# Ecogenomics Reveals Microbial Metabolic Networks in a Psychrophilic Methanogenic Bioreactor Treating Soy Sauce Production Wastewater

**DOI:** 10.1264/jsme2.ME21045

**Published:** 2021-09-30

**Authors:** Kyohei Kuroda, Takashi Narihiro, Masaru K. Nobu, Atsushi Tobo, Masahito Yamauchi, Masayoshi Yamada

**Affiliations:** 1 Bioproduction Research Institute, National Institute of Advanced Industrial Science and Technology (AIST), 2–17–2–1 Tsukisamu‐Higashi, Toyohira‐ku, Sapporo, Hokkaido, 062–8517 Japan; 2 Bioproduction Research Institute, National Institute of Advanced Industrial Science and Technology (AIST), Central 6, Higashi, Tsukuba, Ibaraki 305–8566, Japan; 3 Department of Urban Environmental Design and Engineering, National Institute of Technology, Kagoshima College, 1460–1 Shinko Hayato-cho, Kirishima, 899–5193, Japan

**Keywords:** wastewater treatment, psychrophilic, methanogenic ecosystem, ecogenomics, amino acid degradation

## Abstract

An ecogenomic analysis of the methanogenic microbial community in a laboratory-scale up-flow anaerobic sludge blanket (UASB) reactor treating soy sauce-processing wastewater revealed a synergistic metabolic network. Granular sludge samples were collected from the UASB reactor operated under psychrophilic (20°C) conditions with a COD removal rate >75%. A 16S rRNA gene amplicon sequencing-based microbial community analysis classified the major microbial taxa as *Methanothrix*, *Methanobacterium*, *Pelotomaculaceae*, *Syntrophomonadaceae*, *Solidesulfovibrio*, and members of the phyla *Synergistota* and *Bacteroidota*. Draft genomes of dominant microbial populations were recovered by metagenomic shotgun sequencing. Metagenomic- and metatranscriptomic-assisted metabolic reconstructions indicated that *Synergistota*- and *Bacteroidota*-related organisms play major roles in the degradation of amino acids. A metagenomic bin of the uncultured *Bacteroidales* 4484-276 clade encodes genes for proteins that may function in the catabolism of phenylalanine and tyrosine under microaerobic conditions. *Syntrophomonadaceae* and *Pelotomaculaceae* oxidize fatty acid byproducts presumably derived from the degradation of amino acids in syntrophic association with aceticlastic and hydrogenotrophic methanogen populations. *Solidesulfovibrio* organisms are responsible for the reduction of sulfite and may support the activity of hydrogenotrophic methanogens and other microbial populations by providing hydrogen and ammonia using nitrogen fixation-related proteins. Overall, functionally diverse anaerobic organisms unite to form a metabolic network that performs the complete degradation of amino acids in the psychrophilic methanogenic microbiota.

Soy sauce is a fermented product of soybean that is used as a seasoning for cooking (Lioe *et al.*, 2010). In Japan, there are more than 1,100 soy sauce-processing manufacturing plants and annual production is approximately 744 million liters (Soy Sauce Information Center, 2021, https://www.soysauce.or.jp/). Soy sauce production incurs the discharge of wastewater containing various amino acids with high chemical oxygen demand (COD) concentrations (~150,000‍ ‍mg COD L^–1^) at psychrophilic temperatures (*e.g.*, 16 to 20°C) (Kuroda *et al.*, 2017). To treat this high-strength organic wastewater at a low temperature, an anaerobic treatment system, such as up-flow anaerobic sludge blanket (UASB)- and expanded granular sludge bed (EGSB)-type methanogenic bioreactors, is a promising technology due to its high capacity to degrade concentrated substrates and low energy requirements (Lettinga, 1995; Lettinga *et al.*, 1999, 2001). Although growth and methane production activities are lower than those under mesophilic or thermophilic conditions (Lettinga *et al.*, 2001), previous studies using anaerobic bioreactors operated under psychrophilic conditions showed good efficiency for the treatment of various organic wastewater, including citric acid-processing wastewater (Collins *et al.*, 2006), brewery wastewater (Xing *et al.*, 2009), synthetic fatty acid-based wastewater (Collins *et al.*, 2003; Gunnigle *et al.*, 2015), dairy wastewater (McAteer *et al.*, 2020; Paulo *et al.*, 2020), and low-strength sewage wastewater (Keating *et al.*, 2016, 2018; Petropoulos *et al.*, 2017; Ribera-Pi *et al.*, 2020).

In the methanogenic wastewater treatment system, even if temperature conditions vary between psychrophilic, mesophilic, and thermophilic, the three major trophic groups basically form a synergistic microbial interaction to degrade organic compounds in wastewater into methane and carbon dioxide; fermenters degrade organic compounds (*e.g.*, carbohydrates, proteins, amino acids, and lipids) to fatty acids (*e.g.*, propionate, butyrate, and acetate as volatile fatty acids [VFA]; isovalerate, 2-methylbutyrate, and isobutyrate as branched-chain fatty acids [BCFA]) and hydrogen; syntrophic substrate oxidizers (syntrophs) degrade fatty acids to acetate and hydrogen; and methanogenic archaea (methanogens) further utilize acetate and hydrogen with the production of methane and carbon dioxide (Schink and Stams, 2013). To date, major microbial constituents inhabiting psychrophilic methanogenic bioreactors have been identified by 16S rRNA-targeting molecular approaches (Collins *et al.*, 2006; Xing *et al.*, 2009; Gunnigle *et al.*, 2015; Petropoulos *et al.*, 2017; Keating *et al.*, 2018; Paulo *et al.*, 2020; Ribera-Pi *et al.*, 2020). However, there is currently no information on the metabolic functions of the methanogenic microbiota that proliferates in psychrophilic bioreactors treating high-concentration organic wastewater. The metabolic strategies and interactions employed by microorganisms to accomplish anaerobic amino acid degradation under psychrophilic conditions remain poorly understood. As temperature decreases, methanogens may consume hydrogen to lower concentrations (Conrad and Wetter, 1990), potentially influencing how syntrophic organotrophy is accomplished. Wastewater treatment bioreactors represent rare amino acid-rich ecosystems that have sufficient metabolic activity and biomass to allow omics-based investigations of the above behavior.

We herein attempted to clarify the diversity and metabolic functions of dominant microorganisms in an UASB-type methanogenic bioreactor treating synthetic soy sauce-processing wastewater operated at a psychrophilic temperature (20°C) using an ecogenomic approach with a 16S rRNA gene amplicon, shotgun metagenomic, and metatranscriptomic sequence dataset. Ecogenomics reveals the microorganisms involved in the decomposition of each amino acid, those that may utilize fatty acid byproducts in association with methanogens, and those that may have the ability to support the entire microbial interaction, thereby contributing towards a more detailed understanding of methanogenic amino acid degradation under psychrophilic conditions.

## Materials and Methods

### Reactor operation

The specifications and operation conditions of an UASB-type anaerobic bioreactor were previously described (Kuroda *et al.*, 2017). Briefly, a lab-scale UASB reactor (working volume of 7.0‍ ‍L, H×L×W; 0.7×0.1×0.1 m) was operated at 20°C using a water jacket (Fig. S1). Reactors were operated under different hydraulic retention time (HRT) and volumetric COD loading rates ranging between 6 and 8.6 days and between 3.6 and 24‍ ‍kg COD m^–3^ d^–1^, respectively (Table S1 and Fig. S2). Seed sludge samples were taken from an UASB reactor treating synthetic soy sauce wastewater at 35°C. The reactor was fed with synthetic wastewater that mimicked the composition of wastewater discharged from a soy sauce-processing factory (Table S2). Synthetic wastewater was prepared by the dilution of soy sauce (Koikuchi shoyu, Kikkoman) with tap water. Trace elements of CoCl_2_·6H_2_O (0.17‍ ‍mg L^–1^) and NiCl_2_·6H_2_O (0.04‍ ‍mg L^–1^) were added to synthetic wastewater.

### Analytical methods

Dichromate chemical oxygen demand (COD_Cr_), nitrate, and nitrite were analyzed by a DR3900 spectrophotometer using the COD2 HR kit, NITRA Ver kit, and NITRI Ver3 kit, respectively (Hach). Sulfite and sodium sulfite were analyzed using the Sulfite Test Kit Model SU-5 (Hach). Ammonia and amino acid concentrations were measured by the ACQUITY UPLC H-Class system using the AccQ•Tag Ultra kit according to the manufacturer’s instructions (Waters).

### Granule sludge sampling and DNA/RNA extraction

Sludge samples were collected in triplicate from the middle of the reactor (Port 3, Fig. S1) after 905 days of operation at the maximum COD loading rate (6,000‍ ‍mg COD L^–1^, Table S1). Samples were stored at –80°C prior to use for DNA/RNA extraction. After centrifugation (8,500×*g*, 3‍ ‍min), precipitated sludge samples were collected and stored in a –80°C freezer until DNA extraction. Total DNA and RNA were separately extracted from sludge samples using the FastDNA SPIN Kit for Soil kit (MP Biomedicals) and acid-phenol/beads-beating extraction method (Uyeno *et al.*, 2004), respectively.

### 16S rRNA gene-based microbial community profiling

PCR and amplicon sequencing were performed as previously described (Kuroda *et al.*, 2015, 2016). Briefly, the PCR amplification of 16S rRNA genes was performed with the universal primer set Univ515F/Univ806R. The PCR reaction mixture (20‍ ‍μL) contained 2.0‍ ‍μL of template DNA (10‍ ‍ng μL^–1^), 0.5‍ ‍μM of forward and reverse primers, and 10‍ ‍μL of the Premix Ex Taq Hot Start Version (TaKaRa Bio). PCR was conducted using a thermal cycler (Veriti200, Applied Biosystems) with the following conditions: initial denaturation at 94°C for 3‍ ‍min, denaturation at 94°C for 45‍ ‍s, annealing at 50°C for 60‍ ‍s, elongation at 72°C for 90‍ ‍s, and a final extension at 72°C for 10‍ ‍min. The number of PCR cycles was 25. The purification of PCR products was conducted using a QIAquick PCR purification kit (QIAGEN) following the manufacturer’s protocol. Amplicon sequencing was performed on MiSeq with the MiSeq Reagent v2 kit (Illumina).

Raw 16S rRNA gene reads were processed and trimmed with QIIME 1.9.1 (Caporaso *et al.*, 2010) using sequence length (≥150‍ ‍nt) and quality score (≥25) cut-offs. Screened sequence data were grouped into operational taxonomic units (OTUs) with the UCLUST algorithm using a 97% sequence identity cut-off (Edgar, 2010). Chimeric sequences for each OTU were removed using ChimeraSlayer (Haas *et al.*, 2011). Taxonomy was assigned using classify-sklearn retained on SILVA database version 138 (Yilmaz *et al.*, 2014).

### Metagenomic shotgun sequencing, assembly, and binning

Libraries were prepared with the Nextera XT Library Prep kit (Illumina) with a genomic DNA fragment size ranging between 200 and 1,000 bp. Prepared libraries were sequenced on MiSeq with the MiSeq Reagent v3 kit (Illumina), generating paired-end reads up to 300 bp each, at FASMAC. Assembly and binning were performed as previously described (Nobu *et al.*, 2015, 2016). Briefly, the reads obtained were trimmed using Trimmomatic v0.33 (Bolger *et al.*, 2014); digitally normalized and partitioned using the khmer package (Pell *et al.*, 2012); and assembled using SPAdes v.3.5.0 (Bankevich *et al.*, 2012). The assembled contigs were binned using MetaBAT (Kang *et al.*, 2015) and MaxBin 2.0 (Wu *et al.*, 2014, 2016) with manual curation to eliminate contaminated contigs based on rRNA genes as phylogenetic markers. Short contigs (<1,000 bp) were removed. Genes were predicted using Prodigal v2.5 (Hyatt *et al.*, 2010) and annotated using KEGG (Ogata *et al.*, 1999), BLAST KOALA (Altschul *et al.*, 1990; Kanehisa *et al.*, 2016), IMG/M (Markowitz *et al.*, 2014), and Prokka (Seemann, 2014). The phylogeny and taxonomy of each bin were estimated using PhyloPhlAn software (Segata *et al.*, 2013) and GTDBtk v1.4.1 (GTDB release95; default parameters) (Parks *et al.*, 2018), respectively.

### Metatranscriptomic sequencing and mapping

Ribosomal RNA was removed from extracted total RNA by Ribo-Zero Magnetic Kit Bacteria (Illumina). Libraries were prepared with the KAPA RNA HyperPrep Kit (Thermo Fisher Scientific). Prepared libraries were sequenced on NextSeq 500 (Illumina), generating paired-end reads up to 75 bp each, at the Bioengineering Lab. The reads obtained were trimmed using Sickle v1.33 (Joshi and Fass, 2011); mapped using bowtie2 (Langmead and Salzberg, 2012); converted from SAM to BAM formats using Samtools v1.5 (Li *et al.*, 2009); and RPKM were counted using featureCounts v1.5.3 (Liao *et al.*, 2014).

### Nucleotide sequence accession numbers

The sequence data obtained in the present study have been deposited under DDBJ/EMBL/GenBank accession no. DRA012164.

## Results and Discussion

### Reactor operation

The operational performance of the UASB reactor treating synthetic soy sauce-processing wastewater is shown in Table S1 and Fig. S2. Influent synthetic soy sauce wastewater contained VFA, amino acids, and sulfite (Table S2). The reactor was continuously operated for more than 1,000 days. After the start-up period (Phase 1), the removal efficiencies of COD and VFA were consistently maintained at 60–93%. Granular sludge samples were collected in triplicate for technical replicates after 905 days of operation, at which point operation achieved the maximum COD loading rate (6,000‍ ‍mg COD L^–1^) and a ≥75% soluble COD removal rate, suggesting that major organic compounds in the wastewater were mostly degraded.

### Major microbial constituents and their draft genomes

An overview of the 16S rRNA gene amplicon, shotgun metagenomic, and metatranscriptomic sequencing of sludge samples taken from the UASB reactor treating soy sauce wastewater is shown in Table S3. 16S rRNA gene-based microbial community profiling indicated that the OTUs classified as *Firmicutes* (28.6%), *Synergistota* (14.2%), *Bacteroidota* (12.1%), *Halobacteriota* (14.6%), and *Methanobacteriota* (15.5%) predominated in the reactor (Fig. S3 and Table S4). These dominant microbial populations were previously observed in psychrophilic methanogenic bioreactors treating various types of organic wastewater (Collins *et al.*, 2003, 2006; Xing *et al.*, 2009; Gunnigle *et al.*, 2015; Keating *et al.*, 2016, 2018; Petropoulos *et al.*, 2017; McAteer *et al.*, 2020; Paulo *et al.*, 2020; Ribera-Pi *et al.*, 2020).

**Fig. 1.**
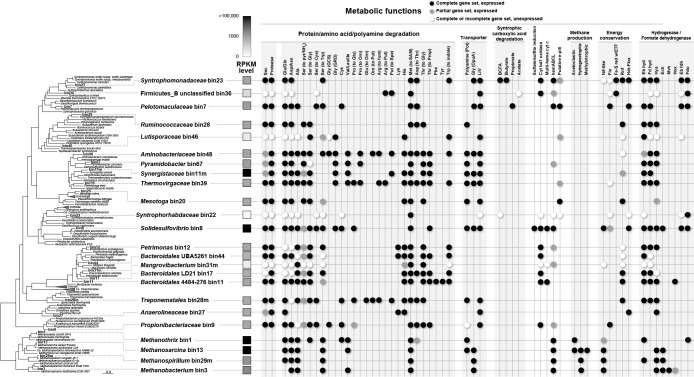
Phylogenetic affiliation and metabolic functions of major microbial constituents in a psychrophilic up-flow anaerobic sludge blanket (UASB) reactor treating synthetic soy sauce wastewater. A distance matrix tree was constructed based on the amino acid sequences of 400 conserved proteins using PhyloPhlAn software. Boldface in the tree indicates the bins obtained in the present study. The averaged RNA expression level (RPKM in triplicate) of each bin is shown as a grayscale heatmap. The metabolic functions of each bin are shown as follows: closed circle, the bin has a complete gene set for each function, and corresponding RNAs were expressed; gray circle, the bin has a partial gene set for each function, and corresponding RNAs were expressed; open circle, although the bin has a complete or partial gene set for each function, corresponding RNAs were not expressed. Amino acids are shown as common three-letter abbreviations according to the International Union of Pure and Applied Chemistry (IUPAC). Other abbreviations: Sec, bacterial secretion system; Pyr, pyruvate; GCS, glycine cleavage system; GRD, glycine reductase; Put, putrescine; Spe, spermidine; SAM, S-adenosyl-l-methionine; Pot, ABC-type polyamine transporter; OpuA, osmotically regulated binding protein-dependent transport system for the osmoprotectant glycine betaine; LIV, branched-chain amino acid transport system; Fum, fumarate; Prop, propionate; BCFA, branched-chain fatty acid; Cyt, cytochrome; Nif, nitrogen fixation complex; ETF, electron transfer flavoprotein; Fix, ETF-oxidizing hydrogenase complex; Fe-S red, Fe-S reductase; Hdr-Flox, heterodisulphide reductase-flavin oxidoreductase complex, Rnf, *Rhodobacter* nitrogen fixation complex; Eb hyd, electron-bifurcating hydrogenase; Fd hyd, ferredoxin-dependent [NiFe]-hydrogenase; Hya, NADH/quinone-dependent [NiFe]-hydrogenase; Ech, energy-conserving hydrogenase; Mvh, methyl-viologen-reducing hydrogenase; Hox, bidirectional soluble hydrogenase; Eb fdh, electron-bifurcating formate dehydrogenase; Fdo, formate dehydrogenase O.

We successfully obtained 24 high quality (*i.e.*, >80% completeness) draft genomes of major microbial taxa from shotgun metagenomic sequence data (Table S5); *i.e.*, *Syntrophomonadaceae* (bin23), unclassified Firmicutes_B clade (bin36), *Pelotomaculaceae* (bin7), *Ruminococcaceae* (bin28), and *Lutisporaceae* (bin46) for *Firmicutes*; *Aminobacteriaceae* (bin48), *Pyramidobacter* (bin67), *Synergistaceae* (bin11m), and *Thermovirgaceae* (bin39) for *Synergistota*; *Petrimonas* (bin12), *Mangrovibacterium* (bin31m), and uncultured *Bacteroidales* clades (bin11, bin17, and bin44) for *Bacteroidota*; *Methanothrix* (bin1), *Methanosarcina* (bin13), and *Methanospirillum* (bin29m) for *Halobacteriota*; and *Methanobacterium* (bin3) for *Methanobacteriota*. Draft genomes associated with *Desulfobacterota* (*Solidesulfovibrio* bin8 and *Syntrophorhabdaceae* bin22), *Chloroflexota* (*Anaerolineaceae* bin27), *Spirochaetota* (*Treponematales* bin28m), *Thermotogota* (*Mesotoga* bin20), and *Actinobacteriota* (*Propionibacteriaceae* bin9) were also recovered from the metagenomic sequence pool. The phylogenetic relationship of the metagenomic bins and previously isolated representatives is shown in Fig. 1.

**Fig. 2.**
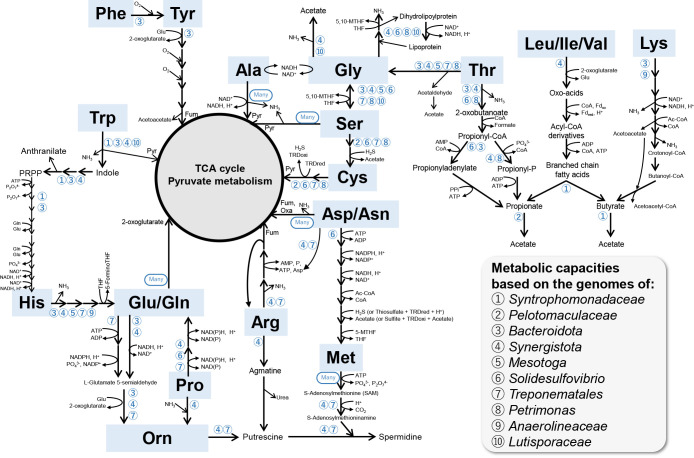
Proposed scheme for the methanogenic degradation of amino acids in a psychrophilic up-flow anaerobic sludge blanket (UASB) reactor treating synthetic soy sauce wastewater. In each pathway, representative microbial population(s) are shown as a number in a panel. “Many” indicates that more than 7 representative populations have the corresponding metabolic capacities. Amino acids are shown as a common three-letter abbreviation according to the International Union of Pure and Applied Chemistry (IUPAC). Other abbreviations: PRPP, 5-phospho-alpha-d-ribose 1-diphosphate; THF, tetrahydrofolate; 5-MTHF, 5-methyltetrahydrofolate TRDred, reduced thioredoxin; TRDoxi, oxidized thioredoxin; Fum, fumarate; Pyr, pyruvate; Oxa, oxaloacetate.

Transcript levels of annotated genes encoded in each bin are shown in Supplemental Table S5–S30. Among these bins, aceticlastic (*Methanothrix* bin1), hydrogenotrophic (*Methanobacterium* bin3 and *Methanospirillum* bin29m), and methylotrophic (*Methanosarcina* bin13) methanogens showed relatively high RNA expression levels in methanogenesis-related protein complexes, such as methyl-coenzyme M reductase (Mcr), tetrahydromethanopterin S-methyltransferase (Mtr), CoB--CoM heterodisulfide reductase (Hdr), coenzyme F420-reducing hydrogenase, and formate dehydrogenase (Table S7–S10), indicating stable methanogenic bioconversion. Within the domain *Bacteria*, *Synergistota*-related bins (bin11m, bin48, and bin67), *Solidesulfovibrio* bin8, *Pelotomaculaceae* bin7, and *Syntrophomonadaceae* bin23 recorded relatively high transcript levels. They have been suggested to play a role in amino acid degradation (Bhandari and Gupta, 2012), sulfite reduction (Baffert *et al.*, 2019), syntrophic propionate oxidation (Hidalgo-Ahumada *et al.*, 2018), and syntrophic butyrate oxidation (Sieber *et al.*, 2010; Narihiro *et al.*, 2016), respectively, which were inferred from the major components of soy sauce wastewater (Table S1). The potential metabolic functions of dominant bacteria are shown in Fig. 1. Details with a special emphasis on the degradation of amino acids are described in the following sections according to metagenome- and metatranscriptome-informed metabolic reconstruction (Fig. 2, Table S11–S30).

### Metabolic capacities of dominant organisms

#### Glutamine, glutamic acid, asparagine, aspartic acid, and alanine

Many microbial constituents utilized Glu/Gln, Asp/Asn, and Ala and produced pyruvate, fumarate, oxaloacetate, and 2-oxoglutarate, which were then used in the citric acid cycle. Gln was generated from Glu and 2-oxoglutarate by glutamate synthase using NAD(P)H as an electron donor. Glutamate dehydrogenase catalyzed the production of 2-oxoglutarate from Gln. Asp was converted to Asn by asparaginase, and aspartate transaminase catalyzed the conversion of Asn to oxaloacetate via an amino-group transfer reaction using 2-oxoglutarate as the substrate and glutamate as the byproduct. Fumarate was produced from Asn by a two-step reaction catalyzed by adenylosuccinate synthetase and adenylosuccinate lyase. Ala may be converted to pyruvate by NADH-dependent alanine dehydrogenase. Glutamate-pyruvate aminotransferase also catalyzed the conversion of Ala to pyruvate via an amino-group transfer reaction via a similar mechanism to aspartate transaminase.

#### Serine, glycine, threonine, and cysteine

Many bacterial bins harbor a l-serine dehydratase gene cluster that is responsible for the conversion of Ser to pyruvate and NH_3_ and a serine hydroxymethyltransferase gene for the reversible conversion of Ser and Gly with the co-factor tetrahydrofolate (THF). Gly is further degraded through the glycine cleavage system (GCS) or Stickland reaction with a glycine reductase (GRD) complex. *Solidesulfovibrio* bin8, *Aminobacteriaceae* bin48, *Lutisporaceae* bin46, and *Propionibacteriaceae* bin9 possess a gene set for GCS (*i.e.*, glycine dehydrogenase, aminomethyltransferase, dihydrolipoyl dehydrogenase, and glycine cleavage system H protein) that catalyzes Gly to NH_3_ and CO_2_. The draft genomes of four *Synergistota* bins and *Lutisporaceae* bin46 encode a GRD gene cluster (GrdABC) for the conversion of Gly to acetate with the coupled oxidation reaction of some amino acids (*e.g.*, Ala). The partial selenoprotein biosynthetic gene cluster *selABCD* (l-seryl-tRNA[Sec] selenium transferase, *selA*; selenocysteine-specific elongation factor, *selB*; tRNA-Sec, *selC* selenide, water dikinase, *selD*) was detected in these GRD-encoded bins because the GrdA subunit contains selenocysteine. Gly was also derived from Thr by l-threonine aldolase found in some of the bacterial bins. Thr may be converted to 2-oxobutanoate and NH_3_ by threonine ammonia-lyase encoded in the genomes of *Bacteroidota*, *Synergistota*, and *Propionibacteriaceae*, and propionate is then produced via the fermentative degradation of 2-oxobutanoate.

Serine o-acetyltransferase and cysteine synthase are responsible for the conversion of Ser to Cys. Furthermore, l-cysteine:2-oxoglutarate aminotransferase may catalyze Cys and 2-oxoglutarate to 3-mercaptopyruvate and Glu, and 3-mercaptopyruvate may then be transformed to pyruvate by 3-mercaptopyruvate sulfurtransferase with reduced thioredoxin. *Solidesulfovibrio* bin8, *Propionibacteriaceae* bin9, *Treponematales* bin28m, *Syntrophorhabdaceae* bin22, and *Pelotomaculaceae* bin7 possess these Ser/Cys utilization enzymes.

#### Lysine, branched-chain amino acids (BCAA), and fatty acids

The Lys fermentation pathway comprises five enzymes: l-lysine-2,3-aminomutase, β-l-lysine-5,6-aminomutase, 3,5-diaminohexanoate dehydrogenase, 3-keto-5-aminohexanoate cleavage enzyme, and 3-aminobutyryl-CoA ammonia lyase. Through this pathway, Lys was fermented to crotonyl-CoA, and may then be further degraded to acetate via the β-oxidation pathway. Among these enzymes, 3,5-diaminohexanoate dehydrogenase requires NAD^+^ as a co-factor. Metagenomic bins of *Petrimonas* bin12, *Bacteroidales* UBA5261 bin44, and *Anaerolineaceae* bin27 contain a complete gene set for Lys fermentation, implying their involvement in Lys degradation and butyrate production in this reactor.

Four bins of *Synergistota* organisms encode BCAA aminotransferase and ferredoxin (Fd)-dependent branched-chain oxo-acid reductase for the degradation of Leu, Ile, and Val to correspond to BCFA: isovalerate, 2-methylbutyrate, and isobutyrate, respectively. The gene cassette for the BCAA transporter (*LivFGHKM*) was also found in these *Synergistota* metagenomes. They have no ability to oxidize BCFA due to the lack of 2-methylbutyryl-CoA dehydrogenase, 3-methylbutyryl-CoA dehydrogenase, or isobutyryl-CoA mutase. Multiple active homologs of acyl-CoA dehydrogenase and a gene cassette of isobutyryl-CoA mutase were instead found in *Syntrophomonadaceae* bin23. In addition, other key enzymes for β-oxidation (*i.e.*, enoyl-CoA hydratase, 3-hydroxybutyryl-CoA dehydrogenase, acetyl-CoA acetyltransferase, phosphotransacetylase, and acetate kinase), the electron transfer flavoprotein (ETF)-oxidizing hydrogenase complex (FixABCX), the heterodisulphide reductase-flavin oxidoreductase (Hdr-Flox) complex, *Rhodobacter* nitrogen fixation (Rnf) complex, hydrogenases, and formate dehydrogenases were found in *Syntrophomonadaceae* organisms (Table S11), which are in accordance with the genomic traits of *Syntrophomonas* strains (Sieber *et al.*, 2010; Narihiro *et al.*, 2016), suggesting its role in the syntrophic oxidation of BCFA and butyrate.

Propionate may be produced by the degradation of 2-methylbutyrate from Ile as well as the degradation of 2-oxobutanoate from Thr, and propionate is then oxidized to acetate via the methylmaronyl-CoA pathway encoded by *Pelotomaculaceae* bin7. *Pelotomaculaceae* organisms encode Hdr-Flox, FixABCX, ETF-linked acyl-CoA dehydrogenase, hydrogenases, and formate dehydrogenases (Table S13). Among these energy-conserving systems, the activities of cytoplasmic and membrane-bound formate dehydrogenases were higher than those of hydrogenases. Formate-dominated interspecies electron transfer was demonstrated in our previous ecogenomic studies using a co-culture of *Pelotomaculum* strains with hydrogenotrophic methanogen (Hidalgo-Ahumada *et al.*, 2018), a lab-scale propionate-fed chemostat (Chen *et al.*, 2020), and full-scale anaerobic digesters (Nobu *et al.*, 2020). In addition to hydrogen/formate-mediated interspecies electron transfer, direct interspecies electron transfer (DIET) employing conductive pili (e-pili) has been investigated (Summers *et al.*, 2010). *Pelotomaculaceae* bin7 possesses a pilus assembly gene cassette with PilA, which meets the criteria for the e-pili protein (SOY3_bin007_00676, Fig. S4) (Holmes *et al.*, 2017; Walker *et al.*, 2018); however, the transcript level of this cassette is low. In a previous study, the effects of granular-activated carbon (GAC) on *pilA* gene expression in a psychrophilic (20°C) UASB reactor treating municipal sewage was estimated using a RT-qPCR approach (Zhang *et al.*, 2020). The findings obtained confirmed that the addition of GAC up-regulated *pilA* gene expression, while the expression levels of *pilA* were 36-fold lower in non-GAC-amended UASB granular sludge. Therefore, we presume that *Pelotomaculaceae* bin7 in our psychrophilic UASB reactor has the potential for an e-pili-mediated DIET reaction; however, the transcription level without mediator material is not high. Therefore, the genomic features of *Pelotomaculaceae* bin7 indicate its versatile syntrophic lifestyle for propionate oxidation in the reactor.

#### Tryptophan and histidine

The draft genomes of *Lutisporaceae* bin46, *Aminobacteriaceae* bin48, *Thermovirgaceae* bin39, and five *Bacteroidota* bins harbored tryptophanase, which may catalyze Try to indole, pyruvate, and NH_3_. Some of these bins possess the ability to convert indole to 5-phospho-alpha-d-ribose 1-diphosphate (PRPP) and anthranilate (2-aminobenzoate). PRPP is a common compound for nucleotide metabolism. Indole and anthranilate are important intermediates for the biosynthesis of alkaloid compounds. Although the absolute abundance and ecophysiological role of indole and its derivatives in methanogenic ecosystems, including our UASB reactor, remain unknown, previous studies reported that these compounds are associated with bacterial signaling in *Escherichia coli* and *Ralstonia solanacearum* (Pinero-Fernandez *et al.*, 2011; Song *et al.*, 2020). PRPP may be further converted to His through a 10-step reaction employing ATP, Glu, and NAD^+^. His may be converted to Glu via a four-step reaction employing histidine ammonia-lyase, urocanate hydratase, imidazolonepropionase, and glutamate formimidoyltransferase by organisms associated with *Synergistota*, *Lutisporaceae*, *Mesotoga*, *Bacteroidota*, *Treponematales*, and *Anaerolineaceae*.

#### Arginine, methionine, proline, and ornithine

*Solidesulfovibrio*, *Thermovirgaceae*, and *Treponematales* bins harbor two NAD(P)H-dependent enzymes, pyrroline-5-carboxylate reductase and l-glutamate gamma-semialdehyde dehydrogenase, to produce the anaplerotic amino acid Glu from Pro. Ornithine, which is a non-proteinogenic amino acid, may be generated from Glu via a three-step reaction using glutamate 5-kinase, glutamate-5-semialdehyde dehydrogenase, and ornithine aminotransferase, which are encoded within *Treponematales* bin28m. Three bins of *Synergistota* organisms (bin48, bin67, and bin39) possess ornithine cyclodeaminase, which catalyzes Pro to Orn. Orn may be converted to putrescine by ornithine decarboxylase encoded in *Aminobacteriaceae* bin48 and *Treponematales* bin28m. Putrescine was also produced from Arg by arginine decarboxylase and agmatinase, which are encoded within *Thermovirgaceae* bin39 and unclassified Firmicutes_B bin36. Four draft genomes (*Aminobacteriaceae* bin48, *Synergistaceae* bin11m, *Treponematales* bin28m, and unclassified Firmicutes_B bin36) encode spermidine synthase, which may catalyze putrescine with S-adenosyl-l-methionine (SAM) to spermidine. Most of the metagenomic draft genomes harbor S-adenosylmethionine synthase to produce SAM from Met. Several organisms possess a gene cassette encoding potential ABC-type polyamine transporter subunits (Pot). Spermidine is a polyamine that is involved in the anaerobic growth of *E. coli* (Chattopadhyay *et al.*, 2009) and *Anaerovibrio lipolytica* (Hirao *et al.*, 2000), the microaerobic growth of *Borrelia burgdorferi* (Bontemps-Gallo *et al.*, 2018), biofilm formation by *Yersinia pestis* (Patel *et al.*, 2006) and *Bacillus subtilis* (Burrell *et al.*, 2010), and other physiological functions (Michael, 2018). Zhu *et al.* (2015) reported that spermidine promoted gene expression and the replacement of damaged proteins in cyanobacteria under cold stress conditions. Some psychrophilic bacteria were shown to accumulate spermidine as a predominant polyamine within their cells (Margesin *et al.*, 2012; Zhang *et al.*, 2012; Dahal *et al.*, 2019; Kumar *et al.*, 2020). These findings imply that spermidine acts as a supportive agent for microbial metabolic activities in methanogenic microbiota in our UASB reactor operated under psychrophilic conditions. Further studies employing a community-level exometabolome approach (Kell *et al.*, 2005; Douglas, 2020) are needed to clarify this assumption.

#### Phenylalanine and tyrosine

**Fig. 3.**
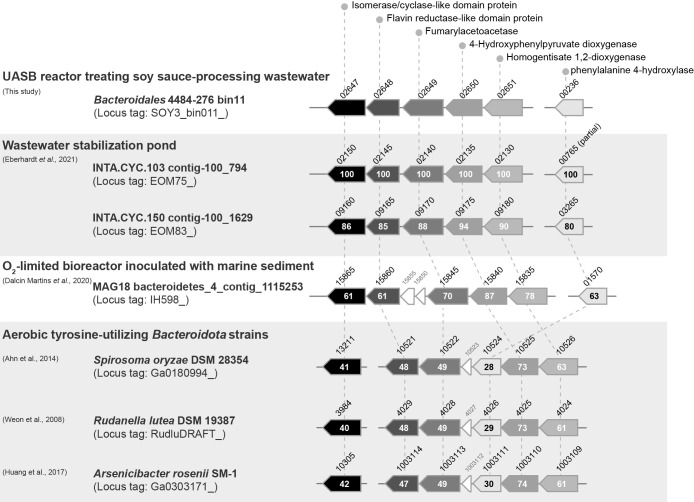
Comparison of gene cassettes associated with the bioconversion of 4-hydroxyphenylpyruvate to acetoacetate/fumarate found in uncultured *Bacteroidales* 4484-276 bin11, previously reported metagenomic contigs derived from probable microaerobic ecosystems (Eberhardt *et al.*, 2020; Dalcin Martins *et al.*, 2021), and aerobic *Bacteroidota* isolates (Weon *et al.*, 2008; Ahn *et al.*, 2014; Huang *et al.*, 2017). An uncultured *Bacteroidales* 4484-276 bin11-associated cassette encodes homogentisate 1,2-dioxygenase, 4-hydroxyphenylpyruvate dioxygenase, fumarylacetoacetase, flavin reductase-like domain protein, and putative isomerase/cyclase-like domain protein. Phenylalanine 4-hydroxylase is also shown. Abbreviated locus tags are shown (*e.g.*, ‘SOY3_bin011_02651’ as ‘02651’ in the row of *Bacteroidales* 4484-276 bin11). Numbers in gene boxes indicate amino acid sequence identity (%) to the corresponding gene of uncultured *Bacteroidales* 4484-276 bin11. The contigs INTA.CYC.103 contig-100_794 (37,009 bp), INTA.CYC.150 contig-100_1629 (24,086 bp), and MAG18 bacteroidetes_4_contig_1115253 (29,273 bp) were originally named as *Candidatus* Falkowbacteria bacterium (accession no. SABQ01000012), *Clostridia* bacterium (SABY01000055), and *Bacteroidales* bacterium (JACZJL010000162), respectively, in the NCBI database. We checked the phylogenetic assignment of these contigs by a blastp search (Altschul *et al.*, 1990) for some functionally important enzymes; *i.e.*, RNA polymerase (locus tag: EOM75_02175), DNA repair protein (EOM75_02190), and chromosomal replication initiator protein (EOM75_02085) for INTA.CYC.103 contig-100_794; RNA polymerase (EOM83_09135), DNA repair protein (EOM83_09120), and aminotransferase (EOM83_09145) for INTA.CYC.150 contig-100_1629; 5′/3′-nucleotidase (IH598_15930), transketolase (IH598_15890), and catalase (IH598_15870) for MAG18 bacteroidetes_4_contig_1115253. Since all enzymes are closely related to *Bacteroidota* organisms, we temporarily concluded that these contigs may be derived from *Bacteroidota*-related microorganisms.

Although the degradation pathway of Phe and Tyr has not yet been elucidated in detail in a methanogenic environment, a bin of uncultured *Bacteroidales* 4484-276 (bin11) encodes some genes for the aerobic utilization of Phe and Tyr. Phe is degraded to fumarate and acetoacetate by a six-step reaction catalyzed by phenylalanine 4-hydroxylase, l-tyrosine:2-oxoglutarate aminotransferase, 4-hydroxyphenylpyruvate dioxygenase, homogentisate 1,2-dioxygenase, maleylacetoacetate isomerase, and fumarylacetoacetase (Arias-Barrau *et al.*, 2004). Despite rigorous explorations of the gene repertory of all metagenomic sequence data retrieved from UASB granular sludge samples, we did not identify any maleylacetoacetate isomerase gene with significant amino acid similarity to previously reported genes. We found the active RNA expression of a gene encoding the putative isomerase/cyclase-like domain-containing protein (SOY3_bin011_02647) along with a flavin reductase gene (SOY3_bin011_02648), which are adjacent to the genes for homogentisate 1,2-dioxygenase (SOY3_bin011_02651), 4-hydroxyphenylpyruvate dioxygenase (SOY3_bin011_02650), and fumarylacetoacetase (SOY3_bin011_02649) (Fig. 3). The amino acid sequence of the protein SOY3_bin011_02647 showed relatively low identity (~40%) with the proteins of tyrosine-utilizing *Bacteroidota* isolates, which possess a gene cassette encoding phenylalanine 4-hydroxylase, 4-hydroxyphenylpyruvate dioxygenase, homogentisate 1,2-dioxygenase, and fumarylacetoacetase (Weon *et al.*, 2008; Ahn *et al.*, 2014; Huang *et al.*, 2017). By expanding to the public environmental metagenomic database, three similar gene clusters consisting of homologs of putative isomerase/cyclase-like domain-containing proteins, flavin reductase, homogentisate 1,2-dioxygenase, 4-hydroxyphenylpyruvate dioxygenase, and fumarylacetoacetase, were found in contigs associated with *Bacteroidota* populations inhabiting ecosystems at low oxygen levels (Eberhardt *et al.*, 2020; Dalcin Martins *et al.*, 2021) (Fig. 3). Among these enzymes, phenylalanine 4-hydroxylase, 4-hydroxyphenylpyruvate dioxygenase, and homogentisate 1,2-dioxygenase require molecular oxygen for reactions to proceed. In our previous ecogenomic study, we revealed that bacterial populations in an anaerobic digester decomposing excess sludge derived from a municipal sewage treatment process survived microaerobic conditions by employing cytochrome *bd* oxidase, which is a terminal oxidase for aerobic respiration (Nobu *et al.*, 2020). Uncultured *Bacteroidales* 4484-276 bin11 as well as *Syntrophomonadaceae* bin23, *Solidesulfovibrio* bin8, *Propionibacteriaceae* bin9, and other *Bacteroidota* bins encode a cytochrome *bd* oxidase complex that utilizes nanomolar-level oxygen as an electron acceptor to promote oxygen-dependent respiration. Although the exact function of the putative isomerase/cyclase-like domain-containing protein remains unclear, uncultured *Bacteroidales* 4484-276 bin11 organisms may be responsible for the degradation of Phe and Tyr in the UASB reactor.

### Sulfite reduction and possible nitrogen fixation

Soy sauce-processing wastewater contains a high concentration of sodium sulfite (Table S2). Among the major bacterial constituents, only *Solidesulfovibrio* bin8 encodes the complete gene set for sulfate/sulfite reduction comprising sulfate adenylyltransferase (*sat*), adenylyl-sulfate reductase (*aprAB*), the dissimilatory sulfite reductase catalytic subunit (*dsrABCD*), and membrane-bound sulfite reductase-associated electron transfer protein subunit (*dsrMKJOP*) with relatively high transcript levels. According to its genome information, this *Solidesulfovibrio*-related organism may utilize Gly, Ser, Cys, Pro, Thr, Asp/Asn, and Glu/Gln, but does not oxidize fatty acids (*e.g.*, propionate and butyrate). The feature of amino acid utilization is in partial agreement with the ability of previously known amino acid-utilizing *Solidesulfovibrio* (formerly known as *Desulfovibrio*) spp. isolated from anaerobic wastewater treatment systems (Baena *et al.*, 1998; Hernandez-Eugenio *et al.*, 2000) and a natural psychrophilic environment (Pecheritsyna *et al.*, 2012). The presence of a *Desulfovibrionaceae*-related population was previously reported in the 16S rRNA-based microbial community profiling of psychrophilic anaerobic bioreactor ecosystems (McKeown *et al.*, 2009; Keating *et al.*, 2016, 2018; McAteer *et al.*, 2020; Ribera-Pi *et al.*, 2020); however, their ecophysiological function has yet to be clarified. Sulfate-reducing bacteria and methanogenic archaea compete for a carbon source and/or H_2_ in an anaerobic bioreactor treating sulfate-/sulfite-rich wastewater (Raskin *et al.*, 1996; Omil *et al.*, 1998; Lu *et al.*, 2017; Wu *et al.*, 2018). Through a genome analysis of *Solidesulfovibrio* bin8, we found the Hdr-Flox complex, an energy-conserving hydrogenase (Ech), membrane-bound cytochrome, and carbon monoxide oxidation (Coo)-hydrogenase with carbon monoxide dehydrogenase, which may catalyze the reversible conversion between CO and CO_2_, flavoredoxin, and NADH dehydrogenase, all of which are responsible for acquiring ATP coupling with sulfate/sulfite reduction, in members of *Desulfovibrionaceae* (Chen *et al.*, 1993; Voordouw, 2002; Meyer *et al.*, 2014; Hadj-Said *et al.*, 2015). A nitrogenase-like gene cluster was detected in the genome along with molybdate and ammonia transporters (Table S25). Previous microbial genome analyses indicated that nitrogen fixation-related proteins (Nif) are distributed in functionally diverse microorganisms including fermentative bacteria, syntrophic substrate oxidizers, methanogenic archaea, and sulfate-reducing bacteria (Dos Santos *et al.*, 2012; Narihiro *et al.*, 2016). The transcript levels of nitrogenase-like gene clusters were abundant in *Solidesulfovibrio* bin8 (Table S25) as well as in the methanogenic archaea (*Methanothrix* bin1, *Methanobacterium* bin3, *Methanospirillum* bin29m, and *Methanosarcina* bin13) (Table S7–10). We speculated that nitrogen fixation in some syntrophs having Nif-related genes may serve as a mechanism to tolerate acidification by providing hydrogen and ammonia for partner hydrogenotrophic methanogens to survive under hydrogen/ammonia-limited conditions (Narihiro *et al.*, 2016). Sayavedra *et al.* (2021) recently reported that the sulfate-reducing bacterium *Desulfovibrio diazotrophicus* strain QI0027 isolated from the human gut exhibited nitrogen fixation activity. These findings suggest that *Desulfovibrionaceae* organisms interact with other trophic populations in the UASB reactor by providing hydrogen and ammonia, particularly under psychrophilic conditions in which the growth rate and metabolic activity of methanogens are lower than those under mesophilic and thermophilic conditions (Lettinga *et al.*, 2001).

In summary, microbial metabolic functions associated with the methanogenic degradation of amino acids in a psychrophilic UASB reactor treating soy sauce-processing wastewater were investigated using an ecogenomic approach. The results obtained demonstrated that members of *Bacteroidota*, *Synergistota*, *Treponematales*, and *Propionibacteriaceae* were the major active populations for the anaerobic fermentative degradation of amino acids. Exceptionally, Phe and Tyr were degraded by the unique enzymes encoded in the uncultured *Bacteroidota*-related organism that may function under microaerobic conditions. Fatty acids resulting from the degradation of the BCAA, Lys and Thr were further oxidized by *Pelotomaculaceae*- and *Syntrophomonadaceae*-related bacteria in syntrophic association with partner hydrogenotrophic methanogens. *Solidesulfovibrio* organisms play a major role in the reduction of sulfite, which is abundant in soy sauce wastewater, and may support the activity of hydrogenotrophic methanogens and other microbial populations by providing hydrogen and ammonia via the Nif-like complex. We consider the maintenance of this sophisticated metabolic network of functionally diverse microbes to be essential for the methanogenic wastewater treatment process under low temperature conditions.

## Citation

Kuroda, K., Narihiro, T., Nobu, M. K.., Tobo, A., Yamauchi, M., and Yamada, M. (2021) Ecogenomics Reveals Microbial Metabolic Networks in a Psychrophilic Methanogenic Bioreactor Treating Soy Sauce Production Wastewater. *Microbes Environ ***36**: ME21045.

https://doi.org/10.1264/jsme2.ME21045

## Supplementary Material

Supplementary Material
