# Supplementary material for: Ecogenomics Reveals Microbial Metabolic Networks in a Psychrophilic Methanogenic Bioreactor Treating Soy Sauce Production Wastewater
Source: Microbes Environ. 2021 Sep 30;36(4):ME21045. doi: 10.1264/jsme2.ME21045 (PMC8674449; doi:10.1264/jsme2.ME21045)
Supplement: Supplementary file 1 — Supplementary Material [file 36_21045_s1.pdf]

Supplemental Infomation

Ecogenomics reveals microbial metabolic networks in a psychrophilic methanogenic bioreactor treating soy sauce production wastewater

Kyohei Kuroda [1], Takashi Narihiro [1*], Masaru K. Nobu [2], Atsushi Tobo [3], Masahito Yamauchi [3], Masayoshi Yamada [3]

[1]Bioproduction Research Institute, National Institute of Advanced Industrial Science and Technology (AIST), 2-17-2-1 Tsukisamu-Higashi, Toyohira-ku, Sapporo, Hokkaido, 062-8517 Japan

[2] Bioproduction Research Institute, National Institute of Advanced Industrial Science and Technology (AIST), Central 6, Higashi, Tsukuba, Ibaraki 305-8566, Japan

[3] Department of Urban Environmental Design and Engineering, National Institute of Technology, Kagoshima College, 1460-1 Shinko Hayato-cho, Kirishima, 899-5193, Japan

[*]Corresponding author. +81-29-861-9443, t.narihiro@aist.go.jp

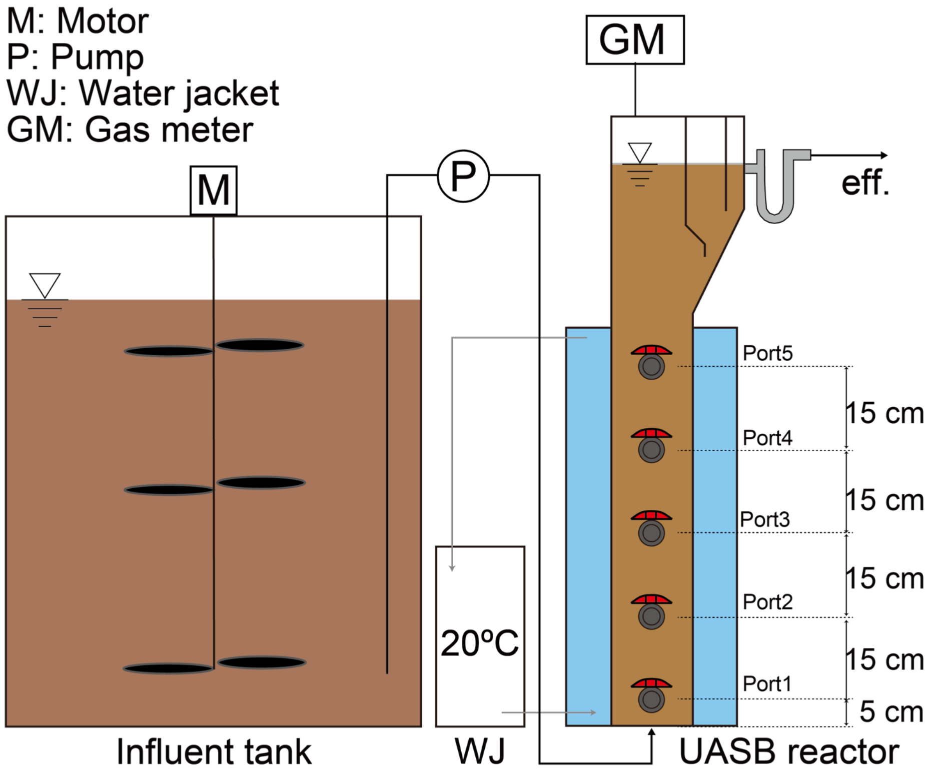

Fig. S1. Cross-section illustration of the lab-scale up-flow anaerobic sludge blanket (UASB) reactor. The reactor was equipped with water jacket (WJ) and heated by water heater to kept at 20°C.

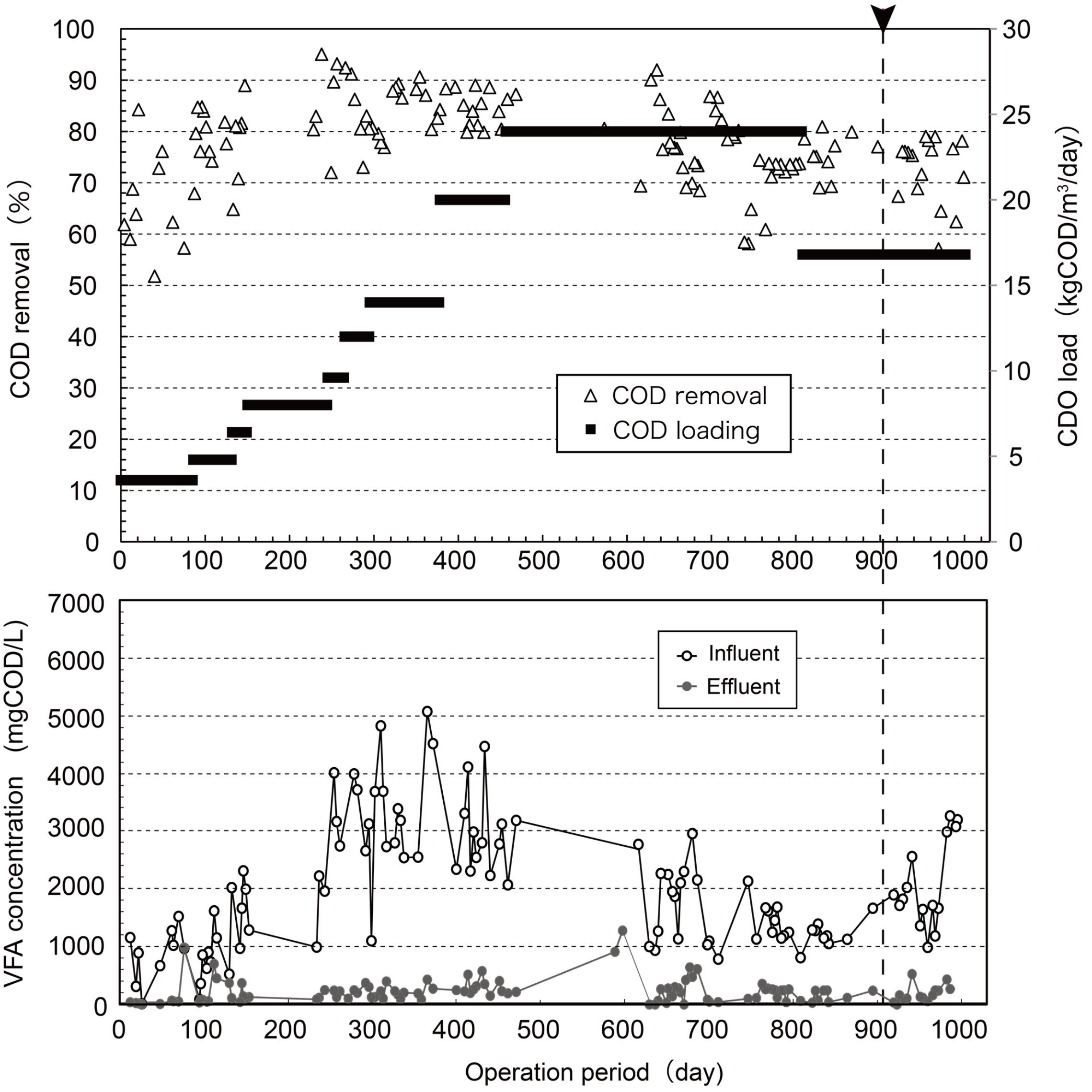

Fig. S2. Changes in operational parameters of up-flow anaerobic sludge blanket (UASB) reactor. Open triangle, COD removal (%); closed square, volumetric COD loading rate (kgCOD m^{-3} d^{-1}); open circle, total influent volatile fatty acid (VFA) concentration (mgCOD L^{-1}); closed circle, total effluent VFA concentration (mgCOD L^{-1}). The reactors were operated at different volumetric COD loading rate ranging from 3.6 to 24 kgCOD m^{-3} d^{-1}. The arrow indicates the periods for biomass sampling from the reactor.

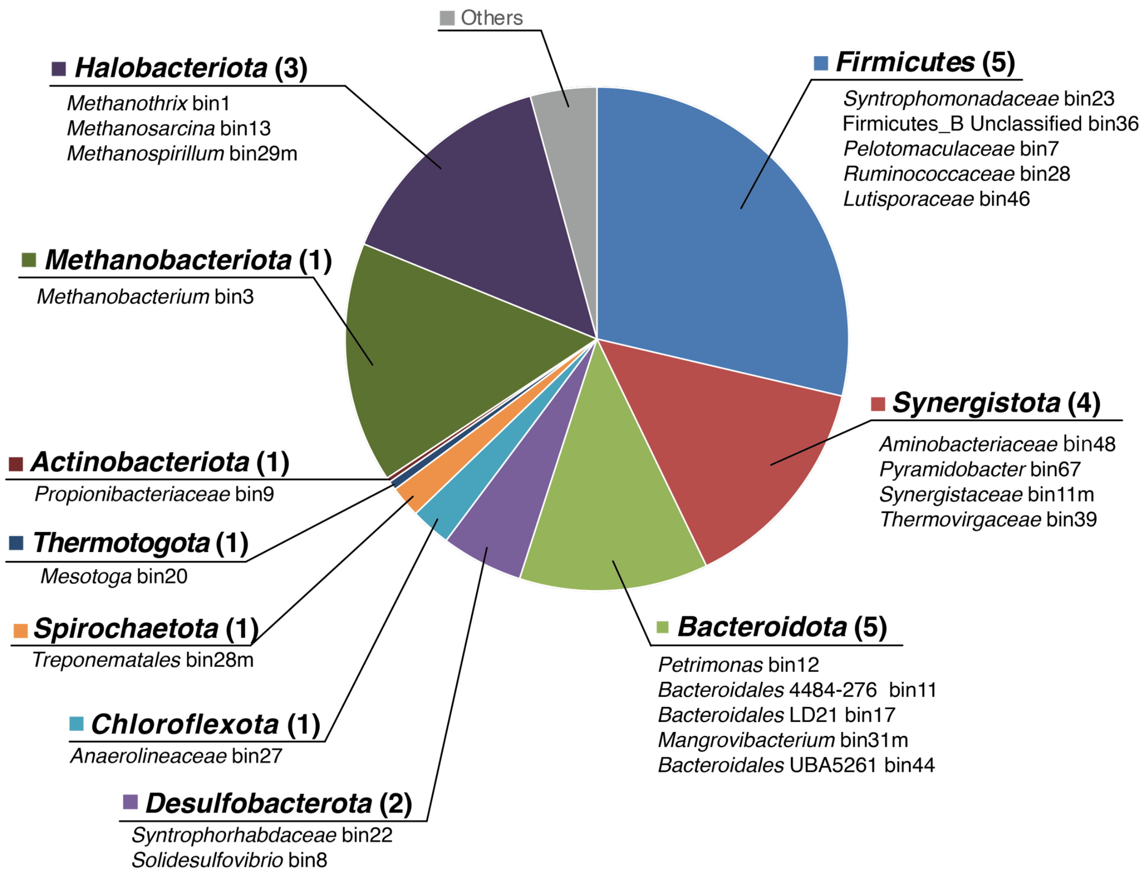

Fig. S3. Pie chart indicates the 16S rRNA gene-based relative abundances of major microbial constituents at phylum/class level retrieved from an up-flow anaerobic sludge blanket (UASB) reactor treating synthetic soy sauce wastewater. The numbers in parentheses indicate the number of metagenomic draft genome bins retrieved from granular sludge taken from the reactor. The taxonomic assignments and bin IDs are shown below the phylum/class name.

>SOY3_bin007_00676 hypothetical protein, putative prepilin-type N-terminal cleavage/methylation domain-containing protein PilW

```
MCEFTGNKGF TLVEAIVSLT IFVLAVSAVL TVYLQGYAGY LENNQKIEVQ EGLRIALNRI
SRDLRQAKSE PEIFKQDGSL SRDGTGSKIS FLTASGETVT YSFDRRDREL EVRRGAAGSF
QPVVSHIEDL EFQFDSDKKI VAIYIRGKKH NSNPPVVYEM KTKVQARARK AGV
```

Fig. S4. Amino acid sequence of putative conductive pili (e-pili) found in the *Pelotomaculum* bin7. Three criteria of conductivity for *Geobacter sulfurreducens* e-pili have been proposed (Holmes *et al.*, 2017; Walker *et al.*, 2018): (1) aromatic amino acids (F, phenylalanine; Y, tyrosine; H, histidine; W, tryptophan; shown in red character) should be located in the key positions, underbar indicated; (2) the abundance of aromatic amino acids greater than 9%, 9.8% and (3) no aromatic-free gaps of greater than 40 amino acids, the maximum gap is 33 amino acids shown in gray colored.

References

Holmes, D.E., Shrestha, P.M., Walker, D.J.F., Dang, Y., Nevin, K.P., Woodard, T.L., and Lovley, D.R. (2017) Metatranscriptomic Evidence for Direct Interspecies Electron Transfer between Geobacter and Methanothrix Species in Methanogenic Rice Paddy Soils. Appl Environ Microbiol 83.

Walker, D.J., Adhikari, R.Y., Holmes, D.E., Ward, J.E., Woodard, T.L., Nevin, K.P., and Lovley, D.R. (2018) Electrically conductive pili from pilin genes of phylogenetically diverse microorganisms. ISME J 12: 48-58.

Table S1. Reactor operation

Phase	Day	Influent COD concentration (mgCOD/L)	HRT (hr)	Volumetric COD loading rate (kgCOD/m3/day)	Total COD removal rate (%)	Soluble COD removal rate (%)	Volatile fatty acids removal rate (%)	Gas production (NL/day)	Methane gas Production (NL/day)
1	1-85	1200	8	3.6	54 ± 15	62 ± 16	71 ± 40	0.6 ± 1.0	0.1
2	86-131	1200	6	4.8	65 ± 13	79 ± 5	76 ± 19	4.8 ± 2.9	4.1 ± 2.3
3	132-149	1600	6	6.4	65 ± 19	78 ± 9	90 ± 5	13.5 ± 4.1	10.4 ± 0.4
4	150-244	2000	6	8	82 ± 3	86 ± 8	93 ± 7	12.2 ± 7.3	6.3 ± 0.6
5	245-264	2400	6	9.6	69 ± 5	85 ± 11	92 ± 3	19.0 ± 7.8	15.9 ± 3.8
6	265-294	3000	6	12	78 ± 5	84 ± 7	81 ± 18	19.9 ± 5.2	18.8 ± 4.5
7	295-377	3500	6	14	76 ± 7	84 ± 5	91 ± 5	21.9 ± 6.8	20.4 ± 5.5
8	378-455	5000	6	20	75 ± 5	84 ± 3	87 ± 5	25.6 ± 12.1	22.6 ± 9.3
9	456-807	6000	6	24	60 ± 16	75 ± 11	80 ± 24	43.7 ± 13.2	40.1 ± 11.7
10	807-1004	6000	8.6	16.8	66 ± 3	75 ± 4	89 ± 7	N.D.	N.D.

N.D., not determined

Table S2. Physico-chemical characteristics of influent synthetic soy sauce wastewater at Phase 9

Parameter	Value
pH	7.86
CODcr (mg/L)	6,550
Volatile fatty acids (mgCOD/L)	
Acetate	1,224.3
Propionate	598.2
isobutyrate	103.9
n-butyrate	296.6
isovalerate	228.1
n-valerate	76.0
isocaproate	7.6
Amino acids (mgCOD/L)	
His	5.1
Ser	5.9
Arg	9.5
Gly	2.9
Asp	1.8
Glu	19.1
Thr	6.1
Ala	14.2
Pro	11.4
Cys	0.1
Lys	9.9
Tyr	2.3
Met	3.3
Val	13.0
Ile	13.2
Leu	20.5
Phe	13.5
Ammonia (mg/L)	4.7
Nitrite (mg/L)	N.D.
Nitrate (mg/L)	0.5
Sulfate (mg/L)	N.D.
Sulfite (mg/L)	896
Sodium sulfite (mg/L)	1,400

N.D., not detected

Table S3. Overview of sequencing

	Samples in triplicate		
	1	2	3
16S rRNA gene amplicon sequencing			
Total generated reads	32,497	33,626	32,481
OTU number	1,087	1,082	1,240
Metagenomic sequencing			
Total generated reads	8,933,746	10,328,001	8,576,690
Trimmed reads	6,848,602	7,852,694	6,678,214
Metatranscriptomic sequencing			
Total generated reads	21,891,528	22,040,758	20,827,923
Trimmed reads	21,233,560	21,332,631	20,157,236
Mapped reads	5,912,448	6,854,211	6,436,033

Table S4. Microbial community structures of granular sludge samples taken from up-flow anaerobic sludge blanket (UASB) reactor treating synthetic soy sauce processing wastewater based on 16S rRNA gene amplicon sequencing.

#OTU ID	Relative abuncance in triplicate			Average	STDEV	Phylogenetic affiliation						
	1	2	3			kingdom	Phylum	Class	Order	Family	Genus	Species
denovo4221	11.4134%	13.6085%	14.0205%	13.0141%	1.4015%	Archaea	Halobacterota	Methanosarcinia	Methanosarciniales	Methanosaetaceae	Methanosaeta	
denovo3208	7.9330%	12.8710%	6.6839%	9.1626%	3.2717%	Archaea	Euryarchaeota	Methanobacteria	Methanobacteriales	Methanobacteriaceae	Methanobacterium	archaeon_enrichment
denovo8447	9.2501%	11.4881%	3.5190%	8.0857%	4.1102%	Bacteria	Firmicutes	Desulfotomaculia	Desulfotomaculales	Desulfotomaculales	Pelotomaculum	uncultured_bacterium
denovo4217	6.6406%	6.2422%	7.6999%	6.8609%	0.7534%	Bacteria	Synergistota	Synergistia	Synergistales	Synergistaceae	uncultured	bacterium_enrichment
denovo1682	3.9911%	2.7984%	5.5571%	4.1156%	1.3835%	Archaea	Euryarchaeota	Methanobacteria	Methanobacteriales	Methanobacteriaceae	Methanobacterium	
denovo7624	3.3080%	2.4178%	4.7166%	3.4808%	1.1591%	Bacteria	Synergistota	Synergistia	Synergistales	Synergistaceae	Syner-01	
denovo1175	1.6648%	2.3910%	4.6366%	2.8974%	1.5493%	Bacteria	Firmicutes	Syntrophomonadia	Syntrophomonadales	Syntrophomonadaceae	Syntrophomonas	
denovo6767	1.3324%	1.1539%	2.8078%	1.7647%	0.9077%	Bacteria	Firmicutes	Clostridia	Peptococcales	Peptococcaceae	uncultured	uncultured_Peptococcaceae
denovo7510	1.4709%	2.3137%	1.2900%	1.6915%	0.5463%	Bacteria	Spirochaetota	Spirochaetia	Spirochaetales	Spirochaetaceae	uncultured	uncultured_Treponema
denovo50	1.0739%	1.3531%	2.5338%	1.6536%	0.7749%	Bacteria	Bacteroidota	Bacteroidia	Bacteroidales	Bacteroidaceae	Bacteroides	Bacteroides_graminisolvens
denovo4057	1.8617%	1.7516%	1.2900%	1.6344%	0.3033%	Bacteria	Bacteroidota	Bacteroidia	Bacteroidales	Dysgonomonadaceae	Petrimonas	
denovo2469	1.0678%	0.6959%	3.0264%	1.5967%	1.2520%	Archaea	Euryarchaeota	Methanobacteria	Methanobacteriales	Methanobacteriaceae	Methanobacterium	
denovo4383	1.0678%	0.7256%	2.2505%	1.3480%	0.8001%	Bacteria	Desulfobacterota	Desulfovibrionia	Desulfovibrionales	Desulfovibrionaceae	Desulfovibrio	
denovo8609	1.2647%	1.4602%	1.0806%	1.2685%	0.1898%	Bacteria	Firmicutes	Syntrophomonadia	Syntrophomonadales	Syntrophomonadaceae	Syntrophomonas	
denovo8608	1.3509%	1.4542%	0.9636%	1.2563%	0.2586%	Bacteria	Firmicutes	Bacilli	Lactobacillales	Enterococcaceae	Enterococcus	
denovo2903	1.6248%	1.3977%	0.6157%	1.2127%	0.5293%	Bacteria	Synergistota	Synergistia	Synergistales	Synergistaceae	Syner-01	uncultured_bacterium
denovo4515	2.9757%	0.4788%	0.0862%	1.1802%	1.5672%	Bacteria	Bacteroidota	Bacteroidia	Bacteroidales	Prolixibacteraceae	Mangrovibacterium	uncultured_bacterium
denovo4219	1.5971%	0.7197%	1.0960%	1.1376%	0.4402%	Bacteria	Bacteroidota	Bacteroidia	Bacteroidales	Bacteroidetes_vadinHA17	Bacteroidetes_vadinHA17	
denovo7936	1.7663%	0.7108%	0.7851%	1.0874%	0.5891%	Bacteria	Synergistota	Synergistia	Synergistales	Synergistaceae	Syner-01	uncultured_bacterium
denovo499	0.8308%	1.8557%	0.3171%	1.0012%	0.7833%	Bacteria	Firmicutes	Clostridia	Clostridia	Gracilibacteraceae	Gracilibacter	uncultured_Gracilibacter
denovo7509	1.5509%	1.0498%	0.3017%	0.9675%	0.6287%	Bacteria	Desulfobacterota	Syntrophobacteria	Syntrophobacterales	Syntrophobacteraceae	Syntrophobacter	
denovo8795	0.8001%	0.8595%	1.2346%	0.9647%	0.2356%	Bacteria	Firmicutes	Clostridia	Oscillospirales			
denovo6919	0.9539%	1.2907%	0.5819%	0.9422%	0.3545%	Bacteria	Proteobacteria	Gammaproteobacteria	Enterobacterales	Enterobacteriaceae		
denovo1773	0.6585%	0.9844%	0.9852%	0.8760%	0.1884%	Archaea	Halobacterota	Methanomicrobia	Methanomicrobiales	Methanospirillaceae	Methanospirillum	uncultured_bacterium
denovo6559	0.2708%	1.6029%	0.6435%	0.8391%	0.6873%	Bacteria	Bacteroidota	Bacteroidia	Bacteroidales	Williamwhitmaniaceae	Blvii28_wastewater-sludge_group	uncultured_bacterium
denovo46	0.6370%	0.6245%	1.0129%	0.7581%	0.2207%	Bacteria	Bacteroidota	Bacteroidia	Sphingobacteriales	ST-12K33	ST-12K33	
denovo7512	0.6739%	1.1747%	0.3140%	0.7209%	0.4322%	Bacteria	Synergistota	Synergistia	Synergistales	Synergistaceae	Thermovirga	
denovo6619	0.9232%	0.5621%	0.3787%	0.6213%	0.2770%	Bacteria	Bacteroidota	Bacteroidia	Bacteroidales	Bacteroidetes_vadinHA17	Bacteroidetes_vadinHA17	uncultured_bacterium
denovo3211	0.7108%	0.8059%	0.3233%	0.6133%	0.2557%	Bacteria	Chloroflexi	Anaerolineae	Anaerolineales	Anaerolineaceae	Flexilinea	
denovo4792	1.1632%	0.4728%	0.1108%	0.5823%	0.5346%	Bacteria	Chloroflexi	Anaerolineae	Anaerolineales	Anaerolineaceae	uncultured	
denovo4058	0.6862%	0.6007%	0.2986%	0.5285%	0.2036%	Bacteria	Thermotogota	Thermotogae	Kosmotogales	Kosmotogaceae	Mesotoga	
denovo5023	0.3693%	0.4580%	0.7574%	0.5282%	0.2034%	Bacteria	Firmicutes	Syntrophomonadia	Syntrophomonadales	Syntrophomonadaceae	Syntrophomonas	
denovo5359	0.6862%	0.5115%	0.3356%	0.5111%	0.1753%	Bacteria	Bacteroidota	Bacteroidia	Bacteroidales	Bacteroidetes_vadinHA17	Bacteroidetes_vadinHA17	
denovo45	0.1292%	0.3420%	1.0345%	0.5019%	0.4733%	Bacteria	Synergistota	Synergistia	Synergistales	Synergistaceae	Pyramidobacter	uncultured_bacterium
denovo4056	0.2062%	0.3926%	0.8682%	0.4890%	0.3414%	Bacteria	Firmicutes	Clostridia	Eubacteriales	Eubacteriaceae	Eubacterium	
denovo627	0.6739%	0.4372%	0.2678%	0.4596%	0.2040%	Bacteria	Firmicutes	Clostridia	Christensenellales	Christensenellaceae	Christensenellaceae_R-7_group	
denovo749	0.7478%	0.2795%	0.3448%	0.4574%	0.2536%	Bacteria	Desulfobacterota	Syntrophorhabdia	Syntrophorhabdales	Syntrophorhabdaceae	Syntrophorhabdus	uncultured_Syntrophorhabdus
denovo844	0.1108%	0.2349%	0.9883%	0.4447%	0.4749%	Bacteria	Firmicutes	Bacilli	Izemoplasmatales	Izemoplasmatales	Izemoplasmatales	uncultured_bacterium
denovo7419	0.7139%	0.2439%	0.2432%	0.4003%	0.2716%	Archaea	Euryarchaeota	Methanobacteria	Methanobacteriales	Methanobacteriaceae	Methanobrevibacter	Methanobrevibacter_arboriphilus
denovo2298	0.1569%	0.4193%	0.6188%	0.3984%	0.2317%	Archaea	Halobacterota	Methanosarcinia	Methanosarciniales	Methanosarcinaceae	Methanosarcina	
denovo51	0.5047%	0.4372%	0.2247%	0.3889%	0.1461%	Bacteria	Firmicutes	BRH-c20a	BRH-c20a	BRH-c20a	BRH-c20a	uncultured_bacterium
denovo3535	0.2400%	0.4015%	0.4710%	0.3708%	0.1185%	Bacteria	Firmicutes	Clostridia	Eubacteriales	Eubacteriaceae	Acetobacterium	
denovo2664	0.2985%	0.4580%	0.2894%	0.3486%	0.0948%	Bacteria	Firmicutes	Clostridia	Peptostreptococcales-Tissierellales	Peptostreptococcaceae	Peptoclostridium	uncultured_Tissierellia
denovo5082	0.3693%	0.2825%	0.3356%	0.3291%	0.0437%	Bacteria	Bacteroidota	Bacteroidia	Bacteroidales	Bacteroidetes_vadinHA17	Bacteroidetes_vadinHA17	
denovo4607	0.6493%	0.2171%	0.1201%	0.3288%	0.2817%	Bacteria	Firmicutes	Clostridia	Christensenellales	Christensenellaceae	Christensenellaceae_R-7_group	uncultured_Caloramator
denovo7206	0.3816%	0.4699%	0.1139%	0.3218%	0.1854%	Bacteria	Bacteroidota	Bacteroidia	Bacteroidales	Bacteroidetes_vadinHA17	Bacteroidetes_vadinHA17	
denovo6240	0.0985%	0.2855%	0.5788%	0.3209%	0.2421%	Bacteria	Firmicutes	Bacilli	Erysipelotrichales	Erysipelatoclostridiaceae	UCG-004	uncultured_bacterium
denovo3214	0.4031%	0.2022%	0.3325%	0.3126%	0.1019%	Bacteria	Bacteroidota	Bacteroidia	Bacteroidales	Rikenellaceae	DMER64	
denovo3827	0.2985%	0.2528%	0.3541%	0.3018%	0.0507%	Bacteria	Chloroflexi	Anaerolineae	Anaerolineales	Anaerolineaceae	Flexilinea	
denovo2384	0.2677%	0.1071%	0.5080%	0.2943%	0.2018%	Bacteria	Bacteroidota	Bacteroidia	Bacteroidales	Rikenellaceae	uncultured	uncultured_Bacteroidales
denovo7627	0.2000%	0.2320%	0.4403%	0.2907%	0.1305%	Bacteria	Bacteroidota	Bacteroidia	Bacteroidales	Dysgonomonadaceae	Proteiniphilum	
denovo1486	0.7816%	0.0416%	0.0369%	0.2867%	0.4286%	Bacteria	Firmicutes	Clostridia	Clostridiales	Clostridiaceae	Clostridium_sensu_stricto_13	
denovo7933	0.4031%	0.0833%	0.2678%	0.2514%	0.1606%	Bacteria	Desulfobacterota	Desulfovibrionia	Desulfovibrionales	Desulfovibrionaceae	Desulfovibrio	
denovo4943	0.2862%	0.2498%	0.2155%	0.2505%	0.0353%	Bacteria	Bacteroidota	Bacteroidia	Bacteroidales	Dysgonomonadaceae		
denovo6660	0.3016%	0.2082%	0.2371%	0.2489%	0.0478%	Bacteria	Bacteroidota	Bacteroidia	Sphingobacteriales	Lentimicrobiaceae	Lentimicrobium	
denovo1295	0.4523%	0.1665%	0.0800%	0.2330%	0.1948%	Bacteria	Chloroflexi	Anaerolineae	Anaerolineales	Anaerolineaceae	Flexilinea	
denovo4620	0.3354%	0.2111%	0.1324%	0.2263%	0.1024%	Bacteria	Firmicutes	Clostridia	Peptococcales	Peptococcaceae	uncultured	

denovo966	0.3908%	0.1487%	0.1262%	0.2219%	0.1467%	Bacteria	Firmicutes	Clostridia	Eubacteriales	Anaerofustaceae	Anaerofustis	uncultured_bacterium
denovo1681	0.2339%	0.1814%	0.2401%	0.2185%	0.0323%	Bacteria	Bacteroidota	Bacteroidia	Bacteroidales	Tannerellaceae	Macellibacteroides	
denovo7938	0.1508%	0.1665%	0.3202%	0.2125%	0.0936%	Bacteria	Bacteroidota	Kapabacteria	Kapabacteriales	Kapabacteriales	Kapabacteriales	uncultured_bacterium
denovo6385	0.4308%	0.0981%	0.0585%	0.1958%	0.2045%	Bacteria	Firmicutes	Clostridia	Peptostreptococcales-Tissierellales	Peptostreptococcaceae	Romboutsia	
denovo6891	0.0985%	0.2022%	0.2802%	0.1936%	0.0912%	Bacteria	Firmicutes	Clostridia	Christensenellales	Christensenellaceae	Christensenellaceae_R-7_group	
denovo8557	0.2616%	0.2022%	0.1139%	0.1926%	0.0743%	Bacteria	Fusobacteriota	Fusobacteriia	Fusobacteriales	Fusobacteriaceae	Fusobacterium	
denovo4464	0.4093%	0.1457%	0.0092%	0.1881%	0.2034%	Archaea	Halobacterota	Methanomicrobia	Methanomicrobiales	Methanospirillaceae	Methanospirillum	uncultured_archaeon
denovo5186	0.0708%	0.3479%	0.1447%	0.1878%	0.1435%	Bacteria	Chloroflexi	Anaerolineae	Anaerolineales	Anaerolineaceae	Pelolinea	uncultured_bacterium
denovo8448	0.0338%	0.0773%	0.4403%	0.1838%	0.2232%	Bacteria	Firmicutes	Clostridia	Oscillospirales	Ruminococcaceae	Anaerofilum	
denovo2443	0.2062%	0.1993%	0.1447%	0.1834%	0.0337%	Bacteria	Desulfobacterota	Syntrophia	Syntrophales	Syntrophaceae	Syntrophus	
denovo500	0.1939%	0.1725%	0.1385%	0.1683%	0.0279%	Bacteria	Chloroflexi	Anaerolineae	Anaerolineales	Anaerolineaceae		
denovo2879	0.2400%	0.0952%	0.1601%	0.1651%	0.0726%	Archaea	Thermoplasmatota	Thermoplasmata	Methanomassiliicoccales	Methanomethylophilaceae	RumEn_M2	uncultured_archaeon
denovo3943	0.0215%	0.0833%	0.3818%	0.1622%	0.1926%	Bacteria	Firmicutes	Syntrophomonadia	Syntrophomonadales	Syntrophomonadaceae	Syntrophomonas	
denovo6035	0.1692%	0.2260%	0.0677%	0.1543%	0.0802%	Bacteria	Caldatribacteriota	Caldatribacteriia	Caldatribacteriales	Caldatribacteriaceae	Candidatus_Caldatribacterium	Atribacteria_bacterium
denovo1868	0.1692%	0.1011%	0.1816%	0.1507%	0.0434%	Bacteria	Firmicutes	Clostridia	Oscillospirales	Ruminococcaceae	uncultured	
denovo6113	0.1139%	0.1457%	0.1816%	0.1471%	0.0339%	Bacteria	Firmicutes	Clostridia	Peptostreptococcales-Tissierellales	Anaerovoracaceae	Anaerovorax	uncultured_Anaerovorax
denovo6958	0.2431%	0.0862%	0.0954%	0.1416%	0.0880%	Bacteria	Firmicutes	Clostridia	Peptostreptococcales-Tissierellales	Anaerovoracaceae	uncultured	
denovo7392	0.0215%	0.0803%	0.3202%	0.1407%	0.1582%	Bacteria	Proteobacteria	Alphaproteobacteria	Rhodospirillales	Magnetospirillaceae	Telmatospirillum	
denovo5634	0.0554%	0.1219%	0.2217%	0.1330%	0.0837%	Bacteria	Firmicutes	Clostridia	Oscillospirales	Oscillospiraceae	Oscillibacter	
denovo3236	0.2677%	0.0625%	0.0677%	0.1326%	0.1170%	Bacteria	Firmicutes	Clostridia	Peptostreptococcales-Tissierellales	Peptostreptococcaceae	Terrisporobacter	
denovo2630	0.3016%	0.0565%	0.0216%	0.1265%	0.1526%	Bacteria	Caldatribacteriota	JS1	JS1	JS1	JS1	uncultured_organism
denovo7937	0.1262%	0.1665%	0.0739%	0.1222%	0.0465%	Bacteria	Desulfobacterota	Syntrophobacteria	Syntrophobacterales	Syntrophobacteraceae	Syntrophobacter	Syntrophobacter_sulfatireducens
denovo1994	0.0369%	0.0238%	0.3017%	0.1208%	0.1568%	Bacteria	Bacteroidota	Bacteroidia	Bacteroidales	Williamwhitmaniaceae	Blvii28_wastewater-sludge_group	uncultured_bacterium
denovo1876	0.2462%	0.0654%	0.0431%	0.1182%	0.1114%	Bacteria	Firmicutes	Clostridia	Clostridiales	Clostridiaceae	Clostridium_sensu_stricto_1	
denovo760	0.2246%	0.1011%	0.0216%	0.1158%	0.1023%	Bacteria	Firmicutes	Clostridia	Peptostreptococcales-Tissierellales	Sedimentibacteraceae	Sedimentibacter	
denovo1869	0.1323%	0.0773%	0.1170%	0.1089%	0.0284%	Bacteria	Firmicutes	Clostridia	Oscillospirales	Ruminococcaceae	uncultured	
denovo3462	0.0954%	0.0892%	0.1201%	0.1016%	0.0163%	Bacteria	Firmicutes	Clostridia	Christensenellales	Christensenellaceae		
denovo5313	0.1662%	0.0922%	0.0462%	0.1015%	0.0605%	Bacteria	Firmicutes	uncultured	uncultured	uncultured	uncultured	
denovo2317	0.0277%	0.1576%	0.1139%	0.0997%	0.0661%	Bacteria	Bacteroidota	Bacteroidia	Bacteroidales	Dysgonomonadaceae	Proteiniphilum	uncultured_Porphyromonadaceae
denovo5314	0.0523%	0.2052%	0.0400%	0.0992%	0.0920%	Bacteria	uncultured	uncultured	uncultured	uncultured	uncultured	uncultured_bacterium
denovo2899	0.0431%	0.0654%	0.1847%	0.0977%	0.0762%	Bacteria	Firmicutes	Clostridia	Christensenellales	Christensenellaceae	Christensenellaceae_R-7_group	uncultured_sludge
denovo4320	0.0523%	0.0743%	0.1663%	0.0976%	0.0604%	Bacteria	Firmicutes	Clostridia	Christensenellales	Christensenellaceae	Christensenellaceae_R-7_group	uncultured_soil
denovo2896	0.0892%	0.0773%	0.1262%	0.0976%	0.0255%	Bacteria	Chloroflexi	Anaerolineae	Anaerolineales	Anaerolineaceae	Flexilinea	
denovo9012	0.0585%	0.1011%	0.1231%	0.0942%	0.0329%	Bacteria	Firmicutes	Clostridia	Oscillospirales	uncultured	uncultured	uncultured_Clostridium
denovo4213	0.0338%	0.0862%	0.1601%	0.0934%	0.0634%	Bacteria	Firmicutes	Clostridia	Lachnospirales	Lachnospiraceae	Lachnoclostridium	
denovo4988	0.0400%	0.0862%	0.1509%	0.0924%	0.0557%	Bacteria	Firmicutes	Clostridia	Oscillospirales	Ruminococcaceae		
denovo5760	0.0523%	0.0625%	0.1539%	0.0896%	0.0560%	Bacteria	Firmicutes	Clostridia	Eubacteriales	Eubacteriaceae	uncultured	uncultured_bacterium
denovo3835	0.0369%	0.1517%	0.0800%	0.0895%	0.0580%	Bacteria	Firmicutes	Clostridia	Oscillospirales	Oscillospiraceae	Colidextribacter	uncultured_Firmicutes
denovo5735	0.2431%	0.0178%	0.0062%	0.0890%	0.1336%	Bacteria	Firmicutes	Syntrophomonadia	Syntrophomonadales	Syntrophomonadaceae		
denovo4993	0.0123%	0.0327%	0.2217%	0.0889%	0.1154%	Bacteria	Elusimicrobiota	Endomicrobia	Endomicrobiales	Endomicrobiaceae	Endomicrobium	
denovo2671	0.0492%	0.0833%	0.1324%	0.0883%	0.0418%	Bacteria	Firmicutes	Clostridia	Christensenellales	Christensenellaceae	Christensenellaceae_R-7_group	
denovo1998	0.0492%	0.0387%	0.1693%	0.0857%	0.0726%	Bacteria	Elusimicrobiota	Endomicrobia	Endomicrobiales	Endomicrobiaceae	Endomicrobium	
denovo3064	0.0615%	0.0862%	0.1078%	0.0852%	0.0231%	Bacteria	Proteobacteria	Gammaproteobacteria	Burkholderiales	Nitrosomonadaceae	Nitrosomonas	
denovo6662	0.1754%	0.0327%	0.0462%	0.0848%	0.0788%	Bacteria	Bacteroidota	Bacteroidia	Bacteroidales	SB-5	SB-5	
denovo7121	0.1508%	0.0684%	0.0339%	0.0843%	0.0601%	Bacteria	Firmicutes	Clostridia	Christensenellales	Christensenellaceae	uncultured	uncultured_bacterium
denovo8022	0.0800%	0.0862%	0.0862%	0.0842%	0.0036%	Bacteria	Firmicutes	Clostridia	Oscillospirales	Ruminococcaceae	uncultured	
denovo1078	0.0739%	0.0952%	0.0677%	0.0789%	0.0144%	Bacteria	Firmicutes	Clostridia	Peptostreptococcales-Tissierellales	Sedimentibacteraceae	Sedimentibacter	uncultured_Sedimentibacter
denovo1320	0.0985%	0.0654%	0.0647%	0.0762%	0.0193%	Bacteria	Bacteroidota	Bacteroidia	Bacteroidales	Dysgonomonadaceae	Proteiniphilum	uncultured_Ruminobacillus
denovo7521	0.0646%	0.1041%	0.0585%	0.0757%	0.0247%	Bacteria	Chloroflexi	Anaerolineae	Anaerolineales	Anaerolineaceae		
denovo998	0.0769%	0.1190%	0.0277%	0.0745%	0.0457%	Bacteria	Firmicutes	Clostridia	Christensenellales	Christensenellaceae	Christensenellaceae_R-7_group	
denovo5859	0.0554%	0.0416%	0.1262%	0.0744%	0.0454%	Bacteria	Verrucomicrobiota	Verrucomicrobiae	Pedosphaerales	Pedosphaeraceae	DEV114	bacterium_enrichment
denovo2366	0.0400%	0.0535%	0.1262%	0.0733%	0.0464%	Bacteria	Synergistota	Synergistia	Synergistales	Synergistaceae	Cloacibacillus	Cloacibacillus_evryensis
denovo2271	0.0892%	0.0327%	0.0862%	0.0694%	0.0318%	Bacteria	Firmicutes	Clostridia	Oscillospirales	Ruminococcaceae	uncultured	uncultured_Firmicutes
denovo5456	0.0523%	0.0743%	0.0770%	0.0679%	0.0135%	Bacteria	Firmicutes	Syntrophomonadia	Syntrophomonadales	Syntrophomonadaceae		
denovo757	0.0308%	0.0446%	0.1231%	0.0662%	0.0498%	Bacteria	Firmicutes	Clostridia	Oscillospirales	Ruminococcaceae		
denovo1619	0.1662%	0.0268%	0.0031%	0.0653%	0.0881%	Bacteria	Caldatribacteriota	JS1	JS1	JS1	JS1	uncultured_organism
denovo536	0.1539%	0.0357%	0.0031%	0.0642%	0.0793%	Bacteria	Cloacimonadota	Cloacimonadia	Cloacimonadales	Cloacimonadaceae	LNR_A2-18	uncultured_bacterium
denovo7287	0.1262%	0.0446%	0.0216%	0.0641%	0.0550%	Bacteria	Bacteroidota	Bacteroidia	Bacteroidales			
denovo4381	0.0246%	0.0506%	0.1139%	0.0630%	0.0459%	Bacteria	Firmicutes	Negativicutes	Acidaminococcales	Acidaminococcaceae	Acidaminococcus	
denovo6976	0.0492%	0.0714%	0.0677%	0.0628%	0.0119%	Bacteria	Firmicutes	Clostridia	Christensenellales	Christensenellaceae	Christensenellaceae_R-7_group	uncultured_Lachnospiraceae

denovo3156	0.0646%	0.0327%	0.0862%	0.0612%	0.0269%	Bacteria	Firmicutes	Clostridia	Peptococcales	Peptococcaceae	uncultured	
denovo4060	0.0308%	0.0595%	0.0893%	0.0598%	0.0293%	Bacteria	Firmicutes	Clostridia	Peptostreptococcales-Tissierellales	Anaerovoracaceae		
denovo8879	0.0123%	0.0862%	0.0800%	0.0595%	0.0410%	Bacteria	Bacteroidota	Bacteroidia	Bacteroidales	Tannerellaceae	Parabacteroides	Blattella_germanica
denovo5442	0.0769%	0.0773%	0.0216%	0.0586%	0.0321%	Bacteria	Bacteroidota	Bacteroidia	Bacteroidales	Bacteroidetes_vadinHA17	Bacteroidetes_vadinHA17	
denovo5891	0.1385%	0.0149%	0.0216%	0.0583%	0.0695%	Bacteria	Firmicutes	Clostridia	Christensenellales	Christensenellaceae	Christensenellaceae_R-7_group	
denovo8285	0.0985%	0.0595%	0.0154%	0.0578%	0.0416%	Bacteria	Proteobacteria	Gammaproteobacteria	Burkholderiales	Aquaspirillaceae	Microvirgula	Microvirgula_aerodenitrificans
denovo1426	0.0615%	0.0416%	0.0677%	0.0570%	0.0136%	Bacteria	Bacteroidota	Bacteroidia	Bacteroidales	Rikenellaceae	Anaerocella	uncultured_bacterium
denovo5411	0.0308%	0.0803%	0.0585%	0.0565%	0.0248%	Bacteria	Firmicutes	Clostridia	Lachnospirales	Lachnospiraceae	Lachnoclostridium	
denovo2897	0.0338%	0.0476%	0.0862%	0.0559%	0.0271%	Bacteria	Firmicutes	Clostridia	Oscillospirales	Ruminococcaceae	Harryflintia	
denovo7836	0.0862%	0.0357%	0.0431%	0.0550%	0.0273%	Bacteria	Desulfobacterota	Desulfobacteria	Desulfobacterales	Desulfosarcinaceae		
denovo2981	0.0400%	0.0416%	0.0831%	0.0549%	0.0244%	Bacteria	Spirochaetota	Spirochaetia	Spirochaetales	Spirochaetaceae	Treponema	metagenome
denovo1779	0.0431%	0.0446%	0.0739%	0.0539%	0.0174%	Bacteria	Firmicutes	Clostridia	Oscillospirales	Ruminococcaceae	uncultured	
denovo9062	0.0523%	0.0952%	0.0123%	0.0533%	0.0414%	Bacteria	Firmicutes	Clostridia	Clostridiales	Clostridiaceae	Clostridium_sensu_stricto_1	
denovo2902	0.0369%	0.0506%	0.0677%	0.0517%	0.0154%	Bacteria	Firmicutes	Clostridia	Oscillospirales	Oscillospiraceae		
denovo5779	0.0646%	0.0268%	0.0616%	0.0510%	0.0210%	Bacteria	Chloroflexi	Anaerolineae	Anaerolineales	Anaerolineaceae	Anaerolineaceae_UCG-001	Anaerolineales_bacterium
denovo1109	0.0677%	0.0654%	0.0185%	0.0505%	0.0278%	Bacteria	Synergistota	Synergistia	Synergistales	Synergistaceae	uncultured	uncultured_bacterium
denovo8719	0.0154%	0.0952%	0.0400%	0.0502%	0.0408%	Bacteria	Spirochaetota	Spirochaetia	Spirochaetales	Spirochaetaceae	uncultured	uncultured_prokaryote
denovo4839	0.0739%	0.0327%	0.0431%	0.0499%	0.0214%	Bacteria	Firmicutes	Clostridia	Peptostreptococcales-Tissierellales	Peptostreptococcaceae	Acetoanaerobium	
denovo1197	0.0185%	0.0387%	0.0924%	0.0498%	0.0382%	Bacteria	Verrucomicrobiota	Verrucomicrobiae	Opitutales	Puniceicoccaceae	Coraliomargarita	Coraliomargarita_sp.
denovo1640	0.0615%	0.0476%	0.0369%	0.0487%	0.0123%	Bacteria	Firmicutes	Clostridia	Oscillospirales	Oscillospirales	Hydrogenoanaerobacterium	uncultured_bacterium
denovo7614	0.0462%	0.0625%	0.0369%	0.0485%	0.0129%	Bacteria	Firmicutes	Bacilli	Erysipelotrichales	Erysipelotrichaceae		
denovo3441	0.0246%	0.0476%	0.0677%	0.0466%	0.0216%	Bacteria	Firmicutes	Clostridia	Christensenellales	Christensenellaceae	Christensenellaceae_R-7_group	
denovo4130	0.0431%	0.0684%	0.0277%	0.0464%	0.0205%	Bacteria	Firmicutes	uncultured	uncultured	uncultured	uncultured	uncultured_Firmicutes
denovo3618	0.0708%	0.0446%	0.0216%	0.0456%	0.0246%	Bacteria	Armatimonadota	uncultured	uncultured	uncultured	uncultured	uncultured_Armatimonadetes
denovo5297	0.0215%	0.0238%	0.0893%	0.0449%	0.0385%	Bacteria	Firmicutes	Clostridia	Christensenellales	Christensenellaceae	uncultured	
denovo6162	0.0585%	0.0387%	0.0369%	0.0447%	0.0120%	Bacteria	Planctomycetota	vadinHA49	vadinHA49	vadinHA49	vadinHA49	uncultured_Planctomycetaceae
denovo5188	0.0431%	0.0565%	0.0339%	0.0445%	0.0114%	Bacteria	Firmicutes	Clostridia	Oscillospirales	Oscillospiraceae		
denovo348	0.0554%	0.0208%	0.0554%	0.0439%	0.0200%	Bacteria	Firmicutes	Clostridia	Oscillospirales	Ruminococcaceae	uncultured	uncultured_Clostridiales
denovo6896	0.0462%	0.0446%	0.0369%	0.0426%	0.0049%	Bacteria	Proteobacteria	Gammaproteobacteria	Enterobacterales	Morganellaceae	Morganella	
denovo635	0.0062%	0.0119%	0.1047%	0.0409%	0.0553%	Bacteria	Firmicutes	Syntrophomonadia	Syntrophomonadales	Syntrophomonadaceae	Pelospora	
denovo4234	0.0031%	0.0119%	0.1016%	0.0389%	0.0545%	Bacteria	Firmicutes	Clostridia	Oscillospirales	Oscillospiraceae	Colidextribacter	
denovo5497	0.0862%	0.0119%	0.0185%	0.0388%	0.0411%	Bacteria	Actinobacteriota	Coriobacteriia	Coriobacteriales	uncultured	uncultured	uncultured_bacterium
denovo7222	0.0062%	0.0178%	0.0924%	0.0388%	0.0468%	Bacteria	Bacteroidota	Bacteroidia	Bacteroidales	Paludibacteraceae	uncultured	
denovo797	0.0492%	0.0238%	0.0431%	0.0387%	0.0133%	Bacteria	Bacteroidota	Bacteroidia	Bacteroidales	Dysgonomonadaceae	uncultured	uncultured_soil
denovo8012	0.0585%	0.0268%	0.0308%	0.0387%	0.0173%	Bacteria	Actinobacteriota	Actinobacteria	Actinomycetales	Actinomycetaceae	uncultured	uncultured_bacterium
denovo6365	0.0308%	0.0416%	0.0431%	0.0385%	0.0067%	Bacteria	Firmicutes	Clostridia	Clostridia	Hungateiclostridiaceae	uncultured	uncultured_bacterium
denovo956	0.0554%	0.0446%	0.0154%	0.0385%	0.0207%	Bacteria	Chloroflexi	Anaerolineae	Anaerolineales	Anaerolineaceae		
denovo2231	0.0400%	0.0476%	0.0277%	0.0384%	0.0100%	Bacteria	Actinobacteriota	Coriobacteriia	Coriobacteriales	Eggerthellaceae	Gordonibacter	uncultured_bacterium
denovo6892	0.0739%	0.0149%	0.0246%	0.0378%	0.0316%	Bacteria	Actinobacteriota	Coriobacteriia	Coriobacteriales	Atopobiaceae	Olsenella	
denovo7277	0.0092%	0.0208%	0.0831%	0.0377%	0.0397%	Bacteria	Firmicutes	Clostridia	Christensenellales	Christensenellaceae	Christensenellaceae_R-7_group	uncultured_Christensenellaceae
denovo1422	0.0185%	0.0238%	0.0708%	0.0377%	0.0288%	Bacteria	Synergistota	Synergistia	Synergistales	Synergistaceae	uncultured	
denovo1423	0.0246%	0.0357%	0.0523%	0.0375%	0.0140%	Bacteria	Firmicutes	uncultured	uncultured	uncultured	uncultured	uncultured_bacterium
denovo1045	0.0400%	0.0535%	0.0185%	0.0373%	0.0177%	Bacteria	Firmicutes	Clostridia				
denovo4264	0.0923%	0.0089%	0.0062%	0.0358%	0.0490%	Bacteria	Armatimonadota	uncultured	uncultured	uncultured	uncultured	
denovo2979	0.0092%	0.0297%	0.0677%	0.0356%	0.0297%	Archaea	Euryarchaeota	Methanobacteria	Methanobacteriales	Methanobacteriaceae	Methanobacterium	
denovo3926	0.0338%	0.0446%	0.0277%	0.0354%	0.0086%	Bacteria	Bacteroidota	Bacteroidia	Bacteroidales	Marinilabiliaceae	Mangroviflexus	uncultured_bacterium
denovo1111	0.0492%	0.0089%	0.0462%	0.0348%	0.0224%	Bacteria	Spirochaetota	Spirochaetia	Spirochaetales	Spirochaetaceae	Spirochaeta	uncultured_bacterium
denovo1685	0.0062%	0.0178%	0.0800%	0.0347%	0.0397%	Bacteria	Firmicutes	Clostridia	Peptococcales	Peptococcaceae	uncultured	uncultured_Dehalobacterium
denovo6759	0.0646%	0.0268%	0.0123%	0.0346%	0.0270%	Bacteria	Firmicutes	Bacilli	Lactobacillales	Carnobacteriaceae	Trichococcus	
denovo8724	0.0523%	0.0387%	0.0123%	0.0344%	0.0203%	Bacteria	Firmicutes	Clostridia	Christensenellales	Christensenellaceae		
denovo2415	0.0154%	0.0476%	0.0400%	0.0343%	0.0168%	Bacteria	Firmicutes	Desulfitobacteriia	Desulfitobacteriales	Syntrophobotulaceae	Syntrophobotulus	Syntrophobotulus_glycolicus
denovo2398	0.0492%	0.0238%	0.0277%	0.0336%	0.0137%	Bacteria	Firmicutes	Clostridia	Christensenellales	Christensenellaceae	uncultured	
denovo1702	0.0092%	0.0327%	0.0585%	0.0335%	0.0246%	Bacteria	Firmicutes	Clostridia	Peptostreptococcales-Tissierellales	Anaerovoracaceae		
denovo512	0.0246%	0.0327%	0.0431%	0.0335%	0.0093%	Bacteria	Firmicutes	Syntrophomonadia	Syntrophomonadales	Syntrophomonadaceae	Syntrophomonas	anaerobic_digester
denovo5204	0.0185%	0.0059%	0.0739%	0.0328%	0.0362%	Bacteria	Bacteroidota	Bacteroidia	Bacteroidales	Bacteroidaceae	Bacteroides	
denovo3172	0.0308%	0.0119%	0.0554%	0.0327%	0.0218%	Bacteria	Firmicutes	Clostridia	Oscillospirales	Ethanoligenenaceae	uncultured	
denovo1419	0.0308%	0.0149%	0.0523%	0.0327%	0.0188%	Bacteria	Bacteroidota	Bacteroidia	Bacteroidales	Dysgonomonadaceae	Dysgonomonadaceae	uncultured_bacterium
denovo5454	0.0769%	0.0149%	0.0062%	0.0327%	0.0386%	Bacteria	WS1	WS1	WS1	WS1	WS1	uncultured_bacterium
denovo3750	0.0523%	0.0327%	0.0123%	0.0324%	0.0200%	Bacteria	Bacteroidota	Ignavibacteria	Ignavibacteriales	PHOS-HE36	PHOS-HE36	uncultured_bacterium
denovo1452	0.0831%	0.0089%	0.0031%	0.0317%	0.0446%	Bacteria	Desulfobacterota	Syntrophia	Syntrophales	uncultured	uncultured	

denovo4853	0.0092%	0.0238%	0.0616%	0.0315%	0.0270%	Bacteria	Bacteroidota	Bacteroidia	Bacteroidales	Dysgonomonadaceae	Proteiniphilum	
denovo5632	0.0246%	0.0238%	0.0462%	0.0315%	0.0127%	Bacteria	Firmicutes	Clostridia	Oscillospirales	Ruminococcaceae	uncultured	
denovo2514	0.0062%	0.0119%	0.0739%	0.0306%	0.0376%	Bacteria	Desulfobacterota	Desulfuromonadia	Geobacterales	Geobacteraceae	uncultured	anaerobic_syntrophic
denovo7477	0.0246%	0.0149%	0.0523%	0.0306%	0.0194%	Bacteria	Firmicutes	Clostridia	Christensenellales	Christensenellaceae	Christensenellaceae_R-7_group	
denovo3500	0.0154%	0.0208%	0.0554%	0.0305%	0.0217%	Bacteria	Firmicutes	Clostridia	Oscillospirales	Ruminococcaceae	uncultured	
denovo5534	0.0215%	0.0268%	0.0431%	0.0305%	0.0112%	Unassigned						
denovo6335	0.0185%	0.0387%	0.0339%	0.0303%	0.0106%	Bacteria	Chloroflexi	Anaerolineae	Anaerolineales	Anaerolineaceae		
denovo7628	0.0123%	0.0416%	0.0369%	0.0303%	0.0158%	Bacteria	Bacteroidota	Bacteroidia	Bacteroidales	Tannerellaceae		
denovo594	0.0769%	0.0089%	0.0031%	0.0296%	0.0411%	Archaea	Halobacterota	Methanomicrobia	Methanomicrobiales	Methanoregulaceae	Methanolinea	
denovo7104	0.0492%	0.0387%	0.0000%	0.0293%	0.0259%	Bacteria	Chloroflexi	Anaerolineae	Anaerolineales	Anaerolineaceae	Pelolinea	uncultured_bacterium
denovo4354	0.0185%	0.0030%	0.0647%	0.0287%	0.0321%	Bacteria	Thermotogota	Thermotogae	Petrotogales	Petrotogaceae	AUTHM297	uncultured_bacterium
denovo6471	0.0000%	0.0089%	0.0770%	0.0286%	0.0421%	Bacteria	Firmicutes	Syntrophomonadia	Syntrophomonadales	Syntrophomonadaceae	Syntrophomonas	uncultured_Syntrophomonas
denovo6831	0.0400%	0.0238%	0.0216%	0.0284%	0.0101%	Bacteria	Sumerlaeota	Sumerlaeia	Sumerlaeales	Sumerlaeaceae	Sumerlaea	uncultured_bacterium
denovo6029	0.0246%	0.0357%	0.0246%	0.0283%	0.0064%	Bacteria	Firmicutes	Clostridia	Lachnospirales	Lachnospiraceae	Lachnoclostridium	Faecalicatena_orotica
denovo3160	0.0400%	0.0357%	0.0092%	0.0283%	0.0167%	Bacteria	Synergistota	Synergistia	Synergistales	Synergistaceae		
denovo2562	0.0246%	0.0416%	0.0185%	0.0282%	0.0120%	Bacteria	Patescibacteria	Parcubacteria				
denovo6454	0.0246%	0.0089%	0.0493%	0.0276%	0.0203%	Bacteria	Desulfobacterota	Desulfuromonadia	Geobacterales	Geobacteraceae	Geobacter	
denovo6532	0.0677%	0.0089%	0.0062%	0.0276%	0.0348%	Bacteria	Bacteroidota	Bacteroidia	Bacteroidales	Bacteroidetes_vadinHA17	Bacteroidetes_vadinHA17	
denovo1042	0.0092%	0.0208%	0.0523%	0.0275%	0.0223%	Bacteria	Armatimonadota	uncultured	uncultured	uncultured	uncultured	
denovo1135	0.0215%	0.0268%	0.0339%	0.0274%	0.0062%	Bacteria	Firmicutes	Clostridia	Clostridia	Hungateiclostridiaceae	Ercella	
denovo5387	0.0554%	0.0268%	0.0000%	0.0274%	0.0277%	Archaea	Euryarchaeota	Thermococci	Methanofastidiosales	Methanofastidiosaceae	Candidatus_Methanofastidiosum	uncultured_euryarchaeote
denovo8913	0.0154%	0.0089%	0.0554%	0.0266%	0.0252%	Bacteria	Desulfobacterota	Desulfovibrionia	Desulfovibrionales	Desulfovibrionaceae		
denovo4570	0.0215%	0.0149%	0.0431%	0.0265%	0.0148%	Bacteria	Firmicutes	Clostridia	Eubacteriales	Anaerofustaceae	Anaerofustis	uncultured_bacterium
denovo6614	0.0554%	0.0238%	0.0000%	0.0264%	0.0278%	Bacteria	Bacteroidota	Bacteroidia	Bacteroidales	Bacteroidetes_vadinHA17	Bacteroidetes_vadinHA17	
denovo1951	0.0154%	0.0357%	0.0277%	0.0263%	0.0102%	Bacteria	Bacteroidota	Bacteroidia	Bacteroidales	Dysgonomonadaceae	uncultured	metagenome
denovo9007	0.0308%	0.0208%	0.0246%	0.0254%	0.0050%	Bacteria	Firmicutes	Clostridia	Lachnospirales	Lachnospiraceae		
denovo4642	0.0369%	0.0208%	0.0185%	0.0254%	0.0100%	Bacteria	Firmicutes	Bacilli	Lactobacillales	Streptococcaceae	Lactococcus	
denovo8274	0.0338%	0.0238%	0.0185%	0.0254%	0.0078%	Bacteria	Bacteroidota	Bacteroidia	Bacteroidales	Dysgonomonadaceae	Dysgonomonas	
denovo9034	0.0369%	0.0238%	0.0154%	0.0254%	0.0109%	Bacteria	Firmicutes	Clostridia	Clostridia	Hungateiclostridiaceae	uncultured	
denovo2279	0.0246%	0.0268%	0.0246%	0.0253%	0.0012%	Bacteria	Bacteroidota	Bacteroidia	Bacteroidales	Rikenellaceae	uncultured	bacterium_enrichment
denovo5493	0.0031%	0.0119%	0.0585%	0.0245%	0.0298%	Bacteria	Firmicutes	Clostridia	Lachnospirales	Lachnospiraceae	Lachnoclostridium	
denovo8910	0.0215%	0.0238%	0.0277%	0.0243%	0.0031%	Bacteria	Firmicutes	Clostridia	Christensenellales	Christensenellaceae	Christensenellaceae_R-7_group	uncultured_Bacteroidetes
denovo9023	0.0246%	0.0238%	0.0246%	0.0243%	0.0005%	Bacteria	Desulfobacterota	Desulfovibrionia	Desulfovibrionales	Desulfovibrionaceae	Desulfovibrio	
denovo935	0.0369%	0.0268%	0.0092%	0.0243%	0.0140%	Bacteria	Firmicutes	Clostridia	Peptostreptococcales-Tissierellales	Anaerovoracaceae	Anaerovorax	
denovo1781	0.0246%	0.0297%	0.0185%	0.0243%	0.0056%	Bacteria	Firmicutes	Clostridia	Christensenellales	Christensenellaceae	uncultured	
denovo997	0.0154%	0.0327%	0.0246%	0.0242%	0.0087%	Bacteria	Spirochaetota	Spirochaetia	Spirochaetales	Spirochaetaceae	Sediminispirochaeta	uncultured_bacterium
denovo6781	0.0185%	0.0357%	0.0185%	0.0242%	0.0099%	Bacteria	Firmicutes	Clostridia	Christensenellales	Christensenellaceae	Christensenellaceae_R-7_group	
denovo6254	0.0154%	0.0416%	0.0154%	0.0241%	0.0152%	Bacteria	Firmicutes	Clostridia	Peptostreptococcales-Tissierellales	Sedimentibacteraceae	Sedimentibacter	
denovo395	0.0523%	0.0149%	0.0031%	0.0234%	0.0257%	Bacteria	Firmicutes	Clostridia	Peptostreptococcales-Tissierellales	Sedimentibacteraceae	Sedimentibacter	
denovo5402	0.0400%	0.0178%	0.0123%	0.0234%	0.0147%	Bacteria	Bacteroidota	Bacteroidia	Bacteroidales	Bacteroidetes_vadinHA17	Bacteroidetes_vadinHA17	
denovo1132	0.0523%	0.0178%	0.0000%	0.0234%	0.0266%	Bacteria	Planctomycetota	vadinHA49	vadinHA49	vadinHA49	vadinHA49	uncultured_Planctomycetaceae
denovo963	0.0154%	0.0297%	0.0246%	0.0233%	0.0073%	Bacteria	Bacteroidota	Bacteroidia	Bacteroidales	Bacteroidaceae	Bacteroides	
denovo9019	0.0000%	0.0000%	0.0677%	0.0226%	0.0391%	Bacteria	Bacteroidota	Bacteroidia	Bacteroidales	Rikenellaceae	DMER64	bacterium_enrichment
denovo5914	0.0092%	0.0059%	0.0523%	0.0225%	0.0259%	Bacteria	Elusimicrobiota	Endomicrobia	Endomicrobiales	Endomicrobiaceae	Endomicrobium	uncultured_Endomicrobia
denovo8138	0.0123%	0.0208%	0.0339%	0.0223%	0.0109%	Unassigned						
denovo3861	0.0154%	0.0357%	0.0154%	0.0222%	0.0117%	Bacteria	Firmicutes	Clostridia	Christensenellales	Christensenellaceae	Catabacter	
denovo5976	0.0308%	0.0149%	0.0185%	0.0214%	0.0083%	Bacteria	Firmicutes	Clostridia	Lachnospirales	Lachnospiraceae	Anaerostignum	
denovo2764	0.0338%	0.0149%	0.0154%	0.0214%	0.0108%	Bacteria	Firmicutes	Clostridia	Oscillospirales	Ruminococcaceae		
denovo1308	0.0215%	0.0178%	0.0246%	0.0213%	0.0034%	Bacteria	Planctomycetota	vadinHA49	vadinHA49	vadinHA49	vadinHA49	uncultured_planctomycete
denovo1501	0.0277%	0.0178%	0.0185%	0.0213%	0.0055%	Bacteria	Bacteroidota	Bacteroidia	Bacteroidales	Bacteroidaceae	Bacteroides	
denovo7447	0.0277%	0.0208%	0.0154%	0.0213%	0.0062%	Bacteria	Actinobacteriota	Actinobacteria	Actinomycetales	Actinomycetaceae	Actinomyces	uncultured_Actinomycetales
denovo739	0.0215%	0.0238%	0.0185%	0.0213%	0.0027%	Bacteria	Firmicutes	Clostridia	Christensenellales	Christensenellaceae	Christensenellaceae_R-7_group	gut_metagenome
denovo3850	0.0338%	0.0238%	0.0062%	0.0213%	0.0140%	Bacteria	Patescibacteria	Microgenomatia	Candidatus_Shapirobacteria	Candidatus_Shapirobacteria	Candidatus_Shapirobacteria	uncultured_bacterium
denovo449	0.0585%	0.0000%	0.0031%	0.0205%	0.0329%	Bacteria						
denovo5500	0.0000%	0.0059%	0.0554%	0.0205%	0.0304%	Bacteria						
denovo8750	0.0123%	0.0119%	0.0369%	0.0204%	0.0143%	Bacteria	Desulfobacterota	Desulfuromonadia	Geobacterales	Geobacteraceae	Geobacter	
denovo881	0.0431%	0.0119%	0.0062%	0.0204%	0.0199%	Bacteria	Firmicutes	Syntrophomonadia	Syntrophomonadales	Syntrophomonadaceae	Syntrophomonas	
denovo612	0.0123%	0.0149%	0.0339%	0.0203%	0.0118%	Bacteria	Spirochaetota	Leptospirae	Leptospirales	Leptospiraceae	RBG-16-49-21	
denovo3370	0.0246%	0.0149%	0.0216%	0.0203%	0.0050%	Bacteria	Spirochaetota	MVP-15	MVP-15	MVP-15	MVP-15	
denovo1990	0.0369%	0.0178%	0.0062%	0.0203%	0.0155%	Bacteria	Firmicutes	Clostridia	Peptostreptococcales-Tissierellales	Anaerovoracaceae	Anaerovorax	uncultured_Firmicutes

denovo6242	0.0277%	0.0208%	0.0123%	0.0203%	0.0077%	Bacteria	Desulfobacterota	Syntrophia	Syntrophales	Syntrophaceae	Syntrophus	
denovo2282	0.0123%	0.0238%	0.0246%	0.0202%	0.0069%	Bacteria	Bacteroidota	Bacteroidia	Bacteroidales	Dysgonomonadaceae	Dysgonomonas	uncultured_Porphyromonadaceae
denovo5024	0.0215%	0.0238%	0.0154%	0.0202%	0.0043%	Bacteria	Firmicutes	uncultured	uncultured	uncultured	uncultured	
denovo5920	0.0338%	0.0238%	0.0031%	0.0202%	0.0157%	Bacteria	Actinobacteriota	Thermoleophilia	Gaiellales	uncultured	uncultured	metagenome
denovo6006	0.0062%	0.0297%	0.0246%	0.0202%	0.0124%	Bacteria	Firmicutes	Clostridia	Clostridia	Gracilibacteraceae		
denovo5766	0.0554%	0.0000%	0.0031%	0.0195%	0.0311%	Bacteria	Actinobacteriota	Thermoleophilia	Gaiellales	uncultured	uncultured	
denovo7124	0.0554%	0.0000%	0.0031%	0.0195%	0.0311%	Bacteria	Firmicutes	Clostridia	Christensenellales	Christensenellaceae	Christensenellaceae_R-7_group	
denovo4394	0.0215%	0.0059%	0.0308%	0.0194%	0.0126%	Bacteria	Bacteroidota	Bacteroidia	Bacteroidales	Rikenellaceae	Bact-08	
denovo1512	0.0092%	0.0089%	0.0400%	0.0194%	0.0179%	Bacteria	Spirochaetota	Spirochaetia	Spirochaetales	Spirochaetaceae	Treponema	
denovo5327	0.0123%	0.0089%	0.0369%	0.0194%	0.0153%	Archaea	Euryarchaeota	Methanobacteria	Methanobacteriales	Methanobacteriaceae	Methanobacterium	
denovo6363	0.0492%	0.0089%	0.0000%	0.0194%	0.0262%	Bacteria	Cloacimonadota	Cloacimonadia	Cloacimonadales	Cloacimonadaceae	LNR_A2-18	uncultured_bacterium
denovo4401	0.0185%	0.0149%	0.0246%	0.0193%	0.0049%	Bacteria	Desulfobacterota	Desulfovibrionia	Desulfovibrionales	Desulfovibrionaceae	uncultured	uncultured_delta
denovo8638	0.0000%	0.0208%	0.0369%	0.0193%	0.0185%	Bacteria	Firmicutes	Clostridia	Lachnospirales	Lachnospiraceae	Lachnoclostridium	
denovo8180	0.0092%	0.0208%	0.0277%	0.0193%	0.0093%	Archaea	Halobacterota	Methanomicrobia	Methanomicrobiales	Methanocorpusculaceae	Methanocorpusculum	uncultured_Methanocorpusculum
denovo7771	0.0031%	0.0238%	0.0308%	0.0192%	0.0144%	Bacteria	Chloroflexi					
denovo4006	0.0215%	0.0238%	0.0123%	0.0192%	0.0061%	Bacteria	Firmicutes	Clostridia	Peptococcales	Peptococcaceae	uncultured	
denovo6145	0.0185%	0.0059%	0.0308%	0.0184%	0.0124%	Bacteria	Bacteroidota	Bacteroidia	Bacteroidales	Rikenellaceae	Anaerocella	uncultured_Bacteroidales
denovo1878	0.0123%	0.0089%	0.0339%	0.0184%	0.0135%	Bacteria	Bacteroidota	Bacteroidia	Bacteroidales			
denovo2537	0.0123%	0.0089%	0.0339%	0.0184%	0.0135%	Bacteria	Firmicutes	uncultured	uncultured	uncultured	uncultured	bacterium_MB7-1
denovo4396	0.0123%	0.0089%	0.0339%	0.0184%	0.0135%	Archaea	Thermoplasmatota	Thermoplasmata	Methanomassiliicoccales	Methanomethylophilaceae	Methanomethylophilaceae	uncultured_euryarchaeote
denovo7003	0.0277%	0.0178%	0.0092%	0.0183%	0.0092%	Bacteria	Firmicutes	Clostridia				
denovo7188	0.0308%	0.0208%	0.0031%	0.0182%	0.0140%	Bacteria	Chloroflexi	Anaerolineae	Anaerolineales	Anaerolineaceae		
denovo3025	0.0062%	0.0268%	0.0216%	0.0182%	0.0107%	Bacteria	Firmicutes	Clostridia	Clostridia_vadinBB60_group	Clostridia_vadinBB60_group	Clostridia_vadinBB60_group	
denovo2268	0.0400%	0.0030%	0.0092%	0.0174%	0.0198%	Bacteria	Spirochaetota	Spirochaetia	Spirochaetales	Spirochaetaceae	Spirochaeta	
denovo3443	0.0215%	0.0119%	0.0185%	0.0173%	0.0049%	Bacteria	Firmicutes	Clostridia	Lachnospirales	Lachnospiraceae		
denovo2950	0.0062%	0.0149%	0.0308%	0.0173%	0.0125%	Bacteria	Firmicutes	Clostridia	Oscillospirales	Ruminococcaceae	uncultured	uncultured_Firmicutes
denovo5761	0.0185%	0.0149%	0.0185%	0.0173%	0.0021%	Bacteria	Desulfobacterota	Syntrophia	Syntrophales	Syntrophaceae	Syntrophus	
denovo7659	0.0185%	0.0149%	0.0185%	0.0173%	0.0021%	Eukaryota						
denovo1528	0.0246%	0.0178%	0.0092%	0.0172%	0.0077%	Bacteria	Firmicutes	Clostridia	Oscillospirales	Ruminococcaceae	uncultured	
denovo6104	0.0154%	0.0208%	0.0154%	0.0172%	0.0031%	Bacteria	Firmicutes	Clostridia	Oscillospirales	Butyricicoccaceae	UCG-009	
denovo850	0.0185%	0.0208%	0.0123%	0.0172%	0.0044%	Bacteria	Firmicutes	Clostridia	Christensenellales	Christensenellaceae	Christensenellaceae_R-7_group	
denovo8728	0.0215%	0.0208%	0.0092%	0.0172%	0.0069%	Bacteria	Firmicutes	uncultured	uncultured	uncultured	uncultured	uncultured_bacterium
denovo3557	0.0462%	0.0030%	0.0000%	0.0164%	0.0258%	Bacteria	Caldatribacteriota	JS1	JS1	JS1	JS1	uncultured_organism
denovo6779	0.0308%	0.0059%	0.0123%	0.0163%	0.0129%	Bacteria	Firmicutes	Clostridia	Christensenellales	Christensenellaceae	uncultured	
denovo4025	0.0123%	0.0119%	0.0246%	0.0163%	0.0072%	Unassigned						
denovo4382	0.0185%	0.0119%	0.0185%	0.0163%	0.0038%	Bacteria	Firmicutes	Clostridia	Oscillospirales	UCG-010	UCG-010	unidentified
denovo1671	0.0215%	0.0119%	0.0154%	0.0163%	0.0049%	Bacteria	Firmicutes	Clostridia	Peptococcales	Peptococcaceae	uncultured	
denovo8116	0.0246%	0.0238%	0.0000%	0.0161%	0.0140%	Bacteria	Firmicutes	Clostridia	Peptostreptococcales-Tissierellales	Anaerovoracaceae	[Eubacterium]_brachy_group	Peptostreptococcaceae_bacterium
denovo2765	0.0246%	0.0030%	0.0185%	0.0154%	0.0112%	Bacteria	Firmicutes	Clostridia	Oscillospirales	Ruminococcaceae	uncultured	uncultured_Firmicutes
denovo4726	0.0431%	0.0030%	0.0000%	0.0154%	0.0241%	Archaea	Euryarchaeota	Thermococci	Methanofastidiosales	Methanofastidiosaceae	Candidatus_Methanofastidiosum	uncultured_archaeon
denovo5501	0.0154%	0.0089%	0.0216%	0.0153%	0.0063%	Bacteria	Firmicutes	Clostridia	Christensenellales	Christensenellaceae	Christensenellaceae_R-7_group	uncultured_microorganism
denovo8580	0.0246%	0.0089%	0.0123%	0.0153%	0.0083%	Bacteria	Bacteroidota	Bacteroidia	Bacteroidales	M2PB4-65_termite_group	M2PB4-65_termite_group	metagenome
denovo3771	0.0215%	0.0119%	0.0123%	0.0153%	0.0055%	Bacteria	Firmicutes	Desulfitobacteriia	Desulfitobacteriales	Syntrophobotulaceae	Dehalobacter	uncultured_Dehalobacter
denovo6476	0.0092%	0.0149%	0.0216%	0.0152%	0.0062%	Bacteria	Firmicutes	Clostridia	Peptostreptococcales-Tissierellales	Anaerovoracaceae		
denovo3440	0.0308%	0.0149%	0.0000%	0.0152%	0.0154%	Bacteria	Chloroflexi	Anaerolineae	Anaerolineales	Anaerolineaceae		
denovo2385	0.0092%	0.0178%	0.0185%	0.0152%	0.0052%	Bacteria	Firmicutes	Clostridia	Eubacteriales	Eubacteriaceae	Eubacterium	
denovo7393	0.0185%	0.0238%	0.0031%	0.0151%	0.0108%	Bacteria						
denovo8189	0.0215%	0.0238%	0.0000%	0.0151%	0.0131%	Bacteria	Firmicutes	Clostridia	Oscillospirales			
denovo8622	0.0031%	0.0268%	0.0154%	0.0151%	0.0118%	Bacteria	Firmicutes	Clostridia	Peptostreptococcales-Tissierellales	Anaerovoracaceae	Anaerovorax	
denovo1281	0.0031%	0.0357%	0.0062%	0.0150%	0.0180%	Bacteria	Synergistota	Synergistia	Synergistales	Synergistaceae	Thermovirga	uncultured_Synergistetes
denovo7324	0.0400%	0.0000%	0.0031%	0.0144%	0.0223%	Bacteria	Firmicutes	Clostridia	Clostridiales	Clostridiaceae	Clostridium_sensu_stricto_10	
denovo7999	0.0031%	0.0030%	0.0369%	0.0143%	0.0196%	Bacteria	Desulfobacterota	Desulfovibrionia	Desulfovibrionales	Desulfovibrionaceae	Desulfovibrio	uncultured_Desulfovibrionaceae
denovo199	0.0185%	0.0030%	0.0216%	0.0143%	0.0100%	Bacteria	Firmicutes	Clostridia	Oscillospirales			
denovo3796	0.0123%	0.0059%	0.0246%	0.0143%	0.0095%	Bacteria	Firmicutes	Clostridia	Oscillospirales	Ruminococcaceae	uncultured	
denovo8381	0.0123%	0.0059%	0.0246%	0.0143%	0.0095%	Unassigned						
denovo2052	0.0308%	0.0059%	0.0062%	0.0143%	0.0143%	Bacteria	Firmicutes	Clostridia				
denovo1131	0.0000%	0.0089%	0.0339%	0.0143%	0.0176%	Bacteria	Bacteroidota	Bacteroidia	Bacteroidales	Tannerellaceae	uncultured	
denovo1031	0.0062%	0.0089%	0.0277%	0.0143%	0.0117%	Bacteria	Firmicutes	Clostridia				
denovo270	0.0308%	0.0089%	0.0031%	0.0143%	0.0146%	Bacteria	Thermotogota	Thermotogae	Kosmotogales	Kosmotogaceae	Mesotoga	
denovo8470	0.0123%	0.0119%	0.0185%	0.0142%	0.0037%	Unassigned						

denovo4174	0.0154%	0.0119%	0.0154%	0.0142%	0.0020%	Unassigned						
denovo8862	0.0154%	0.0119%	0.0154%	0.0142%	0.0020%	Eukaryota						
denovo8612	0.0185%	0.0119%	0.0123%	0.0142%	0.0037%	Bacteria	Firmicutes	Clostridia	Oscillospirales			
denovo30	0.0000%	0.0149%	0.0277%	0.0142%	0.0139%	Archaea	Thermoplasmatota	Thermoplasmata	Methanomassiliicoccales	Methanomethylophilaceae	uncultured	unidentified_archaeon
denovo8457	0.0092%	0.0178%	0.0154%	0.0142%	0.0044%	Bacteria						
denovo2536	0.0123%	0.0238%	0.0062%	0.0141%	0.0090%	Bacteria	Spirochaetota	Spirochaetia	Spirochaetales	Spirochaetaceae	Sphaerochaeta	uncultured_spirochete
denovo2089	0.0062%	0.0089%	0.0246%	0.0132%	0.0100%	Bacteria	Bacteroidota	Bacteroidia	Bacteroidales	Rikenellaceae	uncultured	
denovo282	0.0062%	0.0089%	0.0246%	0.0132%	0.0100%	Unassigned						
denovo7135	0.0092%	0.0089%	0.0216%	0.0132%	0.0072%	Unassigned						
denovo5756	0.0154%	0.0089%	0.0154%	0.0132%	0.0037%	Unassigned						
denovo9008	0.0154%	0.0089%	0.0154%	0.0132%	0.0037%	Archaea						
denovo2386	0.0031%	0.0119%	0.0246%	0.0132%	0.0108%	Unassigned						
denovo8769	0.0062%	0.0119%	0.0216%	0.0132%	0.0078%	Eukaryota						
denovo700	0.0123%	0.0119%	0.0154%	0.0132%	0.0019%	Bacteria	Desulfobacterota	Desulfovibrionia	Desulfovibrionales	Desulfovibrionaceae	Desulfovibrio	
denovo4357	0.0277%	0.0119%	0.0000%	0.0132%	0.0139%	Bacteria	Actinobacteriota	Actinobacteria	Propionibacteriales	Propionibacteriaceae	uncultured	uncultured_Propionibacteriaceae
denovo7409	0.0277%	0.0119%	0.0000%	0.0132%	0.0139%	Bacteria	Actinobacteriota	Thermoleophilia	Gaiellales	uncultured	uncultured	
denovo643	0.0123%	0.0149%	0.0123%	0.0132%	0.0015%	Bacteria	Firmicutes	Clostridia				
denovo3193	0.0154%	0.0149%	0.0092%	0.0132%	0.0034%	Bacteria	Bacteroidota	Bacteroidia	Bacteroidales	Dysgonomonadaceae	Dysgonomonas	
denovo5178	0.0185%	0.0149%	0.0062%	0.0132%	0.0063%	Bacteria	Firmicutes	Clostridia	Lachnospirales	Lachnospiraceae		
denovo1692	0.0062%	0.0178%	0.0154%	0.0131%	0.0062%	Bacteria	Firmicutes	Clostridia	Eubacteriales	Anaerofustaceae	Anaerofustis	uncultured_bacterium
denovo3416	0.0123%	0.0208%	0.0062%	0.0131%	0.0074%	Bacteria	Firmicutes	Clostridia	uncultured	uncultured	uncultured	uncultured_organism
denovo1620	0.0154%	0.0208%	0.0031%	0.0131%	0.0091%	Bacteria	Bacteroidota	Kryptonia	Kryptoniales	MSB-3C8	MSB-3C8	uncultured_bacterium
denovo5138	0.0215%	0.0030%	0.0123%	0.0123%	0.0093%	Bacteria	Firmicutes	Clostridia	Oscillospirales	Ruminococcaceae	uncultured	uncultured_Firmicutes
denovo1068	0.0277%	0.0030%	0.0062%	0.0123%	0.0134%	Bacteria	Firmicutes	Clostridia	Oscillospirales	Ruminococcaceae	uncultured	
denovo296	0.0092%	0.0059%	0.0216%	0.0122%	0.0082%	Bacteria	Firmicutes	Clostridia	Oscillospirales	Ruminococcaceae		
denovo2049	0.0154%	0.0059%	0.0154%	0.0122%	0.0055%	Bacteria	Spirochaetota	Spirochaetia	Spirochaetales	Spirochaetaceae	Sphaerochaeta	Sphaerochaeta_globosa
denovo3376	0.0246%	0.0059%	0.0062%	0.0122%	0.0107%	Bacteria	Firmicutes	Clostridia	Oscillospirales			
denovo7024	0.0246%	0.0059%	0.0062%	0.0122%	0.0107%	Bacteria	Bacteroidota	Bacteroidia	Bacteroidales	Marinilabiliaceae	uncultured	
denovo6554	0.0062%	0.0089%	0.0216%	0.0122%	0.0082%	Unassigned						
denovo5788	0.0092%	0.0089%	0.0185%	0.0122%	0.0054%	Unassigned						
denovo6013	0.0092%	0.0089%	0.0185%	0.0122%	0.0054%	Unassigned						
denovo1627	0.0123%	0.0089%	0.0154%	0.0122%	0.0032%	Bacteria	Verrucomicrobiota	Kiritimatiellae	WCHB1-41	WCHB1-41	WCHB1-41	uncultured_bacterium
denovo5775	0.0277%	0.0089%	0.0000%	0.0122%	0.0141%	Bacteria	Chloroflexi	Anaerolineae	Anaerolineales	Anaerolineaceae	uncultured	
denovo5146	0.0154%	0.0119%	0.0092%	0.0122%	0.0031%	Bacteria	Firmicutes	Clostridia	Christensenellales	Christensenellaceae		
denovo3315	0.0031%	0.0149%	0.0185%	0.0121%	0.0081%	Bacteria	Firmicutes	Clostridia	Oscillospirales	Butyricicoccaceae	UCG-009	uncultured_Firmicutes
denovo1346	0.0154%	0.0149%	0.0062%	0.0121%	0.0052%	Bacteria	Firmicutes	Clostridia	Oscillospirales	Ruminococcaceae	uncultured	uncultured_Firmicutes
denovo6509	0.0185%	0.0149%	0.0031%	0.0121%	0.0080%	Bacteria	Actinobacteriota	WCHB1-81	WCHB1-81	WCHB1-81	WCHB1-81	uncultured_eubacterium
denovo2369	0.0092%	0.0178%	0.0092%	0.0121%	0.0050%	Bacteria	Proteobacteria	Gammaproteobacteria	Enterobacterales	Enterobacteriaceae		
denovo4482	0.0154%	0.0178%	0.0031%	0.0121%	0.0079%	Bacteria	Firmicutes	Clostridia	Oscillospirales	Ruminococcaceae		
denovo2123	0.0031%	0.0268%	0.0062%	0.0120%	0.0129%	Bacteria	Firmicutes	Clostridia	Peptostreptococcales-Tissierellales	Anaerovoracaceae	Anaerovorax	
denovo1383	0.0031%	0.0000%	0.0308%	0.0113%	0.0170%	Bacteria	Bacteroidota	Bacteroidia	Bacteroidales	Dysgonomonadaceae		
denovo1002	0.0246%	0.0000%	0.0092%	0.0113%	0.0124%	Bacteria	Firmicutes	Bacilli	Erysipelotrichales	Erysipelatoclostridiaceae	UCG-004	uncultured_bacterium
denovo3273	0.0092%	0.0030%	0.0216%	0.0113%	0.0095%	Bacteria	Firmicutes	Clostridia	Oscillospirales			
denovo4995	0.0123%	0.0030%	0.0185%	0.0113%	0.0078%	Bacteria	Cloacimonadota	Cloacimonadia	Cloacimonadales	GZKB75	GZKB75	uncultured_bacterium
denovo1934	0.0246%	0.0030%	0.0062%	0.0112%	0.0117%	Bacteria	Firmicutes	Clostridia	Clostridiales	Clostridiaceae	Clostridium_sensu_stricto_8	
denovo2529	0.0000%	0.0059%	0.0277%	0.0112%	0.0146%	Bacteria	Spirochaetota	Spirochaetia	Spirochaetales	Spirochaetaceae	uncultured	uncultured_Treponema
denovo6397	0.0185%	0.0059%	0.0092%	0.0112%	0.0065%	Bacteria	Firmicutes	Clostridia	Oscillospirales	Oscillospiraceae		
denovo8709	0.0246%	0.0059%	0.0031%	0.0112%	0.0117%	Unassigned						
denovo5781	0.0000%	0.0089%	0.0246%	0.0112%	0.0125%	Bacteria	Firmicutes	Clostridia	Christensenellales	Christensenellaceae	uncultured	uncultured_bacterium
denovo2805	0.0123%	0.0089%	0.0123%	0.0112%	0.0020%	Bacteria	Firmicutes	Clostridia	Oscillospirales	Butyricicoccaceae	UCG-009	
denovo8245	0.0154%	0.0089%	0.0092%	0.0112%	0.0036%	Bacteria	Firmicutes	Syntrophomonadia	Syntrophomonadales	Syntrophomonadaceae	Syntrophomonas	
denovo230	0.0246%	0.0089%	0.0000%	0.0112%	0.0125%	Bacteria	Chloroflexi	Anaerolineae	Anaerolineales	Anaerolineaceae	Flexilinea	
denovo7170	0.0062%	0.0119%	0.0154%	0.0111%	0.0047%	Unassigned						
denovo7140	0.0154%	0.0119%	0.0062%	0.0111%	0.0047%	Unassigned						
denovo7853	0.0215%	0.0119%	0.0000%	0.0111%	0.0108%	Bacteria	Chloroflexi	Anaerolineae	SBR1031	SBR1031	SBR1031	terrestrial_metagenome
denovo6845	0.0092%	0.0149%	0.0092%	0.0111%	0.0033%	Bacteria	Firmicutes	Clostridia	Christensenellales	Christensenellaceae	Christensenellaceae_R-7_group	
denovo1637	0.0062%	0.0208%	0.0062%	0.0110%	0.0085%	Eukaryota						
denovo4665	0.0031%	0.0238%	0.0062%	0.0110%	0.0112%	Bacteria	Synergistota	Synergistia	Synergistales	Synergistaceae	Aminobacterium	uncultured_bacterium
denovo4131	0.0031%	0.0000%	0.0277%	0.0103%	0.0152%	Archaea	Euryarchaeota	Methanobacteria	Methanobacteriales	Methanobacteriaceae	Methanobacterium	
denovo6004	0.0092%	0.0000%	0.0216%	0.0103%	0.0108%	Bacteria	Bacteroidota	Bacteroidia	Bacteroidales	Rikenellaceae	Alistipes	

denovo7676	0.0123%	0.0000%	0.0185%	0.0103%	0.0094%	Bacteria	Desulfobacterota	Syntrophia	Syntrophales	Smithellaceae	Smithella	
denovo3508	0.0215%	0.0000%	0.0092%	0.0103%	0.0108%	Bacteria	Firmicutes	Clostridia	Oscillospirales	Butyricicoccaceae		
denovo1904	0.0031%	0.0030%	0.0246%	0.0102%	0.0125%	Bacteria	Bacteroidota	Bacteroidia	Bacteroidales	Bacteroidaceae	Bacteroides	
denovo7383	0.0092%	0.0030%	0.0185%	0.0102%	0.0078%	Bacteria	Firmicutes	Clostridia	Oscillospirales	Ruminococcaceae	Candidatus_Soleaferrea	
denovo3391	0.0031%	0.0059%	0.0216%	0.0102%	0.0099%	Bacteria	Desulfobacterota	Syntrophorhabdia	Syntrophorhabdales	Syntrophorhabdaceae	Syntrophorhabdus	
denovo7547	0.0031%	0.0059%	0.0216%	0.0102%	0.0099%	Bacteria	Firmicutes	Clostridia	Christensenellales	Christensenellaceae	uncultured	uncultured_bacterium
denovo1713	0.0062%	0.0059%	0.0185%	0.0102%	0.0072%	Bacteria	Firmicutes	Clostridia	Oscillospirales			
denovo2611	0.0154%	0.0059%	0.0092%	0.0102%	0.0048%	Unassigned						
denovo7877	0.0154%	0.0059%	0.0092%	0.0102%	0.0048%	Bacteria	Firmicutes	Bacilli	Lactobacillales			
denovo3482	0.0031%	0.0089%	0.0185%	0.0102%	0.0078%	Unassigned						
denovo805	0.0031%	0.0089%	0.0185%	0.0102%	0.0078%	Unassigned						
denovo3955	0.0062%	0.0089%	0.0154%	0.0102%	0.0047%	Unassigned						
denovo7426	0.0092%	0.0089%	0.0123%	0.0102%	0.0019%	Bacteria	Firmicutes	Clostridia	Christensenellales	Christensenellaceae		
denovo4571	0.0123%	0.0089%	0.0092%	0.0102%	0.0019%	Bacteria	Acidobacteriota	Aminicenantia	Aminicenantales	Aminicenantales	Aminicenantales	metagenome
denovo8370	0.0123%	0.0089%	0.0092%	0.0102%	0.0019%	Unassigned						
denovo7774	0.0185%	0.0089%	0.0031%	0.0102%	0.0078%	Bacteria	Firmicutes	Clostridia	Lachnospirales	Lachnospiraceae		
denovo5626	0.0031%	0.0119%	0.0154%	0.0101%	0.0063%	Bacteria	Bacteroidota	Bacteroidia	Bacteroidales	Dysgonomonadaceae	Dysgonomonas	
denovo5846	0.0062%	0.0119%	0.0123%	0.0101%	0.0034%	Bacteria	Firmicutes	Clostridia	Christensenellales	Christensenellaceae		
denovo8347	0.0062%	0.0119%	0.0123%	0.0101%	0.0034%	Bacteria	Firmicutes	Clostridia	Christensenellales	Christensenellaceae	Christensenellaceae_R-7_group	
denovo4908	0.0092%	0.0119%	0.0092%	0.0101%	0.0015%	Unassigned						
denovo7345	0.0000%	0.0149%	0.0154%	0.0101%	0.0087%	Bacteria						
denovo753	0.0000%	0.0149%	0.0154%	0.0101%	0.0087%	Unassigned						
denovo2538	0.0062%	0.0149%	0.0092%	0.0101%	0.0044%	Bacteria	Bacteroidota	Bacteroidia	Bacteroidales	Paludibacteraceae	uncultured	
denovo5084	0.0031%	0.0178%	0.0092%	0.0101%	0.0074%	Bacteria	Firmicutes	Syntrophomonadia	Syntrophomonadales	Syntrophomonadaceae	Syntrophomonas	Syntrophomonas_wolfei
denovo5587	0.0031%	0.0178%	0.0092%	0.0101%	0.0074%	Unassigned						
denovo7259	0.0031%	0.0208%	0.0062%	0.0100%	0.0095%	Bacteria	Patescibacteria	Parcubacteria	Candidatus_Nomurabacteria	Candidatus_Nomurabacteria	Candidatus_Nomurabacteria	uncultured_bacterium
denovo5533	0.0062%	0.0238%	0.0000%	0.0100%	0.0123%	Bacteria	Proteobacteria	Gammaproteobacteria	Enterobacterales	Enterobacteriaceae		
denovo6999	0.0123%	0.0000%	0.0154%	0.0092%	0.0081%	Bacteria	Firmicutes	Clostridia	Christensenellales	Christensenellaceae	Christensenellaceae_R-7_group	
denovo6850	0.0000%	0.0030%	0.0246%	0.0092%	0.0134%	Unassigned						
denovo571	0.0062%	0.0030%	0.0185%	0.0092%	0.0082%	Bacteria	Firmicutes	Clostridia	Oscillospirales			
denovo7213	0.0123%	0.0030%	0.0123%	0.0092%	0.0054%	Bacteria	Firmicutes	Clostridia	Christensenellales	Christensenellaceae		
denovo4976	0.0246%	0.0030%	0.0000%	0.0092%	0.0134%	Bacteria	Myxococcota	Myxococcia	Myxococcales	Myxococcaceae	uncultured	
denovo1914	0.0031%	0.0059%	0.0185%	0.0092%	0.0082%	Unassigned						
denovo6607	0.0031%	0.0059%	0.0185%	0.0092%	0.0082%	Bacteria	Firmicutes	Clostridia	Christensenellales	Christensenellaceae	Christensenellaceae_R-7_group	
denovo2642	0.0062%	0.0059%	0.0154%	0.0092%	0.0054%	Unassigned						
denovo3342	0.0062%	0.0059%	0.0154%	0.0092%	0.0054%	Unassigned						
denovo3725	0.0062%	0.0059%	0.0154%	0.0092%	0.0054%	Unassigned						
denovo4762	0.0062%	0.0059%	0.0154%	0.0092%	0.0054%	Bacteria	Bacteroidota	SJA-28	SJA-28	SJA-28	SJA-28	uncultured_Bacteroidetes
denovo1696	0.0123%	0.0059%	0.0092%	0.0092%	0.0032%	Unassigned						
denovo999	0.0154%	0.0059%	0.0062%	0.0092%	0.0054%	Bacteria	Firmicutes	Clostridia	Peptostreptococcales-Tissierellales	Anaerovoracaceae	Anaerovorax	
denovo7875	0.0215%	0.0059%	0.0000%	0.0092%	0.0111%	Bacteria	Synergistota	Synergistia	Synergistales	Synergistaceae	uncultured	uncultured_bacterium
denovo6465	0.0000%	0.0089%	0.0185%	0.0091%	0.0092%	Bacteria	Desulfobacterota	Desulfovibrionia	Desulfovibrionales	Desulfovibrionaceae	Desulfovibrio	uncultured_proteobacterium
denovo8791	0.0000%	0.0089%	0.0185%	0.0091%	0.0092%	Unassigned						
denovo3368	0.0031%	0.0089%	0.0154%	0.0091%	0.0062%	Unassigned						
denovo5389	0.0031%	0.0089%	0.0154%	0.0091%	0.0062%	Unassigned						
denovo1398	0.0062%	0.0089%	0.0123%	0.0091%	0.0031%	Unassigned						
denovo1966	0.0062%	0.0089%	0.0123%	0.0091%	0.0031%	Unassigned						
denovo6650	0.0062%	0.0089%	0.0123%	0.0091%	0.0031%	Unassigned						
denovo4072	0.0092%	0.0089%	0.0092%	0.0091%	0.0002%	Bacteria	Desulfobacterota	Desulfovibrionia	Desulfovibrionales	Desulfovibrionaceae	Desulfovibrio	
denovo2417	0.0123%	0.0089%	0.0062%	0.0091%	0.0031%	Bacteria	Actinobacteriota	WCHB1-81	WCHB1-81	WCHB1-81	WCHB1-81	
denovo6386	0.0123%	0.0089%	0.0062%	0.0091%	0.0031%	Archaea	Halobacterota	Methanosarcinia	Methanosarciniales	Methanosaetaceae	Methanosaeta	Methanomicrobia_archaeon
denovo7989	0.0123%	0.0089%	0.0062%	0.0091%	0.0031%	Unassigned						
denovo1537	0.0031%	0.0119%	0.0123%	0.0091%	0.0052%	Unassigned						
denovo1074	0.0062%	0.0119%	0.0092%	0.0091%	0.0029%	Bacteria	Firmicutes	Clostridia	Oscillospirales	Ruminococcaceae	uncultured	
denovo689	0.0062%	0.0119%	0.0092%	0.0091%	0.0029%	Unassigned						
denovo3042	0.0031%	0.0149%	0.0092%	0.0091%	0.0059%	Bacteria	Firmicutes	Clostridia	Clostridia	Gracilibacteraceae	Lutispora	Clostridium
denovo3470	0.0092%	0.0149%	0.0031%	0.0091%	0.0059%	Bacteria	Actinobacteriota	Coriobacteriia	Coriobacteriales	Coriobacteriales_Incertae_Sedis	uncultured	
denovo187	0.0062%	0.0000%	0.0185%	0.0082%	0.0094%	Eukaryota						
denovo6347	0.0154%	0.0000%	0.0092%	0.0082%	0.0077%	Bacteria	Proteobacteria	Gammaproteobacteria	Burkholderiales	Comamonadaceae	Comamonas	
denovo5575	0.0246%	0.0000%	0.0000%	0.0082%	0.0142%	Bacteria	Firmicutes	Incertae_Sedis	DTU014	DTU014	DTU014	uncultured_Selenomonadales

denovo4166	0.0000%	0.0030%	0.0216%	0.0082%	0.0117%	Bacteria	Firmicutes	Clostridia	Oscillospirales	Ruminococcaceae	Negativibacillus	uncultured_Anaerotruncus
denovo460	0.0031%	0.0030%	0.0185%	0.0082%	0.0089%	Bacteria	Firmicutes	Clostridia	Eubacteriales	Anaerofustaceae	Anaerofustis	uncultured_organism
denovo1063	0.0062%	0.0030%	0.0154%	0.0082%	0.0065%	Unassigned						
denovo1200	0.0062%	0.0030%	0.0154%	0.0082%	0.0065%	Unassigned						
denovo1432	0.0092%	0.0030%	0.0123%	0.0082%	0.0048%	Unassigned						
denovo31	0.0123%	0.0030%	0.0092%	0.0082%	0.0048%	Bacteria	Firmicutes	Clostridia	Clostridia	Hungateiclostridiaceae	uncultured	uncultured_microorganism
denovo7821	0.0123%	0.0030%	0.0092%	0.0082%	0.0048%	Bacteria	Firmicutes	Syntrophomonadia	Syntrophomonadales	Syntrophomonadaceae	Syntrophomonas	
denovo1536	0.0154%	0.0030%	0.0062%	0.0082%	0.0064%	Unassigned						
denovo2018	0.0185%	0.0030%	0.0031%	0.0082%	0.0089%	Bacteria	Desulfobacterota	Desulfovibrionia	Desulfovibrionales	Desulfovibrionaceae	Desulfovibrio	uncultured_proteobacterium
denovo4113	0.0031%	0.0059%	0.0154%	0.0081%	0.0064%	Unassigned						
denovo5422	0.0031%	0.0059%	0.0154%	0.0081%	0.0064%	Unassigned						
denovo7929	0.0031%	0.0059%	0.0154%	0.0081%	0.0064%	Bacteria	Bacteroidota	Bacteroidia	Bacteroidales	Dysgonomonadaceae	Dysgonomonadaceae	uncultured_compost
denovo2119	0.0092%	0.0059%	0.0092%	0.0081%	0.0019%	Unassigned						
denovo4462	0.0092%	0.0059%	0.0092%	0.0081%	0.0019%	Unassigned						
denovo64	0.0092%	0.0059%	0.0092%	0.0081%	0.0019%	Unassigned						
denovo7166	0.0092%	0.0059%	0.0092%	0.0081%	0.0019%	Unassigned						
denovo7232	0.0092%	0.0059%	0.0092%	0.0081%	0.0019%	Bacteria	Firmicutes	Clostridia	Peptococcales	Peptococcaceae	uncultured	
denovo8018	0.0092%	0.0059%	0.0092%	0.0081%	0.0019%	Eukaryota						
denovo6881	0.0123%	0.0059%	0.0062%	0.0081%	0.0036%	Bacteria	Planctomycetota	vadinHA49	vadinHA49	vadinHA49	vadinHA49	uncultured_Planctomycetaceae
denovo258	0.0154%	0.0059%	0.0031%	0.0081%	0.0064%	Bacteria	Firmicutes	Clostridia				
denovo4344	0.0031%	0.0089%	0.0123%	0.0081%	0.0047%	Unassigned						
denovo8044	0.0031%	0.0089%	0.0123%	0.0081%	0.0047%	Unassigned						
denovo8717	0.0062%	0.0089%	0.0092%	0.0081%	0.0017%	Unassigned						
denovo1809	0.0092%	0.0089%	0.0062%	0.0081%	0.0017%	Bacteria	Firmicutes	Clostridia	Peptococcales	Peptococcaceae	uncultured	
denovo7934	0.0092%	0.0089%	0.0062%	0.0081%	0.0017%	Unassigned						
denovo7433	0.0031%	0.0119%	0.0092%	0.0081%	0.0045%	Unassigned						
denovo3973	0.0062%	0.0119%	0.0062%	0.0081%	0.0033%	Bacteria	Firmicutes	Clostridia	Peptococcales	Peptococcaceae	uncultured	
denovo5552	0.0062%	0.0119%	0.0062%	0.0081%	0.0033%	Eukaryota						
denovo5630	0.0062%	0.0119%	0.0062%	0.0081%	0.0033%	Archaea	Euryarchaeota	Methanobacteria	Methanobacteriales	Methanobacteriaceae	Methanobacterium	
denovo6566	0.0062%	0.0119%	0.0062%	0.0081%	0.0033%	Unassigned						
denovo8901	0.0123%	0.0119%	0.0000%	0.0081%	0.0070%	Unassigned						
denovo1368	0.0062%	0.0149%	0.0031%	0.0080%	0.0061%	Bacteria	Firmicutes	Clostridia				
denovo7388	0.0062%	0.0149%	0.0031%	0.0080%	0.0061%	Unassigned						
denovo2199	0.0062%	0.0178%	0.0000%	0.0080%	0.0091%	Bacteria	Firmicutes	Clostridia	Clostridia	Hungateiclostridiaceae	Ruminiclostridium	
denovo323	0.0000%	0.0000%	0.0216%	0.0072%	0.0124%	Bacteria	Bacteroidota	Bacteroidia	Bacteroidales	Paludibacteraceae		
denovo8411	0.0031%	0.0000%	0.0185%	0.0072%	0.0099%	Archaea	Euryarchaeota	Methanobacteria	Methanobacteriales	Methanobacteriaceae	Methanobacterium	
denovo3472	0.0062%	0.0000%	0.0154%	0.0072%	0.0077%	Bacteria	Firmicutes	Clostridia	Oscillospirales	Ruminococcaceae	Candidatus_Soleaferrea	
denovo8132	0.0185%	0.0000%	0.0031%	0.0072%	0.0099%	Bacteria	Firmicutes	Clostridia	Christensenellales	Christensenellaceae	Christensenellaceae_R-7_group	
denovo6093	0.0215%	0.0000%	0.0000%	0.0072%	0.0124%	Bacteria	Synergistota	Synergistia	Synergistales	Synergistaceae	Aminivibrio	
denovo8040	0.0031%	0.0030%	0.0154%	0.0071%	0.0071%	Eukaryota						
denovo2991	0.0062%	0.0030%	0.0123%	0.0071%	0.0047%	Bacteria	Firmicutes	Clostridia	Peptostreptococcales-Tissierellales	Anaerovoracaceae		
denovo6274	0.0062%	0.0030%	0.0123%	0.0071%	0.0047%	Unassigned						
denovo6258	0.0092%	0.0030%	0.0092%	0.0071%	0.0036%	Bacteria	FW113	FW113	FW113	FW113	FW113	uncultured_bacterium
denovo6675	0.0092%	0.0030%	0.0092%	0.0071%	0.0036%	Unassigned						
denovo7214	0.0123%	0.0030%	0.0062%	0.0071%	0.0047%	Bacteria	Firmicutes	Clostridia	Oscillospirales	Ruminococcaceae		
denovo8511	0.0123%	0.0030%	0.0062%	0.0071%	0.0047%	Bacteria						
denovo965	0.0000%	0.0059%	0.0154%	0.0071%	0.0078%	Unassigned						
denovo2920	0.0031%	0.0059%	0.0123%	0.0071%	0.0047%	Unassigned						
denovo7296	0.0031%	0.0059%	0.0123%	0.0071%	0.0047%	Bacteria						
denovo6047	0.0062%	0.0059%	0.0092%	0.0071%	0.0018%	Unassigned						
denovo1395	0.0092%	0.0059%	0.0062%	0.0071%	0.0018%	Bacteria	Firmicutes	Clostridia	Oscillospirales	Oscillospiraceae		
denovo2740	0.0092%	0.0059%	0.0062%	0.0071%	0.0018%	Unassigned						
denovo3729	0.0092%	0.0059%	0.0062%	0.0071%	0.0018%	Bacteria	Firmicutes	Clostridia	Oscillospirales	Ruminococcaceae	Incertae_Sedis	
denovo4402	0.0092%	0.0059%	0.0062%	0.0071%	0.0018%	Eukaryota						
denovo2739	0.0123%	0.0059%	0.0031%	0.0071%	0.0047%	Bacteria	Firmicutes	Desulfotomaculia	Desulfotomaculales	Desulfotomaculales	Pelotomaculum	uncultured_bacterium
denovo5337	0.0000%	0.0089%	0.0123%	0.0071%	0.0064%	Unassigned						
denovo5923	0.0000%	0.0089%	0.0123%	0.0071%	0.0064%	Unassigned						
denovo358	0.0031%	0.0089%	0.0092%	0.0071%	0.0035%	Unassigned						
denovo5542	0.0031%	0.0089%	0.0092%	0.0071%	0.0035%	Bacteria	Firmicutes	Clostridia	Christensenellales	Christensenellaceae	Christensenellaceae_R-7_group	
denovo5800	0.0031%	0.0089%	0.0092%	0.0071%	0.0035%	Unassigned						

denovo4047	0.0062%	0.0089%	0.0062%	0.0071%	0.0016%	Unassigned						
denovo4397	0.0092%	0.0089%	0.0031%	0.0071%	0.0035%	Archaea	Euryarchaeota	Methanobacteria	Methanobacteriales	Methanobacteriaceae	Methanobacterium	
denovo4796	0.0092%	0.0089%	0.0031%	0.0071%	0.0035%	Unassigned						
denovo8911	0.0092%	0.0089%	0.0031%	0.0071%	0.0035%	Bacteria	Firmicutes	Clostridia	Oscillospirales	Ruminococcaceae	uncultured	
denovo2869	0.0000%	0.0119%	0.0092%	0.0070%	0.0062%	Unassigned						
denovo7722	0.0031%	0.0119%	0.0062%	0.0070%	0.0045%	Unassigned						
denovo2430	0.0092%	0.0119%	0.0000%	0.0070%	0.0062%	Bacteria	Firmicutes	Clostridia	Oscillospirales			
denovo4215	0.0092%	0.0119%	0.0000%	0.0070%	0.0062%	Bacteria	Firmicutes	Clostridia	Clostridia	Hungateiclostridiaceae	uncultured	uncultured_Acetivibrio
denovo551	0.0092%	0.0119%	0.0000%	0.0070%	0.0062%	Bacteria	Planctomycetota	Phycisphaerae	MSBL9	KCLunmb-38-53	KCLunmb-38-53	uncultured_bacterium
denovo1502	0.0000%	0.0149%	0.0062%	0.0070%	0.0075%	Bacteria	Firmicutes	Clostridia				
denovo4531	0.0000%	0.0149%	0.0062%	0.0070%	0.0075%	Bacteria	Firmicutes	Clostridia	Christensenellales	Christensenellaceae	Christensenellaceae_R-7_group	
denovo5048	0.0000%	0.0149%	0.0062%	0.0070%	0.0075%	Eukaryota						
denovo8601	0.0031%	0.0149%	0.0031%	0.0070%	0.0068%	Bacteria	Bacteroidota	Kapabacteria	Kapabacteriales	Kapabacteriales	Kapabacteriales	uncultured_organism
denovo7678	0.0000%	0.0208%	0.0000%	0.0069%	0.0120%	Bacteria	Cloacimonadota	Cloacimonadia	Cloacimonadales	Cloacimonadaceae	Candidatus_Cloacimonas	uncultured_bacterium
denovo7300	0.0000%	0.0000%	0.0185%	0.0062%	0.0107%	Archaea	Euryarchaeota	Methanobacteria	Methanobacteriales	Methanobacteriaceae	Methanobacterium	uncultured_Methanobacteriales
denovo265	0.0031%	0.0000%	0.0154%	0.0062%	0.0081%	Eukaryota						
denovo3521	0.0031%	0.0000%	0.0154%	0.0062%	0.0081%	Bacteria	Bacteroidota	Bacteroidia	Bacteroidales	Rikenellaceae	DMER64	bacterium_enrichment
denovo5918	0.0062%	0.0000%	0.0123%	0.0062%	0.0062%	Unassigned						
denovo6538	0.0062%	0.0000%	0.0123%	0.0062%	0.0062%	Unassigned						
denovo7240	0.0062%	0.0000%	0.0123%	0.0062%	0.0062%	Bacteria						
denovo8512	0.0062%	0.0000%	0.0123%	0.0062%	0.0062%	Bacteria	Firmicutes	Clostridia	Christensenellales	Christensenellaceae	Christensenellaceae_R-7_group	
denovo4035	0.0092%	0.0000%	0.0092%	0.0062%	0.0053%	Unassigned						
denovo6626	0.0092%	0.0000%	0.0092%	0.0062%	0.0053%	Bacteria	Firmicutes	Clostridia	Oscillospirales	Ruminococcaceae	Candidatus_Soleaferrea	
denovo8086	0.0092%	0.0000%	0.0092%	0.0062%	0.0053%	Unassigned						
denovo3986	0.0123%	0.0000%	0.0062%	0.0062%	0.0062%	Unassigned						
denovo1718	0.0185%	0.0000%	0.0000%	0.0062%	0.0107%	Archaea	Euryarchaeota	Methanobacteria	Methanobacteriales	Methanobacteriaceae	Methanobacterium	
denovo979	0.0000%	0.0030%	0.0154%	0.0061%	0.0082%	Bacteria	Bacteroidota	Bacteroidia	Bacteroidales	Prolixibacteraceae	Meniscus	
denovo2477	0.0031%	0.0030%	0.0123%	0.0061%	0.0054%	Unassigned						
denovo2843	0.0031%	0.0030%	0.0123%	0.0061%	0.0054%	Unassigned						
denovo4980	0.0031%	0.0030%	0.0123%	0.0061%	0.0054%	Unassigned						
denovo3091	0.0062%	0.0030%	0.0092%	0.0061%	0.0031%	Unassigned						
denovo322	0.0062%	0.0030%	0.0092%	0.0061%	0.0031%	Bacteria	Planctomycetota	vadinHA49	vadinHA49	vadinHA49	vadinHA49	
denovo7270	0.0062%	0.0030%	0.0092%	0.0061%	0.0031%	Unassigned						
denovo3484	0.0092%	0.0030%	0.0062%	0.0061%	0.0031%	Bacteria	Desulfobacterota	Desulfuromonadia	Desulfuromonadia	Desulfuromonadaceae		
denovo1601	0.0123%	0.0030%	0.0031%	0.0061%	0.0054%	Bacteria						
denovo5240	0.0123%	0.0030%	0.0031%	0.0061%	0.0054%	Bacteria	Bacteroidota	Bacteroidia	Bacteroidales	Prevotellaceae		
denovo5360	0.0154%	0.0030%	0.0000%	0.0061%	0.0082%	Archaea	Thermoplasmatota	Thermoplasmata	Methanomassiliicoccales	Methanomassiliicoccaceae	Methanomassiliicoccus	uncultured_archaeon
denovo1526	0.0000%	0.0059%	0.0123%	0.0061%	0.0062%	Unassigned						
denovo4533	0.0000%	0.0059%	0.0123%	0.0061%	0.0062%	Bacteria	Firmicutes	Clostridia	Peptostreptococcales-Tissierellales	Sedimentibacteraceae	Sedimentibacter	bacterium_enrichment
denovo8243	0.0000%	0.0059%	0.0123%	0.0061%	0.0062%	Unassigned						
denovo2685	0.0031%	0.0059%	0.0092%	0.0061%	0.0031%	Eukaryota						
denovo420	0.0031%	0.0059%	0.0092%	0.0061%	0.0031%	Unassigned						
denovo431	0.0031%	0.0059%	0.0092%	0.0061%	0.0031%	Unassigned						
denovo5769	0.0031%	0.0059%	0.0092%	0.0061%	0.0031%	Bacteria	Bacteroidota	Bacteroidia	Bacteroidales	Bacteroidetes_vadinHA17	Bacteroidetes_vadinHA17	uncultured_bacterium
denovo6071	0.0031%	0.0059%	0.0092%	0.0061%	0.0031%	Unassigned						
denovo8095	0.0031%	0.0059%	0.0092%	0.0061%	0.0031%	Bacteria						
denovo522	0.0062%	0.0059%	0.0062%	0.0061%	0.0001%	Eukaryota						
denovo5265	0.0062%	0.0059%	0.0062%	0.0061%	0.0001%	Unassigned						
denovo5708	0.0062%	0.0059%	0.0062%	0.0061%	0.0001%	Eukaryota						
denovo6010	0.0062%	0.0059%	0.0062%	0.0061%	0.0001%	Bacteria	Bacteroidota	Bacteroidia	Bacteroidales	Dysgonomonadaceae	Dysgonomonas	uncultured_Dysgonomonas
denovo1943	0.0092%	0.0059%	0.0031%	0.0061%	0.0031%	Bacteria	Firmicutes	Clostridia	Peptococcales	Peptococcaceae	uncultured	
denovo2554	0.0092%	0.0059%	0.0031%	0.0061%	0.0031%	Bacteria	Firmicutes	uncultured	uncultured	uncultured	uncultured	uncultured_bacterium
denovo2944	0.0092%	0.0059%	0.0031%	0.0061%	0.0031%	Bacteria	Synergistota	Synergistia	Synergistales	Synergistaceae	uncultured	bacterium_enrichment
denovo234	0.0123%	0.0059%	0.0000%	0.0061%	0.0062%	Bacteria	Desulfobacterota	Syntrophobacteria	Syntrophobacterales	Syntrophobacteraceae	Syntrophobacter	uncultured_bacterium
denovo1946	0.0000%	0.0089%	0.0092%	0.0061%	0.0052%	Eukaryota						
denovo2601	0.0000%	0.0089%	0.0092%	0.0061%	0.0052%	Bacteria						
denovo6783	0.0000%	0.0089%	0.0092%	0.0061%	0.0052%	Bacteria	Firmicutes	Clostridia	Oscillospirales	Oscillospiraceae	NK4A214_group	
denovo6869	0.0000%	0.0089%	0.0092%	0.0061%	0.0052%	Bacteria	Firmicutes	Clostridia	Oscillospirales	Butyricicoccaceae	UCG-009	uncultured_bacterium
denovo7197	0.0000%	0.0089%	0.0092%	0.0061%	0.0052%	Unassigned						
denovo228	0.0031%	0.0089%	0.0062%	0.0061%	0.0029%	Unassigned						

denovo1103	0.0062%	0.0089%	0.0031%	0.0061%	0.0029%	Unassigned						
denovo5942	0.0062%	0.0089%	0.0031%	0.0061%	0.0029%	Unassigned						
denovo1522	0.0000%	0.0119%	0.0062%	0.0060%	0.0059%	Bacteria	Firmicutes	Clostridia	Clostridia_UCG-014	Clostridia_UCG-014	Clostridia_UCG-014	uncultured_Firmicutes
denovo6324	0.0000%	0.0119%	0.0062%	0.0060%	0.0059%	Unassigned						
denovo8277	0.0031%	0.0119%	0.0031%	0.0060%	0.0051%	Bacteria	Firmicutes	Incertae_Sedis	DTU014	DTU014	DTU014	unidentified
denovo462	0.0062%	0.0119%	0.0000%	0.0060%	0.0059%	Unassigned						
denovo2566	0.0031%	0.0149%	0.0000%	0.0060%	0.0078%	Unassigned						
denovo2973	0.0000%	0.0000%	0.0154%	0.0051%	0.0089%	Bacteria	Firmicutes	Clostridia	Lachnospirales	Lachnospiraceae		
denovo3571	0.0000%	0.0000%	0.0154%	0.0051%	0.0089%	Bacteria	Bacteroidota	Bacteroidia	Bacteroidales	Rikenellaceae	uncultured	
denovo4758	0.0000%	0.0000%	0.0154%	0.0051%	0.0089%	Bacteria	Proteobacteria	Alphaproteobacteria	Rhodospirillales	uncultured	uncultured	
denovo5003	0.0000%	0.0000%	0.0154%	0.0051%	0.0089%	Unassigned						
denovo1348	0.0031%	0.0000%	0.0123%	0.0051%	0.0064%	Unassigned						
denovo2143	0.0031%	0.0000%	0.0123%	0.0051%	0.0064%	Bacteria	Firmicutes	Clostridia	Lachnospirales	Lachnospiraceae	Eisenbergiella	
denovo6058	0.0031%	0.0000%	0.0123%	0.0051%	0.0064%	Unassigned						
denovo1871	0.0062%	0.0000%	0.0092%	0.0051%	0.0047%	Bacteria	Desulfobacterota	Desulfobacteria	Desulfobacterales	Desulfosarcinaceae		
denovo2982	0.0062%	0.0000%	0.0092%	0.0051%	0.0047%	Bacteria	Firmicutes	Clostridia	Christensenellales	Christensenellaceae	Christensenellaceae_R-7_group	uncultured_Clostridiaceae
denovo7132	0.0062%	0.0000%	0.0092%	0.0051%	0.0047%	Unassigned						
denovo7656	0.0062%	0.0000%	0.0092%	0.0051%	0.0047%	Unassigned						
denovo7914	0.0062%	0.0000%	0.0092%	0.0051%	0.0047%	Bacteria						
denovo8166	0.0062%	0.0000%	0.0092%	0.0051%	0.0047%	Unassigned						
denovo4576	0.0092%	0.0000%	0.0062%	0.0051%	0.0047%	Bacteria	Firmicutes	Clostridia	Oscillospirales	Ruminococcaceae	uncultured	
denovo5757	0.0092%	0.0000%	0.0062%	0.0051%	0.0047%	Bacteria	Spirochaetota	Spirochaetia	Spirochaetales	Spirochaetaceae	Spirochaeta	
denovo8606	0.0123%	0.0000%	0.0031%	0.0051%	0.0064%	Unassigned						
denovo4222	0.0154%	0.0000%	0.0000%	0.0051%	0.0089%	Bacteria	Firmicutes	Clostridia	Oscillospirales			
denovo4740	0.0154%	0.0000%	0.0000%	0.0051%	0.0089%	Bacteria	Firmicutes	Clostridia	Christensenellales	Christensenellaceae	uncultured	
denovo4958	0.0154%	0.0000%	0.0000%	0.0051%	0.0089%	Bacteria	Elusimicrobiota	Endomicrobia	Endomicrobiales	Endomicrobiaceae	Endomicrobium	uncultured_bacterium
denovo1558	0.0000%	0.0030%	0.0123%	0.0051%	0.0064%	Bacteria	Firmicutes	Clostridia	Oscillospirales	Ruminococcaceae	uncultured	uncultured_Firmicutes
denovo5291	0.0000%	0.0030%	0.0123%	0.0051%	0.0064%	Bacteria	Proteobacteria	Gammaproteobacteria	Xanthomonadales	Rhodanobacteraceae	Dokdonella	
denovo543	0.0000%	0.0030%	0.0123%	0.0051%	0.0064%	Bacteria	Firmicutes	Clostridia	Peptococcales	Peptococcaceae	uncultured	
denovo5447	0.0000%	0.0030%	0.0123%	0.0051%	0.0064%	Unassigned						
denovo3290	0.0031%	0.0030%	0.0092%	0.0051%	0.0036%	Bacteria	Firmicutes	Clostridia	Oscillospirales	Oscillospirales	Hydrogenoanaerobacterium	uncultured_bacterium
denovo3998	0.0031%	0.0030%	0.0092%	0.0051%	0.0036%	Unassigned						
denovo4275	0.0031%	0.0030%	0.0092%	0.0051%	0.0036%	Bacteria	Chloroflexi	Anaerolineae	Anaerolineales	Anaerolineaceae	Pelolinea	
denovo4919	0.0031%	0.0030%	0.0092%	0.0051%	0.0036%	Unassigned						
denovo6016	0.0031%	0.0030%	0.0092%	0.0051%	0.0036%	Unassigned						
denovo7249	0.0031%	0.0030%	0.0092%	0.0051%	0.0036%	Archaea	Halobacterota					
denovo579	0.0062%	0.0030%	0.0062%	0.0051%	0.0018%	Bacteria	Firmicutes	Negativicutes	Veillonellales-Selenomonadales	Sporomusaceae	uncultured	
denovo6729	0.0062%	0.0030%	0.0062%	0.0051%	0.0018%	Unassigned						
denovo7680	0.0062%	0.0030%	0.0062%	0.0051%	0.0018%	Bacteria	Firmicutes	Clostridia	Clostridia	Hungateiclostridiaceae	uncultured	
denovo3355	0.0092%	0.0030%	0.0031%	0.0051%	0.0036%	Bacteria	Firmicutes	Syntrophomonadia	Syntrophomonadales	Syntrophomonadaceae	Syntrophomonas	
denovo5421	0.0092%	0.0030%	0.0031%	0.0051%	0.0036%	Bacteria	Firmicutes	Clostridia	Christensenellales	Christensenellaceae	uncultured	uncultured_Clostridiales
denovo8640	0.0092%	0.0030%	0.0031%	0.0051%	0.0036%	Eukaryota						
denovo9053	0.0092%	0.0030%	0.0031%	0.0051%	0.0036%	Eukaryota						
denovo3259	0.0123%	0.0030%	0.0000%	0.0051%	0.0064%	Archaea	Crenarchaeota	Bathyarchaeia	Bathyarchaeia	Bathyarchaeia	Bathyarchaeia	archaeon_enrichment
denovo39	0.0123%	0.0030%	0.0000%	0.0051%	0.0064%	Bacteria	Bacteroidota	Kapabacteria	Kapabacteriales	Kapabacteriales	Kapabacteriales	
denovo4384	0.0123%	0.0030%	0.0000%	0.0051%	0.0064%	Bacteria	Chloroflexi	Anaerolineae	Anaerolineales	Anaerolineaceae	uncultured	
denovo9057	0.0123%	0.0030%	0.0000%	0.0051%	0.0064%	Bacteria	Proteobacteria	Gammaproteobacteria	Pseudomonadales	Pseudomonadaceae	Pseudomonas	
denovo1717	0.0000%	0.0059%	0.0092%	0.0051%	0.0047%	Bacteria	Firmicutes	Clostridia	Oscillospirales	Ruminococcaceae	uncultured	uncultured_Firmicutes
denovo2296	0.0000%	0.0059%	0.0092%	0.0051%	0.0047%	Unassigned						
denovo3882	0.0000%	0.0059%	0.0092%	0.0051%	0.0047%	Bacteria	Firmicutes	Clostridia				
denovo4220	0.0000%	0.0059%	0.0092%	0.0051%	0.0047%	Unassigned						
denovo6399	0.0000%	0.0059%	0.0092%	0.0051%	0.0047%	Archaea	Thermoplasmatota	Thermoplasmata	Methanomassiliicoccales	Methanomethylophilaceae	RumEn_M2	archaeon_PY-11
denovo1009	0.0031%	0.0059%	0.0062%	0.0051%	0.0017%	Unassigned						
denovo1173	0.0031%	0.0059%	0.0062%	0.0051%	0.0017%	Unassigned						
denovo1510	0.0031%	0.0059%	0.0062%	0.0051%	0.0017%	Unassigned						
denovo2129	0.0031%	0.0059%	0.0062%	0.0051%	0.0017%	Unassigned						
denovo3057	0.0031%	0.0059%	0.0062%	0.0051%	0.0017%	Unassigned						
denovo3957	0.0031%	0.0059%	0.0062%	0.0051%	0.0017%	Unassigned						
denovo4721	0.0031%	0.0059%	0.0062%	0.0051%	0.0017%	Unassigned						
denovo5015	0.0031%	0.0059%	0.0062%	0.0051%	0.0017%	Bacteria						

denovo5273	0.0031%	0.0059%	0.0062%	0.0051%	0.0017%	Unassigned						
denovo5434	0.0031%	0.0059%	0.0062%	0.0051%	0.0017%	Unassigned						
denovo8279	0.0031%	0.0059%	0.0062%	0.0051%	0.0017%	Unassigned						
denovo8974	0.0031%	0.0059%	0.0062%	0.0051%	0.0017%	Bacteria	Proteobacteria	Gammaproteobacteria	Burkholderiales	Oxalobacteraceae		
denovo3887	0.0062%	0.0059%	0.0031%	0.0051%	0.0017%	Bacteria	Firmicutes	Desulfotomaculia	Desulfotomaculales	Desulfotomaculales	Pelotomaculum	uncultured_bacterium
denovo6496	0.0062%	0.0059%	0.0031%	0.0051%	0.0017%	Bacteria	Firmicutes	Bacilli	Lactobacillales	Enterococcaceae	Enterococcus	
denovo8433	0.0062%	0.0059%	0.0031%	0.0051%	0.0017%	Bacteria	Firmicutes	Clostridia	Oscillospirales			
denovo2023	0.0092%	0.0059%	0.0000%	0.0051%	0.0047%	Bacteria	Desulfobacterota	Syntrophia	Syntrophales	Syntrophaceae	Syntrophus	
denovo2117	0.0092%	0.0059%	0.0000%	0.0051%	0.0047%	Bacteria	Firmicutes	Clostridia	Oscillospirales	UCG-010	UCG-010	uncultured_bacterium
denovo4589	0.0092%	0.0059%	0.0000%	0.0051%	0.0047%	Bacteria	Synergistota	Synergistia	Synergistales	Synergistaceae	uncultured	bacterium_enrichment
denovo3578	0.0000%	0.0089%	0.0062%	0.0050%	0.0046%	Eukaryota						
denovo4997	0.0000%	0.0089%	0.0062%	0.0050%	0.0046%	Unassigned						
denovo5827	0.0000%	0.0089%	0.0062%	0.0050%	0.0046%	Bacteria	Actinobacteriota	Actinobacteria	Actinomycetales	Actinomycetaceae	Actinomyces	
denovo5876	0.0000%	0.0089%	0.0062%	0.0050%	0.0046%	Bacteria	Firmicutes	Clostridia	Oscillospirales	Ruminococcaceae	Angelakisella	
denovo5369	0.0031%	0.0089%	0.0031%	0.0050%	0.0034%	Bacteria	Firmicutes	Bacilli	Erysipelotrichales	Erysipelotrichaceae	Erysipelothrix	uncultured_Erysipelotrichia
denovo6564	0.0031%	0.0089%	0.0031%	0.0050%	0.0034%	Unassigned						
denovo7734	0.0031%	0.0089%	0.0031%	0.0050%	0.0034%	Unassigned						
denovo8558	0.0031%	0.0089%	0.0031%	0.0050%	0.0034%	Bacteria	Firmicutes	Clostridia	Oscillospirales	Ruminococcaceae	uncultured	
denovo8620	0.0031%	0.0089%	0.0031%	0.0050%	0.0034%	Unassigned						
denovo982	0.0031%	0.0089%	0.0031%	0.0050%	0.0034%	Bacteria	Bacteroidota	Bacteroidia	Bacteroidales	Rikenellaceae	Alistipes	uncultured_Bacteroidales
denovo4152	0.0062%	0.0089%	0.0000%	0.0050%	0.0046%	Bacteria	Spirochaetota	Spirochaetia	Spirochaetales	Spirochaetaceae	Treponema	
denovo5135	0.0062%	0.0089%	0.0000%	0.0050%	0.0046%	Eukaryota						
denovo1986	0.0031%	0.0119%	0.0000%	0.0050%	0.0062%	Bacteria	Firmicutes	Clostridia	Oscillospirales	Ruminococcaceae	uncultured	
denovo7729	0.0031%	0.0119%	0.0000%	0.0050%	0.0062%	Bacteria	Firmicutes	Clostridia	Lachnospirales	Lachnospiraceae	Lachnoclostridium	bacterium_PFB2-27
denovo2071	0.0000%	0.0000%	0.0123%	0.0041%	0.0071%	Bacteria	Bacteroidota	Bacteroidia	Sphingobacteriales	GWF2-29-10	GWF2-29-10	Bacteroidetes_bacterium
denovo3107	0.0000%	0.0000%	0.0123%	0.0041%	0.0071%	Archaea	Euryarchaeota	Methanobacteria	Methanobacteriales	Methanobacteriaceae	Methanobacterium	
denovo5352	0.0000%	0.0000%	0.0123%	0.0041%	0.0071%	Bacteria	Firmicutes	Clostridia	Eubacteriales	Anaerofustaceae	Anaerofustis	
denovo8767	0.0000%	0.0000%	0.0123%	0.0041%	0.0071%	Bacteria	Firmicutes	Syntrophomonadia	Syntrophomonadales	Syntrophomonadaceae	Syntrophomonas	
denovo1350	0.0031%	0.0000%	0.0092%	0.0041%	0.0047%	Bacteria	Bacteroidota	Bacteroidia	Bacteroidales			
denovo1715	0.0031%	0.0000%	0.0092%	0.0041%	0.0047%	Unassigned						
denovo1967	0.0031%	0.0000%	0.0092%	0.0041%	0.0047%	Unassigned						
denovo4883	0.0031%	0.0000%	0.0092%	0.0041%	0.0047%	Unassigned						
denovo6692	0.0031%	0.0000%	0.0092%	0.0041%	0.0047%	Unassigned						
denovo6989	0.0031%	0.0000%	0.0092%	0.0041%	0.0047%	Unassigned						
denovo9040	0.0031%	0.0000%	0.0092%	0.0041%	0.0047%	Bacteria	Spirochaetota	Spirochaetia	Spirochaetales	Spirochaetaceae	uncultured	uncultured_Treponema
denovo2355	0.0062%	0.0000%	0.0062%	0.0041%	0.0036%	Archaea	Halobacterota					
denovo6307	0.0062%	0.0000%	0.0062%	0.0041%	0.0036%	Unassigned						
denovo6816	0.0062%	0.0000%	0.0062%	0.0041%	0.0036%	Unassigned						
denovo6859	0.0062%	0.0000%	0.0062%	0.0041%	0.0036%	Unassigned						
denovo8844	0.0062%	0.0000%	0.0062%	0.0041%	0.0036%	Unassigned						
denovo3031	0.0092%	0.0000%	0.0031%	0.0041%	0.0047%	Unassigned						
denovo5306	0.0092%	0.0000%	0.0031%	0.0041%	0.0047%	Eukaryota						
denovo2189	0.0123%	0.0000%	0.0000%	0.0041%	0.0071%	Bacteria	Caldatribacteriota	JS1	JS1	JS1	JS1	uncultured_organism
denovo6505	0.0123%	0.0000%	0.0000%	0.0041%	0.0071%	Archaea	Halobacterota	Methanosarcinia	Methanosarciniales	Methanosarcinaceae	Methanomethylovorans	uncultured_bacterium
denovo6920	0.0123%	0.0000%	0.0000%	0.0041%	0.0071%	Eukaryota	Parabasalia					
denovo7596	0.0123%	0.0000%	0.0000%	0.0041%	0.0071%	Bacteria	Firmicutes	Clostridia	Oscillospirales	Butyricicoccaceae	UCG-009	uncultured_bacterium
denovo7598	0.0123%	0.0000%	0.0000%	0.0041%	0.0071%	Bacteria	Bacteroidota	Bacteroidia	Bacteroidales	Prolixibacteraceae	Mangrovibacterium	uncultured_bacterium
denovo2292	0.0000%	0.0030%	0.0092%	0.0041%	0.0047%	Bacteria	Firmicutes	Clostridia	Oscillospirales	Oscillospiraceae	Sporobacter	
denovo2755	0.0000%	0.0030%	0.0092%	0.0041%	0.0047%	Bacteria	Firmicutes	Desulfitobacteriia	Desulfitobacteriales	Desulfitobacteriaceae	Desulfosporosinus	
denovo3124	0.0000%	0.0030%	0.0092%	0.0041%	0.0047%	Unassigned						
denovo3188	0.0000%	0.0030%	0.0092%	0.0041%	0.0047%	Unassigned						
denovo3229	0.0000%	0.0030%	0.0092%	0.0041%	0.0047%	Archaea	Euryarchaeota	Methanobacteria	Methanobacteriales	Methanobacteriaceae	Methanobacterium	
denovo3958	0.0000%	0.0030%	0.0092%	0.0041%	0.0047%	Bacteria						
denovo3993	0.0000%	0.0030%	0.0092%	0.0041%	0.0047%	Unassigned						
denovo6353	0.0000%	0.0030%	0.0092%	0.0041%	0.0047%	Unassigned						
denovo6577	0.0000%	0.0030%	0.0092%	0.0041%	0.0047%	Unassigned						
denovo8034	0.0000%	0.0030%	0.0092%	0.0041%	0.0047%	Unassigned						
denovo8546	0.0000%	0.0030%	0.0092%	0.0041%	0.0047%	Unassigned						
denovo1266	0.0031%	0.0030%	0.0062%	0.0041%	0.0018%	Unassigned						
denovo2586	0.0031%	0.0030%	0.0062%	0.0041%	0.0018%	Unassigned						

denovo6980	0.0031%	0.0030%	0.0062%	0.0041%	0.0018%	Bacteria	Proteobacteria	Gammaproteobacteria	Enterobacterales	Enterobacteriaceae		
denovo7637	0.0031%	0.0030%	0.0062%	0.0041%	0.0018%	Bacteria	Desulfobacterota	Desulfovibrionia	Desulfovibrionales	Desulfovibrionaceae	Desulfovibrio	
denovo8502	0.0031%	0.0030%	0.0062%	0.0041%	0.0018%	Unassigned						
denovo8953	0.0031%	0.0030%	0.0062%	0.0041%	0.0018%	Unassigned						
denovo994	0.0031%	0.0030%	0.0062%	0.0041%	0.0018%	Bacteria	Firmicutes	Clostridia				
denovo1820	0.0062%	0.0030%	0.0031%	0.0041%	0.0018%	Bacteria	Firmicutes	Clostridia				
denovo1922	0.0062%	0.0030%	0.0031%	0.0041%	0.0018%	Bacteria	Chloroflexi	Anaerolineae	SJA-15	SJA-15	SJA-15	
denovo2050	0.0062%	0.0030%	0.0031%	0.0041%	0.0018%	Eukaryota						
denovo2249	0.0062%	0.0030%	0.0031%	0.0041%	0.0018%	Unassigned						
denovo2408	0.0062%	0.0030%	0.0031%	0.0041%	0.0018%	Unassigned						
denovo4598	0.0062%	0.0030%	0.0031%	0.0041%	0.0018%	Bacteria	Firmicutes	Bacilli	Acholeplasmatales	Acholeplasmataceae	Acholeplasma	uncultured_compost
denovo5391	0.0062%	0.0030%	0.0031%	0.0041%	0.0018%	Eukaryota						
denovo5812	0.0062%	0.0030%	0.0031%	0.0041%	0.0018%	Unassigned						
denovo7169	0.0062%	0.0030%	0.0031%	0.0041%	0.0018%	Unassigned						
denovo7484	0.0062%	0.0030%	0.0031%	0.0041%	0.0018%	Bacteria	Firmicutes	Clostridia	Christensenellales	Christensenellaceae		
denovo6409	0.0092%	0.0030%	0.0000%	0.0041%	0.0047%	Unassigned						
denovo6688	0.0092%	0.0030%	0.0000%	0.0041%	0.0047%	Bacteria	Bacteroidota	Bacteroidia	Bacteroidales	Dysgonomonadaceae	Petrimonas	
denovo106	0.0000%	0.0059%	0.0062%	0.0040%	0.0035%	Unassigned						
denovo1684	0.0000%	0.0059%	0.0062%	0.0040%	0.0035%	Bacteria	Firmicutes	Clostridia	Christensenellales	Christensenellaceae	Christensenellaceae_R-7_group	
denovo2333	0.0000%	0.0059%	0.0062%	0.0040%	0.0035%	Bacteria	Firmicutes	Clostridia	Oscillospirales			
denovo2921	0.0000%	0.0059%	0.0062%	0.0040%	0.0035%	Unassigned						
denovo3109	0.0000%	0.0059%	0.0062%	0.0040%	0.0035%	Unassigned						
denovo3555	0.0000%	0.0059%	0.0062%	0.0040%	0.0035%	Bacteria	Firmicutes	Negativicutes	Acidaminococcales	Acidaminococcaceae	uncultured	uncultured_Veillonellaceae
denovo4416	0.0000%	0.0059%	0.0062%	0.0040%	0.0035%	Unassigned						
denovo6576	0.0000%	0.0059%	0.0062%	0.0040%	0.0035%	Bacteria	Firmicutes	Clostridia	Oscillospirales			
denovo7184	0.0000%	0.0059%	0.0062%	0.0040%	0.0035%	Unassigned						
denovo7719	0.0000%	0.0059%	0.0062%	0.0040%	0.0035%	Unassigned						
denovo8480	0.0000%	0.0059%	0.0062%	0.0040%	0.0035%	Unassigned						
denovo8682	0.0000%	0.0059%	0.0062%	0.0040%	0.0035%	Eukaryota						
denovo1450	0.0031%	0.0059%	0.0031%	0.0040%	0.0017%	Bacteria						
denovo1805	0.0031%	0.0059%	0.0031%	0.0040%	0.0017%	Unassigned						
denovo1974	0.0031%	0.0059%	0.0031%	0.0040%	0.0017%	Unassigned						
denovo382	0.0031%	0.0059%	0.0031%	0.0040%	0.0017%	Unassigned						
denovo6118	0.0031%	0.0059%	0.0031%	0.0040%	0.0017%	Bacteria	Firmicutes					
denovo6811	0.0031%	0.0059%	0.0031%	0.0040%	0.0017%	Unassigned						
denovo7282	0.0031%	0.0059%	0.0031%	0.0040%	0.0017%	Unassigned						
denovo7701	0.0031%	0.0059%	0.0031%	0.0040%	0.0017%	Bacteria	Desulfobacterota	Desulfovibrionia	Desulfovibrionales	Desulfovibrionaceae	Desulfovibrio	
denovo8270	0.0031%	0.0059%	0.0031%	0.0040%	0.0017%	Unassigned						
denovo3253	0.0062%	0.0059%	0.0000%	0.0040%	0.0035%	Bacteria	Chloroflexi	Anaerolineae	Anaerolineales	Anaerolineaceae		
denovo3764	0.0062%	0.0059%	0.0000%	0.0040%	0.0035%	Bacteria	Chloroflexi	Anaerolineae	Caldilineales	Caldilineaceae	uncultured	
denovo478	0.0062%	0.0059%	0.0000%	0.0040%	0.0035%	Bacteria	Firmicutes	Bacilli	Lactobacillales	Enterococcaceae	Enterococcus	
denovo6329	0.0062%	0.0059%	0.0000%	0.0040%	0.0035%	Bacteria	Bacteroidota	Bacteroidia	Bacteroidales	SB-5	SB-5	
denovo2263	0.0000%	0.0089%	0.0031%	0.0040%	0.0045%	Bacteria	Firmicutes	Clostridia	Clostridia	Hungateiclostridiaceae	HN-HF0106	uncultured_bacterium
denovo2877	0.0000%	0.0089%	0.0031%	0.0040%	0.0045%	Eukaryota						
denovo4974	0.0000%	0.0089%	0.0031%	0.0040%	0.0045%	Unassigned						
denovo5163	0.0000%	0.0089%	0.0031%	0.0040%	0.0045%	Bacteria	Firmicutes	Clostridia	Eubacteriales	Anaerofustaceae	Anaerofustis	uncultured_bacterium
denovo8033	0.0000%	0.0089%	0.0031%	0.0040%	0.0045%	Bacteria	Desulfobacterota	Desulfarculia	Desulfarculales	Desulfarculaceae	Desulfocarbo	uncultured_bacterium
denovo5939	0.0031%	0.0089%	0.0000%	0.0040%	0.0045%	Bacteria	Firmicutes	Clostridia	Christensenellales	Christensenellaceae	Christensenellaceae_R-7_group	uncultured_microorganism
denovo1516	0.0000%	0.0119%	0.0000%	0.0040%	0.0069%	Unassigned						
denovo2494	0.0000%	0.0119%	0.0000%	0.0040%	0.0069%	Bacteria	Synergistota	Synergistia	Synergistales	Synergistaceae	uncultured	uncultured_Aminobacterium
denovo8015	0.0000%	0.0119%	0.0000%	0.0040%	0.0069%	Unassigned						
denovo1484	0.0000%	0.0000%	0.0092%	0.0031%	0.0053%	Unassigned						
denovo2028	0.0000%	0.0000%	0.0092%	0.0031%	0.0053%	Bacteria	Firmicutes	Clostridia	Oscillospirales	Butyricicoccaceae	UCG-009	
denovo284	0.0000%	0.0000%	0.0092%	0.0031%	0.0053%	Bacteria	Firmicutes	Clostridia	Clostridia	Gracilibacteraceae	Gracilibacter	
denovo3074	0.0000%	0.0000%	0.0092%	0.0031%	0.0053%	Bacteria						
denovo4374	0.0000%	0.0000%	0.0092%	0.0031%	0.0053%	Bacteria	Firmicutes	Clostridia	Oscillospirales	Ruminococcaceae	uncultured	
denovo5713	0.0000%	0.0000%	0.0092%	0.0031%	0.0053%	Bacteria	Firmicutes	Clostridia				
denovo6291	0.0000%	0.0000%	0.0092%	0.0031%	0.0053%	Bacteria	Bacteroidota	Bacteroidia	Bacteroidales	Dysgonomonadaceae		
denovo6311	0.0000%	0.0000%	0.0092%	0.0031%	0.0053%	Bacteria	Bacteroidota	Bacteroidia	Bacteroidales	Prolixibacteraceae	Meniscus	uncultured_bacterium
denovo7367	0.0000%	0.0000%	0.0092%	0.0031%	0.0053%	Bacteria	Firmicutes	Clostridia	Oscillospirales			

denovo7641	0.0000%	0.0000%	0.0092%	0.0031%	0.0053%	Unassigned						
denovo8535	0.0000%	0.0000%	0.0092%	0.0031%	0.0053%	Bacteria	Proteobacteria	Gammaproteobacteria	Pseudomonadales	Moraxellaceae	Acinetobacter	
denovo1566	0.0031%	0.0000%	0.0062%	0.0031%	0.0031%	Unassigned						
denovo2084	0.0031%	0.0000%	0.0062%	0.0031%	0.0031%	Bacteria	Firmicutes	Clostridia	Peptococcales	Peptococcaceae	uncultured	
denovo2638	0.0031%	0.0000%	0.0062%	0.0031%	0.0031%	Bacteria	Proteobacteria	Gammaproteobacteria	Burkholderiales	Alcaligenaceae	Derxia	uncultured_Derxia
denovo3596	0.0031%	0.0000%	0.0062%	0.0031%	0.0031%	Bacteria						
denovo3779	0.0031%	0.0000%	0.0062%	0.0031%	0.0031%	Bacteria	Firmicutes	Syntrophomonadia	Syntrophomonadales	Syntrophomonadaceae		
denovo4924	0.0031%	0.0000%	0.0062%	0.0031%	0.0031%	Unassigned						
denovo5374	0.0031%	0.0000%	0.0062%	0.0031%	0.0031%	Unassigned						
denovo6065	0.0031%	0.0000%	0.0062%	0.0031%	0.0031%	Bacteria	Proteobacteria	Gammaproteobacteria	Burkholderiales	Hydrogenophilaceae	Thiobacillus	
denovo7173	0.0031%	0.0000%	0.0062%	0.0031%	0.0031%	Bacteria	Desulfobacterota	Desulfovibrionia	Desulfovibrionales	Desulfovibrionaceae	Desulfovibrio	uncultured_Firmicutes
denovo7601	0.0031%	0.0000%	0.0062%	0.0031%	0.0031%	Bacteria	Proteobacteria	Alphaproteobacteria	Rhizobiales	Rhizobiaceae	Aquamicrobium	
denovo8397	0.0031%	0.0000%	0.0062%	0.0031%	0.0031%	Bacteria	Firmicutes	Clostridia	Christensenellales	Christensenellaceae		
denovo9059	0.0031%	0.0000%	0.0062%	0.0031%	0.0031%	Bacteria	Firmicutes	Clostridia	Lachnospirales	Lachnospiraceae	Lachnospiraceae_UCG-010	uncultured_rumen
denovo1837	0.0062%	0.0000%	0.0031%	0.0031%	0.0031%	Unassigned						
denovo2253	0.0062%	0.0000%	0.0031%	0.0031%	0.0031%	Unassigned						
denovo3322	0.0062%	0.0000%	0.0031%	0.0031%	0.0031%	Bacteria						
denovo5104	0.0062%	0.0000%	0.0031%	0.0031%	0.0031%	Bacteria	Firmicutes	Clostridia	Oscillospirales	Ruminococcaceae	Incertae_Sedis	
denovo5115	0.0062%	0.0000%	0.0031%	0.0031%	0.0031%	Unassigned						
denovo5960	0.0062%	0.0000%	0.0031%	0.0031%	0.0031%	Bacteria	Synergistota	Synergistia	Synergistales	Synergistaceae		
denovo6639	0.0062%	0.0000%	0.0031%	0.0031%	0.0031%	Unassigned						
denovo6906	0.0062%	0.0000%	0.0031%	0.0031%	0.0031%	Bacteria	Firmicutes	Clostridia	Christensenellales	Christensenellaceae	Christensenellaceae_R-7_group	
denovo6951	0.0062%	0.0000%	0.0031%	0.0031%	0.0031%	Bacteria	Desulfobacterota	Desulfobacteria	Desulfobacterales	Desulfosarcinaceae	Sva0081_sediment_group	
denovo7336	0.0062%	0.0000%	0.0031%	0.0031%	0.0031%	Bacteria	Bacteroidota	Ignavibacteria	Ignavibacteriales	Ignavibacteriaceae	Ignavibacterium	uncultured_bacterium
denovo7479	0.0062%	0.0000%	0.0031%	0.0031%	0.0031%	Bacteria	Bacteroidota	Bacteroidia	Bacteroidales	Dysgonomonadaceae		
denovo7787	0.0062%	0.0000%	0.0031%	0.0031%	0.0031%	Bacteria	Armatimonadota	uncultured	uncultured	uncultured	uncultured	
denovo8200	0.0062%	0.0000%	0.0031%	0.0031%	0.0031%	Bacteria	Firmicutes	Clostridia	Christensenellales	Christensenellaceae		
denovo84	0.0062%	0.0000%	0.0031%	0.0031%	0.0031%	Bacteria	Desulfobacterota	Syntrophorhabdia	Syntrophorhabdales	Syntrophorhabdaceae	Syntrophorhabdus	
denovo1439	0.0092%	0.0000%	0.0000%	0.0031%	0.0053%	Bacteria	Firmicutes	Clostridia	Oscillospirales	UCG-010	UCG-010	
denovo2295	0.0092%	0.0000%	0.0000%	0.0031%	0.0053%	Bacteria	Chloroflexi	Anaerolineae	Anaerolineales	Anaerolineaceae		
denovo2531	0.0092%	0.0000%	0.0000%	0.0031%	0.0053%	Bacteria	Firmicutes	Clostridia	Christensenellales	Christensenellaceae	uncultured	
denovo310	0.0092%	0.0000%	0.0000%	0.0031%	0.0053%	Bacteria	Chloroflexi	Anaerolineae	Anaerolineales	Anaerolineaceae	Pelolinea	uncultured_bacterium
denovo4123	0.0092%	0.0000%	0.0000%	0.0031%	0.0053%	Bacteria	Chloroflexi	Anaerolineae	Anaerolineales	Anaerolineaceae	Flexilinea	
denovo5427	0.0092%	0.0000%	0.0000%	0.0031%	0.0053%	Archaea	Halobacterota	Methanomicrobia	Methanomicrobiales	Methanomicrobiaceae	Methanoculleus	
denovo7502	0.0092%	0.0000%	0.0000%	0.0031%	0.0053%	Unassigned						
denovo783	0.0092%	0.0000%	0.0000%	0.0031%	0.0053%	Archaea	Euryarchaeota	Methanobacteria	Methanobacteriales	Methanobacteriaceae	Methanobacterium	archaeon_enrichment
denovo8109	0.0092%	0.0000%	0.0000%	0.0031%	0.0053%	Bacteria	Firmicutes	Clostridia	Clostridiales	Clostridiaceae	Clostridium_sensu_stricto_7	
denovo3065	0.0000%	0.0030%	0.0062%	0.0030%	0.0031%	Unassigned						
denovo3670	0.0000%	0.0030%	0.0062%	0.0030%	0.0031%	Bacteria	Firmicutes	Clostridia	Oscillospirales			
denovo3990	0.0000%	0.0030%	0.0062%	0.0030%	0.0031%	Unassigned						
denovo4304	0.0000%	0.0030%	0.0062%	0.0030%	0.0031%	Unassigned						
denovo5438	0.0000%	0.0030%	0.0062%	0.0030%	0.0031%	Bacteria	Firmicutes	Clostridia	Christensenellales	Christensenellaceae	uncultured	
denovo5638	0.0000%	0.0030%	0.0062%	0.0030%	0.0031%	Unassigned						
denovo8080	0.0000%	0.0030%	0.0062%	0.0030%	0.0031%	Bacteria	Desulfobacterota	Desulfovibrionia	Desulfovibrionales	Desulfovibrionaceae	Desulfovibrio	uncultured_Firmicutes
denovo8508	0.0000%	0.0030%	0.0062%	0.0030%	0.0031%	Unassigned						
denovo1332	0.0031%	0.0030%	0.0031%	0.0030%	0.0001%	Bacteria	Nitrospirota	Nitrospiria	Nitrospirales	Nitrospiraceae	Nitrospira	
denovo1828	0.0031%	0.0030%	0.0031%	0.0030%	0.0001%	Unassigned						
denovo2187	0.0031%	0.0030%	0.0031%	0.0030%	0.0001%	Bacteria	Proteobacteria	Gammaproteobacteria	Enterobacterales	Enterobacteriaceae		
denovo3996	0.0031%	0.0030%	0.0031%	0.0030%	0.0001%	Bacteria	Elusimicrobiota	Elusimicrobia	Elusimicrobiales	Elusimicrobiaceae	Elusimicrobium	uncultured_bacterium
denovo4011	0.0031%	0.0030%	0.0031%	0.0030%	0.0001%	Bacteria	Firmicutes	Clostridia	Peptococcales	Peptococcaceae	uncultured	
denovo4045	0.0031%	0.0030%	0.0031%	0.0030%	0.0001%	Unassigned						
denovo4967	0.0031%	0.0030%	0.0031%	0.0030%	0.0001%	Archaea	Euryarchaeota	Methanobacteria	Methanobacteriales	Methanobacteriaceae	Methanobacterium	
denovo5307	0.0031%	0.0030%	0.0031%	0.0030%	0.0001%	Unassigned						
denovo5926	0.0031%	0.0030%	0.0031%	0.0030%	0.0001%	Unassigned						
denovo6205	0.0031%	0.0030%	0.0031%	0.0030%	0.0001%	Bacteria	Firmicutes	Clostridia				
denovo642	0.0031%	0.0030%	0.0031%	0.0030%	0.0001%	Bacteria	Firmicutes	Clostridia	Christensenellales	Christensenellaceae	Christensenellaceae_R-7_group	
denovo7081	0.0031%	0.0030%	0.0031%	0.0030%	0.0001%	Bacteria	Firmicutes	Clostridia	Oscillospirales	Ruminococcaceae	uncultured	uncultured_Firmicutes
denovo7145	0.0031%	0.0030%	0.0031%	0.0030%	0.0001%	Bacteria	Proteobacteria	Gammaproteobacteria	Burkholderiales	Nitrosomonadaceae	Nitrosomonas	
denovo7440	0.0031%	0.0030%	0.0031%	0.0030%	0.0001%	Bacteria	Synergistota	Synergistia	Synergistales	Synergistaceae	uncultured	bacterium_enrichment
denovo7809	0.0031%	0.0030%	0.0031%	0.0030%	0.0001%	Bacteria	Firmicutes	Clostridia	Peptococcales	Peptococcaceae	uncultured	uncultured_bacterium

denovo8264	0.0031%	0.0030%	0.0031%	0.0030%	0.0001%	Bacteria	Synergistota	Synergistia	Synergistales	Synergistaceae	Syner-01	
denovo2239	0.0062%	0.0030%	0.0000%	0.0030%	0.0031%	Bacteria	Bacteroidota	Bacteroidia	Bacteroidales	Bacteroidetes_BD2-2	Bacteroidetes_BD2-2	
denovo2577	0.0062%	0.0030%	0.0000%	0.0030%	0.0031%	Bacteria	Desulfobacterota	Syntrophia	Syntrophales	uncultured	uncultured	
denovo4591	0.0062%	0.0030%	0.0000%	0.0030%	0.0031%	Unassigned						
denovo4654	0.0062%	0.0030%	0.0000%	0.0030%	0.0031%	Bacteria	Firmicutes	Clostridia	Peptococcales	Peptococcaceae	uncultured	
denovo494	0.0062%	0.0030%	0.0000%	0.0030%	0.0031%	Bacteria	Firmicutes	Clostridia	Oscillospirales	Ruminococcaceae	UBA1819	
denovo5556	0.0062%	0.0030%	0.0000%	0.0030%	0.0031%	Unassigned						
denovo5611	0.0062%	0.0030%	0.0000%	0.0030%	0.0031%	Unassigned						
denovo5964	0.0062%	0.0030%	0.0000%	0.0030%	0.0031%	Unassigned						
denovo6464	0.0062%	0.0030%	0.0000%	0.0030%	0.0031%	Unassigned						
denovo6623	0.0062%	0.0030%	0.0000%	0.0030%	0.0031%	Bacteria	Proteobacteria	Gammaproteobacteria	Burkholderiales	Burkholderiaceae	Limnobacter	
denovo6922	0.0062%	0.0030%	0.0000%	0.0030%	0.0031%	Bacteria	Synergistota	Synergistia	Synergistales	Synergistaceae	uncultured	bacterium_enrichment
denovo7592	0.0062%	0.0030%	0.0000%	0.0030%	0.0031%	Bacteria	Firmicutes	Clostridia	Christensenellales	Christensenellaceae		
denovo7619	0.0062%	0.0030%	0.0000%	0.0030%	0.0031%	Bacteria	Chloroflexi	Anaerolineae	Anaerolineales	Anaerolineaceae	Flexilinea	
denovo7783	0.0062%	0.0030%	0.0000%	0.0030%	0.0031%	Bacteria	Bacteroidota	Bacteroidia	Bacteroidales	SB-5	SB-5	
denovo8271	0.0062%	0.0030%	0.0000%	0.0030%	0.0031%	Unassigned						
denovo4736	0.0000%	0.0059%	0.0031%	0.0030%	0.0030%	Bacteria	Proteobacteria	Gammaproteobacteria	Enterobacterales	Enterobacteriaceae		
denovo5154	0.0000%	0.0059%	0.0031%	0.0030%	0.0030%	Bacteria						
denovo6072	0.0000%	0.0059%	0.0031%	0.0030%	0.0030%	Unassigned						
denovo7545	0.0000%	0.0059%	0.0031%	0.0030%	0.0030%	Bacteria	Firmicutes	Clostridia	Peptococcales	Peptococcaceae	uncultured	
denovo8563	0.0000%	0.0059%	0.0031%	0.0030%	0.0030%	Bacteria	Firmicutes	Clostridia	Oscillospirales	Ruminococcaceae	Candidatus_Soleaferrea	
denovo8830	0.0000%	0.0059%	0.0031%	0.0030%	0.0030%	Unassigned						
denovo8861	0.0000%	0.0059%	0.0031%	0.0030%	0.0030%	Bacteria	Firmicutes	Clostridia				
denovo15	0.0031%	0.0059%	0.0000%	0.0030%	0.0030%	Archaea	Euryarchaeota	Methanobacteria	Methanobacteriales	Methanobacteriaceae	Methanobacterium	uncultured_Methanobacteriales
denovo2392	0.0031%	0.0059%	0.0000%	0.0030%	0.0030%	Unassigned						
denovo7084	0.0031%	0.0059%	0.0000%	0.0030%	0.0030%	Unassigned						
denovo8275	0.0031%	0.0059%	0.0000%	0.0030%	0.0030%	Bacteria	Firmicutes	Clostridia	Peptococcales	Peptococcaceae	uncultured	uncultured_bacterium
denovo8456	0.0031%	0.0059%	0.0000%	0.0030%	0.0030%	Unassigned						
denovo2046	0.0000%	0.0089%	0.0000%	0.0030%	0.0052%	Unassigned						
denovo3375	0.0000%	0.0089%	0.0000%	0.0030%	0.0052%	Bacteria	Firmicutes	Clostridia	Oscillospirales	Ruminococcaceae		
denovo3735	0.0000%	0.0089%	0.0000%	0.0030%	0.0052%	Bacteria	Firmicutes	Desulfotomaculia	Desulfotomaculales	Desulfotomaculales	Cryptanaerobacter	uncultured_bacterium
denovo8267	0.0000%	0.0089%	0.0000%	0.0030%	0.0052%	Bacteria	Firmicutes	D8A-2	D8A-2	D8A-2	D8A-2	uncultured_Thermoanaerobacteraceae
denovo1167	0.0000%	0.0000%	0.0062%	0.0021%	0.0036%	Bacteria	Firmicutes	Clostridia	Oscillospirales	Oscillospiraceae		
denovo1278	0.0000%	0.0000%	0.0062%	0.0021%	0.0036%	Bacteria	Firmicutes	Clostridia	Peptostreptococcales-Tissierellales	Anaerovoracaceae	Anaerovorax	
denovo1407	0.0000%	0.0000%	0.0062%	0.0021%	0.0036%	Unassigned						
denovo1415	0.0000%	0.0000%	0.0062%	0.0021%	0.0036%	Bacteria	Proteobacteria	Gammaproteobacteria	Burkholderiales	Comamonadaceae		
denovo155	0.0000%	0.0000%	0.0062%	0.0021%	0.0036%	Unassigned						
denovo1806	0.0000%	0.0000%	0.0062%	0.0021%	0.0036%	Bacteria	Proteobacteria	Alphaproteobacteria	Rhizobiales	Xanthobacteraceae		
denovo1971	0.0000%	0.0000%	0.0062%	0.0021%	0.0036%	Bacteria	Firmicutes	Clostridia				
denovo2107	0.0000%	0.0000%	0.0062%	0.0021%	0.0036%	Bacteria	Spirochaetota	Spirochaetia	Spirochaetales	Spirochaetaceae	uncultured	uncultured_Treponema
denovo2201	0.0000%	0.0000%	0.0062%	0.0021%	0.0036%	Bacteria						
denovo3078	0.0000%	0.0000%	0.0062%	0.0021%	0.0036%	Unassigned						
denovo381	0.0000%	0.0000%	0.0062%	0.0021%	0.0036%	Bacteria	Firmicutes	Clostridia	Christensenellales	Christensenellaceae	Christensenellaceae_R-7_group	uncultured_marine
denovo3948	0.0000%	0.0000%	0.0062%	0.0021%	0.0036%	Bacteria	Firmicutes	Clostridia				
denovo401	0.0000%	0.0000%	0.0062%	0.0021%	0.0036%	Unassigned						
denovo4038	0.0000%	0.0000%	0.0062%	0.0021%	0.0036%	Unassigned						
denovo4049	0.0000%	0.0000%	0.0062%	0.0021%	0.0036%	Bacteria	Firmicutes	Clostridia	Oscillospirales			
denovo4248	0.0000%	0.0000%	0.0062%	0.0021%	0.0036%	Bacteria	Synergistota	Synergistia	Synergistales	Synergistaceae	uncultured	bacterium_enrichment
denovo4587	0.0000%	0.0000%	0.0062%	0.0021%	0.0036%	Unassigned						
denovo4644	0.0000%	0.0000%	0.0062%	0.0021%	0.0036%	Bacteria	Synergistota	Synergistia	Synergistales	Synergistaceae	uncultured	uncultured_bacterium
denovo4659	0.0000%	0.0000%	0.0062%	0.0021%	0.0036%	Bacteria	Firmicutes	Clostridia	Oscillospirales	Ruminococcaceae	uncultured	
denovo470	0.0000%	0.0000%	0.0062%	0.0021%	0.0036%	Bacteria	Firmicutes	Syntrophomonadia	Syntrophomonadales	Syntrophomonadaceae		
denovo4704	0.0000%	0.0000%	0.0062%	0.0021%	0.0036%	Unassigned						
denovo5267	0.0000%	0.0000%	0.0062%	0.0021%	0.0036%	Bacteria	Firmicutes	Bacilli	Erysipelotrichales	Erysipelatoclostridiaceae	UCG-004	uncultured_bacterium
denovo5296	0.0000%	0.0000%	0.0062%	0.0021%	0.0036%	Archaea	Euryarchaeota	Methanobacteria	Methanobacteriales	Methanobacteriaceae	Methanobacterium	uncultured_Methanobacteriales
denovo5483	0.0000%	0.0000%	0.0062%	0.0021%	0.0036%	Bacteria	Firmicutes	Clostridia	Oscillospirales	Oscillospiraceae		
denovo5505	0.0000%	0.0000%	0.0062%	0.0021%	0.0036%	Bacteria	Firmicutes	Negativicutes	Veillonellales-Selenomonadales	Sporomusaceae	Anaeromusa-Anaeroarcus	
denovo561	0.0000%	0.0000%	0.0062%	0.0021%	0.0036%	Bacteria	Firmicutes	Clostridia	Christensenellales	Christensenellaceae	Christensenellaceae_R-7_group	
denovo5728	0.0000%	0.0000%	0.0062%	0.0021%	0.0036%	Unassigned						
denovo5851	0.0000%	0.0000%	0.0062%	0.0021%	0.0036%	Bacteria	Firmicutes	Clostridia	Peptococcales	Peptococcaceae	uncultured	

denovo6256	0.0000%	0.0000%	0.0062%	0.0021%	0.0036%	Unassigned						
denovo6746	0.0000%	0.0000%	0.0062%	0.0021%	0.0036%	Unassigned						
denovo682	0.0000%	0.0000%	0.0062%	0.0021%	0.0036%	Eukaryota						
denovo6826	0.0000%	0.0000%	0.0062%	0.0021%	0.0036%	Bacteria	Firmicutes	Clostridia	Clostridia_vadinBB60_group	Clostridia_vadinBB60_group	Clostridia_vadinBB60_group	uncultured_bacterium
denovo7552	0.0000%	0.0000%	0.0062%	0.0021%	0.0036%	Bacteria	Firmicutes	Clostridia	Oscillospirales	Ruminococcaceae	Candidatus_Soleaferrea	
denovo773	0.0000%	0.0000%	0.0062%	0.0021%	0.0036%	Bacteria						
denovo7866	0.0000%	0.0000%	0.0062%	0.0021%	0.0036%	Bacteria	Sumerlaeota	Sumerlaeia	Sumerlaeales	Sumerlaeaceae	Sumerlaea	uncultured_bacterium
denovo7983	0.0000%	0.0000%	0.0062%	0.0021%	0.0036%	Bacteria	Firmicutes	Clostridia	Oscillospirales	Oscillospiraceae		
denovo8196	0.0000%	0.0000%	0.0062%	0.0021%	0.0036%	Archaea	Halobacterota					
denovo8299	0.0000%	0.0000%	0.0062%	0.0021%	0.0036%	Bacteria	Bacteroidota	Bacteroidia	Bacteroidales	Barnesiellaceae	uncultured	Bacteroidales_bacterium
denovo83	0.0000%	0.0000%	0.0062%	0.0021%	0.0036%	Unassigned						
denovo8425	0.0000%	0.0000%	0.0062%	0.0021%	0.0036%	Bacteria	Firmicutes	Syntrophomonadia	Syntrophomonadales	Syntrophomonadaceae	Syntrophomonas	
denovo8489	0.0000%	0.0000%	0.0062%	0.0021%	0.0036%	Unassigned						
denovo8806	0.0000%	0.0000%	0.0062%	0.0021%	0.0036%	Bacteria	Firmicutes	Clostridia	Lachnospirales	Lachnospiraceae	[Eubacterium]_fissicatena_group	
denovo8875	0.0000%	0.0000%	0.0062%	0.0021%	0.0036%	Bacteria	Proteobacteria	Gammaproteobacteria	Burkholderiales	Comamonadaceae		
denovo89	0.0000%	0.0000%	0.0062%	0.0021%	0.0036%	Archaea	Euryarchaeota	Methanobacteria	Methanobacteriales	Methanobacteriaceae	Methanobacterium	
denovo8945	0.0000%	0.0000%	0.0062%	0.0021%	0.0036%	Bacteria	Firmicutes	Syntrophomonadia	Syntrophomonadales	Syntrophomonadaceae	Syntrophomonas	
denovo1166	0.0031%	0.0000%	0.0031%	0.0021%	0.0018%	Bacteria	Firmicutes	Clostridia	Oscillospirales			
denovo1273	0.0031%	0.0000%	0.0031%	0.0021%	0.0018%	Unassigned						
denovo168	0.0031%	0.0000%	0.0031%	0.0021%	0.0018%	Eukaryota						
denovo1772	0.0031%	0.0000%	0.0031%	0.0021%	0.0018%	Bacteria	Synergistota	Synergistia	Synergistales	Synergistaceae	Syner-01	uncultured_bacterium
denovo2266	0.0031%	0.0000%	0.0031%	0.0021%	0.0018%	Bacteria	Desulfobacterota	Desulfovibrionia	Desulfovibrionales	Desulfovibrionaceae	Desulfovibrio	
denovo2668	0.0031%	0.0000%	0.0031%	0.0021%	0.0018%	Unassigned						
denovo3117	0.0031%	0.0000%	0.0031%	0.0021%	0.0018%	Archaea	Halobacterota	Methanosarcinia	Methanosarciniales	Methanosaetaceae	Methanosaeta	
denovo3226	0.0031%	0.0000%	0.0031%	0.0021%	0.0018%	Bacteria	Bacteroidota	Bacteroidia	Bacteroidales	Bacteroidetes_vadinHA17	Bacteroidetes_vadinHA17	uncultured_bacterium
denovo3282	0.0031%	0.0000%	0.0031%	0.0021%	0.0018%	Archaea	Euryarchaeota	Methanobacteria	Methanobacteriales	Methanobacteriaceae	Methanobrevibacter	Methanobrevibacter_arboriphilus
denovo3797	0.0031%	0.0000%	0.0031%	0.0021%	0.0018%	Bacteria	Synergistota	Synergistia	Synergistales	Synergistaceae	uncultured	bacterium_enrichment
denovo3889	0.0031%	0.0000%	0.0031%	0.0021%	0.0018%	Bacteria	Planctomycetota	vadinHA49	vadinHA49	vadinHA49	vadinHA49	uncultured_Planctomycetaceae
denovo397	0.0031%	0.0000%	0.0031%	0.0021%	0.0018%	Unassigned						
denovo3976	0.0031%	0.0000%	0.0031%	0.0021%	0.0018%	Bacteria	Firmicutes	Clostridia	Peptococcales	Peptococcaceae	uncultured	uncultured_Peptococcaceae
denovo4193	0.0031%	0.0000%	0.0031%	0.0021%	0.0018%	Bacteria	Verrucomicrobiota	Verrucomicrobiae	Opitutales	Puniceicoccaceae		
denovo4233	0.0031%	0.0000%	0.0031%	0.0021%	0.0018%	Bacteria	Firmicutes	Clostridia	Clostridia	Gracilibacteraceae	Lutispora	
denovo4257	0.0031%	0.0000%	0.0031%	0.0021%	0.0018%	Bacteria	Firmicutes	Clostridia	Oscillospirales	Ruminococcaceae	uncultured	uncultured_Firmicutes
denovo4502	0.0031%	0.0000%	0.0031%	0.0021%	0.0018%	Bacteria	Desulfobacterota	Desulfovibrionia	Desulfovibrionales	Desulfovibrionaceae	Desulfovibrio	Desulfovibrio_aminophilus
denovo4519	0.0031%	0.0000%	0.0031%	0.0021%	0.0018%	Bacteria	Firmicutes	Syntrophomonadia	Syntrophomonadales	Syntrophomonadaceae	uncultured	uncultured_Syntrophomonadaceae
denovo4719	0.0031%	0.0000%	0.0031%	0.0021%	0.0018%	Bacteria	Firmicutes	Clostridia				
denovo4793	0.0031%	0.0000%	0.0031%	0.0021%	0.0018%	Bacteria	Firmicutes	Clostridia	Oscillospirales	[Eubacterium]_coprostanoligenes_group	[Eubacterium]_coprostanoligenes_group	uncultured_Clostridia
denovo483	0.0031%	0.0000%	0.0031%	0.0021%	0.0018%	Bacteria	Firmicutes	Clostridia				
denovo5182	0.0031%	0.0000%	0.0031%	0.0021%	0.0018%	Unassigned						
denovo5372	0.0031%	0.0000%	0.0031%	0.0021%	0.0018%	Unassigned						
denovo5749	0.0031%	0.0000%	0.0031%	0.0021%	0.0018%	Bacteria	Firmicutes	Clostridia	Peptococcales	Peptococcaceae	uncultured	
denovo6282	0.0031%	0.0000%	0.0031%	0.0021%	0.0018%	Unassigned						
denovo6348	0.0031%	0.0000%	0.0031%	0.0021%	0.0018%	Bacteria	Firmicutes	Clostridia	Oscillospirales	Ruminococcaceae	uncultured	
denovo6563	0.0031%	0.0000%	0.0031%	0.0021%	0.0018%	Bacteria	Firmicutes	Syntrophomonadia	Syntrophomonadales	Syntrophomonadaceae	Syntrophomonas	
denovo7316	0.0031%	0.0000%	0.0031%	0.0021%	0.0018%	Unassigned						
denovo7406	0.0031%	0.0000%	0.0031%	0.0021%	0.0018%	Bacteria	Bacteroidota	Bacteroidia	Bacteroidales	Bacteroidetes_vadinHA17	Bacteroidetes_vadinHA17	metagenome
denovo7768	0.0031%	0.0000%	0.0031%	0.0021%	0.0018%	Bacteria	Bacteroidota	Bacteroidia	Bacteroidales	Paludibacteraceae	Paludibacter	uncultured_Bacteroides
denovo7794	0.0031%	0.0000%	0.0031%	0.0021%	0.0018%	Bacteria	Firmicutes	Syntrophomonadia	Syntrophomonadales	Syntrophomonadaceae	Syntrophomonas	
denovo8140	0.0031%	0.0000%	0.0031%	0.0021%	0.0018%	Bacteria	Firmicutes	Clostridia	Christensenellales	Christensenellaceae	uncultured	uncultured_Firmicutes
denovo8371	0.0031%	0.0000%	0.0031%	0.0021%	0.0018%	Unassigned						
denovo8903	0.0031%	0.0000%	0.0031%	0.0021%	0.0018%	Bacteria	Firmicutes					
denovo8935	0.0031%	0.0000%	0.0031%	0.0021%	0.0018%	Bacteria	Firmicutes	Bacilli	Erysipelotrichales	Erysipelotrichaceae	[Anaerorhabdus]_furcosa_group	
denovo1058	0.0062%	0.0000%	0.0000%	0.0021%	0.0036%	Bacteria	Spirochaetota	Spirochaetia	Spirochaetales	Spirochaetaceae	uncultured	
denovo1420	0.0062%	0.0000%	0.0000%	0.0021%	0.0036%	Bacteria	Bacteroidota	Bacteroidia	Bacteroidales	Williamwhitmaniaceae	Blvii28_wastewater-sludge_group	uncultured_bacterium
denovo1711	0.0062%	0.0000%	0.0000%	0.0021%	0.0036%	Unassigned						
denovo2343	0.0062%	0.0000%	0.0000%	0.0021%	0.0036%	Bacteria	Caldatribacteriota	JS1	JS1	JS1	JS1	
denovo292	0.0062%	0.0000%	0.0000%	0.0021%	0.0036%	Unassigned						
denovo3052	0.0062%	0.0000%	0.0000%	0.0021%	0.0036%	Archaea	Euryarchaeota	Methanobacteria	Methanobacteriales	Methanobacteriaceae	Methanobacterium	archaeon_enrichment
denovo3088	0.0062%	0.0000%	0.0000%	0.0021%	0.0036%	Bacteria	Chloroflexi	Anaerolineae	Anaerolineales	Anaerolineaceae	Flexilinea	
denovo3095	0.0062%	0.0000%	0.0000%	0.0021%	0.0036%	Bacteria	Caldatribacteriota	JS1	JS1	JS1	JS1	uncultured_organism

denovo3213	0.0062%	0.0000%	0.0000%	0.0021%	0.0036%	Bacteria	Synergistota	Synergistia	Synergistales	Synergistaceae		
denovo3525	0.0062%	0.0000%	0.0000%	0.0021%	0.0036%	Unassigned						
denovo417	0.0062%	0.0000%	0.0000%	0.0021%	0.0036%	Bacteria	Planctomycetota	Phycisphaerae	MSBL9	SM23-30	SM23-30	uncultured_bacterium
denovo4400	0.0062%	0.0000%	0.0000%	0.0021%	0.0036%	Unassigned						
denovo5320	0.0062%	0.0000%	0.0000%	0.0021%	0.0036%	Bacteria	Firmicutes	Clostridia	Lachnospirales	Lachnospiraceae		
denovo559	0.0062%	0.0000%	0.0000%	0.0021%	0.0036%	Bacteria	Firmicutes	Desulfotomaculia	Desulfotomaculales	Desulfotomaculales	Desulfurispora	uncultured_bacterium
denovo5772	0.0062%	0.0000%	0.0000%	0.0021%	0.0036%	Bacteria	Proteobacteria	Alphaproteobacteria	Caulobacterales	Caulobacteraceae	Caulobacter	
denovo597	0.0062%	0.0000%	0.0000%	0.0021%	0.0036%	Bacteria	Desulfobacterota	Desulfovibrionia	Desulfovibrionales	Desulfovibrionaceae	Desulfovibrio	
denovo6050	0.0062%	0.0000%	0.0000%	0.0021%	0.0036%	Unassigned						
denovo6207	0.0062%	0.0000%	0.0000%	0.0021%	0.0036%	Bacteria	Firmicutes	Clostridia	Oscillospirales	Oscillospirales	Hydrogenoanaerobacterium	uncultured_bacterium
denovo6436	0.0062%	0.0000%	0.0000%	0.0021%	0.0036%	Bacteria	Patescibacteria	Parcubacteria	Candidatus_Nomurabacteria	Candidatus_Nomurabacteria	Candidatus_Nomurabacteria	uncultured_bacterium
denovo6705	0.0062%	0.0000%	0.0000%	0.0021%	0.0036%	Bacteria	Desulfobacterota	Desulfobulbia	Desulfobulbales	Desulfobulbaceae	Desulfobulbus	
denovo7305	0.0062%	0.0000%	0.0000%	0.0021%	0.0036%	Bacteria	Chloroflexi	Anaerolineae	Anaerolineales	Anaerolineaceae	uncultured	
denovo7385	0.0062%	0.0000%	0.0000%	0.0021%	0.0036%	Unassigned						
denovo7549	0.0062%	0.0000%	0.0000%	0.0021%	0.0036%	Bacteria	Firmicutes	Clostridia	Christensenellales	Christensenellaceae	Christensenellaceae_R-7_group	
denovo7911	0.0062%	0.0000%	0.0000%	0.0021%	0.0036%	Bacteria	Desulfobacterota	Syntrophia	Syntrophales	Smithellaceae	Smithella	
denovo7967	0.0062%	0.0000%	0.0000%	0.0021%	0.0036%	Bacteria	Chloroflexi	Anaerolineae	RBG-13-54-9	RBG-13-54-9	RBG-13-54-9	uncultured_bacterium
denovo8073	0.0062%	0.0000%	0.0000%	0.0021%	0.0036%	Bacteria	Synergistota	Synergistia	Synergistales	Synergistaceae		
denovo8751	0.0062%	0.0000%	0.0000%	0.0021%	0.0036%	Bacteria	Chloroflexi	Anaerolineae	Anaerolineales	Anaerolineaceae	uncultured	
denovo898	0.0062%	0.0000%	0.0000%	0.0021%	0.0036%	Bacteria	Planctomycetota	Planctomycetes	Planctomycetales	Rubinisphaeraceae	Planctomicrobium	
denovo1144	0.0000%	0.0030%	0.0031%	0.0020%	0.0017%	Unassigned						
denovo1251	0.0000%	0.0030%	0.0031%	0.0020%	0.0017%	Bacteria	Firmicutes	Clostridia	Lachnospirales	Lachnospiraceae		
denovo1335	0.0000%	0.0030%	0.0031%	0.0020%	0.0017%	Bacteria	Firmicutes	Clostridia	Oscillospirales			
denovo160	0.0000%	0.0030%	0.0031%	0.0020%	0.0017%	Archaea	Halobacterota					
denovo2209	0.0000%	0.0030%	0.0031%	0.0020%	0.0017%	Bacteria	Firmicutes	Clostridia				
denovo2267	0.0000%	0.0030%	0.0031%	0.0020%	0.0017%	Bacteria	Bacteroidota	Bacteroidia	Bacteroidales	Dysgonomonadaceae	Proteiniphilum	
denovo2325	0.0000%	0.0030%	0.0031%	0.0020%	0.0017%	Bacteria	Firmicutes	Clostridia	Oscillospirales	Ruminococcaceae	Pygmaiobacter	Pygmaiobacter_massiliensis
denovo2383	0.0000%	0.0030%	0.0031%	0.0020%	0.0017%	Unassigned						
denovo2845	0.0000%	0.0030%	0.0031%	0.0020%	0.0017%	Bacteria	Bacteroidota	Bacteroidia	Bacteroidales	Williamwhitmaniaceae	Blvii28_wastewater-sludge_group	
denovo3152	0.0000%	0.0030%	0.0031%	0.0020%	0.0017%	Bacteria	Firmicutes	Clostridia	Christensenellales	Christensenellaceae	uncultured	
denovo350	0.0000%	0.0030%	0.0031%	0.0020%	0.0017%	Bacteria	Firmicutes	Clostridia	Peptococcales	Peptococcaceae	uncultured	uncultured_Soehngenia
denovo3870	0.0000%	0.0030%	0.0031%	0.0020%	0.0017%	Bacteria	Firmicutes	Clostridia	Oscillospirales	Oscillospiraceae		
denovo4160	0.0000%	0.0030%	0.0031%	0.0020%	0.0017%	Unassigned						
denovo4366	0.0000%	0.0030%	0.0031%	0.0020%	0.0017%	Unassigned						
denovo4388	0.0000%	0.0030%	0.0031%	0.0020%	0.0017%	Bacteria	Proteobacteria	Alphaproteobacteria	Rhizobiales	Beijerinckiaceae	Bosea	
denovo4392	0.0000%	0.0030%	0.0031%	0.0020%	0.0017%	Unassigned						
denovo4520	0.0000%	0.0030%	0.0031%	0.0020%	0.0017%	Bacteria	Firmicutes	Clostridia	Clostridia	Hungateiclostridiaceae		
denovo4572	0.0000%	0.0030%	0.0031%	0.0020%	0.0017%	Unassigned						
denovo4737	0.0000%	0.0030%	0.0031%	0.0020%	0.0017%	Unassigned						
denovo4743	0.0000%	0.0030%	0.0031%	0.0020%	0.0017%	Unassigned						
denovo54	0.0000%	0.0030%	0.0031%	0.0020%	0.0017%	Bacteria	Desulfobacterota	Desulfobacteria	Desulfobacterales	Desulfosarcinaceae	Desulfosarcina	
denovo5425	0.0000%	0.0030%	0.0031%	0.0020%	0.0017%	Unassigned						
denovo5571	0.0000%	0.0030%	0.0031%	0.0020%	0.0017%	Unassigned						
denovo5715	0.0000%	0.0030%	0.0031%	0.0020%	0.0017%	Unassigned						
denovo5721	0.0000%	0.0030%	0.0031%	0.0020%	0.0017%	Bacteria	Verrucomicrobiota	Lentisphaeria	Oligosphaerales	Lenti-02	Lenti-02	
denovo5727	0.0000%	0.0030%	0.0031%	0.0020%	0.0017%	Bacteria	Firmicutes	Clostridia				
denovo5913	0.0000%	0.0030%	0.0031%	0.0020%	0.0017%	Bacteria	Firmicutes	Clostridia	Oscillospirales	Ruminococcaceae	uncultured	
denovo6568	0.0000%	0.0030%	0.0031%	0.0020%	0.0017%	Bacteria	Firmicutes	Clostridia	Lachnospirales	Lachnospiraceae	Tuzzerella	uncultured_Clostridium
denovo6618	0.0000%	0.0030%	0.0031%	0.0020%	0.0017%	Unassigned						
denovo6631	0.0000%	0.0030%	0.0031%	0.0020%	0.0017%	Unassigned						
denovo6863	0.0000%	0.0030%	0.0031%	0.0020%	0.0017%	Bacteria						
denovo7006	0.0000%	0.0030%	0.0031%	0.0020%	0.0017%	Bacteria	Firmicutes	Clostridia	Peptococcales	Peptococcaceae	uncultured	
denovo7077	0.0000%	0.0030%	0.0031%	0.0020%	0.0017%	Unassigned						
denovo7139	0.0000%	0.0030%	0.0031%	0.0020%	0.0017%	Bacteria	Firmicutes	Clostridia	Christensenellales	Christensenellaceae	Christensenellaceae_R-7_group	
denovo7504	0.0000%	0.0030%	0.0031%	0.0020%	0.0017%	Unassigned						
denovo7582	0.0000%	0.0030%	0.0031%	0.0020%	0.0017%	Bacteria	Firmicutes	Clostridia	Oscillospirales	Ruminococcaceae	uncultured	uncultured_Firmicutes
denovo7753	0.0000%	0.0030%	0.0031%	0.0020%	0.0017%	Unassigned						
denovo7844	0.0000%	0.0030%	0.0031%	0.0020%	0.0017%	Bacteria	Firmicutes	Clostridia				
denovo7871	0.0000%	0.0030%	0.0031%	0.0020%	0.0017%	Bacteria	Firmicutes	Clostridia	Christensenellales	Christensenellaceae	Christensenellaceae_R-7_group	uncultured_soil
denovo800	0.0000%	0.0030%	0.0031%	0.0020%	0.0017%	Bacteria	Firmicutes	Clostridia	Oscillospirales	Ruminococcaceae	uncultured	

denovo8413	0.0000%	0.0030%	0.0031%	0.0020%	0.0017%	Unassigned					
denovo1099	0.0031%	0.0030%	0.0000%	0.0020%	0.0017%	Bacteria	Firmicutes	Clostridia	Peptostreptococcales-Tissierellales	Peptostreptococcales-Tissierellales		
denovo141	0.0031%	0.0030%	0.0000%	0.0020%	0.0017%	Bacteria	Spirochaetota	Spirochaetia	Spirochaetales	Spirochaetaceae	uncultured	uncultured_bacterium
denovo152	0.0031%	0.0030%	0.0000%	0.0020%	0.0017%	Eukaryota						
denovo1665	0.0031%	0.0030%	0.0000%	0.0020%	0.0017%	Bacteria	Firmicutes	Desulfotomaculia	Desulfotomaculales	Desulfotomaculales	Pelotomaculum	uncultured_bacterium
denovo1725	0.0031%	0.0030%	0.0000%	0.0020%	0.0017%	Bacteria	Firmicutes	Clostridia	Clostridiales	Clostridiaceae	Clostridium_sensu_stricto_1	
denovo1825	0.0031%	0.0030%	0.0000%	0.0020%	0.0017%	Bacteria	Firmicutes	Clostridia	Oscillospirales	Oscillospiraceae	NK4A214_group	uncultured_Firmicutes
denovo2151	0.0031%	0.0030%	0.0000%	0.0020%	0.0017%	Eukaryota						
denovo2211	0.0031%	0.0030%	0.0000%	0.0020%	0.0017%	Bacteria	Chloroflexi	Anaerolineae	Anaerolineales	Anaerolineaceae	Pelolinea	
denovo2404	0.0031%	0.0030%	0.0000%	0.0020%	0.0017%	Unassigned						
denovo2621	0.0031%	0.0030%	0.0000%	0.0020%	0.0017%	Bacteria	Firmicutes	Clostridia	Christensenellales	Christensenellaceae	Christensenellaceae_R-7_group	
denovo2675	0.0031%	0.0030%	0.0000%	0.0020%	0.0017%	Bacteria						
denovo2833	0.0031%	0.0030%	0.0000%	0.0020%	0.0017%	Unassigned						
denovo2900	0.0031%	0.0030%	0.0000%	0.0020%	0.0017%	Bacteria	Firmicutes	Clostridia	Oscillospirales	Oscillospiraceae		
denovo2922	0.0031%	0.0030%	0.0000%	0.0020%	0.0017%	Bacteria	Firmicutes	Bacilli	Erysipelotrichales	Erysipelatoclostridiaceae	UCG-004	
denovo3003	0.0031%	0.0030%	0.0000%	0.0020%	0.0017%	Bacteria	Firmicutes	Clostridia	Oscillospirales			
denovo3438	0.0031%	0.0030%	0.0000%	0.0020%	0.0017%	Bacteria	WPS-2	WPS-2	WPS-2	WPS-2	WPS-2	Burkholderiales_bacterium
denovo3532	0.0031%	0.0030%	0.0000%	0.0020%	0.0017%	Unassigned						
denovo3630	0.0031%	0.0030%	0.0000%	0.0020%	0.0017%	Bacteria	Desulfobacterota	Syntrophobacteria	Syntrophobacterales	Syntrophobacteraceae	Syntrophobacter	
denovo3784	0.0031%	0.0030%	0.0000%	0.0020%	0.0017%	Bacteria	Synergistota	Synergistia	Synergistales	Synergistaceae	Aminivibrio	
denovo3942	0.0031%	0.0030%	0.0000%	0.0020%	0.0017%	Bacteria	Bacteroidota	SJA-28	SJA-28	SJA-28	SJA-28	uncultured_bacterium
denovo430	0.0031%	0.0030%	0.0000%	0.0020%	0.0017%	Bacteria	Actinobacteriota	Coriobacteriia	Coriobacteriales	Coriobacteriales_Incertae_Sedis	Raoultibacter	uncultured_bacterium
denovo4390	0.0031%	0.0030%	0.0000%	0.0020%	0.0017%	Archaea	Euryarchaeota	Methanobacteria	Methanobacteriales	Methanobacteriaceae	Methanobacterium	archaeon_enrichment
denovo444	0.0031%	0.0030%	0.0000%	0.0020%	0.0017%	Unassigned						
denovo4521	0.0031%	0.0030%	0.0000%	0.0020%	0.0017%	Unassigned						
denovo4552	0.0031%	0.0030%	0.0000%	0.0020%	0.0017%	Archaea	Halobacterota					
denovo5617	0.0031%	0.0030%	0.0000%	0.0020%	0.0017%	Bacteria	Firmicutes	Syntrophomonadia	Syntrophomonadales	Syntrophomonadaceae	Syntrophomonas	
denovo6014	0.0031%	0.0030%	0.0000%	0.0020%	0.0017%	Bacteria	Firmicutes	Clostridia	Oscillospirales			
denovo6723	0.0031%	0.0030%	0.0000%	0.0020%	0.0017%	Bacteria	Proteobacteria	Alphaproteobacteria	Rickettsiales	Mitochondria	Mitochondria	uncultured_bacterium
denovo6841	0.0031%	0.0030%	0.0000%	0.0020%	0.0017%	Bacteria	Firmicutes	Syntrophomonadia	Syntrophomonadales	Syntrophomonadaceae		
denovo7035	0.0031%	0.0030%	0.0000%	0.0020%	0.0017%	Bacteria	Desulfobacterota	Syntrophia	Syntrophales	Syntrophaceae	Syntrophus	
denovo7302	0.0031%	0.0030%	0.0000%	0.0020%	0.0017%	Bacteria	Firmicutes	Clostridia	Peptostreptococcales-Tissierellales	Anaerovoracaceae		
denovo7344	0.0031%	0.0030%	0.0000%	0.0020%	0.0017%	Bacteria	Actinobacteriota	Thermoleophilia	Gaiellales	uncultured	uncultured	uncultured_prokaryote
denovo7672	0.0031%	0.0030%	0.0000%	0.0020%	0.0017%	Bacteria	Firmicutes	Clostridia	Oscillospirales	Ruminococcaceae	uncultured	uncultured_Firmicutes
denovo7709	0.0031%	0.0030%	0.0000%	0.0020%	0.0017%	Bacteria	Firmicutes	Clostridia	Eubacteriales	Anaerofustaceae	Anaerofustis	uncultured_Clostridium
denovo7713	0.0031%	0.0030%	0.0000%	0.0020%	0.0017%	Archaea	Euryarchaeota	Methanobacteria	Methanobacteriales	Methanobacteriaceae	Methanobacterium	
denovo8337	0.0031%	0.0030%	0.0000%	0.0020%	0.0017%	Bacteria	Firmicutes	Clostridia	Clostridiales	Clostridiaceae	Clostridium_sensu_stricto_13	
denovo839	0.0031%	0.0030%	0.0000%	0.0020%	0.0017%	Bacteria	Actinobacteriota	Actinobacteria				
denovo863	0.0031%	0.0030%	0.0000%	0.0020%	0.0017%	Bacteria	Firmicutes	Clostridia	Oscillospirales			
denovo8778	0.0031%	0.0030%	0.0000%	0.0020%	0.0017%	Unassigned						
denovo8890	0.0031%	0.0030%	0.0000%	0.0020%	0.0017%	Bacteria	Firmicutes	Clostridia	Oscillospirales	Ruminococcaceae	Incertae_Sedis	
denovo903	0.0031%	0.0030%	0.0000%	0.0020%	0.0017%	Unassigned						
denovo1660	0.0000%	0.0059%	0.0000%	0.0020%	0.0034%	Unassigned						
denovo1726	0.0000%	0.0059%	0.0000%	0.0020%	0.0034%	Unassigned						
denovo1756	0.0000%	0.0059%	0.0000%	0.0020%	0.0034%	Bacteria	Bacteroidota	Bacteroidia	Bacteroidales	Rikenellaceae	Alistipes	uncultured_Bacteroidetes
denovo2463	0.0000%	0.0059%	0.0000%	0.0020%	0.0034%	Bacteria	Proteobacteria	Gammaproteobacteria	Enterobacterales	Enterobacteriaceae		
denovo2549	0.0000%	0.0059%	0.0000%	0.0020%	0.0034%	Bacteria	Firmicutes	Clostridia	Christensenellales	Christensenellaceae	Christensenellaceae_R-7_group	
denovo2570	0.0000%	0.0059%	0.0000%	0.0020%	0.0034%	Unassigned						
denovo2856	0.0000%	0.0059%	0.0000%	0.0020%	0.0034%	Bacteria	Spirochaetota	Spirochaetia	Spirochaetales	Spirochaetaceae	Spirochaeta	
denovo2895	0.0000%	0.0059%	0.0000%	0.0020%	0.0034%	Bacteria	Firmicutes	Clostridia	Christensenellales	Christensenellaceae	Christensenellaceae_R-7_group	
denovo3403	0.0000%	0.0059%	0.0000%	0.0020%	0.0034%	Bacteria	WS1	WS1	WS1	WS1	WS1	uncultured_bacterium
denovo3872	0.0000%	0.0059%	0.0000%	0.0020%	0.0034%	Unassigned						
denovo4164	0.0000%	0.0059%	0.0000%	0.0020%	0.0034%	Bacteria	Firmicutes	uncultured	uncultured	uncultured	uncultured	
denovo4418	0.0000%	0.0059%	0.0000%	0.0020%	0.0034%	Unassigned						
denovo454	0.0000%	0.0059%	0.0000%	0.0020%	0.0034%	Bacteria	Desulfobacterota	Desulfuromonadia	Sva1033	Sva1033	Sva1033	
denovo5547	0.0000%	0.0059%	0.0000%	0.0020%	0.0034%	Bacteria	Firmicutes	Bacilli	Erysipelotrichales	Erysipelatoclostridiaceae	UCG-004	uncultured_bacterium
denovo5994	0.0000%	0.0059%	0.0000%	0.0020%	0.0034%	Bacteria	Bacteroidota	Bacteroidia	Flavobacteriales	Weeksellaceae	Soonwooa	
denovo6416	0.0000%	0.0059%	0.0000%	0.0020%	0.0034%	Bacteria	Firmicutes	Bacilli	Lactobacillales	Enterococcaceae	Enterococcus	
denovo6506	0.0000%	0.0059%	0.0000%	0.0020%	0.0034%	Bacteria	Spirochaetota	Spirochaetia	Spirochaetales	Spirochaetaceae	uncultured	
denovo6581	0.0000%	0.0059%	0.0000%	0.0020%	0.0034%	Unassigned						

denovo6870	0.0000%	0.0059%	0.0000%	0.0020%	0.0034%	Bacteria	Chloroflexi	Anaerolineae	Anaerolineales	Anaerolineaceae	uncultured	
denovo7307	0.0000%	0.0059%	0.0000%	0.0020%	0.0034%	Unassigned						
denovo7435	0.0000%	0.0059%	0.0000%	0.0020%	0.0034%	Bacteria	Firmicutes	Clostridia	Peptostreptococcales-Tissierellales	Anaerovoracaceae		
denovo7613	0.0000%	0.0059%	0.0000%	0.0020%	0.0034%	Bacteria	Armatimonadota	uncultured	uncultured	uncultured	uncultured	uncultured_Armatimonadetes
denovo778	0.0000%	0.0059%	0.0000%	0.0020%	0.0034%	Unassigned						
denovo7954	0.0000%	0.0059%	0.0000%	0.0020%	0.0034%	Bacteria	Firmicutes	Clostridia	Clostridia	Hungateiclostridiaceae		
denovo7966	0.0000%	0.0059%	0.0000%	0.0020%	0.0034%	Bacteria	Firmicutes	Desulfotomaculia	Desulfotomaculales	Desulfotomaculales	Pelotomaculum	
denovo8067	0.0000%	0.0059%	0.0000%	0.0020%	0.0034%	Bacteria	Firmicutes	Clostridia	Oscillospirales	Ruminococcaceae		
denovo8394	0.0000%	0.0059%	0.0000%	0.0020%	0.0034%	Archaea	Euryarchaeota	Methanobacteria	Methanobacteriales	Methanobacteriaceae	Methanobacterium	
denovo8550	0.0000%	0.0059%	0.0000%	0.0020%	0.0034%	Bacteria	Bacteroidota	Bacteroidia	Bacteroidales	Dysgonomonadaceae	Petrimonas	
denovo8895	0.0000%	0.0059%	0.0000%	0.0020%	0.0034%	Bacteria	Firmicutes	Clostridia	Peptostreptococcales-Tissierellales	Peptostreptococcaceae	Terrisporobacter	
denovo8919	0.0000%	0.0059%	0.0000%	0.0020%	0.0034%	Bacteria	Firmicutes	Clostridia	Oscillospirales	Ruminococcaceae	Candidatus_Soleaferrea	
denovo8980	0.0000%	0.0059%	0.0000%	0.0020%	0.0034%	Bacteria						
denovo1022	0.0000%	0.0000%	0.0031%	0.0010%	0.0018%	Bacteria	Firmicutes	Syntrophomonadia	Syntrophomonadales	Syntrophomonadaceae	Syntrophomonas	Syntrophomonas_wolfei
denovo103	0.0000%	0.0000%	0.0031%	0.0010%	0.0018%	Bacteria	Desulfobacterota	Desulfovibrionia	Desulfovibrionales	Desulfovibrionaceae	Desulfovibrio	
denovo1034	0.0000%	0.0000%	0.0031%	0.0010%	0.0018%	Bacteria	Desulfobacterota	Desulfovibrionia	Desulfovibrionales	Desulfovibrionaceae	Desulfovibrio	
denovo1046	0.0000%	0.0000%	0.0031%	0.0010%	0.0018%	Unassigned						
denovo1056	0.0000%	0.0000%	0.0031%	0.0010%	0.0018%	Bacteria	Bacteroidota	Bacteroidia	Bacteroidales	Rikenellaceae	DMER64	bacterium_enrichment
denovo1059	0.0000%	0.0000%	0.0031%	0.0010%	0.0018%	Archaea	Halobacterota	Methanomicrobia	Methanomicrobiales	Methanomicrobiaceae	Methanomicrobium	
denovo1067	0.0000%	0.0000%	0.0031%	0.0010%	0.0018%	Bacteria	Firmicutes					
denovo1073	0.0000%	0.0000%	0.0031%	0.0010%	0.0018%	Bacteria	Bacteroidota	Bacteroidia	Bacteroidales	Williamwhitmaniaceae	Blvii28_wastewater-sludge_group	uncultured_bacterium
denovo1084	0.0000%	0.0000%	0.0031%	0.0010%	0.0018%	Bacteria	Spirochaetota	Spirochaetia	Spirochaetales	Spirochaetaceae	uncultured	uncultured_bacterium
denovo1087	0.0000%	0.0000%	0.0031%	0.0010%	0.0018%	Bacteria	Firmicutes	Bacilli	Erysipelotrichales	Erysipelatoclostridiaceae	Erysipelatoclostridium	
denovo1104	0.0000%	0.0000%	0.0031%	0.0010%	0.0018%	Bacteria	Bacteroidota	Bacteroidia				
denovo1116	0.0000%	0.0000%	0.0031%	0.0010%	0.0018%	Bacteria	Bacteroidota	Bacteroidia	Bacteroidales	Dysgonomonadaceae	Proteiniphilum	
denovo1119	0.0000%	0.0000%	0.0031%	0.0010%	0.0018%	Bacteria	Synergistota	Synergistia	Synergistales	Synergistaceae	uncultured	uncultured_bacterium
denovo1127	0.0000%	0.0000%	0.0031%	0.0010%	0.0018%	Unassigned						
denovo1133	0.0000%	0.0000%	0.0031%	0.0010%	0.0018%	Unassigned						
denovo1140	0.0000%	0.0000%	0.0031%	0.0010%	0.0018%	Bacteria	Synergistota	Synergistia	Synergistales	Synergistaceae	Syner-01	
denovo1222	0.0000%	0.0000%	0.0031%	0.0010%	0.0018%	Bacteria	Firmicutes	Clostridia	Christensenellales	Christensenellaceae	Christensenellaceae_R-7_group	
denovo1224	0.0000%	0.0000%	0.0031%	0.0010%	0.0018%	Bacteria	Desulfobacterota	Syntrophorhabdia	Syntrophorhabdales	Syntrophorhabdaceae	Syntrophorhabdus	
denovo1236	0.0000%	0.0000%	0.0031%	0.0010%	0.0018%	Bacteria	Firmicutes	Bacilli	Izemoplasmatales	Izemoplasmatales	Izemoplasmatales	uncultured_bacterium
denovo1260	0.0000%	0.0000%	0.0031%	0.0010%	0.0018%	Bacteria	Synergistota	Synergistia	Synergistales	Synergistaceae	Thermovirga	
denovo1270	0.0000%	0.0000%	0.0031%	0.0010%	0.0018%	Bacteria						
denovo1272	0.0000%	0.0000%	0.0031%	0.0010%	0.0018%	Bacteria	Firmicutes	Syntrophomonadia	Syntrophomonadales	Syntrophomonadaceae	Syntrophomonas	
denovo1283	0.0000%	0.0000%	0.0031%	0.0010%	0.0018%	Bacteria						
denovo1314	0.0000%	0.0000%	0.0031%	0.0010%	0.0018%	Bacteria	Bacteroidota	Bacteroidia	Chitinophagales	Chitinophagaceae	uncultured	
denovo1321	0.0000%	0.0000%	0.0031%	0.0010%	0.0018%	Bacteria	Firmicutes					
denovo1328	0.0000%	0.0000%	0.0031%	0.0010%	0.0018%	Bacteria	Firmicutes	Clostridia	Lachnospirales	Lachnospiraceae	Robinsoniella	
denovo1334	0.0000%	0.0000%	0.0031%	0.0010%	0.0018%	Bacteria	Proteobacteria	Alphaproteobacteria	Rhodospirillales	Magnetospirillaceae	Telmatospirillum	
denovo1360	0.0000%	0.0000%	0.0031%	0.0010%	0.0018%	Bacteria	Bacteroidota	Bacteroidia	Bacteroidales			
denovo138	0.0000%	0.0000%	0.0031%	0.0010%	0.0018%	Bacteria	Firmicutes	Bacilli	Lactobacillales	Enterococcaceae	Enterococcus	
denovo1405	0.0000%	0.0000%	0.0031%	0.0010%	0.0018%	Bacteria	Firmicutes	Clostridia	Eubacteriales	Eubacteriaceae	Eubacterium	
denovo1411	0.0000%	0.0000%	0.0031%	0.0010%	0.0018%	Bacteria	Firmicutes	Syntrophomonadia	Syntrophomonadales	Syntrophomonadaceae	Syntrophomonas	
denovo1429	0.0000%	0.0000%	0.0031%	0.0010%	0.0018%	Bacteria	Bacteroidota	Bacteroidia	Sphingobacteriales			
denovo1437	0.0000%	0.0000%	0.0031%	0.0010%	0.0018%	Bacteria	Firmicutes	Clostridia				
denovo1441	0.0000%	0.0000%	0.0031%	0.0010%	0.0018%	Bacteria	Firmicutes	Clostridia	Christensenellales	Christensenellaceae		
denovo1473	0.0000%	0.0000%	0.0031%	0.0010%	0.0018%	Bacteria	Firmicutes	Bacilli				
denovo1482	0.0000%	0.0000%	0.0031%	0.0010%	0.0018%	Bacteria	Firmicutes	Bacilli	Lactobacillales	Enterococcaceae	Enterococcus	
denovo1527	0.0000%	0.0000%	0.0031%	0.0010%	0.0018%	Bacteria						
denovo1541	0.0000%	0.0000%	0.0031%	0.0010%	0.0018%	Bacteria	Firmicutes	Clostridia				
denovo1546	0.0000%	0.0000%	0.0031%	0.0010%	0.0018%	Bacteria	Firmicutes	Clostridia				
denovo1547	0.0000%	0.0000%	0.0031%	0.0010%	0.0018%	Bacteria	Bacteroidota	Bacteroidia	Bacteroidales	Bacteroidetes_vadinHA17	Bacteroidetes_vadinHA17	uncultured_bacterium
denovo1563	0.0000%	0.0000%	0.0031%	0.0010%	0.0018%	Bacteria	Firmicutes	Clostridia	Peptostreptococcales-Tissierellales	Anaerovoracaceae		
denovo1577	0.0000%	0.0000%	0.0031%	0.0010%	0.0018%	Bacteria	Firmicutes	Clostridia				
denovo1592	0.0000%	0.0000%	0.0031%	0.0010%	0.0018%	Bacteria	Bacteroidota	Bacteroidia	Bacteroidales	Dysgonomonadaceae	Petrimonas	
denovo1607	0.0000%	0.0000%	0.0031%	0.0010%	0.0018%	Bacteria	Firmicutes	Clostridia				
denovo1628	0.0000%	0.0000%	0.0031%	0.0010%	0.0018%	Bacteria	Firmicutes	Bacilli	Izemoplasmatales	Izemoplasmatales	Izemoplasmatales	uncultured_bacterium
denovo1647	0.0000%	0.0000%	0.0031%	0.0010%	0.0018%	Unassigned						
denovo170	0.0000%	0.0000%	0.0031%	0.0010%	0.0018%	Bacteria	Firmicutes	Syntrophomonadia	Syntrophomonadales	Syntrophomonadaceae	Syntrophomonas	

denovo1701	0.0000%	0.0000%	0.0031%	0.0010%	0.0018%	Bacteria	Bacteroidota	Bacteroidia	Bacteroidales	Bacteroidetes_vadinHA17	Bacteroidetes_vadinHA17	
denovo1709	0.0000%	0.0000%	0.0031%	0.0010%	0.0018%	Bacteria	Synergistota	Synergistia	Synergistales	Synergistaceae		
denovo1747	0.0000%	0.0000%	0.0031%	0.0010%	0.0018%	Bacteria	Desulfobacterota	Syntrophorhabdia	Syntrophorhabdales	Syntrophorhabdaceae	Syntrophorhabdus	
denovo1750	0.0000%	0.0000%	0.0031%	0.0010%	0.0018%	Bacteria	Firmicutes	Clostridia	Christensenellales	Christensenellaceae	Christensenellaceae_R-7_group	
denovo1774	0.0000%	0.0000%	0.0031%	0.0010%	0.0018%	Bacteria	Firmicutes	Clostridia	Oscillospirales	Ruminococcaceae	uncultured	
denovo1786	0.0000%	0.0000%	0.0031%	0.0010%	0.0018%	Bacteria	Firmicutes	Clostridia	Oscillospirales			
denovo1831	0.0000%	0.0000%	0.0031%	0.0010%	0.0018%	Bacteria	Firmicutes	Clostridia	Christensenellales	Christensenellaceae	Christensenellaceae_R-7_group	
denovo1842	0.0000%	0.0000%	0.0031%	0.0010%	0.0018%	Bacteria	Firmicutes	Syntrophomonadia	Syntrophomonadales	Syntrophomonadaceae	Syntrophomonas	
denovo1850	0.0000%	0.0000%	0.0031%	0.0010%	0.0018%	Unassigned						
denovo1863	0.0000%	0.0000%	0.0031%	0.0010%	0.0018%	Bacteria	Firmicutes	Clostridia	Oscillospirales			
denovo1891	0.0000%	0.0000%	0.0031%	0.0010%	0.0018%	Unassigned						
denovo1912	0.0000%	0.0000%	0.0031%	0.0010%	0.0018%	Bacteria	Chloroflexi	Anaerolineae	Anaerolineales	Anaerolineaceae	Flexilinea	
denovo1917	0.0000%	0.0000%	0.0031%	0.0010%	0.0018%	Bacteria	Firmicutes	Clostridia	Oscillospirales	Ruminococcaceae		
denovo1923	0.0000%	0.0000%	0.0031%	0.0010%	0.0018%	Bacteria	Firmicutes	Bacilli	Lactobacillales	Enterococcaceae	Enterococcus	
denovo1927	0.0000%	0.0000%	0.0031%	0.0010%	0.0018%	Bacteria	Thermotogota	Thermotogae	Kosmotogales	Kosmotogaceae	Mesotoga	
denovo1938	0.0000%	0.0000%	0.0031%	0.0010%	0.0018%	Bacteria	Proteobacteria	Alphaproteobacteria	Rhizobiales	Rhizobiaceae		
denovo1949	0.0000%	0.0000%	0.0031%	0.0010%	0.0018%	Bacteria	Patescibacteria	ABY1	Candidatus_Falkowbacteria	Candidatus_Falkowbacteria	Candidatus_Falkowbacteria	
denovo1963	0.0000%	0.0000%	0.0031%	0.0010%	0.0018%	Archaea	Nanoarchaeota	Nanoarchaeia	Woesearchaeales	Woesearchaeales	Woesearchaeales	
denovo1996	0.0000%	0.0000%	0.0031%	0.0010%	0.0018%	Bacteria	Firmicutes	Syntrophomonadia	Syntrophomonadales	Syntrophomonadaceae		
denovo2008	0.0000%	0.0000%	0.0031%	0.0010%	0.0018%	Bacteria	Firmicutes	Clostridia	Christensenellales	Christensenellaceae	Christensenellaceae_R-7_group	
denovo2012	0.0000%	0.0000%	0.0031%	0.0010%	0.0018%	Bacteria	Bacteroidota	Bacteroidia	Bacteroidales	Bacteroidaceae	Bacteroides	
denovo2034	0.0000%	0.0000%	0.0031%	0.0010%	0.0018%	Bacteria	Firmicutes					
denovo2085	0.0000%	0.0000%	0.0031%	0.0010%	0.0018%	Bacteria	Desulfobacterota	Desulfuromonadia	Geobacterales	Geobacteraceae		
denovo2139	0.0000%	0.0000%	0.0031%	0.0010%	0.0018%	Bacteria	Firmicutes	Clostridia	Oscillospirales	Oscillospiraceae		
denovo2150	0.0000%	0.0000%	0.0031%	0.0010%	0.0018%	Bacteria	Proteobacteria	Gammaproteobacteria	Burkholderiales	Rhodocyclaceae		
denovo2167	0.0000%	0.0000%	0.0031%	0.0010%	0.0018%	Bacteria	Synergistota	Synergistia	Synergistales	Synergistaceae	uncultured	bacterium_enrichment
denovo2190	0.0000%	0.0000%	0.0031%	0.0010%	0.0018%	Unassigned						
denovo2193	0.0000%	0.0000%	0.0031%	0.0010%	0.0018%	Bacteria	Synergistota	Synergistia	Synergistales	Synergistaceae	Thermovirga	
denovo2218	0.0000%	0.0000%	0.0031%	0.0010%	0.0018%	Bacteria	Acidobacteriota	Holophagae	Holophagales	Holophagaceae	Holophaga	uncultured_bacterium
denovo2230	0.0000%	0.0000%	0.0031%	0.0010%	0.0018%	Bacteria	Firmicutes	Clostridia	Oscillospirales	Ruminococcaceae	uncultured	
denovo2251	0.0000%	0.0000%	0.0031%	0.0010%	0.0018%	Bacteria	Firmicutes	Clostridia	Peptococcales	Peptococcaceae	uncultured	uncultured_Peptococcaceae
denovo2258	0.0000%	0.0000%	0.0031%	0.0010%	0.0018%	Bacteria	Firmicutes	Clostridia	Oscillospirales	Ruminococcaceae	uncultured	uncultured_Firmicutes
denovo2265	0.0000%	0.0000%	0.0031%	0.0010%	0.0018%	Archaea	Euryarchaeota	Methanobacteria	Methanobacteriales	Methanobacteriaceae	Methanobacterium	
denovo2291	0.0000%	0.0000%	0.0031%	0.0010%	0.0018%	Bacteria	Synergistota	Synergistia	Synergistales	Synergistaceae	uncultured	
denovo2297	0.0000%	0.0000%	0.0031%	0.0010%	0.0018%	Bacteria	Chloroflexi	Anaerolineae				
denovo2338	0.0000%	0.0000%	0.0031%	0.0010%	0.0018%	Bacteria	Proteobacteria	Gammaproteobacteria	Xanthomonadales			
denovo2409	0.0000%	0.0000%	0.0031%	0.0010%	0.0018%	Bacteria	Bacteroidota	Bacteroidia	Bacteroidales	Bacteroidaceae	Bacteroides	
denovo2410	0.0000%	0.0000%	0.0031%	0.0010%	0.0018%	Bacteria	Bacteroidota	Bacteroidia	Bacteroidales	Bacteroidetes_vadinHA17	Bacteroidetes_vadinHA17	
denovo2413	0.0000%	0.0000%	0.0031%	0.0010%	0.0018%	Bacteria	Desulfobacterota	Syntrophobacteria	Syntrophobacterales	Syntrophobacteraceae	Syntrophobacter	
denovo2434	0.0000%	0.0000%	0.0031%	0.0010%	0.0018%	Bacteria	Firmicutes	Clostridia	Lachnospirales	Lachnospiraceae	[Eubacterium]_fissicatena_group	
denovo2440	0.0000%	0.0000%	0.0031%	0.0010%	0.0018%	Bacteria	Proteobacteria	Gammaproteobacteria	Burkholderiales	Hydrogenophilaceae	Thiobacillus	
denovo2461	0.0000%	0.0000%	0.0031%	0.0010%	0.0018%	Bacteria	Firmicutes	Clostridia	Oscillospirales			
denovo2493	0.0000%	0.0000%	0.0031%	0.0010%	0.0018%	Bacteria	Proteobacteria	Gammaproteobacteria	Legionellales	Legionellaceae	Legionella	
denovo250	0.0000%	0.0000%	0.0031%	0.0010%	0.0018%	Bacteria	Proteobacteria	Gammaproteobacteria	Enterobacterales	Enterobacteriaceae		
denovo2520	0.0000%	0.0000%	0.0031%	0.0010%	0.0018%	Bacteria	Firmicutes	Bacilli	Izemoplasmatales	Izemoplasmatales	Izemoplasmatales	uncultured_bacterium
denovo2526	0.0000%	0.0000%	0.0031%	0.0010%	0.0018%	Bacteria	Bacteroidota	Bacteroidia	Bacteroidales	Rikenellaceae	DMER64	bacterium_enrichment
denovo2618	0.0000%	0.0000%	0.0031%	0.0010%	0.0018%	Unassigned						
denovo2625	0.0000%	0.0000%	0.0031%	0.0010%	0.0018%	Bacteria	Firmicutes	Clostridia	Peptococcales	Peptococcaceae	uncultured	uncultured_Peptococcaceae
denovo2627	0.0000%	0.0000%	0.0031%	0.0010%	0.0018%	Bacteria	Firmicutes	Syntrophomonadia	Syntrophomonadales	Syntrophomonadaceae	Syntrophomonas	
denovo2654	0.0000%	0.0000%	0.0031%	0.0010%	0.0018%	Unassigned						
denovo2677	0.0000%	0.0000%	0.0031%	0.0010%	0.0018%	Bacteria	Bacteroidota	Bacteroidia	Bacteroidales			
denovo2679	0.0000%	0.0000%	0.0031%	0.0010%	0.0018%	Bacteria	Synergistota	Synergistia	Synergistales	Synergistaceae	uncultured	
denovo2689	0.0000%	0.0000%	0.0031%	0.0010%	0.0018%	Bacteria	Proteobacteria	Gammaproteobacteria	Enterobacterales	Enterobacteriaceae		
denovo2728	0.0000%	0.0000%	0.0031%	0.0010%	0.0018%	Unassigned						
denovo2785	0.0000%	0.0000%	0.0031%	0.0010%	0.0018%	Bacteria	Desulfobacterota	Syntrophobacteria	Syntrophobacterales	Syntrophobacteraceae		
denovo2789	0.0000%	0.0000%	0.0031%	0.0010%	0.0018%	Eukaryota						
denovo279	0.0000%	0.0000%	0.0031%	0.0010%	0.0018%	Bacteria	Firmicutes	Syntrophomonadia	Syntrophomonadales	Syntrophomonadaceae		
denovo28	0.0000%	0.0000%	0.0031%	0.0010%	0.0018%	Archaea	Halobacterota	Methanomicrobia	Methanomicrobiales	Methanospirillaceae	Methanospirillum	
denovo2806	0.0000%	0.0000%	0.0031%	0.0010%	0.0018%	Bacteria	Firmicutes	Clostridia	Oscillospirales	Oscillospiraceae		
denovo2864	0.0000%	0.0000%	0.0031%	0.0010%	0.0018%	Bacteria	Proteobacteria	Alphaproteobacteria	Rhodospirillales	Magnetospirillaceae	Telmatospirillum	

denovo2904	0.0000%	0.0000%	0.0031%	0.0010%	0.0018%	Bacteria	Bacteroidota	Bacteroidia				
denovo2910	0.0000%	0.0000%	0.0031%	0.0010%	0.0018%	Bacteria	Synergistota	Synergistia	Synergistales	Synergistaceae	Syner-01	uncultured_bacterium
denovo2946	0.0000%	0.0000%	0.0031%	0.0010%	0.0018%	Archaea	Halobacterota					
denovo3021	0.0000%	0.0000%	0.0031%	0.0010%	0.0018%	Bacteria	Synergistota	Synergistia	Synergistales	Synergistaceae	uncultured	bacterium_enrichment
denovo3027	0.0000%	0.0000%	0.0031%	0.0010%	0.0018%	Bacteria	Firmicutes	Clostridia	Christensenellales	Christensenellaceae	Christensenellaceae_R-7_group	
denovo308	0.0000%	0.0000%	0.0031%	0.0010%	0.0018%	Eukaryota						
denovo3092	0.0000%	0.0000%	0.0031%	0.0010%	0.0018%	Bacteria	Firmicutes	Syntrophomonadia	Syntrophomonadales	Syntrophomonadaceae	Syntrophomonas	
denovo3104	0.0000%	0.0000%	0.0031%	0.0010%	0.0018%	Bacteria	Desulfobacterota	Desulfovibrionia	Desulfovibrionales	Desulfovibrionaceae	Desulfovibrio	
denovo3108	0.0000%	0.0000%	0.0031%	0.0010%	0.0018%	Bacteria	Synergistota	Synergistia	Synergistales	Synergistaceae	uncultured	bacterium_enrichment
denovo3125	0.0000%	0.0000%	0.0031%	0.0010%	0.0018%	Bacteria	Firmicutes	Clostridia	Oscillospirales	Ruminococcaceae	uncultured	
denovo3126	0.0000%	0.0000%	0.0031%	0.0010%	0.0018%	Bacteria	Firmicutes	Clostridia	Oscillospirales	Ruminococcaceae	uncultured	
denovo3142	0.0000%	0.0000%	0.0031%	0.0010%	0.0018%	Bacteria	Proteobacteria	Alphaproteobacteria	Rhizobiales	Xanthobacteraceae		
denovo3162	0.0000%	0.0000%	0.0031%	0.0010%	0.0018%	Bacteria	Bacteroidota	Bacteroidia	Bacteroidales	Dysgonomonadaceae	Petrimonas	
denovo3203	0.0000%	0.0000%	0.0031%	0.0010%	0.0018%	Bacteria	Firmicutes	Syntrophomonadia	Syntrophomonadales	Syntrophomonadaceae		
denovo3218	0.0000%	0.0000%	0.0031%	0.0010%	0.0018%	Bacteria	Firmicutes	Clostridia	Clostridia			
denovo3266	0.0000%	0.0000%	0.0031%	0.0010%	0.0018%	Bacteria	Proteobacteria	Alphaproteobacteria	Rhodospirillales	Magnetospirillaceae	Telmatospirillum	
denovo3308	0.0000%	0.0000%	0.0031%	0.0010%	0.0018%	Bacteria	Bacteroidota	Bacteroidia	Bacteroidales	Bacteroidaceae	Bacteroides	
denovo3325	0.0000%	0.0000%	0.0031%	0.0010%	0.0018%	Bacteria	Firmicutes	Clostridia	Eubacteriales	Eubacteriaceae		
denovo3338	0.0000%	0.0000%	0.0031%	0.0010%	0.0018%	Bacteria	Bacteroidota	Bacteroidia	Bacteroidales	Bacteroidaceae	Bacteroides	
denovo3386	0.0000%	0.0000%	0.0031%	0.0010%	0.0018%	Bacteria	Firmicutes	Clostridia	Oscillospirales			
denovo3387	0.0000%	0.0000%	0.0031%	0.0010%	0.0018%	Bacteria	Firmicutes	Clostridia	Eubacteriales	Eubacteriaceae	Eubacterium	
denovo3401	0.0000%	0.0000%	0.0031%	0.0010%	0.0018%	Bacteria						
denovo3424	0.0000%	0.0000%	0.0031%	0.0010%	0.0018%	Bacteria	Synergistota	Synergistia	Synergistales	Synergistaceae	uncultured	
denovo3454	0.0000%	0.0000%	0.0031%	0.0010%	0.0018%	Bacteria	Synergistota	Synergistia	Synergistales	Synergistaceae	uncultured	
denovo3545	0.0000%	0.0000%	0.0031%	0.0010%	0.0018%	Bacteria	Chloroflexi	Anaerolineae	Anaerolineales	Anaerolineaceae	Levilinea	uncultured_bacterium
denovo3547	0.0000%	0.0000%	0.0031%	0.0010%	0.0018%	Bacteria	Firmicutes					
denovo3556	0.0000%	0.0000%	0.0031%	0.0010%	0.0018%	Unassigned						
denovo3599	0.0000%	0.0000%	0.0031%	0.0010%	0.0018%	Bacteria	Firmicutes	Desulfotomaculia	Desulfotomaculales	Desulfotomaculales		
denovo3609	0.0000%	0.0000%	0.0031%	0.0010%	0.0018%	Unassigned						
denovo3659	0.0000%	0.0000%	0.0031%	0.0010%	0.0018%	Bacteria	Bacteroidota	Bacteroidia	Bacteroidales	Bacteroidaceae	Bacteroides	
denovo3683	0.0000%	0.0000%	0.0031%	0.0010%	0.0018%	Unassigned						
denovo3688	0.0000%	0.0000%	0.0031%	0.0010%	0.0018%	Bacteria	Desulfobacterota	Desulfovibrionia	Desulfovibrionales	Desulfovibrionaceae	Desulfovibrio	
denovo3733	0.0000%	0.0000%	0.0031%	0.0010%	0.0018%	Bacteria	Firmicutes	Bacilli	Lactobacillales	Enterococcaceae	Enterococcus	
denovo3740	0.0000%	0.0000%	0.0031%	0.0010%	0.0018%	Bacteria	Desulfobacterota	Desulfovibrionia	Desulfovibrionales	Desulfovibrionaceae	Desulfovibrio	
denovo3746	0.0000%	0.0000%	0.0031%	0.0010%	0.0018%	Bacteria	Firmicutes	Clostridia	Christensenellales	Christensenellaceae	Christensenellaceae_R-7_group	
denovo3747	0.0000%	0.0000%	0.0031%	0.0010%	0.0018%	Bacteria	Firmicutes					
denovo3755	0.0000%	0.0000%	0.0031%	0.0010%	0.0018%	Unassigned						
denovo3769	0.0000%	0.0000%	0.0031%	0.0010%	0.0018%	Bacteria	Firmicutes	Clostridia	Oscillospirales	Ruminococcaceae	Candidatus_Soleaferrea	uncultured_bacterium
denovo378	0.0000%	0.0000%	0.0031%	0.0010%	0.0018%	Bacteria	Proteobacteria	Gammaproteobacteria	Burkholderiales	Nitrosomonadaceae	Nitrosomonas	
denovo3793	0.0000%	0.0000%	0.0031%	0.0010%	0.0018%	Bacteria	Chloroflexi	Anaerolineae	Anaerolineales	Anaerolineaceae	Anaerolinea	
denovo3833	0.0000%	0.0000%	0.0031%	0.0010%	0.0018%	Bacteria	Bacteroidota	Bacteroidia	Bacteroidales	Dysgonomonadaceae	Proteiniphilum	
denovo3843	0.0000%	0.0000%	0.0031%	0.0010%	0.0018%	Bacteria	Firmicutes	Clostridia	Christensenellales	Christensenellaceae	Christensenellaceae_R-7_group	
denovo3847	0.0000%	0.0000%	0.0031%	0.0010%	0.0018%	Bacteria	Bacteroidota	Bacteroidia	Bacteroidales	Dysgonomonadaceae		
denovo3851	0.0000%	0.0000%	0.0031%	0.0010%	0.0018%	Bacteria	Firmicutes	Syntrophomonadia	Syntrophomonadales	Syntrophomonadaceae		
denovo3859	0.0000%	0.0000%	0.0031%	0.0010%	0.0018%	Bacteria	Firmicutes					
denovo3873	0.0000%	0.0000%	0.0031%	0.0010%	0.0018%	Bacteria	Desulfobacterota	Syntrophorhabdia	Syntrophorhabdales	Syntrophorhabdaceae	Syntrophorhabdus	
denovo3883	0.0000%	0.0000%	0.0031%	0.0010%	0.0018%	Bacteria	Synergistota	Synergistia	Synergistales	Synergistaceae	uncultured	uncultured_bacterium
denovo391	0.0000%	0.0000%	0.0031%	0.0010%	0.0018%	Bacteria	Bacteroidota	Bacteroidia				
denovo3935	0.0000%	0.0000%	0.0031%	0.0010%	0.0018%	Bacteria	Synergistota	Synergistia	Synergistales	Synergistaceae	Cloacibacillus	Cloacibacillus_evryensis
denovo3965	0.0000%	0.0000%	0.0031%	0.0010%	0.0018%	Bacteria	Desulfobacterota	Desulfovibrionia	Desulfovibrionales	Desulfovibrionaceae	Desulfovibrio	
denovo399	0.0000%	0.0000%	0.0031%	0.0010%	0.0018%	Bacteria	Firmicutes	Clostridia	Caldicoprobacterales	Caldicoprobacteraceae	Caldicoprobacter	
denovo4032	0.0000%	0.0000%	0.0031%	0.0010%	0.0018%	Bacteria	Firmicutes	Clostridia				
denovo4062	0.0000%	0.0000%	0.0031%	0.0010%	0.0018%	Bacteria	Bacteroidota	Bacteroidia	Bacteroidales	Bacteroidaceae	Bacteroides	
denovo4077	0.0000%	0.0000%	0.0031%	0.0010%	0.0018%	Bacteria	Bacteroidota	Bacteroidia	Bacteroidales	Dysgonomonadaceae	Dysgonomonas	
denovo4099	0.0000%	0.0000%	0.0031%	0.0010%	0.0018%	Bacteria	Synergistota	Synergistia	Synergistales	Synergistaceae	uncultured	uncultured_bacterium
denovo4110	0.0000%	0.0000%	0.0031%	0.0010%	0.0018%	Bacteria	Firmicutes	Bacilli	Izemoplasmatales	Izemoplasmatales	Izemoplasmatales	uncultured_bacterium
denovo4121	0.0000%	0.0000%	0.0031%	0.0010%	0.0018%	Bacteria						
denovo4127	0.0000%	0.0000%	0.0031%	0.0010%	0.0018%	Archaea	Euryarchaeota	Methanobacteria	Methanobacteriales	Methanobacteriaceae	Methanobacterium	
denovo4135	0.0000%	0.0000%	0.0031%	0.0010%	0.0018%	Bacteria	Firmicutes	Clostridia	Oscillospirales			
denovo4158	0.0000%	0.0000%	0.0031%	0.0010%	0.0018%	Bacteria	Synergistota	Synergistia	Synergistales	Synergistaceae	Pyramidobacter	uncultured_bacterium

denovo4175	0.0000%	0.0000%	0.0031%	0.0010%	0.0018%	Bacteria	Chloroflexi	Anaerolineae	Anaerolineales	Anaerolineaceae	uncultured	
denovo4199	0.0000%	0.0000%	0.0031%	0.0010%	0.0018%	Bacteria	GN01	GN01	GN01	GN01	GN01	uncultured_bacterium
denovo423	0.0000%	0.0000%	0.0031%	0.0010%	0.0018%	Unassigned						
denovo4250	0.0000%	0.0000%	0.0031%	0.0010%	0.0018%	Bacteria	Firmicutes	Desulfotomaculia	Desulfotomaculales	Desulfotomaculales	Pelotomaculum	uncultured_bacterium
denovo4266	0.0000%	0.0000%	0.0031%	0.0010%	0.0018%	Bacteria						
denovo4269	0.0000%	0.0000%	0.0031%	0.0010%	0.0018%	Bacteria	Desulfobacterota	Desulfovibrionia	Desulfovibrionales	Desulfovibrionaceae	Desulfovibrio	
denovo4281	0.0000%	0.0000%	0.0031%	0.0010%	0.0018%	Bacteria						
denovo4283	0.0000%	0.0000%	0.0031%	0.0010%	0.0018%	Bacteria	Bacteroidota	Bacteroidia	Bacteroidales	Dysgonomonadaceae		
denovo4298	0.0000%	0.0000%	0.0031%	0.0010%	0.0018%	Bacteria	Firmicutes	Syntrophomonadia	Syntrophomonadales	Syntrophomonadaceae	Syntrophomonas	
denovo4299	0.0000%	0.0000%	0.0031%	0.0010%	0.0018%	Bacteria	Chloroflexi	Anaerolineae	Anaerolineales	Anaerolineaceae	uncultured	
denovo4306	0.0000%	0.0000%	0.0031%	0.0010%	0.0018%	Bacteria	Bacteroidota	Bacteroidia	Bacteroidales	Bacteroidaceae	Bacteroides	
denovo4329	0.0000%	0.0000%	0.0031%	0.0010%	0.0018%	Bacteria	Synergistota	Synergistia	Synergistales	Synergistaceae	EBM-39	uncultured_bacterium
denovo4336	0.0000%	0.0000%	0.0031%	0.0010%	0.0018%	Bacteria	Bacteroidota	Bacteroidia	Bacteroidales	Bacteroidaceae	Bacteroides	
denovo4351	0.0000%	0.0000%	0.0031%	0.0010%	0.0018%	Bacteria	Firmicutes	Desulfotomaculia	Desulfotomaculales	Desulfotomaculales	Cryptanaerobacter	
denovo4419	0.0000%	0.0000%	0.0031%	0.0010%	0.0018%	Bacteria						
denovo4426	0.0000%	0.0000%	0.0031%	0.0010%	0.0018%	Bacteria	Desulfobacterota	Desulfovibrionia	Desulfovibrionales	Desulfovibrionaceae	Desulfovibrio	
denovo451	0.0000%	0.0000%	0.0031%	0.0010%	0.0018%	Bacteria						
denovo4514	0.0000%	0.0000%	0.0031%	0.0010%	0.0018%	Bacteria	Bacteroidota	Bacteroidia	Bacteroidales			
denovo4671	0.0000%	0.0000%	0.0031%	0.0010%	0.0018%	Unassigned						
denovo4725	0.0000%	0.0000%	0.0031%	0.0010%	0.0018%	Unassigned						
denovo4728	0.0000%	0.0000%	0.0031%	0.0010%	0.0018%	Bacteria	Proteobacteria	Gammaproteobacteria	Chromatiales	Sedimenticolaceae	Sedimenticola	
denovo4754	0.0000%	0.0000%	0.0031%	0.0010%	0.0018%	Bacteria	Thermotogota	Thermotogae	Petrotogales	Petrotogaceae	AUTHM297	uncultured_bacterium
denovo4781	0.0000%	0.0000%	0.0031%	0.0010%	0.0018%	Bacteria	Rs-K70_termite_group	Rs-K70_termite_group	Rs-K70_termite_group	Rs-K70_termite_group	Rs-K70_termite_group	
denovo4782	0.0000%	0.0000%	0.0031%	0.0010%	0.0018%	Bacteria	Bacteroidota	Bacteroidia	Bacteroidales	Bacteroidaceae	Bacteroides	
denovo479	0.0000%	0.0000%	0.0031%	0.0010%	0.0018%	Bacteria	Bacteroidota	Bacteroidia	Bacteroidales	Dysgonomonadaceae		
denovo4797	0.0000%	0.0000%	0.0031%	0.0010%	0.0018%	Bacteria	Firmicutes	Syntrophomonadia	Syntrophomonadales	Syntrophomonadaceae	Syntrophomonas	
denovo4819	0.0000%	0.0000%	0.0031%	0.0010%	0.0018%	Bacteria	Desulfobacterota	Desulfovibrionia	Desulfovibrionales	Desulfovibrionaceae	Desulfovibrio	
denovo4822	0.0000%	0.0000%	0.0031%	0.0010%	0.0018%	Unassigned						
denovo4826	0.0000%	0.0000%	0.0031%	0.0010%	0.0018%	Bacteria	Bacteroidota	Bacteroidia	Bacteroidales			
denovo487	0.0000%	0.0000%	0.0031%	0.0010%	0.0018%	Bacteria	Firmicutes	Syntrophomonadia	Syntrophomonadales	Syntrophomonadaceae	Syntrophomonas	
denovo4884	0.0000%	0.0000%	0.0031%	0.0010%	0.0018%	Unassigned						
denovo4912	0.0000%	0.0000%	0.0031%	0.0010%	0.0018%	Bacteria	Firmicutes	Clostridia				
denovo492	0.0000%	0.0000%	0.0031%	0.0010%	0.0018%	Bacteria	Firmicutes	Clostridia				
denovo4932	0.0000%	0.0000%	0.0031%	0.0010%	0.0018%	Eukaryota						
denovo4954	0.0000%	0.0000%	0.0031%	0.0010%	0.0018%	Bacteria	Firmicutes	Clostridia	Eubacteriales	Anaerofustaceae	Anaerofustis	uncultured_bacterium
denovo498	0.0000%	0.0000%	0.0031%	0.0010%	0.0018%	Unassigned						
denovo4990	0.0000%	0.0000%	0.0031%	0.0010%	0.0018%	Bacteria	Firmicutes	Syntrophomonadia	Syntrophomonadales	Syntrophomonadaceae	Syntrophomonas	
denovo5028	0.0000%	0.0000%	0.0031%	0.0010%	0.0018%	Bacteria	Proteobacteria	Gammaproteobacteria	Burkholderiales	Aquaspirillaceae		
denovo5033	0.0000%	0.0000%	0.0031%	0.0010%	0.0018%	Bacteria	Synergistota	Synergistia	Synergistales	Synergistaceae	uncultured	
denovo5040	0.0000%	0.0000%	0.0031%	0.0010%	0.0018%	Bacteria	Firmicutes	Clostridia	Christensenellales	Christensenellaceae	Christensenellaceae_R-7_group	
denovo5052	0.0000%	0.0000%	0.0031%	0.0010%	0.0018%	Unassigned						
denovo5060	0.0000%	0.0000%	0.0031%	0.0010%	0.0018%	Bacteria	Firmicutes	Desulfotomaculia	Desulfotomaculales	Desulfotomaculales		
denovo5067	0.0000%	0.0000%	0.0031%	0.0010%	0.0018%	Bacteria	Firmicutes	Clostridia	Peptococcales	Peptococcaceae	uncultured	
denovo5078	0.0000%	0.0000%	0.0031%	0.0010%	0.0018%	Bacteria	Proteobacteria	Gammaproteobacteria	Enterobacterales	Enterobacteriaceae		
denovo5100	0.0000%	0.0000%	0.0031%	0.0010%	0.0018%	Bacteria	Firmicutes	Clostridia				
denovo5113	0.0000%	0.0000%	0.0031%	0.0010%	0.0018%	Bacteria	Firmicutes	Clostridia	Lachnospirales	Lachnospiraceae		
denovo5122	0.0000%	0.0000%	0.0031%	0.0010%	0.0018%	Bacteria	Synergistota	Synergistia	Synergistales	Synergistaceae	uncultured	uncultured_bacterium
denovo5157	0.0000%	0.0000%	0.0031%	0.0010%	0.0018%	Bacteria	Verrucomicrobiota	Lentisphaeria	Victivallales	Victivallaceae	Victivallis	uncultured_bacterium
denovo5205	0.0000%	0.0000%	0.0031%	0.0010%	0.0018%	Unassigned						
denovo5210	0.0000%	0.0000%	0.0031%	0.0010%	0.0018%	Bacteria	Firmicutes	Syntrophomonadia	Syntrophomonadales	Syntrophomonadaceae	Syntrophomonas	
denovo5257	0.0000%	0.0000%	0.0031%	0.0010%	0.0018%	Bacteria	Firmicutes	Syntrophomonadia	Syntrophomonadales	Syntrophomonadaceae		
denovo5285	0.0000%	0.0000%	0.0031%	0.0010%	0.0018%	Bacteria	Bacteroidota	Bacteroidia	Bacteroidales	Bacteroidetes_vadinHA17	Bacteroidetes_vadinHA17	
denovo5309	0.0000%	0.0000%	0.0031%	0.0010%	0.0018%	Bacteria	Firmicutes	Clostridia				
denovo5362	0.0000%	0.0000%	0.0031%	0.0010%	0.0018%	Bacteria	Firmicutes	Clostridia	Lachnospirales	Lachnospiraceae	uncultured	bacterium_enrichment
denovo5401	0.0000%	0.0000%	0.0031%	0.0010%	0.0018%	Bacteria	Firmicutes	Clostridia	Christensenellales	Christensenellaceae	Christensenellaceae_R-7_group	
denovo5416	0.0000%	0.0000%	0.0031%	0.0010%	0.0018%	Bacteria	Firmicutes	Clostridia	Christensenellales	Christensenellaceae	Christensenellaceae_R-7_group	
denovo5432	0.0000%	0.0000%	0.0031%	0.0010%	0.0018%	Bacteria	Firmicutes	Clostridia	Christensenellales	Christensenellaceae	Christensenellaceae_R-7_group	
denovo5436	0.0000%	0.0000%	0.0031%	0.0010%	0.0018%	Bacteria	Firmicutes	Clostridia	Oscillospirales	Ruminococcaceae	Anaerofilum	
denovo5451	0.0000%	0.0000%	0.0031%	0.0010%	0.0018%	Bacteria	Firmicutes	Clostridia	Peptostreptococcales-Tissierellales	Anaerovoracaceae		
denovo5496	0.0000%	0.0000%	0.0031%	0.0010%	0.0018%	Bacteria	Firmicutes	Syntrophomonadia	Syntrophomonadales	Syntrophomonadaceae	Syntrophomonas	

denovo5527	0.0000%	0.0000%	0.0031%	0.0010%	0.0018%	Bacteria	Spirochaetota	Spirochaetia	Spirochaetales	Spirochaetaceae	Sphaerochaeta	bacterium_enrichment
denovo5530	0.0000%	0.0000%	0.0031%	0.0010%	0.0018%	Bacteria	Synergistota	Synergistia	Synergistales	Synergistaceae	uncultured	uncultured_bacterium
denovo5553	0.0000%	0.0000%	0.0031%	0.0010%	0.0018%	Unassigned						
denovo5577	0.0000%	0.0000%	0.0031%	0.0010%	0.0018%	Bacteria	Proteobacteria	Gammaproteobacteria	Burkholderiales	Comamonadaceae		
denovo5579	0.0000%	0.0000%	0.0031%	0.0010%	0.0018%	Bacteria						
denovo5596	0.0000%	0.0000%	0.0031%	0.0010%	0.0018%	Bacteria	Synergistota	Synergistia	Synergistales	Synergistaceae	Aminiphilus	uncultured_bacterium
denovo5601	0.0000%	0.0000%	0.0031%	0.0010%	0.0018%	Bacteria	Spirochaetota	Spirochaetia	Spirochaetales	Spirochaetaceae	uncultured	uncultured_Treponema
denovo5608	0.0000%	0.0000%	0.0031%	0.0010%	0.0018%	Bacteria	Spirochaetota	Spirochaetia	Spirochaetales	Spirochaetaceae	uncultured	
denovo5609	0.0000%	0.0000%	0.0031%	0.0010%	0.0018%	Bacteria	Planctomycetota	vadinHA49	vadinHA49	vadinHA49	vadinHA49	uncultured_organism
denovo5635	0.0000%	0.0000%	0.0031%	0.0010%	0.0018%	Bacteria	Bacteroidota	Kapabacteria	Kapabacteriales	Kapabacteriales	Kapabacteriales	
denovo5720	0.0000%	0.0000%	0.0031%	0.0010%	0.0018%	Unassigned						
denovo5739	0.0000%	0.0000%	0.0031%	0.0010%	0.0018%	Unassigned						
denovo5746	0.0000%	0.0000%	0.0031%	0.0010%	0.0018%	Bacteria	Bacteroidota	Bacteroidia	Bacteroidales	Dysgonomonadaceae		
denovo5755	0.0000%	0.0000%	0.0031%	0.0010%	0.0018%	Bacteria	Bacteroidota	Kapabacteria	Kapabacteriales	Kapabacteriales	Kapabacteriales	uncultured_bacterium
denovo5763	0.0000%	0.0000%	0.0031%	0.0010%	0.0018%	Bacteria	Firmicutes	Clostridia	Peptococcales	Peptococcaceae	uncultured	
denovo5834	0.0000%	0.0000%	0.0031%	0.0010%	0.0018%	Bacteria	Proteobacteria	Alphaproteobacteria	Rhizobiales			
denovo5847	0.0000%	0.0000%	0.0031%	0.0010%	0.0018%	Bacteria	Desulfobacterota	Desulfovibrionia	Desulfovibrionales	Desulfovibrionaceae	Desulfovibrio	uncultured_Firmicutes
denovo5853	0.0000%	0.0000%	0.0031%	0.0010%	0.0018%	Bacteria	Chloroflexi	Anaerolineae	Anaerolineales	Anaerolineaceae	uncultured	uncultured_hydrocarbon
denovo5887	0.0000%	0.0000%	0.0031%	0.0010%	0.0018%	Bacteria	Synergistota	Synergistia	Synergistales	Synergistaceae	Syner-01	uncultured_bacterium
denovo5931	0.0000%	0.0000%	0.0031%	0.0010%	0.0018%	Bacteria	FCPU426	FCPU426	FCPU426	FCPU426	FCPU426	uncultured_bacterium
denovo5959	0.0000%	0.0000%	0.0031%	0.0010%	0.0018%	Unassigned						
denovo5961	0.0000%	0.0000%	0.0031%	0.0010%	0.0018%	Bacteria	Firmicutes	Clostridia				
denovo5988	0.0000%	0.0000%	0.0031%	0.0010%	0.0018%	Bacteria	Firmicutes	Syntrophomonadia	Syntrophomonadales	Syntrophomonadaceae	Syntrophomonas	
denovo6057	0.0000%	0.0000%	0.0031%	0.0010%	0.0018%	Bacteria	Firmicutes	Bacilli	Lactobacillales	Enterococcaceae	Enterococcus	
denovo6062	0.0000%	0.0000%	0.0031%	0.0010%	0.0018%	Bacteria	Bacteroidota	Bacteroidia				
denovo6077	0.0000%	0.0000%	0.0031%	0.0010%	0.0018%	Bacteria	Firmicutes	Clostridia	Christensenellales	Christensenellaceae		
denovo6081	0.0000%	0.0000%	0.0031%	0.0010%	0.0018%	Bacteria	Bacteroidota	Bacteroidia	Bacteroidales	Bacteroidetes_BD2-2	Bacteroidetes_BD2-2	
denovo6142	0.0000%	0.0000%	0.0031%	0.0010%	0.0018%	Bacteria	Synergistota	Synergistia	Synergistales	Synergistaceae	uncultured	bacterium_enrichment
denovo6153	0.0000%	0.0000%	0.0031%	0.0010%	0.0018%	Bacteria	Firmicutes	Clostridia	Oscillospirales	Oscillospiraceae		
denovo6194	0.0000%	0.0000%	0.0031%	0.0010%	0.0018%	Bacteria	Bacteroidota	Bacteroidia				
denovo6195	0.0000%	0.0000%	0.0031%	0.0010%	0.0018%	Bacteria	Bacteroidota	Bacteroidia	Bacteroidales	Bacteroidaceae	Bacteroides	
denovo6211	0.0000%	0.0000%	0.0031%	0.0010%	0.0018%	Archaea	Halobacterota	Methanomicrobia	Methanomicrobiales	Methanomicrobiaceae	Methanomicrobium	
denovo6223	0.0000%	0.0000%	0.0031%	0.0010%	0.0018%	Unassigned						
denovo6225	0.0000%	0.0000%	0.0031%	0.0010%	0.0018%	Bacteria	Firmicutes	Clostridia	Peptococcales	Peptococcaceae	uncultured	uncultured_Dehalobacterium
denovo6227	0.0000%	0.0000%	0.0031%	0.0010%	0.0018%	Bacteria	Firmicutes	Clostridia	Oscillospirales	Ruminococcaceae	uncultured	uncultured_Firmicutes
denovo6247	0.0000%	0.0000%	0.0031%	0.0010%	0.0018%	Bacteria	Bacteroidota	Bacteroidia	Sphingobacteriales	Lentimicrobiaceae	Lentimicrobium	
denovo6251	0.0000%	0.0000%	0.0031%	0.0010%	0.0018%	Unassigned						
denovo6293	0.0000%	0.0000%	0.0031%	0.0010%	0.0018%	Bacteria	Bacteroidota	Bacteroidia	Bacteroidales	Bacteroidetes_vadinHA17	Bacteroidetes_vadinHA17	
denovo630	0.0000%	0.0000%	0.0031%	0.0010%	0.0018%	Bacteria	Actinobacteriota	Coriobacteriia	Coriobacteriales	uncultured	uncultured	
denovo6301	0.0000%	0.0000%	0.0031%	0.0010%	0.0018%	Bacteria	Firmicutes	Clostridia				
denovo6309	0.0000%	0.0000%	0.0031%	0.0010%	0.0018%	Bacteria	Bacteroidota	Bacteroidia	Bacteroidales	Dysgonomonadaceae	Petrimonas	
denovo631	0.0000%	0.0000%	0.0031%	0.0010%	0.0018%	Bacteria	Desulfobacterota					
denovo6339	0.0000%	0.0000%	0.0031%	0.0010%	0.0018%	Bacteria	Firmicutes	Clostridia				
denovo6352	0.0000%	0.0000%	0.0031%	0.0010%	0.0018%	Bacteria	Acidobacteriota	Acidobacteriae	Bryobacterales	Bryobacteraceae	Bryobacter	
denovo6380	0.0000%	0.0000%	0.0031%	0.0010%	0.0018%	Bacteria	Synergistota	Synergistia	Synergistales	Synergistaceae	uncultured	uncultured_bacterium
denovo6406	0.0000%	0.0000%	0.0031%	0.0010%	0.0018%	Unassigned						
denovo6430	0.0000%	0.0000%	0.0031%	0.0010%	0.0018%	Archaea	Euryarchaeota	Methanobacteria	Methanobacteriales	Methanobacteriaceae	Methanobacterium	
denovo6440	0.0000%	0.0000%	0.0031%	0.0010%	0.0018%	Bacteria	Bacteroidota	Bacteroidia	Bacteroidales	Williamwhitmaniaceae	Blvii28_wastewater-sludge_group	
denovo6467	0.0000%	0.0000%	0.0031%	0.0010%	0.0018%	Bacteria	Firmicutes	Clostridia	Christensenellales	Christensenellaceae	Christensenellaceae_R-7_group	uncultured_soil
denovo6523	0.0000%	0.0000%	0.0031%	0.0010%	0.0018%	Bacteria	Synergistota	Synergistia	Synergistales	Synergistaceae	uncultured	
denovo6524	0.0000%	0.0000%	0.0031%	0.0010%	0.0018%	Bacteria	Firmicutes	Clostridia	Oscillospirales	Oscillospiraceae		
denovo6548	0.0000%	0.0000%	0.0031%	0.0010%	0.0018%	Bacteria	Firmicutes	Clostridia	Peptococcales	Peptococcaceae	uncultured	
denovo6588	0.0000%	0.0000%	0.0031%	0.0010%	0.0018%	Bacteria	Firmicutes	Clostridia	Peptococcales	Peptococcaceae	uncultured	
denovo6612	0.0000%	0.0000%	0.0031%	0.0010%	0.0018%	Bacteria	Bacteroidota	Bacteroidia	Bacteroidales	Bacteroidaceae	Bacteroides	
denovo6655	0.0000%	0.0000%	0.0031%	0.0010%	0.0018%	Bacteria	Firmicutes	Syntrophomonadia	Syntrophomonadales	Syntrophomonadaceae	Syntrophomonas	
denovo6679	0.0000%	0.0000%	0.0031%	0.0010%	0.0018%	Bacteria	Chloroflexi	Anaerolineae	SBR1031	SBR1031	SBR1031	
denovo6702	0.0000%	0.0000%	0.0031%	0.0010%	0.0018%	Bacteria	Firmicutes	Syntrophomonadia	Syntrophomonadales	Syntrophomonadaceae	Syntrophomonas	
denovo673	0.0000%	0.0000%	0.0031%	0.0010%	0.0018%	Bacteria	Myxococcota	Polyangia	UASB-TL25	UASB-TL25	UASB-TL25	uncultured_bacterium
denovo6738	0.0000%	0.0000%	0.0031%	0.0010%	0.0018%	Bacteria	Firmicutes	Clostridia	Peptococcales	Peptococcaceae	uncultured	uncultured_Peptococcaceae
denovo6757	0.0000%	0.0000%	0.0031%	0.0010%	0.0018%	Bacteria	Bacteroidota	Bacteroidia	Bacteroidales	Dysgonomonadaceae	Proteiniphilum	

denovo6829	0.0000%	0.0000%	0.0031%	0.0010%	0.0018%	Bacteria	Synergistota	Synergistia	Synergistales	Synergistaceae	Syner-01	
denovo6849	0.0000%	0.0000%	0.0031%	0.0010%	0.0018%	Bacteria	Bacteroidota	Ignavibacteria	Ignavibacteriales			
denovo6876	0.0000%	0.0000%	0.0031%	0.0010%	0.0018%	Bacteria	Firmicutes	Clostridia				
denovo6898	0.0000%	0.0000%	0.0031%	0.0010%	0.0018%	Unassigned						
denovo6933	0.0000%	0.0000%	0.0031%	0.0010%	0.0018%	Bacteria	Desulfobacterota	Desulfuromonadia	Geobacterales	Geobacteraceae	Geobacter	
denovo6942	0.0000%	0.0000%	0.0031%	0.0010%	0.0018%	Bacteria	Nitrospirota	Nitrospiria	Nitrospirales	Nitrospiraceae	Nitrospira	
denovo6995	0.0000%	0.0000%	0.0031%	0.0010%	0.0018%	Bacteria	Firmicutes					
denovo7005	0.0000%	0.0000%	0.0031%	0.0010%	0.0018%	Unassigned						
denovo708	0.0000%	0.0000%	0.0031%	0.0010%	0.0018%	Bacteria	Firmicutes	BRH-c20a	BRH-c20a	BRH-c20a	BRH-c20a	uncultured_bacterium
denovo7098	0.0000%	0.0000%	0.0031%	0.0010%	0.0018%	Bacteria	Spirochaetota	Spirochaetia	Spirochaetales	Spirochaetaceae	uncultured	
denovo7178	0.0000%	0.0000%	0.0031%	0.0010%	0.0018%	Bacteria	Bacteroidota	Bacteroidia	Bacteroidales	Dysgonomonadaceae		
denovo719	0.0000%	0.0000%	0.0031%	0.0010%	0.0018%	Bacteria	Firmicutes	Syntrophomonadia	Syntrophomonadales	Syntrophomonadaceae	Syntrophomonas	
denovo7193	0.0000%	0.0000%	0.0031%	0.0010%	0.0018%	Bacteria						
denovo720	0.0000%	0.0000%	0.0031%	0.0010%	0.0018%	Bacteria	Firmicutes	Clostridia	Oscillospirales	Ruminococcaceae	Anaerotruncus	Anaerotruncus_sp.
denovo7203	0.0000%	0.0000%	0.0031%	0.0010%	0.0018%	Bacteria	Bacteroidota	Bacteroidia	Bacteroidales	Dysgonomonadaceae	Petrimonas	
denovo7230	0.0000%	0.0000%	0.0031%	0.0010%	0.0018%	Bacteria	Proteobacteria	Alphaproteobacteria	Rhizobiales	Rhizobiales_Incertae_Sedis	Anderseniella	uncultured_bacterium
denovo7237	0.0000%	0.0000%	0.0031%	0.0010%	0.0018%	Bacteria	Desulfobacterota	Desulfobacteria	Desulfobacterales	Desulfosarcinaceae	uncultured	
denovo7264	0.0000%	0.0000%	0.0031%	0.0010%	0.0018%	Unassigned						
denovo73	0.0000%	0.0000%	0.0031%	0.0010%	0.0018%	Unassigned						
denovo7312	0.0000%	0.0000%	0.0031%	0.0010%	0.0018%	Bacteria	Firmicutes	Clostridia	Oscillospirales			
denovo7333	0.0000%	0.0000%	0.0031%	0.0010%	0.0018%	Bacteria	Desulfobacterota	Desulfovibrionia	Desulfovibrionales	Desulfovibrionaceae	Desulfovibrio	
denovo7341	0.0000%	0.0000%	0.0031%	0.0010%	0.0018%	Bacteria	Firmicutes	Clostridia				
denovo7351	0.0000%	0.0000%	0.0031%	0.0010%	0.0018%	Bacteria	Proteobacteria	Gammaproteobacteria	B2M28	B2M28	B2M28	
denovo7369	0.0000%	0.0000%	0.0031%	0.0010%	0.0018%	Bacteria	Bacteroidota	Bacteroidia				
denovo737	0.0000%	0.0000%	0.0031%	0.0010%	0.0018%	Bacteria	Spirochaetota	Spirochaetia	Spirochaetales	Spirochaetaceae	uncultured	
denovo7370	0.0000%	0.0000%	0.0031%	0.0010%	0.0018%	Bacteria	Bacteroidota	Bacteroidia				
denovo7404	0.0000%	0.0000%	0.0031%	0.0010%	0.0018%	Bacteria	Firmicutes	Clostridia	Clostridia	Gracilibacteraceae	uncultured	uncultured_bacterium
denovo7418	0.0000%	0.0000%	0.0031%	0.0010%	0.0018%	Bacteria	Spirochaetota	Spirochaetia	Spirochaetales	Spirochaetaceae	uncultured	uncultured_Treponema
denovo7438	0.0000%	0.0000%	0.0031%	0.0010%	0.0018%	Bacteria	Firmicutes	Clostridia				
denovo7456	0.0000%	0.0000%	0.0031%	0.0010%	0.0018%	Bacteria	Firmicutes	Clostridia	Oscillospirales	Oscillospiraceae		
denovo752	0.0000%	0.0000%	0.0031%	0.0010%	0.0018%	Bacteria	Bacteroidota	Bacteroidia	Bacteroidales	Dysgonomonadaceae	Proteiniphilum	
denovo7530	0.0000%	0.0000%	0.0031%	0.0010%	0.0018%	Archaea	Euryarchaeota	Methanobacteria	Methanobacteriales	Methanobacteriaceae	Methanobacterium	
denovo7536	0.0000%	0.0000%	0.0031%	0.0010%	0.0018%	Bacteria	Firmicutes	Clostridia				
denovo7537	0.0000%	0.0000%	0.0031%	0.0010%	0.0018%	Bacteria	Bacteroidota	Bacteroidia	Bacteroidales	Dysgonomonadaceae	Petrimonas	
denovo7538	0.0000%	0.0000%	0.0031%	0.0010%	0.0018%	Unassigned						
denovo7543	0.0000%	0.0000%	0.0031%	0.0010%	0.0018%	Bacteria	Firmicutes	Clostridia				
denovo7550	0.0000%	0.0000%	0.0031%	0.0010%	0.0018%	Bacteria	Firmicutes	Clostridia	Peptostreptococcales-Tissierellales	Anaerovoracaceae	Anaerovorax	
denovo7607	0.0000%	0.0000%	0.0031%	0.0010%	0.0018%	Bacteria	Synergistota	Synergistia	Synergistales	Synergistaceae	Thermovirga	uncultured_Synergistes
denovo762	0.0000%	0.0000%	0.0031%	0.0010%	0.0018%	Bacteria	Bacteroidota	Bacteroidia	Bacteroidales	Dysgonomonadaceae		
denovo7660	0.0000%	0.0000%	0.0031%	0.0010%	0.0018%	Bacteria	Synergistota	Synergistia	Synergistales	Synergistaceae	uncultured	bacterium_enrichment
denovo7681	0.0000%	0.0000%	0.0031%	0.0010%	0.0018%	Bacteria	Bacteroidota	Bacteroidia	Bacteroidales	Bacteroidetes_vadinHA17	Bacteroidetes_vadinHA17	
denovo7695	0.0000%	0.0000%	0.0031%	0.0010%	0.0018%	Bacteria						
denovo7715	0.0000%	0.0000%	0.0031%	0.0010%	0.0018%	Bacteria	Bacteroidota	Bacteroidia	Bacteroidales	Tannerellaceae	Parabacteroides	Blattella_germanica
denovo7755	0.0000%	0.0000%	0.0031%	0.0010%	0.0018%	Bacteria	Bacteroidota	Bacteroidia	Bacteroidales	Bacteroidaceae	Bacteroides	
denovo7767	0.0000%	0.0000%	0.0031%	0.0010%	0.0018%	Unassigned						
denovo7778	0.0000%	0.0000%	0.0031%	0.0010%	0.0018%	Bacteria	Bacteroidota	Bacteroidia	Bacteroidales	Dysgonomonadaceae		
denovo7784	0.0000%	0.0000%	0.0031%	0.0010%	0.0018%	Bacteria	Proteobacteria	Gammaproteobacteria	Burkholderiales	Comamonadaceae		
denovo7906	0.0000%	0.0000%	0.0031%	0.0010%	0.0018%	Bacteria	Bacteroidota	Bacteroidia	Bacteroidales	Dysgonomonadaceae	Petrimonas	
denovo7910	0.0000%	0.0000%	0.0031%	0.0010%	0.0018%	Bacteria	Synergistota	Synergistia	Synergistales	Synergistaceae	Syner-01	
denovo7962	0.0000%	0.0000%	0.0031%	0.0010%	0.0018%	Bacteria	Planctomycetota	vadinHA49	vadinHA49	vadinHA49	vadinHA49	
denovo7969	0.0000%	0.0000%	0.0031%	0.0010%	0.0018%	Bacteria	Bacteroidota	Bacteroidia	Bacteroidales			
denovo7978	0.0000%	0.0000%	0.0031%	0.0010%	0.0018%	Bacteria	Firmicutes	Clostridia	Christensenellales	Christensenellaceae	Christensenellaceae_R-7_group	
denovo7982	0.0000%	0.0000%	0.0031%	0.0010%	0.0018%	Bacteria	Firmicutes	Clostridia	Oscillospirales	Ruminococcaceae		
denovo7988	0.0000%	0.0000%	0.0031%	0.0010%	0.0018%	Bacteria	Firmicutes	Clostridia	Eubacteriales	Eubacteriaceae	uncultured	uncultured_bacterium
denovo801	0.0000%	0.0000%	0.0031%	0.0010%	0.0018%	Bacteria	Firmicutes	Clostridia	Oscillospirales	Ruminococcaceae	uncultured	
denovo8024	0.0000%	0.0000%	0.0031%	0.0010%	0.0018%	Bacteria	Bacteroidota	Bacteroidia	Bacteroidales	Bacteroidetes_vadinHA17	Bacteroidetes_vadinHA17	
denovo8026	0.0000%	0.0000%	0.0031%	0.0010%	0.0018%	Bacteria						
denovo8054	0.0000%	0.0000%	0.0031%	0.0010%	0.0018%	Bacteria	Firmicutes	Clostridia				
denovo8057	0.0000%	0.0000%	0.0031%	0.0010%	0.0018%	Bacteria	Firmicutes	Clostridia	Christensenellales	Christensenellaceae		
denovo8101	0.0000%	0.0000%	0.0031%	0.0010%	0.0018%	Bacteria	Firmicutes	Syntrophomonadia	Syntrophomonadales	Syntrophomonadaceae	Syntrophomonas	

denovo8125	0.0000%	0.0000%	0.0031%	0.0010%	0.0018%	Bacteria	Chloroflexi	Anaerolineae				
denovo8170	0.0000%	0.0000%	0.0031%	0.0010%	0.0018%	Bacteria	Firmicutes	Clostridia	Christensenellales	Christensenellaceae	Christensenellaceae_R-7_group	
denovo8188	0.0000%	0.0000%	0.0031%	0.0010%	0.0018%	Bacteria	Bacteroidota	Bacteroidia	Bacteroidales	Williamwhitmaniaceae	Blvii28_wastewater-sludge_group	uncultured_bacterium
denovo821	0.0000%	0.0000%	0.0031%	0.0010%	0.0018%	Bacteria	Firmicutes	Clostridia	Christensenellales	Christensenellaceae		
denovo8248	0.0000%	0.0000%	0.0031%	0.0010%	0.0018%	Bacteria	Desulfobacterota	Syntrophobacteria	Syntrophobacterales	Syntrophobacteraceae	Syntrophobacter	
denovo8268	0.0000%	0.0000%	0.0031%	0.0010%	0.0018%	Archaea	Euryarchaeota	Methanobacteria	Methanobacteriales	Methanobacteriaceae	Methanobacterium	
denovo8278	0.0000%	0.0000%	0.0031%	0.0010%	0.0018%	Archaea	Euryarchaeota	Methanobacteria	Methanobacteriales	Methanobacteriaceae	Methanobacterium	
denovo8348	0.0000%	0.0000%	0.0031%	0.0010%	0.0018%	Bacteria						
denovo8350	0.0000%	0.0000%	0.0031%	0.0010%	0.0018%	Bacteria	Firmicutes	Bacilli	Izemoplasmatales	Izemoplasmatales	Izemoplasmatales	uncultured_bacterium
denovo8363	0.0000%	0.0000%	0.0031%	0.0010%	0.0018%	Bacteria	Bacteroidota	Bacteroidia	Bacteroidales	Bacteroidaceae	Bacteroides	
denovo838	0.0000%	0.0000%	0.0031%	0.0010%	0.0018%	Bacteria	Firmicutes	Clostridia	Lachnospirales	Lachnospiraceae		
denovo8385	0.0000%	0.0000%	0.0031%	0.0010%	0.0018%	Bacteria	Firmicutes	Bacilli	Lactobacillales	Enterococcaceae	Enterococcus	
denovo8389	0.0000%	0.0000%	0.0031%	0.0010%	0.0018%	Bacteria	Firmicutes	Desulfotomaculia	Desulfotomaculales			
denovo8442	0.0000%	0.0000%	0.0031%	0.0010%	0.0018%	Bacteria	Firmicutes	Clostridia				
denovo8450	0.0000%	0.0000%	0.0031%	0.0010%	0.0018%	Bacteria	Firmicutes	Desulfotomaculia	Desulfotomaculales	Desulfotomaculales	Pelotomaculum	
denovo8513	0.0000%	0.0000%	0.0031%	0.0010%	0.0018%	Bacteria	Synergistota	Synergistia	Synergistales	Synergistaceae		
denovo8531	0.0000%	0.0000%	0.0031%	0.0010%	0.0018%	Bacteria	Firmicutes	Clostridia	Christensenellales	Christensenellaceae	Christensenellaceae_R-7_group	
denovo8536	0.0000%	0.0000%	0.0031%	0.0010%	0.0018%	Bacteria	Firmicutes	Clostridia	Oscillospirales			
denovo8537	0.0000%	0.0000%	0.0031%	0.0010%	0.0018%	Bacteria	Desulfobacterota	Desulfovibrionia	Desulfovibrionales	Desulfovibrionaceae	Desulfovibrio	
denovo8538	0.0000%	0.0000%	0.0031%	0.0010%	0.0018%	Bacteria	Desulfobacterota	Desulfovibrionia	Desulfovibrionales	Desulfovibrionaceae	Desulfovibrio	
denovo8549	0.0000%	0.0000%	0.0031%	0.0010%	0.0018%	Bacteria	Firmicutes	Bacilli	Lactobacillales			
denovo8566	0.0000%	0.0000%	0.0031%	0.0010%	0.0018%	Bacteria	Bacteroidota	Bacteroidia	Bacteroidales			
denovo8571	0.0000%	0.0000%	0.0031%	0.0010%	0.0018%	Bacteria	Proteobacteria	Gammaproteobacteria	Burkholderiales	Rhodocyclaceae	Thauera	
denovo861	0.0000%	0.0000%	0.0031%	0.0010%	0.0018%	Bacteria	Desulfobacterota	Desulfovibrionia	Desulfovibrionales	Desulfovibrionaceae	Desulfovibrio	
denovo8614	0.0000%	0.0000%	0.0031%	0.0010%	0.0018%	Unassigned						
denovo8636	0.0000%	0.0000%	0.0031%	0.0010%	0.0018%	Bacteria	Synergistota	Synergistia	Synergistales	Synergistaceae	uncultured	
denovo8649	0.0000%	0.0000%	0.0031%	0.0010%	0.0018%	Bacteria	Desulfobacterota	Desulfovibrionia	Desulfovibrionales	Desulfovibrionaceae	Desulfovibrio	
denovo8662	0.0000%	0.0000%	0.0031%	0.0010%	0.0018%	Bacteria	Synergistota	Synergistia	Synergistales	Synergistaceae		
denovo8672	0.0000%	0.0000%	0.0031%	0.0010%	0.0018%	Bacteria	Bacteroidota	Bacteroidia	Bacteroidales	Dysgonomonadaceae	Petrimonas	
denovo8675	0.0000%	0.0000%	0.0031%	0.0010%	0.0018%	Bacteria	Firmicutes	Syntrophomonadia	Syntrophomonadales	Syntrophomonadaceae	Syntrophomonas	
denovo8691	0.0000%	0.0000%	0.0031%	0.0010%	0.0018%	Bacteria	Synergistota	Synergistia	Synergistales	Synergistaceae		
denovo8710	0.0000%	0.0000%	0.0031%	0.0010%	0.0018%	Bacteria	Desulfobacterota	Desulfuromonadia	Geobacterales	Geobacteraceae	Geobacter	
denovo8752	0.0000%	0.0000%	0.0031%	0.0010%	0.0018%	Bacteria						
denovo8754	0.0000%	0.0000%	0.0031%	0.0010%	0.0018%	Bacteria						
denovo8786	0.0000%	0.0000%	0.0031%	0.0010%	0.0018%	Bacteria						
denovo8799	0.0000%	0.0000%	0.0031%	0.0010%	0.0018%	Bacteria	Patescibacteria	Microgenomatia	Candidatus_Woesebacteria	Candidatus_Woesebacteria	Candidatus_Woesebacteria	uncultured_bacterium
denovo88	0.0000%	0.0000%	0.0031%	0.0010%	0.0018%	Bacteria	Firmicutes	Syntrophomonadia	Syntrophomonadales	Syntrophomonadaceae		
denovo8825	0.0000%	0.0000%	0.0031%	0.0010%	0.0018%	Bacteria	Firmicutes	Clostridia	Oscillospirales	Oscillospiraceae	Colidextribacter	
denovo8832	0.0000%	0.0000%	0.0031%	0.0010%	0.0018%	Bacteria						
denovo8886	0.0000%	0.0000%	0.0031%	0.0010%	0.0018%	Bacteria	Firmicutes	Clostridia	Christensenellales	Christensenellaceae	uncultured	
denovo8912	0.0000%	0.0000%	0.0031%	0.0010%	0.0018%	Bacteria	Bacteroidota	Bacteroidia	Bacteroidales	Bacteroidaceae	Bacteroides	
denovo8915	0.0000%	0.0000%	0.0031%	0.0010%	0.0018%	Bacteria	Firmicutes	Syntrophomonadia	Syntrophomonadales	Syntrophomonadaceae	Syntrophomonas	
denovo8944	0.0000%	0.0000%	0.0031%	0.0010%	0.0018%	Bacteria	Firmicutes	Desulfotomaculia	Desulfotomaculales	Desulfotomaculales	Pelotomaculum	
denovo8967	0.0000%	0.0000%	0.0031%	0.0010%	0.0018%	Archaea	Euryarchaeota	Methanobacteria	Methanobacteriales	Methanobacteriaceae	Methanobacterium	archaeon_enrichment
denovo8970	0.0000%	0.0000%	0.0031%	0.0010%	0.0018%	Bacteria	Firmicutes	uncultured	uncultured	uncultured	uncultured	
denovo9003	0.0000%	0.0000%	0.0031%	0.0010%	0.0018%	Bacteria	Firmicutes	Syntrophomonadia	Syntrophomonadales	Syntrophomonadaceae	Syntrophomonas	
denovo9009	0.0000%	0.0000%	0.0031%	0.0010%	0.0018%	Unassigned						
denovo9010	0.0000%	0.0000%	0.0031%	0.0010%	0.0018%	Eukaryota						
denovo9017	0.0000%	0.0000%	0.0031%	0.0010%	0.0018%	Bacteria	Firmicutes	Desulfotomaculia	Desulfotomaculales	Desulfotomaculales	Cryptanaerobacter	
denovo9029	0.0000%	0.0000%	0.0031%	0.0010%	0.0018%	Bacteria	Bacteroidota	Bacteroidia	Bacteroidales			
denovo9030	0.0000%	0.0000%	0.0031%	0.0010%	0.0018%	Bacteria	Synergistota	Synergistia	Synergistales	Synergistaceae	Pyramidobacter	uncultured_bacterium
denovo9036	0.0000%	0.0000%	0.0031%	0.0010%	0.0018%	Bacteria	Firmicutes	Clostridia	Oscillospirales	Ruminococcaceae	uncultured	
denovo9046	0.0000%	0.0000%	0.0031%	0.0010%	0.0018%	Bacteria						
denovo948	0.0000%	0.0000%	0.0031%	0.0010%	0.0018%	Bacteria	Firmicutes	uncultured	uncultured	uncultured	uncultured	
denovo959	0.0000%	0.0000%	0.0031%	0.0010%	0.0018%	Bacteria	Firmicutes	Clostridia				
denovo978	0.0000%	0.0000%	0.0031%	0.0010%	0.0018%	Bacteria	Firmicutes					
denovo1008	0.0031%	0.0000%	0.0000%	0.0010%	0.0018%	Unassigned						
denovo1027	0.0031%	0.0000%	0.0000%	0.0010%	0.0018%	Bacteria	Thermotogota	Thermotogae	Kosmotogales	Kosmotogaceae	Mesotoga	
denovo1118	0.0031%	0.0000%	0.0000%	0.0010%	0.0018%	Bacteria	Firmicutes	Clostridia	Oscillospirales	Ruminococcaceae	Ruminococcus	
denovo1151	0.0031%	0.0000%	0.0000%	0.0010%	0.0018%	Archaea	Euryarchaeota	Methanobacteria	Methanobacteriales	Methanobacteriaceae	Methanobrevibacter	Methanobrevibacter_arboriphilus

denovo1154	0.0031%	0.0000%	0.0000%	0.0010%	0.0018%	Unassigned						
denovo1199	0.0031%	0.0000%	0.0000%	0.0010%	0.0018%	Unassigned						
denovo1213	0.0031%	0.0000%	0.0000%	0.0010%	0.0018%	Bacteria	Bacteroidota	Bacteroidia	Bacteroidales	Williamwhitmaniaceae	Blvii28_wastewater-sludge_group	
denovo1232	0.0031%	0.0000%	0.0000%	0.0010%	0.0018%	Bacteria	Planctomycetota	vadinHA49	vadinHA49	vadinHA49	vadinHA49	uncultured_Planctomycetaceae
denovo1280	0.0031%	0.0000%	0.0000%	0.0010%	0.0018%	Bacteria	Firmicutes	Clostridia	Christensenellales	Christensenellaceae	Christensenellaceae_R-7_group	
denovo1294	0.0031%	0.0000%	0.0000%	0.0010%	0.0018%	Eukaryota						
denovo1304	0.0031%	0.0000%	0.0000%	0.0010%	0.0018%	Bacteria	Firmicutes	Bacilli	Lactobacillales	Enterococcaceae	Enterococcus	
denovo1319	0.0031%	0.0000%	0.0000%	0.0010%	0.0018%	Bacteria	Bacteroidota	Bacteroidia	Bacteroidales	Bacteroidetes_vadinHA17	Bacteroidetes_vadinHA17	
denovo1323	0.0031%	0.0000%	0.0000%	0.0010%	0.0018%	Bacteria	Firmicutes	Syntrophomonadia	Syntrophomonadales	Syntrophomonadaceae		
denovo1333	0.0031%	0.0000%	0.0000%	0.0010%	0.0018%	Bacteria	Firmicutes	Clostridia	Oscillospirales			
denovo1352	0.0031%	0.0000%	0.0000%	0.0010%	0.0018%	Bacteria	Firmicutes	Clostridia	Oscillospirales	Ruminococcaceae	uncultured	
denovo137	0.0031%	0.0000%	0.0000%	0.0010%	0.0018%	Bacteria	Firmicutes	Clostridia	Christensenellales	Christensenellaceae	Christensenellaceae_R-7_group	
denovo142	0.0031%	0.0000%	0.0000%	0.0010%	0.0018%	Bacteria	Caldatribacteriota	JS1	JS1	JS1	JS1	
denovo1459	0.0031%	0.0000%	0.0000%	0.0010%	0.0018%	Bacteria	Acidobacteriota	Thermoanaerobaculia	Thermoanaerobaculales	Thermoanaerobaculaceae	Subgroup_10	
denovo1534	0.0031%	0.0000%	0.0000%	0.0010%	0.0018%	Archaea	Halobacterota	Methanomicrobia	Methanomicrobiales	Methanospirillaceae	Methanospirillum	archaeon_enrichment
denovo1548	0.0031%	0.0000%	0.0000%	0.0010%	0.0018%	Bacteria	Firmicutes	Clostridia				
denovo1597	0.0031%	0.0000%	0.0000%	0.0010%	0.0018%	Bacteria	Firmicutes	Clostridia	Clostridia	Hungateiclostridiaceae	uncultured	
denovo16	0.0031%	0.0000%	0.0000%	0.0010%	0.0018%	Bacteria	Firmicutes	Clostridia	Oscillospirales	Ruminococcaceae	uncultured	
denovo1603	0.0031%	0.0000%	0.0000%	0.0010%	0.0018%	Bacteria	Firmicutes	Clostridia				
denovo1629	0.0031%	0.0000%	0.0000%	0.0010%	0.0018%	Bacteria	Firmicutes	Clostridia	Peptostreptococcales-Tissierellales	Peptostreptococcaceae	Romboutsia	
denovo1631	0.0031%	0.0000%	0.0000%	0.0010%	0.0018%	Bacteria	Bacteroidota	Bacteroidia	Bacteroidales	Bacteroidetes_vadinHA17	Bacteroidetes_vadinHA17	
denovo1639	0.0031%	0.0000%	0.0000%	0.0010%	0.0018%	Bacteria	Spirochaetota	Spirochaetia	Spirochaetales	Spirochaetaceae	Treponema	
denovo1650	0.0031%	0.0000%	0.0000%	0.0010%	0.0018%	Bacteria	Firmicutes	Clostridia	Clostridiales	Clostridiaceae	Clostridium_sensu_stricto_1	
denovo169	0.0031%	0.0000%	0.0000%	0.0010%	0.0018%	Bacteria	Firmicutes	Clostridia				
denovo1704	0.0031%	0.0000%	0.0000%	0.0010%	0.0018%	Bacteria	Firmicutes	Clostridia	Oscillospirales	Oscillospiraceae	Colidextribacter	
denovo1719	0.0031%	0.0000%	0.0000%	0.0010%	0.0018%	Bacteria	Firmicutes	Clostridia	Christensenellales	Christensenellaceae	Christensenellaceae_R-7_group	uncultured_Caloramator
denovo1777	0.0031%	0.0000%	0.0000%	0.0010%	0.0018%	Bacteria	Firmicutes	Clostridia				
denovo178	0.0031%	0.0000%	0.0000%	0.0010%	0.0018%	Bacteria	Firmicutes	Clostridia	Clostridia	Gracilibacteraceae		
denovo1829	0.0031%	0.0000%	0.0000%	0.0010%	0.0018%	Bacteria	Firmicutes	Syntrophomonadia	Syntrophomonadales	Syntrophomonadaceae	Syntrophomonas	
denovo1855	0.0031%	0.0000%	0.0000%	0.0010%	0.0018%	Bacteria	Desulfobacterota	Syntrophobacteria	Syntrophobacterales	Syntrophobacteraceae	Syntrophobacter	
denovo196	0.0031%	0.0000%	0.0000%	0.0010%	0.0018%	Bacteria	Firmicutes	Clostridia	Christensenellales	Christensenellaceae	Christensenellaceae	Christensenella_sp.
denovo1981	0.0031%	0.0000%	0.0000%	0.0010%	0.0018%	Bacteria	Synergistota	Synergistia	Synergistales	Synergistaceae	uncultured	bacterium_enrichment
denovo1983	0.0031%	0.0000%	0.0000%	0.0010%	0.0018%	Bacteria	Firmicutes	Clostridia	Christensenellales	Christensenellaceae	Christensenellaceae_R-7_group	
denovo205	0.0031%	0.0000%	0.0000%	0.0010%	0.0018%	Bacteria	Bacteroidota	Bacteroidia	Sphingobacteriales			
denovo2093	0.0031%	0.0000%	0.0000%	0.0010%	0.0018%	Bacteria	Firmicutes	Clostridia	Clostridia	Hungateiclostridiaceae		
denovo2145	0.0031%	0.0000%	0.0000%	0.0010%	0.0018%	Bacteria						
denovo216	0.0031%	0.0000%	0.0000%	0.0010%	0.0018%	Bacteria	Firmicutes	Clostridia	Christensenellales	Christensenellaceae	Christensenellaceae_R-7_group	
denovo2183	0.0031%	0.0000%	0.0000%	0.0010%	0.0018%	Bacteria	Proteobacteria	Gammaproteobacteria	Enterobacterales	Enterobacteriaceae		
denovo2192	0.0031%	0.0000%	0.0000%	0.0010%	0.0018%	Bacteria	Desulfobacterota					
denovo2276	0.0031%	0.0000%	0.0000%	0.0010%	0.0018%	Archaea	Halobacterota	Methanosarcinia	Methanosarciniales	Methanosaetaceae	Methanosaeta	
denovo2281	0.0031%	0.0000%	0.0000%	0.0010%	0.0018%	Bacteria	Chloroflexi	Anaerolineae	Anaerolineales	Anaerolineaceae		
denovo2335	0.0031%	0.0000%	0.0000%	0.0010%	0.0018%	Bacteria	Firmicutes	Clostridia	Oscillospirales	Ruminococcaceae	uncultured	
denovo2341	0.0031%	0.0000%	0.0000%	0.0010%	0.0018%	Bacteria	Bacteroidota	Bacteroidia	Bacteroidales	Bacteroidetes_vadinHA17	Bacteroidetes_vadinHA17	
denovo2381	0.0031%	0.0000%	0.0000%	0.0010%	0.0018%	Bacteria	Firmicutes	Bacilli	Lactobacillales	Enterococcaceae		
denovo2387	0.0031%	0.0000%	0.0000%	0.0010%	0.0018%	Bacteria	Verrucomicrobiota	Verrucomicrobiae	Pedosphaerales	Pedosphaeraceae	Pedosphaeraceae	
denovo2396	0.0031%	0.0000%	0.0000%	0.0010%	0.0018%	Bacteria	Firmicutes	uncultured	uncultured	uncultured	uncultured	uncultured_organism
denovo2436	0.0031%	0.0000%	0.0000%	0.0010%	0.0018%	Bacteria	Firmicutes					
denovo2442	0.0031%	0.0000%	0.0000%	0.0010%	0.0018%	Unassigned						
denovo2488	0.0031%	0.0000%	0.0000%	0.0010%	0.0018%	Bacteria	Firmicutes	Clostridia	Oscillospirales			
denovo2521	0.0031%	0.0000%	0.0000%	0.0010%	0.0018%	Bacteria	Desulfobacterota	Desulfobacteria	Desulfobacterales	Desulfosarcinaceae	Sva0081_sediment_group	
denovo2527	0.0031%	0.0000%	0.0000%	0.0010%	0.0018%	Bacteria	Bacteroidota	Bacteroidia	Bacteroidales	Dysgonomonadaceae	Proteiniphilum	
denovo2588	0.0031%	0.0000%	0.0000%	0.0010%	0.0018%	Bacteria	Desulfobacterota	Syntrophia	Syntrophales	Syntrophaceae	Syntrophus	
denovo2647	0.0031%	0.0000%	0.0000%	0.0010%	0.0018%	Bacteria	Firmicutes	Clostridia	Oscillospirales	Oscillospiraceae		
denovo269	0.0031%	0.0000%	0.0000%	0.0010%	0.0018%	Bacteria	Acidobacteriota	Blastocatellia	Blastocatellales	Blastocatellaceae	OLB17	uncultured_bacterium
denovo2745	0.0031%	0.0000%	0.0000%	0.0010%	0.0018%	Bacteria	Desulfobacterota	Syntrophobacteria	Syntrophobacterales	Syntrophobacteraceae	Syntrophobacter	uncultured_bacterium
denovo2754	0.0031%	0.0000%	0.0000%	0.0010%	0.0018%	Bacteria	Bacteroidota	Bacteroidia	Bacteroidales	Bacteroidetes_vadinHA17	Bacteroidetes_vadinHA17	
denovo2782	0.0031%	0.0000%	0.0000%	0.0010%	0.0018%	Bacteria	Chloroflexi	Anaerolineae	SBR1031	A4b	A4b	
denovo2810	0.0031%	0.0000%	0.0000%	0.0010%	0.0018%	Bacteria	Actinobacteriota	Thermoleophilia	Gaiellales	uncultured	uncultured	
denovo2812	0.0031%	0.0000%	0.0000%	0.0010%	0.0018%	Bacteria	Firmicutes	Bacilli	Lactobacillales			
denovo2863	0.0031%	0.0000%	0.0000%	0.0010%	0.0018%	Bacteria	Bacteroidota	Bacteroidia	Sphingobacteriales	Lentimicrobiaceae	Lentimicrobiaceae	

denovo287	0.0031%	0.0000%	0.0000%	0.0010%	0.0018%	Bacteria	Bacteroidota	Bacteroidia	Bacteroidales			
denovo2930	0.0031%	0.0000%	0.0000%	0.0010%	0.0018%	Bacteria	Firmicutes	Bacilli	Lactobacillales	Enterococcaceae	Enterococcus	
denovo294	0.0031%	0.0000%	0.0000%	0.0010%	0.0018%	Bacteria	Chloroflexi	Anaerolineae	Anaerolineales	Anaerolineaceae	Anaerolinea	uncultured_bacterium
denovo2953	0.0031%	0.0000%	0.0000%	0.0010%	0.0018%	Bacteria	Verrucomicrobiota	Kiritimatiellae	WCHB1-41	WCHB1-41	WCHB1-41	uncultured_organism
denovo2960	0.0031%	0.0000%	0.0000%	0.0010%	0.0018%	Bacteria	Firmicutes	Clostridia	Lachnospirales	Lachnospiraceae		
denovo3038	0.0031%	0.0000%	0.0000%	0.0010%	0.0018%	Bacteria	Chloroflexi	Anaerolineae	Anaerolineales	Anaerolineaceae	uncultured	uncultured_organism
denovo3216	0.0031%	0.0000%	0.0000%	0.0010%	0.0018%	Eukaryota						
denovo3225	0.0031%	0.0000%	0.0000%	0.0010%	0.0018%	Bacteria	Bacteroidota	Bacteroidia	Bacteroidales	Dysgonomonadaceae	Proteiniphilum	
denovo3241	0.0031%	0.0000%	0.0000%	0.0010%	0.0018%	Bacteria	Firmicutes	Clostridia	Oscillospirales	Ruminococcaceae	uncultured	
denovo3275	0.0031%	0.0000%	0.0000%	0.0010%	0.0018%	Bacteria	Firmicutes	Clostridia				
denovo3283	0.0031%	0.0000%	0.0000%	0.0010%	0.0018%	Bacteria	Firmicutes	Clostridia				
denovo3307	0.0031%	0.0000%	0.0000%	0.0010%	0.0018%	Bacteria	Planctomycetota	vadinHA49	vadinHA49	vadinHA49	vadinHA49	uncultured_Planctomycetaceae
denovo3319	0.0031%	0.0000%	0.0000%	0.0010%	0.0018%	Unassigned						
denovo3320	0.0031%	0.0000%	0.0000%	0.0010%	0.0018%	Bacteria	Verrucomicrobiota	Lentisphaeria	Oligosphaerales	Oligosphaeraceae	Oligosphaera	
denovo3377	0.0031%	0.0000%	0.0000%	0.0010%	0.0018%	Bacteria	Proteobacteria	Alphaproteobacteria	Rhizobiales	Xanthobacteraceae		
denovo3406	0.0031%	0.0000%	0.0000%	0.0010%	0.0018%	Bacteria	Firmicutes	Clostridia				
denovo3408	0.0031%	0.0000%	0.0000%	0.0010%	0.0018%	Bacteria	Firmicutes	Syntrophomonadia	Syntrophomonadales	Syntrophomonadaceae	Syntrophomonas	
denovo3495	0.0031%	0.0000%	0.0000%	0.0010%	0.0018%	Bacteria	Firmicutes	Clostridia	Peptostreptococcales-Tissierellales	Anaerovoracaceae		
denovo3496	0.0031%	0.0000%	0.0000%	0.0010%	0.0018%	Bacteria	Chloroflexi	Anaerolineae	Anaerolineales	Anaerolineaceae	uncultured	
denovo3548	0.0031%	0.0000%	0.0000%	0.0010%	0.0018%	Bacteria						
denovo3562	0.0031%	0.0000%	0.0000%	0.0010%	0.0018%	Bacteria	Firmicutes	Clostridia	Christensenellales	Christensenellaceae	Christensenellaceae_R-7_group	uncultured_Caloramator
denovo3563	0.0031%	0.0000%	0.0000%	0.0010%	0.0018%	Bacteria	Firmicutes	Clostridia	Oscillospirales	Oscillospiraceae	Oscillibacter	uncultured_Oscillibacter
denovo3567	0.0031%	0.0000%	0.0000%	0.0010%	0.0018%	Bacteria	Firmicutes	Clostridia	Christensenellales	Christensenellaceae	Christensenella	
denovo3568	0.0031%	0.0000%	0.0000%	0.0010%	0.0018%	Bacteria	Bacteroidota	Kapabacteria	Kapabacteriales	Kapabacteriales	Kapabacteriales	uncultured_bacterium
denovo3572	0.0031%	0.0000%	0.0000%	0.0010%	0.0018%	Bacteria	Firmicutes	Clostridia	Oscillospirales	Ruminococcaceae	uncultured	uncultured_Firmicutes
denovo3598	0.0031%	0.0000%	0.0000%	0.0010%	0.0018%	Bacteria	Spirochaetota	Spirochaetia	Spirochaetales	Spirochaetaceae	uncultured	uncultured_bacterium
denovo3624	0.0031%	0.0000%	0.0000%	0.0010%	0.0018%	Bacteria	Desulfobacterota	Syntrophorhabdia	Syntrophorhabdales	Syntrophorhabdaceae	Syntrophorhabdus	uncultured_bacterium
denovo3701	0.0031%	0.0000%	0.0000%	0.0010%	0.0018%	Bacteria	Firmicutes	Desulfotomaculia	Desulfotomaculales	Desulfotomaculales	Pelotomaculum	
denovo373	0.0031%	0.0000%	0.0000%	0.0010%	0.0018%	Bacteria	Proteobacteria	Gammaproteobacteria	Enterobacterales			
denovo3732	0.0031%	0.0000%	0.0000%	0.0010%	0.0018%	Bacteria	Chloroflexi	Anaerolineae	Anaerolineales	Anaerolineaceae		
denovo3758	0.0031%	0.0000%	0.0000%	0.0010%	0.0018%	Bacteria	Firmicutes	Clostridia	Lachnospirales	Lachnospiraceae		
denovo3763	0.0031%	0.0000%	0.0000%	0.0010%	0.0018%	Bacteria	Bacteroidota	Bacteroidia	Bacteroidales	Dysgonomonadaceae	Petrimonas	
denovo3806	0.0031%	0.0000%	0.0000%	0.0010%	0.0018%	Unassigned						
denovo3892	0.0031%	0.0000%	0.0000%	0.0010%	0.0018%	Bacteria	Thermotogota	Thermotogae	Kosmotogales	Kosmotogaceae	Mesotoga	
denovo4004	0.0031%	0.0000%	0.0000%	0.0010%	0.0018%	Bacteria	Firmicutes					
denovo405	0.0031%	0.0000%	0.0000%	0.0010%	0.0018%	Unassigned						
denovo4079	0.0031%	0.0000%	0.0000%	0.0010%	0.0018%	Bacteria	Verrucomicrobiota	Lentisphaeria	Victivallales	Victivallaceae	Victivallaceae	uncultured_Lentisphaerae
denovo4116	0.0031%	0.0000%	0.0000%	0.0010%	0.0018%	Bacteria	Bacteroidota	Bacteroidia	Bacteroidales	Dysgonomonadaceae	Petrimonas	
denovo4128	0.0031%	0.0000%	0.0000%	0.0010%	0.0018%	Unassigned						
denovo4148	0.0031%	0.0000%	0.0000%	0.0010%	0.0018%	Bacteria	Firmicutes	Clostridia	Christensenellales	Christensenellaceae		
denovo4254	0.0031%	0.0000%	0.0000%	0.0010%	0.0018%	Bacteria	Firmicutes	Clostridia				
denovo4259	0.0031%	0.0000%	0.0000%	0.0010%	0.0018%	Bacteria	Firmicutes	Clostridia	Peptostreptococcales-Tissierellales	Peptostreptococcaceae		
denovo427	0.0031%	0.0000%	0.0000%	0.0010%	0.0018%	Bacteria	Bacteroidota	Bacteroidia	Bacteroidales	Dysgonomonadaceae	Petrimonas	
denovo4295	0.0031%	0.0000%	0.0000%	0.0010%	0.0018%	Bacteria	Firmicutes	Clostridia	Oscillospirales	Ruminococcaceae	Candidatus_Soleaferrea	
denovo4303	0.0031%	0.0000%	0.0000%	0.0010%	0.0018%	Bacteria	Actinobacteriota	Actinobacteria	Micrococcales			
denovo4332	0.0031%	0.0000%	0.0000%	0.0010%	0.0018%	Bacteria	Firmicutes	Clostridia	Peptococcales	Peptococcaceae	uncultured	
denovo4347	0.0031%	0.0000%	0.0000%	0.0010%	0.0018%	Bacteria	Chloroflexi	Anaerolineae	Anaerolineales	Anaerolineaceae	Flexilinea	
denovo4364	0.0031%	0.0000%	0.0000%	0.0010%	0.0018%	Bacteria	Firmicutes	Clostridia	Peptococcales	Peptococcaceae	uncultured	
denovo438	0.0031%	0.0000%	0.0000%	0.0010%	0.0018%	Bacteria	Actinobacteriota	Thermoleophilia	Gaiellales	uncultured	uncultured	
denovo4444	0.0031%	0.0000%	0.0000%	0.0010%	0.0018%	Bacteria	Firmicutes	Clostridia	Christensenellales	Christensenellaceae	uncultured	
denovo4449	0.0031%	0.0000%	0.0000%	0.0010%	0.0018%	Bacteria	Firmicutes	Desulfotomaculia	Desulfotomaculales	Desulfotomaculales	Pelotomaculum	
denovo4486	0.0031%	0.0000%	0.0000%	0.0010%	0.0018%	Bacteria	Desulfobacterota	Syntrophobacteria	Syntrophobacterales	Syntrophobacteraceae	Syntrophobacter	
denovo4505	0.0031%	0.0000%	0.0000%	0.0010%	0.0018%	Unassigned						
denovo4617	0.0031%	0.0000%	0.0000%	0.0010%	0.0018%	Bacteria	Firmicutes	Clostridia	Oscillospirales	Ruminococcaceae	uncultured	uncultured_Firmicutes
denovo4626	0.0031%	0.0000%	0.0000%	0.0010%	0.0018%	Bacteria	Firmicutes	Clostridia	Clostridiales	Clostridiaceae		
denovo4777	0.0031%	0.0000%	0.0000%	0.0010%	0.0018%	Bacteria	Bacteroidota	Bacteroidia	Bacteroidales	Prolixibacteraceae		
denovo4808	0.0031%	0.0000%	0.0000%	0.0010%	0.0018%	Bacteria	Bacteroidota	Bacteroidia	Bacteroidales	Bacteroidetes_vadinHA17	Bacteroidetes_vadinHA17	
denovo4836	0.0031%	0.0000%	0.0000%	0.0010%	0.0018%	Bacteria	Firmicutes	Clostridia	Oscillospirales	Ruminococcaceae	Candidatus_Soleaferrea	
denovo4838	0.0031%	0.0000%	0.0000%	0.0010%	0.0018%	Bacteria	Firmicutes	Bacilli	Bacillales			
denovo485	0.0031%	0.0000%	0.0000%	0.0010%	0.0018%	Archaea	Halobacterota	Methanomicrobia	Methanomicrobiales	Methanospirillaceae	Methanospirillum	

denovo4876	0.0031%	0.0000%	0.0000%	0.0010%	0.0018%	Bacteria	Firmicutes	Clostridia				
denovo491	0.0031%	0.0000%	0.0000%	0.0010%	0.0018%	Archaea	Halobacterota	Methanosarcinia	Methanosarciniales	Methanosarcinaceae		
denovo4945	0.0031%	0.0000%	0.0000%	0.0010%	0.0018%	Bacteria	Firmicutes	Clostridia	Christensenellales	Christensenellaceae	uncultured	
denovo4948	0.0031%	0.0000%	0.0000%	0.0010%	0.0018%	Bacteria	Firmicutes	Clostridia	Christensenellales	Christensenellaceae	Christensenellaceae_R-7_group	
denovo495	0.0031%	0.0000%	0.0000%	0.0010%	0.0018%	Bacteria						
denovo4956	0.0031%	0.0000%	0.0000%	0.0010%	0.0018%	Archaea	Euryarchaeota	Thermococci	Methanofastidiosales	Methanofastidiosaceae	Candidatus_Methanofastidiosum	uncultured_euryarchaeote
denovo4972	0.0031%	0.0000%	0.0000%	0.0010%	0.0018%	Unassigned						
denovo5011	0.0031%	0.0000%	0.0000%	0.0010%	0.0018%	Bacteria	Firmicutes	Clostridia	Christensenellales	Christensenellaceae	Christensenellaceae_R-7_group	uncultured_Caloramator
denovo5014	0.0031%	0.0000%	0.0000%	0.0010%	0.0018%	Bacteria	Chloroflexi	Anaerolineae	ADurb.Bin180	ADurb.Bin180	ADurb.Bin180	uncultured_organism
denovo5025	0.0031%	0.0000%	0.0000%	0.0010%	0.0018%	Archaea	Euryarchaeota	Methanobacteria	Methanobacteriales	Methanobacteriaceae	Methanobacterium	
denovo5036	0.0031%	0.0000%	0.0000%	0.0010%	0.0018%	Bacteria	Firmicutes	Syntrophomonadia	Syntrophomonadales	Syntrophomonadaceae		
denovo5090	0.0031%	0.0000%	0.0000%	0.0010%	0.0018%	Bacteria	Proteobacteria	Gammaproteobacteria	Enterobacterales	Enterobacteriaceae		
denovo5102	0.0031%	0.0000%	0.0000%	0.0010%	0.0018%	Eukaryota						
denovo5208	0.0031%	0.0000%	0.0000%	0.0010%	0.0018%	Bacteria	Firmicutes	Clostridia	Clostridiales	Clostridiaceae	Clostridium_sensu_stricto_13	
denovo5232	0.0031%	0.0000%	0.0000%	0.0010%	0.0018%	Bacteria	Firmicutes	uncultured	uncultured	uncultured	uncultured	
denovo5233	0.0031%	0.0000%	0.0000%	0.0010%	0.0018%	Bacteria	Sumerlaeota	Sumerlaeia	Sumerlaeales	Sumerlaeaceae	Sumerlaea	uncultured_bacterium
denovo5338	0.0031%	0.0000%	0.0000%	0.0010%	0.0018%	Bacteria	Firmicutes	Clostridia	Oscillospirales	Butyricicoccaceae	Butyricicoccus	bacterium_NLAE-zl-C313
denovo5351	0.0031%	0.0000%	0.0000%	0.0010%	0.0018%	Bacteria	Bacteroidota	Bacteroidia	Bacteroidales	Dysgonomonadaceae		
denovo5370	0.0031%	0.0000%	0.0000%	0.0010%	0.0018%	Unassigned						
denovo5382	0.0031%	0.0000%	0.0000%	0.0010%	0.0018%	Bacteria	Bacteroidota	Bacteroidia	Bacteroidales	Bacteroidetes_vadinHA17	Bacteroidetes_vadinHA17	
denovo5396	0.0031%	0.0000%	0.0000%	0.0010%	0.0018%	Bacteria	Firmicutes	Clostridia	Christensenellales	Christensenellaceae		
denovo5426	0.0031%	0.0000%	0.0000%	0.0010%	0.0018%	Bacteria	Bacteroidota	Bacteroidia	Bacteroidales	Bacteroidetes_vadinHA17	Bacteroidetes_vadinHA17	uncultured_bacterium
denovo5462	0.0031%	0.0000%	0.0000%	0.0010%	0.0018%	Bacteria	Bacteroidota	Bacteroidia	Bacteroidales	Tannerellaceae	Macellibacteroides	
denovo5508	0.0031%	0.0000%	0.0000%	0.0010%	0.0018%	Bacteria	Firmicutes	Clostridia	Clostridia	Gracilibacteraceae		
denovo5509	0.0031%	0.0000%	0.0000%	0.0010%	0.0018%	Bacteria	Desulfobacterota	Desulfovibrionia	Desulfovibrionales	Desulfovibrionaceae	Desulfovibrio	
denovo5549	0.0031%	0.0000%	0.0000%	0.0010%	0.0018%	Bacteria	Desulfobacterota	Desulfuromonadia	Geobacterales	Geobacteraceae	Geobacter	
denovo558	0.0031%	0.0000%	0.0000%	0.0010%	0.0018%	Bacteria	Actinobacteriota	Actinobacteria	Micrococcales	Microbacteriaceae	Leucobacter	
denovo5580	0.0031%	0.0000%	0.0000%	0.0010%	0.0018%	Bacteria	Proteobacteria	Gammaproteobacteria				
denovo5592	0.0031%	0.0000%	0.0000%	0.0010%	0.0018%	Bacteria	Firmicutes	Clostridia	Christensenellales	Christensenellaceae	Christensenellaceae	uncultured_bacterium
denovo5620	0.0031%	0.0000%	0.0000%	0.0010%	0.0018%	Bacteria	Firmicutes	Clostridia	Christensenellales	Christensenellaceae	uncultured	uncultured_bacterium
denovo5650	0.0031%	0.0000%	0.0000%	0.0010%	0.0018%	Unassigned						
denovo5653	0.0031%	0.0000%	0.0000%	0.0010%	0.0018%	Archaea	Euryarchaeota	Methanobacteria	Methanobacteriales	Methanobacteriaceae	Methanobacterium	
denovo5658	0.0031%	0.0000%	0.0000%	0.0010%	0.0018%	Bacteria	Thermotogota	Thermotogae	Kosmotogales	Kosmotogaceae	Mesotoga	
denovo5672	0.0031%	0.0000%	0.0000%	0.0010%	0.0018%	Bacteria	Firmicutes	Syntrophomonadia	Syntrophomonadales	Syntrophomonadaceae	Syntrophomonas	
denovo5674	0.0031%	0.0000%	0.0000%	0.0010%	0.0018%	Bacteria	Actinobacteriota	Actinobacteria	Propionibacteriales	Propionibacteriaceae	Propionibacterium	Propionibacterium_freudenreichii
denovo5705	0.0031%	0.0000%	0.0000%	0.0010%	0.0018%	Bacteria	Firmicutes	Desulfotomaculia	Desulfotomaculales	Desulfotomaculales	Pelotomaculum	
denovo5778	0.0031%	0.0000%	0.0000%	0.0010%	0.0018%	Bacteria	Bacteroidota	Bacteroidia	Bacteroidales	Dysgonomonadaceae		
denovo5862	0.0031%	0.0000%	0.0000%	0.0010%	0.0018%	Bacteria	Firmicutes	Clostridia	Christensenellales	Christensenellaceae		
denovo5871	0.0031%	0.0000%	0.0000%	0.0010%	0.0018%	Eukaryota	Basidiomycota	Tremellomycetes	Trichosporonales	Trichosporonaceae		
denovo5924	0.0031%	0.0000%	0.0000%	0.0010%	0.0018%	Bacteria	Firmicutes	Syntrophomonadia	Syntrophomonadales	Syntrophomonadaceae	Syntrophomonas	
denovo5953	0.0031%	0.0000%	0.0000%	0.0010%	0.0018%	Bacteria	Firmicutes	Clostridia	Clostridiales	Clostridiaceae	Clostridium_sensu_stricto_3	
denovo6030	0.0031%	0.0000%	0.0000%	0.0010%	0.0018%	Bacteria	Firmicutes	Bacilli	Lactobacillales	Enterococcaceae	Enterococcus	
denovo6042	0.0031%	0.0000%	0.0000%	0.0010%	0.0018%	Bacteria	Proteobacteria	Gammaproteobacteria	Xanthomonadales	Xanthomonadaceae	Pseudoxanthomonas	
denovo6043	0.0031%	0.0000%	0.0000%	0.0010%	0.0018%	Bacteria	Bacteroidota	Ignavibacteria	Ignavibacteriales	Melioribacteraceae	IheB3-7	
denovo6099	0.0031%	0.0000%	0.0000%	0.0010%	0.0018%	Bacteria	Spirochaetota	Leptospirae	Leptospirales	Leptospiraceae	RBG-16-49-21	uncultured_spirochete
denovo6100	0.0031%	0.0000%	0.0000%	0.0010%	0.0018%	Bacteria	Bacteroidota	Bacteroidia	Bacteroidales			
denovo6101	0.0031%	0.0000%	0.0000%	0.0010%	0.0018%	Unassigned						
denovo6176	0.0031%	0.0000%	0.0000%	0.0010%	0.0018%	Bacteria	Bacteroidota	Bacteroidia	Bacteroidales			
denovo6192	0.0031%	0.0000%	0.0000%	0.0010%	0.0018%	Bacteria	Bacteroidota	Bacteroidia	Bacteroidales	Prolixibacteraceae	Draconibacterium	
denovo6206	0.0031%	0.0000%	0.0000%	0.0010%	0.0018%	Bacteria	Desulfobacterota	Syntrophia	Syntrophales	Syntrophaceae	Syntrophus	
denovo6229	0.0031%	0.0000%	0.0000%	0.0010%	0.0018%	Bacteria	Firmicutes	Clostridia				
denovo6276	0.0031%	0.0000%	0.0000%	0.0010%	0.0018%	Bacteria	Firmicutes	Clostridia	Lachnospirales	Lachnospiraceae		
denovo6289	0.0031%	0.0000%	0.0000%	0.0010%	0.0018%	Bacteria	Sumerlaeota	Sumerlaeia	uncultured	uncultured	uncultured	
denovo6294	0.0031%	0.0000%	0.0000%	0.0010%	0.0018%	Bacteria	Firmicutes	Clostridia				
denovo6358	0.0031%	0.0000%	0.0000%	0.0010%	0.0018%	Bacteria	Chloroflexi	Anaerolineae	Anaerolineales	Anaerolineaceae	uncultured	
denovo6387	0.0031%	0.0000%	0.0000%	0.0010%	0.0018%	Bacteria	Firmicutes	Clostridia	Oscillospirales	Ruminococcaceae	uncultured	
denovo6403	0.0031%	0.0000%	0.0000%	0.0010%	0.0018%	Bacteria	Bacteroidota	Bacteroidia	Bacteroidales	Dysgonomonadaceae		
denovo6422	0.0031%	0.0000%	0.0000%	0.0010%	0.0018%	Bacteria	Bacteroidota	Bacteroidia	Bacteroidales	Bacteroidetes_vadinHA17	Bacteroidetes_vadinHA17	
denovo6489	0.0031%	0.0000%	0.0000%	0.0010%	0.0018%	Bacteria	Firmicutes	Bacilli	Lactobacillales			
denovo6673	0.0031%	0.0000%	0.0000%	0.0010%	0.0018%	Bacteria						

denovo6685	0.0031%	0.0000%	0.0000%	0.0010%	0.0018%	Bacteria	Firmicutes	Clostridia				
denovo6709	0.0031%	0.0000%	0.0000%	0.0010%	0.0018%	Unassigned						
denovo6720	0.0031%	0.0000%	0.0000%	0.0010%	0.0018%	Bacteria	Firmicutes	Clostridia				
denovo6772	0.0031%	0.0000%	0.0000%	0.0010%	0.0018%	Unassigned						
denovo6801	0.0031%	0.0000%	0.0000%	0.0010%	0.0018%	Bacteria	Firmicutes	Clostridia	Oscillospirales	Ruminococcaceae	uncultured	
denovo6857	0.0031%	0.0000%	0.0000%	0.0010%	0.0018%	Archaea	Euryarchaeota	Methanobacteria	Methanobacteriales	Methanobacteriaceae	Methanobacterium	
denovo6858	0.0031%	0.0000%	0.0000%	0.0010%	0.0018%	Bacteria	Acidobacteriota	Acidobacteriae	Acidobacteriae	Acidobacteriae	Paludibaculum	
denovo6874	0.0031%	0.0000%	0.0000%	0.0010%	0.0018%	Bacteria	Bacteroidota	Bacteroidia	Bacteroidales	Bacteroidaceae	Bacteroides	
denovo6927	0.0031%	0.0000%	0.0000%	0.0010%	0.0018%	Bacteria	Firmicutes	Clostridia				
denovo694	0.0031%	0.0000%	0.0000%	0.0010%	0.0018%	Bacteria	Bacteroidota	Bacteroidia	Bacteroidales	Bacteroidetes_vadinHA17	Bacteroidetes_vadinHA17	
denovo6947	0.0031%	0.0000%	0.0000%	0.0010%	0.0018%	Unassigned						
denovo7022	0.0031%	0.0000%	0.0000%	0.0010%	0.0018%	Bacteria	Patescibacteria	ABY1	Candidatus_Falkowbacteria	Candidatus_Falkowbacteria	Candidatus_Falkowbacteria	uncultured_Parcubacteria
denovo7032	0.0031%	0.0000%	0.0000%	0.0010%	0.0018%	Bacteria	Bacteroidota	Bacteroidia	Bacteroidales	Rikenellaceae	DMER64	bacterium_enrichment
denovo7034	0.0031%	0.0000%	0.0000%	0.0010%	0.0018%	Bacteria	Firmicutes	Desulfotomaculia	Desulfotomaculales	Desulfotomaculales		
denovo7089	0.0031%	0.0000%	0.0000%	0.0010%	0.0018%	Unassigned						
denovo709	0.0031%	0.0000%	0.0000%	0.0010%	0.0018%	Bacteria	Bacteroidota	Bacteroidia	Bacteroidales			
denovo71	0.0031%	0.0000%	0.0000%	0.0010%	0.0018%	Bacteria	Synergistota	Synergistia	Synergistales	Synergistaceae	uncultured	bacterium_enrichment
denovo712	0.0031%	0.0000%	0.0000%	0.0010%	0.0018%	Unassigned						
denovo7156	0.0031%	0.0000%	0.0000%	0.0010%	0.0018%	Bacteria	Firmicutes	Clostridia				
denovo7161	0.0031%	0.0000%	0.0000%	0.0010%	0.0018%	Unassigned						
denovo7195	0.0031%	0.0000%	0.0000%	0.0010%	0.0018%	Unassigned						
denovo7200	0.0031%	0.0000%	0.0000%	0.0010%	0.0018%	Bacteria	Firmicutes	Syntrophomonadia	Syntrophomonadales	Syntrophomonadaceae		
denovo7207	0.0031%	0.0000%	0.0000%	0.0010%	0.0018%	Bacteria	Firmicutes	Clostridia	Clostridiales	Clostridiaceae	Clostridium_sensu_stricto_12	Clostridium_magnum
denovo7219	0.0031%	0.0000%	0.0000%	0.0010%	0.0018%	Bacteria	Firmicutes	Desulfotomaculia	Desulfotomaculales	Desulfotomaculales	Pelotomaculum	uncultured_bacterium
denovo7221	0.0031%	0.0000%	0.0000%	0.0010%	0.0018%	Bacteria	Firmicutes	Desulfotomaculia	Desulfotomaculales	Desulfotomaculales	Pelotomaculum	uncultured_bacterium
denovo7227	0.0031%	0.0000%	0.0000%	0.0010%	0.0018%	Bacteria	Desulfobacterota	Syntrophia	Syntrophales	uncultured	uncultured	
denovo7236	0.0031%	0.0000%	0.0000%	0.0010%	0.0018%	Bacteria	Chloroflexi	Anaerolineae	Anaerolineales	Anaerolineaceae	Pelolinea	
denovo7243	0.0031%	0.0000%	0.0000%	0.0010%	0.0018%	Bacteria	Firmicutes	Clostridia				
denovo7262	0.0031%	0.0000%	0.0000%	0.0010%	0.0018%	Bacteria	Bacteroidota	Bacteroidia	Bacteroidales	Bacteroidetes_vadinHA17	Bacteroidetes_vadinHA17	
denovo7269	0.0031%	0.0000%	0.0000%	0.0010%	0.0018%	Bacteria	Bacteroidota	Bacteroidia	Bacteroidales	Bacteroidetes_vadinHA17	Bacteroidetes_vadinHA17	
denovo727	0.0031%	0.0000%	0.0000%	0.0010%	0.0018%	Bacteria	Desulfobacterota					
denovo730	0.0031%	0.0000%	0.0000%	0.0010%	0.0018%	Bacteria	Firmicutes	Clostridia	Clostridiales	Clostridiaceae		
denovo7317	0.0031%	0.0000%	0.0000%	0.0010%	0.0018%	Bacteria	Chloroflexi	Anaerolineae	Anaerolineales	Anaerolineaceae	uncultured	
denovo7340	0.0031%	0.0000%	0.0000%	0.0010%	0.0018%	Bacteria	Firmicutes	Clostridia	Clostridiales	Clostridiaceae	Clostridium_sensu_stricto_1	
denovo7362	0.0031%	0.0000%	0.0000%	0.0010%	0.0018%	Bacteria	Firmicutes	Bacilli	Lactobacillales	Enterococcaceae	Enterococcus	
denovo7452	0.0031%	0.0000%	0.0000%	0.0010%	0.0018%	Bacteria	Bacteroidota	Bacteroidia	Bacteroidales	Dysgonomonadaceae	Proteiniphilum	
denovo7468	0.0031%	0.0000%	0.0000%	0.0010%	0.0018%	Bacteria	Chloroflexi	Anaerolineae	Anaerolineales	Anaerolineaceae		
denovo7471	0.0031%	0.0000%	0.0000%	0.0010%	0.0018%	Bacteria	Chloroflexi	Anaerolineae	SBR1031	SBR1031	SBR1031	
denovo750	0.0031%	0.0000%	0.0000%	0.0010%	0.0018%	Bacteria	Spirochaetota	Spirochaetia	Spirochaetales	Spirochaetaceae	uncultured	
denovo754	0.0031%	0.0000%	0.0000%	0.0010%	0.0018%	Bacteria	Firmicutes	Syntrophomonadia	Syntrophomonadales	Syntrophomonadaceae		
denovo7541	0.0031%	0.0000%	0.0000%	0.0010%	0.0018%	Bacteria	Firmicutes	Desulfotomaculia	Desulfotomaculales	Desulfotomaculales	Pelotomaculum	uncultured_bacterium
denovo7542	0.0031%	0.0000%	0.0000%	0.0010%	0.0018%	Bacteria	Desulfobacterota	Syntrophorhabdia	Syntrophorhabdales	Syntrophorhabdaceae	Syntrophorhabdus	
denovo7551	0.0031%	0.0000%	0.0000%	0.0010%	0.0018%	Bacteria	Firmicutes	Desulfotomaculia	Desulfotomaculales	Desulfotomaculales	Pelotomaculum	uncultured_bacterium
denovo7556	0.0031%	0.0000%	0.0000%	0.0010%	0.0018%	Bacteria	Planctomycetota	Phycisphaerae	MSBL9	KCLunmb-38-53	KCLunmb-38-53	uncultured_bacterium
denovo7557	0.0031%	0.0000%	0.0000%	0.0010%	0.0018%	Bacteria						
denovo7625	0.0031%	0.0000%	0.0000%	0.0010%	0.0018%	Bacteria	Spirochaetota	Leptospirae	Leptospirales	Leptospiraceae	RBG-16-49-21	bacterium_enrichment
denovo7642	0.0031%	0.0000%	0.0000%	0.0010%	0.0018%	Bacteria	Firmicutes	Clostridia				
denovo77	0.0031%	0.0000%	0.0000%	0.0010%	0.0018%	Bacteria	Firmicutes	Clostridia	Lachnospirales	Lachnospiraceae	Anaerostignum	
denovo7773	0.0031%	0.0000%	0.0000%	0.0010%	0.0018%	Bacteria	Firmicutes	Clostridia	Christensenellales	Christensenellaceae	Christensenellaceae_R-7_group	
denovo7788	0.0031%	0.0000%	0.0000%	0.0010%	0.0018%	Bacteria	Firmicutes	Clostridia	Peptococcales	Peptococcaceae	Dehalobacterium	uncultured_bacterium
denovo7792	0.0031%	0.0000%	0.0000%	0.0010%	0.0018%	Bacteria	Firmicutes	Clostridia	Peptococcales	Peptococcaceae	uncultured	metagenome
denovo7804	0.0031%	0.0000%	0.0000%	0.0010%	0.0018%	Bacteria	Bacteroidota	Bacteroidia	Bacteroidales	Dysgonomonadaceae	Proteiniphilum	
denovo7814	0.0031%	0.0000%	0.0000%	0.0010%	0.0018%	Bacteria						
denovo7825	0.0031%	0.0000%	0.0000%	0.0010%	0.0018%	Bacteria	Desulfobacterota	Desulfovibrionia	Desulfovibrionales	Desulfovibrionaceae	Desulfovibrio	
denovo788	0.0031%	0.0000%	0.0000%	0.0010%	0.0018%	Unassigned						
denovo7886	0.0031%	0.0000%	0.0000%	0.0010%	0.0018%	Bacteria	Synergistota	Synergistia	Synergistales	Synergistaceae	Syner-01	uncultured_bacterium
denovo7986	0.0031%	0.0000%	0.0000%	0.0010%	0.0018%	Bacteria	Firmicutes					
denovo8006	0.0031%	0.0000%	0.0000%	0.0010%	0.0018%	Bacteria	Spirochaetota	Spirochaetia	Spirochaetales	Spirochaetaceae	Spirochaeta	
denovo8020	0.0031%	0.0000%	0.0000%	0.0010%	0.0018%	Bacteria	Chloroflexi	Chloroflexia	Thermomicrobiales	JG30-KF-CM45	JG30-KF-CM45	metagenome
denovo8028	0.0031%	0.0000%	0.0000%	0.0010%	0.0018%	Unassigned						

denovo8055	0.0031%	0.0000%	0.0000%	0.0010%	0.0018%	Bacteria	Bacteroidota	Bacteroidia	Flavobacteriales			
denovo8060	0.0031%	0.0000%	0.0000%	0.0010%	0.0018%	Bacteria						
denovo8062	0.0031%	0.0000%	0.0000%	0.0010%	0.0018%	Bacteria	Firmicutes	Clostridia	Oscillospirales	Butyricicoccaceae	UCG-009	uncultured_Ruminococcaceae
denovo8063	0.0031%	0.0000%	0.0000%	0.0010%	0.0018%	Bacteria	Firmicutes	Clostridia	Peptococcales	Peptococcaceae	uncultured	metagenome
denovo8081	0.0031%	0.0000%	0.0000%	0.0010%	0.0018%	Bacteria	Bacteroidota	Bacteroidia	Bacteroidales			
denovo8091	0.0031%	0.0000%	0.0000%	0.0010%	0.0018%	Unassigned						
denovo810	0.0031%	0.0000%	0.0000%	0.0010%	0.0018%	Bacteria	Chloroflexi	Anaerolineae				
denovo8114	0.0031%	0.0000%	0.0000%	0.0010%	0.0018%	Bacteria	Desulfobacterota	Desulfobulbia	Desulfobulbales			
denovo8120	0.0031%	0.0000%	0.0000%	0.0010%	0.0018%	Bacteria	Chloroflexi	Anaerolineae	Anaerolineales	Anaerolineaceae	Flexilinea	
denovo8135	0.0031%	0.0000%	0.0000%	0.0010%	0.0018%	Bacteria	Desulfobacterota	Syntrophobacteria	Syntrophobacterales	Syntrophobacteraceae	Syntrophobacter	
denovo8162	0.0031%	0.0000%	0.0000%	0.0010%	0.0018%	Bacteria	Synergistota	Synergistia	Synergistales	Synergistaceae	Thermovirga	
denovo8187	0.0031%	0.0000%	0.0000%	0.0010%	0.0018%	Bacteria	Bacteroidota	Bacteroidia	Bacteroidales			
denovo820	0.0031%	0.0000%	0.0000%	0.0010%	0.0018%	Bacteria	Firmicutes	Clostridia	Peptostreptococcales-Tissierellales	Anaerovoracaceae		
denovo8229	0.0031%	0.0000%	0.0000%	0.0010%	0.0018%	Bacteria	Bacteroidota	Bacteroidia	Sphingobacteriales	Lentimicrobiaceae	Lentimicrobium	
denovo8234	0.0031%	0.0000%	0.0000%	0.0010%	0.0018%	Archaea	Halobacterota	Methanomicrobia	Methanomicrobiales	Methanospirillaceae	Methanospirillum	uncultured_bacterium
denovo825	0.0031%	0.0000%	0.0000%	0.0010%	0.0018%	Bacteria	Desulfobacterota	Syntrophorhabdia	Syntrophorhabdales	Syntrophorhabdaceae	Syntrophorhabdus	
denovo8314	0.0031%	0.0000%	0.0000%	0.0010%	0.0018%	Bacteria	Firmicutes	Clostridia	Christensenellales	Christensenellaceae	Christensenellaceae_R-7_group	uncultured_soil
denovo8317	0.0031%	0.0000%	0.0000%	0.0010%	0.0018%	Bacteria	Bacteroidota	Bacteroidia	Bacteroidales	Rikenellaceae	DMER64	
denovo8343	0.0031%	0.0000%	0.0000%	0.0010%	0.0018%	Bacteria	Desulfobacterota	Desulfovibrionia	Desulfovibrionales	Desulfovibrionaceae	Desulfovibrio	
denovo8376	0.0031%	0.0000%	0.0000%	0.0010%	0.0018%	Unassigned						
denovo8432	0.0031%	0.0000%	0.0000%	0.0010%	0.0018%	Bacteria	Firmicutes	Clostridia	Oscillospirales	Oscillospiraceae		
denovo8469	0.0031%	0.0000%	0.0000%	0.0010%	0.0018%	Bacteria	Firmicutes	Syntrophomonadia	Syntrophomonadales	Syntrophomonadaceae		
denovo8544	0.0031%	0.0000%	0.0000%	0.0010%	0.0018%	Bacteria	Bacteroidota	Bacteroidia	Bacteroidales	Dysgonomonadaceae	Petrimonas	
denovo856	0.0031%	0.0000%	0.0000%	0.0010%	0.0018%	Bacteria	Actinobacteriota	Actinobacteria	Actinomycetales	Actinomycetaceae	Actinomyces	
denovo8591	0.0031%	0.0000%	0.0000%	0.0010%	0.0018%	Unassigned						
denovo8597	0.0031%	0.0000%	0.0000%	0.0010%	0.0018%	Unassigned						
denovo862	0.0031%	0.0000%	0.0000%	0.0010%	0.0018%	Bacteria						
denovo8626	0.0031%	0.0000%	0.0000%	0.0010%	0.0018%	Bacteria						
denovo8684	0.0031%	0.0000%	0.0000%	0.0010%	0.0018%	Unassigned						
denovo8776	0.0031%	0.0000%	0.0000%	0.0010%	0.0018%	Unassigned						
denovo8815	0.0031%	0.0000%	0.0000%	0.0010%	0.0018%	Bacteria	Bacteroidota	Bacteroidia	Bacteroidales			
denovo8838	0.0031%	0.0000%	0.0000%	0.0010%	0.0018%	Unassigned						
denovo8859	0.0031%	0.0000%	0.0000%	0.0010%	0.0018%	Bacteria	Bacteroidota	Bacteroidia				
denovo8860	0.0031%	0.0000%	0.0000%	0.0010%	0.0018%	Bacteria	Firmicutes	Clostridia	Clostridiales	Clostridiaceae		
denovo8891	0.0031%	0.0000%	0.0000%	0.0010%	0.0018%	Bacteria	Firmicutes	Clostridia	Christensenellales	Christensenellaceae		
denovo8931	0.0031%	0.0000%	0.0000%	0.0010%	0.0018%	Bacteria	Desulfobacterota	Syntrophobacteria	Syntrophobacterales	Syntrophobacteraceae	Syntrophobacter	uncultured_soil
denovo9005	0.0031%	0.0000%	0.0000%	0.0010%	0.0018%	Bacteria	Firmicutes	Clostridia	Christensenellales	Christensenellaceae	Christensenellaceae_R-7_group	
denovo9047	0.0031%	0.0000%	0.0000%	0.0010%	0.0018%	Bacteria	Firmicutes	Clostridia	Christensenellales	Christensenellaceae		
denovo9049	0.0031%	0.0000%	0.0000%	0.0010%	0.0018%	Bacteria	Synergistota	Synergistia	Synergistales	Synergistaceae	EBM-39	
denovo9063	0.0031%	0.0000%	0.0000%	0.0010%	0.0018%	Bacteria	Proteobacteria	Gammaproteobacteria	Xanthomonadales	Xanthomonadaceae	Stenotrophomonas	
denovo923	0.0031%	0.0000%	0.0000%	0.0010%	0.0018%	Bacteria	Firmicutes	Clostridia	Oscillospirales	Ruminococcaceae	uncultured	
denovo924	0.0031%	0.0000%	0.0000%	0.0010%	0.0018%	Archaea	Halobacterota					
denovo927	0.0031%	0.0000%	0.0000%	0.0010%	0.0018%	Bacteria	Bdellovibrionota	Bdellovibrionia	Bdellovibrionales	Bdellovibrionaceae	Bdellovibrio	Bdellovibrio_bacteriovorus
denovo934	0.0031%	0.0000%	0.0000%	0.0010%	0.0018%	Bacteria	Synergistota	Synergistia	Synergistales	Synergistaceae	Aminivibrio	
denovo946	0.0031%	0.0000%	0.0000%	0.0010%	0.0018%	Bacteria	Calditrichota	Calditrichia	Calditrichales	Calditrichaceae	SM23-31	uncultured_bacterium
denovo985	0.0031%	0.0000%	0.0000%	0.0010%	0.0018%	Bacteria	Desulfobacterota	Syntrophobacteria	Syntrophobacterales	Syntrophobacteraceae	Syntrophobacter	
denovo99	0.0031%	0.0000%	0.0000%	0.0010%	0.0018%	Bacteria	Firmicutes	Clostridia	Clostridia_vadinBB60_group	Clostridia_vadinBB60_group	Clostridia_vadinBB60_group	uncultured_prokaryote
denovo1000	0.0000%	0.0030%	0.0000%	0.0010%	0.0017%	Bacteria	Firmicutes	Desulfotomaculia	Desulfotomaculales	Desulfotomaculales	Pelotomaculum	
denovo1023	0.0000%	0.0030%	0.0000%	0.0010%	0.0017%	Bacteria	Desulfobacterota	Desulfovibrionia	Desulfovibrionales	Desulfovibrionaceae	Desulfovibrio	
denovo1112	0.0000%	0.0030%	0.0000%	0.0010%	0.0017%	Bacteria	Firmicutes	Clostridia	Oscillospirales			
denovo1138	0.0000%	0.0030%	0.0000%	0.0010%	0.0017%	Bacteria	Firmicutes	Clostridia	Peptococcales	Peptococcaceae	uncultured	uncultured_Firmicutes
denovo1149	0.0000%	0.0030%	0.0000%	0.0010%	0.0017%	Bacteria	Bacteroidota	Bacteroidia	Bacteroidales	Bacteroidetes_vadinHA17	Bacteroidetes_vadinHA17	
denovo1209	0.0000%	0.0030%	0.0000%	0.0010%	0.0017%	Bacteria	Desulfobacterota	Syntrophobacteria	Syntrophobacterales	Syntrophobacteraceae	Syntrophobacter	uncultured_bacterium
denovo1211	0.0000%	0.0030%	0.0000%	0.0010%	0.0017%	Bacteria	Firmicutes	Clostridia	Oscillospirales	Ruminococcaceae	uncultured	
denovo1214	0.0000%	0.0030%	0.0000%	0.0010%	0.0017%	Bacteria	Proteobacteria	Gammaproteobacteria	Enterobacterales	Enterobacteriaceae		
denovo1267	0.0000%	0.0030%	0.0000%	0.0010%	0.0017%	Bacteria	Firmicutes					
denovo1305	0.0000%	0.0030%	0.0000%	0.0010%	0.0017%	Bacteria	Bacteroidota	Bacteroidia	Bacteroidales	Prolixibacteraceae	Mangrovibacterium	
denovo1307	0.0000%	0.0030%	0.0000%	0.0010%	0.0017%	Bacteria	Firmicutes	Clostridia	Oscillospirales			
denovo1351	0.0000%	0.0030%	0.0000%	0.0010%	0.0017%	Bacteria	Firmicutes	Bacilli	Erysipelotrichales	Erysipelotrichaceae		
denovo1377	0.0000%	0.0030%	0.0000%	0.0010%	0.0017%	Bacteria	Firmicutes	Clostridia				

denovo144	0.0000%	0.0030%	0.0000%	0.0010%	0.0017%	Archaea	Crenarchaeota	Bathyarchaeia	Bathyarchaeia	Bathyarchaeia	Bathyarchaeia	uncultured_methanogenic
denovo1454	0.0000%	0.0030%	0.0000%	0.0010%	0.0017%	Bacteria	Bacteroidota	Bacteroidia	Bacteroidales	Bacteroidetes_vadinHA17	Bacteroidetes_vadinHA17	
denovo1458	0.0000%	0.0030%	0.0000%	0.0010%	0.0017%	Bacteria	Firmicutes	Clostridia	Oscillospirales	Oscillospiraceae		
denovo1508	0.0000%	0.0030%	0.0000%	0.0010%	0.0017%	Bacteria	Firmicutes	Clostridia	Clostridia	Gracilibacteraceae		
denovo1568	0.0000%	0.0030%	0.0000%	0.0010%	0.0017%	Bacteria	Firmicutes	Bacilli	Bacillales			
denovo1618	0.0000%	0.0030%	0.0000%	0.0010%	0.0017%	Bacteria	Firmicutes	Bacilli	Lactobacillales			
denovo1758	0.0000%	0.0030%	0.0000%	0.0010%	0.0017%	Bacteria	Synergistota	Synergistia	Synergistales	Synergistaceae	Thermovirga	uncultured_Synergistetes
denovo1778	0.0000%	0.0030%	0.0000%	0.0010%	0.0017%	Bacteria	Firmicutes	Clostridia	Clostridia	Gracilibacteraceae	uncultured	uncultured_bacterium
denovo1880	0.0000%	0.0030%	0.0000%	0.0010%	0.0017%	Bacteria	Firmicutes	Clostridia	Christensenellales	Christensenellaceae	uncultured	
denovo1883	0.0000%	0.0030%	0.0000%	0.0010%	0.0017%	Bacteria	Desulfobacterota	Desulfovibrionia	Desulfovibrionales	Desulfovibrionaceae	Desulfovibrio	
denovo1907	0.0000%	0.0030%	0.0000%	0.0010%	0.0017%	Bacteria	Desulfobacterota					
denovo1909	0.0000%	0.0030%	0.0000%	0.0010%	0.0017%	Unassigned						
denovo1926	0.0000%	0.0030%	0.0000%	0.0010%	0.0017%	Bacteria	Bacteroidota	Bacteroidia	Bacteroidales	Bacteroidetes_vadinHA17	Bacteroidetes_vadinHA17	
denovo1976	0.0000%	0.0030%	0.0000%	0.0010%	0.0017%	Bacteria	Bacteroidota	Bacteroidia	Bacteroidales	Prolixibacteraceae		
denovo2001	0.0000%	0.0030%	0.0000%	0.0010%	0.0017%	Bacteria	Firmicutes	Clostridia	Oscillospirales	Ruminococcaceae	uncultured	
denovo2038	0.0000%	0.0030%	0.0000%	0.0010%	0.0017%	Unassigned						
denovo2059	0.0000%	0.0030%	0.0000%	0.0010%	0.0017%	Bacteria	Spirochaetota	Spirochaetia	Spirochaetales	Spirochaetaceae	uncultured	uncultured_bacterium
denovo209	0.0000%	0.0030%	0.0000%	0.0010%	0.0017%	Bacteria	Firmicutes	Syntrophomonadia	Syntrophomonadales	Syntrophomonadaceae	Syntrophomonas	
denovo21	0.0000%	0.0030%	0.0000%	0.0010%	0.0017%	Bacteria	Chloroflexi	Anaerolineae	Anaerolineales	Anaerolineaceae		
denovo2120	0.0000%	0.0030%	0.0000%	0.0010%	0.0017%	Bacteria	Chloroflexi	Anaerolineae	RBG-13-54-9	RBG-13-54-9	RBG-13-54-9	
denovo2144	0.0000%	0.0030%	0.0000%	0.0010%	0.0017%	Bacteria	Desulfobacterota	Syntrophobacteria	Syntrophobacterales	Syntrophobacteraceae	Syntrophobacter	
denovo2161	0.0000%	0.0030%	0.0000%	0.0010%	0.0017%	Bacteria	Firmicutes	Clostridia	Peptococcales	Peptococcaceae	uncultured	
denovo2196	0.0000%	0.0030%	0.0000%	0.0010%	0.0017%	Bacteria	Firmicutes	Clostridia				
denovo225	0.0000%	0.0030%	0.0000%	0.0010%	0.0017%	Bacteria	Nitrospirota	Nitrospiria	Nitrospirales	Nitrospiraceae	Nitrospira	
denovo2299	0.0000%	0.0030%	0.0000%	0.0010%	0.0017%	Unassigned						
denovo2305	0.0000%	0.0030%	0.0000%	0.0010%	0.0017%	Unassigned						
denovo2308	0.0000%	0.0030%	0.0000%	0.0010%	0.0017%	Bacteria	Firmicutes	D8A-2	D8A-2	D8A-2	D8A-2	uncultured_bacterium
denovo2368	0.0000%	0.0030%	0.0000%	0.0010%	0.0017%	Bacteria	Firmicutes	Syntrophomonadia	Syntrophomonadales	Syntrophomonadaceae	Syntrophomonas	
denovo2371	0.0000%	0.0030%	0.0000%	0.0010%	0.0017%	Bacteria	Bacteroidota	Bacteroidia	Bacteroidales	Bacteroidetes_vadinHA17	Bacteroidetes_vadinHA17	
denovo2412	0.0000%	0.0030%	0.0000%	0.0010%	0.0017%	Bacteria	Firmicutes	Clostridia	Oscillospirales			
denovo2427	0.0000%	0.0030%	0.0000%	0.0010%	0.0017%	Bacteria	Spirochaetota	Spirochaetia	Spirochaetales	Spirochaetaceae	Treponema	Treponema_caldarium
denovo2437	0.0000%	0.0030%	0.0000%	0.0010%	0.0017%	Unassigned						
denovo2439	0.0000%	0.0030%	0.0000%	0.0010%	0.0017%	Bacteria	Firmicutes	Clostridia	Christensenellales	Christensenellaceae		
denovo2452	0.0000%	0.0030%	0.0000%	0.0010%	0.0017%	Bacteria	Firmicutes	Desulfotomaculia	Desulfotomaculales	Desulfotomaculales	Pelotomaculum	
denovo2460	0.0000%	0.0030%	0.0000%	0.0010%	0.0017%	Bacteria	Bacteroidota	Bacteroidia	Bacteroidales	Bacteroidetes_vadinHA17	Bacteroidetes_vadinHA17	
denovo2489	0.0000%	0.0030%	0.0000%	0.0010%	0.0017%	Bacteria	Proteobacteria	Gammaproteobacteria				
denovo2491	0.0000%	0.0030%	0.0000%	0.0010%	0.0017%	Bacteria	Firmicutes					
denovo251	0.0000%	0.0030%	0.0000%	0.0010%	0.0017%	Bacteria	Firmicutes	Clostridia	Oscillospirales	Ruminococcaceae		
denovo2515	0.0000%	0.0030%	0.0000%	0.0010%	0.0017%	Bacteria	Firmicutes	Clostridia				
denovo2528	0.0000%	0.0030%	0.0000%	0.0010%	0.0017%	Bacteria	Chloroflexi					
denovo2541	0.0000%	0.0030%	0.0000%	0.0010%	0.0017%	Archaea	Halobacterota	Methanosarcinia	Methanosarciniales	Methanosarcinaceae	Methanosarcina	
denovo2543	0.0000%	0.0030%	0.0000%	0.0010%	0.0017%	Bacteria	Firmicutes	Clostridia	Clostridiales	Clostridiaceae	Clostridium_sensu_stricto_1	
denovo2551	0.0000%	0.0030%	0.0000%	0.0010%	0.0017%	Bacteria	Proteobacteria	Alphaproteobacteria	Rickettsiales	Rickettsiales	Candidatus_Jidaibacter	endosymbiont_of
denovo2589	0.0000%	0.0030%	0.0000%	0.0010%	0.0017%	Bacteria	Bacteroidota	Bacteroidia	Bacteroidales	Dysgonomonadaceae	Petrimonas	
denovo2602	0.0000%	0.0030%	0.0000%	0.0010%	0.0017%	Bacteria	Spirochaetota	Spirochaetia	Spirochaetales	Spirochaetaceae	uncultured	
denovo2661	0.0000%	0.0030%	0.0000%	0.0010%	0.0017%	Bacteria	Firmicutes	Syntrophomonadia	Syntrophomonadales	Syntrophomonadaceae	Syntrophomonas	Syntrophomonas_wolfei
denovo2669	0.0000%	0.0030%	0.0000%	0.0010%	0.0017%	Bacteria	Firmicutes	Bacilli	Lactobacillales	Enterococcaceae	Enterococcus	
denovo2692	0.0000%	0.0030%	0.0000%	0.0010%	0.0017%	Eukaryota						
denovo2708	0.0000%	0.0030%	0.0000%	0.0010%	0.0017%	Bacteria	Firmicutes	Clostridia	Clostridia	Gracilibacteraceae		
denovo2769	0.0000%	0.0030%	0.0000%	0.0010%	0.0017%	Bacteria	Firmicutes	Clostridia	Oscillospirales			
denovo2777	0.0000%	0.0030%	0.0000%	0.0010%	0.0017%	Bacteria	Firmicutes	Clostridia	Christensenellales	Christensenellaceae	Christensenellaceae_R-7_group	
denovo2792	0.0000%	0.0030%	0.0000%	0.0010%	0.0017%	Bacteria						
denovo285	0.0000%	0.0030%	0.0000%	0.0010%	0.0017%	Bacteria	Proteobacteria	Gammaproteobacteria	Enterobacterales			
denovo2851	0.0000%	0.0030%	0.0000%	0.0010%	0.0017%	Bacteria	Firmicutes	Clostridia	Oscillospirales	Oscillospiraceae		
denovo286	0.0000%	0.0030%	0.0000%	0.0010%	0.0017%	Bacteria	Chloroflexi	Anaerolineae	Anaerolineales	Anaerolineaceae		
denovo2891	0.0000%	0.0030%	0.0000%	0.0010%	0.0017%	Unassigned						
denovo2936	0.0000%	0.0030%	0.0000%	0.0010%	0.0017%	Bacteria	Firmicutes	Clostridia	Peptostreptococcales-Tissierellales	Peptostreptococcaceae	Intestinibacter	
denovo2939	0.0000%	0.0030%	0.0000%	0.0010%	0.0017%	Bacteria	Firmicutes	Clostridia				
denovo2994	0.0000%	0.0030%	0.0000%	0.0010%	0.0017%	Bacteria						
denovo2999	0.0000%	0.0030%	0.0000%	0.0010%	0.0017%	Bacteria	Firmicutes	Desulfotomaculia	Desulfotomaculales	Desulfotomaculales	Pelotomaculum	uncultured_bacterium

denovo3017	0.0000%	0.0030%	0.0000%	0.0010%	0.0017%	Bacteria	Firmicutes	Clostridia	Oscillospirales	[Eubacterium]_coprostanoligenes_group	[Eubacterium]_coprostanoligenes_group	
denovo3054	0.0000%	0.0030%	0.0000%	0.0010%	0.0017%	Unassigned						
denovo3110	0.0000%	0.0030%	0.0000%	0.0010%	0.0017%	Bacteria	Firmicutes	uncultured	uncultured	uncultured	uncultured	uncultured_Clostridiales
denovo3114	0.0000%	0.0030%	0.0000%	0.0010%	0.0017%	Bacteria	Synergistota	Synergistia	Synergistales	Synergistaceae	Syner-01	
denovo317	0.0000%	0.0030%	0.0000%	0.0010%	0.0017%	Bacteria	Bacteroidota	Bacteroidia	Bacteroidales	Williamwhitmaniaceae	Blvii28_wastewater-sludge_group	uncultured_Rikenellaceae
denovo3171	0.0000%	0.0030%	0.0000%	0.0010%	0.0017%	Bacteria						
denovo3234	0.0000%	0.0030%	0.0000%	0.0010%	0.0017%	Bacteria	Firmicutes	Clostridia	Lachnospirales	Lachnospiraceae		
denovo3239	0.0000%	0.0030%	0.0000%	0.0010%	0.0017%	Bacteria	Firmicutes	Desulfotomaculia	Desulfotomaculales	Desulfotomaculales	Pelotomaculum	uncultured_bacterium
denovo3287	0.0000%	0.0030%	0.0000%	0.0010%	0.0017%	Bacteria	Firmicutes	Bacilli	Erysipelotrichales	Erysipelatoclostridiaceae	UCG-004	Tenericutes_bacterium
denovo3291	0.0000%	0.0030%	0.0000%	0.0010%	0.0017%	Bacteria	Proteobacteria	Gammaproteobacteria	Burkholderiales			
denovo332	0.0000%	0.0030%	0.0000%	0.0010%	0.0017%	Bacteria	Firmicutes	Syntrophomonadia	Syntrophomonadales	Syntrophomonadaceae	Syntrophomonas	
denovo3329	0.0000%	0.0030%	0.0000%	0.0010%	0.0017%	Bacteria	Firmicutes	Clostridia				
denovo3330	0.0000%	0.0030%	0.0000%	0.0010%	0.0017%	Bacteria	Desulfobacterota	Desulfovibrionia	Desulfovibrionales	Desulfovibrionaceae	Desulfovibrio	
denovo3337	0.0000%	0.0030%	0.0000%	0.0010%	0.0017%	Bacteria	Firmicutes	Clostridia	Christensenellales	Christensenellaceae	Christensenellaceae_R-7_group	
denovo3357	0.0000%	0.0030%	0.0000%	0.0010%	0.0017%	Unassigned						
denovo3384	0.0000%	0.0030%	0.0000%	0.0010%	0.0017%	Unassigned						
denovo3385	0.0000%	0.0030%	0.0000%	0.0010%	0.0017%	Bacteria	Firmicutes	Clostridia	Oscillospirales	Oscillospiraceae		
denovo3390	0.0000%	0.0030%	0.0000%	0.0010%	0.0017%	Bacteria	Firmicutes	Clostridia	Peptostreptococcales-Tissierellales			
denovo3444	0.0000%	0.0030%	0.0000%	0.0010%	0.0017%	Unassigned						
denovo3483	0.0000%	0.0030%	0.0000%	0.0010%	0.0017%	Bacteria	Desulfobacterota	Syntrophobacteria	Syntrophobacterales	Syntrophobacteraceae	Syntrophobacter	
denovo3507	0.0000%	0.0030%	0.0000%	0.0010%	0.0017%	Bacteria						
denovo3559	0.0000%	0.0030%	0.0000%	0.0010%	0.0017%	Bacteria	Firmicutes	Clostridia	Christensenellales	Christensenellaceae		
denovo3560	0.0000%	0.0030%	0.0000%	0.0010%	0.0017%	Bacteria	Proteobacteria	Gammaproteobacteria	Enterobacterales	Enterobacteriaceae		
denovo359	0.0000%	0.0030%	0.0000%	0.0010%	0.0017%	Bacteria	Firmicutes	Clostridia	Oscillospirales	Ruminococcaceae	Incertae_Sedis	uncultured_Ruminococcaceae
denovo3635	0.0000%	0.0030%	0.0000%	0.0010%	0.0017%	Bacteria	Bacteroidota	Bacteroidia	Bacteroidales	Bacteroidaceae	Bacteroides	
denovo3662	0.0000%	0.0030%	0.0000%	0.0010%	0.0017%	Bacteria	Firmicutes	Clostridia				
denovo3665	0.0000%	0.0030%	0.0000%	0.0010%	0.0017%	Bacteria	Proteobacteria	Alphaproteobacteria				
denovo3676	0.0000%	0.0030%	0.0000%	0.0010%	0.0017%	Bacteria	Chloroflexi	Anaerolineae	Anaerolineales	Anaerolineaceae	Flexilinea	
denovo3782	0.0000%	0.0030%	0.0000%	0.0010%	0.0017%	Bacteria	Bacteroidota	Bacteroidia	Bacteroidales			
denovo3785	0.0000%	0.0030%	0.0000%	0.0010%	0.0017%	Bacteria	Firmicutes	Clostridia				
denovo3858	0.0000%	0.0030%	0.0000%	0.0010%	0.0017%	Bacteria	Bacteroidota	Bacteroidia	Bacteroidales	Dysgonomonadaceae		
denovo3862	0.0000%	0.0030%	0.0000%	0.0010%	0.0017%	Bacteria	Desulfobacterota	Desulfuromonadia	Geobacterales	Geobacteraceae		
denovo3871	0.0000%	0.0030%	0.0000%	0.0010%	0.0017%	Bacteria	Firmicutes					
denovo3888	0.0000%	0.0030%	0.0000%	0.0010%	0.0017%	Archaea	Euryarchaeota	Methanobacteria	Methanobacteriales	Methanobacteriaceae	Methanobacterium	uncultured_Methanobacteriales
denovo3895	0.0000%	0.0030%	0.0000%	0.0010%	0.0017%	Bacteria						
denovo3941	0.0000%	0.0030%	0.0000%	0.0010%	0.0017%	Bacteria	Firmicutes	Syntrophomonadia	Syntrophomonadales	Syntrophomonadaceae	Syntrophomonas	
denovo3947	0.0000%	0.0030%	0.0000%	0.0010%	0.0017%	Bacteria	Bacteroidota	Kryptonia	Kryptoniales	MSB-3C8	MSB-3C8	metagenome
denovo3970	0.0000%	0.0030%	0.0000%	0.0010%	0.0017%	Bacteria	Chloroflexi	Anaerolineae	Anaerolineales	Anaerolineaceae	Flexilinea	
denovo4051	0.0000%	0.0030%	0.0000%	0.0010%	0.0017%	Bacteria						
denovo4075	0.0000%	0.0030%	0.0000%	0.0010%	0.0017%	Archaea	Halobacterota	Methanosarcinia	Methanosarciniales	Methanosaetaceae	Methanosaeta	
denovo4106	0.0000%	0.0030%	0.0000%	0.0010%	0.0017%	Bacteria	Proteobacteria	Gammaproteobacteria	Enterobacterales	Enterobacteriaceae		
denovo4144	0.0000%	0.0030%	0.0000%	0.0010%	0.0017%	Bacteria	Synergistota	Synergistia	Synergistales	Synergistaceae	uncultured	
denovo4187	0.0000%	0.0030%	0.0000%	0.0010%	0.0017%	Bacteria	Firmicutes	BRH-c20a	BRH-c20a	BRH-c20a	BRH-c20a	uncultured_bacterium
denovo421	0.0000%	0.0030%	0.0000%	0.0010%	0.0017%	Bacteria	Synergistota	Synergistia	Synergistales	Synergistaceae	Syner-01	
denovo426	0.0000%	0.0030%	0.0000%	0.0010%	0.0017%	Bacteria	Spirochaetota	Spirochaetia	Spirochaetales	Spirochaetaceae	uncultured	uncultured_bacterium
denovo4270	0.0000%	0.0030%	0.0000%	0.0010%	0.0017%	Bacteria	Desulfobacterota	Syntrophobacteria	Syntrophobacterales	Syntrophobacteraceae	Syntrophobacter	
denovo4272	0.0000%	0.0030%	0.0000%	0.0010%	0.0017%	Bacteria	Firmicutes	Desulfotomaculia	Desulfotomaculales	Desulfotomaculales		
denovo4273	0.0000%	0.0030%	0.0000%	0.0010%	0.0017%	Unassigned						
denovo433	0.0000%	0.0030%	0.0000%	0.0010%	0.0017%	Bacteria	Firmicutes	Clostridia	Eubacteriales	Eubacteriaceae	Eubacterium	
denovo4361	0.0000%	0.0030%	0.0000%	0.0010%	0.0017%	Bacteria						
denovo4369	0.0000%	0.0030%	0.0000%	0.0010%	0.0017%	Bacteria	Synergistota	Synergistia	Synergistales	Synergistaceae	Thermovirga	
denovo44	0.0000%	0.0030%	0.0000%	0.0010%	0.0017%	Bacteria	Bacteroidota	Bacteroidia	Sphingobacteriales	ST-12K33	ST-12K33	
denovo4405	0.0000%	0.0030%	0.0000%	0.0010%	0.0017%	Archaea	Halobacterota					
denovo441	0.0000%	0.0030%	0.0000%	0.0010%	0.0017%	Bacteria	Firmicutes	Clostridia				
denovo4413	0.0000%	0.0030%	0.0000%	0.0010%	0.0017%	Bacteria	Firmicutes	Clostridia	Christensenellales	Christensenellaceae	uncultured	uncultured_low
denovo4452	0.0000%	0.0030%	0.0000%	0.0010%	0.0017%	Bacteria	Firmicutes	Clostridia				
denovo453	0.0000%	0.0030%	0.0000%	0.0010%	0.0017%	Bacteria	Synergistota	Synergistia	Synergistales	Synergistaceae	uncultured	bacterium_enrichment
denovo4535	0.0000%	0.0030%	0.0000%	0.0010%	0.0017%	Bacteria	Firmicutes					
denovo4594	0.0000%	0.0030%	0.0000%	0.0010%	0.0017%	Bacteria	Desulfobacterota	Desulfobulbia	Desulfobulbales	Desulfobulbaceae		
denovo4608	0.0000%	0.0030%	0.0000%	0.0010%	0.0017%	Bacteria	Firmicutes	Desulfotomaculia	Desulfotomaculales	Desulfotomaculales	Pelotomaculum	uncultured_bacterium

denovo4621	0.0000%	0.0030%	0.0000%	0.0010%	0.0017%	Bacteria	Firmicutes	Clostridia				
denovo4634	0.0000%	0.0030%	0.0000%	0.0010%	0.0017%	Bacteria	Spirochaetota	Spirochaetia	Spirochaetales	Spirochaetaceae	Sphaerochaeta	
denovo4694	0.0000%	0.0030%	0.0000%	0.0010%	0.0017%	Bacteria	Firmicutes	Clostridia	Christensenellales	Christensenellaceae	Christensenella	Christensenella_minuta
denovo4720	0.0000%	0.0030%	0.0000%	0.0010%	0.0017%	Bacteria	Bacteroidota	Bacteroidia	Sphingobacteriales	Lentimicrobiaceae	Lentimicrobium	
denovo4723	0.0000%	0.0030%	0.0000%	0.0010%	0.0017%	Bacteria	Bacteroidota	Bacteroidia	Bacteroidales	Williamwhitmaniaceae	Blvii28_wastewater-sludge_group	uncultured_bacterium
denovo4739	0.0000%	0.0030%	0.0000%	0.0010%	0.0017%	Eukaryota						
denovo475	0.0000%	0.0030%	0.0000%	0.0010%	0.0017%	Unassigned						
denovo477	0.0000%	0.0030%	0.0000%	0.0010%	0.0017%	Bacteria	Bacteroidota	Bacteroidia	Bacteroidales	Bacteroidaceae	Bacteroides	
denovo4810	0.0000%	0.0030%	0.0000%	0.0010%	0.0017%	Bacteria	Desulfobacterota	Desulfovibrionia	Desulfovibrionales	Desulfovibrionaceae	Desulfovibrio	
denovo4837	0.0000%	0.0030%	0.0000%	0.0010%	0.0017%	Bacteria	Desulfobacterota	Syntrophobacteria	Syntrophobacterales	Syntrophobacteraceae	Syntrophobacter	
denovo4841	0.0000%	0.0030%	0.0000%	0.0010%	0.0017%	Unassigned						
denovo4843	0.0000%	0.0030%	0.0000%	0.0010%	0.0017%	Bacteria	Firmicutes	Clostridia	Peptostreptococcales-Tissierellales	Anaerovoracaceae		
denovo4933	0.0000%	0.0030%	0.0000%	0.0010%	0.0017%	Bacteria	Chloroflexi	Anaerolineae	Anaerolineales	Anaerolineaceae		
denovo4962	0.0000%	0.0030%	0.0000%	0.0010%	0.0017%	Unassigned						
denovo4999	0.0000%	0.0030%	0.0000%	0.0010%	0.0017%	Bacteria	Firmicutes	Clostridia				
denovo5005	0.0000%	0.0030%	0.0000%	0.0010%	0.0017%	Bacteria	Proteobacteria	Gammaproteobacteria				
denovo5022	0.0000%	0.0030%	0.0000%	0.0010%	0.0017%	Unassigned						
denovo5029	0.0000%	0.0030%	0.0000%	0.0010%	0.0017%	Bacteria	Chloroflexi	Anaerolineae	Anaerolineales	Anaerolineaceae	Leptolinea	uncultured_bacterium
denovo5042	0.0000%	0.0030%	0.0000%	0.0010%	0.0017%	Bacteria	Desulfobacterota	Syntrophobacteria	Syntrophobacterales	Syntrophobacteraceae	Syntrophobacter	
denovo5058	0.0000%	0.0030%	0.0000%	0.0010%	0.0017%	Bacteria	Bacteroidota	Bacteroidia	Bacteroidales	Williamwhitmaniaceae	Blvii28_wastewater-sludge_group	uncultured_bacterium
denovo5080	0.0000%	0.0030%	0.0000%	0.0010%	0.0017%	Bacteria	Firmicutes	Bacilli	Lactobacillales	Enterococcaceae	Enterococcus	
denovo5111	0.0000%	0.0030%	0.0000%	0.0010%	0.0017%	Bacteria	Firmicutes	Clostridia	Peptococcales	Peptococcaceae	uncultured	uncultured_Peptococcaceae
denovo5147	0.0000%	0.0030%	0.0000%	0.0010%	0.0017%	Bacteria	Firmicutes	Desulfotomaculia	Desulfotomaculales	Desulfotomaculales	Pelotomaculum	
denovo5152	0.0000%	0.0030%	0.0000%	0.0010%	0.0017%	Bacteria	Firmicutes	Clostridia	Clostridia			
denovo5156	0.0000%	0.0030%	0.0000%	0.0010%	0.0017%	Bacteria	Firmicutes	Clostridia	Eubacteriales	Anaerofustaceae	Anaerofustis	
denovo5170	0.0000%	0.0030%	0.0000%	0.0010%	0.0017%	Bacteria	Firmicutes	Bacilli	Lactobacillales	Enterococcaceae		
denovo5197	0.0000%	0.0030%	0.0000%	0.0010%	0.0017%	Bacteria	Desulfobacterota	Syntrophia	Syntrophales	Syntrophaceae	Syntrophus	
denovo5213	0.0000%	0.0030%	0.0000%	0.0010%	0.0017%	Bacteria	Thermotogota	Thermotogae	Kosmotogales	Kosmotogaceae	Mesotoga	
denovo5237	0.0000%	0.0030%	0.0000%	0.0010%	0.0017%	Bacteria	Firmicutes	Syntrophomonadia	Syntrophomonadales	Syntrophomonadaceae		
denovo5247	0.0000%	0.0030%	0.0000%	0.0010%	0.0017%	Bacteria	Bacteroidota	Bacteroidia	Bacteroidales	Williamwhitmaniaceae	Blvii28_wastewater-sludge_group	
denovo5268	0.0000%	0.0030%	0.0000%	0.0010%	0.0017%	Bacteria	Firmicutes	Desulfotomaculia	Desulfotomaculales	Desulfotomaculales	Pelotomaculum	
denovo5270	0.0000%	0.0030%	0.0000%	0.0010%	0.0017%	Bacteria	Firmicutes	Clostridia	Christensenellales	Christensenellaceae	Christensenellaceae_R-7_group	uncultured_Christensenellaceae
denovo5299	0.0000%	0.0030%	0.0000%	0.0010%	0.0017%	Bacteria	Firmicutes	Clostridia				
denovo5375	0.0000%	0.0030%	0.0000%	0.0010%	0.0017%	Bacteria	Firmicutes					
denovo5383	0.0000%	0.0030%	0.0000%	0.0010%	0.0017%	Bacteria	Firmicutes	Clostridia	Oscillospirales	Ruminococcaceae	uncultured	uncultured_bacterium
denovo5397	0.0000%	0.0030%	0.0000%	0.0010%	0.0017%	Unassigned						
denovo5418	0.0000%	0.0030%	0.0000%	0.0010%	0.0017%	Unassigned						
denovo5477	0.0000%	0.0030%	0.0000%	0.0010%	0.0017%	Unassigned						
denovo5551	0.0000%	0.0030%	0.0000%	0.0010%	0.0017%	Bacteria	Bacteroidota	Bacteroidia				
denovo5585	0.0000%	0.0030%	0.0000%	0.0010%	0.0017%	Bacteria	Synergistota	Synergistia	Synergistales	Synergistaceae	Thermovirga	
denovo5637	0.0000%	0.0030%	0.0000%	0.0010%	0.0017%	Bacteria						
denovo5648	0.0000%	0.0030%	0.0000%	0.0010%	0.0017%	Eukaryota						
denovo5680	0.0000%	0.0030%	0.0000%	0.0010%	0.0017%	Bacteria	Firmicutes	Clostridia	Peptococcales	Peptococcaceae	uncultured	uncultured_bacterium
denovo5711	0.0000%	0.0030%	0.0000%	0.0010%	0.0017%	Archaea	Halobacterota	Methanosarcinia	Methanosarciniales	Methanosaetaceae	Methanosaeta	
denovo5774	0.0000%	0.0030%	0.0000%	0.0010%	0.0017%	Bacteria	Bacteroidota	Bacteroidia	Bacteroidales	Bacteroidetes_vadinHA17	Bacteroidetes_vadinHA17	uncultured_Geobacter
denovo580	0.0000%	0.0030%	0.0000%	0.0010%	0.0017%	Bacteria	Bacteroidota	Bacteroidia	Bacteroidales	Bacteroidaceae	Bacteroides	
denovo5820	0.0000%	0.0030%	0.0000%	0.0010%	0.0017%	Bacteria	Firmicutes	Syntrophomonadia	Syntrophomonadales	Syntrophomonadaceae	Syntrophomonas	uncultured_bacterium
denovo5843	0.0000%	0.0030%	0.0000%	0.0010%	0.0017%	Bacteria	Firmicutes	Clostridia	Clostridia	Hungateiclostridiaceae		
denovo588	0.0000%	0.0030%	0.0000%	0.0010%	0.0017%	Bacteria	Chloroflexi	Anaerolineae	Anaerolineales	Anaerolineaceae		
denovo5885	0.0000%	0.0030%	0.0000%	0.0010%	0.0017%	Bacteria	Desulfobacterota	Desulfovibrionia	Desulfovibrionales	Desulfovibrionaceae	Desulfovibrio	
denovo5889	0.0000%	0.0030%	0.0000%	0.0010%	0.0017%	Bacteria	Bacteroidota	Bacteroidia	Bacteroidales	Bacteroidetes_vadinHA17	Bacteroidetes_vadinHA17	
denovo5915	0.0000%	0.0030%	0.0000%	0.0010%	0.0017%	Eukaryota						
denovo5937	0.0000%	0.0030%	0.0000%	0.0010%	0.0017%	Bacteria	Firmicutes	Syntrophomonadia	Syntrophomonadales	Syntrophomonadaceae	Syntrophomonas	
denovo5987	0.0000%	0.0030%	0.0000%	0.0010%	0.0017%	Bacteria	Firmicutes	Clostridia	Oscillospirales	Oscillospiraceae		
denovo6060	0.0000%	0.0030%	0.0000%	0.0010%	0.0017%	Bacteria	Actinobacteriota	Thermoleophilia	Gaiellales	uncultured	uncultured	
denovo6096	0.0000%	0.0030%	0.0000%	0.0010%	0.0017%	Bacteria	Firmicutes	Clostridia				
denovo6127	0.0000%	0.0030%	0.0000%	0.0010%	0.0017%	Bacteria	Firmicutes					
denovo6146	0.0000%	0.0030%	0.0000%	0.0010%	0.0017%	Bacteria	Firmicutes	Clostridia	Christensenellales	Christensenellaceae	Christensenellaceae_R-7_group	
denovo62	0.0000%	0.0030%	0.0000%	0.0010%	0.0017%	Bacteria	Firmicutes	Clostridia	Oscillospirales	UCG-010	UCG-010	uncultured_bacterium
denovo6234	0.0000%	0.0030%	0.0000%	0.0010%	0.0017%	Bacteria	Firmicutes	Clostridia	Oscillospirales	Ruminococcaceae	uncultured	

denovo6245	0.0000%	0.0030%	0.0000%	0.0010%	0.0017%	Bacteria	Firmicutes	Clostridia	Christensenellales	Christensenellaceae	Christensenella	Christensenella_minuta
denovo634	0.0000%	0.0030%	0.0000%	0.0010%	0.0017%	Bacteria	Firmicutes	uncultured	uncultured	uncultured	uncultured	uncultured_bacterium
denovo6345	0.0000%	0.0030%	0.0000%	0.0010%	0.0017%	Bacteria	Firmicutes	Clostridia	Clostridia	Gracilibacteraceae		
denovo6376	0.0000%	0.0030%	0.0000%	0.0010%	0.0017%	Unassigned						
denovo6378	0.0000%	0.0030%	0.0000%	0.0010%	0.0017%	Bacteria	Firmicutes	Desulfotomaculia	Desulfotomaculales	Desulfotomaculales		
denovo638	0.0000%	0.0030%	0.0000%	0.0010%	0.0017%	Bacteria	Firmicutes	Clostridia	Oscillospirales			
denovo6463	0.0000%	0.0030%	0.0000%	0.0010%	0.0017%	Bacteria	Firmicutes	Clostridia	Christensenellales	Christensenellaceae		
denovo6479	0.0000%	0.0030%	0.0000%	0.0010%	0.0017%	Bacteria	Firmicutes	Syntrophomonadia	Syntrophomonadales	Syntrophomonadaceae	Syntrophomonas	
denovo6491	0.0000%	0.0030%	0.0000%	0.0010%	0.0017%	Bacteria	Synergistota	Synergistia	Synergistales	Synergistaceae	uncultured	
denovo6510	0.0000%	0.0030%	0.0000%	0.0010%	0.0017%	Bacteria						
denovo6557	0.0000%	0.0030%	0.0000%	0.0010%	0.0017%	Bacteria	Firmicutes	Clostridia	Eubacteriales	Eubacteriaceae		
denovo6570	0.0000%	0.0030%	0.0000%	0.0010%	0.0017%	Bacteria	Verrucomicrobiota	Verrucomicrobiae	Opitutales	Opitutaceae	Diplosphaera	uncultured_bacterium
denovo659	0.0000%	0.0030%	0.0000%	0.0010%	0.0017%	Bacteria	Firmicutes	Clostridia				
denovo6609	0.0000%	0.0030%	0.0000%	0.0010%	0.0017%	Bacteria	Firmicutes	Clostridia				
denovo6627	0.0000%	0.0030%	0.0000%	0.0010%	0.0017%	Bacteria	Bacteroidota	Bacteroidia	Bacteroidales	Bacteroidaceae	Bacteroides	
denovo6656	0.0000%	0.0030%	0.0000%	0.0010%	0.0017%	Bacteria	Bacteroidota	Bacteroidia	Bacteroidales	Tannerellaceae	Macellibacteroides	
denovo6661	0.0000%	0.0030%	0.0000%	0.0010%	0.0017%	Bacteria	Firmicutes	Syntrophomonadia	Syntrophomonadales	Syntrophomonadaceae	Syntrophomonas	
denovo6706	0.0000%	0.0030%	0.0000%	0.0010%	0.0017%	Bacteria	Bacteroidota	Bacteroidia	Bacteroidales	Williamwhitmaniaceae	Blvii28_wastewater-sludge_group	
denovo6725	0.0000%	0.0030%	0.0000%	0.0010%	0.0017%	Bacteria	Proteobacteria	Gammaproteobacteria	Enterobacterales	Enterobacteriaceae		
denovo6734	0.0000%	0.0030%	0.0000%	0.0010%	0.0017%	Bacteria	Bacteroidota	Bacteroidia	Bacteroidales	Bacteroidetes_vadinHA17	Bacteroidetes_vadinHA17	
denovo6744	0.0000%	0.0030%	0.0000%	0.0010%	0.0017%	Bacteria	Firmicutes	Clostridia	Oscillospirales	Ruminococcaceae		
denovo679	0.0000%	0.0030%	0.0000%	0.0010%	0.0017%	Unassigned						
denovo6838	0.0000%	0.0030%	0.0000%	0.0010%	0.0017%	Bacteria	Synergistota	Synergistia	Synergistales	Synergistaceae		
denovo6872	0.0000%	0.0030%	0.0000%	0.0010%	0.0017%	Bacteria	Bacteroidota	Bacteroidia	Bacteroidales	Bacteroidetes_vadinHA17	Bacteroidetes_vadinHA17	
denovo6879	0.0000%	0.0030%	0.0000%	0.0010%	0.0017%	Bacteria	Firmicutes	Clostridia	Lachnospirales	Lachnospiraceae		
denovo6900	0.0000%	0.0030%	0.0000%	0.0010%	0.0017%	Unassigned						
denovo6932	0.0000%	0.0030%	0.0000%	0.0010%	0.0017%	Bacteria	Verrucomicrobiota	Lentisphaeria	Victivallales	Victivallaceae	Victivallaceae	
denovo7019	0.0000%	0.0030%	0.0000%	0.0010%	0.0017%	Bacteria	Firmicutes	Clostridia	Oscillospirales			
denovo7020	0.0000%	0.0030%	0.0000%	0.0010%	0.0017%	Bacteria	Firmicutes	Bacilli	Lactobacillales	Enterococcaceae	Enterococcus	
denovo7045	0.0000%	0.0030%	0.0000%	0.0010%	0.0017%	Bacteria	Spirochaetota	Spirochaetia	Spirochaetales	Spirochaetaceae		
denovo7048	0.0000%	0.0030%	0.0000%	0.0010%	0.0017%	Eukaryota						
denovo7107	0.0000%	0.0030%	0.0000%	0.0010%	0.0017%	Bacteria	Firmicutes					
denovo7129	0.0000%	0.0030%	0.0000%	0.0010%	0.0017%	Bacteria	Firmicutes	Clostridia	Clostridia	Hungateiclostridiaceae	HN-HF0106	uncultured_bacterium
denovo7130	0.0000%	0.0030%	0.0000%	0.0010%	0.0017%	Bacteria	Firmicutes	Clostridia				
denovo7179	0.0000%	0.0030%	0.0000%	0.0010%	0.0017%	Bacteria	Bacteroidota	Bacteroidia	Bacteroidales	Dysgonomonadaceae	Petrimonas	
denovo729	0.0000%	0.0030%	0.0000%	0.0010%	0.0017%	Bacteria	Firmicutes	Clostridia	Peptostreptococcales-Tissierellales	Anaerovoracaceae	uncultured	
denovo7308	0.0000%	0.0030%	0.0000%	0.0010%	0.0017%	Unassigned						
denovo7356	0.0000%	0.0030%	0.0000%	0.0010%	0.0017%	Bacteria	Firmicutes	Clostridia	Christensenellales	Christensenellaceae	Christensenellaceae_R-7_group	uncultured_Lachnospiraceae
denovo7424	0.0000%	0.0030%	0.0000%	0.0010%	0.0017%	Bacteria	Firmicutes					
denovo7480	0.0000%	0.0030%	0.0000%	0.0010%	0.0017%	Bacteria						
denovo764	0.0000%	0.0030%	0.0000%	0.0010%	0.0017%	Bacteria	Firmicutes	Clostridia				
denovo7761	0.0000%	0.0030%	0.0000%	0.0010%	0.0017%	Bacteria	Chloroflexi	Anaerolineae	Anaerolineales	Anaerolineaceae		
denovo7786	0.0000%	0.0030%	0.0000%	0.0010%	0.0017%	Bacteria	Firmicutes	Clostridia	Clostridia	Gracilibacteraceae	uncultured	uncultured_bacterium
denovo7835	0.0000%	0.0030%	0.0000%	0.0010%	0.0017%	Bacteria	Proteobacteria	Gammaproteobacteria	Enterobacterales	Enterobacteriaceae		
denovo7838	0.0000%	0.0030%	0.0000%	0.0010%	0.0017%	Bacteria	Firmicutes	Clostridia	Oscillospirales	Ruminococcaceae	uncultured	uncultured_Clostridiales
denovo790	0.0000%	0.0030%	0.0000%	0.0010%	0.0017%	Unassigned						
denovo7903	0.0000%	0.0030%	0.0000%	0.0010%	0.0017%	Bacteria	Firmicutes	Clostridia	Christensenellales	Christensenellaceae	Christensenellaceae_R-7_group	
denovo7917	0.0000%	0.0030%	0.0000%	0.0010%	0.0017%	Bacteria	Firmicutes	Clostridia	Oscillospirales	Butyricicoccaceae		
denovo7963	0.0000%	0.0030%	0.0000%	0.0010%	0.0017%	Bacteria	Bacteroidota	Bacteroidia	Bacteroidales	Tannerellaceae	Macellibacteroides	
denovo7972	0.0000%	0.0030%	0.0000%	0.0010%	0.0017%	Bacteria	Bacteroidota	Bacteroidia	Bacteroidales	Tannerellaceae	Macellibacteroides	
denovo7984	0.0000%	0.0030%	0.0000%	0.0010%	0.0017%	Bacteria	Chloroflexi	Anaerolineae	Anaerolineales	Anaerolineaceae	Ornatilinea	uncultured_bacterium
denovo8016	0.0000%	0.0030%	0.0000%	0.0010%	0.0017%	Bacteria	Proteobacteria	Gammaproteobacteria	Burkholderiales	Burkholderiaceae	Burkholderia-Caballeronia-Paraburkholderia	
denovo8031	0.0000%	0.0030%	0.0000%	0.0010%	0.0017%	Bacteria	Firmicutes	Clostridia				
denovo8056	0.0000%	0.0030%	0.0000%	0.0010%	0.0017%	Bacteria	Verrucomicrobiota	Lentisphaeria	Victivallales	vadinBE97	vadinBE97	uncultured_rumen
denovo8077	0.0000%	0.0030%	0.0000%	0.0010%	0.0017%	Bacteria	Firmicutes	Clostridia	Peptococcales	Peptococcaceae	uncultured	uncultured_Peptococcaceae
denovo8089	0.0000%	0.0030%	0.0000%	0.0010%	0.0017%	Bacteria	Firmicutes	Clostridia	Peptococcales	Peptococcaceae	uncultured	
denovo8167	0.0000%	0.0030%	0.0000%	0.0010%	0.0017%	Bacteria	Firmicutes	Clostridia	Eubacteriales	Eubacteriaceae	Acetobacterium	
denovo8219	0.0000%	0.0030%	0.0000%	0.0010%	0.0017%	Archaea	Euryarchaeota	Methanobacteria	Methanobacteriales	Methanobacteriaceae	Methanobacterium	
denovo823	0.0000%	0.0030%	0.0000%	0.0010%	0.0017%	Bacteria	Desulfobacterota	Desulfovibrionia	Desulfovibrionales	Desulfovibrionaceae	Desulfovibrio	
denovo8233	0.0000%	0.0030%	0.0000%	0.0010%	0.0017%	Bacteria	Synergistota	Synergistia	Synergistales	Synergistaceae	uncultured	bacterium_enrichment

denovo8246	0.0000%	0.0030%	0.0000%	0.0010%	0.0017%	Bacteria	Chloroflexi	Anaerolineae	Anaerolineales	Anaerolineaceae	Pelolinea	
denovo8283	0.0000%	0.0030%	0.0000%	0.0010%	0.0017%	Bacteria	Firmicutes	Clostridia	Peptostreptococcales-Tissierellales	Sedimentibacteraceae	Sedimentibacter	Sedimentibacter_sp.
denovo8289	0.0000%	0.0030%	0.0000%	0.0010%	0.0017%	Unassigned						
denovo831	0.0000%	0.0030%	0.0000%	0.0010%	0.0017%	Bacteria	Bacteroidota	Bacteroidia	Bacteroidales	Williamwhitmaniaceae	Blvii28_wastewater-sludge_group	uncultured_bacterium
denovo8398	0.0000%	0.0030%	0.0000%	0.0010%	0.0017%	Unassigned						
denovo8421	0.0000%	0.0030%	0.0000%	0.0010%	0.0017%	Unassigned						
denovo8567	0.0000%	0.0030%	0.0000%	0.0010%	0.0017%	Bacteria	Firmicutes	Clostridia	Clostridia	Gracilibacteraceae		
denovo8582	0.0000%	0.0030%	0.0000%	0.0010%	0.0017%	Bacteria	Bacteroidota	Bacteroidia	Bacteroidales	Tannerellaceae	Parabacteroides	
denovo8613	0.0000%	0.0030%	0.0000%	0.0010%	0.0017%	Archaea	Euryarchaeota	Methanobacteria	Methanobacteriales	Methanobacteriaceae	Methanobacterium	
denovo8652	0.0000%	0.0030%	0.0000%	0.0010%	0.0017%	Bacteria	Verrucomicrobiota	Kiritimatiellae	WCHB1-41	WCHB1-41	WCHB1-41	metagenome
denovo8653	0.0000%	0.0030%	0.0000%	0.0010%	0.0017%	Bacteria	Firmicutes	Negativicutes	Acidaminococcales	Acidaminococcaceae	Phascolarctobacterium	
denovo8749	0.0000%	0.0030%	0.0000%	0.0010%	0.0017%	Archaea	Euryarchaeota	Methanobacteria	Methanobacteriales	Methanobacteriaceae	Methanobacterium	
denovo8777	0.0000%	0.0030%	0.0000%	0.0010%	0.0017%	Archaea	Halobacterota	Methanomicrobia	Methanomicrobiales	Methanospirillaceae	Methanospirillum	
denovo8837	0.0000%	0.0030%	0.0000%	0.0010%	0.0017%	Bacteria	Synergistota	Synergistia	Synergistales	Synergistaceae	uncultured	uncultured_bacterium
denovo8870	0.0000%	0.0030%	0.0000%	0.0010%	0.0017%	Bacteria	Chloroflexi	Anaerolineae	uncultured	uncultured	uncultured	uncultured_bacterium
denovo8893	0.0000%	0.0030%	0.0000%	0.0010%	0.0017%	Bacteria	Proteobacteria	Gammaproteobacteria	Enterobacterales	Enterobacteriaceae		
denovo8929	0.0000%	0.0030%	0.0000%	0.0010%	0.0017%	Unassigned						
denovo8938	0.0000%	0.0030%	0.0000%	0.0010%	0.0017%	Bacteria	Firmicutes	Clostridia	Oscillospirales	Ruminococcaceae	uncultured	
denovo894	0.0000%	0.0030%	0.0000%	0.0010%	0.0017%	Bacteria	Firmicutes	Clostridia	Oscillospirales	Ruminococcaceae		
denovo8965	0.0000%	0.0030%	0.0000%	0.0010%	0.0017%	Bacteria	Bacteroidota	Bacteroidia	Bacteroidales	Bacteroidaceae	Bacteroides	
denovo8966	0.0000%	0.0030%	0.0000%	0.0010%	0.0017%	Bacteria	Firmicutes	Clostridia	Peptococcales	Peptococcaceae	uncultured	
denovo900	0.0000%	0.0030%	0.0000%	0.0010%	0.0017%	Bacteria	Bacteroidota	Bacteroidia	Bacteroidales	Williamwhitmaniaceae	Blvii28_wastewater-sludge_group	
denovo9028	0.0000%	0.0030%	0.0000%	0.0010%	0.0017%	Bacteria	Desulfobacterota	Syntrophobacteria	Syntrophobacterales	Syntrophobacteraceae	Syntrophobacter	
denovo944	0.0000%	0.0030%	0.0000%	0.0010%	0.0017%	Bacteria	Firmicutes	Clostridia	Christensenellales	Christensenellaceae		
denovo960	0.0000%	0.0030%	0.0000%	0.0010%	0.0017%	Bacteria	Firmicutes	Clostridia				
denovo969	0.0000%	0.0030%	0.0000%	0.0010%	0.0017%	Bacteria	Proteobacteria	Gammaproteobacteria	uncultured	uncultured	uncultured	
denovo976	0.0000%	0.0030%	0.0000%	0.0010%	0.0017%	Bacteria	Firmicutes	Bacilli	Lactobacillales	Enterococcaceae	Enterococcus	

Table S5. Metagenomic bins and their RNA expression

Taxonomy	Bin ID	Draft genome size (bp)	Number of contigs	Average contig length (bp)	Maximum contig length (bp)	N50 (bp)	Averaged read coverage	Completeness (%)	Contamination (%)	RNA expression level (RPKM)				Details are shown in Suppelental Table no.:
										1	2	3	Average	
Archaea														
Halobacteriota														
Methanosarcinia														
Methanothrix	bin1	3,280,471	845	3,882	46,564	5,966	345.52	93.14	14.38	1059657.1	1027061.5	1028869.5	1038529.4	S7
Methanosarcina	bin13	4,483,056	687	6,526	36,492	8,723	7.10	99.18	6.48	59072.3	76985.9	62422.8	66160.3	S8
Methanomicrobia														
Methanospirillum	bin29m	3,196,263	171	18,692	127,323	31,149	21.56	98.37	2.34	7946.4	16363.8	35675.8	19995.3	S9
Methanobacteriota														
Methanobacteria														
Methanobacterium	bin3	2,343,722	317	7,393	83,135	11,659	47.93	95.51	3.42	30016.0	31331.5	12850.6	24732.7	S10
Bacteria														
Firmicutes_A														
Clostridia														
Ruminococcaceae	bin28	2,242,081	83	27,013	136,366	47,149	4.06	96.98	2.35	3962.9	8109.1	7168.6	6413.5	S14
Lutisporaceae	bin46	2,824,039	820	3,444	28,775	4,521	2.68	88.47	5.04	199.3	440.1	184.9	274.8	S15
Firmicutes_B														
Desulfotomaculia														
Pelotomaculaceae	bin7	3,034,394	288	10,536	139,820	28,796	18.72	99.19	2.85	7892.0	21411.9	8639.4	12647.8	S13
Syntrophomonadia														
Syntrophomonadaceae	bin23	3,426,709	181	18,932	115,164	30,440	5.88	87.93	6.76	8022.0	5098.9	4431.0	5850.6	S11
Unclassified														
Unclassified	bin36	1,875,869	317	5,918	28,804	6,517	3.10	80.38	4.09	205.6	336.0	863.0	468.2	S12
Synergistota														
Aminobacteriaceae	bin48	2,402,370	416	5,775	40,701	8,928	2.91	96.61	6.78	5132.6	8005.1	4960.0	6032.6	S16
Pyramidobacter	bin67	2,409,090	477	5,051	19,041	5,371	1.83	81.43	4.39	10473.8	11806.1	10429.3	10903.1	S17
Synergistaceae	bin11m	1,793,169	476	3,767	43,604	5,772	30.58	86.13	2.85	130308.8	127255.2	119797.2	125787.1	S18
Thermovirgaceae	bin39	2,119,592	165	12,846	85,261	27,301	3.17	100	3.7	2327.8	2726.1	1723.3	2259.1	S19
Bacteroidota														
Bacteroidia														
Petrimonas	bin12	3,403,984	338	10,071	64,365	16,843	7.14	99.45	5.81	3074.6	2899.5	2574.2	2849.4	S20
UBA5261	bin44	3,257,960	716	4,550	36,809	6,262	3.40	96.19	4.67	591.9	1256.3	877.4	908.5	S21
Mangrovibacterium	bin31m	4,136,457	672	6,155	48,300	9,153	2.50	91.31	6.33	231.8	249.5	212.6	231.3	S22
LD21	bin17	3,010,302	107	28,134	129,470	42,159	6.70	97.62	6.25	942.5	1036.1	900.9	959.8	S23
4484-276	bin11	4,383,655	252	17,396	129,850	31,002	7.61	96.77	1.81	4137.3	4983.3	5727.6	4949.4	S24
Desulfobacterota														
Desulfovibrionia														
Solidesulfovibrio	bin8	3,934,415	84	46,838	217,249	81,336	16.87	100	0.02	51125.8	89780.3	113040.3	84648.8	S25
Syntrophorhabdia														
Syntrophorhabdaceae	bin22	3,889,831	237	16,413	98,783	27,803	4.75	95.38	4.2	165.8	72.0	36.5	91.4	S26
Actinobacteriota														
Propionibacteriaceae	bin9	3,553,477	397	8,951	111,400	17,337	8.30	98.45	4.7	2555.5	3869.2	2510.0	2978.2	S27
Chloroflexota														
Anaerolineaceae	bin27	2,019,215	311	6,493	25,109	7,556	4.51	82.73	6.22	1197.9	1450.6	819.9	1156.1	S28
Spirochaetota														
Treponematales	bin28m	2,801,615	144	19,456	133,476	43,162	7.14	98.85	1.99	1715.8	2565.7	1813.6	2031.7	S29
Thermotogota														
Mesotoga	bin20	2,353,721	70	33,625	288,174	66,038	5.47	98.28	1.72	3954.1	3657.5	2256.8	3289.5	S30

Table S6. Transcript levels of the genes in metagenomic bins.

Bin no.	Gene ID	Length	Read count in sample no.: 1	2	3	RPKM in sample no.: 1	2	3	Description
bin001	SOY3_bin001_00001	195	33	26	28	20.231	13.524	15.253	hypothetical protein
bin001	SOY3_bin001_00002	2934	1467	1149	1547	59.774	39.720	56.009	Type IIS restriction enzyme Eco57I
bin001	SOY3_bin001_00003	384	935	522	680	291.089	137.878	188.108	hypothetical protein
bin001	SOY3_bin001_00004	975	1106	582	735	135.611	60.544	80.078	Caspase domain protein
bin001	SOY3_bin001_00005	234	94	48	78	48.024	20.806	35.409	Transposase, Mutator family
bin001	SOY3_bin001_00006	261	86	39	77	39.392	15.156	31.339	hypothetical protein
bin001	SOY3_bin001_00007	1305	1533	781	2048	140.436	60.701	166.705	putative ABC transporter solute-binding protein YclQ precursor
bin001	SOY3_bin001_00008	471	22	11	7	5.584	2.369	1.579	hypothetical protein
bin001	SOY3_bin001_00009	180	514	509	628	341.378	286.814	370.610	hypothetical protein
bin001	SOY3_bin001_00010	282	4	5	7	1.696	1.798	2.637	hypothetical protein
bin001	SOY3_bin001_00011	993	34	24	30	4.093	2.451	3.209	CRISPR-associated endonuclease Cas1
bin001	SOY3_bin001_00012	267	17	21	23	7.612	7.977	9.151	CRISPR-associated endoribonuclease Cas2
bin001	SOY3_bin001_00013	837	27	40	38	3.856	4.847	4.823	CRISPR-associated protein Cas4/endonuclease Cas1 fusion
bin001	SOY3_bin001_00014	603	334	333	380	66.218	56.012	66.942	hypothetical protein
bin001	SOY3_bin001_00015	231	383	362	470	198.213	158.947	216.130	hypothetical protein
bin001	SOY3_bin001_00016	210	189	251	364	107.594	121.230	184.125	hypothetical protein
bin001	SOY3_bin001_00017	459	95	150	176	24.743	33.146	40.731	MarR family protein
bin001	SOY3_bin001_00018	252	1082	1200	1426	513.301	482.987	601.103	hypothetical protein
bin001	SOY3_bin001_00019	462	339	482	548	87.721	105.818	125.999	hypothetical protein
bin001	SOY3_bin001_00020	1596	1156	958	1055	86.591	60.882	70.218	hypothetical protein
bin001	SOY3_bin001_00021	2469	4719	6413	5819	228.494	263.448	250.355	Isoleucine--tRNA ligase
bin001	SOY3_bin001_00022	477	1131	1702	1450	283.459	361.906	322.908	hypothetical protein
bin001	SOY3_bin001_00023	228	441	498	474	231.233	221.539	220.838	hypothetical protein
bin001	SOY3_bin001_00024	867	1540	1972	1898	212.347	230.697	232.545	hypothetical protein
bin001	SOY3_bin001_00025	633	119	148	141	22.474	23.714	23.662	tRNA 2'-O-methylase
bin001	SOY3_bin001_00026	696	1997	1967	1730	343.016	286.649	264.038	Cobalt transport protein CbiM precursor
bin001	SOY3_bin001_00027	1242	1079	1348	1248	103.859	110.084	106.739	Exodeoxyribonuclease 7 large subunit
bin001	SOY3_bin001_00028	210	93	97	88	52.943	46.850	44.514	Exodeoxyribonuclease 7 small subunit
bin001	SOY3_bin001_00029	627	468	842	1062	89.233	136.207	179.923	hypothetical protein
bin001	SOY3_bin001_00030	642	126	549	868	23.463	86.735	143.620	N-(5'-phosphoribosyl)anthranilate isomerase
bin001	SOY3_bin001_00031	1455	458	1552	2087	37.631	108.189	152.366	Anthranilate synthase component 1
bin001	SOY3_bin001_00032	582	140	456	630	28.757	79.469	114.987	Aminodeoxychorismate/anthranilate synthase component 2
bin001	SOY3_bin001_00033	732	256	401	557	41.809	55.563	80.830	Indole-3-glycerol phosphate synthase
bin001	SOY3_bin001_00034	216	1122	1564	1349	620.990	734.409	663.419	hypothetical protein
bin001	SOY3_bin001_00035	975	1893	2643	2347	232.109	274.946	255.704	tRNA N6-adenosine threonylcarbamoyltransferase
bin001	SOY3_bin001_00036	1428	2265	3557	3201	189.621	252.645	238.115	Phosphoribosylamine--glycine ligase
bin001	SOY3_bin001_00037	1326	3484	5054	4606	314.109	386.586	368.986	Glutamine synthetase
bin001	SOY3_bin001_00038	75	92	87	118	146.647	117.656	167.129	tRNA-Lys(ctt)
bin001	SOY3_bin001_00039	309	1550	1610	1461	599.679	528.472	502.252	hypothetical protein
bin001	SOY3_bin001_00040	285	1301	1802	1740	545.730	641.305	648.536	hypothetical protein
bin001	SOY3_bin001_00041	411	2305	3081	2936	670.463	760.334	758.829	hypothetical protein
bin001	SOY3_bin001_00042	1503	2112	3004	2835	167.989	202.720	200.366	Ribosomal protein S12 methylthiotransferase RimO
bin001	SOY3_bin001_00043	303	1969	1754	1897	776.870	587.140	665.050	hypothetical protein
bin001	SOY3_bin001_00044	1140	463	692	611	48.554	61.568	56.933	Aminodeoxyfutalosine synthase
bin001	SOY3_bin001_00045	1785	2104	2688	2528	140.914	152.738	150.442	Pyruvate synthase subunit PorD
bin001	SOY3_bin001_00046	588	654	866	743	132.968	149.381	134.227	Pyruvate synthase subunit PorC
bin001	SOY3_bin001_00047	630	3882	4792	3958	736.649	771.491	667.368	HEAT repeat protein
bin001	SOY3_bin001_00048	237	1335	1176	1051	673.408	503.285	471.069	hypothetical protein
bin001	SOY3_bin001_00049	441	55	40	38	14.910	9.200	9.153	hypothetical protein
bin001	SOY3_bin001_00050	606	1934	1057	1005	381.531	176.912	176.166	Phthiotriol/phenolphthiotriol dimycocerosates methyltransferase
bin001	SOY3_bin001_00051	558	12433	10936	10459	2663.712	1987.829	1991.067	30S ribosomal protein S7
bin001	SOY3_bin001_00052	429	8398	7550	6931	2340.260	1785.025	1716.202	30S ribosomal protein S12
bin001	SOY3_bin001_00053	432	4112	3499	3044	1137.928	821.514	748.498	hypothetical protein
bin001	SOY3_bin001_00054	309	2477	1976	1814	958.326	648.609	623.604	50S ribosomal protein L30e
bin001	SOY3_bin001_00055	1140	11360	9165	8547	1191.294	815.422	796.414	DNA-directed RNA polymerase subunit beta'
bin001	SOY3_bin001_00056	2628	19384	15332	14085	881.787	591.736	569.326	DNA-directed RNA polymerase subunit beta'
bin001	SOY3_bin001_00057	1830	12323	9349	8687	805.028	518.166	504.253	DNA-directed RNA polymerase subunit beta
bin001	SOY3_bin001_00058	1491	8264	6509	6066	662.610	442.783	432.170	DNA-directed RNA polymerase subunit beta
bin001	SOY3_bin001_00059	237	1254	1000	862	632.550	427.963	386.357	DNA-directed RNA polymerase subunit H
bin001	SOY3_bin001_00060	1125	7933	6815	6827	843.006	614.424	644.625	D-malate dehydrogenase [decarboxylating]
bin001	SOY3_bin001_00061	495	4172	3715	3452	1007.592	761.217	740.791	2,3-dimethylmalate dehydratase small subunit
bin001	SOY3_bin001_00062	1146	3159	3018	2844	329.542	267.110	263.618	carbamoyl phosphate synthase-like protein
bin001	SOY3_bin001_00063	441	1027	968	934	278.405	222.634	224.977	hypothetical protein
bin001	SOY3_bin001_00064	579	299	235	225	61.736	41.167	41.279	hypothetical protein

bin001	SOY3_bin001_00065	87	49	58	47	67.332	67.618	57.386	tRNA-Ser(cag)
bin001	SOY3_bin001_00066	1671	4421	5747	5348	316.293	348.835	339.973	Succinyl-CoA ligase [ADP-forming] subunit alpha
bin001	SOY3_bin001_00067	435	330	681	542	90.692	158.786	132.355	Nitric oxide reductase
bin001	SOY3_bin001_00068	816	774	714	732	113.396	88.749	95.291	tRNA pseudouridine synthase A
bin001	SOY3_bin001_00069	1269	2720	3511	3274	256.243	280.623	274.061	2,3-dimethylmalate dehydratase large subunit
bin001	SOY3_bin001_00070	237	209	239	225	105.425	102.283	100.847	Uracil DNA glycosylase superfamily protein
bin001	SOY3_bin001_00071	714	16095	17716	16824	2694.873	2516.646	2503.000	50S ribosomal protein L2
bin001	SOY3_bin001_00072	249	6205	6593	6594	2979.120	2685.582	2813.063	50S ribosomal protein L23
bin001	SOY3_bin001_00073	774	18038	18437	17393	2786.076	2416.040	2387.060	50S ribosomal protein L4P
bin001	SOY3_bin001_00074	1005	20164	20637	20032	2398.591	2082.741	2117.328	50S ribosomal protein L3
bin001	SOY3_bin001_00075	840	21754	22447	21945	3096.031	2710.402	2775.148	SpoU rRNA Methylase family protein
bin001	SOY3_bin001_00076	621	4645	3463	3244	894.210	565.608	554.906	Proteasome subunit beta precursor
bin001	SOY3_bin001_00077	1908	7936	5405	5048	497.243	287.324	281.042	Ribonuclease
bin001	SOY3_bin001_00078	519	2654	2533	2282	611.335	495.020	467.066	ArsR transcriptional regulator
bin001	SOY3_bin001_00079	789	2962	2503	2078	448.801	321.765	279.768	manganese transport regulator MntR
bin001	SOY3_bin001_00080	2442	3232	2322	2225	158.224	96.443	96.786	ATP-dependent DNA helicase RecQ
bin001	SOY3_bin001_00081	1188	640	1006	825	64.403	85.889	73.768	DNA primase small subunit
bin001	SOY3_bin001_00082	567	4486	4336	4062	945.849	775.641	761.004	hypothetical protein
bin001	SOY3_bin001_00083	276	6058	5955	5456	2624.011	2188.404	2099.883	50S ribosomal protein L44e
bin001	SOY3_bin001_00084	168	3068	2798	2455	2183.190	1689.247	1552.287	30S ribosomal protein S27e
bin001	SOY3_bin001_00085	774	11790	10494	9363	1821.035	1375.165	1285.002	General stress protein 13
bin001	SOY3_bin001_00086	162	1956	1690	1467	1443.442	1058.099	961.933	H/ACA RNA-protein complex component Nop10p
bin001	SOY3_bin001_00087	771	7421	6144	5523	1150.677	808.261	760.940	PAC2 family protein
bin001	SOY3_bin001_00088	393	1763	2215	2158	536.297	571.658	583.296	Magnesium transporter MgtE
bin001	SOY3_bin001_00089	387	1299	1509	1397	401.276	395.488	383.456	Ferredoxin
bin001	SOY3_bin001_00090	3960	2255	3430	2641	68.076	87.852	70.844	Response regulator aspartate phosphatase I
bin001	SOY3_bin001_00091	909	580	852	496	76.280	95.067	57.963	putative ABC transporter ATP-binding protein YbhF
bin001	SOY3_bin001_00092	1107	1435	2407	1229	154.971	220.538	117.933	NPCBM-associated, NEW3 domain of alpha-galactosidase
bin001	SOY3_bin001_00093	612	429	578	356	83.801	95.792	61.791	Ergosterol biosynthesis ERG4/ERG24 family protein
bin001	SOY3_bin001_00094	711	371	508	519	62.381	72.468	77.540	hypothetical protein
bin001	SOY3_bin001_00095	303	352	359	395	138.882	120.173	138.479	Stress responsive A/B Barrel Domain protein
bin001	SOY3_bin001_00096	2085	1469	1598	1458	84.229	77.737	74.282	Peptidase family U32
bin001	SOY3_bin001_00097	1737	1332	1947	1687	91.675	113.690	103.168	Adenine deaminase
bin001	SOY3_bin001_00098	2214	5935	9131	7630	320.471	418.307	366.081	ATP-dependent zinc metalloprotease FtsH
bin001	SOY3_bin001_00099	255	292	385	308	136.895	153.135	128.304	PRC-barrel domain protein
bin001	SOY3_bin001_00100	540	1966	2466	2206	435.246	463.185	433.952	putative peptidyl-prolyl cis-trans isomerase
bin001	SOY3_bin001_00101	480	1751	2187	1854	436.104	462.128	410.297	putative peptidyl-prolyl cis-trans isomerase
bin001	SOY3_bin001_00102	429	429	417	373	119.549	98.590	92.359	hypothetical protein
bin001	SOY3_bin001_00103	318	480	421	396	180.451	134.280	132.281	hypothetical protein
bin001	SOY3_bin001_00104	645	1711	1715	1544	317.129	269.686	254.283	Putative transposase DNA-binding domain protein
bin001	SOY3_bin001_00105	867	400	535	319	55.155	62.588	39.084	Inner membrane transport permease YbhR
bin001	SOY3_bin001_00106	834	1004	919	873	143.917	111.765	111.193	putative inorganic polyphosphate/ATP-NAD kinase
bin001	SOY3_bin001_00107	819	1519	1274	1270	221.727	157.776	164.721	Inositol-1-monophosphatase
bin001	SOY3_bin001_00108	1188	5935	4352	4513	597.241	371.559	403.533	Putative transposase DNA-binding domain protein
bin001	SOY3_bin001_00109	1569	2295	2393	2448	174.866	154.694	165.737	Sodium/pantothenate symporter
bin001	SOY3_bin001_00110	74	31	30	41	50.081	41.119	58.855	tRNA-Val(tac)
bin001	SOY3_bin001_00111	1245	382	383	410	36.681	31.202	34.982	MgtC family protein
bin001	SOY3_bin001_00112	675	937	1014	1011	165.952	152.366	159.103	Ultraviolet N-glycosylase/AP lyase
bin001	SOY3_bin001_00113	75	45	52	49	71.729	70.323	69.401	tRNA-Glu(ctc)
bin001	SOY3_bin001_00114	315	1228	1368	1219	466.051	440.484	411.077	hypothetical protein
bin001	SOY3_bin001_00115	360	1113	1002	824	369.605	282.306	243.139	hypothetical protein
bin001	SOY3_bin001_00116	1074	1316	1138	981	146.486	107.471	97.027	homoserine kinase
bin001	SOY3_bin001_00117	420	188	202	181	53.512	48.782	45.778	Ferredoxin-2
bin001	SOY3_bin001_00118	438	166	163	170	45.308	37.746	41.229	Iron-sulfur protein
bin001	SOY3_bin001_00119	819	279	282	261	40.725	34.924	33.852	Glutamate synthase [NADPH] large chain
bin001	SOY3_bin001_00120	1515	588	631	636	46.399	42.245	44.594	Glutamate synthase [NADPH] large chain precursor
bin001	SOY3_bin001_00121	1119	427	399	405	45.619	36.166	38.446	Ferredoxin-dependent glutamate synthase 1
bin001	SOY3_bin001_00122	1182	402	429	432	40.659	36.812	38.824	Glutamine synthetase
bin001	SOY3_bin001_00123	537	1280	1484	1465	284.958	280.294	289.797	hypothetical protein
bin001	SOY3_bin001_00124	285	639	799	731	268.041	284.352	272.460	hypothetical protein
bin001	SOY3_bin001_00125	1836	5005	6320	6100	325.894	349.140	352.929	DNA topoisomerase VI subunit B
bin001	SOY3_bin001_00126	231	3113	3845	3544	1611.064	1688.259	1629.715	leucine-responsive transcriptional regulator
bin001	SOY3_bin001_00127	891	3844	3848	3001	515.764	438.038	357.782	hypothetical protein
bin001	SOY3_bin001_00128	1230	7545	7393	6234	733.330	609.636	538.383	replication factor A
bin001	SOY3_bin001_00129	261	3787	2366	2396	1734.604	919.452	975.161	hypothetical protein
bin001	SOY3_bin001_00130	414	158	264	217	45.625	64.678	55.679	hypothetical protein
bin001	SOY3_bin001_00131	1215	459	506	523	45.163	42.240	45.725	Metal binding domain of Ada

bin001	SOY3_bin001_00132	573	869	1134	1022	181.305	200.730	189.464	putative cobalt-precorrin-6Y C(5)-methyltransferase
bin001	SOY3_bin001_00133	1026	1756	2508	2260	204.608	247.933	233.987	cobalt-precorrin-6A synthase
bin001	SOY3_bin001_00134	633	1110	1475	1355	209.635	236.343	227.387	Precorrin-8X methylmutase
bin001	SOY3_bin001_00135	1431	3142	4168	3960	262.490	295.422	293.958	Cobalt-precorrin-3B C(17)-methyltransferase
bin001	SOY3_bin001_00136	768	2076	2623	2512	323.156	346.411	347.447	cobalamin biosynthesis protein CbiG
bin001	SOY3_bin001_00137	738	165331	169030	171570	26782.028	23230.694	24695.329	hypothetical protein
bin001	SOY3_bin001_00138	738	139296	135199	136133	22564.609	18581.119	19594.621	hypothetical protein
bin001	SOY3_bin001_00139	783	7293	8870	7442	1113.499	1148.991	1009.619	hypothetical protein
bin001	SOY3_bin001_00140	777	7123	10344	8989	1095.942	1350.275	1228.910	hypothetical protein
bin001	SOY3_bin001_00141	1182	6346	7532	6890	641.842	646.320	619.201	hypothetical protein
bin001	SOY3_bin001_00142	711	6984	7528	6613	1174.303	1073.902	988.004	hypothetical protein
bin001	SOY3_bin001_00143	2064	2374	3022	2972	137.504	148.504	152.957	Oligosaccharyl transferase STT3 subunit
bin001	SOY3_bin001_00144	5310	16672	17630	15081	375.352	336.754	301.693	Serine-aspartate repeat-containing protein D precursor
bin001	SOY3_bin001_00145	393	617	679	646	187.689	175.239	174.610	hypothetical protein
bin001	SOY3_bin001_00146	1209	762	762	693	75.348	63.927	60.889	Inner membrane transport protein YajR
bin001	SOY3_bin001_00147	234	474	634	581	242.163	274.807	263.749	AsnC family protein
bin001	SOY3_bin001_00148	432	586	930	843	162.166	218.350	207.288	Lipoprotein NlpI precursor
bin001	SOY3_bin001_00149	345	491	693	655	170.141	203.737	201.675	hypothetical protein
bin001	SOY3_bin001_00150	1188	1317	1375	1266	132.530	117.393	113.200	Carbamoyl-phosphate synthase small chain
bin001	SOY3_bin001_00151	3243	2911	3142	2834	107.310	98.268	92.829	Carbamoyl-phosphate synthase large chain
bin001	SOY3_bin001_00152	76	95	126	96	149.436	168.156	134.180	tRNA-Val(gac)
bin001	SOY3_bin001_00153	73	3	5	9	4.913	6.947	13.096	tRNA-Phe(gaa)
bin001	SOY3_bin001_00154	723	425	483	474	70.274	67.758	69.642	TPR repeat-containing protein YrrB
bin001	SOY3_bin001_00155	513	327	379	342	76.204	74.934	70.817	Nucleoside-triphosphatase THEP1
bin001	SOY3_bin001_00156	441	181	211	191	49.067	48.529	46.007	hypothetical protein
bin001	SOY3_bin001_00157	330	140	202	175	50.718	62.086	56.332	hypothetical protein
bin001	SOY3_bin001_00158	285	572	670	622	239.937	238.443	231.833	Plasmid stabilisation system protein
bin001	SOY3_bin001_00159	1227	1409	1686	1424	137.281	139.370	123.281	hypothetical protein
bin001	SOY3_bin001_00160	459	2726	3976	3197	710.001	878.594	739.877	Acid shock protein
bin001	SOY3_bin001_00161	333	244	231	224	87.597	70.359	71.455	D-hydantoinase
bin001	SOY3_bin001_00162	906	741	672	621	97.777	75.231	72.810	Formate dehydrogenase H
bin001	SOY3_bin001_00163	303	399	468	390	157.426	156.660	136.726	Alkyl hydroperoxide reductase AhpD
bin001	SOY3_bin001_00164	396	84	189	139	25.359	48.408	37.286	hypothetical protein
bin001	SOY3_bin001_00165	1026	163	957	1784	18.993	94.606	184.704	Anthranilate phosphoribosyltransferase
bin001	SOY3_bin001_00166	882	767	1141	1036	103.961	131.211	124.773	Molybdate-binding periplasmic protein precursor
bin001	SOY3_bin001_00167	762	639	796	738	100.252	105.953	102.880	hypothetical protein
bin001	SOY3_bin001_00168	279	333	565	378	142.687	205.399	143.919	hypothetical protein
bin001	SOY3_bin001_00169	471	568	635	629	144.169	136.744	141.860	Acetyltransferase YpeA
bin001	SOY3_bin001_00170	417	1096	1594	1511	314.210	387.710	384.909	hypothetical protein
bin001	SOY3_bin001_00171	1176	1585	2393	2211	161.127	206.391	199.715	Argininosuccinate synthase
bin001	SOY3_bin001_00172	447	684	940	818	182.934	213.292	194.391	hypothetical protein
bin001	SOY3_bin001_00173	768	2638	3176	2990	410.638	419.444	413.561	hypothetical protein
bin001	SOY3_bin001_00174	957	1109	1060	963	138.537	112.344	106.892	hypothetical protein
bin001	SOY3_bin001_00175	273	924	1076	1004	404.627	399.765	390.662	hypothetical protein
bin001	SOY3_bin001_00176	516	238	255	226	55.141	50.124	46.525	Eukaryotic cytochrome b561
bin001	SOY3_bin001_00177	453	595	515	503	157.023	115.309	117.950	NifU-like protein
bin001	SOY3_bin001_00178	876	3558	3555	3138	485.565	411.614	380.521	Acetylglutamate kinase
bin001	SOY3_bin001_00179	570	3065	2861	2629	642.837	509.094	489.943	2-oxoglutarate carboxylase large subunit
bin001	SOY3_bin001_00180	1497	12711	16742	16189	1015.087	1134.332	1148.757	NADH-quinone oxidoreductase subunit N
bin001	SOY3_bin001_00181	1503	10825	13569	13254	861.022	915.680	936.737	NAD(P)H-quinone oxidoreductase chain 4 1
bin001	SOY3_bin001_00182	2040	10629	13247	12401	622.885	658.631	645.738	NADH-quinone oxidoreductase subunit L
bin001	SOY3_bin001_00183	303	2031	2379	2284	801.333	796.355	800.725	NADH-quinone oxidoreductase subunit K
bin001	SOY3_bin001_00184	264	1723	1838	1875	780.238	706.149	754.444	NADH:ubiquinone oxidoreductase subunit J
bin001	SOY3_bin001_00185	432	2845	3079	3033	787.307	722.904	745.793	NADH-quinone oxidoreductase subunit 10
bin001	SOY3_bin001_00186	582	5428	6176	5859	1114.968	1076.314	1069.376	NAD(P)H-quinone oxidoreductase subunit I
bin001	SOY3_bin001_00187	1053	8043	9194	8541	913.135	885.586	861.609	NADH-quinone oxidoreductase subunit H
bin001	SOY3_bin001_00188	1713	17856	20671	20035	1246.156	1223.936	1242.401	NADH-quinone oxidoreductase subunit 4
bin001	SOY3_bin001_00189	573	6348	7398	7284	1324.426	1309.527	1350.346	NADH-quinone oxidoreductase subunit B
bin001	SOY3_bin001_00190	405	3760	4276	4528	1109.886	1070.872	1187.630	NAD(P)H-quinone oxidoreductase subunit 3
bin001	SOY3_bin001_00191	945	342	307	312	43.265	32.950	35.071	putative deoxyhypusine synthase
bin001	SOY3_bin001_00192	741	342	421	387	55.176	57.626	55.478	putative nucleotidyltransferase
bin001	SOY3_bin001_00193	558	371	385	370	79.485	69.981	70.436	Phenolic acid decarboxylase subunit B
bin001	SOY3_bin001_00194	219	1059	1593	1521	578.093	737.779	737.760	ClpX C4-type zinc finger
bin001	SOY3_bin001_00195	2046	4328	4878	4862	252.887	241.819	252.429	Phosphoserine phosphatase RsbU
bin001	SOY3_bin001_00196	183	394	426	464	257.389	236.109	269.337	hypothetical protein
bin001	SOY3_bin001_00197	261	75	97	64	34.353	37.695	26.048	hypothetical protein
bin001	SOY3_bin001_00198	696	475	596	440	81.589	86.854	67.154	2,5-diamino-6-ribosylamino-4(3H)-pyrimidinone 5'-phosphate reductase

bin001	SOY3_bin001_00199	1269	215	271	247	20.255	21.660	20.676	L-2,4-diaminobutyrate decarboxylase
bin001	SOY3_bin001_00200	489	29	34	27	7.090	7.052	5.865	Hemin import ATP-binding protein HmuV
bin001	SOY3_bin001_00201	2061	234	265	259	13.573	13.041	13.349	Magnesium-chelatase 38 kDa subunit
bin001	SOY3_bin001_00202	912	123	91	104	16.123	10.120	12.113	Daunorubicin/doxorubicin resistance ATP-binding protein DrrA
bin001	SOY3_bin001_00203	2370	6083	7214	6637	306.842	308.733	297.477	Valine--tRNA ligase
bin001	SOY3_bin001_00204	528	1246	1358	1299	282.117	260.868	261.339	CYTH domain protein
bin001	SOY3_bin001_00205	1185	2011	2162	1930	202.880	185.051	173.009	hypothetical protein
bin001	SOY3_bin001_00206	924	3305	3811	3868	427.607	418.333	444.677	tetrahydromethanopterin S-methyltransferase subunit H
bin001	SOY3_bin001_00207	621	2508	3448	3331	482.816	563.158	569.787	tetrahydromethanopterin S-methyltransferase subunit A
bin001	SOY3_bin001_00208	375	324	457	407	103.290	123.606	115.290	FaeA-like protein
bin001	SOY3_bin001_00209	1371	1515	1825	1718	132.105	135.014	133.111	DNA repair protein RadA
bin001	SOY3_bin001_00210	1986	59247	111641	103783	3566.421	5701.633	5551.071	Acetyl-coenzyme A synthetase
bin001	SOY3_bin001_00211	231	265	312	356	137.145	136.993	163.707	Ribbon-helix-helix protein, copG family
bin001	SOY3_bin001_00212	213	118	132	167	66.229	62.856	83.285	Formate dehydrogenase, nitrate-inducible, iron-sulfur subunit
bin001	SOY3_bin001_00213	1707	984	1229	1170	68.914	73.025	72.809	hypothetical protein
bin001	SOY3_bin001_00214	945	653	621	625	82.609	66.652	70.255	phosphoenolpyruvate carboxykinase
bin001	SOY3_bin001_00215	618	355	386	419	68.673	63.351	72.020	2,3-bisphosphoglycerate-dependent phosphoglycerate mutase
bin001	SOY3_bin001_00216	285	554	726	702	232.386	258.373	261.651	Peptidyl-prolyl cis-trans isomerase C
bin001	SOY3_bin001_00217	723	1100	2261	1976	181.886	317.188	290.321	Kynurenine formamidase
bin001	SOY3_bin001_00218	3273	2336	3517	3023	85.324	108.989	98.112	Aerobic respiration control sensor protein ArcB
bin001	SOY3_bin001_00219	1536	667	741	778	51.913	48.931	53.804	putative amino-acid-binding protein YxeM precursor
bin001	SOY3_bin001_00220	1197	485	610	663	48.439	51.688	58.837	hypothetical protein
bin001	SOY3_bin001_00221	2595	2410	2815	2870	111.026	110.026	117.483	Arylsulfatase
bin001	SOY3_bin001_00222	1287	537	579	665	49.882	45.630	54.887	Anaerobic sulfatase-maturating enzyme
bin001	SOY3_bin001_00223	426	316	347	340	88.679	82.618	84.781	Metallothiol transferase FosB 2
bin001	SOY3_bin001_00224	693	789	858	881	136.110	125.577	135.043	hypothetical protein
bin001	SOY3_bin001_00225	696	437	628	488	75.062	91.518	74.480	Alanine--tRNA ligase
bin001	SOY3_bin001_00226	876	766	792	829	104.537	91.701	100.526	prenyltransferase
bin001	SOY3_bin001_00227	1422	3451	4576	4185	290.129	326.393	312.626	Glutamate synthase [NADPH] small chain
bin001	SOY3_bin001_00228	927	2799	3327	3006	360.968	364.022	344.460	Dihydroorotate dehydrogenase B (NAD(+)), electron transfer subunit
bin001	SOY3_bin001_00229	849	1855	2475	2522	261.205	295.680	315.549	putative formate transporter 1
bin001	SOY3_bin001_00230	1251	1156	1203	1094	110.470	97.536	92.894	putative MFS-type transporter YhjX
bin001	SOY3_bin001_00231	639	1036	1267	999	193.823	201.109	166.071	GMP/IMP nucleotidase YrfG
bin001	SOY3_bin001_00232	768	444	899	537	69.114	118.728	74.275	hypothetical protein
bin001	SOY3_bin001_00233	150	102	114	87	81.293	77.085	61.611	hypothetical protein
bin001	SOY3_bin001_00234	615	478	518	412	92.918	85.430	71.163	Putative pseudouridine methyltransferase
bin001	SOY3_bin001_00235	246	310	318	294	150.651	131.113	126.953	TM2 domain protein
bin001	SOY3_bin001_00236	1356	5714	6917	6466	503.763	517.384	506.531	H/ACA RNA-protein complex component Cbf5p
bin001	SOY3_bin001_00237	291	4664	5429	5240	1916.069	1892.263	1912.794	50S ribosomal protein L21e
bin001	SOY3_bin001_00238	345	6079	6669	6285	2106.486	1960.633	1935.156	fructose-6-phosphate aldolase
bin001	SOY3_bin001_00239	582	4960	4465	4176	1018.836	778.132	762.197	Helix-hairpin-helix motif protein
bin001	SOY3_bin001_00240	744	1914	1421	1331	307.549	193.721	190.036	Ribosomal RNA small subunit methyltransferase A
bin001	SOY3_bin001_00241	1434	959	1055	1032	79.949	74.620	76.447	Succinate-semialdehyde dehydrogenase [NADP(+)] GabD
bin001	SOY3_bin001_00242	327	288	404	386	105.291	125.311	125.392	Helix-turn-helix
bin001	SOY3_bin001_00243	807	372	402	336	55.108	50.525	44.228	NH(3)-dependent NAD(+) synthetase
bin001	SOY3_bin001_00244	1290	396	456	426	36.699	35.853	35.079	putative cysteine desulfurase
bin001	SOY3_bin001_00245	756	479	606	577	75.746	81.303	81.074	formate dehydrogenase accessory protein
bin001	SOY3_bin001_00246	903	332	339	327	43.954	38.077	38.467	tRNA 2-thiocytidine biosynthesis protein TtcA
bin001	SOY3_bin001_00247	348	203	180	137	69.737	52.462	41.819	hypothetical protein
bin001	SOY3_bin001_00248	294	315	337	246	128.088	116.262	88.883	Nucleotidyltransferase domain protein
bin001	SOY3_bin001_00249	636	168	329	192	31.579	52.468	32.068	hypothetical protein
bin001	SOY3_bin001_00250	243	64	91	64	31.486	37.983	27.977	hypothetical protein
bin001	SOY3_bin001_00251	903	1793	2737	2539	237.377	307.427	298.679	Quinolinate synthase A
bin001	SOY3_bin001_00252	279	217	198	187	92.982	71.981	71.198	Sulfur carrier protein CysO
bin001	SOY3_bin001_00253	654	972	1133	880	177.678	175.714	142.934	TfuA-like protein
bin001	SOY3_bin001_00254	1221	2184	2800	2639	213.837	232.593	229.590	YcaO-like family protein
bin001	SOY3_bin001_00255	441	353	496	496	95.693	114.077	119.474	Acyl-coenzyme A thioesterase PaaI
bin001	SOY3_bin001_00256	504	384	447	411	91.085	89.956	86.625	hypothetical protein
bin001	SOY3_bin001_00257	1104	2380	2408	2292	257.723	221.229	220.534	Aspartate aminotransferase
bin001	SOY3_bin001_00258	384	5302	5727	5327	1650.646	1512.693	1473.606	translation initiation factor IF-5A
bin001	SOY3_bin001_00259	786	2303	1836	1685	350.281	236.922	227.723	putative S-adenosylmethionine-dependent methyltransferase/MSMEI_2290
bin001	SOY3_bin001_00260	672	590	411	365	104.961	62.034	57.697	cyclic 3',5'-adenosine monophosphate phosphodiesterase
bin001	SOY3_bin001_00261	723	970	858	668	160.391	120.366	98.145	hypothetical protein
bin001	SOY3_bin001_00262	627	59	60	74	11.249	9.706	12.537	Putative electron transport protein YccM
bin001	SOY3_bin001_00263	1557	3638	3553	2884	279.331	231.452	196.760	2-isopropylmalate synthase
bin001	SOY3_bin001_00264	831	2301	2399	2052	331.025	292.809	262.305	putative deoxyribonuclease YcfH
bin001	SOY3_bin001_00265	549	1618	1997	1701	352.332	368.944	329.126	NYN domain protein

bin001	SOY3_bin001_00266	750	1266	1318	1189	201.799	178.242	168.403	Rhomboid protease GlpG
bin001	SOY3_bin001_00267	255	5	5	9	2.344	1.989	3.749	hypothetical protein
bin001	SOY3_bin001_00268	708	548	716	748	92.532	102.573	112.227	Carbamoyltransferase HypF
bin001	SOY3_bin001_00269	1146	2057	2132	2035	214.583	188.694	188.630	Poly(beta-D-mannuronate) C5 epimerase precursor
bin001	SOY3_bin001_00270	1251	3471	4022	3874	331.698	326.092	328.952	Threonine--tRNA ligase 2
bin001	SOY3_bin001_00271	342	504	641	596	176.177	190.102	185.119	Iron hydrogenase 1
bin001	SOY3_bin001_00272	1785	3564	3966	3951	238.696	225.356	235.125	Formyltransferase/hydrolase complex Fhc subunit A
bin001	SOY3_bin001_00273	810	1711	2064	1836	252.529	258.452	240.778	Formyltransferase/hydrolase complex Fhc subunit C
bin001	SOY3_bin001_00274	399	752	891	796	225.315	226.495	211.919	Molydopterin dinucleotide binding domain protein
bin001	SOY3_bin001_00275	225	397	506	517	210.937	228.099	244.083	hypothetical protein
bin001	SOY3_bin001_00276	726	1133	1634	1444	186.569	228.281	211.281	putative adenylyltransferase/sulfurtransferase MoeZ
bin001	SOY3_bin001_00277	450	785	911	808	208.546	205.334	190.734	hypothetical protein
bin001	SOY3_bin001_00278	375	707	805	715	225.389	217.731	202.537	hypothetical protein
bin001	SOY3_bin001_00279	279	396	437	373	169.682	158.866	142.015	Molybdopterin synthase sulfur carrier subunit
bin001	SOY3_bin001_00280	396	303	336	264	91.473	86.060	70.817	Molybdopterin synthase catalytic subunit
bin001	SOY3_bin001_00281	393	144	182	150	43.804	46.971	40.544	Divalent-cation tolerance protein CutA
bin001	SOY3_bin001_00282	348	257	378	302	88.288	110.171	92.184	hypothetical protein
bin001	SOY3_bin001_00283	804	368	485	446	54.719	61.184	58.926	Dihydrolipoyllysine-residue acetyltransferase component of acetoin cleaving system
bin001	SOY3_bin001_00284	291	460	498	396	188.978	173.577	144.555	hypothetical protein
bin001	SOY3_bin001_00285	2208	2767	3084	3094	149.815	141.667	148.851	Maltodextrin phosphorylase
bin001	SOY3_bin001_00286	420	497	524	475	141.466	126.543	120.136	hypothetical protein
bin001	SOY3_bin001_00287	195	197	327	337	120.775	170.086	183.580	hypothetical protein
bin001	SOY3_bin001_00288	1986	1498	1957	1800	90.173	99.946	96.277	Pectinesterase
bin001	SOY3_bin001_00289	198	969	1080	1048	585.065	553.240	562.245	preprotein translocase subunit SecG
bin001	SOY3_bin001_00290	1203	2534	3466	3132	251.818	292.225	276.558	Glutamate decarboxylase
bin001	SOY3_bin001_00291	2283	7945	12547	11448	416.038	557.428	532.664	Phosphoenolpyruvate synthase
bin001	SOY3_bin001_00292	1155	1364	1876	1623	141.182	164.742	149.268	Molybdopterin molybdenumtransferase
bin001	SOY3_bin001_00293	288	247	280	249	102.530	98.610	91.841	hypothetical protein
bin001	SOY3_bin001_00294	642	897	1008	798	167.033	159.250	132.038	HTH-type transcriptional regulator
bin001	SOY3_bin001_00295	2298	5947	6785	6348	309.381	299.471	293.438	ATP-dependent zinc metalloprotease FtsH 2
bin001	SOY3_bin001_00296	1905	14092	17445	15201	884.348	928.818	847.631	Lon protease 2
bin001	SOY3_bin001_00297	1320	5201	5304	4377	471.041	407.553	352.235	protease TldD
bin001	SOY3_bin001_00298	798	1338	1256	1203	200.447	159.640	160.137	hypothetical protein
bin001	SOY3_bin001_00299	369	485	600	586	157.131	164.922	168.695	hypothetical protein
bin001	SOY3_bin001_00300	900	722	814	868	95.905	91.735	102.449	Sirohydrochlorin cobaltochelatase CbiKP precursor
bin001	SOY3_bin001_00301	411	501	604	597	145.727	149.056	154.299	Alkyl hydroperoxide reductase AhpD
bin001	SOY3_bin001_00302	912	465	553	471	60.954	61.501	54.860	Multidrug-efflux transporter 1 regulator
bin001	SOY3_bin001_00303	1626	733	595	558	53.893	37.115	36.454	hypothetical protein
bin001	SOY3_bin001_00304	1320	899	1029	938	81.420	79.067	75.485	hypothetical protein
bin001	SOY3_bin001_00305	819	529	570	516	77.218	70.590	66.926	putative oxidoreductase
bin001	SOY3_bin001_00306	870	415	477	386	57.026	55.610	47.130	acetoacetate decarboxylase
bin001	SOY3_bin001_00307	1944	1068	1228	1069	65.678	64.070	58.413	NADH oxidase
bin001	SOY3_bin001_00308	447	88	106	113	23.535	24.052	26.853	putative transcriptional regulator
bin001	SOY3_bin001_00309	783	204	301	227	31.147	38.991	30.796	hypothetical protein
bin001	SOY3_bin001_00310	216	396	511	463	219.173	239.951	227.697	Copper chaperone CopZ
bin001	SOY3_bin001_00311	162	26	18	16	19.187	11.270	10.491	Flavin reductase like domain protein
bin001	SOY3_bin001_00312	495	990	1358	1332	239.098	278.259	285.844	hypothetical protein
bin001	SOY3_bin001_00313	570	283	424	308	59.355	75.448	57.399	UDP-3-O-[3-hydroxymyristoyl] glucosamine N-acyltransferase
bin001	SOY3_bin001_00314	1245	1012	1033	1330	97.175	84.156	113.478	putative MFS-type transporter YhjX
bin001	SOY3_bin001_00315	1410	8784	8512	8467	744.764	612.304	637.882	Amidophosphoribosyltransferase precursor
bin001	SOY3_bin001_00316	171	3223	3443	3464	2253.252	2042.188	2151.848	50S ribosomal protein L37e
bin001	SOY3_bin001_00317	219	4529	4860	4674	2472.315	2250.852	2267.120	small nuclear ribonucleoprotein
bin001	SOY3_bin001_00318	483	306	387	298	75.739	81.268	65.539	H/ACA RNA-protein complex component Cbf5p
bin001	SOY3_bin001_00319	744	204	227	195	32.780	30.946	27.841	hypothetical protein
bin001	SOY3_bin001_00320	570	1564	1263	1185	328.025	224.741	220.838	L-2,4-diaminobutyric acid acetyltransferase
bin001	SOY3_bin001_00321	1353	2966	2292	2005	262.071	171.819	157.415	Diaminobutyrate--2-oxoglutarate transaminase
bin001	SOY3_bin001_00322	462	800	644	580	207.011	141.383	133.357	L-ectoine synthase
bin001	SOY3_bin001_00323	1308	1783	1240	1139	162.963	96.154	92.501	pyruvate oxidase
bin001	SOY3_bin001_00324	894	2574	2912	2886	344.205	330.376	342.917	hypothetical protein
bin001	SOY3_bin001_00325	282	532	511	472	225.532	183.792	177.796	hypothetical protein
bin001	SOY3_bin001_00326	483	1144	1202	1055	283.155	252.413	232.025	Adenosine specific kinase
bin001	SOY3_bin001_00327	861	2984	3296	3083	414.325	388.274	380.365	Inosine-5'-monophosphate dehydrogenase
bin001	SOY3_bin001_00328	438	1588	1927	1798	433.433	446.234	436.059	Stress response protein NhaX
bin001	SOY3_bin001_00329	264	623	692	704	282.117	265.862	283.269	Atrazine chlorohydrolase
bin001	SOY3_bin001_00330	585	96	77	71	19.618	13.350	12.892	hypothetical protein
bin001	SOY3_bin001_00331	486	171	243	193	42.063	50.714	42.184	Stigma-specific protein, Stig1
bin001	SOY3_bin001_00332	300	93	163	167	37.060	55.109	59.132	hypothetical protein

bin001	SOY3_bin001_00333	516	715	695	659	165.654	136.612	135.664	hypothetical protein
bin001	SOY3_bin001_00334	558	640	695	634	137.117	126.330	120.694	Succinoglycan biosynthesis protein ExoI
bin001	SOY3_bin001_00335	2412	775	872	630	38.412	36.669	27.746	Magnesium-transporting ATPase, P-type 1
bin001	SOY3_bin001_00336	3384	16101	18719	18487	568.811	561.057	580.318	PEGA domain protein
bin001	SOY3_bin001_00337	1047	2360	1956	1852	269.470	189.486	187.899	Daunorubicin/doxorubicin resistance ATP-binding protein DrrA
bin001	SOY3_bin001_00338	786	978	798	822	148.752	102.976	111.091	Inner membrane transport permease YadH
bin001	SOY3_bin001_00339	621	633	457	451	121.859	74.641	77.146	hypothetical protein
bin001	SOY3_bin001_00340	237	866	698	659	436.833	298.718	295.370	hypothetical protein
bin001	SOY3_bin001_00341	270	3553	4183	4006	1573.175	1571.371	1576.075	elongation factor 1-beta
bin001	SOY3_bin001_00342	171	2776	3182	3124	1940.747	1887.378	1940.639	hypothetical protein
bin001	SOY3_bin001_00343	651	156	204	174	28.648	31.784	28.392	uridylate kinase
bin001	SOY3_bin001_00344	429	143	196	161	39.850	46.340	39.866	Bifunctional NMN adenylyltransferase/Nudix hydrolase
bin001	SOY3_bin001_00345	1680	2817	4117	3694	200.458	248.557	233.570	Glutamine--tRNA ligase
bin001	SOY3_bin001_00346	195	3343	6560	5769	2049.497	3412.118	3142.648	Cold shock protein CspD
bin001	SOY3_bin001_00347	1902	1711	1434	1179	107.544	76.470	65.847	Phosphoadenosine phosphosulfate reductase
bin001	SOY3_bin001_00348	417	1879	3439	2953	538.687	836.471	752.241	hypothetical protein
bin001	SOY3_bin001_00349	420	310	389	337	88.238	93.941	85.234	hypothetical protein
bin001	SOY3_bin001_00350	903	1294	1469	1440	171.314	165.002	169.397	Arsenical pump-driving ATPase
bin001	SOY3_bin001_00351	2181	1020	1184	1024	55.910	55.062	49.874	Vault protein inter-alpha-trypsin
bin001	SOY3_bin001_00352	945	381	463	414	48.199	49.694	46.537	Universal stress protein/MT2085
bin001	SOY3_bin001_00353	603	173	189	148	34.298	31.791	26.072	hypothetical protein
bin001	SOY3_bin001_00354	381	1416	1800	1674	444.308	479.184	466.724	hybrid sensory histidine kinase BarA
bin001	SOY3_bin001_00355	111	2347	3065	2839	2527.759	2800.672	2716.891	hypothetical protein
bin001	SOY3_bin001_00356	1053	1963	2712	2729	222.863	261.226	275.299	Putative aminopeptidase YsdC
bin001	SOY3_bin001_00357	1272	752	1514	1169	70.677	120.724	97.624	3-hydroxy-3-methylglutaryl-coenzyme A reductase
bin001	SOY3_bin001_00358	819	471	759	612	68.752	93.997	79.377	Prephenate dehydratase
bin001	SOY3_bin001_00359	231	867	1506	1491	448.696	661.253	685.639	Histone-like transcription factor (CBF/NF-Y) and archaeal histone
bin001	SOY3_bin001_00360	336	874	950	911	310.969	286.774	288.011	hypothetical protein
bin001	SOY3_bin001_00361	249	690	739	660	331.280	301.023	281.562	glutaredoxin-like protein
bin001	SOY3_bin001_00362	666	767	874	805	137.679	133.104	128.396	hypothetical protein
bin001	SOY3_bin001_00363	309	912	1106	1045	352.843	363.037	359.242	Transcription factor S-II (TFIIS)
bin001	SOY3_bin001_00364	735	5595	7040	6974	910.035	971.494	1007.916	DNA polymerase sliding clamp
bin001	SOY3_bin001_00365	1026	2390	2167	2103	278.481	214.223	217.732	DNA primase large subunit
bin001	SOY3_bin001_00366	1770	1993	2033	1881	134.611	116.498	112.887	Molybdopterin molybdenumtransferase
bin001	SOY3_bin001_00367	1089	1348	1654	1515	147.982	154.050	147.780	Chorismate synthase
bin001	SOY3_bin001_00368	1254	1749	2809	2204	166.739	227.200	186.700	Transcriptional regulatory protein AfsQ1
bin001	SOY3_bin001_00369	456	655	813	723	171.720	180.834	168.424	hypothetical protein
bin001	SOY3_bin001_00370	978	1209	1156	1092	147.786	119.887	118.608	Hydroxyacylglutathione hydrolase
bin001	SOY3_bin001_00371	273	713	965	796	312.228	358.525	309.728	Thioredoxin
bin001	SOY3_bin001_00372	465	912	1186	986	234.470	258.694	225.244	hypothetical protein
bin001	SOY3_bin001_00373	966	1995	2462	2314	246.894	258.503	254.458	Antilisterial bacteriocin subtilosin biosynthesis protein AlbA
bin001	SOY3_bin001_00374	519	1040	1358	1305	239.558	265.392	267.099	Aspartate-semialdehyde dehydrogenase
bin001	SOY3_bin001_00375	339	198	293	315	69.825	87.664	98.705	phosphoribosyl-AMP cyclohydrolase
bin001	SOY3_bin001_00376	1137	650	819	779	68.344	73.060	72.779	Antilisterial bacteriocin subtilosin biosynthesis protein AlbA
bin001	SOY3_bin001_00377	747	711	695	705	113.787	94.367	100.253	hypothetical protein
bin001	SOY3_bin001_00378	858	4844	5458	4897	674.936	645.210	606.279	hypothetical protein
bin001	SOY3_bin001_00379	369	10799	11269	10704	3498.668	3097.517	3081.410	Ribosome-associated protein L7Ae-like protein
bin001	SOY3_bin001_00380	213	8135	8427	7978	4565.869	4012.806	3978.728	30S ribosomal protein S28e
bin001	SOY3_bin001_00381	171	4763	4942	4616	3329.891	2931.308	2867.474	50S ribosomal protein L24e
bin001	SOY3_bin001_00382	432	7235	7257	6677	2002.167	1703.837	1641.827	Nucleoside diphosphate kinase
bin001	SOY3_bin001_00383	1809	9330	7937	7334	616.579	445.013	430.658	Translation initiation factor IF-2
bin001	SOY3_bin001_00384	600	1561	1131	1054	311.026	191.190	186.603	Sulfite exporter TauE/SafE
bin001	SOY3_bin001_00385	195	16868	15773	15138	10341.285	8204.166	8246.387	Cold shock protein 2
bin001	SOY3_bin001_00386	1680	21483	15293	14111	1528.731	923.290	892.233	60 kDa chaperonin
bin001	SOY3_bin001_00387	786	872	1371	1179	132.629	176.917	159.339	Putative cardiolipin synthase YbhO
bin001	SOY3_bin001_00388	504	780	790	703	185.016	158.983	148.168	Ribosomal RNA small subunit methyltransferase C
bin001	SOY3_bin001_00389	963	1448	1497	1471	179.758	157.670	162.262	GTPase Obg
bin001	SOY3_bin001_00390	285	2738	2043	2028	1148.508	727.073	755.880	H/ACA RNA-protein complex component Gar1
bin001	SOY3_bin001_00391	495	1141	1713	1604	275.566	351.000	344.214	hypothetical protein
bin001	SOY3_bin001_00392	471	1199	1872	1862	304.329	403.125	419.941	hypothetical protein
bin001	SOY3_bin001_00393	291	770	1188	1130	316.332	414.074	412.492	hypothetical protein
bin001	SOY3_bin001_00394	960	7542	8766	8639	939.206	926.158	955.921	corrinoid ABC transporter substrate-binding protein
bin001	SOY3_bin001_00395	216	371	487	388	205.336	228.681	190.813	adenine phosphoribosyltransferase
bin001	SOY3_bin001_00396	1086	797	749	654	87.735	69.953	63.970	putative permease
bin001	SOY3_bin001_00397	345	133	94	100	46.087	27.635	30.790	hypothetical protein
bin001	SOY3_bin001_00398	351	81	144	94	27.588	41.611	28.448	hypothetical protein
bin001	SOY3_bin001_00399	273	171	108	80	74.882	40.125	31.128	MtN3/saliva family protein

bin001	SOY3_bin001_00400	1242	4373	5101	5026	420.924	416.570	429.864	L-asparaginase 1
bin001	SOY3_bin001_00401	516	7838	8134	7429	1815.938	1598.856	1529.363	50S ribosomal protein L16
bin001	SOY3_bin001_00402	630	10198	9870	9102	1935.174	1589.027	1534.709	23S rRNA (uracil-C(5))-methyltransferase RlmCD
bin001	SOY3_bin001_00403	234	2504	2052	1953	1279.275	889.439	886.576	hypothetical protein
bin001	SOY3_bin001_00404	741	2466	1650	1516	397.851	225.850	217.326	Coenzyme F420:L-glutamate ligase
bin001	SOY3_bin001_00405	135	76	68	73	67.302	51.089	57.441	hypothetical protein
bin001	SOY3_bin001_00406	711	894	1014	1199	150.319	144.652	179.135	DNA alkylation repair enzyme
bin001	SOY3_bin001_00407	270	90	96	124	39.850	36.063	48.785	hypothetical protein
bin001	SOY3_bin001_00408	2031	466	818	644	27.430	40.851	33.683	Ribonucleoside-diphosphate reductase NrdZ
bin001	SOY3_bin001_00409	777	1680	1677	1573	258.484	218.911	215.049	Septum site-determining protein MinD
bin001	SOY3_bin001_00410	996	2779	3180	2905	333.561	323.834	309.825	Phosphoribosylformylglycinamidine cyclo-ligase
bin001	SOY3_bin001_00411	1395	4833	6527	6299	414.179	474.563	479.653	Bifunctional aspartokinase/homoserine dehydrogenase 1
bin001	SOY3_bin001_00412	2196	33504	32924	30529	1823.937	1520.670	1476.760	Elongation factor G
bin001	SOY3_bin001_00413	1275	37482	39416	37050	3514.456	3135.574	3086.795	Elongation factor Tu-B
bin001	SOY3_bin001_00414	309	8020	7947	7552	3102.855	2608.552	2596.171	30S ribosomal protein S10
bin001	SOY3_bin001_00415	76	66	44	35	103.819	58.721	48.920	tRNA-Arg(ccg)
bin001	SOY3_bin001_00416	201	115	249	213	68.399	125.649	112.568	hypothetical protein
bin001	SOY3_bin001_00417	702	1402	1799	1564	238.757	259.925	236.663	hypothetical protein
bin001	SOY3_bin001_00418	864	1448	1700	1671	200.355	199.568	205.444	putative copper-importing P-type ATPase A
bin001	SOY3_bin001_00419	555	4343	3480	3249	935.497	635.976	621.851	hypothetical protein
bin001	SOY3_bin001_00420	2346	2095	1934	1674	106.758	83.615	75.798	Putative membrane protein YdgH
bin001	SOY3_bin001_00421	222	629	829	803	338.722	378.753	384.231	hypothetical protein
bin001	SOY3_bin001_00422	609	1790	2592	2390	351.383	431.690	416.879	Cobalt-precorrin-2 C(20)-methyltransferase
bin001	SOY3_bin001_00423	513	1551	2175	2159	361.443	430.028	447.059	putative cobalt-precorrin-6Y C(15)-methyltransferase [decarboxylating]
bin001	SOY3_bin001_00424	915	607	736	685	79.307	81.585	79.524	D-alanine--D-alanine ligase
bin001	SOY3_bin001_00425	159	260	264	312	195.489	168.408	208.443	hypothetical protein
bin001	SOY3_bin001_00426	162	360	400	399	265.664	250.438	261.630	hypothetical protein
bin001	SOY3_bin001_00427	438	841	1000	965	229.545	231.569	234.036	putative sugar kinase YdjH
bin001	SOY3_bin001_00428	225	495	675	573	263.008	304.282	270.522	hypothetical protein
bin001	SOY3_bin001_00429	606	482	506	477	95.087	84.690	83.613	hypothetical protein
bin001	SOY3_bin001_00430	2091	3593	4830	4403	205.423	234.287	223.679	Formate dehydrogenase H
bin001	SOY3_bin001_00431	1761	5449	6684	6319	369.916	384.974	381.170	Thiamine import ATP-binding protein ThiQ
bin001	SOY3_bin001_00432	435	2371	3107	3002	651.610	724.447	733.080	hypothetical protein
bin001	SOY3_bin001_00433	459	1296	1524	1355	337.550	336.765	313.586	hypothetical protein
bin001	SOY3_bin001_00434	618	2042	2223	1941	395.014	364.843	333.631	Bifunctional protein PyrR
bin001	SOY3_bin001_00435	390	9529	9674	9102	2920.978	2515.917	2479.146	30S ribosomal protein S8
bin001	SOY3_bin001_00436	531	12720	12389	11901	2863.770	2366.445	2380.777	50S ribosomal protein L6
bin001	SOY3_bin001_00437	375	9761	9752	9299	3111.778	2637.650	2634.115	50S ribosomal protein L32e
bin001	SOY3_bin001_00438	462	12058	11592	10837	3120.174	2544.903	2491.707	50S ribosomal protein L19e
bin001	SOY3_bin001_00439	528	12204	12114	11543	2763.209	2327.064	2322.280	50S ribosomal protein L18P
bin001	SOY3_bin001_00440	624	16535	16084	15452	3167.854	2614.353	2630.449	30S ribosomal protein S5
bin001	SOY3_bin001_00441	456	11582	11319	10932	3036.437	2517.665	2546.623	50S ribosomal protein L30P
bin001	SOY3_bin001_00442	378	10173	10412	9841	3217.383	2793.812	2765.523	50S ribosomal protein L15
bin001	SOY3_bin001_00443	1614	22196	21902	21054	1644.056	1376.369	1385.673	preprotein translocase subunit SecY
bin001	SOY3_bin001_00444	366	511	536	554	166.911	148.538	160.790	hypothetical protein
bin001	SOY3_bin001_00445	2085	3196	3544	3472	183.251	172.402	176.890	MCM2/3/5 family protein
bin001	SOY3_bin001_00446	1317	1773	2059	1827	160.942	158.572	147.361	hypothetical protein
bin001	SOY3_bin001_00447	852	1920	2427	2196	269.406	288.925	273.793	Modulator of FtsH protease HflK
bin001	SOY3_bin001_00448	183	2411	2480	2316	1575.040	1374.534	1344.365	30S ribosomal protein S27ae
bin001	SOY3_bin001_00449	309	4046	4296	3929	1565.355	1410.135	1350.683	30S ribosomal protein S24e
bin001	SOY3_bin001_00450	531	4681	5327	4891	1053.876	1017.520	978.437	hypothetical protein
bin001	SOY3_bin001_00451	186	1393	1746	1556	895.331	952.108	888.641	DNA-directed RNA polymerase subunit E''
bin001	SOY3_bin001_00452	585	4890	5796	5263	999.306	1004.910	955.669	Ribonuclease R
bin001	SOY3_bin001_00453	408	1927	1900	1748	564.634	472.333	455.104	Fcf1
bin001	SOY3_bin001_00454	1296	6586	6516	6140	607.522	509.954	503.261	Elongation factor Tu
bin001	SOY3_bin001_00455	963	646	1017	1016	80.196	107.115	112.072	Chagasin family peptidase inhibitor I42
bin001	SOY3_bin001_00456	930	145	156	166	18.639	17.014	18.961	Thiamine-monophosphate kinase
bin001	SOY3_bin001_00457	123	12	15	18	11.663	12.369	15.545	putative monovalent cation/H+ antiporter subunit B
bin001	SOY3_bin001_00458	525	38	163	98	8.653	31.491	19.829	Na(+)/H(+) antiporter subunit E
bin001	SOY3_bin001_00459	315	27	210	121	10.247	67.618	40.804	Na(+)/H(+) antiporter subunit G1
bin001	SOY3_bin001_00460	270	9	168	91	3.985	63.110	35.802	Na(+)/H(+) antiporter subunit F
bin001	SOY3_bin001_00461	351	8	92	51	2.725	26.585	15.435	hypothetical protein
bin001	SOY3_bin001_00462	267	119	179	149	53.282	67.998	59.280	hypothetical protein
bin001	SOY3_bin001_00463	561	335	531	509	71.388	96.003	96.380	hypothetical protein
bin001	SOY3_bin001_00464	408	1796	2423	2608	526.250	602.349	679.012	Putative nickel-responsive regulator
bin001	SOY3_bin001_00465	681	1994	2591	2683	350.045	385.900	418.508	fibrillarin
bin001	SOY3_bin001_00466	774	1242	1423	1331	191.834	186.474	182.670	Peptidase S24-like protein

bin001	SOY3_bin001_00467	1623	1742	2355	2107	128.314	147.173	137.904	DNA polymerase II small subunit
bin001	SOY3_bin001_00468	390	7191	11899	10641	2204.298	3094.572	2898.329	hypothetical protein
bin001	SOY3_bin001_00469	516	1657	2082	1822	383.900	409.247	375.084	hypothetical protein
bin001	SOY3_bin001_00470	1290	4173	5347	4915	386.727	420.412	404.728	Phosphomethylpyrimidine synthase
bin001	SOY3_bin001_00471	639	715	847	783	133.768	134.443	130.164	putative polyketide biosynthesis zinc-dependent hydrolase PksB
bin001	SOY3_bin001_00472	1164	2801	4039	3626	287.677	351.946	330.906	Acetylornithine aminotransferase
bin001	SOY3_bin001_00473	1056	2436	3413	3190	275.778	327.814	320.890	Histidinol-phosphate aminotransferase
bin001	SOY3_bin001_00474	504	1242	1283	1150	294.603	258.197	242.380	putative kinase
bin001	SOY3_bin001_00475	852	5316	3823	3774	745.918	455.113	470.535	Ribose-phosphate pyrophosphokinase
bin001	SOY3_bin001_00476	309	2905	2157	2123	1123.914	708.021	729.829	hypothetical protein
bin001	SOY3_bin001_00477	645	2371	1995	1834	439.458	313.717	302.043	Methionine aminopeptidase
bin001	SOY3_bin001_00478	600	3229	3381	3342	643.372	571.543	591.677	hypothetical protein
bin001	SOY3_bin001_00479	510	2711	2862	2978	635.484	569.186	620.275	Kinase binding protein CGI-121
bin001	SOY3_bin001_00480	861	16880	17731	15407	2343.769	2088.742	1900.836	hypothetical protein
bin001	SOY3_bin001_00481	924	15307	14975	14657	1980.449	1643.802	1685.012	hypothetical protein
bin001	SOY3_bin001_00482	633	1803	1385	1244	340.516	221.922	208.760	hypothetical protein
bin001	SOY3_bin001_00483	636	1911	1478	1435	359.211	235.707	239.676	Thymidylate kinase
bin001	SOY3_bin001_00484	936	10554	13607	13617	1347.990	1474.488	1545.380	Manganese-dependent inorganic pyrophosphatase
bin001	SOY3_bin001_00485	1338	378	529	404	33.774	40.101	32.074	hypothetical protein
bin001	SOY3_bin001_00486	1239	1554	2069	1812	149.943	169.373	155.352	Cytochrome C biogenesis protein transmembrane region
bin001	SOY3_bin001_00487	588	1736	3006	2365	352.954	518.521	427.252	hypothetical protein
bin001	SOY3_bin001_00488	1530	1654	2222	1907	129.238	147.302	132.400	Poly(beta-D-mannuronate) C5 epimerase precursor
bin001	SOY3_bin001_00489	1149	1230	1453	1455	127.977	128.263	134.516	Putative thiosulfate sulfurtransferase
bin001	SOY3_bin001_00490	258	355	476	447	164.496	187.129	184.042	hypothetical protein
bin001	SOY3_bin001_00491	585	843	1004	1041	172.273	174.073	189.027	hypothetical protein
bin001	SOY3_bin001_00492	2046	2682	3273	3037	156.711	162.254	157.677	Putative membrane protein YdgH
bin001	SOY3_bin001_00493	1053	1634	1841	1871	185.511	177.329	188.745	NPCBM-associated, NEW3 domain of alpha-galactosidase
bin001	SOY3_bin001_00494	1986	431	528	523	25.944	26.966	27.974	Copper-exporting P-type ATPase B
bin001	SOY3_bin001_00495	1452	537	687	610	44.213	47.989	44.627	hypothetical protein
bin001	SOY3_bin001_00496	921	1747	1912	1813	226.766	210.563	209.107	hypothetical protein
bin001	SOY3_bin001_00497	486	989	1231	1013	243.280	256.907	221.413	AP-4-A phosphorylase
bin001	SOY3_bin001_00498	615	1267	1570	1257	246.290	258.928	217.115	Phosphoribosylglycinamide formyltransferase
bin001	SOY3_bin001_00499	270	753	918	884	333.409	344.853	347.791	DNA/RNA-binding protein albA
bin001	SOY3_bin001_00500	579	379	426	357	78.254	74.625	65.497	bifunctional UGMP family protein/serine/threonine protein kinase
bin001	SOY3_bin001_00501	1038	774	804	762	89.143	78.562	77.981	hypothetical protein
bin001	SOY3_bin001_00502	477	405	539	565	101.504	114.611	125.823	Molybdenum cofactor biosynthesis protein B
bin001	SOY3_bin001_00503	555	3070	5551	4770	661.288	1014.455	912.967	Hypoxic response protein 1
bin001	SOY3_bin001_00504	780	2117	4064	3449	324.468	528.462	469.708	Hypoxic response protein 1
bin001	SOY3_bin001_00505	978	2977	4897	4176	363.903	507.862	453.577	Hypoxic response protein 1
bin001	SOY3_bin001_00506	861	3793	5492	4914	526.654	646.967	606.264	Inosine-5'-monophosphate dehydrogenase
bin001	SOY3_bin001_00507	780	3392	4999	4492	519.884	650.045	611.751	inosine 5'-monophosphate dehydrogenase
bin001	SOY3_bin001_00508	1122	1451	1866	1741	154.604	168.684	164.830	ATP dependent DNA ligase domain protein
bin001	SOY3_bin001_00509	1236	4316	4651	4490	417.454	381.665	385.885	pyruvate oxidase
bin001	SOY3_bin001_00510	91	71	90	73	93.274	100.313	85.214	tRNA-Tyr(gta)
bin001	SOY3_bin001_00511	552	2473	1881	1806	535.588	345.624	347.543	transcription factor
bin001	SOY3_bin001_00512	2676	4751	5543	4986	212.248	210.094	197.923	DNA polymerase II
bin001	SOY3_bin001_00513	648	783	683	612	144.455	106.906	100.324	hypothetical protein
bin001	SOY3_bin001_00514	1071	920	1454	1099	102.694	137.699	109.003	hypothetical protein
bin001	SOY3_bin001_00515	1980	1500	1596	1434	90.567	81.757	76.933	Poly(beta-D-mannuronate) C5 epimerase precursor
bin001	SOY3_bin001_00516	195	34	25	31	20.844	13.003	16.887	putative protease YdeA
bin001	SOY3_bin001_00517	552	117	122	115	25.339	22.417	22.130	Putative ribosomal N-acetyltransferase YdaF
bin001	SOY3_bin001_00518	528	391	456	431	88.530	87.596	86.711	Imidazoleglycerol-phosphate dehydratase
bin001	SOY3_bin001_00519	2976	4792	5245	5222	192.499	178.759	186.395	hypothetical protein
bin001	SOY3_bin001_00520	816	1242	1564	1412	181.960	194.402	183.812	Nitrogenase iron protein 1
bin001	SOY3_bin001_00521	1401	1373	1758	1554	117.160	127.273	117.826	Cobyrinic acid A,C-diamide synthase
bin001	SOY3_bin001_00522	405	1543	2394	2060	455.466	599.548	540.309	pyridoxamine 5'-phosphate oxidase
bin001	SOY3_bin001_00523	882	757	1311	1026	102.606	150.761	123.569	dihydroxynaphthoic acid synthetase
bin001	SOY3_bin001_00524	150	88	138	80	70.135	93.313	56.654	hypothetical protein
bin001	SOY3_bin001_00525	336	187	262	176	66.535	79.089	55.642	hypothetical protein
bin001	SOY3_bin001_00526	468	204	224	202	52.111	48.546	45.850	hypothetical protein
bin001	SOY3_bin001_00527	339	801	1126	845	282.474	336.894	264.781	Rubrerythrin
bin001	SOY3_bin001_00528	600	651	577	511	129.711	97.539	90.469	Putative oxidoreductase/MT0587
bin001	SOY3_bin001_00529	402	6872	6674	6317	2043.632	1683.895	1669.224	30S ribosomal protein S6e
bin001	SOY3_bin001_00530	999	1180	1508	1502	141.209	153.105	159.711	Ribonuclease R winged-helix domain protein
bin001	SOY3_bin001_00531	74	36	30	24	58.159	41.119	34.452	tRNA-Gln(ttg)
bin001	SOY3_bin001_00532	396	575	685	629	173.587	175.449	168.727	hypothetical protein
bin001	SOY3_bin001_00533	390	832	1251	1014	255.038	325.347	276.187	hypothetical protein

bin001	SOY3_bin001_00534	744	1625	2326	2152	261.111	317.097	307.255	PAC2 family protein
bin001	SOY3_bin001_00535	2076	2753	3627	3184	158.535	177.205	162.920	Succinyl-CoA ligase [ADP-forming] subunit alpha
bin001	SOY3_bin001_00536	594	2897	3450	3264	583.052	589.098	583.705	hypothetical protein
bin001	SOY3_bin001_00537	564	852	719	684	180.595	129.302	128.827	hypothetical protein
bin001	SOY3_bin001_00538	1338	4286	5425	4939	382.950	411.243	392.114	Signal recognition particle protein
bin001	SOY3_bin001_00539	573	261	345	306	54.454	61.069	56.728	hypothetical protein
bin001	SOY3_bin001_00540	1044	507	692	569	58.057	67.230	57.895	Papain family cysteine protease
bin001	SOY3_bin001_00541	450	6977	4867	4478	1853.539	1096.992	1057.064	hypothetical protein
bin001	SOY3_bin001_00542	363	616	534	510	202.871	149.207	149.243	hypothetical protein
bin001	SOY3_bin001_00543	588	885	1059	1008	179.933	182.673	182.101	molybdopterin-guanine dinucleotide biosynthesis protein MobA
bin001	SOY3_bin001_00544	765	1073	1381	1237	167.681	183.099	171.766	Cobalamin synthase
bin001	SOY3_bin001_00545	480	888	1199	1124	221.165	253.357	248.745	Phosphatidylglycerophosphatase A
bin001	SOY3_bin001_00546	351	922	1283	1108	314.029	370.744	335.322	hypothetical protein
bin001	SOY3_bin001_00547	228	2741	3257	2798	1437.208	1448.898	1303.595	Fe/S biogenesis protein NfuA
bin001	SOY3_bin001_00548	225	2303	2706	2400	1223.649	1219.832	1133.075	small nuclear ribonucleoprotein
bin001	SOY3_bin001_00549	402	3348	3605	3271	995.646	909.565	864.339	B-block binding subunit of TFIIIC
bin001	SOY3_bin001_00550	297	3861	4085	3869	1554.136	1395.052	1383.796	translation initiation factor Sui1
bin001	SOY3_bin001_00551	2361	1265	1613	1414	64.053	69.294	63.618	von Willebrand factor type A domain protein
bin001	SOY3_bin001_00552	357	183	316	303	61.281	89.779	90.158	hypothetical protein
bin001	SOY3_bin001_00553	417	4655	4166	4231	1334.533	1013.300	1077.797	hypothetical protein
bin001	SOY3_bin001_00554	678	4129	3408	3336	728.049	509.829	522.668	ribonuclease P protein component 3
bin001	SOY3_bin001_00555	498	2210	1795	1795	530.528	365.586	382.882	ribonuclease P protein component 2
bin001	SOY3_bin001_00556	774	4524	4699	4223	698.759	615.771	579.575	Proteasome subunit beta precursor
bin001	SOY3_bin001_00557	699	4522	4502	4236	773.391	653.255	643.737	Shwachman-Bodian-Diamond syndrome (SBDS) protein
bin001	SOY3_bin001_00558	666	5769	5579	5185	1035.552	849.644	826.998	Polyribonucleotide nucleotidyltransferase
bin001	SOY3_bin001_00559	762	7449	7196	6860	1168.661	957.836	956.311	Ribonuclease PH
bin001	SOY3_bin001_00560	777	7179	7223	6688	1104.558	942.869	914.334	Ribonuclease PH
bin001	SOY3_bin001_00561	282	6249	7231	6738	2649.152	2600.782	2538.117	50S ribosomal protein L37Ae
bin001	SOY3_bin001_00562	138	2351	2703	2592	2036.663	1986.652	1995.197	DNA-directed RNA polymerase subunit P
bin001	SOY3_bin001_00563	411	7278	7962	7230	2116.975	1964.876	1868.643	ribosomal biogenesis protein
bin001	SOY3_bin001_00564	228	3583	3755	3557	1878.700	1670.436	1657.215	Transcription factor Pcc1
bin001	SOY3_bin001_00565	363	5380	5840	5545	1771.826	1631.778	1622.650	prefoldin subunit beta
bin001	SOY3_bin001_00566	77	159	163	157	246.861	214.710	216.590	tRNA-Arg(tct)
bin001	SOY3_bin001_00567	186	2030	1714	1625	1304.754	934.658	928.048	putative RNA-binding protein
bin001	SOY3_bin001_00568	570	6341	5688	5521	1329.929	1012.137	1028.899	hypothetical protein
bin001	SOY3_bin001_00569	1026	1317	1590	1396	153.456	157.183	144.533	putative deoxyhypusine synthase
bin001	SOY3_bin001_00570	1110	2376	2730	2391	255.899	249.456	228.816	Putative transposase DNA-binding domain protein
bin001	SOY3_bin001_00571	321	351	320	304	130.722	101.111	100.600	hypothetical protein
bin001	SOY3_bin001_00572	633	1504	1517	1455	284.047	243.073	244.168	DNA polymerase/3'-5' exonuclease PolX
bin001	SOY3_bin001_00573	315	797	1000	882	302.478	321.991	297.432	signal recognition particle protein Srp19
bin001	SOY3_bin001_00574	909	2710	3745	3593	356.411	417.871	419.878	FeMo cofactor biosynthesis protein NifB
bin001	SOY3_bin001_00575	222	871	1664	1606	469.041	760.248	768.462	hypothetical protein
bin001	SOY3_bin001_00576	981	1049	1469	1327	127.836	151.882	143.692	L-lysine cyclodeaminase
bin001	SOY3_bin001_00577	504	839	869	877	199.011	174.882	184.841	hypothetical protein
bin001	SOY3_bin001_00578	612	1451	1830	1700	283.440	303.287	295.072	ATP-dependent zinc metalloprotease FtsH 2
bin001	SOY3_bin001_00579	3183	3949	6574	6912	148.319	209.483	230.673	hypothetical protein
bin001	SOY3_bin001_00580	357	2092	1760	1602	700.550	500.034	476.677	hypothetical protein
bin001	SOY3_bin001_00581	273	1574	1389	1278	689.267	516.053	497.277	Glutamine amidotransferase subunit PdxT
bin001	SOY3_bin001_00582	777	952	1182	1196	146.474	154.295	163.508	Transmembrane exosortase (Exosortase_EpsH)
bin001	SOY3_bin001_00583	666	788	857	951	141.448	130.515	151.683	phosphatidylserine decarboxylase
bin001	SOY3_bin001_00584	300	381	395	438	151.827	133.546	155.090	Glutamine--fructose-6-phosphate aminotransferase [isomerizing]
bin001	SOY3_bin001_00585	996	1222	1377	1211	146.675	140.226	129.156	putative glycosyl transferase
bin001	SOY3_bin001_00586	1077	1209	1573	1329	134.201	148.138	131.081	UDP-2,3-diacetamido-2,3-dideoxy-D-glucuronate 2-epimerase
bin001	SOY3_bin001_00587	1197	1275	1732	1587	127.339	146.760	140.836	UDP-N-acetyl-D-glucosamine 6-dehydrogenase
bin001	SOY3_bin001_00588	618	911	1146	1139	176.228	188.084	195.779	dTDP-3-amino-3,6-dideoxy-alpha-D-galactopyranose 3-N-acetyltransferase
bin001	SOY3_bin001_00589	936	1780	2285	2123	227.347	247.608	240.937	Putative oxidoreductase YceM
bin001	SOY3_bin001_00590	1101	2099	2827	2719	227.914	260.431	262.332	UDP-2-acetamido-2-deoxy-3-oxo-D-glucuronate aminotransferase
bin001	SOY3_bin001_00591	1215	1359	1887	1569	133.718	157.525	137.175	Bifunctional protein GlmU
bin001	SOY3_bin001_00592	684	382	670	611	66.766	99.351	94.889	hypothetical protein
bin001	SOY3_bin001_00593	321	45	69	55	16.759	21.802	18.201	hypothetical protein
bin001	SOY3_bin001_00594	375	29	64	45	9.245	17.310	12.747	hypothetical protein
bin001	SOY3_bin001_00595	243	2	1	1	0.984	0.417	0.437	hypothetical protein
bin001	SOY3_bin001_00596	1272	250	438	347	23.496	34.925	28.978	Tripartite tricarboxylate transporter TctA family protein
bin001	SOY3_bin001_00597	360	4630	5460	5099	1537.531	1538.314	1504.570	50S ribosomal protein L15
bin001	SOY3_bin001_00598	420	5741	6098	5737	1634.119	1472.627	1450.993	50S ribosomal protein L13
bin001	SOY3_bin001_00599	399	7708	7418	7236	2309.481	1885.683	1926.440	30S ribosomal protein S9
bin001	SOY3_bin001_00600	186	3362	3376	3032	2160.878	1840.960	1731.594	DNA-directed RNA polymerase subunit N

bin001	SOY3_bin001_00601	75	110	124	111	175.338	167.693	157.214	tRNA-Pro(cgg)
bin001	SOY3_bin001_00602	180	2044	1973	1902	1357.544	1111.756	1122.452	DNA-directed RNA polymerase subunit K
bin001	SOY3_bin001_00603	603	10474	10652	10334	2076.542	1791.714	1820.459	30S ribosomal protein S2
bin001	SOY3_bin001_00604	789	8930	8484	8261	1353.069	1090.632	1112.207	hypothetical protein
bin001	SOY3_bin001_00605	954	4631	4072	4186	580.326	432.926	466.102	D-glycero-alpha-D-manno-heptose 7-phosphate kinase
bin001	SOY3_bin001_00606	753	3251	2788	2836	516.140	375.537	400.075	acetylglutamate kinase
bin001	SOY3_bin001_00607	1098	2138	1723	1743	232.783	159.161	168.626	Isopentenyl-diphosphate delta-isomerase
bin001	SOY3_bin001_00608	1338	5553	4907	4911	496.155	371.976	389.891	Ribonuclease J 1
bin001	SOY3_bin001_00609	963	4089	3613	3383	507.617	380.537	373.169	(2E,6E)-farnesyl diphosphate synthase
bin001	SOY3_bin001_00610	609	35644	35809	36254	6997.045	5963.890	6323.659	hypothetical protein
bin001	SOY3_bin001_00611	1062	63669	66899	67365	7167.192	6389.250	6738.134	hypothetical protein
bin001	SOY3_bin001_00612	2076	1644	3519	3546	94.672	171.928	181.443	Acetyl-coenzyme A synthetase
bin001	SOY3_bin001_00613	2010	982	9275	5384	58.406	468.029	284.537	Acetyl-coenzyme A synthetase
bin001	SOY3_bin001_00614	1314	438	483	470	39.850	37.283	37.996	(Dimethylallyl)adenosine tRNA methylthiotransferase MiaB
bin001	SOY3_bin001_00615	834	512	726	701	73.392	88.293	89.286	putative inner membrane protein
bin001	SOY3_bin001_00616	390	382	464	400	117.097	120.672	108.949	hypothetical protein
bin001	SOY3_bin001_00617	615	826	983	856	160.565	162.119	147.852	Superoxide dismutase [Mn]
bin001	SOY3_bin001_00618	726	805	1011	927	132.558	141.244	135.635	Alkyl hydroperoxide reductase subunit C
bin001	SOY3_bin001_00619	489	197	227	190	48.162	47.084	41.274	Transcriptional regulator PerR
bin001	SOY3_bin001_00620	831	3260	5448	4863	468.988	664.953	621.632	hypothetical protein
bin001	SOY3_bin001_00621	708	562	616	549	94.896	88.247	82.370	Rhomboid protease GluP
bin001	SOY3_bin001_00622	678	2153	1923	1822	379.629	287.676	285.462	TPR repeat-containing protein YrrB
bin001	SOY3_bin001_00623	90	439	331	321	583.133	373.027	378.872	hypothetical protein
bin001	SOY3_bin001_00624	1167	10163	9151	8369	1041.110	795.339	761.785	Signal recognition particle protein
bin001	SOY3_bin001_00625	456	5132	5306	4609	1345.449	1180.204	1073.672	prefoldin subunit alpha
bin001	SOY3_bin001_00626	189	1880	1986	1859	1189.164	1065.791	1044.834	50S ribosomal protein LX
bin001	SOY3_bin001_00627	699	7246	7076	6491	1239.272	1026.752	986.425	translation initiation factor IF-6
bin001	SOY3_bin001_00628	267	2761	2883	2722	1236.234	1095.187	1082.946	50S ribosomal protein L31e
bin001	SOY3_bin001_00629	684	7400	7468	6879	1293.365	1107.396	1068.314	7-cyano-7-deazaguanine synthase
bin001	SOY3_bin001_00630	342	4408	4334	4024	1540.852	1285.339	1249.861	hypothetical protein
bin001	SOY3_bin001_00631	264	3969	3916	3903	1797.309	1504.505	1570.451	30S ribosomal protein S19e
bin001	SOY3_bin001_00632	390	865	1722	924	265.153	447.840	251.673	hypothetical protein
bin001	SOY3_bin001_00633	1230	2694	4460	2436	261.841	367.777	210.379	Macrolide export ATP-binding/permease protein MacB
bin001	SOY3_bin001_00634	324	548	839	513	202.200	262.647	168.191	hypothetical protein
bin001	SOY3_bin001_00635	825	644	766	562	93.321	94.174	72.362	hypothetical protein
bin001	SOY3_bin001_00636	783	235	225	194	35.880	29.146	26.319	Ferredoxin-1
bin001	SOY3_bin001_00637	288	295	237	239	122.455	83.466	88.153	hypothetical protein
bin001	SOY3_bin001_00638	198	1276	1533	1637	770.426	785.293	878.240	Transposase zinc-ribbon domain protein
bin001	SOY3_bin001_00639	1122	3722	4240	4208	396.578	383.290	398.394	Acetoin utilization protein AcuC
bin001	SOY3_bin001_00640	813	1825	2384	2426	268.360	297.420	316.979	L-aspartate dehydrogenase
bin001	SOY3_bin001_00641	1467	2704	3270	3132	220.355	226.085	226.789	Argininosuccinate lyase 1
bin001	SOY3_bin001_00642	1164	1924	2431	2279	197.605	211.830	207.980	Arginine biosynthesis bifunctional protein ArgJ
bin001	SOY3_bin001_00643	483	900	1108	1015	222.762	232.674	223.228	Hypoxic response protein 1
bin001	SOY3_bin001_00644	1026	1913	2249	2230	222.902	222.329	230.881	N-acetyl-gamma-glutamyl-phosphate reductase
bin001	SOY3_bin001_00645	966	1086	1116	1145	134.400	117.177	125.909	ADP-ribosyl-[dinitrogen reductase] glycohydrolase
bin001	SOY3_bin001_00646	1176	1518	1704	1710	154.316	146.966	154.461	2,3-bisphosphoglycerate-independent phosphoglycerate mutase
bin001	SOY3_bin001_00647	654	785	1026	897	143.495	159.120	145.695	Methyl-coenzyme M reductase operon protein C
bin001	SOY3_bin001_00648	798	610	747	637	91.384	94.945	84.794	diphthine synthase
bin001	SOY3_bin001_00649	171	354	725	559	247.487	430.028	347.253	hypothetical protein
bin001	SOY3_bin001_00650	189	357	607	483	225.815	325.748	271.466	hypothetical protein
bin001	SOY3_bin001_00651	219	100	160	117	54.589	74.102	56.751	hypothetical protein
bin001	SOY3_bin001_00652	414	355	660	478	102.512	161.696	122.647	hypothetical protein
bin001	SOY3_bin001_00653	531	481	836	761	108.292	159.686	152.237	hypothetical protein
bin001	SOY3_bin001_00654	411	134	146	181	38.977	36.030	46.781	hypothetical protein
bin001	SOY3_bin001_00655	2472	504	480	492	24.374	19.695	21.142	CHAT domain protein
bin001	SOY3_bin001_00656	636	286	296	258	53.759	47.205	43.092	Putative phosphoribosyl transferase/MT0597
bin001	SOY3_bin001_00657	939	1515	1776	1719	192.882	191.837	194.464	putative manganese-dependent inorganic pyrophosphatase
bin001	SOY3_bin001_00658	1221	5141	6880	6765	503.359	571.515	588.548	Bifunctional PGK/TIM
bin001	SOY3_bin001_00659	219	71	49	42	38.758	22.694	20.372	hypothetical protein
bin001	SOY3_bin001_00660	201	5474	6378	6345	3255.774	3218.424	3353.246	hypothetical protein
bin001	SOY3_bin001_00661	897	216	165	165	28.788	18.657	19.540	hypothetical protein
bin001	SOY3_bin001_00662	3651	5016	4526	3636	164.245	125.735	105.789	translocation protein TolB
bin001	SOY3_bin001_00663	894	4676	4802	4358	625.291	544.803	517.821	Pyridoxal biosynthesis lyase PdxS
bin001	SOY3_bin001_00664	372	2131	1975	1802	684.835	538.492	514.567	hypothetical protein
bin001	SOY3_bin001_00665	960	1941	2238	2044	241.713	236.452	226.172	Selenocysteine-specific elongation factor
bin001	SOY3_bin001_00666	255	1188	1285	1162	556.957	511.114	484.056	hypothetical protein
bin001	SOY3_bin001_00667	972	4395	4505	4321	540.553	470.092	472.224	Methenyltetrahydromethanopterin cyclohydrolase

bin001	SOY3_bin001_00668	567	2413	2366	2296	508.768	423.240	430.149	hypothetical protein
bin001	SOY3_bin001_00669	777	1179	1306	1153	181.400	170.481	157.630	Lipoprotein-releasing system ATP-binding protein LolD
bin001	SOY3_bin001_00670	1158	1146	1174	1141	118.310	102.829	104.666	Lipoprotein-releasing system transmembrane protein LolE
bin001	SOY3_bin001_00671	1188	948	864	852	95.398	73.765	76.182	Lipoprotein-releasing system transmembrane protein LolE
bin001	SOY3_bin001_00672	636	1264	970	796	237.594	154.693	132.949	hypothetical protein
bin001	SOY3_bin001_00673	1188	2418	2029	1650	243.324	173.229	147.536	hypothetical protein
bin001	SOY3_bin001_00674	1524	5465	6109	5235	428.697	406.574	364.890	2-isopropylmalate synthase
bin001	SOY3_bin001_00675	1800	4836	5593	5043	321.188	315.157	297.609	Acetolactate synthase large subunit IlvB1
bin001	SOY3_bin001_00676	492	1519	1652	1486	369.095	340.565	320.836	Putative acetolactate synthase small subunit
bin001	SOY3_bin001_00677	735	552	837	718	89.784	115.503	103.769	2-oxoglutaramate amidase
bin001	SOY3_bin001_00678	864	474	594	503	65.586	69.731	61.842	Cyclic nucleotide-gated potassium channel
bin001	SOY3_bin001_00679	765	373	349	306	58.290	46.272	42.490	hypothetical protein
bin001	SOY3_bin001_00680	1245	10096	8732	10355	969.450	711.376	883.508	High-affinity heme uptake system protein IsdE precursor
bin001	SOY3_bin001_00681	288	1985	1777	2182	823.974	625.820	804.808	hypothetical protein
bin001	SOY3_bin001_00682	534	651	785	745	145.742	149.102	148.199	riboflavin synthase
bin001	SOY3_bin001_00683	1983	2045	2544	2351	123.287	130.122	125.939	Aerobic respiration control sensor protein ArcB
bin001	SOY3_bin001_00684	1008	5510	5138	5033	653.486	516.997	530.391	hypothetical protein
bin001	SOY3_bin001_00685	498	338	513	483	81.140	104.482	103.026	Rubrerythrin-2
bin001	SOY3_bin001_00686	555	934	1099	957	201.187	200.844	183.168	hypothetical protein
bin001	SOY3_bin001_00687	228	13	27	16	6.816	12.011	7.454	hypothetical protein
bin001	SOY3_bin001_00688	738	1401	1677	1620	226.948	230.479	233.178	Thymidylate synthase
bin001	SOY3_bin001_00689	1014	703	647	556	82.883	64.717	58.246	hypothetical protein
bin001	SOY3_bin001_00690	3573	1952	2508	2089	65.312	71.195	62.106	Autoinducer 2 sensor kinase/phosphatase LuxQ
bin001	SOY3_bin001_00691	474	464	480	453	117.027	102.711	101.520	Methylthioribulose-1-phosphate dehydratase
bin001	SOY3_bin001_00692	1101	1166	1327	1200	126.607	122.247	115.777	Putative zinc metalloprotease Rip3
bin001	SOY3_bin001_00693	1290	2335	2439	2164	216.393	191.768	178.196	TraB family protein
bin001	SOY3_bin001_00694	672	962	1018	929	171.140	153.650	146.851	prephenate dehydrogenase
bin001	SOY3_bin001_00695	1461	2637	2549	2365	215.777	176.960	171.953	coproporphyrinogen III oxidase
bin001	SOY3_bin001_00696	1374	3967	4170	3978	345.160	307.825	307.544	Radical SAM superfamily protein
bin001	SOY3_bin001_00697	309	404	276	287	156.303	90.595	98.663	hypothetical protein
bin001	SOY3_bin001_00698	537	796	624	524	177.208	117.860	103.654	hypothetical protein
bin001	SOY3_bin001_00699	1674	2808	4399	3817	200.534	266.534	242.212	hypothetical protein
bin001	SOY3_bin001_00700	291	377	530	513	154.879	184.730	187.264	hypothetical protein
bin001	SOY3_bin001_00701	252	5248	6104	5946	2489.653	2456.794	2506.422	Cna protein B-type domain protein
bin001	SOY3_bin001_00702	504	534	683	555	126.665	137.450	116.975	hypothetical protein
bin001	SOY3_bin001_00703	1125	561	694	616	59.615	62.569	58.165	Putative oxidoreductase/MT0587
bin001	SOY3_bin001_00704	330	65	82	51	23.548	25.203	16.417	Nitrogen regulatory protein P-II
bin001	SOY3_bin001_00705	1209	124	135	132	12.261	11.326	11.598	Ammonium transporter NrgA
bin001	SOY3_bin001_00706	720	2916	3104	2821	484.173	437.264	416.198	hypothetical protein
bin001	SOY3_bin001_00707	1818	9992	11086	9927	657.059	618.494	580.035	nucleoside triphosphate hydrolase domain-containing protein
bin001	SOY3_bin001_00708	501	1839	1825	1627	438.823	369.471	344.969	hypothetical protein
bin001	SOY3_bin001_00709	696	1905	1637	1381	327.214	238.558	210.773	hypothetical protein
bin001	SOY3_bin001_00710	2634	2308	3359	3209	104.753	129.345	129.415	DNA mismatch repair protein MutS
bin001	SOY3_bin001_00711	1716	1052	1572	1550	73.290	92.916	95.950	DNA mismatch repair protein MutL
bin001	SOY3_bin001_00712	1323	987	1220	1068	89.187	93.531	85.751	Phosphoribosylformylglycinamidine synthase 2
bin001	SOY3_bin001_00713	390	539	1639	1172	165.223	426.255	319.222	hypothetical protein
bin001	SOY3_bin001_00714	624	1866	2159	1791	357.497	350.932	304.888	transcription initiation factor E subunit alpha
bin001	SOY3_bin001_00715	402	751	903	777	223.336	227.833	205.317	hypothetical protein
bin001	SOY3_bin001_00716	306	373	584	534	145.725	193.574	185.374	hypothetical protein
bin001	SOY3_bin001_00717	444	84	101	93	22.617	23.072	22.250	DNA-directed RNA polymerase subunit P
bin001	SOY3_bin001_00718	378	686	1093	942	216.959	293.280	264.721	HTH-type transcriptional repressor CzrA
bin001	SOY3_bin001_00719	762	1037	1097	1245	162.693	146.018	173.558	Teichoic acid translocation permease protein TagG
bin001	SOY3_bin001_00720	825	940	893	1028	136.213	109.787	132.364	Teichoic acids export ATP-binding protein TagH
bin001	SOY3_bin001_00721	435	2357	4417	3330	647.763	1029.895	813.176	Intermediate filament tail domain protein
bin001	SOY3_bin001_00722	1038	341	321	311	39.274	31.366	31.827	hypothetical protein
bin001	SOY3_bin001_00723	249	35	27	19	16.804	10.998	8.106	hypothetical protein
bin001	SOY3_bin001_00724	537	2104	3331	3060	468.400	629.151	605.309	NADH-dependent phenylglyoxylate dehydrogenase subunit gamma
bin001	SOY3_bin001_00725	258	992	1520	1351	459.661	597.556	556.244	Pyruvate synthase subunit PorD
bin001	SOY3_bin001_00726	1218	4974	7251	6768	488.207	603.817	590.259	Pyruvate synthase subunit PorA
bin001	SOY3_bin001_00727	876	3937	6016	5660	537.288	696.560	686.344	Pyruvate synthase subunit PorB
bin001	SOY3_bin001_00728	612	2004	2831	2769	391.464	469.184	480.619	IMP cyclohydrolase
bin001	SOY3_bin001_00729	891	1968	2759	2504	264.054	314.072	298.529	Formyltransferase/hydrolase complex subunit D
bin001	SOY3_bin001_00730	513	572	561	501	133.298	110.918	103.741	hypothetical protein
bin001	SOY3_bin001_00731	702	3845	4676	4247	654.794	675.604	642.651	26 kDa periplasmic immunogenic protein precursor
bin001	SOY3_bin001_00732	699	2400	3214	2966	410.468	466.362	450.738	26 kDa periplasmic immunogenic protein precursor
bin001	SOY3_bin001_00733	798	3064	3826	3658	459.020	486.292	486.935	2-amino-4,5-dihydroxy-6-one-heptanoic acid-7-phosphate synthase
bin001	SOY3_bin001_00734	780	1224	1995	1637	187.600	259.420	222.938	TPR repeat-containing protein YrrB

bin001	SOY3_bin001_00735	447	515	741	617	137.735	168.138	146.625	RDD family protein
bin001	SOY3_bin001_00736	74	43	55	49	69.468	75.385	70.339	tRNA-Thr(ggt)
bin001	SOY3_bin001_00737	1152	2431	2096	1879	252.277	184.541	173.262	Glycogen synthase
bin001	SOY3_bin001_00738	984	839	809	805	101.932	83.389	86.902	Galactose-1-phosphate uridylyltransferase
bin001	SOY3_bin001_00739	1248	1434	1522	1559	137.366	123.696	132.697	Glycogen synthase
bin001	SOY3_bin001_00740	1785	2181	2794	2453	146.071	158.761	145.979	Glycogen synthase
bin001	SOY3_bin001_00741	729	1000	1421	1291	163.990	197.707	188.117	Circadian clock protein kinase KaiC
bin001	SOY3_bin001_00742	1605	1754	2589	2199	130.647	163.611	145.539	replication factor C large subunit
bin001	SOY3_bin001_00743	438	454	801	746	123.916	185.487	180.923	Pyridoxamine 5'-phosphate oxidase
bin001	SOY3_bin001_00744	1491	5049	5475	5142	404.831	372.444	366.340	Biotin carboxylase
bin001	SOY3_bin001_00745	870	1713	1699	1564	235.388	198.075	190.962	Bifunctional ligase/repressor BirA
bin001	SOY3_bin001_00746	417	815	759	656	233.651	184.612	167.108	hypothetical protein
bin001	SOY3_bin001_00747	432	863	696	645	238.821	163.411	158.601	Bifunctional protein HldE
bin001	SOY3_bin001_00748	465	3119	3592	3293	801.877	783.498	752.261	Glycerol-3-phosphate cytidylyltransferase
bin001	SOY3_bin001_00749	1065	4724	5148	5018	530.281	490.279	500.508	NADH dehydrogenase-like protein
bin001	SOY3_bin001_00750	912	358	232	336	46.928	25.802	39.136	hypothetical protein
bin001	SOY3_bin001_00751	114	17	10	17	17.827	8.897	15.841	hypothetical protein
bin001	SOY3_bin001_00752	447	161	135	160	43.059	30.632	38.023	hypothetical protein
bin001	SOY3_bin001_00753	252	90	80	78	42.696	32.199	32.879	hypothetical protein
bin001	SOY3_bin001_00754	450	140	189	151	37.193	42.599	35.645	bifunctional RNase H/acid phosphatase
bin001	SOY3_bin001_00755	1143	1088	1113	938	113.796	98.765	87.174	hypothetical protein
bin001	SOY3_bin001_00756	447	307	206	190	82.106	46.743	45.152	hypothetical protein
bin001	SOY3_bin001_00757	957	1693	1801	1709	211.490	190.878	189.697	Delta-aminolevulinic acid dehydratase
bin001	SOY3_bin001_00758	1146	1299	1677	1608	135.510	148.424	149.050	Glutamyl-tRNA reductase
bin001	SOY3_bin001_00759	861	656	956	675	91.085	112.618	83.278	Anaerobic sulfite reductase subunit C
bin001	SOY3_bin001_00760	987	568	745	606	68.798	76.559	65.221	Daunorubicin/doxorubicin resistance ATP-binding protein DrrA
bin001	SOY3_bin001_00761	732	292	380	297	47.689	52.654	43.100	Inner membrane transport permease YadH
bin001	SOY3_bin001_00762	333	154	184	160	55.287	56.044	51.039	CGGC domain protein
bin001	SOY3_bin001_00763	165	26	38	35	18.838	23.359	22.533	hypothetical protein
bin001	SOY3_bin001_00764	153	4204	4160	3954	3284.860	2757.761	2745.207	30S ribosomal protein S14P
bin001	SOY3_bin001_00765	516	13665	13459	12449	3165.960	2645.561	2562.799	50S ribosomal protein L5
bin001	SOY3_bin001_00766	705	15284	15618	14034	2591.752	2246.938	2114.571	30S ribosomal protein S4e
bin001	SOY3_bin001_00767	360	9867	9780	9171	3276.636	2755.441	2706.101	50S ribosomal protein L24P
bin001	SOY3_bin001_00768	399	9031	9113	8629	2705.880	2316.558	2297.298	50S ribosomal protein L14
bin001	SOY3_bin001_00769	321	6758	6921	6653	2516.858	2186.848	2201.620	30S ribosomal protein S17
bin001	SOY3_bin001_00770	294	9839	9833	9464	4000.821	3392.294	3419.458	ribonuclease P protein component 1
bin001	SOY3_bin001_00771	201	6671	6938	6754	3967.714	3501.007	3569.397	50S ribosomal protein L29
bin001	SOY3_bin001_00772	843	22489	22687	21480	3189.247	2729.633	2706.678	30S ribosomal protein S3
bin001	SOY3_bin001_00773	462	14580	15107	14402	3772.776	3316.584	3311.392	50S ribosomal protein L22
bin001	SOY3_bin001_00774	345	9601	10562	9954	3326.924	3105.145	3064.844	30S ribosomal protein S19
bin001	SOY3_bin001_00775	1122	1995	2349	2226	212.567	212.346	210.747	deoxyguanosinetriphosphate triphosphohydrolase-like protein
bin001	SOY3_bin001_00776	450	1012	1105	1131	268.852	249.060	266.981	hypothetical protein
bin001	SOY3_bin001_00777	552	1040	1124	1124	225.237	206.529	216.300	hypothetical protein
bin001	SOY3_bin001_00778	1350	784	723	738	69.427	54.320	58.070	Hydroxymethylpyrimidine/phosphomethylpyrimidine kinase
bin001	SOY3_bin001_00779	216	1197	1266	1289	662.500	594.477	633.912	30S ribosomal protein S17e
bin001	SOY3_bin001_00780	876	3697	3598	3658	504.535	416.593	443.577	4-hydroxy-tetrahydrodipicolinate synthase
bin001	SOY3_bin001_00781	786	2026	1939	1980	308.150	250.213	267.592	4-hydroxy-tetrahydrodipicolinate reductase
bin001	SOY3_bin001_00782	117	186	272	229	190.052	235.797	207.912	2-ketoisovalerate ferredoxin reductase
bin001	SOY3_bin001_00783	1446	2291	3671	3066	189.410	257.496	225.234	2-oxoglutarate oxidoreductase subunit KorB
bin001	SOY3_bin001_00784	207	117	164	167	67.571	80.358	85.699	hypothetical protein
bin001	SOY3_bin001_00785	396	150	187	189	45.284	47.896	50.699	tRNA(fMet)-specific endonuclease VapC
bin001	SOY3_bin001_00786	225	200	200	188	106.266	90.158	88.758	hypothetical protein
bin001	SOY3_bin001_00787	609	1073	1080	980	210.634	179.871	170.938	CDP-diacylglycerol--inositol 3-phosphatidyltransferase
bin001	SOY3_bin001_00788	1209	2249	2321	2116	222.387	194.717	185.917	Putative N-acetyl-LL-diaminopimelate aminotransferase
bin001	SOY3_bin001_00789	480	1215	1109	1046	302.608	234.339	231.484	Regulatory protein AsnC
bin001	SOY3_bin001_00790	75	13	12	13	20.722	16.228	18.412	tRNA-Thr(tgt)
bin001	SOY3_bin001_00791	819	543	594	493	79.261	73.563	63.943	tRNA 2-thiocytidine biosynthesis protein TtcA
bin001	SOY3_bin001_00792	1038	970	1257	1066	111.717	122.827	109.091	peptide chain release factor 1
bin001	SOY3_bin001_00793	89	821	785	728	1102.805	894.611	868.903	tRNA-Asn(gtt)
bin001	SOY3_bin001_00794	77	191	187	202	296.543	246.323	278.670	tRNA-Met(cat)
bin001	SOY3_bin001_00795	561	1805	2000	1825	384.645	361.595	345.565	DNA topoisomerase 1
bin001	SOY3_bin001_00796	960	2898	3209	2889	360.888	339.042	319.673	hypothetical protein
bin001	SOY3_bin001_00797	894	2477	2345	2194	331.233	266.048	260.693	2-phospho-L-lactate transferase
bin001	SOY3_bin001_00798	570	1951	2156	2018	409.193	383.644	376.076	ZPR1 zinc-finger domain protein
bin001	SOY3_bin001_00799	366	1408	1560	1456	459.904	432.313	422.581	hypothetical protein
bin001	SOY3_bin001_00800	195	757	775	712	464.095	403.108	387.860	hypothetical protein
bin001	SOY3_bin001_00801	1002	1064	907	855	126.946	91.811	90.642	2-iminoacetate synthase

bin001	SOY3_bin001_00802	450	1154	1185	1076	306.576	267.092	253.998	hypothetical protein
bin001	SOY3_bin001_00803	1032	2433	2917	2698	281.843	286.689	277.710	Glycerol-1-phosphate dehydrogenase [NAD(P)+]
bin001	SOY3_bin001_00804	759	1881	2084	1949	296.273	278.491	272.772	Taurine import ATP-binding protein TauB
bin001	SOY3_bin001_00805	825	1826	2097	1867	264.602	257.810	240.392	F420-dependent methylenetetrahydromethanopterin dehydrogenase
bin001	SOY3_bin001_00806	1668	7703	6733	6535	552.089	409.418	416.178	Putative zinc metalloprotease
bin001	SOY3_bin001_00807	543	3386	2710	2694	745.474	506.202	527.021	Polyribonucleotide nucleotidyltransferase
bin001	SOY3_bin001_00808	801	4471	3329	3175	667.295	421.537	421.057	bifunctional UGMP family protein/serine/threonine protein kinase
bin001	SOY3_bin001_00809	417	402	420	488	115.249	102.157	124.312	antiporter inner membrane protein
bin001	SOY3_bin001_00810	276	1406	2351	2591	609.006	863.969	997.214	hypothetical protein
bin001	SOY3_bin001_00811	846	902	957	895	127.462	114.735	112.378	hypothetical protein
bin001	SOY3_bin001_00812	1335	1897	2404	2102	169.876	182.645	167.256	(Dimethylallyl)adenosine tRNA methylthiotransferase MiaB
bin001	SOY3_bin001_00813	1680	126462	145538	131578	8999.042	8786.621	8319.626	hypothetical protein
bin001	SOY3_bin001_00814	768	64755	76761	69344	10079.932	10137.577	9591.300	Methyl-coenzyme M reductase gamma subunit
bin001	SOY3_bin001_00815	504	37112	49777	44322	8802.973	10017.352	9341.543	Methyl-coenzyme M reductase operon protein D
bin001	SOY3_bin001_00816	1305	97329	124765	113600	8916.148	9696.991	9246.931	hypothetical protein
bin001	SOY3_bin001_00817	573	163	145	121	34.008	25.667	22.432	Amylopullulanase precursor
bin001	SOY3_bin001_00818	2085	12	15	14	0.688	0.730	0.713	Prolyl tripeptidyl peptidase precursor
bin001	SOY3_bin001_00819	120	0	0	0	0.000	0.000	0.000	hypothetical protein
bin001	SOY3_bin001_00820	309	21	8	4	8.125	2.626	1.375	hypothetical protein
bin001	SOY3_bin001_00821	408	7	50	8	2.051	12.430	2.083	hypothetical protein
bin001	SOY3_bin001_00822	417	19	168	45	5.447	40.863	11.463	hypothetical protein
bin001	SOY3_bin001_00823	174	6	18	12	4.122	10.492	7.326	hypothetical protein
bin001	SOY3_bin001_00824	516	36	95	31	8.341	18.674	6.382	hypothetical protein
bin001	SOY3_bin001_00825	432	5	8	7	1.384	1.878	1.721	hypothetical protein
bin001	SOY3_bin001_00826	516	36	91	44	8.341	17.887	9.058	hypothetical protein
bin001	SOY3_bin001_00827	564	30	63	23	6.359	11.330	4.332	hypothetical protein
bin001	SOY3_bin001_00828	321	1	4	1	0.372	1.264	0.331	hypothetical protein
bin001	SOY3_bin001_00829	3039	13	27	18	0.511	0.901	0.629	hypothetical protein
bin001	SOY3_bin001_00830	1296	26	30	29	2.398	2.348	2.377	hypothetical protein
bin001	SOY3_bin001_00831	195	2	15	3	1.226	7.802	1.634	hypothetical protein
bin001	SOY3_bin001_00832	636	0	7	2	0.000	1.116	0.334	hypothetical protein
bin001	SOY3_bin001_00833	162	20	84	32	14.759	52.592	20.983	Phosphoglycerate kinase
bin001	SOY3_bin001_00834	714	10	17	6	1.674	2.415	0.893	hypothetical protein
bin001	SOY3_bin001_00835	363	8	6	6	2.635	1.676	1.756	hypothetical protein
bin001	SOY3_bin001_00836	687	478	452	478	83.180	66.732	73.910	hypothetical protein
bin001	SOY3_bin001_00837	684	866	718	714	151.359	106.469	110.885	hypothetical protein
bin001	SOY3_bin001_00838	375	376	378	389	119.868	102.239	110.192	hypothetical protein
bin001	SOY3_bin001_00839	219	49	29	23	26.748	13.431	11.156	hypothetical protein
bin001	SOY3_bin001_00840	354	129	49	108	43.564	14.039	32.408	hypothetical protein
bin001	SOY3_bin001_00841	657	313	190	240	56.954	29.332	38.804	Sporulation initiation inhibitor protein Soj
bin001	SOY3_bin001_00842	561	24	79	69	5.114	14.283	13.065	Benzoylformate decarboxylase
bin001	SOY3_bin001_00843	498	39	86	52	9.362	17.516	11.092	hypothetical protein
bin001	SOY3_bin001_00844	438	273	292	244	74.513	67.618	59.176	Bifunctional ligase/repressor BirA
bin001	SOY3_bin001_00845	2328	1353	2193	1128	69.480	95.546	51.470	Putative membrane protein YdgH
bin001	SOY3_bin001_00846	1053	585	840	425	66.416	80.911	42.874	NPCBM-associated, NEW3 domain of alpha-galactosidase
bin001	SOY3_bin001_00847	405	128	168	67	37.783	42.074	17.573	RDD family protein
bin001	SOY3_bin001_00848	501	161	225	112	38.418	45.551	23.747	MarR family protein
bin001	SOY3_bin001_00849	1209	369	557	296	36.488	46.729	26.007	Glycogen synthase
bin001	SOY3_bin001_00850	1545	515	627	346	39.850	41.162	23.789	Cyclic pyranopterin monophosphate synthase 1
bin001	SOY3_bin001_00851	384	138	163	80	42.963	43.054	22.130	hypothetical protein
bin001	SOY3_bin001_00852	804	74	75	43	11.003	9.461	5.681	Putative glycosyltransferase EpsH
bin001	SOY3_bin001_00853	1209	106	89	57	10.482	7.467	5.008	Protoporphyrinogen oxidase
bin001	SOY3_bin001_00854	1014	96	104	92	11.318	10.403	9.638	hypothetical protein
bin001	SOY3_bin001_00855	381	93	115	94	29.181	30.615	26.208	Transposase DDE domain protein
bin001	SOY3_bin001_00856	693	160	178	132	27.601	26.052	20.233	hypothetical protein
bin001	SOY3_bin001_00857	354	193	249	314	65.178	71.343	94.223	Manganese transport system membrane protein MntB
bin001	SOY3_bin001_00858	450	136	176	230	36.130	39.669	54.293	hypothetical protein
bin001	SOY3_bin001_00859	879	1462	1647	3170	198.840	190.046	383.089	Periplasmic solute binding protein family protein
bin001	SOY3_bin001_00860	417	287	353	450	82.279	85.860	114.632	Aerobic respiration control sensor protein ArcB
bin001	SOY3_bin001_00861	258	301	345	404	139.474	135.629	166.338	hypothetical protein
bin001	SOY3_bin001_00862	171	21	79	53	14.681	46.858	32.924	hypothetical protein
bin001	SOY3_bin001_00863	318	1	3	4	0.376	0.957	1.336	molybdate ABC transporter periplasmic molybdate-binding protein
bin001	SOY3_bin001_00864	894	4	8	9	0.535	0.908	1.069	Molybdenum transport system permease protein ModB
bin001	SOY3_bin001_00865	714	45	61	98	7.535	8.665	14.580	Spermidine/putrescine import ATP-binding protein PotA
bin001	SOY3_bin001_00866	1821	278	278	366	18.251	15.484	21.350	Glycine--tRNA ligase
bin001	SOY3_bin001_00867	507	105	249	98	24.759	49.813	20.533	Imidazole glycerol phosphate synthase subunit HisF
bin001	SOY3_bin001_00868	702	295	312	320	50.238	45.079	48.422	putative type I restriction enzymeP M protein

bin001	SOY3_bin001_00869	1164	170	168	194	17.460	14.639	17.704	Putative type I restriction enzyme specificity protein MPN_638
bin001	SOY3_bin001_00870	513	162	178	179	37.752	35.193	37.065	hypothetical protein
bin001	SOY3_bin001_00871	300	219	238	208	87.271	80.466	73.650	hypothetical protein
bin001	SOY3_bin001_00872	1047	1643	1858	1798	187.602	179.992	182.420	hypothetical protein
bin001	SOY3_bin001_00873	375	155	238	298	49.414	64.373	84.414	hypothetical protein
bin001	SOY3_bin001_00874	270	68	69	40	30.109	25.920	15.737	hypothetical protein
bin001	SOY3_bin001_00875	489	2137	1780	1686	522.446	369.204	366.251	MarR family protein
bin001	SOY3_bin001_00876	1029	5003	4723	4526	581.247	465.540	467.228	hypothetical protein
bin001	SOY3_bin001_00877	915	3352	2813	2764	437.954	311.820	320.883	Phosphoribosylaminoimidazole-succinocarboxamide synthase
bin001	SOY3_bin001_00878	1638	33805	24032	22205	2467.247	1488.095	1440.014	60 kDa chaperonin
bin001	SOY3_bin001_00879	927	49	97	51	6.319	10.613	5.844	hypothetical protein
bin001	SOY3_bin001_00880	300	101	187	86	40.248	63.223	30.451	hypothetical protein
bin001	SOY3_bin001_00881	960	13	16	15	1.619	1.690	1.660	Integrase core domain protein
bin001	SOY3_bin001_00882	153	0	0	1	0.000	0.000	0.694	hypothetical protein
bin001	SOY3_bin001_00883	153	394	472	386	307.858	312.900	267.994	Creatinine amidohydrolase
bin001	SOY3_bin001_00884	189	1192	1290	1235	753.980	692.281	694.121	hypothetical protein
bin001	SOY3_bin001_00885	543	952	892	873	209.596	166.617	170.783	hypothetical protein
bin001	SOY3_bin001_00886	435	1626	2320	2268	446.865	540.945	553.839	hypothetical protein
bin001	SOY3_bin001_00887	1305	4176	5879	6076	382.556	456.928	494.581	Phenylacetate-coenzyme A ligase
bin001	SOY3_bin001_00888	813	38	36	30	5.588	4.491	3.920	hypothetical protein
bin001	SOY3_bin001_00889	1257	25	27	26	2.378	2.179	2.197	hypothetical protein
bin001	SOY3_bin001_00890	390	3	2	1	0.920	0.520	0.272	hypothetical protein
bin001	SOY3_bin001_00891	1455	1033	1548	1355	84.876	107.910	98.925	putative multidrug resistance protein EmrY
bin001	SOY3_bin001_00892	213	64	75	60	35.921	35.714	29.923	hypothetical protein
bin001	SOY3_bin001_00893	225	120	139	154	63.759	62.660	72.706	hypothetical protein
bin001	SOY3_bin001_00894	270	9	7	13	3.985	2.630	5.115	hypothetical protein
bin001	SOY3_bin001_00895	288	11	12	15	4.566	4.226	5.533	SWIM zinc finger
bin001	SOY3_bin001_00896	171	85	123	106	59.425	72.956	65.848	hypothetical protein
bin001	SOY3_bin001_00897	774	364	376	528	56.222	49.272	72.464	hypothetical protein
bin001	SOY3_bin001_00898	588	179	133	163	36.393	22.942	29.447	hypothetical protein
bin001	SOY3_bin001_00899	1362	1491	1373	1806	130.872	102.246	140.854	3-phosphoshikimate 1-carboxyvinyltransferase
bin001	SOY3_bin001_00900	1626	893	774	848	65.656	48.281	55.399	Phosphoenolpyruvate carboxykinase [ATP]
bin001	SOY3_bin001_00901	276	462	530	608	200.114	194.770	234.005	tyrosyl-tRNA synthetase
bin001	SOY3_bin001_00902	1158	227	153	130	23.435	13.401	11.925	hypothetical protein
bin001	SOY3_bin001_00903	1614	409	349	244	30.295	21.932	16.059	Periplasmic copper-binding protein (NosD)
bin001	SOY3_bin001_00904	282	50	22	17	21.197	7.913	6.404	threonine synthase
bin001	SOY3_bin001_00905	360	5	7	6	1.660	1.972	1.770	Sigma-70, region 4
bin001	SOY3_bin001_00906	120	3	0	1	2.989	0.000	0.885	hypothetical protein
bin001	SOY3_bin001_00907	165	8	9	7	5.796	5.532	4.507	hypothetical protein
bin001	SOY3_bin001_00908	936	1911	2329	2600	244.079	252.376	295.072	Oligopeptide transport ATP-binding protein OppD
bin001	SOY3_bin001_00909	801	1358	1627	1773	202.681	206.020	235.129	Oligopeptide transport ATP-binding protein OppF
bin001	SOY3_bin001_00910	1338	1905	1862	2126	170.210	141.149	168.786	hypothetical protein
bin001	SOY3_bin001_00911	135	51	187	94	45.163	140.496	73.965	Carboxymuconolactone decarboxylase family protein
bin001	SOY3_bin001_00912	315	14	12	7	5.313	3.864	2.361	hypothetical protein
bin001	SOY3_bin001_00913	528	24	53	25	5.434	10.181	5.030	Polyketide cyclase / dehydrase and lipid transport
bin001	SOY3_bin001_00914	831	14	26	9	2.014	3.173	1.150	CAAX amino terminal protease self- immunity
bin001	SOY3_bin001_00915	1713	1727	2118	1828	120.526	125.407	113.357	Divergent AAA domain protein
bin001	SOY3_bin001_00916	183	297	360	332	194.022	199.529	192.716	hypothetical protein
bin001	SOY3_bin001_00917	216	19	10	13	10.516	4.696	6.393	Transposase
bin001	SOY3_bin001_00918	3072	2082	1535	1948	81.022	50.681	67.359	ATP-dependent helicase/deoxyribonuclease subunit B
bin001	SOY3_bin001_00919	2988	1184	837	1047	47.371	28.412	37.222	ATP-dependent helicase/nuclease subunit A
bin001	SOY3_bin001_00920	870	183	226	328	25.146	26.348	40.048	hypothetical protein
bin001	SOY3_bin001_00921	579	112	127	173	23.125	22.247	31.739	hypothetical protein
bin001	SOY3_bin001_00922	3648	863	841	1213	28.281	23.383	35.321	hypothetical protein
bin001	SOY3_bin001_00923	606	108	107	130	21.306	17.909	22.788	hypothetical protein
bin001	SOY3_bin001_00924	3819	788	757	1018	24.667	20.105	28.316	N-6 DNA Methylase
bin001	SOY3_bin001_00925	450	83	82	101	22.050	18.482	23.842	hypothetical protein
bin001	SOY3_bin001_00926	2622	707	698	933	32.235	27.001	37.799	antiporter inner membrane protein
bin001	SOY3_bin001_00927	567	273	311	347	57.561	55.633	65.009	hypothetical protein
bin001	SOY3_bin001_00928	2580	882	792	1001	40.869	31.136	41.214	PglZ domain protein
bin001	SOY3_bin001_00929	2028	459	366	516	27.058	18.305	27.028	Lon protease
bin001	SOY3_bin001_00930	444	68	48	80	18.309	10.965	19.140	hypothetical protein
bin001	SOY3_bin001_00931	876	39	17	35	5.322	1.968	4.244	DNA primase TraC
bin001	SOY3_bin001_00932	384	5	1	10	1.557	0.264	2.766	hypothetical protein
bin001	SOY3_bin001_00933	183	0	0	1	0.000	0.000	0.580	hypothetical protein
bin001	SOY3_bin001_00934	234	0	1	0	0.000	0.433	0.000	hypothetical protein
bin001	SOY3_bin001_00935	2949	137	82	136	5.554	2.820	4.899	hypothetical protein

bin001	SOY3_bin001_00936	267	1	0	1	0.448	0.000	0.398	hypothetical protein
bin001	SOY3_bin001_00937	963	181	189	172	22.470	19.906	18.973	Tyrosine recombinase XerC
bin001	SOY3_bin001_00938	510	149	120	172	34.927	23.865	35.825	hypothetical protein
bin001	SOY3_bin001_00939	1260	1522	1337	2087	144.407	107.626	175.947	corrinoid ABC transporter substrate-binding protein
bin001	SOY3_bin001_00940	714	4390	5245	7227	735.041	745.079	1075.201	Nickel uptake substrate-specific transmembrane region
bin001	SOY3_bin001_00941	846	266	332	285	37.589	39.804	35.785	TPR repeat-containing protein YrrB
bin001	SOY3_bin001_00942	357	466	436	377	156.050	123.872	112.177	Glyoxalase-like domain protein
bin001	SOY3_bin001_00943	138	373	536	461	323.129	393.949	354.856	hypothetical protein
bin001	SOY3_bin001_00944	387	36	45	27	11.121	11.794	7.411	Nitrogenase molybdenum-iron protein beta chain
bin001	SOY3_bin001_00945	840	453	1061	2031	64.471	128.112	256.839	Molybdate-binding periplasmic protein precursor
bin001	SOY3_bin001_00946	894	426	733	1070	56.966	83.161	127.138	Molybdenum transport system permease protein ModB
bin001	SOY3_bin001_00947	369	1140	2171	1351	369.338	596.744	388.919	Acetyl-coenzyme A synthetase
bin001	SOY3_bin001_00948	417	194	265	221	55.617	64.456	56.297	CoA binding domain protein
bin001	SOY3_bin001_00949	591	55	232	189	11.126	39.816	33.971	hypothetical protein
bin001	SOY3_bin001_00950	228	1256	1818	1735	658.568	808.749	808.341	hypothetical protein
bin001	SOY3_bin001_00951	327	263	416	450	96.151	129.033	146.182	hypothetical protein
bin001	SOY3_bin001_00952	345	156	203	169	54.057	59.680	52.035	hypothetical protein
bin001	SOY3_bin001_00953	273	51	90	77	22.333	33.438	29.961	hypothetical protein
bin001	SOY3_bin001_00954	558	508	426	511	108.837	77.434	97.278	Chaperone protein YajL
bin001	SOY3_bin001_00955	297	72	66	73	28.982	22.539	26.109	Flagellin N-methylase
bin001	SOY3_bin001_00956	492	428	530	723	103.998	109.261	156.100	SprT-like family protein
bin001	SOY3_bin001_00957	393	1506	2355	2129	458.119	607.789	575.457	Sugar-specific transcriptional regulator TrmB
bin001	SOY3_bin001_00958	180	910	1843	1579	604.386	1038.503	931.836	hypothetical protein
bin001	SOY3_bin001_00959	885	433	525	602	58.491	60.169	72.258	hypothetical protein
bin001	SOY3_bin001_00960	270	8	19	11	3.542	7.137	4.328	hypothetical protein
bin001	SOY3_bin001_00961	507	549	547	453	129.452	109.429	94.912	Formaldehyde-activating enzyme
bin001	SOY3_bin001_00962	546	702	925	933	153.706	171.832	181.518	GMP synthase [glutamine-hydrolyzing]
bin001	SOY3_bin001_00963	828	399	479	478	57.609	58.676	61.324	hypothetical protein
bin001	SOY3_bin001_00964	603	431	561	516	85.449	94.363	90.900	2,3-dimethylmalate dehydratase small subunit
bin001	SOY3_bin001_00965	153	12	28	9	9.376	18.562	6.249	hypothetical protein
bin001	SOY3_bin001_00966	528	666	1017	906	150.795	195.363	182.274	flagellar assembly protein H
bin001	SOY3_bin001_00967	1146	713	1351	905	74.379	119.571	83.887	DOMON domain protein
bin001	SOY3_bin001_00968	861	824	1300	951	114.411	153.142	117.329	ribonuclease Z
bin001	SOY3_bin001_00969	969	333	510	429	41.083	53.383	47.029	molybdenum cofactor biosynthesis protein A
bin001	SOY3_bin001_00970	618	957	925	971	185.127	151.813	166.902	hypothetical protein
bin001	SOY3_bin001_00971	855	1483	1333	1300	207.358	158.132	161.513	NADH-dependent phenylglyoxylate dehydrogenase subunit delta
bin001	SOY3_bin001_00972	864	2677	2631	2635	370.408	308.860	323.964	Benzoyl-CoA oxygenase component A
bin001	SOY3_bin001_00973	360	1395	1627	1357	463.252	458.395	400.412	Dinitrogenase iron-molybdenum cofactor
bin001	SOY3_bin001_00974	480	1802	1921	1893	448.806	405.920	418.928	Dinitrogenase iron-molybdenum cofactor
bin001	SOY3_bin001_00975	1239	784	840	769	75.647	68.764	65.930	putative cation efflux system protein/MT2084
bin001	SOY3_bin001_00976	657	689	904	842	125.372	139.559	136.137	hypothetical protein
bin001	SOY3_bin001_00977	231	72	92	106	37.262	40.395	48.744	Zinc transporter ZupT
bin001	SOY3_bin001_00978	354	179	231	208	60.450	66.186	62.415	hypothetical protein
bin001	SOY3_bin001_00979	996	499	504	593	59.894	51.325	63.245	2-dehydropantoate 2-reductase
bin001	SOY3_bin001_00980	621	187	166	208	35.999	27.113	35.580	Isoprenylcysteine carboxyl methyltransferase (ICMT) family protein
bin001	SOY3_bin001_00981	381	140	157	155	43.929	41.795	43.215	putative spore protein YtfJ
bin001	SOY3_bin001_00982	132	49	74	57	44.378	56.861	45.870	molybdopterin biosynthesis protein MoeB
bin001	SOY3_bin001_00983	507	148	211	154	34.898	42.211	32.266	putative inner membrane protein
bin001	SOY3_bin001_00984	549	60	125	74	13.065	23.094	14.318	putative inner membrane protein
bin001	SOY3_bin001_00985	579	742	1173	1095	153.204	205.482	200.893	hypothetical protein
bin001	SOY3_bin001_00986	393	139	77	76	42.283	19.873	20.542	HTH-type transcriptional activator HxlR
bin001	SOY3_bin001_00987	384	89	66	67	27.708	17.433	18.534	Integral membrane protein TerC family protein
bin001	SOY3_bin001_00988	213	80	55	39	44.901	26.190	19.450	Antitoxin HipB
bin001	SOY3_bin001_00989	246	50	28	27	24.299	11.545	11.659	hypothetical protein
bin001	SOY3_bin001_00990	342	57	38	26	19.925	11.270	8.076	hypothetical protein
bin001	SOY3_bin001_00991	690	135	95	61	23.390	13.965	9.391	HTH DNA binding domain protein
bin001	SOY3_bin001_00992	615	17	17	13	3.305	2.804	2.245	hypothetical protein
bin001	SOY3_bin001_00993	348	56	67	47	19.238	19.528	14.347	hypothetical protein
bin001	SOY3_bin001_00994	1167	106	123	83	10.859	10.690	7.555	Lipoprotein-releasing system transmembrane protein LolE
bin001	SOY3_bin001_00995	1161	166	171	170	17.093	14.939	15.554	Lipoprotein-releasing system transmembrane protein LolE
bin001	SOY3_bin001_00996	708	83	69	71	14.015	9.885	10.653	Lipoprotein-releasing system ATP-binding protein LolD
bin001	SOY3_bin001_00997	297	859	1422	1016	345.766	485.621	363.385	hypothetical protein
bin001	SOY3_bin001_00998	474	29	32	27	7.314	6.847	6.051	Chromosome partition protein Smc
bin001	SOY3_bin001_00999	1323	263	602	376	23.765	46.152	30.190	TPR repeat-containing protein YrrB
bin001	SOY3_bin001_01000	651	53	108	52	9.733	16.827	8.485	Dna-J like membrane chaperone protein
bin001	SOY3_bin001_01001	144	377	237	239	312.986	166.932	176.305	hypothetical protein
bin001	SOY3_bin001_01002	108	2543	3612	3182	2814.933	3392.179	3129.725	hypothetical protein

bin001	SOY3_bin001_01003	150	659	1136	880	525.218	768.143	623.191	hypothetical protein
bin001	SOY3_bin001_01004	648	707	607	523	130.434	95.010	85.735	Flagellin N-methylase
bin001	SOY3_bin001_01005	936	24305	27562	24765	3104.312	2986.686	2810.556	tetrahydromethanopterin S-methyltransferase subunit H
bin001	SOY3_bin001_01006	123	3480	3869	3681	3382.359	3190.424	3179.000	tetrahydromethanopterin S-methyltransferase subunit G
bin001	SOY3_bin001_01007	1338	242	320	267	21.622	24.258	21.198	Deoxyribodipyrimidine photo-lyase
bin001	SOY3_bin001_01008	1020	1072	831	678	125.644	82.633	70.609	Putative NAD(P)H nitroreductase/MT3217
bin001	SOY3_bin001_01009	108	35	25	46	38.743	23.479	45.244	hypothetical protein
bin001	SOY3_bin001_01010	1188	4133	4351	3843	415.905	371.473	343.624	Formaldehyde-activating enzyme
bin001	SOY3_bin001_01011	246	49	33	57	23.813	13.606	24.613	hypothetical protein
bin001	SOY3_bin001_01012	573	122	113	106	25.454	20.002	19.651	hypothetical protein
bin001	SOY3_bin001_01013	741	105	143	101	16.940	19.574	14.479	3-mercaptopyruvate sulfurtransferase
bin001	SOY3_bin001_01014	258	73	91	89	33.826	35.775	36.644	hypothetical protein
bin001	SOY3_bin001_01015	420	81	100	68	23.056	24.149	17.198	HTH-type transcriptional repressor CzrA
bin001	SOY3_bin001_01016	177	40	42	30	27.017	24.067	18.004	hypothetical protein
bin001	SOY3_bin001_01017	408	393	984	983	115.154	244.619	255.931	hypothetical protein
bin001	SOY3_bin001_01018	972	207	271	207	25.459	28.279	22.622	5-methyltetrahydropteroyltriglutamate--homocysteine methyltransferase
bin001	SOY3_bin001_01019	381	177	233	177	55.538	62.028	49.349	Threonine synthase
bin001	SOY3_bin001_01020	834	142	430	249	20.355	52.295	31.715	Transposase IS66 family protein
bin001	SOY3_bin001_01021	1671	67	111	60	4.793	6.738	3.814	Type IIS restriction enzyme Eco57I
bin001	SOY3_bin001_01022	252	13	4	4	6.167	1.610	1.686	hypothetical protein
bin001	SOY3_bin001_01023	312	401	451	372	153.651	146.614	126.654	TM2 domain protein
bin001	SOY3_bin001_01024	1263	1287	1854	1698	121.821	148.888	142.812	Ribonuclease R
bin001	SOY3_bin001_01025	327	18825	24124	23772	6882.287	7482.665	7722.320	F0F1 ATP synthase subunit B
bin001	SOY3_bin001_01026	312	59	49	53	22.607	15.929	18.045	2,3-diketo-5-methylthiopentyl-1-phosphate enolase
bin001	SOY3_bin001_01027	477	41	53	50	10.276	11.270	11.135	Ribulose bisphosphate carboxylase large chain
bin001	SOY3_bin001_01028	453	175	192	180	46.183	42.989	42.209	Virulence factors putative positive transcription regulator BvgA
bin001	SOY3_bin001_01029	780	392	831	1001	60.081	108.059	136.323	ribulose-1,5-biphosphate synthetase
bin001	SOY3_bin001_01030	957	481	737	809	60.087	78.111	89.798	UDP-glucose 4-epimerase
bin001	SOY3_bin001_01031	501	244	294	258	58.223	59.520	54.703	acetyltransferase
bin001	SOY3_bin001_01032	1095	1353	2329	2085	147.717	215.730	202.265	Periplasmic copper-binding protein (NosD)
bin001	SOY3_bin001_01033	153	357	462	370	278.947	306.271	256.886	hypothetical protein
bin001	SOY3_bin001_01034	240	263	291	274	131.006	122.981	121.274	PemK-like protein
bin001	SOY3_bin001_01035	471	76	150	77	19.290	32.302	17.366	hypothetical protein
bin001	SOY3_bin001_01036	621	68	96	63	13.091	15.680	10.777	IS1 transposase
bin001	SOY3_bin001_01037	633	107	103	159	20.208	16.504	26.682	hypothetical protein
bin001	SOY3_bin001_01038	1797	751	706	994	49.962	39.848	58.758	TPR repeat-containing protein YrrB
bin001	SOY3_bin001_01039	3435	2968	3239	4108	103.296	95.640	127.038	Type III restriction enzyme, res subunit
bin001	SOY3_bin001_01040	2094	1061	1394	1403	60.574	67.521	71.172	Modification methylase DpnIIB
bin001	SOY3_bin001_01041	111	2	4	2	2.154	3.655	1.914	hypothetical protein
bin001	SOY3_bin001_01042	627	14	18	10	2.669	2.912	1.694	hypothetical protein
bin001	SOY3_bin001_01043	387	2	2	1	0.618	0.524	0.274	hypothetical protein
bin001	SOY3_bin001_01044	402	1	0	4	0.297	0.000	1.057	hypothetical protein
bin001	SOY3_bin001_01045	357	5	2	3	1.674	0.568	0.893	SWIM zinc finger
bin001	SOY3_bin001_01046	183	4	0	2	2.613	0.000	1.161	Putative nickel-responsive regulator
bin001	SOY3_bin001_01047	75	62	51	45	98.827	68.971	63.735	tRNA-Met(cat)
bin001	SOY3_bin001_01048	294	32	36	29	13.012	12.420	10.478	hypothetical protein
bin001	SOY3_bin001_01049	798	775	464	785	116.103	58.975	104.495	2-amino-4,5-dihydroxy-6-one-heptanoic acid-7-phosphate synthase
bin001	SOY3_bin001_01050	606	103	156	87	20.319	26.110	15.250	hypothetical protein
bin001	SOY3_bin001_01051	471	216	346	329	54.825	74.509	74.200	DNA gyrase inhibitor
bin001	SOY3_bin001_01052	474	958	608	565	241.620	130.101	126.619	hypothetical protein
bin001	SOY3_bin001_01053	222	180	511	342	96.932	233.465	163.645	hypothetical protein
bin001	SOY3_bin001_01054	234	104	194	146	53.133	84.089	66.278	Ribbon-helix-helix protein, copG family
bin001	SOY3_bin001_01055	255	64	117	83	30.004	46.537	34.575	Plasmid stabilisation system protein
bin001	SOY3_bin001_01056	237	21	53	40	10.593	22.682	17.928	FeoA domain protein
bin001	SOY3_bin001_01057	525	1996	2556	2574	454.513	493.806	520.810	pyrroloquinoline quinone biosynthesis protein PqqE
bin001	SOY3_bin001_01058	639	1608	2410	2136	300.837	382.535	355.083	GTPase Era
bin001	SOY3_bin001_01059	345	697	1018	849	241.523	299.284	261.408	hypothetical protein
bin001	SOY3_bin001_01060	417	555	729	622	159.112	177.315	158.447	hypothetical protein
bin001	SOY3_bin001_01061	153	976	1369	1250	762.612	907.542	867.857	hypothetical protein
bin001	SOY3_bin001_01062	411	2441	3365	3267	710.021	830.420	844.378	hypothetical protein
bin001	SOY3_bin001_01063	102	1563	4799	3840	1831.911	4772.054	3999.087	hypothetical protein
bin001	SOY3_bin001_01064	867	8193	6806	7154	1129.716	796.210	876.516	Immunoglobulin-binding protein sbi precursor
bin001	SOY3_bin001_01065	228	24390	41923	42548	12788.585	18649.718	19823.216	hypothetical protein
bin001	SOY3_bin001_01066	1413	2188	2734	2479	185.119	196.251	186.365	Cobyrinic acid A,C-diamide synthase
bin001	SOY3_bin001_01067	420	164	198	168	46.681	47.816	42.490	hypothetical protein
bin001	SOY3_bin001_01068	924	199	224	206	25.747	24.588	23.682	Carbamate kinase 1
bin001	SOY3_bin001_01069	1044	829	910	916	94.929	88.409	93.202	RNA 3'-terminal phosphate cyclase

bin001	SOY3_bin001_01070	1260	606	673	676	57.497	54.175	56.991	Bicyclomycin resistance protein
bin001	SOY3_bin001_01071	396	251	354	433	75.775	90.670	116.151	p-benzoquinone reductase
bin001	SOY3_bin001_01072	594	1849	1718	1571	372.131	293.354	280.944	hypothetical protein
bin001	SOY3_bin001_01073	993	1456	1114	1015	175.290	113.786	108.579	triphosphoribosyl-dephospho-CoA synthase
bin001	SOY3_bin001_01074	600	845	820	781	168.365	138.617	138.271	hypothetical protein
bin001	SOY3_bin001_01075	672	909	757	803	161.711	114.257	126.933	hypothetical protein
bin001	SOY3_bin001_01076	417	469	451	434	134.457	109.697	110.556	Mycothiol acetyltransferase
bin001	SOY3_bin001_01077	1710	2627	3271	3231	183.658	194.017	200.711	Long-chain-fatty-acid--CoA ligase
bin001	SOY3_bin001_01078	255	3	4	5	1.406	1.591	2.083	hypothetical protein
bin001	SOY3_bin001_01079	759	68	124	107	10.711	16.570	14.975	hypothetical protein
bin001	SOY3_bin001_01080	2934	3401	4236	4447	138.577	146.437	161.004	hypothetical protein
bin001	SOY3_bin001_01081	843	1807	1800	2292	256.257	216.571	288.813	hypothetical protein
bin001	SOY3_bin001_01082	165	75	89	94	54.340	54.709	60.516	hypothetical protein
bin001	SOY3_bin001_01083	747	282	259	359	45.131	35.167	51.051	hypothetical protein
bin001	SOY3_bin001_01084	1584	4909	6397	6211	370.496	409.615	416.520	D-3-phosphoglycerate dehydrogenase
bin001	SOY3_bin001_01085	693	809	818	761	139.560	119.722	116.649	Iron-sulfur flavoprotein
bin001	SOY3_bin001_01086	555	800	863	831	172.323	157.715	159.052	NYN domain protein
bin001	SOY3_bin001_01087	942	113	134	152	14.341	14.428	17.140	PD-(D/E)XK nuclease superfamily protein
bin001	SOY3_bin001_01088	612	177	245	275	34.575	40.604	47.732	ATP-dependent helicase/nuclease subunit A
bin001	SOY3_bin001_01089	279	112	170	213	47.991	61.802	81.097	CRISPR-associated endoribonuclease Cas2
bin001	SOY3_bin001_01090	1014	466	910	938	54.941	91.024	98.264	CRISPR-associated endonuclease Cas1
bin001	SOY3_bin001_01091	2403	509	759	833	25.323	32.036	36.823	CRISPR-associated endonuclease/helicase Cas3
bin001	SOY3_bin001_01092	1719	382	549	623	26.566	32.393	38.498	hypothetical protein
bin001	SOY3_bin001_01093	960	370	497	554	46.076	52.510	61.301	CRISPR-associated negative autoregulator DevR/Csa2
bin001	SOY3_bin001_01094	744	148	145	199	23.781	19.767	28.413	hypothetical protein
bin001	SOY3_bin001_01095	1143	951	1377	1214	99.467	122.192	112.824	Glycogen phosphorylase
bin001	SOY3_bin001_01096	336	329	393	314	117.058	118.634	99.270	hypothetical protein
bin001	SOY3_bin001_01097	798	263	450	400	39.400	57.196	53.246	hypothetical protein
bin001	SOY3_bin001_01098	420	239	438	422	68.029	105.774	106.732	Sporulation protein YtfJ (Spore_YtfJ)
bin001	SOY3_bin001_01099	957	1211	1885	1664	151.279	199.781	184.702	ATPase RavA
bin001	SOY3_bin001_01100	447	108	85	100	28.884	19.287	23.764	hypothetical protein
bin001	SOY3_bin001_01101	1215	3262	3157	3737	320.962	263.544	326.721	FeS cluster assembly protein SufB
bin001	SOY3_bin001_01102	735	1701	1709	2126	276.670	235.836	307.260	putative ABC transporter ATP-binding protein
bin001	SOY3_bin001_01103	642	302	278	338	56.236	43.920	55.926	hypothetical protein
bin001	SOY3_bin001_01104	324	187	169	226	68.999	52.905	74.096	Diaminopimelate decarboxylase
bin001	SOY3_bin001_01105	678	345	1149	775	60.832	171.888	121.423	hypothetical protein
bin001	SOY3_bin001_01106	345	163	536	380	56.483	157.580	117.002	hypothetical protein
bin001	SOY3_bin001_01107	423	182	244	323	51.437	58.507	81.113	hypothetical protein
bin001	SOY3_bin001_01108	1140	1066	777	892	111.789	69.131	83.117	Adenosine monophosphate-protein transferase SoFic
bin001	SOY3_bin001_01109	2394	7701	5731	5512	384.564	242.807	244.577	DNA gyrase subunit A
bin001	SOY3_bin001_01110	1899	7760	5767	5114	488.520	308.021	286.066	DNA gyrase subunit B
bin001	SOY3_bin001_01111	72	3	12	12	4.981	16.905	17.704	tRNA-Cys(gca)
bin001	SOY3_bin001_01112	321	16	12	17	5.959	3.792	5.626	(Dimethylallyl)adenosine tRNA methylthiotransferase MiaB
bin001	SOY3_bin001_01113	360	82	42	46	27.231	11.833	13.573	hypothetical protein
bin001	SOY3_bin001_01114	912	647	557	708	84.812	61.946	82.465	hypothetical protein
bin001	SOY3_bin001_01115	468	327	272	354	83.531	58.949	80.350	hypothetical protein
bin001	SOY3_bin001_01116	798	593	486	597	88.838	61.771	79.470	Corrinoid/iron-sulfur protein large subunit
bin001	SOY3_bin001_01117	363	303	237	338	99.789	66.221	98.910	sulfur transfer complex subunit TusD
bin001	SOY3_bin001_01118	342	205	153	195	71.659	45.375	60.567	Intracellular sulfur oxidation protein DsrF
bin001	SOY3_bin001_01119	297	82	69	84	33.007	23.564	30.044	DsrH like protein
bin001	SOY3_bin001_01120	2193	249	250	291	13.574	11.563	14.096	CHAT domain protein
bin001	SOY3_bin001_01121	1644	175	156	193	12.726	9.624	12.471	hypothetical protein
bin001	SOY3_bin001_01122	771	1643	2255	2036	254.759	296.652	280.513	Ribosomal RNA large subunit methyltransferase E
bin001	SOY3_bin001_01123	282	1428	1582	1743	605.375	569.000	656.566	DNA/RNA-binding protein albA
bin001	SOY3_bin001_01124	1143	1000	1202	1316	104.592	106.663	122.304	Nuclease SbcCD subunit D
bin001	SOY3_bin001_01125	1599	1430	1755	1996	106.914	111.323	132.600	AAA-like domain protein
bin001	SOY3_bin001_01126	387	313	404	428	96.689	105.883	117.480	NurA domain protein
bin001	SOY3_bin001_01127	3297	1409	1941	1738	51.090	59.712	55.996	Regulatory protein AfsR
bin001	SOY3_bin001_01128	192	292	405	385	181.814	213.948	213.005	hypothetical protein
bin001	SOY3_bin001_01129	1479	1690	2656	1905	136.604	182.144	136.822	Dictyostelium (slime mold) repeat protein
bin001	SOY3_bin001_01130	444	294	413	269	79.161	94.346	64.357	hypothetical protein
bin001	SOY3_bin001_01131	1608	4814	5347	4820	357.903	337.271	318.413	AAA-like domain protein
bin001	SOY3_bin001_01132	219	170	668	429	92.801	309.376	208.086	hypothetical protein
bin001	SOY3_bin001_01133	1398	774	803	717	66.188	58.259	54.481	Polyketide biosynthesis 3-hydroxy-3-methylglutaryl-ACP synthase PksG
bin001	SOY3_bin001_01134	237	33	16	15	16.646	6.847	6.723	hypothetical protein
bin001	SOY3_bin001_01135	468	29	12	9	7.408	2.601	2.043	hypothetical protein
bin001	SOY3_bin001_01136	252	465	526	579	220.596	211.709	244.066	hypothetical protein

bin001	SOY3_bin001_01137	888	1308	1489	1652	176.092	170.073	197.618	Dihydroorotate dehydrogenase B (NAD(+)), electron transfer subunit
bin001	SOY3_bin001_01138	96	7	7	5	8.717	7.396	5.533	AMP-binding domain protein
bin001	SOY3_bin001_01139	1395	1339	1617	1614	114.750	117.568	122.902	D-tagatose-1,6-bisphosphate aldolase subunit KbaY
bin001	SOY3_bin001_01140	939	955	1488	1417	121.586	160.728	160.300	Fructose-1,6-bisphosphatase class 1
bin001	SOY3_bin001_01141	222	87	96	82	46.850	43.860	39.237	Ribulose bisphosphate carboxylase large chain
bin001	SOY3_bin001_01142	543	170	179	184	37.428	33.436	35.995	Ribulose bisphosphate carboxylase large chain 1
bin001	SOY3_bin001_01143	186	321	348	326	206.318	189.767	186.181	NADP-dependent malic enzyme
bin001	SOY3_bin001_01144	984	398	420	373	48.354	43.292	40.266	Homoisocitrate dehydrogenase
bin001	SOY3_bin001_01145	828	153	290	158	22.091	35.524	20.270	hypothetical protein
bin001	SOY3_bin001_01146	603	96	119	143	19.033	20.016	25.191	2,3-dimethylmalate dehydratase small subunit
bin001	SOY3_bin001_01147	273	37	80	77	16.203	29.722	29.961	hypothetical protein
bin001	SOY3_bin001_01148	594	477	749	743	96.001	127.894	132.872	Iron-sulfur flavoprotein
bin001	SOY3_bin001_01149	1116	2028	2525	2756	217.245	229.484	262.328	hypothetical protein
bin001	SOY3_bin001_01150	363	1420	1429	1418	467.657	399.283	414.953	hypothetical protein
bin001	SOY3_bin001_01151	201	319	460	850	189.732	232.122	449.213	H/ACA RNA-protein complex component Cbf5p
bin001	SOY3_bin001_01152	726	562	683	1091	92.543	95.420	159.631	Oligopeptide transport ATP-binding protein OppF
bin001	SOY3_bin001_01153	933	964	1053	1749	123.521	114.473	199.131	Oligopeptide transport ATP-binding protein OppD
bin001	SOY3_bin001_01154	900	867	1016	1649	115.165	114.500	194.629	Glutathione transport system permease protein GsiD
bin001	SOY3_bin001_01155	942	1279	1396	2165	162.317	150.310	244.139	Nickel transport system permease protein NikB
bin001	SOY3_bin001_01156	438	1415	1389	2272	386.214	321.650	551.016	putative D,D-dipeptide-binding periplasmic protein DdpA precursor
bin001	SOY3_bin001_01157	1806	592	704	818	39.188	39.538	48.113	Type-1 restriction enzyme R protein
bin001	SOY3_bin001_01158	480	135	132	130	33.623	27.893	28.769	hypothetical protein
bin001	SOY3_bin001_01159	291	280	385	387	115.030	134.191	141.269	hypothetical protein
bin001	SOY3_bin001_01160	222	165	247	280	88.854	112.849	133.978	YcfA-like protein
bin001	SOY3_bin001_01161	1215	1015	1161	1056	99.870	96.919	92.325	Inner membrane transport protein YajR
bin001	SOY3_bin001_01162	450	728	1076	941	193.404	242.524	222.130	Spore protein SP21
bin001	SOY3_bin001_01163	201	59	74	86	35.091	37.341	45.450	hypothetical protein
bin001	SOY3_bin001_01164	846	2069	1771	1758	292.372	212.326	220.739	Nickel import ATP-binding protein NikO
bin001	SOY3_bin001_01165	939	3908	4035	4430	497.547	435.846	501.150	Nickel transport protein NikQ
bin001	SOY3_bin001_01166	315	1772	1932	2178	672.510	622.087	734.475	Fused nickel transport protein NikMN
bin001	SOY3_bin001_01167	720	3593	3936	4140	596.582	554.469	610.798	Fused nickel transport protein NikMN
bin001	SOY3_bin001_01168	228	254	281	272	133.182	125.005	126.725	hypothetical protein
bin001	SOY3_bin001_01169	255	364	399	352	170.650	158.704	146.633	hypothetical protein
bin001	SOY3_bin001_01170	933	973	990	923	124.674	107.624	105.087	Methylthioribose-1-phosphate isomerase
bin001	SOY3_bin001_01171	474	1195	1462	1368	301.394	312.841	306.576	phosphoribosylformylglycinamidine synthase subunit PurS
bin001	SOY3_bin001_01172	810	1775	2433	2167	261.974	304.657	284.187	Phosphoribosylformylglycinamidine synthase 1
bin001	SOY3_bin001_01173	2367	6060	9118	7875	306.069	390.711	353.413	Phosphoribosylformylglycinamidine synthase 2
bin001	SOY3_bin001_01174	282	230	1026	611	97.504	369.023	230.156	hypothetical protein
bin001	SOY3_bin001_01175	264	23	35	48	10.415	13.447	19.314	hypothetical protein
bin001	SOY3_bin001_01176	753	1285	1947	1401	204.011	262.256	197.639	TPR repeat-containing protein YrrB
bin001	SOY3_bin001_01177	303	1099	1421	1269	433.611	475.670	444.886	hypothetical protein
bin001	SOY3_bin001_01178	576	1540	1321	1414	319.627	232.614	260.769	Rubrerythrin
bin001	SOY3_bin001_01179	1470	1675	1450	1365	136.221	100.047	98.638	Catalase
bin001	SOY3_bin001_01180	798	385	303	313	57.677	38.512	41.665	putative transposase
bin001	SOY3_bin001_01181	165	23	22	22	16.664	13.524	14.163	Dipeptide transport system permease protein DppC
bin001	SOY3_bin001_01182	705	550	757	706	93.265	108.908	106.376	Acylphosphatase
bin001	SOY3_bin001_01183	1266	1014	1106	1013	95.752	88.609	84.997	Anaerobic sulfite reductase subunit C
bin001	SOY3_bin001_01184	177	198	459	469	133.733	263.023	281.468	Pyruvate synthase subunit PorD
bin001	SOY3_bin001_01185	663	758	2110	2061	136.679	322.793	330.213	indolepyruvate oxidoreductase subunit beta
bin001	SOY3_bin001_01186	1284	1373	3177	3159	127.835	250.961	261.345	Phenylacetate-coenzyme A ligase
bin001	SOY3_bin001_01187	1167	1911	3562	2867	195.765	309.584	260.968	hypothetical protein
bin001	SOY3_bin001_01188	789	6467	6280	6649	979.877	807.305	895.177	putative siderophore transport system ATP-binding protein YusV
bin001	SOY3_bin001_01189	255	430	587	698	201.592	233.482	290.767	hypothetical protein
bin001	SOY3_bin001_01190	735	1178	1669	1965	191.604	230.316	283.991	Ferrous iron transport protein B
bin001	SOY3_bin001_01191	264	428	555	723	193.814	213.228	290.914	FeoA domain protein
bin001	SOY3_bin001_01192	237	356	557	587	179.576	238.375	263.099	Ferrous iron transport protein A
bin001	SOY3_bin001_01193	558	127	125	133	27.209	22.721	25.319	hypothetical protein
bin001	SOY3_bin001_01194	891	332	246	239	44.546	28.003	28.494	hypothetical protein
bin001	SOY3_bin001_01195	594	203	249	211	40.856	42.517	37.733	Plasmid pRiA4b ORF-3-like protein
bin001	SOY3_bin001_01196	207	100	122	105	57.753	59.778	53.883	hypothetical protein
bin001	SOY3_bin001_01197	234	225	233	220	114.951	100.994	99.870	Antitoxin HicB
bin001	SOY3_bin001_01198	228	132	120	94	69.213	53.383	43.795	YcfA-like protein
bin001	SOY3_bin001_01199	141	183	241	245	155.159	173.362	184.577	hypothetical protein
bin001	SOY3_bin001_01200	846	1096	1351	1252	154.877	161.972	157.204	succinate dehydrogenase/fumarate reductase iron-sulfur subunit
bin001	SOY3_bin001_01201	510	39	52	65	9.142	10.342	13.539	hypothetical protein
bin001	SOY3_bin001_01202	555	81	92	102	17.448	16.813	19.523	hypothetical protein
bin001	SOY3_bin001_01203	117	897	1411	1376	916.541	1223.196	1249.287	hypothetical protein

bin001	SOY3_bin001_01204	612	769	994	862	150.217	164.736	149.619	Ribbon-helix-helix protein, copG family
bin001	SOY3_bin001_01205	504	236	301	312	55.979	60.575	65.759	hypothetical protein
bin001	SOY3_bin001_01206	603	522	507	515	103.490	85.280	90.723	hypothetical protein
bin001	SOY3_bin001_01207	858	254	346	360	35.391	40.902	44.570	hypothetical protein
bin001	SOY3_bin001_01208	432	202	205	193	55.900	48.131	47.457	hypothetical protein
bin001	SOY3_bin001_01209	1050	107	166	163	12.183	16.035	16.490	MarR family protein
bin001	SOY3_bin001_01210	324	261	310	303	96.303	97.045	99.341	Helix-turn-helix domain protein
bin001	SOY3_bin001_01211	168	460	669	651	327.336	403.898	411.625	Putative nickel-responsive regulator
bin001	SOY3_bin001_01212	1053	2335	2299	2258	265.097	221.445	227.785	tRNA splicing endonuclease
bin001	SOY3_bin001_01213	651	3025	3745	3504	555.507	583.479	571.759	glyceraldehyde-3-phosphate dehydrogenase
bin001	SOY3_bin001_01214	357	985	1103	1063	329.848	313.373	316.297	hypothetical protein
bin001	SOY3_bin001_01215	1092	3370	4328	3889	368.937	401.994	378.308	coenzyme F420-reducing hydrogenase subunit beta
bin001	SOY3_bin001_01216	1026	1052	1407	1371	122.578	139.092	141.945	Hydantoinase/oxoprolinase
bin001	SOY3_bin001_01217	1140	147	401	363	15.416	35.677	33.825	Anthranilate phosphoribosyltransferase 2
bin001	SOY3_bin001_01218	1104	156	842	901	16.893	77.357	86.693	Putative L-galactonate oxidoreductase
bin001	SOY3_bin001_01219	1041	180	1180	1373	20.671	114.970	140.104	hypothetical protein
bin001	SOY3_bin001_01220	753	3559	3099	2572	565.039	417.428	362.832	hypothetical protein
bin001	SOY3_bin001_01221	216	83	121	92	45.938	56.818	45.244	hypothetical protein
bin001	SOY3_bin001_01222	747	183	409	180	29.287	55.534	25.597	hypothetical protein
bin001	SOY3_bin001_01223	210	63	88	77	35.865	42.503	38.949	hypothetical protein
bin001	SOY3_bin001_01224	843	902	1893	832	127.916	227.760	104.840	hypothetical protein
bin001	SOY3_bin001_01225	669	1607	1415	1760	287.168	214.529	279.458	ATP-binding region
bin001	SOY3_bin001_01226	873	28201	34296	37928	3861.853	3984.593	4615.041	hypothetical protein
bin001	SOY3_bin001_01227	504	310	359	335	73.532	72.247	70.606	hypothetical protein
bin001	SOY3_bin001_01228	702	102	137	131	17.370	19.794	19.823	GMP synthase [glutamine-hydrolyzing]
bin001	SOY3_bin001_01229	438	65	81	67	17.741	18.757	16.249	hypothetical protein
bin001	SOY3_bin001_01230	1083	1084	1496	1312	119.659	140.106	128.687	Cell division protein FtsZ
bin001	SOY3_bin001_01231	96	76	84	75	94.643	88.749	82.989	hypothetical protein
bin001	SOY3_bin001_01232	1260	1346	1505	1371	127.709	121.149	115.584	3-octaprenyl-4-hydroxybenzoate carboxy-lyase
bin001	SOY3_bin001_01233	702	495	592	421	84.297	85.534	63.705	hypothetical protein
bin001	SOY3_bin001_01234	1122	1305	1839	1628	139.047	166.243	154.131	3-amino-4-hydroxybenzoic acid synthase
bin001	SOY3_bin001_01235	645	534	648	663	98.975	101.899	109.190	3-dehydroquinate dehydratase
bin001	SOY3_bin001_01236	1344	276	360	341	24.550	27.168	26.952	Radical SAM superfamily protein
bin001	SOY3_bin001_01237	336	567	617	597	201.739	186.252	188.740	hypothetical protein
bin001	SOY3_bin001_01238	213	305	356	343	171.185	169.522	171.058	hypothetical protein
bin001	SOY3_bin001_01239	576	105	173	123	21.793	30.463	22.684	hypothetical protein
bin001	SOY3_bin001_01240	213	78	86	71	43.778	40.952	35.409	hypothetical protein
bin001	SOY3_bin001_01241	2352	451	457	628	22.924	19.708	28.363	hypothetical protein
bin001	SOY3_bin001_01242	2478	678	888	917	32.710	36.347	39.310	Type IIS restriction enzyme Eco57I
bin001	SOY3_bin001_01243	360	201	258	213	66.748	72.690	62.850	hypothetical protein
bin001	SOY3_bin001_01244	720	1251	1543	1404	207.716	217.364	207.140	Helix-turn-helix domain protein
bin001	SOY3_bin001_01245	1038	2316	2581	2519	266.739	252.200	257.787	3-oxoacyl-[acyl-carrier-protein] synthase 3
bin001	SOY3_bin001_01246	1164	1560	1661	1584	160.220	144.734	144.555	Beta-ketoadipyl-CoA thiolase
bin001	SOY3_bin001_01247	444	350	360	350	94.239	82.238	83.737	hypothetical protein
bin001	SOY3_bin001_01248	201	279	366	308	165.941	184.688	162.774	hypothetical protein
bin001	SOY3_bin001_01249	861	865	1199	797	120.104	141.244	98.330	hypothetical protein
bin001	SOY3_bin001_01250	873	3988	5324	5289	546.118	618.555	643.560	hypothetical protein
bin001	SOY3_bin001_01251	1068	1903	2391	2120	213.016	227.072	210.860	hypothetical protein
bin001	SOY3_bin001_01252	150	118	100	122	94.045	67.618	86.397	hypothetical protein
bin001	SOY3_bin001_01253	435	305	375	296	83.822	87.437	72.282	hypothetical protein
bin001	SOY3_bin001_01254	846	1271	1514	1360	179.606	181.514	170.765	Guanine deaminase
bin001	SOY3_bin001_01255	1173	283	245	242	28.843	21.185	21.915	6-phosphogluconolactonase
bin001	SOY3_bin001_01256	74	17	26	18	27.464	35.637	25.839	tRNA-His(gtg)
bin001	SOY3_bin001_01257	1548	542	680	688	41.858	44.555	47.211	hypothetical protein
bin001	SOY3_bin001_01258	765	373	568	570	58.290	75.308	79.149	Ferredoxin-dependent glutamate synthase 2
bin001	SOY3_bin001_01259	1257	891	1402	1390	84.740	113.127	117.465	ATPase family associated with various cellular activities (AAA)
bin001	SOY3_bin001_01260	315	142	171	163	53.892	55.061	54.968	hypothetical protein
bin001	SOY3_bin001_01261	636	921	872	852	173.120	139.064	142.302	Acylphosphatase
bin001	SOY3_bin001_01262	2277	7174	10769	8978	376.655	479.697	418.838	ATP-dependent zinc metalloprotease FtsH
bin001	SOY3_bin001_01263	456	759	841	752	198.986	187.062	175.179	Acetyltransferase (GNAT) family protein
bin001	SOY3_bin001_01264	909	1861	1707	1575	244.753	190.469	184.055	hypothetical protein
bin001	SOY3_bin001_01265	282	49	73	56	20.773	26.256	21.094	hypothetical protein
bin001	SOY3_bin001_01266	924	650	731	727	84.098	80.242	83.578	Methyl-coenzyme M reductase operon protein C
bin001	SOY3_bin001_01267	453	1159	1529	1629	305.866	342.345	381.991	Alkyl hydroperoxide reductase AhpD
bin001	SOY3_bin001_01268	321	217	124	188	80.817	39.181	62.213	Bacterial regulatory protein, arsR family
bin001	SOY3_bin001_01269	219	133	83	106	72.603	38.440	51.415	hypothetical protein
bin001	SOY3_bin001_01270	312	66	38	38	25.289	12.353	12.938	hypothetical protein

bin001	SOY3_bin001_01271	2238	375	238	228	20.032	10.786	10.822	Leupeptin-inactivating enzyme 1 precursor
bin001	SOY3_bin001_01272	453	480	657	588	126.674	147.103	137.882	FKBP-type peptidyl-prolyl cis-trans isomerase SlyD
bin001	SOY3_bin001_01273	1137	2563	3594	4148	269.484	320.607	387.532	Putative transposase DNA-binding domain protein
bin001	SOY3_bin001_01274	843	479	615	605	67.929	73.995	76.236	hypothetical protein
bin001	SOY3_bin001_01275	564	278	269	255	58.927	48.376	48.028	hypothetical protein
bin001	SOY3_bin001_01276	834	482	662	577	69.092	80.509	73.492	Sulfite exporter TauE/SafE
bin001	SOY3_bin001_01277	744	710	1139	920	114.086	155.276	131.354	Nitric oxide reductase
bin001	SOY3_bin001_01278	165	34	35	22	24.634	21.515	14.163	hypothetical protein
bin001	SOY3_bin001_01279	138	5	2	6	4.331	1.470	4.619	hypothetical protein
bin001	SOY3_bin001_01280	144	15	9	18	12.453	6.339	13.278	hypothetical protein
bin001	SOY3_bin001_01281	189	106	121	175	67.049	64.935	98.357	hypothetical protein
bin001	SOY3_bin001_01282	1587	1020	1092	1474	76.837	69.791	98.662	Sodium/proline symporter
bin001	SOY3_bin001_01283	1221	2294	2197	1965	224.607	182.503	170.953	putative transposase
bin001	SOY3_bin001_01284	1113	520	503	585	55.854	45.838	55.833	Putative NAD(P)H nitroreductase/MT3217
bin001	SOY3_bin001_01285	213	101	182	130	56.687	86.666	64.833	hypothetical protein
bin001	SOY3_bin001_01286	759	97	168	82	15.278	22.450	11.476	hypothetical protein
bin001	SOY3_bin001_01287	339	176	223	157	62.067	66.721	49.196	hypothetical protein
bin001	SOY3_bin001_01288	129	30	27	25	27.802	21.229	20.586	hypothetical protein
bin001	SOY3_bin001_01289	159	275	298	228	206.767	190.096	152.324	translation-associated GTPase
bin001	SOY3_bin001_01290	390	236	381	332	72.342	99.087	90.428	hypothetical protein
bin001	SOY3_bin001_01291	375	59	96	49	18.809	25.965	13.880	hypothetical protein
bin001	SOY3_bin001_01292	282	20	60	21	8.479	21.580	7.910	Aspartyl/glutamyl-tRNA(Asn/Gln) amidotransferase subunit C
bin001	SOY3_bin001_01293	1341	336	566	295	29.954	42.810	23.368	Hydroxyneurosporene desaturase
bin001	SOY3_bin001_01294	324	85	49	70	31.363	15.339	22.950	hypothetical protein
bin001	SOY3_bin001_01295	1056	919	1205	922	104.039	115.739	92.746	hypothetical protein
bin001	SOY3_bin001_01296	444	5976	9951	8472	1609.063	2273.205	2026.902	hypothetical protein
bin001	SOY3_bin001_01297	402	68	44	68	20.222	11.101	17.969	LOG family protein ORF6 in fasciation locus
bin001	SOY3_bin001_01298	1131	778	883	751	82.236	79.187	70.535	Hydroxyneurosporene synthase (CrtC)
bin001	SOY3_bin001_01299	330	107	111	94	38.763	34.116	30.258	hypothetical protein
bin001	SOY3_bin001_01300	249	49	73	58	23.526	29.736	24.743	Zinc finger found in FPG and IleRS
bin001	SOY3_bin001_01301	258	413	508	487	191.371	199.710	200.511	hypothetical protein
bin001	SOY3_bin001_01302	3543	1266	1297	1421	42.718	37.130	42.604	Endoglucanase C precursor
bin001	SOY3_bin001_01303	240	295	260	253	146.946	109.880	111.980	hypothetical protein
bin001	SOY3_bin001_01304	579	192	198	190	39.643	34.685	34.858	Pyruvate kinase, alpha/beta domain
bin001	SOY3_bin001_01305	147	20	21	9	16.265	14.490	6.504	hypothetical protein
bin001	SOY3_bin001_01306	513	137	194	149	31.926	38.357	30.853	NADH dehydrogenase
bin001	SOY3_bin001_01307	579	227	284	222	46.870	49.750	40.729	hypothetical protein
bin001	SOY3_bin001_01308	324	226	179	199	83.389	56.035	65.244	hypothetical protein
bin001	SOY3_bin001_01309	441	37	58	66	10.030	13.340	15.898	Polyketide cyclase / dehydrase and lipid transport
bin001	SOY3_bin001_01310	522	324	374	336	74.203	72.670	68.375	Aspartate carbamoyltransferase regulatory chain
bin001	SOY3_bin001_01311	927	607	816	678	78.281	89.282	77.693	Aspartate carbamoyltransferase catalytic chain
bin001	SOY3_bin001_01312	858	2169	2669	2542	302.216	315.512	314.715	Universal stress protein/MSMEI_3859
bin001	SOY3_bin001_01313	1287	1159	1111	1113	107.659	87.557	91.864	Sodium:neurotransmitter symporter family protein
bin001	SOY3_bin001_01314	1686	870	972	967	61.689	58.474	60.925	Na+/Pi-cotransporter
bin001	SOY3_bin001_01315	501	169	269	240	40.327	54.459	50.887	hypothetical protein
bin001	SOY3_bin001_01316	246	73	124	111	35.476	51.126	47.931	hypothetical protein
bin001	SOY3_bin001_01317	900	1026	1234	1130	136.286	139.068	133.372	Dihydroorotate dehydrogenase B (NAD(+)), catalytic subunit
bin001	SOY3_bin001_01318	279	2447	2180	2046	1048.517	792.514	778.989	hypothetical protein
bin001	SOY3_bin001_01319	1332	4174	5167	5034	374.622	393.449	401.457	Phenylacetate-coenzyme A ligase
bin001	SOY3_bin001_01320	255	65	298	142	30.473	118.531	59.153	hypothetical protein
bin001	SOY3_bin001_01321	720	887	1732	1516	147.278	243.989	223.664	Acylphosphatase
bin001	SOY3_bin001_01322	2913	1343	1581	1265	55.116	55.049	46.130	ATP-dependent RNA helicase RhlE
bin001	SOY3_bin001_01323	462	432	343	401	111.786	75.302	92.200	hypothetical protein
bin001	SOY3_bin001_01324	1341	1743	1796	2008	155.387	135.841	159.061	Hydroxyneurosporene desaturase
bin001	SOY3_bin001_01325	282	328	578	478	139.050	207.890	180.056	Aspartyl/glutamyl-tRNA(Asn/Gln) amidotransferase subunit C
bin001	SOY3_bin001_01326	375	368	589	577	117.317	159.308	163.446	hypothetical protein
bin001	SOY3_bin001_01327	300	205	332	330	81.692	112.246	116.848	hypothetical protein
bin001	SOY3_bin001_01328	1017	1683	1984	1764	197.838	197.868	184.250	Putative aliphatic sulfonates-binding protein precursor
bin001	SOY3_bin001_01329	1305	294	515	415	26.933	40.027	33.781	hypothetical protein
bin001	SOY3_bin001_01330	561	376	406	337	80.125	73.404	63.811	Benzoylformate decarboxylase
bin001	SOY3_bin001_01331	498	562	587	503	134.913	119.554	107.292	hypothetical protein
bin001	SOY3_bin001_01332	438	11	30	15	3.002	6.947	3.638	DeoR-like helix-turn-helix domain protein
bin001	SOY3_bin001_01333	318	255	317	261	95.865	101.108	87.185	hypothetical protein
bin001	SOY3_bin001_01334	2277	8219	8613	7451	431.521	383.660	347.601	Anaerobic ribonucleoside-triphosphate reductase
bin001	SOY3_bin001_01335	177	8991	19091	16832	6072.678	10939.820	10101.649	Ferredoxin-2
bin001	SOY3_bin001_01336	675	580	489	500	102.723	73.478	78.686	hypothetical protein
bin001	SOY3_bin001_01337	456	9517	10334	9557	2495.059	2298.573	2226.315	30S ribosomal protein S15P

bin001	SOY3_bin001_01338	1323	10112	9538	8826	913.740	731.227	708.653	DHHA1 domain protein
bin001	SOY3_bin001_01339	231	970	780	785	502.002	342.482	360.984	hypothetical protein
bin001	SOY3_bin001_01340	1503	6939	6183	5962	551.929	417.249	421.369	Amino acid--[acyl-carrier-protein] ligase 1
bin001	SOY3_bin001_01341	351	1208	879	846	411.439	254.002	256.031	hypothetical protein
bin001	SOY3_bin001_01342	183	689	578	472	450.105	320.355	273.981	hypothetical protein
bin001	SOY3_bin001_01343	1443	31	88	34	2.568	6.185	2.503	hypothetical protein
bin001	SOY3_bin001_01344	297	4	7	2	1.610	2.391	0.715	hypothetical protein
bin001	SOY3_bin001_01345	300	116	134	105	46.226	45.304	37.179	hypothetical protein
bin001	SOY3_bin001_01346	294	337	369	322	137.034	127.302	116.342	hypothetical protein
bin001	SOY3_bin001_01347	219	52	55	76	28.386	25.473	36.864	hypothetical protein
bin001	SOY3_bin001_01348	279	4	2	2	1.714	0.727	0.761	hypothetical protein
bin001	SOY3_bin001_01349	1473	1501	2194	3176	121.821	151.074	229.038	TPR repeat-containing protein YrrB
bin001	SOY3_bin001_01350	165	191	174	169	138.387	106.960	108.801	hypothetical protein
bin001	SOY3_bin001_01351	1029	989	1391	1376	114.902	137.109	142.047	Thiosulfate sulfurtransferase
bin001	SOY3_bin001_01352	687	18	5	9	3.132	0.738	1.392	EamA-like transporter family protein
bin001	SOY3_bin001_01353	570	17	8	8	3.565	1.424	1.491	Acylphosphatase
bin001	SOY3_bin001_01354	477	358	403	344	89.724	85.692	76.607	hypothetical protein
bin001	SOY3_bin001_01355	1218	4967	6622	5819	487.520	551.438	507.494	ATP-dependent zinc metalloprotease FtsH
bin001	SOY3_bin001_01356	489	1352	1696	1463	330.532	351.780	317.808	transcription factor
bin001	SOY3_bin001_01357	534	1555	1637	1497	348.125	310.930	297.790	Threonylcarbamoyl-AMP synthase
bin001	SOY3_bin001_01358	936	2454	2713	2381	313.433	293.987	270.217	hypothetical protein
bin001	SOY3_bin001_01359	1515	3294	3763	3517	259.930	251.928	246.598	Pyrimidine-nucleoside phosphorylase
bin001	SOY3_bin001_01360	846	1575	1789	1741	222.564	214.484	218.604	Histone deacetylase-like amidohydrolase
bin001	SOY3_bin001_01361	858	2110	2175	2091	293.996	257.115	258.879	hypothetical protein
bin001	SOY3_bin001_01362	450	1777	1947	1860	472.085	438.842	439.066	PAC2 family protein
bin001	SOY3_bin001_01363	261	96	263	135	43.972	102.204	54.944	hypothetical protein
bin001	SOY3_bin001_01364	522	39763	59722	53054	9106.556	11604.291	10796.362	YcfA-like protein
bin001	SOY3_bin001_01365	141	278	241	158	235.706	173.362	119.033	hypothetical protein
bin001	SOY3_bin001_01366	843	464	870	796	65.802	104.676	100.303	2-hydroxy-6-oxo-6-phenylhexa-2,4-dienoate hydrolase
bin001	SOY3_bin001_01367	240	51	79	86	25.404	33.386	38.064	hypothetical protein
bin001	SOY3_bin001_01368	435	24	39	22	6.596	9.093	5.372	hypothetical protein
bin001	SOY3_bin001_01369	8796	4816	3645	3473	65.456	42.031	41.942	N,N'-diacetylchitobiose phosphorylase
bin001	SOY3_bin001_01370	471	742	875	913	188.334	188.426	205.911	Putative small multi-drug export protein
bin001	SOY3_bin001_01371	444	1193	1498	1501	321.220	342.203	359.110	hypothetical protein
bin001	SOY3_bin001_01372	180	29	45	41	19.261	25.357	24.196	Putative multidrug export ATP-binding/permease protein
bin001	SOY3_bin001_01373	1761	1674	1981	2294	113.643	114.098	138.377	Putative multidrug export ATP-binding/permease protein
bin001	SOY3_bin001_01374	714	1687	1255	3378	282.464	178.279	502.564	Nickel uptake substrate-specific transmembrane region
bin001	SOY3_bin001_01375	1269	328	299	107	30.900	23.898	8.957	iron-enterobactin transporter periplasmic binding protein
bin001	SOY3_bin001_01376	501	413	401	459	98.550	81.182	97.321	hypothetical protein
bin001	SOY3_bin001_01377	303	142	244	190	56.026	81.677	66.610	hypothetical protein
bin001	SOY3_bin001_01378	693	357	453	468	61.586	66.301	71.737	7-cyano-7-deazaguanine synthase
bin001	SOY3_bin001_01379	1308	975	1456	1305	89.113	112.904	105.982	Magnesium and cobalt efflux protein CorC
bin001	SOY3_bin001_01380	438	304	569	387	82.975	131.763	93.857	Large-conductance mechanosensitive channel
bin001	SOY3_bin001_01381	423	503	615	561	142.159	147.465	140.881	Iron-binding zinc finger CDGSH type
bin001	SOY3_bin001_01382	504	617	699	574	146.353	140.670	120.979	hypothetical protein
bin001	SOY3_bin001_01383	1938	1506	1941	1741	92.900	101.584	95.428	hypothetical protein
bin001	SOY3_bin001_01384	261	169	234	253	77.409	90.935	102.970	hypothetical protein
bin001	SOY3_bin001_01385	192	150	429	346	93.398	226.627	191.428	hypothetical protein
bin001	SOY3_bin001_01386	156	496	513	494	380.104	333.540	336.382	hypothetical protein
bin001	SOY3_bin001_01387	984	912	1035	955	110.801	106.684	103.095	Peptide methionine sulfoxide reductase MsrB
bin001	SOY3_bin001_01388	594	19	26	20	3.824	4.440	3.577	3',5'-cyclic adenosine monophosphate phosphodiesterase CpdA
bin001	SOY3_bin001_01389	1227	747	962	495	72.782	79.522	42.854	Glycerophosphoryl diester phosphodiesterase precursor
bin001	SOY3_bin001_01390	1155	403	324	467	41.713	28.452	42.950	Serine dehydrogenase proteinase
bin001	SOY3_bin001_01391	177	213	215	290	143.864	123.203	174.042	hypothetical protein
bin001	SOY3_bin001_01392	3237	1112	1015	1373	41.068	31.804	45.057	23S rRNA m(2)G2445 methyltransferase
bin001	SOY3_bin001_01393	153	43	56	96	33.599	37.124	66.651	nickel responsive regulator
bin001	SOY3_bin001_01394	393	13	11	18	3.955	2.839	4.865	SWIM zinc finger
bin001	SOY3_bin001_01395	318	2	5	6	0.752	1.595	2.004	hypothetical protein
bin001	SOY3_bin001_01396	141	1	0	0	0.848	0.000	0.000	hypothetical protein
bin001	SOY3_bin001_01397	288	1	2	1	0.415	0.704	0.369	hypothetical protein
bin001	SOY3_bin001_01398	129	0	0	0	0.000	0.000	0.000	hypothetical protein
bin001	SOY3_bin001_01399	192	1	2	2	0.623	1.057	1.107	hypothetical protein
bin001	SOY3_bin001_01400	3225	75	55	60	2.780	1.730	1.976	hypothetical protein
bin001	SOY3_bin001_01401	492	82	41	61	19.925	8.452	13.170	hypothetical protein
bin001	SOY3_bin001_01402	222	16	11	9	8.616	5.026	4.306	Helix-turn-helix domain of resolvase
bin001	SOY3_bin001_01403	294	17	10	11	6.913	3.450	3.974	hypothetical protein
bin001	SOY3_bin001_01404	219	22	14	16	12.009	6.484	7.761	hypothetical protein

bin001	SOY3_bin001_01405	924	101	171	162	13.068	18.771	18.624	TPR repeat-containing protein YrrB
bin001	SOY3_bin001_01406	507	16	12	4	3.773	2.401	0.838	hypothetical protein
bin001	SOY3_bin001_01407	1464	20	12	17	1.633	0.831	1.233	hypothetical protein
bin001	SOY3_bin001_01408	1350	29	25	32	2.568	1.878	2.518	hypothetical protein
bin001	SOY3_bin001_01409	1845	12	12	9	0.778	0.660	0.518	Phage Mu protein F like protein
bin001	SOY3_bin001_01410	687	22	8	11	3.828	1.181	1.701	hypothetical protein
bin001	SOY3_bin001_01411	936	54	32	57	6.897	3.468	6.469	hypothetical protein
bin001	SOY3_bin001_01412	333	6	5	2	2.154	1.523	0.638	hypothetical protein
bin001	SOY3_bin001_01413	627	2	6	1	0.381	0.971	0.169	hypothetical protein
bin001	SOY3_bin001_01414	357	1	1	2	0.335	0.284	0.595	hypothetical protein
bin001	SOY3_bin001_01415	360	8	5	12	2.657	1.409	3.541	hypothetical protein
bin001	SOY3_bin001_01416	198	2	3	1	1.208	1.537	0.536	hypothetical protein
bin001	SOY3_bin001_01417	282	6	4	12	2.544	1.439	4.520	hypothetical protein
bin001	SOY3_bin001_01418	321	2	0	0	0.745	0.000	0.000	hypothetical protein
bin001	SOY3_bin001_01419	342	2	3	0	0.699	0.890	0.000	hypothetical protein
bin001	SOY3_bin001_01420	369	1	1	1	0.324	0.275	0.288	hypothetical protein
bin001	SOY3_bin001_01421	435	3	2	3	0.824	0.466	0.733	Phage major tail protein 2
bin001	SOY3_bin001_01422	996	16	12	16	1.920	1.222	1.706	Phage major tail protein 2
bin001	SOY3_bin001_01423	522	19	20	22	4.351	3.886	4.477	hypothetical protein
bin001	SOY3_bin001_01424	3849	115	90	99	3.572	2.372	2.732	Phage-related minor tail protein
bin001	SOY3_bin001_01425	351	7	8	6	2.384	2.312	1.816	hypothetical protein
bin001	SOY3_bin001_01426	1773	8	7	7	0.539	0.400	0.419	hypothetical protein
bin001	SOY3_bin001_01427	309	11	6	5	4.256	1.969	1.719	hypothetical protein
bin001	SOY3_bin001_01428	1185	57	34	49	5.750	2.910	4.392	hypothetical protein
bin001	SOY3_bin001_01429	2385	295	183	203	14.787	7.782	9.041	Pectate lyase superfamily protein
bin001	SOY3_bin001_01430	561	134	89	136	28.555	16.091	25.752	F5/8 type C domain protein
bin001	SOY3_bin001_01431	774	42	54	84	6.487	7.076	11.528	hypothetical protein
bin001	SOY3_bin001_01432	954	133	147	237	16.667	15.629	26.389	hypothetical protein
bin001	SOY3_bin001_01433	495	132	145	192	31.880	29.711	41.203	hypothetical protein
bin001	SOY3_bin001_01434	909	113	80	78	14.861	8.926	9.115	Tyrosine recombinase XerC
bin001	SOY3_bin001_01435	495	90	49	59	21.736	10.040	12.661	hypothetical protein
bin001	SOY3_bin001_01436	486	466	382	351	114.629	79.723	76.719	hypothetical protein
bin001	SOY3_bin001_01437	228	220	209	199	115.354	92.975	92.715	hypothetical protein
bin001	SOY3_bin001_01438	1071	3629	3265	2894	405.082	309.206	287.038	Phosphoribosylamine--glycine ligase
bin001	SOY3_bin001_01439	942	533	565	522	67.643	60.835	58.864	EamA-like transporter family protein
bin001	SOY3_bin001_01440	918	635	909	763	82.694	100.433	88.290	Ribonuclease BN
bin001	SOY3_bin001_01441	630	935	1375	1076	177.426	221.369	181.427	hypothetical protein
bin001	SOY3_bin001_01442	1623	208	371	375	15.321	23.185	24.544	Signal transduction histidine-protein kinase BarA
bin001	SOY3_bin001_01443	1215	131	317	215	12.890	26.463	18.797	Signal transduction histidine-protein kinase BarA
bin001	SOY3_bin001_01444	738	191	259	203	30.940	35.596	29.219	Histone deacetylase-like amidohydrolase
bin001	SOY3_bin001_01445	627	344	386	333	65.590	62.442	56.417	hypothetical protein
bin001	SOY3_bin001_01446	240	23	27	29	11.457	11.411	12.836	MTH865-like family protein
bin001	SOY3_bin001_01447	777	168	173	194	25.848	22.583	26.522	Helix-turn-helix domain protein
bin001	SOY3_bin001_01448	606	192	230	280	37.877	38.496	49.081	FMN-dependent NADH-azoreductase 1
bin001	SOY3_bin001_01449	921	4	7	5	0.519	0.771	0.577	Tyrosine recombinase XerC
bin001	SOY3_bin001_01450	510	4	0	4	0.938	0.000	0.833	hypothetical protein
bin001	SOY3_bin001_01451	792	3168	4376	4004	478.196	560.411	537.030	hypothetical protein
bin001	SOY3_bin001_01452	927	2977	4104	6520	383.923	449.037	747.133	Nickel uptake substrate-specific transmembrane region
bin001	SOY3_bin001_01453	741	614	737	1413	99.059	100.880	202.560	hypothetical protein
bin001	SOY3_bin001_01454	831	669	619	749	96.243	75.552	95.744	Energy-coupling factor transporter ATP-binding protein EcfA3
bin001	SOY3_bin001_01455	750	738	721	881	117.636	97.505	124.780	Energy-coupling factor transporter transmembrane protein EcfT
bin001	SOY3_bin001_01456	423	1206	1133	1485	340.842	271.672	372.920	hypothetical protein
bin001	SOY3_bin001_01457	318	920	985	1194	345.865	314.169	398.848	Nickel uptake substrate-specific transmembrane region
bin001	SOY3_bin001_01458	630	1479	1450	1879	280.655	233.444	316.823	Fused nickel transport protein NikMN
bin001	SOY3_bin001_01459	771	2341	2590	3123	362.988	340.722	430.276	Nickel uptake substrate-specific transmembrane region
bin001	SOY3_bin001_01460	795	6520	7288	12170	980.451	929.814	1626.123	Nickel uptake substrate-specific transmembrane region
bin001	SOY3_bin001_01461	270	96	112	237	42.506	42.074	93.243	hypothetical protein
bin001	SOY3_bin001_01462	789	3985	3211	7357	603.805	412.779	990.498	Nickel uptake substrate-specific transmembrane region
bin001	SOY3_bin001_01463	864	671	756	839	92.844	88.749	103.152	ubiquinone/menaquinone biosynthesis methyltransferase
bin001	SOY3_bin001_01464	408	1044	1203	1286	305.904	299.061	334.819	Putative nickel-responsive regulator
bin001	SOY3_bin001_01465	1458	4690	5160	5191	384.557	358.961	378.202	TPR repeat-containing protein YrrB
bin001	SOY3_bin001_01466	840	3836	4291	4282	545.940	518.124	541.498	hypothetical protein
bin001	SOY3_bin001_01467	468	1103	1640	1541	281.757	355.429	349.773	hypothetical protein
bin001	SOY3_bin001_01468	489	739	1082	965	180.668	224.426	209.628	hypothetical protein
bin001	SOY3_bin001_01469	165	471	664	520	341.258	408.168	334.772	hypothetical protein
bin001	SOY3_bin001_01470	270	41	34	31	18.154	12.772	12.196	hypothetical protein
bin001	SOY3_bin001_01471	408	55	57	62	16.116	14.170	16.142	corrinoid ABC transporter substrate-binding protein

bin001	SOY3_bin001_01472	813	88	103	82	12.940	12.850	10.714	Vitamin B12-binding protein
bin001	SOY3_bin001_01473	1002	70	62	50	8.352	6.276	5.301	Vitamin B12-binding protein precursor
bin001	SOY3_bin001_01474	1092	76	104	81	8.320	9.660	7.879	Fe(3+)-citrate-binding protein YfmC precursor
bin001	SOY3_bin001_01475	1392	110	192	98	9.447	13.990	7.479	Vitamin B12-binding protein precursor
bin001	SOY3_bin001_01476	3804	65	79	49	2.043	2.106	1.368	Aerobic cobaltochelatase subunit CobN
bin001	SOY3_bin001_01477	411	5	7	5	1.454	1.727	1.292	2-oxoglutarate-acceptor oxidoreductase subunit OorD
bin001	SOY3_bin001_01478	1059	68	84	43	7.676	8.045	4.313	Vitamin B12-binding protein precursor
bin001	SOY3_bin001_01479	249	24	49	35	11.523	19.960	14.931	hypothetical protein
bin001	SOY3_bin001_01480	447	526	847	771	140.677	192.190	183.222	Deoxycytidine triphosphate deaminase
bin001	SOY3_bin001_01481	765	40	26	35	6.251	3.447	4.860	Glycine--tRNA ligase
bin001	SOY3_bin001_01482	390	77	90	72	23.603	23.406	19.611	Lactoylglutathione lyase
bin001	SOY3_bin001_01483	840	37	45	36	5.266	5.434	4.553	hypothetical protein
bin001	SOY3_bin001_01484	879	448	842	456	60.930	97.158	55.107	hypothetical protein
bin001	SOY3_bin001_01485	498	197	260	138	47.291	52.954	29.436	hypothetical protein
bin001	SOY3_bin001_01486	408	247	472	211	72.374	117.337	54.935	Thioredoxin
bin001	SOY3_bin001_01487	579	149	307	132	30.765	53.779	24.217	hypothetical protein
bin001	SOY3_bin001_01488	1866	368	816	607	23.577	44.354	34.555	Malto-oligosyltrehalose trehalohydrolase
bin001	SOY3_bin001_01489	465	239	328	283	61.446	71.544	64.649	hypothetical protein
bin001	SOY3_bin001_01490	600	735	968	1037	146.447	163.636	183.594	hypothetical protein
bin001	SOY3_bin001_01491	450	2076	3047	2823	551.519	686.775	666.390	hypothetical protein
bin001	SOY3_bin001_01492	720	1279	1822	1573	212.365	256.667	232.074	glucose-6-phosphate isomerase
bin001	SOY3_bin001_01493	261	271	312	280	124.129	121.246	113.959	hypothetical protein
bin001	SOY3_bin001_01494	840	179	210	215	25.475	25.357	27.189	Transposase IS66 family protein
bin001	SOY3_bin001_01495	246	49	25	36	23.813	10.308	15.545	hypothetical protein
bin001	SOY3_bin001_01496	783	116	94	105	17.711	12.176	14.245	hypothetical protein
bin001	SOY3_bin001_01497	1002	46	39	60	5.488	3.948	6.361	hypothetical protein
bin001	SOY3_bin001_01498	405	60	44	57	17.711	11.019	14.950	MarR family protein
bin001	SOY3_bin001_01499	360	114	83	92	37.857	23.385	27.147	Bacterial regulatory protein, arsR family
bin001	SOY3_bin001_01500	177	186	140	159	125.628	80.225	95.423	hypothetical protein
bin001	SOY3_bin001_01501	195	169	104	111	103.609	54.095	60.467	hypothetical protein
bin001	SOY3_bin001_01502	423	661	456	586	186.813	109.340	147.159	hypothetical protein
bin001	SOY3_bin001_01503	72	56	59	47	92.982	83.114	69.342	tRNA-Gly(gcc)
bin001	SOY3_bin001_01504	2133	844	1212	1116	47.304	57.632	55.578	Cellobiose 2-epimerase
bin001	SOY3_bin001_01505	888	33839	37605	36758	4555.647	4295.239	4397.124	hypothetical protein
bin001	SOY3_bin001_01506	576	3642	4594	4278	755.898	808.953	788.948	Inner membrane protein YaaH
bin001	SOY3_bin001_01507	399	59	121	127	17.678	30.759	33.811	hypothetical protein
bin001	SOY3_bin001_01508	867	1827	1879	1724	251.921	219.818	211.226	Branched-chain-amino-acid aminotransferase
bin001	SOY3_bin001_01509	459	1537	1745	1439	400.319	385.600	333.026	6,7-dimethyl-8-ribityllumazine synthase
bin001	SOY3_bin001_01510	1137	3321	3407	3066	349.184	303.925	286.445	Aspartate aminotransferase
bin001	SOY3_bin001_01511	558	117	123	103	25.067	22.358	19.608	hypothetical protein
bin001	SOY3_bin001_01512	207	563	761	543	325.150	372.880	278.650	hypothetical protein
bin001	SOY3_bin001_01513	1266	3498	4648	4273	330.318	372.381	358.533	Succinyl-CoA ligase [ADP-forming] subunit beta
bin001	SOY3_bin001_01514	999	322	410	322	38.533	41.627	34.239	Ribosomal protein L11 methyltransferase
bin001	SOY3_bin001_01515	77	115	112	85	178.547	147.531	117.262	tRNA-Pro(tgg)
bin001	SOY3_bin001_01516	1131	601	619	618	63.527	55.511	58.044	hypothetical protein
bin001	SOY3_bin001_01517	180	3488	4370	4326	2316.592	2462.429	2552.959	hypothetical protein
bin001	SOY3_bin001_01518	1200	8680	8361	8282	864.737	706.695	733.135	putative tRNA sulfurtransferase
bin001	SOY3_bin001_01519	213	962	677	697	539.934	322.377	347.603	hypothetical protein
bin001	SOY3_bin001_01520	192	31	67	30	19.302	35.394	16.598	Glucose starvation-inducible protein B
bin001	SOY3_bin001_01521	582	270	314	280	55.461	54.722	51.105	Putative DNA ligase-like protein/MT0965
bin001	SOY3_bin001_01522	141	10813	11304	11517	9167.958	8131.446	8676.610	Acetyl-coenzyme A synthetase
bin001	SOY3_bin001_01523	1971	33834	38337	37330	2052.165	1972.815	2011.876	Acetyl-coenzyme A synthetase
bin001	SOY3_bin001_01524	1062	455	483	462	51.219	46.129	46.211	Alpha-aminoadipate--LysW ligase LysX
bin001	SOY3_bin001_01525	1278	5206	5394	5214	486.989	428.090	433.381	Adenylosuccinate synthetase
bin001	SOY3_bin001_01526	1734	1754	2142	1954	120.928	125.293	119.703	DNA polymerase/3'-5' exonuclease PolX
bin001	SOY3_bin001_01527	735	458	623	572	74.494	85.972	82.668	hypothetical protein
bin001	SOY3_bin001_01528	714	467	502	509	78.192	71.312	75.727	hypothetical protein
bin001	SOY3_bin001_01529	3270	847	792	862	30.966	24.566	28.002	Signal transduction histidine-protein kinase BarA
bin001	SOY3_bin001_01530	345	577	741	724	199.941	217.848	222.920	hypothetical protein
bin001	SOY3_bin001_01531	2466	3759	4645	4228	182.232	191.050	182.126	hypothetical protein
bin001	SOY3_bin001_01532	648	447	776	622	82.467	121.462	101.964	hypothetical protein
bin001	SOY3_bin001_01533	1314	505	636	542	45.945	49.093	43.816	hypothetical protein
bin001	SOY3_bin001_01534	729	441	482	488	72.320	67.062	71.109	hypothetical protein
bin001	SOY3_bin001_01535	1521	503	486	519	39.535	32.409	36.247	hypothetical protein
bin001	SOY3_bin001_01536	717	224	613	358	37.349	86.715	53.039	PD-(D/E)XK nuclease superfamily protein
bin001	SOY3_bin001_01537	1287	2152	2181	1846	199.898	171.883	152.364	GTPase Obg
bin001	SOY3_bin001_01538	405	9543	4656	4428	2816.926	1166.038	1161.402	hypothetical protein

bin001	SOY3_bin001_01539	834	3655	3328	2810	523.922	404.736	357.907	Phosphate-binding protein PstS 1 precursor
bin001	SOY3_bin001_01540	1686	28	86	33	1.985	5.174	2.079	Internalin-A precursor
bin001	SOY3_bin001_01541	972	123	264	108	15.128	27.548	11.803	Cyclic pyranopterin monophosphate synthase 1
bin001	SOY3_bin001_01542	1416	220	509	196	18.574	36.459	14.704	Mycothione reductase
bin001	SOY3_bin001_01543	225	1441	1120	1500	765.644	504.882	708.172	hypothetical protein
bin001	SOY3_bin001_01544	1122	5962	5498	6068	635.250	497.012	574.490	Hemin-binding periplasmic protein HmuT precursor
bin001	SOY3_bin001_01545	501	11	22	24	2.625	4.454	5.089	hypothetical protein
bin001	SOY3_bin001_01546	795	16437	14718	14256	2471.730	1877.744	1904.848	Ferredoxin-1
bin001	SOY3_bin001_01547	390	8432	8547	7842	2584.708	2222.818	2135.955	30S ribosomal protein S11
bin001	SOY3_bin001_01548	537	10762	10586	9811	2395.875	1999.458	1940.746	30S ribosomal protein S4
bin001	SOY3_bin001_01549	546	12887	12819	11952	2821.660	2381.312	2325.293	30S ribosomal protein S13
bin001	SOY3_bin001_01550	633	169	152	128	31.917	24.355	21.480	hypothetical protein
bin001	SOY3_bin001_01551	294	136	229	168	55.302	79.003	60.700	hypothetical protein
bin001	SOY3_bin001_01552	1362	951	1233	1102	83.474	91.821	85.948	Magnesium transporter MgtE
bin001	SOY3_bin001_01553	519	248	367	323	57.125	71.722	66.110	Regulatory protein AsnC
bin001	SOY3_bin001_01554	612	118	192	178	23.050	31.820	30.896	hypothetical protein
bin001	SOY3_bin001_01555	2145	199	437	195	11.091	20.664	9.657	SH3 domain of the SH3b1 type
bin001	SOY3_bin001_01556	498	16	46	16	3.841	9.369	3.413	hypothetical protein
bin001	SOY3_bin001_01557	243	469	515	425	230.734	214.959	185.786	hypothetical protein
bin001	SOY3_bin001_01558	219	75	66	71	40.941	30.567	34.438	hypothetical protein
bin001	SOY3_bin001_01559	75	52	102	82	82.887	137.941	116.140	tRNA-Asp(gtc)
bin001	SOY3_bin001_01560	531	615	752	806	138.461	143.641	161.239	phosphodiesterase
bin001	SOY3_bin001_01561	456	948	819	862	248.536	182.169	200.804	Methylated-DNA--protein-cysteine methyltransferase
bin001	SOY3_bin001_01562	1005	3498	2807	2741	416.101	283.290	289.716	hypothetical protein
bin001	SOY3_bin001_01563	654	2303	2056	1926	420.980	318.860	312.830	hypothetical protein
bin001	SOY3_bin001_01564	747	2785	2542	2551	445.708	345.151	362.760	Phosphate import ATP-binding protein PstB 3
bin001	SOY3_bin001_01565	474	447	558	526	112.739	119.402	117.879	hypothetical protein
bin001	SOY3_bin001_01566	255	303	422	398	142.052	167.852	165.795	hypothetical protein
bin001	SOY3_bin001_01567	786	554	646	576	84.262	83.361	77.845	Inner membrane transport permease YadH
bin001	SOY3_bin001_01568	1017	991	1242	1253	116.493	123.867	130.876	Daunorubicin/doxorubicin resistance ATP-binding protein DrrA
bin001	SOY3_bin001_01569	981	728	1022	1036	88.717	105.666	112.181	hypothetical protein
bin001	SOY3_bin001_01570	807	523	512	550	77.477	64.350	72.397	High-affinity zinc uptake system membrane protein ZnuB
bin001	SOY3_bin001_01571	762	734	650	616	115.156	86.519	85.873	High-affinity zinc uptake system ATP-binding protein ZnuC
bin001	SOY3_bin001_01572	999	835	858	746	99.923	87.112	79.324	High-affinity zinc uptake system binding-protein ZnuA precursor
bin001	SOY3_bin001_01573	351	238	252	189	81.062	72.820	57.198	hypothetical protein
bin001	SOY3_bin001_01574	771	686	979	861	106.369	128.790	118.626	hypothetical protein
bin001	SOY3_bin001_01575	1992	1200	1423	1346	72.017	72.455	71.777	Putative agmatine deiminase
bin001	SOY3_bin001_01576	378	324	349	348	102.470	93.646	97.795	PIN domain protein
bin001	SOY3_bin001_01577	177	186	236	227	125.628	135.236	136.233	hypothetical protein
bin001	SOY3_bin001_01578	558	99	183	87	21.210	33.264	16.562	Chaperone protein YajL
bin001	SOY3_bin001_01579	297	31	62	46	12.478	21.173	16.452	hypothetical protein
bin001	SOY3_bin001_01580	492	152	338	160	36.934	69.680	34.545	SprT-like family protein
bin001	SOY3_bin001_01581	234	304	350	329	155.311	151.707	149.352	hypothetical protein
bin001	SOY3_bin001_01582	1122	1874	2110	1981	199.674	190.741	187.552	Cytidine deaminase
bin001	SOY3_bin001_01583	1371	15985	18516	17657	1393.865	1369.823	1368.073	Anaerobic glycerol-3-phosphate dehydrogenase subunit C
bin001	SOY3_bin001_01584	753	7603	9427	9173	1207.079	1269.794	1294.036	Nitrate reductase gamma subunit
bin001	SOY3_bin001_01585	501	922	1930	1106	220.008	390.728	234.502	hypothetical protein
bin001	SOY3_bin001_01586	1605	3855	5855	6857	287.141	370.004	453.826	Heme-binding protein A precursor
bin001	SOY3_bin001_01587	1956	689	753	728	42.111	39.046	39.536	Succinyl-CoA:coenzyme A transferase
bin001	SOY3_bin001_01588	906	5	0	1	0.660	0.000	0.117	hypothetical protein
bin001	SOY3_bin001_01589	777	841	2219	1556	129.396	289.662	212.725	Ribosomal RNA large subunit methyltransferase J
bin001	SOY3_bin001_01590	483	289	652	485	71.531	136.916	106.666	hypothetical protein
bin001	SOY3_bin001_01591	1701	958	1023	982	67.330	60.999	61.325	Ferrous iron transport protein B
bin001	SOY3_bin001_01592	1137	843	1202	1147	88.637	107.226	107.160	hypothetical protein
bin001	SOY3_bin001_01593	942	1775	2615	2170	225.265	281.563	244.703	Immunogenic protein MPT70 precursor
bin001	SOY3_bin001_01594	741	211	376	343	34.042	51.466	49.171	hypothetical protein
bin001	SOY3_bin001_01595	399	34	54	43	10.187	13.727	11.448	hypothetical protein
bin001	SOY3_bin001_01596	234	520	459	521	265.664	198.953	236.511	hypothetical protein
bin001	SOY3_bin001_01597	315	1833	2384	2290	695.661	767.627	772.244	hypothetical protein
bin001	SOY3_bin001_01598	423	921	861	849	260.294	206.451	213.205	hypothetical protein
bin001	SOY3_bin001_01599	849	430	600	523	60.549	71.680	65.437	Type I phosphodiesterase / nucleotide pyrophosphatase
bin001	SOY3_bin001_01600	468	1471	1499	1483	375.762	324.871	336.609	hypothetical protein
bin001	SOY3_bin001_01601	1641	1567	1571	1639	114.158	97.101	106.096	TPR repeat-containing protein YrrB
bin001	SOY3_bin001_01602	549	449	1312	845	97.773	242.391	163.499	Macrolide export ATP-binding/permease protein MacB
bin001	SOY3_bin001_01603	699	521	1240	822	89.106	179.928	124.918	Hydrogenase isoenzymes nickel incorporation protein HypB
bin001	SOY3_bin001_01604	681	300	619	435	52.665	92.193	67.853	ferredoxin
bin001	SOY3_bin001_01605	1068	49	97	84	5.485	9.212	8.355	hypothetical protein

bin001	SOY3_bin001_01606	165	2	2	2	1.449	1.229	1.288	hypothetical protein
bin001	SOY3_bin001_01607	291	235	283	288	96.543	98.639	105.131	CHAT domain protein
bin001	SOY3_bin001_01608	870	1358	1854	1684	186.606	216.145	205.614	Acylphosphatase
bin001	SOY3_bin001_01609	810	1257	1587	1569	185.522	198.722	205.763	Protoheme IX farnesyltransferase
bin001	SOY3_bin001_01610	870	296	789	563	40.674	91.984	68.741	MarR family protein
bin001	SOY3_bin001_01611	555	463	358	352	99.732	65.425	67.372	hypothetical protein
bin001	SOY3_bin001_01612	285	513	498	499	215.188	177.231	185.988	hypothetical protein
bin001	SOY3_bin001_01613	1062	2316	2769	2686	260.711	264.456	268.665	MarR family protein
bin001	SOY3_bin001_01614	663	459	472	482	82.765	72.208	77.226	hypothetical protein
bin001	SOY3_bin001_01615	120	184	191	257	183.308	161.438	227.500	hypothetical protein
bin001	SOY3_bin001_01616	444	9191	10275	10577	2474.716	2347.219	2530.518	hypothetical protein
bin001	SOY3_bin001_01617	915	841	1194	1083	109.880	132.354	125.729	Glycerophosphoryl diester phosphodiesterase
bin001	SOY3_bin001_01618	210	265	341	298	150.859	164.699	150.739	hypothetical protein
bin001	SOY3_bin001_01619	456	783	944	864	205.278	209.972	201.270	hypothetical protein
bin001	SOY3_bin001_01620	1170	2144	2391	2197	219.071	207.276	199.468	Anaerobic sulfatase-maturating enzyme
bin001	SOY3_bin001_01621	1230	4579	5434	5181	445.052	448.094	447.444	Radical SAM superfamily protein
bin001	SOY3_bin001_01622	3516	4226	6976	5507	143.690	201.239	166.378	Chromosome partition protein Smc
bin001	SOY3_bin001_01623	711	585	810	608	98.363	115.550	90.837	Segregation and condensation protein A
bin001	SOY3_bin001_01624	3144	799	1956	862	30.382	63.102	29.124	hypothetical protein
bin001	SOY3_bin001_01625	243	20	38	14	9.839	15.861	6.120	hypothetical protein
bin001	SOY3_bin001_01626	417	31	64	22	8.887	15.567	5.604	hypothetical protein
bin001	SOY3_bin001_01627	2964	205	364	174	8.268	12.456	6.236	D12 class N6 adenine-specific DNA methyltransferase
bin001	SOY3_bin001_01628	1074	56	92	39	6.233	8.688	3.857	ferredoxin-NADP reductase
bin001	SOY3_bin001_01629	573	31	40	23	6.468	7.080	4.264	hypothetical protein
bin001	SOY3_bin001_01630	585	34	63	23	6.948	10.923	4.176	hypothetical protein
bin001	SOY3_bin001_01631	3450	223	521	185	7.727	15.317	5.696	RNA polymerase-associated protein RapA
bin001	SOY3_bin001_01632	1290	1360	1492	1662	126.036	117.310	136.858	Glutathione import ATP-binding protein GsiA
bin001	SOY3_bin001_01633	252	214	219	248	101.522	88.145	104.540	hypothetical protein
bin001	SOY3_bin001_01634	489	257	250	247	62.830	51.854	53.656	TspO/MBR family protein
bin001	SOY3_bin001_01635	261	378	644	602	173.140	250.265	245.011	PRC-barrel domain protein
bin001	SOY3_bin001_01636	702	681	1052	904	115.973	151.996	136.792	tRNAHis guanylyltransferase
bin001	SOY3_bin001_01637	897	524	817	708	69.837	92.381	83.844	Sigma-70, region 4
bin001	SOY3_bin001_01638	1452	1148	1323	1261	94.519	92.416	92.253	Ribulose bisphosphate carboxylase
bin001	SOY3_bin001_01639	285	374	447	428	156.882	159.081	159.525	hypothetical protein
bin001	SOY3_bin001_01640	189	29	36	24	18.343	19.319	13.489	hypothetical protein
bin001	SOY3_bin001_01641	312	175	219	214	67.055	71.194	72.860	hypothetical protein
bin001	SOY3_bin001_01642	1200	1999	2516	2369	199.149	212.659	209.707	Glucose-1-phosphate thymidylyltransferase
bin001	SOY3_bin001_01643	1125	678	619	542	72.048	55.808	51.177	Putative teichuronic acid biosynthesis glycosyltransferase TuaH
bin001	SOY3_bin001_01644	969	324	269	238	39.973	28.157	26.091	D-inositol 3-phosphate glycosyltransferase
bin001	SOY3_bin001_01645	987	742	461	440	89.874	47.374	47.355	Alpha-galactosylglucosyldiacylglycerol synthase
bin001	SOY3_bin001_01646	993	422	370	417	50.805	37.793	44.608	UDP-Glc:alpha-D-GlcNAc-diphosphoundecaprenol beta-1,3-glucosyltransferase WfgD
bin001	SOY3_bin001_01647	1461	347	357	392	28.394	24.784	28.501	colanic acid exporter
bin001	SOY3_bin001_01648	498	709	786	810	170.201	160.084	172.777	dTDP-4-dehydrorhamnose 3,5-epimerase
bin001	SOY3_bin001_01649	873	1099	1369	1196	150.497	159.054	145.528	dTDP-4-dehydrorhamnose reductase
bin001	SOY3_bin001_01650	1002	1925	2535	2291	229.672	256.605	242.877	dTDP-glucose 4,6-dehydratase
bin001	SOY3_bin001_01651	1053	2051	2460	2425	232.854	236.953	244.632	Glucose-1-phosphate thymidylyltransferase
bin001	SOY3_bin001_01652	1185	1387	2886	2155	139.928	247.020	193.178	2-isopropylmalate synthase
bin001	SOY3_bin001_01653	573	186	200	183	38.806	35.402	33.926	GMP synthase [glutamine-hydrolyzing]
bin001	SOY3_bin001_01654	672	256	286	283	45.542	43.167	44.735	ATP-binding region
bin001	SOY3_bin001_01655	1518	400	439	403	31.502	29.332	28.201	Cobyric acid synthase
bin001	SOY3_bin001_01656	558	321	455	430	68.773	82.705	81.859	Iron-sulfur flavoprotein
bin001	SOY3_bin001_01657	795	775	676	753	116.541	86.245	100.614	DNA polymerase IV
bin001	SOY3_bin001_01658	231	452	312	291	233.922	136.993	133.817	hypothetical protein
bin001	SOY3_bin001_01659	354	437	306	349	147.579	87.674	104.725	peptidyl-tRNA hydrolase
bin001	SOY3_bin001_01660	1323	4344	4015	3833	392.532	307.808	307.758	DNA primase
bin001	SOY3_bin001_01661	1188	630	766	756	63.397	65.398	67.598	biotin synthase
bin001	SOY3_bin001_01662	327	532	498	480	194.495	154.467	155.928	Bacilysin biosynthesis protein BacB
bin001	SOY3_bin001_01663	1140	3143	4208	3954	329.598	374.391	368.436	Inositol-3-phosphate synthase
bin001	SOY3_bin001_01664	933	1188	1273	1320	152.223	138.389	150.287	hypothetical protein
bin001	SOY3_bin001_01665	498	134	163	159	32.168	33.198	33.915	hypothetical protein
bin001	SOY3_bin001_01666	756	659	1391	628	104.210	186.621	88.240	hypothetical protein
bin001	SOY3_bin001_01667	1080	315	671	356	34.868	63.016	35.015	hypothetical protein
bin001	SOY3_bin001_01668	216	80	82	64	44.277	38.505	31.474	hypothetical protein
bin001	SOY3_bin001_01669	390	697	778	629	213.655	202.334	171.323	Iron-sulfur cluster insertion protein ErpA
bin001	SOY3_bin001_01670	1011	859	1136	910	101.575	113.968	95.614	flap endonuclease-1
bin001	SOY3_bin001_01671	1020	695	783	586	81.457	77.860	61.028	Putative diphthamide synthesis protein
bin001	SOY3_bin001_01672	849	3217	3265	3896	452.990	390.059	487.462	Magnesium-chelatase 60 kDa subunit

bin001	SOY3_bin001_01673	861	2240	2338	2782	311.021	275.420	343.229	Daunorubicin/doxorubicin resistance ATP-binding protein DrrA
bin001	SOY3_bin001_01674	738	1069	1047	1292	173.168	143.895	185.967	Inner membrane transport permease YadH
bin001	SOY3_bin001_01675	1341	8517	6579	5609	759.283	497.606	444.310	60 kDa chaperonin
bin001	SOY3_bin001_01676	522	2946	2139	1850	674.695	415.619	376.471	heat shock protein GrpE
bin001	SOY3_bin001_01677	1869	12381	9486	8338	791.939	514.788	473.895	Chaperone protein DnaK
bin001	SOY3_bin001_01678	1170	8167	6952	6165	834.492	602.669	559.728	Chaperone protein DnaJ
bin001	SOY3_bin001_01679	621	1503	1268	1128	289.343	207.101	192.951	Flagellin N-methylase
bin001	SOY3_bin001_01680	1170	603	569	651	61.614	49.327	59.105	Bifunctional NAD(P)H-hydrate repair enzyme Nnr
bin001	SOY3_bin001_01681	486	313	282	361	76.993	58.853	78.904	Cyclic pyranopterin monophosphate synthase accessory protein 2
bin001	SOY3_bin001_01682	285	175	102	128	73.407	36.300	47.708	Aspartate aminotransferase
bin001	SOY3_bin001_01683	669	191	154	160	34.131	23.348	25.405	SOUL heme-binding protein
bin001	SOY3_bin001_01684	135	101	90	124	89.440	67.618	97.570	hypothetical protein
bin001	SOY3_bin001_01685	360	131	106	125	43.503	29.865	36.884	Toxin Doc
bin001	SOY3_bin001_01686	213	109	211	268	61.178	100.475	133.655	hypothetical protein
bin001	SOY3_bin001_01687	888	2037	2295	2491	274.235	262.135	297.982	hypothetical protein
bin001	SOY3_bin001_01688	486	785	803	915	193.099	167.585	199.993	hypothetical protein
bin001	SOY3_bin001_01689	408	1083	1307	1417	317.332	324.915	368.926	Thioredoxin
bin001	SOY3_bin001_01690	579	725	796	893	149.694	139.441	163.834	hypothetical protein
bin001	SOY3_bin001_01691	255	403	547	482	188.934	217.571	200.788	Glutamyl-tRNA(Gln) amidotransferase subunit A
bin001	SOY3_bin001_01692	1416	2024	2789	2518	170.881	199.774	188.896	Aspartyl/glutamyl-tRNA(Asn/Gln) amidotransferase subunit B
bin001	SOY3_bin001_01693	198	100	243	204	60.378	124.479	109.445	hypothetical protein
bin001	SOY3_bin001_01694	1155	979	1099	1150	101.332	96.510	105.766	peptide chain release factor 1
bin001	SOY3_bin001_01695	804	624	556	456	92.784	70.141	60.247	Alpha-acetolactate decarboxylase precursor
bin001	SOY3_bin001_01696	1104	590	780	699	63.889	71.661	67.257	Isonitrile hydratase
bin001	SOY3_bin001_01697	552	578	2073	791	125.180	380.904	152.218	hypothetical protein
bin001	SOY3_bin001_01698	303	72	195	82	28.408	65.275	28.748	AsnC family protein
bin001	SOY3_bin001_01699	588	142	241	137	28.871	41.571	24.750	hypothetical protein
bin001	SOY3_bin001_01700	489	31	51	36	7.579	10.578	7.820	tRNA-specific adenosine deaminase
bin001	SOY3_bin001_01701	681	272	771	529	47.749	114.832	82.516	Transglutaminase-like superfamily protein
bin001	SOY3_bin001_01702	510	69	92	81	16.174	18.297	16.871	hypothetical protein
bin001	SOY3_bin001_01703	1284	12237	7380	6212	1139.346	582.970	513.921	ATP-dependent RNA helicase RhlE
bin001	SOY3_bin001_01704	1311	2199	2394	2234	200.525	185.215	181.013	Histidine--tRNA ligase
bin001	SOY3_bin001_01705	2310	1127	1616	1285	58.325	70.955	59.091	ATP-dependent RNA helicase DbpA
bin001	SOY3_bin001_01706	93	0	0	0	0.000	0.000	0.000	hypothetical protein
bin001	SOY3_bin001_01707	399	458	470	442	137.227	119.476	117.674	hypothetical protein
bin001	SOY3_bin001_01708	387	524	439	424	161.870	115.056	116.382	hypothetical protein
bin001	SOY3_bin001_01709	243	820	1013	947	403.416	422.822	413.974	RNA-binding protein Hfq
bin001	SOY3_bin001_01710	1347	492	611	541	43.666	46.007	42.664	Sodium/pantothenate symporter
bin001	SOY3_bin001_01711	411	2392	3725	3177	695.769	919.262	821.117	hypothetical protein
bin001	SOY3_bin001_01712	582	483	649	595	99.213	113.104	108.598	hypothetical protein
bin001	SOY3_bin001_01713	837	194	236	185	27.709	28.598	23.479	Lipoyl synthase
bin001	SOY3_bin001_01714	537	21	15	8	4.675	2.833	1.583	hypothetical protein
bin001	SOY3_bin001_01715	180	13	5	4	8.634	2.817	2.361	hypothetical protein
bin001	SOY3_bin001_01716	939	34	39	24	4.329	4.213	2.715	Modification methylase PvuII
bin001	SOY3_bin001_01717	648	85	71	72	15.682	11.113	11.803	Isoprenylcysteine carboxyl methyltransferase (ICMT) family protein
bin001	SOY3_bin001_01718	333	59	118	90	21.181	35.941	28.710	hypothetical protein
bin001	SOY3_bin001_01719	639	55	131	54	10.290	20.793	8.977	Intermediate filament tail domain protein
bin001	SOY3_bin001_01720	618	90	207	81	17.410	33.973	13.923	Arylesterase precursor
bin001	SOY3_bin001_01721	855	54	87	54	7.550	10.321	6.709	Shikimate dehydrogenase
bin001	SOY3_bin001_01722	1215	532	782	710	52.346	65.281	62.074	hypothetical protein
bin001	SOY3_bin001_01723	612	483	545	494	94.350	90.323	85.744	(2Z,6E)-farnesyl diphosphate synthase
bin001	SOY3_bin001_01724	645	1565	1723	1573	290.068	270.944	259.059	RNA 2'-phosphotransferase
bin001	SOY3_bin001_01725	231	1148	1858	1717	594.122	815.809	789.565	hypothetical protein
bin001	SOY3_bin001_01726	441	63	97	71	17.078	22.309	17.102	hypothetical protein
bin001	SOY3_bin001_01727	2535	2502	2977	2703	117.993	119.112	113.266	AAA-like domain protein
bin001	SOY3_bin001_01728	144	13	18	12	10.793	12.678	8.852	hypothetical protein
bin001	SOY3_bin001_01729	303	24	21	17	9.469	7.030	5.960	hypothetical protein
bin001	SOY3_bin001_01730	255	306	349	355	143.459	138.816	147.883	hypothetical protein
bin001	SOY3_bin001_01731	204	382	441	502	223.861	219.262	261.399	hypothetical protein
bin001	SOY3_bin001_01732	909	355	454	419	46.689	50.658	48.964	2,5-diketo-D-gluconic acid reductase A
bin001	SOY3_bin001_01733	330	37	50	55	13.404	15.368	17.704	Divalent metal cation transporter MntH
bin001	SOY3_bin001_01734	1206	1069	906	1122	105.968	76.197	98.827	Magnesium transport protein CorA
bin001	SOY3_bin001_01735	906	559	641	703	73.761	71.760	82.425	Small-conductance mechanosensitive channel
bin001	SOY3_bin001_01736	570	92	108	34	19.296	19.218	6.336	putative GTP-binding protein EngB
bin001	SOY3_bin001_01737	1176	2031	2744	2428	206.466	236.664	219.316	2-octaprenyl-3-methyl-6-methoxy-1,4-benzoquinol hydroxylase
bin001	SOY3_bin001_01738	549	574	624	655	124.993	115.283	126.736	hypothetical protein
bin001	SOY3_bin001_01739	390	2612	2922	3006	800.671	759.924	818.755	hypothetical protein

bin001	SOY3_bin001_01740	261	517	721	682	236.808	280.188	277.571	FeoA domain protein
bin001	SOY3_bin001_01741	813	252	325	301	37.056	40.546	39.328	Imidazole glycerol phosphate synthase subunit HisF
bin001	SOY3_bin001_01742	606	128	137	154	25.251	22.930	26.995	Imidazole glycerol phosphate synthase subunit HisH 1
bin001	SOY3_bin001_01743	522	1680	1412	1274	384.755	274.359	259.256	hypothetical protein
bin001	SOY3_bin001_01744	366	1601	1469	1242	522.945	407.095	360.471	hypothetical protein
bin001	SOY3_bin001_01745	1509	2755	2314	2210	218.262	155.535	155.573	Phenylalanine--tRNA ligase alpha subunit
bin001	SOY3_bin001_01746	1647	2809	2483	2258	203.894	152.911	145.633	Phenylalanine--tRNA ligase beta subunit
bin001	SOY3_bin001_01747	381	619	791	691	194.228	210.575	192.656	Inosine-5'-monophosphate dehydrogenase
bin001	SOY3_bin001_01748	86	49	55	52	68.115	64.866	64.230	tRNA-Ser(gct)
bin001	SOY3_bin001_01749	2001	468	492	633	27.960	24.939	33.604	hypothetical protein
bin001	SOY3_bin001_01750	264	89	126	144	40.302	48.408	57.941	hypothetical protein
bin001	SOY3_bin001_01751	807	692	938	839	102.513	117.892	110.438	hypothetical protein
bin001	SOY3_bin001_01752	963	929	1533	1337	115.328	161.462	147.481	Phosphoribosylformylglycinamidine synthase 2
bin001	SOY3_bin001_01753	429	351	473	396	97.813	111.830	98.055	hypothetical protein
bin001	SOY3_bin001_01754	3411	3236	3733	3330	113.415	111.002	103.703	DNA polymerase II large subunit
bin001	SOY3_bin001_01755	405	263	372	410	77.633	93.163	107.537	hypothetical protein
bin001	SOY3_bin001_01756	414	89	102	146	25.700	24.989	37.461	hypothetical protein
bin001	SOY3_bin001_01757	870	121	115	122	16.627	13.407	14.896	Aerobic cobaltochelatase subunit CobN
bin001	SOY3_bin001_01758	969	121	115	130	14.928	12.037	14.251	Hemin-binding periplasmic protein HmuT precursor
bin001	SOY3_bin001_01759	1020	151	150	131	17.698	14.916	13.643	High-affinity heme uptake system protein IsdE precursor
bin001	SOY3_bin001_01760	1011	201	207	182	23.768	20.767	19.123	Vitamin B12-binding protein precursor
bin001	SOY3_bin001_01761	672	153	167	137	27.219	25.206	21.656	hypothetical protein
bin001	SOY3_bin001_01762	1221	189	192	202	18.505	15.949	17.574	Adenosylcobinamide amidohydrolase
bin001	SOY3_bin001_01763	1737	187	238	248	12.870	13.897	15.166	(R)-stereoselective amidase
bin001	SOY3_bin001_01764	1278	272	302	282	25.444	23.968	23.439	hypothetical protein
bin001	SOY3_bin001_01765	387	29	30	33	8.958	7.863	9.058	hypothetical protein
bin001	SOY3_bin001_01766	471	352	491	378	89.344	105.734	85.251	Thiamine-phosphate synthase
bin001	SOY3_bin001_01767	156	48	50	19	36.784	32.509	12.938	hypothetical protein
bin001	SOY3_bin001_01768	1560	438	464	404	33.566	30.168	27.510	UvrABC system protein C
bin001	SOY3_bin001_01769	111	16	23	11	17.232	21.016	10.527	hypothetical protein
bin001	SOY3_bin001_01770	471	59	77	57	14.975	16.582	12.855	hypothetical protein
bin001	SOY3_bin001_01771	1191	1131	1272	1342	113.526	108.325	119.694	3-hexulose-6-phosphate isomerase
bin001	SOY3_bin001_01772	546	470	399	443	102.908	74.120	86.187	hypothetical protein
bin001	SOY3_bin001_01773	1410	820	1099	1170	69.525	79.056	88.145	hypothetical protein
bin001	SOY3_bin001_01774	537	147	119	103	32.726	22.476	20.375	GtrA-like protein
bin001	SOY3_bin001_01775	666	471	504	475	84.546	76.756	75.762	Undecaprenyl-phosphate mannosyltransferase
bin001	SOY3_bin001_01776	1680	2885	3524	2968	205.297	212.756	187.665	Putative thiosulfate sulfurtransferase
bin001	SOY3_bin001_01777	1188	2060	2687	2231	207.299	229.407	199.486	Putative thiosulfate sulfurtransferase
bin001	SOY3_bin001_01778	393	657	806	649	199.857	208.016	175.421	DGC domain protein
bin001	SOY3_bin001_01779	237	95	111	109	47.920	47.504	48.855	Glutaredoxin
bin001	SOY3_bin001_01780	1557	797	1611	1244	61.195	104.945	84.871	hypothetical protein
bin001	SOY3_bin001_01781	210	117	191	146	66.606	92.251	73.852	hypothetical protein
bin001	SOY3_bin001_01782	738	332	499	386	53.781	68.580	55.560	Glycerol uptake facilitator protein
bin001	SOY3_bin001_01783	831	999	1133	1019	143.718	138.288	130.258	hypothetical protein
bin001	SOY3_bin001_01784	1101	673	679	632	73.076	62.551	60.976	GDP-mannose-dependent alpha-(1-6)-phosphatidylinositol dimannoside mannosyltransferase
bin001	SOY3_bin001_01785	387	21	16	12	6.487	4.193	3.294	hypothetical protein
bin001	SOY3_bin001_01786	492	153	224	210	37.177	46.178	45.340	hypothetical protein
bin001	SOY3_bin001_01787	516	727	829	745	168.434	162.952	153.369	Spore protein SP21
bin001	SOY3_bin001_01788	1209	1730	1835	1793	171.067	153.945	157.537	Riboflavin biosynthesis protein RibBA
bin001	SOY3_bin001_01789	1020	650	795	736	76.183	79.054	76.649	Cyclic pyranopterin monophosphate synthase
bin001	SOY3_bin001_01790	2097	231253	270569	259412	13183.614	13086.827	13140.789	methionyl-tRNA synthetase
bin001	SOY3_bin001_01791	1020	550	591	557	64.463	58.768	58.008	Histone deacetylase-like amidohydrolase
bin001	SOY3_bin001_01792	1314	2382	2771	2674	216.716	213.893	216.170	tRNA nucleotidyltransferase, second domain
bin001	SOY3_bin001_01793	546	1687	2283	2071	369.375	424.100	402.919	2'-5'-RNA ligase
bin001	SOY3_bin001_01794	1656	5342	6688	6152	385.646	409.629	394.626	Dihydroxy-acid dehydratase
bin001	SOY3_bin001_01795	156	143	264	271	109.586	171.646	184.533	hypothetical protein
bin001	SOY3_bin001_01796	267	191	333	348	85.520	126.499	138.452	hypothetical protein
bin001	SOY3_bin001_01797	216	113	202	202	62.542	94.853	99.341	hypothetical protein
bin001	SOY3_bin001_01798	2100	1151	1523	1387	65.524	73.559	70.160	Aerobic respiration control sensor protein ArcB
bin001	SOY3_bin001_01799	1293	1798	2161	1948	166.240	169.516	160.037	Aspartate aminotransferase
bin001	SOY3_bin001_01800	1797	1124	1358	1253	74.776	76.649	74.068	Glutamate 2,3-aminomutase
bin001	SOY3_bin001_01801	1005	123	213	178	14.631	21.497	18.814	Small-conductance mechanosensitive channel
bin001	SOY3_bin001_01802	1650	356	488	546	25.794	29.998	35.151	Trk system potassium uptake protein TrkG
bin001	SOY3_bin001_01803	408	2529	4094	3917	741.027	1017.753	1019.819	PXA domain protein
bin001	SOY3_bin001_01804	330	637	752	740	230.766	231.131	238.203	IS2 repressor TnpA
bin001	SOY3_bin001_01805	690	275	414	309	47.646	60.856	47.571	Glutamine--fructose-6-phosphate aminotransferase [isomerizing]
bin001	SOY3_bin001_01806	408	66	323	174	19.339	80.297	45.302	hypothetical protein

bin001	SOY3_bin001_01807	972	991	965	968	121.886	100.697	105.789	5-methyltetrahydropteroyltriglutamate--homocysteine methyltransferase
bin001	SOY3_bin001_01808	381	861	938	921	270.162	249.708	256.782	Threonine synthase
bin001	SOY3_bin001_01809	378	364	452	346	115.121	121.283	97.233	Ribosomal protein S6--L-glutamate ligase
bin001	SOY3_bin001_01810	492	300	321	233	72.896	66.175	50.306	Flagellin N-methylase
bin001	SOY3_bin001_01811	1146	591	739	500	61.652	65.406	46.346	Carboxylate-amine ligase YbdK
bin001	SOY3_bin001_01812	1137	801	902	760	84.220	80.464	71.004	Aspartate aminotransferase
bin001	SOY3_bin001_01813	192	91	102	81	56.661	53.883	44.814	hypothetical protein
bin001	SOY3_bin001_01814	261	131	198	161	60.003	76.945	65.526	hypothetical protein
bin001	SOY3_bin001_01815	186	179	196	176	115.050	106.880	100.515	hypothetical protein
bin001	SOY3_bin001_01816	288	259	369	332	107.511	129.954	122.455	hypothetical protein
bin001	SOY3_bin001_01817	306	144	216	173	56.258	71.596	60.056	hypothetical protein
bin001	SOY3_bin001_01818	423	371	485	551	104.853	116.294	138.370	Glutathione transport system permease protein GsiD
bin001	SOY3_bin001_01819	468	98	87	80	25.034	18.855	18.158	hypothetical protein
bin001	SOY3_bin001_01820	1668	645	614	613	46.228	37.336	39.039	putative outer membrane protein PmpB precursor
bin001	SOY3_bin001_01821	132	46	29	35	41.661	22.283	28.166	hypothetical protein
bin001	SOY3_bin001_01822	1296	2942	2281	3260	271.383	178.515	267.204	Periplasmic binding protein
bin001	SOY3_bin001_01823	201	1320	1462	1880	785.097	737.745	993.554	putative RNA-binding protein
bin001	SOY3_bin001_01824	474	3038	3355	4421	766.223	717.908	990.768	CobQ/CobB/MinD/ParA nucleotide binding domain protein
bin001	SOY3_bin001_01825	309	85	130	86	32.886	42.672	29.564	30S ribosomal protein S19e
bin001	SOY3_bin001_01826	1641	1188	1344	1313	86.547	83.070	84.994	ATP-dependent DNA ligase
bin001	SOY3_bin001_01827	855	434	567	564	60.683	67.262	70.072	Radical SAM superfamily protein
bin001	SOY3_bin001_01828	135	1313	1399	1457	1162.724	1051.087	1146.451	hypothetical protein
bin001	SOY3_bin001_01829	759	164	255	271	25.831	34.076	37.928	hypothetical protein
bin001	SOY3_bin001_01830	294	155	218	231	63.027	75.208	83.463	glutaredoxin 3
bin001	SOY3_bin001_01831	534	291	430	471	65.147	81.674	93.694	Reverse rubrerythrin-2
bin001	SOY3_bin001_01832	2490	977	1171	1154	46.907	47.699	49.231	Copper-exporting P-type ATPase A
bin001	SOY3_bin001_01833	180	33	38	46	21.917	21.412	27.147	Copper-transporting P-type ATPase
bin001	SOY3_bin001_01834	216	90	114	103	49.812	53.531	50.654	hypothetical protein
bin001	SOY3_bin001_01835	684	841	1099	1129	146.989	162.966	175.335	Phosphoserine phosphatase
bin001	SOY3_bin001_01836	249	813	1062	925	390.334	432.593	394.614	18 kDa heat shock protein
bin001	SOY3_bin001_01837	1746	3771	4502	3943	258.201	261.527	239.890	Glycine--tRNA ligase
bin001	SOY3_bin001_01838	666	1101	1203	1060	197.633	183.209	169.068	hypothetical protein
bin001	SOY3_bin001_01839	648	7258	10048	10209	1339.021	1572.749	1673.547	Adenylate kinase
bin001	SOY3_bin001_01840	945	259	333	286	32.765	35.741	32.149	Transposase DDE domain protein
bin001	SOY3_bin001_01841	222	26	25	28	14.001	11.422	13.398	hypothetical protein
bin001	SOY3_bin001_01842	495	9	26	43	2.174	5.327	9.228	Spermine/spermidine acetyltransferase
bin001	SOY3_bin001_01843	1443	176	155	245	14.581	10.895	18.036	Beta-lactamase precursor
bin001	SOY3_bin001_01844	2079	444	424	496	25.531	20.686	25.343	Prolyl tripeptidyl peptidase precursor
bin001	SOY3_bin001_01845	597	39	32	52	7.810	5.437	9.252	Fatty acid metabolism regulator protein
bin001	SOY3_bin001_01846	1896	1234	1697	1623	77.808	90.782	90.931	Glutathione import ATP-binding protein GsiA
bin001	SOY3_bin001_01847	261	51	73	37	23.360	28.369	15.059	hypothetical protein
bin001	SOY3_bin001_01848	489	81	145	92	19.803	30.076	19.985	TspO/MBR family protein
bin001	SOY3_bin001_01849	1056	168	357	160	19.019	34.289	16.095	Vitamin B12-binding protein precursor
bin001	SOY3_bin001_01850	1191	143	308	144	14.354	26.230	12.843	Vitamin B12-binding protein precursor
bin001	SOY3_bin001_01851	612	2042	2230	2096	398.887	369.580	363.806	cell division protein FtsZ
bin001	SOY3_bin001_01852	891	1050	1069	1017	140.883	121.690	121.248	GXGXG motif protein
bin001	SOY3_bin001_01853	1104	2222	3151	2630	240.614	289.490	253.056	hypothetical protein
bin001	SOY3_bin001_01854	468	1025	1591	1357	261.832	344.809	308.009	hypothetical protein
bin001	SOY3_bin001_01855	1284	2121	2710	2187	197.479	214.072	180.931	Phosphomethylpyrimidine synthase
bin001	SOY3_bin001_01856	327	446	434	436	163.054	134.616	141.634	hypothetical protein
bin001	SOY3_bin001_01857	1968	515	447	444	31.284	23.038	23.966	DNA polymerase II
bin001	SOY3_bin001_01858	417	250	252	237	71.672	61.294	60.373	Transcriptional activator protein CzcR
bin001	SOY3_bin001_01859	438	326	378	354	88.979	87.533	85.854	Acylphosphatase
bin001	SOY3_bin001_01860	774	605	902	902	93.446	118.201	123.793	Exodeoxyribonuclease
bin001	SOY3_bin001_01861	414	506	582	505	146.115	142.586	129.575	hypothetical protein
bin001	SOY3_bin001_01862	1665	2117	3071	2546	152.003	187.077	162.433	hypothetical protein
bin001	SOY3_bin001_01863	366	288	693	414	94.071	192.047	120.157	hypothetical protein
bin001	SOY3_bin001_01864	564	1055	1532	1470	223.624	275.508	276.865	HTH-type transcriptional regulator Xre
bin001	SOY3_bin001_01865	519	345	463	444	79.469	90.483	90.875	PIN domain protein
bin001	SOY3_bin001_01866	210	107	135	129	60.913	65.203	65.253	hypothetical protein
bin001	SOY3_bin001_01867	357	89	65	81	29.804	18.467	24.102	hypothetical protein
bin001	SOY3_bin001_01868	405	306	547	477	90.326	136.989	125.110	hypothetical protein
bin001	SOY3_bin001_01869	522	485	556	502	111.075	108.034	102.156	hypothetical protein
bin001	SOY3_bin001_01870	636	337	413	394	63.346	65.864	65.807	Protein-L-isoaspartate O-methyltransferase
bin001	SOY3_bin001_01871	564	280	406	353	59.351	73.013	66.485	translation initiation factor IF-2 subunit beta
bin001	SOY3_bin001_01872	213	19	59	32	10.664	28.095	15.959	hypothetical protein
bin001	SOY3_bin001_01873	741	502	820	655	80.990	112.241	93.897	hypothetical protein

bin001	SOY3_bin001_01874	687	549	710	717	95.535	104.823	110.864	putative N-glycosylase/DNA lyase
bin001	SOY3_bin001_01875	1977	2423	3560	3364	146.518	182.641	180.750	UvrABC system protein B
bin001	SOY3_bin001_01876	1395	791	848	820	67.787	61.656	62.441	Multidrug export protein MepA
bin001	SOY3_bin001_01877	870	900	1126	1166	123.671	131.273	142.367	Serine/threonine-protein kinase AfsK
bin001	SOY3_bin001_01878	1992	1170	1201	1208	70.217	61.152	64.418	hypothetical protein
bin001	SOY3_bin001_01879	3171	102	229	119	3.845	7.325	3.986	ATP-dependent helicase/deoxyribonuclease subunit B
bin001	SOY3_bin001_01880	3021	102	204	103	4.036	6.849	3.622	ATP-dependent DNA helicase PcrA
bin001	SOY3_bin001_01881	2226	43	111	46	2.309	5.058	2.195	ATP-dependent RNA helicase DbpA
bin001	SOY3_bin001_01882	1329	62	128	76	5.577	9.769	6.075	Mitochondrial ribosomal death-associated protein 3
bin001	SOY3_bin001_01883	2400	202	403	213	10.062	17.031	9.428	Tellurite resistance protein TerB
bin001	SOY3_bin001_01884	216	9	20	13	4.981	9.391	6.393	hypothetical protein
bin001	SOY3_bin001_01885	633	12826	13274	12531	2422.329	2126.928	2102.867	30S ribosomal protein S3Ae
bin001	SOY3_bin001_01886	396	1727	1365	1293	521.366	349.617	346.843	hypothetical protein
bin001	SOY3_bin001_01887	756	1536	1432	1389	242.893	192.122	195.169	S-methyl-5'-thioinosine phosphorylase
bin001	SOY3_bin001_01888	1017	1593	2266	2038	187.258	225.992	212.869	Methylthioribose-1-phosphate isomerase
bin001	SOY3_bin001_01889	831	458	568	552	65.889	69.327	70.562	Phosphate-binding protein PstS 1 precursor
bin001	SOY3_bin001_01890	1083	198	172	179	21.857	16.108	17.557	Hemin transport system permease protein HmuU
bin001	SOY3_bin001_01891	480	66	61	67	16.438	12.890	14.827	Intermediate filament tail domain protein
bin001	SOY3_bin001_01892	924	107	103	89	13.844	11.306	10.232	Sirohydrochlorin cobaltochelatase CbiKP precursor
bin001	SOY3_bin001_01893	261	123	244	183	56.339	94.821	74.480	hypothetical protein
bin001	SOY3_bin001_01894	492	985	1433	1361	239.341	295.417	293.848	hypothetical protein
bin001	SOY3_bin001_01895	438	335	330	332	91.436	76.418	80.518	hypothetical protein
bin001	SOY3_bin001_01896	372	763	1396	1238	245.204	380.625	353.515	hypothetical protein
bin001	SOY3_bin001_01897	255	513	608	586	240.504	241.834	244.111	hypothetical protein
bin001	SOY3_bin001_01898	669	1042	1141	1086	186.203	172.987	172.438	Peptidase family M50
bin001	SOY3_bin001_01899	228	34	61	43	17.827	27.136	20.034	hypothetical protein
bin001	SOY3_bin001_01900	738	203	260	162	32.884	35.733	23.318	putative ABC transporter ATP-binding protein YbhF
bin001	SOY3_bin001_01901	738	110	119	98	17.819	16.355	14.106	ABC-2 type transporter
bin001	SOY3_bin001_01902	1851	561	828	838	36.233	45.371	48.091	Carbon monoxide dehydrogenase 1
bin001	SOY3_bin001_01903	246	157	150	161	76.297	61.846	69.522	YcfA-like protein
bin001	SOY3_bin001_01904	342	166	206	213	58.027	61.094	66.158	hypothetical protein
bin001	SOY3_bin001_01905	774	109	66	53	16.836	8.649	7.274	photosystem I assembly protein Ycf3
bin001	SOY3_bin001_01906	396	127	182	179	38.340	46.616	48.016	hypothetical protein
bin001	SOY3_bin001_01907	816	245	308	274	35.894	38.284	35.669	hypothetical protein
bin001	SOY3_bin001_01908	417	23	22	16	6.594	5.351	4.076	hypothetical protein
bin001	SOY3_bin001_01909	489	351	296	349	85.811	61.396	75.813	hypothetical protein
bin001	SOY3_bin001_01910	1170	359	275	251	36.682	23.840	22.789	Integrase core domain protein
bin001	SOY3_bin001_01911	147	9	3	6	7.319	2.070	4.336	hypothetical protein
bin001	SOY3_bin001_01912	1077	908	987	1235	100.790	92.951	121.809	hypothetical protein
bin001	SOY3_bin001_01913	288	87	96	141	36.114	33.809	52.006	hypothetical protein
bin001	SOY3_bin001_01914	1656	34454	18879	17119	2487.281	1156.308	1098.115	60 kDa chaperonin
bin001	SOY3_bin001_01915	1011	5299	6285	5717	626.597	630.535	600.685	ATP-dependent zinc metalloprotease FtsH
bin001	SOY3_bin001_01916	486	300	392	352	73.796	81.810	76.937	O-acetyl-ADP-ribose deacetylase
bin001	SOY3_bin001_01917	834	963	787	761	138.040	95.711	96.928	putative methyltransferase YcgJ
bin001	SOY3_bin001_01918	564	65	219	131	13.778	39.384	24.673	hypothetical protein
bin001	SOY3_bin001_01919	375	158	203	168	50.370	54.906	47.589	hypothetical protein
bin001	SOY3_bin001_01920	1425	1266	1520	1376	106.210	108.189	102.573	NADP-reducing hydrogenase subunit HndC
bin001	SOY3_bin001_01921	1887	2475	2992	2534	156.801	160.822	142.648	NADP-reducing hydrogenase subunit HndC
bin001	SOY3_bin001_01922	462	528	942	626	136.627	206.806	143.934	NADP-reducing hydrogenase subunit HndA
bin001	SOY3_bin001_01923	393	241	89	85	73.311	22.970	22.975	hypothetical protein
bin001	SOY3_bin001_01924	255	1258	399	349	589.774	158.704	145.383	hypothetical protein
bin001	SOY3_bin001_01925	474	4982	1216	1139	1256.524	260.202	255.256	hypothetical protein
bin001	SOY3_bin001_01926	363	110	165	137	36.227	46.103	40.091	hypothetical protein
bin001	SOY3_bin001_01927	99	185	283	253	223.399	289.939	271.466	hypothetical protein
bin001	SOY3_bin001_01928	195	283	332	307	173.499	172.686	167.237	hypothetical protein
bin001	SOY3_bin001_01929	258	265	231	214	122.792	90.813	88.110	hypothetical protein
bin001	SOY3_bin001_01930	669	198	264	265	35.382	40.025	42.077	hypothetical protein
bin001	SOY3_bin001_01931	186	2	10	6	1.285	5.453	3.427	hypothetical protein
bin001	SOY3_bin001_01932	945	157	138	130	19.862	14.812	14.613	Nucleotidyltransferase domain protein
bin001	SOY3_bin001_01933	1791	462	658	593	30.838	37.264	35.171	Endonuclease/Exonuclease/phosphatase family protein
bin001	SOY3_bin001_01934	282	180	273	215	76.308	98.190	80.988	hypothetical protein
bin001	SOY3_bin001_01935	390	60	110	86	18.392	28.608	23.424	hypothetical protein
bin001	SOY3_bin001_01936	858	777	761	685	108.263	89.961	84.807	hypothetical protein
bin001	SOY3_bin001_01937	1668	2008	2541	2193	143.917	154.512	139.660	Arginine--tRNA ligase
bin001	SOY3_bin001_01938	1224	884	964	958	86.341	79.882	83.141	hypothetical protein
bin001	SOY3_bin001_01939	270	322	305	309	142.573	114.575	121.569	Glutathione transport system permease protein GsiD
bin001	SOY3_bin001_01940	276	94	69	69	40.716	25.357	26.556	hypothetical protein

bin001	SOY3_bin001_01941	231	75	69	64	38.815	30.296	29.431	hypothetical protein
bin001	SOY3_bin001_01942	237	820	1081	1078	413.629	462.628	483.170	hypothetical protein
bin001	SOY3_bin001_01943	1419	2852	3065	2947	240.277	219.080	220.611	Glutamine-dependent NAD(+) synthetase
bin001	SOY3_bin001_01944	1302	1801	1917	2001	165.367	149.336	163.255	IclR helix-turn-helix domain protein
bin001	SOY3_bin001_01945	597	721	1615	767	144.380	274.380	136.474	hypothetical protein
bin001	SOY3_bin001_01946	459	380	370	472	98.973	81.761	109.234	GDP-mannose-dependent alpha-(1-6)-phosphatidylinositol monomannoside mannosyltransferase
bin001	SOY3_bin001_01947	978	914	1059	974	111.726	109.828	105.791	hypothetical protein
bin001	SOY3_bin001_01948	1680	7641	8350	8166	543.734	504.118	516.333	preprotein translocase subunit SecD
bin001	SOY3_bin001_01949	894	4269	5109	4752	570.866	579.633	564.636	preprotein translocase subunit SecF
bin001	SOY3_bin001_01950	1260	4586	4503	4164	435.120	362.482	351.051	3-hexulose-6-phosphate synthase
bin001	SOY3_bin001_01951	912	1587	1959	1827	208.031	217.868	212.801	Diaminopimelate epimerase
bin001	SOY3_bin001_01952	441	646	722	684	175.121	166.056	164.758	hypothetical protein
bin001	SOY3_bin001_01953	384	441	615	611	137.294	162.442	169.021	ssDNA exonuclease RecJ
bin001	SOY3_bin001_01954	345	20	43	16	6.930	12.642	4.926	hypothetical protein
bin001	SOY3_bin001_01955	321	48	65	42	17.876	20.538	13.899	Helix-turn-helix domain protein
bin001	SOY3_bin001_01956	225	20	23	11	10.627	10.368	5.193	hypothetical protein
bin001	SOY3_bin001_01957	453	309	489	288	81.547	109.488	67.534	Alkyl hydroperoxide reductase AhpD
bin001	SOY3_bin001_01958	924	1026	1054	899	132.746	115.697	103.352	Methyl-coenzyme M reductase operon protein C
bin001	SOY3_bin001_01959	573	1030	1126	1108	214.896	199.314	205.407	hypothetical protein
bin001	SOY3_bin001_01960	1227	3006	3530	3322	292.880	291.800	287.597	Activator of (R)-2-hydroxyglutaryl-CoA dehydratase
bin001	SOY3_bin001_01961	438	1337	1529	1538	364.924	354.069	373.003	hypothetical protein
bin001	SOY3_bin001_01962	414	1367	1474	1566	394.742	361.120	401.810	hypothetical protein
bin001	SOY3_bin001_01963	1524	4895	5730	5407	383.984	381.351	376.878	hypothetical protein
bin001	SOY3_bin001_01964	1602	4082	5453	5007	304.618	345.245	332.005	Glycine betaine transport ATP-binding protein OpuAA
bin001	SOY3_bin001_01965	510	1241	1959	1728	290.902	389.600	359.918	NADPH-dependent oxidoreductase
bin001	SOY3_bin001_01966	567	1058	1432	1294	223.074	256.162	242.427	Flavoredoxin
bin001	SOY3_bin001_01967	312	59	81	75	22.607	26.332	25.535	hypothetical protein
bin001	SOY3_bin001_01968	453	367	394	333	96.853	88.217	78.086	hypothetical protein
bin001	SOY3_bin001_01969	1551	351	395	337	27.055	25.831	23.081	Thiol:disulfide interchange protein DsbD precursor
bin001	SOY3_bin001_01970	573	287	326	238	59.879	57.706	44.122	hypothetical protein
bin001	SOY3_bin001_01971	324	111	137	93	40.957	42.887	30.491	hypothetical protein
bin001	SOY3_bin001_01972	4089	5741	6953	7657	167.848	172.469	198.917	Eco57I restriction-modification methylase
bin001	SOY3_bin001_01973	1128	867	984	1019	91.887	88.479	95.961	Adenosine monophosphate-protein transferase SoFic
bin001	SOY3_bin001_01974	3246	1774	1857	2028	65.336	58.025	66.367	ATP-dependent RNA helicase RhlB
bin001	SOY3_bin001_01975	1020	54	61	55	6.329	6.066	5.728	Vitamin B12-binding protein precursor
bin001	SOY3_bin001_01976	627	350	327	322	66.734	52.897	54.553	Precorrin-8X methylmutase
bin001	SOY3_bin001_01977	1548	335	363	326	25.871	23.784	22.371	Peptidase C13 family protein
bin001	SOY3_bin001_01978	498	56	90	86	13.443	18.330	18.344	hypothetical protein
bin001	SOY3_bin001_01979	2184	23	18	19	1.259	0.836	0.924	hypothetical protein
bin001	SOY3_bin001_01980	1206	330	371	380	32.712	31.202	33.471	hypothetical protein
bin001	SOY3_bin001_01981	780	447	806	715	68.511	104.808	97.374	hypothetical protein
bin001	SOY3_bin001_01982	153	70	82	80	54.696	54.360	55.543	hypothetical protein
bin001	SOY3_bin001_01983	129	57	69	50	52.824	54.252	41.173	hypothetical protein
bin001	SOY3_bin001_01984	630	219	314	219	41.557	50.553	36.926	hypothetical protein
bin001	SOY3_bin001_01985	231	94	155	127	48.648	68.057	58.401	YcfA-like protein
bin001	SOY3_bin001_01986	228	187	307	271	98.051	136.571	126.260	hypothetical protein
bin001	SOY3_bin001_01987	345	131	158	158	45.394	46.451	48.648	hypothetical protein
bin001	SOY3_bin001_01988	540	865	756	718	191.500	141.998	141.241	UvrABC system protein A
bin001	SOY3_bin001_01989	333	24	31	18	8.616	9.442	5.742	Alkyl hydroperoxide reductase AhpD
bin001	SOY3_bin001_01990	1092	200	174	171	21.895	16.161	16.634	hypothetical protein
bin001	SOY3_bin001_01991	165	872	985	1097	631.798	605.490	706.240	hypothetical protein
bin001	SOY3_bin001_01992	300	11	68	32	4.383	22.990	11.331	hypothetical protein
bin001	SOY3_bin001_01993	789	111	159	108	16.819	20.440	14.540	hypothetical protein
bin001	SOY3_bin001_01994	1161	749	882	788	77.125	77.053	72.098	Lipoprotein-releasing system transmembrane protein LolE
bin001	SOY3_bin001_01995	390	342	404	342	104.835	105.068	93.152	Macrolide export ATP-binding/permease protein MacB
bin001	SOY3_bin001_01996	1338	976	955	982	87.205	72.394	77.962	hypothetical protein
bin001	SOY3_bin001_01997	873	1887	1902	1896	258.406	220.979	230.703	ATP phosphoribosyltransferase
bin001	SOY3_bin001_01998	942	206	433	303	26.143	46.622	34.168	UDP-2,3-diacylglucosamine hydrolase
bin001	SOY3_bin001_01999	213	168	253	190	94.292	120.475	94.755	hypothetical protein
bin001	SOY3_bin001_02000	1227	2101	2784	2698	204.704	230.133	233.575	hypothetical protein
bin001	SOY3_bin001_02001	645	1849	2316	2089	342.707	364.195	344.040	hypothetical protein
bin001	SOY3_bin001_02002	558	944	999	1031	202.248	181.588	196.270	Orotate phosphoribosyltransferase
bin001	SOY3_bin001_02003	795	547	529	589	82.256	67.491	78.701	Calcineurin-like phosphoesterase superfamily domain protein
bin001	SOY3_bin001_02004	465	15	25	17	3.856	5.453	3.884	hypothetical protein
bin001	SOY3_bin001_02005	351	107	144	140	36.444	41.611	42.369	Toxin MazF
bin001	SOY3_bin001_02006	429	366	560	451	101.993	132.399	111.673	hypothetical protein
bin001	SOY3_bin001_02007	2157	331	667	320	18.345	31.364	15.759	Regulatory protein AfsR

bin001	SOY3_bin001_02008	186	22	26	14	14.140	14.178	7.995	hypothetical protein
bin001	SOY3_bin001_02009	1146	500	929	444	52.159	82.222	41.156	Antilisterial bacteriocin subtilosin biosynthesis protein AlbA
bin001	SOY3_bin001_02010	330	6	23	20	2.174	7.069	6.438	Divalent metal cation transporter MntH
bin001	SOY3_bin001_02011	1209	223	446	232	22.051	37.417	20.384	Magnesium transport protein CorA
bin001	SOY3_bin001_02012	906	91	202	101	12.008	22.614	11.842	Small-conductance mechanosensitive channel
bin001	SOY3_bin001_02013	570	625	577	556	131.084	102.673	103.617	putative GTP-binding protein EngB
bin001	SOY3_bin001_02014	585	1081	1227	1257	220.910	212.737	228.249	Flagellin N-methylase
bin001	SOY3_bin001_02015	945	937	1010	967	118.537	108.404	108.699	putative phycocyanin operon protein Z
bin001	SOY3_bin001_02016	909	711	1012	854	93.509	112.920	99.798	molybdenum cofactor biosynthesis protein A
bin001	SOY3_bin001_02017	852	717	893	880	100.606	106.308	109.717	Septum site-determining protein MinD
bin001	SOY3_bin001_02018	1023	3820	4675	4054	446.409	463.512	420.957	S-adenosylmethionine synthetase
bin001	SOY3_bin001_02019	1044	135	175	169	15.459	17.002	17.196	hypothetical protein
bin001	SOY3_bin001_02020	1227	298	390	379	29.035	32.238	32.811	putative inner membrane protein
bin001	SOY3_bin001_02021	273	288	246	188	126.118	91.396	73.152	hypothetical protein
bin001	SOY3_bin001_02022	465	651	765	762	167.368	166.864	174.073	Outer membrane efflux protein
bin001	SOY3_bin001_02023	675	496	533	521	87.846	80.090	81.991	Flagellin N-methylase
bin001	SOY3_bin001_02024	696	450	579	571	77.295	84.377	87.148	Sugar phosphatase YidA
bin001	SOY3_bin001_02025	744	581	685	762	93.357	93.384	108.796	Ribonuclease Z
bin001	SOY3_bin001_02026	336	235	311	336	83.613	93.881	106.226	hypothetical protein
bin001	SOY3_bin001_02027	129	68	74	51	63.018	58.183	41.996	hypothetical protein
bin001	SOY3_bin001_02028	699	1194	1380	1227	204.208	200.243	186.465	Ribose-5-phosphate isomerase A
bin001	SOY3_bin001_02029	1611	3394	3889	3716	251.862	244.848	245.025	Phenylalanine--tRNA ligase alpha subunit
bin001	SOY3_bin001_02030	627	1551	1634	1607	295.726	264.326	272.256	Translin family protein
bin001	SOY3_bin001_02031	777	2512	2838	2694	386.495	370.464	368.304	putative endonuclease 4
bin001	SOY3_bin001_02032	501	1805	2145	1962	430.710	434.254	415.998	hypothetical protein
bin001	SOY3_bin001_02033	1242	6259	7578	7141	602.461	618.853	610.755	peptide chain release factor 1
bin001	SOY3_bin001_02034	2361	735	2712	1558	37.217	116.506	70.097	photosystem I assembly protein Ycf3
bin001	SOY3_bin001_02035	597	184	344	248	36.846	58.444	44.127	Ribosomal protein L11 methyltransferase
bin001	SOY3_bin001_02036	561	3872	3684	3702	825.122	666.057	700.976	exosome complex RNA-binding protein Csl4
bin001	SOY3_bin001_02037	276	1730	1561	1580	749.346	573.652	608.104	DNA-directed RNA polymerase subunit L
bin001	SOY3_bin001_02038	717	607	578	598	101.208	81.764	88.596	peptide chain release factor 1
bin001	SOY3_bin001_02039	846	764	813	763	107.961	97.471	95.804	Leucine rich repeat variant
bin001	SOY3_bin001_02040	1062	880	1123	1084	99.061	107.253	108.426	Aminodeoxyfutalosine synthase
bin001	SOY3_bin001_02041	537	409	515	474	91.053	97.272	93.764	rRNA (cytosine-C(5)-)-methyltransferase RsmF
bin001	SOY3_bin001_02042	906	200	263	231	26.390	29.443	27.084	Ribosomal RNA small subunit methyltransferase F
bin001	SOY3_bin001_02043	288	2183	2191	1970	906.164	771.622	726.614	23S rRNA (uracil-C(5))-methyltransferase RlmCD
bin001	SOY3_bin001_02044	228	1659	1446	1308	869.875	643.262	609.400	23S rRNA (uracil-C(5))-methyltransferase RlmCD
bin001	SOY3_bin001_02045	717	2275	2621	2345	379.322	370.768	347.419	DNA-directed RNA polymerase subunit P
bin001	SOY3_bin001_02046	1317	650	897	816	59.003	69.081	65.816	hypothetical protein
bin001	SOY3_bin001_02047	579	1023	963	895	211.224	168.695	164.200	hypothetical protein
bin001	SOY3_bin001_02048	831	1755	1765	1508	252.477	215.426	192.766	hypothetical protein
bin001	SOY3_bin001_02049	264	391	426	362	177.059	163.667	145.658	hypothetical protein
bin001	SOY3_bin001_02050	642	1241	1222	1103	231.091	193.059	182.503	hypothetical protein
bin001	SOY3_bin001_02051	627	51	44	72	9.724	7.118	12.198	hypothetical protein
bin001	SOY3_bin001_02052	111	2	0	1	2.154	0.000	0.957	hypothetical protein
bin001	SOY3_bin001_02053	432	960	1027	1312	265.664	241.125	322.612	hypothetical protein
bin001	SOY3_bin001_02054	513	404	371	535	94.148	73.352	110.781	hypothetical protein
bin001	SOY3_bin001_02055	963	37	53	55	4.593	5.582	6.067	hypothetical protein
bin001	SOY3_bin001_02056	609	4	13	11	0.785	2.165	1.919	CRISPR-associated endoribonuclease Cas2
bin001	SOY3_bin001_02057	1140	1674	1814	1849	175.548	161.394	172.291	hypothetical protein
bin001	SOY3_bin001_02058	648	2215	3131	2593	408.643	490.075	425.067	Chromosome partition protein Smc
bin001	SOY3_bin001_02059	1536	2087	2683	2411	162.434	177.168	166.738	4-diphosphocytidyl-2-C-methyl-D-erythritol kinase
bin001	SOY3_bin001_02060	1773	189	276	250	12.744	15.789	14.978	Sodium-dependent dicarboxylate transporter SdcS
bin001	SOY3_bin001_02061	3228	3629	4509	3974	134.400	141.678	130.775	Signal transduction histidine-protein kinase BarA
bin001	SOY3_bin001_02062	1011	357	390	339	42.215	39.126	35.619	hypothetical protein
bin001	SOY3_bin001_02063	735	184	191	166	29.928	26.357	23.991	hypothetical protein
bin001	SOY3_bin001_02064	897	674	1027	888	89.828	116.127	105.160	Cytidylyltransferase family protein
bin001	SOY3_bin001_02065	1392	443	728	508	38.046	53.045	38.766	soluble pyridine nucleotide transhydrogenase
bin001	SOY3_bin001_02066	1170	1833	1801	2376	187.293	156.129	215.720	Fosmidomycin resistance protein
bin001	SOY3_bin001_02067	1038	2902	4055	5402	334.230	396.231	552.824	FmdE, Molybdenum formylmethanofuran dehydrogenase operon
bin001	SOY3_bin001_02068	570	6022	5905	6180	1263.023	1050.751	1151.711	Flavin reductase like domain protein
bin001	SOY3_bin001_02069	750	7424	8109	8045	1183.375	1096.632	1139.448	Septum site-determining protein MinD
bin001	SOY3_bin001_02070	1590	15856	16547	17367	1192.181	1055.545	1160.266	Periplasmic dipeptide transport protein precursor
bin001	SOY3_bin001_02071	174	6	12	3	4.122	6.995	1.831	hypothetical protein
bin001	SOY3_bin001_02072	873	2353	2450	2528	322.221	284.647	307.604	putative ABC transporter ATP-binding protein YbhF
bin001	SOY3_bin001_02073	807	1153	1132	1111	170.805	142.275	146.241	Inner membrane transport permease YadH
bin001	SOY3_bin001_02074	246	1159	1739	1456	563.240	717.000	628.718	F420H2 dehydrogenase subunit FpoO

bin001	SOY3_bin001_02075	402	418	485	452	124.307	122.369	119.438	OB-fold nucleic acid binding domain protein
bin001	SOY3_bin001_02076	1245	2216	2805	2404	212.787	228.517	205.114	Lysine 6-dehydrogenase
bin001	SOY3_bin001_02077	759	1031	1073	981	162.391	143.388	137.296	Adenosyl-chloride synthase
bin001	SOY3_bin001_02078	213	402	435	435	225.627	207.140	216.940	hypothetical protein
bin001	SOY3_bin001_02079	216	573	717	736	317.137	336.682	361.954	hypothetical protein
bin001	SOY3_bin001_02080	1254	747	785	723	71.215	63.493	61.245	Chagasin family peptidase inhibitor I42
bin001	SOY3_bin001_02081	1251	428	439	268	40.901	35.593	22.757	Transposase IS116/IS110/IS902 family protein
bin001	SOY3_bin001_02082	426	15	22	25	4.209	5.238	6.234	hypothetical protein
bin001	SOY3_bin001_02083	1341	29	35	24	2.585	2.647	1.901	hypothetical protein
bin001	SOY3_bin001_02084	933	125	79	69	16.017	8.588	7.856	hypothetical protein
bin001	SOY3_bin001_02085	1176	276	220	162	28.057	18.974	14.633	hypothetical protein
bin001	SOY3_bin001_02086	1503	242	304	276	19.249	20.515	19.507	Na(+)/H(+) antiporter subunit A
bin001	SOY3_bin001_02087	1263	56	84	54	5.301	6.746	4.542	hypothetical protein
bin001	SOY3_bin001_02088	162	13	9	14	9.593	5.635	9.180	hypothetical protein
bin001	SOY3_bin001_02089	312	27	19	18	10.346	6.177	6.128	hypothetical protein
bin001	SOY3_bin001_02090	1722	151	131	123	10.483	7.716	7.588	Na(+)/H(+) antiporter subunit A
bin001	SOY3_bin001_02091	318	633	806	747	237.970	257.077	249.530	hypothetical protein
bin001	SOY3_bin001_02092	1029	30440	36593	35687	3536.509	3606.927	3684.041	V-type ATP synthase subunit I
bin001	SOY3_bin001_02093	267	11731	15871	15385	5252.539	6029.035	6120.911	ATP synthase subunit c
bin001	SOY3_bin001_02094	549	24351	30572	29722	5302.614	5648.151	5750.896	V-type ATP synthase subunit E
bin001	SOY3_bin001_02095	1050	36096	44461	44528	4109.749	4294.817	4504.781	V-type ATP synthase subunit C
bin001	SOY3_bin001_02096	300	11247	14842	15272	4481.888	5017.945	5407.599	V-type ATP synthase subunit F
bin001	SOY3_bin001_02097	1731	54868	68244	69453	3789.375	3998.731	4262.101	V-type ATP synthase alpha chain
bin001	SOY3_bin001_02098	150	3529	4256	4088	2812.587	2877.830	2895.006	hypothetical protein
bin001	SOY3_bin001_02099	138	34	61	47	29.454	44.834	36.178	hypothetical protein
bin001	SOY3_bin001_02100	402	1773	1682	1558	527.264	424.380	411.691	hypothetical protein
bin001	SOY3_bin001_02101	1416	47128	61597	57379	3978.884	4412.158	4304.469	Corrinoid/iron-sulfur protein large subunit
bin001	SOY3_bin001_02102	1353	37944	48081	44778	3352.670	3604.379	3515.578	Corrinoid/iron-sulfur protein small subunit
bin001	SOY3_bin001_02103	759	25359	32616	30192	3994.256	4358.567	4225.518	Septum site-determining protein MinD
bin001	SOY3_bin001_02104	174	4308	5703	5172	2959.865	3324.366	3157.469	hypothetical protein
bin001	SOY3_bin001_02105	171	8605	12995	11188	6015.896	7707.880	6950.022	acetyl-CoA decarbonylase/synthase complex subunit beta
bin001	SOY3_bin001_02106	1212	46777	63248	54121	4613.975	5292.964	4743.436	Carbon monoxide dehydrogenase/acetyl-CoA synthase subunit alpha
bin001	SOY3_bin001_02107	723	171	259	190	28.275	36.334	27.915	3-oxoacyl-[acyl-carrier-protein] reductase FabG
bin001	SOY3_bin001_02108	1203	236	171	167	23.453	14.417	14.746	Aminopeptidase YwaD precursor
bin001	SOY3_bin001_02109	780	147	138	121	22.530	17.945	16.479	Arylesterase
bin001	SOY3_bin001_02110	717	261	282	305	43.518	39.892	45.187	putative S-adenosyl-L-methionine-dependent methyltransferase TehB
bin001	SOY3_bin001_02111	333	44	59	45	15.796	17.971	14.355	Multidrug transporter EmrE
bin001	SOY3_bin001_02112	2499	835	875	807	39.945	35.514	34.303	Cytochrome c-type biogenesis protein CcmH precursor
bin001	SOY3_bin001_02113	528	131	134	133	29.661	25.741	26.758	photosystem I assembly protein Ycf3
bin001	SOY3_bin001_02114	216	410	524	459	226.921	246.055	225.730	hypothetical protein
bin001	SOY3_bin001_02115	2310	3520	4035	3704	182.170	177.168	170.329	ATP-dependent DNA helicase RecQ
bin001	SOY3_bin001_02116	354	181	199	151	61.125	57.017	45.311	Plasmid stabilisation system protein
bin001	SOY3_bin001_02117	189	1206	1225	1106	762.836	657.399	621.617	hypothetical protein
bin001	SOY3_bin001_02118	213	122	247	163	68.474	117.618	81.290	2-ketoisovalerate ferredoxin reductase
bin001	SOY3_bin001_02119	852	370	763	571	51.917	90.832	71.191	2-oxoglutarate oxidoreductase subunit KorB
bin001	SOY3_bin001_02120	162	645	1110	946	475.982	694.965	620.306	Rubredoxin
bin001	SOY3_bin001_02121	525	1423	2273	1909	324.034	439.132	386.257	Ferritin
bin001	SOY3_bin001_02122	438	1936	2380	2125	528.417	551.135	515.365	Secreted repeat of unknown function
bin001	SOY3_bin001_02123	429	284	304	322	79.142	71.874	79.731	hypothetical protein
bin001	SOY3_bin001_02124	438	110	122	86	30.024	28.251	20.857	hypothetical protein
bin001	SOY3_bin001_02125	1245	887	1073	973	85.173	87.415	83.018	Transposase IS116/IS110/IS902 family protein
bin001	SOY3_bin001_02126	228	11	15	16	5.768	6.673	7.454	manganese transport regulator MntR
bin001	SOY3_bin001_02127	141	38	76	67	32.219	54.670	50.476	hypothetical protein
bin001	SOY3_bin001_02128	225	119	177	146	63.228	79.789	68.929	hypothetical protein
bin001	SOY3_bin001_02129	330	177	183	160	64.122	56.246	51.503	hypothetical protein
bin001	SOY3_bin001_02130	1902	2089	1951	1796	131.303	104.040	100.306	Inner membrane protein YbaL
bin001	SOY3_bin001_02131	264	197	309	269	89.209	118.716	108.238	hypothetical protein
bin001	SOY3_bin001_02132	462	1040	1564	1408	269.114	343.360	323.736	Corrinoid/iron-sulfur protein large subunit
bin001	SOY3_bin001_02133	2091	1541	1384	1414	88.104	67.133	71.833	hypothetical protein
bin001	SOY3_bin001_02134	123	24	22	23	23.327	18.141	19.863	hypothetical protein
bin001	SOY3_bin001_02135	921	65	61	45	8.437	6.718	5.190	hypothetical protein
bin001	SOY3_bin001_02136	1146	312	184	166	32.547	16.285	15.387	hypothetical protein
bin001	SOY3_bin001_02137	1830	2264	2517	2181	147.901	139.504	126.600	Biosynthetic arginine decarboxylase
bin001	SOY3_bin001_02138	1929	951	1230	1100	58.938	64.674	60.575	Calcium-transporting ATPase
bin001	SOY3_bin001_02139	846	16713	17546	22999	2361.726	2103.597	2887.809	Ribosomal protein L11 methyltransferase
bin001	SOY3_bin001_02140	957	20954	21831	28734	2617.583	2313.750	3189.437	Fe(3+)-citrate-binding protein YfmC precursor
bin001	SOY3_bin001_02141	519	801	786	817	184.506	153.607	167.219	adenosine nucleotide hydrolase NudE

bin001	SOY3_bin001_02142	705	1716	2107	1974	290.987	303.131	297.432	hypothetical protein
bin001	SOY3_bin001_02143	690	586	675	636	101.530	99.222	97.912	Phosphoribosyl isomerase A
bin001	SOY3_bin001_02144	804	212	281	261	31.523	35.449	34.484	Demethylrebeccamycin-D-glucose O-methyltransferase
bin001	SOY3_bin001_02145	1005	659	501	471	78.391	50.562	49.783	Inner membrane protein YrbG
bin001	SOY3_bin001_02146	681	380	393	367	66.709	58.533	57.246	K(+)/H(+) antiporter NhaP2
bin001	SOY3_bin001_02147	135	12	20	21	10.627	15.026	16.524	hypothetical protein
bin001	SOY3_bin001_02148	1083	135	164	162	14.902	15.359	15.890	hypothetical protein
bin001	SOY3_bin001_02149	402	167	229	188	49.663	57.778	49.678	hypothetical protein
bin001	SOY3_bin001_02150	258	93	92	80	43.093	36.168	32.938	hypothetical protein
bin001	SOY3_bin001_02151	1056	982	1633	1217	111.171	156.847	122.421	hypothetical protein
bin001	SOY3_bin001_02152	462	148	228	170	38.297	50.055	39.087	hypothetical protein
bin001	SOY3_bin001_02153	1353	5107	7305	6957	451.246	547.617	546.203	Bifunctional purine biosynthesis protein PurH
bin001	SOY3_bin001_02154	732	2379	3695	3109	388.534	511.986	451.169	hypothetical protein
bin001	SOY3_bin001_02155	411	1776	3415	2678	516.591	842.759	692.147	hypothetical protein
bin001	SOY3_bin001_02156	930	746	1239	932	95.896	135.127	106.454	hypothetical protein
bin001	SOY3_bin001_02157	972	1687	2152	1834	207.489	224.559	200.430	Phosphoribulokinase
bin001	SOY3_bin001_02158	675	1403	1700	1590	248.485	255.446	250.221	hypothetical protein
bin001	SOY3_bin001_02159	873	2439	2790	2695	333.997	324.149	327.925	LL-diaminopimelate aminotransferase
bin001	SOY3_bin001_02160	1671	3415	3978	3661	244.320	241.459	232.730	Putative DNA ligase-like protein/MT0965
bin001	SOY3_bin001_02161	582	72	84	86	14.790	14.639	15.697	hypothetical protein
bin001	SOY3_bin001_02162	552	659	913	789	142.722	167.759	151.834	hypothetical protein
bin001	SOY3_bin001_02163	309	155	290	278	59.968	95.191	95.569	hypothetical protein
bin001	SOY3_bin001_02164	138	66	100	101	57.176	73.498	77.745	hypothetical protein
bin001	SOY3_bin001_02165	612	1014	1322	1192	198.076	219.096	206.897	hypothetical protein
bin001	SOY3_bin001_02166	663	1602	2036	1855	288.865	311.472	297.208	Iron-sulfur flavoprotein
bin001	SOY3_bin001_02167	345	718	1049	894	248.800	308.398	275.263	prolyl-tRNA synthetase
bin001	SOY3_bin001_02168	255	727	750	703	340.832	298.316	292.850	hypothetical protein
bin001	SOY3_bin001_02169	150	2475	2851	2686	1972.557	1927.794	1902.149	50S ribosomal protein L40e
bin001	SOY3_bin001_02170	477	246	255	258	61.654	54.222	57.455	hypothetical protein
bin001	SOY3_bin001_02171	819	64	152	120	9.342	18.824	15.564	putative inner membrane protein
bin001	SOY3_bin001_02172	327	1861	2319	2161	680.368	719.296	702.000	hypothetical protein
bin001	SOY3_bin001_02173	126	240	331	322	227.712	266.448	271.466	hypothetical protein
bin001	SOY3_bin001_02174	1626	2856	3949	3766	209.983	246.332	246.031	Aerobic cobaltochelatase subunit CobN
bin001	SOY3_bin001_02175	507	1852	2549	2552	436.695	509.937	534.691	threonine dehydratase
bin001	SOY3_bin001_02176	1008	3264	4247	4118	387.111	427.343	433.966	Homoserine dehydrogenase
bin001	SOY3_bin001_02177	1338	40	48	42	3.574	3.639	3.334	Nitrogenase molybdenum-iron protein alpha chain
bin001	SOY3_bin001_02178	768	134	165	158	20.859	21.791	21.854	Acyltransferase family protein
bin001	SOY3_bin001_02179	177	147	160	162	99.286	91.686	97.224	hypothetical protein
bin001	SOY3_bin001_02180	918	1653	2234	2241	215.266	246.828	259.316	cobalamin biosynthesis protein
bin001	SOY3_bin001_02181	243	805	1106	1116	396.036	461.640	487.852	Cysteine-rich small domain protein
bin001	SOY3_bin001_02182	456	1421	1990	2006	372.542	442.632	467.300	Histidinol-phosphate aminotransferase 2
bin001	SOY3_bin001_02183	318	614	956	799	230.827	304.920	266.901	hypothetical protein
bin001	SOY3_bin001_02184	435	610	757	603	167.643	176.507	147.251	hypothetical protein
bin001	SOY3_bin001_02185	1185	1553	1975	1722	156.675	169.045	154.363	hypothetical protein
bin001	SOY3_bin001_02186	1146	4756	4031	3817	496.138	356.766	353.808	Aminopeptidase
bin001	SOY3_bin001_02187	618	630	847	640	121.870	139.011	110.007	hypothetical protein
bin001	SOY3_bin001_02188	297	483	587	505	194.418	200.464	180.620	hypothetical protein
bin001	SOY3_bin001_02189	843	1365	1477	1349	193.576	177.708	169.986	hypothetical protein
bin001	SOY3_bin001_02190	129	49	86	84	45.410	67.618	69.170	hypothetical protein
bin001	SOY3_bin001_02191	396	3341	4453	4630	1008.618	1140.545	1241.983	Sirohydrochlorin ferrochelatase
bin001	SOY3_bin001_02192	441	2563	3091	3249	694.793	710.911	782.602	hypothetical protein
bin001	SOY3_bin001_02193	576	242	264	259	50.227	46.488	47.765	Fructose-1,6-bisphosphatase
bin001	SOY3_bin001_02194	882	359	344	319	48.660	39.559	38.420	hypothetical protein
bin001	SOY3_bin001_02195	546	1388	1201	1029	303.908	223.103	200.195	hypothetical protein
bin001	SOY3_bin001_02196	486	7191	5556	5096	1768.881	1159.527	1113.840	hypothetical protein
bin001	SOY3_bin001_02197	807	189	245	224	27.998	30.793	29.485	Formamidopyrimidine-DNA glycosylase
bin001	SOY3_bin001_02198	819	602	722	704	87.874	89.415	91.310	Trans-aconitate 2-methyltransferase
bin001	SOY3_bin001_02199	1218	1343	1838	1696	131.818	153.057	147.914	Isocitrate dehydrogenase [NADP]
bin001	SOY3_bin001_02200	1230	2243	3141	2989	218.007	259.011	258.137	Histone deacetylase-like amidohydrolase
bin001	SOY3_bin001_02201	240	86	75	67	42.838	31.696	29.655	hypothetical protein
bin001	SOY3_bin001_02202	423	203	206	208	57.372	49.395	52.234	SnoaL-like polyketide cyclase
bin001	SOY3_bin001_02203	453	258	216	190	68.087	48.363	44.554	Calcium-transporting ATPase 1
bin001	SOY3_bin001_02204	513	624	832	498	145.416	164.498	103.120	hypothetical protein
bin001	SOY3_bin001_02205	315	433	580	373	164.332	186.755	125.785	hypothetical protein
bin001	SOY3_bin001_02206	102	115	101	110	134.786	100.433	114.557	YcfA-like protein
bin001	SOY3_bin001_02207	246	342	300	357	166.202	123.692	154.157	Antitoxin HicB
bin001	SOY3_bin001_02208	1161	664	571	687	68.372	49.884	62.857	MULE transposase domain protein

bin001	SOY3_bin001_02209	1080	1053	994	984	116.560	93.351	96.783	Hemolysin C
bin001	SOY3_bin001_02210	198	175	233	259	105.662	119.356	138.952	hypothetical protein
bin001	SOY3_bin001_02211	366	189	159	175	61.734	44.063	50.791	hypothetical protein
bin001	SOY3_bin001_02212	321	405	451	378	150.833	142.504	125.088	Kanamycin nucleotidyltransferase
bin001	SOY3_bin001_02213	744	2439	1758	1691	391.908	239.663	241.435	Phosphoribosyl 1,2-cyclic phosphodiesterase
bin001	SOY3_bin001_02214	435	2148	1606	1641	590.324	374.465	400.727	ATP-dependent zinc metalloprotease FtsH
bin001	SOY3_bin001_02215	1050	749	743	740	85.278	71.772	74.864	hypothetical protein
bin001	SOY3_bin001_02216	795	34	32	32	5.113	4.083	4.276	Integrase core domain protein
bin001	SOY3_bin001_02217	780	412	516	477	63.146	67.098	64.961	putative ABC transporter ATP-binding protein
bin001	SOY3_bin001_02218	597	348	377	427	69.687	64.050	75.977	putative ABC transporter permease protein
bin001	SOY3_bin001_02219	390	698	681	709	213.962	177.108	193.113	hypothetical protein
bin001	SOY3_bin001_02220	324	730	731	641	269.354	228.837	210.157	Cobalt transport protein CbiN
bin001	SOY3_bin001_02221	795	647	653	526	97.293	83.311	70.283	Cobalt transport protein CbiQ
bin001	SOY3_bin001_02222	1257	3867	4564	4550	367.777	368.269	384.508	Adenosylhomocysteinase
bin001	SOY3_bin001_02223	564	329	375	369	69.737	67.438	69.499	Transposase
bin001	SOY3_bin001_02224	363	2062	2099	1854	679.090	586.490	542.541	hypothetical protein
bin001	SOY3_bin001_02225	1251	3878	5618	5332	370.592	455.490	452.754	Enolase
bin001	SOY3_bin001_02226	216	36	45	27	19.925	21.131	13.278	hypothetical protein
bin001	SOY3_bin001_02227	360	147	99	87	48.816	27.893	25.671	HTH-type transcriptional activator HxlR
bin001	SOY3_bin001_02228	417	5694	5709	4882	1632.401	1388.605	1243.631	Putative transposase DNA-binding domain protein
bin001	SOY3_bin001_02229	945	363	369	392	45.922	39.605	44.064	Cephalosporin-C deacetylase
bin001	SOY3_bin001_02230	252	200	258	229	94.880	103.842	96.531	4Fe-4S binding domain protein
bin001	SOY3_bin001_02231	600	84	111	126	16.737	18.764	22.307	hypothetical protein
bin001	SOY3_bin001_02232	255	1861	2375	2232	872.472	944.666	929.788	hypothetical protein
bin001	SOY3_bin001_02233	285	571	708	640	239.517	251.967	238.542	hypothetical protein
bin001	SOY3_bin001_02234	453	737	834	828	194.498	186.734	194.161	H/ACA RNA-protein complex component Cbf5p
bin001	SOY3_bin001_02235	552	669	806	801	144.888	148.099	154.143	hypothetical protein
bin001	SOY3_bin001_02236	273	393	454	395	172.098	168.674	153.697	hypothetical protein
bin001	SOY3_bin001_02237	906	2029	2514	2218	267.731	281.444	260.054	Valine--tRNA ligase
bin001	SOY3_bin001_02238	507	170	244	295	40.085	48.813	61.808	tRNA(fMet)-specific endonuclease VapC
bin001	SOY3_bin001_02239	351	92	62	45	31.335	17.916	13.619	hypothetical protein
bin001	SOY3_bin001_02240	105	62	67	68	70.591	64.720	68.794	hypothetical protein
bin001	SOY3_bin001_02241	153	92	104	89	71.886	68.944	61.791	hypothetical protein
bin001	SOY3_bin001_02242	177	377	378	391	254.632	216.607	234.657	hypothetical protein
bin001	SOY3_bin001_02243	297	291	317	292	117.134	108.257	104.437	Plasmid stabilisation system protein
bin001	SOY3_bin001_02244	354	1370	1484	1371	462.661	425.192	411.400	hypothetical protein
bin001	SOY3_bin001_02245	237	937	1143	935	472.647	489.162	419.076	hypothetical protein
bin001	SOY3_bin001_02246	801	224	250	228	33.432	31.656	30.237	hypothetical protein
bin001	SOY3_bin001_02247	210	149	209	227	84.823	100.944	114.825	hypothetical protein
bin001	SOY3_bin001_02248	243	260	369	343	127.912	154.019	149.940	phosphate transporter permease subunit PstC
bin001	SOY3_bin001_02249	930	184	291	254	23.653	31.737	29.012	Ferrous iron transport protein B
bin001	SOY3_bin001_02250	207	126	309	365	72.769	151.406	187.306	hypothetical protein
bin001	SOY3_bin001_02251	144	51	168	215	42.340	118.332	158.601	hypothetical protein
bin001	SOY3_bin001_02252	885	2182	2944	2524	294.752	337.403	302.953	hypothetical protein
bin001	SOY3_bin001_02253	396	211	318	310	63.699	81.449	83.157	hypothetical protein
bin001	SOY3_bin001_02254	411	130	154	144	37.814	38.004	37.218	Y_Y_Y domain protein
bin001	SOY3_bin001_02255	750	1325	1465	1338	211.203	198.121	189.507	Serine protease AprX
bin001	SOY3_bin001_02256	768	801	1193	1172	124.686	157.556	162.105	Putative thiosulfate sulfurtransferase
bin001	SOY3_bin001_02257	492	77	58	53	18.710	11.957	11.443	Shikimate kinase 1
bin001	SOY3_bin001_02258	276	185	319	449	80.132	117.229	172.809	Tryptophan synthase beta chain
bin001	SOY3_bin001_02259	777	727	1111	1196	111.856	145.027	163.508	Tryptophan synthase alpha chain
bin001	SOY3_bin001_02260	318	1857	2140	2395	698.120	682.561	800.034	hypothetical protein
bin001	SOY3_bin001_02261	549	1636	2003	1835	356.251	370.053	355.053	hypothetical protein
bin001	SOY3_bin001_02262	177	73	117	122	49.305	67.045	73.218	hypothetical protein
bin001	SOY3_bin001_02263	192	74	96	78	46.076	50.714	43.154	hypothetical protein
bin001	SOY3_bin001_02264	186	49	47	36	31.494	25.629	20.560	hypothetical protein
bin001	SOY3_bin001_02265	516	93	165	143	21.547	32.433	29.439	hypothetical protein
bin001	SOY3_bin001_02266	915	285	247	289	37.237	27.380	33.551	Tyrosine recombinase XerC
bin001	SOY3_bin001_02267	639	176	145	175	32.927	23.016	29.092	hypothetical protein
bin001	SOY3_bin001_02268	147	82	131	124	66.687	90.388	89.605	hypothetical protein
bin001	SOY3_bin001_02269	285	8	12	24	3.356	4.271	8.945	hypothetical protein
bin001	SOY3_bin001_02270	525	48	30	57	10.930	5.796	11.533	hypothetical protein
bin001	SOY3_bin001_02271	1416	1903	2547	2038	160.665	182.440	152.887	hypothetical protein
bin001	SOY3_bin001_02272	174	102	89	75	70.080	51.879	45.787	hypothetical protein
bin001	SOY3_bin001_02273	1470	1186	1357	1404	96.452	93.630	101.456	putative DNA double-strand break repair Rad50 ATPase
bin001	SOY3_bin001_02274	567	793	1002	974	167.200	179.242	182.476	Porphobilinogen deaminase
bin001	SOY3_bin001_02275	1506	2238	2974	2803	177.656	200.295	197.710	Uroporphyrinogen-III C-methyltransferase

bin001	SOY3_bin001_02276	1161	1101	1152	1215	113.371	100.641	111.166	Antilisterial bacteriocin subtilosin biosynthesis protein AlbA
bin001	SOY3_bin001_02277	2418	64337	66404	75949	3180.900	2785.433	3336.534	NADH-quinone oxidoreductase subunit I
bin001	SOY3_bin001_02278	564	17426	22220	20208	3693.721	3995.947	3806.046	acetyl-CoA decarbonylase/synthase complex subunit epsilon
bin001	SOY3_bin001_02279	213	239	311	275	134.142	148.093	137.146	hypothetical protein
bin001	SOY3_bin001_02280	228	87	156	146	45.617	69.398	68.022	hypothetical protein
bin001	SOY3_bin001_02281	531	264	394	417	59.437	75.259	83.420	hypothetical protein
bin001	SOY3_bin001_02282	171	1	3	1	0.699	1.779	0.621	hypothetical protein
bin001	SOY3_bin001_02283	1479	949	1627	1483	76.709	111.577	106.513	Glycosyl hydrolase family 57
bin001	SOY3_bin001_02284	417	342	514	503	98.047	125.021	128.133	hypothetical protein
bin001	SOY3_bin001_02285	654	558	866	775	102.000	134.306	125.879	hypothetical protein
bin001	SOY3_bin001_02286	1053	3482	4388	5032	395.317	422.662	507.624	Vitamin B12-binding protein precursor
bin001	SOY3_bin001_02287	618	1757	2037	2236	339.883	334.316	384.338	Demethylrebeccamycin-D-glucose O-methyltransferase
bin001	SOY3_bin001_02288	315	159	208	215	60.344	66.974	72.503	hypothetical protein
bin001	SOY3_bin001_02289	294	6010	7711	7304	2443.839	2660.224	2639.023	hypothetical protein
bin001	SOY3_bin001_02290	849	269	464	403	37.878	55.433	50.423	CAAX amino terminal protease self- immunity
bin001	SOY3_bin001_02291	774	2808	3225	2924	433.712	422.614	401.297	FKBP-type peptidyl-prolyl cis-trans isomerase SlyD
bin001	SOY3_bin001_02292	411	2085	2908	2415	606.471	717.641	624.173	hypothetical protein
bin001	SOY3_bin001_02293	609	886	983	952	173.925	163.716	166.054	hypothetical protein
bin001	SOY3_bin001_02294	645	2623	2973	2957	486.165	467.509	486.992	hypothetical protein
bin001	SOY3_bin001_02295	723	1232	1323	1304	203.713	185.599	191.588	DNA primase large subunit
bin001	SOY3_bin001_02296	396	621	886	807	187.474	226.931	216.475	hypothetical protein
bin001	SOY3_bin001_02297	252	1362	1347	1159	646.133	542.153	488.554	NAD-specific glutamate dehydrogenase
bin001	SOY3_bin001_02298	147	17	21	9	13.825	14.490	6.504	hypothetical protein
bin001	SOY3_bin001_02299	729	320	502	398	52.477	69.844	57.994	Ferredoxin--NAD(P)(+) reductase (naphthalene dioxygenase/salicylate 5-hydroxylase ferredoxin-specific)
bin001	SOY3_bin001_02300	291	128	147	158	52.585	51.236	57.676	T-protein
bin001	SOY3_bin001_02301	183	146	174	141	95.378	96.439	81.846	hypothetical protein
bin001	SOY3_bin001_02302	432	821	1269	1116	227.198	297.943	274.417	hypothetical protein
bin001	SOY3_bin001_02303	1425	2318	2648	2251	194.466	188.477	167.799	RNA-splicing ligase RtcB
bin001	SOY3_bin001_02304	984	1890	2401	2473	229.621	247.487	266.968	Dipeptide transport system permease protein DppB
bin001	SOY3_bin001_02305	723	455	331	292	75.235	46.435	42.902	hypothetical protein
bin001	SOY3_bin001_02306	273	550	851	814	240.849	316.171	316.732	Non-histone chromosomal protein MC1
bin001	SOY3_bin001_02307	663	834	775	660	150.383	118.561	105.745	2-phospho-L-lactate guanylyltransferase
bin001	SOY3_bin001_02308	498	2625	2975	3376	630.152	605.916	720.117	Periplasmic dipeptide transport protein precursor
bin001	SOY3_bin001_02309	948	2292	2739	3106	289.036	293.048	348.035	Glutathione transport system permease protein GsiC
bin001	SOY3_bin001_02310	216	192	198	310	106.266	92.975	152.454	hypothetical protein
bin001	SOY3_bin001_02311	336	333	377	592	118.481	113.804	187.160	hypothetical protein
bin001	SOY3_bin001_02312	984	700	1170	1079	85.045	120.600	116.481	ATP-dependent RNA helicase DbpA
bin001	SOY3_bin001_02313	894	494	702	594	66.059	79.644	70.580	SWIM zinc finger
bin001	SOY3_bin001_02314	291	124	384	244	50.942	133.842	89.069	hypothetical protein
bin001	SOY3_bin001_02315	393	282	348	331	85.783	89.813	89.467	TM2 domain protein
bin001	SOY3_bin001_02316	861	223	342	215	30.963	40.288	26.526	Putative neutral zinc metallopeptidase
bin001	SOY3_bin001_02317	165	75	38	42	54.340	23.359	27.039	hypothetical protein
bin001	SOY3_bin001_02318	1734	912	954	819	62.877	55.803	50.172	hypothetical protein
bin001	SOY3_bin001_02319	216	30	43	23	16.604	20.192	11.311	Nitrogenase molybdenum-iron protein beta chain
bin001	SOY3_bin001_02320	1428	176	237	212	14.734	16.834	15.770	Nitrogenase molybdenum-iron protein alpha chain
bin001	SOY3_bin001_02321	477	129	140	124	32.331	29.769	27.614	hypothetical protein
bin001	SOY3_bin001_02322	414	963	1184	952	278.081	290.072	244.268	hypothetical protein
bin001	SOY3_bin001_02323	234	747	961	963	381.637	416.545	437.160	hypothetical protein
bin001	SOY3_bin001_02324	1305	305	357	282	27.941	27.747	22.955	2,3-bisphosphoglycerate-independent phosphoglycerate mutase
bin001	SOY3_bin001_02325	186	32	53	49	20.568	28.901	27.984	hypothetical protein
bin001	SOY3_bin001_02326	291	40	53	51	16.433	18.473	18.617	Plasmid stabilisation system protein
bin001	SOY3_bin001_02327	702	272	342	368	46.321	49.413	55.685	hypothetical protein
bin001	SOY3_bin001_02328	876	168	170	160	22.927	19.683	19.402	DNA-binding transcriptional activator MhpR
bin001	SOY3_bin001_02329	168	2	3	0	1.423	1.811	0.000	hypothetical protein
bin001	SOY3_bin001_02330	270	294	422	400	130.175	158.527	157.371	hypothetical protein
bin001	SOY3_bin001_02331	162	83	141	106	61.250	88.279	69.506	hypothetical protein
bin001	SOY3_bin001_02332	309	113	166	116	43.719	54.488	39.878	hypothetical protein
bin001	SOY3_bin001_02333	741	918	783	738	148.105	107.176	105.796	Ribonuclease BN
bin001	SOY3_bin001_02334	216	425	291	332	235.223	136.645	163.273	hypothetical protein
bin001	SOY3_bin001_02335	1665	598	695	718	42.937	42.338	45.808	Endonuclease MutS2
bin001	SOY3_bin001_02336	930	82	77	63	10.541	8.398	7.196	hypothetical protein
bin001	SOY3_bin001_02337	435	285	333	261	78.325	77.644	63.735	Molybdopterin-guanine dinucleotide biosynthesis adapter protein
bin001	SOY3_bin001_02338	966	1176	1322	1110	145.538	138.806	122.061	Succinyl-diaminopimelate desuccinylase
bin001	SOY3_bin001_02339	360	530	761	671	176.003	214.406	197.993	hypothetical protein
bin001	SOY3_bin001_02340	1272	2199	3199	2843	206.673	255.083	237.421	Glutamate-1-semialdehyde 2,1-aminomutase
bin001	SOY3_bin001_02341	1137	1682	2045	1982	176.852	182.426	185.171	Succinyl-CoA:coenzyme A transferase
bin001	SOY3_bin001_02342	480	380	456	403	94.643	96.356	89.185	hypothetical protein

bin001	SOY3_bin001_02343	1878	2746	3916	3441	174.804	211.496	194.634	Ferredoxin
bin001	SOY3_bin001_02344	228	44	29	27	23.071	12.901	12.579	hypothetical protein
bin001	SOY3_bin001_02345	1482	3188	2894	3752	257.167	198.064	268.933	Oligosaccharyl transferase STT3 subunit
bin001	SOY3_bin001_02346	1344	152	169	179	13.520	12.754	14.148	hypothetical protein
bin001	SOY3_bin001_02347	303	194	183	217	76.543	61.258	76.076	hypothetical protein
bin001	SOY3_bin001_02348	1320	1859	2490	2258	168.365	191.329	181.710	Phosphomannomutase/phosphoglucomutase
bin001	SOY3_bin001_02349	1167	1329	1758	1959	136.144	152.793	178.317	Ferrous iron transport protein B
bin001	SOY3_bin001_02350	417	410	586	633	117.542	142.533	161.249	hypothetical protein
bin001	SOY3_bin001_02351	207	339	304	313	195.783	148.956	160.622	hypothetical protein
bin001	SOY3_bin001_02352	387	593	567	646	183.185	148.603	177.317	Acylphosphatase
bin001	SOY3_bin001_02353	978	3936	4391	4056	481.129	455.386	440.544	DNA repair and recombination protein RadA
bin001	SOY3_bin001_02354	1224	978	3980	2263	95.522	329.804	196.396	Threonine synthase
bin001	SOY3_bin001_02355	279	170	549	330	72.843	199.583	125.643	hypothetical protein
bin001	SOY3_bin001_02356	582	75	57	58	15.406	9.934	10.586	Periplasmic copper-binding protein (NosD)
bin001	SOY3_bin001_02357	741	718	908	887	115.838	124.286	127.156	hypothetical protein
bin001	SOY3_bin001_02358	882	210	237	133	28.464	27.254	16.018	TPR repeat-containing protein YrrB
bin001	SOY3_bin001_02359	1617	2104	2745	2575	155.554	172.182	169.160	Carbamoyl-phosphate synthase large chain
bin001	SOY3_bin001_02360	993	347	307	253	41.776	31.358	27.065	lipid A 1-phosphatase
bin001	SOY3_bin001_02361	453	397	441	492	104.770	98.740	115.371	hypothetical protein
bin001	SOY3_bin001_02362	1077	1308	1794	1646	145.190	168.951	162.347	Queuine tRNA-ribosyltransferase
bin001	SOY3_bin001_02363	789	868	1322	1297	131.519	169.945	174.620	hypothetical protein
bin001	SOY3_bin001_02364	468	1194	1622	1489	305.003	351.528	337.970	hypothetical protein
bin001	SOY3_bin001_02365	1101	2579	3008	3014	280.033	277.106	290.794	Nitrogenase molybdenum-iron protein beta chain
bin001	SOY3_bin001_02366	288	235	187	171	97.549	65.857	63.072	hypothetical protein
bin001	SOY3_bin001_02367	189	190	254	260	120.181	136.310	146.131	hypothetical protein
bin001	SOY3_bin001_02368	363	436	532	519	143.590	148.648	151.876	hypothetical protein
bin001	SOY3_bin001_02369	525	828	1096	1098	188.546	211.742	222.164	hypothetical protein
bin001	SOY3_bin001_02370	675	1024	1029	960	181.360	154.620	151.077	lipoprotein NlpI
bin001	SOY3_bin001_02371	1005	1749	1621	1534	208.051	163.596	162.140	hypothetical protein
bin001	SOY3_bin001_02372	471	974	1366	1176	247.220	294.161	265.226	hypothetical protein
bin001	SOY3_bin001_02373	189	338	366	367	213.796	196.415	206.269	hypothetical protein
bin001	SOY3_bin001_02374	147	435	281	301	353.767	193.885	217.510	hypothetical protein
bin001	SOY3_bin001_02375	147	670	541	512	544.883	373.280	369.984	hypothetical protein
bin001	SOY3_bin001_02376	225	1236	1108	1139	656.722	499.473	537.738	hypothetical protein
bin001	SOY3_bin001_02377	549	33	36	30	7.186	6.651	5.805	Deazaflavin-dependent nitroreductase
bin001	SOY3_bin001_02378	1284	1174	1864	3110	109.307	147.243	257.291	High-affinity heme uptake system protein IsdE precursor
bin001	SOY3_bin001_02379	471	456	576	612	115.742	124.038	138.026	hypothetical protein
bin001	SOY3_bin001_02380	345	398	351	432	137.914	103.191	133.013	hypothetical protein
bin001	SOY3_bin001_02381	420	207	143	138	58.921	34.534	34.903	L-threonine dehydratase catabolic TdcB
bin001	SOY3_bin001_02382	195	463	543	569	283.852	282.436	309.961	hypothetical protein
bin001	SOY3_bin001_02383	525	4949	4479	4201	1126.947	865.320	850.008	Adenylate kinase
bin001	SOY3_bin001_02384	501	6525	6252	6557	1556.999	1265.715	1390.264	Membrane protein insertase YidC
bin001	SOY3_bin001_02385	1266	265	284	265	25.024	22.753	22.235	Large cysteine-rich periplasmic protein OmcB, serovars L1/L3 precursor
bin001	SOY3_bin001_02386	291	91	72	98	37.385	25.095	35.774	hypothetical protein
bin001	SOY3_bin001_02387	636	514	605	552	96.617	96.483	92.196	Queuine tRNA-ribosyltransferase
bin001	SOY3_bin001_02388	744	392	449	469	62.988	61.211	66.962	hypothetical protein
bin001	SOY3_bin001_02389	306	399	541	506	155.882	179.321	175.654	hypothetical protein
bin001	SOY3_bin001_02390	171	51	110	105	35.655	65.246	65.226	hypothetical protein
bin001	SOY3_bin001_02391	369	292	389	337	94.602	106.925	97.014	Acetyltransferase (GNAT) family protein
bin001	SOY3_bin001_02392	384	5609	5474	4848	1746.223	1445.867	1341.100	30S ribosomal protein S8e
bin001	SOY3_bin001_02393	210	2392	2007	1754	1361.719	969.355	887.238	hypothetical protein
bin001	SOY3_bin001_02394	321	992	985	1002	369.447	311.233	331.583	Putative esterase/MT1895
bin001	SOY3_bin001_02395	420	1054	1411	1232	300.011	340.747	311.596	hypothetical protein
bin001	SOY3_bin001_02396	894	653	871	801	87.322	98.818	95.175	Putative snoRNA binding domain protein
bin001	SOY3_bin001_02397	942	1294	1500	1327	164.221	161.508	149.641	hypothetical protein
bin001	SOY3_bin001_02398	633	37	47	34	6.988	7.531	5.706	hypothetical protein
bin001	SOY3_bin001_02399	567	142	147	112	29.940	26.296	20.983	hypothetical protein
bin001	SOY3_bin001_02400	420	1295	1318	1252	368.609	318.288	316.654	hypothetical protein
bin001	SOY3_bin001_02401	543	272	399	258	59.885	74.529	50.472	hypothetical protein
bin001	SOY3_bin001_02402	771	220	231	237	34.113	30.389	32.653	Asparagine synthetase [glutamine-hydrolyzing] 1
bin001	SOY3_bin001_02403	915	294	352	332	38.412	39.019	38.543	HI0933-like protein
bin001	SOY3_bin001_02404	270	304	274	316	134.603	102.930	124.323	lipoprotein NlpI
bin001	SOY3_bin001_02405	333	417	572	524	149.705	174.223	167.154	NAD(P)H-quinone oxidoreductase subunit I
bin001	SOY3_bin001_02406	294	534	683	632	217.140	235.629	228.349	hypothetical protein
bin001	SOY3_bin001_02407	264	1070	1327	1040	484.535	509.826	418.465	Ketol-acid reductoisomerase
bin001	SOY3_bin001_02408	414	1871	745	769	540.280	182.520	197.313	hypothetical protein
bin001	SOY3_bin001_02409	837	320	398	338	45.706	48.229	42.896	Lipoyl synthase

bin001	SOY3_bin001_02410	855	494	576	507	69.073	68.330	62.990	Endonuclease 4
bin001	SOY3_bin001_02411	405	527	850	734	155.561	212.872	192.518	Response regulator rcp1
bin001	SOY3_bin001_02412	525	257	312	252	58.522	60.277	50.988	hypothetical protein
bin001	SOY3_bin001_02413	603	393	361	370	77.915	60.722	65.180	hypothetical protein
bin001	SOY3_bin001_02414	312	56	50	31	21.457	16.254	10.554	Isoprenylcysteine carboxyl methyltransferase (ICMT) family protein
bin001	SOY3_bin001_02415	333	111	221	166	39.850	67.314	52.953	hypothetical protein
bin001	SOY3_bin001_02416	609	1863	3433	1865	365.714	571.757	325.305	Macrolide export ATP-binding/permease protein MacB
bin001	SOY3_bin001_02417	264	621	992	587	281.212	381.121	236.191	hypothetical protein
bin001	SOY3_bin001_02418	444	91	88	86	24.502	20.103	20.575	Nitroreductase family protein
bin001	SOY3_bin001_02419	735	140	112	93	22.771	15.456	13.441	Carboxymuconolactone decarboxylase family protein
bin001	SOY3_bin001_02420	198	27	40	18	16.302	20.490	9.657	hypothetical protein
bin001	SOY3_bin001_02421	366	59	130	50	19.272	36.026	14.512	hypothetical protein
bin001	SOY3_bin001_02422	402	1161	1511	1502	345.264	381.235	396.893	hypothetical protein
bin001	SOY3_bin001_02423	183	349	545	549	227.992	302.065	318.677	hypothetical protein
bin001	SOY3_bin001_02424	117	197	170	160	201.292	147.373	145.266	hypothetical protein
bin001	SOY3_bin001_02425	396	672	597	477	202.871	152.909	127.954	hypothetical protein
bin001	SOY3_bin001_02426	813	2213	2227	2367	325.414	277.833	309.270	hypothetical protein
bin001	SOY3_bin001_02427	198	10	27	15	6.038	13.831	8.047	hypothetical protein
bin001	SOY3_bin001_02428	327	31	62	28	11.333	19.231	9.096	hypothetical protein
bin001	SOY3_bin001_02429	975	1676	1726	1561	205.501	179.552	170.070	NMD3 family protein
bin001	SOY3_bin001_02430	510	236	327	317	55.321	65.033	66.027	hypothetical protein
bin001	SOY3_bin001_02431	267	66	50	49	29.551	18.994	19.495	hypothetical protein
bin001	SOY3_bin001_02432	240	80	56	71	39.850	23.666	31.425	hypothetical protein
bin001	SOY3_bin001_02433	405	304	355	338	89.735	88.905	88.653	hypothetical protein
bin001	SOY3_bin001_02434	702	227	284	301	38.658	41.033	45.547	Putative electron transport protein YccM
bin001	SOY3_bin001_02435	201	108	121	110	64.235	61.058	58.133	hypothetical protein
bin001	SOY3_bin001_02436	867	229	238	210	31.576	27.843	25.729	Demethylmenaquinone methyltransferase
bin001	SOY3_bin001_02437	243	83	58	77	40.834	24.209	33.660	hypothetical protein
bin001	SOY3_bin001_02438	519	377	320	416	86.840	62.537	85.144	hypothetical protein
bin001	SOY3_bin001_02439	1020	3877	4454	4080	454.403	442.899	424.903	CTP synthase
bin001	SOY3_bin001_02440	609	192	234	196	37.690	38.972	34.188	hypothetical protein
bin001	SOY3_bin001_02441	645	2961	3277	2973	548.813	515.313	489.627	hypothetical protein
bin001	SOY3_bin001_02442	126	101	149	176	95.829	119.942	148.379	hypothetical protein
bin001	SOY3_bin001_02443	177	404	492	658	272.869	281.933	394.896	hypothetical protein
bin001	SOY3_bin001_02444	648	1653	2087	2816	304.960	326.665	461.623	High-affinity zinc uptake system ATP-binding protein ZnuC
bin001	SOY3_bin001_02445	90	46	63	80	61.103	70.999	94.423	hypothetical protein
bin001	SOY3_bin001_02446	945	2015	2467	2355	254.911	264.784	264.721	GTP cyclohydrolase FolE2
bin001	SOY3_bin001_02447	117	342	548	446	349.451	475.061	404.929	Zinc finger, C2H2 type
bin001	SOY3_bin001_02448	318	334	562	490	125.564	179.252	163.681	High molecular weight rubredoxin
bin001	SOY3_bin001_02449	804	206	205	163	30.631	25.861	21.536	Nitroreductase family protein
bin001	SOY3_bin001_02450	111	11	4	8	11.847	3.655	7.656	hypothetical protein
bin001	SOY3_bin001_02451	489	139	179	152	33.982	37.128	33.019	hypothetical protein
bin001	SOY3_bin001_02452	303	110	115	123	43.401	38.496	43.121	hypothetical protein
bin001	SOY3_bin001_02453	738	179	200	175	28.996	27.487	25.189	H(+)/Cl(-) exchange transporter ClcA
bin001	SOY3_bin001_02454	309	246	318	381	95.175	104.381	130.977	30S ribosomal protein S19e
bin001	SOY3_bin001_02455	192	220	276	222	136.983	145.802	122.824	hypothetical protein
bin001	SOY3_bin001_02456	471	291	269	241	73.861	57.928	54.353	Putative peroxiredoxin bcp
bin001	SOY3_bin001_02457	183	634	822	729	414.175	455.591	423.162	hypothetical protein
bin001	SOY3_bin001_02458	489	71	76	74	17.358	15.764	16.075	hypothetical protein
bin001	SOY3_bin001_02459	951	384	360	469	48.272	38.395	52.387	5-methylthioadenosine/S-adenosylhomocysteine deaminase
bin001	SOY3_bin001_02460	882	1258	1623	1562	170.513	186.640	188.123	hypothetical protein
bin001	SOY3_bin001_02461	828	16460	17160	19075	2376.539	2102.044	2447.169	tetrahydromethanopterin S-methyltransferase subunit C
bin001	SOY3_bin001_02462	855	2103	1991	2048	294.048	236.189	254.445	tRNA pseudouridine synthase B
bin001	SOY3_bin001_02463	696	22452	30313	28736	3856.482	4417.478	4385.781	V-type ATP synthase subunit D
bin001	SOY3_bin001_02464	213	70	114	112	39.288	54.285	55.856	hypothetical protein
bin001	SOY3_bin001_02465	231	127	161	166	65.726	70.692	76.335	YcfA-like protein
bin001	SOY3_bin001_02466	978	332	429	452	40.583	44.491	49.094	hypothetical protein
bin001	SOY3_bin001_02467	651	170	232	208	31.219	36.146	33.940	hypothetical protein
bin001	SOY3_bin001_02468	207	182	240	309	105.111	117.597	158.569	hypothetical protein
bin001	SOY3_bin001_02469	3186	1142	1294	1635	42.851	41.195	54.513	putative symporter YodF
bin001	SOY3_bin001_02470	243	107	102	138	52.641	42.574	60.326	SigmaK-factor processing regulatory protein BofA
bin001	SOY3_bin001_02471	1230	3455	2977	3831	335.806	245.487	330.854	cell division control protein 6
bin001	SOY3_bin001_02472	204	392	597	503	229.721	296.824	261.919	Antitoxin HicB
bin001	SOY3_bin001_02473	222	253	372	259	136.243	169.959	123.930	YcfA-like protein
bin001	SOY3_bin001_02474	600	448	580	508	89.263	98.046	89.938	Acylphosphatase
bin001	SOY3_bin001_02475	333	171	236	124	61.390	71.882	39.556	hypothetical protein
bin001	SOY3_bin001_02476	240	208	294	233	103.609	124.248	103.128	hypothetical protein

bin001	SOY3_bin001_02477	438	256	482	205	69.873	111.616	49.718	hypothetical protein
bin001	SOY3_bin001_02478	1062	57	35	38	6.416	3.343	3.801	Hemin transport system permease protein HmuU
bin001	SOY3_bin001_02479	336	386	599	533	137.339	180.818	168.507	hypothetical protein
bin001	SOY3_bin001_02480	1095	304	292	293	33.190	27.047	28.424	hypothetical protein
bin001	SOY3_bin001_02481	522	72	65	70	16.490	12.630	14.245	Aerobic cobaltochelatase subunit CobN
bin001	SOY3_bin001_02482	804	20	26	43	2.974	3.280	5.681	Transposase
bin001	SOY3_bin001_02483	324	189	173	217	69.737	54.157	71.145	Mannosylfructose-phosphate synthase
bin001	SOY3_bin001_02484	624	92	84	90	17.626	13.654	15.321	hypothetical protein
bin001	SOY3_bin001_02485	1347	1738	1878	1927	154.251	141.411	151.965	Transposase IS66 family protein
bin001	SOY3_bin001_02486	2196	1038	1023	1140	56.508	47.250	55.145	Regulatory protein AfsR
bin001	SOY3_bin001_02487	1140	2303	2115	2468	241.510	188.174	229.969	Antilisterial bacteriocin subtilosin biosynthesis protein AlbA
bin001	SOY3_bin001_02488	135	264	508	466	233.784	381.667	366.676	Carboxymuconolactone decarboxylase family protein
bin001	SOY3_bin001_02489	228	0	0	2	0.000	0.000	0.932	hypothetical protein
bin001	SOY3_bin001_02490	840	29	39	34	4.127	4.709	4.300	CAAX amino terminal protease self- immunity
bin001	SOY3_bin001_02491	186	259	261	326	166.469	142.325	186.181	hypothetical protein
bin001	SOY3_bin001_02492	114	36	44	36	37.752	39.147	33.545	hypothetical protein
bin001	SOY3_bin001_02493	339	9	28	17	3.174	8.377	5.327	Nitrogen regulatory protein P-II
bin001	SOY3_bin001_02494	1611	223	314	257	16.548	19.769	16.946	Nitrogenase molybdenum-iron protein alpha chain
bin001	SOY3_bin001_02495	105	10	16	10	11.386	15.456	10.117	hypothetical protein
bin001	SOY3_bin001_02496	270	47	53	42	20.810	19.910	16.524	hypothetical protein
bin001	SOY3_bin001_02497	600	98	125	106	19.526	21.131	18.767	hypothetical protein
bin001	SOY3_bin001_02498	168	2	6	4	1.423	3.622	2.529	putative monovalent cation/H+ antiporter subunit B
bin001	SOY3_bin001_02499	600	8	17	15	1.594	2.874	2.656	putative monovalent cation/H+ antiporter subunit B
bin001	SOY3_bin001_02500	486	23	25	27	5.658	5.217	5.901	putative monovalent cation/H+ antiporter subunit B
bin001	SOY3_bin001_02501	429	23	28	11	6.409	6.620	2.724	Na(+)/H(+) antiporter subunit C
bin001	SOY3_bin001_02502	165	17	10	8	12.317	6.147	5.150	hypothetical protein
bin001	SOY3_bin001_02503	630	210	162	170	39.850	26.081	28.664	hypothetical protein
bin001	SOY3_bin001_02504	1449	331	232	234	27.309	16.240	17.154	TPR repeat-containing protein YrrB
bin001	SOY3_bin001_02505	966	150	107	144	18.563	11.235	15.835	Dictyostelium (slime mold) repeat protein
bin001	SOY3_bin001_02506	255	173	262	210	81.106	104.212	87.480	Secreted effector protein PipB
bin001	SOY3_bin001_02507	291	157	282	221	64.499	98.290	80.673	hypothetical protein
bin001	SOY3_bin001_02508	753	387	598	500	61.441	80.549	70.535	hypothetical protein
bin001	SOY3_bin001_02509	678	53	42	38	9.345	6.283	5.954	zinc/cadmium-binding protein
bin001	SOY3_bin001_02510	954	1848	2819	3080	231.579	299.710	342.951	Methyl-accepting chemotaxis protein 4
bin001	SOY3_bin001_02511	2211	1169	1229	1276	63.208	56.379	61.304	Internalin-A precursor
bin001	SOY3_bin001_02512	858	911	1127	1020	126.934	133.227	126.282	hypothetical protein
bin001	SOY3_bin001_02513	1254	822	1087	915	78.365	87.920	77.509	hypothetical protein
bin001	SOY3_bin001_02514	213	6	13	10	3.368	6.190	4.987	hypothetical protein
bin001	SOY3_bin001_02515	786	1148	1856	1700	174.608	239.503	229.750	hypothetical protein
bin001	SOY3_bin001_02516	261	99	69	84	45.346	26.814	34.188	hypothetical protein
bin001	SOY3_bin001_02517	165	62	53	37	44.921	32.580	23.820	hypothetical protein
bin001	SOY3_bin001_02518	462	381	391	368	98.589	85.840	84.613	tRNA-specific adenosine deaminase
bin001	SOY3_bin001_02519	675	144	234	190	25.504	35.161	29.901	PAN domain protein
bin001	SOY3_bin001_02520	1158	1481	1619	1556	152.895	141.805	142.735	putative oxidoreductase FixC
bin001	SOY3_bin001_02521	408	4094	6031	4486	1199.591	1499.284	1167.963	hypothetical protein
bin001	SOY3_bin001_02522	360	240	184	180	79.699	51.841	53.113	hypothetical protein
bin001	SOY3_bin001_02523	606	552	481	502	108.896	80.506	87.996	7-carboxy-7-deazaguanine synthase
bin001	SOY3_bin001_02524	576	331	369	371	68.699	64.977	68.420	transcription factor
bin001	SOY3_bin001_02525	915	710	1116	888	92.765	123.708	103.091	hypothetical protein
bin001	SOY3_bin001_02526	702	541	698	768	92.131	100.849	116.213	tryptophanyl-tRNA synthetase
bin001	SOY3_bin001_02527	270	207	194	322	91.654	72.877	126.684	hypothetical protein
bin001	SOY3_bin001_02528	987	1157	1091	1503	140.140	112.115	161.760	corrinoid ABC transporter substrate-binding protein
bin001	SOY3_bin001_02529	252	115	129	130	54.556	51.921	54.799	hypothetical protein
bin001	SOY3_bin001_02530	1101	532	578	599	57.766	53.247	57.792	GDP-mannose-dependent alpha-(1-6)-phosphatidylinositol monomannoside mannosyltransferase
bin001	SOY3_bin001_02531	1941	5438	4037	5326	334.934	210.954	291.478	putative cadmium-transporting ATPase
bin001	SOY3_bin001_02532	678	523	1065	1468	92.218	159.322	229.999	hypothetical protein
bin001	SOY3_bin001_02533	342	248	592	675	86.690	175.570	209.656	hypothetical protein
bin001	SOY3_bin001_02534	291	268	305	254	110.100	106.307	92.719	hypothetical protein
bin001	SOY3_bin001_02535	435	346	361	347	95.089	84.173	84.736	hypothetical protein
bin001	SOY3_bin001_02536	375	454	662	617	144.734	179.053	174.777	hypothetical protein
bin001	SOY3_bin001_02537	180	46	62	44	30.551	34.936	25.966	hypothetical protein
bin001	SOY3_bin001_02538	699	1202	1250	1369	205.576	181.379	208.044	Serine hydroxymethyltransferase
bin001	SOY3_bin001_02539	864	1756	1836	2007	242.972	215.533	246.754	Bifunctional protein FolD protein
bin001	SOY3_bin001_02540	507	642	736	742	151.381	147.240	155.463	hypothetical protein
bin001	SOY3_bin001_02541	543	194	191	162	42.712	35.677	31.692	hypothetical protein
bin001	SOY3_bin001_02542	648	171	173	157	31.548	27.079	25.737	hypothetical protein
bin001	SOY3_bin001_02543	384	223	219	113	69.426	57.845	31.259	Chagasin family peptidase inhibitor I42

bin001	SOY3_bin001_02544	330	9	13	11	3.260	3.996	3.541	Nitrogen regulatory protein P-II
bin001	SOY3_bin001_02545	261	489	503	523	223.982	195.471	212.859	hypothetical protein
bin001	SOY3_bin001_02546	354	1026	1359	1226	346.489	389.378	367.889	nascent polypeptide-associated complex protein
bin001	SOY3_bin001_02547	612	4010	4753	4530	783.319	787.719	786.279	hypothetical protein
bin001	SOY3_bin001_02548	462	413	557	545	106.869	122.284	125.310	Amylo-alpha-1,6-glucosidase
bin001	SOY3_bin001_02549	1266	1808	1377	1199	170.730	110.320	100.604	Trk system potassium uptake protein TrkH
bin001	SOY3_bin001_02550	537	168	191	228	37.401	36.076	45.101	L(+)-tartrate dehydratase subunit beta
bin001	SOY3_bin001_02551	900	681	754	888	90.459	84.974	104.809	L(+)-tartrate dehydratase subunit alpha
bin001	SOY3_bin001_02552	273	183	231	300	80.137	85.823	116.732	hypothetical protein
bin001	SOY3_bin001_02553	186	95	83	90	61.060	45.261	51.400	hypothetical protein
bin001	SOY3_bin001_02554	1104	3775	2340	2882	408.784	214.982	277.303	Putative transposase DNA-binding domain protein
bin001	SOY3_bin001_02555	840	623	678	613	88.665	81.866	77.520	N5-carboxyaminoimidazole ribonucleotide mutase
bin001	SOY3_bin001_02556	1254	70	62	48	6.673	5.015	4.066	sirohydrochlorin cobaltochelatase
bin001	SOY3_bin001_02557	1023	1904	2123	2000	222.503	210.489	207.675	Alanine--tRNA ligase
bin001	SOY3_bin001_02558	318	271	292	293	101.880	93.134	97.875	hypothetical protein
bin001	SOY3_bin001_02559	936	1515	1293	1196	193.501	140.113	135.733	Cyclic pyranopterin monophosphate synthase
bin001	SOY3_bin001_02560	393	686	758	619	208.678	195.628	167.312	hypothetical protein
bin001	SOY3_bin001_02561	225	96	121	84	51.008	54.545	39.658	hypothetical protein
bin001	SOY3_bin001_02562	519	595	794	794	137.055	155.170	162.511	hypothetical protein
bin001	SOY3_bin001_02563	345	20	10	13	6.930	2.940	4.003	hypothetical protein
bin001	SOY3_bin001_02564	921	2005	2058	2165	260.256	226.642	249.705	Nitrate reductase
bin001	SOY3_bin001_02565	153	240	252	250	187.528	167.057	173.571	hydrogenase nickel incorporation protein HypA
bin001	SOY3_bin001_02566	468	489	551	461	124.913	119.415	104.637	hypothetical protein
bin001	SOY3_bin001_02567	765	242	269	209	37.818	35.665	29.021	TPR repeat-containing protein YrrB
bin001	SOY3_bin001_02568	714	134	157	144	22.436	22.303	21.424	hypothetical protein
bin001	SOY3_bin001_02569	840	153	109	88	21.775	13.161	11.128	hypothetical protein
bin001	SOY3_bin001_02570	777	88	145	86	13.540	18.928	11.757	Archaeal putative transposase
bin001	SOY3_bin001_02571	1146	4760	4853	5523	496.556	429.517	511.941	Soluble hydrogenase 42 kDa subunit
bin001	SOY3_bin001_02572	1236	206	210	192	19.925	17.233	16.501	Inner membrane protein YbiR
bin001	SOY3_bin001_02573	297	205	254	224	82.517	86.743	80.116	Cysteine desulfurase
bin001	SOY3_bin001_02574	330	588	874	931	213.014	268.629	299.685	hypothetical protein
bin001	SOY3_bin001_02575	576	328	499	401	68.076	87.868	73.952	2-amino-4-deoxychorismate dehydrogenase
bin001	SOY3_bin001_02576	357	264	365	261	88.406	103.700	77.661	hypothetical protein
bin001	SOY3_bin001_02577	1416	1141	1432	1411	96.331	102.573	105.851	Mycothione reductase
bin001	SOY3_bin001_02578	387	1030	851	1964	318.179	223.035	539.089	hypothetical protein
bin001	SOY3_bin001_02579	942	1197	812	1041	151.911	87.430	117.390	hypothetical protein
bin001	SOY3_bin001_02580	168	959	817	922	682.425	493.250	582.977	hypothetical protein
bin001	SOY3_bin001_02581	741	291	363	408	46.948	49.687	58.489	hypothetical protein
bin001	SOY3_bin001_02582	486	97	126	118	23.861	26.296	25.791	HIT-like protein
bin001	SOY3_bin001_02583	336	68	78	72	24.194	23.546	22.763	Formamidopyrimidine-DNA glycosylase
bin001	SOY3_bin001_02584	840	635	748	712	90.373	90.319	90.039	hypothetical protein
bin001	SOY3_bin001_02585	231	395	502	483	204.423	220.418	222.108	Histidinol dehydrogenase
bin001	SOY3_bin001_02586	1290	1720	1626	1982	159.399	127.846	163.209	corrinoid ABC transporter substrate-binding protein
bin001	SOY3_bin001_02587	1434	4521	6666	6089	376.904	471.488	451.052	Acetophenone carboxylase gamma subunit
bin001	SOY3_bin001_02588	819	455	744	677	66.416	92.139	87.808	sodium/panthothenate symporter
bin001	SOY3_bin001_02589	426	513	630	596	143.964	149.998	148.616	Coenzyme A biosynthesis bifunctional protein CoaBC
bin001	SOY3_bin001_02590	870	679	709	750	93.303	82.657	91.574	homoserine kinase
bin001	SOY3_bin001_02591	390	27	34	27	8.276	8.842	7.354	hypothetical protein
bin001	SOY3_bin001_02592	633	173	261	236	32.673	41.821	39.604	Universal stress protein/MSMEI_3859
bin001	SOY3_bin001_02593	918	254	291	232	33.078	32.152	26.846	enterobactin exporter EntS
bin001	SOY3_bin001_02594	822	1751	1589	1628	254.659	196.068	210.384	UDP-glucose 4-epimerase
bin001	SOY3_bin001_02595	240	300	262	282	149.436	110.725	124.815	GDP-mannose 4,6-dehydratase
bin001	SOY3_bin001_02596	390	363	423	423	111.272	110.010	115.214	hypothetical protein
bin001	SOY3_bin001_02597	1032	1623	2018	1991	188.011	198.334	204.937	Acetyl-coenzyme A synthetase
bin001	SOY3_bin001_02598	144	36	17	16	29.887	11.974	11.803	hypothetical protein
bin001	SOY3_bin001_02599	210	153	213	174	87.100	102.876	88.016	hypothetical protein
bin001	SOY3_bin001_02600	228	201	257	216	105.392	114.328	100.635	YcfA-like protein
bin001	SOY3_bin001_02601	639	322	394	559	60.242	62.539	92.927	Intermediate filament tail domain protein
bin001	SOY3_bin001_02602	618	303	317	480	58.614	52.027	82.505	Esterase TesA precursor
bin001	SOY3_bin001_02603	318	21	27	26	7.895	8.612	8.685	hypothetical protein
bin001	SOY3_bin001_02604	744	514	312	320	82.592	42.534	45.688	Demethylrebeccamycin-D-glucose O-methyltransferase
bin001	SOY3_bin001_02605	219	75	65	67	40.941	30.104	32.498	hypothetical protein
bin001	SOY3_bin001_02606	636	2085	3099	2650	391.917	494.219	442.607	hypothetical protein
bin001	SOY3_bin001_02607	606	91	176	86	17.952	29.457	15.075	Putative snoRNA binding domain protein
bin001	SOY3_bin001_02608	261	55	61	66	25.192	23.705	26.862	Lipoprotein NlpI precursor
bin001	SOY3_bin001_02609	453	1011	1561	1228	266.808	349.510	287.959	hypothetical protein
bin001	SOY3_bin001_02610	513	319	308	405	74.339	60.896	83.862	hypothetical protein

bin001	SOY3_bin001_02611	240	358	469	433	178.327	198.206	191.649	hypothetical protein
bin001	SOY3_bin001_02612	411	514	547	520	149.509	134.990	134.398	Glyoxalase-like domain protein
bin001	SOY3_bin001_02613	771	962	898	885	149.165	118.134	121.932	Small-conductance mechanosensitive channel
bin001	SOY3_bin001_02614	522	706	642	847	161.689	124.744	172.362	glucose-1-phosphate adenylyltransferase
bin001	SOY3_bin001_02615	237	1272	1205	1308	641.629	515.696	586.259	hypothetical protein
bin001	SOY3_bin001_02616	756	654	747	817	103.419	100.220	114.797	Vitamin B12-binding protein
bin001	SOY3_bin001_02617	1167	239	363	254	24.483	31.549	23.120	hypothetical protein
bin001	SOY3_bin001_02618	513	1528	1565	1546	356.083	309.422	320.127	hypothetical protein
bin001	SOY3_bin001_02619	645	648	765	739	120.105	120.297	121.707	Translocon-associated protein beta (TRAPB)
bin001	SOY3_bin001_02620	459	215	268	217	55.998	59.221	50.220	carbamoyl phosphate synthase-like protein
bin001	SOY3_bin001_02621	73	6	2	4	9.826	2.779	5.821	tRNA-Pro(ggg)
bin001	SOY3_bin001_02622	249	235	244	217	112.827	99.391	92.574	hypothetical protein
bin001	SOY3_bin001_02623	684	382	464	491	66.766	68.804	76.253	Xaa-Pro dipeptidase
bin001	SOY3_bin001_02624	531	120	114	129	27.017	21.775	25.806	hypothetical protein
bin001	SOY3_bin001_02625	609	296	375	344	58.106	62.455	60.003	Pyrroline-5-carboxylate reductase
bin001	SOY3_bin001_02626	237	59	44	42	29.761	18.830	18.825	hypothetical protein
bin001	SOY3_bin001_02627	195	758	768	797	464.708	399.467	434.164	hypothetical protein
bin001	SOY3_bin001_02628	753	219	312	273	34.769	42.026	38.512	hypothetical protein
bin001	SOY3_bin001_02629	294	26	44	36	10.572	15.180	13.007	hypothetical protein
bin001	SOY3_bin001_02630	954	802	735	486	100.501	78.144	54.115	Cyclic pyranopterin monophosphate synthase
bin001	SOY3_bin001_02631	102	141	149	143	165.259	148.163	148.924	hypothetical protein
bin001	SOY3_bin001_02632	375	27	32	29	8.608	8.655	8.215	Nitrogen regulatory protein P-II
bin001	SOY3_bin001_02633	705	54	69	56	9.157	9.927	8.438	hypothetical protein
bin001	SOY3_bin001_02634	948	2005	1746	2296	252.843	186.806	257.272	Arginine transport ATP-binding protein ArtM
bin001	SOY3_bin001_02635	117	0	0	1	0.000	0.000	0.908	hypothetical protein
bin001	SOY3_bin001_02636	519	1501	1896	2520	345.747	370.532	515.778	Hemin transport system permease protein HmuU
bin001	SOY3_bin001_02637	318	61	304	165	22.932	96.962	55.117	hypothetical protein
bin001	SOY3_bin001_02638	321	420	625	682	156.419	197.483	225.688	hypothetical protein
bin001	SOY3_bin001_02639	891	17464	16712	19559	2343.212	1902.416	2331.840	tetrahydromethanopterin S-methyltransferase subunit E
bin001	SOY3_bin001_02640	651	53	38	52	9.733	5.920	8.485	EamA-like transporter family protein
bin001	SOY3_bin001_02641	498	503	326	363	120.749	66.396	77.430	Calcium-transporting ATPase
bin001	SOY3_bin001_02642	225	21	30	23	11.158	13.524	10.859	hypothetical protein
bin001	SOY3_bin001_02643	249	154	211	197	73.938	85.948	84.042	hypothetical protein
bin001	SOY3_bin001_02644	705	189	164	144	32.049	23.594	21.697	hypothetical protein
bin001	SOY3_bin001_02645	105	463	556	536	527.154	537.082	542.257	hypothetical protein
bin001	SOY3_bin001_02646	675	1080	1256	1300	191.278	188.730	204.583	Uridylate kinase
bin001	SOY3_bin001_02647	576	696	910	785	144.455	160.241	144.769	SPFH domain / Band 7 family protein
bin001	SOY3_bin001_02648	156	316	331	284	242.163	215.208	193.385	hypothetical protein
bin001	SOY3_bin001_02649	1059	386	979	587	43.575	93.765	58.881	Transposase DDE domain protein
bin001	SOY3_bin001_02650	1257	389	229	587	36.996	18.478	49.606	corrinoid ABC transporter substrate-binding protein
bin001	SOY3_bin001_02651	1128	5780	3692	8800	612.582	331.977	828.712	putative ABC transporter solute-binding protein YclQ precursor
bin001	SOY3_bin001_02652	1080	3844	2301	5687	425.505	216.096	559.357	Hemin transport system permease protein HmuU
bin001	SOY3_bin001_02653	921	2078	1216	2954	269.731	133.915	340.707	putative siderophore transport system ATP-binding protein YusV
bin001	SOY3_bin001_02654	1128	3096	1447	3466	328.124	130.111	326.399	High-affinity heme uptake system protein IsdE precursor
bin001	SOY3_bin001_02655	816	1141	540	1176	167.163	67.121	153.090	putative methyltransferase YcgJ
bin001	SOY3_bin001_02656	864	1127	546	1303	155.939	64.096	160.199	Malonyl-[acyl-carrier protein] O-methyltransferase
bin001	SOY3_bin001_02657	936	1024	482	1161	130.789	52.231	131.761	Demethylmenaquinone methyltransferase
bin001	SOY3_bin001_02658	1287	466	366	568	43.287	28.844	46.881	corrinoid ABC transporter substrate-binding protein
bin001	SOY3_bin001_02659	228	80	39	82	41.947	17.349	38.204	Hemin import ATP-binding protein HmuV
bin001	SOY3_bin001_02660	753	138	99	165	21.909	13.335	23.277	Ubiquinone biosynthesis O-methyltransferase
bin001	SOY3_bin001_02661	135	23	15	13	20.368	11.270	10.229	hypothetical protein
bin001	SOY3_bin001_02662	522	689	1022	1008	157.795	198.580	205.126	hypothetical protein
bin001	SOY3_bin001_02663	447	624	925	999	166.887	209.889	237.404	hypothetical protein
bin001	SOY3_bin001_02664	2640	227	179	288	10.279	6.877	11.588	ABC-type uncharacterized transport system
bin001	SOY3_bin001_02665	2505	356	285	593	16.990	11.540	25.146	ABC-type uncharacterized transport system
bin001	SOY3_bin001_02666	351	1103	1738	3251	375.676	502.224	983.874	hypothetical protein
bin001	SOY3_bin001_02667	1065	11637	11901	15399	1306.282	1133.414	1535.935	TPR repeat-containing protein YrrB
bin001	SOY3_bin001_02668	273	229	400	489	100.281	148.611	190.273	Non-histone chromosomal protein MC1
bin001	SOY3_bin001_02669	1380	2240	2072	3034	194.050	152.288	233.543	High-affinity heme uptake system protein IsdE precursor
bin001	SOY3_bin001_02670	234	195	151	213	99.624	65.451	96.693	hypothetical protein
bin001	SOY3_bin001_02671	507	866	746	783	204.200	149.240	164.053	hypothetical protein
bin001	SOY3_bin001_02672	243	537	409	404	264.188	170.715	176.606	Sugar-specific transcriptional regulator TrmB
bin001	SOY3_bin001_02673	576	3082	4084	3482	639.670	719.148	642.149	Bacterial regulatory proteins, luxR family
bin001	SOY3_bin001_02674	609	50	73	119	9.815	12.158	20.757	hypothetical protein
bin001	SOY3_bin001_02675	1239	32	27	34	3.088	2.210	2.915	Transposase DDE domain protein
bin001	SOY3_bin001_02676	2469	720	863	1200	34.862	35.452	51.629	Modification methylase RsrI
bin001	SOY3_bin001_02677	870	95	114	192	13.054	13.290	23.443	Modification methylase DpnIIB

bin001	SOY3_bin001_02678	456	10215	10585	10092	2678.052	2354.403	2350.944	transcription antitermination protein NusG
bin001	SOY3_bin001_02679	477	9121	9643	8935	2285.965	2050.447	1989.784	50S ribosomal protein L11
bin001	SOY3_bin001_02680	633	24538	22648	21766	4634.266	3628.949	3652.622	50S ribosomal protein L1
bin001	SOY3_bin001_02681	975	28505	25809	24438	3495.119	2684.858	2662.508	50S ribosomal protein L10
bin001	SOY3_bin001_02682	1299	1136	871	1023	104.548	68.009	83.656	Alpha-monoglucosyldiacylglycerol synthase
bin001	SOY3_bin001_02683	1050	1720	1315	1422	195.832	127.026	143.860	Putative glycosyltransferase EpsD
bin001	SOY3_bin001_02684	1338	2796	2768	2897	249.820	209.829	229.997	putative S-adenosylmethionine-dependent methyltransferase/MSMEI_2290
bin001	SOY3_bin001_02685	108	245	157	187	271.199	147.445	183.928	hypothetical protein
bin001	SOY3_bin001_02686	663	4593	4017	4434	828.187	614.530	710.415	hypothetical protein
bin001	SOY3_bin001_02687	204	102	71	76	59.774	35.301	39.574	hypothetical protein
bin001	SOY3_bin001_02688	1368	1143	1256	1438	99.886	93.123	111.661	putative S-adenosylmethionine-dependent methyltransferase/MSMEI_2290
bin001	SOY3_bin001_02689	789	373	323	441	56.517	41.522	59.373	hypothetical protein
bin001	SOY3_bin001_02690	630	148	138	143	28.084	22.217	24.112	DNA-binding transcriptional regulator LysR
bin001	SOY3_bin001_02691	846	263	213	241	37.165	25.537	30.261	hypothetical protein
bin001	SOY3_bin001_02692	2571	701	564	689	32.596	22.250	28.467	hypothetical protein
bin001	SOY3_bin001_02693	444	170	107	165	45.773	24.443	39.476	hypothetical protein
bin001	SOY3_bin001_02694	999	495	300	424	59.236	30.459	45.085	hypothetical protein
bin001	SOY3_bin001_02695	756	1067	991	1039	168.728	132.956	145.990	hypothetical protein
bin001	SOY3_bin001_02696	1758	2508	3451	3224	170.551	199.104	194.808	hypothetical protein
bin001	SOY3_bin001_02697	654	253	132	150	46.248	20.472	24.364	Acetyl-coenzyme A carboxylase carboxyl transferase subunit beta, chloroplastic
bin001	SOY3_bin001_02698	420	353	208	227	100.478	50.231	57.412	Helix-turn-helix domain protein
bin001	SOY3_bin001_02699	381	377	241	299	118.294	64.157	83.364	Transposase IS200 like protein
bin001	SOY3_bin001_02700	822	2	0	1	0.291	0.000	0.129	hypothetical protein
bin001	SOY3_bin001_02701	354	0	0	0	0.000	0.000	0.000	hypothetical protein
bin001	SOY3_bin001_02702	498	101326	90819	104834	24324.117	18497.035	22361.588	hypothetical protein
bin001	SOY3_bin001_02703	1509	16029	14635	14657	1269.880	983.690	1031.777	hypothetical protein
bin001	SOY3_bin001_02704	909	3376	3392	3658	444.001	378.483	427.474	hypothetical protein
bin001	SOY3_bin001_02705	282	382	178	183	161.942	64.021	68.934	tRNA pseudouridine synthase A
bin001	SOY3_bin001_02706	546	304	357	357	66.562	66.318	69.455	hypothetical protein
bin001	SOY3_bin001_02707	1014	510	672	654	60.128	67.218	68.512	Putative thiosulfate sulfurtransferase SseA
bin001	SOY3_bin001_02708	255	1206	777	829	565.396	309.055	345.338	hypothetical protein
bin001	SOY3_bin001_02709	1038	296	258	240	34.091	25.210	24.561	Poly-beta-1,6-N-acetyl-D-glucosamine synthase
bin001	SOY3_bin001_02710	216	416	372	353	230.242	174.680	173.600	hypothetical protein
bin001	SOY3_bin001_02711	309	1088	1229	1207	420.936	403.411	414.934	hypothetical protein
bin001	SOY3_bin001_02712	474	672	1733	1533	169.487	370.830	343.553	hypothetical protein
bin001	SOY3_bin001_02713	471	125	144	111	31.727	31.010	25.034	Phosphate transport system permease protein PstA
bin001	SOY3_bin001_02714	579	73	51	80	15.073	8.934	14.677	Transposase DDE domain protein
bin001	SOY3_bin001_02715	87	53	68	42	72.829	79.276	51.281	tRNA-Ser(cag)
bin001	SOY3_bin001_02716	2157	82	47	58	4.545	2.210	2.856	primosome assembly protein PriA
bin001	SOY3_bin001_02717	789	194	95	117	29.395	12.212	15.752	hypothetical protein
bin001	SOY3_bin001_02718	1014	342	455	463	40.321	45.512	48.503	photosystem I assembly protein Ycf3
bin001	SOY3_bin001_02719	1131	209	277	304	22.092	24.841	28.552	CHAT domain protein
bin001	SOY3_bin001_02720	498	194	209	245	46.571	42.567	52.260	flagellar hook-basal body protein FliE
bin001	SOY3_bin001_02721	318	41	20	28	15.414	6.379	9.353	hypothetical protein
bin001	SOY3_bin001_02722	207	364	336	363	210.221	164.636	186.280	hypothetical protein
bin001	SOY3_bin001_02723	468	144	121	160	36.784	26.224	36.316	Phosphatase/MT3486
bin001	SOY3_bin001_02724	159	29	11	36	21.805	7.017	24.051	hypothetical protein
bin001	SOY3_bin001_02725	228	101	100	147	52.958	44.486	68.488	Acylphosphatase
bin001	SOY3_bin001_02726	828	286	314	252	41.293	38.464	32.330	Ubiquinone/menaquinone biosynthesis C-methyltransferase UbiE
bin001	SOY3_bin001_02727	276	81	100	71	35.085	36.749	27.326	hypothetical protein
bin001	SOY3_bin001_02728	294	77	72	64	31.310	24.839	23.124	hypothetical protein
bin001	SOY3_bin001_02729	420	170	181	153	48.389	43.710	38.697	hypothetical protein
bin001	SOY3_bin001_02730	1068	7945	6370	10288	889.341	604.955	1023.268	FmdE, Molybdenum formylmethanofuran dehydrogenase operon
bin001	SOY3_bin001_02731	552	627	857	774	135.792	157.470	148.947	hypothetical protein
bin001	SOY3_bin001_02732	318	520	654	631	195.489	208.596	210.781	hypothetical protein
bin001	SOY3_bin001_02733	117	40	46	38	40.871	39.877	34.501	hypothetical protein
bin001	SOY3_bin001_02734	603	400	446	502	79.303	75.019	88.433	Putative 5'(3')-deoxyribonucleotidase
bin001	SOY3_bin001_02735	930	83	131	334	10.669	14.287	38.150	corrinoid ABC transporter substrate-binding protein
bin001	SOY3_bin001_02736	402	16	30	74	4.758	7.569	19.554	Periplasmic binding protein
bin001	SOY3_bin001_02737	447	6	13	15	1.605	2.950	3.565	hypothetical protein
bin001	SOY3_bin001_02738	1035	86	132	103	9.934	12.936	10.571	hypothetical protein
bin001	SOY3_bin001_02739	363	229	280	117	75.418	78.236	34.238	Transposase IS200 like protein
bin001	SOY3_bin001_02740	1101	463	593	270	50.274	54.629	26.050	putative transposase
bin001	SOY3_bin001_02741	417	218	314	220	62.498	76.374	56.042	antiporter inner membrane protein
bin001	SOY3_bin001_02742	729	1310	1009	960	214.827	140.384	139.886	hypothetical protein
bin001	SOY3_bin001_02743	201	234	144	188	139.176	72.664	99.355	hypothetical protein
bin001	SOY3_bin001_02744	756	261	432	353	41.273	57.958	49.600	hypothetical protein

bin001	SOY3_bin001_02745	255	291	266	295	136.426	105.803	122.889	hypothetical protein
bin001	SOY3_bin001_02746	552	664	840	790	143.805	154.346	152.026	hypothetical protein
bin001	SOY3_bin001_02747	504	47	72	90	11.148	14.490	18.969	hypothetical protein
bin001	SOY3_bin001_02748	522	86	134	151	19.696	26.037	30.728	Secreted effector protein pipB2
bin001	SOY3_bin001_02749	348	48	37	44	16.490	10.784	13.431	hypothetical protein
bin001	SOY3_bin001_02750	990	1196	1374	1215	144.425	140.769	130.368	UDP-glucose 4-epimerase
bin001	SOY3_bin001_02751	522	198	142	154	45.346	27.591	31.339	photosystem I assembly protein Ycf3
bin001	SOY3_bin001_02752	426	32	52	49	8.980	12.381	12.218	hypothetical protein
bin001	SOY3_bin001_02753	114	40	28	44	41.947	24.912	40.999	hypothetical protein
bin001	SOY3_bin001_02754	624	19	30	11	3.640	4.876	1.873	hypothetical protein
bin001	SOY3_bin001_02755	474	24	54	29	6.053	11.555	6.499	hypothetical protein
bin001	SOY3_bin001_02756	312	121	158	145	46.364	51.364	49.368	hypothetical protein
bin001	SOY3_bin001_02757	558	103	75	106	22.067	13.633	20.179	Transposase IS116/IS110/IS902 family protein
bin001	SOY3_bin001_02758	711	1200	1418	1364	201.770	202.284	203.786	hypothetical protein
bin001	SOY3_bin001_02759	423	722	868	790	204.053	208.130	198.389	Chromosome partition protein Smc
bin001	SOY3_bin001_02760	837	909	795	828	129.833	96.338	105.084	Benzylsuccinate synthase activating enzyme
bin001	SOY3_bin001_02761	624	311	369	333	59.583	59.979	56.688	Adenylate kinase
bin001	SOY3_bin001_02762	1929	853	715	642	52.864	37.595	35.354	hypothetical protein
bin001	SOY3_bin001_02763	555	294	482	413	63.329	88.086	79.047	putative DNA repair protein YkoV
bin001	SOY3_bin001_02764	948	413	620	518	52.082	66.334	58.043	Putative DNA ligase-like protein/MT0965
bin001	SOY3_bin001_02765	156	17	26	12	13.028	16.905	8.171	putative permease
bin001	SOY3_bin001_02766	498	391	345	427	93.863	70.266	91.081	Arsenate-mycothiol transferase ArsC1
bin001	SOY3_bin001_02767	261	400	456	496	183.217	177.206	201.870	Ferredoxin
bin001	SOY3_bin001_02768	297	46	28	43	18.516	9.562	15.379	hypothetical protein
bin001	SOY3_bin001_02769	1422	206	232	247	17.319	16.548	18.451	Thiol-disulfide oxidoreductase ResA
bin001	SOY3_bin001_02770	546	211	203	233	46.199	37.710	45.331	hypothetical protein
bin001	SOY3_bin001_02771	2508	519	454	516	24.739	18.360	21.855	TPR repeat-containing protein YrrB
bin001	SOY3_bin001_02772	381	120	120	159	37.653	31.946	44.330	hypothetical protein
bin001	SOY3_bin001_02773	282	24	43	52	10.174	15.466	19.588	hypothetical protein
bin001	SOY3_bin001_02774	1062	199	259	303	22.401	24.736	30.307	hypothetical protein
bin001	SOY3_bin001_02775	1320	243	179	237	22.008	13.754	19.072	TPR repeat-containing protein YrrB
bin001	SOY3_bin001_02776	528	147	83	116	33.283	15.944	23.337	photosystem I assembly protein Ycf3
bin001	SOY3_bin001_02777	435	219	399	371	60.187	93.033	90.597	hypothetical protein
bin001	SOY3_bin001_02778	228	478	413	352	250.633	183.726	163.998	hypothetical protein
bin001	SOY3_bin001_02779	564	221	314	156	46.845	56.468	29.382	Coenzyme F420:L-glutamate ligase
bin001	SOY3_bin001_02780	468	177	233	127	45.214	50.497	28.826	(S)-2-haloacid dehalogenase 4A
bin001	SOY3_bin001_02781	681	241	234	275	42.307	34.852	42.896	hypothetical protein
bin001	SOY3_bin001_02782	873	111	141	132	15.200	16.382	16.062	Shikimate dehydrogenase
bin001	SOY3_bin001_02783	750	158	221	162	25.185	29.887	22.945	TPR repeat-containing protein YrrB
bin001	SOY3_bin001_02784	228	5	4	1	2.622	1.779	0.466	MarR family protein
bin001	SOY3_bin001_02785	786	2189	2880	2771	332.942	371.642	374.493	5'-nucleotidase SurE
bin001	SOY3_bin001_02786	888	850	1007	1281	114.433	115.019	153.238	hypothetical protein
bin001	SOY3_bin001_02787	1224	70	66	84	6.837	5.469	7.290	putative hydrolase
bin001	SOY3_bin001_02788	183	15	17	16	9.799	9.422	9.287	O-acetylserine/cysteine export protein
bin001	SOY3_bin001_02789	99	0	0	1	0.000	0.000	1.073	hypothetical protein
bin001	SOY3_bin001_02790	129	293	171	217	271.534	134.450	178.690	hypothetical protein
bin001	SOY3_bin001_02791	1134	1589	1516	2124	167.516	135.594	198.963	ATP-NAD kinase
bin001	SOY3_bin001_02792	306	572	682	979	223.470	226.057	339.853	Tryptophan synthase beta chain
bin001	SOY3_bin001_02793	432	255	255	202	70.567	59.870	49.670	hypothetical protein
bin001	SOY3_bin001_02794	261	16	33	12	7.329	12.824	4.884	hypothetical protein
bin001	SOY3_bin001_02795	225	21	13	9	11.158	5.860	4.249	hypothetical protein
bin001	SOY3_bin001_02796	183	1613	1440	1436	1053.729	798.116	833.553	hypothetical protein
bin001	SOY3_bin001_02797	234	1258	1833	1963	642.703	794.514	891.116	Thrombin inhibitor from mosquito
bin001	SOY3_bin001_02798	1413	978	925	956	82.745	66.398	71.870	PKD domain protein
bin001	SOY3_bin001_02799	663	744	607	663	134.154	92.860	106.226	cell division control protein 6
bin001	SOY3_bin001_02800	558	208	459	816	44.563	83.432	155.341	Sodium/pantothenate symporter
bin001	SOY3_bin001_02801	267	1218	2059	2088	545.358	782.168	830.709	Non-histone chromosomal protein MC1
bin001	SOY3_bin001_02802	252	756	811	910	358.647	326.419	383.593	Asparagine--tRNA ligase
bin001	SOY3_bin001_02803	138	39	55	37	33.786	40.424	28.481	hypothetical protein
bin001	SOY3_bin001_02804	801	122	202	141	18.208	25.578	18.699	Ribosomal RNA large subunit methyltransferase K/L
bin001	SOY3_bin001_02805	258	485	565	574	224.733	222.118	236.332	hypothetical protein
bin001	SOY3_bin001_02806	225	188	164	229	99.890	73.929	108.114	hypothetical protein
bin001	SOY3_bin001_02807	231	587	926	804	303.789	406.587	369.721	hypothetical protein
bin001	SOY3_bin001_02808	249	32	37	26	15.364	15.072	11.092	hypothetical protein
bin001	SOY3_bin001_02809	684	138	219	330	24.120	32.475	51.249	Spermidine/putrescine import ATP-binding protein PotA
bin001	SOY3_bin001_02810	507	242	231	352	57.063	46.212	73.750	Imidazole glycerol phosphate synthase subunit HisF
bin001	SOY3_bin001_02811	762	830	449	594	130.217	59.765	82.806	tRNA pseudouridine synthase D

bin001	SOY3_bin001_02812	273	128	316	272	56.052	117.403	105.837	hypothetical protein
bin001	SOY3_bin001_02813	267	257	264	242	115.071	100.288	96.280	hypothetical protein
bin001	SOY3_bin001_02814	459	481	518	506	125.279	114.465	117.103	hypothetical protein
bin001	SOY3_bin001_02815	294	418	484	392	169.971	166.976	141.634	hypothetical protein
bin001	SOY3_bin001_02816	228	697	1347	980	365.463	599.222	456.584	Tetrahydromethanopterin S-methyltransferase, F subunit (MtrF)
bin001	SOY3_bin001_02817	732	16219	17619	18318	2648.857	2441.321	2658.256	tetrahydromethanopterin S-methyltransferase subunit A
bin001	SOY3_bin001_02818	432	264	246	212	73.058	57.757	52.129	hypothetical protein
bin001	SOY3_bin001_02819	411	667	394	473	194.012	97.232	122.250	Nitroreductase family protein
bin001	SOY3_bin001_02820	345	138	148	140	47.820	43.511	43.106	hypothetical protein
bin001	SOY3_bin001_02821	1062	646	840	740	72.720	80.225	74.018	2-octaprenyl-3-methyl-6-methoxy-1,4-benzoquinol hydroxylase
bin001	SOY3_bin001_02822	438	145	207	177	39.577	47.935	42.927	hypothetical protein
bin001	SOY3_bin001_02823	660	402	525	444	72.816	80.681	71.461	Ferrous-iron efflux pump FieF
bin001	SOY3_bin001_02824	693	167	190	149	28.809	27.808	22.839	Acetyltransferase (GNAT) family protein
bin001	SOY3_bin001_02825	381	96	152	99	30.123	40.464	27.602	succinate dehydrogenase/fumarate reductase iron-sulfur subunit
bin001	SOY3_bin001_02826	720	548	621	759	90.990	87.481	111.980	hypothetical protein
bin001	SOY3_bin001_02827	561	104	103	109	22.162	18.622	20.639	2,5-diamino-6-ribosylamino-4(3H)-pyrimidinone 5'-phosphate reductase
bin001	SOY3_bin001_02828	762	2770	2572	3740	434.581	342.350	521.370	Hemin import ATP-binding protein HmuV
bin001	SOY3_bin001_02829	930	1028	1127	1087	132.147	122.912	124.158	Lysine--tRNA ligase
bin001	SOY3_bin001_02830	381	116	117	134	36.398	31.147	37.360	Peroxyureidoacrylate/ureidoacrylate amidohydrolase RutB
bin001	SOY3_bin001_02831	96	54	51	34	67.246	53.883	37.622	hypothetical protein
bin001	SOY3_bin001_02832	312	744	841	863	285.078	273.399	293.823	hypothetical protein
bin001	SOY3_bin001_02833	252	4	10	9	1.898	4.025	3.794	hypothetical protein
bin001	SOY3_bin001_02834	180	176	227	261	116.892	127.911	154.027	Putative multidrug export ATP-binding/permease protein
bin001	SOY3_bin001_02835	639	1078	1374	1244	201.680	218.092	206.799	hypothetical protein
bin001	SOY3_bin001_02836	456	45	67	48	11.798	14.903	11.182	hypothetical protein
bin001	SOY3_bin001_02837	603	6158	12016	9716	1220.866	2021.144	1711.591	hypothetical protein
bin001	SOY3_bin001_02838	86	68	60	48	94.527	70.763	59.289	tRNA-Leu(taa)
bin001	SOY3_bin001_02839	276	12	5	10	5.198	1.837	3.849	hypothetical protein
bin001	SOY3_bin001_02840	2997	37	30	24	1.476	1.015	0.851	hypothetical protein
bin001	SOY3_bin001_02841	156	0	1	1	0.000	0.650	0.681	hypothetical protein
bin001	SOY3_bin001_02842	363	7	10	10	2.305	2.794	2.926	CRS1 / YhbY (CRM) domain protein
bin001	SOY3_bin001_02843	585	20	19	22	4.087	3.294	3.995	chromosome segregation protein
bin001	SOY3_bin001_02844	192	3	2	1	1.868	1.057	0.553	hypothetical protein
bin001	SOY3_bin001_02845	168	20	6	7	14.232	3.622	4.426	hypothetical protein
bin001	SOY3_bin001_02846	2955	28	15	33	1.133	0.515	1.186	DNA primase large subunit
bin001	SOY3_bin001_02847	600	13	9	13	2.590	1.521	2.302	hypothetical protein
bin001	SOY3_bin001_02848	579	7	9	6	1.445	1.577	1.101	hypothetical protein
bin001	SOY3_bin001_02849	222	1	1	1	0.539	0.457	0.478	hypothetical protein
bin001	SOY3_bin001_02850	195	0	1	0	0.000	0.520	0.000	hypothetical protein
bin001	SOY3_bin001_02851	228	2	1	1	1.049	0.445	0.466	hypothetical protein
bin001	SOY3_bin001_02852	363	7	4	6	2.305	1.118	1.756	hypothetical protein
bin001	SOY3_bin001_02853	246	14	14	19	6.804	5.772	8.204	hypothetical protein
bin001	SOY3_bin001_02854	1194	80	91	130	8.010	7.730	11.566	hypothetical protein
bin001	SOY3_bin001_02855	546	119	136	184	26.056	25.264	35.798	CRISPR-associated protein (Cas_NE0113)
bin001	SOY3_bin001_02856	354	154	158	198	52.007	45.270	59.414	hypothetical protein
bin001	SOY3_bin001_02857	981	471	347	444	57.398	35.877	48.078	RAMP superfamily protein
bin001	SOY3_bin001_02858	450	228	204	301	60.571	45.980	71.053	hypothetical protein
bin001	SOY3_bin001_02859	915	346	303	450	45.206	33.587	52.242	RAMP superfamily protein
bin001	SOY3_bin001_02860	819	343	285	474	50.067	35.295	61.479	RAMP superfamily protein
bin001	SOY3_bin001_02861	393	172	201	304	52.322	51.875	82.170	DNA polymerase III subunit delta
bin001	SOY3_bin001_02862	1233	512	488	657	49.642	40.143	56.602	RAMP superfamily protein
bin001	SOY3_bin001_02863	672	305	240	372	54.260	36.224	58.804	RAMP superfamily protein
bin001	SOY3_bin001_02864	1680	619	523	758	44.048	31.575	47.928	hypothetical protein
bin001	SOY3_bin001_02865	849	641	883	1089	90.260	105.489	136.254	CRISPR-associated protein, family (Cas_GSU0054)
bin001	SOY3_bin001_02866	1335	19	14	25	1.701	1.064	1.989	Phenylacetate-coenzyme A ligase
bin001	SOY3_bin001_02867	1164	17	14	24	1.746	1.220	2.190	Putative teichuronic acid biosynthesis glycosyltransferase TuaC
bin001	SOY3_bin001_02868	1692	6	3	4	0.424	0.180	0.251	hypothetical protein
bin001	SOY3_bin001_02869	1254	27	28	36	2.574	2.265	3.050	Polysaccharide biosynthesis protein
bin001	SOY3_bin001_02870	1440	46	34	36	3.819	2.395	2.656	UDP-N-acetyl-D-glucosamine 6-dehydrogenase
bin001	SOY3_bin001_02871	990	56	33	51	6.762	3.381	5.472	UDP-glucose 4-epimerase
bin001	SOY3_bin001_02872	477	242	278	243	60.652	59.113	54.115	hypothetical protein
bin001	SOY3_bin001_02873	1266	118	662	317	11.143	53.037	26.598	Alkaline phosphatase 4 precursor
bin001	SOY3_bin001_02874	234	11	17	13	5.620	7.369	5.901	AsnC family protein
bin001	SOY3_bin001_02875	864	40	30	30	5.535	3.522	3.688	Tyrosine recombinase XerD
bin001	SOY3_bin001_02876	789	172	131	161	26.061	16.840	21.676	hypothetical protein
bin001	SOY3_bin001_02877	1197	737	597	823	73.607	50.587	73.036	Divergent AAA domain protein
bin001	SOY3_bin001_02878	1299	349	403	646	32.119	31.467	52.827	hypothetical protein

bin001	SOY3_bin001_02879	156	23	13	19	17.626	8.452	12.938	hypothetical protein
bin001	SOY3_bin001_02880	858	19	18	19	2.647	2.128	2.352	Tyrosine recombinase XerD
bin001	SOY3_bin001_02881	390	8	8	7	2.452	2.081	1.907	hypothetical protein
bin001	SOY3_bin001_02882	213	1	3	2	0.561	1.429	0.997	hypothetical protein
bin001	SOY3_bin001_02883	270	6	7	15	2.657	2.630	5.901	hypothetical protein
bin001	SOY3_bin001_02884	201	26	13	25	15.464	6.560	13.212	hypothetical protein
bin001	SOY3_bin001_02885	564	45	29	62	9.538	5.215	11.677	hypothetical protein
bin001	SOY3_bin001_02886	276	0	0	2	0.000	0.000	0.770	hypothetical protein
bin001	SOY3_bin001_02887	216	0	0	0	0.000	0.000	0.000	hypothetical protein
bin001	SOY3_bin001_02888	1050	205	193	264	23.340	18.643	26.708	hypothetical protein
bin001	SOY3_bin001_02889	471	46	34	49	11.676	7.322	11.051	hypothetical protein
bin001	SOY3_bin001_02890	642	332	552	263	61.823	87.208	43.516	Undecaprenyl-phosphate mannosyltransferase
bin001	SOY3_bin001_02891	1095	19	9	10	2.074	0.834	0.970	dTDP-3-amino-3,6-dideoxy-alpha-D-galactopyranose transaminase
bin001	SOY3_bin001_02892	975	14	11	9	1.717	1.144	0.981	Inositol 2-dehydrogenase/D-chiro-inositol 3-dehydrogenase
bin001	SOY3_bin001_02893	138	1	2	0	0.866	1.470	0.000	hypothetical protein
bin001	SOY3_bin001_02894	966	11	6	20	1.361	0.630	2.199	Undecaprenyl-phosphate mannosyltransferase
bin001	SOY3_bin001_02895	1089	26	17	30	2.854	1.583	2.926	tRNA-specific 2-thiouridylase MnmA
bin001	SOY3_bin001_02896	1068	15	15	19	1.679	1.425	1.890	hypothetical protein
bin001	SOY3_bin001_02897	2049	570	612	665	33.257	30.295	34.475	Type IIS restriction enzyme Eco57I
bin001	SOY3_bin001_02898	711	339	198	233	57.000	28.246	34.811	hypothetical protein
bin001	SOY3_bin001_02899	2127	288	218	317	16.187	10.395	15.831	ski2-like helicase
bin001	SOY3_bin001_02900	225	32	22	41	17.003	9.917	19.357	hypothetical protein
bin001	SOY3_bin001_02901	579	319	281	274	65.865	49.225	50.269	Rhodopirellula transposase
bin001	SOY3_bin001_02902	306	12	65	30	4.688	21.545	10.414	hypothetical protein
bin001	SOY3_bin001_02903	1425	5	13	14	0.419	0.925	1.044	Transposase DDE domain protein
bin001	SOY3_bin001_02904	83	20	50	38	28.807	61.101	48.633	tRNA-Leu(tag)
bin001	SOY3_bin001_02905	615	0	1	0	0.000	0.165	0.000	Tyrosine recombinase XerC
bin001	SOY3_bin001_02906	387	2	1	1	0.618	0.262	0.274	hypothetical protein
bin001	SOY3_bin001_02907	1035	14	24	16	1.617	2.352	1.642	lipoprotein NlpI
bin001	SOY3_bin001_02908	1356	627	446	644	55.278	33.360	50.449	hypothetical protein
bin001	SOY3_bin001_02909	969	351	279	302	43.304	29.204	33.106	Bacterial regulatory protein, arsR family
bin001	SOY3_bin001_02910	1260	76	51	63	7.211	4.105	5.311	Transposase DDE domain protein
bin001	SOY3_bin001_02911	270	186	126	131	82.356	47.333	51.539	hypothetical protein
bin001	SOY3_bin001_02912	204	140	140	163	82.043	69.607	84.876	hypothetical protein
bin001	SOY3_bin001_02913	3435	244	217	284	8.492	6.407	8.783	hypothetical protein
bin001	SOY3_bin001_02914	684	6	3	8	1.049	0.445	1.242	hypothetical protein
bin001	SOY3_bin001_02915	753	342	225	286	54.297	30.307	40.346	DNA-directed RNA polymerase subunit P
bin001	SOY3_bin001_02916	1236	838	824	1305	81.053	67.618	112.156	AAA-like domain protein
bin001	SOY3_bin001_02917	198	327	337	327	197.437	172.631	175.433	hypothetical protein
bin001	SOY3_bin001_02918	1095	810	1063	1042	88.433	98.463	101.084	N-acetylglucosaminyl-diphospho-decaprenol L-rhamnosyltransferase
bin001	SOY3_bin001_02919	1524	160	192	167	12.551	12.778	11.640	hypothetical protein
bin001	SOY3_bin001_02920	1428	375	517	518	31.394	36.721	38.533	HNH endonuclease
bin001	SOY3_bin001_02921	495	317	519	483	76.560	106.345	103.651	hypothetical protein
bin001	SOY3_bin001_02922	708	19	35	31	3.208	5.014	4.651	hypothetical protein
bin001	SOY3_bin001_02923	339	28	34	27	9.874	10.173	8.460	hypothetical protein
bin001	SOY3_bin001_02924	360	139	150	208	46.159	42.261	61.375	4-amino-4-deoxy-L-arabinose-phosphoundecaprenol flippase subunit ArnF
bin001	SOY3_bin001_02925	663	556	498	683	100.255	76.185	109.430	Error-prone DNA polymerase
bin001	SOY3_bin001_02926	900	812	773	865	107.860	87.115	102.095	Decaprenyl-phosphate phosphoribosyltransferase
bin001	SOY3_bin001_02927	315	5	5	4	1.898	1.610	1.349	hypothetical protein
bin001	SOY3_bin001_02928	180	2	1	1	1.328	0.563	0.590	hypothetical protein
bin001	SOY3_bin001_02929	492	5	2	7	1.215	0.412	1.511	hypothetical protein
bin001	SOY3_bin001_02930	246	4	3	4	1.944	1.237	1.727	hypothetical protein
bin001	SOY3_bin001_02931	363	7	2	4	2.305	0.559	1.171	hypothetical protein
bin001	SOY3_bin001_02932	198	0	2	1	0.000	1.025	0.536	hypothetical protein
bin001	SOY3_bin001_02933	753	192	245	392	30.483	33.001	55.299	hypothetical protein
bin001	SOY3_bin001_02934	1080	118	140	217	13.062	13.148	21.344	hypothetical protein
bin001	SOY3_bin001_02935	153	107	134	99	83.606	88.832	68.734	D-glycero-alpha-D-manno-heptose 1-phosphate guanylyltransferase
bin001	SOY3_bin001_02936	888	540	541	506	72.699	61.793	60.530	GDP-6-deoxy-D-mannose reductase
bin001	SOY3_bin001_02937	399	467	397	395	139.923	100.919	105.161	undecaprenyldiphospho-muramoylpentapeptide beta-N- acetylglucosaminyltransferase
bin001	SOY3_bin001_02938	465	489	373	411	125.719	81.360	93.890	hypothetical protein
bin001	SOY3_bin001_02939	243	9	3	4	4.428	1.252	1.749	hypothetical protein
bin001	SOY3_bin001_02940	993	270	226	354	32.506	23.084	37.869	hypothetical protein
bin001	SOY3_bin001_02941	234	61	52	54	31.164	22.539	24.514	YcfA-like protein
bin001	SOY3_bin001_02942	204	59	65	47	34.575	32.318	24.474	Antitoxin HicB
bin001	SOY3_bin001_02943	144	17	26	26	14.113	18.313	19.180	hypothetical protein
bin001	SOY3_bin001_02944	696	248	216	314	42.598	31.477	47.924	putative DNA-binding protein
bin001	SOY3_bin001_02945	783	90	71	106	13.741	9.197	14.380	hypothetical protein

bin001	SOY3_bin001_02946	618	6	4	5	1.161	0.656	0.859	AAA-like domain protein
bin001	SOY3_bin001_02947	1260	24	18	24	2.277	1.449	2.023	hypothetical protein
bin001	SOY3_bin001_02948	216	76	65	57	42.063	30.522	28.032	hypothetical protein
bin001	SOY3_bin001_02949	105	32	40	29	36.434	38.639	29.339	YcfA-like protein
bin001	SOY3_bin001_02950	696	81	62	94	13.913	9.035	14.347	hypothetical protein
bin001	SOY3_bin001_02951	225	166	255	241	88.201	114.951	113.780	hypothetical protein
bin001	SOY3_bin001_02952	606	11	13	6	2.170	2.176	1.052	Mrr restriction system protein
bin001	SOY3_bin001_02953	813	127	107	137	18.675	13.349	17.900	hypothetical protein
bin001	SOY3_bin001_02954	432	18	19	27	4.981	4.461	6.639	50S ribosomal protein L22/unknown domain fusion protein
bin001	SOY3_bin001_02955	894	7	9	9	0.936	1.021	1.069	Modification methylase DpnIIA
bin001	SOY3_bin001_02956	465	122	101	150	31.366	22.030	34.266	hypothetical protein
bin001	SOY3_bin001_02957	174	30	31	21	20.612	18.070	12.820	hypothetical protein
bin001	SOY3_bin001_02958	537	865	712	759	192.569	134.481	150.140	hypothetical protein
bin001	SOY3_bin001_02959	375	4	5	3	1.275	1.352	0.850	Beta-(1-->2)glucan export ATP-binding/permease protein NdvA
bin001	SOY3_bin001_02960	1263	121	196	150	11.453	15.740	12.616	Ribonuclease J 2
bin001	SOY3_bin001_02961	975	72	79	90	8.828	8.218	9.805	hypothetical protein
bin001	SOY3_bin001_02962	918	245	233	237	31.906	25.744	27.424	D-inositol-3-phosphate glycosyltransferase
bin001	SOY3_bin001_02963	228	118	183	181	61.872	81.409	84.328	hypothetical protein
bin001	SOY3_bin001_02964	519	96	114	117	22.113	22.279	23.947	hypothetical protein
bin001	SOY3_bin001_02965	285	192	208	142	80.538	74.024	52.927	tRNA(fMet)-specific endonuclease VapC
bin001	SOY3_bin001_02966	222	8	13	9	4.308	5.939	4.306	CRISPR-associated endoribonuclease Cas2
bin001	SOY3_bin001_02967	495	56	36	44	13.525	7.377	9.442	hypothetical protein
bin001	SOY3_bin001_02968	264	280	489	475	126.794	187.871	191.126	Molydopterin dinucleotide binding domain protein
bin001	SOY3_bin001_02969	504	0	0	0	0.000	0.000	0.000	hypothetical protein
bin001	SOY3_bin001_02970	318	0	0	0	0.000	0.000	0.000	hypothetical protein
bin001	SOY3_bin001_02971	186	1	0	0	0.643	0.000	0.000	hypothetical protein
bin001	SOY3_bin001_02972	222	0	0	0	0.000	0.000	0.000	hypothetical protein
bin001	SOY3_bin001_02973	969	0	0	0	0.000	0.000	0.000	hypothetical protein
bin001	SOY3_bin001_02974	480	39	32	40	9.713	6.762	8.852	hypothetical protein
bin001	SOY3_bin001_02975	1119	13	19	18	1.389	1.722	1.709	hypothetical protein
bin001	SOY3_bin001_02976	972	620	684	680	76.255	71.375	74.314	Cyclic pyranopterin monophosphate synthase 1
bin001	SOY3_bin001_02977	150	80	110	87	63.759	74.380	61.611	hypothetical protein
bin001	SOY3_bin001_02978	258	298	263	321	138.084	103.393	132.165	hypothetical protein
bin001	SOY3_bin001_02979	258	211	208	246	97.771	81.771	101.285	hypothetical protein
bin001	SOY3_bin001_02980	492	401	412	404	97.437	84.935	87.226	tRNA(fMet)-specific endonuclease VapC
bin001	SOY3_bin001_02981	219	400	430	428	218.354	199.149	207.601	hypothetical protein
bin001	SOY3_bin001_02982	204	66	197	105	38.678	97.947	54.675	hypothetical protein
bin001	SOY3_bin001_02983	510	44	92	57	10.314	18.297	11.872	PIN domain protein
bin001	SOY3_bin001_02984	255	127	117	127	59.540	46.537	52.905	hypothetical protein
bin001	SOY3_bin001_02985	228	110	109	136	57.677	48.489	63.363	YcfA-like protein
bin001	SOY3_bin001_02986	258	188	209	236	87.113	82.164	97.168	hypothetical protein
bin001	SOY3_bin001_02987	2808	444	743	396	18.903	26.838	14.981	hypothetical protein
bin001	SOY3_bin001_02988	243	42	78	44	20.663	32.557	19.234	SigmaK-factor processing regulatory protein BofA
bin001	SOY3_bin001_02989	1230	1609	2498	1284	156.385	205.988	110.889	cell division control protein 6
bin001	SOY3_bin001_02990	573	8	7	4	1.669	1.239	0.742	hypothetical protein
bin001	SOY3_bin001_02991	708	16	12	25	2.702	1.719	3.751	Chromosomal replication initiator protein DnaA
bin001	SOY3_bin001_02992	408	3	2	2	0.879	0.497	0.521	hypothetical protein
bin001	SOY3_bin001_02993	1470	1	1	1	0.081	0.069	0.072	Anaerobic sulfatase-maturating enzyme
bin001	SOY3_bin001_02994	996	1	1	1	0.120	0.102	0.107	putative ABC transporter ATP-binding protein
bin001	SOY3_bin001_02995	273	1144	1316	1723	500.967	488.931	670.428	hypothetical protein
bin001	SOY3_bin001_02996	240	1117	1498	2142	556.400	633.075	948.065	hypothetical protein
bin001	SOY3_bin001_02997	627	2221	2993	3979	423.474	484.166	674.118	putative ABC transporter permease protein
bin001	SOY3_bin001_02998	330	1	1	0	0.362	0.307	0.000	ATP-dependent Clp protease adapter protein ClpS
bin001	SOY3_bin001_02999	555	2	0	2	0.431	0.000	0.383	hypothetical protein
bin001	SOY3_bin001_03000	186	24	58	22	15.426	31.628	12.564	hypothetical protein
bin001	SOY3_bin001_03001	282	51	123	48	21.621	44.240	18.081	hypothetical protein
bin001	SOY3_bin001_03002	1857	97	148	95	6.245	8.084	5.434	Sodium-dependent dicarboxylate transporter SdcS
bin001	SOY3_bin001_03003	1062	192	424	220	21.613	40.495	22.005	hypothetical protein
bin001	SOY3_bin001_03004	225	31	17	28	16.471	7.663	13.219	hypothetical protein
bin001	SOY3_bin001_03005	867	56	67	29	7.722	7.838	3.553	TPR repeat-containing protein YrrB
bin001	SOY3_bin001_03006	1317	98	140	83	8.896	10.782	6.695	TPR repeat-containing protein YrrB
bin001	SOY3_bin001_03007	1020	86	95	55	10.080	9.447	5.728	MarR family protein
bin001	SOY3_bin001_03008	279	6	7	1	2.571	2.545	0.381	hypothetical protein
bin001	SOY3_bin001_03009	1704	1	1	1	0.070	0.060	0.062	N-substituted formamide deformylase precursor
bin001	SOY3_bin001_03010	903	950	908	790	125.771	101.989	92.933	2,6-dihydropseudooxynicotine hydrolase
bin001	SOY3_bin001_03011	1335	83	159	91	7.433	12.080	7.241	von Willebrand factor type A domain protein
bin001	SOY3_bin001_03012	1176	84	92	52	8.539	7.935	4.697	TelA-like protein

bin001	SOY3_bin001_03013	480	701	572	530	174.591	120.868	117.291	Ribosomal RNA large subunit methyltransferase H
bin001	SOY3_bin001_03014	657	1224	959	835	222.721	148.050	135.005	hypothetical protein
bin001	SOY3_bin001_03015	1095	65	68	35	7.097	6.299	3.395	putative permease
bin001	SOY3_bin001_03016	345	8	21	7	2.772	6.174	2.155	hypothetical protein
bin001	SOY3_bin001_03017	768	291	356	302	45.298	47.016	41.771	Ubiquinone biosynthesis O-methyltransferase
bin001	SOY3_bin001_03018	408	91	106	56	26.664	26.351	14.580	hypothetical protein
bin001	SOY3_bin001_03019	1215	751	1314	855	73.894	109.692	74.751	FeS cluster assembly protein SufB
bin001	SOY3_bin001_03020	735	649	1189	745	105.561	164.078	107.671	putative ABC transporter ATP-binding protein
bin001	SOY3_bin001_03021	189	91	178	82	57.561	95.524	46.087	hypothetical protein
bin001	SOY3_bin001_03022	330	64	107	44	23.185	32.887	14.163	mRNA interferase MazF6
bin001	SOY3_bin001_03023	729	283	263	141	46.409	36.592	20.546	tRNA pseudouridine synthase D
bin001	SOY3_bin001_03024	828	3088	4939	2347	445.854	605.011	301.101	tetrahydromethanopterin S-methyltransferase subunit C
bin001	SOY3_bin001_03025	690	15294	16683	16309	2649.827	2452.335	2510.777	tetrahydromethanopterin S-methyltransferase subunit D
bin001	SOY3_bin001_03026	891	4716	7980	5181	632.764	908.406	617.683	tetrahydromethanopterin S-methyltransferase subunit E
bin001	SOY3_bin001_03027	2418	16741	33383	15741	827.696	1400.309	691.522	Photosystem I iron-sulfur center
bin001	SOY3_bin001_03028	603	61	86	49	12.094	14.466	8.632	Aerobic cobaltochelatase subunit CobN
bin001	SOY3_bin001_03029	1095	119	223	113	12.992	20.656	10.962	hypothetical protein
bin001	SOY3_bin001_03030	174	57	112	60	39.163	65.287	36.630	hypothetical protein
bin001	SOY3_bin001_03031	375	15	19	13	4.782	5.139	3.682	hypothetical protein
bin001	SOY3_bin001_03032	798	50	113	57	7.491	14.363	7.588	Corrinoid/iron-sulfur protein large subunit
bin001	SOY3_bin001_03033	363	30	66	27	9.880	18.441	7.901	sulfur transfer complex subunit TusD
bin001	SOY3_bin001_03034	342	17	37	17	5.942	10.973	5.280	Intracellular sulfur oxidation protein DsrF
bin001	SOY3_bin001_03035	297	18	15	5	7.245	5.123	1.788	Protein TusB
bin001	SOY3_bin001_03036	246	127	253	103	61.718	104.313	44.477	Antitoxin HicB
bin001	SOY3_bin001_03037	270	149	243	124	65.973	91.285	48.785	hypothetical protein
bin001	SOY3_bin001_03038	471	179	317	123	45.434	68.264	27.740	tRNA(fMet)-specific endonuclease VapC
bin001	SOY3_bin001_03039	333	358	644	249	128.524	196.154	79.430	UDP-2,3-diacetamido-2,3-dideoxy-D-glucuronate 2-epimerase
bin001	SOY3_bin001_03040	1146	2026	3611	1848	211.349	319.593	171.296	Soluble hydrogenase 42 kDa subunit
bin001	SOY3_bin001_03041	1542	23	32	18	1.783	2.105	1.240	Transposase DDE domain protein
bin001	SOY3_bin001_03042	1482	967	1864	934	78.005	127.571	66.947	Oligosaccharyl transferase STT3 subunit
bin001	SOY3_bin001_03043	129	110	123	80	101.941	96.710	65.876	hypothetical protein
bin001	SOY3_bin001_03044	1125	493	1254	636	52.389	113.058	60.053	ATP-NAD kinase
bin001	SOY3_bin001_03045	306	176	636	295	68.760	210.810	102.407	Tryptophan synthase beta chain
bin001	SOY3_bin001_03046	228	15	37	7	7.865	16.460	3.261	Acylphosphatase
bin001	SOY3_bin001_03047	906	75	87	80	9.896	9.740	9.380	EamA-like transporter family protein
bin001	SOY3_bin001_03048	180	63	60	56	41.842	33.809	33.048	2-dehydropantoate 2-reductase
bin001	SOY3_bin001_03049	315	142	141	100	53.892	45.401	33.722	hypothetical protein
bin001	SOY3_bin001_03050	1311	641	1227	587	58.452	94.929	47.563	Vitamin B12-binding protein
bin001	SOY3_bin001_03051	735	95	175	69	15.452	24.149	9.972	Alkyl hydroperoxide reductase subunit C
bin001	SOY3_bin001_03052	1284	1261	1091	1265	117.407	86.182	104.654	Phosphomethylpyrimidine synthase
bin001	SOY3_bin001_03053	297	181	378	342	72.856	129.089	122.321	hypothetical protein
bin001	SOY3_bin001_03054	663	291	435	271	52.472	66.547	43.420	cell division control protein 6
bin001	SOY3_bin001_03055	507	48	128	81	11.318	25.607	16.971	hypothetical protein
bin001	SOY3_bin001_03056	294	32	27	17	13.012	9.315	6.142	hypothetical protein
bin001	SOY3_bin001_03057	762	768	850	898	120.490	113.141	125.185	Cyclic pyranopterin monophosphate synthase
bin001	SOY3_bin001_03058	363	63	147	55	20.748	41.074	16.095	hypothetical protein
bin001	SOY3_bin001_03059	366	249	354	243	81.332	98.102	70.527	hypothetical protein
bin001	SOY3_bin001_03060	216	195	217	193	107.926	101.897	94.915	hypothetical protein
bin001	SOY3_bin001_03061	375	116	89	31	36.980	24.072	8.781	hypothetical protein
bin001	SOY3_bin001_03062	411	147	211	99	42.758	52.071	25.587	TPR repeat-containing protein YrrB
bin001	SOY3_bin001_03063	732	4672	6269	4271	763.022	868.644	619.795	tetrahydromethanopterin S-methyltransferase subunit A
bin001	SOY3_bin001_03064	228	5833	6091	6256	3058.459	2709.621	2914.686	Tetrahydromethanopterin S-methyltransferase, F subunit (MtrF)
bin001	SOY3_bin001_03065	708	141	291	190	23.808	41.688	28.507	Lon protease 1
bin001	SOY3_bin001_03066	312	113	164	97	43.298	53.314	33.025	TM2 domain protein
bin001	SOY3_bin001_03067	1101	681	612	532	73.944	56.379	51.328	hypothetical protein
bin001	SOY3_bin001_03068	111	41	31	41	44.158	28.327	39.237	ADP-specific phosphofructokinase
bin001	SOY3_bin001_03069	798	754	836	725	112.957	106.257	96.508	Cadmium, cobalt and zinc/H(+)-K(+) antiporter
bin001	SOY3_bin001_03070	489	70	71	86	17.113	14.727	18.682	putative NADH oxidase
bin001	SOY3_bin001_03071	369	346	303	327	112.097	83.286	94.135	hypothetical protein
bin001	SOY3_bin001_03072	627	620	632	842	118.214	102.236	142.651	hypothetical protein
bin001	SOY3_bin001_03073	1026	136	301	151	15.847	29.756	15.634	hypothetical protein
bin001	SOY3_bin001_03074	91	94	135	91	123.490	150.469	106.226	tRNA-Arg(gcg)
bin001	SOY3_bin001_03075	141	13	18	20	11.022	12.948	15.067	hypothetical protein
bin001	SOY3_bin001_03076	408	61	234	107	17.874	58.172	27.858	Iron-sulfur flavoprotein
bin001	SOY3_bin001_03077	1002	981	1324	865	117.043	134.022	91.702	Arginine transport ATP-binding protein ArtM
bin001	SOY3_bin001_03078	240	59	117	52	29.389	49.446	23.016	Glutathione transport system permease protein GsiC
bin001	SOY3_bin001_03079	216	87	139	105	48.152	65.270	51.638	hypothetical protein

bin001	SOY3_bin001_03080	297	65	131	92	26.164	44.737	32.905	hypothetical protein
bin001	SOY3_bin001_03081	942	15	23	12	1.904	2.476	1.353	Transposase DDE domain protein
bin001	SOY3_bin001_03082	390	351	457	395	107.594	118.852	107.588	hypothetical protein
bin001	SOY3_bin001_03083	537	350	509	363	77.918	96.139	71.806	Acetyl-coenzyme A synthetase
bin001	SOY3_bin001_03084	294	1942	2005	2690	789.673	691.706	971.930	hypothetical protein
bin001	SOY3_bin001_03085	195	1263	1193	1648	774.309	620.527	897.744	hypothetical protein
bin001	SOY3_bin001_03086	126	856	816	1103	812.173	656.862	929.897	hypothetical protein
bin001	SOY3_bin001_03087	441	26	46	21	7.048	10.580	5.058	hypothetical protein
bin001	SOY3_bin001_03088	747	1206	1664	1445	193.007	225.937	205.484	Ornithine carbamoyltransferase
bin001	SOY3_bin001_03089	966	56	91	40	6.930	9.555	4.399	Dictyostelium (slime mold) repeat protein
bin001	SOY3_bin001_03090	219	205	234	234	111.906	108.374	113.501	hypothetical protein
bin001	SOY3_bin001_03091	324	111	142	124	40.957	44.453	40.654	Diaminopimelate decarboxylase
bin001	SOY3_bin001_03092	642	157	247	170	29.235	39.023	28.128	hypothetical protein
bin001	SOY3_bin001_03093	114	14	16	13	14.681	14.235	12.113	hypothetical protein
bin001	SOY3_bin001_03094	444	38	102	67	10.232	23.301	16.030	hypothetical protein
bin001	SOY3_bin001_03095	411	117	83	104	34.032	20.483	26.880	tRNA(fMet)-specific endonuclease VapC
bin001	SOY3_bin001_03096	3297	10	6	12	0.363	0.185	0.387	hypothetical protein
bin001	SOY3_bin001_03097	414	0	1	2	0.000	0.245	0.513	hypothetical protein
bin001	SOY3_bin001_03098	972	4	3	4	0.492	0.313	0.437	Tyrosine recombinase XerD
bin001	SOY3_bin001_03099	504	4	2	0	0.949	0.402	0.000	hypothetical protein
bin001	SOY3_bin001_03100	333	53	80	75	19.027	24.367	23.925	Plasmid stabilisation system protein
bin001	SOY3_bin001_03101	1068	33	68	26	3.694	6.458	2.586	Putative ABC transporter substrate-binding lipoprotein YhfQ precursor
bin001	SOY3_bin001_03102	1014	76	108	55	8.960	10.803	5.762	Demethylspheroidene O-methyltransferase
bin001	SOY3_bin001_03103	498	14	11	2	3.361	2.240	0.427	Transcriptional regulator YqjI
bin001	SOY3_bin001_03104	603	8	9	1	1.586	1.514	0.176	Demethylmenaquinone methyltransferase
bin001	SOY3_bin001_03105	1392	4	12	4	0.344	0.874	0.305	Multidrug export protein MepA
bin001	SOY3_bin001_03106	1611	40	49	36	2.968	3.085	2.374	Heme-binding protein A precursor
bin001	SOY3_bin001_03107	522	64	118	43	14.657	22.928	8.750	Transcriptional regulator YqjI
bin001	SOY3_bin001_03108	783	116	189	81	17.711	24.482	10.989	FmdE, Molybdenum formylmethanofuran dehydrogenase operon
bin001	SOY3_bin001_03109	1182	114	188	78	11.530	16.132	7.010	putative tRNA (adenine(37)-N6)-methyltransferase
bin001	SOY3_bin001_03110	1599	261	328	305	19.514	20.806	20.262	Heme-binding protein A precursor
bin001	SOY3_bin001_03111	1074	181	386	176	20.147	36.453	17.408	putative ABC transporter permease protein
bin001	SOY3_bin001_03112	837	146	273	99	20.853	33.082	12.564	putative ABC transporter ATP-binding protein
bin001	SOY3_bin001_03113	1332	120	243	105	10.770	18.504	8.374	Fe(3+)-citrate-binding protein YfmC precursor
bin001	SOY3_bin001_03114	1182	139	224	97	14.059	19.221	8.717	Vitamin B12-binding protein precursor
bin001	SOY3_bin001_03115	840	95	199	84	13.520	24.029	10.623	putative methyltransferase YcgJ
bin001	SOY3_bin001_03116	1182	112	246	92	11.328	21.109	8.268	High-affinity heme uptake system protein IsdE precursor
bin001	SOY3_bin001_03117	360	29	86	34	9.630	24.230	10.032	hypothetical protein
bin001	SOY3_bin001_03118	450	4	10	2	1.063	2.254	0.472	Fe(3+)-citrate-binding protein YfmC precursor
bin001	SOY3_bin001_03119	3243	211	387	250	7.778	12.104	8.189	Type I restriction enzyme EcoR124II R protein
bin001	SOY3_bin001_03120	2103	49	100	50	2.785	4.823	2.526	hypothetical protein
bin001	SOY3_bin001_03121	114	5	9	3	5.243	8.007	2.795	hypothetical protein
bin001	SOY3_bin001_03122	1743	26	67	19	1.783	3.899	1.158	AAA-like domain protein
bin001	SOY3_bin001_03123	1326	9	45	10	0.811	3.442	0.801	hypothetical protein
bin001	SOY3_bin001_03124	1239	55	98	50	5.307	8.022	4.287	Putative type I restriction enzyme specificity protein MPN_638
bin001	SOY3_bin001_03125	945	314	476	228	39.723	51.089	25.629	lipoprotein NlpI
bin001	SOY3_bin001_03126	222	0	0	1	0.000	0.000	0.478	hypothetical protein
bin001	SOY3_bin001_03127	117	6	13	1	6.131	11.270	0.908	hypothetical protein
bin001	SOY3_bin001_03128	519	6	25	11	1.382	4.886	2.251	hypothetical protein
bin001	SOY3_bin001_03129	369	10	7	5	3.240	1.924	1.439	DNA-binding transcriptional repressor ArsR
bin001	SOY3_bin001_03130	1845	21	32	15	1.361	1.759	0.864	Lipid A export ATP-binding/permease protein MsbA
bin001	SOY3_bin001_03131	1488	10	24	7	0.803	1.636	0.500	hypothetical protein
bin001	SOY3_bin001_03132	1140	7	26	9	0.734	2.313	0.839	pyrroloquinoline quinone biosynthesis protein PqqE
bin001	SOY3_bin001_03133	375	21	30	26	6.695	8.114	7.365	hypothetical protein
bin001	SOY3_bin001_03134	171	3	4	3	2.097	2.373	1.864	hypothetical protein
bin001	SOY3_bin001_03135	774	31	75	54	4.788	9.828	7.411	hypothetical protein
bin001	SOY3_bin001_03136	2007	108	181	125	6.433	9.147	6.616	PglZ domain protein
bin001	SOY3_bin001_03137	1086	43	52	36	4.734	4.857	3.521	hypothetical protein
bin001	SOY3_bin001_03138	2748	156	220	164	6.787	8.120	6.340	putative methyltransferase
bin001	SOY3_bin001_03139	135	7	16	10	6.199	12.021	7.869	hypothetical protein
bin001	SOY3_bin001_03140	1824	136	229	169	8.914	12.734	9.842	Divergent AAA domain protein
bin001	SOY3_bin001_03141	630	38	78	46	7.211	12.558	7.756	hypothetical protein
bin001	SOY3_bin001_03142	1101	17	30	22	1.846	2.764	2.123	Chromosome partition protein Smc
bin001	SOY3_bin001_03143	3930	38	83	35	1.156	2.142	0.946	ATP-dependent DNA helicase RecQ
bin001	SOY3_bin001_03144	1479	490	708	524	39.607	48.553	37.635	TPR repeat-containing protein YrrB
bin001	SOY3_bin001_03145	1446	264	230	233	21.826	16.133	17.117	TPR repeat-containing protein YrrB
bin001	SOY3_bin001_03146	276	1	1	0	0.433	0.367	0.000	Cation transport regulator ChaB

bin001	SOY3_bin001_03147	264	0	0	0	0.000	0.000	0.000	hypothetical protein
bin001	SOY3_bin001_03148	1125	29	25	17	3.082	2.254	1.605	putative metallophosphoesterase YhaO
bin001	SOY3_bin001_03149	2106	51	29	25	2.895	1.397	1.261	thiamine transporter ATP-binding subunit
bin001	SOY3_bin001_03150	165	0	1	0	0.000	0.615	0.000	hypothetical protein
bin001	SOY3_bin001_03151	216	8	11	6	4.428	5.165	2.951	hypothetical protein
bin001	SOY3_bin001_03152	1179	610	1036	765	61.853	89.125	68.925	2-aminoadipate transaminase
bin001	SOY3_bin001_03153	1146	9	4	10	0.939	0.354	0.927	hypothetical protein
bin001	SOY3_bin001_03154	435	4	3	2	1.099	0.699	0.488	50S ribosomal protein L22/unknown domain fusion protein
bin001	SOY3_bin001_03155	1323	19	9	16	1.717	0.690	1.285	Type-1 restriction enzyme EcoKI specificity protein
bin001	SOY3_bin001_03156	2892	20	17	50	0.827	0.596	1.837	Type-1 restriction enzyme R protein
bin001	SOY3_bin001_03157	267	7	1	4	3.134	0.380	1.591	Protein SprT
bin001	SOY3_bin001_03158	618	666	762	758	128.834	125.061	130.290	IS2 transposase TnpB
bin001	SOY3_bin001_03159	189	68	160	86	43.012	85.864	48.336	hypothetical protein
bin001	SOY3_bin001_03160	1272	275	664	278	25.846	52.946	23.216	UDP-glucose 6-dehydrogenase TuaD
bin001	SOY3_bin001_03161	741	84	181	86	13.552	24.775	12.328	UTP--glucose-1-phosphate uridylyltransferase
bin001	SOY3_bin001_03162	366	132	213	139	43.116	59.027	40.343	Capsular glucan synthase
bin001	SOY3_bin001_03163	783	397	675	384	60.614	87.437	52.095	Teichoic acid translocation permease protein TagG
bin001	SOY3_bin001_03164	726	438	686	460	72.125	95.839	67.306	Teichoic acids export ATP-binding protein TagH
bin001	SOY3_bin001_03165	693	38	52	22	6.555	7.611	3.372	Ubiquinone biosynthesis O-methyltransferase
bin001	SOY3_bin001_03166	975	29	53	26	3.556	5.513	2.833	hypothetical protein
bin001	SOY3_bin001_03167	3039	220	275	108	8.654	9.178	3.775	Glycosyl hydrolase family 26
bin001	SOY3_bin001_03168	888	139	343	180	18.713	39.177	21.532	hypothetical protein
bin001	SOY3_bin001_03169	192	26	51	39	16.189	26.942	21.577	hypothetical protein
bin001	SOY3_bin001_03170	450	20	31	22	5.313	6.987	5.193	tRNA(fMet)-specific endonuclease VapC
bin001	SOY3_bin001_03171	810	154	314	175	22.729	39.319	22.950	hypothetical protein
bin001	SOY3_bin001_03172	3426	370	758	354	12.911	22.441	10.976	ski2-like helicase
bin001	SOY3_bin001_03173	168	3	13	0	2.135	7.849	0.000	hypothetical protein
bin001	SOY3_bin001_03174	1506	11	46	3	0.873	3.098	0.212	hypothetical protein
bin001	SOY3_bin001_03175	1614	192	406	186	14.221	25.514	12.242	Transposase DDE domain protein
bin001	SOY3_bin001_03176	255	115	118	125	53.914	46.935	52.071	hypothetical protein
bin001	SOY3_bin001_03177	144	22	55	31	18.264	38.740	22.868	hypothetical protein
bin001	SOY3_bin001_03178	390	16	28	12	4.905	7.282	3.268	Fic/DOC family protein
bin001	SOY3_bin001_03179	150	7	7	11	5.579	4.733	7.790	hypothetical protein
bin001	SOY3_bin001_03180	192	61	60	49	37.982	31.696	27.110	hypothetical protein
bin001	SOY3_bin001_03181	171	23	20	26	16.080	11.863	16.151	hypothetical protein
bin001	SOY3_bin001_03182	753	12	21	15	1.905	2.829	2.116	hypothetical protein
bin001	SOY3_bin001_03183	1137	91	131	64	9.568	11.686	5.979	hypothetical protein
bin001	SOY3_bin001_03184	168	7	25	6	4.981	15.093	3.794	hypothetical protein
bin001	SOY3_bin001_03185	159	39	69	25	29.323	44.016	16.702	hypothetical protein
bin001	SOY3_bin001_03186	1359	906	2077	963	79.699	155.014	75.273	inosine 5-monophosphate dehydrogenase
bin001	SOY3_bin001_03187	1479	18	2	3	1.455	0.137	0.215	GDSL-like Lipase/Acylhydrolase
bin001	SOY3_bin001_03188	882	26	3	2	3.524	0.345	0.241	hypothetical protein
bin001	SOY3_bin001_03189	1170	139	325	153	14.203	28.174	13.891	Bifunctional NAD(P)H-hydrate repair enzyme Nnr
bin001	SOY3_bin001_03190	486	45	120	42	11.069	25.044	9.180	Cyclic pyranopterin monophosphate synthase accessory protein 2
bin001	SOY3_bin001_03191	423	6	14	2	1.696	3.357	0.502	SOUL heme-binding protein
bin001	SOY3_bin001_03192	135	4	18	6	3.542	13.524	4.721	hypothetical protein
bin001	SOY3_bin001_03193	360	22	49	12	7.306	13.805	3.541	Fic/DOC family protein
bin001	SOY3_bin001_03194	372	20	35	20	6.427	9.543	5.711	hypothetical protein
bin001	SOY3_bin001_03195	315	11	30	18	4.175	9.660	6.070	hypothetical protein
bin001	SOY3_bin001_03196	213	20	60	32	11.225	28.571	15.959	hypothetical protein
bin001	SOY3_bin001_03197	1248	381	623	296	36.497	50.632	25.195	Transposase IS116/IS110/IS902 family protein
bin001	SOY3_bin001_03198	1566	401	320	368	30.612	20.726	24.962	Transposase DDE domain protein
bin001	SOY3_bin001_03199	195	66	73	95	40.463	37.970	51.751	hypothetical protein
bin001	SOY3_bin001_03200	996	129	110	96	15.484	11.202	10.239	hypothetical protein
bin001	SOY3_bin001_03201	1230	98	166	107	9.525	13.689	9.241	Divergent AAA domain protein
bin001	SOY3_bin001_03202	252	9	22	8	4.270	8.855	3.372	D-inositol-3-phosphate glycosyltransferase
bin001	SOY3_bin001_03203	510	37	45	26	8.673	8.949	5.415	tRNA(fMet)-specific endonuclease VapC
bin001	SOY3_bin001_03204	243	125	173	87	61.496	72.210	38.031	hypothetical protein
bin001	SOY3_bin001_03205	249	43	91	49	20.645	37.068	20.904	hypothetical protein
bin001	SOY3_bin001_03206	237	65	130	56	32.788	55.635	25.100	YcfA-like protein
bin001	SOY3_bin001_03207	255	149	253	124	69.854	100.632	51.655	Antitoxin HicB
bin001	SOY3_bin001_03208	351	138	236	94	47.002	68.196	28.448	tRNA(fMet)-specific endonuclease VapC
bin001	SOY3_bin001_03209	264	190	307	168	86.039	117.948	67.598	hypothetical protein
bin001	SOY3_bin001_03210	1263	358	702	302	33.886	56.375	25.400	TPR repeat-containing protein YrrB
bin001	SOY3_bin001_03211	624	1	1	1	0.192	0.163	0.170	hypothetical protein
bin001	SOY3_bin001_03212	159	0	0	0	0.000	0.000	0.000	hypothetical protein
bin001	SOY3_bin001_03213	165	0	0	0	0.000	0.000	0.000	hypothetical protein

bin001	SOY3_bin001_03214	180	22	40	24	14.612	22.539	14.163	hypothetical protein
bin001	SOY3_bin001_03215	117	24	24	26	24.523	20.806	23.606	hypothetical protein
bin001	SOY3_bin001_03216	573	131	225	118	27.331	39.827	21.875	Putative 5'(3')-deoxyribonucleotidase
bin001	SOY3_bin001_03217	1098	48	72	75	5.226	6.651	7.256	Putative transposase DNA-binding domain protein
bin001	SOY3_bin001_03218	360	18	41	29	5.977	11.551	8.557	hypothetical protein
bin001	SOY3_bin001_03219	183	10	23	21	6.533	12.748	12.190	hypothetical protein
bin001	SOY3_bin001_03220	186	20	41	17	12.855	22.358	9.709	hypothetical protein
bin001	SOY3_bin001_03221	477	61	110	62	15.288	23.390	13.807	ATPase family associated with various cellular activities (AAA)
bin001	SOY3_bin001_03222	303	4	7	2	1.578	2.343	0.701	hypothetical protein
bin001	SOY3_bin001_03223	1236	25	68	37	2.418	5.580	3.180	Inner membrane transport protein YdhC
bin001	SOY3_bin001_03224	264	5	6	2	2.264	2.305	0.805	hypothetical protein
bin001	SOY3_bin001_03225	756	92	185	105	14.548	24.820	14.754	HTH-type transcriptional regulator AcrR
bin001	SOY3_bin001_03226	456	19	33	13	4.981	7.340	3.028	hypothetical protein
bin001	SOY3_bin001_03227	216	70	77	56	38.743	36.157	27.540	hypothetical protein
bin001	SOY3_bin001_03228	2004	355	639	486	21.178	32.341	25.761	Endonuclease MutS2
bin001	SOY3_bin001_03229	885	99	246	139	13.373	28.193	16.684	hypothetical protein
bin001	SOY3_bin001_03230	1848	611	1503	646	39.526	82.492	37.133	hypothetical protein
bin001	SOY3_bin001_03231	1224	228	471	222	22.269	39.030	19.266	hypothetical protein
bin001	SOY3_bin001_03232	162	19	21	17	14.021	13.148	11.147	hypothetical protein
bin001	SOY3_bin001_03233	546	243	387	199	53.206	71.891	38.716	hypothetical protein
bin001	SOY3_bin001_03234	1431	82	147	61	6.850	10.419	4.528	Thiol-disulfide oxidoreductase ResA
bin001	SOY3_bin001_03235	261	45	105	32	20.612	40.804	13.024	Ferredoxin
bin001	SOY3_bin001_03236	474	40	70	26	10.089	14.979	5.827	Arsenate-mycothiol transferase ArsC1
bin001	SOY3_bin001_03237	1569	195	334	195	14.858	21.591	13.202	Regulatory protein AfsR
bin001	SOY3_bin001_03238	1254	112	254	134	10.677	20.544	11.351	cell division control protein 6
bin001	SOY3_bin001_03239	606	31	36	31	6.116	6.025	5.434	FMN-dependent NADH-azoreductase 1
bin001	SOY3_bin001_03240	1191	138	168	107	13.852	14.307	9.543	hypothetical protein
bin001	SOY3_bin001_03241	159	4	3	5	3.008	1.914	3.340	hypothetical protein
bin001	SOY3_bin001_03242	1959	235678	237978	264385	14382.359	12321.317	14336.139	hypothetical protein
bin001	SOY3_bin001_03243	315	312	307	337	118.410	98.851	113.645	HTH domain protein
bin001	SOY3_bin001_03244	120	27	16	19	26.898	13.524	16.819	hypothetical protein
bin001	SOY3_bin001_03245	546	129	83	90	28.245	15.418	17.510	hypothetical protein
bin001	SOY3_bin001_03246	1032	11	11	13	1.274	1.081	1.338	hypothetical protein
bin001	SOY3_bin001_03247	2142	724	787	620	40.408	37.266	30.747	Transglutaminase-like superfamily protein
bin001	SOY3_bin001_03248	501	17	18	8	4.057	3.644	1.696	D-inositol 3-phosphate glycosyltransferase
bin001	SOY3_bin001_03249	987	182	280	168	22.044	28.774	18.081	Alpha-galactosylglucosyldiacylglycerol synthase
bin001	SOY3_bin001_03250	993	107	141	100	12.882	14.402	10.697	UDP-Glc:alpha-D-GlcNAc-diphosphoundecaprenol beta-1,3-glucosyltransferase WfgD
bin001	SOY3_bin001_03251	762	251	411	196	39.379	54.707	27.323	Cyclic pyranopterin monophosphate synthase
bin001	SOY3_bin001_03252	234	158	189	186	80.721	81.922	84.436	Antitoxin HicB
bin001	SOY3_bin001_03253	1224	55	103	48	5.372	8.535	4.166	putative hydrolase
bin001	SOY3_bin001_03254	153	8	10	4	6.251	6.629	2.777	O-acetylserine/cysteine export protein
bin001	SOY3_bin001_03255	699	236	431	274	40.363	62.540	41.639	Serine hydroxymethyltransferase
bin001	SOY3_bin001_03256	864	196	366	205	27.120	42.966	25.204	Bifunctional protein FolD protein
bin001	SOY3_bin001_03257	597	337	534	422	67.484	90.724	75.088	hypothetical protein
bin001	SOY3_bin001_03258	789	27	50	35	4.091	6.428	4.712	Carbamate kinase 1
bin001	SOY3_bin001_03259	384	55	83	57	17.123	21.923	15.768	putative protease YdeA
bin001	SOY3_bin001_03260	150	38	50	32	30.286	33.809	22.661	hypothetical protein
bin001	SOY3_bin001_03261	480	45	84	42	11.208	17.750	9.295	hypothetical protein
bin001	SOY3_bin001_03262	591	30	58	35	6.068	9.954	6.291	Acetyltransferase YpeA
bin001	SOY3_bin001_03263	513	20	41	25	4.661	8.106	5.177	Putative phosphinothricin acetyltransferase YwnH
bin001	SOY3_bin001_03264	492	48	35	26	11.663	7.215	5.614	putative ABC transporter ATP-binding protein
bin001	SOY3_bin001_03265	954	178	290	124	22.306	30.832	13.807	hypothetical protein
bin001	SOY3_bin001_03266	1038	587	434	489	67.606	42.408	50.043	hypothetical protein
bin001	SOY3_bin001_03267	405	1643	1485	1810	484.985	371.900	474.737	hypothetical protein
bin001	SOY3_bin001_03268	396	2	0	1	0.604	0.000	0.268	hypothetical protein
bin001	SOY3_bin001_03269	1260	653	1055	650	61.957	84.925	54.799	putative transposase
bin001	SOY3_bin001_03270	207	190	191	229	109.731	93.587	117.515	hypothetical protein
bin001	SOY3_bin001_03271	789	2099	1972	2687	318.039	253.504	361.760	Hemin import ATP-binding protein HmuV
bin001	SOY3_bin001_03272	1437	22	33	18	1.830	2.329	1.331	Transposase DDE domain protein
bin001	SOY3_bin001_03273	726	475	864	544	78.217	120.707	79.596	hypothetical protein
bin001	SOY3_bin001_03274	708	492	664	628	83.076	95.124	94.223	hypothetical protein
bin001	SOY3_bin001_03275	87	51	55	36	70.080	64.121	43.955	tRNA-Ser(cag)
bin001	SOY3_bin001_03276	1254	36	36	31	3.432	2.912	2.626	sirohydrochlorin cobaltochelatase
bin001	SOY3_bin001_03277	528	57	50	51	12.906	9.605	10.260	hypothetical protein
bin001	SOY3_bin001_03278	381	116	210	101	36.398	55.905	28.160	putative spore protein YtfJ
bin001	SOY3_bin001_03279	990	125	217	103	15.095	22.232	11.052	2-dehydropantoate 2-reductase
bin001	SOY3_bin001_03280	354	23	61	41	7.767	17.478	12.303	hypothetical protein

bin001	SOY3_bin001_03281	672	208	260	221	37.003	39.243	34.934	putative D,D-dipeptide transport ATP-binding protein DdpF
bin001	SOY3_bin001_03282	252	221	230	267	104.842	92.573	112.549	hypothetical protein
bin001	SOY3_bin001_03283	489	294	231	280	71.876	47.913	60.825	TspO/MBR family protein
bin001	SOY3_bin001_03284	726	170	361	237	27.994	50.434	34.677	Periplasmic binding protein
bin001	SOY3_bin001_03285	270	55	121	61	24.353	45.454	23.999	hypothetical protein
bin001	SOY3_bin001_03286	702	201	432	262	34.230	62.417	39.646	tryptophanyl-tRNA synthetase
bin001	SOY3_bin001_03287	264	84	140	108	38.038	53.787	43.456	hypothetical protein
bin001	SOY3_bin001_03288	513	1	2	3	0.233	0.395	0.621	hypothetical protein
bin001	SOY3_bin001_03289	1053	48	39	25	5.450	3.757	2.522	Transposase IS66 family protein
bin001	SOY3_bin001_03290	204	28	43	45	16.409	21.379	23.432	DNA alkylation repair enzyme
bin001	SOY3_bin001_03291	1725	143	285	160	9.910	16.758	9.853	Ribonucleoside-diphosphate reductase NrdZ
bin001	SOY3_bin001_03292	642	68	121	57	12.662	19.116	9.431	putative S-adenosylmethionine-dependent methyltransferase/MSMEI_2290
bin001	SOY3_bin001_03293	195	27	83	37	16.553	43.172	20.156	hypothetical protein
bin001	SOY3_bin001_03294	306	102	151	95	39.850	50.051	32.979	hypothetical protein
bin001	SOY3_bin001_03295	135	96	153	152	85.013	114.951	119.602	hypothetical protein
bin001	SOY3_bin001_03296	516	456	801	662	105.648	157.448	136.282	Putative NAD(P)H nitroreductase YodC
bin001	SOY3_bin001_03297	126	0	1	1	0.000	0.805	0.843	hypothetical protein
bin001	SOY3_bin001_03298	753	920	1427	824	146.062	192.213	116.242	hypothetical protein
bin001	SOY3_bin001_03299	309	161	131	101	62.289	43.000	34.721	hypothetical protein
bin001	SOY3_bin001_03300	273	72	130	76	31.529	48.299	29.572	hypothetical protein
bin001	SOY3_bin001_03301	900	110	245	136	14.612	27.611	16.052	L(+)-tartrate dehydratase subunit alpha
bin001	SOY3_bin001_03302	537	75	145	89	16.697	27.387	17.605	L(+)-tartrate dehydratase subunit beta
bin001	SOY3_bin001_03303	276	101	204	121	43.748	74.968	46.570	tyrosyl-tRNA synthetase
bin001	SOY3_bin001_03304	1272	373	823	338	35.056	65.625	28.227	3-phosphoshikimate 1-carboxyvinyltransferase
bin001	SOY3_bin001_03305	600	981	1458	1315	195.462	246.468	232.811	hypothetical protein
bin001	SOY3_bin001_03306	387	197	311	240	60.856	81.509	65.876	Molydopterin dinucleotide binding domain protein
bin001	SOY3_bin001_03307	213	360	575	560	202.054	273.806	279.279	hypothetical protein
bin001	SOY3_bin001_03308	513	148	197	149	34.490	38.950	30.853	hypothetical protein
bin001	SOY3_bin001_03309	744	127	292	133	20.407	39.807	18.989	Ribonuclease Z
bin001	SOY3_bin001_03310	336	136	233	157	48.389	70.335	49.635	Quercetin 2,3-dioxygenase
bin001	SOY3_bin001_03311	525	152	287	142	34.612	55.447	28.732	hypothetical protein
bin001	SOY3_bin001_03312	453	198	335	149	52.253	75.007	34.940	hypothetical protein
bin001	SOY3_bin001_03313	252	7	8	6	3.321	3.220	2.529	Lipoprotein NlpI precursor
bin001	SOY3_bin001_03314	471	339	286	361	86.045	61.589	81.417	Phosphate transport system permease protein PstA
bin001	SOY3_bin001_03315	474	59	127	116	14.881	27.176	25.996	hypothetical protein
bin001	SOY3_bin001_03316	186	7	5	2	4.499	2.727	1.142	hypothetical protein
bin001	SOY3_bin001_03317	240	24	39	25	11.955	16.482	11.065	CobD/Cbib protein
bin001	SOY3_bin001_03318	585	168	456	224	34.332	79.061	40.674	hypothetical protein
bin001	SOY3_bin001_03319	165	4	17	15	2.898	10.450	9.657	hypothetical protein
bin001	SOY3_bin001_03320	771	48	69	31	7.443	9.077	4.271	regulatory protein UhpC
bin001	SOY3_bin001_03321	234	112	1398	752	57.220	605.963	341.375	hypothetical protein
bin001	SOY3_bin001_03322	270	19	24	19	8.413	9.016	7.475	Dihydroorotate dehydrogenase B (NAD(+)), electron transfer subunit
bin001	SOY3_bin001_03323	1221	47	77	26	4.602	6.396	2.262	Rhodopirellula transposase
bin001	SOY3_bin001_03324	462	329	418	193	85.133	91.768	44.376	tRNA(fMet)-specific endonuclease VapC
bin001	SOY3_bin001_03325	186	138	219	136	88.698	119.422	77.670	SpoVT / AbrB like domain protein
bin001	SOY3_bin001_03326	600	707	1192	532	140.868	201.502	94.187	Acylphosphatase
bin001	SOY3_bin001_03327	435	396	597	256	108.831	139.200	62.514	hypothetical protein
bin001	SOY3_bin001_03328	234	0	0	0	0.000	0.000	0.000	Transposase IS66 family protein
bin001	SOY3_bin001_03329	558	125	286	177	26.781	51.986	33.695	Sodium/pantothenate symporter
bin001	SOY3_bin001_03330	267	556	1314	710	248.948	499.159	282.473	Non-histone chromosomal protein MC1
bin001	SOY3_bin001_03331	252	243	369	275	115.279	148.519	115.921	Asparagine--tRNA ligase
bin001	SOY3_bin001_03332	285	32	71	29	13.423	25.268	10.809	hypothetical protein
bin001	SOY3_bin001_03333	273	14	29	18	6.131	10.774	7.004	von Willebrand factor type A domain protein
bin001	SOY3_bin001_03334	1128	57	110	64	6.041	9.891	6.027	TelA-like protein
bin001	SOY3_bin001_03335	276	45	68	41	19.492	24.989	15.780	hypothetical protein
bin001	SOY3_bin001_03336	570	167	288	147	35.026	51.247	27.395	Glutamyl-tRNA reductase
bin001	SOY3_bin001_03337	741	61	54	24	9.841	7.391	3.441	Inner membrane protein YrbG
bin001	SOY3_bin001_03338	666	53	94	52	9.514	14.316	8.294	K(+)/H(+) antiporter NhaP2
bin001	SOY3_bin001_03339	144	34	33	29	28.227	23.244	21.393	hypothetical protein
bin001	SOY3_bin001_03340	723	49	106	45	8.102	14.870	6.612	hypothetical protein
bin001	SOY3_bin001_03341	168	59	103	65	41.984	62.185	41.099	Tetratricopeptide repeat protein
bin001	SOY3_bin001_03342	291	85	97	97	34.920	33.809	35.409	MarR family protein
bin001	SOY3_bin001_03343	813	17	38	17	2.500	4.741	2.221	hypothetical protein
bin001	SOY3_bin001_03344	411	265	323	298	77.081	79.710	77.020	hypothetical protein
bin001	SOY3_bin001_03345	576	320	380	248	66.416	66.914	45.736	Rubrerythrin
bin001	SOY3_bin001_03346	474	85	81	49	21.438	17.333	10.981	Catalase
bin001	SOY3_bin001_03347	462	477	489	496	123.430	107.355	114.043	tRNA-specific adenosine deaminase

bin001	SOY3_bin001_03348	1167	1328	1569	1580	136.042	136.366	143.819	Lysine--tRNA ligase
bin001	SOY3_bin001_03349	498	147	185	80	35.289	37.679	17.064	Calcium-transporting ATPase
bin001	SOY3_bin001_03350	225	5	11	4	2.657	4.959	1.888	hypothetical protein
bin001	SOY3_bin001_03351	258	19	21	17	8.804	8.256	6.999	DNA polymerase I, thermostable
bin001	SOY3_bin001_03352	216	5	4	5	2.767	1.878	2.459	hypothetical protein
bin001	SOY3_bin001_03353	249	32	79	26	15.364	32.180	11.092	Beta-(1-->2)glucan export ATP-binding/permease protein NdvA
bin001	SOY3_bin001_03354	525	196	335	173	44.632	64.720	35.004	glucose-1-phosphate adenylyltransferase
bin001	SOY3_bin001_03355	237	347	723	324	175.036	309.417	145.220	hypothetical protein
bin001	SOY3_bin001_03356	681	8	6	8	1.404	0.894	1.248	hypothetical protein
bin001	SOY3_bin001_03357	159	2	7	7	1.504	4.465	4.677	hypothetical protein
bin001	SOY3_bin001_03358	684	157	266	169	27.440	39.444	26.246	Xaa-Pro dipeptidase
bin001	SOY3_bin001_03359	531	38	58	30	8.555	11.079	6.001	hypothetical protein
bin001	SOY3_bin001_03360	243	16	26	15	7.872	10.852	6.557	hypothetical protein
bin001	SOY3_bin001_03361	519	68	112	51	15.663	21.888	10.438	hypothetical protein
bin001	SOY3_bin001_03362	420	525	533	466	149.436	128.716	117.860	hypothetical protein
bin001	SOY3_bin001_03363	294	37	37	18	15.045	12.765	6.504	hypothetical protein
bin001	SOY3_bin001_03364	96	335	381	305	417.176	402.539	337.488	hypothetical protein
bin001	SOY3_bin001_03365	840	129	253	135	18.359	30.549	17.072	Ubiquinone biosynthesis O-methyltransferase
bin001	SOY3_bin001_03366	108	92	56	58	101.838	52.592	57.047	hypothetical protein
bin001	SOY3_bin001_03367	870	106	200	115	14.566	23.317	14.041	homoserine kinase
bin001	SOY3_bin001_03368	522	46	87	48	10.535	16.905	9.768	Secreted effector protein pipB2
bin001	SOY3_bin001_03369	348	17	26	13	5.840	7.578	3.968	hypothetical protein
bin001	SOY3_bin001_03370	870	213	375	250	29.269	43.719	30.525	3-deoxy-D-manno-octulosonic-acid kinase
bin001	SOY3_bin001_03371	567	315	487	505	66.416	87.117	94.610	hypothetical protein
bin001	SOY3_bin001_03372	267	2	2	2	0.895	0.760	0.796	Mrr restriction system protein
bin001	SOY3_bin001_03373	297	8	13	14	3.220	4.440	5.007	hypothetical protein
bin001	SOY3_bin001_03374	333	100	155	53	35.901	47.211	16.907	hypothetical protein
bin001	SOY3_bin001_03375	240	131	241	90	65.254	101.850	39.835	hypothetical protein
bin001	SOY3_bin001_03376	768	1775	2709	1567	276.301	357.769	216.739	Immunoglobulin-binding protein sbi precursor
bin001	SOY3_bin001_03377	762	320	444	295	50.204	59.099	41.124	hypothetical protein
bin001	SOY3_bin001_03378	369	157	261	237	50.865	71.741	68.226	hypothetical protein
bin001	SOY3_bin001_03379	354	12	34	17	4.053	9.742	5.101	HIT domain protein
bin001	SOY3_bin001_03380	144	22	32	21	18.264	22.539	15.491	hypothetical protein
bin001	SOY3_bin001_03381	315	69	71	69	26.187	22.861	23.268	putative FAD-linked oxidoreductase
bin001	SOY3_bin001_03382	237	465	1288	610	234.558	551.217	273.408	corrinoid ABC transporter substrate-binding protein
bin001	SOY3_bin001_03383	840	134	213	104	19.071	25.719	13.152	Succinate-semialdehyde dehydrogenase [NADP(+)] GabD
bin001	SOY3_bin001_03384	192	49	48	36	30.510	25.357	19.917	Ribosomal RNA small subunit methyltransferase A
bin001	SOY3_bin001_03385	537	97	145	155	21.594	27.387	30.661	L(+)-tartrate dehydratase subunit beta
bin001	SOY3_bin001_03386	396	23	67	53	6.943	17.161	14.217	L(+)-tartrate dehydratase subunit alpha
bin001	SOY3_bin001_03387	525	473	538	460	107.708	103.939	93.074	Adenylosuccinate lyase
bin001	SOY3_bin001_03388	795	120	178	111	18.045	22.710	14.832	Calcineurin-like phosphoesterase
bin001	SOY3_bin001_03389	534	228	234	237	51.043	44.446	47.145	N5-carboxyaminoimidazole ribonucleotide mutase
bin001	SOY3_bin001_03390	504	151	245	215	35.817	49.305	45.315	TPR repeat-containing protein YrrB
bin001	SOY3_bin001_03391	489	18	46	27	4.401	9.541	5.865	putative NADH oxidase
bin001	SOY3_bin001_03392	369	119	157	99	38.554	43.155	28.500	hypothetical protein
bin001	SOY3_bin001_03393	951	83	142	69	10.434	15.145	7.707	5-methylthioadenosine/S-adenosylhomocysteine deaminase
bin001	SOY3_bin001_03394	105	188	269	174	214.049	259.847	176.031	hypothetical protein
bin001	SOY3_bin001_03395	675	187	294	144	33.119	44.177	22.661	Uridylate kinase
bin001	SOY3_bin001_03396	312	697	881	794	267.069	286.402	270.331	hypothetical protein
bin001	SOY3_bin001_03397	333	102	144	103	36.619	43.860	32.857	hypothetical protein
bin001	SOY3_bin001_03398	639	318	743	418	59.494	117.935	69.487	hypothetical protein
bin003	SOY3_bin003_00001	1659	59	76	15	4.252	4.646	0.960	Long-chain-fatty-acid--CoA ligase
bin003	SOY3_bin003_00002	429	18	32	6	5.016	7.566	1.486	1,4-dihydroxy-2-naphthoyl-CoA hydrolase
bin003	SOY3_bin003_00003	576	16	21	2	3.321	3.698	0.369	(S)-2-hydroxypropylphosphonic acid epoxidase
bin003	SOY3_bin003_00004	276	7	18	1	3.032	6.615	0.385	hypothetical protein
bin003	SOY3_bin003_00005	1779	78	108	18	5.242	6.157	1.075	Glutamine--fructose-6-phosphate aminotransferase [isomerizing]
bin003	SOY3_bin003_00006	756	33	66	10	5.218	8.855	1.405	Phosphoribosylaminoimidazole-succinocarboxamide synthase
bin003	SOY3_bin003_00007	252	8	17	2	3.795	6.842	0.843	phosphoribosylformylglycinamidine synthase subunit PurS
bin003	SOY3_bin003_00008	651	24	41	8	4.407	6.388	1.305	Phosphoribosylformylglycinamidine synthase 1
bin003	SOY3_bin003_00009	708	16	25	6	2.702	3.581	0.900	Uroporphyrinogen-III C-methyltransferase
bin003	SOY3_bin003_00010	783	12	22	6	1.832	2.850	0.814	uroporphyrinogen-III synthase
bin003	SOY3_bin003_00011	273	5	7	0	2.190	2.601	0.000	signal recognition particle protein Srp19
bin003	SOY3_bin003_00012	1398	84	95	26	7.183	6.892	1.976	alanyl-tRNA synthetase
bin003	SOY3_bin003_00013	675	7	11	2	1.240	1.653	0.315	hypothetical protein
bin003	SOY3_bin003_00014	795	40	58	9	6.015	7.400	1.203	manganese transport regulator MntR
bin003	SOY3_bin003_00015	882	15	28	9	2.033	3.220	1.084	mevalonate kinase
bin003	SOY3_bin003_00016	663	16	43	10	2.885	6.578	1.602	DNA polymerase/3'-5' exonuclease PolX

bin003	SOY3_bin003_00017	159	6	8	2	4.511	5.103	1.336	hypothetical protein
bin003	SOY3_bin003_00018	681	4	12	1	0.702	1.787	0.156	2,5-diamino-6-ribosylamino-4(3H)-pyrimidinone 5'-phosphate reductase
bin003	SOY3_bin003_00019	378	2	6	2	0.633	1.610	0.562	Carboxymuconolactone decarboxylase family protein
bin003	SOY3_bin003_00020	1077	13	20	4	1.443	1.884	0.395	Capsule biosynthesis protein CapA
bin003	SOY3_bin003_00021	750	12	26	11	1.913	3.516	1.558	putative deoxyribonuclease YcfH
bin003	SOY3_bin003_00022	768	23	39	4	3.580	5.151	0.553	(2Z,6E)-farnesyl diphosphate synthase
bin003	SOY3_bin003_00023	696	8	10	1	1.374	1.457	0.153	phosphate butyryltransferase
bin003	SOY3_bin003_00024	579	12	24	7	2.478	4.204	1.284	Protein translocase subunit SecE
bin003	SOY3_bin003_00025	1254	12	29	6	1.144	2.346	0.508	Glutamate-1-semialdehyde 2,1-aminomutase
bin003	SOY3_bin003_00026	663	12	29	8	2.164	4.436	1.282	Precorrin-8X methylmutase
bin003	SOY3_bin003_00027	264	11	15	2	4.981	5.763	0.805	hypothetical protein
bin003	SOY3_bin003_00028	579	2	11	3	0.413	1.927	0.550	V4R domain protein
bin003	SOY3_bin003_00029	345	1	4	2	0.347	1.176	0.616	Roadblock/LC7 domain protein
bin003	SOY3_bin003_00030	444	15	25	9	4.039	5.711	2.153	RDD family protein
bin003	SOY3_bin003_00031	1011	40	56	25	4.730	5.618	2.627	Hydrogenase expression/formation protein HypE
bin003	SOY3_bin003_00032	381	32	51	13	10.041	13.577	3.625	30S ribosomal protein S8e
bin003	SOY3_bin003_00033	672	57	76	13	10.140	11.471	2.055	DNA polymerase II
bin003	SOY3_bin003_00034	681	17	35	14	2.984	5.213	2.184	Pyrimidine 5'-nucleotidase YjjG
bin003	SOY3_bin003_00035	1554	29	45	8	2.231	2.937	0.547	putative ATP-dependent protease
bin003	SOY3_bin003_00036	969	13	22	3	1.604	2.303	0.329	Sortase family protein
bin003	SOY3_bin003_00037	867	14	8	3	1.930	0.936	0.368	putative sugar kinase YdjH
bin003	SOY3_bin003_00038	687	19	31	2	3.306	4.577	0.309	Phosphoribosyl isomerase A
bin003	SOY3_bin003_00039	273	9	25	6	3.941	9.288	2.335	hypothetical protein
bin003	SOY3_bin003_00040	363	56	70	22	18.443	19.559	6.438	prefoldin subunit beta
bin003	SOY3_bin003_00041	240	22	40	12	10.959	16.905	5.311	Transcription factor Pcc1
bin003	SOY3_bin003_00042	573	54	107	29	11.266	18.940	5.376	ribosomal biogenesis protein
bin003	SOY3_bin003_00043	270	26	46	14	11.512	17.280	5.508	50S ribosomal protein L37Ae
bin003	SOY3_bin003_00044	807	80	141	36	11.851	17.721	4.739	Ribonuclease PH
bin003	SOY3_bin003_00045	729	56	101	23	9.183	14.052	3.351	Ribonuclease PH
bin003	SOY3_bin003_00046	765	54	95	22	8.439	12.596	3.055	exosome complex RNA-binding protein Rrp4
bin003	SOY3_bin003_00047	693	75	119	30	12.938	17.417	4.599	Shwachman-Bodian-Diamond syndrome (SBDS) protein
bin003	SOY3_bin003_00048	759	51	126	27	8.033	16.838	3.779	Proteasome subunit beta precursor
bin003	SOY3_bin003_00049	381	3	5	2	0.941	1.331	0.558	ribonuclease P protein component 2
bin003	SOY3_bin003_00050	729	21	25	7	3.444	3.478	1.020	ribonuclease P protein component 3
bin003	SOY3_bin003_00051	414	41	78	19	11.839	19.109	4.875	hypothetical protein
bin003	SOY3_bin003_00052	546	61	79	26	13.356	14.675	5.058	50S ribosomal protein L15e
bin003	SOY3_bin003_00053	1437	16	28	5	1.331	1.976	0.370	Multidrug export protein MepA
bin003	SOY3_bin003_00054	321	30	53	14	11.173	16.747	4.633	Carboxymuconolactone decarboxylase family protein
bin003	SOY3_bin003_00055	459	14	13	3	3.646	2.873	0.694	riboflavin synthase
bin003	SOY3_bin003_00056	963	25	71	23	3.104	7.478	2.537	Methenyltetrahydromethanopterin cyclohydrolase
bin003	SOY3_bin003_00057	663	9	14	5	1.623	2.142	0.801	Thymidylate synthase
bin003	SOY3_bin003_00058	978	13	25	4	1.589	2.593	0.434	5-methyltetrahydropteroyltriglutamate--homocysteine methyltransferase
bin003	SOY3_bin003_00059	309	11	24	6	4.256	7.878	2.063	hypothetical protein
bin003	SOY3_bin003_00060	462	17	36	17	4.399	7.903	3.909	hypothetical protein
bin003	SOY3_bin003_00061	1083	48	101	29	5.299	9.459	2.844	hypothetical protein
bin003	SOY3_bin003_00062	492	3	9	2	0.729	1.855	0.432	L-2,4-diaminobutyric acid acetyltransferase
bin003	SOY3_bin003_00063	74	1	0	0	1.616	0.000	0.000	tRNA-Ala(ggc)
bin003	SOY3_bin003_00064	1026	31	62	20	3.612	6.129	2.071	lipid-A-disaccharide synthase
bin003	SOY3_bin003_00065	660	11	32	3	1.992	4.918	0.483	Hydrogenase isoenzymes nickel incorporation protein HypB
bin003	SOY3_bin003_00066	372	3	16	4	0.964	4.362	1.142	Hydrogenase/urease nickel incorporation protein HypA
bin003	SOY3_bin003_00067	480	90	306	68	22.415	64.660	15.049	acid-resistance membrane protein
bin003	SOY3_bin003_00068	150	4	7	3	3.188	4.733	2.125	hypothetical protein
bin003	SOY3_bin003_00069	861	41	70	22	5.693	8.246	2.714	Ribose-phosphate pyrophosphokinase
bin003	SOY3_bin003_00070	369	13	44	11	4.212	12.094	3.167	hypothetical protein
bin003	SOY3_bin003_00071	363	18	37	9	5.928	10.338	2.634	hypothetical protein
bin003	SOY3_bin003_00072	321	5	7	1	1.862	2.212	0.331	hypothetical protein
bin003	SOY3_bin003_00073	1584	71	71	18	5.359	4.546	1.207	DEAD-box ATP-dependent RNA helicase CshA
bin003	SOY3_bin003_00074	1908	215	291	75	13.471	15.469	4.176	Lon protease 2
bin003	SOY3_bin003_00075	1512	24	35	9	1.898	2.348	0.632	Cobyric acid synthase
bin003	SOY3_bin003_00076	654	23	46	10	4.204	7.134	1.624	Trk system potassium uptake protein TrkA
bin003	SOY3_bin003_00077	447	12	21	5	3.209	4.765	1.188	Putative universal stress protein
bin003	SOY3_bin003_00078	1380	16	41	8	1.386	3.013	0.616	Sodium-dependent dicarboxylate transporter SdcS
bin003	SOY3_bin003_00079	432	7	14	1	1.937	3.287	0.246	Universal stress protein F
bin003	SOY3_bin003_00080	1002	10	19	8	1.193	1.923	0.848	Inner membrane protein YrbG
bin003	SOY3_bin003_00081	77	4	4	1	6.210	5.269	1.380	tRNA-Gly(gcc)
bin003	SOY3_bin003_00082	74	1	2	0	1.616	2.741	0.000	tRNA-Gly(tcc)
bin003	SOY3_bin003_00083	405	10	8	4	2.952	2.004	1.049	Magnesium transporter MgtE

bin003	SOY3_bin003_00084	420	27	58	19	7.685	14.007	4.805	Putative nickel-responsive regulator
bin003	SOY3_bin003_00085	774	4	13	4	0.618	1.704	0.549	Ribosomal protein L11 methyltransferase
bin003	SOY3_bin003_00086	549	8	24	3	1.742	4.434	0.580	Hydrogenase 3 maturation protease
bin003	SOY3_bin003_00087	702	14	42	9	2.384	6.068	1.362	hypothetical protein
bin003	SOY3_bin003_00088	1134	21	40	9	2.214	3.578	0.843	D-alanine--D-alanine ligase
bin003	SOY3_bin003_00089	1077	23	40	13	2.553	3.767	1.282	Phospho-N-acetylmuramoyl-pentapeptide-transferase
bin003	SOY3_bin003_00090	1419	37	84	21	3.117	6.004	1.572	UDP-N-acetylmuramoyl-L-alanyl-D-glutamate--L-lysine ligase
bin003	SOY3_bin003_00091	1434	33	47	12	2.751	3.324	0.889	UDP-N-acetylmuramoylalanine--D-glutamate ligase
bin003	SOY3_bin003_00092	1596	32	56	12	2.397	3.559	0.799	UDP-N-acetylmuramate--L-alanine ligase
bin003	SOY3_bin003_00093	423	22	37	10	6.218	8.872	2.511	hypothetical protein
bin003	SOY3_bin003_00094	1278	81	151	26	7.577	11.984	2.161	hypothetical protein
bin003	SOY3_bin003_00095	372	10	21	4	3.214	5.726	1.142	hypothetical protein
bin003	SOY3_bin003_00096	459	10	11	2	2.605	2.431	0.463	transcription factor
bin003	SOY3_bin003_00097	1233	107	177	52	10.374	14.560	4.480	ATP-dependent zinc metalloprotease FtsH 3
bin003	SOY3_bin003_00098	597	0	0	0	0.000	0.000	0.000	Cyclic nucleotide-gated potassium channel
bin003	SOY3_bin003_00099	168	0	0	0	0.000	0.000	0.000	hypothetical protein
bin003	SOY3_bin003_00100	360	44	88	19	14.612	24.793	5.606	O-acetyl-ADP-ribose deacetylase
bin003	SOY3_bin003_00101	279	7	12	1	2.999	4.362	0.381	hypothetical protein
bin003	SOY3_bin003_00102	1827	33	69	11	2.159	3.831	0.640	Threonine--tRNA ligase
bin003	SOY3_bin003_00103	1656	74	75	16	5.342	4.594	1.026	Dihydroxy-acid dehydratase
bin003	SOY3_bin003_00104	438	5	8	8	1.365	1.853	1.940	Signal peptidase I W
bin003	SOY3_bin003_00105	1842	52	56	16	3.375	3.084	0.923	Arginine--tRNA ligase
bin003	SOY3_bin003_00106	906	15	43	6	1.979	4.814	0.703	Ornithine carbamoyltransferase
bin003	SOY3_bin003_00107	1317	24	41	12	2.179	3.158	0.968	Phosphoribosylamine--glycine ligase
bin003	SOY3_bin003_00108	1746	64	152	37	4.382	8.830	2.251	Acetolactate synthase large subunit
bin003	SOY3_bin003_00109	498	20	39	11	4.801	7.943	2.346	Acetolactate synthase small subunit
bin003	SOY3_bin003_00110	981	55	119	35	6.703	12.304	3.790	Ketol-acid reductoisomerase
bin003	SOY3_bin003_00111	993	25	47	21	3.010	4.801	2.246	hypothetical protein
bin003	SOY3_bin003_00112	306	11	21	1	4.298	6.961	0.347	hypothetical protein
bin003	SOY3_bin003_00113	801	12	21	3	1.791	2.659	0.398	5'-nucleotidase SurE
bin003	SOY3_bin003_00114	780	72	134	32	11.035	17.425	4.358	Restriction endonuclease
bin003	SOY3_bin003_00115	75	7	0	2	11.158	0.000	2.833	tRNA-Val(cac)
bin003	SOY3_bin003_00116	72	1	0	3	1.660	0.000	4.426	tRNA-Val(tac)
bin003	SOY3_bin003_00117	924	49	96	18	6.340	10.538	2.069	Branched-chain-amino-acid aminotransferase
bin003	SOY3_bin003_00118	837	26	43	13	3.714	5.211	1.650	Undecaprenyl-diphosphatase
bin003	SOY3_bin003_00119	198	4	5	1	2.415	2.561	0.536	HTH-type transcriptional regulator SinR
bin003	SOY3_bin003_00120	426	4	6	0	1.123	1.429	0.000	hypothetical protein
bin003	SOY3_bin003_00121	1071	24	55	9	2.679	5.209	0.893	hypothetical protein
bin003	SOY3_bin003_00122	1635	43	52	20	3.144	3.226	1.299	tRNA N6-adenosine threonylcarbamoyltransferase
bin003	SOY3_bin003_00123	933	15	29	3	1.922	3.153	0.342	Lipase EstA precursor
bin003	SOY3_bin003_00124	576	275	395	155	57.076	69.555	28.585	Non-canonical purine NTP pyrophosphatase
bin003	SOY3_bin003_00125	402	20	55	12	5.948	13.877	3.171	30S ribosomal protein S15
bin003	SOY3_bin003_00126	1392	72	130	44	6.184	9.472	3.358	ssDNA exonuclease RecJ
bin003	SOY3_bin003_00127	1191	51	110	26	5.119	9.368	2.319	hypothetical protein
bin003	SOY3_bin003_00128	369	0	1	1	0.000	0.275	0.288	hypothetical protein
bin003	SOY3_bin003_00129	2328	41	48	14	2.105	2.091	0.639	ATP-dependent RNA helicase RhlE
bin003	SOY3_bin003_00130	729	8	12	4	1.312	1.670	0.583	tRNA (adenine(58)-N(1))-methyltransferase TrmI
bin003	SOY3_bin003_00131	918	11	25	5	1.433	2.762	0.579	Aspartate carbamoyltransferase catalytic chain
bin003	SOY3_bin003_00132	1149	42	38	16	4.370	3.354	1.479	cell division control protein 6
bin003	SOY3_bin003_00133	489	20	48	6	4.890	9.956	1.303	hypothetical protein
bin003	SOY3_bin003_00134	921	8	20	5	1.038	2.203	0.577	cobalamin biosynthesis protein
bin003	SOY3_bin003_00135	1044	21	17	5	2.405	1.652	0.509	cobalamin biosynthesis protein CbiG
bin003	SOY3_bin003_00136	261	9	16	1	4.122	6.218	0.407	hypothetical protein
bin003	SOY3_bin003_00137	534	5	8	1	1.119	1.520	0.199	L-fuculose phosphate aldolase
bin003	SOY3_bin003_00138	1710	35	67	11	2.447	3.974	0.683	DNA polymerase II small subunit
bin003	SOY3_bin003_00139	522	14	24	2	3.206	4.663	0.407	hypothetical protein
bin003	SOY3_bin003_00140	1065	34	47	6	3.817	4.476	0.598	Cobalt-precorrin-3B C(17)-methyltransferase
bin003	SOY3_bin003_00141	489	7	9	4	1.711	1.867	0.869	lipoprotein NlpI
bin003	SOY3_bin003_00142	372	7	3	1	2.250	0.818	0.286	hypothetical protein
bin003	SOY3_bin003_00143	873	16	20	7	2.191	2.324	0.852	Diaminopimelate epimerase
bin003	SOY3_bin003_00144	1287	14	25	6	1.300	1.970	0.495	Diaminopimelate decarboxylase
bin003	SOY3_bin003_00145	1179	36	48	24	3.650	4.129	2.162	Acetylornithine aminotransferase
bin003	SOY3_bin003_00146	426	15	27	13	4.209	6.428	3.242	Peptidyl-prolyl cis-trans isomerase B
bin003	SOY3_bin003_00147	516	5	5	3	1.158	0.983	0.618	GAF domain protein
bin003	SOY3_bin003_00148	261	6	5	2	2.748	1.943	0.814	hypothetical protein
bin003	SOY3_bin003_00149	660	8	17	1	1.449	2.613	0.161	hypothetical protein
bin003	SOY3_bin003_00150	984	5	13	4	0.607	1.340	0.432	DNA-3-methyladenine glycosylase

bin003	SOY3_bin003_00151	1398	8	28	2	0.684	2.031	0.152	ferrous iron efflux protein F
bin003	SOY3_bin003_00152	627	3	11	1	0.572	1.779	0.169	hypothetical protein
bin003	SOY3_bin003_00153	411	2	2	0	0.582	0.494	0.000	hypothetical protein
bin003	SOY3_bin003_00154	564	2	9	1	0.424	1.619	0.188	DNA-3-methyladenine glycosylase 1
bin003	SOY3_bin003_00155	1542	29	156	38	2.248	10.261	2.618	putative NAD-dependent malic enzyme 2
bin003	SOY3_bin003_00156	327	0	4	0	0.000	1.241	0.000	putative adenylyltransferase/sulfurtransferase MoeZ
bin003	SOY3_bin003_00157	492	14	22	7	3.402	4.535	1.511	hypothetical protein
bin003	SOY3_bin003_00158	825	61	118	17	8.839	14.507	2.189	Imidazole glycerol phosphate synthase subunit HisF
bin003	SOY3_bin003_00159	726	3	9	2	0.494	1.257	0.293	hypothetical protein
bin003	SOY3_bin003_00160	780	6	16	1	0.920	2.081	0.136	hypothetical protein
bin003	SOY3_bin003_00161	633	10	31	5	1.889	4.967	0.839	Flavin reductase like domain protein
bin003	SOY3_bin003_00162	777	12	39	11	1.846	5.091	1.504	putative adenylyl-sulfate kinase
bin003	SOY3_bin003_00163	732	8	19	5	1.307	2.633	0.726	hypothetical protein
bin003	SOY3_bin003_00164	79	2	3	1	3.027	3.852	1.345	tRNA-Arg(tcg)
bin003	SOY3_bin003_00165	303	1	5	1	0.395	1.674	0.351	Alkyl hydroperoxide reductase AhpD
bin003	SOY3_bin003_00166	5190	86	193	45	1.981	3.772	0.921	ATP-dependent RecD-like DNA helicase
bin003	SOY3_bin003_00167	1050	2	12	8	0.228	1.159	0.809	ATP-dependent DNA helicase DinG
bin003	SOY3_bin003_00168	249	3	3	1	1.440	1.222	0.427	fumarate hydratase
bin003	SOY3_bin003_00169	861	12	32	8	1.666	3.770	0.987	Citrate synthase 2
bin003	SOY3_bin003_00170	327	29	50	12	10.602	15.509	3.898	F0F1 ATP synthase subunit B
bin003	SOY3_bin003_00171	2007	139	332	98	8.280	16.778	5.187	V-type ATP synthase subunit I
bin003	SOY3_bin003_00172	486	23	78	31	5.658	16.278	6.776	V-type sodium ATPase subunit K
bin003	SOY3_bin003_00173	624	41	107	24	7.855	17.392	4.086	V-type ATP synthase subunit E
bin003	SOY3_bin003_00174	1158	73	158	42	7.536	13.839	3.853	V-type ATP synthase subunit C
bin003	SOY3_bin003_00175	321	19	55	7	7.076	17.379	2.316	V-type ATP synthase subunit F
bin003	SOY3_bin003_00176	1755	79	275	56	5.381	15.893	3.390	V-type ATP synthase alpha chain
bin003	SOY3_bin003_00177	1401	56	213	43	4.779	15.420	3.260	V-type sodium ATPase subunit B
bin003	SOY3_bin003_00178	648	19	61	13	3.505	9.548	2.131	V-type sodium ATPase subunit D
bin003	SOY3_bin003_00179	384	13	48	11	4.047	12.678	3.043	hypothetical protein
bin003	SOY3_bin003_00180	450	0	0	0	0.000	0.000	0.000	Arsenate-mycothiol transferase ArsC1
bin003	SOY3_bin003_00181	198	1	1	2	0.604	0.512	1.073	hypothetical protein
bin003	SOY3_bin003_00182	402	2	5	0	0.595	1.262	0.000	Methylisocitrate lyase
bin003	SOY3_bin003_00183	771	3	5	1	0.465	0.658	0.138	putative methyltransferase YcgJ
bin003	SOY3_bin003_00184	1101	3	9	2	0.326	0.829	0.193	Sodium Bile acid symporter family protein
bin003	SOY3_bin003_00185	801	37	54	15	5.522	6.838	1.989	Sulfite exporter TauE/SafE
bin003	SOY3_bin003_00186	408	4	11	4	1.172	2.735	1.041	hypothetical protein
bin003	SOY3_bin003_00187	156	2	1	1	1.533	0.650	0.681	hypothetical protein
bin003	SOY3_bin003_00188	1200	8	12	1	0.797	1.014	0.089	carbamoyl phosphate synthase-like protein
bin003	SOY3_bin003_00189	77	2	4	0	3.105	5.269	0.000	tRNA-Gln(ttg)
bin003	SOY3_bin003_00190	666	16	32	10	2.872	4.873	1.595	PD-(D/E)XK nuclease superfamily protein
bin003	SOY3_bin003_00191	570	5	10	4	1.049	1.779	0.745	Tyrosine-protein kinase etk
bin003	SOY3_bin003_00192	315	3	13	1	1.139	4.186	0.337	hypothetical protein
bin003	SOY3_bin003_00193	204	3	0	0	1.758	0.000	0.000	hypothetical protein
bin003	SOY3_bin003_00194	531	5	3	1	1.126	0.573	0.200	adenylate cyclase
bin003	SOY3_bin003_00195	135	2	1	0	1.771	0.751	0.000	hypothetical protein
bin003	SOY3_bin003_00196	74	0	0	1	0.000	0.000	1.435	tRNA-Lys(ctt)
bin003	SOY3_bin003_00197	237	3	9	0	1.513	3.852	0.000	YcfA-like protein
bin003	SOY3_bin003_00198	342	27	23	4	9.438	6.821	1.242	hypothetical protein
bin003	SOY3_bin003_00199	74	1	0	0	1.616	0.000	0.000	tRNA-Lys(ctt)
bin003	SOY3_bin003_00200	1140	13	7	2	1.363	0.623	0.186	hypothetical protein
bin003	SOY3_bin003_00201	765	19	26	7	2.969	3.447	0.972	Inner membrane transport permease YadH
bin003	SOY3_bin003_00202	954	17	26	5	2.130	2.764	0.557	Daunorubicin/doxorubicin resistance ATP-binding protein DrrA
bin003	SOY3_bin003_00203	1467	2	10	3	0.163	0.691	0.217	Sugar-specific transcriptional regulator TrmB
bin003	SOY3_bin003_00204	1368	5	12	1	0.437	0.890	0.078	Beta-lactamase hydrolase-like protein
bin003	SOY3_bin003_00205	1224	0	1	0	0.000	0.083	0.000	hypothetical protein
bin003	SOY3_bin003_00206	213	0	0	0	0.000	0.000	0.000	hypothetical protein
bin003	SOY3_bin003_00207	489	3	12	0	0.733	2.489	0.000	hypothetical protein
bin003	SOY3_bin003_00208	486	11	18	1	2.706	3.757	0.219	TspO/MBR family protein
bin003	SOY3_bin003_00209	207	12	22	7	6.930	10.780	3.592	hypothetical protein
bin003	SOY3_bin003_00210	240	1	4	0	0.498	1.690	0.000	putative regulator PrlF
bin003	SOY3_bin003_00211	387	1	2	1	0.309	0.524	0.274	tRNA(fMet)-specific endonuclease VapC
bin003	SOY3_bin003_00212	690	10	10	2	1.733	1.470	0.308	DNA alkylation repair enzyme
bin003	SOY3_bin003_00213	336	15	10	6	5.337	3.019	1.897	hypothetical protein
bin003	SOY3_bin003_00214	399	5	13	3	1.498	3.305	0.799	hypothetical protein
bin003	SOY3_bin003_00215	387	5	5	1	1.545	1.310	0.274	hypothetical protein
bin003	SOY3_bin003_00216	462	9	16	2	2.329	3.513	0.460	Bacterial transcription activator, effector binding domain
bin003	SOY3_bin003_00217	546	19	9	2	4.160	1.672	0.389	lineage-specific thermal regulator protein

bin003	SOY3_bin003_00218	1725	4	12	2	0.277	0.706	0.123	H(+)/Cl(-) exchange transporter ClcA
bin003	SOY3_bin003_00219	1104	14	30	6	1.516	2.756	0.577	Glucans biosynthesis protein C
bin003	SOY3_bin003_00220	1242	7	15	3	0.674	1.225	0.257	L-lactate dehydrogenase [cytochrome]
bin003	SOY3_bin003_00221	729	2	9	1	0.328	1.252	0.146	Fumarate reductase iron-sulfur subunit
bin003	SOY3_bin003_00222	342	2	8	2	0.699	2.373	0.621	peptidyl-tRNA hydrolase
bin003	SOY3_bin003_00223	1191	41	51	12	4.115	4.343	1.070	YcaO-like family protein
bin003	SOY3_bin003_00224	591	15	29	7	3.034	4.977	1.258	Peptidase family M50
bin003	SOY3_bin003_00225	453	13	9	3	3.431	2.015	0.703	HIT domain protein
bin003	SOY3_bin003_00226	990	9	15	7	1.087	1.537	0.751	putative peptidase
bin003	SOY3_bin003_00227	933	29	68	8	3.716	7.392	0.911	flagellar assembly protein J
bin003	SOY3_bin003_00228	195	3	13	1	1.839	6.762	0.545	Class III signal peptide
bin003	SOY3_bin003_00229	1401	13	45	5	1.109	3.258	0.379	Succinate-semialdehyde dehydrogenase [NADP(+)] GabD
bin003	SOY3_bin003_00230	723	8	17	4	1.323	2.385	0.588	Methionine--tRNA ligase
bin003	SOY3_bin003_00231	246	5	9	1	2.430	3.711	0.432	hypothetical protein
bin003	SOY3_bin003_00232	990	282	951	221	34.053	97.432	23.713	Pseudomurein-binding repeat protein
bin003	SOY3_bin003_00233	942	7	11	4	0.888	1.184	0.451	Carbamate kinase 2
bin003	SOY3_bin003_00234	1299	21	28	4	1.933	2.186	0.327	Arginine deiminase
bin003	SOY3_bin003_00235	336	43	102	20	15.299	30.790	6.323	PRC-barrel domain protein
bin003	SOY3_bin003_00236	765	59	82	17	9.220	10.872	2.361	L-aspartate dehydrogenase
bin003	SOY3_bin003_00237	759	24	23	4	3.780	3.074	0.560	tRNAHis guanylyltransferase
bin003	SOY3_bin003_00238	441	8	7	2	2.169	1.610	0.482	Mov34/MPN/PAD-1 family protein
bin003	SOY3_bin003_00239	2340	88	179	34	4.496	7.759	1.543	Outer cell wall protein precursor
bin003	SOY3_bin003_00240	1572	32	60	25	2.434	3.871	1.689	D-3-phosphoglycerate dehydrogenase
bin003	SOY3_bin003_00241	342	11	26	8	3.845	7.711	2.485	hypothetical protein
bin003	SOY3_bin003_00242	750	17	28	9	2.710	3.787	1.275	hypothetical protein
bin003	SOY3_bin003_00243	990	16	23	12	1.932	2.356	1.288	hypothetical protein
bin003	SOY3_bin003_00244	270	1	2	0	0.443	0.751	0.000	hypothetical protein
bin003	SOY3_bin003_00245	6171	36	70	16	0.697	1.151	0.275	Lactococcin-G-processing and transport ATP-binding protein LagD
bin003	SOY3_bin003_00246	363	1	1	0	0.329	0.279	0.000	hypothetical protein
bin003	SOY3_bin003_00247	735	6	7	4	0.976	0.966	0.578	Peptidase C39 family protein
bin003	SOY3_bin003_00248	621	3	5	0	0.578	0.817	0.000	TPR repeat-containing protein YrrB
bin003	SOY3_bin003_00249	720	0	0	0	0.000	0.000	0.000	hypothetical protein
bin003	SOY3_bin003_00250	312	1	0	0	0.383	0.000	0.000	hypothetical protein
bin003	SOY3_bin003_00251	183	2	1	0	1.307	0.554	0.000	hypothetical protein
bin003	SOY3_bin003_00252	438	0	0	0	0.000	0.000	0.000	hypothetical protein
bin003	SOY3_bin003_00253	228	1	0	0	0.524	0.000	0.000	hypothetical protein
bin003	SOY3_bin003_00254	288	1	0	1	0.415	0.000	0.369	hypothetical protein
bin003	SOY3_bin003_00255	570	2	7	0	0.419	1.246	0.000	hypothetical protein
bin003	SOY3_bin003_00256	192	0	0	0	0.000	0.000	0.000	hypothetical protein
bin003	SOY3_bin003_00257	1545	23	35	15	1.780	2.298	1.031	Lactate utilization protein A
bin003	SOY3_bin003_00258	768	8	6	2	1.245	0.792	0.277	hypothetical protein
bin003	SOY3_bin003_00259	576	6	5	0	1.245	0.880	0.000	WLM domain protein
bin003	SOY3_bin003_00260	1863	49	54	14	3.144	2.940	0.798	2-oxoglutarate oxidoreductase subunit KorB
bin003	SOY3_bin003_00261	594	8	26	2	1.610	4.440	0.358	indolepyruvate oxidoreductase subunit beta
bin003	SOY3_bin003_00262	282	2	1	1	0.848	0.360	0.377	HTH-type transcriptional repressor SmtB
bin003	SOY3_bin003_00263	324	10	16	5	3.690	5.009	1.639	hypothetical protein
bin003	SOY3_bin003_00264	1134	12	26	7	1.265	2.325	0.656	Macrolide export ATP-binding/permease protein MacB
bin003	SOY3_bin003_00265	675	8	28	7	1.417	4.207	1.102	Bacitracin export ATP-binding protein BceA
bin003	SOY3_bin003_00266	384	6	12	1	1.868	3.170	0.277	ACT domain protein
bin003	SOY3_bin003_00267	1302	38	73	15	3.489	5.687	1.224	Phenylacetate-coenzyme A ligase
bin003	SOY3_bin003_00268	948	19	43	12	2.396	4.601	1.345	hypothetical protein
bin003	SOY3_bin003_00269	1614	13	22	7	0.963	1.383	0.461	Sodium/pantothenate symporter
bin003	SOY3_bin003_00270	843	3	11	3	0.425	1.323	0.378	N-acyl homoserine lactonase AttM
bin003	SOY3_bin003_00271	1023	13	21	6	1.519	2.082	0.623	Calcineurin-like phosphoesterase superfamily domain protein
bin003	SOY3_bin003_00272	363	5	8	2	1.647	2.235	0.585	Calcineurin-like phosphoesterase
bin003	SOY3_bin003_00273	366	0	0	0	0.000	0.000	0.000	hypothetical protein
bin003	SOY3_bin003_00274	474	2	1	0	0.504	0.214	0.000	hypothetical protein
bin003	SOY3_bin003_00275	639	3	16	6	0.561	2.540	0.997	hypothetical protein
bin003	SOY3_bin003_00276	186	2	1	0	1.285	0.545	0.000	hypothetical protein
bin003	SOY3_bin003_00277	594	5	7	2	1.006	1.195	0.358	2,6-dihydropseudooxynicotine hydrolase
bin003	SOY3_bin003_00278	768	21	40	16	3.269	5.283	2.213	hypothetical protein
bin003	SOY3_bin003_00279	126	0	1	1	0.000	0.805	0.843	hypothetical protein
bin003	SOY3_bin003_00280	780	10	4	2	1.533	0.520	0.272	Acetyltransferase (GNAT) family protein
bin003	SOY3_bin003_00281	693	10	12	0	1.725	1.756	0.000	hypothetical protein
bin003	SOY3_bin003_00282	1683	47	65	28	3.339	3.917	1.767	Hydroxyneurosporene synthase (CrtC)
bin003	SOY3_bin003_00283	870	19	20	6	2.611	2.332	0.733	Quercetin 2,3-dioxygenase
bin003	SOY3_bin003_00284	1248	18	11	5	1.724	0.894	0.426	Dihydrolipoyl dehydrogenase

bin003	SOY3_bin003_00285	825	18	34	4	2.608	4.180	0.515	hypothetical protein
bin003	SOY3_bin003_00286	327	11	12	4	4.022	3.722	1.299	hypothetical protein
bin003	SOY3_bin003_00287	495	66	97	33	15.940	19.876	7.082	acid-resistance membrane protein
bin003	SOY3_bin003_00288	1527	12	18	5	0.939	1.196	0.348	Antiseptic resistance protein
bin003	SOY3_bin003_00289	342	12	17	6	4.195	5.042	1.864	hypothetical protein
bin003	SOY3_bin003_00290	207	6	15	0	3.465	7.350	0.000	hypothetical protein
bin003	SOY3_bin003_00291	1446	2	1	0	0.165	0.070	0.000	Polysaccharide biosynthesis protein
bin003	SOY3_bin003_00292	504	3	1	0	0.712	0.201	0.000	hypothetical protein
bin003	SOY3_bin003_00293	1011	39	62	14	4.612	6.220	1.471	2-oxoglutarate oxidoreductase subunit KorA
bin003	SOY3_bin003_00294	864	25	54	5	3.459	6.339	0.615	2-oxoglutarate oxidoreductase subunit KorB
bin003	SOY3_bin003_00295	549	15	24	6	3.266	4.434	1.161	NADH-dependent phenylglyoxylate dehydrogenase subunit gamma
bin003	SOY3_bin003_00296	1107	41	46	15	4.428	4.215	1.439	Succinyl-CoA ligase [ADP-forming] subunit beta
bin003	SOY3_bin003_00297	864	37	76	17	5.120	8.922	2.090	hypothetical protein
bin003	SOY3_bin003_00298	915	1	20	7	0.131	2.217	0.813	Wyosine base formation
bin003	SOY3_bin003_00299	831	93	137	31	13.379	16.721	3.963	Pseudomurein-binding repeat protein
bin003	SOY3_bin003_00300	681	12	29	10	2.107	4.319	1.560	Triosephosphate isomerase
bin003	SOY3_bin003_00301	1227	32	72	18	3.118	5.952	1.558	Phosphoglycerate kinase
bin003	SOY3_bin003_00302	75	2	7	2	3.188	9.467	2.833	tRNA-Thr(tgt)
bin003	SOY3_bin003_00303	75	1	1	3	1.594	1.352	4.249	tRNA-Pro(tgg)
bin003	SOY3_bin003_00304	76	1	1	0	1.573	1.335	0.000	tRNA-Tyr(gta)
bin003	SOY3_bin003_00305	74	4	11	5	6.462	15.077	7.177	tRNA-Asp(gtc)
bin003	SOY3_bin003_00306	74	1	3	1	1.616	4.112	1.435	tRNA-Lys(ttt)
bin003	SOY3_bin003_00307	1491	87	155	42	6.976	10.544	2.992	DNA-directed RNA polymerase subunit beta
bin003	SOY3_bin003_00308	1854	98	192	62	6.319	10.504	3.552	DNA-directed RNA polymerase subunit beta
bin003	SOY3_bin003_00309	2613	157	277	55	7.183	10.752	2.236	DNA-directed RNA polymerase subunit beta'
bin003	SOY3_bin003_00310	1167	121	168	41	12.395	14.601	3.732	DNA-directed RNA polymerase subunit beta'
bin003	SOY3_bin003_00311	297	19	38	7	7.648	12.977	2.504	50S ribosomal protein L30e
bin003	SOY3_bin003_00312	432	29	57	14	8.025	13.383	3.443	hypothetical protein
bin003	SOY3_bin003_00313	426	43	102	35	12.067	24.285	8.727	30S ribosomal protein S12
bin003	SOY3_bin003_00314	564	48	135	35	10.174	24.278	6.592	30S ribosomal protein S7
bin003	SOY3_bin003_00315	2175	286	494	152	15.720	23.037	7.424	Elongation factor G
bin003	SOY3_bin003_00316	1242	366	602	169	35.229	49.162	14.454	Elongation factor Tu
bin003	SOY3_bin003_00317	309	57	110	29	22.053	36.107	9.969	30S ribosomal protein S10
bin003	SOY3_bin003_00318	85	0	0	0	0.000	0.000	0.000	tRNA-Ser(tga)
bin003	SOY3_bin003_00319	1677	9	10	8	0.642	0.605	0.507	Beta-lactamase precursor
bin003	SOY3_bin003_00320	1719	11	24	4	0.765	1.416	0.247	Beta-lactamase precursor
bin003	SOY3_bin003_00321	1074	1	4	1	0.111	0.378	0.099	putative inner membrane protein
bin003	SOY3_bin003_00322	471	1	4	0	0.254	0.861	0.000	hypothetical protein
bin003	SOY3_bin003_00323	384	0	1	0	0.000	0.264	0.000	Demethylrebeccamycin-D-glucose O-methyltransferase
bin003	SOY3_bin003_00324	261	0	2	1	0.000	0.777	0.407	arsenite S-adenosylmethyltransferase
bin003	SOY3_bin003_00325	690	1	4	1	0.173	0.588	0.154	putative metallo-hydrolase YflN
bin003	SOY3_bin003_00326	1263	3	4	3	0.284	0.321	0.252	General stress protein 69
bin003	SOY3_bin003_00327	561	2	2	0	0.426	0.362	0.000	HTH-type transcriptional repressor Bm3R1
bin003	SOY3_bin003_00328	834	37	52	7	5.304	6.324	0.892	Hydroxymethylglutaryl-coenzyme A reductase
bin003	SOY3_bin003_00329	579	10	11	3	2.065	1.927	0.550	VIT family protein
bin003	SOY3_bin003_00330	780	6	9	4	0.920	1.170	0.545	Leucine carboxyl methyltransferase
bin003	SOY3_bin003_00331	387	0	1	0	0.000	0.262	0.000	hypothetical protein
bin003	SOY3_bin003_00332	846	13	14	3	1.837	1.678	0.377	putative deoxyribonuclease YjjV
bin003	SOY3_bin003_00333	852	13	20	5	1.824	2.381	0.623	Shikimate dehydrogenase
bin003	SOY3_bin003_00334	387	6	1	2	1.853	0.262	0.549	hypothetical protein
bin003	SOY3_bin003_00335	510	37	42	9	8.673	8.353	1.875	hypothetical protein
bin003	SOY3_bin003_00336	1296	39	61	17	3.598	4.774	1.393	Histidine--tRNA ligase
bin003	SOY3_bin003_00337	405	18	13	4	5.313	3.256	1.049	phosphoribosyl-AMP cyclohydrolase
bin003	SOY3_bin003_00338	1827	85	117	24	5.562	6.495	1.395	polynucleotide phosphorylase/polyadenylase
bin003	SOY3_bin003_00339	783	21	29	9	3.206	3.757	1.221	endonuclease IV
bin003	SOY3_bin003_00340	642	4	1	1	0.745	0.158	0.165	Undecaprenyl-diphosphatase BcrC
bin003	SOY3_bin003_00341	669	15	30	7	2.680	4.548	1.111	prephenate dehydrogenase
bin003	SOY3_bin003_00342	585	11	43	7	2.248	7.455	1.271	3-hexulose-6-phosphate isomerase
bin003	SOY3_bin003_00343	504	10	17	1	2.372	3.421	0.211	tRNA-splicing endonuclease subunit alpha
bin003	SOY3_bin003_00344	1092	22	25	5	2.408	2.322	0.486	Tryptophan--tRNA ligase
bin003	SOY3_bin003_00345	603	18	25	6	3.569	4.205	1.057	hypothetical protein
bin003	SOY3_bin003_00346	1197	33	51	8	3.296	4.321	0.710	Threonine synthase
bin003	SOY3_bin003_00347	207	267	655	168	154.201	320.941	86.212	Histone-like transcription factor (CBF/NF-Y) and archaeal histone
bin003	SOY3_bin003_00348	501	3	12	5	0.716	2.429	1.060	Chagasin family peptidase inhibitor I42
bin003	SOY3_bin003_00349	201	14	14	10	8.327	7.065	5.285	hypothetical protein
bin003	SOY3_bin003_00350	372	58	110	27	18.639	29.992	7.710	Ribosome-associated protein L7Ae-like protein
bin003	SOY3_bin003_00351	207	36	62	19	20.791	30.379	9.750	30S ribosomal protein S28e

bin003	SOY3_bin003_00352	132	11	38	11	9.962	29.199	8.852	50S ribosomal protein L24e
bin003	SOY3_bin003_00353	459	87	123	31	22.660	27.180	7.174	Nucleoside diphosphate kinase
bin003	SOY3_bin003_00354	1779	155	190	46	10.416	10.833	2.747	Translation initiation factor IF-2
bin003	SOY3_bin003_00355	381	45	56	11	14.120	14.908	3.067	30S ribosomal protein S6e
bin003	SOY3_bin003_00356	1227	144	212	58	14.030	17.525	5.021	Selenocysteine-specific elongation factor
bin003	SOY3_bin003_00357	354	32	41	4	10.807	11.747	1.200	Fcf1
bin003	SOY3_bin003_00358	654	36	66	7	6.581	10.236	1.137	Inorganic pyrophosphatase
bin003	SOY3_bin003_00359	603	69	89	23	13.680	14.970	4.052	30S ribosomal protein S1
bin003	SOY3_bin003_00360	183	12	23	8	7.839	12.748	4.644	DNA-directed RNA polymerase subunit E"
bin003	SOY3_bin003_00361	534	62	67	24	13.880	12.726	4.774	hypothetical protein
bin003	SOY3_bin003_00362	354	33	45	12	11.144	12.893	3.601	30S ribosomal protein S24e
bin003	SOY3_bin003_00363	165	11	26	2	7.970	15.982	1.288	30S ribosomal protein S27ae
bin003	SOY3_bin003_00364	1428	80	100	21	6.697	7.103	1.562	Argininosuccinate lyase 1
bin003	SOY3_bin003_00365	195	4	6	1	2.452	3.121	0.545	hypothetical protein
bin003	SOY3_bin003_00366	186	0	6	3	0.000	3.272	1.713	Putative transposase DNA-binding domain protein
bin003	SOY3_bin003_00367	156	12	18	7	9.196	11.703	4.767	hypothetical protein
bin003	SOY3_bin003_00368	1032	19	25	4	2.201	2.457	0.412	tRNA-specific 2-thiouridylase MnmA
bin003	SOY3_bin003_00369	1665	6	16	1	0.431	0.975	0.064	hypothetical protein
bin003	SOY3_bin003_00370	1587	45	73	18	3.390	4.666	1.205	Elongation factor Tu
bin003	SOY3_bin003_00371	333	2	0	0	0.718	0.000	0.000	hypothetical protein
bin003	SOY3_bin003_00372	576	1	5	1	0.208	0.880	0.184	hypothetical protein
bin003	SOY3_bin003_00373	366	6	10	1	1.960	2.771	0.290	hypothetical protein
bin003	SOY3_bin003_00374	291	2	3	0	0.822	1.046	0.000	Bacterial regulatory protein, arsR family
bin003	SOY3_bin003_00375	852	8	11	1	1.123	1.310	0.125	tRNA 2-thiocytidine biosynthesis protein TtcA
bin003	SOY3_bin003_00376	303	1	6	2	0.395	2.008	0.701	hypothetical protein
bin003	SOY3_bin003_00377	1095	32	64	15	3.494	5.928	1.455	UDP-2-acetamido-2-deoxy-3-oxo-D-glucuronate aminotransferase
bin003	SOY3_bin003_00378	1152	12	27	6	1.245	2.377	0.553	tRNA (guanine(26)-N(2)/guanine(27)-N(2))-dimethyltransferase
bin003	SOY3_bin003_00379	675	4	3	0	0.708	0.451	0.000	hypothetical protein
bin003	SOY3_bin003_00380	705	155	458	110	26.284	65.892	16.574	hypothetical protein
bin003	SOY3_bin003_00381	723	5	6	0	0.827	0.842	0.000	hypothetical protein
bin003	SOY3_bin003_00382	1863	20	83	11	1.283	4.519	0.627	Glutamate synthase [NADPH] large chain
bin003	SOY3_bin003_00383	642	3	22	2	0.559	3.476	0.331	Glutamate synthase [NADPH] large chain
bin003	SOY3_bin003_00384	549	5	13	4	1.089	2.402	0.774	Sugar-specific transcriptional regulator TrmB
bin003	SOY3_bin003_00385	219	16	43	17	8.734	19.915	8.246	hypothetical protein
bin003	SOY3_bin003_00386	483	6	16	5	1.485	3.360	1.100	Regulatory protein AsnC
bin003	SOY3_bin003_00387	1089	23	67	11	2.525	6.240	1.073	Aminodeoxyfutalosine synthase
bin003	SOY3_bin003_00388	438	12	30	8	3.275	6.947	1.940	hypothetical protein
bin003	SOY3_bin003_00389	963	32	113	23	3.973	11.902	2.537	GTP cyclohydrolase FolE2
bin003	SOY3_bin003_00390	681	2	11	0	0.351	1.638	0.000	hypothetical protein
bin003	SOY3_bin003_00391	957	8	17	5	0.999	1.802	0.555	Histone deacetylase-like amidohydrolase
bin003	SOY3_bin003_00392	735	10	19	3	1.627	2.622	0.434	23S rRNA m(5)U1939 methyltransferase
bin003	SOY3_bin003_00393	450	9	24	9	2.391	5.409	2.125	NADP-reducing hydrogenase subunit HndA
bin003	SOY3_bin003_00394	624	135	191	53	25.864	31.046	9.022	Proteasome subunit beta precursor
bin003	SOY3_bin003_00395	1905	167	210	32	10.480	11.181	1.784	Ribonuclease
bin003	SOY3_bin003_00396	1029	30	59	5	3.485	5.816	0.516	Phosphoribosylformylglycinamidine cyclo-ligase
bin003	SOY3_bin003_00397	1026	27	36	14	3.146	3.559	1.449	putative oxidoreductase YbiC
bin003	SOY3_bin003_00398	390	6	20	7	1.839	5.201	1.907	Double zinc ribbon
bin003	SOY3_bin003_00399	342	15	43	9	5.243	12.753	2.795	hypothetical protein
bin003	SOY3_bin003_00400	846	174	1039	209	24.588	124.566	26.243	putative formate transporter 1
bin003	SOY3_bin003_00401	2094	248	1516	333	14.159	73.431	16.893	Formate dehydrogenase H
bin003	SOY3_bin003_00402	435	64	368	71	17.589	85.805	17.338	hypothetical protein
bin003	SOY3_bin003_00403	456	2	3	0	0.524	0.667	0.000	Multidrug resistance operon repressor
bin003	SOY3_bin003_00404	261	0	3	0	0.000	1.166	0.000	Multidrug efflux protein YfmO
bin003	SOY3_bin003_00405	294	0	0	0	0.000	0.000	0.000	Multidrug efflux protein YfmO
bin003	SOY3_bin003_00406	261	2	0	0	0.916	0.000	0.000	Hypoxic response protein 1
bin003	SOY3_bin003_00407	99	0	0	0	0.000	0.000	0.000	CBS domain protein
bin003	SOY3_bin003_00408	267	2	10	3	0.895	3.799	1.194	hypothetical protein
bin003	SOY3_bin003_00409	459	4	7	4	1.042	1.547	0.926	putative kinase inhibitor protein
bin003	SOY3_bin003_00410	1689	26	54	9	1.840	3.243	0.566	putative ABC transporter ATP-binding protein YjjK
bin003	SOY3_bin003_00411	408	7	21	5	2.051	5.221	1.302	UDP-2,3-diacylglucosamine hydrolase
bin003	SOY3_bin003_00412	237	0	1	0	0.000	0.428	0.000	UDP-2,3-diacylglucosamine hydrolase
bin003	SOY3_bin003_00413	1155	30	59	15	3.105	5.181	1.380	Aspartate aminotransferase
bin003	SOY3_bin003_00414	705	22	30	14	3.731	4.316	2.109	hypothetical protein
bin003	SOY3_bin003_00415	654	10	23	5	1.828	3.567	0.812	HTH-type transcriptional repressor Bm3R1
bin003	SOY3_bin003_00416	1542	20	21	2	1.551	1.381	0.138	Antilisterial bacteriocin subtilosin biosynthesis protein AlbA
bin003	SOY3_bin003_00417	1452	35	41	8	2.882	2.864	0.585	(Dimethylallyl)adenosine tRNA methylthiotransferase MiaB
bin003	SOY3_bin003_00418	1191	21	50	19	2.108	4.258	1.695	putative peptidase

bin003	SOY3_bin003_00419	594	5	16	0	1.006	2.732	0.000	Threonylcarbamoyl-AMP synthase
bin003	SOY3_bin003_00420	585	6	17	6	1.226	2.947	1.089	CDP-diacylglycerol--inositol 3-phosphatidyltransferase
bin003	SOY3_bin003_00421	234	5	11	3	2.554	4.768	1.362	hypothetical protein
bin003	SOY3_bin003_00422	657	14	27	4	2.547	4.168	0.647	hypothetical protein
bin003	SOY3_bin003_00423	681	10	25	5	1.755	3.723	0.780	hypothetical protein
bin003	SOY3_bin003_00424	1101	85	227	49	9.229	20.912	4.728	Fructose-1,6-bisphosphatase
bin003	SOY3_bin003_00425	990	15	29	5	1.811	2.971	0.536	Homoisocitrate dehydrogenase
bin003	SOY3_bin003_00426	573	34	29	8	7.094	5.133	1.483	putative phycocyanin operon protein Z
bin003	SOY3_bin003_00427	945	38	51	8	4.807	5.474	0.899	L-lactate dehydrogenase
bin003	SOY3_bin003_00428	579	7	15	3	1.445	2.628	0.550	Glutamine amidotransferase subunit PdxT
bin003	SOY3_bin003_00429	918	15	28	3	1.953	3.094	0.347	Amidophosphoribosyltransferase precursor
bin003	SOY3_bin003_00430	690	10	22	12	1.733	3.234	1.847	Formyltransferase/hydrolase complex Fhc subunit C
bin003	SOY3_bin003_00431	1095	18	33	5	1.965	3.057	0.485	coenzyme F420-reducing hydrogenase subunit beta
bin003	SOY3_bin003_00432	1503	75	101	11	5.966	6.816	0.777	Glutamate synthase [NADPH] large chain
bin003	SOY3_bin003_00433	1230	3	5	0	0.292	0.412	0.000	Ammonia channel precursor
bin003	SOY3_bin003_00434	339	36	59	14	12.695	17.653	4.387	Nitrogen regulatory protein P-II
bin003	SOY3_bin003_00435	1230	3	2	0	0.292	0.165	0.000	Ammonium transporter NrgA
bin003	SOY3_bin003_00436	342	2	19	5	0.699	5.635	1.553	Nitrogen regulatory protein P-II
bin003	SOY3_bin003_00437	606	0	2	0	0.000	0.335	0.000	4-pyridoxolactonase
bin003	SOY3_bin003_00438	498	0	0	2	0.000	0.000	0.427	Translation initiation factor IF-2
bin003	SOY3_bin003_00439	210	73	237	46	41.557	114.468	23.268	Histone-like transcription factor (CBF/NF-Y) and archaeal histone
bin003	SOY3_bin003_00440	381	9	26	9	2.824	6.922	2.509	Helix-turn-helix
bin003	SOY3_bin003_00441	714	14	11	5	2.344	1.563	0.744	Serine acetyltransferase
bin003	SOY3_bin003_00442	987	1	6	1	0.121	0.617	0.108	O-acetylserine sulfhydrylase
bin003	SOY3_bin003_00443	768	7	16	3	1.090	2.113	0.415	hypothetical protein
bin003	SOY3_bin003_00444	126	0	1	0	0.000	0.805	0.000	hypothetical protein
bin003	SOY3_bin003_00445	528	1	4	0	0.226	0.768	0.000	Ergosterol biosynthesis ERG4/ERG24 family protein
bin003	SOY3_bin003_00446	225	2	2	2	1.063	0.902	0.944	Sulfurtransferase TusA
bin003	SOY3_bin003_00447	420	1	6	4	0.285	1.449	1.012	DsrE/DsrF-like family protein
bin003	SOY3_bin003_00448	1161	19	56	7	1.956	4.892	0.640	Cysteine desulfurase
bin003	SOY3_bin003_00449	831	19	25	7	2.733	3.051	0.895	Tyrosine-protein kinase YwqD
bin003	SOY3_bin003_00450	495	7	9	3	1.691	1.844	0.644	Putative phosphinothricin acetyltransferase YwnH
bin003	SOY3_bin003_00451	687	18	23	3	3.132	3.396	0.464	Kynurenine formamidase
bin003	SOY3_bin003_00452	348	0	0	0	0.000	0.000	0.000	FeMo cofactor biosynthesis protein NifB
bin003	SOY3_bin003_00453	330	1	1	0	0.362	0.307	0.000	FeMo cofactor biosynthesis protein NifB
bin003	SOY3_bin003_00454	1197	1	3	1	0.100	0.254	0.089	Cystathionine beta-lyase PatB
bin003	SOY3_bin003_00455	990	2	2	0	0.242	0.205	0.000	D-3-phosphoglycerate dehydrogenase
bin003	SOY3_bin003_00456	396	15	27	6	4.528	6.915	1.609	Pyridoxamine 5'-phosphate oxidase
bin003	SOY3_bin003_00457	273	4	5	3	1.752	1.858	1.167	ThiS family protein
bin003	SOY3_bin003_00458	897	1	3	2	0.133	0.339	0.237	Putative aliphatic sulfonates-binding protein precursor
bin003	SOY3_bin003_00459	768	13	25	5	2.024	3.302	0.692	hypothetical protein
bin003	SOY3_bin003_00460	2688	28	448	99	1.245	16.905	3.912	FmdE, Molybdenum formylmethanofuran dehydrogenase operon
bin003	SOY3_bin003_00461	498	1	8	1	0.240	1.629	0.213	hypothetical protein
bin003	SOY3_bin003_00462	624	3	5	1	0.575	0.813	0.170	Cobalt transport protein CbiM precursor
bin003	SOY3_bin003_00463	516	2	18	5	0.463	3.538	1.029	hypothetical protein
bin003	SOY3_bin003_00464	2379	46	75	28	2.312	3.198	1.250	NAD(P)H-quinone oxidoreductase subunit I
bin003	SOY3_bin003_00465	954	15	28	9	1.880	2.977	1.002	Anaerobic glycerol-3-phosphate dehydrogenase subunit C
bin003	SOY3_bin003_00466	621	7	9	2	1.348	1.470	0.342	Anaerobic glycerol-3-phosphate dehydrogenase subunit C
bin003	SOY3_bin003_00467	1011	13	17	1	1.537	1.706	0.105	Sulfur carrier protein ThiS adenylyltransferase
bin003	SOY3_bin003_00468	1350	20	43	6	1.771	3.231	0.472	Fructose dehydrogenase large subunit
bin003	SOY3_bin003_00469	1380	16	16	4	1.386	1.176	0.308	Cysteine--tRNA ligase
bin003	SOY3_bin003_00470	71	0	0	0	0.000	0.000	0.000	tRNA-Cys(gca)
bin003	SOY3_bin003_00471	906	14	17	7	1.847	1.903	0.821	Septum site-determining protein MinD
bin003	SOY3_bin003_00472	1209	16	8	3	1.582	0.671	0.264	putative cation efflux system protein/MT2084
bin003	SOY3_bin003_00473	654	15	13	6	2.742	2.016	0.975	Metallo-beta-lactamase L1 precursor
bin003	SOY3_bin003_00474	459	6	6	4	1.563	1.326	0.926	Transcriptional regulator MntR
bin003	SOY3_bin003_00475	1338	38	59	23	3.395	4.473	1.826	(Dimethylallyl)adenosine tRNA methylthiotransferase MiaB
bin003	SOY3_bin003_00476	1248	85	96	29	8.142	7.802	2.468	Radical SAM superfamily protein
bin003	SOY3_bin003_00477	1329	2163	5794	1334	194.571	442.189	106.625	hypothetical protein
bin003	SOY3_bin003_00478	441	755	2016	445	204.670	463.668	107.189	Methyl-coenzyme M reductase operon protein D
bin003	SOY3_bin003_00479	597	817	2476	568	163.604	420.660	101.066	Methyl-coenzyme M reductase operon protein C
bin003	SOY3_bin003_00480	750	1320	3826	791	210.406	517.414	112.033	Methyl-coenzyme M reductase gamma subunit
bin003	SOY3_bin003_00481	1653	3029	7850	1804	219.064	481.672	115.929	hypothetical protein
bin003	SOY3_bin003_00482	909	400	977	202	52.607	109.015	23.606	tetrahydromethanopterin S-methyltransferase subunit E
bin003	SOY3_bin003_00483	729	317	699	172	51.985	97.253	25.063	tetrahydromethanopterin S-methyltransferase subunit D
bin003	SOY3_bin003_00484	810	296	758	179	43.687	94.916	23.475	tetrahydromethanopterin S-methyltransferase subunit C
bin003	SOY3_bin003_00485	312	110	327	62	42.149	106.304	21.109	tetrahydromethanopterin S-methyltransferase subunit B

bin003	SOY3_bin003_00486	723	318	926	202	52.582	129.905	29.679	tetrahydromethanopterin S-methyltransferase subunit A
bin003	SOY3_bin003_00487	207	110	286	44	63.528	140.136	22.579	Tetrahydromethanopterin S-methyltransferase, F subunit (MtrF)
bin003	SOY3_bin003_00488	252	142	347	88	67.365	139.664	37.095	tetrahydromethanopterin S-methyltransferase subunit G
bin003	SOY3_bin003_00489	954	660	1302	331	82.707	138.426	36.856	tetrahydromethanopterin S-methyltransferase subunit H
bin003	SOY3_bin003_00490	984	13	26	5	1.579	2.680	0.540	Inner membrane protein YrbG
bin003	SOY3_bin003_00491	429	5	11	1	1.393	2.601	0.248	Putative universal stress protein
bin003	SOY3_bin003_00492	1488	46	85	14	3.696	5.794	0.999	hypothetical protein
bin003	SOY3_bin003_00493	114	0	2	1	0.000	1.779	0.932	hypothetical protein
bin003	SOY3_bin003_00494	399	0	4	2	0.000	1.017	0.532	Pyridoxamine 5'-phosphate oxidase
bin003	SOY3_bin003_00495	633	3	16	2	0.567	2.564	0.336	Bifunctional protein GlmU
bin003	SOY3_bin003_00496	270	8	28	4	3.542	10.518	1.574	PRC-barrel domain protein
bin003	SOY3_bin003_00497	498	16	26	1	3.841	5.295	0.213	Formaldehyde-activating enzyme
bin003	SOY3_bin003_00498	1227	36	104	23	3.508	8.597	1.991	FeS cluster assembly protein SufB
bin003	SOY3_bin003_00499	759	15	36	9	2.363	4.811	1.260	putative ABC transporter ATP-binding protein
bin003	SOY3_bin003_00500	477	7	5	2	1.754	1.063	0.445	hypothetical protein
bin003	SOY3_bin003_00501	441	190	414	104	51.506	95.217	25.051	Methyl-viologen-reducing hydrogenase, delta subunit
bin003	SOY3_bin003_00502	930	339	737	152	43.577	80.378	17.362	NAD-reducing hydrogenase HoxS subunit delta
bin003	SOY3_bin003_00503	1413	503	1074	280	42.557	77.093	21.050	NAD-reducing hydrogenase HoxS subunit beta
bin003	SOY3_bin003_00504	1197	340	768	161	33.957	65.076	14.288	Ferredoxin
bin003	SOY3_bin003_00505	174	22	26	6	15.115	15.156	3.663	hypothetical protein
bin003	SOY3_bin003_00506	276	3	9	1	1.299	3.307	0.385	Plasmid stabilisation system protein
bin003	SOY3_bin003_00507	477	7	40	5	1.754	8.505	1.113	Archaebacterial flagellin
bin003	SOY3_bin003_00508	1239	0	0	0	0.000	0.000	0.000	Pseudomurein endo-isopeptidase Pei
bin003	SOY3_bin003_00509	174	1	8	2	0.687	4.663	1.221	hypothetical protein
bin003	SOY3_bin003_00510	405	0	2	0	0.000	0.501	0.000	hypothetical protein
bin003	SOY3_bin003_00511	171	1	11	2	0.699	6.525	1.242	hypothetical protein
bin003	SOY3_bin003_00512	390	22	55	16	6.744	14.304	4.358	hypothetical protein
bin003	SOY3_bin003_00513	285	9	19	6	3.775	6.762	2.236	hypothetical protein
bin003	SOY3_bin003_00514	288	24	41	9	9.962	14.439	3.320	transcriptional regulator NrdR
bin003	SOY3_bin003_00515	1377	21	48	13	1.823	3.536	1.003	Multidrug resistance protein stp
bin003	SOY3_bin003_00516	240	3	5	7	1.494	2.113	3.098	Ferredoxin
bin003	SOY3_bin003_00517	984	16	29	6	1.944	2.989	0.648	Homoserine O-acetyltransferase
bin003	SOY3_bin003_00518	708	6	15	8	1.013	2.149	1.200	putative phycocyanin operon protein Z
bin003	SOY3_bin003_00519	438	8	22	2	2.184	5.095	0.485	hypothetical protein
bin003	SOY3_bin003_00520	384	3	7	1	0.934	1.849	0.277	4-oxalocrotonate tautomerase
bin003	SOY3_bin003_00521	597	22	31	7	4.405	5.267	1.246	Inner membrane protein YaaH
bin003	SOY3_bin003_00522	1902	52	72	15	3.268	3.840	0.838	Acetyl-coenzyme A synthetase
bin003	SOY3_bin003_00523	1644	135	364	80	9.817	22.457	5.169	60 kDa chaperonin
bin003	SOY3_bin003_00524	546	5	2	2	1.095	0.372	0.389	Peptide deformylase
bin003	SOY3_bin003_00525	1422	45	45	17	3.783	3.210	1.270	Cyclic 2,3-diphosphoglycerate synthetase
bin003	SOY3_bin003_00526	345	37	81	13	12.821	23.813	4.003	hypothetical protein
bin003	SOY3_bin003_00527	1290	26	26	11	2.410	2.044	0.906	Histidinol dehydrogenase
bin003	SOY3_bin003_00528	1320	28	64	9	2.536	4.918	0.724	Asparagine--tRNA ligase
bin003	SOY3_bin003_00529	618	40	60	7	7.738	9.847	1.203	hypothetical protein
bin003	SOY3_bin003_00530	795	25	35	11	3.759	4.465	1.470	2-amino-4,5-dihydroxy-6-one-heptanoic acid-7-phosphate synthase
bin003	SOY3_bin003_00531	1125	45	71	15	4.782	6.401	1.416	3-amino-4-hydroxybenzoic acid synthase
bin003	SOY3_bin003_00532	552	21	29	7	4.548	5.329	1.347	2'-5'-RNA ligase
bin003	SOY3_bin003_00533	1029	0	4	1	0.000	0.394	0.103	Putative oxidoreductase/MT0587
bin003	SOY3_bin003_00534	360	5	12	4	1.660	3.381	1.180	hypothetical protein
bin003	SOY3_bin003_00535	90	0	0	0	0.000	0.000	0.000	hypothetical protein
bin003	SOY3_bin003_00536	2106	65	63	21	3.690	3.034	1.059	Phytochrome-like protein cph1
bin003	SOY3_bin003_00537	972	32	49	12	3.936	5.113	1.311	High-affinity zinc uptake system binding-protein ZnuA precursor
bin003	SOY3_bin003_00538	747	13	21	1	2.081	2.851	0.142	High-affinity zinc uptake system ATP-binding protein ZnuC
bin003	SOY3_bin003_00539	795	9	12	5	1.353	1.531	0.668	Manganese transport system membrane protein MntB
bin003	SOY3_bin003_00540	372	0	5	0	0.000	1.363	0.000	hypothetical protein
bin003	SOY3_bin003_00541	291	2	2	2	0.822	0.697	0.730	hypothetical protein
bin003	SOY3_bin003_00542	186	0	1	0	0.000	0.545	0.000	hypothetical protein
bin003	SOY3_bin003_00543	2580	22	28	7	1.019	1.101	0.288	DEAD-box ATP-dependent RNA helicase CshB
bin003	SOY3_bin003_00544	738	7	7	4	1.134	0.962	0.576	Calcineurin-like phosphoesterase
bin003	SOY3_bin003_00545	975	2	3	2	0.245	0.312	0.218	Inner membrane protein YrbG
bin003	SOY3_bin003_00546	675	1	1	6	0.177	0.150	0.944	3-oxoadipate enol-lactonase 2
bin003	SOY3_bin003_00547	135	0	0	0	0.000	0.000	0.000	haloalkane dehalogenase
bin003	SOY3_bin003_00548	993	1	2	0	0.120	0.204	0.000	2-hydroxy-6-oxo-6-phenylhexa-2,4-dienoate hydrolase
bin003	SOY3_bin003_00549	432	1	0	1	0.277	0.000	0.246	hypothetical protein
bin003	SOY3_bin003_00550	423	5	9	3	1.413	2.158	0.753	Enamine/imine deaminase
bin003	SOY3_bin003_00551	462	8	10	3	2.070	2.195	0.690	hypothetical protein
bin003	SOY3_bin003_00552	477	8	7	1	2.005	1.488	0.223	3-demethylubiquinone-9 3-methyltransferase

bin003	SOY3_bin003_00553	540	6	5	1	1.328	0.939	0.197	hypothetical protein
bin003	SOY3_bin003_00554	495	1	4	1	0.242	0.820	0.215	hypothetical protein
bin003	SOY3_bin003_00555	504	7	7	0	1.660	1.409	0.000	hypothetical protein
bin003	SOY3_bin003_00556	813	17	59	7	2.500	7.361	0.915	SPBc2 prophage-derived aminoglycoside N(3')-acetyltransferase-like protein YokD
bin003	SOY3_bin003_00557	810	12	21	4	1.771	2.630	0.525	hypothetical protein
bin003	SOY3_bin003_00558	567	2	10	2	0.422	1.789	0.375	Phycocyanobilin lyase subunit alpha
bin003	SOY3_bin003_00559	234	2	7	0	1.022	3.034	0.000	hypothetical protein
bin003	SOY3_bin003_00560	399	4	2	0	1.198	0.508	0.000	hypothetical protein
bin003	SOY3_bin003_00561	1758	9	26	6	0.612	1.500	0.363	putative sensor histidine kinase pdtaS
bin003	SOY3_bin003_00562	573	0	6	2	0.000	1.062	0.371	RQC domain protein
bin003	SOY3_bin003_00563	753	5	18	3	0.794	2.425	0.423	hypothetical protein
bin003	SOY3_bin003_00564	1413	13	26	5	1.100	1.866	0.376	hypothetical protein
bin003	SOY3_bin003_00565	1263	30	51	15	2.840	4.096	1.262	ferredoxin-type protein
bin003	SOY3_bin003_00566	423	12	25	5	3.391	5.995	1.256	Double zinc ribbon
bin003	SOY3_bin003_00567	1143	204	767	181	21.337	68.062	16.821	Pseudomurein-binding repeat protein
bin003	SOY3_bin003_00568	1035	1	8	3	0.116	0.784	0.308	Pyruvate formate-lyase 1-activating enzyme
bin003	SOY3_bin003_00569	1305	20	34	15	1.832	2.643	1.221	peptidase PmbA
bin003	SOY3_bin003_00570	678	6	17	5	1.058	2.543	0.783	Putative phosphatase
bin003	SOY3_bin003_00571	1050	16	27	13	1.822	2.608	1.315	Hydrogenase expression/formation protein HypD
bin003	SOY3_bin003_00572	1233	12	54	22	1.163	4.442	1.895	UDP-N-acetylmuramoylalanine--D-glutamate ligase
bin003	SOY3_bin003_00573	1191	37	88	13	3.714	7.494	1.159	hypothetical protein
bin003	SOY3_bin003_00574	660	6	6	1	1.087	0.922	0.161	Bifunctional protein GlmU
bin003	SOY3_bin003_00575	840	10	17	1	1.423	2.053	0.126	Succinylglutamate desuccinylase / Aspartoacylase family protein
bin003	SOY3_bin003_00576	690	67	109	29	11.608	16.023	4.465	hypothetical protein
bin003	SOY3_bin003_00577	666	0	1	0	0.000	0.152	0.000	Fatty acid metabolism regulator protein
bin003	SOY3_bin003_00578	855	2	3	1	0.280	0.356	0.124	Ferredoxin
bin003	SOY3_bin003_00579	195	5	2	1	3.065	1.040	0.545	hypothetical protein
bin003	SOY3_bin003_00580	732	47	77	20	7.676	10.669	2.902	Heptaprenylglyceryl phosphate synthase
bin003	SOY3_bin003_00581	147	10	26	8	8.133	17.940	5.781	50S ribosomal protein L40e
bin003	SOY3_bin003_00582	510	29	69	21	6.798	13.723	4.374	hypothetical protein
bin003	SOY3_bin003_00583	690	6	10	2	1.040	1.470	0.308	hypothetical protein
bin003	SOY3_bin003_00584	585	9	15	6	1.839	2.601	1.089	Flavoredoxin
bin003	SOY3_bin003_00585	1998	44	76	19	2.633	3.858	1.010	Magnesium-chelatase 38 kDa subunit
bin003	SOY3_bin003_00586	135	13	29	3	11.512	21.788	2.361	DNA-directed RNA polymerase subunit P
bin003	SOY3_bin003_00587	474	39	62	19	9.836	13.267	4.258	hypothetical protein
bin003	SOY3_bin003_00588	705	31	27	10	5.257	3.884	1.507	hypothetical protein
bin003	SOY3_bin003_00589	732	6	4	1	0.980	0.554	0.145	Sulfite exporter TauE/SafE
bin003	SOY3_bin003_00590	210	3	0	0	1.708	0.000	0.000	hypothetical protein
bin003	SOY3_bin003_00591	198	2	1	0	1.208	0.512	0.000	Antitoxin HipB
bin003	SOY3_bin003_00592	372	1	2	0	0.321	0.545	0.000	hypothetical protein
bin003	SOY3_bin003_00593	468	6	3	1	1.533	0.650	0.227	hypothetical protein
bin003	SOY3_bin003_00594	453	3	1	0	0.792	0.224	0.000	hypothetical protein
bin003	SOY3_bin003_00595	753	1	4	7	0.159	0.539	0.987	Ribonuclease 3
bin003	SOY3_bin003_00596	1167	4	5	0	0.410	0.435	0.000	Tripartite tricarboxylate transporter TctA family protein
bin003	SOY3_bin003_00597	1089	2	11	2	0.220	1.025	0.195	ribosomal protein L11 methyltransferase
bin003	SOY3_bin003_00598	1620	100	172	42	7.380	10.769	2.754	Putative conjugal transfer protein/MT3759
bin003	SOY3_bin003_00599	1203	85	146	35	8.447	12.310	3.091	Bacterial type II secretion system protein F domain protein
bin003	SOY3_bin003_00600	2220	56	102	28	3.016	4.660	1.340	Copper-exporting P-type ATPase B
bin003	SOY3_bin003_00601	969	3	17	6	0.370	1.779	0.658	Energy-coupling factor transporter ATP-binding protein EcfA3
bin003	SOY3_bin003_00602	831	16	17	9	2.302	2.075	1.150	Nickel transport protein NikQ
bin003	SOY3_bin003_00603	300	70	236	49	27.895	79.789	17.350	cobalt transport protein CbiN
bin003	SOY3_bin003_00604	675	103	366	93	18.242	54.996	14.636	Fused nickel transport protein NikMN
bin003	SOY3_bin003_00605	390	6	5	1	1.839	1.300	0.272	hypothetical protein
bin003	SOY3_bin003_00606	1353	5	17	0	0.442	1.274	0.000	Antiseptic resistance protein
bin003	SOY3_bin003_00607	714	0	6	1	0.000	0.852	0.149	Cyclic pyranopterin monophosphate synthase
bin003	SOY3_bin003_00608	408	8	15	1	2.344	3.729	0.260	Putative nickel-responsive regulator
bin003	SOY3_bin003_00609	771	6	13	2	0.930	1.710	0.276	Benzylsuccinate synthase activating enzyme
bin003	SOY3_bin003_00610	1725	2	8	0	0.139	0.470	0.000	Putative multidrug export ATP-binding/permease protein
bin003	SOY3_bin003_00611	1743	2	8	2	0.137	0.466	0.122	Iron import ATP-binding/permease protein IrtA
bin003	SOY3_bin003_00612	1683	0	8	1	0.000	0.482	0.063	Putative HMP/thiamine import ATP-binding protein YkoD
bin003	SOY3_bin003_00613	786	1	4	0	0.152	0.516	0.000	Energy-coupling factor transporter transmembrane protein EcfT
bin003	SOY3_bin003_00614	558	4	14	6	0.857	2.545	1.142	hypothetical protein
bin003	SOY3_bin003_00615	561	9	12	6	1.918	2.170	1.136	hypothetical protein
bin003	SOY3_bin003_00616	846	6	5	0	0.848	0.599	0.000	prenyltransferase
bin003	SOY3_bin003_00617	144	2	3	0	1.660	2.113	0.000	hypothetical protein
bin003	SOY3_bin003_00618	393	0	4	0	0.000	1.032	0.000	Anaerobic glycerol-3-phosphate dehydrogenase subunit C
bin003	SOY3_bin003_00619	444	0	0	0	0.000	0.000	0.000	Methyl-viologen-reducing hydrogenase, delta subunit

bin003	SOY3_bin003_00620	1026	2	8	1	0.233	0.791	0.104	H(2)-dependent methylenetetrahydromethanopterin dehydrogenase-related protein
bin003	SOY3_bin003_00621	1506	1	9	2	0.079	0.606	0.141	Fibrillarin-like archaeal protein
bin003	SOY3_bin003_00622	1332	9	77	75	0.808	5.863	5.981	hypothetical protein
bin003	SOY3_bin003_00623	489	0	3	4	0.000	0.622	0.869	Methyl-coenzyme M reductase operon protein D
bin003	SOY3_bin003_00624	801	2	29	19	0.298	3.672	2.520	Methyl-coenzyme M reductase gamma subunit
bin003	SOY3_bin003_00625	1656	5	71	52	0.361	4.349	3.336	hypothetical protein
bin003	SOY3_bin003_00626	693	9	17	4	1.553	2.488	0.613	HTH-type transcriptional repressor Bm3R1
bin003	SOY3_bin003_00627	1074	14	21	7	1.558	1.983	0.692	Pyruvate formate-lyase-activating enzyme
bin003	SOY3_bin003_00628	510	1	7	1	0.234	1.392	0.208	Acetyltransferase (GNAT) family protein
bin003	SOY3_bin003_00629	210	7	15	3	3.985	7.245	1.518	hypothetical protein
bin003	SOY3_bin003_00630	333	8	23	1	2.872	7.005	0.319	flagellar basal body-associated protein FliL
bin003	SOY3_bin003_00631	675	35	57	10	6.199	8.565	1.574	flagellar motor protein MotA
bin003	SOY3_bin003_00632	714	14	37	17	2.344	5.256	2.529	hypothetical protein
bin003	SOY3_bin003_00633	4629	2	11	9	0.052	0.241	0.207	Aerobic cobaltochelatase subunit CobN
bin003	SOY3_bin003_00634	4617	6	13	4	0.155	0.286	0.092	Aerobic cobaltochelatase subunit CobN
bin003	SOY3_bin003_00635	786	8	25	11	1.217	3.226	1.487	DNA-binding transcriptional activator MhpR
bin003	SOY3_bin003_00636	90	0	2	0	0.000	2.254	0.000	hypothetical protein
bin003	SOY3_bin003_00637	1983	31	251	42	1.869	12.838	2.250	Phosphoribosylformylglycinamidine synthase 1
bin003	SOY3_bin003_00638	1719	69	118	29	4.799	6.962	1.792	2-oxoglutarate carboxylase large subunit
bin003	SOY3_bin003_00639	1602	8	20	6	0.597	1.266	0.398	hypothetical protein
bin003	SOY3_bin003_00640	828	0	0	0	0.000	0.000	0.000	Nitrogenase iron protein 1
bin003	SOY3_bin003_00641	318	1	0	0	0.376	0.000	0.000	Nitrogen regulatory protein P-II
bin003	SOY3_bin003_00642	369	0	0	0	0.000	0.000	0.000	Nitrogen regulatory protein P-II
bin003	SOY3_bin003_00643	1461	1	0	1	0.082	0.000	0.073	Nitrogenase vanadium-iron protein alpha chain
bin003	SOY3_bin003_00644	1386	1	1	1	0.086	0.073	0.077	Nitrogenase iron-iron protein beta chain
bin003	SOY3_bin003_00645	1371	1	4	0	0.087	0.296	0.000	Nitrogenase molybdenum-iron protein alpha chain
bin003	SOY3_bin003_00646	1419	2	0	0	0.168	0.000	0.000	Nitrogenase molybdenum-iron protein beta chain
bin003	SOY3_bin003_00647	321	0	0	1	0.000	0.000	0.331	FeMo cofactor biosynthesis protein NifB
bin003	SOY3_bin003_00648	297	0	0	0	0.000	0.000	0.000	Ferredoxin-3
bin003	SOY3_bin003_00649	1962	0	4	0	0.000	0.207	0.000	Dimethyl sulfoxide/trimethylamine N-oxide reductase precursor
bin003	SOY3_bin003_00650	813	9	30	5	1.323	3.743	0.653	Caspase domain protein
bin003	SOY3_bin003_00651	1104	54	78	30	5.847	7.166	2.887	Inositol-3-phosphate synthase
bin003	SOY3_bin003_00652	441	2	3	2	0.542	0.690	0.482	SNARE associated Golgi protein
bin003	SOY3_bin003_00653	294	3	7	1	1.220	2.415	0.361	hypothetical protein
bin003	SOY3_bin003_00654	300	3	3	1	1.195	1.014	0.354	hypothetical protein
bin003	SOY3_bin003_00655	288	3	5	1	1.245	1.761	0.369	Bacterial regulatory protein, arsR family
bin003	SOY3_bin003_00656	306	1	1	1	0.391	0.331	0.347	hypothetical protein
bin003	SOY3_bin003_00657	915	5	14	0	0.653	1.552	0.000	prenyltransferase
bin003	SOY3_bin003_00658	1086	1	3	0	0.110	0.280	0.000	putative sensor histidine kinase pdtaS
bin003	SOY3_bin003_00659	1203	3	3	1	0.298	0.253	0.088	Transposase IS66 family protein
bin003	SOY3_bin003_00660	570	22	32	9	4.614	5.694	1.677	exosome complex RNA-binding protein Csl4
bin003	SOY3_bin003_00661	306	9	19	1	3.516	6.298	0.347	DNA-directed RNA polymerase subunit L
bin003	SOY3_bin003_00662	609	10	27	4	1.963	4.497	0.698	hypothetical protein
bin003	SOY3_bin003_00663	408	12	23	2	3.516	5.718	0.521	Bifunctional NMN adenylyltransferase/Nudix hydrolase
bin003	SOY3_bin003_00664	318	11	30	6	4.135	9.569	2.004	30S ribosomal protein S27ae
bin003	SOY3_bin003_00665	834	40	53	19	5.734	6.446	2.420	hypothetical protein
bin003	SOY3_bin003_00666	735	73	120	31	11.874	16.560	4.480	DNA polymerase sliding clamp
bin003	SOY3_bin003_00667	1056	66	107	29	7.472	10.277	2.917	hypothetical protein
bin003	SOY3_bin003_00668	195	6	11	2	3.678	5.722	1.089	Putative transposase DNA-binding domain protein
bin003	SOY3_bin003_00669	561	2	4	0	0.426	0.723	0.000	putative transposase
bin003	SOY3_bin003_00670	282	27	50	19	11.446	17.984	7.157	50S ribosomal protein L44e
bin003	SOY3_bin003_00671	186	21	39	17	13.497	21.267	9.709	30S ribosomal protein S27e
bin003	SOY3_bin003_00672	780	75	115	24	11.495	14.954	3.268	Polyribonucleotide nucleotidyltransferase
bin003	SOY3_bin003_00673	180	13	28	2	8.634	15.778	1.180	H/ACA RNA-protein complex component Nop10p
bin003	SOY3_bin003_00674	774	61	88	18	9.422	11.532	2.470	PAC2 family protein
bin003	SOY3_bin003_00675	1218	8	12	4	0.785	0.999	0.349	PHP domain protein
bin003	SOY3_bin003_00676	123	7	4	3	6.804	3.298	2.591	hypothetical protein
bin003	SOY3_bin003_00677	76	1	1	1	1.573	1.335	1.398	tRNA-Arg(cct)
bin003	SOY3_bin003_00678	76	2	2	2	3.146	2.669	2.795	tRNA-Arg(tct)
bin003	SOY3_bin003_00679	720	37	62	13	6.143	8.734	1.918	PAC2 family protein
bin003	SOY3_bin003_00680	1218	474	966	273	46.524	80.442	23.809	NAD-reducing hydrogenase HoxS subunit beta
bin003	SOY3_bin003_00681	471	175	414	92	44.418	89.153	20.749	Hydrogenase 2 maturation protease
bin003	SOY3_bin003_00682	825	393	748	176	56.949	91.961	22.661	NAD-reducing hydrogenase HoxS subunit delta
bin003	SOY3_bin003_00683	885	419	810	182	56.600	92.832	21.845	coenzyme F420-reducing hydrogenase subunit beta
bin003	SOY3_bin003_00684	933	21	33	11	2.691	3.587	1.252	Methionine aminopeptidase
bin003	SOY3_bin003_00685	294	10	25	9	4.066	8.625	3.252	hypothetical protein
bin003	SOY3_bin003_00686	465	44	83	24	11.312	18.104	5.483	hypothetical protein

bin003	SOY3_bin003_00687	579	36	88	17	7.433	15.416	3.119	Poly(hydroxyalcanoate) granule associated protein (phasin)
bin003	SOY3_bin003_00688	75	0	2	1	0.000	2.705	1.416	tRNA-Met(cat)
bin003	SOY3_bin003_00689	798	16	16	3	2.397	2.034	0.399	Sulfite exporter TauE/SafE
bin003	SOY3_bin003_00690	1161	67	74	19	6.899	6.465	1.738	Chaperone protein DnaJ
bin003	SOY3_bin003_00691	402	0	0	0	0.000	0.000	0.000	Transcriptional regulator SlyA
bin003	SOY3_bin003_00692	405	1	4	1	0.295	1.002	0.262	hypothetical protein
bin003	SOY3_bin003_00693	672	18	7	2	3.202	1.057	0.316	hypothetical protein
bin003	SOY3_bin003_00694	336	9	5	2	3.202	1.509	0.632	hypothetical protein
bin003	SOY3_bin003_00695	276	23	25	5	9.962	9.187	1.924	DNA/RNA-binding protein albA
bin003	SOY3_bin003_00696	234	241	213	41	123.125	92.325	18.612	23S rRNA (uracil-C(5))-methyltransferase RlmCD
bin003	SOY3_bin003_00697	660	18	10	2	3.260	1.537	0.322	nitroreductase A
bin003	SOY3_bin003_00698	225	0	2	1	0.000	0.902	0.472	Histone-like transcription factor (CBF/NF-Y) and archaeal histone
bin003	SOY3_bin003_00699	2274	21	29	10	1.104	1.293	0.467	Phytochrome-like protein cph1
bin003	SOY3_bin003_00700	2163	103	119	44	5.693	5.580	2.161	Catalase-peroxidase
bin003	SOY3_bin003_00701	393	2	1	1	0.608	0.258	0.270	Peroxide-responsive repressor PerR
bin003	SOY3_bin003_00702	411	6	22	2	1.745	5.429	0.517	hypothetical protein
bin003	SOY3_bin003_00703	1152	22	41	12	2.283	3.610	1.107	hypothetical protein
bin003	SOY3_bin003_00704	561	15	27	8	3.196	4.882	1.515	V-type ATP synthase subunit D
bin003	SOY3_bin003_00705	561	5	5	0	1.065	0.904	0.000	hypothetical protein
bin003	SOY3_bin003_00706	999	8	20	6	0.957	2.031	0.638	Magnesium transporter MgtE
bin003	SOY3_bin003_00707	546	1	3	1	0.219	0.557	0.195	hypothetical protein
bin003	SOY3_bin003_00708	375	0	2	0	0.000	0.541	0.000	hypothetical protein
bin003	SOY3_bin003_00709	1917	6	24	3	0.374	1.270	0.166	DNA mismatch repair protein MutS
bin003	SOY3_bin003_00710	2634	15	19	5	0.681	0.732	0.202	putative protease YhbU precursor
bin003	SOY3_bin003_00711	390	9	9	2	2.759	2.341	0.545	Cell cycle response regulator CtrA
bin003	SOY3_bin003_00712	840	5	18	5	0.712	2.173	0.632	Protoheme IX farnesyltransferase
bin003	SOY3_bin003_00713	540	6	7	2	1.328	1.315	0.393	Histone methylation protein DOT1
bin003	SOY3_bin003_00714	246	0	1	0	0.000	0.412	0.000	hypothetical protein
bin003	SOY3_bin003_00715	1410	76	62	10	6.444	4.460	0.753	Multidrug export protein MepA
bin003	SOY3_bin003_00716	819	25	11	3	3.649	1.362	0.389	Ferredoxin-1
bin003	SOY3_bin003_00717	1107	13	31	9	1.404	2.840	0.864	Sporulation kinase E
bin003	SOY3_bin003_00718	447	0	3	0	0.000	0.681	0.000	Putative universal stress protein
bin003	SOY3_bin003_00719	1329	5	12	4	0.450	0.916	0.320	Sodium-dependent dicarboxylate transporter SdcS
bin003	SOY3_bin003_00720	270	3	4	0	1.328	1.503	0.000	hypothetical protein
bin003	SOY3_bin003_00721	219	2	3	0	1.092	1.389	0.000	Putative transposase DNA-binding domain protein
bin003	SOY3_bin003_00722	1572	15	33	4	1.141	2.129	0.270	putative sensor histidine kinase pdtaS
bin003	SOY3_bin003_00723	87	9	19	6	12.367	22.151	7.326	tRNA-Ser(cga)
bin003	SOY3_bin003_00724	297	36	74	19	14.491	25.271	6.796	Putative monooxygenase YcnE
bin003	SOY3_bin003_00725	597	19	29	7	3.805	4.927	1.246	HTH-type transcriptional repressor NemR
bin003	SOY3_bin003_00726	1056	715	1300	220	80.945	124.863	22.130	NADP-dependent isopropanol dehydrogenase
bin003	SOY3_bin003_00727	687	221	337	86	38.458	49.754	13.298	prephenate dehydrogenase
bin003	SOY3_bin003_00728	888	81	67	19	10.905	7.653	2.273	L-aspartate dehydrogenase
bin003	SOY3_bin003_00729	2085	30	32	8	1.720	1.557	0.408	Succinyl-CoA ligase [ADP-forming] subunit alpha
bin003	SOY3_bin003_00730	570	11	21	1	2.307	3.737	0.186	hypothetical protein
bin003	SOY3_bin003_00731	585	22	70	15	4.496	12.137	2.724	Rubrerythrin-1
bin003	SOY3_bin003_00732	930	4	4	1	0.514	0.436	0.114	Inner membrane protein YrbG
bin003	SOY3_bin003_00733	279	5	12	3	2.142	4.362	1.142	transcriptional regulator NrdR
bin003	SOY3_bin003_00734	477	1	5	1	0.251	1.063	0.223	hypothetical protein
bin003	SOY3_bin003_00735	768	5	15	2	0.778	1.981	0.277	hypothetical protein
bin003	SOY3_bin003_00736	468	2	23	7	0.511	4.985	1.589	DNA gyrase inhibitor
bin003	SOY3_bin003_00737	1137	22	65	10	2.313	5.798	0.934	putative cysteine desulfurase
bin003	SOY3_bin003_00738	639	4	7	6	0.748	1.111	0.997	Error-prone DNA polymerase
bin003	SOY3_bin003_00739	975	2	12	1	0.245	1.248	0.109	peptide chain release factor 1
bin003	SOY3_bin003_00740	927	1	8	1	0.129	0.875	0.115	hypothetical protein
bin003	SOY3_bin003_00741	1140	0	1	2	0.000	0.089	0.186	Inner membrane transport permease YbhR
bin003	SOY3_bin003_00742	801	3	3	1	0.448	0.380	0.133	putative ABC transporter ATP-binding protein YbhF
bin003	SOY3_bin003_00743	834	4	11	0	0.573	1.338	0.000	HTH-type transcriptional repressor NsrR
bin003	SOY3_bin003_00744	1044	22	59	8	2.519	5.732	0.814	UDP-N-acetylglucosamine--N-acetylmuramyl-(pentapeptide) pyrophosphoryl-undecaprenol N-acetylglucosamine transferase
bin003	SOY3_bin003_00745	1317	21	17	5	1.906	1.309	0.403	Dihydrolipoyl dehydrogenase
bin003	SOY3_bin003_00746	231	15	17	5	7.763	7.464	2.299	Hydrogenase isoenzymes formation protein HypC
bin003	SOY3_bin003_00747	1680	7	28	5	0.498	1.690	0.316	putative sensor histidine kinase pdtaS
bin003	SOY3_bin003_00748	504	1	4	2	0.237	0.805	0.422	ACT domain protein
bin003	SOY3_bin003_00749	1356	2	19	10	0.176	1.421	0.783	Anthranilate synthase component 1
bin003	SOY3_bin003_00750	576	1	5	2	0.208	0.880	0.369	Aminodeoxychorismate/anthranilate synthase component 2
bin003	SOY3_bin003_00751	807	2	17	4	0.296	2.137	0.527	Indole-3-glycerol phosphate synthase
bin003	SOY3_bin003_00752	660	0	9	2	0.000	1.383	0.322	N-(5'-phosphoribosyl)anthranilate isomerase
bin003	SOY3_bin003_00753	1182	0	19	8	0.000	1.630	0.719	Tryptophan synthase beta chain

bin003	SOY3_bin003_00754	816	0	7	1	0.000	0.870	0.130	Tryptophan synthase alpha chain
bin003	SOY3_bin003_00755	1050	0	18	3	0.000	1.739	0.304	Anthranilate phosphoribosyltransferase
bin003	SOY3_bin003_00756	75	1	2	0	1.594	2.705	0.000	tRNA-Ile(gat)
bin003	SOY3_bin003_00757	540	48	106	20	10.627	19.910	3.934	adenylate kinase
bin003	SOY3_bin003_00758	291	26	52	21	10.681	18.124	7.666	hypothetical protein
bin003	SOY3_bin003_00759	915	7	31	10	0.915	3.436	1.161	PUA domain protein
bin003	SOY3_bin003_00760	1494	19	21	3	1.520	1.426	0.213	Glutamate synthase [NADPH] large chain precursor
bin003	SOY3_bin003_00761	429	17	35	7	4.737	8.275	1.733	Pyridoxamine 5'-phosphate oxidase
bin003	SOY3_bin003_00762	1458	18	40	12	1.476	2.783	0.874	Bifunctional NAD(P)H-hydrate repair enzyme Nnr
bin003	SOY3_bin003_00763	1941	2	10	6	0.123	0.523	0.328	Voltage-gated potassium channel Kch
bin003	SOY3_bin003_00764	897	39	120	26	5.198	13.569	3.079	Formyltransferase/hydrolase complex subunit D
bin003	SOY3_bin003_00765	396	11	48	12	3.321	12.294	3.219	Pyridoxamine 5'-phosphate oxidase
bin003	SOY3_bin003_00766	1026	6	27	3	0.699	2.669	0.311	Phosphatidylglycerol lysyltransferase
bin003	SOY3_bin003_00767	384	112	143	40	34.868	37.771	11.065	lipase chaperone
bin003	SOY3_bin003_00768	471	3	8	6	0.761	1.723	1.353	Phosphodiesterase YfcE
bin003	SOY3_bin003_00769	567	7	2	0	1.476	0.358	0.000	Peptidase C39 family protein
bin003	SOY3_bin003_00770	789	18	36	5	2.727	4.628	0.673	Modulator of FtsH protease HflK
bin003	SOY3_bin003_00771	1938	16	20	10	0.987	1.047	0.548	Inner membrane protein YbaL
bin003	SOY3_bin003_00772	618	17	28	10	3.289	4.595	1.719	putative polyketide biosynthesis zinc-dependent hydrolase PksB
bin003	SOY3_bin003_00773	72	0	0	0	0.000	0.000	0.000	tRNA-Cys(gca)
bin003	SOY3_bin003_00774	390	46	72	25	14.101	18.725	6.809	hypothetical protein
bin003	SOY3_bin003_00775	804	1	7	0	0.149	0.883	0.000	Demethylrebeccamycin-D-glucose O-methyltransferase
bin003	SOY3_bin003_00776	744	5	7	1	0.803	0.954	0.143	Phycocyanobilin lyase subunit alpha
bin003	SOY3_bin003_00777	576	0	3	1	0.000	0.528	0.184	hypothetical protein
bin003	SOY3_bin003_00778	567	0	0	0	0.000	0.000	0.000	putative methyltransferase YcgJ
bin003	SOY3_bin003_00779	636	1	1	0	0.188	0.159	0.000	Putative O-methyltransferase/MSMEI_4947
bin003	SOY3_bin003_00780	465	3	2	0	0.771	0.436	0.000	Epoxyqueuosine reductase
bin003	SOY3_bin003_00781	318	1	8	0	0.376	2.552	0.000	SnoaL-like domain protein
bin003	SOY3_bin003_00782	1185	11	14	2	1.110	1.198	0.179	Inner membrane transport permease YbhR
bin003	SOY3_bin003_00783	1017	6	11	6	0.705	1.097	0.627	Daunorubicin/doxorubicin resistance ATP-binding protein DrrA
bin003	SOY3_bin003_00784	525	2	9	0	0.455	1.739	0.000	lineage-specific thermal regulator protein
bin003	SOY3_bin003_00785	1473	55	121	24	4.464	8.332	1.731	hypothetical protein
bin003	SOY3_bin003_00786	1824	6	11	0	0.393	0.612	0.000	hypothetical protein
bin003	SOY3_bin003_00787	1488	17	14	7	1.366	0.954	0.500	Trk system potassium uptake protein TrkG
bin003	SOY3_bin003_00788	243	11	7	0	5.412	2.922	0.000	hypothetical protein
bin003	SOY3_bin003_00789	1008	81	116	31	9.607	11.672	3.267	Arsenical pump-driving ATPase
bin003	SOY3_bin003_00790	813	34	50	14	5.000	6.238	1.829	NH(3)-dependent NAD(+) synthetase
bin003	SOY3_bin003_00791	312	7	7	2	2.682	2.276	0.681	Divalent-cation tolerance protein CutA
bin003	SOY3_bin003_00792	2865	88	114	31	3.672	4.036	1.149	Valine--tRNA ligase
bin003	SOY3_bin003_00793	288	9	30	1	3.736	10.565	0.369	2-nonaprenyl-3-methyl-6-methoxy-1,4-benzoquinol hydroxylase
bin003	SOY3_bin003_00794	561	8	10	1	1.705	1.808	0.189	hypothetical protein
bin003	SOY3_bin003_00795	1251	20	23	2	1.911	1.865	0.170	putative transporter YycB
bin003	SOY3_bin003_00796	780	17	14	6	2.606	1.820	0.817	Ureidoglycolate lyase
bin003	SOY3_bin003_00797	861	20	25	6	2.777	2.945	0.740	ATP phosphoribosyltransferase
bin003	SOY3_bin003_00798	1503	21	45	11	1.670	3.037	0.777	threonine dehydratase
bin003	SOY3_bin003_00799	1293	11	42	4	1.017	3.295	0.329	5-methylthioadenosine/S-adenosylhomocysteine deaminase
bin003	SOY3_bin003_00800	690	7	23	5	1.213	3.381	0.770	Pyrophosphatase PpaX
bin003	SOY3_bin003_00801	759	2	9	0	0.315	1.203	0.000	Inner membrane transport permease YbhS
bin003	SOY3_bin003_00802	783	0	3	1	0.000	0.389	0.136	Daunorubicin/doxorubicin resistance ATP-binding protein DrrA
bin003	SOY3_bin003_00803	183	2	3	1	1.307	1.663	0.580	hypothetical protein
bin003	SOY3_bin003_00804	186	1	2	0	0.643	1.091	0.000	hypothetical protein
bin003	SOY3_bin003_00805	990	4	24	4	0.483	2.459	0.429	ATP phosphoribosyltransferase
bin003	SOY3_bin003_00806	672	2	6	2	0.356	0.906	0.316	Demethylrebeccamycin-D-glucose O-methyltransferase
bin003	SOY3_bin003_00807	1413	169	421	89	14.298	30.220	6.691	Pseudomurein-binding repeat protein
bin003	SOY3_bin003_00808	807	13	43	15	1.926	5.404	1.974	Septum site-determining protein MinD
bin003	SOY3_bin003_00809	567	2	12	1	0.422	2.147	0.187	hypothetical protein
bin003	SOY3_bin003_00810	459	3	25	3	0.781	5.524	0.694	hypothetical protein
bin003	SOY3_bin003_00811	372	3	14	2	0.964	3.817	0.571	Proteasome-associated ATPase
bin003	SOY3_bin003_00812	1269	54	301	62	5.087	24.058	5.190	phosphoribosylformylglycinamidine synthase I
bin003	SOY3_bin003_00813	579	6	7	0	1.239	1.226	0.000	Histone methylation protein DOT1
bin003	SOY3_bin003_00814	303	4	5	1	1.578	1.674	0.351	hypothetical protein
bin003	SOY3_bin003_00815	870	23	31	7	3.160	3.614	0.855	putative transcriptional regulatory protein pdtaR
bin003	SOY3_bin003_00816	450	9	17	5	2.391	3.832	1.180	hypothetical protein
bin003	SOY3_bin003_00817	447	76	107	19	20.326	24.279	4.515	18 kDa heat shock protein
bin003	SOY3_bin003_00818	498	22	36	10	5.281	7.332	2.133	NYN domain protein
bin003	SOY3_bin003_00819	264	5	7	0	2.264	2.689	0.000	Double zinc ribbon
bin003	SOY3_bin003_00820	1239	64	108	25	6.175	8.841	2.143	LL-diaminopimelate aminotransferase

bin003	SOY3_bin003_00821	624	112	244	57	21.457	39.661	9.703	hypothetical protein
bin003	SOY3_bin003_00822	4860	10	33	6	0.246	0.689	0.131	hypothetical protein
bin003	SOY3_bin003_00823	678	8	16	4	1.411	2.394	0.627	fibrillarin
bin003	SOY3_bin003_00824	867	2	11	1	0.276	1.287	0.123	CAAX amino terminal protease self- immunity
bin003	SOY3_bin003_00825	1203	5	15	4	0.497	1.265	0.353	Putative snoRNA binding domain protein
bin003	SOY3_bin003_00826	906	24	28	5	3.167	3.135	0.586	Dihydroorotate dehydrogenase B (NAD(+)), catalytic subunit
bin003	SOY3_bin003_00827	795	6	19	10	0.902	2.424	1.336	Dihydroorotate dehydrogenase B (NAD(+)), electron transfer subunit
bin003	SOY3_bin003_00828	411	7	12	2	2.036	2.961	0.517	Archaeal holliday junction resolvase (hjc)
bin003	SOY3_bin003_00829	330	7	19	5	2.536	5.840	1.609	hypothetical protein
bin003	SOY3_bin003_00830	73	0	3	1	0.000	4.168	1.455	tRNA-His(gtg)
bin003	SOY3_bin003_00831	88	2	9	4	2.717	10.373	4.828	tRNA-Leu(tag)
bin003	SOY3_bin003_00832	77	9	5	8	13.973	6.586	11.036	tRNA-Glu(ttc)
bin003	SOY3_bin003_00833	75	2	18	6	3.188	24.343	8.498	tRNA-Met(cat)
bin003	SOY3_bin003_00834	75	11	12	10	17.534	16.228	14.163	tRNA-Asn(gtt)
bin003	SOY3_bin003_00835	1032	9	38	7	1.043	3.735	0.721	Biotin synthase
bin003	SOY3_bin003_00836	84	1	1	2	1.423	1.207	2.529	tRNA-Ser(cag)
bin003	SOY3_bin003_00837	549	3	2	1	0.653	0.369	0.193	HTH-type transcriptional regulator AcrR
bin003	SOY3_bin003_00838	1356	21	53	13	1.851	3.964	1.018	Aspartyl/glutamyl-tRNA(Asn/Gln) amidotransferase subunit B
bin003	SOY3_bin003_00839	237	7	11	2	3.531	4.708	0.896	hypothetical protein
bin003	SOY3_bin003_00840	798	15	23	3	2.247	2.923	0.399	Inosine-5'-monophosphate dehydrogenase
bin003	SOY3_bin003_00841	291	6	2	2	2.465	0.697	0.730	Phosphoribosyl-ATP pyrophosphatase
bin003	SOY3_bin003_00842	540	6	25	2	1.328	4.696	0.393	Acetolactate synthase isozyme 1 large subunit
bin003	SOY3_bin003_00843	492	19	17	8	4.617	3.505	1.727	acetolactate synthase 1 catalytic subunit
bin003	SOY3_bin003_00844	339	1	2	1	0.353	0.598	0.313	hypothetical protein
bin003	SOY3_bin003_00845	1191	3	11	2	0.301	0.937	0.178	hypothetical protein
bin003	SOY3_bin003_00846	456	0	3	3	0.000	0.667	0.699	tRNA-specific adenosine deaminase
bin003	SOY3_bin003_00847	2307	225	325	84	11.660	14.289	3.868	Anaerobic ribonucleoside-triphosphate reductase
bin003	SOY3_bin003_00848	3264	80	110	15	2.930	3.418	0.488	DNA polymerase II large subunit
bin003	SOY3_bin003_00849	162	5	6	5	3.690	3.757	3.279	preprotein translocase subunit SecG
bin003	SOY3_bin003_00850	1353	68	75	19	6.008	5.622	1.492	Adenylosuccinate lyase
bin003	SOY3_bin003_00851	921	95	198	55	12.331	21.805	6.344	hypothetical protein
bin003	SOY3_bin003_00852	408	1	2	0	0.293	0.497	0.000	hypothetical protein
bin003	SOY3_bin003_00853	417	7	12	2	2.007	2.919	0.509	hypothetical protein
bin003	SOY3_bin003_00854	378	17	55	15	5.377	14.758	4.215	hypothetical protein
bin003	SOY3_bin003_00855	1233	40	40	10	3.878	3.290	0.862	Imidazolonepropionase
bin003	SOY3_bin003_00856	342	5	5	2	1.748	1.483	0.621	HTH-type transcriptional activator HxlR
bin003	SOY3_bin003_00857	2448	48	50	8	2.344	2.072	0.347	Copper-exporting P-type ATPase A
bin003	SOY3_bin003_00858	150	6	4	2	4.782	2.705	1.416	Copper-transporting P-type ATPase
bin003	SOY3_bin003_00859	153	5	7	1	3.907	4.640	0.694	hypothetical protein
bin003	SOY3_bin003_00860	1584	32	69	11	2.415	4.418	0.738	Lysine--tRNA ligase
bin003	SOY3_bin003_00861	273	9	7	4	3.941	2.601	1.556	hypothetical protein
bin003	SOY3_bin003_00862	489	28	49	7	6.845	10.163	1.521	putative monovalent cation/H+ antiporter subunit B
bin003	SOY3_bin003_00863	279	11	12	5	4.713	4.362	1.904	putative monovalent cation/H+ antiporter subunit B
bin003	SOY3_bin003_00864	339	11	11	6	3.879	3.291	1.880	hypothetical protein
bin003	SOY3_bin003_00865	1500	65	102	12	5.180	6.897	0.850	Na(+)/H(+) antiporter subunit D
bin003	SOY3_bin003_00866	372	10	12	3	3.214	3.272	0.857	Na(+)/H(+) antiporter subunit C1
bin003	SOY3_bin003_00867	243	6	12	2	2.952	5.009	0.874	hypothetical protein
bin003	SOY3_bin003_00868	288	12	18	5	4.981	6.339	1.844	putative monovalent cation/H+ antiporter subunit G
bin003	SOY3_bin003_00869	261	21	8	1	9.619	3.109	0.407	putative monovalent cation/H+ antiporter subunit F
bin003	SOY3_bin003_00870	312	37	32	9	14.177	10.403	3.064	Na(+)/H(+) antiporter subunit E
bin003	SOY3_bin003_00871	669	5	0	0	0.893	0.000	0.000	inner membrane protein
bin003	SOY3_bin003_00872	729	22	33	13	3.608	4.591	1.894	succinylglutamate desuccinylase
bin003	SOY3_bin003_00873	1182	47	80	21	4.754	6.865	1.887	Argininosuccinate synthase
bin003	SOY3_bin003_00874	681	2	1	1	0.351	0.149	0.156	HTH-type transcriptional repressor Bm3R1
bin003	SOY3_bin003_00875	1032	2	4	2	0.232	0.393	0.206	HTH-type transcriptional repressor KstR
bin003	SOY3_bin003_00876	489	6	13	4	1.467	2.696	0.869	hypothetical protein
bin003	SOY3_bin003_00877	726	7	26	1	1.153	3.632	0.146	Iron-sulfur flavoprotein
bin003	SOY3_bin003_00878	549	1	0	0	0.218	0.000	0.000	Chaperone protein DnaJ
bin003	SOY3_bin003_00879	1140	1	5	0	0.105	0.445	0.000	Chaperone protein DnaJ
bin003	SOY3_bin003_00880	393	0	0	0	0.000	0.000	0.000	hypothetical protein
bin003	SOY3_bin003_00881	396	12	27	6	3.623	6.915	1.609	Peptidase propeptide and YPEB domain protein
bin003	SOY3_bin003_00882	2274	6	8	1	0.315	0.357	0.047	Copper resistance protein CopC
bin003	SOY3_bin003_00883	1518	39	58	16	3.071	3.875	1.120	2-isopropylmalate synthase
bin003	SOY3_bin003_00884	804	4	28	7	0.595	3.532	0.925	hypothetical protein
bin003	SOY3_bin003_00885	651	10	33	5	1.836	5.141	0.816	Error-prone DNA polymerase
bin003	SOY3_bin003_00886	1026	25	34	5	2.913	3.361	0.518	hypothetical protein
bin003	SOY3_bin003_00887	1293	17	76	14	1.572	5.962	1.150	Tryptophan synthase beta chain

bin003	SOY3_bin003_00888	375	103	354	85	32.836	95.747	24.078	chaperone protein DnaJ
bin003	SOY3_bin003_00889	1170	261	918	178	26.669	79.581	16.161	Pseudomurein-binding repeat protein
bin003	SOY3_bin003_00890	1242	75	143	31	7.219	11.678	2.651	Formaldehyde-activating enzyme
bin003	SOY3_bin003_00891	597	6	7	3	1.201	1.189	0.534	2-amino-4-deoxychorismate dehydrogenase
bin003	SOY3_bin003_00892	228	3	4	2	1.573	1.779	0.932	Ferredoxin-2
bin003	SOY3_bin003_00893	579	9	22	5	1.858	3.854	0.917	Imidazoleglycerol-phosphate dehydratase
bin003	SOY3_bin003_00894	630	6	13	2	1.139	2.093	0.337	hypothetical protein
bin003	SOY3_bin003_00895	831	140	359	91	20.141	43.818	11.632	F420-dependent methylenetetrahydromethanopterin dehydrogenase
bin003	SOY3_bin003_00896	1179	5	14	1	0.507	1.204	0.090	hypothetical protein
bin003	SOY3_bin003_00897	1350	22	41	9	1.948	3.080	0.708	Cobyrinic acid A,C-diamide synthase
bin003	SOY3_bin003_00898	1128	21	27	7	2.226	2.428	0.659	biotin synthase
bin003	SOY3_bin003_00899	429	5	12	3	1.393	2.837	0.743	Poxvirus Late Transcription Factor VLTF3 like protein
bin003	SOY3_bin003_00900	315	1	6	0	0.380	1.932	0.000	Sporulation initiation inhibitor protein Soj
bin003	SOY3_bin003_00901	1605	11	18	5	0.819	1.138	0.331	hypothetical protein
bin003	SOY3_bin003_00902	423	4	1	0	1.130	0.240	0.000	GtrA-like protein
bin003	SOY3_bin003_00903	1605	4	9	1	0.298	0.569	0.066	hypothetical protein
bin003	SOY3_bin003_00904	927	20	47	10	2.579	5.142	1.146	GMP synthase [glutamine-hydrolyzing]
bin003	SOY3_bin003_00905	432	3	1	0	0.830	0.235	0.000	Pyridoxamine 5'-phosphate oxidase
bin003	SOY3_bin003_00906	726	8	16	6	1.317	2.235	0.878	Glycine/sarcosine/dimethylglycine N-methyltransferase
bin003	SOY3_bin003_00907	630	6	16	3	1.139	2.576	0.506	putative metallo-hydrolase YflN
bin003	SOY3_bin003_00908	1272	9	12	12	0.846	0.957	1.002	HTH-type transcriptional repressor KstR2
bin003	SOY3_bin003_00909	456	20	24	2	5.243	5.338	0.466	Peptide methionine sulfoxide reductase MsrB
bin003	SOY3_bin003_00910	861	33	33	11	4.582	3.887	1.357	L-aspartate dehydrogenase
bin003	SOY3_bin003_00911	681	51	74	17	8.953	11.021	2.652	prephenate dehydrogenase
bin003	SOY3_bin003_00912	768	126	175	41	19.613	23.112	5.671	Gluconate 5-dehydrogenase
bin003	SOY3_bin003_00913	717	24	26	6	4.002	3.678	0.889	NADP-dependent isopropanol dehydrogenase
bin003	SOY3_bin003_00914	246	14	14	1	6.804	5.772	0.432	NADP-dependent isopropanol dehydrogenase
bin003	SOY3_bin003_00915	612	16	19	3	3.125	3.149	0.521	HTH-type transcriptional repressor KstR2
bin003	SOY3_bin003_00916	507	8	12	4	1.886	2.401	0.838	General stress protein 18
bin003	SOY3_bin003_00917	885	15	44	10	2.026	5.043	1.200	Chemotaxis protein methyltransferase Cher2
bin003	SOY3_bin003_00918	450	8	15	5	2.125	3.381	1.180	Response regulator rcp1
bin003	SOY3_bin003_00919	243	0	9	0	0.000	3.757	0.000	hypothetical protein
bin003	SOY3_bin003_00920	1602	41	66	19	3.060	4.179	1.260	Sodium/proline symporter
bin003	SOY3_bin003_00921	243	8	16	2	3.936	6.678	0.874	PRC-barrel domain protein
bin003	SOY3_bin003_00922	531	29	29	9	6.529	5.539	1.800	Orotate phosphoribosyltransferase
bin003	SOY3_bin003_00923	543	18	18	8	3.963	3.362	1.565	Molybdenum cofactor biosynthesis protein B
bin003	SOY3_bin003_00924	519	2	6	1	0.461	1.173	0.205	hypothetical protein
bin003	SOY3_bin003_00925	1173	20	20	3	2.038	1.729	0.272	hypothetical protein
bin003	SOY3_bin003_00926	984	18	33	3	2.187	3.402	0.324	Thiamine-monophosphate kinase
bin003	SOY3_bin003_00927	1551	40	74	14	3.083	4.839	0.959	hypothetical protein
bin003	SOY3_bin003_00928	570	12	15	1	2.517	2.669	0.186	hypothetical protein
bin003	SOY3_bin003_00929	381	6	21	5	1.883	5.590	1.394	hypothetical protein
bin003	SOY3_bin003_00930	447	12	27	7	3.209	6.126	1.663	hypothetical protein
bin003	SOY3_bin003_00931	1236	30	71	14	2.902	5.826	1.203	R-phenyllactate dehydratase activator
bin003	SOY3_bin003_00932	576	11	14	5	2.283	2.465	0.922	hypothetical protein
bin003	SOY3_bin003_00933	852	22	42	5	3.087	5.000	0.623	FeMo cofactor biosynthesis protein NifB
bin003	SOY3_bin003_00934	1140	31	67	8	3.251	5.961	0.745	Glycogen synthase
bin003	SOY3_bin003_00935	1128	65	125	34	6.889	11.240	3.202	Undecaprenyl-phosphate mannosyltransferase
bin003	SOY3_bin003_00936	855	15	38	9	2.097	4.508	1.118	UTP--glucose-1-phosphate uridylyltransferase
bin003	SOY3_bin003_00937	1068	11	23	3	1.231	2.184	0.298	Glucose-1-phosphate thymidylyltransferase
bin003	SOY3_bin003_00938	864	8	20	6	1.107	2.348	0.738	dTDP-4-dehydrorhamnose reductase
bin003	SOY3_bin003_00939	468	11	46	7	2.810	9.969	1.589	hypothetical protein
bin003	SOY3_bin003_00940	936	25	70	15	3.193	7.585	1.702	dTDP-glucose 4,6-dehydratase
bin003	SOY3_bin003_00941	315	0	0	1	0.000	0.000	0.337	hypothetical protein
bin003	SOY3_bin003_00942	84	9	10	9	12.809	12.075	11.381	tRNA-Leu(gag)
bin003	SOY3_bin003_00943	429	1	2	1	0.279	0.473	0.248	hypothetical protein
bin003	SOY3_bin003_00944	486	1	1	0	0.246	0.209	0.000	hypothetical protein
bin003	SOY3_bin003_00945	447	0	3	1	0.000	0.681	0.238	GIY-YIG catalytic domain protein
bin003	SOY3_bin003_00946	288	1	2	1	0.415	0.704	0.369	Plasmid stabilisation system protein
bin003	SOY3_bin003_00947	180	2	2	3	1.328	1.127	1.770	hypothetical protein
bin003	SOY3_bin003_00948	843	27	47	9	3.829	5.655	1.134	Nitrogenase iron protein 1
bin003	SOY3_bin003_00949	480	13	19	8	3.238	4.015	1.770	Carnitine transport ATP-binding protein OpuCA
bin003	SOY3_bin003_00950	357	0	4	1	0.000	1.136	0.298	hypothetical protein
bin003	SOY3_bin003_00951	1218	3	26	5	0.294	2.165	0.436	putative protease YhbU precursor
bin003	SOY3_bin003_00952	1398	19	49	14	1.625	3.555	1.064	Amidophosphoribosyltransferase precursor
bin003	SOY3_bin003_00953	894	67	107	26	8.959	12.140	3.089	hypothetical protein
bin003	SOY3_bin003_00954	186	19	71	17	12.212	38.717	9.709	50S ribosomal protein L37e

bin003	SOY3_bin003_00955	636	8	20	5	1.504	3.190	0.835	inosine 5'-monophosphate dehydrogenase
bin003	SOY3_bin003_00956	750	10	20	3	1.594	2.705	0.425	Ferredoxin-3
bin003	SOY3_bin003_00957	936	8	33	6	1.022	3.576	0.681	putative sugar kinase YdjH
bin003	SOY3_bin003_00958	900	15	45	12	1.992	5.071	1.416	Formyltransferase/hydrolase complex subunit D
bin003	SOY3_bin003_00959	1026	14	33	11	1.631	3.262	1.139	haloacid dehalogenase-like hydrolase
bin003	SOY3_bin003_00960	1380	25	80	13	2.166	5.880	1.001	Ferredoxin
bin003	SOY3_bin003_00961	1038	16	57	5	1.843	5.570	0.512	6-hydroxynicotinate reductase
bin003	SOY3_bin003_00962	1155	27	59	22	2.795	5.181	2.023	NADH-quinone oxidoreductase subunit 4
bin003	SOY3_bin003_00963	501	9	23	10	2.148	4.656	2.120	Formate hydrogenlyase subunit 7
bin003	SOY3_bin003_00964	687	9	21	6	1.566	3.100	0.928	hypothetical protein
bin003	SOY3_bin003_00965	327	7	13	3	2.559	4.032	0.975	hypothetical protein
bin003	SOY3_bin003_00966	264	1	13	0	0.453	4.995	0.000	Inhibitor of apoptosis-promoting Bax1
bin003	SOY3_bin003_00967	936	16	34	11	2.044	3.684	1.248	NADH-quinone oxidoreductase subunit H
bin003	SOY3_bin003_00968	216	1	4	3	0.553	1.878	1.475	hypothetical protein
bin003	SOY3_bin003_00969	684	14	29	12	2.447	4.300	1.864	hydrogenase subunit F
bin003	SOY3_bin003_00970	687	30	33	9	5.220	4.872	1.392	hypothetical protein
bin003	SOY3_bin003_00971	480	20	43	12	4.981	9.086	2.656	hypothetical protein
bin003	SOY3_bin003_00972	261	12	12	3	5.497	4.663	1.221	hypothetical protein
bin003	SOY3_bin003_00973	291	12	15	3	4.930	5.228	1.095	hypothetical protein
bin003	SOY3_bin003_00974	144	3	5	2	2.491	3.522	1.475	hypothetical protein
bin003	SOY3_bin003_00975	648	4	3	0	0.738	0.470	0.000	HTH-type transcriptional repressor KstR2
bin003	SOY3_bin003_00976	1395	35	61	19	2.999	4.435	1.447	Glutamate decarboxylase
bin003	SOY3_bin003_00977	318	0	1	0	0.000	0.319	0.000	Flavodoxin-like fold protein
bin003	SOY3_bin003_00978	741	47	57	18	7.583	7.802	2.580	glucose-6-phosphate isomerase
bin003	SOY3_bin003_00979	657	33	51	13	6.005	7.873	2.102	hypothetical protein
bin003	SOY3_bin003_00980	282	24	52	15	10.174	18.703	5.650	hypothetical protein
bin003	SOY3_bin003_00981	504	15	16	2	3.558	3.220	0.422	hypothetical protein
bin003	SOY3_bin003_00982	324	17	41	7	6.273	12.835	2.295	Cupin domain protein
bin003	SOY3_bin003_00983	1284	14	46	17	1.303	3.634	1.406	Homoserine dehydrogenase
bin003	SOY3_bin003_00984	1029	7	18	2	0.813	1.774	0.206	Nucleotidyltransferase domain protein
bin003	SOY3_bin003_00985	588	21	38	11	4.270	6.555	1.987	hypothetical protein
bin003	SOY3_bin003_00986	492	9	19	3	2.187	3.917	0.648	6-carboxy-5,6,7,8-tetrahydropterin synthase
bin003	SOY3_bin003_00987	702	27	24	8	4.598	3.468	1.211	7-carboxy-7-deazaguanine synthase
bin003	SOY3_bin003_00988	552	66	75	14	14.294	13.781	2.694	Hypoxic response protein 1
bin003	SOY3_bin003_00989	825	63	73	19	9.129	8.975	2.446	Inosine-5'-monophosphate dehydrogenase
bin003	SOY3_bin003_00990	936	58	61	15	7.408	6.610	1.702	Hypoxic response protein 1
bin003	SOY3_bin003_00991	840	64	84	21	9.108	10.143	2.656	Hypoxic response protein 1
bin003	SOY3_bin003_00992	822	30	50	9	4.363	6.170	1.163	Hypoxic response protein 1
bin003	SOY3_bin003_00993	267	2	11	1	0.895	4.179	0.398	Ribonuclease Z
bin003	SOY3_bin003_00994	762	11	32	7	1.726	4.259	0.976	Small-conductance mechanosensitive channel
bin003	SOY3_bin003_00995	606	12	13	0	2.367	2.176	0.000	Sortase family protein
bin003	SOY3_bin003_00996	252	13	30	7	6.167	12.075	2.951	Ferredoxin-2
bin003	SOY3_bin003_00997	555	24	75	17	5.170	13.706	3.254	hypothetical protein
bin003	SOY3_bin003_00998	921	49	81	17	6.360	8.920	1.961	NAD-reducing hydrogenase HoxS subunit delta
bin003	SOY3_bin003_00999	1452	137	271	56	11.280	18.930	4.097	NAD-reducing hydrogenase HoxS subunit beta
bin003	SOY3_bin003_01000	1278	29	39	9	2.713	3.095	0.748	Roadblock/LC7 domain protein
bin003	SOY3_bin003_01001	966	22	40	9	2.723	4.200	0.990	DNA polymerase III subunit tau
bin003	SOY3_bin003_01002	1506	37	49	8	2.937	3.300	0.564	ATP-dependent zinc metalloprotease FtsH
bin003	SOY3_bin003_01003	672	4	1	1	0.712	0.151	0.158	hypothetical protein
bin003	SOY3_bin003_01004	1221	4	4	2	0.392	0.332	0.174	putative amino acid permease YhdG
bin003	SOY3_bin003_01005	936	25	33	7	3.193	3.576	0.794	antitoxin MazE
bin003	SOY3_bin003_01006	867	11	22	4	1.517	2.574	0.490	hypothetical protein
bin003	SOY3_bin003_01007	609	32	44	15	6.282	7.328	2.616	Inner membrane protein YqjA
bin003	SOY3_bin003_01008	405	2	2	2	0.590	0.501	0.525	hypothetical protein
bin003	SOY3_bin003_01009	1131	63	105	22	6.659	9.416	2.066	Inner membrane transport permease YbhR
bin003	SOY3_bin003_01010	666	30	49	14	5.385	7.462	2.233	Macrolide export ATP-binding/permease protein MacB
bin003	SOY3_bin003_01011	1083	24	60	12	2.649	5.619	1.177	Macrolide export ATP-binding/permease protein MacB
bin003	SOY3_bin003_01012	243	4	10	0	1.968	4.174	0.000	anaerobic benzoate catabolism transcriptional regulator
bin003	SOY3_bin003_01013	405	1	5	0	0.295	1.252	0.000	hypothetical protein
bin003	SOY3_bin003_01014	720	3	1	2	0.498	0.141	0.295	ABC-2 family transporter protein
bin003	SOY3_bin003_01015	930	4	5	2	0.514	0.545	0.228	Fluoroquinolones export ATP-binding protein/MT2762
bin003	SOY3_bin003_01016	411	2	4	0	0.582	0.987	0.000	hypothetical protein
bin003	SOY3_bin003_01017	1188	11	30	8	1.107	2.561	0.715	Inner membrane transport permease YbhR
bin003	SOY3_bin003_01018	993	18	24	7	2.167	2.451	0.749	putative ABC transporter ATP-binding protein YxlF
bin003	SOY3_bin003_01019	300	10	16	4	3.985	5.409	1.416	hypothetical protein
bin003	SOY3_bin003_01020	846	40	45	11	5.652	5.395	1.381	putative metallophosphoesterase
bin003	SOY3_bin003_01021	486	114	232	54	28.042	48.418	11.803	Inner membrane protein YeeR

bin003	SOY3_bin003_01022	1215	12	29	10	1.181	2.421	0.874	Molybdopterin molybdenumtransferase
bin003	SOY3_bin003_01023	1173	38	74	23	3.873	6.399	2.083	Peptidase family M50
bin003	SOY3_bin003_01024	474	6	6	3	1.513	1.284	0.672	TPR repeat-containing protein YrrB
bin003	SOY3_bin003_01025	1257	8	7	3	0.761	0.565	0.254	Radical SAM superfamily protein
bin003	SOY3_bin003_01026	1260	1	8	3	0.095	0.644	0.253	Radical SAM superfamily protein
bin003	SOY3_bin003_01027	420	1	3	2	0.285	0.724	0.506	hypothetical protein
bin003	SOY3_bin003_01028	2319	12	31	6	0.619	1.356	0.275	Serine/threonine-protein kinase PknB
bin003	SOY3_bin003_01029	237	8	16	2	4.035	6.847	0.896	hypothetical protein
bin003	SOY3_bin003_01030	1719	13	34	11	0.904	2.006	0.680	Immune inhibitor A precursor
bin003	SOY3_bin003_01031	783	11	13	6	1.679	1.684	0.814	diadenosine tetraphosphatase
bin003	SOY3_bin003_01032	360	0	1	0	0.000	0.282	0.000	Double zinc ribbon
bin003	SOY3_bin003_01033	666	9	18	4	1.616	2.741	0.638	TMAO/DMSO reductase
bin003	SOY3_bin003_01034	612	7	14	2	1.367	2.320	0.347	TPR repeat-containing protein YrrB
bin003	SOY3_bin003_01035	828	3	11	3	0.433	1.347	0.385	hypothetical protein
bin003	SOY3_bin003_01036	852	6	6	2	0.842	0.714	0.249	pH-gated potassium channel KcsA
bin003	SOY3_bin003_01037	669	6	10	3	1.072	1.516	0.476	Ribose-5-phosphate isomerase A
bin003	SOY3_bin003_01038	435	6	9	4	1.649	2.098	0.977	hypothetical protein
bin003	SOY3_bin003_01039	537	11	24	9	2.449	4.533	1.780	hypothetical protein
bin003	SOY3_bin003_01040	2379	60	100	27	3.015	4.263	1.206	replication factor A
bin003	SOY3_bin003_01041	420	2	7	2	0.569	1.690	0.506	Transcriptional repressor MprA
bin003	SOY3_bin003_01042	1254	9	19	4	0.858	1.537	0.339	2,3-dimethylmalate dehydratase large subunit
bin003	SOY3_bin003_01043	507	3	15	1	0.707	3.001	0.210	2,3-dimethylmalate dehydratase small subunit
bin003	SOY3_bin003_01044	987	5	18	5	0.606	1.850	0.538	Homoisocitrate dehydrogenase
bin003	SOY3_bin003_01045	1200	34	68	12	3.387	5.748	1.062	putative cysteine desulfurase
bin003	SOY3_bin003_01046	405	10	23	10	2.952	5.760	2.623	6,7-dimethyl-8-ribityllumazine synthase
bin003	SOY3_bin003_01047	933	19	35	10	2.435	3.805	1.139	hypothetical protein
bin003	SOY3_bin003_01048	1713	80	126	26	5.583	7.461	1.612	Glucosyl-3-phosphoglycerate synthase
bin003	SOY3_bin003_01049	1029	16	22	14	1.859	2.169	1.445	N5-carboxyaminoimidazole ribonucleotide mutase
bin003	SOY3_bin003_01050	1308	26	41	10	2.376	3.179	0.812	Phenolic acid decarboxylase subunit C
bin003	SOY3_bin003_01051	306	7	6	5	2.735	1.989	1.736	V4R domain protein
bin003	SOY3_bin003_01052	375	0	7	3	0.000	1.893	0.850	Roadblock/LC7 domain protein
bin003	SOY3_bin003_01053	513	1	5	1	0.233	0.989	0.207	hypothetical protein
bin003	SOY3_bin003_01054	486	18	20	1	4.428	4.174	0.219	hypothetical protein
bin003	SOY3_bin003_01055	498	4	8	1	0.960	1.629	0.213	Translation initiation factor IF-2
bin003	SOY3_bin003_01056	366	2	3	1	0.653	0.831	0.290	Roadblock/LC7 domain protein
bin003	SOY3_bin003_01057	378	1	1	1	0.316	0.268	0.281	Roadblock/LC7 domain protein
bin003	SOY3_bin003_01058	636	6	6	1	1.128	0.957	0.167	Uracil phosphoribosyltransferase
bin003	SOY3_bin003_01059	1053	12	23	2	1.362	2.215	0.202	Soluble aldose sugar dehydrogenase YliI precursor
bin003	SOY3_bin003_01060	150	0	0	0	0.000	0.000	0.000	hypothetical protein
bin003	SOY3_bin003_01061	258	0	0	1	0.000	0.000	0.412	hypothetical protein
bin003	SOY3_bin003_01062	531	6	8	5	1.351	1.528	1.000	tRNA 2'-O-methylase
bin003	SOY3_bin003_01063	792	6	14	3	0.906	1.793	0.402	Cobalamin synthase
bin003	SOY3_bin003_01064	1092	12	29	10	1.314	2.694	0.973	Phosphatidylglycerophosphatase A
bin003	SOY3_bin003_01065	360	11	17	3	3.653	4.790	0.885	nitrogen regulatory protein P-II 1
bin003	SOY3_bin003_01066	201	4	6	1	2.379	3.028	0.528	hypothetical protein
bin003	SOY3_bin003_01067	1491	121	92	16	9.702	6.258	1.140	Hydroxylamine reductase
bin003	SOY3_bin003_01068	753	32	48	20	5.080	6.465	2.821	putative HTH-type transcriptional regulator YgaV
bin003	SOY3_bin003_01069	1215	43	72	17	4.231	6.011	1.486	hypothetical protein
bin003	SOY3_bin003_01070	678	16	12	5	2.821	1.795	0.783	7-cyano-7-deazaguanine synthase
bin003	SOY3_bin003_01071	864	13	19	7	1.799	2.230	0.861	hypothetical protein
bin003	SOY3_bin003_01072	1014	24	27	5	2.830	2.701	0.524	Peptidase family M50
bin003	SOY3_bin003_01073	201	2	7	2	1.190	3.532	1.057	bifunctional sulfur carrier protein/thiazole synthase protein
bin003	SOY3_bin003_01074	972	37	38	6	4.551	3.965	0.656	tRNA 2-thiocytidine biosynthesis protein TtcA
bin003	SOY3_bin003_01075	144	5	4	0	4.151	2.817	0.000	hypothetical protein
bin003	SOY3_bin003_01076	297	0	1	1	0.000	0.342	0.358	hypothetical protein
bin003	SOY3_bin003_01077	177	1	1	1	0.675	0.573	0.600	hypothetical protein
bin003	SOY3_bin003_01078	1554	29	46	10	2.231	3.002	0.684	methyltetrahydrofolate:corrinoid/iron-sulfur protein methyltransferase
bin003	SOY3_bin003_01079	531	12	21	6	2.702	4.011	1.200	Pyruvate synthase subunit PorC
bin003	SOY3_bin003_01080	243	3	5	4	1.476	2.087	1.749	Pyruvate synthase subunit PorD
bin003	SOY3_bin003_01081	1158	45	76	21	4.646	6.657	1.926	Pyruvate synthase subunit PorA
bin003	SOY3_bin003_01082	867	59	84	19	8.135	9.827	2.328	Pyruvate synthase subunit PorB
bin003	SOY3_bin003_01083	543	34	45	7	7.486	8.406	1.369	Iron-sulfur protein
bin003	SOY3_bin003_01084	432	22	28	15	6.088	6.574	3.688	Anaerobic dimethyl sulfoxide reductase chain B
bin003	SOY3_bin003_01085	2370	92	123	13	4.641	5.264	0.583	Carbon monoxide dehydrogenase/acetyl-CoA synthase subunit beta
bin003	SOY3_bin003_01086	516	15	12	9	3.475	2.359	1.853	acetyl-CoA decarbonylase/synthase complex subunit epsilon
bin003	SOY3_bin003_01087	5100	48	88	14	1.125	1.750	0.292	Phytochrome-like protein cph1
bin003	SOY3_bin003_01088	2010	13	27	5	0.773	1.362	0.264	Membrane protein of unknown function

bin003	SOY3_bin003_01089	1326	28	76	12	2.524	5.813	0.961	Glutamine synthetase
bin003	SOY3_bin003_01090	1701	64	140	34	4.498	8.348	2.123	Ribonuclease R winged-helix domain protein
bin003	SOY3_bin003_01091	492	3	17	5	0.729	3.505	1.080	putative tRNA (adenine(37)-N6)-methyltransferase
bin003	SOY3_bin003_01092	210	15	31	9	8.539	14.973	4.553	Molybdenum-pterin-binding protein 2
bin003	SOY3_bin003_01093	195	2	2	1	1.226	1.040	0.545	Iron-sulfur protein
bin003	SOY3_bin003_01094	1005	7	11	1	0.833	1.110	0.106	NADH-quinone oxidoreductase subunit I
bin003	SOY3_bin003_01095	738	13	15	1	2.106	2.062	0.144	Zn-finger in Ran binding protein and others
bin003	SOY3_bin003_01096	1281	21	24	4	1.960	1.900	0.332	(Dimethylallyl)adenosine tRNA methylthiotransferase MiaB
bin003	SOY3_bin003_01097	654	12	18	3	2.194	2.792	0.487	Protein-L-isoaspartate O-methyltransferase
bin003	SOY3_bin003_01098	597	11	19	3	2.203	3.228	0.534	hypothetical protein
bin003	SOY3_bin003_01099	489	28	71	16	6.845	14.727	3.476	2,3-dimethylmalate dehydratase small subunit
bin003	SOY3_bin003_01100	990	74	105	31	8.936	10.757	3.326	4-diphosphocytidyl-2-C-methyl-D-erythritol kinase
bin003	SOY3_bin003_01101	1494	94	166	39	7.522	11.270	2.773	Cyclic pyranopterin monophosphate synthase
bin003	SOY3_bin003_01102	579	22	45	11	4.542	7.883	2.018	hypothetical protein
bin003	SOY3_bin003_01103	1029	31	55	10	3.602	5.421	1.032	Hydantoinase/oxoprolinase
bin003	SOY3_bin003_01104	1029	22	23	6	2.556	2.267	0.619	carbamoyl phosphate synthase-like protein
bin003	SOY3_bin003_01105	1311	24	51	10	2.189	3.946	0.810	UDP-2,3-diacetamido-2,3-dideoxy-D-glucuronate 2-epimerase
bin003	SOY3_bin003_01106	1263	90	95	22	8.519	7.629	1.850	hypothetical protein
bin003	SOY3_bin003_01107	723	1	13	1	0.165	1.824	0.147	tRNA pseudouridine synthase A
bin003	SOY3_bin003_01108	1200	3	3	1	0.299	0.254	0.089	hypothetical protein
bin003	SOY3_bin003_01109	858	191	646	150	26.613	76.366	18.571	Pseudomurein-binding repeat protein
bin003	SOY3_bin003_01110	333	6	2	2	2.154	0.609	0.638	preprotein translocase subunit SecG
bin003	SOY3_bin003_01111	273	17	51	24	7.444	18.948	9.339	DNA/RNA-binding protein albA
bin003	SOY3_bin003_01112	3783	47	80	20	1.485	2.145	0.562	Phytochrome-like protein cph1
bin003	SOY3_bin003_01113	453	2	5	0	0.528	1.120	0.000	Response regulator rcp1
bin003	SOY3_bin003_01114	1209	17	31	2	1.681	2.601	0.176	Phytochrome-like protein cph1
bin003	SOY3_bin003_01115	771	120	131	29	18.607	17.233	3.996	Iron-dependent repressor IdeR
bin003	SOY3_bin003_01116	1983	98	114	24	5.908	5.831	1.286	Ferrous iron transport protein B
bin003	SOY3_bin003_01117	861	2	5	0	0.278	0.589	0.000	Ribonuclease HIII
bin003	SOY3_bin003_01118	255	0	1	0	0.000	0.398	0.000	hypothetical protein
bin003	SOY3_bin003_01119	468	3	7	5	0.766	1.517	1.135	Pyridoxamine 5'-phosphate oxidase
bin003	SOY3_bin003_01120	207	2	0	0	1.155	0.000	0.000	hypothetical protein
bin003	SOY3_bin003_01121	390	2	6	4	0.613	1.560	1.089	RNA polymerase sigma factor SigJ
bin003	SOY3_bin003_01122	447	4	12	5	1.070	2.723	1.188	Protease synthase and sporulation negative regulatory protein PAI 1
bin003	SOY3_bin003_01123	1107	37	91	14	3.996	8.338	1.343	Chorismate synthase
bin003	SOY3_bin003_01124	156	6	4	1	4.598	2.601	0.681	hypothetical protein
bin003	SOY3_bin003_01125	74	1	4	1	1.616	5.483	1.435	tRNA-Thr(cgt)
bin003	SOY3_bin003_01126	153	2	1	1	1.563	0.663	0.694	hypothetical protein
bin003	SOY3_bin003_01127	192	1	1	0	0.623	0.528	0.000	hypothetical protein
bin003	SOY3_bin003_01128	801	9	14	4	1.343	1.773	0.530	ribonuclease Z
bin003	SOY3_bin003_01129	930	16	25	8	2.057	2.727	0.914	hypothetical protein
bin003	SOY3_bin003_01130	921	3	15	1	0.389	1.652	0.115	hypothetical protein
bin003	SOY3_bin003_01131	2904	35	44	9	1.441	1.537	0.329	UvrABC system protein A
bin003	SOY3_bin003_01132	1959	32	61	8	1.953	3.158	0.434	UvrABC system protein B
bin003	SOY3_bin003_01133	825	21	48	10	3.043	5.901	1.288	Glycine betaine/carnitine/choline transport ATP-binding protein OpuCA
bin003	SOY3_bin003_01134	360	0	8	1	0.000	2.254	0.295	Dinitrogenase iron-molybdenum cofactor
bin003	SOY3_bin003_01135	501	16	31	7	3.818	6.276	1.484	hypothetical protein
bin003	SOY3_bin003_01136	1779	40	91	20	2.688	5.188	1.194	hypothetical protein
bin003	SOY3_bin003_01137	1050	0	2	1	0.000	0.193	0.101	Demethylspheroidene O-methyltransferase
bin003	SOY3_bin003_01138	1125	1	9	1	0.106	0.811	0.094	putative ABC transporter solute-binding protein YclQ precursor
bin003	SOY3_bin003_01139	765	6	19	6	0.938	2.519	0.833	putative siderophore transport system ATP-binding protein YusV
bin003	SOY3_bin003_01140	1059	12	31	4	1.355	2.969	0.401	Hemin transport system permease protein HmuU
bin003	SOY3_bin003_01141	1050	5	32	9	0.569	3.091	0.911	Demethylspheroidene O-methyltransferase
bin003	SOY3_bin003_01142	1119	19	40	13	2.030	3.626	1.234	putative ABC transporter solute-binding protein YclQ precursor
bin003	SOY3_bin003_01143	1125	23	83	18	2.444	7.483	1.700	Fe(3+)-citrate-binding protein YfmC precursor
bin003	SOY3_bin003_01144	1101	3	2	3	0.326	0.184	0.289	Fe(3+)-citrate-binding protein YfmC precursor
bin003	SOY3_bin003_01145	1230	5	10	4	0.486	0.825	0.345	DNA polymerase/3'-5' exonuclease PolX
bin003	SOY3_bin003_01146	228	8	4	2	4.195	1.779	0.932	TM2 domain protein
bin003	SOY3_bin003_01147	156	0	4	4	0.000	2.601	2.724	hypothetical protein
bin003	SOY3_bin003_01148	765	5	5	1	0.781	0.663	0.139	hypothetical protein
bin003	SOY3_bin003_01149	360	2	5	0	0.664	1.409	0.000	hypothetical protein
bin003	SOY3_bin003_01150	378	10	14	8	3.163	3.757	2.248	Double zinc ribbon
bin003	SOY3_bin003_01151	891	27	56	7	3.623	6.375	0.835	Sensor histidine kinase TodS
bin003	SOY3_bin003_01152	453	13	19	6	3.431	4.254	1.407	Response regulator rcp1
bin003	SOY3_bin003_01153	330	3	14	4	1.087	4.303	1.288	hypothetical protein
bin003	SOY3_bin003_01154	573	54	74	20	11.266	13.099	3.708	18 kDa heat shock protein
bin003	SOY3_bin003_01155	138	8	3	0	6.930	2.205	0.000	hypothetical protein

bin003	SOY3_bin003_01156	1140	29	31	7	3.041	2.758	0.652	Anaerobic sulfatase-maturating enzyme
bin003	SOY3_bin003_01157	162	1	4	1	0.738	2.504	0.656	30S ribosomal protein S27ae
bin003	SOY3_bin003_01158	147	1	10	2	0.813	6.900	1.445	Ferredoxin
bin003	SOY3_bin003_01159	1185	34	50	14	3.430	4.280	1.255	Putative oxidoreductase/MT0587
bin003	SOY3_bin003_01160	810	25	20	5	3.690	2.504	0.656	phosphoribose diphosphate:decaprenyl-phosphate phosphoribosyltransferase
bin003	SOY3_bin003_01161	1758	33	48	10	2.244	2.769	0.604	Putative thiamine pyrophosphate-containing protein YdaP
bin003	SOY3_bin003_01162	399	6	20	4	1.798	5.084	1.065	DNA repair and recombination protein RadA
bin003	SOY3_bin003_01163	1986	490	1266	309	29.496	64.656	16.528	Hydrogenase-4 component A
bin003	SOY3_bin003_01164	1269	39	75	17	3.674	5.995	1.423	Serine hydroxymethyltransferase
bin003	SOY3_bin003_01165	708	26	47	16	4.390	6.733	2.401	NADH-quinone oxidoreductase subunit I
bin003	SOY3_bin003_01166	288	4	7	1	1.660	2.465	0.369	hypothetical protein
bin003	SOY3_bin003_01167	378	4	10	0	1.265	2.683	0.000	hypothetical protein
bin003	SOY3_bin003_01168	1206	79	120	28	7.831	10.092	2.466	S-adenosylmethionine synthetase
bin003	SOY3_bin003_01169	3162	134	195	42	5.066	6.255	1.411	Isoleucine--tRNA ligase
bin003	SOY3_bin003_01170	4134	27	53	12	0.781	1.300	0.308	hypothetical protein
bin003	SOY3_bin003_01171	885	2	5	2	0.270	0.573	0.240	TPR repeat-containing protein YrrB
bin003	SOY3_bin003_01172	276	1	8	2	0.433	2.940	0.770	hypothetical protein
bin003	SOY3_bin003_01173	168	0	2	0	0.000	1.207	0.000	lipoprotein NlpI
bin003	SOY3_bin003_01174	774	6	14	1	0.927	1.835	0.137	hypothetical protein
bin003	SOY3_bin003_01175	1044	38	59	13	4.351	5.732	1.323	Cyclic pyranopterin monophosphate synthase
bin003	SOY3_bin003_01176	705	28	58	12	4.748	8.344	1.808	Molybdopterin-guanine dinucleotide biosynthesis adapter protein
bin003	SOY3_bin003_01177	462	83	134	41	21.477	29.418	9.427	Ferredoxin
bin003	SOY3_bin003_01178	1059	166	300	68	18.739	28.733	6.821	NADH-quinone oxidoreductase subunit I
bin003	SOY3_bin003_01179	234	42	64	10	21.457	27.741	4.540	Ferredoxin-2
bin003	SOY3_bin003_01180	543	105	170	33	23.117	31.754	6.456	Nitrate reductase
bin003	SOY3_bin003_01181	348	0	3	2	0.000	0.874	0.610	Spore protein SP21
bin003	SOY3_bin003_01182	306	10	9	10	3.907	2.983	3.471	PRC-barrel domain protein
bin003	SOY3_bin003_01183	2178	60	85	19	3.293	3.958	0.927	Chaperone protein DnaJ
bin003	SOY3_bin003_01184	489	44	49	17	10.757	10.163	3.693	hypothetical protein
bin003	SOY3_bin003_01185	384	10	25	9	3.113	6.603	2.490	Desulfoferrodoxin
bin003	SOY3_bin003_01186	576	13	36	5	2.698	6.339	0.922	Rubrerythrin-1
bin003	SOY3_bin003_01187	1236	18	40	13	1.741	3.282	1.117	phosphoribosylformylglycinamidine synthase I
bin003	SOY3_bin003_01188	1296	129	425	112	11.900	33.261	9.180	phosphoribosylformylglycinamidine synthase I
bin003	SOY3_bin003_01189	1368	32	57	16	2.796	4.226	1.242	Multidrug resistance protein 3
bin003	SOY3_bin003_01190	489	13	27	1	3.178	5.600	0.217	hypothetical protein
bin003	SOY3_bin003_01191	1008	14	32	6	1.660	3.220	0.632	Pyruvate formate-lyase 1-activating enzyme
bin003	SOY3_bin003_01192	1047	9	21	5	1.028	2.034	0.507	Thiamine-monophosphate kinase
bin003	SOY3_bin003_01193	495	19	34	8	4.589	6.967	1.717	Sirohydrochlorin cobaltochelatase
bin003	SOY3_bin003_01194	399	10	22	7	2.996	5.592	1.864	hypothetical protein
bin003	SOY3_bin003_01195	993	1	3	0	0.120	0.306	0.000	hypothetical protein
bin003	SOY3_bin003_01196	927	0	3	2	0.000	0.328	0.229	hypothetical protein
bin003	SOY3_bin003_01197	768	14	26	6	2.179	3.434	0.830	hypothetical protein
bin003	SOY3_bin003_01198	474	5	25	3	1.261	5.350	0.672	Regulatory protein AsnC
bin003	SOY3_bin003_01199	1239	25	38	11	2.412	3.111	0.943	Magnesium transporter MgtE
bin003	SOY3_bin003_01200	1257	21	43	15	1.997	3.470	1.268	Divalent metal cation transporter MntH
bin003	SOY3_bin003_01201	594	1	5	1	0.201	0.854	0.179	Ribosomal protein L11 methyltransferase
bin003	SOY3_bin003_01202	510	0	1	0	0.000	0.199	0.000	hypothetical protein
bin003	SOY3_bin003_01203	294	0	0	0	0.000	0.000	0.000	hypothetical protein
bin003	SOY3_bin003_01204	510	1	1	0	0.234	0.199	0.000	hypothetical protein
bin003	SOY3_bin003_01205	972	7	25	1	0.861	2.609	0.109	Sporulenol synthase
bin003	SOY3_bin003_01206	618	40	84	20	7.738	13.786	3.438	Superoxide dismutase [Mn/Fe]
bin003	SOY3_bin003_01207	687	22	72	9	3.828	10.630	1.392	Selenocysteine-containing peroxiredoxin PrxU
bin003	SOY3_bin003_01208	507	16	46	7	3.773	9.202	1.467	Ferritin
bin003	SOY3_bin003_01209	162	4	23	2	2.952	14.400	1.311	Rubredoxin
bin003	SOY3_bin003_01210	171	4	13	1	2.796	7.711	0.621	Rubredoxin
bin003	SOY3_bin003_01211	159	0	7	1	0.000	4.465	0.668	Rubredoxin
bin003	SOY3_bin003_01212	381	1	16	5	0.314	4.259	1.394	Putative universal stress protein
bin003	SOY3_bin003_01213	1743	25	56	20	1.715	3.259	1.219	Methionine import ATP-binding protein MetN
bin003	SOY3_bin003_01214	528	13	27	8	2.943	5.187	1.609	Bifunctional NMN adenylyltransferase/Nudix hydrolase
bin003	SOY3_bin003_01215	1161	32	35	9	3.295	3.058	0.823	deoxyguanosinetriphosphate triphosphohydrolase-like protein
bin003	SOY3_bin003_01216	552	20	23	3	4.331	4.226	0.577	putative aromatic acid decarboxylase
bin003	SOY3_bin003_01217	564	22	42	9	4.663	7.553	1.695	putative cobalt-precorrin-6Y C(15)-methyltransferase [decarboxylating]
bin003	SOY3_bin003_01218	807	0	0	0	0.000	0.000	0.000	manganese transport regulator MntR
bin003	SOY3_bin003_01219	945	13	11	4	1.645	1.181	0.450	Transposase DDE domain protein
bin003	SOY3_bin003_01220	4323	3	7	1	0.083	0.164	0.025	Aerobic cobaltochelatase subunit CobN
bin003	SOY3_bin003_01221	2679	8	20	10	0.357	0.757	0.397	Aerobic cobaltochelatase subunit CobN
bin003	SOY3_bin003_01222	77	13	22	10	20.184	28.979	13.796	tRNA-Ile(gat)

bin003	SOY3_bin003_01223	76	5	24	12	7.865	32.030	16.772	tRNA-Ala(tgc)
bin003	SOY3_bin003_01224	3485	19058	22929	19814	653.763	667.325	603.948	23S ribosomal RNA
bin003	SOY3_bin003_01225	116	1	3	2	1.031	2.623	1.831	5S ribosomal RNA
bin003	SOY3_bin003_01226	1032	27	66	14	3.128	6.487	1.441	Glycine betaine/carnitine transport binding protein GbuC precursor
bin003	SOY3_bin003_01227	843	35	35	7	4.963	4.211	0.882	Glycine betaine transport system permease protein OpuAB
bin003	SOY3_bin003_01228	121	0	0	0	0.000	0.000	0.000	5S ribosomal RNA
bin003	SOY3_bin003_01229	882	50	161	67	6.777	18.515	8.069	D-gamma-glutamyl-meso-diaminopimelic acid endopeptidase CwlS precursor
bin003	SOY3_bin003_01230	732	2	12	2	0.327	1.663	0.290	putative transcriptional regulatory protein
bin003	SOY3_bin003_01231	630	4	7	6	0.759	1.127	1.012	hypothetical protein
bin003	SOY3_bin003_01232	996	12	19	3	1.440	1.935	0.320	Putative diphthamide synthesis protein
bin003	SOY3_bin003_01233	558	8	14	5	1.714	2.545	0.952	Pur operon repressor
bin003	SOY3_bin003_01234	1335	27	44	9	2.418	3.343	0.716	Signal recognition particle protein
bin003	SOY3_bin003_01235	1257	9	17	7	0.856	1.372	0.592	H/ACA RNA-protein complex component Cbf5p
bin003	SOY3_bin003_01236	291	17	34	7	6.984	11.851	2.555	50S ribosomal protein L21e
bin003	SOY3_bin003_01237	339	18	33	5	6.348	9.873	1.567	RNA polymerase Rpb4
bin003	SOY3_bin003_01238	579	27	45	16	5.575	7.883	2.935	hypothetical protein
bin003	SOY3_bin003_01239	828	5	12	2	0.722	1.470	0.257	Ribosomal RNA small subunit methyltransferase A
bin003	SOY3_bin003_01240	501	1	2	0	0.239	0.405	0.000	Multidrug resistance protein MdtA
bin003	SOY3_bin003_01241	324	1	4	1	0.369	1.252	0.328	Carboxymuconolactone decarboxylase family protein
bin003	SOY3_bin003_01242	294	1	5	0	0.407	1.725	0.000	hypothetical protein
bin003	SOY3_bin003_01243	141	3	3	0	2.544	2.158	0.000	hypothetical protein
bin003	SOY3_bin003_01244	363	3	0	2	0.988	0.000	0.585	hypothetical protein
bin003	SOY3_bin003_01245	597	4	10	0	0.801	1.699	0.000	Ribosomal protein L11 methyltransferase
bin003	SOY3_bin003_01246	219	3	1	1	1.638	0.463	0.485	Divalent metal cation transporter MntH
bin003	SOY3_bin003_01247	744	5	11	5	0.803	1.500	0.714	hypothetical protein
bin003	SOY3_bin003_01248	1122	22	36	16	2.344	3.254	1.515	cell division control protein 6
bin003	SOY3_bin003_01249	183	7	11	2	4.573	6.097	1.161	hypothetical protein
bin003	SOY3_bin003_01250	453	2	7	1	0.528	1.567	0.234	hypothetical protein
bin003	SOY3_bin003_01251	1449	36	59	12	2.970	4.130	0.880	RNA-splicing ligase RtcB
bin003	SOY3_bin003_01252	792	15	19	7	2.264	2.433	0.939	S-methyl-5'-thioadenosine phosphorylase
bin003	SOY3_bin003_01253	915	21	36	14	2.744	3.991	1.625	Quinolinate synthase A
bin003	SOY3_bin003_01254	1011	12	25	1	1.419	2.508	0.105	hypothetical protein
bin003	SOY3_bin003_01255	1755	18	56	10	1.226	3.236	0.605	GalNAc(5)-diNAcBac-PP-undecaprenol beta-1,3-glucosyltransferase
bin003	SOY3_bin003_01256	690	3	5	1	0.520	0.735	0.154	hypothetical protein
bin003	SOY3_bin003_01257	357	2	3	1	0.670	0.852	0.298	HTH-type transcriptional repressor CzrA
bin003	SOY3_bin003_01258	1023	62	122	25	7.245	12.096	2.596	Adenylosuccinate synthetase
bin003	SOY3_bin003_01259	558	3	13	2	0.643	2.363	0.381	L-2,4-diaminobutyric acid acetyltransferase
bin003	SOY3_bin003_01260	1440	19	48	11	1.577	3.381	0.811	Diaminobutyrate--2-oxoglutarate transaminase
bin003	SOY3_bin003_01261	393	1	6	2	0.304	1.549	0.541	L-ectoine synthase
bin003	SOY3_bin003_01262	327	6	4	2	2.194	1.241	0.650	Amicyanin precursor
bin003	SOY3_bin003_01263	393	4	7	2	1.217	1.807	0.541	hypothetical protein
bin003	SOY3_bin003_01264	231	9	22	2	4.658	9.660	0.920	hypothetical protein
bin003	SOY3_bin003_01265	72	0	4	0	0.000	5.635	0.000	tRNA-Gln(ctg)
bin003	SOY3_bin003_01266	321	6	6	1	2.235	1.896	0.331	hypothetical protein
bin003	SOY3_bin003_01267	156	0	4	1	0.000	2.601	0.681	hypothetical protein
bin003	SOY3_bin003_01268	288	10	5	3	4.151	1.761	1.107	hypothetical protein
bin003	SOY3_bin003_01269	2874	34	246	78	1.414	8.682	2.883	FmdE, Molybdenum formylmethanofuran dehydrogenase operon
bin003	SOY3_bin003_01270	150	1	2	0	0.797	1.352	0.000	hypothetical protein
bin003	SOY3_bin003_01271	621	3	8	2	0.578	1.307	0.342	hypothetical protein
bin003	SOY3_bin003_01272	471	7	10	5	1.777	2.153	1.128	NAD(P)H dehydrogenase (quinone)
bin003	SOY3_bin003_01273	396	5	16	3	1.509	4.098	0.805	S-adenosyl-L-methionine-binding protein
bin003	SOY3_bin003_01274	882	5	10	1	0.678	1.150	0.120	hypothetical protein
bin003	SOY3_bin003_01275	1617	16	34	6	1.183	2.133	0.394	Adenine deaminase
bin003	SOY3_bin003_01276	1131	2	12	0	0.211	1.076	0.000	putative permease
bin003	SOY3_bin003_01277	366	5	13	4	1.633	3.603	1.161	hypothetical protein
bin003	SOY3_bin003_01278	375	1	0	0	0.319	0.000	0.000	Inner membrane protein YbaN
bin003	SOY3_bin003_01279	231	7	0	1	3.623	0.000	0.460	Ferrous iron transport protein A
bin003	SOY3_bin003_01280	225	5	0	0	2.657	0.000	0.000	FeoA domain protein
bin003	SOY3_bin003_01281	2238	55	9	3	2.938	0.408	0.142	Ferrous iron transport protein B
bin003	SOY3_bin003_01282	117	0	1	0	0.000	0.867	0.000	hypothetical protein
bin003	SOY3_bin003_01283	873	0	5	0	0.000	0.581	0.000	hypothetical protein
bin003	SOY3_bin003_01284	579	0	3	0	0.000	0.526	0.000	tRNA (cmo5U34)-methyltransferase
bin003	SOY3_bin003_01285	327	2	1	0	0.731	0.310	0.000	Sugar-specific transcriptional regulator TrmB
bin003	SOY3_bin003_01286	702	1	1	0	0.170	0.144	0.000	hypothetical protein
bin003	SOY3_bin003_01287	660	0	5	1	0.000	0.768	0.161	MotA/TolQ/ExbB proton channel family protein
bin003	SOY3_bin003_01288	303	1	2	1	0.395	0.669	0.351	flagellar motor protein MotB
bin003	SOY3_bin003_01289	1125	1	12	2	0.106	1.082	0.189	High-affinity heme uptake system protein IsdE precursor

bin003	SOY3_bin003_01290	1044	2	7	3	0.229	0.680	0.305	putative ABC transporter permease protein
bin003	SOY3_bin003_01291	798	1	5	1	0.150	0.636	0.133	putative ABC transporter ATP-binding protein
bin003	SOY3_bin003_01292	573	1	6	0	0.209	1.062	0.000	FmdE, Molybdenum formylmethanofuran dehydrogenase operon
bin003	SOY3_bin003_01293	615	11	30	8	2.138	4.948	1.382	Demethylrebeccamycin-D-glucose O-methyltransferase
bin003	SOY3_bin003_01294	354	3	9	1	1.013	2.579	0.300	Ribbon-helix-helix protein, copG family
bin003	SOY3_bin003_01295	180	2	1	1	1.328	0.563	0.590	hypothetical protein
bin003	SOY3_bin003_01296	723	5	5	1	0.827	0.701	0.147	Macrolide export ATP-binding/permease protein MacB
bin003	SOY3_bin003_01297	1143	1	5	0	0.105	0.444	0.000	putative ABC transporter permease YknZ
bin003	SOY3_bin003_01298	360	13	15	10	4.317	4.226	2.951	hypothetical protein
bin003	SOY3_bin003_01299	285	1	4	1	0.419	1.424	0.373	HTH-type transcriptional repressor CzrA
bin003	SOY3_bin003_01300	690	7	25	6	1.213	3.675	0.924	Anaerobic glycerol-3-phosphate dehydrogenase subunit C
bin003	SOY3_bin003_01301	1221	40	77	13	3.916	6.396	1.131	Lactate utilization protein B
bin003	SOY3_bin003_01302	714	15	24	3	2.512	3.409	0.446	hypothetical protein
bin003	SOY3_bin003_01303	1488	65	149	27	5.222	10.156	1.927	Inosine-5'-monophosphate dehydrogenase
bin003	SOY3_bin003_01304	597	4	12	3	0.801	2.039	0.534	putative molybdenum cofactor guanylyltransferase
bin003	SOY3_bin003_01305	774	5	16	2	0.772	2.097	0.274	tRNA (mo5U34)-methyltransferase
bin003	SOY3_bin003_01306	1647	23	73	15	1.669	4.496	0.967	putative cell division protein YtgP
bin003	SOY3_bin003_01307	351	9	23	2	3.065	6.646	0.605	hypothetical protein
bin003	SOY3_bin003_01308	666	3	4	1	0.539	0.609	0.159	Deoxyribose-phosphate aldolase
bin003	SOY3_bin003_01309	1629	3	22	2	0.220	1.370	0.130	Oxygen-independent coproporphyrinogen-III oxidase 1
bin003	SOY3_bin003_01310	1059	11	21	3	1.242	2.011	0.301	Putative zinc metalloprotease Rip3
bin003	SOY3_bin003_01311	264	9	15	6	4.076	5.763	2.414	PRC-barrel domain protein
bin003	SOY3_bin003_01312	339	22	47	17	7.758	14.062	5.327	hypothetical protein
bin003	SOY3_bin003_01313	615	6	9	3	1.166	1.484	0.518	hypothetical protein
bin003	SOY3_bin003_01314	303	7	19	4	2.762	6.360	1.402	hypothetical protein
bin003	SOY3_bin003_01315	546	2	7	5	0.438	1.300	0.973	hypothetical protein
bin003	SOY3_bin003_01316	372	5	10	4	1.607	2.727	1.142	ski2-like helicase
bin003	SOY3_bin003_01317	843	44	102	30	6.240	12.272	3.780	preprotein translocase subunit SecF
bin003	SOY3_bin003_01318	1011	39	45	11	4.612	4.515	1.156	N-acetyl-gamma-glutamyl-phosphate reductase
bin003	SOY3_bin003_01319	918	27	34	4	3.516	3.757	0.463	Acetylglutamate kinase
bin003	SOY3_bin003_01320	1194	55	65	12	5.507	5.522	1.068	Arginine biosynthesis bifunctional protein ArgJ
bin003	SOY3_bin003_01321	309	23	27	6	8.898	8.863	2.063	hypothetical protein
bin003	SOY3_bin003_01322	1239	112	162	38	10.807	13.262	3.258	hypothetical protein
bin003	SOY3_bin003_01323	1026	146	199	51	17.012	19.673	5.280	Zc3h12a-like Ribonuclease NYN domain protein
bin003	SOY3_bin003_01324	747	38	40	16	6.081	5.431	2.275	3',5'-cyclic adenosine monophosphate phosphodiesterase CpdA
bin003	SOY3_bin003_01325	354	14	26	12	4.728	7.449	3.601	nascent polypeptide-associated complex protein
bin003	SOY3_bin003_01326	1050	19	30	12	2.163	2.898	1.214	H/ACA RNA-protein complex component Cbf5p
bin003	SOY3_bin003_01327	1002	12	12	3	1.432	1.215	0.318	Phosphatidylglycerol lysyltransferase
bin003	SOY3_bin003_01328	684	6	13	4	1.049	1.928	0.621	Undecaprenyl-phosphate mannosyltransferase
bin003	SOY3_bin003_01329	1404	8	9	3	0.681	0.650	0.227	Oligosaccharyl transferase STT3 subunit
bin003	SOY3_bin003_01330	612	7	16	2	1.367	2.652	0.347	HTH-type transcriptional repressor KstR2
bin003	SOY3_bin003_01331	1554	5	16	6	0.385	1.044	0.410	6-pyruvoyl-tetrahydropterin synthase related domain; membrane protein
bin003	SOY3_bin003_01332	1584	9	20	7	0.679	1.281	0.469	N-acetylmuramoyl-L-alanine amidase LytC precursor
bin003	SOY3_bin003_01333	558	9	17	3	1.928	3.090	0.571	Coenzyme A biosynthesis bifunctional protein CoaBC
bin003	SOY3_bin003_01334	1353	44	50	10	3.888	3.748	0.785	Phosphoglucosamine mutase
bin003	SOY3_bin003_01335	699	0	0	0	0.000	0.000	0.000	Energy-coupling factor transporter transmembrane protein EcfT
bin003	SOY3_bin003_01336	1521	1	0	2	0.079	0.000	0.140	Putative HMP/thiamine import ATP-binding protein YkoD
bin003	SOY3_bin003_01337	3603	2	2	0	0.066	0.056	0.000	Aerobic cobaltochelatase subunit CobN
bin003	SOY3_bin003_01338	1125	0	1	0	0.000	0.090	0.000	Adenosylcobinamide amidohydrolase
bin003	SOY3_bin003_01339	642	0	0	0	0.000	0.000	0.000	hypothetical protein
bin003	SOY3_bin003_01340	1770	0	0	0	0.000	0.000	0.000	Putative HMP/thiamine import ATP-binding protein YkoD
bin003	SOY3_bin003_01341	831	4	17	5	0.575	2.075	0.639	Sarcosine/dimethylglycine N-methyltransferase
bin003	SOY3_bin003_01342	1164	15	48	6	1.541	4.183	0.548	Cysteine desulfurase
bin003	SOY3_bin003_01343	381	5	16	4	1.569	4.259	1.115	NifU-like protein
bin003	SOY3_bin003_01344	651	2	8	1	0.367	1.246	0.163	Flagellin N-methylase
bin003	SOY3_bin003_01345	1158	1	12	3	0.103	1.051	0.275	General stress protein 69
bin003	SOY3_bin003_01346	879	32	72	27	4.352	8.308	3.263	Pyridoxal biosynthesis lyase PdxS
bin003	SOY3_bin003_01347	450	12	26	1	3.188	5.860	0.236	MOSC domain protein
bin003	SOY3_bin003_01348	828	1	2	0	0.144	0.245	0.000	Putrescine oxidase
bin003	SOY3_bin003_01349	1161	9	49	7	0.927	4.281	0.640	putative tRNA sulfurtransferase
bin003	SOY3_bin003_01350	1164	1	4	7	0.103	0.349	0.639	biotin synthase
bin003	SOY3_bin003_01351	129	0	1	0	0.000	0.786	0.000	Cystathionine gamma-synthase
bin003	SOY3_bin003_01352	537	6	13	8	1.336	2.455	1.583	CYTH domain protein
bin003	SOY3_bin003_01353	405	3	12	0	0.886	3.005	0.000	hypothetical protein
bin003	SOY3_bin003_01354	546	49	81	24	10.729	15.047	4.669	transcription factor
bin003	SOY3_bin003_01355	1902	98	192	52	6.160	10.239	2.904	Phosphoserine phosphatase
bin003	SOY3_bin003_01356	1524	76	113	29	5.962	7.521	2.021	Multidrug resistance protein stp

bin003	SOY3_bin003_01357	1050	7	13	3	0.797	1.256	0.304	NurA domain protein
bin003	SOY3_bin003_01358	1611	12	19	5	0.890	1.196	0.330	AAA-like domain protein
bin003	SOY3_bin003_01359	372	1	4	1	0.321	1.091	0.286	hypothetical protein
bin003	SOY3_bin003_01360	1044	11	44	9	1.260	4.275	0.916	Aminodeoxyfutalosine synthase
bin003	SOY3_bin003_01361	840	11	25	6	1.566	3.019	0.759	hypothetical protein
bin003	SOY3_bin003_01362	384	5	9	0	1.557	2.377	0.000	Hypoxic response protein 1
bin003	SOY3_bin003_01363	1644	24	22	7	1.745	1.357	0.452	2-oxoglutarate oxidoreductase subunit KorA
bin003	SOY3_bin003_01364	861	11	13	1	1.527	1.531	0.123	2-oxoglutarate oxidoreductase subunit KorB
bin003	SOY3_bin003_01365	366	0	0	1	0.000	0.000	0.290	hypothetical protein
bin003	SOY3_bin003_01366	660	1	2	1	0.181	0.307	0.161	hypothetical protein
bin003	SOY3_bin003_01367	690	4	8	2	0.693	1.176	0.308	hypothetical protein
bin003	SOY3_bin003_01368	909	2	6	2	0.263	0.669	0.234	hypothetical protein
bin003	SOY3_bin003_01369	471	7	19	8	1.777	4.092	1.804	Cyclic pyranopterin monophosphate synthase accessory protein 2
bin003	SOY3_bin003_01370	1071	15	36	10	1.674	3.409	0.992	Putative teichuronic acid biosynthesis glycosyltransferase TuaC
bin003	SOY3_bin003_01371	1095	51	111	26	5.568	10.282	2.522	cobalt-precorrin-6A synthase
bin003	SOY3_bin003_01372	258	11	35	9	5.097	13.760	3.706	Thioredoxin
bin003	SOY3_bin003_01373	882	38	73	15	5.151	8.395	1.807	shikimate kinase
bin003	SOY3_bin003_01374	300	12	31	6	4.782	10.481	2.125	chorismate mutase
bin003	SOY3_bin003_01375	189	14	17	5	8.855	9.123	2.810	30S ribosomal protein S17e
bin003	SOY3_bin003_01376	1221	111	115	33	10.868	9.553	2.871	Aspartokinase
bin003	SOY3_bin003_01377	588	0	1	0	0.000	0.172	0.000	hypothetical protein
bin003	SOY3_bin003_01378	1797	0	0	0	0.000	0.000	0.000	Magnesium-chelatase 38 kDa subunit
bin003	SOY3_bin003_01379	597	1	1	1	0.200	0.170	0.178	tetrahydromethanopterin S-methyltransferase subunit A
bin003	SOY3_bin003_01380	4656	291	962	262	7.472	20.956	5.977	Aerobic cobaltochelatase subunit CobN
bin003	SOY3_bin003_01381	156	0	0	1	0.000	0.000	0.681	hypothetical protein
bin003	SOY3_bin003_01382	279	6	6	2	2.571	2.181	0.761	hypothetical protein
bin003	SOY3_bin003_01383	321	13	31	2	4.842	9.795	0.662	hypothetical protein
bin003	SOY3_bin003_01384	489	3	8	1	0.733	1.659	0.217	Adenosine specific kinase
bin003	SOY3_bin003_01385	543	8	12	5	1.761	2.241	0.978	hypothetical protein
bin003	SOY3_bin003_01386	1683	21	51	11	1.492	3.074	0.694	Acetyl-coenzyme A synthetase
bin003	SOY3_bin003_01387	282	8	8	1	3.391	2.877	0.377	2-oxoglutarate-acceptor oxidoreductase subunit OorD
bin003	SOY3_bin003_01388	1071	22	24	6	2.456	2.273	0.595	2-oxoglutarate oxidoreductase subunit KorA
bin003	SOY3_bin003_01389	1524	68	104	25	5.334	6.922	1.743	2-oxoglutarate oxidoreductase subunit KorB
bin003	SOY3_bin003_01390	471	31	40	18	7.868	8.614	4.060	Response regulator rcp1
bin003	SOY3_bin003_01391	273	7	9	5	3.065	3.344	1.946	hypothetical protein
bin003	SOY3_bin003_01392	831	6	14	2	0.863	1.709	0.256	NADH pyrophosphatase
bin003	SOY3_bin003_01393	774	39	110	24	6.024	14.415	3.294	Aquaporin Z
bin003	SOY3_bin003_01394	210	13	29	6	7.401	14.007	3.035	hypothetical protein
bin003	SOY3_bin003_01395	1500	101	186	28	8.050	12.577	1.983	hypothetical protein
bin003	SOY3_bin003_01396	1197	4	6	1	0.399	0.508	0.089	K(+)/H(+) antiporter NhaP2
bin003	SOY3_bin003_01397	567	5	20	3	1.054	3.578	0.562	2-amino-4-deoxychorismate dehydrogenase
bin003	SOY3_bin003_01398	738	3	9	5	0.486	1.237	0.720	dTDP-3-amino-3,4,6-trideoxy-alpha-D-glucopyranose
bin003	SOY3_bin003_01399	336	2	3	0	0.712	0.906	0.000	Dinitrogenase iron-molybdenum cofactor
bin003	SOY3_bin003_01400	333	18	65	17	6.462	19.798	5.423	hypothetical protein
bin003	SOY3_bin003_01401	657	4	4	1	0.728	0.618	0.162	hypothetical protein
bin003	SOY3_bin003_01402	639	2	5	0	0.374	0.794	0.000	thiamine biosynthesis protein ThiF
bin003	SOY3_bin003_01403	732	1	1	3	0.163	0.139	0.435	Putative GTP cyclohydrolase 1 type 2
bin003	SOY3_bin003_01404	852	1	2	2	0.140	0.238	0.249	hypothetical protein
bin003	SOY3_bin003_01405	480	0	2	1	0.000	0.423	0.221	hypothetical protein
bin003	SOY3_bin003_01406	996	0	1	3	0.000	0.102	0.320	Biotin synthase
bin003	SOY3_bin003_01407	1065	8	33	44	0.898	3.143	4.389	H(2)-dependent methylenetetrahydromethanopterin dehydrogenase
bin003	SOY3_bin003_01408	381	0	2	0	0.000	0.532	0.000	hypothetical protein
bin003	SOY3_bin003_01409	1152	2	4	1	0.208	0.352	0.092	NADH-quinone oxidoreductase chain 3
bin003	SOY3_bin003_01410	690	3	17	2	0.520	2.499	0.308	hypothetical protein
bin003	SOY3_bin003_01411	840	15	33	12	2.135	3.985	1.518	L(+)-tartrate dehydratase subunit alpha
bin003	SOY3_bin003_01412	579	8	15	7	1.652	2.628	1.284	Putative DNA ligase-like protein/MT0965
bin003	SOY3_bin003_01413	663	18	116	25	3.246	17.746	4.005	hypothetical protein
bin003	SOY3_bin003_01414	438	5	6	7	1.365	1.389	1.698	hypothetical protein
bin003	SOY3_bin003_01415	669	9	37	7	1.608	5.610	1.111	hypothetical protein
bin003	SOY3_bin003_01416	759	22	38	4	3.465	5.078	0.560	Phosphate import ATP-binding protein PstB 3
bin003	SOY3_bin003_01417	411	13	52	11	3.781	12.833	2.843	Phosphate transport system permease protein PstA
bin003	SOY3_bin003_01418	630	26	32	7	4.934	5.152	1.180	Ribonuclease J 2
bin003	SOY3_bin003_01419	987	26	29	6	3.149	2.980	0.646	Octaprenyl-diphosphate synthase
bin003	SOY3_bin003_01420	1680	32	41	8	2.277	2.475	0.506	Glutamine--tRNA ligase
bin003	SOY3_bin003_01421	384	109	224	56	33.934	59.166	15.491	HTH-type transcriptional regulator
bin003	SOY3_bin003_01422	2403	8	24	5	0.398	1.013	0.221	Lon protease 1
bin003	SOY3_bin003_01423	1452	4	3	0	0.329	0.210	0.000	replication factor C large subunit

bin003	SOY3_bin003_01424	723	8	15	7	1.323	2.104	1.028	Asparagine synthetase B [glutamine-hydrolyzing]
bin003	SOY3_bin003_01425	216	9	22	5	4.981	10.331	2.459	aspartyl/glutamyl-tRNA amidotransferase subunit C
bin003	SOY3_bin003_01426	489	8	22	6	1.956	4.563	1.303	threonine dehydratase
bin003	SOY3_bin003_01427	1011	40	58	18	4.730	5.819	1.891	Homoserine dehydrogenase
bin003	SOY3_bin003_01428	1221	33	61	10	3.231	5.067	0.870	2,3-bisphosphoglycerate-independent phosphoglycerate mutase
bin003	SOY3_bin003_01429	1611	65	129	23	4.824	8.122	1.517	CTP synthase
bin003	SOY3_bin003_01430	1164	11	17	9	1.130	1.481	0.821	hypothetical protein
bin003	SOY3_bin003_01431	453	28	59	17	7.389	13.210	3.986	Class III signal peptide
bin003	SOY3_bin003_01432	198	1	1	2	0.604	0.512	1.073	hypothetical protein
bin003	SOY3_bin003_01433	74	0	1	1	0.000	1.371	1.435	tRNA-Ala(cgc)
bin003	SOY3_bin003_01434	720	5	16	4	0.830	2.254	0.590	Iron-binding zinc finger CDGSH type
bin003	SOY3_bin003_01435	681	5	21	5	0.878	3.128	0.780	SNARE associated Golgi protein
bin003	SOY3_bin003_01436	876	14	22	2	1.911	2.547	0.243	Cadmium, cobalt and zinc/H(+)-K(+) antiporter
bin003	SOY3_bin003_01437	576	23	32	11	4.774	5.635	2.029	Thymidylate kinase
bin003	SOY3_bin003_01438	417	8	12	4	2.294	2.919	1.019	hypothetical protein
bin003	SOY3_bin003_01439	213	10	14	5	5.613	6.667	2.494	hypothetical protein
bin003	SOY3_bin003_01440	963	27	57	13	3.352	6.003	1.434	Tyrosine--tRNA ligase
bin003	SOY3_bin003_01441	1062	43	42	5	4.840	4.011	0.500	NMD3 family protein
bin003	SOY3_bin003_01442	408	22	29	15	6.446	7.209	3.905	translation initiation factor IF-2 subunit beta
bin003	SOY3_bin003_01443	405	24	16	8	7.084	4.007	2.098	Carbon monoxide dehydrogenase/acetyl-CoA synthase subunit alpha
bin003	SOY3_bin003_01444	756	27	45	10	4.270	6.037	1.405	Septum site-determining protein MinD
bin003	SOY3_bin003_01445	1152	65	90	12	6.745	7.924	1.107	Corrinoid/iron-sulfur protein small subunit
bin003	SOY3_bin003_01446	1386	98	95	18	8.453	6.952	1.380	Corrinoid/iron-sulfur protein large subunit
bin003	SOY3_bin003_01447	474	27	32	10	6.810	6.847	2.241	Anaerobic dimethyl sulfoxide reductase chain B
bin003	SOY3_bin003_01448	600	11	21	8	2.192	3.550	1.416	Methylated-DNA--protein-cysteine methyltransferase
bin003	SOY3_bin003_01449	1362	5	14	2	0.439	1.043	0.156	Phycocyanobilin lyase subunit alpha
bin003	SOY3_bin003_01450	1167	1	2	2	0.102	0.174	0.182	Putative oxidoreductase/MT0587
bin003	SOY3_bin003_01451	1482	9	19	3	0.726	1.300	0.215	Ribonuclease J 2
bin003	SOY3_bin003_01452	312	2	5	0	0.766	1.625	0.000	hypothetical protein
bin003	SOY3_bin003_01453	891	5	9	2	0.671	1.025	0.238	Putative DNA ligase-like protein/MT0965
bin003	SOY3_bin003_01454	771	9	16	4	1.396	2.105	0.551	putative DNA repair protein YkoV
bin003	SOY3_bin003_01455	855	2	6	3	0.280	0.712	0.373	hypothetical protein
bin003	SOY3_bin003_01456	315	3	5	0	1.139	1.610	0.000	Tetraspanin family protein
bin003	SOY3_bin003_01457	867	0	1	0	0.000	0.117	0.000	hypothetical protein
bin003	SOY3_bin003_01458	159	5	11	1	3.759	7.017	0.668	4-hydroxy-tetrahydrodipicolinate synthase
bin003	SOY3_bin003_01459	822	32	63	12	4.654	7.774	1.551	4-hydroxy-tetrahydrodipicolinate reductase
bin003	SOY3_bin003_01460	1047	79	124	31	9.020	12.012	3.145	Aspartate-semialdehyde dehydrogenase 2
bin003	SOY3_bin003_01461	1485	41	68	16	3.301	4.644	1.145	Copper resistance protein CopC
bin003	SOY3_bin003_01462	501	40	78	11	9.545	15.791	2.332	hypothetical protein
bin003	SOY3_bin003_01463	1635	389	977	315	28.443	60.608	20.466	60 kDa chaperonin
bin003	SOY3_bin003_01464	171	0	0	0	0.000	0.000	0.000	hypothetical protein
bin003	SOY3_bin003_01465	549	2	3	1	0.436	0.554	0.193	HTH-type transcriptional regulator AcrR
bin003	SOY3_bin003_01466	195	0	4	2	0.000	2.081	1.089	putative RNA-binding protein
bin003	SOY3_bin003_01467	168	0	3	2	0.000	1.811	1.265	Epoxyqueuosine reductase
bin003	SOY3_bin003_01468	201	0	4	1	0.000	2.018	0.528	hypothetical protein
bin003	SOY3_bin003_01469	525	4	6	0	0.911	1.159	0.000	hypothetical protein
bin003	SOY3_bin003_01470	240	3	2	0	1.494	0.845	0.000	HTH-type transcriptional regulator MgrA
bin003	SOY3_bin003_01471	3735	495	629	345	15.844	17.081	9.812	UDP-Glc:alpha-D-GlcNAc-diphosphoundecaprenol beta-1,3-glucosyltransferase WfgD
bin003	SOY3_bin003_01472	1518	45	90	44	3.544	6.013	3.079	Dolichyl-phosphate-mannose-protein mannosyltransferase
bin003	SOY3_bin003_01473	948	80	128	61	10.089	13.695	6.835	Undecaprenyl-phosphate 4-deoxy-4-formamido-L-arabinose transferase
bin003	SOY3_bin003_01474	267	34	56	31	15.223	21.273	12.333	dTDP-glucose 4,6-dehydratase
bin003	SOY3_bin003_01475	861	43	49	14	5.971	5.772	1.727	Succinyl-CoA ligase [ADP-forming] subunit alpha
bin003	SOY3_bin003_01476	726	26	38	8	4.281	5.309	1.171	hypothetical protein
bin003	SOY3_bin003_01477	924	44	47	9	5.693	5.159	1.035	putative ABC transporter ATP-binding protein YbhF
bin003	SOY3_bin003_01478	717	17	23	7	2.834	3.254	1.037	ABC-2 family transporter protein
bin003	SOY3_bin003_01479	675	12	29	7	2.125	4.358	1.102	3-dehydroquinate dehydratase
bin003	SOY3_bin003_01480	612	5	7	1	0.977	1.160	0.174	Signal peptidase I V
bin003	SOY3_bin003_01481	960	73	132	31	9.091	13.946	3.430	hypothetical protein
bin003	SOY3_bin003_01482	336	1	2	1	0.356	0.604	0.316	Nucleotidyltransferase domain protein
bin003	SOY3_bin003_01483	159	0	2	0	0.000	1.276	0.000	hypothetical protein
bin003	SOY3_bin003_01484	984	35	70	13	4.252	7.215	1.403	Delta-aminolevulinic acid dehydratase
bin003	SOY3_bin003_01485	906	10	14	3	1.320	1.567	0.352	2-(5''-triphosphoribosyl)-3'-dephosphocoenzyme-A synthase
bin003	SOY3_bin003_01486	1527	27	66	19	2.114	4.384	1.322	Phenylalanine--tRNA ligase alpha subunit
bin003	SOY3_bin003_01487	471	6	8	3	1.523	1.723	0.677	Organic hydroperoxide resistance transcriptional regulator
bin003	SOY3_bin003_01488	510	7	9	3	1.641	1.790	0.625	Albonoursin synthase
bin003	SOY3_bin003_01489	570	9	9	4	1.888	1.601	0.745	Flavoredoxin
bin003	SOY3_bin003_01490	252	4	11	2	1.898	4.427	0.843	MTH865-like family protein

bin003	SOY3_bin003_01491	321	0	0	0	0.000	0.000	0.000	hypothetical protein
bin003	SOY3_bin003_01492	1983	90	170	44	5.426	8.695	2.357	hypothetical protein
bin003	SOY3_bin003_01493	882	4	2	0	0.542	0.230	0.000	hypothetical protein
bin003	SOY3_bin003_01494	333	0	1	0	0.000	0.305	0.000	hypothetical protein
bin003	SOY3_bin003_01495	285	2	2	2	0.839	0.712	0.745	Bacterial regulatory protein, arsR family
bin003	SOY3_bin003_01496	819	7	16	1	1.022	1.981	0.130	NADH-plastoquinone oxidoreductase subunit
bin003	SOY3_bin003_01497	468	4	7	5	1.022	1.517	1.135	Flavodoxin
bin003	SOY3_bin003_01498	579	14	32	10	2.891	5.606	1.835	hypothetical protein
bin003	SOY3_bin003_01499	540	1	4	4	0.221	0.751	0.787	2-hydroxy-6-oxo-6-(2'-aminophenyl)hexa-2,4-dienoic acid hydrolase
bin003	SOY3_bin003_01500	1134	12	58	12	1.265	5.188	1.124	GDP-mannose-dependent alpha-(1-2)-phosphatidylinositol mannosyltransferase
bin003	SOY3_bin003_01501	2124	13	33	7	0.732	1.576	0.350	hypothetical protein
bin003	SOY3_bin003_01502	1179	8	12	1	0.811	1.032	0.090	Alpha-D-kanosaminyltransferase
bin003	SOY3_bin003_01503	243	2	16	3	0.984	6.678	1.311	hypothetical protein
bin003	SOY3_bin003_01504	963	9	12	10	1.117	1.264	1.103	Rhamnosyltransferase WbbL
bin003	SOY3_bin003_01505	1020	6	16	6	0.703	1.591	0.625	N-glycosyltransferase
bin003	SOY3_bin003_01506	519	1	12	0	0.230	2.345	0.000	hypothetical protein
bin003	SOY3_bin003_01507	1230	31	42	10	3.013	3.463	0.864	Nitric oxide reductase
bin003	SOY3_bin003_01508	975	2	6	0	0.245	0.624	0.000	Sulfate permease CysP
bin003	SOY3_bin003_01509	627	7	10	5	1.335	1.618	0.847	Protein archease
bin003	SOY3_bin003_01510	651	1	3	0	0.184	0.467	0.000	hypothetical protein
bin003	SOY3_bin003_01511	1164	5	7	5	0.514	0.610	0.456	hypothetical protein
bin003	SOY3_bin003_01512	1320	21	35	6	1.902	2.689	0.483	Magnesium and cobalt efflux protein CorC
bin003	SOY3_bin003_01513	87	1	5	3	1.374	5.829	3.663	tRNA-Ser(gct)
bin003	SOY3_bin003_01514	318	21	30	9	7.895	9.569	3.006	hypothetical protein
bin003	SOY3_bin003_01515	426	5	14	3	1.403	3.333	0.748	Cytochrome b6-f complex subunit 6
bin003	SOY3_bin003_01516	933	7	29	6	0.897	3.153	0.683	UDP-glucose 4-epimerase
bin003	SOY3_bin003_01517	1434	20	24	2	1.667	1.698	0.148	Lipopolysaccharide biosynthesis protein WzxC
bin003	SOY3_bin003_01518	849	5	12	1	0.704	1.434	0.125	Putative glycosyltransferase EpsH
bin003	SOY3_bin003_01519	1155	1	10	2	0.104	0.878	0.184	D-inositol 3-phosphate glycosyltransferase
bin003	SOY3_bin003_01520	228	1	6	0	0.524	2.669	0.000	Transcription factor Pcc1
bin003	SOY3_bin003_01521	789	5	12	4	0.758	1.543	0.539	Septum site-determining protein MinD
bin003	SOY3_bin003_01522	483	36	66	22	8.910	13.860	4.838	50S ribosomal protein L16
bin003	SOY3_bin003_01523	2295	225	311	76	11.720	13.745	3.518	Phosphoenolpyruvate synthase
bin003	SOY3_bin003_01524	1149	32	35	9	3.329	3.090	0.832	Glutamate decarboxylase
bin003	SOY3_bin003_01525	348	2	3	0	0.687	0.874	0.000	Bacterial regulatory protein, arsR family
bin003	SOY3_bin003_01526	618	5	3	0	0.967	0.492	0.000	hypothetical protein
bin003	SOY3_bin003_01527	363	3	3	0	0.988	0.838	0.000	lineage-specific thermal regulator protein
bin003	SOY3_bin003_01528	609	3	2	3	0.589	0.333	0.523	Spermine/spermidine acetyltransferase
bin003	SOY3_bin003_01529	1788	37	52	6	2.474	2.950	0.356	DNA polymerase II
bin003	SOY3_bin003_01530	510	3	6	2	0.703	1.193	0.417	ribosomal-protein-alanine N-acetyltransferase
bin003	SOY3_bin003_01531	699	20	17	2	3.421	2.467	0.304	Epoxyqueuosine reductase
bin003	SOY3_bin003_01532	450	4	8	3	1.063	1.803	0.708	Putative phosphinothricin acetyltransferase YwnH
bin003	SOY3_bin003_01533	513	17	5	3	3.962	0.989	0.621	FMN reductase [NAD(P)H]
bin003	SOY3_bin003_01534	93	25	24	5	32.137	26.175	5.711	hypothetical protein
bin003	SOY3_bin003_01535	549	3	10	2	0.653	1.847	0.387	DNA-binding transcriptional repressor PuuR
bin003	SOY3_bin003_01536	273	0	1	0	0.000	0.372	0.000	Protein MraZ
bin003	SOY3_bin003_01537	447	1	1	0	0.267	0.227	0.000	tRNA(fMet)-specific endonuclease VapC
bin003	SOY3_bin003_01538	1989	27	43	15	1.623	2.193	0.801	formamidopyrimidine/5-formyluracil/ 5-hydroxymethyluracil DNA glycosylase
bin003	SOY3_bin003_01539	279	14	20	4	5.999	7.271	1.523	hypothetical protein
bin003	SOY3_bin003_01540	1485	54	102	19	4.347	6.967	1.359	hypothetical protein
bin003	SOY3_bin003_01541	1257	61	147	27	5.801	11.861	2.282	hypothetical protein
bin003	SOY3_bin003_01542	354	14	31	3	4.728	8.882	0.900	Protease 4
bin003	SOY3_bin003_01543	1053	80	78	22	9.083	7.513	2.219	homoserine dehydrogenase
bin003	SOY3_bin003_01544	348	13	22	12	4.466	6.412	3.663	Roadblock/LC7 domain protein
bin003	SOY3_bin003_01545	966	34	71	5	4.208	7.455	0.550	putative transcription repressor NiaR
bin003	SOY3_bin003_01546	561	4	5	3	0.852	0.904	0.568	ZPR1 zinc-finger domain protein
bin003	SOY3_bin003_01547	468	75	56	17	19.158	12.137	3.859	Histone-like transcription factor (CBF/NF-Y) and archaeal histone
bin003	SOY3_bin003_01548	600	15	17	5	2.989	2.874	0.885	putative nicotinate-nucleotide pyrophosphorylase [carboxylating]
bin003	SOY3_bin003_01549	471	2	9	3	0.508	1.938	0.677	hypothetical protein
bin003	SOY3_bin003_01550	105	0	0	0	0.000	0.000	0.000	hypothetical protein
bin003	SOY3_bin003_01551	723	3	10	1	0.496	1.403	0.147	hypothetical protein
bin003	SOY3_bin003_01552	1197	28	142	28	2.796	12.032	2.485	Pseudomurein-binding repeat protein
bin003	SOY3_bin003_01553	171	0	0	0	0.000	0.000	0.000	hypothetical protein
bin003	SOY3_bin003_01554	1101	80	323	86	8.687	29.756	8.297	Transglutaminase-like superfamily protein
bin003	SOY3_bin003_01555	1071	12	41	10	1.339	3.883	0.992	Transglutaminase-like superfamily protein
bin003	SOY3_bin003_01556	741	0	1	0	0.000	0.137	0.000	Sodium Bile acid symporter family protein
bin003	SOY3_bin003_01557	1218	17	31	9	1.669	2.581	0.785	putative deoxyhypusine synthase

bin003	SOY3_bin003_01558	96	3	5	1	3.736	5.283	1.107	hypothetical protein
bin003	SOY3_bin003_01559	861	38	46	19	5.276	5.419	2.344	Agmatinase
bin003	SOY3_bin003_01560	390	23	37	8	7.050	9.623	2.179	Elongation factor P
bin003	SOY3_bin003_01561	444	5	4	1	1.346	0.914	0.239	pyruvoyl-dependent arginine decarboxylase
bin003	SOY3_bin003_01562	831	12	21	4	1.726	2.563	0.511	Inositol-1-monophosphatase
bin003	SOY3_bin003_01563	402	4	8	4	1.190	2.018	1.057	tRNA(fMet)-specific endonuclease VapC
bin003	SOY3_bin003_01564	291	2	6	1	0.822	2.091	0.365	hypothetical protein
bin003	SOY3_bin003_01565	3498	7	51	10	0.239	1.479	0.304	PKD domain protein
bin003	SOY3_bin003_01566	1044	2	3	2	0.229	0.291	0.203	PKD domain protein
bin003	SOY3_bin003_01567	321	10	11	3	3.724	3.476	0.993	GTPase Era
bin003	SOY3_bin003_01568	423	16	29	2	4.522	6.954	0.502	18 kDa heat shock protein
bin003	SOY3_bin003_01569	561	11	15	4	2.344	2.712	0.757	Bifunctional thiamine biosynthesis protein ThiDN
bin003	SOY3_bin003_01570	1764	301	773	197	20.399	44.446	11.863	Pseudomurein-binding repeat protein
bin003	SOY3_bin003_01571	444	13	22	9	3.500	5.026	2.153	D-beta-D-heptose 1-phosphate adenylyltransferase
bin003	SOY3_bin003_01572	1470	44	92	21	3.578	6.348	1.518	putative bifunctional UDP-N-acetylmuramoylalanyl-D-glutamate--2,6-diaminopimelate ligase/UDP-N-acetylmuramoyl-tripeptide:D-alanyl-D-alanine ligase
bin003	SOY3_bin003_01573	1020	42	134	31	4.923	13.325	3.228	glyceraldehyde-3-phosphate dehydrogenase
bin003	SOY3_bin003_01574	1074	72	85	27	8.014	8.027	2.670	DNA topoisomerase VI subunit A
bin003	SOY3_bin003_01575	1599	89	106	30	6.654	6.724	1.993	DNA topoisomerase VI subunit B
bin003	SOY3_bin003_01576	582	25	31	11	5.135	5.402	2.008	polynucleotide phosphorylase/polyadenylase
bin003	SOY3_bin003_01577	768	31	14	3	4.826	1.849	0.415	bifunctional UGMP family protein/serine/threonine protein kinase
bin003	SOY3_bin003_01578	306	23	20	2	8.986	6.629	0.694	translation initiation factor IF-1A
bin003	SOY3_bin003_01579	1239	20	30	10	1.930	2.456	0.857	hypothetical protein
bin003	SOY3_bin003_01580	465	0	1	2	0.000	0.218	0.457	hypothetical protein
bin003	SOY3_bin003_01581	1299	5	9	3	0.460	0.703	0.245	Dihydroorotase
bin003	SOY3_bin003_01582	2742	147	444	91	6.409	16.424	3.525	hypothetical protein
bin003	SOY3_bin003_01583	552	5	7	2	1.083	1.286	0.385	hypothetical protein
bin003	SOY3_bin003_01584	444	1	9	1	0.269	2.056	0.239	hypothetical protein
bin003	SOY3_bin003_01585	1362	42	107	30	3.687	7.968	2.340	Phosphoribosylformylglycinamidine synthase 2
bin003	SOY3_bin003_01586	612	11	10	4	2.149	1.657	0.694	Imidazole glycerol phosphate synthase subunit HisH 1
bin003	SOY3_bin003_01587	1008	29	43	11	3.439	4.327	1.159	D-glycero-alpha-D-manno-heptose 1-phosphate guanylyltransferase
bin003	SOY3_bin003_01588	1086	32	43	12	3.523	4.016	1.174	Nitrogenase molybdenum-iron protein alpha chain
bin003	SOY3_bin003_01589	552	11	14	3	2.382	2.572	0.577	Bacterial transcription activator, effector binding domain
bin003	SOY3_bin003_01590	954	2	9	1	0.251	0.957	0.111	putative tRNA (adenine(37)-N6)-methyltransferase
bin003	SOY3_bin003_01591	384	0	2	1	0.000	0.528	0.277	putative HTH-type transcriptional regulator YgaV
bin003	SOY3_bin003_01592	960	8	5	2	0.996	0.528	0.221	UDP-N-acetylbacillosamine transaminase
bin003	SOY3_bin003_01593	498	3	7	3	0.720	1.426	0.640	Kinase binding protein CGI-121
bin003	SOY3_bin003_01594	1503	39	59	19	3.102	3.982	1.343	2-isopropylmalate synthase
bin003	SOY3_bin003_01595	606	8	9	5	1.578	1.506	0.876	Modification methylase VspI
bin003	SOY3_bin003_01596	5520	56	178	34	1.213	3.271	0.654	Microbial collagenase precursor
bin003	SOY3_bin003_01597	1140	3	39	13	0.315	3.470	1.211	Inner membrane transport permease YbhR
bin003	SOY3_bin003_01598	1077	7	30	7	0.777	2.825	0.690	putative ABC transporter ATP-binding protein YbhF
bin003	SOY3_bin003_01599	486	2	14	4	0.492	2.922	0.874	Transcriptional regulator YqjI
bin003	SOY3_bin003_01600	771	5	3	5	0.775	0.395	0.689	endonuclease IV
bin003	SOY3_bin003_01601	1107	27	30	11	2.916	2.749	1.056	Ferredoxin
bin003	SOY3_bin003_01602	627	4	11	5	0.763	1.779	0.847	hypothetical protein
bin003	SOY3_bin003_01603	429	77	348	52	21.457	82.277	12.876	sn-glycerol-3-phosphate dehydrogenase subunit C
bin003	SOY3_bin003_01604	429	11	55	11	3.065	13.003	2.724	Methyl-viologen-reducing hydrogenase, delta subunit
bin003	SOY3_bin003_01605	1200	81	340	99	8.070	28.738	8.764	coenzyme F420-reducing hydrogenase subunit beta
bin003	SOY3_bin003_01606	528	12	34	9	2.717	6.531	1.811	Beta-carbonic anhydrase 1
bin003	SOY3_bin003_01607	1092	134	230	68	14.670	21.363	6.615	hypothetical protein
bin003	SOY3_bin003_01608	1446	6	19	5	0.496	1.333	0.367	Inner membrane protein YccS
bin003	SOY3_bin003_01609	546	22	28	3	4.817	5.201	0.584	hypothetical protein
bin003	SOY3_bin003_01610	837	52	61	17	7.427	7.392	2.158	Inosine-5'-monophosphate dehydrogenase
bin003	SOY3_bin003_01611	450	17	24	10	4.516	5.409	2.361	Putative universal stress protein
bin003	SOY3_bin003_01612	1158	37	38	12	3.820	3.328	1.101	Isoxanthopterin deaminase
bin003	SOY3_bin003_01613	2214	95	123	36	5.130	5.635	1.727	Carbamoyl-phosphate synthase large chain
bin003	SOY3_bin003_01614	615	23	29	12	4.471	4.783	2.073	hypothetical protein
bin003	SOY3_bin003_01615	1254	61	106	20	5.815	8.574	1.694	Adenosylhomocysteinase
bin003	SOY3_bin003_01616	2175	62	39	7	3.408	1.819	0.342	ATP-dependent zinc metalloprotease FtsH
bin003	SOY3_bin003_01617	444	2	3	2	0.539	0.685	0.478	hypothetical protein
bin003	SOY3_bin003_01618	417	9	6	8	2.580	1.459	2.038	prephenate dehydrogenase
bin003	SOY3_bin003_01619	720	102	284	161	16.936	40.007	23.753	hypothetical protein
bin003	SOY3_bin003_01620	1038	111	220	143	12.784	21.497	14.634	NurA domain protein
bin003	SOY3_bin003_01621	1623	282	570	368	20.772	35.621	24.086	AAA-like domain protein
bin003	SOY3_bin003_01622	1149	111	246	110	11.549	21.716	10.170	Nuclease SbcCD subunit D
bin003	SOY3_bin003_01623	1104	2	13	2	0.217	1.194	0.192	D-alanyl-D-alanine-carboxypeptidase/endopeptidase AmpH precursor
bin003	SOY3_bin003_01624	759	7	8	1	1.103	1.069	0.140	Non-heme chloroperoxidase

bin003	SOY3_bin003_01625	1113	26	41	8	2.793	3.736	0.764	Nitric oxide reductase
bin003	SOY3_bin003_01626	657	1	1	2	0.182	0.154	0.323	Demethylrebeccamycin-D-glucose O-methyltransferase
bin003	SOY3_bin003_01627	300	0	0	0	0.000	0.000	0.000	putative metallo-hydrolase YflN
bin003	SOY3_bin003_01628	402	0	1	1	0.000	0.252	0.264	putative metallo-hydrolase YflN
bin003	SOY3_bin003_01629	366	0	0	0	0.000	0.000	0.000	sn-glycerol-3-phosphate dehydrogenase subunit C
bin003	SOY3_bin003_01630	1773	62	140	34	4.181	8.009	2.037	hypothetical protein
bin003	SOY3_bin003_01631	717	90	139	38	15.006	19.663	5.630	hypothetical protein
bin003	SOY3_bin003_01632	693	8	8	2	1.380	1.171	0.307	hypothetical protein
bin003	SOY3_bin003_01633	798	12	53	10	1.798	6.736	1.331	Polysialic acid transport protein KpsM
bin003	SOY3_bin003_01634	1179	16	43	10	1.622	3.699	0.901	Teichoic acids export ATP-binding protein TagH
bin003	SOY3_bin003_01635	972	11	25	4	1.353	2.609	0.437	N-acetylglucosaminyl-diphospho-decaprenol L-rhamnosyltransferase
bin003	SOY3_bin003_01636	1239	24	52	10	2.316	4.257	0.857	hypothetical protein
bin003	SOY3_bin003_01637	270	9	28	4	3.985	10.518	1.574	hypothetical protein
bin003	SOY3_bin003_01638	876	17	75	13	2.320	8.684	1.576	Phosphate transport system permease protein PstC
bin003	SOY3_bin003_01639	804	6	15	5	0.892	1.892	0.661	fructoselysine 3-epimerase
bin003	SOY3_bin003_01640	792	120	789	150	18.113	101.043	20.119	Phosphate-binding protein PstS 1 precursor
bin003	SOY3_bin003_01641	786	1	3	0	0.152	0.387	0.000	Citrate synthase 2
bin003	SOY3_bin003_01642	927	0	3	0	0.000	0.328	0.000	Antiseptic resistance protein
bin003	SOY3_bin003_01643	678	12	36	10	2.116	5.386	1.567	hypothetical protein
bin003	SOY3_bin003_01644	789	1	0	0	0.152	0.000	0.000	3-oxoadipate enol-lactonase 2
bin003	SOY3_bin003_01645	765	10	15	2	1.563	1.989	0.278	Precorrin-6A reductase
bin003	SOY3_bin003_01646	2361	53	115	37	2.684	4.940	1.665	Calcium-transporting ATPase 1
bin003	SOY3_bin003_01647	267	8	13	1	3.582	4.938	0.398	precorrin-2 dehydrogenase
bin003	SOY3_bin003_01648	537	15	31	7	3.339	5.855	1.385	hypothetical protein
bin003	SOY3_bin003_01649	669	16	27	5	2.859	4.093	0.794	3',5'-cyclic adenosine monophosphate phosphodiesterase CpdA
bin003	SOY3_bin003_01650	894	47	58	16	6.285	6.580	1.901	2-phosphoglycerate kinase
bin003	SOY3_bin003_01651	393	22	31	7	6.692	8.001	1.892	Inosine-5'-monophosphate dehydrogenase
bin003	SOY3_bin003_01652	1152	49	64	20	5.085	5.635	1.844	putative sensor histidine kinase pdtaS
bin003	SOY3_bin003_01653	441	14	24	6	3.795	5.520	1.445	Response regulator rcp1
bin003	SOY3_bin003_01654	756	2	2	1	0.316	0.268	0.141	Energy-coupling factor transporter ATP-binding protein EcfA3
bin003	SOY3_bin003_01655	693	1	4	0	0.173	0.585	0.000	Nickel/cobalt efflux system RcnA
bin003	SOY3_bin003_01656	846	17	59	8	2.402	7.074	1.004	Energy-coupling factor transporter ATP-binding protein EcfA3
bin003	SOY3_bin003_01657	387	15	40	12	4.634	10.483	3.294	Ferredoxin-2
bin003	SOY3_bin003_01658	1539	63	148	32	4.894	9.754	2.209	Inosine-5'-monophosphate dehydrogenase
bin003	SOY3_bin003_01659	342	15	81	23	5.243	24.022	7.144	hypothetical protein
bin003	SOY3_bin003_01660	1011	22	49	12	2.601	4.916	1.261	50S ribosomal protein L3
bin003	SOY3_bin003_01661	789	103	144	38	15.607	18.511	5.116	50S ribosomal protein L4
bin003	SOY3_bin003_01662	261	27	42	15	12.367	16.322	6.105	50S ribosomal protein L23
bin003	SOY3_bin003_01663	726	63	100	28	10.374	13.971	4.097	50S ribosomal protein L2
bin003	SOY3_bin003_01664	411	42	57	9	12.217	14.067	2.326	30S ribosomal protein S19
bin003	SOY3_bin003_01665	459	65	99	25	16.930	21.876	5.786	50S ribosomal protein L22
bin003	SOY3_bin003_01666	1122	49	64	16	5.221	5.786	1.515	30S ribosomal protein S3
bin003	SOY3_bin003_01667	399	0	22	1	0.000	5.592	0.266	hypothetical protein
bin003	SOY3_bin003_01668	684	33	46	16	5.768	6.821	2.485	hypothetical protein
bin003	SOY3_bin003_01669	1209	52	58	19	5.142	4.866	1.669	2,3-bisphosphoglycerate-independent phosphoglycerate mutase
bin003	SOY3_bin003_01670	1353	45	75	17	3.976	5.622	1.335	Phosphoglucosamine mutase
bin003	SOY3_bin003_01671	1278	41	52	17	3.835	4.127	1.413	Bifunctional protein GlmU
bin003	SOY3_bin003_01672	966	15	22	4	1.856	2.310	0.440	putative undecaprenyl-phosphate N-acetylglucosaminyl 1-phosphate transferase
bin003	SOY3_bin003_01673	468	9	20	6	2.299	4.334	1.362	RNA polymerase sigma factor
bin003	SOY3_bin003_01674	600	12	21	5	2.391	3.550	0.885	hypothetical protein
bin003	SOY3_bin003_01675	951	6	44	11	0.754	4.693	1.229	hypothetical protein
bin003	SOY3_bin003_01676	408	1	20	7	0.293	4.972	1.823	hypothetical protein
bin003	SOY3_bin003_01677	78	16	31	8	24.523	40.311	10.895	tRNA-Ala(tgc)
bin003	SOY3_bin003_01678	2957	243221	142217	105936	9833.209	4878.148	3805.591	23S ribosomal RNA
bin003	SOY3_bin003_01679	120	8973	8292	4018	8939.268	7008.625	3556.792	5S ribosomal RNA
bin003	SOY3_bin003_01680	939	8	12	2	1.019	1.296	0.226	Daunorubicin/doxorubicin resistance ATP-binding protein DrrA
bin003	SOY3_bin003_01681	1263	17	28	11	1.609	2.249	0.925	Inner membrane transport permease YbhS
bin003	SOY3_bin003_01682	582	4	16	2	0.822	2.788	0.365	putative methyltransferase YcgJ
bin003	SOY3_bin003_01683	732	30	33	10	4.900	4.573	1.451	Queuine tRNA-ribosyltransferase
bin003	SOY3_bin003_01684	522	0	0	0	0.000	0.000	0.000	CDP-diacylglycerol--glycerol-3-phosphate 3-phosphatidyltransferase
bin003	SOY3_bin003_01685	1248	7	11	3	0.671	0.894	0.255	putative amino acid permease YhdG
bin003	SOY3_bin003_01686	1416	35	69	18	2.955	4.942	1.350	Poly(beta-D-mannuronate) C5 epimerase 6
bin003	SOY3_bin003_01687	1551	27	50	15	2.081	3.270	1.027	(Dimethylallyl)adenosine tRNA methylthiotransferase MiaB
bin003	SOY3_bin003_01688	324	0	0	0	0.000	0.000	0.000	hypothetical protein
bin003	SOY3_bin003_01689	609	1	4	0	0.196	0.666	0.000	Ribosomal protein L11 methyltransferase
bin003	SOY3_bin003_01690	1551	56	98	23	4.316	6.409	1.575	Biotin carboxylase
bin003	SOY3_bin003_01691	864	22	33	11	3.044	3.874	1.352	Bifunctional ligase/repressor BirA

bin003	SOY3_bin003_01692	924	5	9	2	0.647	0.988	0.230	RNA 3'-terminal phosphate cyclase
bin003	SOY3_bin003_01693	648	5	5	5	0.922	0.783	0.820	TfuA-like protein
bin003	SOY3_bin003_01694	477	14	13	0	3.509	2.764	0.000	tRNA 2'-O-methylase
bin003	SOY3_bin003_01695	489	70	99	31	17.113	20.534	6.734	transcription initiation factor E subunit alpha
bin003	SOY3_bin003_01696	852	0	8	2	0.000	0.952	0.249	Coenzyme F420:L-glutamate ligase
bin003	SOY3_bin003_01697	702	9	12	3	1.533	1.734	0.454	tRNA nucleotidyltransferase, second domain
bin003	SOY3_bin003_01698	981	22	27	6	2.681	2.792	0.650	DNA primase small subunit
bin003	SOY3_bin003_01699	1383	23	33	10	1.988	2.420	0.768	DNA primase large subunit
bin003	SOY3_bin003_01700	594	58	91	28	11.673	15.539	5.007	tRNA-specific 2-thiouridylase MnmA
bin003	SOY3_bin003_01701	348	38	66	17	13.054	19.236	5.189	hypothetical protein
bin003	SOY3_bin003_01702	438	40	81	20	10.918	18.757	4.850	30S ribosomal protein S19e
bin003	SOY3_bin003_01703	264	30	23	4	13.585	8.836	1.609	RNA-binding protein
bin003	SOY3_bin003_01704	372	43	47	18	13.819	12.815	5.140	ribonuclease P protein component 4
bin003	SOY3_bin003_01705	537	15	23	7	3.339	4.344	1.385	Shikimate kinase
bin003	SOY3_bin003_01706	777	13	33	2	2.000	4.308	0.273	ribulose-1,5-biphosphate synthetase
bin003	SOY3_bin003_01707	639	86	150	76	16.090	23.809	12.634	50S ribosomal protein L1
bin003	SOY3_bin003_01708	489	23	34	10	5.623	7.052	2.172	50S ribosomal protein L11
bin003	SOY3_bin003_01709	453	15	35	9	3.959	7.837	2.110	transcription antitermination protein NusG
bin003	SOY3_bin003_01710	186	6	15	2	3.856	8.180	1.142	preprotein translocase subunit SecE
bin003	SOY3_bin003_01711	1149	235	573	171	24.451	50.581	15.809	Cell division protein FtsZ
bin003	SOY3_bin003_01712	921	24	37	13	3.115	4.075	1.499	Histidinol-phosphate aminotransferase
bin003	SOY3_bin003_01713	702	15	17	5	2.554	2.456	0.757	Pyruvate formate-lyase 1-activating enzyme
bin003	SOY3_bin003_01714	768	12	17	8	1.868	2.245	1.107	TPR repeat-containing protein YrrB
bin003	SOY3_bin003_01715	441	10	21	6	2.711	4.830	1.445	hypothetical protein
bin003	SOY3_bin003_01716	657	14	24	3	2.547	3.705	0.485	Biotin transporter BioY
bin003	SOY3_bin003_01717	1008	79	134	34	9.369	13.483	3.583	preprotein translocase subunit SecD
bin003	SOY3_bin003_01718	465	46	83	18	11.826	18.104	4.112	Aspartate carbamoyltransferase regulatory chain
bin003	SOY3_bin003_01719	411	7	7	2	2.036	1.727	0.517	hypothetical protein
bin003	SOY3_bin003_01720	504	9	17	4	2.135	3.421	0.843	CTP pyrophosphohydrolase
bin003	SOY3_bin003_01721	402	9	13	5	2.676	3.280	1.321	Nucleotidyltransferase domain protein
bin003	SOY3_bin003_01722	420	4	2	2	1.139	0.483	0.506	hypothetical protein
bin003	SOY3_bin003_01723	2499	119	243	58	5.693	9.863	2.465	Undecaprenyl-diphosphooligosaccharide--protein glycotransferase
bin003	SOY3_bin003_01724	915	17	45	13	2.221	4.988	1.509	DNA topoisomerase 1
bin003	SOY3_bin003_01725	366	27	44	21	8.819	12.193	6.095	hypothetical protein
bin003	SOY3_bin003_01726	555	14	23	5	3.016	4.203	0.957	hypothetical protein
bin003	SOY3_bin003_01727	465	12	19	2	3.085	4.144	0.457	hypothetical protein
bin003	SOY3_bin003_01728	117	2	8	1	2.044	6.935	0.908	hypothetical protein
bin003	SOY3_bin003_01729	597	11	25	5	2.203	4.247	0.890	Orotate phosphoribosyltransferase
bin003	SOY3_bin003_01730	954	16	25	10	2.005	2.658	1.113	1,5-anhydro-D-fructose reductase
bin003	SOY3_bin003_01731	870	11	34	8	1.512	3.964	0.977	Porphobilinogen deaminase
bin003	SOY3_bin003_01732	693	15	22	1	2.588	3.220	0.153	UDP-N-acetylmuramate--L-alanine ligase
bin003	SOY3_bin003_01733	699	15	22	5	2.565	3.192	0.760	Cobalt-precorrin-2 C(20)-methyltransferase
bin003	SOY3_bin003_01734	786	13	24	5	1.977	3.097	0.676	V4R domain protein
bin003	SOY3_bin003_01735	1224	6	36	5	0.586	2.983	0.434	Nitric oxide reductase
bin003	SOY3_bin003_01736	435	8	23	6	2.199	5.363	1.465	hypothetical protein
bin003	SOY3_bin003_01737	1131	29	62	17	3.065	5.560	1.597	Acyltransferase family protein
bin003	SOY3_bin003_01738	600	1	6	0	0.199	1.014	0.000	Ribosomal RNA large subunit methyltransferase E
bin003	SOY3_bin003_01739	534	3	20	2	0.672	3.799	0.398	hypothetical protein
bin003	SOY3_bin003_01740	342	3	13	1	1.049	3.855	0.311	hypothetical protein
bin003	SOY3_bin003_01741	936	64	89	34	8.174	9.644	3.859	transcription initiation factor IIB
bin003	SOY3_bin003_01742	2331	40	68	21	2.051	2.959	0.957	Phytochrome-like protein cph1
bin003	SOY3_bin003_01743	180	0	0	0	0.000	0.000	0.000	hypothetical protein
bin003	SOY3_bin003_01744	885	5	2	0	0.675	0.229	0.000	Ferredoxin-2
bin003	SOY3_bin003_01745	669	2	1	1	0.357	0.152	0.159	HTH-type transcriptional repressor KstR2
bin003	SOY3_bin003_01746	444	25	25	5	6.731	5.711	1.196	hypothetical protein
bin003	SOY3_bin003_01747	465	14	25	8	3.599	5.453	1.828	Peptidase C39 family protein
bin003	SOY3_bin003_01748	1176	22	52	15	2.236	4.485	1.355	2-isopropylmalate synthase
bin003	SOY3_bin003_01749	1245	31	77	17	2.977	6.273	1.450	2,3-dimethylmalate dehydratase large subunit
bin003	SOY3_bin003_01750	534	8	16	6	1.791	3.039	1.194	Chorismate pyruvate-lyase
bin003	SOY3_bin003_01751	987	9	26	6	1.090	2.672	0.646	flap endonuclease-1
bin003	SOY3_bin003_01752	444	18	14	4	4.847	3.198	0.957	Lactoylglutathione lyase
bin003	SOY3_bin003_01753	153	6	4	0	4.688	2.652	0.000	hypothetical protein
bin003	SOY3_bin003_01754	1722	46	64	21	3.194	3.770	1.295	Glycine--tRNA ligase
bin003	SOY3_bin003_01755	615	14	23	6	2.721	3.793	1.036	Deoxycytidine triphosphate deaminase
bin003	SOY3_bin003_01756	783	17	18	3	2.596	2.332	0.407	hypothetical protein
bin003	SOY3_bin003_01757	234	50	105	17	25.545	45.512	7.717	23S rRNA (uracil-C(5))-methyltransferase RlmCD
bin003	SOY3_bin003_01758	651	13	22	6	2.387	3.428	0.979	Orotidine 5'-phosphate decarboxylase

bin003	SOY3_bin003_01759	618	15	12	10	2.902	1.969	1.719	hypothetical protein
bin003	SOY3_bin003_01760	924	20	24	3	2.588	2.634	0.345	putative deoxyhypusine synthase
bin003	SOY3_bin003_01761	882	43	47	10	5.828	5.405	1.204	Arabinose 5-phosphate isomerase KdsD
bin003	SOY3_bin003_01762	432	13	17	3	3.598	3.991	0.738	Nucleotidyltransferase domain protein
bin003	SOY3_bin003_01763	429	7	11	3	1.951	2.601	0.743	Nucleotidyltransferase substrate binding protein like protein
bin003	SOY3_bin003_01764	1758	12	44	12	0.816	2.539	0.725	Long-chain-fatty-acid--CoA ligase
bin003	SOY3_bin003_01765	699	8	11	6	1.368	1.596	0.912	branched-chain alpha-keto acid dehydrogenase subunit E2
bin003	SOY3_bin003_01766	213	3	6	1	1.684	2.857	0.499	hypothetical protein
bin003	SOY3_bin003_01767	624	10	8	1	1.916	1.300	0.170	HTH-type transcriptional regulator AcrR
bin003	SOY3_bin003_01768	1737	31	55	18	2.134	3.212	1.101	Antiseptic resistance protein
bin003	SOY3_bin003_01769	969	18	42	5	2.221	4.396	0.548	UDP-glucose 4-epimerase
bin003	SOY3_bin003_01770	255	7	15	1	3.282	5.966	0.417	hypothetical protein
bin003	SOY3_bin003_01771	489	4	3	1	0.978	0.622	0.217	Methyl-coenzyme M reductase operon protein C
bin003	SOY3_bin003_01772	513	3	2	0	0.699	0.395	0.000	tRNA sulfurtransferase
bin003	SOY3_bin003_01773	465	0	0	0	0.000	0.000	0.000	hypothetical protein
bin003	SOY3_bin003_01774	513	1	1	0	0.233	0.198	0.000	hypothetical protein
bin003	SOY3_bin003_01775	828	20	42	9	2.888	5.145	1.155	Glutamine transport ATP-binding protein GlnQ
bin003	SOY3_bin003_01776	477	13	38	6	3.258	8.080	1.336	hypothetical protein
bin003	SOY3_bin003_01777	618	12	21	3	2.321	3.447	0.516	Precorrin-2 dehydrogenase
bin003	SOY3_bin003_01778	1194	20	27	7	2.002	2.294	0.623	Glutamyl-tRNA reductase
bin003	SOY3_bin003_01779	432	54	83	17	14.944	19.487	4.180	hypothetical protein
bin003	SOY3_bin003_01780	510	51	65	28	11.955	12.927	5.832	50S ribosomal protein L5
bin003	SOY3_bin003_01781	729	79	113	29	12.955	15.722	4.226	30S ribosomal protein S4e
bin003	SOY3_bin003_01782	354	35	76	13	11.820	21.775	3.901	50S ribosomal protein L24
bin003	SOY3_bin003_01783	399	47	49	18	14.082	12.456	4.792	50S ribosomal protein L14
bin003	SOY3_bin003_01784	318	43	60	18	16.165	19.137	6.013	30S ribosomal protein S17
bin003	SOY3_bin003_01785	285	37	45	16	15.520	16.015	5.964	ribonuclease P protein component 1
bin003	SOY3_bin003_01786	342	47	77	25	16.429	22.836	7.765	translation initiation factor Sui1
bin003	SOY3_bin003_01787	213	40	59	10	22.450	28.095	4.987	50S ribosomal protein L29
bin003	SOY3_bin003_01788	1038	4	4	0	0.461	0.391	0.000	2-dehydropantoate 2-reductase
bin003	SOY3_bin003_01789	813	3	4	1	0.441	0.499	0.131	putative acetoacetate decarboxylase
bin003	SOY3_bin003_01790	795	0	2	0	0.000	0.255	0.000	putative acetoacetate decarboxylase
bin003	SOY3_bin003_01791	834	1	0	0	0.143	0.000	0.000	putative oxidoreductase
bin003	SOY3_bin003_01792	447	51	114	23	13.640	25.867	5.466	hypothetical protein
bin003	SOY3_bin003_01793	723	45	91	30	7.441	12.766	4.408	Phosphatidylcholine synthase
bin003	SOY3_bin003_01794	630	16	28	5	3.036	4.508	0.843	hypothetical protein
bin003	SOY3_bin003_01795	1074	81	181	46	9.016	17.093	4.550	Rod shape-determining protein MreB
bin003	SOY3_bin003_01796	420	55	68	18	15.655	16.422	4.553	Ribonuclease M5
bin003	SOY3_bin003_01797	1161	63	90	24	6.487	7.863	2.196	DNA primase
bin003	SOY3_bin003_01798	1512	163	173	37	12.888	11.605	2.599	ATP-dependent zinc metalloprotease FtsH 1
bin003	SOY3_bin003_01799	192	12	29	7	7.472	15.320	3.873	2-hydroxymuconate tautomerase
bin003	SOY3_bin003_01800	2100	45	60	16	2.562	2.898	0.809	Cellobiose 2-epimerase
bin003	SOY3_bin003_01801	384	9	4	1	2.802	1.057	0.277	Putative nickel-responsive regulator
bin003	SOY3_bin003_01802	1281	20	26	7	1.866	2.059	0.580	Phosphomethylpyrimidine synthase
bin003	SOY3_bin003_01803	279	15	21	4	6.427	7.634	1.523	transcriptional regulator Spx
bin003	SOY3_bin003_01804	516	29	31	5	6.719	6.093	1.029	Reverse rubrerythrin-2
bin003	SOY3_bin003_01805	168	0	0	0	0.000	0.000	0.000	hypothetical protein
bin003	SOY3_bin003_01806	372	3	5	1	0.964	1.363	0.286	hypothetical protein
bin003	SOY3_bin003_01807	600	0	9	1	0.000	1.521	0.177	Sulfate transport system permease protein CysW
bin003	SOY3_bin003_01808	1107	6	8	2	0.648	0.733	0.192	Putative 2-aminoethylphosphonate import ATP-binding protein PhnT
bin003	SOY3_bin003_01809	693	6	19	9	1.035	2.781	1.380	DNA-binding transcriptional regulator ModE
bin003	SOY3_bin003_01810	315	11	21	4	4.175	6.762	1.349	hypothetical protein
bin003	SOY3_bin003_01811	300	3	18	3	1.195	6.086	1.062	hypothetical protein
bin003	SOY3_bin003_01812	1704	12	49	7	0.842	2.917	0.436	Succinate dehydrogenase flavoprotein subunit
bin003	SOY3_bin003_01813	1434	15	32	8	1.251	2.263	0.593	Fumarate hydratase class II
bin003	SOY3_bin003_01814	927	13	56	11	1.677	6.127	1.260	hypothetical protein
bin003	SOY3_bin003_01815	168	15	14	5	10.674	8.452	3.161	DNA-directed RNA polymerase subunit N
bin003	SOY3_bin003_01816	216	13	15	1	7.195	7.044	0.492	DNA-directed RNA polymerase subunit K
bin003	SOY3_bin003_01817	1257	106	146	31	10.081	11.781	2.620	Enolase
bin003	SOY3_bin003_01818	192	12	14	6	7.472	7.396	3.320	Ferredoxin-3
bin003	SOY3_bin003_01819	597	49	81	19	9.812	13.761	3.381	30S ribosomal protein S2
bin003	SOY3_bin003_01820	213	14	12	5	7.858	5.714	2.494	Ferredoxin-2
bin003	SOY3_bin003_01821	375	4	1	3	1.275	0.270	0.850	Carboxymuconolactone decarboxylase family protein
bin003	SOY3_bin003_01822	492	36	50	16	8.747	10.308	3.454	Rubrerythrin-2
bin003	SOY3_bin003_01823	378	4	8	3	1.265	2.147	0.843	hypothetical protein
bin003	SOY3_bin003_01824	363	14	54	5	4.611	15.088	1.463	hypothetical protein
bin003	SOY3_bin003_01825	369	12	21	3	3.888	5.772	0.864	hypothetical protein

bin003	SOY3_bin003_01826	459	17	46	15	4.428	10.165	3.471	Sortase family protein
bin003	SOY3_bin003_01827	384	23	39	15	7.160	10.301	4.149	putative transcriptional regulatory protein pdtaR
bin003	SOY3_bin003_01828	1203	42	28	13	4.174	2.361	1.148	Nitrogenase molybdenum-iron protein alpha chain
bin003	SOY3_bin003_01829	276	22	42	10	9.529	15.435	3.849	DNA/RNA-binding protein albA
bin003	SOY3_bin003_01830	978	5	38	11	0.611	3.941	1.195	Outer membrane protein assembly factor BamB precursor
bin003	SOY3_bin003_01831	71	0	0	0	0.000	0.000	0.000	tRNA-Cys(gca)
bin003	SOY3_bin003_01832	1308	1	7	2	0.091	0.543	0.162	Methionine gamma-lyase
bin003	SOY3_bin003_01833	1476	22	29	5	1.782	1.993	0.360	Homoserine O-acetyltransferase
bin003	SOY3_bin003_01834	156	19	45	10	14.560	29.258	6.809	50S ribosomal protein L39e
bin003	SOY3_bin003_01835	246	19	50	15	9.233	20.615	6.477	50S ribosomal protein L31e
bin003	SOY3_bin003_01836	675	80	96	38	14.169	14.425	5.980	translation initiation factor IF-6
bin003	SOY3_bin003_01837	231	27	31	4	13.973	13.611	1.839	50S ribosomal protein LX
bin003	SOY3_bin003_01838	435	28	59	22	7.695	13.757	5.372	prefoldin subunit alpha
bin003	SOY3_bin003_01839	1215	45	78	19	4.428	6.511	1.661	Signal recognition particle receptor FtsY
bin003	SOY3_bin003_01840	177	3	4	3	2.026	2.292	1.800	hypothetical protein
bin003	SOY3_bin003_01841	492	1	7	5	0.243	1.443	1.080	hypothetical protein
bin003	SOY3_bin003_01842	258	2	13	1	0.927	5.111	0.412	hypothetical protein
bin003	SOY3_bin003_01843	654	11	13	1	2.011	2.016	0.162	Leucine efflux protein
bin003	SOY3_bin003_01844	993	18	52	4	2.167	5.311	0.428	L-lysine cyclodeaminase
bin003	SOY3_bin003_01845	792	2	7	2	0.302	0.896	0.268	Sulfate transport system permease protein CysW
bin003	SOY3_bin003_01846	1047	4	12	3	0.457	1.162	0.304	Spermidine/putrescine import ATP-binding protein PotA
bin003	SOY3_bin003_01847	813	9	33	6	1.323	4.117	0.784	Putative binding protein precursor
bin003	SOY3_bin003_01848	1377	21	37	6	1.823	2.725	0.463	Glutamyl-tRNA(Gln) amidotransferase subunit A
bin003	SOY3_bin003_01849	1512	28	51	14	2.214	3.421	0.984	Phosphoribosylformylglycinamidine synthase 1
bin003	SOY3_bin003_01850	1914	11	9	3	0.687	0.477	0.166	NADP-reducing hydrogenase subunit HndC
bin003	SOY3_bin003_01851	675	30	67	12	5.313	10.068	1.888	Uridylate kinase
bin003	SOY3_bin003_01852	201	2	15	2	1.190	7.569	1.057	DNA gyrase inhibitor YacG
bin003	SOY3_bin003_01853	201	7	11	3	4.163	5.551	1.585	hypothetical protein
bin003	SOY3_bin003_01854	537	14	17	5	3.117	3.211	0.989	hypothetical protein
bin003	SOY3_bin003_01855	705	102	161	45	17.296	23.163	6.780	Gas vesicle protein G
bin003	SOY3_bin003_01856	810	10	11	4	1.476	1.377	0.525	Prephenate dehydratase
bin003	SOY3_bin003_01857	429	8	7	2	2.229	1.655	0.495	Ornithine aminotransferase
bin003	SOY3_bin003_01858	534	71	69	25	15.895	13.106	4.973	50S ribosomal protein L6
bin003	SOY3_bin003_01859	330	42	55	15	15.215	16.905	4.828	50S ribosomal protein L32e
bin003	SOY3_bin003_01860	447	37	53	16	9.896	12.026	3.802	50S ribosomal protein L19e
bin003	SOY3_bin003_01861	582	65	109	33	13.352	18.996	6.023	50S ribosomal protein L18
bin003	SOY3_bin003_01862	684	70	89	26	12.235	13.197	4.038	30S ribosomal protein S5
bin003	SOY3_bin003_01863	1146	39	51	12	4.068	4.514	1.112	Hydrogenase-4 component G
bin003	SOY3_bin003_01864	999	59	55	9	7.060	5.584	0.957	NADH:ubiquinone oxidoreductase subunit H
bin003	SOY3_bin003_01865	273	10	4	5	4.379	1.486	1.946	Energy-converting hydrogenase B subunit P (EhbP)
bin003	SOY3_bin003_01866	453	3	11	1	0.792	2.463	0.234	NAD(P)H:quinone oxidoreductase
bin003	SOY3_bin003_01867	717	0	2	0	0.000	0.283	0.000	Putative NAD(P)H-dependent FMN-containing oxidoreductase YwqN
bin003	SOY3_bin003_01868	879	2	4	0	0.272	0.462	0.000	Putative S-adenosyl-L-methionine-dependent methyltransferase
bin003	SOY3_bin003_01869	648	4	5	1	0.738	0.783	0.164	HTH-type transcriptional regulator TtgR
bin003	SOY3_bin003_01870	609	2	5	1	0.393	0.833	0.174	5-formyltetrahydrofolate cyclo-ligase family protein
bin003	SOY3_bin003_01871	360	4	2	1	1.328	0.563	0.295	hypothetical protein
bin003	SOY3_bin003_01872	753	8	7	1	1.270	0.943	0.141	Bacterial regulatory protein, arsR family
bin003	SOY3_bin003_01873	240	0	3	0	0.000	1.268	0.000	MTH865-like family protein
bin003	SOY3_bin003_01874	450	70	162	28	18.596	36.514	6.610	Nitrate reductase
bin003	SOY3_bin003_01875	1713	255	466	97	17.796	27.592	6.015	Formyltransferase/hydrolase complex Fhc subunit A
bin003	SOY3_bin003_01876	267	24	55	14	10.746	20.893	5.570	acidic ribosomal protein P0
bin003	SOY3_bin003_01877	318	38	84	18	14.286	26.792	6.013	50S ribosomal protein L12P
bin003	SOY3_bin003_01878	189	28	41	11	17.711	22.003	6.182	hypothetical protein
bin003	SOY3_bin003_01879	1314	43	68	22	3.912	5.249	1.779	L-asparaginase 1
bin003	SOY3_bin003_01880	342	5	6	1	1.748	1.779	0.311	Exodeoxyribonuclease
bin003	SOY3_bin003_01881	1863	4125	12004	2194	264.702	653.534	125.099	Large cysteine-rich periplasmic protein OmcB precursor
bin003	SOY3_bin003_01882	738	5	9	2	0.810	1.237	0.288	Prenyltransferase and squalene oxidase repeat protein
bin003	SOY3_bin003_01883	657	1	4	0	0.182	0.618	0.000	Lipoprotein-releasing system ATP-binding protein LolD
bin003	SOY3_bin003_01884	1053	3	4	0	0.341	0.385	0.000	ABC transporter permease YtrF precursor
bin003	SOY3_bin003_01885	216	2	3	1	1.107	1.409	0.492	hypothetical protein
bin003	SOY3_bin003_01886	1296	56	42	3	5.166	3.287	0.246	TraB family protein
bin003	SOY3_bin003_01887	669	25	18	6	4.467	2.729	0.953	putative 2-phosphosulfolactate phosphatase
bin003	SOY3_bin003_01888	210	58	124	19	33.018	59.890	9.611	23S rRNA (uracil-C(5))-methyltransferase RlmCD
bin003	SOY3_bin003_01889	1275	7	13	4	0.656	1.034	0.333	Integrase core domain protein
bin003	SOY3_bin003_01890	804	4	12	0	0.595	1.514	0.000	cell division control protein 6
bin003	SOY3_bin003_01891	255	0	4	0	0.000	1.591	0.000	hypothetical protein
bin003	SOY3_bin003_01892	267	57	61	32	25.522	23.173	12.731	50S ribosomal protein L34e

bin003	SOY3_bin003_01893	519	41	82	20	9.444	16.025	4.093	Adenylate kinase
bin003	SOY3_bin003_01894	231	20	30	1	10.351	13.172	0.460	50S ribosomal protein L14e
bin003	SOY3_bin003_01895	963	222	225	61	27.560	23.698	6.729	tRNA pseudouridine synthase B
bin003	SOY3_bin003_01896	1245	11	5	5	1.056	0.407	0.427	colanic acid biosynthesis protein
bin003	SOY3_bin003_01897	606	4	24	7	0.789	4.017	1.227	hypothetical protein
bin003	SOY3_bin003_01898	657	17	30	15	3.093	4.631	2.425	Nitroreductase family protein
bin003	SOY3_bin003_01899	681	21	60	22	3.687	8.936	3.432	Riboflavin biosynthesis protein RibBA
bin003	SOY3_bin003_01900	396	9	14	2	2.717	3.586	0.536	hypothetical protein
bin003	SOY3_bin003_01901	678	19	24	3	3.350	3.590	0.470	Endonuclease V
bin003	SOY3_bin003_01902	903	63	133	34	8.341	14.939	4.000	2-phospho-L-lactate transferase
bin003	SOY3_bin003_01903	765	11	42	11	1.719	5.569	1.527	Coenzyme F420:L-glutamate ligase
bin003	SOY3_bin003_01904	1860	82	125	30	5.270	6.816	1.713	MCM2/3/5 family protein
bin003	SOY3_bin003_01905	2127	9	32	7	0.506	1.526	0.350	chromosome segregation protein
bin003	SOY3_bin003_01906	1260	88	175	63	8.349	14.087	5.311	Formyltransferase/hydrolase complex Fhc subunit A
bin003	SOY3_bin003_01907	1515	17	35	5	1.341	2.343	0.351	ATP-dependent DNA helicase RecQ
bin003	SOY3_bin003_01908	348	2	1	1	0.687	0.291	0.305	LemA family protein
bin003	SOY3_bin003_01909	522	36	58	23	8.245	11.270	4.680	Soluble hydrogenase 42 kDa subunit
bin003	SOY3_bin003_01910	690	14	14	8	2.426	2.058	1.232	3-oxoadipate enol-lactonase 2
bin003	SOY3_bin003_01911	678	26	56	11	4.584	8.377	1.723	Oxygen-independent coproporphyrinogen-III oxidase 1
bin003	SOY3_bin003_01912	546	3	0	0	0.657	0.000	0.000	Cobalt transport protein CbiM
bin003	SOY3_bin003_01913	306	5	4	2	1.953	1.326	0.694	Cobalt transport protein CbiN
bin003	SOY3_bin003_01914	801	8	7	0	1.194	0.886	0.000	Cobalt transport protein CbiQ
bin003	SOY3_bin003_01915	864	17	29	8	2.352	3.404	0.984	Tetrathionate reductase subunit B precursor
bin003	SOY3_bin003_01916	501	15	20	1	3.579	4.049	0.212	Periplasmic [Fe] hydrogenase large subunit
bin003	SOY3_bin003_01917	447	34	31	2	9.093	7.034	0.475	Formate hydrogenlyase subunit 7
bin003	SOY3_bin003_01918	630	5	4	2	0.949	0.644	0.337	undecaprenyldiphospho-muramoylpentapeptide beta-N-acetylglucosaminyltransferase
bin003	SOY3_bin003_01919	774	8	13	2	1.236	1.704	0.274	Phosphosulfolactate synthase
bin003	SOY3_bin003_01920	900	6	18	4	0.797	2.029	0.472	Peptidase family U32
bin003	SOY3_bin003_01921	447	1	1	0	0.267	0.227	0.000	hypothetical protein
bin003	SOY3_bin003_01922	174	0	3	0	0.000	1.749	0.000	putative ABC transporter ATP-binding protein
bin003	SOY3_bin003_01923	1305	34	43	12	3.115	3.342	0.977	Alanine--tRNA ligase
bin003	SOY3_bin003_01924	351	6	17	3	2.044	4.912	0.908	Tetratricopeptide repeat protein
bin003	SOY3_bin003_01925	1023	9	16	6	1.052	1.586	0.623	Acetyl-coenzyme A synthetase
bin003	SOY3_bin003_01926	315	5	14	4	1.898	4.508	1.349	DNA-directed RNA polymerase subunit P
bin003	SOY3_bin003_01927	399	14	30	10	4.195	7.626	2.662	hypothetical protein
bin003	SOY3_bin003_01928	447	1	3	0	0.267	0.681	0.000	Polyketide cyclase / dehydrase and lipid transport
bin003	SOY3_bin003_01929	1017	5	18	0	0.588	1.795	0.000	Oxygen-dependent choline dehydrogenase
bin003	SOY3_bin003_01930	903	8	22	6	1.059	2.471	0.706	putative cation efflux system protein/MT2084
bin003	SOY3_bin003_01931	459	0	1	0	0.000	0.221	0.000	hypothetical protein
bin003	SOY3_bin003_01932	264	0	0	0	0.000	0.000	0.000	hypothetical protein
bin003	SOY3_bin003_01933	1380	42	49	18	3.638	3.601	1.386	Multidrug resistance protein stp
bin003	SOY3_bin003_01934	330	0	2	0	0.000	0.615	0.000	hypothetical protein
bin003	SOY3_bin003_01935	501	52	71	18	12.408	14.374	3.816	hypothetical protein
bin003	SOY3_bin003_01936	468	25	32	5	6.386	6.935	1.135	heat shock protein GrpE
bin003	SOY3_bin003_01937	414	6	18	2	1.733	4.410	0.513	hypothetical protein
bin003	SOY3_bin003_01938	939	18	29	7	2.292	3.132	0.792	Phytochrome-like protein cph1
bin003	SOY3_bin003_01939	87	1	6	2	1.374	6.995	2.442	tRNA-Ser(gga)
bin003	SOY3_bin003_01940	453	75	72	30	19.793	16.121	7.035	30S ribosomal protein S13
bin003	SOY3_bin003_01941	528	89	112	19	20.151	21.515	3.823	30S ribosomal protein S4
bin003	SOY3_bin003_01942	393	63	63	18	19.164	16.259	4.865	30S ribosomal protein S11
bin003	SOY3_bin003_01943	390	14	27	8	4.291	7.022	2.179	GTP cyclohydrolase III
bin003	SOY3_bin003_01944	756	11	18	7	1.739	2.415	0.984	tRNA-dihydrouridine synthase C
bin003	SOY3_bin003_01945	861	13	15	2	1.805	1.767	0.247	protease TldD
bin003	SOY3_bin003_01946	561	1	7	1	0.213	1.266	0.189	hypothetical protein
bin003	SOY3_bin003_01947	396	0	2	0	0.000	0.512	0.000	phosphodiesterase
bin003	SOY3_bin003_01948	399	7	11	1	2.097	2.796	0.266	hypothetical protein
bin003	SOY3_bin003_01949	300	0	1	1	0.000	0.338	0.354	hypothetical protein
bin003	SOY3_bin003_01950	1152	3	9	4	0.311	0.792	0.369	Pyridoxal 4-dehydrogenase
bin003	SOY3_bin003_01951	1458	5	11	6	0.410	0.765	0.437	Transposase DDE domain protein
bin003	SOY3_bin003_01952	1437	93	252	52	7.737	17.787	3.844	Filamentous hemagglutinin
bin003	SOY3_bin003_01953	612	3	3	0	0.586	0.497	0.000	Epoxyqueuosine reductase
bin003	SOY3_bin003_01954	636	33	54	11	6.203	8.612	1.837	tRNA modification GTPase MnmE
bin003	SOY3_bin003_01955	699	28	48	17	4.789	6.965	2.583	Metallo-beta-lactamase L1 precursor
bin003	SOY3_bin003_01956	1047	11	18	3	1.256	1.744	0.304	Acetolactate synthase large subunit
bin003	SOY3_bin003_01957	1281	15	33	14	1.400	2.613	1.161	Transposase IS116/IS110/IS902 family protein
bin003	SOY3_bin003_01958	1248	108	204	417	10.346	16.579	35.494	Transposase IS116/IS110/IS902 family protein
bin003	SOY3_bin003_01959	1101	7	11	6	0.760	1.013	0.579	OB-fold nucleic acid binding domain protein

bin003	SOY3_bin003_01960	540	9	19	5	1.992	3.569	0.984	PsbP
bin003	SOY3_bin003_01961	882	50	66	12	6.777	7.590	1.445	Glycerol-1-phosphate dehydrogenase [NAD(P)+]
bin003	SOY3_bin003_01962	252	0	4	1	0.000	1.610	0.422	hypothetical protein
bin003	SOY3_bin003_01963	660	15	27	6	2.717	4.149	0.966	4-hydroxy-4-methyl-2-oxoglutarate aldolase
bin003	SOY3_bin003_01964	171	5	9	1	3.496	5.338	0.621	hypothetical protein
bin003	SOY3_bin003_01965	765	19	33	8	2.969	4.375	1.111	hypothetical protein
bin003	SOY3_bin003_01966	240	81	225	43	40.348	95.088	19.032	Pseudomurein-binding repeat protein
bin003	SOY3_bin003_01967	549	42	63	33	9.146	11.639	6.385	Transposase IS66 family protein
bin003	SOY3_bin003_01968	165	0	0	0	0.000	0.000	0.000	hypothetical protein
bin003	SOY3_bin003_01969	978	21	25	5	2.567	2.593	0.543	Aspartyl/glutamyl-tRNA(Asn/Gln) amidotransferase subunit B
bin003	SOY3_bin003_01970	1122	29	48	10	3.090	4.339	0.947	2-isopropylmalate synthase
bin003	SOY3_bin003_01971	963	41	87	36	5.090	9.163	3.971	hypothetical protein
bin003	SOY3_bin003_01972	603	0	0	1	0.000	0.000	0.176	hypothetical protein
bin003	SOY3_bin003_01973	552	56	97	19	12.128	17.823	3.656	adenylate kinase
bin003	SOY3_bin003_01974	597	66	103	22	13.216	17.499	3.915	60Kd inner membrane protein
bin003	SOY3_bin003_01975	255	1	1	0	0.469	0.398	0.000	hypothetical protein
bin003	SOY3_bin003_01976	1134	6	6	1	0.633	0.537	0.094	2-methylcitrate dehydratase
bin003	SOY3_bin003_01977	477	10	17	8	2.506	3.615	1.782	Methionine--tRNA ligase
bin003	SOY3_bin003_01978	591	12	16	3	2.427	2.746	0.539	Phosphoribosylformylglycinamidine synthase 2
bin003	SOY3_bin003_01979	582	10	19	5	2.054	3.311	0.913	Cytidylyltransferase family protein
bin003	SOY3_bin003_01980	702	19	21	6	3.236	3.034	0.908	Carbamoyl-phosphate synthase small chain
bin003	SOY3_bin003_01981	726	8	10	0	1.317	1.397	0.000	tRNA pseudouridine synthase D
bin003	SOY3_bin003_01982	807	19	25	5	2.815	3.142	0.658	Oxygen-independent coproporphyrinogen-III oxidase 1
bin003	SOY3_bin003_01983	249	2	7	4	0.960	2.851	1.706	hypothetical protein
bin003	SOY3_bin003_01984	948	9	21	3	1.135	2.247	0.336	Rhamnosyltransferase WbbL
bin003	SOY3_bin003_01985	966	45	184	63	5.569	19.319	6.928	putative transposase
bin003	SOY3_bin003_01986	441	2	5	6	0.542	1.150	1.445	2-(S)-hydroxypropyl-CoM dehydrogenase
bin003	SOY3_bin003_01987	423	54	76	19	15.262	18.223	4.771	50S ribosomal protein L13
bin003	SOY3_bin003_01988	357	48	54	8	16.074	15.342	2.380	50S ribosomal protein L18e
bin003	SOY3_bin003_01989	186	18	35	3	11.569	19.086	1.713	DNA-directed RNA polymerase subunit D
bin003	SOY3_bin003_01990	429	2	10	1	0.557	2.364	0.248	hypothetical protein
bin003	SOY3_bin003_01991	390	2	2	2	0.613	0.520	0.545	L(+)-tartrate dehydratase subunit beta
bin003	SOY3_bin003_01992	645	14	28	4	2.595	4.403	0.659	Proline--tRNA ligase
bin003	SOY3_bin003_01993	609	14	18	6	2.748	2.998	1.047	hypothetical protein
bin003	SOY3_bin003_01994	1026	73	123	33	8.506	12.159	3.417	30S ribosomal protein S3
bin003	SOY3_bin003_01995	588	5	48	11	1.017	8.280	1.987	hypothetical protein
bin003	SOY3_bin003_01996	372	1	1	0	0.321	0.273	0.000	Nucleotidyltransferase domain protein
bin003	SOY3_bin003_01997	162	0	0	0	0.000	0.000	0.000	hypothetical protein
bin003	SOY3_bin003_01998	1458	5	9	2	0.410	0.626	0.146	Transcriptional regulator
bin003	SOY3_bin003_01999	1185	14	21	7	1.412	1.797	0.627	cell division control protein 6
bin003	SOY3_bin003_02000	192	0	4	0	0.000	2.113	0.000	hypothetical protein
bin003	SOY3_bin003_02001	444	1	3	1	0.269	0.685	0.239	DNA-damage-inducible protein D
bin003	SOY3_bin003_02002	585	1	2	1	0.204	0.347	0.182	hypothetical protein
bin003	SOY3_bin003_02003	123	0	0	1	0.000	0.000	0.864	2-oxoacid ferredoxin oxidoreductase subunit beta
bin003	SOY3_bin003_02004	339	3	4	0	1.058	1.197	0.000	hypothetical protein
bin003	SOY3_bin003_02005	531	0	6	2	0.000	1.146	0.400	PsbP
bin003	SOY3_bin003_02006	321	0	0	0	0.000	0.000	0.000	hypothetical protein
bin003	SOY3_bin003_02007	660	1	1	0	0.181	0.154	0.000	hypothetical protein
bin003	SOY3_bin003_02008	1068	14	20	1	1.567	1.899	0.099	Radical SAM superfamily protein
bin003	SOY3_bin003_02009	1125	12	30	8	1.275	2.705	0.755	hypothetical protein
bin003	SOY3_bin003_02010	213	13	4	1	7.296	1.905	0.499	HTH-type transcriptional activator HxlR
bin003	SOY3_bin003_02011	759	6	9	3	0.945	1.203	0.420	N5-carboxyaminoimidazole ribonucleotide mutase
bin003	SOY3_bin003_02012	1545	4	7	1	0.310	0.460	0.069	Stage V sporulation protein K
bin003	SOY3_bin003_02013	1233	3	7	3	0.291	0.576	0.258	Radical SAM superfamily protein
bin003	SOY3_bin003_02014	1146	53	139	26	5.529	12.302	2.410	Serine protease Do-like HtrB
bin003	SOY3_bin003_02015	342	1	5	0	0.350	1.483	0.000	hypothetical protein
bin003	SOY3_bin003_02016	414	3	4	1	0.866	0.980	0.257	LexA repressor
bin003	SOY3_bin003_02017	177	0	0	0	0.000	0.000	0.000	hypothetical protein
bin003	SOY3_bin003_02018	282	29	125	17	12.294	44.959	6.404	anaerobic ribonucleoside triphosphate reductase
bin003	SOY3_bin003_02019	420	13	15	3	3.700	3.622	0.759	hypothetical protein
bin003	SOY3_bin003_02020	378	2	9	2	0.633	2.415	0.562	chromosome segregation protein
bin003	SOY3_bin003_02021	969	0	2	0	0.000	0.209	0.000	hypothetical protein
bin003	SOY3_bin003_02022	504	3	10	2	0.712	2.012	0.422	SpoIVB peptidase S55
bin003	SOY3_bin003_02023	720	17	24	5	2.823	3.381	0.738	Putative phosphoribosyl transferase/MT0597
bin003	SOY3_bin003_02024	456	8	12	5	2.097	2.669	1.165	Deoxycytidine triphosphate deaminase
bin003	SOY3_bin003_02025	1314	7	14	5	0.637	1.081	0.404	Magnesium-chelatase 60 kDa subunit
bin003	SOY3_bin003_02026	954	34	79	20	4.261	8.399	2.227	Inosine-5'-monophosphate dehydrogenase

bin003	SOY3_bin003_02027	690	2	8	1	0.347	1.176	0.154	Molybdenum-pterin-binding protein MopA
bin003	SOY3_bin003_02028	330	8	18	2	2.898	5.532	0.644	Carboxymuconolactone decarboxylase family protein
bin003	SOY3_bin003_02029	276	1	1	1	0.433	0.367	0.385	hypothetical protein
bin003	SOY3_bin003_02030	198	3	4	3	1.811	2.049	1.609	hypothetical protein
bin003	SOY3_bin003_02031	591	2	11	2	0.405	1.888	0.359	Flavodoxin-like fold protein
bin003	SOY3_bin003_02032	507	5	18	3	1.179	3.601	0.629	hypothetical protein
bin003	SOY3_bin003_02033	882	1	6	2	0.136	0.690	0.241	putative oxidoreductase
bin003	SOY3_bin003_02034	1572	34	64	10	2.586	4.129	0.676	Antiseptic resistance protein
bin003	SOY3_bin003_02035	1242	14	12	4	1.348	0.980	0.342	hypothetical protein
bin003	SOY3_bin003_02036	423	2	4	0	0.565	0.959	0.000	hypothetical protein
bin003	SOY3_bin003_02037	354	0	3	1	0.000	0.860	0.300	Nitroreductase family protein
bin003	SOY3_bin003_02038	456	0	3	3	0.000	0.667	0.699	hypothetical protein
bin003	SOY3_bin003_02039	441	2	3	0	0.542	0.690	0.000	Protoporphyrinogen IX dehydrogenase [menaquinone]
bin003	SOY3_bin003_02040	282	0	1	1	0.000	0.360	0.377	Transcriptional repressor SdpR
bin003	SOY3_bin003_02041	123	0	0	0	0.000	0.000	0.000	hypothetical protein
bin003	SOY3_bin003_02042	699	4	14	1	0.684	2.031	0.152	hypothetical protein
bin003	SOY3_bin003_02043	186	0	0	0	0.000	0.000	0.000	hypothetical protein
bin003	SOY3_bin003_02044	195	0	1	0	0.000	0.520	0.000	hypothetical protein
bin003	SOY3_bin003_02045	1392	12	20	4	1.031	1.457	0.305	D-inositol-3-phosphate glycosyltransferase
bin003	SOY3_bin003_02046	1014	4	15	4	0.472	1.500	0.419	Galactofuranosyl transferase GlfT1
bin003	SOY3_bin003_02047	909	13	10	3	1.710	1.116	0.351	Benzylsuccinate synthase activating enzyme
bin003	SOY3_bin003_02048	1980	18	34	8	1.087	1.742	0.429	4-hydroxyphenylacetate decarboxylase large subunit
bin003	SOY3_bin003_02049	1269	6	12	3	0.565	0.959	0.251	Putative O-antigen transporter
bin003	SOY3_bin003_02050	375	3	9	0	0.956	2.434	0.000	hypothetical protein
bin003	SOY3_bin003_02051	735	6	11	7	0.976	1.518	1.012	Undecaprenyl-phosphate mannosyltransferase
bin003	SOY3_bin003_02052	459	27	35	6	7.032	7.734	1.389	Transposase zinc-ribbon domain protein
bin003	SOY3_bin003_02053	399	14	31	3	4.195	7.880	0.799	ISXO2-like transposase domain protein
bin003	SOY3_bin003_02054	252	12	22	3	5.693	8.855	1.265	Glucosyl-3-phosphoglycerate synthase
bin003	SOY3_bin003_02055	162	5	11	4	3.690	6.887	2.623	hypothetical protein
bin003	SOY3_bin003_02056	198	1	2	1	0.604	1.025	0.536	hypothetical protein
bin003	SOY3_bin003_02057	837	2	9	3	0.286	1.091	0.381	Sporulenol synthase
bin003	SOY3_bin003_02058	1218	3	8	5	0.294	0.666	0.436	MULE transposase domain protein
bin003	SOY3_bin003_02059	282	1	0	0	0.424	0.000	0.000	Acyl-coenzyme A thioesterase PaaI
bin003	SOY3_bin003_02060	492	2	6	1	0.486	1.237	0.216	Peptide methionine sulfoxide reductase MsrA
bin003	SOY3_bin003_02061	1428	10	10	3	0.837	0.710	0.223	putative amino acid permease YhdG
bin003	SOY3_bin003_02062	939	16	23	4	2.037	2.484	0.453	DNA polymerase I
bin003	SOY3_bin003_02063	552	28	69	7	6.064	12.678	1.347	hypothetical protein
bin003	SOY3_bin003_02064	77	1	0	0	1.553	0.000	0.000	tRNA-Glu(ctc)
bin003	SOY3_bin003_02065	330	9	11	2	3.260	3.381	0.644	hypothetical protein
bin003	SOY3_bin003_02066	399	8	12	5	2.397	3.050	1.331	hypothetical protein
bin003	SOY3_bin003_02067	744	1	11	3	0.161	1.500	0.428	hypothetical protein
bin003	SOY3_bin003_02068	300	1	2	1	0.398	0.676	0.354	hypothetical protein
bin003	SOY3_bin003_02069	393	0	5	0	0.000	1.290	0.000	tRNA(fMet)-specific endonuclease VapC
bin003	SOY3_bin003_02070	267	1	1	0	0.448	0.380	0.000	hypothetical protein
bin003	SOY3_bin003_02071	429	0	10	3	0.000	2.364	0.743	phenylalanyl-tRNA synthetase subunit alpha
bin003	SOY3_bin003_02072	198	0	3	2	0.000	1.537	1.073	hypothetical protein
bin003	SOY3_bin003_02073	249	2	4	0	0.960	1.629	0.000	hypothetical protein
bin003	SOY3_bin003_02074	126	0	4	0	0.000	3.220	0.000	hypothetical protein
bin003	SOY3_bin003_02075	303	0	0	1	0.000	0.000	0.351	hypothetical protein
bin003	SOY3_bin003_02076	234	1	1	1	0.511	0.433	0.454	hypothetical protein
bin003	SOY3_bin003_02077	258	0	2	2	0.000	0.786	0.823	hypothetical protein
bin003	SOY3_bin003_02078	390	1	4	0	0.307	1.040	0.000	Ribonuclease VapC20
bin003	SOY3_bin003_02079	294	0	4	1	0.000	1.380	0.361	hypothetical protein
bin003	SOY3_bin003_02080	429	5	14	3	1.393	3.310	0.743	Helix-turn-helix domain protein
bin003	SOY3_bin003_02081	348	1	0	0	0.344	0.000	0.000	hypothetical protein
bin003	SOY3_bin003_02082	741	3	7	1	0.484	0.958	0.143	hypothetical protein
bin003	SOY3_bin003_02083	675	0	2	1	0.000	0.301	0.157	hypothetical protein
bin003	SOY3_bin003_02084	1029	7	9	1	0.813	0.887	0.103	Adenosine monophosphate-protein transferase SoFic
bin003	SOY3_bin003_02085	177	0	0	0	0.000	0.000	0.000	Ferredoxin
bin003	SOY3_bin003_02086	651	15	21	11	2.755	3.272	1.795	hypothetical protein
bin003	SOY3_bin003_02087	894	37	47	15	4.948	5.332	1.782	hypothetical protein
bin003	SOY3_bin003_02088	360	10	4	4	3.321	1.127	1.180	hypothetical protein
bin003	SOY3_bin003_02089	705	2	7	2	0.339	1.007	0.301	hypothetical protein
bin003	SOY3_bin003_02090	735	4	5	2	0.651	0.690	0.289	hypothetical protein
bin003	SOY3_bin003_02091	954	5	13	2	0.627	1.382	0.223	Secreted effector protein pipB2
bin003	SOY3_bin003_02092	393	13	15	5	3.955	3.871	1.351	hypothetical protein
bin003	SOY3_bin003_02093	1158	4	10	3	0.413	0.876	0.275	Lon protease 2

bin003	SOY3_bin003_02094	1107	5	11	4	0.540	1.008	0.384	MULE transposase domain protein
bin003	SOY3_bin003_02095	153	0	1	0	0.000	0.663	0.000	Ribbon-helix-helix domain protein
bin003	SOY3_bin003_02096	576	3	12	3	0.623	2.113	0.553	FmdE, Molybdenum formylmethanofuran dehydrogenase operon
bin003	SOY3_bin003_02097	882	1	3	0	0.136	0.345	0.000	Magnesium-protoporphyrin O-methyltransferase
bin003	SOY3_bin003_02098	213	0	0	0	0.000	0.000	0.000	hypothetical protein
bin003	SOY3_bin003_02099	459	0	0	0	0.000	0.000	0.000	hypothetical protein
bin003	SOY3_bin003_02100	195	1	0	0	0.613	0.000	0.000	hypothetical protein
bin003	SOY3_bin003_02101	609	1	0	1	0.196	0.000	0.174	hypothetical protein
bin003	SOY3_bin003_02102	834	5	9	3	0.717	1.095	0.382	Non-haem bromoperoxidase BPO-A2
bin003	SOY3_bin003_02103	246	5	13	4	2.430	5.360	1.727	hypothetical protein
bin003	SOY3_bin003_02104	291	2	3	1	0.822	1.046	0.365	hypothetical protein
bin003	SOY3_bin003_02105	1227	1	1	0	0.097	0.083	0.000	General stress protein 69
bin003	SOY3_bin003_02106	561	0	3	0	0.000	0.542	0.000	HTH-type transcriptional repressor Bm3R1
bin003	SOY3_bin003_02107	771	5	3	0	0.775	0.395	0.000	endonuclease IV
bin003	SOY3_bin003_02108	486	1	5	0	0.246	1.043	0.000	Transcriptional regulator YqjI
bin003	SOY3_bin003_02109	1077	9	13	4	0.999	1.224	0.395	putative ABC transporter ATP-binding protein YbhF
bin003	SOY3_bin003_02110	1140	11	19	5	1.154	1.690	0.466	Inner membrane transport permease YbhR
bin003	SOY3_bin003_02111	861	14	13	0	1.944	1.531	0.000	Anaerobic sulfite reductase subunit C
bin003	SOY3_bin003_02112	192	7	4	0	4.359	2.113	0.000	Ferredoxin-1
bin003	SOY3_bin003_02113	105	5	3	0	5.693	2.898	0.000	hypothetical protein
bin003	SOY3_bin003_02114	417	9	11	2	2.580	2.676	0.509	Dinitrogenase iron-molybdenum cofactor
bin003	SOY3_bin003_02115	852	27	19	7	3.789	2.262	0.873	Septum site-determining protein MinD
bin003	SOY3_bin003_02116	375	19	17	4	6.057	4.598	1.133	Dinitrogenase iron-molybdenum cofactor
bin003	SOY3_bin003_02117	606	19	22	4	3.748	3.682	0.701	hypothetical protein
bin003	SOY3_bin003_02118	402	3	6	1	0.892	1.514	0.264	putative HTH-type transcriptional regulator YybR
bin003	SOY3_bin003_02119	168	7	13	3	4.981	7.849	1.897	Prefoldin subunit alpha
bin003	SOY3_bin003_02120	924	9	6	3	1.164	0.659	0.345	Daunorubicin/doxorubicin resistance ATP-binding protein DrrA
bin003	SOY3_bin003_02121	756	3	0	1	0.474	0.000	0.141	Inner membrane transport permease YadH
bin003	SOY3_bin003_02122	285	0	5	0	0.000	1.779	0.000	hypothetical protein
bin003	SOY3_bin003_02123	231	0	1	0	0.000	0.439	0.000	hypothetical protein
bin003	SOY3_bin003_02124	309	1	5	2	0.387	1.641	0.688	hydrogenase nickel incorporation protein HypA
bin003	SOY3_bin003_02125	621	17	49	8	3.273	8.003	1.368	FmdE, Molybdenum formylmethanofuran dehydrogenase operon
bin003	SOY3_bin003_02126	573	6	13	3	1.252	2.301	0.556	hypothetical protein
bin003	SOY3_bin003_02127	189	0	3	0	0.000	1.610	0.000	Cation efflux family protein
bin003	SOY3_bin003_02128	612	2	3	0	0.391	0.497	0.000	Demethylmenaquinone methyltransferase
bin003	SOY3_bin003_02129	768	2	5	0	0.311	0.660	0.000	putative adenylyl-sulfate kinase
bin003	SOY3_bin003_02130	483	2	4	1	0.495	0.840	0.220	Transcriptional regulator YqjI
bin003	SOY3_bin003_02131	852	1	2	1	0.140	0.238	0.125	Nitrogen regulatory protein P-II
bin003	SOY3_bin003_02132	780	8	13	3	1.226	1.690	0.409	Formamidopyrimidine-DNA glycosylase
bin003	SOY3_bin003_02133	1266	12	28	10	1.133	2.243	0.839	3-phosphoshikimate 1-carboxyvinyltransferase
bin003	SOY3_bin003_02134	594	1	2	0	0.201	0.342	0.000	helix-turn-helix, Psq domain
bin003	SOY3_bin003_02135	462	0	2	1	0.000	0.439	0.230	HTH-type transcriptional activator HxlR
bin003	SOY3_bin003_02136	687	0	1	0	0.000	0.148	0.000	short chain dehydrogenase
bin003	SOY3_bin003_02137	870	0	1	0	0.000	0.117	0.000	L-aspartate dehydrogenase
bin003	SOY3_bin003_02138	765	1	1	0	0.156	0.133	0.000	3-oxoacyl-[acyl-carrier-protein] reductase FabG
bin003	SOY3_bin003_02139	438	2	3	1	0.546	0.695	0.243	Methyl-viologen-reducing hydrogenase, delta subunit
bin003	SOY3_bin003_02140	147	0	0	0	0.000	0.000	0.000	H(2)-dependent methylenetetrahydromethanopterin dehydrogenase-related protein
bin003	SOY3_bin003_02141	222	10	14	0	5.385	6.396	0.000	hypothetical protein
bin003	SOY3_bin003_02142	393	1	3	2	0.304	0.774	0.541	hypothetical protein
bin003	SOY3_bin003_02143	189	0	0	0	0.000	0.000	0.000	tRNA(fMet)-specific endonuclease VapC
bin003	SOY3_bin003_02144	189	1	2	0	0.633	1.073	0.000	hypothetical protein
bin003	SOY3_bin003_02145	144	0	0	0	0.000	0.000	0.000	hypothetical protein
bin003	SOY3_bin003_02146	186	1	3	2	0.643	1.636	1.142	hypothetical protein
bin003	SOY3_bin003_02147	381	4	1	0	1.255	0.266	0.000	HEPN domain protein
bin003	SOY3_bin003_02148	345	0	8	1	0.000	2.352	0.308	Nucleotidyltransferase domain protein
bin003	SOY3_bin003_02149	423	0	7	0	0.000	1.678	0.000	hypothetical protein
bin003	SOY3_bin003_02150	402	1	5	2	0.297	1.262	0.528	Nucleotidyltransferase domain protein
bin003	SOY3_bin003_02151	702	2	2	0	0.341	0.289	0.000	hypothetical protein
bin003	SOY3_bin003_02152	681	1	1	1	0.176	0.149	0.156	tRNA s(4)U8 sulfurtransferase
bin003	SOY3_bin003_02153	381	4	5	1	1.255	1.331	0.279	hypothetical protein
bin003	SOY3_bin003_02154	549	4	12	7	0.871	2.217	1.354	hypothetical protein
bin003	SOY3_bin003_02155	762	6	15	3	0.941	1.997	0.418	hypothetical protein
bin003	SOY3_bin003_02156	372	0	0	0	0.000	0.000	0.000	hypothetical protein
bin003	SOY3_bin003_02157	1143	1	10	3	0.105	0.887	0.279	UDP-galactopyranose mutase precursor
bin003	SOY3_bin003_02158	1044	4	4	2	0.458	0.389	0.203	Galactofuranosyl transferase GlfT1
bin003	SOY3_bin003_02159	1218	18	25	9	1.767	2.082	0.785	N-acetylgalactosamine-N,N'-diacetylbacillosaminyl-diphospho-undecaprenol 4-alpha-N-acetylgalactosaminyltransferase
bin003	SOY3_bin003_02160	1227	6	5	3	0.585	0.413	0.260	N-acetylgalactosamine-N,N'-diacetylbacillosaminyl-diphospho-undecaprenol 4-alpha-N-acetylgalactosaminyltransferase

bin003	SOY3_bin003_02161	327	0	1	1	0.000	0.310	0.325	hypothetical protein
bin003	SOY3_bin003_02162	1122	7	20	5	0.746	1.808	0.473	ATP-dependent zinc metalloprotease FtsH
bin003	SOY3_bin003_02163	843	3	3	0	0.425	0.361	0.000	putative endonuclease 4
bin003	SOY3_bin003_02164	678	5	4	2	0.882	0.598	0.313	Thioredoxin reductase
bin003	SOY3_bin003_02165	255	0	0	0	0.000	0.000	0.000	hypothetical protein
bin003	SOY3_bin003_02166	345	0	0	1	0.000	0.000	0.308	hypothetical protein
bin003	SOY3_bin003_02167	219	1	0	1	0.546	0.000	0.485	hypothetical protein
bin003	SOY3_bin003_02168	1149	34	45	13	3.538	3.972	1.202	Coenzyme A biosynthesis bifunctional protein CoaBC
bin003	SOY3_bin003_02169	822	8	59	13	1.163	7.280	1.680	Copper resistance protein CopC
bin003	SOY3_bin003_02170	480	12	25	9	2.989	5.283	1.992	hypothetical protein
bin003	SOY3_bin003_02171	273	11	35	4	4.817	13.003	1.556	hypothetical protein
bin003	SOY3_bin003_02172	159	0	0	0	0.000	0.000	0.000	hypothetical protein
bin003	SOY3_bin003_02173	210	0	2	1	0.000	0.966	0.506	50S ribosomal protein L9
bin003	SOY3_bin003_02174	219	4	3	0	2.184	1.389	0.000	hypothetical protein
bin003	SOY3_bin003_02175	624	1	4	1	0.192	0.650	0.170	hypothetical protein
bin003	SOY3_bin003_02176	207	4	3	1	2.310	1.470	0.513	hypothetical protein
bin003	SOY3_bin003_02177	543	1	2	0	0.220	0.374	0.000	Adenosine monophosphate-protein transferase SoFic
bin003	SOY3_bin003_02178	450	4	2	2	1.063	0.451	0.472	Lipoprotein-releasing system ATP-binding protein LolD
bin003	SOY3_bin003_02179	384	1	1	2	0.311	0.264	0.553	Lipoprotein-releasing system ATP-binding protein LolD
bin003	SOY3_bin003_02180	1113	2	2	2	0.215	0.182	0.191	Macrolide export ATP-binding/permease protein MacB
bin003	SOY3_bin003_02181	579	0	0	0	0.000	0.000	0.000	Fatty acid metabolism regulator protein
bin003	SOY3_bin003_02182	141	0	2	0	0.000	1.439	0.000	hypothetical protein
bin003	SOY3_bin003_02183	441	9	22	4	2.440	5.060	0.963	hypothetical protein
bin003	SOY3_bin003_02184	675	1	6	4	0.177	0.902	0.629	hypothetical protein
bin003	SOY3_bin003_02185	525	0	4	2	0.000	0.773	0.405	Inorganic pyrophosphatase
bin003	SOY3_bin003_02186	276	0	0	0	0.000	0.000	0.000	hypothetical protein
bin003	SOY3_bin003_02187	270	0	1	0	0.000	0.376	0.000	Inorganic pyrophosphatase
bin003	SOY3_bin003_02188	264	2	2	0	0.906	0.768	0.000	CRISPR-associated endoribonuclease Cas2
bin003	SOY3_bin003_02189	984	8	7	1	0.972	0.722	0.108	CRISPR-associated endonuclease Cas1
bin003	SOY3_bin003_02190	519	3	7	3	0.691	1.368	0.614	PD-(D/E)XK nuclease superfamily protein
bin003	SOY3_bin003_02191	267	4	4	2	1.791	1.520	0.796	mRNA interferase RelE
bin003	SOY3_bin003_02192	183	9	12	2	5.879	6.651	1.161	hypothetical protein
bin003	SOY3_bin003_02193	192	6	8	2	3.736	4.226	1.107	hypothetical protein
bin003	SOY3_bin003_02194	468	4	6	4	1.022	1.300	0.908	Tetratricopeptide repeat protein
bin003	SOY3_bin003_02195	588	11	17	0	2.236	2.932	0.000	Plasmid pRiA4b ORF-3-like protein
bin003	SOY3_bin003_02196	132	0	1	1	0.000	0.768	0.805	hypothetical protein
bin003	SOY3_bin003_02197	3504	3	10	1	0.102	0.289	0.030	Serine/threonine-protein kinase AfsK
bin003	SOY3_bin003_02198	267	0	0	2	0.000	0.000	0.796	hypothetical protein
bin003	SOY3_bin003_02199	573	6	7	0	1.252	1.239	0.000	Sugar-specific transcriptional regulator TrmB
bin003	SOY3_bin003_02200	303	4	1	1	1.578	0.335	0.351	hypothetical protein
bin003	SOY3_bin003_02201	813	113	227	66	16.616	28.320	8.623	hypothetical protein
bin003	SOY3_bin003_02202	1011	55	86	28	6.504	8.628	2.942	50S ribosomal protein L3
bin003	SOY3_bin003_02203	765	5	16	0	0.781	2.121	0.000	hypothetical protein
bin003	SOY3_bin003_02204	549	3	4	1	0.653	0.739	0.193	Dephospho-CoA kinase
bin003	SOY3_bin003_02205	381	2	8	1	0.628	2.130	0.279	hypothetical protein
bin003	SOY3_bin003_02206	681	7	16	5	1.229	2.383	0.780	tRNA s(4)U8 sulfurtransferase
bin003	SOY3_bin003_02207	816	31	46	9	4.542	5.718	1.172	hypothetical protein
bin003	SOY3_bin003_02208	402	7	11	2	2.082	2.775	0.528	Peptidase propeptide and YPEB domain protein
bin003	SOY3_bin003_02209	402	4	10	2	1.190	2.523	0.528	Outer membrane protein assembly factor BamB
bin003	SOY3_bin003_02210	1167	19	32	12	1.946	2.781	1.092	hypothetical protein
bin003	SOY3_bin003_02211	2070	34	67	19	1.964	3.283	0.975	hypothetical protein
bin003	SOY3_bin003_02212	900	21	26	2	2.789	2.930	0.236	hypothetical protein
bin003	SOY3_bin003_02213	411	65	67	25	18.907	16.534	6.461	Class III signal peptide
bin003	SOY3_bin003_02214	1662	4	20	2	0.288	1.221	0.128	Penicillin-binding protein 4*
bin003	SOY3_bin003_02215	579	5	9	3	1.032	1.577	0.550	hypothetical protein
bin003	SOY3_bin003_02216	426	3	4	5	0.842	0.952	1.247	hypothetical protein
bin003	SOY3_bin003_02217	86	3	1	1	4.170	1.179	1.235	tRNA-Ser(tga)
bin003	SOY3_bin003_02218	258	3	6	3	1.390	2.359	1.235	putative selenate reductase subunit YgfK
bin003	SOY3_bin003_02219	1188	11	14	2	1.107	1.195	0.179	Ribosome-binding ATPase YchF
bin003	SOY3_bin003_02220	5727	2	5	5	0.042	0.089	0.093	Virginiamycin B lyase
bin003	SOY3_bin003_02221	975	0	0	0	0.000	0.000	0.000	hypothetical protein
bin003	SOY3_bin003_02222	837	0	1	0	0.000	0.121	0.000	hypothetical protein
bin003	SOY3_bin003_02223	987	0	0	0	0.000	0.000	0.000	hypothetical protein
bin003	SOY3_bin003_02224	297	0	3	1	0.000	1.025	0.358	protease TldD
bin003	SOY3_bin003_02225	561	4	10	0	0.852	1.808	0.000	hypothetical protein
bin003	SOY3_bin003_02226	432	2	1	0	0.553	0.235	0.000	hypothetical protein
bin003	SOY3_bin003_02227	414	2	6	2	0.578	1.470	0.513	Nucleotidyltransferase domain protein

bin003	SOY3_bin003_02228	960	8	31	9	0.996	3.275	0.996	Putative aliphatic sulfonates-binding protein precursor
bin003	SOY3_bin003_02229	798	3	11	2	0.449	1.398	0.266	Bicarbonate transport system permease protein CmpB
bin003	SOY3_bin003_02230	741	3	4	2	0.484	0.548	0.287	Bicarbonate transport ATP-binding protein CmpD
bin003	SOY3_bin003_02231	396	0	0	0	0.000	0.000	0.000	hypothetical protein
bin003	SOY3_bin003_02232	345	19	34	7	6.584	9.996	2.155	hypothetical protein
bin003	SOY3_bin003_02233	198	12	13	5	7.245	6.659	2.682	30S ribosomal protein S27ae
bin003	SOY3_bin003_02234	399	33	74	19	9.888	18.811	5.058	putative spore protein YtfJ
bin003	SOY3_bin003_02235	591	13	24	4	2.630	4.119	0.719	hypothetical protein
bin003	SOY3_bin003_02236	408	6	7	2	1.758	1.740	0.521	hypothetical protein
bin003	SOY3_bin003_02237	207	335	1431	303	193.473	701.171	155.490	Histone-like transcription factor (CBF/NF-Y) and archaeal histone
bin003	SOY3_bin003_02238	399	1	6	2	0.300	1.525	0.532	Peptidase propeptide and YPEB domain protein
bin003	SOY3_bin003_02239	420	4	9	2	1.139	2.173	0.506	hypothetical protein
bin003	SOY3_bin003_02240	1065	12	28	6	1.347	2.667	0.598	hypothetical protein
bin003	SOY3_bin003_02241	2175	38	104	19	2.089	4.850	0.928	hypothetical protein
bin003	SOY3_bin003_02242	888	29	44	9	3.904	5.026	1.077	hypothetical protein
bin003	SOY3_bin003_02243	468	11	17	4	2.810	3.684	0.908	Class III signal peptide
bin003	SOY3_bin003_02244	1278	3	2	3	0.281	0.159	0.249	Enolase
bin003	SOY3_bin003_02245	780	1	0	0	0.153	0.000	0.000	Prenyltransferase and squalene oxidase repeat protein
bin003	SOY3_bin003_02246	1143	1	6	0	0.105	0.532	0.000	Sodium/hydrogen exchanger family protein
bin003	SOY3_bin003_02247	579	10	16	7	2.065	2.803	1.284	hypothetical protein
bin003	SOY3_bin003_02248	1683	1	13	2	0.071	0.783	0.126	Penicillin-binding protein 4*
bin003	SOY3_bin003_02249	906	5	0	2	0.660	0.000	0.234	Modification methylase DpnIIA
bin003	SOY3_bin003_02250	333	2	4	1	0.718	1.218	0.319	hypothetical protein
bin003	SOY3_bin003_02251	444	4	5	3	1.077	1.142	0.718	phenylalanyl-tRNA synthetase subunit alpha
bin003	SOY3_bin003_02252	330	2	0	1	0.725	0.000	0.322	hypothetical protein
bin003	SOY3_bin003_02253	153	1	0	0	0.781	0.000	0.000	Benzoate--CoA ligase
bin003	SOY3_bin003_02254	327	3	2	2	1.097	0.620	0.650	Quaternary ammonium compound-resistance protein QacC
bin003	SOY3_bin003_02255	1686	11	28	9	0.780	1.684	0.567	Beta-lactamase precursor
bin003	SOY3_bin003_02256	360	1	2	2	0.332	0.563	0.590	hypothetical protein
bin003	SOY3_bin003_02257	2160	2	5	2	0.111	0.235	0.098	putative cadmium-transporting ATPase
bin003	SOY3_bin003_02258	240	8	23	3	3.985	9.720	1.328	hypothetical protein
bin003	SOY3_bin003_02259	288	1	1	2	0.415	0.352	0.738	Transcriptional repressor SdpR
bin003	SOY3_bin003_02260	447	2	1	0	0.535	0.227	0.000	Protoporphyrinogen IX dehydrogenase [menaquinone]
bin003	SOY3_bin003_02261	657	0	2	0	0.000	0.309	0.000	Lipoprotein-releasing system ATP-binding protein LolD
bin003	SOY3_bin003_02262	1056	1	4	2	0.113	0.384	0.201	Macrolide export ATP-binding/permease protein MacB
bin003	SOY3_bin003_02263	525	3	4	0	0.683	0.773	0.000	PRC-barrel domain protein
bin003	SOY3_bin003_02264	528	7	10	3	1.585	1.921	0.604	hypothetical protein
bin003	SOY3_bin003_02265	345	3	11	1	1.040	3.234	0.308	hypothetical protein
bin003	SOY3_bin003_02266	249	3	12	2	1.440	4.888	0.853	Bacterial Ig-like domain (group 1)
bin003	SOY3_bin003_02267	2628	17	11	3	0.773	0.425	0.121	hypothetical protein
bin003	SOY3_bin003_02268	888	1	0	0	0.135	0.000	0.000	TPR repeat-containing protein YrrB
bin003	SOY3_bin003_02269	73	1	2	1	1.638	2.779	1.455	tRNA-Arg(gcg)
bin003	SOY3_bin003_02270	1200	4	6	3	0.398	0.507	0.266	Putative oxidoreductase/MT0587
bin003	SOY3_bin003_02271	168	0	0	0	0.000	0.000	0.000	Ferredoxin-1
bin003	SOY3_bin003_02272	636	0	9	1	0.000	1.435	0.167	HTH-type transcriptional repressor ComR
bin003	SOY3_bin003_02273	1170	2	14	3	0.204	1.214	0.272	hypothetical protein
bin003	SOY3_bin003_02274	780	3	7	2	0.460	0.910	0.272	Ferredoxin-2
bin003	SOY3_bin003_02275	603	1	4	3	0.198	0.673	0.528	lipid A 1-phosphatase
bin003	SOY3_bin003_02276	549	30	34	4	6.533	6.281	0.774	tetrahydromethanopterin S-methyltransferase subunit A
bin003	SOY3_bin003_02277	465	4	6	0	1.028	1.309	0.000	hypothetical protein
bin003	SOY3_bin003_02278	207	0	2	2	0.000	0.980	1.026	Antitoxin HicB
bin003	SOY3_bin003_02279	321	2	4	0	0.745	1.264	0.000	hypothetical protein
bin003	SOY3_bin003_02280	618	1	2	0	0.193	0.328	0.000	hypothetical protein
bin003	SOY3_bin003_02281	1152	5	3	1	0.519	0.264	0.092	hypothetical protein
bin003	SOY3_bin003_02282	1140	14	21	6	1.468	1.868	0.559	VWA domain containing CoxE-like protein
bin003	SOY3_bin003_02283	855	12	21	4	1.678	2.491	0.497	Chaperone protein ClpB
bin003	SOY3_bin003_02284	642	2	10	2	0.372	1.580	0.331	hypothetical protein
bin003	SOY3_bin003_02285	561	69	127	27	14.704	22.961	5.112	LemA family protein
bin003	SOY3_bin003_02286	372	2	8	2	0.643	2.181	0.571	5-nitrosalicylic acid 1,2-dioxygenase
bin003	SOY3_bin003_02287	624	12	17	4	2.299	2.763	0.681	putative cobalt-precorrin-6Y C(5)-methyltransferase
bin003	SOY3_bin003_02288	1734	15	22	6	1.034	1.287	0.368	hypothetical protein
bin003	SOY3_bin003_02289	972	1	4	2	0.123	0.417	0.219	hypothetical protein
bin003	SOY3_bin003_02290	966	1	1	0	0.124	0.105	0.000	hypothetical protein
bin003	SOY3_bin003_02291	1305	2	2	0	0.183	0.155	0.000	hypothetical protein
bin003	SOY3_bin003_02292	1137	0	1	0	0.000	0.089	0.000	hypothetical protein
bin003	SOY3_bin003_02293	891	2	0	0	0.268	0.000	0.000	hypothetical protein
bin003	SOY3_bin003_02294	1224	39	74	17	3.809	6.132	1.475	M protein, serotype 5 precursor

bin003	SOY3_bin003_02295	927	15	11	11	1.934	1.204	1.260	hypothetical protein
bin003	SOY3_bin003_02296	1770	7	20	7	0.473	1.146	0.420	hypothetical protein
bin003	SOY3_bin003_02297	1404	19	41	2	1.618	2.962	0.151	UDP-glucose:undecaprenyl-phosphate glucose-1-phosphate transferase
bin003	SOY3_bin003_02298	858	6	7	4	0.836	0.827	0.495	hypothetical protein
bin003	SOY3_bin003_02299	2424	11	31	8	0.543	1.297	0.351	Putative glycosyltransferase EpsH
bin003	SOY3_bin003_02300	1305	7	19	4	0.641	1.477	0.326	UDP-N-acetyl-D-glucosamine 6-dehydrogenase
bin003	SOY3_bin003_02301	1347	2	10	1	0.178	0.753	0.079	Glycosyl transferase family 2
bin003	SOY3_bin003_02302	591	13	38	4	2.630	6.522	0.719	hypothetical protein
bin003	SOY3_bin003_02303	1080	14	35	1	1.550	3.287	0.098	putative metallophosphoesterase
bin003	SOY3_bin003_02304	459	11	28	6	2.865	6.187	1.389	hypothetical protein
bin003	SOY3_bin003_02305	912	17	31	13	2.228	3.448	1.514	Methyl-coenzyme M reductase operon protein C
bin003	SOY3_bin003_02306	870	9	17	2	1.237	1.982	0.244	CAAX amino terminal protease self- immunity
bin003	SOY3_bin003_02307	549	8	17	5	1.742	3.141	0.967	hypothetical protein
bin003	SOY3_bin003_02308	438	0	1	0	0.000	0.232	0.000	Nucleotidyltransferase domain protein
bin003	SOY3_bin003_02309	813	9	11	1	1.323	1.372	0.131	hypothetical protein
bin003	SOY3_bin003_02310	1707	13	26	4	0.910	1.545	0.249	hypothetical protein
bin003	SOY3_bin003_02311	1011	5	3	1	0.591	0.301	0.105	hypothetical protein
bin003	SOY3_bin003_02312	1188	3	4	1	0.302	0.342	0.089	Ribosome-binding ATPase YchF
bin003	SOY3_bin003_02313	267	0	0	1	0.000	0.000	0.398	hypothetical protein
bin003	SOY3_bin003_02314	198	2	7	2	1.208	3.586	1.073	hypothetical protein
bin003	SOY3_bin003_02315	504	0	0	0	0.000	0.000	0.000	hypothetical protein
bin003	SOY3_bin003_02316	189	0	1	0	0.000	0.537	0.000	cobalt transport protein CbiM
bin003	SOY3_bin003_02317	498	0	0	0	0.000	0.000	0.000	Cobalt transport protein CbiM precursor
bin003	SOY3_bin003_02318	435	0	3	1	0.000	0.699	0.244	hypothetical protein
bin003	SOY3_bin003_02319	582	0	0	0	0.000	0.000	0.000	putative xanthine dehydrogenase subunit A
bin003	SOY3_bin003_02320	420	10	11	4	2.846	2.656	1.012	hypothetical protein
bin003	SOY3_bin003_02321	1059	11	15	4	1.242	1.437	0.401	Antilisterial bacteriocin subtilosin biosynthesis protein AlbA
bin003	SOY3_bin003_02322	744	1	5	1	0.161	0.682	0.143	hypothetical protein
bin003	SOY3_bin003_02323	573	0	9	0	0.000	1.593	0.000	FmdE, Molybdenum formylmethanofuran dehydrogenase operon
bin003	SOY3_bin003_02324	699	5	19	8	0.855	2.757	1.216	Magnesium-protoporphyrin O-methyltransferase
bin003	SOY3_bin003_02325	576	5	18	5	1.038	3.170	0.922	FmdE, Molybdenum formylmethanofuran dehydrogenase operon
bin003	SOY3_bin003_02326	606	4	6	0	0.789	1.004	0.000	Glycine/sarcosine/dimethylglycine N-methyltransferase
bin003	SOY3_bin003_02327	1086	2	2	0	0.220	0.187	0.000	High-affinity heme uptake system protein IsdE precursor
bin003	SOY3_bin003_02328	1020	0	0	0	0.000	0.000	0.000	putative ABC transporter permease protein
bin003	SOY3_bin003_02329	774	0	0	0	0.000	0.000	0.000	putative ABC transporter ATP-binding protein
bin003	SOY3_bin003_02330	168	0	1	0	0.000	0.604	0.000	Ribbon-helix-helix domain protein
bin003	SOY3_bin003_02331	378	2	0	2	0.633	0.000	0.562	50S ribosomal protein L40e
bin003	SOY3_bin003_02332	390	3	6	0	0.920	1.560	0.000	tRNA(fMet)-specific endonuclease VapC
bin003	SOY3_bin003_02333	246	2	6	0	0.972	2.474	0.000	SpoVT / AbrB like domain protein
bin003	SOY3_bin003_02334	426	5	2	0	1.403	0.476	0.000	hypothetical protein
bin003	SOY3_bin003_02335	264	5	5	2	2.264	1.921	0.805	hypothetical protein
bin003	SOY3_bin003_02336	384	4	4	0	1.245	1.057	0.000	Ribonuclease VapC20
bin003	SOY3_bin003_02337	312	2	0	0	0.766	0.000	0.000	hypothetical protein
bin003	SOY3_bin003_02338	1191	1	2	2	0.100	0.170	0.178	cell division control protein 6
bin003	SOY3_bin003_02339	780	3	10	7	0.460	1.300	0.953	CAAX amino terminal protease self- immunity
bin003	SOY3_bin003_02340	237	3	3	0	1.513	1.284	0.000	putative regulator PrlF
bin003	SOY3_bin003_02341	390	4	3	0	1.226	0.780	0.000	Ribonuclease VapC20
bin003	SOY3_bin003_02342	1170	2	0	0	0.204	0.000	0.000	site-specific tyrosine recombinase XerD
bin003	SOY3_bin003_02343	489	1	0	0	0.244	0.000	0.000	hypothetical protein
bin003	SOY3_bin003_02344	252	0	0	0	0.000	0.000	0.000	hypothetical protein
bin003	SOY3_bin003_02345	147	0	0	0	0.000	0.000	0.000	hypothetical protein
bin003	SOY3_bin003_02346	192	28	40	2	17.434	21.131	1.107	hypothetical protein
bin003	SOY3_bin003_02347	423	72	84	21	20.349	20.142	5.274	hypothetical protein
bin003	SOY3_bin003_02348	354	10	11	1	3.377	3.152	0.300	Alkaline phosphatase synthesis transcriptional regulatory protein PhoP
bin003	SOY3_bin003_02349	924	17	22	1	2.199	2.415	0.115	hypothetical protein
bin003	SOY3_bin003_02350	249	5	5	0	2.401	2.037	0.000	50S ribosomal protein L40e
bin003	SOY3_bin003_02351	681	10	18	5	1.755	2.681	0.780	Zinc dependent phospholipase C
bin003	SOY3_bin003_02352	537	4	10	1	0.890	1.889	0.198	N-glycosyltransferase
bin003	SOY3_bin003_02353	1230	34	41	11	3.305	3.381	0.950	peptide chain release factor 1
bin003	SOY3_bin003_02354	1782	18	26	7	1.208	1.480	0.417	Phytochrome-like protein cph1
bin003	SOY3_bin003_02355	141	3	3	0	2.544	2.158	0.000	hypothetical protein
bin003	SOY3_bin003_02356	225	3	5	1	1.594	2.254	0.472	hypothetical protein
bin003	SOY3_bin003_02357	822	40	89	20	5.817	10.982	2.585	Chaperone protein DnaJ
bin003	SOY3_bin003_02358	219	3	9	3	1.638	4.168	1.455	2-hydroxymuconate tautomerase
bin003	SOY3_bin003_02359	537	4	8	0	0.890	1.511	0.000	HEPN domain protein
bin003	SOY3_bin003_02360	579	3	4	0	0.619	0.701	0.000	Nucleotidyltransferase domain protein
bin003	SOY3_bin003_02361	153	0	0	0	0.000	0.000	0.000	4-oxalocrotonate tautomerase

bin003	SOY3_bin003_02362	186	2	15	1	1.285	8.180	0.571	hypothetical protein
bin003	SOY3_bin003_02363	399	0	1	0	0.000	0.254	0.000	hypothetical protein
bin003	SOY3_bin003_02364	1371	18	38	4	1.570	2.811	0.310	Multidrug resistance protein 3
bin003	SOY3_bin003_02365	372	2	2	1	0.643	0.545	0.286	UvrABC system protein C
bin003	SOY3_bin003_02366	1263	24	31	10	2.272	2.490	0.841	putative transcriptional regulatory protein pdtaR
bin003	SOY3_bin003_02367	753	21	32	10	3.334	4.310	1.411	Circadian clock protein kinase KaiC
bin003	SOY3_bin003_02368	330	3	3	2	1.087	0.922	0.644	Benzene 1,2-dioxygenase system ferredoxin subunit
bin003	SOY3_bin003_02369	1122	22	35	11	2.344	3.164	1.041	ATP-dependent zinc metalloprotease FtsH
bin003	SOY3_bin003_02370	843	8	5	2	1.135	0.602	0.252	putative endonuclease 4
bin003	SOY3_bin003_02371	678	6	12	5	1.058	1.795	0.783	Thioredoxin reductase
bin003	SOY3_bin003_02372	483	0	3	0	0.000	0.630	0.000	hypothetical protein
bin003	SOY3_bin003_02373	516	1	1	0	0.232	0.197	0.000	hypothetical protein
bin003	SOY3_bin003_02374	939	4	6	3	0.509	0.648	0.339	hypothetical protein
bin003	SOY3_bin003_02375	777	1	1	1	0.154	0.131	0.137	hypothetical protein
bin003	SOY3_bin003_02376	762	0	0	0	0.000	0.000	0.000	hypothetical protein
bin003	SOY3_bin003_02377	462	1	1	1	0.259	0.220	0.230	hypothetical protein
bin003	SOY3_bin003_02378	492	4	6	7	0.972	1.237	1.511	Methylthioribose-1-phosphate isomerase
bin003	SOY3_bin003_02379	435	3	3	2	0.824	0.699	0.488	Uracil DNA glycosylase superfamily protein
bin003	SOY3_bin003_02380	582	7	9	7	1.438	1.568	1.278	TPR repeat-containing protein YrrB
bin003	SOY3_bin003_02381	930	6	24	3	0.771	2.617	0.343	CAAX amino terminal protease self- immunity
bin003	SOY3_bin003_02382	1023	10	20	6	1.169	1.983	0.623	23S rRNA (uracil-C(5))-methyltransferase RlmCD
bin003	SOY3_bin003_02383	792	6	9	2	0.906	1.153	0.268	diphthine synthase
bin003	SOY3_bin003_02384	1281	5	9	3	0.467	0.713	0.249	hypothetical protein
bin003	SOY3_bin003_02385	852	3	7	2	0.421	0.833	0.249	Modification methylase DpnIIA
bin003	SOY3_bin003_02386	1365	155	608	141	13.575	45.178	10.973	Pseudomurein-binding repeat protein
bin003	SOY3_bin003_02387	963	13	37	6	1.614	3.897	0.662	Modulator of FtsH protease HflK
bin003	SOY3_bin003_02388	438	7	30	8	1.911	6.947	1.940	Inner membrane protein YbbJ
bin003	SOY3_bin003_02389	882	11	25	7	1.491	2.875	0.843	N-acetylglucosaminyl-diphospho-decaprenol L-rhamnosyltransferase
bin003	SOY3_bin003_02390	1113	6	23	7	0.644	2.096	0.668	UDP-galactopyranose mutase
bin003	SOY3_bin003_02391	4560	30	106	26	0.787	2.358	0.606	putative glycosyltransferase EpsJ
bin003	SOY3_bin003_02392	117	0	2	0	0.000	1.734	0.000	hypothetical protein
bin003	SOY3_bin003_02393	597	2	14	3	0.400	2.379	0.534	Ultraviolet N-glycosylase/AP lyase
bin003	SOY3_bin003_02394	468	8	15	0	2.044	3.251	0.000	Translation initiation factor IF-2
bin003	SOY3_bin003_02395	1176	4	2	2	0.407	0.172	0.181	hypothetical protein
bin003	SOY3_bin003_02396	966	1013	2283	1032	125.365	239.709	113.483	Phthiodiolone/phenolphthiodiolone dimycocerosates ketoreductase
bin003	SOY3_bin003_02397	615	5	5	1	0.972	0.825	0.173	Thiol:disulfide interchange protein DsbD precursor
bin003	SOY3_bin003_02398	420	6	7	3	1.708	1.690	0.759	Thioredoxin
bin003	SOY3_bin003_02399	798	12	17	5	1.798	2.161	0.666	Radical SAM superfamily protein
bin003	SOY3_bin003_02400	645	3	6	1	0.556	0.944	0.165	putative methyltransferase YcgJ
bin003	SOY3_bin003_02401	288	6	19	6	2.491	6.691	2.213	hypothetical protein
bin003	SOY3_bin003_02402	141	2	5	0	1.696	3.597	0.000	putative oxidoreductase
bin007	SOY3_bin007_00001	105	0	0	0	0.000	0.000	0.000	5S ribosomal RNA
bin007	SOY3_bin007_00002	453	0	0	0	0.000	0.000	0.000	hypothetical protein
bin007	SOY3_bin007_00003	1599	5193	5357	4896	388.254	339.804	325.254	16S ribosomal RNA
bin007	SOY3_bin007_00004	585	0	9	4	0.000	1.560	0.726	hypothetical protein
bin007	SOY3_bin007_00005	1284	4	20	6	0.372	1.580	0.496	Bacillibactin exporter
bin007	SOY3_bin007_00006	978	81	366	125	9.901	37.957	13.577	Fructose-1,6-bisphosphatase class 2
bin007	SOY3_bin007_00007	783	14	40	16	2.138	5.181	2.171	hypothetical protein
bin007	SOY3_bin007_00008	432	2	19	5	0.553	4.461	1.229	hypothetical protein
bin007	SOY3_bin007_00009	408	2	15	3	0.586	3.729	0.781	Putative universal stress protein
bin007	SOY3_bin007_00010	438	3	16	4	0.819	3.705	0.970	Stress response protein NhaX
bin007	SOY3_bin007_00011	2079	6	42	10	0.345	2.049	0.511	DNA topoisomerase 3
bin007	SOY3_bin007_00012	1095	4	15	7	0.437	1.389	0.679	Antilisterial bacteriocin subtilosin biosynthesis protein AlbA
bin007	SOY3_bin007_00013	285	1	6	2	0.419	2.135	0.745	hypothetical protein
bin007	SOY3_bin007_00014	714	0	5	2	0.000	0.710	0.298	Phospholipase C precursor
bin007	SOY3_bin007_00015	3516	700	1256	349	23.801	36.232	10.544	Pyruvate-flavodoxin oxidoreductase
bin007	SOY3_bin007_00016	1401	470	656	223	40.106	47.492	16.908	Methylmalonyl-CoA carboxyltransferase 5S subunit
bin007	SOY3_bin007_00017	939	281	419	134	35.776	45.259	15.159	Malate dehydrogenase
bin007	SOY3_bin007_00018	414	143	212	61	41.293	51.939	15.652	Glutaconyl-CoA decarboxylase subunit gamma
bin007	SOY3_bin007_00019	189	97	138	44	61.356	74.058	24.730	hypothetical protein
bin007	SOY3_bin007_00020	1527	564	779	230	44.156	51.743	16.000	Methylmalonyl-CoA carboxyltransferase 12S subunit
bin007	SOY3_bin007_00021	402	136	254	64	40.444	64.086	16.912	4-hydroxymandelate synthase
bin007	SOY3_bin007_00022	399	212	231	71	63.520	58.721	18.902	Methylmalonyl-CoA mutase
bin007	SOY3_bin007_00023	1674	628	833	250	44.849	50.471	15.864	Methylmalonyl-CoA mutase
bin007	SOY3_bin007_00024	873	251	394	112	34.372	45.776	13.628	Succinyl-CoA ligase [ADP-forming] subunit alpha
bin007	SOY3_bin007_00025	1116	363	478	147	38.886	43.443	13.992	Succinyl-CoA ligase [ADP-forming] subunit beta
bin007	SOY3_bin007_00026	555	135	125	45	29.079	22.844	8.613	Fumarate hydratase class I, aerobic

bin007	SOY3_bin007_00027	912	121	122	41	15.861	13.568	4.775	L(+)-tartrate dehydratase subunit alpha
bin007	SOY3_bin007_00028	1707	3	8	6	0.210	0.475	0.373	Transcriptional regulatory protein ZraR
bin007	SOY3_bin007_00029	612	20	42	14	3.907	6.961	2.430	LexA repressor
bin007	SOY3_bin007_00030	1038	8	55	20	0.921	5.374	2.047	C4-dicarboxylate-binding periplasmic protein precursor
bin007	SOY3_bin007_00031	678	1	10	3	0.176	1.496	0.470	Transcriptional regulatory protein DcuR
bin007	SOY3_bin007_00032	1641	4	53	10	0.291	3.276	0.647	Sensor histidine kinase DcuS
bin007	SOY3_bin007_00033	918	16	52	20	2.084	5.745	2.314	HTH-type transcriptional activator CmpR
bin007	SOY3_bin007_00034	1251	21	60	17	2.007	4.865	1.444	Methionine gamma-lyase
bin007	SOY3_bin007_00035	957	20	43	16	2.498	4.557	1.776	Stage V sporulation protein K
bin007	SOY3_bin007_00036	243	7	21	3	3.444	8.765	1.311	RNA-binding protein Hfq
bin007	SOY3_bin007_00037	954	23	54	12	2.882	5.741	1.336	tRNA dimethylallyltransferase
bin007	SOY3_bin007_00038	837	24	43	16	3.428	5.211	2.031	Ribosomal RNA small subunit methyltransferase J
bin007	SOY3_bin007_00039	1833	50	128	40	3.261	7.083	2.318	DNA mismatch repair protein MutL
bin007	SOY3_bin007_00040	1368	26	75	21	2.272	5.561	1.631	Histone deacetylase-like amidohydrolase
bin007	SOY3_bin007_00041	1677	47	99	28	3.351	5.988	1.774	Acetophenone carboxylase alpha subunit
bin007	SOY3_bin007_00042	2616	164	390	132	7.495	15.121	5.360	DNA mismatch repair protein MutS
bin007	SOY3_bin007_00043	396	32	110	41	9.661	28.174	10.998	hypothetical protein
bin007	SOY3_bin007_00044	1323	18	86	26	1.627	6.593	2.088	(Dimethylallyl)adenosine tRNA methylthiotransferase MiaB
bin007	SOY3_bin007_00045	357	1	8	2	0.335	2.273	0.595	hypothetical protein
bin007	SOY3_bin007_00046	636	8	31	5	1.504	4.944	0.835	hypothetical protein
bin007	SOY3_bin007_00047	441	6	28	5	1.627	6.440	1.204	Stress response protein NhaX
bin007	SOY3_bin007_00048	876	4	12	2	0.546	1.389	0.243	Citrate lyase subunit beta
bin007	SOY3_bin007_00049	1251	8	31	9	0.765	2.513	0.764	hypothetical protein
bin007	SOY3_bin007_00050	390	2	5	2	0.613	1.300	0.545	8-oxo-dGTP diphosphatase
bin007	SOY3_bin007_00051	654	13	66	16	2.376	10.236	2.599	putative septum site-determining protein MinC
bin007	SOY3_bin007_00052	561	2	13	3	0.426	2.350	0.568	ADP-ribose pyrophosphatase
bin007	SOY3_bin007_00053	444	2	3	2	0.539	0.685	0.478	Spore protein SP21
bin007	SOY3_bin007_00054	999	14	36	15	1.675	3.655	1.595	2-hydroxyglutaryl-CoA dehydratase, D-component
bin007	SOY3_bin007_00055	1104	24	63	31	2.599	5.788	2.983	hypothetical protein
bin007	SOY3_bin007_00056	1185	18	61	21	1.816	5.221	1.882	putative cysteine desulfurase
bin007	SOY3_bin007_00057	1398	3	36	15	0.257	2.612	1.140	DNA-directed RNA polymerase subunit delta
bin007	SOY3_bin007_00058	621	1	68	4	0.193	11.106	0.684	Acetoin utilization protein AcuA
bin007	SOY3_bin007_00059	1551	39	100	46	3.006	6.539	3.150	Inosine-5'-monophosphate dehydrogenase
bin007	SOY3_bin007_00060	234	0	1	0	0.000	0.433	0.000	hypothetical protein
bin007	SOY3_bin007_00061	129	0	0	0	0.000	0.000	0.000	hypothetical protein
bin007	SOY3_bin007_00062	633	0	1	0	0.000	0.160	0.000	Phosphoserine phosphatase 1
bin007	SOY3_bin007_00063	1128	2	9	4	0.212	0.809	0.377	Threonine-phosphate decarboxylase
bin007	SOY3_bin007_00064	750	0	2	6	0.000	0.270	0.850	Cobalamin synthase
bin007	SOY3_bin007_00065	966	4	5	2	0.495	0.525	0.220	cobalamin biosynthesis protein
bin007	SOY3_bin007_00066	1536	5	11	3	0.389	0.726	0.207	Cobyric acid synthase
bin007	SOY3_bin007_00067	573	7	10	1	1.460	1.770	0.185	Bifunctional adenosylcobalamin biosynthesis protein CobP
bin007	SOY3_bin007_00068	429	4	12	4	1.115	2.837	0.990	hypothetical protein
bin007	SOY3_bin007_00069	279	9	17	9	3.856	6.180	3.427	hypothetical protein
bin007	SOY3_bin007_00070	1173	8	21	12	0.815	1.816	1.087	LL-diaminopimelate aminotransferase
bin007	SOY3_bin007_00071	975	8	23	5	0.981	2.393	0.545	Error-prone DNA polymerase
bin007	SOY3_bin007_00072	261	14	39	15	6.413	15.156	6.105	Stage V sporulation protein S
bin007	SOY3_bin007_00073	783	6	34	7	0.916	4.404	0.950	hypothetical protein
bin007	SOY3_bin007_00074	531	5	23	7	1.126	4.393	1.400	hypothetical protein
bin007	SOY3_bin007_00075	1539	32	87	34	2.486	5.734	2.347	Ribonuclease Y
bin007	SOY3_bin007_00076	492	9	38	13	2.187	7.834	2.807	Regulatory protein RecX
bin007	SOY3_bin007_00077	1044	29	131	50	3.321	12.727	5.087	recombinase A
bin007	SOY3_bin007_00078	1596	10	24	10	0.749	1.525	0.666	DEAD-box ATP-dependent RNA helicase CshA
bin007	SOY3_bin007_00079	510	7	25	10	1.641	4.972	2.083	Nicotinamide-nucleotide amidohydrolase PncC
bin007	SOY3_bin007_00080	1470	4	17	13	0.325	1.173	0.939	ATP-dependent zinc metalloprotease FtsH
bin007	SOY3_bin007_00081	576	2	12	1	0.415	2.113	0.184	hypothetical protein
bin007	SOY3_bin007_00082	1800	2	2	3	0.133	0.113	0.177	Transposase DDE domain protein
bin007	SOY3_bin007_00083	546	1	6	4	0.219	1.115	0.778	CDP-diacylglycerol--glycerol-3-phosphate 3-phosphatidyltransferase
bin007	SOY3_bin007_00084	1326	5	13	5	0.451	0.994	0.401	Ribosomal protein S12 methylthiotransferase RimO
bin007	SOY3_bin007_00085	507	3	7	2	0.707	1.400	0.419	putative nucleotide-binding protein
bin007	SOY3_bin007_00086	99	0	1	1	0.000	1.025	1.073	hypothetical protein
bin007	SOY3_bin007_00087	1617	3	8	3	0.222	0.502	0.197	DAK2 domain protein
bin007	SOY3_bin007_00088	840	2	7	5	0.285	0.845	0.632	DegV domain-containing protein
bin007	SOY3_bin007_00089	774	9	22	6	1.390	2.883	0.823	Cytoskeleton protein RodZ
bin007	SOY3_bin007_00090	2262	19	51	11	1.004	2.287	0.517	DNA translocase SpoIIIE
bin007	SOY3_bin007_00091	786	2	10	5	0.304	1.290	0.676	Undecaprenyl-diphosphatase
bin007	SOY3_bin007_00092	213	2	2	1	1.123	0.952	0.499	hypothetical protein
bin007	SOY3_bin007_00093	846	2	9	2	0.283	1.079	0.251	Translocation-enhancing protein TepA

bin007	SOY3_bin007_00094	1668	88	234	69	6.307	14.229	4.394	Ribonuclease J 1
bin007	SOY3_bin007_00095	885	12	67	18	1.621	7.679	2.161	4-hydroxy-tetrahydrodipicolinate synthase
bin007	SOY3_bin007_00096	1227	14	60	22	1.364	4.960	1.905	Aspartokinase
bin007	SOY3_bin007_00097	1020	18	54	26	2.110	5.370	2.708	Aspartate-semialdehyde dehydrogenase
bin007	SOY3_bin007_00098	594	5	17	4	1.006	2.903	0.715	Dipicolinate synthase subunit B
bin007	SOY3_bin007_00099	885	16	77	28	2.161	8.825	3.361	Dipicolinate synthase subunit A
bin007	SOY3_bin007_00100	807	2	17	3	0.296	2.137	0.395	4-hydroxy-tetrahydrodipicolinate reductase
bin007	SOY3_bin007_00101	114	0	0	0	0.000	0.000	0.000	hypothetical protein
bin007	SOY3_bin007_00102	204	0	0	0	0.000	0.000	0.000	hypothetical protein
bin007	SOY3_bin007_00103	1020	4	26	9	0.469	2.585	0.937	Serine dehydrogenase proteinase
bin007	SOY3_bin007_00104	255	0	4	2	0.000	1.591	0.833	Spo0E like sporulation regulatory protein
bin007	SOY3_bin007_00105	852	1	7	4	0.140	0.833	0.499	2-amino-4-deoxychorismate dehydrogenase
bin007	SOY3_bin007_00106	1299	1	9	4	0.092	0.703	0.327	Sporulation kinase E
bin007	SOY3_bin007_00107	123	6	49	17	5.832	40.406	14.682	hypothetical protein
bin007	SOY3_bin007_00108	645	10	77	23	1.853	12.108	3.788	Accessory gene regulator protein B
bin007	SOY3_bin007_00109	462	1	4	10	0.259	0.878	2.299	hypothetical protein
bin007	SOY3_bin007_00110	261	0	4	2	0.000	1.554	0.814	GIY-YIG nuclease superfamily protein
bin007	SOY3_bin007_00111	786	16	42	18	2.434	5.420	2.433	hypothetical protein
bin007	SOY3_bin007_00112	1194	7	19	3	0.701	1.614	0.267	Xaa-Pro dipeptidase
bin007	SOY3_bin007_00113	1392	3	12	2	0.258	0.874	0.153	Asparagine--tRNA ligase
bin007	SOY3_bin007_00114	1077	3	8	7	0.333	0.753	0.690	Putative ammonia monooxygenase
bin007	SOY3_bin007_00115	468	3	15	0	0.766	3.251	0.000	Bacterial transcription activator, effector binding domain
bin007	SOY3_bin007_00116	585	1	1	0	0.204	0.173	0.000	HTH-type transcriptional regulator MtrR
bin007	SOY3_bin007_00117	1131	4	9	2	0.423	0.807	0.188	Macrolide export protein MacA
bin007	SOY3_bin007_00118	3108	3	18	4	0.115	0.587	0.137	Swarming motility protein SwrC
bin007	SOY3_bin007_00119	456	9	26	5	2.360	5.783	1.165	acyl-CoA thioesterase YbgC
bin007	SOY3_bin007_00120	1749	17	66	26	1.162	3.827	1.579	Gamma-D-glutamyl-L-lysine endopeptidase
bin007	SOY3_bin007_00121	765	2	25	2	0.313	3.315	0.278	Phosphotransferase enzyme family protein
bin007	SOY3_bin007_00122	753	0	1	1	0.000	0.135	0.141	Molybdopterin-guanine dinucleotide biosynthesis adapter protein
bin007	SOY3_bin007_00123	543	0	0	1	0.000	0.000	0.196	Heptaprenyl diphosphate synthase component I
bin007	SOY3_bin007_00124	408	0	4	0	0.000	0.994	0.000	hypothetical protein
bin007	SOY3_bin007_00125	735	0	6	1	0.000	0.828	0.145	Energy-coupling factor transporter transmembrane protein EcfT
bin007	SOY3_bin007_00126	681	0	3	1	0.000	0.447	0.156	Energy-coupling factor transporter ATP-binding protein EcfA2
bin007	SOY3_bin007_00127	705	0	7	2	0.000	1.007	0.301	Energy-coupling factor transporter ATP-binding protein EcfA1
bin007	SOY3_bin007_00128	1566	4	22	10	0.305	1.425	0.678	Alkaline phosphatase precursor
bin007	SOY3_bin007_00129	1419	5	63	19	0.421	4.503	1.422	Endoglucanase precursor
bin007	SOY3_bin007_00130	519	2	22	5	0.461	4.299	1.023	Oxidoreductase molybdopterin binding domain protein
bin007	SOY3_bin007_00131	876	17	52	19	2.320	6.021	2.304	Putative electron transport protein YccM
bin007	SOY3_bin007_00132	621	6	22	17	1.155	3.593	2.908	electron transport complex protein RnfG
bin007	SOY3_bin007_00133	939	12	56	15	1.528	6.049	1.697	Oxidoreductase molybdopterin binding domain protein
bin007	SOY3_bin007_00134	783	16	95	27	2.443	12.306	3.663	hypothetical protein
bin007	SOY3_bin007_00135	699	3	12	8	0.513	1.741	1.216	Undecaprenol kinase
bin007	SOY3_bin007_00136	459	2	7	2	0.521	1.547	0.463	Endoribonuclease YbeY
bin007	SOY3_bin007_00137	2181	9	27	21	0.493	1.256	1.023	hypothetical protein
bin007	SOY3_bin007_00138	981	6	6	5	0.731	0.620	0.541	PhoH-like protein
bin007	SOY3_bin007_00139	1257	4	9	6	0.380	0.726	0.507	Putative stage IV sporulation protein YqfD
bin007	SOY3_bin007_00140	282	0	0	0	0.000	0.000	0.000	YabP family protein
bin007	SOY3_bin007_00141	504	25	47	22	5.930	9.458	4.637	hypothetical protein
bin007	SOY3_bin007_00142	1002	22	55	20	2.625	5.567	2.120	SigmaW regulon antibacterial
bin007	SOY3_bin007_00143	501	5	12	8	1.193	2.429	1.696	hypothetical protein
bin007	SOY3_bin007_00144	177	17	83	33	11.482	47.562	19.805	30S ribosomal protein S21
bin007	SOY3_bin007_00145	345	22	64	20	7.623	18.815	6.158	HIT-like protein
bin007	SOY3_bin007_00146	1311	11	32	14	1.003	2.476	1.134	Threonylcarbamoyladenosine tRNA methylthiotransferase MtaB
bin007	SOY3_bin007_00147	753	18	27	9	2.858	3.637	1.270	Ribosomal RNA small subunit methyltransferase E
bin007	SOY3_bin007_00148	204	3	11	5	1.758	5.469	2.604	putative tautomerase
bin007	SOY3_bin007_00149	924	16	41	17	2.070	4.501	1.954	Ribosomal protein L11 methyltransferase
bin007	SOY3_bin007_00150	1137	33	67	32	3.470	5.977	2.990	Chaperone protein DnaJ
bin007	SOY3_bin007_00151	1839	25	105	37	1.625	5.791	2.137	Chaperone protein DnaK
bin007	SOY3_bin007_00152	609	6	22	12	1.178	3.664	2.093	heat shock protein GrpE
bin007	SOY3_bin007_00153	1569	8	37	10	0.610	2.392	0.677	60 kDa chaperonin
bin007	SOY3_bin007_00154	1047	2	124	24	0.228	12.012	2.435	Heat-inducible transcription repressor HrcA
bin007	SOY3_bin007_00155	1137	11	26	21	1.157	2.319	1.962	Oxygen-independent coproporphyrinogen-III oxidase 1
bin007	SOY3_bin007_00156	1806	5	44	20	0.331	2.471	1.176	Elongation factor 4
bin007	SOY3_bin007_00157	414	1	3	0	0.289	0.735	0.000	hypothetical protein
bin007	SOY3_bin007_00158	1107	10	24	4	1.080	2.199	0.384	Stage II sporulation protein P (SpoIIP)
bin007	SOY3_bin007_00159	339	4	13	8	1.411	3.890	2.507	Membrane protein of unknown function
bin007	SOY3_bin007_00160	267	14	71	33	6.268	26.971	13.129	30S ribosomal protein S20

bin007	SOY3_bin007_00161	1056	6	35	10	0.679	3.362	1.006	DNA polymerase III subunit delta
bin007	SOY3_bin007_00162	333	1	10	3	0.359	3.046	0.957	hypothetical protein
bin007	SOY3_bin007_00163	1533	3	15	3	0.234	0.992	0.208	2-isopropylmalate synthase
bin007	SOY3_bin007_00164	1806	10	28	12	0.662	1.573	0.706	Oligoendopeptidase F, plasmid
bin007	SOY3_bin007_00165	165	5	11	8	3.623	6.762	5.150	hypothetical protein
bin007	SOY3_bin007_00166	96	4	4	1	4.981	4.226	1.107	hypothetical protein
bin007	SOY3_bin007_00167	285	5	30	11	2.097	10.677	4.100	hypothetical protein
bin007	SOY3_bin007_00168	1902	2	1	3	0.126	0.053	0.168	putative ABC transporter ATP-binding protein
bin007	SOY3_bin007_00169	1731	2	4	3	0.138	0.234	0.184	putative ABC transporter ATP-binding protein
bin007	SOY3_bin007_00170	465	0	0	0	0.000	0.000	0.000	putative HTH-type transcriptional regulator YusO
bin007	SOY3_bin007_00171	882	7	55	23	0.949	6.325	2.770	Vitamin B12-binding protein precursor
bin007	SOY3_bin007_00172	1176	6	16	7	0.610	1.380	0.632	Multidrug resistance protein MdtE precursor
bin007	SOY3_bin007_00173	3120	17	43	11	0.651	1.398	0.375	Multidrug resistance protein MdtB
bin007	SOY3_bin007_00174	726	0	4	1	0.000	0.559	0.146	Demethylmenaquinone methyltransferase
bin007	SOY3_bin007_00175	1116	0	15	1	0.000	1.363	0.095	HTH-type transcriptional regulator Xre
bin007	SOY3_bin007_00176	201	0	1	0	0.000	0.505	0.000	hypothetical protein
bin007	SOY3_bin007_00177	3486	9	36	10	0.309	1.047	0.305	Immune inhibitor A precursor
bin007	SOY3_bin007_00178	1308	40	94	40	3.656	7.289	3.248	hypothetical protein
bin007	SOY3_bin007_00179	423	8	20	10	2.261	4.796	2.511	hypothetical protein
bin007	SOY3_bin007_00180	552	6	30	5	1.299	5.512	0.962	Pyruvate kinase, alpha/beta domain
bin007	SOY3_bin007_00181	873	4	33	22	0.548	3.834	2.677	ATP phosphoribosyltransferase
bin007	SOY3_bin007_00182	1167	5	7	2	0.512	0.608	0.182	putative cell wall hydrolase LytN precursor
bin007	SOY3_bin007_00183	195	1	12	4	0.613	6.242	2.179	hypothetical protein
bin007	SOY3_bin007_00184	1191	15	43	16	1.506	3.662	1.427	Aspartate aminotransferase
bin007	SOY3_bin007_00185	480	5	10	1	1.245	2.113	0.221	hypothetical protein
bin007	SOY3_bin007_00186	198	2	6	3	1.208	3.074	1.609	hypothetical protein
bin007	SOY3_bin007_00187	768	3	11	5	0.467	1.453	0.692	Germination-specific N-acetylmuramoyl-L-alanine amidase precursor
bin007	SOY3_bin007_00188	1146	1	9	3	0.104	0.797	0.278	Putative oxidoreductase/MT0587
bin007	SOY3_bin007_00189	1902	40	101	31	2.514	5.386	1.731	Selenocysteine-specific elongation factor
bin007	SOY3_bin007_00190	1422	5	24	7	0.420	1.712	0.523	L-seryl-tRNA(Sec) selenium transferase
bin007	SOY3_bin007_00191	2838	51	163	65	2.148	5.825	2.433	Phosphoribosylformylglycinamidine synthase 2
bin007	SOY3_bin007_00192	780	11	38	15	1.686	4.941	2.043	Phosphoribosylformylglycinamidine synthase 1
bin007	SOY3_bin007_00193	777	2	11	3	0.308	1.436	0.410	Putative L,D-transpeptidase YkuD
bin007	SOY3_bin007_00194	840	9	28	13	1.281	3.381	1.644	Chorismate dehydratase
bin007	SOY3_bin007_00195	777	10	29	16	1.539	3.786	2.187	Octaprenyl-diphosphate synthase
bin007	SOY3_bin007_00196	381	34	221	81	10.668	58.833	22.583	S-adenosylmethionine decarboxylase proenzyme precursor
bin007	SOY3_bin007_00197	756	6	8	7	0.949	1.073	0.984	Ureidoglycolate lyase
bin007	SOY3_bin007_00198	189	11	26	9	6.958	13.953	5.058	hypothetical protein
bin007	SOY3_bin007_00199	198	7	6	2	4.226	3.074	1.073	hypothetical protein
bin007	SOY3_bin007_00200	153	21	50	30	16.409	33.146	20.829	hypothetical protein
bin007	SOY3_bin007_00201	141	0	5	0	0.000	3.597	0.000	hypothetical protein
bin007	SOY3_bin007_00202	189	2	6	1	1.265	3.220	0.562	Ferredoxin
bin007	SOY3_bin007_00203	642	2	9	6	0.372	1.422	0.993	Succinate dehydrogenase iron-sulfur subunit
bin007	SOY3_bin007_00204	1683	7	32	13	0.497	1.929	0.821	Succinate dehydrogenase flavoprotein subunit
bin007	SOY3_bin007_00205	1338	10	36	12	0.893	2.729	0.953	2-methylcitrate dehydratase
bin007	SOY3_bin007_00206	1935	63	216	101	3.892	11.322	5.545	NADH oxidase
bin007	SOY3_bin007_00207	123	0	3	1	0.000	2.474	0.864	hypothetical protein
bin007	SOY3_bin007_00208	1965	8	24	7	0.487	1.239	0.378	Acetoin dehydrogenase operon transcriptional activator AcoR
bin007	SOY3_bin007_00209	486	0	5	1	0.000	1.043	0.219	Cys-tRNA(Pro)/Cys-tRNA(Cys) deacylase YbaK
bin007	SOY3_bin007_00210	78	0	6	3	0.000	7.802	4.086	tRNA-Pro(ggg)
bin007	SOY3_bin007_00211	504	0	13	1	0.000	2.616	0.211	GDP-mannose mannosyl hydrolase
bin007	SOY3_bin007_00212	639	39	175	63	7.296	27.777	10.473	Formate dehydrogenase subunit alpha precursor
bin007	SOY3_bin007_00213	2370	193	749	246	9.735	32.054	11.026	Formate dehydrogenase, nitrate-inducible, major subunit precursor
bin007	SOY3_bin007_00214	76	0	0	0	0.000	0.000	0.000	tRNA-Ala(ggc)
bin007	SOY3_bin007_00215	855	3	16	3	0.419	1.898	0.373	Glycine/sarcosine/dimethylglycine N-methyltransferase
bin007	SOY3_bin007_00216	1053	0	6	4	0.000	0.578	0.404	Mannosyltransferase (PIG-V))
bin007	SOY3_bin007_00217	423	2	8	0	0.565	1.918	0.000	GtrA-like protein
bin007	SOY3_bin007_00218	756	3	7	6	0.474	0.939	0.843	Undecaprenyl-phosphate 4-deoxy-4-formamido-L-arabinose transferase
bin007	SOY3_bin007_00219	288	36	90	31	14.944	31.696	11.434	molybdopterin synthase small subunit
bin007	SOY3_bin007_00220	228	4	15	2	2.097	6.673	0.932	hypothetical protein
bin007	SOY3_bin007_00221	156	5	7	5	3.832	4.551	3.405	hypothetical protein
bin007	SOY3_bin007_00222	261	12	57	20	5.497	22.151	8.140	Transposase zinc-ribbon domain protein
bin007	SOY3_bin007_00223	669	8	44	26	1.430	6.671	4.128	LexA repressor
bin007	SOY3_bin007_00224	429	3	7	1	0.836	1.655	0.248	Peroxide-responsive repressor PerR
bin007	SOY3_bin007_00225	1095	4	6	1	0.437	0.556	0.097	High-affinity zinc uptake system binding-protein ZnuA precursor
bin007	SOY3_bin007_00226	738	4	4	1	0.648	0.550	0.144	High-affinity zinc uptake system ATP-binding protein ZnuC
bin007	SOY3_bin007_00227	840	5	6	5	0.712	0.724	0.632	High-affinity zinc uptake system membrane protein ZnuB

bin007	SOY3_bin007_00228	1206	42	120	40	4.163	10.092	3.523	hypothetical protein
bin007	SOY3_bin007_00229	402	30	49	13	8.922	12.363	3.435	Ferredoxin-2
bin007	SOY3_bin007_00230	747	44	102	33	7.042	13.850	4.693	hypothetical protein
bin007	SOY3_bin007_00231	330	3	4	3	1.087	1.229	0.966	hypothetical protein
bin007	SOY3_bin007_00232	1209	16	134	53	1.582	11.242	4.657	DNA polymerase IV
bin007	SOY3_bin007_00233	612	14	136	55	2.735	22.539	9.546	Sulfurtransferase TusA
bin007	SOY3_bin007_00234	873	2	7	4	0.274	0.813	0.487	33 kDa chaperonin
bin007	SOY3_bin007_00235	735	3	10	3	0.488	1.380	0.434	Nitrite reductase [NAD(P)H]
bin007	SOY3_bin007_00236	324	0	5	3	0.000	1.565	0.984	Diguanylate cyclase DosC
bin007	SOY3_bin007_00237	648	33	117	40	6.088	18.313	6.557	hypothetical protein
bin007	SOY3_bin007_00238	903	1	14	5	0.132	1.573	0.588	hypothetical protein
bin007	SOY3_bin007_00239	696	2	6	3	0.344	0.874	0.458	putative membrane protein YdfK
bin007	SOY3_bin007_00240	390	1	2	0	0.307	0.520	0.000	HTH-type transcriptional repressor YtrA
bin007	SOY3_bin007_00241	900	3	4	3	0.398	0.451	0.354	ABC transporter ATP-binding protein YtrB
bin007	SOY3_bin007_00242	816	1	12	1	0.147	1.492	0.130	Phosphosulfolactate synthase
bin007	SOY3_bin007_00243	456	3	17	6	0.787	3.781	1.398	hypothetical protein
bin007	SOY3_bin007_00244	924	32	107	40	4.140	11.745	4.599	Ribosomal large subunit pseudouridine synthase D
bin007	SOY3_bin007_00245	456	8	39	10	2.097	8.675	2.330	Lipoprotein signal peptidase
bin007	SOY3_bin007_00246	1032	2	12	3	0.232	1.179	0.309	hypothetical protein
bin007	SOY3_bin007_00247	480	1	3	2	0.249	0.634	0.443	bifunctional uroporphyrinogen-III synthetase/uroporphyrin-III C-methyltransferase
bin007	SOY3_bin007_00248	2781	8	37	15	0.344	1.349	0.573	Isoleucine--tRNA ligase
bin007	SOY3_bin007_00249	294	6	8	6	2.440	2.760	2.168	hypothetical protein
bin007	SOY3_bin007_00250	636	9	23	8	1.692	3.668	1.336	putative NUDIX hydrolase
bin007	SOY3_bin007_00251	456	1	25	3	0.262	5.561	0.699	Septum site-determining protein DivIVA
bin007	SOY3_bin007_00252	255	6	14	9	2.813	5.569	3.749	hypothetical protein
bin007	SOY3_bin007_00253	279	9	22	10	3.856	7.998	3.807	YGGT family protein
bin007	SOY3_bin007_00254	819	13	60	15	1.898	7.431	1.946	Pyrroline-5-carboxylate reductase
bin007	SOY3_bin007_00255	468	6	30	6	1.533	6.502	1.362	Cell division protein SepF
bin007	SOY3_bin007_00256	684	26	79	37	4.544	11.715	5.746	hypothetical protein
bin007	SOY3_bin007_00257	966	15	90	37	1.856	9.450	4.069	HlyD family secretion protein
bin007	SOY3_bin007_00258	1419	4	26	7	0.337	1.858	0.524	Alkaline phosphatase synthesis sensor protein PhoR
bin007	SOY3_bin007_00259	714	1	23	6	0.167	3.267	0.893	Alkaline phosphatase synthesis transcriptional regulatory protein PhoP
bin007	SOY3_bin007_00260	819	1	12	5	0.146	1.486	0.649	Laccase domain protein
bin007	SOY3_bin007_00261	1560	3	28	7	0.230	1.820	0.477	Ribonuclease
bin007	SOY3_bin007_00262	222	30	93	49	16.155	42.490	23.446	Zinc ribbon domain protein
bin007	SOY3_bin007_00263	1320	16	37	15	1.449	2.843	1.207	Intracellular proteinase inhibitor
bin007	SOY3_bin007_00264	507	3	13	6	0.707	2.601	1.257	K(+)/H(+) antiporter subunit KhtT
bin007	SOY3_bin007_00265	1212	8	25	8	0.789	2.092	0.701	K(+)/H(+) antiporter YhaU
bin007	SOY3_bin007_00266	750	6	52	28	0.956	7.032	3.966	Tyrosine-protein phosphatase YwqE
bin007	SOY3_bin007_00267	279	2	1	0	0.857	0.364	0.000	PRC-barrel domain protein
bin007	SOY3_bin007_00268	774	8	17	3	1.236	2.228	0.412	RNA polymerase sigma-F factor
bin007	SOY3_bin007_00269	732	1	11	3	0.163	1.524	0.435	RNA polymerase sigma-E factor precursor
bin007	SOY3_bin007_00270	918	14	27	5	1.823	2.983	0.579	Sporulation factor SpoIIGA
bin007	SOY3_bin007_00271	927	4	19	13	0.516	2.079	1.490	Selenide, water dikinase
bin007	SOY3_bin007_00272	1050	21	94	37	2.391	9.080	3.743	Cell division protein FtsZ
bin007	SOY3_bin007_00273	1197	85	280	131	8.489	23.726	11.625	Cell division protein FtsA
bin007	SOY3_bin007_00274	342	9	40	14	3.146	11.863	4.348	hypothetical protein
bin007	SOY3_bin007_00275	711	8	27	14	1.345	3.852	2.092	Cell division protein FtsL
bin007	SOY3_bin007_00276	696	9	24	9	1.546	3.497	1.374	hypothetical protein
bin007	SOY3_bin007_00277	744	9	33	8	1.446	4.499	1.142	Cell division protein DivIB
bin007	SOY3_bin007_00278	141	2	12	6	1.696	8.632	4.520	hypothetical protein
bin007	SOY3_bin007_00279	129	2	7	1	1.853	5.504	0.823	hypothetical protein
bin007	SOY3_bin007_00280	1260	9	21	8	0.854	1.690	0.674	UDP-N-acetylglucosamine 1-carboxyvinyltransferase 1
bin007	SOY3_bin007_00281	912	11	23	10	1.442	2.558	1.165	UDP-N-acetylenolpyruvoylglucosamine reductase
bin007	SOY3_bin007_00282	1338	17	34	11	1.519	2.577	0.873	UDP-N-acetylmuramate--L-alanine ligase
bin007	SOY3_bin007_00283	1113	8	7	2	0.859	0.638	0.191	UDP-N-acetylglucosamine--N-acetylmuramyl-(pentapeptide) pyrophosphoryl-undecaprenol N-acetylglucosamine transferase
bin007	SOY3_bin007_00284	1104	8	20	7	0.866	1.837	0.674	Lipid II flippase FtsW
bin007	SOY3_bin007_00285	1359	11	33	8	0.968	2.463	0.625	UDP-N-acetylmuramoylalanine--D-glutamate ligase
bin007	SOY3_bin007_00286	1071	12	17	12	1.339	1.610	1.190	Phospho-N-acetylmuramoyl-pentapeptide-transferase
bin007	SOY3_bin007_00287	1407	18	27	9	1.529	1.946	0.679	UDP-N-acetylmuramoyl-tripeptide--D-alanyl-D-alanine ligase
bin007	SOY3_bin007_00288	1497	17	32	16	1.358	2.168	1.135	UDP-N-acetylmuramoyl-L-alanyl-D-glutamate--2,6-diaminopimelate ligase
bin007	SOY3_bin007_00289	1989	19	42	13	1.142	2.142	0.694	Stage V sporulation protein D
bin007	SOY3_bin007_00290	654	12	18	10	2.194	2.792	1.624	Cell division protein FtsL
bin007	SOY3_bin007_00291	936	20	49	18	2.554	5.310	2.043	Ribosomal RNA small subunit methyltransferase H
bin007	SOY3_bin007_00292	486	39	59	26	9.593	12.313	5.683	cell division protein MraZ
bin007	SOY3_bin007_00293	816	12	41	15	1.758	5.096	1.953	Histidinol-phosphatase
bin007	SOY3_bin007_00294	900	23	82	33	3.055	9.241	3.895	hypothetical protein

bin007	SOY3_bin007_00295	2826	30	117	46	1.269	4.199	1.729	1,4-alpha-glucan branching enzyme
bin007	SOY3_bin007_00296	450	7	51	29	1.860	11.495	6.846	Ferric uptake regulation protein
bin007	SOY3_bin007_00297	1242	10	41	12	0.963	3.348	1.026	D-alanyl-D-alanine carboxypeptidase DacB precursor
bin007	SOY3_bin007_00298	1944	28	82	24	1.722	4.278	1.311	Ferrous iron transport protein B
bin007	SOY3_bin007_00299	219	7	25	10	3.821	11.578	4.850	FeoA domain protein
bin007	SOY3_bin007_00300	1347	6	1	0	0.533	0.075	0.000	Putative sporulation-specific glycosylase YdhD
bin007	SOY3_bin007_00301	1194	11	49	24	1.101	4.162	2.135	putative glycolipid permease LtaA
bin007	SOY3_bin007_00302	1350	4	24	16	0.354	1.803	1.259	Peptidoglycan-N-acetylglucosamine deacetylase
bin007	SOY3_bin007_00303	1428	5	20	5	0.419	1.421	0.372	putative FAD-linked oxidoreductase
bin007	SOY3_bin007_00304	1218	1	10	1	0.098	0.833	0.087	Anaerobic glycerol-3-phosphate dehydrogenase subunit C
bin007	SOY3_bin007_00305	144	0	4	1	0.000	2.817	0.738	hypothetical protein
bin007	SOY3_bin007_00306	144	0	2	0	0.000	1.409	0.000	hypothetical protein
bin007	SOY3_bin007_00307	132	2	10	2	1.811	7.684	1.609	hypothetical protein
bin007	SOY3_bin007_00308	156	10	39	17	7.663	25.357	11.576	hypothetical protein
bin007	SOY3_bin007_00309	537	1	2	1	0.223	0.378	0.198	hypothetical protein
bin007	SOY3_bin007_00310	1143	1	9	2	0.105	0.799	0.186	putative periplasmic serine endoprotease DegP-like precursor
bin007	SOY3_bin007_00311	150	0	0	0	0.000	0.000	0.000	hypothetical protein
bin007	SOY3_bin007_00312	2178	8	26	13	0.439	1.211	0.634	Penicillin-binding protein 1F
bin007	SOY3_bin007_00313	579	4	19	9	0.826	3.328	1.651	2-amino-4-deoxychorismate dehydrogenase
bin007	SOY3_bin007_00314	198	4	5	2	2.415	2.561	1.073	DNA-binding transcriptional activator PspC
bin007	SOY3_bin007_00315	876	6	35	11	0.819	4.052	1.334	sn-glycerol-3-phosphate dehydrogenase subunit C
bin007	SOY3_bin007_00316	576	5	16	9	1.038	2.817	1.660	succinate dehydrogenase iron-sulfur subunit
bin007	SOY3_bin007_00317	2166	62	110	40	3.422	5.151	1.962	Periplasmic [Fe] hydrogenase large subunit
bin007	SOY3_bin007_00318	1296	42	79	29	3.874	6.183	2.377	ribulose-1,5-biphosphate synthetase
bin007	SOY3_bin007_00319	1848	49	131	37	3.170	7.190	2.127	adenylylsulfate reductase subunit alpha
bin007	SOY3_bin007_00320	444	17	32	7	4.577	7.310	1.675	Pyruvate synthase subunit PorD
bin007	SOY3_bin007_00321	189	3	6	2	1.898	3.220	1.124	hypothetical protein
bin007	SOY3_bin007_00322	366	7	13	6	2.286	3.603	1.741	hypothetical protein
bin007	SOY3_bin007_00323	294	0	7	0	0.000	2.415	0.000	hypothetical protein
bin007	SOY3_bin007_00324	89	1	7	4	1.343	7.977	4.774	tRNA-Leu(taa)
bin007	SOY3_bin007_00325	165	1	14	5	0.725	8.606	3.219	Rubredoxin
bin007	SOY3_bin007_00326	738	11	44	15	1.782	6.047	2.159	Glutamine transport ATP-binding protein GlnQ
bin007	SOY3_bin007_00327	783	13	39	9	1.985	5.052	1.221	Inner membrane amino-acid ABC transporter permease protein YecS
bin007	SOY3_bin007_00328	825	45	135	50	6.521	16.597	6.438	Arginine-binding extracellular protein ArtP precursor
bin007	SOY3_bin007_00329	267	0	0	1	0.000	0.000	0.398	Ser-Thr-rich glycosyl-phosphatidyl-inositol-anchored membrane family protein
bin007	SOY3_bin007_00330	135	0	2	0	0.000	1.503	0.000	hypothetical protein
bin007	SOY3_bin007_00331	294	0	1	2	0.000	0.345	0.723	hypothetical protein
bin007	SOY3_bin007_00332	945	2	11	6	0.253	1.181	0.674	3'-5' exoribonuclease YhaM
bin007	SOY3_bin007_00333	168	14	48	21	9.962	28.979	13.278	hypothetical protein
bin007	SOY3_bin007_00334	1068	3	16	2	0.336	1.520	0.199	hypothetical protein
bin007	SOY3_bin007_00335	2013	44	231	108	2.613	11.639	5.699	K(+)-insensitive pyrophosphate-energized proton pump
bin007	SOY3_bin007_00336	2430	28	91	22	1.378	3.798	0.962	Endo-1,4-beta-xylanase A precursor
bin007	SOY3_bin007_00337	882	29	71	24	3.931	8.165	2.890	hypothetical protein
bin007	SOY3_bin007_00338	519	9	46	14	2.073	8.990	2.865	Primary amine oxidase precursor
bin007	SOY3_bin007_00339	282	4	0	0	1.696	0.000	0.000	(2R)-sulfolactate sulfo-lyase subunit beta
bin007	SOY3_bin007_00340	489	12	2	3	2.934	0.415	0.652	Nicotinate dehydrogenase small FeS subunit
bin007	SOY3_bin007_00341	2484	70	31	14	3.369	1.266	0.599	4-hydroxybenzoyl-CoA reductase subunit alpha
bin007	SOY3_bin007_00342	972	37	13	5	4.551	1.357	0.546	4-hydroxybenzoyl-CoA reductase subunit beta
bin007	SOY3_bin007_00343	795	11	1	0	1.654	0.128	0.000	Acryloyl-CoA reductase electron transfer subunit gamma
bin007	SOY3_bin007_00344	999	7	4	4	0.838	0.406	0.425	Acryloyl-CoA reductase electron transfer subunit beta
bin007	SOY3_bin007_00345	1128	16	9	4	1.696	0.809	0.377	hypothetical protein
bin007	SOY3_bin007_00346	1026	6	1	2	0.699	0.099	0.207	hypothetical protein
bin007	SOY3_bin007_00347	2100	5	9	2	0.285	0.435	0.101	Succinyl-CoA ligase [ADP-forming] subunit alpha
bin007	SOY3_bin007_00348	1056	7	2	2	0.792	0.192	0.201	Phosphate acetyltransferase
bin007	SOY3_bin007_00349	741	6	1	3	0.968	0.137	0.430	Peptidoglycan-N-acetylmuramic acid deacetylase PdaA precursor
bin007	SOY3_bin007_00350	1260	2	15	6	0.190	1.207	0.506	Protease 3 precursor
bin007	SOY3_bin007_00351	465	0	5	2	0.000	1.091	0.457	Deoxyuridine 5'-triphosphate nucleotidohydrolase
bin007	SOY3_bin007_00352	336	0	2	3	0.000	0.604	0.948	hypothetical protein
bin007	SOY3_bin007_00353	270	1	5	1	0.443	1.878	0.393	PRC-barrel domain protein
bin007	SOY3_bin007_00354	3195	14	38	15	0.524	1.206	0.499	Maltooligosyl trehalose synthase
bin007	SOY3_bin007_00355	2439	11	30	5	0.539	1.248	0.218	Alpha-amylase 1
bin007	SOY3_bin007_00356	1863	13	29	3	0.834	1.579	0.171	Malto-oligosyltrehalose trehalohydrolase
bin007	SOY3_bin007_00357	2142	10	32	7	0.558	1.515	0.347	4-alpha-glucanotransferase
bin007	SOY3_bin007_00358	867	1	24	4	0.138	2.808	0.490	hypothetical protein
bin007	SOY3_bin007_00359	1218	46	85	35	4.515	7.078	3.052	Trehalose synthase
bin007	SOY3_bin007_00360	390	7	33	16	2.146	8.582	4.358	hydrogenase nickel incorporation protein HypA
bin007	SOY3_bin007_00361	993	10	29	5	1.204	2.962	0.535	UDP-glucose 4-epimerase

bin007	SOY3_bin007_00362	1140	16	22	6	1.678	1.957	0.559	hypothetical protein
bin007	SOY3_bin007_00363	576	5	9	3	1.038	1.585	0.553	Inner membrane protein YaaH
bin007	SOY3_bin007_00364	957	3	30	9	0.375	3.180	0.999	Nitronate monooxygenase
bin007	SOY3_bin007_00365	306	1	1	1	0.391	0.331	0.347	hypothetical protein
bin007	SOY3_bin007_00366	1704	13	54	21	0.912	3.214	1.309	Long-chain-fatty-acid--CoA ligase
bin007	SOY3_bin007_00367	1029	8	34	1	0.929	3.351	0.103	Magnesium transport protein CorA
bin007	SOY3_bin007_00368	1155	6	37	13	0.621	3.249	1.196	Putative serine protease HtrA
bin007	SOY3_bin007_00369	675	0	7	1	0.000	1.052	0.157	Response regulator MprA
bin007	SOY3_bin007_00370	1419	7	33	11	0.590	2.359	0.823	putative sensor histidine kinase TcrY
bin007	SOY3_bin007_00371	531	3	11	5	0.675	2.101	1.000	hypothetical protein
bin007	SOY3_bin007_00372	105	0	0	0	0.000	0.000	0.000	hypothetical protein
bin007	SOY3_bin007_00373	1221	82	93	31	8.029	7.725	2.697	Leucine-, isoleucine-, valine-, threonine-, and alanine-binding protein precursor
bin007	SOY3_bin007_00374	876	23	30	9	3.139	3.474	1.091	High-affinity branched-chain amino acid transport system permease protein LivH
bin007	SOY3_bin007_00375	954	21	37	7	2.632	3.934	0.779	High-affinity branched-chain amino acid transport system permease protein LivH
bin007	SOY3_bin007_00376	720	21	40	10	3.487	5.635	1.475	Lipopolysaccharide export system ATP-binding protein LptB
bin007	SOY3_bin007_00377	705	26	43	9	4.409	6.186	1.356	High-affinity branched-chain amino acid transport ATP-binding protein LivF
bin007	SOY3_bin007_00378	438	2	6	4	0.546	1.389	0.970	Stress response protein NhaX
bin007	SOY3_bin007_00379	1665	11	29	18	0.790	1.767	1.148	Methyl-accepting chemotaxis protein McpB
bin007	SOY3_bin007_00380	633	8	19	4	1.511	3.044	0.671	leucine export protein LeuE
bin007	SOY3_bin007_00381	1401	16	40	11	1.365	2.896	0.834	2-aminophenol 1,6-dioxygenase subunit beta
bin007	SOY3_bin007_00382	999	7	24	6	0.838	2.437	0.638	4-hydroxyphenylacetate decarboxylase activating enzyme
bin007	SOY3_bin007_00383	867	22	60	27	3.034	7.019	3.308	PBP superfamily domain protein
bin007	SOY3_bin007_00384	654	4	33	17	0.731	5.118	2.761	hypothetical protein
bin007	SOY3_bin007_00385	2553	7	42	29	0.328	1.669	1.207	Maltodextrin phosphorylase
bin007	SOY3_bin007_00386	564	1	5	4	0.212	0.899	0.753	hypothetical protein
bin007	SOY3_bin007_00387	1389	2	12	6	0.172	0.876	0.459	Cyclic di-GMP phosphodiesterase response regulator RpfG
bin007	SOY3_bin007_00388	1629	5	22	8	0.367	1.370	0.522	Oligopeptide-binding protein OppA precursor
bin007	SOY3_bin007_00389	924	5	15	11	0.647	1.647	1.265	Oligopeptide transport system permease protein OppB
bin007	SOY3_bin007_00390	840	0	5	0	0.000	0.604	0.000	Oligopeptide transport system permease protein OppC
bin007	SOY3_bin007_00391	963	2	0	1	0.248	0.000	0.110	Oligopeptide transport ATP-binding protein OppD
bin007	SOY3_bin007_00392	756	0	0	0	0.000	0.000	0.000	Oligopeptide transport ATP-binding protein OppF
bin007	SOY3_bin007_00393	504	1	11	2	0.237	2.214	0.422	hypothetical protein
bin007	SOY3_bin007_00394	975	9	38	11	1.104	3.953	1.198	GTPase Obg
bin007	SOY3_bin007_00395	1239	11	27	21	1.061	2.210	1.800	Beta-monoglucosyldiacylglycerol synthase
bin007	SOY3_bin007_00396	750	0	6	2	0.000	0.811	0.283	Sulfate/thiosulfate import ATP-binding protein CysA
bin007	SOY3_bin007_00397	1194	0	2	0	0.000	0.170	0.000	Leucine-, isoleucine-, valine-, threonine-, and alanine-binding protein precursor
bin007	SOY3_bin007_00398	882	0	2	2	0.000	0.230	0.241	High-affinity branched-chain amino acid transport system permease protein LivH
bin007	SOY3_bin007_00399	1005	2	3	1	0.238	0.303	0.106	High-affinity branched-chain amino acid transport system permease protein LivH
bin007	SOY3_bin007_00400	768	1	4	1	0.156	0.528	0.138	Lipopolysaccharide export system ATP-binding protein LptB
bin007	SOY3_bin007_00401	741	6	29	12	0.968	3.969	1.720	High-affinity branched-chain amino acid transport ATP-binding protein LivF
bin007	SOY3_bin007_00402	1335	24	82	24	2.149	6.230	1.910	Phenylacetate-coenzyme A ligase
bin007	SOY3_bin007_00403	1062	10	41	16	1.126	3.916	1.600	Peptidoglycan-N-acetylmuramic acid deacetylase PdaA precursor
bin007	SOY3_bin007_00404	1443	1	14	1	0.083	0.984	0.074	Sodium/glucose cotransporter
bin007	SOY3_bin007_00405	1044	13	46	13	1.489	4.469	1.323	N-acetyl-gamma-glutamyl-phosphate reductase
bin007	SOY3_bin007_00406	1236	18	63	16	1.741	5.170	1.375	Arginine biosynthesis bifunctional protein ArgJ
bin007	SOY3_bin007_00407	933	10	52	23	1.281	5.653	2.619	Acetylglutamate kinase
bin007	SOY3_bin007_00408	1209	20	85	30	1.978	7.131	2.636	Acetylornithine aminotransferase
bin007	SOY3_bin007_00409	942	8	41	16	1.015	4.415	1.804	Ornithine carbamoyltransferase
bin007	SOY3_bin007_00410	1206	18	75	22	1.784	6.308	1.938	Argininosuccinate synthase
bin007	SOY3_bin007_00411	429	4	14	3	1.115	3.310	0.743	Organic hydroperoxide resistance transcriptional regulator
bin007	SOY3_bin007_00412	240	0	1	0	0.000	0.423	0.000	hypothetical protein
bin007	SOY3_bin007_00413	2196	7	23	6	0.381	1.062	0.290	ATP-dependent DNA helicase RecQ
bin007	SOY3_bin007_00414	183	5	19	5	3.266	10.531	2.902	hypothetical protein
bin007	SOY3_bin007_00415	126	3	7	4	2.846	5.635	3.372	hypothetical protein
bin007	SOY3_bin007_00416	1380	70	157	69	6.064	11.539	5.311	Argininosuccinate lyase 1
bin007	SOY3_bin007_00417	438	0	0	0	0.000	0.000	0.000	hypothetical protein
bin007	SOY3_bin007_00418	1032	0	0	1	0.000	0.000	0.103	Spore germination protein B3 precursor
bin007	SOY3_bin007_00419	237	1	0	0	0.504	0.000	0.000	hypothetical protein
bin007	SOY3_bin007_00420	258	5	18	6	2.317	7.076	2.470	hypothetical protein
bin007	SOY3_bin007_00421	579	11	42	13	2.271	7.357	2.385	Septum formation protein Maf
bin007	SOY3_bin007_00422	738	22	68	26	3.564	9.346	3.742	hypothetical protein
bin007	SOY3_bin007_00423	1032	15	46	21	1.738	4.521	2.162	Rod shape-determining protein MreB
bin007	SOY3_bin007_00424	858	16	41	19	2.229	4.847	2.352	Cell shape-determining protein MreC precursor
bin007	SOY3_bin007_00425	492	1	7	8	0.243	1.443	1.727	rod shape-determining protein MreD
bin007	SOY3_bin007_00426	1959	8	46	12	0.488	2.382	0.651	Stage V sporulation protein D
bin007	SOY3_bin007_00427	444	5	18	8	1.346	4.112	1.914	Septum site-determining protein MinC
bin007	SOY3_bin007_00428	795	6	21	15	0.902	2.679	2.004	Septum site-determining protein MinD

bin007	SOY3_bin007_00429	267	4	15	7	1.791	5.698	2.785	Cell division topological specificity factor
bin007	SOY3_bin007_00430	1182	1	7	1	0.101	0.601	0.090	Rod shape-determining protein RodA
bin007	SOY3_bin007_00431	738	5	1	1	0.810	0.137	0.144	Murein hydrolase activator NlpD precursor
bin007	SOY3_bin007_00432	891	3	3	2	0.403	0.342	0.238	Putative zinc metalloprotease Rip3
bin007	SOY3_bin007_00433	1863	27	73	23	1.733	3.974	1.311	Threonylcarbamoyladenosine tRNA methylthiotransferase MtaB
bin007	SOY3_bin007_00434	711	7	27	15	1.177	3.852	2.241	hypothetical protein
bin007	SOY3_bin007_00435	1695	11	56	26	0.776	3.351	1.629	Ribonuclease G
bin007	SOY3_bin007_00436	312	15	40	18	5.748	13.003	6.128	50S ribosomal protein L21
bin007	SOY3_bin007_00437	270	21	84	41	9.298	31.555	16.131	50S ribosomal protein L27
bin007	SOY3_bin007_00438	165	1	2	0	0.725	1.229	0.000	hypothetical protein
bin007	SOY3_bin007_00439	567	2	9	3	0.422	1.610	0.562	Sensor histidine kinase DcuS
bin007	SOY3_bin007_00440	1269	6	23	8	0.565	1.838	0.670	GTPase ObgE
bin007	SOY3_bin007_00441	1128	3	22	5	0.318	1.978	0.471	Glutamate 5-kinase
bin007	SOY3_bin007_00442	615	4	37	20	0.778	6.102	3.454	Nicotinate-nucleotide adenylyltransferase
bin007	SOY3_bin007_00443	258	40	196	110	18.535	77.053	45.290	RNA recognition motif. (a.k.a. RRM, RBD, or RNP domain)
bin007	SOY3_bin007_00444	357	14	37	13	4.688	10.512	3.868	Ribosomal silencing factor RsfS
bin007	SOY3_bin007_00445	2484	34	119	43	1.636	4.859	1.839	Leucine--tRNA ligase
bin007	SOY3_bin007_00446	1797	23	50	22	1.530	2.822	1.300	Transcriptional regulatory protein ZraR
bin007	SOY3_bin007_00447	546	34	70	27	7.444	13.003	5.253	NADH-dependent phenylglyoxylate dehydrogenase subunit gamma
bin007	SOY3_bin007_00448	807	33	77	34	4.889	9.678	4.475	2-oxoglutarate oxidoreductase subunit KorB
bin007	SOY3_bin007_00449	1140	59	131	50	6.187	11.655	4.659	2-oxoglutarate oxidoreductase subunit KorA
bin007	SOY3_bin007_00450	225	62	109	49	32.942	49.136	23.134	Ferredoxin-2
bin007	SOY3_bin007_00451	669	0	1	0	0.000	0.152	0.000	hypothetical protein
bin007	SOY3_bin007_00452	660	1	12	2	0.181	1.844	0.322	ComE operon protein 1
bin007	SOY3_bin007_00453	201	196	724	245	116.575	365.340	129.479	hypothetical protein
bin007	SOY3_bin007_00454	798	4	13	1	0.599	1.652	0.133	S-adenosyl-L-methionine-binding protein
bin007	SOY3_bin007_00455	792	20	58	16	3.019	7.428	2.146	formate dehydrogenase accessory protein
bin007	SOY3_bin007_00456	477	22	38	16	5.514	8.080	3.563	hypothetical protein
bin007	SOY3_bin007_00457	1200	56	143	53	5.579	12.087	4.692	Molybdopterin molybdenumtransferase
bin007	SOY3_bin007_00458	1026	139	407	135	16.196	40.235	13.977	Molybdopterin molybdenumtransferase
bin007	SOY3_bin007_00459	1572	262	722	227	19.925	46.584	15.339	Formate dehydrogenase H
bin007	SOY3_bin007_00460	1059	182	391	153	20.546	37.449	15.347	Putative formate dehydrogenase
bin007	SOY3_bin007_00461	2439	1	11	7	0.049	0.457	0.305	ComEC family competence protein
bin007	SOY3_bin007_00462	1044	4	6	4	0.458	0.583	0.407	Glutaconyl-CoA decarboxylase subunit beta
bin007	SOY3_bin007_00463	447	2	3	5	0.535	0.681	1.188	Glutaconyl-CoA decarboxylase subunit gamma
bin007	SOY3_bin007_00464	366	0	3	3	0.000	0.831	0.871	oxaloacetate decarboxylase subunit gamma
bin007	SOY3_bin007_00465	1554	7	77	23	0.539	5.026	1.572	Methylmalonyl-CoA carboxyltransferase 12S subunit
bin007	SOY3_bin007_00466	267	1	1	1	0.448	0.380	0.398	hypothetical protein
bin007	SOY3_bin007_00467	1926	14	50	28	0.869	2.633	1.544	putative cadmium-transporting ATPase
bin007	SOY3_bin007_00468	438	3	2	3	0.819	0.463	0.728	HTH-type transcriptional regulator MhqR
bin007	SOY3_bin007_00469	339	0	1	1	0.000	0.299	0.313	Copper-sensing transcriptional repressor CsoR
bin007	SOY3_bin007_00470	2433	12	43	20	0.590	1.793	0.873	Copper-exporting P-type ATPase A
bin007	SOY3_bin007_00471	201	2	12	6	1.190	6.055	3.171	Mercuric transport protein periplasmic component precursor
bin007	SOY3_bin007_00472	1410	8	37	15	0.678	2.662	1.130	Multidrug export protein MepA
bin007	SOY3_bin007_00473	456	1	1	1	0.262	0.222	0.233	Multiple antibiotic resistance protein MarR
bin007	SOY3_bin007_00474	120	4	11	3	3.985	9.298	2.656	hypothetical protein
bin007	SOY3_bin007_00475	771	1	8	1	0.155	1.052	0.138	Prolipoprotein diacylglyceryl transferase
bin007	SOY3_bin007_00476	687	0	3	1	0.000	0.443	0.155	Protein-L-isoaspartate O-methyltransferase
bin007	SOY3_bin007_00477	600	0	0	1	0.000	0.000	0.177	hypothetical protein
bin007	SOY3_bin007_00478	585	0	1	0	0.000	0.173	0.000	hypothetical protein
bin007	SOY3_bin007_00479	3552	5	17	7	0.168	0.485	0.209	hypothetical protein
bin007	SOY3_bin007_00480	3678	3	20	8	0.098	0.552	0.231	N5-glutamine S-adenosyl-L-methionine-dependent methyltransferase
bin007	SOY3_bin007_00481	210	0	0	1	0.000	0.000	0.506	hypothetical protein
bin007	SOY3_bin007_00482	2559	4	19	5	0.187	0.753	0.208	PglZ domain protein
bin007	SOY3_bin007_00483	1716	0	5	3	0.000	0.296	0.186	Divergent AAA domain protein
bin007	SOY3_bin007_00484	2031	2	13	8	0.118	0.649	0.418	DNA-binding ATP-dependent protease La
bin007	SOY3_bin007_00485	273	1	0	1	0.438	0.000	0.389	Putative ribosome biogenesis GTPase RsgA
bin007	SOY3_bin007_00486	327	0	4	0	0.000	1.241	0.000	Nucleotidyltransferase domain protein
bin007	SOY3_bin007_00487	189	0	3	0	0.000	1.610	0.000	hypothetical protein
bin007	SOY3_bin007_00488	294	0	4	0	0.000	1.380	0.000	hypothetical protein
bin007	SOY3_bin007_00489	237	0	0	0	0.000	0.000	0.000	hypothetical protein
bin007	SOY3_bin007_00490	597	0	10	4	0.000	1.699	0.712	Sensor histidine kinase YycG
bin007	SOY3_bin007_00491	699	104	492	168	17.787	71.391	25.531	Sensory transduction protein regX3
bin007	SOY3_bin007_00492	489	0	2	0	0.000	0.415	0.000	hypothetical protein
bin007	SOY3_bin007_00493	1365	2	10	1	0.175	0.743	0.078	hypothetical protein
bin007	SOY3_bin007_00494	828	2	27	4	0.289	3.307	0.513	Chondramide synthase cmdD
bin007	SOY3_bin007_00495	333	2	16	0	0.718	4.873	0.000	hypothetical protein

bin007	SOY3_bin007_00496	315	1	24	2	0.380	7.728	0.674	Nucleotidyltransferase domain protein
bin007	SOY3_bin007_00497	333	1	1	0	0.359	0.305	0.000	hypothetical protein
bin007	SOY3_bin007_00498	210	1	1	0	0.569	0.483	0.000	hypothetical protein
bin007	SOY3_bin007_00499	525	23	40	23	5.237	7.728	4.654	Bacterioferritin
bin007	SOY3_bin007_00500	1644	9	18	7	0.654	1.111	0.452	Long-chain-fatty-acid--CoA ligase FadD13
bin007	SOY3_bin007_00501	876	4	14	4	0.546	1.621	0.485	putative metallo-hydrolase YflN
bin007	SOY3_bin007_00502	1752	9	46	10	0.614	2.663	0.606	Glutamine--tRNA ligase
bin007	SOY3_bin007_00503	2043	12	69	14	0.702	3.426	0.728	putative ATP-dependent helicase DinG
bin007	SOY3_bin007_00504	663	11	16	3	1.983	2.448	0.481	Inner membrane protein YabI
bin007	SOY3_bin007_00505	306	1	2	0	0.391	0.663	0.000	NADH dehydrogenase subunit E
bin007	SOY3_bin007_00506	1707	2	22	12	0.140	1.307	0.747	Iron hydrogenase 1
bin007	SOY3_bin007_00507	1158	1	8	10	0.103	0.701	0.917	Stage II sporulation protein E (SpoIIE)
bin007	SOY3_bin007_00508	627	1	11	4	0.191	1.779	0.678	Inosine-5'-monophosphate dehydrogenase
bin007	SOY3_bin007_00509	1221	10	13	1	0.979	1.080	0.087	hypothetical protein
bin007	SOY3_bin007_00510	90	3	5	2	3.985	5.635	2.361	tRNA-Ser(gga)
bin007	SOY3_bin007_00511	1893	29	98	33	1.831	5.251	1.852	DNA polymerase III subunit tau
bin007	SOY3_bin007_00512	315	1	18	8	0.380	5.796	2.698	Nucleoid-associated protein
bin007	SOY3_bin007_00513	603	3	33	12	0.595	5.551	2.114	Recombination protein RecR
bin007	SOY3_bin007_00514	279	5	8	3	2.142	2.908	1.142	Sigma-K factor-processing regulatory protein BofA
bin007	SOY3_bin007_00515	666	8	6	1	1.436	0.914	0.159	hypothetical protein
bin007	SOY3_bin007_00516	1089	5	13	5	0.549	1.211	0.488	2-oxoglutarate oxidoreductase subunit KorA
bin007	SOY3_bin007_00517	765	3	10	4	0.469	1.326	0.555	2-oxoglutarate oxidoreductase subunit KorB
bin007	SOY3_bin007_00518	582	5	19	3	1.027	3.311	0.548	NADH-dependent phenylglyoxylate dehydrogenase subunit gamma
bin007	SOY3_bin007_00519	252	2	6	4	0.949	2.415	1.686	Ferredoxin
bin007	SOY3_bin007_00520	192	3	19	5	1.868	10.037	2.766	Inhibitor of sigma-G Gin
bin007	SOY3_bin007_00521	270	1	3	1	0.443	1.127	0.393	hypothetical protein
bin007	SOY3_bin007_00522	1446	19	57	19	1.571	3.998	1.396	Arginine decarboxylase
bin007	SOY3_bin007_00523	651	15	59	16	2.755	9.192	2.611	Thymidylate kinase
bin007	SOY3_bin007_00524	996	24	105	38	2.881	10.693	4.053	DNA polymerase III subunit tau
bin007	SOY3_bin007_00525	870	9	29	15	1.237	3.381	1.831	hypothetical protein
bin007	SOY3_bin007_00526	357	1	12	9	0.335	3.409	2.678	Initiation-control protein YabA
bin007	SOY3_bin007_00527	915	10	36	22	1.307	3.991	2.554	Ribosomal RNA small subunit methyltransferase I
bin007	SOY3_bin007_00528	255	126	423	132	59.071	168.250	54.987	Transition state regulatory protein AbrB
bin007	SOY3_bin007_00529	1545	16	52	17	1.238	3.414	1.169	Methionine--tRNA ligase
bin007	SOY3_bin007_00530	771	6	14	4	0.930	1.842	0.551	putative deoxyribonuclease YcfH
bin007	SOY3_bin007_00531	540	5	9	2	1.107	1.690	0.393	Cob(I)yrinic acid a,c-diamide adenosyltransferase
bin007	SOY3_bin007_00532	459	4	19	9	1.042	4.199	2.083	hypothetical protein
bin007	SOY3_bin007_00533	1020	114	203	75	13.361	20.186	7.811	Cell wall-binding protein YocH precursor
bin007	SOY3_bin007_00534	867	5	15	4	0.689	1.755	0.490	Quercetin 2,3-dioxygenase
bin007	SOY3_bin007_00535	888	13	38	21	1.750	4.340	2.512	Ribosomal RNA small subunit methyltransferase A
bin007	SOY3_bin007_00536	471	3	16	6	0.761	3.446	1.353	hypothetical protein
bin007	SOY3_bin007_00537	792	2	8	3	0.302	1.025	0.402	4,4'-diaponeurosporenoate glycosyltransferase
bin007	SOY3_bin007_00538	915	1	3	1	0.131	0.333	0.116	Sporulation-specific protease YabG
bin007	SOY3_bin007_00539	1005	0	18	7	0.000	1.817	0.740	hypothetical protein
bin007	SOY3_bin007_00540	417	0	5	2	0.000	1.216	0.509	hypothetical protein
bin007	SOY3_bin007_00541	393	3	2	3	0.913	0.516	0.811	flagellar assembly protein H
bin007	SOY3_bin007_00542	1011	1	1	0	0.118	0.100	0.000	adhesin
bin007	SOY3_bin007_00543	1551	84	276	96	6.475	18.049	6.575	putative cell wall hydrolase LytN precursor
bin007	SOY3_bin007_00544	1065	94	226	95	10.552	21.524	9.476	hypothetical protein
bin007	SOY3_bin007_00545	303	1	9	0	0.395	3.013	0.000	Nucleoid-associated protein
bin007	SOY3_bin007_00546	612	6	14	12	1.172	2.320	2.083	hypothetical protein
bin007	SOY3_bin007_00547	315	1	4	2	0.380	1.288	0.674	hypothetical protein
bin007	SOY3_bin007_00548	207	1	6	3	0.578	2.940	1.540	hypothetical protein
bin007	SOY3_bin007_00549	225	0	2	0	0.000	0.902	0.000	hypothetical protein
bin007	SOY3_bin007_00550	447	2	7	3	0.535	1.588	0.713	Photosystem II reaction center protein H
bin007	SOY3_bin007_00551	720	11	14	12	1.826	1.972	1.770	Zinc transporter ZupT
bin007	SOY3_bin007_00552	585	3	11	2	0.613	1.907	0.363	Putative L,D-transpeptidase YkuD
bin007	SOY3_bin007_00553	465	3	2	0	0.771	0.436	0.000	Cyanophycin synthetase
bin007	SOY3_bin007_00554	903	4	25	7	0.530	2.808	0.823	4-diphosphocytidyl-2-C-methyl-D-erythritol kinase
bin007	SOY3_bin007_00555	678	13	53	17	2.292	7.929	2.663	putative HTH-type transcriptional regulator YdfH
bin007	SOY3_bin007_00556	759	9	59	18	1.418	7.884	2.519	2-phospho-L-lactate guanylyltransferase
bin007	SOY3_bin007_00557	1140	2	12	5	0.210	1.068	0.466	Acetyltransferase (GNAT) family protein
bin007	SOY3_bin007_00558	255	62	125	51	29.067	49.719	21.245	Putative septation protein SpoVG
bin007	SOY3_bin007_00559	1368	31	143	64	2.709	10.602	4.970	Bifunctional protein GlmU
bin007	SOY3_bin007_00560	948	20	72	31	2.522	7.703	3.474	Ribose-phosphate pyrophosphokinase
bin007	SOY3_bin007_00561	753	12	44	15	1.905	5.927	2.116	Mannosylfructose-phosphate phosphatase
bin007	SOY3_bin007_00562	1452	92	360	109	7.575	25.147	7.974	Cellulosome-anchoring protein precursor

bin007	SOY3_bin007_00563	771	17	84	31	2.636	11.050	4.271	Cohesin domain protein
bin007	SOY3_bin007_00564	894	48	126	51	6.419	14.295	6.060	hypothetical protein
bin007	SOY3_bin007_00565	1332	92	302	94	8.257	22.996	7.496	Cellulosome-anchoring protein precursor
bin007	SOY3_bin007_00566	990	91	228	82	10.989	23.359	8.798	Cellulosome-anchoring protein precursor
bin007	SOY3_bin007_00567	2910	2576	9076	3290	105.827	316.342	120.097	hypothetical protein
bin007	SOY3_bin007_00568	1143	31	82	29	3.242	7.276	2.695	Ribosomal RNA large subunit methyltransferase K/L
bin007	SOY3_bin007_00569	1584	108	306	120	8.151	19.594	8.047	L-aspartate oxidase
bin007	SOY3_bin007_00570	897	15	71	20	1.999	8.028	2.368	Quinolinate synthase A
bin007	SOY3_bin007_00571	384	12	37	8	3.736	9.773	2.213	Aspartate 1-decarboxylase precursor
bin007	SOY3_bin007_00572	846	77	219	89	10.881	26.256	11.175	Pantothenate synthetase
bin007	SOY3_bin007_00573	837	27	98	20	3.856	11.876	2.538	3-methyl-2-oxobutanoate hydroxymethyltransferase
bin007	SOY3_bin007_00574	933	32	92	37	4.100	10.001	4.213	prephenate dehydrogenase
bin007	SOY3_bin007_00575	1020	6	25	10	0.703	2.486	1.041	Spore photoproduct lyase
bin007	SOY3_bin007_00576	492	8	13	3	1.944	2.680	0.648	2-amino-4-hydroxy-6-hydroxymethyldihydropteridine pyrophosphokinase
bin007	SOY3_bin007_00577	375	6	29	14	1.913	7.844	3.966	Dihydroneopterin aldolase
bin007	SOY3_bin007_00578	1212	26	77	39	2.565	6.444	3.418	Dihydropteroate synthase
bin007	SOY3_bin007_00579	1761	41	130	39	2.783	7.488	2.353	Formate--tetrahydrofolate ligase
bin007	SOY3_bin007_00580	459	2	15	6	0.521	3.315	1.389	Nucleoside diphosphate kinase
bin007	SOY3_bin007_00581	1830	42	148	52	2.744	8.203	3.018	ATP-dependent zinc metalloprotease FtsH
bin007	SOY3_bin007_00582	1398	12	97	29	1.026	7.038	2.204	tRNA(Ile)-lysidine synthase
bin007	SOY3_bin007_00583	2274	600	2783	867	31.543	124.130	40.500	Alpha-2-macroglobulin MG1 domain protein
bin007	SOY3_bin007_00584	2463	26	81	25	1.262	3.336	1.078	Stage II sporulation protein E
bin007	SOY3_bin007_00585	903	7	16	8	0.927	1.797	0.941	Guanosine-5'-triphosphate,3'-diphosphate pyrophosphatase
bin007	SOY3_bin007_00586	384	10	22	15	3.113	5.811	4.149	General stress protein 13
bin007	SOY3_bin007_00587	423	0	6	6	0.000	1.439	1.507	cell division protein FtsB
bin007	SOY3_bin007_00588	246	0	1	2	0.000	0.412	0.864	RNA polymerase sigma factor SigX
bin007	SOY3_bin007_00589	867	3	6	3	0.414	0.702	0.368	Stage II sporulation protein R (spore_II_R)
bin007	SOY3_bin007_00590	1176	12	31	9	1.220	2.674	0.813	Putative pyridoxal phosphate-dependent aminotransferase EpsN
bin007	SOY3_bin007_00591	1029	3	5	3	0.349	0.493	0.310	hypothetical protein
bin007	SOY3_bin007_00592	519	0	8	0	0.000	1.563	0.000	HTH-type transcriptional regulator MhqR
bin007	SOY3_bin007_00593	324	1	2	1	0.369	0.626	0.328	hypothetical protein
bin007	SOY3_bin007_00594	558	3	10	3	0.643	1.818	0.571	Spore protein YabQ
bin007	SOY3_bin007_00595	279	6	13	4	2.571	4.726	1.523	Spore protein YabP
bin007	SOY3_bin007_00596	1320	14	98	26	1.268	7.530	2.092	Cohesin domain protein
bin007	SOY3_bin007_00597	948	2	18	4	0.252	1.926	0.448	Amidase enhancer precursor
bin007	SOY3_bin007_00598	252	19	78	36	9.014	31.394	15.175	Heat shock protein 15
bin007	SOY3_bin007_00599	276	21	89	34	9.096	32.707	13.086	DNA-binding protein HU
bin007	SOY3_bin007_00600	1698	20	43	17	1.408	2.569	1.064	Nucleoside triphosphate pyrophosphohydrolase
bin007	SOY3_bin007_00601	561	38	97	26	8.098	17.537	4.923	Stage V sporulation protein T
bin007	SOY3_bin007_00602	1002	5	33	17	0.597	3.340	1.802	Putative peptidyl-prolyl cis-trans isomerase Cbf2 precursor
bin007	SOY3_bin007_00603	3585	21	53	15	0.700	1.499	0.444	Transcription-repair-coupling factor
bin007	SOY3_bin007_00604	231	13	41	16	6.728	18.002	7.358	hypothetical protein
bin007	SOY3_bin007_00605	567	17	34	12	3.584	6.082	2.248	Peptidyl-tRNA hydrolase
bin007	SOY3_bin007_00606	630	5	43	10	0.949	6.923	1.686	50S ribosomal protein L25
bin007	SOY3_bin007_00607	801	22	49	14	3.283	6.205	1.857	PRC-barrel domain protein
bin007	SOY3_bin007_00608	564	20	59	29	4.239	10.610	5.462	NADH dehydrogenase
bin007	SOY3_bin007_00609	1278	40	90	38	3.742	7.143	3.159	putative siderophore transport system ATP-binding protein YusV
bin007	SOY3_bin007_00610	1053	20	73	35	2.271	7.032	3.531	Hemin transport system permease protein HmuU
bin007	SOY3_bin007_00611	963	14	26	15	1.738	2.738	1.655	Vitamin B12-binding protein precursor
bin007	SOY3_bin007_00612	534	10	12	4	2.239	2.279	0.796	Adenosylcobinamide amidohydrolase
bin007	SOY3_bin007_00613	354	17	87	34	5.741	24.927	10.202	hypothetical protein
bin007	SOY3_bin007_00614	978	2	4	1	0.244	0.415	0.109	Activator of (R)-2-hydroxyglutaryl-CoA dehydratase
bin007	SOY3_bin007_00615	1095	2	3	0	0.218	0.278	0.000	2-hydroxyglutaryl-CoA dehydratase, D-component
bin007	SOY3_bin007_00616	999	0	5	2	0.000	0.508	0.213	2-hydroxyglutaryl-CoA dehydratase, D-component
bin007	SOY3_bin007_00617	582	3	2	1	0.616	0.349	0.183	hypothetical protein
bin007	SOY3_bin007_00618	195	1	2	2	0.613	1.040	1.089	hypothetical protein
bin007	SOY3_bin007_00619	771	5	11	5	0.775	1.447	0.689	RNA polymerase sigma-F factor
bin007	SOY3_bin007_00620	459	2	14	9	0.521	3.094	2.083	Anti-sigma F factor
bin007	SOY3_bin007_00621	339	2	14	5	0.705	4.189	1.567	Anti-sigma F factor antagonist
bin007	SOY3_bin007_00622	1185	6	7	3	0.605	0.599	0.269	D-alanyl-D-alanine carboxypeptidase DacF precursor
bin007	SOY3_bin007_00623	1302	5	19	2	0.459	1.480	0.163	Pyrimidine-nucleoside phosphorylase
bin007	SOY3_bin007_00624	1200	6	27	4	0.598	2.282	0.354	Phosphopentomutase
bin007	SOY3_bin007_00625	888	8	19	5	1.077	2.170	0.598	Tyrosine recombinase XerD
bin007	SOY3_bin007_00626	630	1	3	0	0.190	0.483	0.000	Stage II sporulation protein M
bin007	SOY3_bin007_00627	132	1	2	0	0.906	1.537	0.000	hypothetical protein
bin007	SOY3_bin007_00628	1137	12	56	25	1.262	4.996	2.336	Carboxypeptidase G2 precursor
bin007	SOY3_bin007_00629	561	3	15	3	0.639	2.712	0.568	Pyruvate synthase subunit PorC

bin007	SOY3_bin007_00630	747	7	22	7	1.120	2.987	0.995	2-oxoglutarate oxidoreductase subunit KorB
bin007	SOY3_bin007_00631	1056	6	44	10	0.679	4.226	1.006	NADH-dependent phenylglyoxylate dehydrogenase subunit alpha
bin007	SOY3_bin007_00632	219	0	7	5	0.000	3.242	2.425	NADH-quinone oxidoreductase subunit I
bin007	SOY3_bin007_00633	693	7	21	6	1.208	3.074	0.920	Flagellum site-determining protein YlxH
bin007	SOY3_bin007_00634	771	11	83	29	1.706	10.919	3.996	Stage 0 sporulation protein A
bin007	SOY3_bin007_00635	1326	1	13	5	0.090	0.994	0.401	SpoIVB peptidase precursor
bin007	SOY3_bin007_00636	1683	0	12	6	0.000	0.723	0.379	DNA repair protein RecN
bin007	SOY3_bin007_00637	462	3	5	5	0.776	1.098	1.150	Arginine repressor
bin007	SOY3_bin007_00638	582	4	8	8	0.822	1.394	1.460	Ubiquinone/menaquinone biosynthesis C-methyltransferase UbiE
bin007	SOY3_bin007_00639	870	9	18	10	1.237	2.098	1.221	putative inorganic polyphosphate/ATP-NAD kinase
bin007	SOY3_bin007_00640	846	6	16	3	0.848	1.918	0.377	16S/23S rRNA (cytidine-2'-O)-methyltransferase TlyA
bin007	SOY3_bin007_00641	1908	27	88	27	1.692	4.678	1.503	1-deoxy-D-xylulose-5-phosphate synthase
bin007	SOY3_bin007_00642	594	7	29	10	1.409	4.952	1.788	hypothetical protein
bin007	SOY3_bin007_00643	885	8	32	12	1.081	3.667	1.440	Farnesyl diphosphate synthase
bin007	SOY3_bin007_00644	270	3	11	3	1.328	4.132	1.180	Exodeoxyribonuclease 7 small subunit
bin007	SOY3_bin007_00645	633	2	12	10	0.378	1.923	1.678	Methenyltetrahydrofolate cyclohydrolase
bin007	SOY3_bin007_00646	861	7	25	8	0.972	2.945	0.987	Bifunctional protein FolD protein
bin007	SOY3_bin007_00647	1203	19	41	12	1.888	3.457	1.060	Exodeoxyribonuclease 7 large subunit
bin007	SOY3_bin007_00648	1410	18	50	11	1.526	3.597	0.829	Glutamate synthase [NADPH] small chain
bin007	SOY3_bin007_00649	864	12	18	4	1.660	2.113	0.492	Dihydroorotate dehydrogenase B (NAD(+)), electron transfer subunit
bin007	SOY3_bin007_00650	420	3	17	7	0.854	4.105	1.770	hypothetical protein
bin007	SOY3_bin007_00651	213	3	3	1	1.684	1.429	0.499	hypothetical protein
bin007	SOY3_bin007_00652	531	8	16	5	1.801	3.056	1.000	hypothetical protein
bin007	SOY3_bin007_00653	399	63	152	49	18.876	38.639	13.045	Alkaline shock protein 23
bin007	SOY3_bin007_00654	1353	20	83	17	1.767	6.222	1.335	Biotin carboxylase
bin007	SOY3_bin007_00655	1971	35	157	43	2.123	8.079	2.317	2-oxoglutarate carboxylase large subunit
bin007	SOY3_bin007_00656	597	2	5	1	0.400	0.849	0.178	Stage III sporulation protein AH
bin007	SOY3_bin007_00657	624	5	4	4	0.958	0.650	0.681	hypothetical protein
bin007	SOY3_bin007_00658	636	3	3	2	0.564	0.478	0.334	Stage III sporulation protein AF (Spore_III_AF)
bin007	SOY3_bin007_00659	1167	4	1	1	0.410	0.087	0.091	Stage III sporulation protein AE precursor
bin007	SOY3_bin007_00660	387	1	0	0	0.309	0.000	0.000	Stage III sporulation protein AC/AD protein family protein
bin007	SOY3_bin007_00661	204	0	2	0	0.000	0.994	0.000	Stage III sporulation protein AC/AD protein family protein
bin007	SOY3_bin007_00662	519	1	1	0	0.230	0.195	0.000	stage III sporulation protein SpoAB
bin007	SOY3_bin007_00663	1014	5	7	1	0.589	0.700	0.105	NTPase
bin007	SOY3_bin007_00664	432	9	4	4	2.491	0.939	0.984	hypothetical protein
bin007	SOY3_bin007_00665	465	6	44	11	1.543	9.597	2.513	DNA gyrase inhibitor
bin007	SOY3_bin007_00666	558	3	17	3	0.643	3.090	0.571	Elongation factor P
bin007	SOY3_bin007_00667	1074	4	31	19	0.445	2.928	1.879	putative peptidase
bin007	SOY3_bin007_00668	453	1	12	8	0.264	2.687	1.876	3-dehydroquinate dehydratase
bin007	SOY3_bin007_00669	1350	8	33	20	0.708	2.479	1.574	peptidase PmbA
bin007	SOY3_bin007_00670	246	0	1	0	0.000	0.412	0.000	Sporulation-specific protease YabG
bin007	SOY3_bin007_00671	1419	12	47	14	1.011	3.359	1.048	protease TldD
bin007	SOY3_bin007_00672	876	3	17	4	0.409	1.968	0.485	General secretion pathway, M protein
bin007	SOY3_bin007_00673	645	0	4	1	0.000	0.629	0.165	Fimbrial assembly protein (PilN)
bin007	SOY3_bin007_00674	1104	2	7	3	0.217	0.643	0.289	Cell division protein FtsA
bin007	SOY3_bin007_00675	1305	10	19	5	0.916	1.477	0.407	Polymer-forming cytoskeletal
bin007	SOY3_bin007_00676	522	4	6	1	0.916	1.166	0.203	hypothetical protein
bin007	SOY3_bin007_00677	789	3	9	4	0.455	1.157	0.539	hypothetical protein
bin007	SOY3_bin007_00678	603	1	10	3	0.198	1.682	0.528	hypothetical protein
bin007	SOY3_bin007_00679	417	84	361	132	24.082	87.806	33.625	Type II secretion system protein G precursor
bin007	SOY3_bin007_00680	309	4	10	0	1.548	3.282	0.000	Long-chain-fatty-acid--CoA ligase
bin007	SOY3_bin007_00681	417	4	12	3	1.147	2.919	0.764	hypothetical protein
bin007	SOY3_bin007_00682	450	16	44	9	4.251	9.917	2.125	18 kDa heat shock protein
bin007	SOY3_bin007_00683	2604	16	17	6	0.735	0.662	0.245	Chaperone protein ClpB 1
bin007	SOY3_bin007_00684	1263	1	12	2	0.095	0.964	0.168	GTPase HflX
bin007	SOY3_bin007_00685	759	1	4	3	0.158	0.535	0.420	Calcineurin-like phosphoesterase
bin007	SOY3_bin007_00686	630	0	0	0	0.000	0.000	0.000	Pyrophosphatase PpaX
bin007	SOY3_bin007_00687	354	0	2	0	0.000	0.573	0.000	hypothetical protein
bin007	SOY3_bin007_00688	519	3	5	4	0.691	0.977	0.819	Putative redox-active protein (C_GCAxxG_C_C)
bin007	SOY3_bin007_00689	483	30	63	24	7.425	13.230	5.278	Coenzyme A disulfide reductase
bin007	SOY3_bin007_00690	750	2	7	2	0.319	0.947	0.283	Epoxyqueuosine reductase
bin007	SOY3_bin007_00691	918	3	11	1	0.391	1.215	0.116	O-acetylserine/cysteine export protein
bin007	SOY3_bin007_00692	516	1	23	2	0.232	4.521	0.412	Leucine-specific-binding protein precursor
bin007	SOY3_bin007_00693	633	4	19	6	0.755	3.044	1.007	hypothetical protein
bin007	SOY3_bin007_00694	876	0	1	0	0.000	0.116	0.000	High-affinity branched-chain amino acid transport system permease protein LivH
bin007	SOY3_bin007_00695	891	0	2	0	0.000	0.228	0.000	High-affinity branched-chain amino acid transport system permease protein LivH
bin007	SOY3_bin007_00696	768	1	3	2	0.156	0.396	0.277	Lipopolysaccharide export system ATP-binding protein LptB

bin007	SOY3_bin007_00697	708	3	1	6	0.507	0.143	0.900	High-affinity branched-chain amino acid transport ATP-binding protein LivF
bin007	SOY3_bin007_00698	1725	12	55	23	0.832	3.234	1.416	putative oxidoreductase YdhV
bin007	SOY3_bin007_00699	312	0	3	0	0.000	0.975	0.000	hypothetical protein
bin007	SOY3_bin007_00700	1293	168	502	176	15.533	39.379	14.459	putative MFS-type transporter YhjX
bin007	SOY3_bin007_00701	807	135	230	88	19.999	28.907	11.583	Glutaconate CoA-transferase subunit B
bin007	SOY3_bin007_00702	1017	144	334	121	16.927	33.310	12.638	3-oxoadipate CoA-transferase subunit A
bin007	SOY3_bin007_00703	1062	91	398	79	10.244	38.011	7.902	3-oxoadipate CoA-transferase subunit A
bin007	SOY3_bin007_00704	786	80	229	62	12.168	29.551	8.379	3-oxoadipate CoA-transferase subunit B
bin007	SOY3_bin007_00705	672	3	9	2	0.534	1.358	0.316	Response regulator protein VraR
bin007	SOY3_bin007_00706	1308	6	21	10	0.548	1.628	0.812	Sensor protein ZraS
bin007	SOY3_bin007_00707	516	2	17	7	0.463	3.342	1.441	2,3,4,5-tetrahydropyridine-2,6-dicarboxylate N-acetyltransferase
bin007	SOY3_bin007_00708	1008	9	97	35	1.067	9.760	3.688	Low-affinity inorganic phosphate transporter 1
bin007	SOY3_bin007_00709	615	3	88	27	0.583	14.513	4.664	Putative pit accessory protein
bin007	SOY3_bin007_00710	264	1	2	1	0.453	0.768	0.402	hypothetical protein
bin007	SOY3_bin007_00711	453	3	4	2	0.792	0.896	0.469	putative Mg(2+) transport ATPase
bin007	SOY3_bin007_00712	183	356	753	296	232.565	417.348	171.819	Small, acid-soluble spore proteins, alpha/beta type
bin007	SOY3_bin007_00713	867	1	5	0	0.138	0.585	0.000	hypothetical protein
bin007	SOY3_bin007_00714	1995	3	25	7	0.180	1.271	0.373	Glucoamylase precursor
bin007	SOY3_bin007_00715	321	3	10	6	1.117	3.160	1.986	T-protein
bin007	SOY3_bin007_00716	1041	5	42	14	0.574	4.092	1.429	hypothetical protein
bin007	SOY3_bin007_00717	306	4	13	9	1.563	4.309	3.124	hypothetical protein
bin007	SOY3_bin007_00718	567	14	19	3	2.952	3.399	0.562	Spore coat protein F precursor
bin007	SOY3_bin007_00719	270	7	8	2	3.099	3.005	0.787	hypothetical protein
bin007	SOY3_bin007_00720	300	2	6	0	0.797	2.029	0.000	galactokinase
bin007	SOY3_bin007_00721	195	0	2	0	0.000	1.040	0.000	hypothetical protein
bin007	SOY3_bin007_00722	2358	10	21	12	0.507	0.903	0.541	Bacterial dynamin-like protein
bin007	SOY3_bin007_00723	867	5	13	2	0.689	1.521	0.245	Helix-turn-helix domain protein
bin007	SOY3_bin007_00724	972	3	14	5	0.369	1.461	0.546	NAD-dependent protein deacetylase
bin007	SOY3_bin007_00725	1542	19	103	17	1.473	6.775	1.171	ski2-like helicase
bin007	SOY3_bin007_00726	2109	39	120	55	2.211	5.771	2.770	ski2-like helicase
bin007	SOY3_bin007_00727	114	2	8	1	2.097	7.118	0.932	hypothetical protein
bin007	SOY3_bin007_00728	114	2	7	0	2.097	6.228	0.000	hypothetical protein
bin007	SOY3_bin007_00729	1242	1	10	1	0.096	0.817	0.086	Dehydrosqualene desaturase
bin007	SOY3_bin007_00730	165	2	10	4	1.449	6.147	2.575	hypothetical protein
bin007	SOY3_bin007_00731	489	10	131	42	2.445	27.172	9.124	NADP-reducing hydrogenase subunit HndA
bin007	SOY3_bin007_00732	1698	36	459	143	2.535	27.418	8.946	NADP-reducing hydrogenase subunit HndC
bin007	SOY3_bin007_00733	1731	62	614	184	4.282	35.977	11.291	NADP-reducing hydrogenase subunit HndC
bin007	SOY3_bin007_00734	333	0	0	4	0.000	0.000	1.276	Phosphoenolpyruvate carboxykinase [ATP]
bin007	SOY3_bin007_00735	1113	5	9	3	0.537	0.820	0.286	Cysteine synthase
bin007	SOY3_bin007_00736	693	1	17	8	0.173	2.488	1.226	Pyrimidine 5'-nucleotidase YjjG
bin007	SOY3_bin007_00737	1161	3	19	8	0.309	1.660	0.732	General stress protein 69
bin007	SOY3_bin007_00738	651	2	8	1	0.367	1.246	0.163	Haemolysin-III related
bin007	SOY3_bin007_00739	825	7	21	11	1.014	2.582	1.416	DNA replication protein DnaD
bin007	SOY3_bin007_00740	744	11	16	9	1.768	2.181	1.285	Primosomal protein DnaI
bin007	SOY3_bin007_00741	696	18	22	11	3.092	3.206	1.679	Spore cortex-lytic enzyme precursor
bin007	SOY3_bin007_00742	840	9	25	11	1.281	3.019	1.391	hypothetical protein
bin007	SOY3_bin007_00743	2037	13	18	6	0.763	0.896	0.313	Penicillin-binding protein 2D
bin007	SOY3_bin007_00744	258	1	7	2	0.463	2.752	0.823	hypothetical protein
bin007	SOY3_bin007_00745	546	5	17	4	1.095	3.158	0.778	Galactoside O-acetyltransferase
bin007	SOY3_bin007_00746	77	0	1	1	0.000	1.317	1.380	tRNA-Pro(cgg)
bin007	SOY3_bin007_00747	2310	13	32	20	0.673	1.405	0.920	Extracellular serine protease precursor
bin007	SOY3_bin007_00748	915	4	2	0	0.523	0.222	0.000	Isoaspartyl peptidase precursor
bin007	SOY3_bin007_00749	645	3	32	10	0.556	5.032	1.647	Adenylate kinase
bin007	SOY3_bin007_00750	924	0	6	1	0.000	0.659	0.115	Thioredoxin reductase
bin007	SOY3_bin007_00751	972	33	143	28	4.059	14.922	3.060	putative GTPase/MT1543
bin007	SOY3_bin007_00752	681	14	21	9	2.458	3.128	1.404	putative L-ascorbate-6-phosphate lactonase UlaG
bin007	SOY3_bin007_00753	300	0	5	8	0.000	1.690	2.833	hypothetical protein
bin007	SOY3_bin007_00754	2841	29	146	53	1.220	5.212	1.982	hypothetical protein
bin007	SOY3_bin007_00755	582	40	155	62	8.216	27.012	11.316	sn-glycerol-3-phosphate dehydrogenase subunit C
bin007	SOY3_bin007_00756	864	52	244	84	7.195	28.644	10.328	succinate dehydrogenase/fumarate reductase iron-sulfur subunit
bin007	SOY3_bin007_00757	585	32	163	60	6.539	28.261	10.895	Dehydrosqualene desaturase
bin007	SOY3_bin007_00758	1383	78	390	119	6.742	28.602	9.140	ferredoxin
bin007	SOY3_bin007_00759	213	12	54	20	6.735	25.714	9.974	Methyl-viologen-reducing hydrogenase, delta subunit
bin007	SOY3_bin007_00760	963	66	274	82	8.193	28.859	9.045	electron transport complex protein RnfC
bin007	SOY3_bin007_00761	1041	84	337	96	9.647	32.835	9.796	Anaerobic sulfite reductase subunit A
bin007	SOY3_bin007_00762	843	69	303	93	9.785	36.456	11.719	Anaerobic sulfite reductase subunit B
bin007	SOY3_bin007_00763	393	10	13	2	3.042	3.355	0.541	hypothetical protein

bin007	SOY3_bin007_00764	174	0	7	0	0.000	4.080	0.000	hypothetical protein
bin007	SOY3_bin007_00765	597	1	11	3	0.200	1.869	0.534	GTP cyclohydrolase 1
bin007	SOY3_bin007_00766	174	1	5	2	0.687	2.915	1.221	hypothetical protein
bin007	SOY3_bin007_00767	765	6	45	19	0.938	5.966	2.638	5'-nucleotidase SurE
bin007	SOY3_bin007_00768	1527	22	61	18	1.722	4.052	1.252	Stage V sporulation protein B
bin007	SOY3_bin007_00769	2016	33	153	60	1.957	7.698	3.161	Elongation factor G
bin007	SOY3_bin007_00770	198	9	25	9	5.434	12.806	4.828	hypothetical protein
bin007	SOY3_bin007_00771	76	0	0	0	0.000	0.000	0.000	tRNA-Trp(cca)
bin007	SOY3_bin007_00772	939	3	15	8	0.382	1.620	0.905	D-alanine--D-alanine ligase B
bin007	SOY3_bin007_00773	714	6	20	8	1.005	2.841	1.190	Deoxyribose-phosphate aldolase
bin007	SOY3_bin007_00774	135	0	0	0	0.000	0.000	0.000	hypothetical protein
bin007	SOY3_bin007_00775	192	0	20	5	0.000	10.565	2.766	hypothetical protein
bin007	SOY3_bin007_00776	2748	35	146	40	1.523	5.389	1.546	hypothetical protein
bin007	SOY3_bin007_00777	1101	6	24	7	0.651	2.211	0.675	6-phosphofructokinase 1
bin007	SOY3_bin007_00778	459	1	12	6	0.260	2.652	1.389	hypothetical protein
bin007	SOY3_bin007_00779	1164	25	63	19	2.568	5.490	1.734	Alpha-monoglucosyldiacylglycerol synthase
bin007	SOY3_bin007_00780	585	0	5	2	0.000	0.867	0.363	NADP-reducing hydrogenase subunit HndA
bin007	SOY3_bin007_00781	1956	2	5	1	0.122	0.259	0.054	NADP-reducing hydrogenase subunit HndC
bin007	SOY3_bin007_00782	1896	3	17	9	0.189	0.909	0.504	NADP-reducing hydrogenase subunit HndC
bin007	SOY3_bin007_00783	1458	8	22	3	0.656	1.530	0.219	Long-chain-fatty-acid--CoA ligase
bin007	SOY3_bin007_00784	585	37	163	39	7.561	28.261	7.082	Tryptophan synthase beta chain
bin007	SOY3_bin007_00785	2640	14	52	17	0.634	1.998	0.684	CCA-adding enzyme
bin007	SOY3_bin007_00786	615	1	1	2	0.194	0.165	0.345	Peptidase family M50
bin007	SOY3_bin007_00787	975	2	9	2	0.245	0.936	0.218	Tryptophan--tRNA ligase
bin007	SOY3_bin007_00788	681	0	1	0	0.000	0.149	0.000	hypothetical protein
bin007	SOY3_bin007_00789	462	7	24	6	1.811	5.269	1.380	putative spore protein YtfJ
bin007	SOY3_bin007_00790	843	1	3	0	0.142	0.361	0.000	putative DNA repair protein YkoV
bin007	SOY3_bin007_00791	978	1	3	0	0.122	0.311	0.000	Putative DNA ligase-like protein/MT0965
bin007	SOY3_bin007_00792	900	1	2	0	0.133	0.225	0.000	putative ATP-dependent DNA ligase YkoU
bin007	SOY3_bin007_00793	882	1	5	0	0.136	0.575	0.000	Pseudouridine-5'-phosphate glycosidase
bin007	SOY3_bin007_00794	1065	5	14	7	0.561	1.333	0.698	hypothetical protein
bin007	SOY3_bin007_00795	2118	9	35	7	0.508	1.676	0.351	DNA topoisomerase 1
bin007	SOY3_bin007_00796	1362	19	36	17	1.668	2.681	1.326	Methylenetetrahydrofolate--tRNA-(uracil-5-)-methyltransferase TrmFO
bin007	SOY3_bin007_00797	975	1	14	7	0.123	1.456	0.763	Tyrosine recombinase XerC
bin007	SOY3_bin007_00798	531	2	4	1	0.450	0.764	0.200	ATP-dependent protease subunit ClpQ
bin007	SOY3_bin007_00799	1395	3	30	8	0.257	2.181	0.609	ATP-dependent protease ATPase subunit ClpY
bin007	SOY3_bin007_00800	783	35	263	39	5.344	34.068	5.291	GTP-sensing transcriptional pleiotropic repressor CodY
bin007	SOY3_bin007_00801	699	56	227	111	9.578	32.938	16.868	30S ribosomal protein S2
bin007	SOY3_bin007_00802	660	6	21	12	1.087	3.227	1.931	Elongation factor Ts
bin007	SOY3_bin007_00803	744	10	81	31	1.607	11.042	4.426	Uridylate kinase
bin007	SOY3_bin007_00804	561	5	58	20	1.065	10.486	3.787	Ribosome-recycling factor
bin007	SOY3_bin007_00805	378	42	220	80	13.283	59.032	22.482	Ferredoxin
bin007	SOY3_bin007_00806	813	3	13	7	0.441	1.622	0.915	Isoprenyl transferase
bin007	SOY3_bin007_00807	792	4	6	10	0.604	0.768	1.341	Phosphatidate cytidylyltransferase
bin007	SOY3_bin007_00808	1050	2	10	2	0.228	0.966	0.202	putative inner membrane protein
bin007	SOY3_bin007_00809	1158	3	15	4	0.310	1.314	0.367	1-deoxy-D-xylulose 5-phosphate reductoisomerase
bin007	SOY3_bin007_00810	1047	5	20	6	0.571	1.937	0.609	Regulator of sigma-W protease RasP
bin007	SOY3_bin007_00811	987	4	18	12	0.484	1.850	1.291	4-hydroxy-3-methylbut-2-en-1-yl diphosphate synthase
bin007	SOY3_bin007_00812	1713	5	38	9	0.349	2.250	0.558	Proline--tRNA ligase
bin007	SOY3_bin007_00813	1089	3	15	8	0.329	1.397	0.780	Processive diacylglycerol beta-glucosyltransferase
bin007	SOY3_bin007_00814	465	5	11	4	1.285	2.399	0.914	Ribosome maturation factor RimP
bin007	SOY3_bin007_00815	1083	10	35	12	1.104	3.278	1.177	hypothetical protein
bin007	SOY3_bin007_00816	270	4	7	2	1.771	2.630	0.787	hypothetical protein
bin007	SOY3_bin007_00817	309	8	20	7	3.095	6.565	2.406	putative ribosomal protein YlxQ
bin007	SOY3_bin007_00818	2946	81	270	102	3.287	9.296	3.678	Translation initiation factor IF-2
bin007	SOY3_bin007_00819	354	9	31	14	3.039	8.882	4.201	Ribosome-binding factor A
bin007	SOY3_bin007_00820	984	10	57	12	1.215	5.875	1.295	Bifunctional oligoribonuclease and PAP phosphatase NrnA
bin007	SOY3_bin007_00821	924	10	24	12	1.294	2.634	1.380	tRNA pseudouridine synthase B
bin007	SOY3_bin007_00822	939	7	26	8	0.891	2.808	0.905	Riboflavin biosynthesis protein RibF
bin007	SOY3_bin007_00823	222	9	31	11	4.847	14.163	5.263	hypothetical protein
bin007	SOY3_bin007_00824	270	27	77	26	11.955	28.926	10.229	30S ribosomal protein S15
bin007	SOY3_bin007_00825	2205	67	208	86	3.633	9.568	4.143	Polyribonucleotide nucleotidyltransferase
bin007	SOY3_bin007_00826	276	1	12	1	0.433	4.410	0.385	Altronate dehydratase
bin007	SOY3_bin007_00827	1206	11	33	9	1.090	2.775	0.793	(2R)-sulfolactate sulfo-lyase subunit beta
bin007	SOY3_bin007_00828	387	1	8	4	0.309	2.097	1.098	NifU-like protein
bin007	SOY3_bin007_00829	1704	5	17	2	0.351	1.012	0.125	Limonene hydroxylase
bin007	SOY3_bin007_00830	285	1	0	0	0.419	0.000	0.000	Altronate dehydratase

bin007	SOY3_bin007_00831	201	1	0	1	0.595	0.000	0.528	hypothetical protein
bin007	SOY3_bin007_00832	435	1	1	0	0.275	0.233	0.000	hypothetical protein
bin007	SOY3_bin007_00833	834	0	3	2	0.000	0.365	0.255	Amidohydrolase
bin007	SOY3_bin007_00834	75	10	25	13	15.940	33.809	18.412	tRNA-Cys(gca)
bin007	SOY3_bin007_00835	75	7	12	3	11.158	16.228	4.249	tRNA-Gly(gcc)
bin007	SOY3_bin007_00836	76	2	14	2	3.146	18.684	2.795	tRNA-Phe(gaa)
bin007	SOY3_bin007_00837	77	18	23	4	27.946	30.296	5.518	tRNA-Asp(gtc)
bin007	SOY3_bin007_00838	897	2	15	7	0.267	1.696	0.829	Agmatinase
bin007	SOY3_bin007_00839	840	3	21	9	0.427	2.536	1.138	Spermidine synthase
bin007	SOY3_bin007_00840	459	3	8	5	0.781	1.768	1.157	pyruvoyl-dependent arginine decarboxylase
bin007	SOY3_bin007_00841	486	7	13	1	1.722	2.713	0.219	Heat induced stress protein YflT
bin007	SOY3_bin007_00842	864	0	3	0	0.000	0.352	0.000	putative endonuclease 4
bin007	SOY3_bin007_00843	1596	1	2	1	0.075	0.127	0.067	Periplasmic dipeptide transport protein precursor
bin007	SOY3_bin007_00844	831	21	74	25	3.021	9.032	3.196	hypothetical protein
bin007	SOY3_bin007_00845	252	29	81	25	13.758	32.602	10.538	hypothetical protein
bin007	SOY3_bin007_00846	636	0	8	1	0.000	1.276	0.167	EcsC protein family protein
bin007	SOY3_bin007_00847	76	2	1	1	3.146	1.335	1.398	tRNA-Ala(cgc)
bin007	SOY3_bin007_00848	1674	0	2	1	0.000	0.121	0.063	Putative penicillin-binding protein PbpX
bin007	SOY3_bin007_00849	678	1	3	1	0.176	0.449	0.157	Tetratricopeptide repeat protein
bin007	SOY3_bin007_00850	834	21	65	15	3.010	7.905	1.911	formate dehydrogenase accessory protein FdhE
bin007	SOY3_bin007_00851	216	10	61	21	5.535	28.644	10.328	Sec-independent protein translocase protein TatAd
bin007	SOY3_bin007_00852	768	14	61	31	2.179	8.056	4.288	Sec-independent protein translocase protein TatCd
bin007	SOY3_bin007_00853	633	36	181	46	6.799	29.002	7.719	Formate dehydrogenase subunit alpha precursor
bin007	SOY3_bin007_00854	2406	136	592	194	6.758	24.956	8.565	Formate dehydrogenase, nitrate-inducible, major subunit precursor
bin007	SOY3_bin007_00855	816	31	200	63	4.542	24.860	8.201	Formate dehydrogenase-O iron-sulfur subunit
bin007	SOY3_bin007_00856	1170	54	288	91	5.518	24.967	8.262	putative Ni/Fe-hydrogenase 2 b-type cytochrome subunit
bin007	SOY3_bin007_00857	255	4	11	9	1.875	4.375	3.749	Sec-independent protein translocase protein TatAd
bin007	SOY3_bin007_00858	345	1	12	1	0.347	3.528	0.308	Membrane protein of unknown function
bin007	SOY3_bin007_00859	1074	0	8	4	0.000	0.756	0.396	Nicotinate-nucleotide--dimethylbenzimidazole phosphoribosyltransferase
bin007	SOY3_bin007_00860	2712	39	81	23	1.719	3.029	0.901	Endoglucanase precursor
bin007	SOY3_bin007_00861	1122	24	66	18	2.557	5.966	1.704	Ribosome-binding ATPase YchF
bin007	SOY3_bin007_00862	354	1	3	3	0.338	0.860	0.900	hypothetical protein
bin007	SOY3_bin007_00863	933	8	16	7	1.025	1.739	0.797	hypothetical protein
bin007	SOY3_bin007_00864	174	0	0	0	0.000	0.000	0.000	hypothetical protein
bin007	SOY3_bin007_00865	222	13	40	10	7.001	18.275	4.785	hypothetical protein
bin007	SOY3_bin007_00866	318	6	11	5	2.256	3.508	1.670	Glutamate racemase 2
bin007	SOY3_bin007_00867	1407	8	34	12	0.680	2.451	0.906	Inner membrane protein YbiR
bin007	SOY3_bin007_00868	597	4	9	1	0.801	1.529	0.178	L-fuculose phosphate aldolase
bin007	SOY3_bin007_00869	537	1	4	1	0.223	0.756	0.198	hypothetical protein
bin007	SOY3_bin007_00870	1950	23	75	23	1.410	3.901	1.253	Glutamine-dependent NAD(+) synthetase
bin007	SOY3_bin007_00871	435	2	10	4	0.550	2.332	0.977	Diadenosine hexaphosphate hydrolase
bin007	SOY3_bin007_00872	741	2	13	4	0.323	1.779	0.573	Histone deacetylase-like amidohydrolase
bin007	SOY3_bin007_00873	1200	5	7	1	0.498	0.592	0.089	Fosmidomycin resistance protein
bin007	SOY3_bin007_00874	519	0	4	1	0.000	0.782	0.205	Signal peptidase I T
bin007	SOY3_bin007_00875	615	6	29	5	1.166	4.783	0.864	Coenzyme F420:L-glutamate ligase
bin007	SOY3_bin007_00876	783	3	9	0	0.458	1.166	0.000	Carboxylesterase
bin007	SOY3_bin007_00877	699	12	25	8	2.052	3.628	1.216	Succinyl-CoA ligase [ADP-forming] subunit beta
bin007	SOY3_bin007_00878	1392	5	11	1	0.429	0.802	0.076	succinyl-CoA synthetase subunit alpha
bin007	SOY3_bin007_00879	357	0	0	0	0.000	0.000	0.000	Cadmium resistance transcriptional regulatory protein CadC
bin007	SOY3_bin007_00880	2415	1	6	0	0.050	0.252	0.000	putative cadmium-transporting ATPase
bin007	SOY3_bin007_00881	156	0	1	0	0.000	0.650	0.000	hypothetical protein
bin007	SOY3_bin007_00882	435	1	2	0	0.275	0.466	0.000	primosome assembly protein PriA
bin007	SOY3_bin007_00883	222	0	0	0	0.000	0.000	0.000	hypothetical protein
bin007	SOY3_bin007_00884	144	0	1	0	0.000	0.704	0.000	hypothetical protein
bin007	SOY3_bin007_00885	1779	0	6	2	0.000	0.342	0.119	hypothetical protein
bin007	SOY3_bin007_00886	915	0	1	1	0.000	0.111	0.116	ATP-dependent DNA helicase PcrA
bin007	SOY3_bin007_00887	2949	1	7	3	0.041	0.241	0.108	DNA topoisomerase I
bin007	SOY3_bin007_00888	474	0	7	4	0.000	1.498	0.896	hypothetical protein
bin007	SOY3_bin007_00889	1347	8	14	3	0.710	1.054	0.237	replication factor C large subunit
bin007	SOY3_bin007_00890	501	2	3	1	0.477	0.607	0.212	Endonuclease V
bin007	SOY3_bin007_00891	1926	29	53	32	1.800	2.791	1.765	NADH oxidase
bin007	SOY3_bin007_00892	3906	2	12	8	0.061	0.312	0.218	Sporulation kinase A
bin007	SOY3_bin007_00893	759	0	3	0	0.000	0.401	0.000	Transposase IS200 like protein
bin007	SOY3_bin007_00894	1092	2	13	3	0.219	1.207	0.292	hypothetical protein
bin007	SOY3_bin007_00895	537	0	5	0	0.000	0.944	0.000	Methylated-DNA--protein-cysteine methyltransferase
bin007	SOY3_bin007_00896	1485	11	25	8	0.886	1.708	0.572	DNA-3-methyladenine glycosylase 2
bin007	SOY3_bin007_00897	1218	3	34	7	0.294	2.831	0.610	Sensor histidine kinase YpdA

bin007	SOY3_bin007_00898	831	2	26	7	0.288	3.173	0.895	putative response regulatory protein
bin007	SOY3_bin007_00899	2409	2	17	7	0.099	0.716	0.309	Benzylsuccinate synthase alpha subunit
bin007	SOY3_bin007_00900	903	0	2	0	0.000	0.225	0.000	Benzylsuccinate synthase activating enzyme
bin007	SOY3_bin007_00901	1026	0	2	0	0.000	0.198	0.000	hypothetical protein
bin007	SOY3_bin007_00902	195	0	4	2	0.000	2.081	1.089	2-hydroxymuconate tautomerase
bin007	SOY3_bin007_00903	201	1	4	2	0.595	2.018	1.057	hypothetical protein
bin007	SOY3_bin007_00904	1230	7	32	7	0.680	2.639	0.605	LL-diaminopimelate aminotransferase
bin007	SOY3_bin007_00905	2415	51	107	55	2.525	4.494	2.419	Methionine synthase
bin007	SOY3_bin007_00906	795	12	40	8	1.805	5.103	1.069	Vitamin B12 dependent methionine synthase, activation domain
bin007	SOY3_bin007_00907	579	2	8	8	0.413	1.401	1.468	TVP38/TMEM64 family inner membrane protein YdjZ
bin007	SOY3_bin007_00908	666	15	66	21	2.693	10.051	3.349	Nitrite reductase [NAD(P)H]
bin007	SOY3_bin007_00909	348	2	0	0	0.687	0.000	0.000	Transcriptional repressor SdpR
bin007	SOY3_bin007_00910	1005	1	1	0	0.119	0.101	0.000	putative permease
bin007	SOY3_bin007_00911	231	0	1	0	0.000	0.439	0.000	hypothetical protein
bin007	SOY3_bin007_00912	579	1	24	11	0.206	4.204	2.018	R-phenyllactate dehydratase beta subunit
bin007	SOY3_bin007_00913	342	0	17	5	0.000	5.042	1.553	R-phenyllactate dehydratase activator
bin007	SOY3_bin007_00914	1143	4	141	38	0.418	12.512	3.532	hypothetical protein
bin007	SOY3_bin007_00915	1584	1	26	5	0.075	1.665	0.335	Xylose import ATP-binding protein XylG
bin007	SOY3_bin007_00916	1032	1	11	7	0.116	1.081	0.721	D-allose transport system permease protein AlsC
bin007	SOY3_bin007_00917	1056	0	7	0	0.000	0.672	0.000	Ribose transport system permease protein RbsC
bin007	SOY3_bin007_00918	363	0	1	0	0.000	0.279	0.000	hypothetical protein
bin007	SOY3_bin007_00919	681	0	0	0	0.000	0.000	0.000	putative Mg(2+) transport ATPase
bin007	SOY3_bin007_00920	663	0	0	0	0.000	0.000	0.000	VTC domain protein
bin007	SOY3_bin007_00921	1425	12	23	9	1.007	1.637	0.671	Sensor histidine kinase CssS
bin007	SOY3_bin007_00922	699	2	18	8	0.342	2.612	1.216	Transcriptional regulatory protein CseB
bin007	SOY3_bin007_00923	273	252	883	347	110.353	328.060	135.020	hypothetical protein
bin007	SOY3_bin007_00924	486	0	3	3	0.000	0.626	0.656	HTH-type transcriptional regulator ImmR
bin007	SOY3_bin007_00925	630	3	5	1	0.569	0.805	0.169	2-amino-4-deoxychorismate dehydrogenase
bin007	SOY3_bin007_00926	966	5	9	4	0.619	0.945	0.440	Amidase enhancer precursor
bin007	SOY3_bin007_00927	1248	7	8	3	0.671	0.650	0.255	UDP-N-acetylglucosamine 1-carboxyvinyltransferase 1
bin007	SOY3_bin007_00928	801	0	11	4	0.000	1.393	0.530	hypothetical protein
bin007	SOY3_bin007_00929	411	81	395	158	23.561	97.479	40.836	ATP synthase epsilon chain
bin007	SOY3_bin007_00930	1413	138	654	235	11.676	46.945	17.667	ATP synthase subunit beta
bin007	SOY3_bin007_00931	864	114	449	149	15.774	52.709	18.319	ATP synthase gamma chain
bin007	SOY3_bin007_00932	1518	133	622	214	10.474	41.560	14.975	ATP synthase subunit alpha
bin007	SOY3_bin007_00933	546	45	212	76	9.853	39.382	14.786	ATP synthase subunit delta
bin007	SOY3_bin007_00934	486	33	243	59	8.118	50.714	12.896	ATP synthase subunit b, sodium ion specific
bin007	SOY3_bin007_00935	234	64	303	112	32.697	131.335	50.843	ATP synthase subunit c
bin007	SOY3_bin007_00936	735	55	363	119	8.946	50.093	17.198	ATP synthase subunit a
bin007	SOY3_bin007_00937	405	15	44	14	4.428	11.019	3.672	ATP synthase I chain
bin007	SOY3_bin007_00938	264	15	55	24	6.793	21.131	9.657	Putative F0F1-ATPase subunit (ATPase_gene1)
bin007	SOY3_bin007_00939	1155	10	33	14	1.035	2.898	1.288	UDP-N-acetylglucosamine 2-epimerase
bin007	SOY3_bin007_00940	315	1	8	5	0.380	2.576	1.686	MazG nucleotide pyrophosphohydrolase domain protein
bin007	SOY3_bin007_00941	600	9	32	24	1.793	5.409	4.249	tRNA-specific adenosine deaminase
bin007	SOY3_bin007_00942	1248	5	28	9	0.479	2.276	0.766	Serine hydroxymethyltransferase
bin007	SOY3_bin007_00943	459	2	15	2	0.521	3.315	0.463	Putative sugar phosphate isomerase YwlF
bin007	SOY3_bin007_00944	453	0	9	2	0.000	2.015	0.469	Low molecular weight protein-tyrosine-phosphatase YwlE
bin007	SOY3_bin007_00945	543	0	10	1	0.000	1.868	0.196	putative manganese efflux pump MntP
bin007	SOY3_bin007_00946	1071	14	64	33	1.563	6.061	3.273	Threonylcarbamoyl-AMP synthase
bin007	SOY3_bin007_00947	867	5	14	9	0.689	1.638	1.103	Release factor glutamine methyltransferase
bin007	SOY3_bin007_00948	1068	14	59	19	1.567	5.603	1.890	Peptide chain release factor 1
bin007	SOY3_bin007_00949	870	11	28	4	1.512	3.264	0.488	hypothetical protein
bin007	SOY3_bin007_00950	204	16	52	27	9.376	25.854	14.059	50S ribosomal protein L31
bin007	SOY3_bin007_00951	1251	24	78	27	2.294	6.324	2.293	Cyclic pyranopterin monophosphate synthase
bin007	SOY3_bin007_00952	387	1	5	4	0.309	1.310	1.098	hypothetical protein
bin007	SOY3_bin007_00953	117	0	0	0	0.000	0.000	0.000	hypothetical protein
bin007	SOY3_bin007_00954	1434	0	2	1	0.000	0.141	0.074	Phenolic acid decarboxylase subunit C
bin007	SOY3_bin007_00955	228	0	1	0	0.000	0.445	0.000	Phenolic acid decarboxylase subunit D
bin007	SOY3_bin007_00956	555	0	3	0	0.000	0.548	0.000	Phenolic acid decarboxylase subunit B
bin007	SOY3_bin007_00957	189	0	1	0	0.000	0.537	0.000	hypothetical protein
bin007	SOY3_bin007_00958	975	0	1	0	0.000	0.104	0.000	HTH-type transcriptional regulator CynR
bin007	SOY3_bin007_00959	525	1	5	0	0.228	0.966	0.000	Isopentenyl-diphosphate Delta-isomerase
bin007	SOY3_bin007_00960	1953	5	16	2	0.306	0.831	0.109	Purine catabolism regulatory protein
bin007	SOY3_bin007_00961	228	0	4	1	0.000	1.779	0.466	Phenolic acid decarboxylase subunit D
bin007	SOY3_bin007_00962	1425	0	14	7	0.000	0.996	0.522	Phenolic acid decarboxylase subunit C
bin007	SOY3_bin007_00963	573	0	5	4	0.000	0.885	0.742	Phenolic acid decarboxylase subunit B
bin007	SOY3_bin007_00964	546	9	7	4	1.971	1.300	0.778	Murein DD-endopeptidase MepM

bin007	SOY3_bin007_00965	714	3	12	2	0.502	1.705	0.298	Serine/threonine phosphatase stp
bin007	SOY3_bin007_00966	501	0	9	1	0.000	1.822	0.212	FHA domain-containing protein FhaB
bin007	SOY3_bin007_00967	762	2	18	5	0.314	2.396	0.697	FHA domain-containing protein FhaB
bin007	SOY3_bin007_00968	1101	9	33	10	0.977	3.040	0.965	putative dual-specificity RNA methyltransferase RlmN
bin007	SOY3_bin007_00969	1368	7	51	17	0.612	3.781	1.320	Ribosomal RNA small subunit methyltransferase B
bin007	SOY3_bin007_00970	777	6	32	13	0.923	4.177	1.777	hypothetical protein
bin007	SOY3_bin007_00971	960	12	60	23	1.494	6.339	2.545	Methionyl-tRNA formyltransferase
bin007	SOY3_bin007_00972	453	2	13	10	0.528	2.911	2.345	Peptide deformylase
bin007	SOY3_bin007_00973	2238	33	101	51	1.763	4.577	2.421	Primosomal protein N'
bin007	SOY3_bin007_00974	183	0	3	1	0.000	1.663	0.580	hypothetical protein
bin007	SOY3_bin007_00975	1203	14	111	41	1.391	9.359	3.620	Coenzyme A biosynthesis bifunctional protein CoaBC
bin007	SOY3_bin007_00976	207	0	18	4	0.000	8.820	2.053	DNA-directed RNA polymerase subunit omega
bin007	SOY3_bin007_00977	606	11	45	21	2.170	7.532	3.681	Guanylate kinase
bin007	SOY3_bin007_00978	273	4	26	10	1.752	9.660	3.891	hypothetical protein
bin007	SOY3_bin007_00979	879	9	58	23	1.224	6.693	2.780	hypothetical protein
bin007	SOY3_bin007_00980	1194	26	156	74	2.603	13.252	6.584	Aspartate aminotransferase
bin007	SOY3_bin007_00981	846	30	59	21	4.239	7.074	2.637	Diaminopimelate epimerase
bin007	SOY3_bin007_00982	2751	19	50	14	0.826	1.843	0.541	Calcium-transporting ATPase
bin007	SOY3_bin007_00983	498	5	18	5	1.200	3.666	1.067	mannosyl-3-phosphoglycerate phosphatase
bin007	SOY3_bin007_00984	951	16	37	10	2.011	3.946	1.117	Activator of (R)-2-hydroxyglutaryl-CoA dehydratase
bin007	SOY3_bin007_00985	438	2	16	5	0.546	3.705	1.213	NifU-like protein
bin007	SOY3_bin007_00986	414	5	16	4	1.444	3.920	1.026	NifU-like protein
bin007	SOY3_bin007_00987	76	5	5	5	7.865	6.673	6.989	tRNA-Val(tac)
bin007	SOY3_bin007_00988	76	1	2	1	1.573	2.669	1.398	tRNA-Glu(ttc)
bin007	SOY3_bin007_00989	75	2	12	4	3.188	16.228	5.665	tRNA-Met(cat)
bin007	SOY3_bin007_00990	75	4	10	4	6.376	13.524	5.665	tRNA-Asn(gtt)
bin007	SOY3_bin007_00991	1095	12	45	20	1.310	4.168	1.940	RNA polymerase sigma factor SigA
bin007	SOY3_bin007_00992	1893	26	71	22	1.642	3.804	1.235	DNA primase
bin007	SOY3_bin007_00993	129	0	1	0	0.000	0.786	0.000	hypothetical protein
bin007	SOY3_bin007_00994	1002	6	13	4	0.716	1.316	0.424	Deoxyguanosinetriphosphate triphosphohydrolase
bin007	SOY3_bin007_00995	588	0	16	0	0.000	2.760	0.000	2'-5'-RNA ligase
bin007	SOY3_bin007_00996	2658	30	99	29	1.349	3.778	1.159	Pyruvate, phosphate dikinase
bin007	SOY3_bin007_00997	867	4	13	9	0.552	1.521	1.103	Putative pyruvate, phosphate dikinase regulatory protein
bin007	SOY3_bin007_00998	639	3	13	7	0.561	2.063	1.164	Transcriptional repressor CcpN
bin007	SOY3_bin007_00999	2118	9	37	15	0.508	1.772	0.752	Glycine--tRNA ligase beta subunit
bin007	SOY3_bin007_01000	879	4	17	4	0.544	1.962	0.483	Glycine--tRNA ligase alpha subunit
bin007	SOY3_bin007_01001	423	2	4	0	0.565	0.959	0.000	nascent polypeptide-associated complex protein
bin007	SOY3_bin007_01002	771	3	5	5	0.465	0.658	0.689	DNA repair protein RecO
bin007	SOY3_bin007_01003	1365	3	14	7	0.263	1.040	0.545	Magnesium transporter MgtE
bin007	SOY3_bin007_01004	909	1	13	4	0.132	1.451	0.467	GTPase Era
bin007	SOY3_bin007_01005	429	0	3	0	0.000	0.709	0.000	Cytidine deaminase
bin007	SOY3_bin007_01006	699	0	10	5	0.000	1.451	0.760	Undecaprenol kinase
bin007	SOY3_bin007_01007	459	1	1	2	0.260	0.221	0.463	Endoribonuclease YbeY
bin007	SOY3_bin007_01008	189	1	5	0	0.633	2.683	0.000	hypothetical protein
bin007	SOY3_bin007_01009	327	1	6	1	0.366	1.861	0.325	hypothetical protein
bin007	SOY3_bin007_01010	579	3	23	11	0.619	4.029	2.018	Desulfoferrodoxin
bin007	SOY3_bin007_01011	2034	1	1	2	0.059	0.050	0.104	Denitrification regulatory protein NirQ
bin007	SOY3_bin007_01012	465	1	0	0	0.257	0.000	0.000	hypothetical protein
bin007	SOY3_bin007_01013	348	0	1	0	0.000	0.291	0.000	hypothetical protein
bin007	SOY3_bin007_01014	210	0	0	1	0.000	0.000	0.506	Virginiamycin A acetyltransferase
bin007	SOY3_bin007_01015	498	0	0	2	0.000	0.000	0.427	Lincosamide resistance protein
bin007	SOY3_bin007_01016	1392	1	8	0	0.086	0.583	0.000	23S rRNA (uracil-C(5))-methyltransferase RlmCD
bin007	SOY3_bin007_01017	198	0	0	1	0.000	0.000	0.536	hypothetical protein
bin007	SOY3_bin007_01018	453	2	6	1	0.528	1.343	0.234	Nucleotidyltransferase domain protein
bin007	SOY3_bin007_01019	429	0	7	2	0.000	1.655	0.495	hypothetical protein
bin007	SOY3_bin007_01020	729	2	9	1	0.328	1.252	0.146	NADH pyrophosphatase
bin007	SOY3_bin007_01021	3231	13	64	31	0.481	2.009	1.019	Carbamoyl-phosphate synthase large chain
bin007	SOY3_bin007_01022	405	3	2	1	0.886	0.501	0.262	hypothetical protein
bin007	SOY3_bin007_01023	1194	73	246	72	7.309	20.897	6.406	Anaerobic nitric oxide reductase flavorubredoxin
bin007	SOY3_bin007_01024	528	1	12	3	0.226	2.305	0.604	hypothetical protein
bin007	SOY3_bin007_01025	372	5	38	13	1.607	10.361	3.712	hypothetical protein
bin007	SOY3_bin007_01026	1329	1	19	7	0.090	1.450	0.560	Arsenical pump membrane protein
bin007	SOY3_bin007_01027	306	0	5	3	0.000	1.657	1.041	hypothetical protein
bin007	SOY3_bin007_01028	2199	7	33	9	0.381	1.522	0.435	Ribonucleoside-diphosphate reductase NrdZ
bin007	SOY3_bin007_01029	492	2	4	2	0.486	0.825	0.432	hypothetical protein
bin007	SOY3_bin007_01030	1110	2	9	10	0.215	0.822	0.957	hypothetical protein
bin007	SOY3_bin007_01031	2241	1	8	0	0.053	0.362	0.000	Malto-oligosyltrehalose trehalohydrolase

bin007	SOY3_bin007_01032	216	0	1	0	0.000	0.470	0.000	hypothetical protein
bin007	SOY3_bin007_01033	321	1	4	1	0.372	1.264	0.331	hypothetical protein
bin007	SOY3_bin007_01034	1104	1	13	5	0.108	1.194	0.481	Response regulator PleD
bin007	SOY3_bin007_01035	243	0	3	1	0.000	1.252	0.437	Small, acid-soluble spore protein H
bin007	SOY3_bin007_01036	129	0	0	1	0.000	0.000	0.823	hypothetical protein
bin007	SOY3_bin007_01037	474	1	1	4	0.252	0.214	0.896	Spore germination protein YaaH
bin007	SOY3_bin007_01038	1296	5	18	5	0.461	1.409	0.410	Gamma-glutamyl phosphate reductase
bin007	SOY3_bin007_01039	711	1	9	2	0.168	1.284	0.299	chromosome segregation protein
bin007	SOY3_bin007_01040	1119	18	36	11	1.923	3.263	1.044	Putative GTP cyclohydrolase 1 type 2
bin007	SOY3_bin007_01041	699	41	108	33	7.012	15.671	5.015	tRNA (adenine(22)-N(1))-methyltransferase
bin007	SOY3_bin007_01042	408	2	8	3	0.586	1.989	0.781	hypothetical protein
bin007	SOY3_bin007_01043	1794	8	26	19	0.533	1.470	1.125	hypothetical protein
bin007	SOY3_bin007_01044	576	0	8	1	0.000	1.409	0.184	Orotate phosphoribosyltransferase
bin007	SOY3_bin007_01045	1179	3	10	6	0.304	0.860	0.541	putative metallophosphoesterase
bin007	SOY3_bin007_01046	1575	9	32	6	0.683	2.061	0.405	Protease inhibitor precursor
bin007	SOY3_bin007_01047	819	7	25	13	1.022	3.096	1.686	Exoglucanase B precursor
bin007	SOY3_bin007_01048	1623	1	4	0	0.074	0.250	0.000	Spore germination protein A1
bin007	SOY3_bin007_01049	1524	5	46	18	0.392	3.061	1.255	Folylpolyglutamate synthase
bin007	SOY3_bin007_01050	2649	14	62	19	0.632	2.374	0.762	Valine--tRNA ligase
bin007	SOY3_bin007_01051	837	2	8	3	0.286	0.969	0.381	Modification methylase DpnIIA
bin007	SOY3_bin007_01052	465	2	5	1	0.514	1.091	0.228	hypothetical protein
bin007	SOY3_bin007_01053	1086	3	10	0	0.330	0.934	0.000	NMT1/THI5 like protein
bin007	SOY3_bin007_01054	693	3	11	1	0.518	1.610	0.153	hypothetical protein
bin007	SOY3_bin007_01055	606	20	28	8	3.946	4.686	1.402	putative GTP-binding protein EngB
bin007	SOY3_bin007_01056	2421	50	111	46	2.469	4.650	2.018	Lon protease 1
bin007	SOY3_bin007_01057	1725	9	6	5	0.624	0.353	0.308	Lon protease 2
bin007	SOY3_bin007_01058	648	6	25	5	1.107	3.913	0.820	Inner membrane protein YaaH
bin007	SOY3_bin007_01059	201	0	2	1	0.000	1.009	0.528	hypothetical protein
bin007	SOY3_bin007_01060	420	0	7	1	0.000	1.690	0.253	hypothetical protein
bin007	SOY3_bin007_01061	288	0	1	3	0.000	0.352	1.107	hypothetical protein
bin007	SOY3_bin007_01062	312	0	1	0	0.000	0.325	0.000	Toxin ParE1
bin007	SOY3_bin007_01063	267	0	2	0	0.000	0.760	0.000	Phd_YefM
bin007	SOY3_bin007_01064	726	4	13	3	0.659	1.816	0.439	Energy-coupling factor transporter transmembrane protein EcfT
bin007	SOY3_bin007_01065	693	0	20	3	0.000	2.927	0.460	Nickel import ATP-binding protein NikO
bin007	SOY3_bin007_01066	747	3	31	15	0.480	4.209	2.133	Energy-coupling factor transporter ATP-binding protein EcfA2
bin007	SOY3_bin007_01067	612	0	18	12	0.000	2.983	2.083	hypothetical protein
bin007	SOY3_bin007_01068	1140	1	57	22	0.105	5.071	2.050	Cysteine desulfurase
bin007	SOY3_bin007_01069	261	0	12	8	0.000	4.663	3.256	hypothetical protein
bin007	SOY3_bin007_01070	855	1	66	42	0.140	7.829	5.218	putative tRNA (adenine(37)-N6)-methyltransferase
bin007	SOY3_bin007_01071	744	0	48	31	0.000	6.544	4.426	hypothetical protein
bin007	SOY3_bin007_01072	1365	1	119	50	0.088	8.842	3.891	Endoglucanase precursor
bin007	SOY3_bin007_01073	5637	2	295	134	0.042	5.308	2.525	Bacterial Ig-like domain (group 2)
bin007	SOY3_bin007_01074	5922	1	636	236	0.020	10.893	4.233	Kappa-carrageenase precursor
bin007	SOY3_bin007_01075	3333	1	164	57	0.036	4.991	1.817	Calcineurin-like phosphoesterase
bin007	SOY3_bin007_01076	1473	13	31	9	1.055	2.135	0.649	Phosphoglucosamine mutase
bin007	SOY3_bin007_01077	261	0	0	1	0.000	0.000	0.407	Ferrous iron transport protein A
bin007	SOY3_bin007_01078	246	0	1	0	0.000	0.412	0.000	FeoA domain protein
bin007	SOY3_bin007_01079	2052	3	16	5	0.175	0.791	0.259	Ferrous iron transport protein B
bin007	SOY3_bin007_01080	288	0	5	1	0.000	1.761	0.369	hypothetical protein
bin007	SOY3_bin007_01081	1614	3	15	6	0.222	0.943	0.395	1,4-alpha-glucan branching enzyme
bin007	SOY3_bin007_01082	753	2	5	0	0.318	0.673	0.000	hypothetical protein
bin007	SOY3_bin007_01083	543	3	6	1	0.660	1.121	0.196	Endonuclease III
bin007	SOY3_bin007_01084	459	3	6	1	0.781	1.326	0.231	tRNA (cytidine(34)-2'-O)-methyltransferase
bin007	SOY3_bin007_01085	462	0	2	2	0.000	0.439	0.460	Cys-tRNA(Pro)/Cys-tRNA(Cys) deacylase YbaK
bin007	SOY3_bin007_01086	3603	16	51	16	0.531	1.436	0.472	DNA polymerase III subunit alpha
bin007	SOY3_bin007_01087	207	1	1	1	0.578	0.490	0.513	Phosphatidylglycerophosphatase A
bin007	SOY3_bin007_01088	234	12	37	19	6.131	16.038	8.625	Transcription attenuation protein MtrB
bin007	SOY3_bin007_01089	192	5	10	4	3.113	5.283	2.213	hypothetical protein
bin007	SOY3_bin007_01090	852	11	27	12	1.543	3.214	1.496	Acetyl-coenzyme A carboxylase carboxyl transferase subunit beta
bin007	SOY3_bin007_01091	957	6	30	9	0.750	3.180	0.999	Acetyl-coenzyme A carboxylase carboxyl transferase subunit alpha
bin007	SOY3_bin007_01092	963	11	39	15	1.366	4.108	1.655	6-phosphofructokinase
bin007	SOY3_bin007_01093	1755	33	120	51	2.248	6.935	3.087	Pyruvate kinase
bin007	SOY3_bin007_01094	603	2	4	0	0.397	0.673	0.000	Inner membrane protein YabI
bin007	SOY3_bin007_01095	1083	20	50	32	2.208	4.683	3.139	O-acetyl-ADP-ribose deacetylase
bin007	SOY3_bin007_01096	294	3	16	4	1.220	5.520	1.445	hypothetical protein
bin007	SOY3_bin007_01097	507	2	15	5	0.472	3.001	1.048	hypothetical protein
bin007	SOY3_bin007_01098	300	1	11	7	0.398	3.719	2.479	FHA domain protein

bin007	SOY3_bin007_01099	219	3	9	5	1.638	4.168	2.425	hypothetical protein
bin007	SOY3_bin007_01100	216	17	43	17	9.409	20.192	8.360	hypothetical protein
bin007	SOY3_bin007_01101	1371	8	26	4	0.698	1.923	0.310	Multidrug resistance protein stp
bin007	SOY3_bin007_01102	681	2	5	3	0.351	0.745	0.468	hypothetical protein
bin007	SOY3_bin007_01103	381	2	2	1	0.628	0.532	0.279	hypothetical protein
bin007	SOY3_bin007_01104	315	21	63	22	7.970	20.285	7.419	hypothetical protein
bin007	SOY3_bin007_01105	363	3	62	6	0.988	17.324	1.756	Dinitrogenase iron-molybdenum cofactor
bin007	SOY3_bin007_01106	333	7	65	15	2.513	19.798	4.785	hypothetical protein
bin007	SOY3_bin007_01107	360	8	79	25	2.657	22.258	7.377	hypothetical protein
bin007	SOY3_bin007_01108	867	27	184	60	3.723	21.526	7.351	Septum site-determining protein MinD
bin007	SOY3_bin007_01109	909	22	77	34	2.893	8.592	3.973	Pyruvate synthase subunit PorD
bin007	SOY3_bin007_01110	342	10	27	7	3.496	8.007	2.174	Dinitrogenase iron-molybdenum cofactor
bin007	SOY3_bin007_01111	942	24	90	40	3.046	9.691	4.511	Flagellum site-determining protein YlxH
bin007	SOY3_bin007_01112	399	11	68	23	3.296	17.286	6.123	hypothetical protein
bin007	SOY3_bin007_01113	402	0	5	3	0.000	1.262	0.793	hypothetical protein
bin007	SOY3_bin007_01114	594	0	5	1	0.000	0.854	0.179	Glutathione-regulated potassium-efflux system ancillary protein KefF
bin007	SOY3_bin007_01115	714	1	2	0	0.167	0.284	0.000	Cypemycin methyltransferase
bin007	SOY3_bin007_01116	1836	1	13	7	0.065	0.718	0.405	Primary amine oxidase precursor
bin007	SOY3_bin007_01117	408	2	17	2	0.586	4.226	0.521	Chromosome partition protein Smc
bin007	SOY3_bin007_01118	267	2	11	4	0.895	4.179	1.591	hypothetical protein
bin007	SOY3_bin007_01119	444	0	5	1	0.000	1.142	0.239	hypothetical protein
bin007	SOY3_bin007_01120	1092	1	0	0	0.109	0.000	0.000	hypothetical protein
bin007	SOY3_bin007_01121	291	3	8	4	1.232	2.788	1.460	Phd_YefM
bin007	SOY3_bin007_01122	423	0	0	0	0.000	0.000	0.000	putative diguanylate cyclase YegE
bin007	SOY3_bin007_01123	77	1	5	1	1.553	6.586	1.380	tRNA-Met(cat)
bin007	SOY3_bin007_01124	76	0	1	1	0.000	1.335	1.398	tRNA-Thr(ggt)
bin007	SOY3_bin007_01125	76	2	7	3	3.146	9.342	4.193	tRNA-Met(cat)
bin007	SOY3_bin007_01126	85	0	0	2	0.000	0.000	2.499	tRNA-Tyr(gta)
bin007	SOY3_bin007_01127	76	7	9	9	11.011	12.011	12.579	tRNA-Thr(tgt)
bin007	SOY3_bin007_01128	645	42	129	58	7.785	20.285	9.552	RNA polymerase sigma-H factor
bin007	SOY3_bin007_01129	510	1	5	5	0.234	0.994	1.041	YacP-like NYN domain protein
bin007	SOY3_bin007_01130	618	11	26	15	2.128	4.267	2.578	Putative TrmH family tRNA/rRNA methyltransferase
bin007	SOY3_bin007_01131	966	15	32	13	1.856	3.360	1.430	Thymidylate synthase ThyX
bin007	SOY3_bin007_01132	516	15	43	11	3.475	8.452	2.265	Mini-ribonuclease 3
bin007	SOY3_bin007_01133	1587	38	78	26	2.863	4.985	1.740	Cysteine--tRNA ligase
bin007	SOY3_bin007_01134	1467	33	77	38	2.689	5.324	2.752	Glutamate--tRNA ligase
bin007	SOY3_bin007_01135	471	7	15	12	1.777	3.230	2.706	2-C-methyl-D-erythritol 2,4-cyclodiphosphate synthase
bin007	SOY3_bin007_01136	624	3	9	5	0.575	1.463	0.851	hypothetical protein
bin007	SOY3_bin007_01137	738	81	124	50	13.121	17.042	7.197	2-C-methyl-D-erythritol 4-phosphate cytidylyltransferase
bin007	SOY3_bin007_01138	1146	24	95	39	2.504	8.408	3.615	putative PIN and TRAM-domain containing protein precursor
bin007	SOY3_bin007_01139	477	68	95	45	17.043	20.200	10.021	RNA polymerase-binding transcription factor CarD
bin007	SOY3_bin007_01140	1077	9	40	20	0.999	3.767	1.973	DNA integrity scanning protein DisA
bin007	SOY3_bin007_01141	1353	29	88	36	2.562	6.597	2.826	hypothetical protein
bin007	SOY3_bin007_01142	546	17	43	14	3.722	7.988	2.724	hypothetical protein
bin007	SOY3_bin007_01143	2442	23	48	20	1.126	1.994	0.870	Negative regulator of genetic competence ClpC/MecB
bin007	SOY3_bin007_01144	1071	11	21	7	1.228	1.989	0.694	Putative ATP:guanido phosphotransferase
bin007	SOY3_bin007_01145	537	6	12	4	1.336	2.267	0.791	excinuclease ABC subunit B
bin007	SOY3_bin007_01146	462	4	5	0	1.035	1.098	0.000	Transcriptional regulator CtsR
bin007	SOY3_bin007_01147	75	3	10	5	4.782	13.524	7.082	tRNA-Asn(gtt)
bin007	SOY3_bin007_01148	1854	7	65	41	0.451	3.556	2.349	NADH oxidoreductase hcr
bin007	SOY3_bin007_01149	1035	6	54	34	0.693	5.292	3.490	methylcobalamin:coenzyme M methyltransferase
bin007	SOY3_bin007_01150	957	1	32	15	0.125	3.392	1.665	Benzylsuccinate synthase activating enzyme
bin007	SOY3_bin007_01151	2400	1	167	128	0.050	7.058	5.665	4-hydroxyphenylacetate decarboxylase large subunit
bin007	SOY3_bin007_01152	648	3	14	5	0.553	2.191	0.820	hypothetical protein
bin007	SOY3_bin007_01153	1608	1	5	4	0.074	0.315	0.264	HTH-type transcriptional regulator YesS
bin007	SOY3_bin007_01154	1851	1	9	1	0.065	0.493	0.057	Sensor histidine kinase YpdA
bin007	SOY3_bin007_01155	138	1	0	2	0.866	0.000	1.540	hypothetical protein
bin007	SOY3_bin007_01156	852	0	3	2	0.000	0.357	0.249	Putative electron transport protein YccM
bin007	SOY3_bin007_01157	804	3	30	3	0.446	3.785	0.396	Thiol-disulfide oxidoreductase ResA
bin007	SOY3_bin007_01158	582	28	82	31	5.751	14.290	5.658	Acetyltransferase YpeA
bin007	SOY3_bin007_01159	87	0	0	0	0.000	0.000	0.000	5S ribosomal RNA
bin007	SOY3_bin007_01160	483	4	18	7	0.990	3.780	1.540	hypothetical protein
bin007	SOY3_bin007_01161	489	1	10	1	0.244	2.074	0.217	Shikimate kinase 1
bin007	SOY3_bin007_01162	1161	12	30	9	1.236	2.621	0.823	Chorismate synthase
bin007	SOY3_bin007_01163	885	16	44	16	2.161	5.043	1.920	Shikimate dehydrogenase
bin007	SOY3_bin007_01164	525	2	15	3	0.455	2.898	0.607	putative hydrolase YutF
bin007	SOY3_bin007_01165	606	8	21	11	1.578	3.515	1.928	putative molybdenum cofactor guanylyltransferase

bin007	SOY3_bin007_01166	708	1	5	3	0.169	0.716	0.450	RNA polymerase sigma-28 factor precursor
bin007	SOY3_bin007_01167	93	0	0	0	0.000	0.000	0.000	hypothetical protein
bin007	SOY3_bin007_01168	1944	3	5	2	0.184	0.261	0.109	C1q domain protein
bin007	SOY3_bin007_01169	654	9	35	13	1.645	5.428	2.112	Nitrite reductase [NAD(P)H]
bin007	SOY3_bin007_01170	135	0	0	1	0.000	0.000	0.787	hypothetical protein
bin007	SOY3_bin007_01171	1173	7	32	10	0.713	2.767	0.906	Sporulation-specific N-acetylmuramoyl-L-alanine amidase
bin007	SOY3_bin007_01172	693	2	1	1	0.345	0.146	0.153	WbqC-like protein family protein
bin007	SOY3_bin007_01173	936	7	12	2	0.894	1.300	0.227	dTDP-glucose 4,6-dehydratase
bin007	SOY3_bin007_01174	1257	1	4	1	0.095	0.323	0.085	Undecaprenyl phosphate N,N'-diacetylbacillosamine 1-phosphate transferase
bin007	SOY3_bin007_01175	1302	11	28	13	1.010	2.181	1.061	hypothetical protein
bin007	SOY3_bin007_01176	642	2	13	2	0.372	2.054	0.331	Carbohydrate binding domain protein
bin007	SOY3_bin007_01177	1155	2	7	3	0.207	0.615	0.276	Beta-monoglucosyldiacylglycerol synthase
bin007	SOY3_bin007_01178	2877	1	1	1	0.042	0.035	0.037	Y_Y_Y domain protein
bin007	SOY3_bin007_01179	2925	1	2	6	0.041	0.069	0.218	Serine acetyltransferase
bin007	SOY3_bin007_01180	972	2	21	3	0.246	2.191	0.328	Calcineurin-like phosphoesterase
bin007	SOY3_bin007_01181	630	0	3	1	0.000	0.483	0.169	glucosamine-6-phosphate deaminase-like protein
bin007	SOY3_bin007_01182	1209	1	3	3	0.099	0.252	0.264	Putative teichuronic acid biosynthesis glycosyltransferase TuaC
bin007	SOY3_bin007_01183	1395	1	3	0	0.086	0.218	0.000	Teichuronic acid biosynthesis protein TuaE
bin007	SOY3_bin007_01184	1491	2	5	2	0.160	0.340	0.142	colanic acid exporter
bin007	SOY3_bin007_01185	1095	4	2	1	0.437	0.185	0.097	UDP-2-acetamido-2-deoxy-3-oxo-D-glucuronate aminotransferase
bin007	SOY3_bin007_01186	1326	4	6	1	0.361	0.459	0.080	UDP-N-acetyl-D-glucosamine 6-dehydrogenase
bin007	SOY3_bin007_01187	1830	0	7	2	0.000	0.388	0.116	UDP-N-acetyl-alpha-D-glucosamine C6 dehydratase
bin007	SOY3_bin007_01188	207	8	74	38	4.620	36.259	19.500	hypothetical protein
bin007	SOY3_bin007_01189	1023	3	27	13	0.351	2.677	1.350	C4-dicarboxylate-binding periplasmic protein precursor
bin007	SOY3_bin007_01190	537	1	8	4	0.223	1.511	0.791	Sialic acid TRAP transporter permease protein SiaT
bin007	SOY3_bin007_01191	1281	1	19	5	0.093	1.504	0.415	Sialic acid TRAP transporter permease protein SiaT
bin007	SOY3_bin007_01192	804	0	40	23	0.000	5.046	3.039	putative ABC transporter ATP-binding protein
bin007	SOY3_bin007_01193	1116	0	80	26	0.000	7.271	2.475	Fe(3+)-citrate-binding protein YfmC precursor
bin007	SOY3_bin007_01194	624	0	42	21	0.000	6.827	3.575	Malonyl-[acyl-carrier protein] O-methyltransferase
bin007	SOY3_bin007_01195	1785	2	101	38	0.134	5.739	2.261	corrinoid ABC transporter substrate-binding protein
bin007	SOY3_bin007_01196	1695	4	128	65	0.282	7.659	4.074	3',5'-cyclic adenosine monophosphate phosphodiesterase CpdA
bin007	SOY3_bin007_01197	711	0	9	1	0.000	1.284	0.149	Lipoprotein-releasing system ATP-binding protein LolD
bin007	SOY3_bin007_01198	2379	8	45	14	0.402	1.919	0.625	Macrolide export ATP-binding/permease protein MacB
bin007	SOY3_bin007_01199	1170	3	18	6	0.307	1.560	0.545	Macrolide export protein MacA
bin007	SOY3_bin007_01200	1704	15	66	24	1.052	3.929	1.496	Dihydroxy-acid dehydratase
bin007	SOY3_bin007_01201	879	30	42	27	4.080	4.846	3.263	O-acetylserine sulfhydrylase
bin007	SOY3_bin007_01202	456	11	20	9	2.884	4.449	2.097	HTH-type transcriptional regulator CymR
bin007	SOY3_bin007_01203	1170	7	20	12	0.715	1.734	1.089	Cysteine desulfurase
bin007	SOY3_bin007_01204	729	12	15	6	1.968	2.087	0.874	Serine acetyltransferase
bin007	SOY3_bin007_01205	678	1	11	5	0.176	1.646	0.783	Ditrans,polycis-undecaprenyl-diphosphate synthase ((2E,6E)-farnesyl-diphosphate specific)
bin007	SOY3_bin007_01206	396	3	5	1	0.906	1.281	0.268	Cupin domain protein
bin007	SOY3_bin007_01207	369	1	5	5	0.324	1.374	1.439	hypothetical protein
bin007	SOY3_bin007_01208	372	1	7	4	0.321	1.909	1.142	hypothetical protein
bin007	SOY3_bin007_01209	1431	8	28	11	0.668	1.985	0.817	Nicotinate phosphoribosyltransferase pncB2
bin007	SOY3_bin007_01210	201	0	0	1	0.000	0.000	0.528	hypothetical protein
bin007	SOY3_bin007_01211	963	0	4	1	0.000	0.421	0.110	Beta-lactamase NDM-1 precursor
bin007	SOY3_bin007_01212	198	1	2	3	0.604	1.025	1.609	hypothetical protein
bin007	SOY3_bin007_01213	492	2	3	4	0.486	0.618	0.864	AP-4-A phosphorylase
bin007	SOY3_bin007_01214	378	0	9	6	0.000	2.415	1.686	hypothetical protein
bin007	SOY3_bin007_01215	612	0	1	0	0.000	0.166	0.000	hypothetical protein
bin007	SOY3_bin007_01216	480	0	1	2	0.000	0.211	0.443	hypothetical protein
bin007	SOY3_bin007_01217	77	4	6	2	6.210	7.903	2.759	tRNA-His(gtg)
bin007	SOY3_bin007_01218	75	0	4	1	0.000	5.409	1.416	tRNA-Gln(ttg)
bin007	SOY3_bin007_01219	76	0	0	1	0.000	0.000	1.398	tRNA-Lys(ttt)
bin007	SOY3_bin007_01220	85	1	0	0	1.406	0.000	0.000	tRNA-Leu(tag)
bin007	SOY3_bin007_01221	1008	17	72	31	2.016	7.245	3.267	molybdate ABC transporter periplasmic substrate-binding protein
bin007	SOY3_bin007_01222	792	1	4	2	0.151	0.512	0.268	Sulfate transport system permease protein CysT
bin007	SOY3_bin007_01223	1023	0	4	4	0.000	0.397	0.415	Sulfate/thiosulfate import ATP-binding protein CysA
bin007	SOY3_bin007_01224	1329	21	129	44	1.889	9.845	3.517	Trigger factor
bin007	SOY3_bin007_01225	591	33	97	31	6.675	16.647	5.572	ATP-dependent Clp protease proteolytic subunit precursor
bin007	SOY3_bin007_01226	1251	42	181	66	4.014	14.675	5.604	ATP-dependent Clp protease ATP-binding subunit ClpX
bin007	SOY3_bin007_01227	345	0	0	0	0.000	0.000	0.000	hypothetical protein
bin007	SOY3_bin007_01228	270	0	0	0	0.000	0.000	0.000	hypothetical protein
bin007	SOY3_bin007_01229	846	1	5	4	0.141	0.599	0.502	hypothetical protein
bin007	SOY3_bin007_01230	717	2	8	5	0.333	1.132	0.741	2-deoxyglucose-6-phosphate phosphatase
bin007	SOY3_bin007_01231	144	1	4	0	0.830	2.817	0.000	hypothetical protein
bin007	SOY3_bin007_01232	963	15	18	4	1.862	1.896	0.441	Anaerobic sulfite reductase subunit C

bin007	SOY3_bin007_01233	795	4	10	1	0.602	1.276	0.134	Anaerobic sulfite reductase subunit B
bin007	SOY3_bin007_01234	1023	8	7	2	0.935	0.694	0.208	Anaerobic sulfite reductase subunit A
bin007	SOY3_bin007_01235	192	0	0	0	0.000	0.000	0.000	hypothetical protein
bin007	SOY3_bin007_01236	1326	0	0	1	0.000	0.000	0.080	Phenylacetate-coenzyme A ligase
bin007	SOY3_bin007_01237	375	0	6	2	0.000	1.623	0.567	hypothetical protein
bin007	SOY3_bin007_01238	855	3	19	7	0.419	2.254	0.870	hypothetical protein
bin007	SOY3_bin007_01239	1134	2	11	4	0.211	0.984	0.375	Processive diacylglycerol beta-glucosyltransferase
bin007	SOY3_bin007_01240	1080	0	15	11	0.000	1.409	1.082	Arginine transport ATP-binding protein ArtM
bin007	SOY3_bin007_01241	543	1	11	8	0.220	2.055	1.565	putative glutamine ABC transporter permease protein GlnP
bin007	SOY3_bin007_01242	240	1	5	0	0.498	2.113	0.000	Double zinc ribbon
bin007	SOY3_bin007_01243	1140	4	28	12	0.419	2.491	1.118	Acyl-CoA dehydrogenase
bin007	SOY3_bin007_01244	795	2	14	3	0.301	1.786	0.401	Acryloyl-CoA reductase electron transfer subunit gamma
bin007	SOY3_bin007_01245	1008	4	27	5	0.474	2.717	0.527	Acryloyl-CoA reductase electron transfer subunit beta
bin007	SOY3_bin007_01246	990	0	5	4	0.000	0.512	0.429	putative metallo-hydrolase YflN
bin007	SOY3_bin007_01247	303	0	3	0	0.000	1.004	0.000	hypothetical protein
bin007	SOY3_bin007_01248	1374	0	3	0	0.000	0.221	0.000	Phenylacetate-coenzyme A ligase
bin007	SOY3_bin007_01249	996	1	8	1	0.120	0.815	0.107	Hydroxyacylglutathione hydrolase
bin007	SOY3_bin007_01250	1866	5	16	10	0.320	0.870	0.569	putative oxidoreductase YdhV
bin007	SOY3_bin007_01251	246	0	0	0	0.000	0.000	0.000	Pyruvate-flavodoxin oxidoreductase
bin007	SOY3_bin007_01252	1341	2	2	2	0.178	0.151	0.158	Propionyl-CoA:succinate CoA transferase
bin007	SOY3_bin007_01253	1155	1	5	1	0.104	0.439	0.092	Cysteine desulfurase
bin007	SOY3_bin007_01254	1149	0	3	1	0.000	0.265	0.092	putative xanthine dehydrogenase subunit A
bin007	SOY3_bin007_01255	2349	5	110	40	0.254	4.750	1.809	4-hydroxybenzoyl-CoA reductase subunit alpha
bin007	SOY3_bin007_01256	498	3	14	8	0.720	2.851	1.706	Carbon monoxide dehydrogenase small chain
bin007	SOY3_bin007_01257	867	2	34	11	0.276	3.978	1.348	Carbon monoxide dehydrogenase medium chain
bin007	SOY3_bin007_01258	957	1	2	1	0.125	0.212	0.111	HTH-type transcriptional regulator CynR
bin007	SOY3_bin007_01259	375	2	8	3	0.638	2.164	0.850	Chromosome partition protein Smc
bin007	SOY3_bin007_01260	267	10	34	5	4.477	12.916	1.989	hypothetical protein
bin007	SOY3_bin007_01261	75	1	1	0	1.594	1.352	0.000	tRNA-Val(gac)
bin007	SOY3_bin007_01262	1137	1	10	3	0.105	0.892	0.280	D-alanyl-D-alanine carboxypeptidase DacB precursor
bin007	SOY3_bin007_01263	747	2	6	2	0.320	0.815	0.284	Peptidoglycan-N-acetylglucosamine deacetylase
bin007	SOY3_bin007_01264	504	11	27	6	2.609	5.434	1.265	Lipoprotein YhcN precursor
bin007	SOY3_bin007_01265	609	13	19	14	2.552	3.164	2.442	Inner membrane protein YohD
bin007	SOY3_bin007_01266	1533	63	157	62	4.913	10.388	4.296	SEC-C motif protein
bin007	SOY3_bin007_01267	1503	8	31	8	0.636	2.092	0.565	putative CtpA-like serine protease
bin007	SOY3_bin007_01268	2715	11	27	6	0.484	1.009	0.235	DNA polymerase I
bin007	SOY3_bin007_01269	831	2	9	2	0.288	1.098	0.256	Formamidopyrimidine-DNA glycosylase
bin007	SOY3_bin007_01270	645	7	11	7	1.297	1.730	1.153	hypothetical protein
bin007	SOY3_bin007_01271	636	3	9	1	0.564	1.435	0.167	Dephospho-CoA kinase
bin007	SOY3_bin007_01272	582	3	9	3	0.616	1.568	0.548	Soluble lytic murein transglycosylase precursor
bin007	SOY3_bin007_01273	1479	8	29	18	0.647	1.989	1.293	Sodium/pantothenate symporter
bin007	SOY3_bin007_01274	189	1	3	3	0.633	1.610	1.686	hypothetical protein
bin007	SOY3_bin007_01275	645	3	7	2	0.556	1.101	0.329	Spore cortex-lytic enzyme precursor
bin007	SOY3_bin007_01276	720	3	10	7	0.498	1.409	1.033	phosphatidylserine decarboxylase
bin007	SOY3_bin007_01277	546	1	10	2	0.219	1.858	0.389	Phosphatidylcholine synthase
bin007	SOY3_bin007_01278	765	0	2	1	0.000	0.265	0.139	hypothetical protein
bin007	SOY3_bin007_01279	83	0	2	1	0.000	2.444	1.280	tRNA-Leu(gag)
bin007	SOY3_bin007_01280	1959	2	8	4	0.122	0.414	0.217	Lon protease
bin007	SOY3_bin007_01281	2589	3	12	6	0.139	0.470	0.246	Modification methylase MboII
bin007	SOY3_bin007_01282	2922	0	16	9	0.000	0.555	0.327	PglZ domain protein
bin007	SOY3_bin007_01283	4335	4	26	6	0.110	0.608	0.147	hypothetical protein
bin007	SOY3_bin007_01284	462	2	2	0	0.518	0.439	0.000	hypothetical protein
bin007	SOY3_bin007_01285	1206	2	6	1	0.198	0.505	0.088	hypothetical protein
bin007	SOY3_bin007_01286	3156	27	83	22	1.023	2.667	0.740	R-phenyllactate dehydratase activator
bin007	SOY3_bin007_01287	837	3	16	11	0.428	1.939	1.396	Ribonuclease
bin007	SOY3_bin007_01288	336	1	2	1	0.356	0.604	0.316	hypothetical protein
bin007	SOY3_bin007_01289	1161	6	16	8	0.618	1.398	0.732	putative MscS family protein YkuT
bin007	SOY3_bin007_01290	546	2	4	1	0.438	0.743	0.195	hypothetical protein
bin007	SOY3_bin007_01291	1119	21	60	24	2.244	5.438	2.278	Ferredoxin-1
bin007	SOY3_bin007_01292	717	7	46	16	1.167	6.507	2.370	Amidohydrolase
bin007	SOY3_bin007_01293	465	1	7	5	0.257	1.527	1.142	LysM domain/BON superfamily protein
bin007	SOY3_bin007_01294	333	3	13	4	1.077	3.960	1.276	hypothetical protein
bin007	SOY3_bin007_01295	1383	2	11	5	0.173	0.807	0.384	Ktr system potassium uptake protein B
bin007	SOY3_bin007_01296	987	5	15	7	0.606	1.541	0.753	Chaperone protein DnaJ
bin007	SOY3_bin007_01297	309	1	7	4	0.387	2.298	1.375	chaperone-modulator protein CbpM
bin007	SOY3_bin007_01298	1212	16	43	22	1.578	3.598	1.928	Molybdopterin molybdenumtransferase
bin007	SOY3_bin007_01299	1047	6	30	10	0.685	2.906	1.015	Phosphate acetyltransferase

bin007	SOY3_bin007_01300	2106	1	18	5	0.057	0.867	0.252	Succinyl-CoA ligase [ADP-forming] subunit alpha
bin007	SOY3_bin007_01301	1965	3	12	8	0.183	0.619	0.432	Acetoin dehydrogenase operon transcriptional activator AcoR
bin007	SOY3_bin007_01302	537	0	0	0	0.000	0.000	0.000	hypothetical protein
bin007	SOY3_bin007_01303	780	0	0	0	0.000	0.000	0.000	putative enoyl-CoA hydratase
bin007	SOY3_bin007_01304	846	0	0	0	0.000	0.000	0.000	putative 3-hydroxybutyryl-CoA dehydrogenase
bin007	SOY3_bin007_01305	1206	0	0	0	0.000	0.000	0.000	Acetyl-CoA acetyltransferase
bin007	SOY3_bin007_01306	765	0	0	0	0.000	0.000	0.000	2-dehydro-3-deoxy-D-gluconate 5-dehydrogenase
bin007	SOY3_bin007_01307	864	1	1	0	0.138	0.117	0.000	3-oxoadipate CoA-transferase subunit B
bin007	SOY3_bin007_01308	921	0	1	0	0.000	0.110	0.000	3-oxoadipate CoA-transferase subunit A
bin007	SOY3_bin007_01309	309	0	0	0	0.000	0.000	0.000	hypothetical protein
bin007	SOY3_bin007_01310	1440	1	4	2	0.083	0.282	0.148	4-hydroxybutyryl-CoA dehydratase/vinylacetyl-CoA-Delta-isomerase
bin007	SOY3_bin007_01311	573	7	34	4	1.460	6.018	0.742	Rubrerythrin
bin007	SOY3_bin007_01312	465	2	2	1	0.514	0.436	0.228	hypothetical protein
bin007	SOY3_bin007_01313	165	1	17	3	0.725	10.450	1.931	hypothetical protein
bin007	SOY3_bin007_01314	804	9	63	19	1.338	7.948	2.510	(-)-trans-carveol dehydrogenase
bin007	SOY3_bin007_01315	2073	38	59	7	2.191	2.887	0.359	Thiol:disulfide interchange protein DsbD
bin007	SOY3_bin007_01316	1272	22	96	15	2.068	7.655	1.253	Glutamate 2,3-aminomutase
bin007	SOY3_bin007_01317	207	0	0	0	0.000	0.000	0.000	hypothetical protein
bin007	SOY3_bin007_01318	567	2	22	4	0.422	3.935	0.749	Flavoredoxin
bin007	SOY3_bin007_01319	558	9	14	8	1.928	2.545	1.523	O-acetyl-ADP-ribose deacetylase
bin007	SOY3_bin007_01320	951	49	57	18	6.160	6.079	2.011	Diflavin flavoprotein A 1
bin007	SOY3_bin007_01321	1314	30	56	13	2.729	4.323	1.051	Sensor histidine kinase YehU
bin007	SOY3_bin007_01322	789	16	23	10	2.424	2.957	1.346	Sensory transduction protein LytR
bin007	SOY3_bin007_01323	408	12	35	17	3.516	8.701	4.426	Holo-[acyl-carrier-protein] synthase
bin007	SOY3_bin007_01324	1548	23	103	40	1.776	6.749	2.745	Bifunctional NAD(P)H-hydrate repair enzyme Nnr
bin007	SOY3_bin007_01325	1119	37	60	23	3.953	5.438	2.183	Alanine dehydrogenase
bin007	SOY3_bin007_01326	300	6	9	5	2.391	3.043	1.770	hypothetical protein
bin007	SOY3_bin007_01327	1137	38	110	36	3.995	9.813	3.363	Alanine racemase
bin007	SOY3_bin007_01328	537	0	0	2	0.000	0.000	0.396	ECF RNA polymerase sigma factor SigW
bin007	SOY3_bin007_01329	1233	5	10	13	0.485	0.823	1.120	Anti-sigma-W factor RsiW
bin007	SOY3_bin007_01330	1131	15	31	14	1.586	2.780	1.315	hypothetical protein
bin007	SOY3_bin007_01331	1449	28	83	37	2.310	5.810	2.712	Glutamine synthetase
bin007	SOY3_bin007_01332	501	8	28	9	1.909	5.669	1.908	Putative acetolactate synthase small subunit
bin007	SOY3_bin007_01333	276	5	26	8	2.166	9.555	3.079	Antitoxin EndoAI
bin007	SOY3_bin007_01334	351	30	112	41	10.218	32.364	12.408	mRNA interferase EndoA
bin007	SOY3_bin007_01335	714	1	2	0	0.167	0.284	0.000	Putative glutamine amidotransferase
bin007	SOY3_bin007_01336	1203	6	18	10	0.596	1.518	0.883	Imidazolonepropionase
bin007	SOY3_bin007_01337	402	3	21	12	0.892	5.298	3.171	Sporulation initiation phosphotransferase F
bin007	SOY3_bin007_01338	480	4	16	6	0.996	3.381	1.328	tRNA threonylcarbamoyladenosine biosynthesis protein TsaE
bin007	SOY3_bin007_01339	723	6	17	11	0.992	2.385	1.616	tRNA threonylcarbamoyladenosine biosynthesis protein TsaB
bin007	SOY3_bin007_01340	486	1	9	5	0.246	1.878	1.093	L-2,4-diaminobutyric acid acetyltransferase
bin007	SOY3_bin007_01341	147	0	6	3	0.000	4.140	2.168	hypothetical protein
bin007	SOY3_bin007_01342	540	8	16	6	1.771	3.005	1.180	ECF RNA polymerase sigma factor SigW
bin007	SOY3_bin007_01343	1140	12	23	6	1.258	2.046	0.559	hypothetical protein
bin007	SOY3_bin007_01344	915	5	7	3	0.653	0.776	0.348	putative ABC transporter ATP-binding protein YxlF
bin007	SOY3_bin007_01345	825	2	4	5	0.290	0.492	0.644	ABC-2 family transporter protein
bin007	SOY3_bin007_01346	75	2	5	1	3.188	6.762	1.416	tRNA-Arg(ccg)
bin007	SOY3_bin007_01347	498	5	21	7	1.200	4.277	1.493	chromosome segregation protein
bin007	SOY3_bin007_01348	1056	0	0	3	0.000	0.000	0.302	Glutamate synthase [NADPH] small chain
bin007	SOY3_bin007_01349	1125	0	5	1	0.000	0.451	0.094	Spore germination protein YndE
bin007	SOY3_bin007_01350	912	3	20	4	0.393	2.224	0.466	Signal recognition particle receptor FtsY
bin007	SOY3_bin007_01351	723	3	22	6	0.496	3.086	0.882	Ribonuclease 3
bin007	SOY3_bin007_01352	1236	8	43	14	0.774	3.529	1.203	3-oxoacyl-[acyl-carrier-protein] synthase 2
bin007	SOY3_bin007_01353	231	5	32	14	2.588	14.051	6.438	Acyl carrier protein
bin007	SOY3_bin007_01354	747	17	56	32	2.721	7.604	4.551	3-oxoacyl-[acyl-carrier-protein] reductase FabG
bin007	SOY3_bin007_01355	942	7	29	7	0.888	3.122	0.789	Malonyl CoA-acyl carrier protein transacylase
bin007	SOY3_bin007_01356	951	11	29	16	1.383	3.093	1.787	Nitronate monooxygenase
bin007	SOY3_bin007_01357	993	12	31	13	1.445	3.166	1.391	3-oxoacyl-[acyl-carrier-protein] synthase 3
bin007	SOY3_bin007_01358	1032	7	27	15	0.811	2.654	1.544	Phosphate acyltransferase
bin007	SOY3_bin007_01359	579	3	12	8	0.619	2.102	1.468	Transcription factor FapR
bin007	SOY3_bin007_01360	180	10	71	36	6.642	40.007	21.245	50S ribosomal protein L32
bin007	SOY3_bin007_01361	540	4	34	6	0.886	6.386	1.180	hypothetical protein
bin007	SOY3_bin007_01362	585	0	5	2	0.000	0.867	0.363	Spore germination protein GerM
bin007	SOY3_bin007_01363	1266	5	13	6	0.472	1.042	0.503	Sporulation integral membrane protein YlbJ
bin007	SOY3_bin007_01364	1305	2	12	3	0.183	0.933	0.244	putative sulfoacetate transporter SauU
bin007	SOY3_bin007_01365	2103	10	26	9	0.568	1.254	0.455	Succinyl-CoA ligase [ADP-forming] subunit beta
bin007	SOY3_bin007_01366	1053	3	12	2	0.341	1.156	0.202	Phosphate acetyltransferase

bin007	SOY3_bin007_01367	447	20	61	24	5.349	13.841	5.703	hypothetical protein
bin007	SOY3_bin007_01368	483	12	51	17	2.970	10.710	3.739	Phosphopantetheine adenylyltransferase
bin007	SOY3_bin007_01369	516	29	71	20	6.719	13.956	4.117	Ribosomal RNA small subunit methyltransferase D
bin007	SOY3_bin007_01370	984	4	15	6	0.486	1.546	0.648	Germination protease precursor
bin007	SOY3_bin007_01371	258	40	119	42	18.535	46.782	17.293	Small, acid-soluble spore protein C2
bin007	SOY3_bin007_01372	192	28	116	53	17.434	61.279	29.323	50S ribosomal protein L28
bin007	SOY3_bin007_01373	471	6	18	13	1.523	3.876	2.932	6,7-dimethyl-8-ribityllumazine synthase
bin007	SOY3_bin007_01374	1209	7	40	11	0.692	3.356	0.966	Riboflavin biosynthesis protein RibBA
bin007	SOY3_bin007_01375	651	1	18	5	0.184	2.804	0.816	Riboflavin synthase
bin007	SOY3_bin007_01376	1113	6	36	11	0.644	3.281	1.050	Riboflavin biosynthesis protein RibD
bin007	SOY3_bin007_01377	414	0	10	5	0.000	2.450	1.283	HTH-type transcriptional regulator FrlR
bin007	SOY3_bin007_01378	2403	3	11	1	0.149	0.464	0.044	Beta propeller domain protein
bin007	SOY3_bin007_01379	1296	16	38	22	1.476	2.974	1.803	Bis(5'-nucleosyl)-tetraphosphatase PrpE [asymmetrical]
bin007	SOY3_bin007_01380	939	11	39	23	1.400	4.213	2.602	1-deoxy-D-xylulose-5-phosphate synthase
bin007	SOY3_bin007_01381	864	6	21	16	0.830	2.465	1.967	Transketolase 2
bin007	SOY3_bin007_01382	840	10	35	14	1.423	4.226	1.770	hypothetical protein
bin007	SOY3_bin007_01383	993	29	88	21	3.491	8.989	2.246	Peptide chain release factor 2
bin007	SOY3_bin007_01384	2664	57	139	37	2.558	5.292	1.475	preprotein translocase subunit SecA
bin007	SOY3_bin007_01385	525	17	47	22	3.871	9.080	4.451	Putative sigma-54 modulation protein
bin007	SOY3_bin007_01386	198	10	24	12	6.038	12.294	6.438	Cold shock protein CspB
bin007	SOY3_bin007_01387	756	4	8	4	0.633	1.073	0.562	DNA utilization protein GntX
bin007	SOY3_bin007_01388	1704	4	14	7	0.281	0.833	0.436	Phytochrome-like protein cph2
bin007	SOY3_bin007_01389	429	1	1	2	0.279	0.236	0.495	hypothetical protein
bin007	SOY3_bin007_01390	1191	24	62	35	2.409	5.280	3.122	S-adenosylmethionine synthase
bin007	SOY3_bin007_01391	234	1	5	3	0.511	2.167	1.362	PRC-barrel domain protein
bin007	SOY3_bin007_01392	1560	9	27	17	0.690	1.755	1.158	putative peptidoglycan biosynthesis protein MurJ
bin007	SOY3_bin007_01393	1428	16	52	21	1.339	3.693	1.562	Phosphoglucomutase
bin007	SOY3_bin007_01394	150	2	1	0	1.594	0.676	0.000	hypothetical protein
bin007	SOY3_bin007_01395	114	0	1	2	0.000	0.890	1.864	hypothetical protein
bin007	SOY3_bin007_01396	762	6	24	4	0.941	3.195	0.558	AIG2-like family protein
bin007	SOY3_bin007_01397	924	9	74	17	1.164	8.123	1.954	UDP-glucose 4-epimerase
bin007	SOY3_bin007_01398	894	5	46	17	0.669	5.219	2.020	UTP--glucose-1-phosphate uridylyltransferase
bin007	SOY3_bin007_01399	1077	30	111	39	3.330	10.454	3.847	putative undecaprenyl-phosphate N-acetylglucosaminyl 1-phosphate transferase
bin007	SOY3_bin007_01400	426	3	16	8	0.842	3.809	1.995	3-hydroxyacyl-[acyl-carrier-protein] dehydratase FabZ
bin007	SOY3_bin007_01401	480	7	39	19	1.743	8.241	4.205	hypothetical protein
bin007	SOY3_bin007_01402	1173	15	69	28	1.529	5.966	2.536	Putative teichuronic acid biosynthesis glycosyltransferase TuaH
bin007	SOY3_bin007_01403	1134	12	36	13	1.265	3.220	1.218	Alpha-D-kanosaminyltransferase
bin007	SOY3_bin007_01404	1524	8	84	21	0.628	5.590	1.464	O-Antigen ligase
bin007	SOY3_bin007_01405	1500	59	93	38	4.702	6.288	2.691	Putative transcriptional regulator YwtF
bin007	SOY3_bin007_01406	423	9	25	11	2.544	5.995	2.762	hypothetical protein
bin007	SOY3_bin007_01407	312	0	8	2	0.000	2.601	0.681	colanic acid biosynthesis protein
bin007	SOY3_bin007_01408	1815	14	52	15	0.922	2.906	0.878	Putative thiamine pyrophosphate-containing protein YdaP
bin007	SOY3_bin007_01409	591	5	15	6	1.011	2.574	1.078	NADH-dependent phenylglyoxylate dehydrogenase subunit gamma
bin007	SOY3_bin007_01410	1281	9	47	11	0.840	3.721	0.912	Phenylacetate-coenzyme A ligase
bin007	SOY3_bin007_01411	1680	12	48	21	0.854	2.898	1.328	Sporulation kinase E
bin007	SOY3_bin007_01412	546	1	13	3	0.219	2.415	0.584	putative isochorismatase
bin007	SOY3_bin007_01413	528	2	10	7	0.453	1.921	1.408	hypothetical protein
bin007	SOY3_bin007_01414	4356	89	156	61	2.443	3.632	1.488	ATP-dependent helicase/nuclease subunit A
bin007	SOY3_bin007_01415	3474	36	78	23	1.239	2.277	0.703	ATP-dependent helicase/deoxyribonuclease subunit B
bin007	SOY3_bin007_01416	360	23	52	17	7.638	14.651	5.016	hypothetical protein
bin007	SOY3_bin007_01417	798	14	49	9	2.097	6.228	1.198	Calcineurin-like phosphoesterase superfamily domain protein
bin007	SOY3_bin007_01418	477	0	2	0	0.000	0.425	0.000	hypothetical protein
bin007	SOY3_bin007_01419	1230	0	0	0	0.000	0.000	0.000	Anti-sigma-I factor RsgI
bin007	SOY3_bin007_01420	735	1	4	0	0.163	0.552	0.000	RNA polymerase sigma factor SigI
bin007	SOY3_bin007_01421	1137	21	73	33	2.208	6.512	3.083	Virulence sensor protein BvgS precursor
bin007	SOY3_bin007_01422	864	2	11	2	0.277	1.291	0.246	hypothetical protein
bin007	SOY3_bin007_01423	114	9	7	1	9.438	6.228	0.932	hypothetical protein
bin007	SOY3_bin007_01424	117	0	4	2	0.000	3.468	1.816	hypothetical protein
bin007	SOY3_bin007_01425	504	4	17	7	0.949	3.421	1.475	Ribonuclease HI
bin007	SOY3_bin007_01426	183	0	0	0	0.000	0.000	0.000	hypothetical protein
bin007	SOY3_bin007_01427	1578	4	21	5	0.303	1.350	0.337	Competence protein ComM
bin007	SOY3_bin007_01428	267	0	3	0	0.000	1.140	0.000	NADP-reducing hydrogenase subunit HndA
bin007	SOY3_bin007_01429	171	0	1	0	0.000	0.593	0.000	hypothetical protein
bin007	SOY3_bin007_01430	1032	4	64	14	0.463	6.290	1.441	Iron deficiency-induced protein A precursor
bin007	SOY3_bin007_01431	621	0	18	11	0.000	2.940	1.882	DNA polymerase III PolC
bin007	SOY3_bin007_01432	153	0	0	0	0.000	0.000	0.000	hypothetical protein
bin007	SOY3_bin007_01433	2184	40	19	14	2.190	0.882	0.681	Catalase-peroxidase

bin007	SOY3_bin007_01434	423	1	0	2	0.283	0.000	0.502	Peroxide-responsive repressor PerR
bin007	SOY3_bin007_01435	1443	12	53	27	0.994	3.725	1.988	Aspartyl/glutamyl-tRNA(Asn/Gln) amidotransferase subunit B
bin007	SOY3_bin007_01436	1440	9	34	11	0.747	2.395	0.811	Glutamyl-tRNA(Gln) amidotransferase subunit A
bin007	SOY3_bin007_01437	285	5	12	4	2.097	4.271	1.491	Aspartyl/glutamyl-tRNA(Asn/Gln) amidotransferase subunit C
bin007	SOY3_bin007_01438	465	0	5	4	0.000	1.091	0.914	hypothetical protein
bin007	SOY3_bin007_01439	402	0	2	0	0.000	0.505	0.000	MoaE protein
bin007	SOY3_bin007_01440	327	0	0	0	0.000	0.000	0.000	hypothetical protein
bin007	SOY3_bin007_01441	1017	0	1	0	0.000	0.100	0.000	putative ABC transporter ATP-binding protein YbhF
bin007	SOY3_bin007_01442	810	0	2	0	0.000	0.250	0.000	hypothetical protein
bin007	SOY3_bin007_01443	804	0	5	1	0.000	0.631	0.132	hypothetical protein
bin007	SOY3_bin007_01444	900	2	7	2	0.266	0.789	0.236	putative inner membrane transporter YicL
bin007	SOY3_bin007_01445	723	3	7	3	0.496	0.982	0.441	Arginine transport ATP-binding protein ArtM
bin007	SOY3_bin007_01446	678	1	3	1	0.176	0.449	0.157	Inner membrane amino-acid ABC transporter permease protein YecS
bin007	SOY3_bin007_01447	801	30	82	32	4.477	10.383	4.244	Glutamine-binding periplasmic protein precursor
bin007	SOY3_bin007_01448	867	0	7	3	0.000	0.819	0.368	putative inner membrane transporter yiJE
bin007	SOY3_bin007_01449	720	0	14	2	0.000	1.972	0.295	N-(5'-phosphoribosyl)anthranilate isomerase
bin007	SOY3_bin007_01450	708	1	11	1	0.169	1.576	0.150	Indole-3-glycerol phosphate synthase
bin007	SOY3_bin007_01451	1086	0	13	5	0.000	1.214	0.489	Anthranilate phosphoribosyltransferase
bin007	SOY3_bin007_01452	831	1	6	2	0.144	0.732	0.256	Tryptophan synthase alpha chain
bin007	SOY3_bin007_01453	1269	0	21	6	0.000	1.678	0.502	Tryptophan synthase beta chain
bin007	SOY3_bin007_01454	450	1	8	1	0.266	1.803	0.236	Guanine deaminase
bin007	SOY3_bin007_01455	294	0	0	0	0.000	0.000	0.000	hypothetical protein
bin007	SOY3_bin007_01456	267	3	7	3	1.343	2.659	1.194	hypothetical protein
bin007	SOY3_bin007_01457	1185	14	91	34	1.412	7.789	3.048	Leucine-, isoleucine-, valine-, threonine-, and alanine-binding protein precursor
bin007	SOY3_bin007_01458	882	1	7	6	0.136	0.805	0.723	High-affinity branched-chain amino acid transport system permease protein LivH
bin007	SOY3_bin007_01459	969	2	9	5	0.247	0.942	0.548	High-affinity branched-chain amino acid transport system permease protein LivH
bin007	SOY3_bin007_01460	768	2	4	3	0.311	0.528	0.415	Lipopolysaccharide export system ATP-binding protein LptB
bin007	SOY3_bin007_01461	705	0	8	2	0.000	1.151	0.301	High-affinity branched-chain amino acid transport ATP-binding protein LivF
bin007	SOY3_bin007_01462	663	3	9	6	0.541	1.377	0.961	Hypoxic response protein 1
bin007	SOY3_bin007_01463	411	2	30	13	0.582	7.403	3.360	hypothetical protein
bin007	SOY3_bin007_01464	1125	19	116	52	2.019	10.458	4.910	Glutamine--fructose-6-phosphate aminotransferase [isomerizing]
bin007	SOY3_bin007_01465	1338	20	97	32	1.787	7.353	2.541	Phosphoglucosamine mutase
bin007	SOY3_bin007_01466	969	1	7	1	0.123	0.733	0.110	YbbR-like protein
bin007	SOY3_bin007_01467	834	2	11	1	0.287	1.338	0.127	DNA integrity scanning protein DisA
bin007	SOY3_bin007_01468	1590	26	82	42	1.955	5.231	2.806	2-isopropylmalate synthase
bin007	SOY3_bin007_01469	1080	10	34	13	1.107	3.193	1.279	3-isopropylmalate dehydrogenase
bin007	SOY3_bin007_01470	501	4	20	4	0.954	4.049	0.848	2,3-dimethylmalate dehydratase small subunit
bin007	SOY3_bin007_01471	1263	10	84	20	0.947	6.746	1.682	2,3-dimethylmalate dehydratase large subunit
bin007	SOY3_bin007_01472	1545	13	65	24	1.006	4.267	1.650	2-isopropylmalate synthase
bin007	SOY3_bin007_01473	1662	11	71	34	0.791	4.333	2.173	Acetolactate synthase large subunit
bin007	SOY3_bin007_01474	993	13	69	28	1.565	7.048	2.995	Ketol-acid reductoisomerase
bin007	SOY3_bin007_01475	630	3	22	9	0.569	3.542	1.518	Putative acetolactate synthase small subunit
bin007	SOY3_bin007_01476	1737	17	104	39	1.170	6.073	2.385	Acetolactate synthase large subunit
bin007	SOY3_bin007_01477	873	6	32	10	0.822	3.718	1.217	Branched-chain-amino-acid aminotransferase
bin007	SOY3_bin007_01478	351	8	6	6	2.725	1.734	1.816	hypothetical protein
bin007	SOY3_bin007_01479	87	3	9	9	4.122	10.492	10.989	tRNA-Ser(cag)
bin007	SOY3_bin007_01480	147	0	0	2	0.000	0.000	1.445	hypothetical protein
bin007	SOY3_bin007_01481	930	3	19	5	0.386	2.072	0.571	Putative serine protease HtrA
bin007	SOY3_bin007_01482	876	2	17	4	0.273	1.968	0.485	hypothetical protein
bin007	SOY3_bin007_01483	789	9	20	5	1.364	2.571	0.673	1,2-epoxyphenylacetyl-CoA isomerase
bin007	SOY3_bin007_01484	804	5	29	3	0.743	3.658	0.396	Hydroxymethylpyrimidine/phosphomethylpyrimidine kinase
bin007	SOY3_bin007_01485	801	3	20	5	0.448	2.533	0.663	Hydroxyethylthiazole kinase
bin007	SOY3_bin007_01486	636	1	12	2	0.188	1.914	0.334	Thiamine-phosphate synthase
bin007	SOY3_bin007_01487	636	1	9	2	0.188	1.435	0.334	2-hydroxy-3-keto-5-methylthiopentenyl-1-phosphate phosphatase
bin007	SOY3_bin007_01488	76	0	1	0	0.000	1.335	0.000	tRNA-Lys(ctt)
bin007	SOY3_bin007_01489	76	1	6	2	1.573	8.007	2.795	tRNA-Gln(ctg)
bin007	SOY3_bin007_01490	76	4	5	7	6.292	6.673	9.784	tRNA-Glu(ttc)
bin007	SOY3_bin007_01491	78	5	19	4	7.663	24.707	5.447	tRNA-Pro(tgg)
bin007	SOY3_bin007_01492	1548	15	24	12	1.158	1.573	0.823	Transcriptional regulatory protein ZraR
bin007	SOY3_bin007_01493	1164	138	1322	584	14.173	115.195	53.295	Aldehyde-alcohol dehydrogenase
bin007	SOY3_bin007_01494	1164	138	1361	538	14.173	118.593	49.097	1,3-propanediol dehydrogenase
bin007	SOY3_bin007_01495	732	5	29	7	0.817	4.018	1.016	CobQ/CobB/MinD/ParA nucleotide binding domain protein
bin007	SOY3_bin007_01496	1242	7	53	19	0.674	4.328	1.625	Anaerobic glycerol-3-phosphate dehydrogenase subunit C
bin007	SOY3_bin007_01497	3027	24	148	52	0.948	4.959	1.825	NADH-dependent phenylglyoxylate dehydrogenase subunit delta
bin007	SOY3_bin007_01498	414	1	11	2	0.289	2.695	0.513	Methyl-viologen-reducing hydrogenase, delta subunit
bin007	SOY3_bin007_01499	669	3	19	7	0.536	2.881	1.111	hypothetical protein
bin007	SOY3_bin007_01500	921	4	28	14	0.519	3.084	1.615	Bifunctional homocysteine S-methyltransferase/5,10-methylenetetrahydrofolate reductase

bin007	SOY3_bin007_01501	1017	1	9	0	0.118	0.898	0.000	Glyceraldehyde-3-phosphate dehydrogenase
bin007	SOY3_bin007_01502	1548	0	8	5	0.000	0.524	0.343	Phosphoenolpyruvate carboxykinase [ATP]
bin007	SOY3_bin007_01503	309	1	2	0	0.387	0.656	0.000	hypothetical protein
bin007	SOY3_bin007_01504	732	0	5	2	0.000	0.693	0.290	General stress protein 16O
bin007	SOY3_bin007_01505	480	10	20	5	2.491	4.226	1.107	hypothetical protein
bin007	SOY3_bin007_01506	963	0	5	1	0.000	0.527	0.110	Ferrous-iron efflux pump FieF
bin007	SOY3_bin007_01507	276	1	2	0	0.433	0.735	0.000	TFIIE alpha subunit
bin007	SOY3_bin007_01508	2634	44	136	49	1.997	5.237	1.976	Single-stranded-DNA-specific exonuclease RecJ
bin007	SOY3_bin007_01509	519	2	7	6	0.461	1.368	1.228	Adenine phosphoribosyltransferase
bin007	SOY3_bin007_01510	2307	15	37	15	0.777	1.627	0.691	GTP pyrophosphokinase
bin007	SOY3_bin007_01511	450	2	14	5	0.531	3.156	1.180	D-tyrosyl-tRNA(Tyr) deacylase
bin007	SOY3_bin007_01512	624	20	41	22	3.832	6.664	3.745	putative metallo-hydrolase
bin007	SOY3_bin007_01513	408	7	43	9	2.051	10.690	2.343	hypothetical protein
bin007	SOY3_bin007_01514	1275	3	12	4	0.281	0.955	0.333	Histidine--tRNA ligase
bin007	SOY3_bin007_01515	1845	10	48	18	0.648	2.639	1.036	Aspartate--tRNA ligase
bin007	SOY3_bin007_01516	228	2303	5927	2771	1207.549	2636.664	1291.016	hypothetical protein
bin007	SOY3_bin007_01517	759	0	1	0	0.000	0.134	0.000	tRNA threonylcarbamoyladenosine dehydratase
bin007	SOY3_bin007_01518	330	18	119	59	6.521	36.575	18.992	Thioredoxin
bin007	SOY3_bin007_01519	1320	3	8	3	0.272	0.615	0.241	Replication-associated recombination protein A
bin007	SOY3_bin007_01520	429	10	43	21	2.787	10.166	5.200	HTH-type transcriptional regulator CymR
bin007	SOY3_bin007_01521	372	3	19	7	0.964	5.180	1.999	NifU-like protein
bin007	SOY3_bin007_01522	1086	6	43	17	0.660	4.016	1.663	tRNA-specific 2-thiouridylase MnmA
bin007	SOY3_bin007_01523	174	3	8	2	2.061	4.663	1.221	hypothetical protein
bin007	SOY3_bin007_01524	192	5	14	6	3.113	7.396	3.320	hypothetical protein
bin007	SOY3_bin007_01525	1041	0	4	0	0.000	0.390	0.000	AI-2 transport protein TqsA
bin007	SOY3_bin007_01526	291	2	5	0	0.822	1.743	0.000	hypothetical protein
bin007	SOY3_bin007_01527	360	2	7	1	0.664	1.972	0.295	hypothetical protein
bin007	SOY3_bin007_01528	255	3	3	0	1.406	1.193	0.000	hypothetical protein
bin007	SOY3_bin007_01529	297	1	29	14	0.403	9.904	5.007	Putative anti-sigma factor antagonist BtrV
bin007	SOY3_bin007_01530	1722	19	77	24	1.319	4.535	1.480	Multidrug export protein MepA
bin007	SOY3_bin007_01531	870	7	21	15	0.962	2.448	1.831	tRNA (mo5U34)-methyltransferase
bin007	SOY3_bin007_01532	603	6	13	8	1.190	2.187	1.409	hypothetical protein
bin007	SOY3_bin007_01533	2022	2	20	13	0.118	1.003	0.683	Phosphoserine phosphatase RsbU
bin007	SOY3_bin007_01534	375	1	3	1	0.319	0.811	0.283	hypothetical protein
bin007	SOY3_bin007_01535	4830	1	20	6	0.025	0.420	0.132	ATP-dependent DNA helicase RecQ
bin007	SOY3_bin007_01536	150	4	11	4	3.188	7.438	2.833	hypothetical protein
bin007	SOY3_bin007_01537	1032	10	33	12	1.158	3.243	1.235	Methionine import ATP-binding protein MetN
bin007	SOY3_bin007_01538	678	11	47	12	1.940	7.031	1.880	D-methionine transport system permease protein MetI
bin007	SOY3_bin007_01539	831	17	61	31	2.446	7.445	3.963	Methionine-binding lipoprotein MetQ precursor
bin007	SOY3_bin007_01540	453	5	23	8	1.320	5.150	1.876	hypothetical protein
bin007	SOY3_bin007_01541	681	3	20	5	0.527	2.979	0.780	hypothetical protein
bin007	SOY3_bin007_01542	1002	9	42	11	1.074	4.251	1.166	hypothetical protein
bin007	SOY3_bin007_01543	894	4	40	9	0.535	4.538	1.069	23S rRNA (guanine(745)-N(1))-methyltransferase
bin007	SOY3_bin007_01544	228	4	9	7	2.097	4.004	3.261	hypothetical protein
bin007	SOY3_bin007_01545	1035	0	27	14	0.000	2.646	1.437	putative ABC transporter permease protein
bin007	SOY3_bin007_01546	486	1	5	1	0.246	1.043	0.219	Mercuric resistance operon regulatory protein
bin007	SOY3_bin007_01547	2433	6	33	9	0.295	1.376	0.393	Vault protein inter-alpha-trypsin
bin007	SOY3_bin007_01548	261	0	2	0	0.000	0.777	0.000	hypothetical protein
bin007	SOY3_bin007_01549	1077	11	30	18	1.221	2.825	1.775	Putative ribosome biogenesis GTPase RsgA
bin007	SOY3_bin007_01550	3192	1	16	9	0.037	0.508	0.300	Sporulation kinase E
bin007	SOY3_bin007_01551	3060	8	11	5	0.313	0.365	0.174	hypothetical protein
bin007	SOY3_bin007_01552	546	2	4	1	0.438	0.743	0.195	hypothetical protein
bin007	SOY3_bin007_01553	2871	1	6	1	0.042	0.212	0.037	hypothetical protein
bin007	SOY3_bin007_01554	2757	1	11	1	0.043	0.405	0.039	RNA polymerase-associated protein RapA
bin007	SOY3_bin007_01555	765	1	13	3	0.156	1.724	0.417	Uracil DNA glycosylase superfamily protein
bin007	SOY3_bin007_01556	441	0	7	2	0.000	1.610	0.482	hypothetical protein
bin007	SOY3_bin007_01557	720	0	5	2	0.000	0.704	0.295	hypothetical protein
bin007	SOY3_bin007_01558	1044	1	1	1	0.115	0.097	0.102	Histone deacetylase-like amidohydrolase
bin007	SOY3_bin007_01559	846	7	8	1	0.989	0.959	0.126	Protease 4
bin007	SOY3_bin007_01560	258	0	2	0	0.000	0.786	0.000	hypothetical protein
bin007	SOY3_bin007_01561	1821	0	6	0	0.000	0.334	0.000	ABC transporter permease YtrF precursor
bin007	SOY3_bin007_01562	114	0	0	0	0.000	0.000	0.000	hypothetical protein
bin007	SOY3_bin007_01563	216	0	5	1	0.000	2.348	0.492	hypothetical protein
bin007	SOY3_bin007_01564	771	1	19	8	0.155	2.500	1.102	Bicarbonate transport ATP-binding protein CmpD
bin007	SOY3_bin007_01565	837	2	26	11	0.286	3.151	1.396	Putative aliphatic sulfonates transport permease protein SsuC
bin007	SOY3_bin007_01566	1107	5	113	32	0.540	10.353	3.071	Nitrate transport protein NrtA precursor
bin007	SOY3_bin007_01567	747	6	97	23	0.960	13.171	3.271	Phosphate import ATP-binding protein PstB 3

bin007	SOY3_bin007_01568	846	7	108	25	0.989	12.948	3.139	Phosphate transport system permease protein PstA
bin007	SOY3_bin007_01569	870	13	152	55	1.786	17.721	6.715	Phosphate transport system permease protein PstC
bin007	SOY3_bin007_01570	903	24	272	90	3.177	30.552	10.587	Phosphate-binding protein PstS precursor
bin007	SOY3_bin007_01571	1278	30	488	151	2.806	38.730	12.551	R-phenyllactate dehydratase beta subunit
bin007	SOY3_bin007_01572	840	4	51	16	0.569	6.158	2.023	R-phenyllactate dehydratase activator
bin007	SOY3_bin007_01573	765	3	16	7	0.469	2.121	0.972	Aliphatic sulfonates import ATP-binding protein SsuB
bin007	SOY3_bin007_01574	795	1	10	4	0.150	1.276	0.534	Putative aliphatic sulfonates transport permease protein SsuC
bin007	SOY3_bin007_01575	966	0	16	2	0.000	1.680	0.220	Putative aliphatic sulfonates-binding protein precursor
bin007	SOY3_bin007_01576	1458	0	2	0	0.000	0.139	0.000	Biotin synthase
bin007	SOY3_bin007_01577	300	12	20	11	4.782	6.762	3.895	hypothetical protein
bin007	SOY3_bin007_01578	1374	23	52	18	2.001	3.839	1.392	NADH peroxidase
bin007	SOY3_bin007_01579	444	6	17	6	1.616	3.883	1.435	hypothetical protein
bin007	SOY3_bin007_01580	327	12	15	6	4.387	4.653	1.949	hypothetical protein
bin007	SOY3_bin007_01581	432	8	27	8	2.214	6.339	1.967	Large-conductance mechanosensitive channel
bin007	SOY3_bin007_01582	1764	5	9	5	0.339	0.517	0.301	Maltodextrin phosphorylase
bin007	SOY3_bin007_01583	507	2	3	0	0.472	0.600	0.000	putative lysine decarboxylase
bin007	SOY3_bin007_01584	243	21	72	20	10.331	30.053	8.743	hypothetical protein
bin007	SOY3_bin007_01585	1953	0	10	2	0.000	0.519	0.109	Spore germination protein B1
bin007	SOY3_bin007_01586	1098	1	3	2	0.109	0.277	0.193	Spore germination protein YndE
bin007	SOY3_bin007_01587	1281	1	2	2	0.093	0.158	0.166	Spore germination protein B3 precursor
bin007	SOY3_bin007_01588	318	0	0	1	0.000	0.000	0.334	hypothetical protein
bin007	SOY3_bin007_01589	276	8	13	5	3.465	4.777	1.924	Transposase zinc-ribbon domain protein
bin007	SOY3_bin007_01590	1467	10	66	26	0.815	4.563	1.883	Long-chain-fatty-acid--CoA ligase
bin007	SOY3_bin007_01591	1401	2	4	1	0.171	0.290	0.076	DNA recombination protein RmuC
bin007	SOY3_bin007_01592	432	6	12	5	1.660	2.817	1.229	Peptidyl-prolyl cis-trans isomerase B
bin007	SOY3_bin007_01593	552	1	48	10	0.217	8.820	1.924	lineage-specific thermal regulator protein
bin007	SOY3_bin007_01594	561	4	31	6	0.852	5.605	1.136	Acyl-coenzyme A thioesterase PaaI
bin007	SOY3_bin007_01595	102	1	0	0	1.172	0.000	0.000	hypothetical protein
bin007	SOY3_bin007_01596	1044	18	66	33	2.061	6.412	3.358	Molybdopterin molybdenumtransferase
bin007	SOY3_bin007_01597	828	22	61	23	3.176	7.472	2.951	hypothetical protein
bin007	SOY3_bin007_01598	750	7	20	4	1.116	2.705	0.567	hypothetical protein
bin007	SOY3_bin007_01599	516	0	8	1	0.000	1.573	0.206	Nicotine blue oxidoreductase
bin007	SOY3_bin007_01600	1563	2	47	15	0.153	3.050	1.019	Acetate CoA-transferase YdiF
bin007	SOY3_bin007_01601	300	0	3	2	0.000	1.014	0.708	hypothetical protein
bin007	SOY3_bin007_01602	270	0	0	3	0.000	0.000	1.180	hypothetical protein
bin007	SOY3_bin007_01603	7176	4	5	2	0.067	0.071	0.030	Cadherin-like beta sandwich domain protein
bin007	SOY3_bin007_01604	135	6	26	13	5.313	19.534	10.229	hypothetical protein
bin007	SOY3_bin007_01605	1218	1	7	0	0.098	0.583	0.000	Enterobactin exporter EntS
bin007	SOY3_bin007_01606	1077	0	5	3	0.000	0.471	0.296	NADH oxidase
bin007	SOY3_bin007_01607	1626	10	43	18	0.735	2.682	1.176	SWIM zinc finger
bin007	SOY3_bin007_01608	3138	50	103	33	1.905	3.329	1.117	ATP-dependent helicase HepA
bin007	SOY3_bin007_01609	201	3	2	1	1.784	1.009	0.528	hypothetical protein
bin007	SOY3_bin007_01610	165	1	1	0	0.725	0.615	0.000	Chaperone protein DnaK
bin007	SOY3_bin007_01611	1008	3	21	9	0.356	2.113	0.948	L-glyceraldehyde 3-phosphate reductase
bin007	SOY3_bin007_01612	1110	2	10	2	0.215	0.914	0.191	Cobalt-zinc-cadmium resistance protein CzcB
bin007	SOY3_bin007_01613	237	1	3	1	0.504	1.284	0.448	hypothetical protein
bin007	SOY3_bin007_01614	240	0	2	2	0.000	0.845	0.885	hypothetical protein
bin007	SOY3_bin007_01615	288	1	5	6	0.415	1.761	2.213	hypothetical protein
bin007	SOY3_bin007_01616	1521	9	18	10	0.707	1.200	0.698	Lysine--tRNA ligase
bin007	SOY3_bin007_01617	477	3	9	6	0.752	1.914	1.336	Transcription elongation factor GreA
bin007	SOY3_bin007_01618	1092	12	58	23	1.314	5.387	2.237	Long-chain acyl-[acyl-carrier-protein] reductase
bin007	SOY3_bin007_01619	525	3	22	6	0.683	4.250	1.214	hypothetical protein
bin007	SOY3_bin007_01620	963	31	106	46	3.848	11.164	5.074	tRNA-dihydrouridine synthase C
bin007	SOY3_bin007_01621	771	16	68	21	2.481	8.946	2.893	Type III pantothenate kinase
bin007	SOY3_bin007_01622	1167	4	6	0	0.410	0.521	0.000	Sulfate adenylyltransferase
bin007	SOY3_bin007_01623	711	3	5	5	0.504	0.713	0.747	Bicarbonate transport system permease protein CmpB
bin007	SOY3_bin007_01624	837	9	7	4	1.285	0.848	0.508	Taurine import ATP-binding protein TauB
bin007	SOY3_bin007_01625	1044	3	17	6	0.344	1.652	0.610	Putative aliphatic sulfonates-binding protein precursor
bin007	SOY3_bin007_01626	594	14	58	27	2.818	9.904	4.828	Biotin transporter BioY
bin007	SOY3_bin007_01627	990	31	128	46	3.743	13.114	4.936	Bifunctional ligase/repressor BirA
bin007	SOY3_bin007_01628	852	10	50	12	1.403	5.952	1.496	putative nicotinate-nucleotide pyrophosphorylase [carboxylating]
bin007	SOY3_bin007_01629	1068	10	33	20	1.119	3.134	1.989	hypothetical protein
bin007	SOY3_bin007_01630	423	3	28	9	0.848	6.714	2.260	hypothetical protein
bin007	SOY3_bin007_01631	873	16	98	33	2.191	11.386	4.015	Endoglucanase precursor
bin007	SOY3_bin007_01632	1089	25	88	38	2.744	8.196	3.707	HIUase/Transthyretin family protein
bin007	SOY3_bin007_01633	1857	167	735	232	10.751	40.145	13.271	Endoglucanase precursor
bin007	SOY3_bin007_01634	2532	0	20	4	0.000	0.801	0.168	Long-chain-fatty-acid--CoA ligase

bin007	SOY3_bin007_01635	198	0	4	0	0.000	2.049	0.000	hypothetical protein
bin007	SOY3_bin007_01636	933	61	258	87	7.816	28.047	9.905	HTH-type transcriptional regulator CynR
bin007	SOY3_bin007_01637	1245	12	27	12	1.152	2.200	1.024	hypothetical protein
bin007	SOY3_bin007_01638	561	8	13	6	1.705	2.350	1.136	LemA family protein
bin007	SOY3_bin007_01639	1938	30	39	20	1.851	2.041	1.096	hypothetical protein
bin007	SOY3_bin007_01640	276	0	4	2	0.000	1.470	0.770	hypothetical protein
bin007	SOY3_bin007_01641	1068	2	6	5	0.224	0.570	0.497	Membrane lipoprotein TmpC precursor
bin007	SOY3_bin007_01642	1428	5	18	9	0.419	1.278	0.669	RNA-splicing ligase RtcB
bin007	SOY3_bin007_01643	240	2	2	1	0.996	0.845	0.443	hypothetical protein
bin007	SOY3_bin007_01644	537	5	10	8	1.113	1.889	1.583	Invasion gene expression up-regulator, SirB
bin007	SOY3_bin007_01645	510	2	13	5	0.469	2.585	1.041	FMN reductase [NAD(P)H]
bin007	SOY3_bin007_01646	1254	19	68	26	1.811	5.500	2.202	Diaminopimelate decarboxylase
bin007	SOY3_bin007_01647	909	40	89	47	5.261	9.931	5.492	Cysteine synthase B
bin007	SOY3_bin007_01648	480	5	8	1	1.245	1.690	0.221	hypothetical protein
bin007	SOY3_bin007_01649	339	3	5	2	1.058	1.496	0.627	hypothetical protein
bin007	SOY3_bin007_01650	171	0	1	0	0.000	0.593	0.000	hypothetical protein
bin007	SOY3_bin007_01651	381	1	5	2	0.314	1.331	0.558	hypothetical protein
bin007	SOY3_bin007_01652	1590	5	15	3	0.376	0.957	0.200	Ribose import ATP-binding protein RbsA
bin007	SOY3_bin007_01653	1011	4	11	1	0.473	1.104	0.105	Sorbitol dehydrogenase
bin007	SOY3_bin007_01654	1311	4	16	6	0.365	1.238	0.486	Histidinol dehydrogenase
bin007	SOY3_bin007_01655	600	7	25	7	1.395	4.226	1.239	Carbonic anhydrase
bin007	SOY3_bin007_01656	270	8	15	8	3.542	5.635	3.147	TFIIE alpha subunit
bin007	SOY3_bin007_01657	1872	8	33	9	0.511	1.788	0.511	Chaperone protein HtpG
bin007	SOY3_bin007_01658	246	0	0	0	0.000	0.000	0.000	hypothetical protein
bin007	SOY3_bin007_01659	459	0	0	1	0.000	0.000	0.231	Sporulation lipoprotein YhcN/YlaJ (Spore_YhcN_YlaJ)
bin007	SOY3_bin007_01660	354	0	1	1	0.000	0.287	0.300	SpoVA protein
bin007	SOY3_bin007_01661	1014	0	3	1	0.000	0.300	0.105	Stage V sporulation protein AD
bin007	SOY3_bin007_01662	480	1	2	2	0.249	0.423	0.443	SpoVA protein
bin007	SOY3_bin007_01663	861	1	7	3	0.139	0.825	0.370	hypothetical protein
bin007	SOY3_bin007_01664	207	0	1	1	0.000	0.490	0.513	hypothetical protein
bin007	SOY3_bin007_01665	207	3	1	2	1.733	0.490	1.026	hypothetical protein
bin007	SOY3_bin007_01666	168	0	0	0	0.000	0.000	0.000	hypothetical protein
bin007	SOY3_bin007_01667	243	162	151	65	79.699	63.027	28.414	Selenocysteine-containing peroxiredoxin PrxU
bin007	SOY3_bin007_01668	411	227	221	88	66.028	54.539	22.744	Selenocysteine-containing peroxiredoxin PrxU
bin007	SOY3_bin007_01669	234	0	1	0	0.000	0.433	0.000	hypothetical protein
bin007	SOY3_bin007_01670	381	1	3	1	0.314	0.799	0.279	HicB family protein
bin007	SOY3_bin007_01671	183	0	2	1	0.000	1.108	0.580	hypothetical protein
bin007	SOY3_bin007_01672	1311	43	118	11	3.921	9.129	0.891	Sialic acid TRAP transporter permease protein SiaT
bin007	SOY3_bin007_01673	492	7	46	5	1.701	9.483	1.080	Sialic acid TRAP transporter permease protein SiaT
bin007	SOY3_bin007_01674	1089	8	56	5	0.878	5.216	0.488	2,3-diketo-L-gulonate-binding periplasmic protein YiaO precursor
bin007	SOY3_bin007_01675	516	15	15	12	3.475	2.948	2.470	hypothetical protein
bin007	SOY3_bin007_01676	375	0	8	0	0.000	2.164	0.000	hypothetical protein
bin007	SOY3_bin007_01677	954	9	16	9	1.128	1.701	1.002	NIF3 (NGG1p interacting factor 3)
bin007	SOY3_bin007_01678	612	6	21	8	1.172	3.480	1.389	3-methyladenine DNA glycosylase
bin007	SOY3_bin007_01679	1704	11	34	3	0.772	2.024	0.187	DNA polymerase/3'-5' exonuclease PolX
bin007	SOY3_bin007_01680	276	14	70	28	6.064	25.724	10.777	Cell division protein ZapA
bin007	SOY3_bin007_01681	990	1	2	0	0.121	0.205	0.000	putative oxidoreductase YhhX
bin007	SOY3_bin007_01682	255	1	1	0	0.469	0.398	0.000	dTDP-3-amino-3,6-dideoxy-alpha-D-galactopyranose 3-N-acetyltransferase
bin007	SOY3_bin007_01683	2421	23	68	15	1.136	2.849	0.658	Phenylalanine--tRNA ligase beta subunit
bin007	SOY3_bin007_01684	1026	4	7	3	0.466	0.692	0.311	Phenylalanine--tRNA ligase alpha subunit
bin007	SOY3_bin007_01685	810	8	25	6	1.181	3.130	0.787	23S rRNA (uridine(2479)-2'-O)-methyltransferase
bin007	SOY3_bin007_01686	648	6	21	7	1.107	3.287	1.148	Ktr system potassium uptake protein A
bin007	SOY3_bin007_01687	354	9	39	20	3.039	11.174	6.001	50S ribosomal protein L20
bin007	SOY3_bin007_01688	192	2	24	5	1.245	12.678	2.766	50S ribosomal protein L35
bin007	SOY3_bin007_01689	432	12	38	7	3.321	8.922	1.721	Translation initiation factor IF-3
bin007	SOY3_bin007_01690	2727	6	27	16	0.263	1.004	0.623	The GLUG motif protein
bin007	SOY3_bin007_01691	798	1	6	2	0.150	0.763	0.266	hypothetical protein
bin007	SOY3_bin007_01692	348	6	60	6	2.061	17.487	1.831	hypothetical protein
bin007	SOY3_bin007_01693	1689	18	71	23	1.274	4.264	1.447	Arginine--tRNA ligase
bin007	SOY3_bin007_01694	594	3	22	9	0.604	3.757	1.609	indolepyruvate oxidoreductase subunit beta
bin007	SOY3_bin007_01695	1764	28	62	35	1.898	3.565	2.108	Ferredoxin
bin007	SOY3_bin007_01696	1251	0	2	5	0.000	0.162	0.425	Putative N-acetyl-LL-diaminopimelate aminotransferase
bin007	SOY3_bin007_01697	480	0	1	2	0.000	0.211	0.443	Regulatory protein AsnC
bin007	SOY3_bin007_01698	123	4	26	7	3.888	21.440	6.045	hypothetical protein
bin007	SOY3_bin007_01699	510	3	5	1	0.703	0.994	0.208	hypothetical protein
bin007	SOY3_bin007_01700	324	2	0	2	0.738	0.000	0.656	Sporulation membrane protein YtrH
bin007	SOY3_bin007_01701	1362	4	10	2	0.351	0.745	0.156	Sporulation protein YpeB

bin007	SOY3_bin007_01702	711	5	2	0	0.841	0.285	0.000	Spore cortex-lytic enzyme precursor
bin007	SOY3_bin007_01703	318	8	40	12	3.008	12.758	4.009	Sulfite reductase, dissimilatory-type subunit gamma
bin007	SOY3_bin007_01704	882	1	10	2	0.136	1.150	0.241	hypothetical protein
bin007	SOY3_bin007_01705	483	1	7	1	0.248	1.470	0.220	Ribosomal RNA large subunit methyltransferase H
bin007	SOY3_bin007_01706	1257	8	63	30	0.761	5.083	2.535	UDP-N-acetylglucosamine 1-carboxyvinyltransferase 1
bin007	SOY3_bin007_01707	939	8	23	12	1.019	2.484	1.358	hypothetical protein
bin007	SOY3_bin007_01708	585	16	60	26	3.270	10.403	4.721	formate-dependent nitrite reductase complex subunit NrfG
bin007	SOY3_bin007_01709	1407	56	197	83	4.758	14.201	6.266	Murein DD-endopeptidase MepM
bin007	SOY3_bin007_01710	246	2	4	1	0.972	1.649	0.432	hypothetical protein
bin007	SOY3_bin007_01711	1377	15	41	15	1.302	3.020	1.157	Antilisterial bacteriocin subtilosin biosynthesis protein AlbA
bin007	SOY3_bin007_01712	1068	14	36	11	1.567	3.419	1.094	tRNA N6-adenosine threonylcarbamoyltransferase
bin007	SOY3_bin007_01713	258	2	6	0	0.927	2.359	0.000	hypothetical protein
bin007	SOY3_bin007_01714	741	1	6	0	0.161	0.821	0.000	Alpha/beta hydrolase family protein
bin007	SOY3_bin007_01715	228	1	1	0	0.524	0.445	0.000	hypothetical protein
bin007	SOY3_bin007_01716	186	2	8	2	1.285	4.362	1.142	hypothetical protein
bin007	SOY3_bin007_01717	660	0	13	10	0.000	1.998	1.609	Rhomboid protease GluP
bin007	SOY3_bin007_01718	747	8	33	6	1.280	4.481	0.853	NAD-dependent protein deacetylase
bin007	SOY3_bin007_01719	747	3	11	9	0.480	1.494	1.280	tRNA 2-thiocytidine biosynthesis protein TtcA
bin007	SOY3_bin007_01720	822	3	7	1	0.436	0.864	0.129	Peptidoglycan-N-acetylglucosamine deacetylase
bin007	SOY3_bin007_01721	1059	3	9	7	0.339	0.862	0.702	hypothetical protein
bin007	SOY3_bin007_01722	1671	11	43	9	0.787	2.610	0.572	Threonine synthase
bin007	SOY3_bin007_01723	327	7	59	33	2.559	18.300	10.720	hypothetical protein
bin007	SOY3_bin007_01724	318	1	4	8	0.376	1.276	2.672	hypothetical protein
bin007	SOY3_bin007_01725	144	1	1	3	0.830	0.704	2.213	hypothetical protein
bin007	SOY3_bin007_01726	1920	22	35	12	1.370	1.849	0.664	putative ABC transporter ATP-binding protein YheS
bin007	SOY3_bin007_01727	1443	12	28	13	0.994	1.968	0.957	Antiseptic resistance protein
bin007	SOY3_bin007_01728	1125	3	25	9	0.319	2.254	0.850	ABC transporter periplasmic-binding protein YtfQ precursor
bin007	SOY3_bin007_01729	330	7	14	1	2.536	4.303	0.322	hypothetical protein
bin007	SOY3_bin007_01730	402	7	16	8	2.082	4.037	2.114	Acyl-coenzyme A thioesterase PaaI
bin007	SOY3_bin007_01731	198	3	11	2	1.811	5.635	1.073	hypothetical protein
bin007	SOY3_bin007_01732	1167	5	17	10	0.512	1.478	0.910	Dockerin type I repeat protein
bin007	SOY3_bin007_01733	1221	9	39	18	0.881	3.240	1.566	hypothetical protein
bin007	SOY3_bin007_01734	1374	0	2	0	0.000	0.148	0.000	CRISPR-associated protein (Cas_Csm6)
bin007	SOY3_bin007_01735	336	0	0	0	0.000	0.000	0.000	CRISPR-associated endoribonuclease Cas2 3
bin007	SOY3_bin007_01736	1011	0	1	0	0.000	0.100	0.000	CRISPR-associated protein Cas4/endonuclease Cas1 fusion
bin007	SOY3_bin007_01737	747	3	7	1	0.480	0.950	0.142	hypothetical protein
bin007	SOY3_bin007_01738	1170	7	15	2	0.715	1.300	0.182	RAMP superfamily protein
bin007	SOY3_bin007_01739	942	5	14	0	0.635	1.507	0.000	hypothetical protein
bin007	SOY3_bin007_01740	693	2	8	3	0.345	1.171	0.460	RAMP superfamily protein
bin007	SOY3_bin007_01741	477	3	5	0	0.752	1.063	0.000	hypothetical protein
bin007	SOY3_bin007_01742	2475	8	34	14	0.386	1.393	0.601	phosphodiesterase
bin007	SOY3_bin007_01743	1095	0	8	0	0.000	0.741	0.000	2-iminoacetate synthase
bin007	SOY3_bin007_01744	1299	2	16	4	0.184	1.249	0.327	tRNA modification GTPase MnmE
bin007	SOY3_bin007_01745	255	0	3	0	0.000	1.193	0.000	nickel responsive regulator
bin007	SOY3_bin007_01746	1731	21	53	20	1.450	3.106	1.227	Glutamate-1-semialdehyde 2,1-aminomutase
bin007	SOY3_bin007_01747	456	2	13	2	0.524	2.892	0.466	DNA-binding transcriptional regulator AsnC
bin007	SOY3_bin007_01748	993	10	18	10	1.204	1.839	1.070	Antilisterial bacteriocin subtilosin biosynthesis protein AlbA
bin007	SOY3_bin007_01749	975	13	23	8	1.594	2.393	0.872	Delta-aminolevulinic acid dehydratase
bin007	SOY3_bin007_01750	1179	12	26	8	1.217	2.237	0.721	Antilisterial bacteriocin subtilosin biosynthesis protein AlbA
bin007	SOY3_bin007_01751	1539	18	42	21	1.398	2.768	1.449	Uroporphyrinogen-III C-methyltransferase
bin007	SOY3_bin007_01752	942	10	36	15	1.269	3.876	1.691	Porphobilinogen deaminase
bin007	SOY3_bin007_01753	1335	4	38	14	0.358	2.887	1.114	Glutamyl-tRNA reductase
bin007	SOY3_bin007_01754	642	6	19	8	1.117	3.002	1.324	Siroheme synthase
bin007	SOY3_bin007_01755	966	5	36	11	0.619	3.780	1.210	Heptaprenyl diphosphate synthase component 2
bin007	SOY3_bin007_01756	252	0	0	0	0.000	0.000	0.000	hypothetical protein
bin007	SOY3_bin007_01757	97	4	10	5	4.930	10.456	5.476	tRNA-seC(tca)
bin007	SOY3_bin007_01758	1566	4	18	6	0.305	1.166	0.407	Primary amine oxidase precursor
bin007	SOY3_bin007_01759	186	0	0	1	0.000	0.000	0.571	hypothetical protein
bin007	SOY3_bin007_01760	540	2	2	2	0.443	0.376	0.393	hypothetical protein
bin007	SOY3_bin007_01761	576	15	54	15	3.113	9.509	2.766	Primary amine oxidase precursor
bin007	SOY3_bin007_01762	153	1	9	1	0.781	5.966	0.694	hypothetical protein
bin007	SOY3_bin007_01763	339	3	17	11	1.058	5.086	3.447	hypothetical protein
bin007	SOY3_bin007_01764	117	3	4	3	3.065	3.468	2.724	hypothetical protein
bin007	SOY3_bin007_01765	837	0	12	9	0.000	1.454	1.142	hypothetical protein
bin007	SOY3_bin007_01766	741	0	6	4	0.000	0.821	0.573	hypothetical protein
bin007	SOY3_bin007_01767	543	1	21	4	0.220	3.923	0.783	Foldase protein PrsA
bin007	SOY3_bin007_01768	1413	5	10	0	0.423	0.718	0.000	Glutamate synthase [NADPH] large chain

bin007	SOY3_bin007_01769	306	1	0	1	0.391	0.000	0.347	hypothetical protein
bin007	SOY3_bin007_01770	369	2	2	1	0.648	0.550	0.288	hypothetical protein
bin007	SOY3_bin007_01771	636	2	21	7	0.376	3.349	1.169	Pyrophosphatase PpaX
bin007	SOY3_bin007_01772	1398	9	42	17	0.770	3.047	1.292	putative FAD-linked oxidoreductase
bin007	SOY3_bin007_01773	1263	10	33	5	0.947	2.650	0.421	Anaerobic glycerol-3-phosphate dehydrogenase subunit C
bin007	SOY3_bin007_01774	1470	4	23	5	0.325	1.587	0.361	Poly-beta-1,6-N-acetyl-D-glucosamine synthase
bin007	SOY3_bin007_01775	1044	1	18	6	0.115	1.749	0.610	Antilisterial bacteriocin subtilosin biosynthesis protein AlbA
bin007	SOY3_bin007_01776	252	1	7	1	0.474	2.817	0.422	hypothetical protein
bin007	SOY3_bin007_01777	909	9	31	12	1.184	3.459	1.402	Acryloyl-CoA reductase electron transfer subunit gamma
bin007	SOY3_bin007_01778	273	2	13	5	0.876	4.830	1.946	formate dehydrogenase-H ferredoxin subunit
bin007	SOY3_bin007_01779	1308	5	57	16	0.457	4.420	1.299	Electron transfer flavoprotein-ubiquinone oxidoreductase
bin007	SOY3_bin007_01780	279	1	3	0	0.428	1.091	0.000	Acryloyl-CoA reductase electron transfer subunit beta
bin007	SOY3_bin007_01781	369	5	43	21	1.620	11.819	6.045	hypothetical protein
bin007	SOY3_bin007_01782	609	1	1	2	0.196	0.167	0.349	Malonyl-[acyl-carrier protein] O-methyltransferase
bin007	SOY3_bin007_01783	201	0	1	0	0.000	0.505	0.000	Glutamine-binding periplasmic protein precursor
bin007	SOY3_bin007_01784	738	0	5	0	0.000	0.687	0.000	hypothetical protein
bin007	SOY3_bin007_01785	213	0	2	0	0.000	0.952	0.000	Regulatory protein MgsR
bin007	SOY3_bin007_01786	2157	20	56	21	1.108	2.633	1.034	30S ribosomal protein S1
bin007	SOY3_bin007_01787	900	3	11	8	0.398	1.240	0.944	HTH-type transcriptional regulator PuuR
bin007	SOY3_bin007_01788	774	1	15	6	0.154	1.966	0.823	Transposase IS200 like protein
bin007	SOY3_bin007_01789	3993	1	14	2	0.030	0.356	0.053	Maltogenic alpha-amylase precursor
bin007	SOY3_bin007_01790	2118	5	17	2	0.282	0.814	0.100	Catalase HPII
bin007	SOY3_bin007_01791	588	1	5	1	0.203	0.862	0.181	hypothetical protein
bin007	SOY3_bin007_01792	564	0	8	0	0.000	1.439	0.000	hypothetical protein
bin007	SOY3_bin007_01793	138	8	27	9	6.930	19.844	6.928	Elongation factor Tu
bin007	SOY3_bin007_01794	306	15	42	11	5.860	13.921	3.819	30S ribosomal protein S10
bin007	SOY3_bin007_01795	633	48	193	74	9.065	30.925	12.418	50S ribosomal protein L3
bin007	SOY3_bin007_01796	621	29	103	36	5.583	16.823	6.158	50S ribosomal protein L4
bin007	SOY3_bin007_01797	285	11	48	12	4.614	17.082	4.473	50S ribosomal protein L23
bin007	SOY3_bin007_01798	825	32	122	34	4.637	14.999	4.378	50S ribosomal protein L2
bin007	SOY3_bin007_01799	285	8	48	16	3.356	17.082	5.964	30S ribosomal protein S19
bin007	SOY3_bin007_01800	363	14	51	13	4.611	14.250	3.804	50S ribosomal protein L22
bin007	SOY3_bin007_01801	696	16	94	29	2.748	13.699	4.426	30S ribosomal protein S3
bin007	SOY3_bin007_01802	432	22	66	17	6.088	15.496	4.180	50S ribosomal protein L16
bin007	SOY3_bin007_01803	204	8	39	12	4.688	19.391	6.249	50S ribosomal protein L29
bin007	SOY3_bin007_01804	264	7	48	25	3.170	18.441	10.059	30S ribosomal protein S17
bin007	SOY3_bin007_01805	369	14	58	30	4.536	15.942	8.636	50S ribosomal protein L14
bin007	SOY3_bin007_01806	348	9	55	29	3.092	16.030	8.852	50S ribosomal protein L24
bin007	SOY3_bin007_01807	543	13	86	31	2.862	16.064	6.064	50S ribosomal protein L5
bin007	SOY3_bin007_01808	186	6	27	4	3.856	14.723	2.284	30S ribosomal protein S14 type Z
bin007	SOY3_bin007_01809	399	10	59	17	2.996	14.998	4.526	30S ribosomal protein S8
bin007	SOY3_bin007_01810	549	24	93	28	5.226	17.182	5.418	50S ribosomal protein L6
bin007	SOY3_bin007_01811	369	8	48	19	2.592	13.194	5.470	50S ribosomal protein L18
bin007	SOY3_bin007_01812	504	14	71	25	3.321	14.288	5.269	30S ribosomal protein S5
bin007	SOY3_bin007_01813	180	7	37	13	4.649	20.849	7.672	50S ribosomal protein L30
bin007	SOY3_bin007_01814	441	23	61	25	6.235	14.030	6.022	50S ribosomal protein L15
bin007	SOY3_bin007_01815	1260	40	190	64	3.795	15.295	5.396	preprotein translocase subunit SecY
bin007	SOY3_bin007_01816	747	29	95	44	4.641	12.899	6.257	Methionine aminopeptidase 1
bin007	SOY3_bin007_01817	387	12	58	24	3.707	15.201	6.588	hypothetical protein
bin007	SOY3_bin007_01818	219	6	23	10	3.275	10.652	4.850	Translation initiation factor IF-1
bin007	SOY3_bin007_01819	372	18	64	29	5.785	17.450	8.281	30S ribosomal protein S13
bin007	SOY3_bin007_01820	390	14	68	11	4.291	17.685	2.996	30S ribosomal protein S11
bin007	SOY3_bin007_01821	627	39	134	35	7.436	21.677	5.930	30S ribosomal protein S4
bin007	SOY3_bin007_01822	948	60	192	67	7.566	20.542	7.508	DNA-directed RNA polymerase subunit alpha
bin007	SOY3_bin007_01823	339	25	85	41	8.816	25.432	12.847	50S ribosomal protein L17
bin007	SOY3_bin007_01824	543	10	15	4	2.202	2.802	0.783	hypothetical protein
bin007	SOY3_bin007_01825	834	11	23	19	1.577	2.797	2.420	NAD-dependent protein deacylase
bin007	SOY3_bin007_01826	324	97	327	162	35.791	102.366	53.113	hypothetical protein
bin007	SOY3_bin007_01827	1398	2	13	8	0.171	0.943	0.608	Sodium/proline symporter
bin007	SOY3_bin007_01828	744	1	6	4	0.161	0.818	0.571	Aliphatic sulfonates import ATP-binding protein SsuB
bin007	SOY3_bin007_01829	753	0	4	2	0.000	0.539	0.282	Putative aliphatic sulfonates transport permease protein SsuC
bin007	SOY3_bin007_01830	981	8	35	9	0.975	3.619	0.975	NMT1/THI5 like protein
bin007	SOY3_bin007_01831	1122	20	53	25	2.131	4.791	2.367	hypothetical protein
bin007	SOY3_bin007_01832	492	33	139	40	8.019	28.655	8.636	Nigerythrin
bin007	SOY3_bin007_01833	486	7	37	16	1.722	7.722	3.497	Flagellin N-methylase
bin007	SOY3_bin007_01834	1290	11	38	12	1.019	2.988	0.988	hypothetical protein
bin007	SOY3_bin007_01835	996	2	16	6	0.240	1.629	0.640	Sulfite exporter TauE/SafE

bin007	SOY3_bin007_01836	1137	1	9	2	0.105	0.803	0.187	putative transporter
bin007	SOY3_bin007_01837	234	1	2	3	0.511	0.867	1.362	hypothetical protein
bin007	SOY3_bin007_01838	276	0	2	1	0.000	0.735	0.385	hypothetical protein
bin007	SOY3_bin007_01839	243	3	9	4	1.476	3.757	1.749	DNA-directed RNA polymerase subunit P
bin007	SOY3_bin007_01840	759	1	14	6	0.158	1.871	0.840	hypothetical protein
bin007	SOY3_bin007_01841	537	3	13	2	0.668	2.455	0.396	hypothetical protein
bin007	SOY3_bin007_01842	876	5	3	2	0.682	0.347	0.243	hypothetical protein
bin007	SOY3_bin007_01843	2169	7	43	20	0.386	2.011	0.979	ATP-dependent DNA helicase PcrA
bin007	SOY3_bin007_01844	390	0	8	6	0.000	2.081	1.634	phosphoribosyl-AMP cyclohydrolase
bin007	SOY3_bin007_01845	759	2	19	5	0.315	2.539	0.700	1-(5-phosphoribosyl)-5-[(5-phosphoribosylamino)methylideneamino] imidazole-4-carboxamide isomerase
bin007	SOY3_bin007_01846	588	2	11	4	0.407	1.897	0.723	Imidazoleglycerol-phosphate dehydratase
bin007	SOY3_bin007_01847	1089	11	51	17	1.208	4.750	1.658	Histidinol-phosphate aminotransferase 2
bin007	SOY3_bin007_01848	1329	5	12	5	0.450	0.916	0.400	Histidinol dehydrogenase
bin007	SOY3_bin007_01849	306	0	2	0	0.000	0.663	0.000	Trp operon repressor
bin007	SOY3_bin007_01850	1281	10	37	8	0.933	2.930	0.663	Phosphoribosylamine--glycine ligase
bin007	SOY3_bin007_01851	1542	11	26	11	0.853	1.710	0.758	Bifunctional purine biosynthesis protein PurH
bin007	SOY3_bin007_01852	609	2	11	6	0.393	1.832	1.047	Phosphoribosylglycinamide formyltransferase
bin007	SOY3_bin007_01853	870	4	14	7	0.550	1.632	0.855	Phosphoribosylformylglycinamidine cyclo-ligase
bin007	SOY3_bin007_01854	474	3	40	15	0.757	8.559	3.362	DNA-binding transcriptional regulator DsdC
bin007	SOY3_bin007_01855	456	1	16	3	0.262	3.559	0.699	hypothetical protein
bin007	SOY3_bin007_01856	1626	1	13	7	0.074	0.811	0.457	Proton/sodium-glutamate symport protein
bin007	SOY3_bin007_01857	1608	2	13	2	0.149	0.820	0.132	Proton/sodium-glutamate symport protein
bin007	SOY3_bin007_01858	882	5	18	9	0.678	2.070	1.084	Nicotinate dehydrogenase FAD-subunit
bin007	SOY3_bin007_01859	666	1	16	5	0.180	2.437	0.797	Carbon monoxide dehydrogenase small chain
bin007	SOY3_bin007_01860	2376	5	28	9	0.252	1.195	0.402	putative xanthine dehydrogenase subunit D
bin007	SOY3_bin007_01861	1317	56	113	37	5.083	8.703	2.984	hypothetical protein
bin007	SOY3_bin007_01862	1107	13	40	8	1.404	3.665	0.768	Histidinol-phosphate aminotransferase 2
bin007	SOY3_bin007_01863	1206	15	18	12	1.487	1.514	1.057	major facilitator superfamily transporter
bin007	SOY3_bin007_01864	1044	1	31	10	0.115	3.012	1.017	hypothetical protein
bin007	SOY3_bin007_01865	717	13	17	4	2.168	2.405	0.593	Demethylrebeccamycin-D-glucose O-methyltransferase
bin007	SOY3_bin007_01866	1020	15	45	21	1.758	4.475	2.187	hypothetical protein
bin007	SOY3_bin007_01867	834	15	67	34	2.150	8.148	4.331	N5-carboxyaminoimidazole ribonucleotide mutase
bin007	SOY3_bin007_01868	345	6	25	14	2.079	7.350	4.311	hypothetical protein
bin007	SOY3_bin007_01869	303	13	26	17	5.129	8.703	5.960	hypothetical protein
bin007	SOY3_bin007_01870	876	19	48	25	2.593	5.558	3.032	3 beta-hydroxysteroid dehydrogenase/Delta 5-->4-isomerase
bin007	SOY3_bin007_01871	879	12	57	27	1.632	6.577	3.263	Septum site-determining protein MinD
bin007	SOY3_bin007_01872	1116	29	90	39	3.107	8.180	3.712	Anaerobic sulfite reductase subunit C
bin007	SOY3_bin007_01873	1344	111	469	201	9.873	35.394	15.886	ferredoxin
bin007	SOY3_bin007_01874	1545	36	110	57	2.786	7.221	3.919	Nitrogen regulation protein NR(I)
bin007	SOY3_bin007_01875	1668	15	42	17	1.075	2.554	1.083	Sensor protein ZraS
bin007	SOY3_bin007_01876	1149	15	33	19	1.561	2.913	1.757	1,3-propanediol dehydrogenase
bin007	SOY3_bin007_01877	264	1	7	3	0.453	2.689	1.207	hypothetical protein
bin007	SOY3_bin007_01878	624	7	43	17	1.341	6.989	2.894	Nitrite reductase [NAD(P)H]
bin007	SOY3_bin007_01879	501	20	36	15	4.772	7.288	3.180	Molybdopterin-guanine dinucleotide biosynthesis adapter protein
bin007	SOY3_bin007_01880	957	3	35	9	0.375	3.709	0.999	FeS cluster assembly protein SufB
bin007	SOY3_bin007_01881	732	5	14	7	0.817	1.940	1.016	putative ABC transporter ATP-binding protein
bin007	SOY3_bin007_01882	189	0	0	0	0.000	0.000	0.000	hypothetical protein
bin007	SOY3_bin007_01883	1452	14	55	22	1.153	3.842	1.609	Aspartate ammonia-lyase
bin007	SOY3_bin007_01884	600	0	9	1	0.000	1.521	0.177	Maleamate amidohydrolase
bin007	SOY3_bin007_01885	546	3	6	5	0.657	1.115	0.973	hypothetical protein
bin007	SOY3_bin007_01886	399	1	12	4	0.300	3.050	1.065	Transcriptional regulator YqjI
bin007	SOY3_bin007_01887	1221	7	38	8	0.685	3.157	0.696	Macrolide export protein MacA
bin007	SOY3_bin007_01888	1173	5	22	10	0.510	1.902	0.906	Lipoprotein-releasing system transmembrane protein LolE
bin007	SOY3_bin007_01889	765	2	15	7	0.313	1.989	0.972	Lipoprotein-releasing system ATP-binding protein LolD
bin007	SOY3_bin007_01890	1164	2	6	3	0.205	0.523	0.274	Bacillibactin exporter
bin007	SOY3_bin007_01891	684	0	4	1	0.000	0.593	0.155	Ferredoxin
bin007	SOY3_bin007_01892	426	3	4	3	0.842	0.952	0.748	Transposase IS66 family protein
bin007	SOY3_bin007_01893	312	1	3	0	0.383	0.975	0.000	3'-5' exoribonuclease YhaM
bin007	SOY3_bin007_01894	987	2	5	3	0.242	0.514	0.323	hypothetical protein
bin007	SOY3_bin007_01895	498	2	11	4	0.480	2.240	0.853	hypothetical protein
bin007	SOY3_bin007_01896	174	1	2	1	0.687	1.166	0.610	hypothetical protein
bin007	SOY3_bin007_01897	76	3	7	5	4.719	9.342	6.989	tRNA-Val(tac)
bin007	SOY3_bin007_01898	900	7	9	4	0.930	1.014	0.472	hypothetical protein
bin007	SOY3_bin007_01899	231	3	6	0	1.553	2.634	0.000	hypothetical protein
bin007	SOY3_bin007_01900	276	0	7	1	0.000	2.572	0.385	Phd_YefM
bin007	SOY3_bin007_01901	1038	1	2	5	0.115	0.195	0.512	hypothetical protein
bin007	SOY3_bin007_01902	1611	1	4	1	0.074	0.252	0.066	hypothetical protein

bin007	SOY3_bin007_01903	693	1	0	0	0.173	0.000	0.000	hypothetical protein
bin007	SOY3_bin007_01904	4407	10	18	5	0.271	0.414	0.121	Chromosome partition protein Smc
bin007	SOY3_bin007_01905	1137	34	124	49	3.575	11.062	4.578	hypothetical protein
bin007	SOY3_bin007_01906	792	3	5	2	0.453	0.640	0.268	hypothetical protein
bin007	SOY3_bin007_01907	1296	3	16	5	0.277	1.252	0.410	Stage II sporulation protein P (SpoIIP)
bin007	SOY3_bin007_01908	945	8	13	0	1.012	1.395	0.000	hypothetical protein
bin007	SOY3_bin007_01909	192	0	1	1	0.000	0.528	0.553	hypothetical protein
bin007	SOY3_bin007_01910	633	1	2	1	0.189	0.320	0.168	hypothetical protein
bin007	SOY3_bin007_01911	339	0	0	0	0.000	0.000	0.000	hypothetical protein
bin007	SOY3_bin007_01912	1344	7	39	13	0.623	2.943	1.027	Putative serine protease HhoA precursor
bin007	SOY3_bin007_01913	1323	7	24	8	0.633	1.840	0.642	GTPase Der
bin007	SOY3_bin007_01914	588	0	8	3	0.000	1.380	0.542	Glycerol-3-phosphate acyltransferase
bin007	SOY3_bin007_01915	1044	3	26	7	0.344	2.526	0.712	Glycerol-3-phosphate dehydrogenase [NAD(P)+]
bin007	SOY3_bin007_01916	1479	101	235	62	8.164	16.116	4.453	Stage IV sporulation protein A
bin007	SOY3_bin007_01917	453	1	4	4	0.264	0.896	0.938	hypothetical protein
bin007	SOY3_bin007_01918	393	1	9	3	0.304	2.323	0.811	hypothetical protein
bin007	SOY3_bin007_01919	237	11	103	31	5.549	44.080	13.895	hypothetical protein
bin007	SOY3_bin007_01920	1584	29	166	65	2.189	10.629	4.359	Endo-1,4-beta-xylanase A precursor
bin007	SOY3_bin007_01921	216	0	2	0	0.000	0.939	0.000	hypothetical protein
bin007	SOY3_bin007_01922	2787	19	46	29	0.815	1.674	1.105	Calcium-transporting ATPase 1
bin007	SOY3_bin007_01923	345	1	4	3	0.347	1.176	0.924	Nitrogen regulatory protein P-II
bin007	SOY3_bin007_01924	738	3	13	9	0.486	1.787	1.295	hypothetical protein
bin007	SOY3_bin007_01925	693	0	22	8	0.000	3.220	1.226	hypothetical protein
bin007	SOY3_bin007_01926	522	3	7	5	0.687	1.360	1.017	FMN reductase [NAD(P)H]
bin007	SOY3_bin007_01927	1311	5	6	1	0.456	0.464	0.081	Macrolide export protein MacA
bin007	SOY3_bin007_01928	768	3	6	4	0.467	0.792	0.553	Lipoprotein-releasing system ATP-binding protein LolD
bin007	SOY3_bin007_01929	1182	1	8	1	0.101	0.686	0.090	putative ABC transporter permease YknZ
bin007	SOY3_bin007_01930	1110	13	46	21	1.400	4.203	2.010	DNA replication and repair protein RecF
bin007	SOY3_bin007_01931	195	3	2	0	1.839	1.040	0.000	ribosome-associated protein
bin007	SOY3_bin007_01932	1110	6	31	8	0.646	2.833	0.766	DNA polymerase III subunit beta
bin007	SOY3_bin007_01933	1362	7	41	14	0.614	3.053	1.092	Chromosomal replication initiator protein DnaA
bin007	SOY3_bin007_01934	339	7	32	13	2.469	9.574	4.074	Ribonuclease P protein component
bin007	SOY3_bin007_01935	669	22	78	24	3.931	11.826	3.811	Membrane protein insertase YidC
bin007	SOY3_bin007_01936	621	12	57	24	2.310	9.310	4.105	30S ribosomal protein S3
bin007	SOY3_bin007_01937	1386	2	9	2	0.173	0.659	0.153	tRNA modification GTPase MnmE
bin007	SOY3_bin007_01938	1947	4	14	11	0.246	0.729	0.600	tRNA uridine 5-carboxymethylaminomethyl modification enzyme MnmG
bin007	SOY3_bin007_01939	678	2	12	1	0.353	1.795	0.157	Ribosomal RNA small subunit methyltransferase G
bin007	SOY3_bin007_01940	834	1	12	3	0.143	1.459	0.382	Sporulation initiation inhibitor protein Soj
bin007	SOY3_bin007_01941	870	1	19	2	0.137	2.215	0.244	putative chromosome-partitioning protein ParB
bin007	SOY3_bin007_01942	792	1	228	96	0.151	29.199	12.876	Molybdate-binding periplasmic protein precursor
bin007	SOY3_bin007_01943	675	3	217	143	0.531	32.607	22.504	Molybdenum transport system permease protein ModB
bin007	SOY3_bin007_01944	711	1	130	63	0.168	18.545	9.412	Sulfate/thiosulfate import ATP-binding protein CysA
bin007	SOY3_bin007_01945	810	1	142	59	0.148	17.781	7.737	Putative binding protein precursor
bin007	SOY3_bin007_01946	867	0	108	36	0.000	12.635	4.411	Sulfate transport system permease protein CysW
bin007	SOY3_bin007_01947	1137	4	198	104	0.421	17.663	9.716	Sulfate/thiosulfate import ATP-binding protein CysA
bin007	SOY3_bin007_01948	759	2	168	68	0.315	22.450	9.517	hypothetical protein
bin007	SOY3_bin007_01949	240	6	5	3	2.989	2.113	1.328	ThiS family protein
bin007	SOY3_bin007_01950	543	2	15	5	0.440	2.802	0.978	Molybdenum-pterin-binding protein MopB
bin007	SOY3_bin007_01951	702	3	11	1	0.511	1.589	0.151	Sulfate transport system permease protein CysW
bin007	SOY3_bin007_01952	981	34	148	52	4.143	15.302	5.631	Cyclic pyranopterin monophosphate synthase
bin007	SOY3_bin007_01953	183	2	1	1	1.307	0.554	0.580	hypothetical protein
bin007	SOY3_bin007_01954	771	5	22	11	0.775	2.894	1.516	Molybdopterin-synthase adenylyltransferase
bin007	SOY3_bin007_01955	177	1	2	1	0.675	1.146	0.600	hypothetical protein
bin007	SOY3_bin007_01956	1242	12	36	12	1.155	2.940	1.026	Molybdopterin molybdenumtransferase
bin007	SOY3_bin007_01957	687	9	45	22	1.566	6.644	3.402	Cell division ATP-binding protein FtsE
bin007	SOY3_bin007_01958	912	4	21	13	0.524	2.335	1.514	Cell division protein FtsX
bin007	SOY3_bin007_01959	1131	5	33	22	0.529	2.959	2.066	Murein DD-endopeptidase MepM
bin007	SOY3_bin007_01960	1152	20	39	21	2.076	3.434	1.936	Carboxy-terminal processing protease CtpB precursor
bin007	SOY3_bin007_01961	1257	12	43	12	1.141	3.470	1.014	Cell division topological determinant MinJ
bin007	SOY3_bin007_01962	195	52	160	71	31.880	83.222	38.677	putative RNA-binding protein
bin007	SOY3_bin007_01963	621	37	88	47	7.123	14.373	8.040	Uracil DNA glycosylase superfamily protein
bin007	SOY3_bin007_01964	345	11	35	4	3.812	10.290	1.232	hypothetical protein
bin007	SOY3_bin007_01965	2031	24	53	29	1.413	2.647	1.517	UvrABC system protein B
bin007	SOY3_bin007_01966	498	7	6	5	1.680	1.222	1.067	hypothetical protein
bin007	SOY3_bin007_01967	219	2	2	1	1.092	0.926	0.485	hypothetical protein
bin007	SOY3_bin007_01968	819	6	14	3	0.876	1.734	0.389	hypothetical protein
bin007	SOY3_bin007_01969	2871	50	137	52	2.082	4.840	1.924	Nuclease SbcCD subunit C

bin007	SOY3_bin007_01970	2550	7	59	19	0.328	2.347	0.791	5-methylcytosine-specific restriction enzyme B
bin007	SOY3_bin007_01971	1317	10	31	14	0.908	2.387	1.129	5-methylcytosine-specific restriction enzyme subunit McrC
bin007	SOY3_bin007_01972	816	2	11	4	0.293	1.367	0.521	Zinc transporter ZupT
bin007	SOY3_bin007_01973	750	27	74	30	4.304	10.007	4.249	hypothetical protein
bin007	SOY3_bin007_01974	1032	0	0	1	0.000	0.000	0.103	Phospho-2-dehydro-3-deoxyheptonate aldolase
bin007	SOY3_bin007_01975	945	1	1	0	0.127	0.107	0.000	Prephenate dehydratase
bin007	SOY3_bin007_01976	351	7	31	10	2.384	8.958	3.026	hypothetical protein
bin007	SOY3_bin007_01977	519	16	43	18	3.686	8.403	3.684	hypothetical protein
bin007	SOY3_bin007_01978	426	11	45	21	3.087	10.714	5.236	hypothetical protein
bin007	SOY3_bin007_01979	453	7	33	7	1.847	7.389	1.641	hypothetical protein
bin007	SOY3_bin007_01980	1116	1	19	5	0.107	1.727	0.476	Oxidoreductase molybdopterin binding domain protein
bin007	SOY3_bin007_01981	912	4	11	6	0.524	1.223	0.699	Oxidoreductase molybdopterin binding domain protein
bin007	SOY3_bin007_01982	1530	12	24	4	0.938	1.591	0.278	Cellulosome-anchoring protein precursor
bin007	SOY3_bin007_01983	318	1	3	2	0.376	0.957	0.668	hypothetical protein
bin007	SOY3_bin007_01984	657	0	5	3	0.000	0.772	0.485	MotA/TolQ/ExbB proton channel family protein
bin007	SOY3_bin007_01985	1824	4	27	11	0.262	1.501	0.641	Kappa-carrageenase precursor
bin007	SOY3_bin007_01986	219	1	5	0	0.546	2.316	0.000	Cellulosome-anchoring protein precursor
bin007	SOY3_bin007_01987	1425	17	54	27	1.426	3.844	2.013	UDP-N-acetylgalactosamine-undecaprenyl-phosphate N-acetylgalactosaminephosphotransferase
bin007	SOY3_bin007_01988	1161	6	12	9	0.618	1.048	0.823	N,N'-diacetylbacillosaminyl-diphospho-undecaprenol alpha-1,3-N-acetylgalactosaminyltransferase
bin007	SOY3_bin007_01989	2490	2	11	6	0.096	0.448	0.256	O-Antigen ligase
bin007	SOY3_bin007_01990	750	5	15	5	0.797	2.029	0.708	putative 2-phosphosulfolactate phosphatase
bin007	SOY3_bin007_01991	75	2	3	1	3.188	4.057	1.416	tRNA-Val(cac)
bin007	SOY3_bin007_01992	2739	2	11	5	0.087	0.407	0.194	Cyclic di-GMP phosphodiesterase response regulator RpfG
bin007	SOY3_bin007_01993	258	0	0	2	0.000	0.000	0.823	hypothetical protein
bin007	SOY3_bin007_01994	624	5	16	9	0.958	2.601	1.532	5' nucleotidase, deoxy (Pyrimidine), cytosolic type C protein (NT5C)
bin007	SOY3_bin007_01995	162	1	5	4	0.738	3.130	2.623	hypothetical protein
bin007	SOY3_bin007_01996	414	8	39	8	2.310	9.555	2.053	Regulator of nucleoside diphosphate kinase
bin007	SOY3_bin007_01997	438	2	6	3	0.546	1.389	0.728	transcription elongation factor GreA
bin007	SOY3_bin007_01998	696	2	5	3	0.344	0.729	0.458	Global nitrogen regulator
bin007	SOY3_bin007_01999	723	4	9	0	0.661	1.263	0.000	Ferredoxin-1
bin007	SOY3_bin007_02000	1566	8	25	13	0.611	1.619	0.882	Hydroxylamine reductase
bin007	SOY3_bin007_02001	297	9	27	10	3.623	9.221	3.577	Carbohydrate binding domain (family 25)
bin007	SOY3_bin007_02002	537	88	192	54	19.591	36.265	10.682	Stage V sporulation protein T
bin007	SOY3_bin007_02003	198	32	90	38	19.321	46.103	20.387	Cold shock protein CspB
bin007	SOY3_bin007_02004	600	42	59	16	8.368	9.974	2.833	Superoxide dismutase [Mn/Fe]
bin007	SOY3_bin007_02005	585	5	18	6	1.022	3.121	1.089	Superoxide dismutase [Fe]
bin007	SOY3_bin007_02006	705	4	29	6	0.678	4.172	0.904	Transcriptional regulatory protein SrrA
bin007	SOY3_bin007_02007	1623	11	31	16	0.810	1.937	1.047	Non-motile and phage-resistance protein
bin007	SOY3_bin007_02008	168	1	4	1	0.712	2.415	0.632	hypothetical protein
bin007	SOY3_bin007_02009	321	1	1	0	0.372	0.316	0.000	hypothetical protein
bin007	SOY3_bin007_02010	276	1	2	1	0.433	0.735	0.385	hypothetical protein
bin007	SOY3_bin007_02011	546	32	70	25	7.007	13.003	4.864	Bifunctional protein PyrR
bin007	SOY3_bin007_02012	942	8	21	5	1.015	2.261	0.564	Aspartate carbamoyltransferase
bin007	SOY3_bin007_02013	1272	11	34	12	1.034	2.711	1.002	Dihydroorotase
bin007	SOY3_bin007_02014	978	18	43	8	2.200	4.459	0.869	Dihydroorotate dehydrogenase B (NAD(+)), electron transfer subunit
bin007	SOY3_bin007_02015	951	28	48	16	3.520	5.119	1.787	Dihydroorotate dehydrogenase B (NAD(+)), catalytic subunit
bin007	SOY3_bin007_02016	732	7	14	2	1.143	1.940	0.290	Orotidine 5'-phosphate decarboxylase
bin007	SOY3_bin007_02017	822	4	12	2	0.582	1.481	0.258	Amidohydrolase
bin007	SOY3_bin007_02018	3579	5	9	1	0.167	0.255	0.030	RNA polymerase-associated protein RapA
bin007	SOY3_bin007_02019	3045	1	3	1	0.039	0.100	0.035	D12 class N6 adenine-specific DNA methyltransferase
bin007	SOY3_bin007_02020	3363	1	2	0	0.036	0.060	0.000	hypothetical protein
bin007	SOY3_bin007_02021	330	0	2	0	0.000	0.615	0.000	hypothetical protein
bin007	SOY3_bin007_02022	375	0	12	0	0.000	3.246	0.000	Helix-turn-helix domain protein
bin007	SOY3_bin007_02023	216	0	6	1	0.000	2.817	0.492	hypothetical protein
bin007	SOY3_bin007_02024	726	0	3	0	0.000	0.419	0.000	hypothetical protein
bin007	SOY3_bin007_02025	315	2	3	0	0.759	0.966	0.000	Plasmid stabilisation system protein
bin007	SOY3_bin007_02026	273	4	12	2	1.752	4.458	0.778	Antitoxin RelF
bin007	SOY3_bin007_02027	94	0	1	2	0.000	1.079	2.260	tRNA-Ser(cga)
bin007	SOY3_bin007_02028	1284	7	20	5	0.652	1.580	0.414	Putative purine permease YbbY
bin007	SOY3_bin007_02029	159	0	8	2	0.000	5.103	1.336	hypothetical protein
bin007	SOY3_bin007_02030	756	23	63	17	3.637	8.452	2.389	Modulator of FtsH protease HflK
bin007	SOY3_bin007_02031	1353	23	43	15	2.032	3.223	1.178	ATP-dependent Clp protease proteolytic subunit
bin007	SOY3_bin007_02032	954	2	1	0	0.251	0.106	0.000	putative DMT superfamily transporter inner membrane protein
bin007	SOY3_bin007_02033	77	0	1	0	0.000	1.317	0.000	tRNA-Arg(cct)
bin007	SOY3_bin007_02034	243	1	8	0	0.492	3.339	0.000	hypothetical protein
bin007	SOY3_bin007_02035	429	2	11	1	0.557	2.601	0.248	Peptidase family U32
bin007	SOY3_bin007_02036	2358	26	80	30	1.318	3.441	1.351	Endonuclease MutS2

bin007	SOY3_bin007_02037	348	9	27	9	3.092	7.869	2.747	hypothetical protein
bin007	SOY3_bin007_02038	312	13	24	8	4.981	7.802	2.724	Carbohydrate binding domain (family 25)
bin007	SOY3_bin007_02039	2310	18	56	15	0.932	2.459	0.690	Penicillin-binding protein 1F
bin007	SOY3_bin007_02040	393	3	9	5	0.913	2.323	1.351	N-acetylmuramoyl-L-alanine amidase sle1 precursor
bin007	SOY3_bin007_02041	2106	4	20	13	0.227	0.963	0.656	succinyl-CoA synthetase subunit alpha
bin007	SOY3_bin007_02042	1059	4	23	8	0.452	2.203	0.802	Phosphate acetyltransferase
bin007	SOY3_bin007_02043	594	4	18	7	0.805	3.074	1.252	hypothetical protein
bin007	SOY3_bin007_02044	849	3	8	4	0.422	0.956	0.500	hypothetical protein
bin007	SOY3_bin007_02045	1209	4	22	8	0.396	1.846	0.703	Zonular occludens toxin (Zot)
bin007	SOY3_bin007_02046	1278	7	17	8	0.655	1.349	0.665	primosomal protein DnaI
bin007	SOY3_bin007_02047	2589	22	27	16	1.016	1.058	0.656	DNA topoisomerase 1
bin007	SOY3_bin007_02048	303	53	155	63	20.911	51.885	22.087	hypothetical protein
bin007	SOY3_bin007_02049	1464	0	6	0	0.000	0.416	0.000	Anthranilate synthase component 1
bin007	SOY3_bin007_02050	573	0	0	0	0.000	0.000	0.000	Aminodeoxychorismate/anthranilate synthase component 2
bin007	SOY3_bin007_02051	1023	1	5	0	0.117	0.496	0.000	Anthranilate phosphoribosyltransferase
bin007	SOY3_bin007_02052	1074	1	12	7	0.111	1.133	0.692	2-iminoacetate synthase
bin007	SOY3_bin007_02053	321	1	14	4	0.372	4.424	1.324	hypothetical protein
bin007	SOY3_bin007_02054	651	12	40	17	2.204	6.232	2.774	Redox-sensing transcriptional repressor Rex
bin007	SOY3_bin007_02055	408	13	27	8	3.809	6.712	2.083	hypothetical protein
bin007	SOY3_bin007_02056	1359	11	56	18	0.968	4.179	1.407	hypothetical protein
bin007	SOY3_bin007_02057	306	1	6	0	0.391	1.989	0.000	hypothetical protein
bin007	SOY3_bin007_02058	1125	0	8	0	0.000	0.721	0.000	Patatin-like phospholipase
bin007	SOY3_bin007_02059	183	2	6	3	1.307	3.325	1.741	hypothetical protein
bin007	SOY3_bin007_02060	918	4	53	25	0.521	5.856	2.893	Selenide, water dikinase
bin007	SOY3_bin007_02061	858	8	64	21	1.115	7.566	2.600	hydroxyethylthiazole kinase
bin007	SOY3_bin007_02062	537	7	47	21	1.558	8.877	4.154	hypothetical protein
bin007	SOY3_bin007_02063	222	5	49	21	2.693	22.387	10.048	Sulfurtransferase TusA
bin007	SOY3_bin007_02064	1095	20	235	101	2.184	21.767	9.798	putative inner membrane protein
bin007	SOY3_bin007_02065	1065	3	15	3	0.337	1.429	0.299	15-cis-phytoene desaturase
bin007	SOY3_bin007_02066	336	0	7	2	0.000	2.113	0.632	hypothetical protein
bin007	SOY3_bin007_02067	717	97	234	73	16.173	33.102	10.815	Fumarate reductase cytochrome b subunit
bin007	SOY3_bin007_02068	1824	353	820	283	23.136	45.598	16.481	Fumarate reductase flavoprotein subunit
bin007	SOY3_bin007_02069	750	207	434	153	32.995	58.693	21.670	Fumarate reductase iron-sulfur subunit
bin007	SOY3_bin007_02070	246	4	10	4	1.944	4.123	1.727	hypothetical protein
bin007	SOY3_bin007_02071	819	2	5	3	0.292	0.619	0.389	D-aminopeptidase
bin007	SOY3_bin007_02072	1221	2	26	6	0.196	2.160	0.522	Tyrosine--tRNA ligase
bin007	SOY3_bin007_02073	657	1	1	0	0.182	0.154	0.000	Sporulation protein YunB
bin007	SOY3_bin007_02074	897	9	18	5	1.199	2.035	0.592	Murein DD-endopeptidase MepM
bin007	SOY3_bin007_02075	1305	15	34	14	1.374	2.643	1.140	hypothetical protein
bin007	SOY3_bin007_02076	489	8	19	7	1.956	3.941	1.521	hypothetical protein
bin007	SOY3_bin007_02077	648	19	67	19	3.505	10.487	3.115	Transaldolase
bin007	SOY3_bin007_02078	855	51	142	49	7.131	16.845	6.088	Fructose-bisphosphate aldolase
bin007	SOY3_bin007_02079	708	54	209	69	9.118	29.941	10.353	DNL zinc finger
bin007	SOY3_bin007_02080	408	10	49	15	2.930	12.181	3.905	Sporulation initiation phosphotransferase F
bin007	SOY3_bin007_02081	816	4	2	2	0.586	0.249	0.260	Yip1 domain protein
bin007	SOY3_bin007_02082	924	2	5	0	0.259	0.549	0.000	Putative signal peptide peptidase SppA
bin007	SOY3_bin007_02083	1614	36	147	68	2.667	9.238	4.475	CTP synthase
bin007	SOY3_bin007_02084	885	6	62	35	0.811	7.106	4.201	Pyridoxal biosynthesis lyase PdxS
bin007	SOY3_bin007_02085	1152	28	129	53	2.906	11.358	4.887	Soluble hydrogenase 42 kDa subunit
bin007	SOY3_bin007_02086	1584	27	197	101	2.038	12.614	6.773	D-3-phosphoglycerate dehydrogenase
bin007	SOY3_bin007_02087	1269	35	110	48	3.297	8.792	4.018	Serine--tRNA ligase
bin007	SOY3_bin007_02088	90	12	33	6	15.940	37.190	7.082	tRNA-Ser(tga)
bin007	SOY3_bin007_02089	94	9	30	10	11.446	32.370	11.301	tRNA-Ser(gct)
bin007	SOY3_bin007_02090	891	0	9	3	0.000	1.025	0.358	Putative glycosyltransferase EpsH
bin007	SOY3_bin007_02091	77	0	0	0	0.000	0.000	0.000	tRNA-Arg(acg)
bin007	SOY3_bin007_02092	216	0	3	2	0.000	1.409	0.984	Tyrosine recombinase XerC
bin007	SOY3_bin007_02093	174	1	5	2	0.687	2.915	1.221	hypothetical protein
bin007	SOY3_bin007_02094	282	2	4	1	0.848	1.439	0.377	Toxin RelE
bin007	SOY3_bin007_02095	216	2	5	2	1.107	2.348	0.984	hypothetical protein
bin007	SOY3_bin007_02096	342	2	19	4	0.699	5.635	1.242	hypothetical protein
bin007	SOY3_bin007_02097	474	1	0	1	0.252	0.000	0.224	S-adenosyl-L-methionine-binding protein
bin007	SOY3_bin007_02098	720	0	2	0	0.000	0.282	0.000	ski2-like helicase
bin007	SOY3_bin007_02099	237	0	1	1	0.000	0.428	0.448	Ribbon-helix-helix protein, copG family
bin007	SOY3_bin007_02100	273	0	5	0	0.000	1.858	0.000	Toxin RelG
bin007	SOY3_bin007_02101	213	0	2	0	0.000	0.952	0.000	hypothetical protein
bin007	SOY3_bin007_02102	147	0	2	0	0.000	1.380	0.000	Endodeoxyribonuclease RusA
bin007	SOY3_bin007_02103	141	1	4	6	0.848	2.877	4.520	hypothetical protein

bin007	SOY3_bin007_02104	198	0	9	2	0.000	4.610	1.073	hypothetical protein
bin007	SOY3_bin007_02105	279	5	15	7	2.142	5.453	2.665	hypothetical protein
bin007	SOY3_bin007_02106	891	9	5	1	1.208	0.569	0.119	N-acetyltransferase YodP
bin007	SOY3_bin007_02107	1290	16	4	0	1.483	0.315	0.000	L-lysine 2,3-aminomutase
bin007	SOY3_bin007_02108	417	2	12	4	0.573	2.919	1.019	hypothetical protein
bin007	SOY3_bin007_02109	1299	1	9	6	0.092	0.703	0.491	Phosphomethylpyrimidine synthase
bin007	SOY3_bin007_02110	495	2	5	1	0.483	1.025	0.215	hypothetical protein
bin007	SOY3_bin007_02111	237	1	2	2	0.504	0.856	0.896	hypothetical protein
bin007	SOY3_bin007_02112	1344	33	146	54	2.935	11.018	4.268	NAD(P)-specific glutamate dehydrogenase
bin007	SOY3_bin007_02113	2292	42	95	25	2.191	4.204	1.159	Endo-1,4-beta-xylanase A precursor
bin007	SOY3_bin007_02114	2286	20	34	22	1.046	1.509	1.022	Endo-1,4-beta-xylanase A precursor
bin007	SOY3_bin007_02115	219	1	1	0	0.546	0.463	0.000	hypothetical protein
bin007	SOY3_bin007_02116	204	0	1	0	0.000	0.497	0.000	hypothetical protein
bin007	SOY3_bin007_02117	945	4	14	5	0.506	1.503	0.562	YtxC-like family protein
bin007	SOY3_bin007_02118	198	0	0	0	0.000	0.000	0.000	hypothetical protein
bin007	SOY3_bin007_02119	1161	23	89	13	2.368	7.775	1.189	(2R)-sulfolactate sulfo-lyase subunit beta
bin007	SOY3_bin007_02120	285	7	21	6	2.936	7.474	2.236	Altronate dehydratase
bin007	SOY3_bin007_02121	1251	20	92	19	1.911	7.459	1.613	hypothetical protein
bin007	SOY3_bin007_02122	411	4	25	1	1.163	6.170	0.258	hypothetical protein
bin007	SOY3_bin007_02123	843	28	75	20	3.971	9.024	2.520	hypothetical protein
bin007	SOY3_bin007_02124	999	6	36	8	0.718	3.655	0.851	Inner membrane protein YrbG
bin007	SOY3_bin007_02125	972	1	4	7	0.123	0.417	0.765	L-aspartate oxidase
bin007	SOY3_bin007_02126	489	1	5	2	0.244	1.037	0.434	Crossover junction endodeoxyribonuclease RuvC
bin007	SOY3_bin007_02127	591	0	2	0	0.000	0.343	0.000	Holliday junction ATP-dependent DNA helicase RuvA
bin007	SOY3_bin007_02128	1035	3	18	7	0.347	1.764	0.718	Holliday junction ATP-dependent DNA helicase RuvB
bin007	SOY3_bin007_02129	558	0	7	1	0.000	1.272	0.190	hypothetical protein
bin007	SOY3_bin007_02130	216	1	2	0	0.553	0.939	0.000	hypothetical protein
bin007	SOY3_bin007_02131	1380	8	21	13	0.693	1.543	1.001	Amidase enhancer precursor
bin007	SOY3_bin007_02132	945	4	31	11	0.506	3.327	1.236	S-adenosylmethionine:tRNA ribosyltransferase-isomerase
bin007	SOY3_bin007_02133	1110	7	43	13	0.754	3.929	1.244	Queuine tRNA-ribosyltransferase
bin007	SOY3_bin007_02134	285	5	18	10	2.097	6.406	3.727	preprotein translocase subunit YajC
bin007	SOY3_bin007_02135	660	2	32	14	0.362	4.918	2.253	putative nicotinate-nucleotide adenylyltransferase
bin007	SOY3_bin007_02136	525	1	16	6	0.228	3.091	1.214	hypothetical protein
bin007	SOY3_bin007_02137	1245	4	4	1	0.384	0.326	0.085	Energy-coupling factor transporter ATP-binding protein EcfA1
bin007	SOY3_bin007_02138	630	1	1	0	0.190	0.161	0.000	hypothetical protein
bin007	SOY3_bin007_02139	1287	1	2	0	0.093	0.158	0.000	Arylsulfotransferase (ASST)
bin007	SOY3_bin007_02140	153	1	0	0	0.781	0.000	0.000	hypothetical protein
bin007	SOY3_bin007_02141	345	0	2	0	0.000	0.588	0.000	hypothetical protein
bin007	SOY3_bin007_02142	1659	11	19	11	0.793	1.162	0.704	Endo-1,4-beta-xylanase A precursor
bin007	SOY3_bin007_02143	888	6	20	8	0.808	2.284	0.957	preprotein translocase subunit SecG
bin007	SOY3_bin007_02144	891	7	17	3	0.939	1.935	0.358	Energy-coupling factor transporter transmembrane protein EcfT
bin007	SOY3_bin007_02145	360	2	15	4	0.664	4.226	1.180	hypothetical protein
bin007	SOY3_bin007_02146	126	0	1	0	0.000	0.805	0.000	hypothetical protein
bin007	SOY3_bin007_02147	750	1	13	1	0.159	1.758	0.142	hypothetical protein
bin007	SOY3_bin007_02148	711	1	1	0	0.168	0.143	0.000	hypothetical protein
bin007	SOY3_bin007_02149	723	6	13	3	0.992	1.824	0.441	hypothetical protein
bin007	SOY3_bin007_02150	360	6	5	2	1.992	1.409	0.590	Arginine transport ATP-binding protein ArtM
bin007	SOY3_bin007_02151	1542	5	19	5	0.388	1.250	0.344	L-cystine transport system permease protein TcyB
bin007	SOY3_bin007_02152	129	0	0	0	0.000	0.000	0.000	hypothetical protein
bin007	SOY3_bin007_02153	165	0	1	0	0.000	0.615	0.000	hypothetical protein
bin007	SOY3_bin007_02154	1143	1	7	2	0.105	0.621	0.186	NAD-dependent dihydropyrimidine dehydrogenase subunit PreA
bin007	SOY3_bin007_02155	420	2	2	1	0.569	0.483	0.253	hypothetical protein
bin007	SOY3_bin007_02156	399	0	6	0	0.000	1.525	0.000	hypothetical protein
bin007	SOY3_bin007_02157	219	0	0	0	0.000	0.000	0.000	hypothetical protein
bin007	SOY3_bin007_02158	2169	1	7	0	0.055	0.327	0.000	ATP-dependent RecD-like DNA helicase
bin007	SOY3_bin007_02159	453	2	120	7	0.528	26.868	1.641	hypothetical protein
bin007	SOY3_bin007_02160	390	11	125	15	3.372	32.509	4.086	hypothetical protein
bin007	SOY3_bin007_02161	912	13	40	13	1.704	4.449	1.514	hypothetical protein
bin007	SOY3_bin007_02162	354	7	17	8	2.364	4.871	2.401	SCP-2 sterol transfer family protein
bin007	SOY3_bin007_02163	1359	11	26	12	0.968	1.940	0.938	putative amino acid permease YhdG
bin007	SOY3_bin007_02164	651	1	4	4	0.184	0.623	0.653	Spore germination protein GerM
bin007	SOY3_bin007_02165	1809	47	57	25	3.106	3.196	1.468	N-acetylmuramoyl-L-alanine amidase AmiC precursor
bin007	SOY3_bin007_02166	1224	25	167	46	2.442	13.839	3.992	flagellar assembly protein H
bin007	SOY3_bin007_02167	177	1	5	3	0.675	2.865	1.800	hypothetical protein
bin007	SOY3_bin007_02168	735	1	11	4	0.163	1.518	0.578	Phosphoribosylaminoimidazole-succinocarboxamide synthase
bin007	SOY3_bin007_02169	1458	0	0	0	0.000	0.000	0.000	Inner membrane protein YbhI
bin007	SOY3_bin007_02170	639	21	70	23	3.929	11.111	3.823	hypothetical protein

bin007	SOY3_bin007_02171	1389	5	18	7	0.430	1.314	0.535	Amidophosphoribosyltransferase precursor
bin007	SOY3_bin007_02172	927	1	3	2	0.129	0.328	0.229	hypothetical protein
bin007	SOY3_bin007_02173	894	32	91	32	4.279	10.324	3.802	Primary amine oxidase precursor
bin007	SOY3_bin007_02174	1122	109	758	167	11.614	68.522	15.811	hypothetical protein
bin007	SOY3_bin007_02175	612	62	432	92	12.111	71.596	15.969	ECF RNA polymerase sigma-E factor
bin007	SOY3_bin007_02176	2352	2227	5415	2162	113.195	233.516	97.645	hypothetical protein
bin007	SOY3_bin007_02177	1152	29	76	30	3.009	6.691	2.766	hypothetical protein
bin007	SOY3_bin007_02178	2427	32	106	54	1.576	4.430	2.363	DNA gyrase subunit A
bin007	SOY3_bin007_02179	294	41	360	124	16.672	124.197	44.803	hypothetical protein
bin007	SOY3_bin007_02180	672	1	18	7	0.178	2.717	1.107	hypothetical protein
bin007	SOY3_bin007_02181	228	6	12	4	3.146	5.338	1.864	hypothetical protein
bin007	SOY3_bin007_02182	1929	37	74	27	2.293	3.891	1.487	DNA gyrase subunit B
bin007	SOY3_bin007_02183	249	2	2	3	0.960	0.815	1.280	hypothetical protein
bin007	SOY3_bin007_02184	702	15	84	29	2.554	12.137	4.388	High molecular weight rubredoxin
bin007	SOY3_bin007_02185	1785	2	18	11	0.134	1.023	0.655	hypothetical protein
bin007	SOY3_bin007_02186	1467	1	6	6	0.081	0.415	0.434	Spore germination protein GerE
bin007	SOY3_bin007_02187	303	0	6	2	0.000	2.008	0.701	SpoVT / AbrB like domain protein
bin007	SOY3_bin007_02188	258	0	3	1	0.000	1.179	0.412	hypothetical protein
bin007	SOY3_bin007_02189	1113	0	6	3	0.000	0.547	0.286	putative diguanylate cyclase YcdT
bin007	SOY3_bin007_02190	900	0	15	3	0.000	1.690	0.354	Sensor protein CitS
bin007	SOY3_bin007_02191	765	2	7	2	0.313	0.928	0.278	Transcriptional regulatory protein YpdB
bin007	SOY3_bin007_02192	576	4	17	7	0.830	2.994	1.291	Glucokinase
bin007	SOY3_bin007_02193	2010	31	99	27	1.844	4.996	1.427	Amylo-alpha-1,6-glucosidase
bin007	SOY3_bin007_02194	1074	7	32	14	0.779	3.022	1.385	HTH-type transcriptional regulator MurR
bin007	SOY3_bin007_02195	633	7	29	9	1.322	4.647	1.510	putative phosphatase YcdX
bin007	SOY3_bin007_02196	909	8	27	15	1.052	3.013	1.753	glmZ(sRNA)-inactivating NTPase
bin007	SOY3_bin007_02197	1359	9	23	15	0.792	1.717	1.172	Gluconeogenesis factor
bin007	SOY3_bin007_02198	948	67	198	38	8.449	21.184	4.258	Sporulation transcription regulator WhiA
bin007	SOY3_bin007_02199	549	35	93	24	7.622	17.182	4.644	Galactoside O-acetyltransferase
bin007	SOY3_bin007_02200	234	4	16	7	2.044	6.935	3.178	Melamine deaminase
bin007	SOY3_bin007_02201	1422	21	83	29	1.765	5.920	2.166	Putrescine aminotransferase
bin007	SOY3_bin007_02202	354	11	25	13	3.715	7.163	3.901	putative DNA-binding protein
bin007	SOY3_bin007_02203	1350	23	97	37	2.037	7.288	2.911	Signal recognition particle protein
bin007	SOY3_bin007_02204	276	13	37	14	5.631	13.597	5.388	30S ribosomal protein S16
bin007	SOY3_bin007_02205	228	12	35	18	6.292	15.570	8.386	hypothetical protein
bin007	SOY3_bin007_02206	438	19	59	23	5.186	13.663	5.578	hypothetical protein
bin007	SOY3_bin007_02207	510	19	70	25	4.454	13.921	5.207	Ribosome maturation factor RimM
bin007	SOY3_bin007_02208	753	31	75	36	4.922	10.102	5.079	tRNA (guanine-N(1)-)-methyltransferase
bin007	SOY3_bin007_02209	342	12	31	16	4.195	9.194	4.970	50S ribosomal protein L19
bin007	SOY3_bin007_02210	573	3	12	2	0.626	2.124	0.371	Signal peptidase I T
bin007	SOY3_bin007_02211	855	9	28	18	1.258	3.322	2.236	Ribosome biogenesis GTPase A
bin007	SOY3_bin007_02212	1629	5	14	6	0.367	0.872	0.391	Chemotaxis protein CheY
bin007	SOY3_bin007_02213	1281	1	11	2	0.093	0.871	0.166	Sensor histidine kinase YpdA
bin007	SOY3_bin007_02214	2511	1	4	1	0.048	0.162	0.042	4-hydroxyphenylacetate decarboxylase large subunit
bin007	SOY3_bin007_02215	405	0	0	0	0.000	0.000	0.000	hypothetical protein
bin007	SOY3_bin007_02216	984	0	2	1	0.000	0.206	0.108	Benzylsuccinate synthase activating enzyme
bin007	SOY3_bin007_02217	321	0	2	0	0.000	0.632	0.000	hypothetical protein
bin007	SOY3_bin007_02218	243	0	1	0	0.000	0.417	0.000	hypothetical protein
bin007	SOY3_bin007_02219	1479	1	3	3	0.081	0.206	0.215	Type III restriction enzyme, res subunit
bin007	SOY3_bin007_02220	657	0	0	0	0.000	0.000	0.000	HTH-type transcriptional repressor KstR2
bin007	SOY3_bin007_02221	2670	2	3	3	0.090	0.114	0.119	Phosphoenolpyruvate synthase
bin007	SOY3_bin007_02222	207	0	1	1	0.000	0.490	0.513	hypothetical protein
bin007	SOY3_bin007_02223	510	3	1	0	0.703	0.199	0.000	Methylated-DNA--protein-cysteine methyltransferase
bin007	SOY3_bin007_02224	1545	4	9	7	0.310	0.591	0.481	DNA-3-methyladenine glycosylase 2
bin007	SOY3_bin007_02225	711	1	7	2	0.168	0.999	0.299	hypothetical protein
bin007	SOY3_bin007_02226	264	4	0	0	1.811	0.000	0.000	Ferredoxin-2
bin007	SOY3_bin007_02227	735	4	4	1	0.651	0.552	0.145	N-acetylmuramoyl-L-alanine amidase LytC precursor
bin007	SOY3_bin007_02228	567	0	4	0	0.000	0.716	0.000	putative xanthine dehydrogenase subunit E
bin007	SOY3_bin007_02229	867	0	2	0	0.000	0.234	0.000	Nicotinate dehydrogenase FAD-subunit
bin007	SOY3_bin007_02230	2337	1	4	3	0.051	0.174	0.136	Xanthine dehydrogenase molybdenum-binding subunit
bin007	SOY3_bin007_02231	243	0	0	0	0.000	0.000	0.000	hypothetical protein
bin007	SOY3_bin007_02232	738	0	0	0	0.000	0.000	0.000	Glutamine transport ATP-binding protein GlnQ
bin007	SOY3_bin007_02233	786	1	7	5	0.152	0.903	0.676	putative glutamine ABC transporter permease protein GlnM
bin007	SOY3_bin007_02234	807	3	3	3	0.444	0.377	0.395	Glutamine-binding periplasmic protein precursor
bin007	SOY3_bin007_02235	2517	41	92	27	1.947	3.707	1.139	hypothetical protein
bin007	SOY3_bin007_02236	939	7	16	5	0.891	1.728	0.566	1,4-dihydroxy-2-naphthoate octaprenyltransferase
bin007	SOY3_bin007_02237	1191	7	16	10	0.703	1.363	0.892	hypothetical protein

bin007	SOY3_bin007_02238	1032	17	45	8	1.969	4.423	0.823	replication factor C large subunit
bin007	SOY3_bin007_02239	552	5	13	8	1.083	2.389	1.540	hypothetical protein
bin007	SOY3_bin007_02240	354	2	1	1	0.675	0.287	0.300	hypothetical protein
bin007	SOY3_bin007_02241	627	4	0	1	0.763	0.000	0.169	formyl-coenzyme A transferase
bin007	SOY3_bin007_02242	390	0	2	0	0.000	0.520	0.000	Molybdenum transport system permease protein ModB
bin007	SOY3_bin007_02243	1050	1	17	3	0.114	1.642	0.304	Spermidine/putrescine import ATP-binding protein PotA
bin007	SOY3_bin007_02244	141	0	3	2	0.000	2.158	1.507	hypothetical protein
bin007	SOY3_bin007_02245	273	2	9	6	0.876	3.344	2.335	Phd_YefM
bin007	SOY3_bin007_02246	1284	2	6	5	0.186	0.474	0.414	Serpin (serine protease inhibitor)
bin007	SOY3_bin007_02247	255	1	4	1	0.469	1.591	0.417	YcfA-like protein
bin007	SOY3_bin007_02248	366	0	5	0	0.000	1.386	0.000	HicB family protein
bin007	SOY3_bin007_02249	1755	1	4	1	0.068	0.231	0.061	hypothetical protein
bin007	SOY3_bin007_02250	216	0	0	1	0.000	0.000	0.492	hypothetical protein
bin007	SOY3_bin007_02251	834	1	4	1	0.143	0.486	0.127	Molybdenum storage protein subunit beta
bin007	SOY3_bin007_02252	561	1	1	0	0.213	0.181	0.000	Spore coat assembly protein ExsA
bin007	SOY3_bin007_02253	744	1	2	0	0.161	0.273	0.000	hypothetical protein
bin007	SOY3_bin007_02254	1116	0	1	1	0.000	0.091	0.095	corrinoid ABC transporter substrate-binding protein
bin007	SOY3_bin007_02255	810	0	0	0	0.000	0.000	0.000	putative ABC transporter ATP-binding protein
bin007	SOY3_bin007_02256	1014	1	1	0	0.118	0.100	0.000	putative ABC transporter permease protein
bin007	SOY3_bin007_02257	927	1	4	1	0.129	0.438	0.115	hypothetical protein
bin007	SOY3_bin007_02258	741	5	42	10	0.807	5.749	1.434	Bicarbonate transport ATP-binding protein CmpD
bin007	SOY3_bin007_02259	1008	6	36	9	0.712	3.622	0.948	Putative thiamine biosynthesis protein
bin007	SOY3_bin007_02260	765	3	16	6	0.469	2.121	0.833	Putative aliphatic sulfonates transport permease protein SsuC
bin007	SOY3_bin007_02261	285	2	9	3	0.839	3.203	1.118	hypothetical protein
bin007	SOY3_bin007_02262	411	3	17	1	0.873	4.195	0.258	Cupin domain protein
bin007	SOY3_bin007_02263	606	32	76	27	6.313	12.720	4.733	FmdE, Molybdenum formylmethanofuran dehydrogenase operon
bin007	SOY3_bin007_02264	1866	95	221	71	6.086	12.013	4.042	Glutathione import ATP-binding protein GsiA
bin007	SOY3_bin007_02265	855	47	114	36	6.572	13.524	4.473	Oligopeptide transport system permease protein OppC
bin007	SOY3_bin007_02266	1977	1	5	1	0.060	0.257	0.054	helicase Cas3
bin007	SOY3_bin007_02267	720	2	2	2	0.332	0.282	0.295	hypothetical protein
bin007	SOY3_bin007_02268	924	1	5	0	0.129	0.549	0.000	hypothetical protein
bin007	SOY3_bin007_02269	645	0	1	0	0.000	0.157	0.000	hypothetical protein
bin007	SOY3_bin007_02270	2625	1	6	3	0.046	0.232	0.121	hypothetical protein
bin007	SOY3_bin007_02271	474	6	9	4	1.513	1.926	0.896	Fatty acid metabolism regulator protein
bin007	SOY3_bin007_02272	1071	15	26	27	1.674	2.462	2.678	Macrolide export protein MacA
bin007	SOY3_bin007_02273	768	7	8	3	1.090	1.057	0.415	putative ABC transporter ATP-binding protein YbhF
bin007	SOY3_bin007_02274	1164	6	10	2	0.616	0.871	0.183	Inner membrane transport permease YbhR
bin007	SOY3_bin007_02275	1197	1	4	1	0.100	0.339	0.089	Inner membrane transport permease YbhR
bin007	SOY3_bin007_02276	687	20	86	31	3.480	12.697	4.793	hypothetical protein
bin007	SOY3_bin007_02277	438	1	5	1	0.273	1.158	0.243	hypothetical protein
bin007	SOY3_bin007_02278	2151	11	20	4	0.611	0.943	0.198	hypothetical protein
bin007	SOY3_bin007_02279	771	2	16	10	0.310	2.105	1.378	hypothetical protein
bin007	SOY3_bin007_02280	492	1	9	2	0.243	1.855	0.432	Flavoredoxin
bin007	SOY3_bin007_02281	1095	2	20	3	0.218	1.853	0.291	Peptidoglycan-N-acetylmuramic acid deacetylase PdaC
bin007	SOY3_bin007_02282	1074	2	16	6	0.223	1.511	0.593	Putative sporulation-specific glycosylase YdhD
bin007	SOY3_bin007_02283	774	1	0	2	0.154	0.000	0.274	Spore cortex-lytic enzyme precursor
bin007	SOY3_bin007_02284	390	28	153	54	8.583	39.791	14.708	chromosome segregation protein
bin007	SOY3_bin007_02285	444	7	45	23	1.885	10.280	5.503	bifunctional RNase H/acid phosphatase
bin007	SOY3_bin007_02286	315	31	71	39	11.765	22.861	13.152	hypothetical protein
bin007	SOY3_bin007_02287	216	1	0	0	0.553	0.000	0.000	hypothetical protein
bin007	SOY3_bin007_02288	261	13	37	13	5.955	14.379	5.291	hypothetical protein
bin007	SOY3_bin007_02289	1047	5	3	0	0.571	0.291	0.000	Fibronectin type III domain protein
bin007	SOY3_bin007_02290	351	0	1	2	0.000	0.289	0.605	hypothetical protein
bin007	SOY3_bin007_02291	630	1	4	0	0.190	0.644	0.000	hypothetical protein
bin007	SOY3_bin007_02292	765	0	1	0	0.000	0.133	0.000	hypothetical protein
bin007	SOY3_bin007_02293	1674	1	2	0	0.071	0.121	0.000	Endo-1,4-beta-xylanase A precursor
bin007	SOY3_bin007_02294	441	0	2	0	0.000	0.460	0.000	hypothetical protein
bin007	SOY3_bin007_02295	567	0	0	0	0.000	0.000	0.000	hypothetical protein
bin007	SOY3_bin007_02296	885	0	0	0	0.000	0.000	0.000	hypothetical protein
bin007	SOY3_bin007_02297	297	0	0	0	0.000	0.000	0.000	hypothetical protein
bin007	SOY3_bin007_02298	813	2	16	4	0.294	1.996	0.523	(R)-stereoselective amidase
bin007	SOY3_bin007_02299	885	40	124	28	5.403	14.211	3.361	Peptidoglycan deacetylase
bin007	SOY3_bin007_02300	552	9	28	8	1.949	5.145	1.540	hypothetical protein
bin007	SOY3_bin007_02301	966	1	3	1	0.124	0.315	0.110	Putative aliphatic sulfonates-binding protein precursor
bin007	SOY3_bin007_02302	756	0	2	0	0.000	0.268	0.000	Putative aliphatic sulfonates transport permease protein SsuC
bin007	SOY3_bin007_02303	750	2	4	0	0.319	0.541	0.000	Taurine import ATP-binding protein TauB
bin007	SOY3_bin007_02304	606	1	3	2	0.197	0.502	0.351	putative HTH-type transcriptional regulator YvdT

bin007	SOY3_bin007_02305	771	2	29	15	0.310	3.815	2.067	hypothetical protein
bin007	SOY3_bin007_02306	1938	13	54	23	0.802	2.826	1.261	Lon protease 2
bin007	SOY3_bin007_02307	447	1	16	5	0.267	3.631	1.188	50S ribosomal protein L9
bin007	SOY3_bin007_02308	972	10	47	19	1.230	4.904	2.076	hypothetical protein
bin007	SOY3_bin007_02309	318	8	29	7	3.008	9.250	2.338	MazG-like family protein
bin007	SOY3_bin007_02310	231	13	65	30	6.728	28.540	13.796	30S ribosomal protein S18
bin007	SOY3_bin007_02311	414	17	99	37	4.909	24.254	9.494	Single-stranded DNA-binding protein ssb
bin007	SOY3_bin007_02312	288	10	64	18	4.151	22.539	6.639	30S ribosomal protein S6
bin007	SOY3_bin007_02313	687	1	10	0	0.174	1.476	0.000	hypothetical protein
bin007	SOY3_bin007_02314	1488	7	7	5	0.562	0.477	0.357	RAMP superfamily protein
bin007	SOY3_bin007_02315	633	2	4	2	0.378	0.641	0.336	hypothetical protein
bin007	SOY3_bin007_02316	1983	4	24	8	0.241	1.228	0.429	RAMP superfamily protein
bin007	SOY3_bin007_02317	1299	2	12	1	0.184	0.937	0.082	CRISPR-associated protein (Cas_Cas02710)
bin007	SOY3_bin007_02318	1272	6	16	16	0.564	1.276	1.336	hypothetical protein
bin007	SOY3_bin007_02319	657	5	46	26	0.910	7.101	4.204	hypothetical protein
bin007	SOY3_bin007_02320	600	11	61	15	2.192	10.312	2.656	Signal peptidase I W
bin007	SOY3_bin007_02321	1926	12	36	14	0.745	1.896	0.772	Threonine--tRNA ligase 2
bin007	SOY3_bin007_02322	645	4	27	5	0.741	4.246	0.823	Group II intron-encoded protein LtrA
bin007	SOY3_bin007_02323	303	3	9	4	1.184	3.013	1.402	hypothetical protein
bin007	SOY3_bin007_02324	747	2	15	7	0.320	2.037	0.995	Group II intron-encoded protein LtrA
bin007	SOY3_bin007_02325	1011	1	4	1	0.118	0.401	0.105	Tyrosine recombinase XerC
bin007	SOY3_bin007_02326	1236	1	0	0	0.097	0.000	0.000	Tyrosine recombinase XerC
bin007	SOY3_bin007_02327	1086	78	296	103	8.586	27.645	10.075	Transposase IS66 family protein
bin007	SOY3_bin007_02328	213	0	5	2	0.000	2.381	0.997	hypothetical protein
bin007	SOY3_bin007_02329	921	4	21	3	0.519	2.313	0.346	Putative S-adenosyl-L-methionine-dependent methyltransferase
bin007	SOY3_bin007_02330	819	9	55	10	1.314	6.811	1.297	Demethylrebeccamycin-D-glucose O-methyltransferase
bin007	SOY3_bin007_02331	294	4	29	4	1.627	10.005	1.445	hypothetical protein
bin007	SOY3_bin007_02332	720	3	27	4	0.498	3.804	0.590	hypothetical protein
bin007	SOY3_bin007_02333	282	0	1	0	0.000	0.360	0.000	hypothetical protein
bin007	SOY3_bin007_02334	315	0	1	0	0.000	0.322	0.000	hypothetical protein
bin007	SOY3_bin007_02335	261	0	5	0	0.000	1.943	0.000	hypothetical protein
bin007	SOY3_bin007_02336	279	0	10	1	0.000	3.635	0.381	antitoxin ChpS
bin007	SOY3_bin007_02337	1266	1	7	3	0.094	0.561	0.252	Radical SAM superfamily protein
bin007	SOY3_bin007_02338	4026	27	61	14	0.802	1.537	0.369	Serine protease AprX
bin007	SOY3_bin007_02339	1032	18	65	25	2.085	6.388	2.573	Rod shape-determining protein MreB
bin007	SOY3_bin007_02340	252	3	10	5	1.423	4.025	2.108	Stage III sporulation protein D
bin007	SOY3_bin007_02341	2361	0	5	7	0.000	0.215	0.315	CRISPR-associated endonuclease/helicase Cas3
bin007	SOY3_bin007_02342	759	0	0	1	0.000	0.000	0.140	hypothetical protein
bin007	SOY3_bin007_02343	1071	3	3	2	0.335	0.284	0.198	hypothetical protein
bin007	SOY3_bin007_02344	1509	3	4	3	0.238	0.269	0.211	hypothetical protein
bin007	SOY3_bin007_02345	741	26	39	20	4.195	5.338	2.867	hypothetical protein
bin007	SOY3_bin007_02346	3489	44	76	32	1.508	2.209	0.974	Maltogenic alpha-amylase precursor
bin007	SOY3_bin007_02347	1296	17	39	15	1.568	3.052	1.229	Putative competence-damage inducible protein
bin007	SOY3_bin007_02348	663	3	9	4	0.541	1.377	0.641	Fatty acid metabolism regulator protein
bin007	SOY3_bin007_02349	1110	2	7	2	0.215	0.640	0.191	Inner membrane transport permease YbhR
bin007	SOY3_bin007_02350	918	3	5	2	0.391	0.552	0.231	putative ABC transporter ATP-binding protein YbhF
bin007	SOY3_bin007_02351	912	1	6	0	0.131	0.667	0.000	putative ABC transporter ATP-binding protein YbhF
bin007	SOY3_bin007_02352	978	1	5	2	0.122	0.519	0.217	Macrolide export protein MacA
bin007	SOY3_bin007_02353	507	5	19	2	1.179	3.801	0.419	hypothetical protein
bin007	SOY3_bin007_02354	1158	14	22	2	1.445	1.927	0.183	hypothetical protein
bin007	SOY3_bin007_02355	1896	26	58	11	1.639	3.103	0.616	PrkA AAA domain protein
bin007	SOY3_bin007_02356	123	3	16	7	2.916	13.194	6.045	hypothetical protein
bin007	SOY3_bin007_02357	732	6	12	7	0.980	1.663	1.016	Cobyric acid synthase
bin007	SOY3_bin007_02358	255	3	12	0	1.406	4.773	0.000	hypothetical protein
bin007	SOY3_bin007_02359	1017	7	38	12	0.823	3.790	1.253	Acryloyl-CoA reductase electron transfer subunit beta
bin007	SOY3_bin007_02360	1956	11	71	21	0.672	3.682	1.140	NADH oxidase
bin007	SOY3_bin007_02361	1947	8	52	11	0.491	2.709	0.600	putative N-methylproline demethylase
bin007	SOY3_bin007_02362	738	5	21	8	0.810	2.886	1.151	Lactate utilization protein A
bin007	SOY3_bin007_02363	339	1	3	1	0.353	0.898	0.313	hypothetical protein
bin007	SOY3_bin007_02364	603	4	13	4	0.793	2.187	0.705	germination protease
bin007	SOY3_bin007_02365	1086	2	1	0	0.220	0.093	0.000	hypothetical protein
bin007	SOY3_bin007_02366	822	9	20	7	1.309	2.468	0.905	colicin V production protein
bin007	SOY3_bin007_02367	1419	2	35	12	0.168	2.502	0.898	putative M18 family aminopeptidase 1
bin007	SOY3_bin007_02368	213	2	4	1	1.123	1.905	0.499	DNA-directed RNA polymerase subunit P
bin007	SOY3_bin007_02369	399	0	0	0	0.000	0.000	0.000	Serine/threonine-protein phosphatase 1
bin007	SOY3_bin007_02370	447	0	0	0	0.000	0.000	0.000	hypothetical protein
bin007	SOY3_bin007_02371	306	1	6	0	0.391	1.989	0.000	Antitoxin DinJ

bin007	SOY3_bin007_02372	336	1	11	1	0.356	3.321	0.316	Plasmid stabilisation system protein
bin007	SOY3_bin007_02373	246	0	2	2	0.000	0.825	0.864	hypothetical protein
bin007	SOY3_bin007_02374	77	0	2	0	0.000	2.634	0.000	tRNA-Arg(tct)
bin007	SOY3_bin007_02375	74	0	4	0	0.000	5.483	0.000	tRNA-Gly(tcc)
bin007	SOY3_bin007_02376	477	3	7	2	0.752	1.488	0.445	hypothetical protein
bin007	SOY3_bin007_02377	615	6	21	7	1.166	3.463	1.209	dITP/XTP pyrophosphatase
bin007	SOY3_bin007_02378	774	6	10	4	0.927	1.310	0.549	Ribonuclease PH
bin007	SOY3_bin007_02379	396	0	2	2	0.000	0.512	0.536	Ribonuclease Z
bin007	SOY3_bin007_02380	171	4	13	2	2.796	7.711	1.242	hypothetical protein
bin007	SOY3_bin007_02381	1647	8	17	2	0.581	1.047	0.129	Formate hydrogenlyase transcriptional activator
bin007	SOY3_bin007_02382	2037	65	188	86	3.815	9.361	4.485	Carbon monoxide dehydrogenase 1
bin007	SOY3_bin007_02383	804	6	18	8	0.892	2.271	1.057	HPP family protein
bin007	SOY3_bin007_02384	141	0	5	1	0.000	3.597	0.753	hypothetical protein
bin007	SOY3_bin007_02385	651	1	0	0	0.184	0.000	0.000	hypothetical protein
bin007	SOY3_bin007_02386	4593	12	7	7	0.312	0.155	0.162	Cadherin-like beta sandwich domain protein
bin007	SOY3_bin007_02387	1707	12	31	22	0.840	1.842	1.369	Vancomycin B-type resistance protein VanW
bin007	SOY3_bin007_02388	264	12	38	13	5.434	14.599	5.231	hypothetical protein
bin007	SOY3_bin007_02389	441	1	3	4	0.271	0.690	0.963	Putative Holliday junction resolvase
bin007	SOY3_bin007_02390	948	5	30	19	0.631	3.210	2.129	General stress protein 69
bin007	SOY3_bin007_02391	261	3	15	3	1.374	5.829	1.221	hypothetical protein
bin007	SOY3_bin007_02392	468	3	14	6	0.766	3.034	1.362	Amino-acid acetyltransferase
bin007	SOY3_bin007_02393	861	2	9	2	0.278	1.060	0.247	Alanine--tRNA ligase
bin007	SOY3_bin007_02394	291	1	1	1	0.411	0.349	0.365	hypothetical protein
bin007	SOY3_bin007_02395	1179	1	4	2	0.101	0.344	0.180	Transposase from transposon Tn916
bin007	SOY3_bin007_02396	942	0	0	1	0.000	0.000	0.113	hypothetical protein
bin007	SOY3_bin007_02397	174	0	0	0	0.000	0.000	0.000	hypothetical protein
bin007	SOY3_bin007_02398	222	0	0	0	0.000	0.000	0.000	Helix-turn-helix domain protein
bin007	SOY3_bin007_02399	1806	0	0	0	0.000	0.000	0.000	DNA repair protein RadA
bin007	SOY3_bin007_02400	438	0	0	1	0.000	0.000	0.243	hypothetical protein
bin007	SOY3_bin007_02401	88	1	2	1	1.359	2.305	1.207	tRNA-Leu(caa)
bin007	SOY3_bin007_02402	489	115	497	169	28.115	103.087	36.712	NADP-reducing hydrogenase subunit HndA
bin007	SOY3_bin007_02403	1854	464	2107	659	29.919	115.268	37.758	NADP-reducing hydrogenase subunit HndC
bin007	SOY3_bin007_02404	930	247	1224	382	31.751	133.491	43.633	NADP-reducing hydrogenase subunit HndC
bin007	SOY3_bin007_02405	1065	208	950	331	23.349	90.475	33.015	Putative formate dehydrogenase
bin007	SOY3_bin007_02406	807	7	53	23	1.037	6.661	3.027	tRNA-specific 2-thiouridylase MnmA
bin007	SOY3_bin007_02407	2784	514	4099	1646	22.072	149.336	62.804	Aldehyde oxidoreductase
bin007	SOY3_bin007_02408	177	5	25	11	3.377	14.326	6.602	hypothetical protein
bin007	SOY3_bin007_02409	462	18	43	11	4.658	9.440	2.529	hypothetical protein
bin007	SOY3_bin007_02410	543	16	45	10	3.523	8.406	1.956	hypothetical protein
bin007	SOY3_bin007_02411	1515	25	67	23	1.973	4.486	1.613	Tyrosine-protein kinase YwqD
bin007	SOY3_bin007_02412	1737	17	55	24	1.170	3.212	1.468	Putative multidrug export ATP-binding/permease protein
bin007	SOY3_bin007_02413	186	7	24	14	4.499	13.087	7.995	hypothetical protein
bin007	SOY3_bin007_02414	423	13	37	15	3.674	8.872	3.767	hypothetical protein
bin007	SOY3_bin007_02415	591	13	36	15	2.630	6.178	2.696	Demethylrebeccamycin-D-glucose O-methyltransferase
bin007	SOY3_bin007_02416	447	1	38	14	0.267	8.622	3.327	Putative redox-active protein (C_GCAxxG_C_C)
bin007	SOY3_bin007_02417	1401	18	97	34	1.536	7.022	2.578	Cyclic pyranopterin monophosphate synthase
bin007	SOY3_bin007_02418	1350	9	63	27	0.797	4.733	2.125	Phenylacetate-coenzyme A ligase
bin007	SOY3_bin007_02419	1095	9	42	18	0.983	3.890	1.746	putative xanthine dehydrogenase subunit A
bin007	SOY3_bin007_02420	1500	25	93	18	1.992	6.288	1.275	2,3-bisphosphoglycerate-independent phosphoglycerate mutase
bin007	SOY3_bin007_02421	1290	10	58	15	0.927	4.560	1.235	Enolase
bin007	SOY3_bin007_02422	237	5	22	11	2.522	9.415	4.930	preprotein translocase subunit SecG
bin007	SOY3_bin007_02423	477	3	30	10	0.752	6.379	2.227	Thioesterase superfamily protein
bin007	SOY3_bin007_02424	552	6	38	12	1.299	6.982	2.309	hypothetical protein
bin007	SOY3_bin007_02425	522	0	5	1	0.000	0.972	0.203	nitrogen regulation protein NR(II)
bin007	SOY3_bin007_02426	1149	0	6	0	0.000	0.530	0.000	Stage II sporulation protein E
bin007	SOY3_bin007_02427	2115	12	52	15	0.678	2.494	0.753	Succinyl-CoA ligase [ADP-forming] subunit alpha
bin007	SOY3_bin007_02428	363	2	8	1	0.659	2.235	0.293	hypothetical protein
bin007	SOY3_bin007_02429	834	7	15	9	1.003	1.824	1.146	Ribonuclease HII
bin007	SOY3_bin007_02430	1356	6	4	2	0.529	0.299	0.157	hypothetical protein
bin007	SOY3_bin007_02431	1206	6	18	7	0.595	1.514	0.617	2,3-bisphosphoglycerate-independent phosphoglycerate mutase
bin007	SOY3_bin007_02432	1614	4	17	7	0.296	1.068	0.461	Multidrug export protein EmrB
bin007	SOY3_bin007_02433	135	0	0	0	0.000	0.000	0.000	hypothetical protein
bin007	SOY3_bin007_02434	669	0	1	0	0.000	0.152	0.000	hypothetical protein
bin007	SOY3_bin007_02435	2274	1	3	1	0.053	0.134	0.047	Methicillin resistance mecR1 protein
bin007	SOY3_bin007_02436	360	0	1	0	0.000	0.282	0.000	Penicillinase repressor
bin007	SOY3_bin007_02437	378	4	12	1	1.265	3.220	0.281	UDP-glucose 4-epimerase
bin007	SOY3_bin007_02438	138	2	17	4	1.733	12.495	3.079	Elongation factor Tu

bin007	SOY3_bin007_02439	150	7	28	9	5.579	18.933	6.374	50S ribosomal protein L33
bin007	SOY3_bin007_02440	372	5	46	22	1.607	12.542	6.282	preprotein translocase subunit SecE
bin007	SOY3_bin007_02441	537	9	33	18	2.004	6.233	3.561	hypothetical protein
bin007	SOY3_bin007_02442	429	9	52	14	2.508	12.294	3.467	50S ribosomal protein L11
bin007	SOY3_bin007_02443	699	27	98	38	4.618	14.220	5.775	50S ribosomal protein L1
bin007	SOY3_bin007_02444	537	16	78	39	3.562	14.732	7.715	50S ribosomal protein L10
bin007	SOY3_bin007_02445	384	27	93	41	8.406	24.564	11.342	50S ribosomal protein L7/L12
bin007	SOY3_bin007_02446	291	11	8	6	4.519	2.788	2.190	50S ribosomal protein L7/L12
bin007	SOY3_bin007_02447	429	7	31	5	1.951	7.329	1.238	flagellar assembly protein H
bin007	SOY3_bin007_02448	1047	15	73	21	1.713	7.072	2.131	Cysteine-rich secretory protein family protein
bin007	SOY3_bin007_02449	2142	20	59	18	1.116	2.794	0.893	Tyrosine-protein kinase YwqD
bin007	SOY3_bin007_02450	219	0	1	2	0.000	0.463	0.970	Replicative DNA helicase
bin007	SOY3_bin007_02451	1389	26	85	31	2.238	6.207	2.371	soluble pyridine nucleotide transhydrogenase
bin007	SOY3_bin007_02452	1287	33	76	32	3.065	5.989	2.641	Adenylosuccinate synthetase
bin007	SOY3_bin007_02453	76	0	0	0	0.000	0.000	0.000	tRNA-Lys(ttt)
bin007	SOY3_bin007_02454	76	1	3	4	1.573	4.004	5.591	tRNA-Glu(ttc)
bin007	SOY3_bin007_02455	75	1	0	0	1.594	0.000	0.000	tRNA-Gly(ccc)
bin007	SOY3_bin007_02456	831	8	12	10	1.151	1.465	1.278	hypothetical protein
bin007	SOY3_bin007_02457	240	0	0	1	0.000	0.000	0.443	hypothetical protein
bin007	SOY3_bin007_02458	285	0	8	3	0.000	2.847	1.118	hypothetical protein
bin007	SOY3_bin007_02459	486	0	5	4	0.000	1.043	0.874	VanZ like family protein
bin007	SOY3_bin007_02460	1068	4	17	6	0.448	1.614	0.597	Putative glycosyltransferase EpsH
bin007	SOY3_bin007_02461	1140	1	4	3	0.105	0.356	0.280	GDP-mannose-dependent alpha-(1-6)-phosphatidylinositol monomannoside mannosyltransferase
bin007	SOY3_bin007_02462	2997	0	3	0	0.000	0.102	0.000	Endo-1,4-beta-xylanase Y precursor
bin007	SOY3_bin007_02463	219	3	4	0	1.638	1.853	0.000	hypothetical protein
bin007	SOY3_bin007_02464	441	6	25	3	1.627	5.750	0.723	Coat F domain protein
bin007	SOY3_bin007_02465	2004	234	357	163	13.959	18.069	8.640	Acetyl-coenzyme A synthetase
bin007	SOY3_bin007_02466	1251	8	9	12	0.765	0.730	1.019	hypothetical protein
bin007	SOY3_bin007_02467	1302	8	29	11	0.735	2.259	0.897	Gamma-glutamyl phosphate reductase
bin007	SOY3_bin007_02468	894	4	10	4	0.535	1.135	0.475	1,4-dihydroxy-2-naphthoate octaprenyltransferase
bin007	SOY3_bin007_02469	522	1	11	2	0.229	2.137	0.407	Carbamoyl-phosphate synthase large chain
bin007	SOY3_bin007_02470	348	0	9	2	0.000	2.623	0.610	hypothetical protein
bin007	SOY3_bin007_02471	732	2	18	1	0.327	2.494	0.145	tRNA nuclease WapA precursor
bin007	SOY3_bin007_02472	774	16	41	9	2.471	5.373	1.235	Triosephosphate isomerase
bin007	SOY3_bin007_02473	1185	12	74	23	1.211	6.334	2.062	Phosphoglycerate kinase
bin007	SOY3_bin007_02474	1053	41	119	45	4.655	11.462	4.540	Glyceraldehyde-3-phosphate dehydrogenase
bin007	SOY3_bin007_02475	1425	50	111	42	4.195	7.901	3.131	RNA polymerase sigma-54 factor
bin007	SOY3_bin007_02476	417	3	11	5	0.860	2.676	1.274	Methylated-DNA--protein-cysteine methyltransferase
bin007	SOY3_bin007_02477	879	13	27	7	1.768	3.116	0.846	Glycine betaine/carnitine transport binding protein GbuC precursor
bin007	SOY3_bin007_02478	834	16	8	6	2.294	0.973	0.764	Glycine betaine transport system permease protein OpuAB
bin007	SOY3_bin007_02479	1191	23	49	9	2.309	4.173	0.803	Glycine betaine/carnitine transport ATP-binding protein GbuA
bin007	SOY3_bin007_02480	648	43	116	50	7.933	18.157	8.196	Energy-coupling factor transporter ATP-binding protein EcfA1
bin007	SOY3_bin007_02481	879	9	20	6	1.224	2.308	0.725	Energy-coupling factor transporter ATP-binding protein EcfA2
bin007	SOY3_bin007_02482	783	14	27	6	2.138	3.497	0.814	Energy-coupling factor transporter transmembrane protein EcfT
bin007	SOY3_bin007_02483	759	5	17	4	0.788	2.272	0.560	tRNA pseudouridine synthase A
bin007	SOY3_bin007_02484	216	0	6	0	0.000	2.817	0.000	RNA polymerases M/15 Kd subunit
bin007	SOY3_bin007_02485	1668	1	13	4	0.072	0.791	0.255	tetratricopeptide repeat protein
bin007	SOY3_bin007_02486	294	1	3	1	0.407	1.035	0.361	hypothetical protein
bin007	SOY3_bin007_02487	651	2	9	4	0.367	1.402	0.653	hypothetical protein
bin007	SOY3_bin007_02488	528	2	18	5	0.453	3.458	1.006	Serpin (serine protease inhibitor)
bin007	SOY3_bin007_02489	561	0	8	3	0.000	1.446	0.568	Isoprenylcysteine carboxyl methyltransferase (ICMT) family protein
bin007	SOY3_bin007_02490	729	3	10	3	0.492	1.391	0.437	UvrABC system protein A
bin007	SOY3_bin007_02491	1887	34	83	29	2.154	4.461	1.633	UvrABC system protein C
bin007	SOY3_bin007_02492	1140	6	26	6	0.629	2.313	0.559	putative xanthine dehydrogenase subunit A
bin007	SOY3_bin007_02493	177	2	2	5	1.351	1.146	3.001	hypothetical protein
bin007	SOY3_bin007_02494	807	6	16	11	0.889	2.011	1.448	tRNA-specific 2-thiouridylase MnmA
bin007	SOY3_bin007_02495	2784	217	1645	640	9.318	59.931	24.420	Aldehyde oxidoreductase
bin007	SOY3_bin007_02496	444	0	1	0	0.000	0.228	0.000	hypothetical protein
bin007	SOY3_bin007_02497	252	0	0	0	0.000	0.000	0.000	hypothetical protein
bin007	SOY3_bin007_02498	423	0	0	0	0.000	0.000	0.000	hypothetical protein
bin007	SOY3_bin007_02499	1647	3	11	1	0.218	0.677	0.064	Cellulosome-anchoring protein precursor
bin007	SOY3_bin007_02500	504	4	2	0	0.949	0.402	0.000	hypothetical protein
bin007	SOY3_bin007_02501	768	7	17	4	1.090	2.245	0.553	translocation protein TolB
bin007	SOY3_bin007_02502	345	0	1	0	0.000	0.294	0.000	mRNA interferase EndoA
bin007	SOY3_bin007_02503	270	0	6	0	0.000	2.254	0.000	hypothetical protein
bin007	SOY3_bin007_02504	750	2	2	2	0.319	0.270	0.283	Helix-turn-helix domain protein
bin007	SOY3_bin007_02505	300	0	4	1	0.000	1.352	0.354	Glycerol dehydrogenase

bin007	SOY3_bin007_02506	336	0	1	0	0.000	0.302	0.000	Glycerol dehydrogenase
bin007	SOY3_bin007_02507	609	1	5	0	0.196	0.833	0.000	multidrug efflux protein
bin007	SOY3_bin007_02508	126	0	4	0	0.000	3.220	0.000	3-dehydroquinate synthase
bin007	SOY3_bin007_02509	762	3	36	12	0.471	4.792	1.673	Type 4 prepilin-like proteins leader peptide-processing enzyme
bin007	SOY3_bin007_02510	1773	7	27	7	0.472	1.545	0.419	Type II secretion system protein E
bin007	SOY3_bin007_02511	1101	3	16	6	0.326	1.474	0.579	Twitching mobility protein
bin007	SOY3_bin007_02512	891	0	5	0	0.000	0.569	0.000	DNA polymerase III PolC-type
bin007	SOY3_bin007_02513	756	1	1	1	0.158	0.134	0.141	CRISPR-associated endonuclease Cas1
bin007	SOY3_bin007_02514	897	1	1	0	0.133	0.113	0.000	Plasmid recombination enzyme
bin007	SOY3_bin007_02515	327	0	0	0	0.000	0.000	0.000	hypothetical protein
bin007	SOY3_bin007_02516	1263	0	0	0	0.000	0.000	0.000	Putative prophage phiRv2 integrase
bin007	SOY3_bin007_02517	504	3	5	0	0.712	1.006	0.000	HTH-type transcriptional regulator Xre
bin007	SOY3_bin007_02518	171	2	9	7	1.398	5.338	4.348	GMP synthase [glutamine-hydrolyzing]
bin007	SOY3_bin007_02519	1296	8	38	12	0.738	2.974	0.984	3-phosphoshikimate 1-carboxyvinyltransferase 1
bin007	SOY3_bin007_02520	675	14	30	7	2.480	4.508	1.102	Cytidylate kinase
bin007	SOY3_bin007_02521	576	1	11	5	0.208	1.937	0.922	1-acyl-sn-glycerol-3-phosphate acyltransferase
bin007	SOY3_bin007_02522	474	7	19	10	1.765	4.066	2.241	hypothetical protein
bin007	SOY3_bin007_02523	2100	18	46	13	1.025	2.222	0.658	Cyclomaltodextrin glucanotransferase precursor
bin007	SOY3_bin007_02524	405	1	9	3	0.295	2.254	0.787	hypothetical protein
bin007	SOY3_bin007_02525	528	21	52	8	4.755	9.989	1.609	hypothetical protein
bin007	SOY3_bin007_02526	2076	60	335	120	3.455	16.367	6.140	Elongation factor G
bin007	SOY3_bin007_02527	471	16	77	33	4.061	16.582	7.443	30S ribosomal protein S7
bin007	SOY3_bin007_02528	375	37	117	23	11.795	31.645	6.515	30S ribosomal protein S12
bin007	SOY3_bin007_02529	252	17	58	30	8.065	23.344	12.646	Ribosome-associated protein L7Ae-like protein
bin007	SOY3_bin007_02530	240	3	6	2	1.494	2.536	0.885	hypothetical protein
bin007	SOY3_bin007_02531	657	0	0	0	0.000	0.000	0.000	Periplasmic beta-glucosidase precursor
bin007	SOY3_bin007_02532	1125	1	0	0	0.106	0.000	0.000	Putative transposase
bin007	SOY3_bin007_02533	759	0	0	0	0.000	0.000	0.000	Tyrosine recombinase XerD
bin007	SOY3_bin007_02534	801	0	0	0	0.000	0.000	0.000	Recombinase
bin007	SOY3_bin007_02535	2463	9	326	155	0.437	13.425	6.685	The GLUG motif protein
bin007	SOY3_bin007_02536	498	3	12	5	0.720	2.444	1.067	Cyclic pyranopterin monophosphate synthase accessory protein
bin007	SOY3_bin007_02537	486	1	23	5	0.246	4.800	1.093	Molybdopterin adenylyltransferase
bin007	SOY3_bin007_02538	285	49	181	67	20.554	64.415	24.972	10 kDa chaperonin
bin007	SOY3_bin007_02539	1644	302	729	258	21.961	44.976	16.670	60 kDa chaperonin
bin007	SOY3_bin007_02540	780	1	5	0	0.153	0.650	0.000	hypothetical protein
bin007	SOY3_bin007_02541	1593	7	22	7	0.525	1.401	0.467	hypothetical protein
bin007	SOY3_bin007_02542	648	1	8	3	0.184	1.252	0.492	RAMP superfamily protein
bin007	SOY3_bin007_02543	456	0	19	7	0.000	4.226	1.631	hypothetical protein
bin007	SOY3_bin007_02544	210	0	0	0	0.000	0.000	0.000	RNA polymerase sigma factor SigI
bin007	SOY3_bin007_02545	345	0	0	0	0.000	0.000	0.000	hypothetical protein
bin007	SOY3_bin007_02546	309	8	14	3	3.095	4.595	1.031	Plasmid stabilisation system protein
bin007	SOY3_bin007_02547	288	6	26	9	2.491	9.157	3.320	Phd_YefM
bin007	SOY3_bin007_02548	378	1	7	2	0.316	1.878	0.562	Carbohydrate binding domain (family 25)
bin007	SOY3_bin007_02549	252	0	9	5	0.000	3.622	2.108	hypothetical protein
bin007	SOY3_bin007_02550	1008	33	106	50	3.914	10.666	5.269	Isocitrate dehydrogenase [NADP]
bin007	SOY3_bin007_02551	504	13	37	15	3.084	7.446	3.161	2,3-dimethylmalate dehydratase small subunit
bin007	SOY3_bin007_02552	483	41	71	32	10.148	14.910	7.038	Homoaconitase large subunit
bin007	SOY3_bin007_02553	402	0	4	1	0.000	1.009	0.264	MoaE protein
bin007	SOY3_bin007_02554	1983	5	17	13	0.301	0.870	0.696	Acetyl-coenzyme A synthetase
bin007	SOY3_bin007_02555	552	1	4	1	0.217	0.735	0.192	putative inner membrane transporter YicL
bin007	SOY3_bin007_02556	639	25	50	22	4.677	7.936	3.657	Outer membrane efflux protein
bin007	SOY3_bin007_02557	813	5	20	17	0.735	2.495	2.221	hypothetical protein
bin007	SOY3_bin007_02558	480	9	16	3	2.242	3.381	0.664	putative HTH-type transcriptional regulator YusO
bin007	SOY3_bin007_02559	1191	10	27	9	1.004	2.299	0.803	Macrolide export protein MacA
bin007	SOY3_bin007_02560	315	4	0	0	1.518	0.000	0.000	Germination-specific N-acetylmuramoyl-L-alanine amidase precursor
bin007	SOY3_bin007_02561	789	12	25	9	1.818	3.214	1.212	Tyrosine-protein phosphatase YwqE
bin007	SOY3_bin007_02562	942	29	85	24	3.680	9.152	2.706	Capsular polysaccharide type 8 biosynthesis protein cap8A
bin007	SOY3_bin007_02563	828	27	48	21	3.898	5.880	2.694	Tyrosine-protein kinase YwqD
bin007	SOY3_bin007_02564	1665	1	4	0	0.072	0.244	0.000	Chondramide synthase cmdD
bin007	SOY3_bin007_02565	663	0	11	0	0.000	1.683	0.000	Aspartate racemase
bin007	SOY3_bin007_02566	732	0	8	4	0.000	1.108	0.580	Sugar fermentation stimulation protein A
bin007	SOY3_bin007_02567	1170	0	10	4	0.000	0.867	0.363	Peptide methionine sulfoxide reductase MsrA/MsrB
bin007	SOY3_bin007_02568	135	0	1	0	0.000	0.751	0.000	hypothetical protein
bin007	SOY3_bin007_02569	114	0	0	0	0.000	0.000	0.000	hypothetical protein
bin007	SOY3_bin007_02570	738	1	10	1	0.162	1.374	0.144	hypothetical protein
bin007	SOY3_bin007_02571	420	2	6	3	0.569	1.449	0.759	hypothetical protein
bin007	SOY3_bin007_02572	426	1	10	1	0.281	2.381	0.249	Nucleotidyltransferase domain protein

bin007	SOY3_bin007_02573	1134	6	10	0	0.633	0.894	0.000	putative GTPase/MT1543
bin007	SOY3_bin007_02574	249	0	1	0	0.000	0.407	0.000	Methylmalonyl-CoA mutase
bin007	SOY3_bin007_02575	723	16	42	20	2.646	5.892	2.938	Putative N-acetylmannosaminyltransferase
bin007	SOY3_bin007_02576	1059	8	31	8	0.903	2.969	0.802	Endoglucanase precursor
bin007	SOY3_bin007_02577	339	4	6	3	1.411	1.795	0.940	Multidrug export protein EmrA
bin007	SOY3_bin007_02578	456	9	59	18	2.360	13.123	4.193	50S ribosomal protein L13
bin007	SOY3_bin007_02579	393	19	98	38	5.780	25.292	10.271	30S ribosomal protein S9
bin007	SOY3_bin007_02580	456	2	9	2	0.524	2.002	0.466	hypothetical protein
bin007	SOY3_bin007_02581	873	4	15	7	0.548	1.743	0.852	hypothetical protein
bin007	SOY3_bin007_02582	606	7	4	2	1.381	0.669	0.351	hypothetical protein
bin007	SOY3_bin007_02583	699	0	2	0	0.000	0.290	0.000	hypothetical protein
bin007	SOY3_bin007_02584	318	1	3	1	0.376	0.957	0.334	Transposase
bin007	SOY3_bin007_02585	354	1	5	3	0.338	1.433	0.900	IS66 Orf2 like protein
bin007	SOY3_bin007_02586	1593	21	83	29	1.576	5.285	1.934	Transposase IS66 family protein
bin007	SOY3_bin007_02587	918	5	18	10	0.651	1.989	1.157	Methylthioribose-1-phosphate isomerase
bin007	SOY3_bin007_02588	1248	11	26	9	1.054	2.113	0.766	Adenosylhomocysteinase
bin007	SOY3_bin007_02589	762	7	34	12	1.098	4.526	1.673	hypothetical protein
bin007	SOY3_bin007_02590	1224	17	42	20	1.660	3.480	1.736	Aspartokinase
bin007	SOY3_bin007_02591	903	11	46	13	1.456	5.167	1.529	Homoserine kinase
bin007	SOY3_bin007_02592	504	4	13	6	0.949	2.616	1.265	Homoserine dehydrogenase
bin007	SOY3_bin007_02593	1497	9	29	11	0.719	1.965	0.781	L-cystine transport system permease protein TcyB
bin007	SOY3_bin007_02594	360	0	0	0	0.000	0.000	0.000	hypothetical protein
bin007	SOY3_bin007_02595	561	0	2	0	0.000	0.362	0.000	putative HTH-type transcriptional regulator YttP
bin007	SOY3_bin007_02596	627	1	3	1	0.191	0.485	0.169	F420-0:Gamma-glutamyl ligase
bin007	SOY3_bin007_02597	486	1	8	0	0.246	1.670	0.000	pyruvoyl-dependent arginine decarboxylase
bin007	SOY3_bin007_02598	642	23	101	38	4.283	15.957	6.288	Protease inhibitor precursor
bin007	SOY3_bin007_02599	378	4	22	3	1.265	5.903	0.843	hypothetical protein
bin007	SOY3_bin007_02600	2208	35	120	33	1.895	5.512	1.588	hypothetical protein
bin007	SOY3_bin007_02601	891	0	53	11	0.000	6.033	1.311	hypothetical protein
bin007	SOY3_bin007_02602	1116	0	71	18	0.000	6.453	1.713	Oxidoreductase molybdopterin binding domain protein
bin007	SOY3_bin007_02603	534	0	37	22	0.000	7.028	4.376	Oxidoreductase molybdopterin binding domain protein
bin007	SOY3_bin007_02604	417	20	62	20	5.734	15.080	5.095	hypothetical protein
bin007	SOY3_bin007_02605	447	24	56	25	6.419	12.707	5.941	hypothetical protein
bin007	SOY3_bin007_02606	333	9	26	15	3.231	7.919	4.785	hypothetical protein
bin007	SOY3_bin007_02607	288	5	17	2	2.076	5.987	0.738	hypothetical protein
bin007	SOY3_bin007_02608	1017	10	40	6	1.176	3.989	0.627	Phospho-2-dehydro-3-deoxyheptonate aldolase
bin007	SOY3_bin007_02609	375	5	11	2	1.594	2.975	0.567	HutP
bin007	SOY3_bin007_02610	1395	9	35	17	0.771	2.545	1.295	Penicillin-binding protein A
bin007	SOY3_bin007_02611	378	0	2	1	0.000	0.537	0.281	Lipid II flippase FtsW
bin007	SOY3_bin007_02612	624	3	29	11	0.575	4.714	1.873	hypothetical protein
bin007	SOY3_bin007_02613	1092	24	62	6	2.627	5.759	0.584	Protease inhibitor precursor
bin007	SOY3_bin007_02614	654	6	2	2	1.097	0.310	0.325	hypothetical protein
bin007	SOY3_bin007_02615	540	3	5	1	0.664	0.939	0.197	hypothetical protein
bin007	SOY3_bin007_02616	1389	0	22	5	0.000	1.606	0.382	Deoxyguanosinetriphosphate triphosphohydrolase
bin007	SOY3_bin007_02617	105	0	0	0	0.000	0.000	0.000	hypothetical protein
bin007	SOY3_bin007_02618	1764	225	1036	357	15.249	59.568	21.498	Fibronectin type III domain protein
bin007	SOY3_bin007_02619	342	11	21	5	3.845	6.228	1.553	hypothetical protein
bin007	SOY3_bin007_02620	204	19	62	16	11.134	30.826	8.331	Molybdenum-pterin-binding protein 2
bin007	SOY3_bin007_02621	987	6	104	22	0.727	10.687	2.368	PBP superfamily domain protein
bin007	SOY3_bin007_02622	564	2	8	3	0.424	1.439	0.565	Coenzyme F420:L-glutamate ligase
bin007	SOY3_bin007_02623	969	4	13	3	0.493	1.361	0.329	Thiamine biosynthesis lipoprotein ApbE precursor
bin007	SOY3_bin007_02624	594	1	8	1	0.201	1.366	0.179	ABC-type cobalt transport system, permease component
bin007	SOY3_bin007_02625	264	0	2	1	0.000	0.768	0.402	Lipoprotein signal peptidase
bin007	SOY3_bin007_02626	138	0	2	1	0.000	1.470	0.770	hypothetical protein
bin007	SOY3_bin007_02627	993	11	50	13	1.324	5.107	1.391	Pyruvate dehydrogenase E1 component subunit beta
bin007	SOY3_bin007_02628	960	8	36	18	0.996	3.804	1.992	Acetoin:2,6-dichlorophenolindophenol oxidoreductase subunit alpha
bin007	SOY3_bin007_02629	795	5	18	7	0.752	2.296	0.935	S-methyl-5'-thioadenosine phosphorylase
bin007	SOY3_bin007_02630	258	0	0	0	0.000	0.000	0.000	hypothetical protein
bin007	SOY3_bin007_02631	210	1	1	0	0.569	0.483	0.000	hypothetical protein
bin007	SOY3_bin007_02632	225	1	0	0	0.531	0.000	0.000	hypothetical protein
bin007	SOY3_bin007_02633	717	0	0	0	0.000	0.000	0.000	hypothetical protein
bin007	SOY3_bin007_02634	729	16	26	12	2.624	3.617	1.749	putative murein peptide carboxypeptidase
bin007	SOY3_bin007_02635	288	0	2	0	0.000	0.704	0.000	Acylphosphatase
bin007	SOY3_bin007_02636	963	3	11	3	0.372	1.159	0.331	hydroxyacylglutathione hydrolase
bin007	SOY3_bin007_02637	1593	24	58	12	1.801	3.693	0.800	hypothetical protein
bin007	SOY3_bin007_02638	318	2	23	9	0.752	7.336	3.006	nucleoside triphosphate pyrophosphohydrolase
bin007	SOY3_bin007_02639	717	75	107	42	12.505	15.136	6.222	hypothetical protein

bin007	SOY3_bin007_02640	2811	2	139	48	0.085	5.015	1.814	Cohesin domain protein
bin007	SOY3_bin007_02641	1065	2	6	2	0.225	0.571	0.199	Proline/betaine transporter
bin007	SOY3_bin007_02642	609	2	3	1	0.393	0.500	0.174	ECF RNA polymerase sigma-E factor
bin007	SOY3_bin007_02643	768	1	3	1	0.156	0.396	0.138	argininosuccinate lyase
bin007	SOY3_bin007_02644	1101	1	6	3	0.109	0.553	0.289	dTDP-4-amino-4,6-dideoxy-D-glucose transaminase
bin007	SOY3_bin007_02645	207	0	0	0	0.000	0.000	0.000	hypothetical protein
bin007	SOY3_bin007_02646	807	4	6	2	0.593	0.754	0.263	Diguanylate cyclase DosC
bin007	SOY3_bin007_02647	384	0	2	0	0.000	0.528	0.000	hypothetical protein
bin007	SOY3_bin007_02648	555	1	5	1	0.215	0.914	0.191	hypothetical protein
bin007	SOY3_bin007_02649	429	1	2	1	0.279	0.473	0.248	macrolide transporter subunit MacA
bin007	SOY3_bin007_02650	756	7	4	7	1.107	0.537	0.984	Macrolide export ATP-binding/permease protein MacB
bin007	SOY3_bin007_02651	1218	9	18	4	0.883	1.499	0.349	Macrolide export ATP-binding/permease protein MacB
bin007	SOY3_bin007_02652	153	0	6	2	0.000	3.978	1.389	hypothetical protein
bin007	SOY3_bin007_02653	744	6	35	17	0.964	4.771	2.427	Ribosomal large subunit pseudouridine synthase B
bin007	SOY3_bin007_02654	468	5	15	9	1.277	3.251	2.043	hypothetical protein
bin007	SOY3_bin007_02655	534	3	13	9	0.672	2.469	1.790	hypothetical protein
bin007	SOY3_bin007_02656	798	3	12	7	0.449	1.525	0.932	AB hydrolase superfamily protein YvaM
bin007	SOY3_bin007_02657	900	3	5	3	0.398	0.563	0.354	Radical SAM superfamily protein
bin007	SOY3_bin007_02658	435	1	1	3	0.275	0.233	0.733	Type I restriction enzyme EcoR124II R protein
bin007	SOY3_bin007_02659	88	1	2	0	1.359	2.305	0.000	tRNA-Leu(caa)
bin007	SOY3_bin007_02660	204	0	1	0	0.000	0.497	0.000	F0F1 ATP synthase subunit B
bin007	SOY3_bin007_02661	1314	2	6	2	0.182	0.463	0.162	hypothetical protein
bin007	SOY3_bin007_02662	303	1	0	0	0.395	0.000	0.000	hypothetical protein
bin007	SOY3_bin007_02663	483	1	1	1	0.248	0.210	0.220	hypothetical protein
bin007	SOY3_bin007_02664	441	1	19	3	0.271	4.370	0.723	hypothetical protein
bin007	SOY3_bin007_02665	573	2	98	61	0.417	17.347	11.309	hypothetical protein
bin007	SOY3_bin007_02666	1167	14	39	17	1.434	3.390	1.547	hypothetical protein
bin007	SOY3_bin007_02667	447	5	34	3	1.337	7.715	0.713	LexA repressor
bin007	SOY3_bin007_02668	876	28	159	55	3.821	18.410	6.669	hypothetical protein
bin007	SOY3_bin007_02669	465	1	3	1	0.257	0.654	0.228	putative Helix-turn-helix domain of transposase IS66
bin007	SOY3_bin007_02670	477	3	13	3	0.752	2.764	0.668	Transposase IS66 family protein
bin007	SOY3_bin007_02671	483	0	3	1	0.000	0.630	0.220	Transposase IS66 family protein
bin007	SOY3_bin007_02672	2493	38	91	27	1.822	3.702	1.150	Maltogenic alpha-amylase precursor
bin007	SOY3_bin007_02673	780	14	14	10	2.146	1.820	1.362	Ribonuclease Z
bin007	SOY3_bin007_02674	852	7	24	8	0.982	2.857	0.997	Glutamate racemase
bin007	SOY3_bin007_02675	2013	0	196	96	0.000	9.876	5.066	Cellulosome-anchoring protein precursor
bin007	SOY3_bin007_02676	207	0	30	7	0.000	14.700	3.592	hypothetical protein
bin007	SOY3_bin007_02677	153	0	0	0	0.000	0.000	0.000	hypothetical protein
bin007	SOY3_bin007_02678	357	0	1	0	0.000	0.284	0.000	hypothetical protein
bin007	SOY3_bin007_02679	1098	3	4	0	0.327	0.369	0.000	hypothetical protein
bin007	SOY3_bin007_02680	351	5	6	13	1.703	1.734	3.934	hypothetical protein
bin007	SOY3_bin007_02681	354	4	6	10	1.351	1.719	3.001	IS66 Orf2 like protein
bin007	SOY3_bin007_02682	1572	19	85	81	1.445	5.484	5.473	Transposase IS66 family protein
bin007	SOY3_bin007_02683	912	1	11	2	0.131	1.223	0.233	Putative ribosome biogenesis GTPase RsgA
bin007	SOY3_bin007_02684	696	3	11	2	0.515	1.603	0.305	Serine/threonine-protein kinase PK-1
bin007	SOY3_bin007_02685	174	0	0	0	0.000	0.000	0.000	hypothetical protein
bin007	SOY3_bin007_02686	504	0	3	1	0.000	0.604	0.211	hypothetical protein
bin007	SOY3_bin007_02687	354	3	2	2	1.013	0.573	0.600	hypothetical protein
bin007	SOY3_bin007_02688	246	0	3	0	0.000	1.237	0.000	hypothetical protein
bin007	SOY3_bin007_02689	375	5	3	1	1.594	0.811	0.283	hypothetical protein
bin007	SOY3_bin007_02690	1056	0	14	4	0.000	1.345	0.402	Phospho-2-dehydro-3-deoxyheptonate aldolase
bin007	SOY3_bin007_02691	615	0	3	0	0.000	0.495	0.000	N-(5'-phosphoribosyl)anthranilate isomerase
bin007	SOY3_bin007_02692	315	0	2	0	0.000	0.644	0.000	Indole-3-glycerol phosphate synthase
bin007	SOY3_bin007_02693	75	0	0	0	0.000	0.000	0.000	tRNA-Thr(cgt)
bin007	SOY3_bin007_02694	390	2	3	1	0.613	0.780	0.272	hypothetical protein
bin007	SOY3_bin007_02695	174	3	4	3	2.061	2.332	1.831	manganese transport transcriptional regulator
bin007	SOY3_bin007_02696	1938	10	22	6	0.617	1.151	0.329	Aldehyde-alcohol dehydrogenase
bin007	SOY3_bin007_02697	1293	3	7	4	0.277	0.549	0.329	Methylenetetrahydrofolate--tRNA-(uracil-5-)-methyltransferase TrmFO
bin007	SOY3_bin007_02698	96	1	0	1	1.245	0.000	1.107	hypothetical protein
bin007	SOY3_bin007_02699	444	3	5	1	0.808	1.142	0.239	Cob(I)yrinic acid a,c-diamide adenosyltransferase
bin007	SOY3_bin007_02700	381	0	2	0	0.000	0.532	0.000	hypothetical protein
bin007	SOY3_bin007_02701	486	3	8	5	0.738	1.670	1.093	hypothetical protein
bin007	SOY3_bin007_02702	2091	29	47	16	1.658	2.280	0.813	Cyclomaltodextrin glucanotransferase precursor
bin007	SOY3_bin007_02703	513	6	16	10	1.398	3.163	2.071	hypothetical protein
bin007	SOY3_bin007_02704	546	4	7	3	0.876	1.300	0.584	Hypoxanthine-guanine phosphoribosyltransferase
bin007	SOY3_bin007_02705	357	0	3	1	0.000	0.852	0.298	hypothetical protein
bin007	SOY3_bin007_02706	324	0	1	1	0.000	0.313	0.328	Branched-chain amino acid transport protein (AzlD)

bin007	SOY3_bin007_02707	687	0	3	3	0.000	0.443	0.464	Inner membrane protein YgaZ
bin007	SOY3_bin007_02708	2118	24	35	13	1.355	1.676	0.652	Cyclomaltodextrin glucanotransferase precursor
bin007	SOY3_bin007_02709	276	74	381	119	32.053	140.014	45.800	hypothetical protein
bin007	SOY3_bin007_02710	549	169	768	220	36.801	141.887	42.568	putative Helix-turn-helix domain of transposase IS66
bin007	SOY3_bin007_02711	654	77	316	95	14.075	49.008	15.430	Transposase IS66 family protein
bin007	SOY3_bin007_02712	168	0	0	0	0.000	0.000	0.000	hypothetical protein
bin007	SOY3_bin007_02713	1665	1	1	1	0.072	0.061	0.064	Endo-1,4-beta-xylanase A precursor
bin007	SOY3_bin007_02714	495	2	3	0	0.483	0.615	0.000	hypothetical protein
bin007	SOY3_bin007_02715	252	0	2	0	0.000	0.805	0.000	hypothetical protein
bin007	SOY3_bin007_02716	333	0	4	1	0.000	1.218	0.319	hypothetical protein
bin007	SOY3_bin007_02717	1011	1	7	0	0.118	0.702	0.000	Glycogen synthase
bin007	SOY3_bin007_02718	1443	24	168	63	1.988	11.809	4.638	Anaerobic glycerol-3-phosphate dehydrogenase subunit C
bin007	SOY3_bin007_02719	213	4	24	11	2.245	11.428	5.486	hypothetical protein
bin007	SOY3_bin007_02720	213	0	1	0	0.000	0.476	0.000	hypothetical protein
bin007	SOY3_bin007_02721	213	0	1	2	0.000	0.476	0.997	hypothetical protein
bin007	SOY3_bin007_02722	1056	7	18	4	0.792	1.729	0.402	Macrolide export ATP-binding/permease protein MacB
bin007	SOY3_bin007_02723	762	6	9	10	0.941	1.198	1.394	hypothetical protein
bin007	SOY3_bin007_02724	297	4	5	4	1.610	1.708	1.431	hypothetical protein
bin007	SOY3_bin007_02725	135	52	231	94	46.048	173.553	73.965	Fe/S biogenesis protein NfuA
bin007	SOY3_bin007_02726	582	19	100	10	3.903	17.427	1.825	hypothetical protein
bin007	SOY3_bin007_02727	270	3	12	3	1.328	4.508	1.180	Lactate utilization protein C
bin007	SOY3_bin007_02728	1572	15	65	32	1.141	4.194	2.162	hypothetical protein
bin007	SOY3_bin007_02729	1461	1	5	2	0.082	0.347	0.145	Teichuronic acid biosynthesis protein TuaB
bin007	SOY3_bin007_02730	465	8	14	4	2.057	3.054	0.914	Glycerol-3-phosphate cytidylyltransferase
bin007	SOY3_bin007_02731	459	6	5	0	1.563	1.105	0.000	tRNA-specific adenosine deaminase
bin007	SOY3_bin007_02732	1188	9	14	0	0.906	1.195	0.000	hypothetical protein
bin007	SOY3_bin007_02733	222	4	44	14	2.154	20.103	6.699	hypothetical protein
bin007	SOY3_bin007_02734	258	0	5	1	0.000	1.966	0.412	hypothetical protein
bin007	SOY3_bin007_02735	1080	1	2	0	0.111	0.188	0.000	dTDP-glucose 4,6-dehydratase
bin007	SOY3_bin007_02736	387	0	0	0	0.000	0.000	0.000	hypothetical protein
bin007	SOY3_bin007_02737	918	209	1288	441	27.218	142.308	51.030	putative formate transporter 1
bin007	SOY3_bin007_02738	465	66	336	106	16.968	73.289	24.215	OB-fold nucleic acid binding domain protein
bin007	SOY3_bin007_02739	918	111	529	147	14.455	58.448	17.010	putative formate transporter 1
bin007	SOY3_bin007_02740	465	29	199	61	7.456	43.407	13.935	OB-fold nucleic acid binding domain protein
bin007	SOY3_bin007_02741	1176	1	9	6	0.102	0.776	0.542	hypothetical protein
bin007	SOY3_bin007_02742	534	3	8	7	0.672	1.520	1.392	N5-carboxyaminoimidazole ribonucleotide mutase
bin007	SOY3_bin007_02743	309	1	8	4	0.387	2.626	1.375	hypothetical protein
bin007	SOY3_bin007_02744	342	0	2	2	0.000	0.593	0.621	Endoglucanase precursor
bin007	SOY3_bin007_02745	783	3	5	5	0.458	0.648	0.678	hypothetical protein
bin007	SOY3_bin007_02746	252	0	1	1	0.000	0.402	0.422	hypothetical protein
bin007	SOY3_bin007_02747	1431	5	65	41	0.418	4.607	3.044	Endoglucanase D precursor
bin007	SOY3_bin007_02748	378	0	0	0	0.000	0.000	0.000	Lipid II flippase FtsW
bin007	SOY3_bin007_02749	552	0	2	3	0.000	0.367	0.577	hypothetical protein
bin007	SOY3_bin007_02750	1236	9	38	11	0.871	3.118	0.945	Transposase DDE domain protein
bin007	SOY3_bin007_02751	402	0	0	0	0.000	0.000	0.000	Ribonuclease BN
bin007	SOY3_bin007_02752	207	0	0	0	0.000	0.000	0.000	hypothetical protein
bin007	SOY3_bin007_02753	1137	24	107	33	2.523	9.545	3.083	2-isopropylmalate synthase
bin007	SOY3_bin007_02754	372	14	39	9	4.499	10.634	2.570	hypothetical protein
bin007	SOY3_bin007_02755	603	49	146	49	9.715	24.558	8.632	putative 5-formyltetrahydrofolate cyclo-ligase
bin007	SOY3_bin007_02756	459	10	22	14	2.605	4.861	3.240	hypothetical protein
bin007	SOY3_bin007_02757	231	13	26	8	6.728	11.416	3.679	hypothetical protein
bin007	SOY3_bin007_02758	252	5	15	6	2.372	6.037	2.529	hypothetical protein
bin007	SOY3_bin007_02759	261	2	8	1	0.916	3.109	0.407	Ferrous iron transport protein A
bin007	SOY3_bin007_02760	795	0	3	3	0.000	0.383	0.401	Ferrous iron transport protein B
bin007	SOY3_bin007_02761	417	1	8	2	0.287	1.946	0.509	hypothetical protein
bin007	SOY3_bin007_02762	504	1	7	1	0.237	1.409	0.211	Adenylosuccinate lyase
bin007	SOY3_bin007_02763	669	2	1	1	0.357	0.152	0.159	hypothetical protein
bin007	SOY3_bin007_02764	642	1	2	1	0.186	0.316	0.165	Energy-coupling factor transporter transmembrane protein EcfT
bin007	SOY3_bin007_02765	1146	4	16	2	0.417	1.416	0.185	UDP-N-acetylgalactosamine-undecaprenyl-phosphate N-acetylgalactosaminephosphotransferase
bin007	SOY3_bin007_02766	750	7	22	10	1.116	2.975	1.416	putative aminodeoxychorismate lyase
bin007	SOY3_bin007_02767	297	1	8	3	0.403	2.732	1.073	hypothetical protein
bin007	SOY3_bin007_02768	561	11	21	7	2.344	3.797	1.325	hypothetical protein
bin007	SOY3_bin007_02769	504	2	5	1	0.474	1.006	0.211	Homoserine dehydrogenase
bin007	SOY3_bin007_02770	579	2	4	3	0.413	0.701	0.550	Type II secretion system protein F
bin007	SOY3_bin007_02771	1014	13	64	15	1.533	6.402	1.571	Outer cell wall protein precursor
bin007	SOY3_bin007_02772	693	1	4	3	0.173	0.585	0.460	Ferrous iron transport protein B
bin007	SOY3_bin007_02773	630	5	15	6	0.949	2.415	1.012	Arginine-binding extracellular protein ArtP precursor

bin007	SOY3_bin007_02774	258	1	10	2	0.463	3.931	0.823	Endo-1,4-beta-xylanase A precursor
bin007	SOY3_bin007_02775	573	0	3	0	0.000	0.531	0.000	PD-(D/E)XK nuclease superfamily protein
bin007	SOY3_bin007_02776	267	1	0	0	0.448	0.000	0.000	CRISPR-associated endoribonuclease Cas2
bin007	SOY3_bin007_02777	357	2	0	0	0.670	0.000	0.000	CRISPR-associated endonuclease Cas1
bin007	SOY3_bin007_02778	777	4	17	5	0.615	2.219	0.684	ribulose-1,5-biphosphate synthetase
bin007	SOY3_bin007_02779	483	1	2	4	0.248	0.420	0.880	hypothetical protein
bin007	SOY3_bin007_02780	882	1	1	2	0.136	0.115	0.241	Tyrosine recombinase XerC
bin007	SOY3_bin007_02781	192	0	0	0	0.000	0.000	0.000	hypothetical protein
bin007	SOY3_bin007_02782	585	0	2	1	0.000	0.347	0.182	hypothetical protein
bin007	SOY3_bin007_02783	258	0	0	0	0.000	0.000	0.000	hypothetical protein
bin007	SOY3_bin007_02784	864	4	8	1	0.553	0.939	0.123	Nitrilase
bin007	SOY3_bin007_02785	903	2	25	7	0.265	2.808	0.823	preprotein translocase subunit SecF
bin007	SOY3_bin007_02786	207	2	3	2	1.155	1.470	1.026	bifunctional preprotein translocase subunit SecD/SecF
bin007	SOY3_bin007_02787	633	7	13	2	1.322	2.083	0.336	hypothetical protein
bin007	SOY3_bin007_02788	789	17	30	17	2.576	3.857	2.289	hypothetical protein
bin007	SOY3_bin007_02789	348	6	34	9	2.061	9.910	2.747	flagellar assembly protein H
bin007	SOY3_bin007_02790	591	2	13	4	0.405	2.231	0.719	hypothetical protein
bin007	SOY3_bin007_02791	756	17	150	28	2.688	20.124	3.934	flagellar assembly protein H
bin007	SOY3_bin007_02792	171	2	3	0	1.398	1.779	0.000	hypothetical protein
bin007	SOY3_bin007_02793	351	3	16	2	1.022	4.623	0.605	hypothetical protein
bin007	SOY3_bin007_02794	945	0	2	1	0.000	0.215	0.112	Benzoate membrane transport protein
bin007	SOY3_bin007_02795	669	2	11	1	0.357	1.668	0.159	hypothetical protein
bin007	SOY3_bin007_02796	291	4	8	4	1.643	2.788	1.460	IS2 repressor TnpA
bin007	SOY3_bin007_02797	318	6	6	1	2.256	1.914	0.334	IS2 transposase TnpB
bin007	SOY3_bin007_02798	435	0	11	2	0.000	2.565	0.488	hypothetical protein
bin007	SOY3_bin007_02799	165	0	2	0	0.000	1.229	0.000	hypothetical protein
bin007	SOY3_bin007_02800	249	1	1	1	0.480	0.407	0.427	hypothetical protein
bin007	SOY3_bin007_02801	1071	13	17	5	1.451	1.610	0.496	Inner membrane protein YbiR
bin007	SOY3_bin007_02802	540	27	28	10	5.977	5.259	1.967	N-formyl-4-amino-5-aminomethyl-2-methylpyrimidine deformylase
bin007	SOY3_bin007_02803	675	1	24	8	0.177	3.606	1.259	MotA/TolQ/ExbB proton channel family protein
bin007	SOY3_bin007_02804	204	0	9	12	0.000	4.475	6.249	hypothetical protein
bin007	SOY3_bin007_02805	1056	1	0	0	0.113	0.000	0.000	Glutamate synthase [NADPH] small chain
bin007	SOY3_bin007_02806	408	1	7	1	0.293	1.740	0.260	C4-dicarboxylate transporter/malic acid transport protein
bin007	SOY3_bin007_02807	420	2	4	2	0.569	0.966	0.506	Imidazole glycerol phosphate synthase subunit HisF
bin007	SOY3_bin007_02808	417	0	0	0	0.000	0.000	0.000	C4-dicarboxylate transporter/malic acid transport protein
bin007	SOY3_bin007_02809	420	0	3	0	0.000	0.724	0.000	Imidazole glycerol phosphate synthase subunit HisF
bin007	SOY3_bin007_02810	624	4	18	10	0.766	2.926	1.702	5' nucleotidase, deoxy (Pyrimidine), cytosolic type C protein (NT5C)
bin007	SOY3_bin007_02811	300	0	4	3	0.000	1.352	1.062	hypothetical protein
bin007	SOY3_bin007_02812	270	0	3	2	0.000	1.127	0.787	hypothetical protein
bin007	SOY3_bin007_02813	195	3	8	5	1.839	4.161	2.724	hypothetical protein
bin007	SOY3_bin007_02814	195	6	16	12	3.678	8.322	6.537	hypothetical protein
bin007	SOY3_bin007_02815	360	4	8	1	1.328	2.254	0.295	hypothetical protein
bin007	SOY3_bin007_02816	543	0	5	1	0.000	0.934	0.196	Spore maturation protein B
bin008	SOY3_bin008_00001	885	2	4	7	0.270	0.458	0.840	hypothetical protein
bin008	SOY3_bin008_00002	261	2	4	4	0.916	1.554	1.628	Toxin YoeB
bin008	SOY3_bin008_00003	225	6	6	6	3.188	2.705	2.833	Phd_YefM
bin008	SOY3_bin008_00004	1179	14	47	47	1.420	4.043	4.235	Tyrosine recombinase XerC
bin008	SOY3_bin008_00005	1131	34	132	172	3.594	11.838	16.155	Glutamine amidotransferases class-II
bin008	SOY3_bin008_00006	1635	10	105	105	0.731	6.514	6.822	Glutamate synthase [NADPH] large chain
bin008	SOY3_bin008_00007	2331	16	147	171	0.821	6.396	7.793	NAD-dependent dihydropyrimidine dehydrogenase subunit PreT
bin008	SOY3_bin008_00008	2109	5	22	15	0.283	1.058	0.756	Succinyl-CoA ligase [ADP-forming] subunit alpha
bin008	SOY3_bin008_00009	1065	8	27	49	0.898	2.571	4.887	Phosphate acetyltransferase
bin008	SOY3_bin008_00010	966	2	40	68	0.248	4.200	7.478	hypothetical protein
bin008	SOY3_bin008_00011	2085	280	625	683	16.055	30.404	34.797	Acetoin dehydrogenase operon transcriptional activator AcoR
bin008	SOY3_bin008_00012	1236	32	250	407	3.095	20.515	34.979	Dihydrolipoyllysine-residue acetyltransferase component of pyruvate dehydrogenase complex
bin008	SOY3_bin008_00013	429	4	83	152	1.115	19.623	37.637	hypothetical protein
bin008	SOY3_bin008_00014	963	63	681	1086	7.821	71.726	119.794	Acetoin:2,6-dichlorophenolindophenol oxidoreductase subunit alpha
bin008	SOY3_bin008_00015	972	61	581	963	7.503	60.627	105.242	Acetoin:2,6-dichlorophenolindophenol oxidoreductase subunit beta
bin008	SOY3_bin008_00016	996	29	197	276	3.481	20.061	29.436	ATP-NAD kinase
bin008	SOY3_bin008_00017	306	44	234	328	17.190	77.562	113.863	hypothetical protein
bin008	SOY3_bin008_00018	1275	19	145	179	1.782	11.535	14.913	Phosphomethylpyrimidine synthase
bin008	SOY3_bin008_00019	780	313	526	991	47.973	68.398	134.961	Diacetyl reductase [(S)-acetoin forming]
bin008	SOY3_bin008_00020	1341	14	115	140	1.248	8.698	11.090	Dihydrolipoyllysine-residue acetyltransferase component of pyruvate dehydrogenase complex
bin008	SOY3_bin008_00021	1377	21	187	256	1.823	13.774	19.749	Dihydrolipoyl dehydrogenase
bin008	SOY3_bin008_00022	1572	7	36	40	0.532	2.323	2.703	Lipoyl synthase
bin008	SOY3_bin008_00023	1797	11	59	87	0.732	3.330	5.143	Fumarate reductase flavoprotein subunit precursor
bin008	SOY3_bin008_00024	426	30	183	291	8.419	43.571	72.563	Glycine cleavage system H protein

bin008	SOY3_bin008_00025	1188	65	392	539	6.541	33.468	48.195	Anaerobic glycerol-3-phosphate dehydrogenase subunit C
bin008	SOY3_bin008_00026	1566	258	567	839	19.696	36.724	56.911	Lipoate-protein ligase LplJ
bin008	SOY3_bin008_00027	1068	14	47	69	1.567	4.464	6.863	putative monooxygenase VioC
bin008	SOY3_bin008_00028	945	12	32	53	1.518	3.435	5.958	biotin synthase
bin008	SOY3_bin008_00029	1149	42	138	192	4.370	12.182	17.751	biotin synthase
bin008	SOY3_bin008_00030	792	22	119	109	3.321	15.240	14.619	Putrescine oxidase
bin008	SOY3_bin008_00031	360	10	27	32	3.321	7.607	9.442	hypothetical protein
bin008	SOY3_bin008_00032	804	94	195	252	13.977	24.600	33.295	Phosphate-binding protein PstS precursor
bin008	SOY3_bin008_00033	894	24	38	45	3.209	4.311	5.347	Phosphate transport system permease protein PstC
bin008	SOY3_bin008_00034	879	12	36	44	1.632	4.154	5.317	Phosphate transport system permease protein PstA
bin008	SOY3_bin008_00035	1209	24	37	52	2.373	3.104	4.569	PhoH-like protein
bin008	SOY3_bin008_00036	177	1	4	3	0.675	2.292	1.800	hypothetical protein
bin008	SOY3_bin008_00037	3210	339	438	538	12.625	13.840	17.804	Sensor histidine kinase TmoS
bin008	SOY3_bin008_00038	540	77	125	165	17.047	23.479	32.458	hypothetical protein
bin008	SOY3_bin008_00039	1179	90	153	163	9.126	13.162	14.686	hypothetical protein
bin008	SOY3_bin008_00040	285	12	22	30	5.034	7.829	11.182	hypothetical protein
bin008	SOY3_bin008_00041	1653	55	82	120	3.978	5.031	7.711	Phosphate-specific transport system accessory protein PhoU
bin008	SOY3_bin008_00042	636	46	70	95	8.647	11.163	15.867	His Kinase A (phospho-acceptor) domain protein
bin008	SOY3_bin008_00043	354	19	71	77	6.416	20.343	23.106	Sporulation initiation phosphotransferase F
bin008	SOY3_bin008_00044	1743	38	125	125	2.606	7.274	7.618	Sensor protein ZraS
bin008	SOY3_bin008_00045	1230	35	156	186	3.402	12.864	16.063	Sporulation initiation phosphotransferase F
bin008	SOY3_bin008_00046	2130	15	74	80	0.842	3.524	3.990	putative sulfate transporter/MT1781
bin008	SOY3_bin008_00047	597	14	20	26	2.803	3.398	4.626	putative HTH-type transcriptional regulator YfiR
bin008	SOY3_bin008_00048	435	86	132	147	23.635	30.778	35.897	HTH-type transcriptional regulator CymR
bin008	SOY3_bin008_00049	2400	492	978	1213	24.508	41.332	53.688	Sporulation kinase E
bin008	SOY3_bin008_00050	1224	96	136	209	9.376	11.270	18.138	Hydrogenase transcriptional regulatory protein hupR1
bin008	SOY3_bin008_00051	1140	59	133	145	6.187	11.833	13.511	Membrane-bound lytic murein transglycosylase A precursor
bin008	SOY3_bin008_00052	381	169	323	491	53.028	85.987	136.895	hypothetical protein
bin008	SOY3_bin008_00053	375	16	31	30	5.101	8.385	8.498	hypothetical protein
bin008	SOY3_bin008_00054	1062	33	49	49	3.715	4.680	4.901	hypothetical protein
bin008	SOY3_bin008_00055	2472	145	175	205	7.012	7.180	8.809	Phosphoenolpyruvate synthase
bin008	SOY3_bin008_00056	369	41	55	62	13.283	15.118	17.848	Transcriptional regulatory protein ZraR
bin008	SOY3_bin008_00057	2049	50	106	113	2.917	5.247	5.858	Blue-light-activated protein
bin008	SOY3_bin008_00058	933	33	52	66	4.228	5.653	7.514	Phosphate-import protein PhnD precursor
bin008	SOY3_bin008_00059	570	13	42	54	2.727	7.474	10.063	hypothetical protein
bin008	SOY3_bin008_00060	1146	625	968	1384	65.199	85.673	128.287	Sulfite exporter TauE/SafE
bin008	SOY3_bin008_00061	504	2	4	3	0.474	0.805	0.632	hypothetical protein
bin008	SOY3_bin008_00062	1509	43	256	325	3.407	17.207	22.878	hypothetical protein
bin008	SOY3_bin008_00063	1671	871	1136	1430	62.314	68.954	90.905	Cytochrome c bacterial
bin008	SOY3_bin008_00064	660	463	493	704	83.865	75.763	113.307	putative Ni/Fe-hydrogenase B-type cytochrome subunit
bin008	SOY3_bin008_00065	1809	13	70	100	0.859	3.925	5.872	corrinoid ABC transporter substrate-binding protein
bin008	SOY3_bin008_00066	648	1	38	55	0.184	5.948	9.016	Demethylmenaquinone methyltransferase
bin008	SOY3_bin008_00067	870	2	19	13	0.275	2.215	1.587	putative ABC transporter ATP-binding protein
bin008	SOY3_bin008_00068	1050	3	15	30	0.342	1.449	3.035	putative ABC transporter permease protein
bin008	SOY3_bin008_00069	1065	5	37	24	0.561	3.524	2.394	Vitamin B12-binding protein precursor
bin008	SOY3_bin008_00070	2760	23	53	72	0.996	1.948	2.771	Autoinducer 2 sensor kinase/phosphatase LuxQ
bin008	SOY3_bin008_00071	660	2	10	12	0.362	1.537	1.931	Redox-sensing transcriptional repressor Rex
bin008	SOY3_bin008_00072	342	60	642	660	20.973	190.399	204.997	ATP synthase subunit c
bin008	SOY3_bin008_00073	717	11	144	115	1.834	20.370	17.038	ATP synthase subunit a
bin008	SOY3_bin008_00074	453	3	32	26	0.792	7.165	6.097	ATP synthase I chain
bin008	SOY3_bin008_00075	267	3	16	22	1.343	6.078	8.753	ATP synthase protein I
bin008	SOY3_bin008_00076	432	50	194	218	13.837	45.548	53.605	hypothetical protein
bin008	SOY3_bin008_00077	213	31	97	67	17.399	46.190	33.414	hypothetical protein
bin008	SOY3_bin008_00078	867	18	48	46	2.482	5.615	5.636	Oxygen-independent coproporphyrinogen-III oxidase 1
bin008	SOY3_bin008_00079	837	1	11	5	0.143	1.333	0.635	Sulfotransferase domain protein
bin008	SOY3_bin008_00080	2535	260	603	710	12.261	24.126	29.752	Lon protease
bin008	SOY3_bin008_00081	276	45	92	107	19.492	33.809	41.182	Acylphosphatase
bin008	SOY3_bin008_00082	687	57	113	149	9.919	16.683	23.039	hypothetical protein
bin008	SOY3_bin008_00083	99	0	2	3	0.000	2.049	3.219	hypothetical protein
bin008	SOY3_bin008_00084	993	26	38	81	3.130	3.881	8.665	DNA polymerase III subunit delta
bin008	SOY3_bin008_00085	2058	5	23	25	0.290	1.134	1.290	hypothetical protein
bin008	SOY3_bin008_00086	2049	67	172	199	3.909	8.514	10.317	Anaerobic ribonucleoside-triphosphate reductase
bin008	SOY3_bin008_00087	378	52	66	86	16.446	17.710	24.168	putative transcriptional regulatory protein pdtaR
bin008	SOY3_bin008_00088	1548	26	39	38	2.008	2.555	2.608	hypothetical protein
bin008	SOY3_bin008_00089	723	16	48	69	2.646	6.734	10.138	Galactoside O-acetyltransferase
bin008	SOY3_bin008_00090	879	22	81	101	2.992	9.347	12.206	hypothetical protein
bin008	SOY3_bin008_00091	1407	33	81	114	2.804	5.839	8.607	hypothetical protein

bin008	SOY3_bin008_00092	2478	8	68	69	0.386	2.783	2.958	Aerobic respiration control sensor protein ArcB
bin008	SOY3_bin008_00093	2667	15	192	214	0.672	7.302	8.524	Valine--tRNA ligase
bin008	SOY3_bin008_00094	633	6	85	81	1.133	13.620	13.593	Lipoprotein NlpI precursor
bin008	SOY3_bin008_00095	603	8	49	58	1.586	8.242	10.217	Endonuclease YncB precursor
bin008	SOY3_bin008_00096	2736	35	98	118	1.529	3.633	4.581	Phosphoenolpyruvate synthase
bin008	SOY3_bin008_00097	363	16	21	8	5.269	5.868	2.341	Transcriptional regulator PadR-like family protein
bin008	SOY3_bin008_00098	1164	20	40	45	2.054	3.485	4.107	putative chromate transport protein
bin008	SOY3_bin008_00099	300	8	20	34	3.188	6.762	12.039	Phytochrome-like protein cph2
bin008	SOY3_bin008_00100	801	31	95	99	4.627	12.029	13.129	hypothetical protein
bin008	SOY3_bin008_00101	2508	64	202	245	3.051	8.169	10.377	Lon protease
bin008	SOY3_bin008_00102	771	19	77	96	2.946	10.130	13.227	hypothetical protein
bin008	SOY3_bin008_00103	336	7	24	32	2.491	7.245	10.117	Acid stress chaperone HdeA
bin008	SOY3_bin008_00104	1218	8	27	31	0.785	2.248	2.704	Cyclic nucleotide-gated potassium channel
bin008	SOY3_bin008_00105	528	5	19	18	1.132	3.650	3.621	cAMP receptor protein
bin008	SOY3_bin008_00106	2067	90	110	136	5.205	5.398	6.989	Cyclic di-GMP phosphodiesterase Gmr
bin008	SOY3_bin008_00107	711	28	73	104	4.708	10.414	15.538	RlpA-like protein precursor
bin008	SOY3_bin008_00108	303	92	369	408	36.299	123.520	143.037	Integration host factor subunit alpha
bin008	SOY3_bin008_00109	423	94	368	522	26.566	88.239	131.087	HIT-like protein
bin008	SOY3_bin008_00110	1095	6	7	8	0.655	0.648	0.776	Oxygen-independent coproporphyrinogen-III oxidase 1
bin008	SOY3_bin008_00111	510	24	58	80	5.626	11.535	16.663	hypothetical protein
bin008	SOY3_bin008_00112	1191	27	41	62	2.710	3.492	5.530	Aminodeoxyfutalosine deaminase
bin008	SOY3_bin008_00113	624	21	67	67	4.023	10.890	11.406	dITP/XTP pyrophosphatase
bin008	SOY3_bin008_00114	1452	73	148	172	6.010	10.338	12.583	Sensor protein ZraS
bin008	SOY3_bin008_00115	1299	2	19	23	0.184	1.484	1.881	Ribosomal protein S12 methylthiotransferase RimO
bin008	SOY3_bin008_00116	1731	82	1180	1241	5.663	69.142	76.156	30S ribosomal protein S1
bin008	SOY3_bin008_00117	930	6	62	71	0.771	6.762	8.110	Putative signal peptide peptidase SppA
bin008	SOY3_bin008_00118	876	74	249	261	10.099	28.830	31.649	Ureidoglycolate lyase
bin008	SOY3_bin008_00119	933	38	123	124	4.869	13.371	14.118	membrane-bound lytic murein transglycosylase D
bin008	SOY3_bin008_00120	1038	3326	12805	14238	383.063	1251.230	1457.073	Outer membrane protein P6 precursor
bin008	SOY3_bin008_00121	2913	26	101	108	1.067	3.517	3.938	Peptidase M16C associated
bin008	SOY3_bin008_00122	1173	5	35	47	0.510	3.026	4.256	Putative oxidoreductase/MT0587
bin008	SOY3_bin008_00123	729	42	78	94	6.888	10.852	13.697	Phosphoglycolate phosphatase
bin008	SOY3_bin008_00124	753	15	32	55	2.381	4.310	7.759	hypothetical protein
bin008	SOY3_bin008_00125	813	57	89	107	8.382	11.103	13.981	Tetratricopeptide repeat protein
bin008	SOY3_bin008_00126	2598	18	59	81	0.828	2.303	3.312	DNA polymerase I, thermostable
bin008	SOY3_bin008_00127	1545	50	285	347	3.869	18.710	23.858	Outer membrane protein OprM precursor
bin008	SOY3_bin008_00128	192	1	11	7	0.623	5.811	3.873	hypothetical protein
bin008	SOY3_bin008_00129	1278	9	55	60	0.842	4.365	4.987	Serine--tRNA ligase
bin008	SOY3_bin008_00130	501	9	16	27	2.148	3.239	5.725	Nicotinamide-nucleotide amidohydrolase PncC
bin008	SOY3_bin008_00131	258	7	6	9	3.244	2.359	3.706	hypothetical protein
bin008	SOY3_bin008_00132	540	14	50	46	3.099	9.391	9.049	hypothetical protein
bin008	SOY3_bin008_00133	1002	61	154	187	7.278	15.589	19.825	Respiratory nitrate reductase 1 gamma chain
bin008	SOY3_bin008_00134	1614	92	231	278	6.814	14.517	18.297	succinate dehydrogenase/fumarate reductase iron-sulfur subunit
bin008	SOY3_bin008_00135	417	23	100	98	6.594	24.323	24.964	hypothetical protein
bin008	SOY3_bin008_00136	777	37	104	116	5.693	13.576	15.859	Tetrathionate reductase subunit B precursor
bin008	SOY3_bin008_00137	1188	49	136	133	4.931	11.611	11.892	Polysulphide reductase, NrfD
bin008	SOY3_bin008_00138	582	28	92	103	5.751	16.033	18.799	Glutathione transferase FosA
bin008	SOY3_bin008_00139	852	6	39	31	0.842	4.643	3.865	Diaminopimelate epimerase
bin008	SOY3_bin008_00140	1767	8	17	22	0.541	0.976	1.323	Aminodeoxychorismate synthase component 1
bin008	SOY3_bin008_00141	1002	45	133	176	5.369	13.463	18.658	putative lipoprotein YiaD precursor
bin008	SOY3_bin008_00142	2481	105	292	334	5.060	11.937	14.300	Maltodextrin phosphorylase
bin008	SOY3_bin008_00143	216	42	306	354	23.246	143.689	174.092	hypothetical protein
bin008	SOY3_bin008_00144	1173	30	99	129	3.058	8.560	11.682	DNA polymerase IV
bin008	SOY3_bin008_00145	1011	57	135	139	6.740	13.544	14.605	Transcriptional regulatory protein ZraR
bin008	SOY3_bin008_00146	1722	83	215	203	5.762	12.664	12.523	Wide host range VirA protein
bin008	SOY3_bin008_00147	489	17	34	44	4.156	7.052	9.558	hypothetical protein
bin008	SOY3_bin008_00148	792	24	73	74	3.623	9.349	9.925	Phosphatidate cytidylyltransferase
bin008	SOY3_bin008_00149	1536	39	108	126	3.035	7.132	8.714	hypothetical protein
bin008	SOY3_bin008_00150	1278	15	33	46	1.403	2.619	3.823	1-deoxy-D-xylulose 5-phosphate reductoisomerase
bin008	SOY3_bin008_00151	1080	21	81	91	2.325	7.607	8.951	Metalloprotease MmpA
bin008	SOY3_bin008_00152	777	28	75	74	4.308	9.790	10.117	tRNA threonylcarbamoyladenosine biosynthesis protein TsaB
bin008	SOY3_bin008_00153	900	67	429	535	8.900	48.347	63.145	Flagellar filament 33 kDa core protein
bin008	SOY3_bin008_00154	1689	29	158	198	2.053	9.488	12.453	Flagellar hook-associated protein 2
bin008	SOY3_bin008_00155	726	40	157	177	6.587	21.934	25.898	Flagellar protein FliS
bin008	SOY3_bin008_00156	2511	8	88	84	0.381	3.555	3.554	Leucine--tRNA ligase
bin008	SOY3_bin008_00157	498	8	24	34	1.920	4.888	7.252	hypothetical protein
bin008	SOY3_bin008_00158	471	7	30	31	1.777	6.460	6.992	6,7-dimethyl-8-ribityllumazine synthase

bin008	SOY3_bin008_00159	1227	13	97	97	1.267	8.018	8.398	Riboflavin biosynthesis protein RibBA
bin008	SOY3_bin008_00160	477	4	23	32	1.003	4.891	7.126	Glyoxalase-like domain protein
bin008	SOY3_bin008_00161	660	6	26	28	1.087	3.996	4.507	Riboflavin synthase
bin008	SOY3_bin008_00162	489	3	26	15	0.733	5.393	3.258	Methylated-DNA--protein-cysteine methyltransferase
bin008	SOY3_bin008_00163	915	6	36	38	0.784	3.991	4.412	(Dimethylallyl)adenosine tRNA methylthiotransferase MiaB
bin008	SOY3_bin008_00164	1041	73	245	286	8.383	23.871	29.184	Rod shape-determining protein MreB
bin008	SOY3_bin008_00165	927	23	82	110	2.966	8.972	12.605	Cell shape-determining protein MreC
bin008	SOY3_bin008_00166	471	6	25	31	1.523	5.384	6.992	hypothetical protein
bin008	SOY3_bin008_00167	1890	21	69	65	1.328	3.703	3.653	Stage V sporulation protein D
bin008	SOY3_bin008_00168	1113	12	32	41	1.289	2.916	3.913	Rod shape-determining protein RodA
bin008	SOY3_bin008_00169	387	2	15	16	0.618	3.931	4.392	Polymer-forming cytoskeletal
bin008	SOY3_bin008_00170	426	39	152	151	10.945	36.190	37.653	ATP synthase subunit b, sodium ion specific
bin008	SOY3_bin008_00171	573	50	269	257	10.432	47.616	47.644	ATP synthase subunit b
bin008	SOY3_bin008_00172	552	42	222	194	9.096	40.791	37.333	ATP synthase subunit delta
bin008	SOY3_bin008_00173	1509	150	746	734	11.884	50.142	51.670	ATP synthase subunit alpha
bin008	SOY3_bin008_00174	882	80	387	387	10.843	44.504	46.609	ATP synthase gamma chain
bin008	SOY3_bin008_00175	1413	158	1042	1106	13.368	74.796	83.146	ATP synthase subunit beta
bin008	SOY3_bin008_00176	426	56	389	433	15.715	92.618	107.971	ATP synthase epsilon chain
bin008	SOY3_bin008_00177	450	14	68	86	3.719	15.327	20.301	ATP synthase epsilon chain
bin008	SOY3_bin008_00178	1365	34	117	138	2.978	8.694	10.739	Bifunctional protein GlmU
bin008	SOY3_bin008_00179	180	85	242	282	56.454	136.363	166.420	Cell division protein ZapB
bin008	SOY3_bin008_00180	255	124	306	321	58.134	121.713	133.719	Cell division protein ZapA
bin008	SOY3_bin008_00181	1560	76	303	339	5.824	19.700	23.084	Ribonuclease Y
bin008	SOY3_bin008_00182	789	4	14	29	0.606	1.800	3.904	hypothetical protein
bin008	SOY3_bin008_00183	477	4	35	37	1.003	7.442	8.240	hypothetical protein
bin008	SOY3_bin008_00184	351	1	22	33	0.341	6.357	9.987	Sporulation related domain protein
bin008	SOY3_bin008_00185	1440	16	56	64	1.328	3.944	4.721	Tyrosine--tRNA ligase
bin008	SOY3_bin008_00186	1851	54	152	212	3.488	8.329	12.166	Electron transfer flavoprotein-ubiquinone oxidoreductase
bin008	SOY3_bin008_00187	2049	52	187	239	3.034	9.257	12.390	Acryloyl-CoA reductase (NADH)
bin008	SOY3_bin008_00188	1113	120	202	265	12.889	18.408	25.292	hypothetical protein
bin008	SOY3_bin008_00189	267	55	91	100	24.626	34.569	39.785	hypothetical protein
bin008	SOY3_bin008_00190	363	16	34	46	5.269	9.500	13.461	Putative anti-sigma factor antagonist
bin008	SOY3_bin008_00191	237	35	125	146	17.655	53.495	65.439	hypothetical protein
bin008	SOY3_bin008_00192	663	143	508	695	25.785	77.715	111.353	Inosine-5'-monophosphate dehydrogenase
bin008	SOY3_bin008_00193	1215	4	3	12	0.394	0.250	1.049	D-arabitol-phosphate dehydrogenase
bin008	SOY3_bin008_00194	2052	1	6	16	0.058	0.297	0.828	hypothetical protein
bin008	SOY3_bin008_00195	462	22	74	92	5.693	16.246	21.153	putative kinase inhibitor protein
bin008	SOY3_bin008_00196	1275	84	305	333	7.876	24.263	27.744	Fructose-bisphosphate aldolase
bin008	SOY3_bin008_00197	441	25	72	89	6.777	16.560	21.438	hypothetical protein
bin008	SOY3_bin008_00198	798	46	207	275	6.891	26.310	36.607	Disulfide bond formation protein D precursor
bin008	SOY3_bin008_00199	1155	12	34	38	1.242	2.986	3.495	rare lipoprotein A
bin008	SOY3_bin008_00200	1368	30	102	116	2.622	7.563	9.007	TPR repeat-containing protein YrrB
bin008	SOY3_bin008_00201	1221	157	263	321	15.372	21.847	27.927	Cyclic di-GMP phosphodiesterase response regulator RpfG
bin008	SOY3_bin008_00202	1716	116	252	296	8.081	14.895	18.323	Wide host range VirA protein
bin008	SOY3_bin008_00203	828	6	13	28	0.866	1.592	3.592	bacteriophage N4 adsorption protein B
bin008	SOY3_bin008_00204	816	7	24	26	1.026	2.983	3.385	tetratricopeptide repeat protein
bin008	SOY3_bin008_00205	1200	61	147	178	6.077	12.425	15.757	Hydrogenase transcriptional regulatory protein hupR1
bin008	SOY3_bin008_00206	297	6	25	33	2.415	8.538	11.803	GGDEF domain protein
bin008	SOY3_bin008_00207	1278	24	221	201	2.245	17.539	16.707	8-amino-7-oxononanoate synthase
bin008	SOY3_bin008_00208	1251	4	61	45	0.382	4.946	3.821	Macrolide export ATP-binding/permease protein MacB
bin008	SOY3_bin008_00209	717	20	93	93	3.335	13.156	13.778	ABC transporter ATP-binding protein YtrE
bin008	SOY3_bin008_00210	1668	51	269	289	3.655	16.357	18.405	Outer membrane efflux protein
bin008	SOY3_bin008_00211	948	40	121	132	5.044	12.946	14.791	Macrolide export protein MacA
bin008	SOY3_bin008_00212	1146	23	62	62	2.399	5.487	5.747	hypothetical protein
bin008	SOY3_bin008_00213	1656	3	9	11	0.217	0.551	0.706	hypothetical protein
bin008	SOY3_bin008_00214	831	7	21	27	1.007	2.563	3.451	Phosphoadenosine phosphosulfate reductase
bin008	SOY3_bin008_00215	342	7	24	31	2.447	7.118	9.629	hypothetical protein
bin008	SOY3_bin008_00216	732	27	99	92	4.410	13.718	13.351	Malonyl-[acyl-carrier protein] O-methyltransferase
bin008	SOY3_bin008_00217	1677	47	233	281	3.351	14.092	17.799	Dihydroxy-acid dehydratase
bin008	SOY3_bin008_00218	1011	22	51	54	2.601	5.117	5.674	Glycosyl transferases group 1
bin008	SOY3_bin008_00219	387	11	42	39	3.398	11.008	10.705	hypothetical protein
bin008	SOY3_bin008_00220	642	11	59	64	2.048	9.321	10.589	hypothetical protein
bin008	SOY3_bin008_00221	912	14	53	61	1.835	5.894	7.105	mce related protein
bin008	SOY3_bin008_00222	855	12	30	39	1.678	3.559	4.845	putative ABC transporter ATP-binding protein
bin008	SOY3_bin008_00223	771	4	20	19	0.620	2.631	2.618	putative phospholipid ABC transporter permease protein MlaE
bin008	SOY3_bin008_00224	333	6	40	55	2.154	12.183	17.545	Anti-sigma-B factor antagonist
bin008	SOY3_bin008_00225	1047	21	55	58	2.398	5.328	5.885	Bifunctional protein HldE

bin008	SOY3_bin008_00226	942	10	42	47	1.269	4.522	5.300	Chromosome-partitioning protein Spo0J
bin008	SOY3_bin008_00227	432	2	36	35	0.553	8.452	8.606	hypothetical protein
bin008	SOY3_bin008_00228	777	4	11	24	0.615	1.436	3.281	Chromosome partitioning protein ParA
bin008	SOY3_bin008_00229	291	11	77	80	4.519	26.838	29.203	hypothetical protein
bin008	SOY3_bin008_00230	1008	44	89	133	5.218	8.955	14.016	UDP-glucose 4-epimerase
bin008	SOY3_bin008_00231	840	149	257	330	21.206	31.032	41.732	Modulator of FtsH protease HflK
bin008	SOY3_bin008_00232	1338	23	63	89	2.055	4.776	7.066	hypothetical protein
bin008	SOY3_bin008_00233	591	5	20	43	1.011	3.432	7.729	hypothetical protein
bin008	SOY3_bin008_00234	1221	21	77	63	2.056	6.396	5.481	Coenzyme A biosynthesis bifunctional protein CoaBC
bin008	SOY3_bin008_00235	741	2	10	11	0.323	1.369	1.577	hypothetical protein
bin008	SOY3_bin008_00236	879	2	34	34	0.272	3.923	4.109	hypothetical protein
bin008	SOY3_bin008_00237	795	10	34	39	1.504	4.338	5.211	hypothetical protein
bin008	SOY3_bin008_00238	2433	29	100	96	1.425	4.169	4.191	DNA gyrase subunit A
bin008	SOY3_bin008_00239	2415	17	93	96	0.842	3.906	4.223	DNA gyrase subunit B
bin008	SOY3_bin008_00240	1191	6	54	67	0.602	4.599	5.976	DNA polymerase III subunit beta
bin008	SOY3_bin008_00241	1314	11	30	46	1.001	2.316	3.719	Chromosomal replication initiator protein DnaA
bin008	SOY3_bin008_00242	1167	10	71	87	1.024	6.171	7.919	hypothetical protein
bin008	SOY3_bin008_00243	837	0	8	4	0.000	0.969	0.508	fructoselysine 3-epimerase
bin008	SOY3_bin008_00244	750	2	33	21	0.319	4.463	2.974	hypothetical protein
bin008	SOY3_bin008_00245	2142	24	145	134	1.339	6.866	6.645	L,D-transpeptidase catalytic domain
bin008	SOY3_bin008_00246	597	13	61	67	2.603	10.364	11.921	Orotate phosphoribosyltransferase
bin008	SOY3_bin008_00247	1293	15	106	148	1.387	8.315	12.159	Adenylosuccinate lyase
bin008	SOY3_bin008_00248	354	7	64	53	2.364	18.337	15.904	Zinc ribbon domain protein
bin008	SOY3_bin008_00249	114	11	57	58	11.535	50.714	54.045	hypothetical protein
bin008	SOY3_bin008_00250	1065	1	23	18	0.112	2.190	1.795	undecaprenyldiphospho-muramoylpentapeptide beta-N- acetylglucosaminyltransferase
bin008	SOY3_bin008_00251	2289	7	69	53	0.366	3.057	2.460	DNA topoisomerase 1
bin008	SOY3_bin008_00252	885	62	152	178	8.375	17.420	21.365	GTPase Era
bin008	SOY3_bin008_00253	693	83	229	271	14.318	33.516	41.540	hypothetical protein
bin008	SOY3_bin008_00254	582	37	107	119	7.600	18.647	21.720	flagellar basal body-associated protein FliL
bin008	SOY3_bin008_00255	582	35	110	142	7.189	19.170	25.918	Flagellar motor switch protein FliN
bin008	SOY3_bin008_00256	387	12	41	51	3.707	10.746	13.999	Flagellar protein FliO
bin008	SOY3_bin008_00257	657	5	26	43	0.910	4.014	6.952	Flagellar biosynthetic protein FliP precursor
bin008	SOY3_bin008_00258	270	5	8	11	2.214	3.005	4.328	Flagellar biosynthetic protein FliQ
bin008	SOY3_bin008_00259	270	49	153	173	21.696	57.475	68.063	Glutaredoxin-like protein NrdH
bin008	SOY3_bin008_00260	1416	92	184	253	7.767	13.180	18.980	Major cardiolipin synthase ClsA
bin008	SOY3_bin008_00261	603	21	72	94	4.163	12.111	16.559	PRC-barrel domain protein
bin008	SOY3_bin008_00262	1269	68	131	179	6.406	10.470	14.984	hypothetical protein
bin008	SOY3_bin008_00263	855	21	60	82	2.936	7.118	10.188	Molybdopterin-synthase adenylyltransferase
bin008	SOY3_bin008_00264	225	5	15	16	2.657	6.762	7.554	ThiS family protein
bin008	SOY3_bin008_00265	300	7	35	31	2.789	11.833	10.977	hypothetical protein
bin008	SOY3_bin008_00266	525	25	77	106	5.693	14.876	21.447	putative acyl-CoA thioester hydrolase
bin008	SOY3_bin008_00267	237	7	56	55	3.531	23.966	24.652	hypothetical protein
bin008	SOY3_bin008_00268	927	30	62	86	3.869	6.784	9.855	putative periplasmic serine endoprotease DegP-like precursor
bin008	SOY3_bin008_00269	597	9	26	33	1.802	4.417	5.872	CheY-P phosphatase CheC
bin008	SOY3_bin008_00270	2028	38	70	94	2.240	3.501	4.924	putative diguanylate cyclase
bin008	SOY3_bin008_00271	1029	58	129	139	6.738	12.715	14.349	phosphodiesterase
bin008	SOY3_bin008_00272	609	19	25	26	3.730	4.164	4.535	hypothetical protein
bin008	SOY3_bin008_00273	417	7	32	22	2.007	7.783	5.604	hypothetical protein
bin008	SOY3_bin008_00274	390	5	45	38	1.533	11.703	10.350	Nitrogen assimilation regulatory protein
bin008	SOY3_bin008_00275	804	58	141	169	8.624	17.788	22.329	3'(2'),5'-bisphosphate nucleotidase CysQ
bin008	SOY3_bin008_00276	282	11	59	73	4.663	21.221	27.498	Circadian clock protein KaiB
bin008	SOY3_bin008_00277	1041	50	114	143	5.742	11.107	14.592	Putative agmatine deiminase
bin008	SOY3_bin008_00278	1377	49	169	241	4.254	12.448	18.591	hypothetical protein
bin008	SOY3_bin008_00279	288	3	22	20	1.245	7.748	7.377	EF hand
bin008	SOY3_bin008_00280	1008	26	67	74	3.084	6.742	7.798	copper/silver efflux system membrane fusion protein CusB
bin008	SOY3_bin008_00281	516	6	10	15	1.390	1.966	3.088	Chemoreceptor glutamine deamidase CheD
bin008	SOY3_bin008_00282	441	8	10	8	2.169	2.300	1.927	Chemotaxis protein CheY
bin008	SOY3_bin008_00283	1701	18	50	57	1.265	2.981	3.560	Chemotaxis protein CheA
bin008	SOY3_bin008_00284	279	10	26	22	4.285	9.452	8.376	hypothetical protein
bin008	SOY3_bin008_00285	471	22	85	78	5.584	18.304	17.592	hypothetical protein
bin008	SOY3_bin008_00286	507	23	54	61	5.423	10.803	12.781	hypothetical protein
bin008	SOY3_bin008_00287	1092	10	29	38	1.095	2.694	3.697	Spore photoproduct lyase
bin008	SOY3_bin008_00288	996	14	18	31	1.680	1.833	3.306	hypothetical protein
bin008	SOY3_bin008_00289	2073	50	725	751	2.883	35.473	38.483	Elongation factor G
bin008	SOY3_bin008_00290	1092	5	32	38	0.547	2.972	3.697	hypothetical protein
bin008	SOY3_bin008_00291	1404	7	46	37	0.596	3.323	2.799	Transcriptional regulatory protein ZraR
bin008	SOY3_bin008_00292	1128	5	39	46	0.530	3.507	4.332	Antilisterial bacteriocin subtilosin biosynthesis protein AlbA

bin008	SOY3_bin008_00293	1053	92	258	372	10.445	24.851	37.527	Fructose-bisphosphate aldolase class 1
bin008	SOY3_bin008_00294	2106	187	352	431	10.615	16.953	21.739	Chemotaxis protein CheA
bin008	SOY3_bin008_00295	234	6	14	8	3.065	6.068	3.632	hypothetical protein
bin008	SOY3_bin008_00296	447	18	40	56	4.814	9.076	13.308	Ferric uptake regulation protein
bin008	SOY3_bin008_00297	699	27	43	51	4.618	6.239	7.750	Inward rectifier potassium channel Kirbac3.1
bin008	SOY3_bin008_00298	1812	46	62	74	3.035	3.470	4.338	putative sulfate transporter/MT1781
bin008	SOY3_bin008_00299	438	23	43	51	6.278	9.957	12.369	Thioredoxin-2
bin008	SOY3_bin008_00300	879	0	6	7	0.000	0.692	0.846	Inner membrane protein YtfF
bin008	SOY3_bin008_00301	372	5	74	70	1.607	20.176	19.989	DNA-directed RNA polymerase subunit P
bin008	SOY3_bin008_00302	2604	3	30	28	0.138	1.169	1.142	Cobyric acid synthase
bin008	SOY3_bin008_00303	1071	3	21	32	0.335	1.989	3.174	Methyl-accepting chemotaxis protein McpC
bin008	SOY3_bin008_00304	435	1	10	7	0.275	2.332	1.709	hypothetical protein
bin008	SOY3_bin008_00305	1632	608	1170	1427	44.538	72.714	92.882	Isopenicillin N epimerase
bin008	SOY3_bin008_00306	1641	122	1358	1409	8.888	83.936	91.208	60 kDa chaperonin 1
bin008	SOY3_bin008_00307	291	31	288	286	12.735	100.382	104.401	10 kDa chaperonin
bin008	SOY3_bin008_00308	1923	458	2322	2449	28.473	122.472	135.282	Na(+)/H(+) antiporter subunit A
bin008	SOY3_bin008_00309	855	152	754	773	21.253	89.446	96.038	Formate hydrogenlyase subunit 4
bin008	SOY3_bin008_00310	444	74	496	429	19.925	113.306	102.637	Formate hydrogenlyase subunit 7
bin008	SOY3_bin008_00311	366	74	458	493	24.171	126.923	143.086	NADH-quinone oxidoreductase subunit C
bin008	SOY3_bin008_00312	1227	287	1636	1660	27.963	135.236	143.712	Formate hydrogenlyase subunit 5 precursor
bin008	SOY3_bin008_00313	366	133	734	749	43.443	203.409	217.385	NAD(P)H-quinone oxidoreductase subunit I
bin008	SOY3_bin008_00314	891	5	18	12	0.671	2.049	1.431	DNA polymerase III subunit tau
bin008	SOY3_bin008_00315	1317	59	228	267	5.356	17.559	21.536	Adenylosuccinate synthetase
bin008	SOY3_bin008_00316	342	36	81	111	12.584	24.022	34.477	hypothetical protein
bin008	SOY3_bin008_00317	1281	7	101	87	0.653	7.997	7.214	Bifunctional purine biosynthesis protein PurH
bin008	SOY3_bin008_00318	2124	12	59	49	0.675	2.817	2.451	Ribonuclease R
bin008	SOY3_bin008_00319	603	19	41	52	3.767	6.896	9.160	putative glycerol-3-phosphate acyltransferase
bin008	SOY3_bin008_00320	375	13	32	35	4.144	8.655	9.914	hypothetical protein
bin008	SOY3_bin008_00321	537	8	35	30	1.781	6.611	5.934	Tetratricopeptide repeat protein
bin008	SOY3_bin008_00322	1266	46	155	188	4.344	12.418	15.774	Ribonuclease Y
bin008	SOY3_bin008_00323	192	7	12	18	4.359	6.339	9.959	hypothetical protein
bin008	SOY3_bin008_00324	204	22	57	58	12.893	28.340	30.201	hypothetical protein
bin008	SOY3_bin008_00325	870	107	189	261	14.703	22.034	31.868	Universal stress protein family protein
bin008	SOY3_bin008_00326	189	8	26	19	5.060	13.953	10.679	Virus attachment protein p12 family protein
bin008	SOY3_bin008_00327	1290	12	28	15	1.112	2.202	1.235	succinate dehydrogenase/fumarate reductase iron-sulfur subunit
bin008	SOY3_bin008_00328	705	2	11	7	0.339	1.583	1.055	Nitrate reductase gamma subunit
bin008	SOY3_bin008_00329	552	5	10	8	1.083	1.837	1.540	Dihaem cytochrome c
bin008	SOY3_bin008_00330	681	4	24	16	0.702	3.575	2.496	Phosphoribosylglycinamide formyltransferase
bin008	SOY3_bin008_00331	1518	2	82	73	0.158	5.479	5.108	Uroporphyrinogen-III C-methyltransferase
bin008	SOY3_bin008_00332	369	0	3	3	0.000	0.825	0.864	hypothetical protein
bin008	SOY3_bin008_00333	3747	15	69	66	0.479	1.868	1.871	putative assembly protein
bin008	SOY3_bin008_00334	1686	26	34	48	1.844	2.045	3.024	Spore protein YkvP
bin008	SOY3_bin008_00335	771	27	104	136	4.187	13.682	18.738	Immunoglobulin G-binding protein A precursor
bin008	SOY3_bin008_00336	1641	16	40	48	1.166	2.472	3.107	N-acetylglucosaminyl-diphospho-decaprenol L-rhamnosyltransferase
bin008	SOY3_bin008_00337	870	8	30	37	1.099	3.497	4.518	Murein DD-endopeptidase MepM
bin008	SOY3_bin008_00338	2541	37	86	109	1.741	3.433	4.557	putative ABC transporter ATP-binding protein
bin008	SOY3_bin008_00339	531	7	23	31	1.576	4.393	6.202	Cyclic nucleotide-binding domain protein
bin008	SOY3_bin008_00340	774	15	45	44	2.317	5.897	6.039	Trans-aconitate 2-methyltransferase
bin008	SOY3_bin008_00341	849	4	30	29	0.563	3.584	3.628	Glycine/sarcosine/dimethylglycine N-methyltransferase
bin008	SOY3_bin008_00342	95	3	2	2	3.775	2.135	2.236	tRNA-Ile(gat)
bin008	SOY3_bin008_00343	330	37	89	135	13.404	27.355	43.456	aerobic respiration control sensor protein ArcB
bin008	SOY3_bin008_00344	2028	90	141	238	5.305	7.052	12.466	Autoinducer 2 sensor kinase/phosphatase LuxQ
bin008	SOY3_bin008_00345	675	38	85	121	6.730	12.772	19.042	Ribulose-phosphate 3-epimerase
bin008	SOY3_bin008_00346	729	12	26	16	1.968	3.617	2.331	putative 2-phosphosulfolactate phosphatase
bin008	SOY3_bin008_00347	984	4	25	26	0.486	2.577	2.807	Cyclic di-GMP phosphodiesterase response regulator RpfG
bin008	SOY3_bin008_00348	213	1	11	24	0.561	5.238	11.969	hypothetical protein
bin008	SOY3_bin008_00349	264	6	25	40	2.717	9.605	16.095	hypothetical protein
bin008	SOY3_bin008_00350	1233	21	99	94	2.036	8.144	8.098	Phytochrome-like protein cph2
bin008	SOY3_bin008_00351	1224	1	10	13	0.098	0.829	1.128	putative calcium/sodium:proton antiporter
bin008	SOY3_bin008_00352	669	0	14	4	0.000	2.123	0.635	hypothetical protein
bin008	SOY3_bin008_00353	366	1	17	17	0.327	4.711	4.934	Dinitrogenase iron-molybdenum cofactor
bin008	SOY3_bin008_00354	981	57	298	312	6.946	30.811	33.784	Delta-aminolevulinic acid dehydratase
bin008	SOY3_bin008_00355	1245	114	448	542	10.947	36.498	46.244	phosphodiesterase
bin008	SOY3_bin008_00356	276	3	20	19	1.299	7.350	7.313	hypothetical protein
bin008	SOY3_bin008_00357	1212	7	14	16	0.690	1.172	1.402	hypothetical protein
bin008	SOY3_bin008_00358	777	20	45	48	3.077	5.874	6.562	hypothetical protein
bin008	SOY3_bin008_00359	1098	80	145	159	8.710	13.394	15.382	tol-pal system protein YbgF

bin008	SOY3_bin008_00360	933	1	20	30	0.128	2.174	3.416	hypothetical protein
bin008	SOY3_bin008_00361	672	3	11	15	0.534	1.660	2.371	hypothetical protein
bin008	SOY3_bin008_00362	567	4	9	11	0.843	1.610	2.061	HTH-type transcriptional repressor NicS
bin008	SOY3_bin008_00363	306	165	468	682	64.463	155.124	236.752	Putative monooxygenase
bin008	SOY3_bin008_00364	1200	18	48	61	1.793	4.057	5.400	Alkaline phosphatase synthesis sensor protein PhoR
bin008	SOY3_bin008_00365	300	37	83	99	14.744	28.062	35.054	Acetolactate synthase isozyme 1 small subunit
bin008	SOY3_bin008_00366	1689	172	254	406	12.174	15.253	25.534	Acetolactate synthase isozyme 1 large subunit
bin008	SOY3_bin008_00367	597	405	455	639	81.101	77.302	113.699	HTH-type transcriptional repressor KstR2
bin008	SOY3_bin008_00368	3012	97	144	211	3.850	4.849	7.441	PD-(D/E)XK nuclease superfamily protein
bin008	SOY3_bin008_00369	3186	52	150	194	1.951	4.775	6.468	ATP-dependent helicase/nuclease subunit A
bin008	SOY3_bin008_00370	1020	16	18	29	1.875	1.790	3.020	Ribosomal large subunit pseudouridine synthase D
bin008	SOY3_bin008_00371	183	10	26	49	6.533	14.410	28.443	Anti-sigma-28 factor, FlgM
bin008	SOY3_bin008_00372	678	30	120	134	5.290	17.952	20.994	putative HTH-type transcriptional regulator
bin008	SOY3_bin008_00373	165	18	52	37	13.042	31.965	23.820	hypothetical protein
bin008	SOY3_bin008_00374	378	6	34	32	1.898	9.123	8.993	flagellar basal body-associated protein FliL
bin008	SOY3_bin008_00375	645	6	24	24	1.112	3.774	3.953	Glucosyl-3-phosphoglycerate phosphatase
bin008	SOY3_bin008_00376	447	15	72	90	4.012	16.337	21.388	hypothetical protein
bin008	SOY3_bin008_00377	654	20	95	135	3.656	14.733	21.927	hypothetical protein
bin008	SOY3_bin008_00378	714	53	128	150	8.874	18.183	22.316	hypothetical protein
bin008	SOY3_bin008_00379	1158	30	90	137	3.097	7.883	12.567	hypothetical protein
bin008	SOY3_bin008_00380	1308	97	190	217	8.866	14.733	17.623	phosphodiesterase
bin008	SOY3_bin008_00381	1410	60	162	197	5.087	11.653	14.841	Transcriptional regulatory protein ZraR
bin008	SOY3_bin008_00382	1023	9	107	127	1.052	10.609	13.187	flagellar basal body P-ring biosynthesis protein FlgA
bin008	SOY3_bin008_00383	1452	24	138	207	1.976	9.640	15.144	Putative type II secretion system protein D precursor
bin008	SOY3_bin008_00384	270	8	44	60	3.542	16.529	23.606	hypothetical protein
bin008	SOY3_bin008_00385	1176	71	218	242	7.218	18.802	21.859	Sporulation initiation inhibitor protein Soj
bin008	SOY3_bin008_00386	1368	18	85	113	1.573	6.302	8.774	Putative conjugal transfer protein/MT3759
bin008	SOY3_bin008_00387	984	8	45	54	0.972	4.638	5.829	Bacterial type II secretion system protein F domain protein
bin008	SOY3_bin008_00388	948	5	31	37	0.631	3.317	4.146	Bacterial type II secretion system protein F domain protein
bin008	SOY3_bin008_00389	330	10	32	29	3.623	9.835	9.335	hypothetical protein
bin008	SOY3_bin008_00390	483	10	36	44	2.475	7.560	9.677	TadE-like protein
bin008	SOY3_bin008_00391	1290	39	64	98	3.614	5.032	8.070	von Willebrand factor type A domain protein
bin008	SOY3_bin008_00392	429	7	28	29	1.951	6.620	7.181	TadE-like protein
bin008	SOY3_bin008_00393	1101	11	30	38	1.194	2.764	3.666	photosystem I assembly protein Ycf3
bin008	SOY3_bin008_00394	2136	29	71	100	1.623	3.371	4.973	Blue-light-activated protein
bin008	SOY3_bin008_00395	1587	16	45	74	1.205	2.876	4.953	DNA translocase FtsK
bin008	SOY3_bin008_00396	699	40	101	110	6.841	14.655	16.716	Soluble lytic murein transglycosylase precursor
bin008	SOY3_bin008_00397	492	21	32	47	5.103	6.597	10.148	Putative peroxiredoxin bcp
bin008	SOY3_bin008_00398	990	46	66	91	5.555	6.762	9.764	pyrroloquinoline quinone biosynthesis protein PqqE
bin008	SOY3_bin008_00399	1689	75	345	432	5.309	20.718	27.170	Thioredoxin reductase
bin008	SOY3_bin008_00400	126	42	49	58	39.850	39.444	48.898	hypothetical protein
bin008	SOY3_bin008_00401	996	317	386	509	38.049	39.308	54.286	Biotin synthase
bin008	SOY3_bin008_00402	171	30	37	61	20.973	21.946	37.893	hypothetical protein
bin008	SOY3_bin008_00403	801	193	192	261	28.805	24.312	34.613	Benzoyl-CoA oxygenase component A
bin008	SOY3_bin008_00404	2760	29	67	48	1.256	2.462	1.847	UvrABC system protein A
bin008	SOY3_bin008_00405	1056	46	176	220	5.208	16.905	22.130	hypothetical protein
bin008	SOY3_bin008_00406	165	12	18	30	8.694	11.065	19.314	hypothetical protein
bin008	SOY3_bin008_00407	1215	19	58	55	1.869	4.842	4.809	Phosphopentomutase
bin008	SOY3_bin008_00408	435	2	20	26	0.550	4.663	6.349	Acyl-CoA thioester hydrolase YbgC
bin008	SOY3_bin008_00409	786	2	9	14	0.304	1.161	1.892	Amidohydrolase
bin008	SOY3_bin008_00410	2328	22	123	125	1.130	5.359	5.704	Methyl-accepting chemotaxis protein McpS
bin008	SOY3_bin008_00411	1776	134	272	362	9.020	15.534	21.652	Methyl-accepting chemotaxis protein McpS
bin008	SOY3_bin008_00412	876	15	50	53	2.047	5.789	6.427	Phospholipase YtpA
bin008	SOY3_bin008_00413	552	18	35	29	3.898	6.431	5.581	hypothetical protein
bin008	SOY3_bin008_00414	243	332	1283	1384	163.334	535.519	605.006	hypothetical protein
bin008	SOY3_bin008_00415	996	482	896	1190	57.854	91.244	126.916	tRNA1(Val) (adenine(37)-N6)-methyltransferase
bin008	SOY3_bin008_00416	711	2	8	9	0.336	1.141	1.345	Integral membrane protein TerC family protein
bin008	SOY3_bin008_00417	1539	9	11	19	0.699	0.725	1.311	putative peptidoglycan biosynthesis protein MurJ
bin008	SOY3_bin008_00418	846	5	13	14	0.707	1.559	1.758	Formamidopyrimidine-DNA glycosylase
bin008	SOY3_bin008_00419	1224	33	48	52	3.223	3.978	4.513	PDZ domain (Also known as DHR or GLGF)
bin008	SOY3_bin008_00420	1266	50	121	159	4.722	9.694	13.341	Phenylacetate-coenzyme A ligase
bin008	SOY3_bin008_00421	1632	209	240	345	15.310	14.916	22.456	Sensory/regulatory protein RpfC
bin008	SOY3_bin008_00422	888	0	13	12	0.000	1.485	1.435	tRNA (adenine(58)-N(1))-methyltransferase TrmI
bin008	SOY3_bin008_00423	1020	6	31	30	0.703	3.083	3.124	molybdenum cofactor biosynthesis protein A
bin008	SOY3_bin008_00424	1461	9	51	51	0.736	3.541	3.708	Ribonuclease E
bin008	SOY3_bin008_00425	582	3	7	16	0.616	1.220	2.920	hypothetical protein
bin008	SOY3_bin008_00426	942	11	69	113	1.396	7.429	12.743	cobalamin biosynthesis protein

bin008	SOY3_bin008_00427	207	531	1564	1889	306.669	766.339	969.374	Cold shock protein CspB
bin008	SOY3_bin008_00428	801	23	83	111	3.433	10.510	14.720	Pyrroline-5-carboxylate reductase
bin008	SOY3_bin008_00429	417	11	77	70	3.154	18.729	17.832	Nucleoside diphosphate kinase
bin008	SOY3_bin008_00430	1227	96	230	242	9.353	19.012	20.951	Hypoxic response protein 1
bin008	SOY3_bin008_00431	378	32	61	60	10.121	16.368	16.861	Putative fluoride ion transporter CrcB
bin008	SOY3_bin008_00432	843	59	151	205	8.367	18.168	25.832	NifU-like protein
bin008	SOY3_bin008_00433	1185	94	216	272	9.483	18.488	24.383	Cysteine desulfurase
bin008	SOY3_bin008_00434	930	56	160	161	7.199	17.450	18.390	O-acetylserine sulfhydrylase
bin008	SOY3_bin008_00435	942	79	151	213	10.026	16.259	24.019	Serine acetyltransferase
bin008	SOY3_bin008_00436	309	16	27	51	6.190	8.863	17.532	Double zinc ribbon
bin008	SOY3_bin008_00437	333	4	14	19	1.436	4.264	6.061	tRNA-binding protein YgjH
bin008	SOY3_bin008_00438	642	9	20	22	1.676	3.160	3.640	putative adenylyl-sulfate kinase
bin008	SOY3_bin008_00439	1116	28	69	70	2.999	6.271	6.663	Phosphotransferase enzyme family protein
bin008	SOY3_bin008_00440	951	17	81	69	2.137	8.639	7.707	Adenosine kinase
bin008	SOY3_bin008_00441	327	26	60	47	9.505	18.611	15.268	Divalent-cation tolerance protein CutA
bin008	SOY3_bin008_00442	1974	10	22	17	0.606	1.130	0.915	Oxygen sensor histidine kinase NreB
bin008	SOY3_bin008_00443	645	0	1	5	0.000	0.157	0.823	Transcriptional regulatory protein UhpA
bin008	SOY3_bin008_00444	651	5	20	25	0.918	3.116	4.079	Ultraviolet N-glycosylase/AP lyase
bin008	SOY3_bin008_00445	783	13	69	80	1.985	8.938	10.853	Murein hydrolase activator EnvC precursor
bin008	SOY3_bin008_00446	1287	13	193	201	1.208	15.210	16.590	Carboxy-terminal processing protease CtpB precursor
bin008	SOY3_bin008_00447	1158	7	38	38	0.723	3.328	3.486	Divergent polysaccharide deacetylase
bin008	SOY3_bin008_00448	1131	23	71	89	2.431	6.367	8.359	O-acetyltransferase OatA
bin008	SOY3_bin008_00449	777	30	51	49	4.616	6.657	6.699	Ribonuclease Z
bin008	SOY3_bin008_00450	1542	17	21	29	1.318	1.381	1.998	BNR/Asp-box repeat protein
bin008	SOY3_bin008_00451	1041	7	22	21	0.804	2.144	2.143	hypothetical protein
bin008	SOY3_bin008_00452	693	21	118	89	3.623	17.270	13.642	Murein DD-endopeptidase MepH precursor
bin008	SOY3_bin008_00453	1572	48	106	109	3.650	6.839	7.366	Na(+)-translocating NADH-quinone reductase subunit F
bin008	SOY3_bin008_00454	1398	57	83	130	4.874	6.022	9.878	Mitochondrial small ribosomal subunit Rsm22
bin008	SOY3_bin008_00455	558	8	25	34	1.714	4.544	6.473	dTDP-4-dehydrorhamnose 3,5-epimerase
bin008	SOY3_bin008_00456	255	0	10	9	0.000	3.978	3.749	hypothetical protein
bin008	SOY3_bin008_00457	570	39	82	108	8.180	14.591	20.127	Class III cytochrome C family protein
bin008	SOY3_bin008_00458	2154	90	212	224	4.995	9.983	11.047	Polysulfide reductase chain A precursor
bin008	SOY3_bin008_00459	759	29	59	73	4.568	7.884	10.217	Tetrathionate reductase subunit B precursor
bin008	SOY3_bin008_00460	1245	36	108	102	3.457	8.799	8.703	putative Ni/Fe-hydrogenase 2 b-type cytochrome subunit
bin008	SOY3_bin008_00461	474	1	8	3	0.252	1.712	0.672	Cys-tRNA(Pro)/Cys-tRNA(Cys) deacylase YbaK
bin008	SOY3_bin008_00462	2235	12	47	38	0.642	2.133	1.806	ATP-dependent DNA helicase RecQ
bin008	SOY3_bin008_00463	1512	72	138	160	5.693	9.257	11.241	FHA domain protein
bin008	SOY3_bin008_00464	264	0	1	3	0.000	0.384	1.207	hypothetical protein
bin008	SOY3_bin008_00465	789	0	2	0	0.000	0.257	0.000	Agglutinin receptor precursor
bin008	SOY3_bin008_00466	1236	0	2	1	0.000	0.164	0.086	Cyclic pyranopterin monophosphate synthase
bin008	SOY3_bin008_00467	606	17	51	61	3.354	8.536	10.693	hypothetical protein
bin008	SOY3_bin008_00468	384	338	763	956	105.228	201.534	264.458	hypothetical protein
bin008	SOY3_bin008_00469	255	130	277	375	60.946	110.178	156.214	hypothetical protein
bin008	SOY3_bin008_00470	1116	11	37	53	1.178	3.363	5.045	HPr kinase/phosphorylase
bin008	SOY3_bin008_00471	885	10	15	12	1.351	1.719	1.440	Alpha-aminoadipate--LysW ligase LysX
bin008	SOY3_bin008_00472	1635	13	22	27	0.951	1.365	1.754	hypothetical protein
bin008	SOY3_bin008_00473	288	4	5	4	1.660	1.761	1.475	hypothetical protein
bin008	SOY3_bin008_00474	3393	32	87	114	1.127	2.601	3.569	Sensory/regulatory protein RpfC
bin008	SOY3_bin008_00475	1014	0	6	6	0.000	0.600	0.629	Putative glycosyltransferase EpsE
bin008	SOY3_bin008_00476	708	58	652	752	9.794	93.405	112.827	Sulfate/thiosulfate import ATP-binding protein CysA
bin008	SOY3_bin008_00477	747	27	877	991	4.321	119.079	140.923	Molybdate-binding periplasmic protein precursor
bin008	SOY3_bin008_00478	669	11	222	270	1.966	33.657	42.871	Molybdenum transport system permease protein ModB
bin008	SOY3_bin008_00479	663	3	17	13	0.541	2.601	2.083	Cyclic di-GMP binding protein
bin008	SOY3_bin008_00480	825	16	72	66	2.319	8.852	8.498	tetratricopeptide repeat protein
bin008	SOY3_bin008_00481	1278	1	6	9	0.094	0.476	0.748	FeMo cofactor biosynthesis protein NifB
bin008	SOY3_bin008_00482	1356	3	9	8	0.264	0.673	0.627	Nitrogenase molybdenum-iron protein beta chain
bin008	SOY3_bin008_00483	1398	2	8	8	0.171	0.580	0.608	Nitrogenase molybdenum-iron protein alpha chain
bin008	SOY3_bin008_00484	810	8	18	17	1.181	2.254	2.229	Motility protein B
bin008	SOY3_bin008_00485	792	8	19	20	1.208	2.433	2.682	Chemotaxis protein PomA
bin008	SOY3_bin008_00486	1206	21	136	144	2.082	11.438	12.684	cAMP receptor protein
bin008	SOY3_bin008_00487	795	5	39	38	0.752	4.976	5.077	Dihydroorotate dehydrogenase B (NAD(+)), electron transfer subunit
bin008	SOY3_bin008_00488	927	8	36	44	1.032	3.939	5.042	Dihydroorotate dehydrogenase B (NAD(+)), catalytic subunit
bin008	SOY3_bin008_00489	399	86	367	384	25.767	93.293	102.232	Transcriptional activator protein CzcR
bin008	SOY3_bin008_00490	714	31	91	164	5.190	12.927	24.399	translation initiation factor Sui1
bin008	SOY3_bin008_00491	1260	35	128	209	3.321	10.304	17.620	Sulfite exporter TauE/SafE
bin008	SOY3_bin008_00492	375	4	16	26	1.275	4.328	7.365	Nitrogen assimilation regulatory protein
bin008	SOY3_bin008_00493	1437	8	27	40	0.666	1.906	2.957	Transcriptional regulatory protein ZraR

bin008	SOY3_bin008_00494	1518	20	42	42	1.575	2.806	2.939	Sensor protein ZraS
bin008	SOY3_bin008_00495	1998	24	22	28	1.436	1.117	1.489	Methyl-accepting chemotaxis protein PctC
bin008	SOY3_bin008_00496	852	11	23	25	1.543	2.738	3.117	Tim44-like domain protein
bin008	SOY3_bin008_00497	1080	11	34	44	1.218	3.193	4.328	Chemotaxis protein CheY
bin008	SOY3_bin008_00498	789	8	20	36	1.212	2.571	4.847	HD domain protein
bin008	SOY3_bin008_00499	267	8	13	12	3.582	4.938	4.774	hypothetical protein
bin008	SOY3_bin008_00500	1167	2	8	10	0.205	0.695	0.910	Macrolide export ATP-binding/permease protein MacB
bin008	SOY3_bin008_00501	1158	0	1	6	0.000	0.088	0.550	Lipoprotein-releasing system transmembrane protein LolE
bin008	SOY3_bin008_00502	714	4	6	13	0.670	0.852	1.934	ABC transporter ATP-binding protein YtrE
bin008	SOY3_bin008_00503	1206	15	25	24	1.487	2.103	2.114	Macrolide export protein MacA
bin008	SOY3_bin008_00504	1101	8	36	45	0.869	3.316	4.342	putative A/G-specific adenine glycosylase YfhQ
bin008	SOY3_bin008_00505	1860	4	20	25	0.257	1.091	1.428	Diguanylate cyclase DosC
bin008	SOY3_bin008_00506	1278	21	42	59	1.964	3.333	4.904	Bifunctional enzyme IspD/IspF
bin008	SOY3_bin008_00507	4128	156	420	578	4.518	10.320	14.874	hypothetical protein
bin008	SOY3_bin008_00508	438	12	18	27	3.275	4.168	6.548	HTH-type transcriptional regulator CymR
bin008	SOY3_bin008_00509	858	5	15	10	0.697	1.773	1.238	Chorismate dehydratase
bin008	SOY3_bin008_00510	1767	43	164	133	2.909	9.414	7.995	Adenylate kinase
bin008	SOY3_bin008_00511	336	7	9	9	2.491	2.717	2.845	hypothetical protein
bin008	SOY3_bin008_00512	1362	27	57	56	2.370	4.245	4.368	Glutamyl-tRNA reductase
bin008	SOY3_bin008_00513	870	25	59	71	3.435	6.878	8.669	Cytochrome c biogenesis protein CcsA
bin008	SOY3_bin008_00514	693	12	51	70	2.070	7.464	10.730	Siroheme synthase
bin008	SOY3_bin008_00515	1266	17	50	55	1.605	4.006	4.615	ADP-heptose--LPS heptosyltransferase 2
bin008	SOY3_bin008_00516	1278	14	38	52	1.310	3.016	4.322	hypothetical protein
bin008	SOY3_bin008_00517	1566	17	55	63	1.298	3.562	4.273	Flagellar hook protein FlgE
bin008	SOY3_bin008_00518	2403	24	75	87	1.194	3.166	3.846	Chemotaxis protein methyltransferase
bin008	SOY3_bin008_00519	180	1	0	0	0.664	0.000	0.000	hypothetical protein
bin008	SOY3_bin008_00520	279	12	24	18	5.142	8.725	6.853	hypothetical protein
bin008	SOY3_bin008_00521	8649	629	528	636	8.694	6.192	7.811	N,N'-diacetylchitobiose phosphorylase
bin008	SOY3_bin008_00522	1527	155	368	335	12.135	24.444	23.304	Threonylcarbamoyladenosine tRNA methylthiotransferase MtaB
bin008	SOY3_bin008_00523	996	18	185	229	2.161	18.839	24.423	General stress protein 69
bin008	SOY3_bin008_00524	87	0	1	1	0.000	1.166	1.221	tRNA-Leu(taa)
bin008	SOY3_bin008_00525	624	1	3	2	0.192	0.488	0.340	Phosphoglycolate phosphatase
bin008	SOY3_bin008_00526	1002	3	5	11	0.358	0.506	1.166	Putative prophage phiRv2 integrase
bin008	SOY3_bin008_00527	807	5	25	24	0.741	3.142	3.159	hypothetical protein
bin008	SOY3_bin008_00528	207	0	1	1	0.000	0.490	0.513	MbeD/MobD like protein
bin008	SOY3_bin008_00529	531	0	0	1	0.000	0.000	0.200	Cell division protein FtsL
bin008	SOY3_bin008_00530	384	0	0	0	0.000	0.000	0.000	Bacterial mobilisation protein (MobC)
bin008	SOY3_bin008_00531	1383	0	1	1	0.000	0.073	0.077	hypothetical protein
bin008	SOY3_bin008_00532	288	1	0	0	0.415	0.000	0.000	Helix-turn-helix domain protein
bin008	SOY3_bin008_00533	303	4	48	38	1.578	16.068	13.322	mRNA interferase MqsR
bin008	SOY3_bin008_00534	423	18	85	45	5.087	20.381	11.301	Antitoxin MqsA
bin008	SOY3_bin008_00535	180	3	6	2	1.992	3.381	1.180	hypothetical protein
bin008	SOY3_bin008_00536	1380	36	58	71	3.119	4.263	5.465	putative diguanylate cyclase YcdT
bin008	SOY3_bin008_00537	615	9	24	41	1.749	3.958	7.082	translation initiation factor Sui1
bin008	SOY3_bin008_00538	753	17	48	72	2.699	6.465	10.157	N-acyl homoserine lactonase
bin008	SOY3_bin008_00539	1950	37	275	261	2.268	14.304	14.218	Peptidyl-prolyl cis-trans isomerase D
bin008	SOY3_bin008_00540	1920	14	110	118	0.872	5.811	6.528	2,3-dimethylmalate dehydratase large subunit
bin008	SOY3_bin008_00541	666	32	82	97	5.744	12.488	15.471	NADH dehydrogenase
bin008	SOY3_bin008_00542	252	10	12	15	4.744	4.830	6.323	hypothetical protein
bin008	SOY3_bin008_00543	1368	4	30	22	0.350	2.224	1.708	Succinate semialdehyde dehydrogenase [NAD(P)+] Sad
bin008	SOY3_bin008_00544	1293	350	830	1044	32.360	65.108	85.769	Sulfate adenylyltransferase
bin008	SOY3_bin008_00545	1803	10	38	42	0.663	2.138	2.474	Elongation factor 4
bin008	SOY3_bin008_00546	600	2	35	26	0.398	5.917	4.603	Signal peptidase I
bin008	SOY3_bin008_00547	801	26	77	121	3.880	9.750	16.047	Molybdopterin adenylyltransferase
bin008	SOY3_bin008_00548	870	28	78	115	3.848	9.093	14.041	Glucose-1-phosphate thymidylyltransferase
bin008	SOY3_bin008_00549	150	17	44	49	13.549	29.752	34.700	hypothetical protein
bin008	SOY3_bin008_00550	2532	297	588	664	14.023	23.554	27.857	Sensory/regulatory protein RpfC
bin008	SOY3_bin008_00551	1113	43	62	81	4.619	5.650	7.731	Alpha/beta hydrolase family protein
bin008	SOY3_bin008_00552	744	8	38	43	1.285	5.180	6.139	Methionine aminopeptidase 2
bin008	SOY3_bin008_00553	1536	9	28	29	0.700	1.849	2.006	Competence protein ComM
bin008	SOY3_bin008_00554	1938	14	38	43	0.864	1.989	2.357	Non-motile and phage-resistance protein
bin008	SOY3_bin008_00555	3162	53	160	170	2.004	5.132	5.711	ATP-dependent DNA helicase PcrA
bin008	SOY3_bin008_00556	144	1	5	3	0.830	3.522	2.213	hypothetical protein
bin008	SOY3_bin008_00557	765	4	3	10	0.625	0.398	1.389	Xylose isomerase-like TIM barrel
bin008	SOY3_bin008_00558	525	2	11	15	0.455	2.125	3.035	Bifunctional adenosylcobalamin biosynthesis protein CobP
bin008	SOY3_bin008_00559	1005	24	47	45	2.855	4.743	4.756	putative manganese-dependent inorganic pyrophosphatase
bin008	SOY3_bin008_00560	375	16	35	38	5.101	9.467	10.764	Type II secretion system protein G precursor

bin008	SOY3_bin008_00561	405	8	20	10	2.361	5.009	2.623	Type II secretion system protein G precursor
bin008	SOY3_bin008_00562	1449	25	69	118	2.063	4.830	8.651	Polymer-forming cytoskeletal
bin008	SOY3_bin008_00563	849	22	40	62	3.098	4.779	7.757	Error-prone DNA polymerase
bin008	SOY3_bin008_00564	216	4	16	17	2.214	7.513	8.360	hypothetical protein
bin008	SOY3_bin008_00565	432	4	40	28	1.107	9.391	6.885	Spore protein SP21
bin008	SOY3_bin008_00566	1353	35	103	121	3.093	7.721	9.500	Periplasmic pH-dependent serine endoprotease DegQ precursor
bin008	SOY3_bin008_00567	942	30	133	166	3.807	14.320	18.719	Glycosyl transferase family 2
bin008	SOY3_bin008_00568	189	5	51	60	3.163	27.369	33.722	Ferredoxin-3
bin008	SOY3_bin008_00569	825	5	23	30	0.725	2.828	3.863	Flagellin N-methylase
bin008	SOY3_bin008_00570	360	2	10	12	0.664	2.817	3.541	hypothetical protein
bin008	SOY3_bin008_00571	627	9	36	40	1.716	5.824	6.777	TPR repeat-containing protein YrrB
bin008	SOY3_bin008_00572	708	12	25	24	2.026	3.581	3.601	hypothetical protein
bin008	SOY3_bin008_00573	423	0	10	4	0.000	2.398	1.004	hypothetical protein
bin008	SOY3_bin008_00574	345	83	311	237	28.761	91.432	72.972	hypothetical protein
bin008	SOY3_bin008_00575	567	447	1093	1364	94.248	195.520	255.541	EF hand
bin008	SOY3_bin008_00576	432	50	154	110	13.837	36.157	27.048	HTH-type transcriptional regulator CymR
bin008	SOY3_bin008_00577	1302	172	470	466	15.793	36.614	38.019	Cytochrome bd-I ubiquinol oxidase subunit 1
bin008	SOY3_bin008_00578	1029	112	283	317	13.012	27.895	32.725	Cytochrome bd-I ubiquinol oxidase subunit 2
bin008	SOY3_bin008_00579	714	4	9	5	0.670	1.278	0.744	Cyclic di-GMP phosphodiesterase response regulator RpfG
bin008	SOY3_bin008_00580	582	32	64	69	6.573	11.154	12.594	HTH-type transcriptional repressor KstR2
bin008	SOY3_bin008_00581	1245	19	32	41	1.824	2.607	3.498	Efflux pump periplasmic linker BepD precursor
bin008	SOY3_bin008_00582	3129	47	78	87	1.796	2.528	2.954	Efflux pump membrane transporter BepE
bin008	SOY3_bin008_00583	1221	0	12	12	0.000	0.997	1.044	Phosphoserine phosphatase
bin008	SOY3_bin008_00584	750	50	100	130	7.970	13.524	18.412	hypothetical protein
bin008	SOY3_bin008_00585	849	48	60	110	6.759	7.168	13.763	Peptidase family M13
bin008	SOY3_bin008_00586	1017	12	45	52	1.411	4.488	5.431	putative aminodeoxychorismate lyase
bin008	SOY3_bin008_00587	420	3	18	21	0.854	4.347	5.311	Putative Holliday junction resolvase
bin008	SOY3_bin008_00588	3534	12	101	90	0.406	2.899	2.705	putative FAD-linked oxidoreductase
bin008	SOY3_bin008_00589	174	18	54	49	12.367	31.477	29.914	hypothetical protein
bin008	SOY3_bin008_00590	1440	150	442	447	12.453	31.133	32.974	hypothetical protein
bin008	SOY3_bin008_00591	2496	9	30	50	0.431	1.219	2.128	Sensor protein ZraS
bin008	SOY3_bin008_00592	1404	12	40	55	1.022	2.890	4.161	Transcriptional regulatory protein QseF
bin008	SOY3_bin008_00593	612	13	43	44	2.539	7.126	7.637	Homoserine/homoserine lactone efflux protein
bin008	SOY3_bin008_00594	1659	51	147	168	3.675	8.987	10.757	Ribosomal protein S12 methylthiotransferase RimO
bin008	SOY3_bin008_00595	1287	182	539	655	16.906	42.478	54.062	Cell division protein FtsZ
bin008	SOY3_bin008_00596	1227	88	280	365	8.574	23.146	31.599	Cell division protein FtsA
bin008	SOY3_bin008_00597	942	87	167	190	11.041	17.981	21.426	Cell division protein DivIB
bin008	SOY3_bin008_00598	888	51	136	176	6.866	15.534	21.054	UDP-N-acetylenolpyruvoylglucosamine reductase
bin008	SOY3_bin008_00599	1416	68	176	226	5.741	12.607	16.954	UDP-N-acetylmuramate--L-alanine ligase
bin008	SOY3_bin008_00600	1107	40	134	189	4.320	12.278	18.136	UDP-N-acetylglucosamine--N-acetylmuramyl-(pentapeptide) pyrophosphoryl-undecaprenol N-acetylglucosamine transferase
bin008	SOY3_bin008_00601	1125	26	70	89	2.763	6.311	8.404	Lipid II flippase FtsW
bin008	SOY3_bin008_00602	1329	60	154	191	5.397	11.753	15.266	UDP-N-acetylmuramoylalanine--D-glutamate ligase
bin008	SOY3_bin008_00603	1077	37	114	142	4.107	10.736	14.006	Phospho-N-acetylmuramoyl-pentapeptide-transferase
bin008	SOY3_bin008_00604	1407	79	168	219	6.712	12.111	16.534	UDP-N-acetylmuramoyl-tripeptide--D-alanyl-D-alanine ligase
bin008	SOY3_bin008_00605	1464	121	255	327	9.881	17.667	23.727	UDP-N-acetylmuramoyl-L-alanyl-D-glutamate--2,6-diaminopimelate ligase
bin008	SOY3_bin008_00606	2019	126	282	300	7.461	14.167	15.784	Peptidoglycan synthase FtsI precursor
bin008	SOY3_bin008_00607	393	46	170	200	13.993	43.874	54.059	Cell division protein FtsL
bin008	SOY3_bin008_00608	975	645	1197	1409	79.086	124.521	153.510	Ribosomal RNA small subunit methyltransferase H
bin008	SOY3_bin008_00609	411	49	126	121	14.253	31.094	31.273	cell division protein MraZ
bin008	SOY3_bin008_00610	1020	36	127	149	4.219	12.629	15.517	Cyclic di-GMP phosphodiesterase response regulator RpfG
bin008	SOY3_bin008_00611	273	19	50	40	8.320	18.576	15.564	Mor transcription activator family protein
bin008	SOY3_bin008_00612	708	15	10	12	2.533	1.433	1.800	Glycine/sarcosine/dimethylglycine N-methyltransferase
bin008	SOY3_bin008_00613	1302	24	71	70	2.204	5.531	5.711	Sulfite exporter TauE/SafE
bin008	SOY3_bin008_00614	378	12	18	29	3.795	4.830	8.150	Alkyl hydroperoxide reductase AhpD
bin008	SOY3_bin008_00615	930	9	10	23	1.157	1.091	2.627	Hca operon transcriptional activator
bin008	SOY3_bin008_00616	1032	1	8	8	0.116	0.786	0.823	hypothetical protein
bin008	SOY3_bin008_00617	1872	3	32	35	0.192	1.734	1.986	Asparagine synthetase [glutamine-hydrolyzing] 1
bin008	SOY3_bin008_00618	729	5	20	17	0.820	2.783	2.477	Bifunctional polymyxin resistance protein ArnA
bin008	SOY3_bin008_00619	933	9	57	48	1.153	6.197	5.465	Cyclic pyranopterin monophosphate synthase 1
bin008	SOY3_bin008_00620	957	8	57	62	0.999	6.041	6.882	Antilisterial bacteriocin subtilosin biosynthesis protein AlbA
bin008	SOY3_bin008_00621	900	6	36	37	0.797	4.057	4.367	Undecaprenyl-phosphate mannosyltransferase
bin008	SOY3_bin008_00622	693	5	35	30	0.863	5.123	4.599	WbqC-like protein family protein
bin008	SOY3_bin008_00623	1041	2	34	40	0.230	3.313	4.082	dTDP-glucose 4,6-dehydratase
bin008	SOY3_bin008_00624	1137	5	51	48	0.526	4.550	4.484	UDP-4-amino-4-deoxy-L-arabinose--oxoglutarate aminotransferase
bin008	SOY3_bin008_00625	1563	5	14	17	0.382	0.908	1.155	hypothetical protein
bin008	SOY3_bin008_00626	1323	25	24	44	2.259	1.840	3.533	Type I phosphodiesterase / nucleotide pyrophosphatase
bin008	SOY3_bin008_00627	2151	31	38	50	1.723	1.792	2.469	ATP-dependent DNA helicase PcrA

bin008	SOY3_bin008_00628	177	4	12	13	2.702	6.876	7.802	hypothetical protein
bin008	SOY3_bin008_00629	402	38	106	121	11.301	26.745	31.973	Transcriptional activator protein CzcR
bin008	SOY3_bin008_00630	741	52	111	123	8.389	15.194	17.633	Cytochrome b5-like Heme/Steroid binding domain protein
bin008	SOY3_bin008_00631	2115	145	298	347	8.196	14.291	17.428	Sporulation kinase E
bin008	SOY3_bin008_00632	954	24	45	52	3.008	4.784	5.790	prenyltransferase
bin008	SOY3_bin008_00633	1020	44	96	150	5.157	9.546	15.621	Cytochrome bd-I ubiquinol oxidase subunit 2
bin008	SOY3_bin008_00634	1368	80	158	221	6.991	11.715	17.161	Cytochrome bd-II ubiquinol oxidase subunit 1
bin008	SOY3_bin008_00635	603	28	69	100	5.551	11.606	17.616	Phthiotriol/phenolphthiotriol dimycocerosates methyltransferase
bin008	SOY3_bin008_00636	690	0	7	8	0.000	1.029	1.232	Demethylmenaquinone methyltransferase
bin008	SOY3_bin008_00637	432	1	15	16	0.277	3.522	3.934	hypothetical protein
bin008	SOY3_bin008_00638	1170	3	17	25	0.307	1.474	2.270	Chromosome-partitioning ATPase Soj
bin008	SOY3_bin008_00639	831	21	59	62	3.021	7.201	7.925	Poly-beta-1,6-N-acetyl-D-glucosamine N-deacetylase precursor
bin008	SOY3_bin008_00640	1146	17	45	54	1.773	3.983	5.005	Membrane-bound lytic murein transglycosylase B precursor
bin008	SOY3_bin008_00641	954	9	25	27	1.128	2.658	3.006	Nucleoside recognition
bin008	SOY3_bin008_00642	489	5	10	24	1.222	2.074	5.214	hypothetical protein
bin008	SOY3_bin008_00643	342	16	120	149	5.593	35.589	46.280	Alkyl hydroperoxide reductase AhpD
bin008	SOY3_bin008_00644	936	4	14	13	0.511	1.517	1.475	HTH-type transcriptional regulator CysL
bin008	SOY3_bin008_00645	459	25	215	211	6.511	47.510	48.831	50S ribosomal protein L17
bin008	SOY3_bin008_00646	1044	46	487	488	5.267	47.313	49.653	DNA-directed RNA polymerase subunit alpha
bin008	SOY3_bin008_00647	627	31	306	355	5.911	49.500	60.144	30S ribosomal protein S4
bin008	SOY3_bin008_00648	387	24	133	149	7.414	34.857	40.898	30S ribosomal protein S11
bin008	SOY3_bin008_00649	372	24	203	222	7.713	55.349	63.393	30S ribosomal protein S13
bin008	SOY3_bin008_00650	114	9	85	89	9.438	75.626	82.931	50S ribosomal protein L36
bin008	SOY3_bin008_00651	717	30	229	260	5.002	32.394	38.520	Methionine aminopeptidase 1
bin008	SOY3_bin008_00652	1314	48	265	285	4.367	20.455	23.040	preprotein translocase subunit SecY
bin008	SOY3_bin008_00653	480	18	246	271	4.483	51.981	59.973	50S ribosomal protein L15
bin008	SOY3_bin008_00654	180	7	102	95	4.649	57.475	56.064	50S ribosomal protein L30
bin008	SOY3_bin008_00655	534	35	286	320	7.836	54.322	63.656	30S ribosomal protein S5
bin008	SOY3_bin008_00656	360	14	211	237	4.649	59.448	69.932	50S ribosomal protein L18
bin008	SOY3_bin008_00657	540	26	255	268	5.756	47.896	52.719	50S ribosomal protein L6
bin008	SOY3_bin008_00658	384	29	243	276	9.028	64.184	76.350	30S ribosomal protein S8
bin008	SOY3_bin008_00659	186	18	108	119	11.569	58.893	67.962	30S ribosomal protein S14 type Z
bin008	SOY3_bin008_00660	540	16	228	233	3.542	42.825	45.834	50S ribosomal protein L5
bin008	SOY3_bin008_00661	324	8	151	148	2.952	47.270	48.523	50S ribosomal protein L24
bin008	SOY3_bin008_00662	369	15	200	232	4.860	54.974	66.787	50S ribosomal protein L14
bin008	SOY3_bin008_00663	261	15	197	183	6.871	76.556	74.480	30S ribosomal protein S17
bin008	SOY3_bin008_00664	195	10	110	123	6.131	57.215	67.004	50S ribosomal protein L29
bin008	SOY3_bin008_00665	420	25	239	242	7.116	57.717	61.206	50S ribosomal protein L16
bin008	SOY3_bin008_00666	642	33	323	296	6.145	51.030	48.976	30S ribosomal protein S3
bin008	SOY3_bin008_00667	333	34	243	238	12.206	74.014	75.921	50S ribosomal protein L22
bin008	SOY3_bin008_00668	282	19	238	192	8.055	85.602	72.324	30S ribosomal protein S19
bin008	SOY3_bin008_00669	831	49	431	460	7.049	52.605	58.801	50S ribosomal protein L2
bin008	SOY3_bin008_00670	291	14	200	218	5.751	69.709	79.578	50S ribosomal protein L23
bin008	SOY3_bin008_00671	633	29	384	394	5.477	61.529	66.118	50S ribosomal protein L4
bin008	SOY3_bin008_00672	633	45	445	479	8.499	71.304	80.383	50S ribosomal protein L3
bin008	SOY3_bin008_00673	318	24	225	240	9.023	71.765	80.170	30S ribosomal protein S10
bin008	SOY3_bin008_00674	138	12	126	124	10.396	92.608	95.449	Elongation factor Tu
bin008	SOY3_bin008_00675	1143	11	39	58	1.151	3.461	5.390	30S ribosomal protein S5
bin008	SOY3_bin008_00676	444	6	17	26	1.616	3.883	6.220	Ferredoxin
bin008	SOY3_bin008_00677	525	7	31	29	1.594	5.989	5.868	PilZ domain protein
bin008	SOY3_bin008_00678	735	13	52	66	2.114	7.176	9.539	chemotaxis regulator CheZ
bin008	SOY3_bin008_00679	801	18	64	84	2.686	8.104	11.140	Cobalt import ATP-binding protein CbiO
bin008	SOY3_bin008_00680	768	13	78	109	2.024	10.301	15.076	Nickel transport protein NikQ
bin008	SOY3_bin008_00681	675	18	163	218	3.188	24.493	34.307	hypothetical protein
bin008	SOY3_bin008_00682	657	7	113	162	1.274	17.445	26.193	Cobalt transport protein CbiM
bin008	SOY3_bin008_00683	795	89	1373	1991	13.383	175.169	266.032	Nickel uptake substrate-specific transmembrane region
bin008	SOY3_bin008_00684	2472	13	29	34	0.629	1.190	1.461	GDP-mannose-dependent alpha-mannosyltransferase
bin008	SOY3_bin008_00685	201	1	3	3	0.595	1.514	1.585	hypothetical protein
bin008	SOY3_bin008_00686	1935	13	77	69	0.803	4.036	3.788	DNA recombination protein RmuC
bin008	SOY3_bin008_00687	126	0	3	5	0.000	2.415	4.215	hypothetical protein
bin008	SOY3_bin008_00688	1797	7	20	23	0.466	1.129	1.360	UvrABC system protein C
bin008	SOY3_bin008_00689	480	27	67	98	6.725	14.158	21.688	LOG family protein ORF6 in fasciation locus
bin008	SOY3_bin008_00690	501	27	54	65	6.443	10.932	13.782	AP-4-A phosphorylase
bin008	SOY3_bin008_00691	411	54	132	150	15.707	32.575	38.769	hypothetical protein
bin008	SOY3_bin008_00692	2607	11	55	84	0.504	2.140	3.423	DNA mismatch repair protein MutS
bin008	SOY3_bin008_00693	1251	12	70	68	1.147	5.675	5.774	Diaminopimelate decarboxylase
bin008	SOY3_bin008_00694	537	20	49	75	4.452	9.255	14.836	GDSL-like Lipase/Acylhydrolase

bin008	SOY3_bin008_00695	354	11	34	30	3.715	9.742	9.002	hypothetical protein
bin008	SOY3_bin008_00696	930	6	24	29	0.771	2.617	3.312	hypothetical protein
bin008	SOY3_bin008_00697	2070	2	17	28	0.116	0.833	1.437	Prolyl tripeptidyl peptidase precursor
bin008	SOY3_bin008_00698	1605	12	188	190	0.894	11.881	12.575	Swarming motility protein SwrC
bin008	SOY3_bin008_00699	369	8	148	171	2.592	40.681	49.227	preprotein translocase subunit YajC
bin008	SOY3_bin008_00700	1767	29	178	205	1.962	10.217	12.324	RNA polymerase sigma factor RpoD
bin008	SOY3_bin008_00701	1725	22	75	90	1.525	4.410	5.542	DNA primase
bin008	SOY3_bin008_00702	2331	29	99	108	1.487	4.308	4.922	Endonuclease MutS2
bin008	SOY3_bin008_00703	447	93	604	647	24.873	137.052	153.754	glutamyl-tRNA(Gln) amidotransferase subunit E
bin008	SOY3_bin008_00704	201	37	329	323	22.007	166.018	170.701	30S ribosomal protein S21
bin008	SOY3_bin008_00705	183	11	103	106	7.186	57.087	61.530	hypothetical protein
bin008	SOY3_bin008_00706	273	35	195	215	15.327	72.448	83.658	DNA-binding protein HRL53
bin008	SOY3_bin008_00707	843	0	23	34	0.000	2.767	4.284	Ribosomal RNA small subunit methyltransferase A
bin008	SOY3_bin008_00708	1716	5	20	15	0.348	1.182	0.929	Spore coat protein SA
bin008	SOY3_bin008_00709	1503	24	128	199	1.909	8.638	14.064	Response regulator PleD
bin008	SOY3_bin008_00710	768	67	369	434	10.429	48.733	60.029	hypothetical protein
bin008	SOY3_bin008_00711	1137	14	42	43	1.472	3.747	4.017	Alanine racemase
bin008	SOY3_bin008_00712	1935	69	190	193	4.263	9.959	10.595	DNA mismatch repair protein MutL
bin008	SOY3_bin008_00713	975	9	24	37	1.104	2.497	4.031	ADP-L-glycero-D-manno-heptose-6-epimerase
bin008	SOY3_bin008_00714	633	2	14	17	0.378	2.243	2.853	Imidazole glycerol phosphate synthase subunit HisH 1
bin008	SOY3_bin008_00715	780	5	32	27	0.766	4.161	3.677	Imidazole glycerol phosphate synthase subunit HisF
bin008	SOY3_bin008_00716	1095	15	121	131	1.638	11.208	12.708	preprotein translocase subunit SecF
bin008	SOY3_bin008_00717	282	3	30	29	1.272	10.790	10.924	hypothetical protein
bin008	SOY3_bin008_00718	849	15	47	65	2.112	5.615	8.133	Modulator of FtsH protease HflC
bin008	SOY3_bin008_00719	1125	22	83	87	2.338	7.483	8.215	Modulator of FtsH protease HflK
bin008	SOY3_bin008_00720	1377	14	51	62	1.215	3.757	4.783	Phosphomannomutase/phosphoglucomutase
bin008	SOY3_bin008_00721	498	4	21	20	0.960	4.277	4.266	hypothetical protein
bin008	SOY3_bin008_00722	2034	248	713	968	14.576	35.554	50.554	Methyl-accepting chemotaxis protein I
bin008	SOY3_bin008_00723	2565	1	15	15	0.047	0.593	0.621	cell division ABC transporter subunit FtsX
bin008	SOY3_bin008_00724	555	2	7	5	0.431	1.279	0.957	ECF RNA polymerase sigma factor SigE
bin008	SOY3_bin008_00725	525	1	10	16	0.228	1.932	3.237	Shikimate kinase 2
bin008	SOY3_bin008_00726	1338	38	68	82	3.395	5.155	6.510	Pyrophosphate--fructose 6-phosphate 1-phosphotransferase
bin008	SOY3_bin008_00727	513	20	65	80	4.661	12.851	16.565	Cyclic pyranopterin monophosphate synthase accessory protein
bin008	SOY3_bin008_00728	423	17	62	56	4.805	14.866	14.063	photosystem I assembly protein Ycf3
bin008	SOY3_bin008_00729	1110	36	157	162	3.877	14.346	15.503	Chaperone protein DnaJ
bin008	SOY3_bin008_00730	225	16	62	56	8.501	27.949	26.438	DNA-directed RNA polymerase subunit omega
bin008	SOY3_bin008_00731	1401	9	55	52	0.768	3.982	3.943	Phosphoserine phosphatase RsbU
bin008	SOY3_bin008_00732	1959	7	26	27	0.427	1.346	1.464	OPT oligopeptide transporter protein
bin008	SOY3_bin008_00733	1983	5	22	30	0.301	1.125	1.607	hypothetical protein
bin008	SOY3_bin008_00734	4995	23	82	102	0.550	1.665	2.169	ABC transporter permease YtrF precursor
bin008	SOY3_bin008_00735	741	10	41	43	1.613	5.612	6.164	Lipoprotein-releasing system ATP-binding protein LolD
bin008	SOY3_bin008_00736	717	8	22	31	1.334	3.112	4.593	ABC transporter permease YtrF precursor
bin008	SOY3_bin008_00737	1359	31	53	64	2.727	3.956	5.003	Transcriptional regulatory protein ZraR
bin008	SOY3_bin008_00738	624	30	95	73	5.748	15.442	12.427	hypothetical protein
bin008	SOY3_bin008_00739	1458	1	15	15	0.082	1.043	1.093	Alkaline phosphatase synthesis sensor protein PhoR
bin008	SOY3_bin008_00740	504	5	27	29	1.186	5.434	6.112	hypothetical protein
bin008	SOY3_bin008_00741	891	7	13	26	0.939	1.480	3.100	Rhomboid protease GluP
bin008	SOY3_bin008_00742	1089	6	26	40	0.659	2.422	3.902	putative inner membrane protein
bin008	SOY3_bin008_00743	213	60	222	267	33.676	105.713	133.156	Rubredoxin-2
bin008	SOY3_bin008_00744	567	192	609	686	40.482	108.940	128.520	Rubrerythrin-2
bin008	SOY3_bin008_00745	468	79	300	323	20.180	65.017	73.314	Transcriptional regulator PerR
bin008	SOY3_bin008_00746	1551	2	5	3	0.154	0.327	0.205	Cation/acetate symporter ActP
bin008	SOY3_bin008_00747	312	0	1	1	0.000	0.325	0.340	Inner membrane protein YjcH
bin008	SOY3_bin008_00748	1440	28	31	40	2.325	2.184	2.951	Bifunctional protein GlmU
bin008	SOY3_bin008_00749	378	8	15	16	2.530	4.025	4.496	Transcriptional regulatory protein ZraR
bin008	SOY3_bin008_00750	1503	4	24	23	0.318	1.620	1.626	Na(+)/H(+) antiporter NhaB
bin008	SOY3_bin008_00751	2031	90	202	218	5.298	10.088	11.402	hypothetical protein
bin008	SOY3_bin008_00752	1032	9	57	63	1.043	5.602	6.485	L-lactate dehydrogenase [cytochrome]
bin008	SOY3_bin008_00753	3426	54	108	134	1.884	3.197	4.155	Blue-light-activated protein
bin008	SOY3_bin008_00754	1419	11	25	19	0.927	1.787	1.422	Transcriptional regulatory protein ZraR
bin008	SOY3_bin008_00755	3642	841	1947	2295	27.606	54.223	66.938	Pyruvate-flavodoxin oxidoreductase
bin008	SOY3_bin008_00756	1380	669	1027	1446	57.955	75.482	111.306	putative FAD-linked oxidoreductase
bin008	SOY3_bin008_00757	1308	219	507	585	20.016	39.315	47.509	Lactate utilization protein A
bin008	SOY3_bin008_00758	2109	372	691	792	21.087	33.232	39.891	Succinyl-CoA ligase [ADP-forming] subunit alpha
bin008	SOY3_bin008_00759	1065	275	555	646	30.869	52.856	64.434	Phosphate acetyltransferase
bin008	SOY3_bin008_00760	1632	1122	1304	1876	82.190	81.042	122.108	Glycolate permease GlcA
bin008	SOY3_bin008_00761	627	225	206	273	42.900	33.324	46.251	Lactate utilization protein C

bin008	SOY3_bin008_00762	2157	756	709	1136	41.900	33.339	55.945	Lactate utilization protein B
bin008	SOY3_bin008_00763	864	6	28	25	0.830	3.287	3.074	hypothetical protein
bin008	SOY3_bin008_00764	519	5	24	22	1.152	4.690	4.503	hypothetical protein
bin008	SOY3_bin008_00765	1824	260	518	683	17.041	28.804	39.776	Methyl-accepting chemotaxis protein McpS
bin008	SOY3_bin008_00766	486	142	207	287	34.930	43.201	62.730	Zinc resistance-associated protein precursor
bin008	SOY3_bin008_00767	663	4	65	50	0.721	9.944	8.011	Nitrite reductase [NAD(P)H]
bin008	SOY3_bin008_00768	288	230	169	149	95.473	59.518	54.957	hypothetical protein
bin008	SOY3_bin008_00769	99	23	30	19	27.774	30.736	20.387	hypothetical protein
bin008	SOY3_bin008_00770	801	15	21	26	2.239	2.659	3.448	2-amino-4,5-dihydroxy-6-one-heptanoic acid-7-phosphate synthase
bin008	SOY3_bin008_00771	906	4	11	18	0.528	1.231	2.110	hypothetical protein
bin008	SOY3_bin008_00772	1236	2	15	29	0.193	1.231	2.492	D-serine dehydratase
bin008	SOY3_bin008_00773	921	123	152	188	15.966	16.739	21.683	Manganese-dependent inorganic pyrophosphatase
bin008	SOY3_bin008_00774	1170	22	48	73	2.248	4.161	6.628	Multifunctional CCA protein
bin008	SOY3_bin008_00775	915	30	85	110	3.920	9.422	12.770	Bifunctional ligase/repressor BirA
bin008	SOY3_bin008_00776	3696	64	386	373	2.070	10.593	10.720	2-oxoglutarate carboxylase small subunit
bin008	SOY3_bin008_00777	3588	171	849	924	5.698	24.000	27.356	Phosphoenolpyruvate synthase
bin008	SOY3_bin008_00778	612	32	49	43	6.251	8.121	7.464	putative S-adenosylmethionine-dependent methyltransferase/MSMEI_2290
bin008	SOY3_bin008_00779	1248	79	142	140	7.568	11.541	11.916	Homoserine O-acetyltransferase
bin008	SOY3_bin008_00780	1056	11	93	80	1.245	8.933	8.047	D-gamma-glutamyl-meso-diaminopimelic acid endopeptidase CwlS precursor
bin008	SOY3_bin008_00781	504	14	57	66	3.321	11.471	13.911	Methylamine utilization protein MauE
bin008	SOY3_bin008_00782	522	32	179	181	7.329	34.781	36.833	molybdopterin biosynthesis protein MoeB
bin008	SOY3_bin008_00783	561	0	8	8	0.000	1.446	1.515	Putative redox-active protein (C_GCAxxG_C_C)
bin008	SOY3_bin008_00784	864	0	15	19	0.000	1.761	2.336	hypothetical protein
bin008	SOY3_bin008_00785	945	1271	1183	1263	160.790	126.972	141.972	Periplasmic [NiFe] hydrogenase small subunit precursor
bin008	SOY3_bin008_00786	1695	2215	2058	2118	156.225	123.149	132.735	Periplasmic [NiFe] hydrogenase large subunit precursor
bin008	SOY3_bin008_00787	504	4	9	7	0.949	1.811	1.475	Hydrogenase 2 maturation protease
bin008	SOY3_bin008_00788	780	2	3	1	0.307	0.390	0.136	Type 4 prepilin-like proteins leader peptide-processing enzyme
bin008	SOY3_bin008_00789	276	0	21	17	0.000	7.717	6.543	hypothetical protein
bin008	SOY3_bin008_00790	1437	3	120	111	0.250	8.470	8.205	2-iminoacetate synthase
bin008	SOY3_bin008_00791	597	65	130	180	13.016	22.086	32.028	hypothetical protein
bin008	SOY3_bin008_00792	1014	22	54	84	2.594	5.401	8.800	Paraquat-inducible protein B
bin008	SOY3_bin008_00793	777	16	25	43	2.462	3.263	5.879	Glycine betaine transport ATP-binding protein OpuAA
bin008	SOY3_bin008_00794	1161	43	79	103	4.428	6.902	9.424	putative phospholipid ABC transporter permease protein MlaE
bin008	SOY3_bin008_00795	1074	60	87	158	6.679	8.216	15.627	Diguanylate cyclase DosC
bin008	SOY3_bin008_00796	465	53	91	112	13.626	19.849	25.586	inner membrane protein
bin008	SOY3_bin008_00797	285	63	69	101	26.427	24.556	37.645	hypothetical protein
bin008	SOY3_bin008_00798	837	98	147	177	13.997	17.813	22.464	Bacterial extracellular solute-binding proteins, family 3
bin008	SOY3_bin008_00799	981	46	119	120	5.606	12.304	12.994	Chemotaxis protein CheV
bin008	SOY3_bin008_00800	771	37	80	104	5.737	10.524	14.329	N-acetylmuramoyl-L-alanine amidase AmiC precursor
bin008	SOY3_bin008_00801	294	37	147	193	15.045	50.714	69.733	hypothetical protein
bin008	SOY3_bin008_00802	2337	12	43	51	0.614	1.866	2.318	Penicillin-binding protein 1F
bin008	SOY3_bin008_00803	789	10	36	38	1.515	4.628	5.116	hypothetical protein
bin008	SOY3_bin008_00804	1452	6	13	24	0.494	0.908	1.756	hypothetical protein
bin008	SOY3_bin008_00805	1140	21	59	69	2.202	5.249	6.429	Capsular glucan synthase
bin008	SOY3_bin008_00806	1272	43	175	225	4.041	13.954	18.790	GDP-mannose-dependent alpha-(1-6)-phosphatidylinositol monomannoside mannosyltransferase
bin008	SOY3_bin008_00807	1566	34	85	89	2.596	5.505	6.037	hypothetical protein
bin008	SOY3_bin008_00808	441	39	30	30	10.572	6.900	7.226	hypothetical protein
bin008	SOY3_bin008_00809	2634	42	101	99	1.906	3.889	3.993	Protease 3 precursor
bin008	SOY3_bin008_00810	2559	5	29	31	0.234	1.149	1.287	(Dimethylallyl)adenosine tRNA methylthiotransferase MiaB
bin008	SOY3_bin008_00811	345	5	46	51	1.733	13.524	15.703	hypothetical protein
bin008	SOY3_bin008_00812	495	3	19	27	0.725	3.893	5.794	Zinc uptake regulation protein
bin008	SOY3_bin008_00813	816	3	8	9	0.440	0.994	1.172	Manganese transport system membrane protein MntB
bin008	SOY3_bin008_00814	795	2	14	14	0.301	1.786	1.871	High-affinity zinc uptake system ATP-binding protein ZnuC
bin008	SOY3_bin008_00815	921	7	19	26	0.909	2.092	2.999	High-affinity zinc uptake system binding-protein ZnuA precursor
bin008	SOY3_bin008_00816	201	4	6	7	2.379	3.028	3.699	hypothetical protein
bin008	SOY3_bin008_00817	1695	42	153	160	2.962	9.155	10.027	NADH peroxidase
bin008	SOY3_bin008_00818	1113	7	26	31	0.752	2.369	2.959	hypothetical protein
bin008	SOY3_bin008_00819	282	7	9	6	2.968	3.237	2.260	CRISPR-associated endoribonuclease Cas2
bin008	SOY3_bin008_00820	1032	41	40	50	4.750	3.931	5.147	CRISPR-associated endonuclease Cas1
bin008	SOY3_bin008_00821	630	91	121	148	17.268	19.480	24.955	PD-(D/E)XK nuclease superfamily protein
bin008	SOY3_bin008_00822	870	174	304	380	23.910	35.441	46.397	hypothetical protein
bin008	SOY3_bin008_00823	1701	132	312	336	9.277	18.604	20.983	CRISPR-associated protein (Cas_Csd1)
bin008	SOY3_bin008_00824	651	97	252	278	17.813	39.262	45.362	CRISPR-associated protein Cas5
bin008	SOY3_bin008_00825	97	153	311	405	188.567	325.195	443.520	tRNA-Ala(tgc)
bin008	SOY3_bin008_00826	2220	30	80	76	1.616	3.655	3.637	helicase Cas3
bin008	SOY3_bin008_00827	77	0	0	0	0.000	0.000	0.000	tRNA-Arg(cct)
bin008	SOY3_bin008_00828	1464	109	232	305	8.901	16.073	22.130	putative periplasmic serine endoprotease DegP-like precursor

bin008	SOY3_bin008_00829	891	29	90	93	3.891	10.245	11.088	4-diphosphocytidyl-2-C-methyl-D-erythritol kinase
bin008	SOY3_bin008_00830	76	0	2	2	0.000	2.669	2.795	tRNA-Gln(ttg)
bin008	SOY3_bin008_00831	945	13	164	167	1.645	17.602	18.772	Ribose-phosphate pyrophosphokinase
bin008	SOY3_bin008_00832	624	28	177	192	5.364	28.770	32.685	50S ribosomal protein L25
bin008	SOY3_bin008_00833	621	0	3	3	0.000	0.490	0.513	Peptidyl-tRNA hydrolase
bin008	SOY3_bin008_00834	513	75	233	289	17.478	46.067	59.843	RNA polymerase-binding transcription factor CarD
bin008	SOY3_bin008_00835	1248	42	129	116	4.023	10.484	9.874	hypothetical protein
bin008	SOY3_bin008_00836	1464	20	60	84	1.633	4.157	6.095	TPR repeat-containing protein YfgC precursor
bin008	SOY3_bin008_00837	417	10	22	29	2.867	5.351	7.387	hypothetical protein
bin008	SOY3_bin008_00838	843	6	28	33	0.851	3.369	4.158	hypothetical protein
bin008	SOY3_bin008_00839	5490	108	287	371	2.352	5.302	7.178	hypothetical protein
bin008	SOY3_bin008_00840	609	14	216	200	2.748	35.974	34.885	Peptidyl-prolyl cis-trans isomerase cyp18
bin008	SOY3_bin008_00841	279	17	18	28	7.284	6.544	10.661	Periplasmic [Fe] hydrogenase large subunit
bin008	SOY3_bin008_00842	1140	27	46	65	2.831	4.093	6.057	2-oxoglutarate oxidoreductase subunit KorA
bin008	SOY3_bin008_00843	840	77	77	114	10.959	9.298	14.416	2-oxoglutarate oxidoreductase subunit KorB
bin008	SOY3_bin008_00844	657	43	49	81	7.824	7.565	13.096	Pyruvate synthase subunit PorC
bin008	SOY3_bin008_00845	399	2	10	15	0.599	2.542	3.993	Transcriptional regulatory protein ros
bin008	SOY3_bin008_00846	732	11	24	48	1.796	3.325	6.966	Phytochrome-like protein cph2
bin008	SOY3_bin008_00847	159	0	1	0	0.000	0.638	0.000	hypothetical protein
bin008	SOY3_bin008_00848	1344	3	8	3	0.267	0.604	0.237	Threonylcarbamoyladenosine tRNA methylthiotransferase MtaB
bin008	SOY3_bin008_00849	549	1	5	7	0.218	0.924	1.354	hypothetical protein
bin008	SOY3_bin008_00850	882	2	23	33	0.271	2.645	3.974	hypothetical protein
bin008	SOY3_bin008_00851	264	1	13	14	0.453	4.995	5.633	hypothetical protein
bin008	SOY3_bin008_00852	630	1	7	9	0.190	1.127	1.518	Guanylate kinase
bin008	SOY3_bin008_00853	870	23	92	107	3.160	10.726	13.065	tetratricopeptide repeat protein
bin008	SOY3_bin008_00854	861	15	15	18	2.083	1.767	2.221	putative nicotinate-nucleotide adenylyltransferase
bin008	SOY3_bin008_00855	1713	30	61	69	2.094	3.612	4.279	Single-stranded-DNA-specific exonuclease RecJ
bin008	SOY3_bin008_00856	1494	2254	8299	10785	180.364	563.417	766.830	hypothetical protein
bin008	SOY3_bin008_00857	525	3	15	15	0.683	2.898	3.035	hypothetical protein
bin008	SOY3_bin008_00858	276	2	5	8	0.866	1.837	3.079	hypothetical protein
bin008	SOY3_bin008_00859	741	4	15	20	0.645	2.053	2.867	putative sulfate transport protein CysZ
bin008	SOY3_bin008_00860	1893	6	32	43	0.379	1.715	2.413	potassium transport protein Kup
bin008	SOY3_bin008_00861	1053	4	14	29	0.454	1.349	2.925	Sec-independent protein translocase protein TatC
bin008	SOY3_bin008_00862	498	17	27	60	4.081	5.499	12.798	hypothetical protein
bin008	SOY3_bin008_00863	201	7	14	12	4.163	7.065	6.342	Divalent metal cation transporter MntH
bin008	SOY3_bin008_00864	1287	59	151	161	5.480	11.900	13.289	Citrate synthase 1
bin008	SOY3_bin008_00865	912	3	6	18	0.393	0.667	2.097	formate dehydrogenase accessory protein FdhE
bin008	SOY3_bin008_00866	384	188	186	299	58.529	49.129	82.712	Formate dehydrogenase subunit alpha precursor
bin008	SOY3_bin008_00867	696	136	135	197	23.360	19.673	30.067	7-cyano-7-deazaguanine synthase
bin008	SOY3_bin008_00868	636	25	48	60	4.699	7.655	10.021	7-carboxy-7-deazaguanine synthase
bin008	SOY3_bin008_00869	375	16	37	56	5.101	10.007	15.863	6-carboxy-5,6,7,8-tetrahydropterin synthase
bin008	SOY3_bin008_00870	423	5	30	25	1.413	7.193	6.278	NADPH-dependent 7-cyano-7-deazaguanine reductase
bin008	SOY3_bin008_00871	2406	76	216	271	3.776	9.106	11.965	Autoinducer 2 sensor kinase/phosphatase LuxQ
bin008	SOY3_bin008_00872	1593	32	70	82	2.401	4.457	5.468	(Dimethylallyl)adenosine tRNA methylthiotransferase MiaB
bin008	SOY3_bin008_00873	2460	73	285	327	3.548	11.751	14.120	Lon protease 1
bin008	SOY3_bin008_00874	1233	46	242	250	4.460	19.907	21.538	ATP-dependent Clp protease ATP-binding subunit ClpX
bin008	SOY3_bin008_00875	615	17	87	87	3.305	14.348	15.027	ATP-dependent Clp protease proteolytic subunit precursor
bin008	SOY3_bin008_00876	1320	13	210	221	1.177	16.136	17.785	Trigger factor
bin008	SOY3_bin008_00877	1590	16	52	40	1.203	3.317	2.672	Acetophenone carboxylase delta subunit
bin008	SOY3_bin008_00878	462	30	42	48	7.763	9.221	11.036	hypothetical protein
bin008	SOY3_bin008_00879	270	19	71	79	8.413	26.672	31.081	hypothetical protein
bin008	SOY3_bin008_00880	441	26	40	38	7.048	9.200	9.153	Polymer-forming cytoskeletal
bin008	SOY3_bin008_00881	225	14	6	14	7.439	2.705	6.610	hypothetical protein
bin008	SOY3_bin008_00882	744	6	62	60	0.964	8.452	8.567	S-methyl-5'-thioinosine phosphorylase
bin008	SOY3_bin008_00883	516	4	25	31	0.927	4.914	6.382	Adenine phosphoribosyltransferase
bin008	SOY3_bin008_00884	1323	3	17	15	0.271	1.303	1.204	5-methylthioadenosine/S-adenosylhomocysteine deaminase
bin008	SOY3_bin008_00885	243	3	69	49	1.476	28.800	21.420	hypothetical protein
bin008	SOY3_bin008_00886	1227	10	56	53	0.974	4.629	4.588	Succinyl-diaminopimelate desuccinylase
bin008	SOY3_bin008_00887	453	1	8	12	0.264	1.791	2.814	Guanine deaminase
bin008	SOY3_bin008_00888	1902	11	26	31	0.691	1.386	1.731	tRNA uridine 5-carboxymethylaminomethyl modification enzyme MnmG
bin008	SOY3_bin008_00889	1014	93	357	334	10.965	35.710	34.990	hypothetical protein
bin008	SOY3_bin008_00890	816	26	87	95	3.809	10.814	12.367	Magnesium and cobalt efflux protein CorC
bin008	SOY3_bin008_00891	1530	33	61	68	2.579	4.044	4.721	Apolipoprotein N-acyltransferase
bin008	SOY3_bin008_00892	1053	24	44	70	2.725	4.238	7.062	Peptide chain release factor 2
bin008	SOY3_bin008_00893	867	11	29	31	1.517	3.393	3.798	putative diguanylate cyclase YcdT
bin008	SOY3_bin008_00894	831	9	31	29	1.295	3.784	3.707	Flagellum site-determining protein YlxH
bin008	SOY3_bin008_00895	276	114	266	360	49.379	97.752	138.555	Integration host factor subunit beta

bin008	SOY3_bin008_00896	867	69	145	176	9.514	16.963	21.564	hypothetical protein
bin008	SOY3_bin008_00897	147	1	4	4	0.813	2.760	2.890	hypothetical protein
bin008	SOY3_bin008_00898	570	3	19	19	0.629	3.381	3.541	hypothetical protein
bin008	SOY3_bin008_00899	879	124	334	374	16.865	38.540	45.197	4-hydroxy-tetrahydrodipicolinate synthase
bin008	SOY3_bin008_00900	1521	48	114	85	3.773	7.602	5.936	putative ABC transporter ATP-binding protein YlmA
bin008	SOY3_bin008_00901	906	71	131	141	9.369	14.666	16.532	putative endopeptidase p60 precursor
bin008	SOY3_bin008_00902	438	10	44	47	2.729	10.189	11.399	flagellar basal body-associated protein FliL
bin008	SOY3_bin008_00903	2184	125	224	311	6.842	10.403	15.126	Malate synthase G
bin008	SOY3_bin008_00904	510	14	28	38	3.282	5.569	7.915	hypothetical protein
bin008	SOY3_bin008_00905	636	49	139	184	9.211	22.167	30.732	NADH dehydrogenase
bin008	SOY3_bin008_00906	405	8	17	30	2.361	4.257	7.869	Virulence regulon transcriptional activator VirF
bin008	SOY3_bin008_00907	771	4	21	38	0.620	2.763	5.236	Hemolysin A
bin008	SOY3_bin008_00908	1353	24	66	73	2.121	4.948	5.731	Transcriptional regulatory protein ZraR
bin008	SOY3_bin008_00909	561	0	1	0	0.000	0.181	0.000	hypothetical protein
bin008	SOY3_bin008_00910	1452	43	178	203	3.540	12.434	14.851	Threonine synthase
bin008	SOY3_bin008_00911	507	3	12	21	0.707	2.401	4.400	Glycine zipper 2TM domain protein
bin008	SOY3_bin008_00912	1299	6	33	32	0.552	2.577	2.617	Phenylacetate-coenzyme A ligase
bin008	SOY3_bin008_00913	1458	21	143	157	1.722	9.948	11.439	Replicative DNA helicase
bin008	SOY3_bin008_00914	525	15	352	336	3.416	68.005	67.984	50S ribosomal protein L9
bin008	SOY3_bin008_00915	258	8	167	160	3.707	65.653	65.876	30S ribosomal protein S18
bin008	SOY3_bin008_00916	309	12	205	227	4.643	67.290	78.036	30S ribosomal protein S6
bin008	SOY3_bin008_00917	2124	116	239	324	6.529	11.413	16.204	Phosphoserine phosphatase RsbU
bin008	SOY3_bin008_00918	390	52	93	103	15.940	24.187	28.054	Endonuclease YhcR precursor
bin008	SOY3_bin008_00919	396	24	82	87	7.245	21.003	23.337	Signal transduction histidine-protein kinase BarA
bin008	SOY3_bin008_00920	774	33	78	122	5.097	10.221	16.744	Coenzyme F420:L-glutamate ligase
bin008	SOY3_bin008_00921	786	99	154	195	15.058	19.873	26.354	Ferredoxin-3
bin008	SOY3_bin008_00922	1710	27	113	147	1.888	6.703	9.132	GTPase Era
bin008	SOY3_bin008_00923	621	7	68	72	1.348	11.106	12.316	hypothetical protein
bin008	SOY3_bin008_00924	336	12	42	46	4.270	12.678	14.543	Putative anti-sigma factor antagonist BtrV
bin008	SOY3_bin008_00925	438	13	46	35	3.548	10.652	8.488	Serine/threonine-protein kinase BtrW
bin008	SOY3_bin008_00926	810	7	38	43	1.033	4.758	5.639	Flagellin N-methylase
bin008	SOY3_bin008_00927	522	25	79	119	5.726	15.350	24.216	Bifunctional protein HldE
bin008	SOY3_bin008_00928	1086	44	120	153	4.844	11.207	14.966	hypothetical protein
bin008	SOY3_bin008_00929	546	10	92	114	2.190	17.090	22.179	hypothetical protein
bin008	SOY3_bin008_00930	543	117	536	674	25.759	100.120	131.853	Chemotaxis protein CheW
bin008	SOY3_bin008_00931	2106	488	1048	1255	27.702	50.473	63.302	Methyl-accepting chemotaxis protein III
bin008	SOY3_bin008_00932	2166	372	886	1164	20.532	41.489	57.085	Chemotaxis protein CheA
bin008	SOY3_bin008_00933	324	75	169	203	27.673	52.905	66.555	STAS domain protein
bin008	SOY3_bin008_00934	363	86	186	221	28.323	51.971	64.672	hypothetical protein
bin008	SOY3_bin008_00935	129	3	5	5	2.780	3.931	4.117	hypothetical protein
bin008	SOY3_bin008_00936	1359	27	89	112	2.375	6.642	8.754	Putative hydroxypyruvate reductase
bin008	SOY3_bin008_00937	1131	8	11	11	0.846	0.986	1.033	ABC transporter permease YtrF precursor
bin008	SOY3_bin008_00938	702	0	8	11	0.000	1.156	1.665	ABC transporter ATP-binding protein YtrE
bin008	SOY3_bin008_00939	1107	1	12	15	0.108	1.099	1.439	Multidrug export protein EmrA
bin008	SOY3_bin008_00940	870	10	42	51	1.374	4.896	6.227	NADH pyrophosphatase
bin008	SOY3_bin008_00941	2406	33	92	113	1.640	3.878	4.989	Phenylalanine--tRNA ligase beta subunit
bin008	SOY3_bin008_00942	1047	22	32	31	2.512	3.100	3.145	Phenylalanine--tRNA ligase alpha subunit
bin008	SOY3_bin008_00943	354	24	189	186	8.105	54.152	55.814	50S ribosomal protein L20
bin008	SOY3_bin008_00944	198	26	145	151	15.698	74.278	81.011	50S ribosomal protein L35
bin008	SOY3_bin008_00945	474	68	397	424	17.150	84.951	95.021	Translation initiation factor IF-3
bin008	SOY3_bin008_00946	1944	65	436	466	3.997	22.748	25.464	Threonine--tRNA ligase 2
bin008	SOY3_bin008_00947	75	0	5	4	0.000	6.762	5.665	tRNA-Val(gac)
bin008	SOY3_bin008_00948	609	9	22	27	1.767	3.664	4.710	Prefoldin subunit alpha
bin008	SOY3_bin008_00949	243	9	20	30	4.428	8.348	13.114	hypothetical protein
bin008	SOY3_bin008_00950	1215	20	75	133	1.968	6.261	11.628	hypothetical protein
bin008	SOY3_bin008_00951	903	31	161	152	4.104	18.084	17.881	hypothetical protein
bin008	SOY3_bin008_00952	318	115	350	404	43.233	111.634	134.953	hypothetical protein
bin008	SOY3_bin008_00953	537	212	384	479	47.196	72.529	94.753	Transposase, Mutator family
bin008	SOY3_bin008_00954	501	4	11	16	0.954	2.227	3.392	hypothetical protein
bin008	SOY3_bin008_00955	492	4	9	11	0.972	1.855	2.375	hypothetical protein
bin008	SOY3_bin008_00956	1362	3	19	23	0.263	1.415	1.794	WD40-like Beta Propeller Repeat protein
bin008	SOY3_bin008_00957	546	45	96	127	9.853	17.833	24.708	Cytochrome c-type protein TorC
bin008	SOY3_bin008_00958	1437	275	428	572	22.878	30.209	42.283	Hydroxylamine oxidoreductase precursor
bin008	SOY3_bin008_00959	1095	11	89	82	1.201	8.244	7.955	Glycerol dehydrogenase
bin008	SOY3_bin008_00960	4536	150	422	443	3.953	9.436	10.374	Signal transduction histidine-protein kinase BarA
bin008	SOY3_bin008_00961	1125	53	120	150	5.632	10.819	14.163	Cyclic di-GMP phosphodiesterase response regulator RpfG
bin008	SOY3_bin008_00962	1146	56	107	148	5.842	9.470	13.719	Response regulator PleD

bin008	SOY3_bin008_00963	723	71	118	181	11.740	16.554	26.593	hypothetical protein
bin008	SOY3_bin008_00964	294	14	33	58	5.693	11.385	20.956	hypothetical protein
bin008	SOY3_bin008_00965	468	4	24	34	1.022	5.201	7.717	Phosphatidylglycerophosphatase A
bin008	SOY3_bin008_00966	657	29	27	34	5.277	4.168	5.497	Magnesium transporter MgtE
bin008	SOY3_bin008_00967	711	5	7	5	0.841	0.999	0.747	High-affinity branched-chain amino acid transport ATP-binding protein LivF
bin008	SOY3_bin008_00968	729	2	6	3	0.328	0.835	0.437	Lipopolysaccharide export system ATP-binding protein LptB
bin008	SOY3_bin008_00969	1023	2	17	13	0.234	1.685	1.350	High-affinity branched-chain amino acid transport system permease protein LivH
bin008	SOY3_bin008_00970	885	10	14	11	1.351	1.604	1.320	High-affinity branched-chain amino acid transport system permease protein LivH
bin008	SOY3_bin008_00971	1248	4	5	8	0.383	0.406	0.681	hypothetical protein
bin008	SOY3_bin008_00972	1326	2	4	4	0.180	0.306	0.320	4-aminobutyrate aminotransferase GabT
bin008	SOY3_bin008_00973	1701	3	16	25	0.211	0.954	1.561	Limonene hydroxylase
bin008	SOY3_bin008_00974	1455	5	28	28	0.411	1.952	2.044	Succinate-semialdehyde dehydrogenase [NADP(+)] GabD
bin008	SOY3_bin008_00975	1530	20	95	101	1.563	6.298	7.012	Nitrogen assimilation regulatory protein
bin008	SOY3_bin008_00976	315	161	305	401	61.103	98.207	135.227	PTS system glucitol/sorbitol-specific transporter subunit IIA
bin008	SOY3_bin008_00977	597	61	144	141	12.215	24.465	25.088	Transglutaminase-like superfamily protein
bin008	SOY3_bin008_00978	564	107	125	178	22.680	22.479	33.525	CYTH domain protein
bin008	SOY3_bin008_00979	864	3	10	20	0.415	1.174	2.459	putative inner membrane transporter yiJE
bin008	SOY3_bin008_00980	945	1	13	9	0.127	1.395	1.012	hypothetical protein
bin008	SOY3_bin008_00981	753	3	5	4	0.476	0.673	0.564	N5-carboxyaminoimidazole ribonucleotide mutase
bin008	SOY3_bin008_00982	840	0	5	7	0.000	0.604	0.885	Argininosuccinate synthase
bin008	SOY3_bin008_00983	1227	3	16	23	0.292	1.323	1.991	hypothetical protein
bin008	SOY3_bin008_00984	1446	16	12	27	1.323	0.842	1.983	C4-dicarboxylate transport transcriptional regulatory protein DctD
bin008	SOY3_bin008_00985	1884	14	28	48	0.888	1.507	2.706	Sporulation kinase E
bin008	SOY3_bin008_00986	402	12	6	15	3.569	1.514	3.964	hypothetical protein
bin008	SOY3_bin008_00987	2391	64	45	46	3.200	1.909	2.044	Benzylsuccinate synthase alpha subunit
bin008	SOY3_bin008_00988	963	16	11	19	1.986	1.159	2.096	4-hydroxyphenylacetate decarboxylase activating enzyme
bin008	SOY3_bin008_00989	87	4	4	3	5.497	4.663	3.663	tRNA-Ser(cag)
bin008	SOY3_bin008_00990	591	5	19	28	1.011	3.261	5.033	Septum formation protein Maf
bin008	SOY3_bin008_00991	819	5	40	51	0.730	4.954	6.615	hypothetical protein
bin008	SOY3_bin008_00992	1371	24	93	115	2.093	6.880	8.910	Dihydrolipoyl dehydrogenase
bin008	SOY3_bin008_00993	1449	29	174	177	2.393	12.180	12.976	putative glycine dehydrogenase (decarboxylating) subunit 2
bin008	SOY3_bin008_00994	1332	22	195	194	1.975	14.849	15.471	putative glycine dehydrogenase (decarboxylating) subunit 1
bin008	SOY3_bin008_00995	375	10	102	105	3.188	27.588	29.743	Glycine cleavage system H protein
bin008	SOY3_bin008_00996	696	20	94	133	3.435	13.699	20.299	Cypemycin methyltransferase
bin008	SOY3_bin008_00997	420	10	57	57	2.846	13.765	14.416	Putative nickel-responsive regulator
bin008	SOY3_bin008_00998	798	4	36	50	0.599	4.576	6.656	GTP cyclohydrolase FolE2
bin008	SOY3_bin008_00999	1956	23	54	64	1.406	2.800	3.476	Autoinducer 2 sensor kinase/phosphatase LuxQ
bin008	SOY3_bin008_01000	627	7	5	17	1.335	0.809	2.880	hypothetical protein
bin008	SOY3_bin008_01001	405	15	26	50	4.428	6.511	13.114	Flagellar basal-body rod protein FlgC
bin008	SOY3_bin008_01002	1011	12	58	77	1.419	5.819	8.090	Transcriptional regulatory protein ZraR
bin008	SOY3_bin008_01003	2967	18	69	75	0.725	2.359	2.685	tetratricopeptide repeat protein
bin008	SOY3_bin008_01004	837	183	273	416	26.138	33.082	52.796	hypothetical protein
bin008	SOY3_bin008_01005	747	153	217	331	24.486	29.464	47.069	hypothetical protein
bin008	SOY3_bin008_01006	2250	18	231	224	0.956	10.413	10.575	Polyribonucleotide nucleotidyltransferase
bin008	SOY3_bin008_01007	270	5	95	80	2.214	35.687	31.474	30S ribosomal protein S15
bin008	SOY3_bin008_01008	981	16	190	204	1.950	19.644	22.090	tRNA pseudouridine synthase B
bin008	SOY3_bin008_01009	978	4	34	60	0.489	3.526	6.517	Bifunctional oligoribonuclease and PAP phosphatase NrnA
bin008	SOY3_bin008_01010	336	1	2	8	0.356	0.604	2.529	Ribosome-binding factor A
bin008	SOY3_bin008_01011	288	5	11	6	2.076	3.874	2.213	hypothetical protein
bin008	SOY3_bin008_01012	2943	35	459	532	1.422	15.819	19.202	Translation initiation factor IF-2
bin008	SOY3_bin008_01013	1404	14	152	209	1.192	10.981	15.813	hypothetical protein
bin008	SOY3_bin008_01014	567	0	28	29	0.000	5.009	5.433	Ribosome maturation factor RimP
bin008	SOY3_bin008_01015	76	1	3	2	1.573	4.004	2.795	tRNA-Gly(ccc)
bin008	SOY3_bin008_01016	786	20	72	95	3.042	9.291	12.839	Flagellar basal-body rod protein FlgG
bin008	SOY3_bin008_01017	783	8	48	53	1.221	6.218	7.190	Flagellar basal-body rod protein FlgG
bin008	SOY3_bin008_01018	972	9	26	45	1.107	2.713	4.918	flagellar basal body P-ring biosynthesis protein FlgA
bin008	SOY3_bin008_01019	723	9	30	37	1.488	4.209	5.436	Flagellar L-ring protein precursor
bin008	SOY3_bin008_01020	1152	16	62	58	1.660	5.459	5.348	Flagellar P-ring protein precursor
bin008	SOY3_bin008_01021	972	34	94	108	4.182	9.809	11.803	Murein DD-endopeptidase MepM
bin008	SOY3_bin008_01022	477	9	26	27	2.256	5.529	6.013	FlgN protein
bin008	SOY3_bin008_01023	2085	34	113	120	1.949	5.497	6.114	Flagellar hook-associated protein 1
bin008	SOY3_bin008_01024	1563	10	34	49	0.765	2.206	3.330	Flagellar hook-associated protein 3
bin008	SOY3_bin008_01025	240	219	586	697	109.088	247.652	308.497	hypothetical protein
bin008	SOY3_bin008_01026	531	64	190	253	14.409	36.292	50.612	Flagellar assembly factor FliW
bin008	SOY3_bin008_01027	318	37	204	221	13.910	65.067	73.824	anti-sigma28 factor FlgM
bin008	SOY3_bin008_01028	426	24	127	145	6.735	30.238	36.157	hypothetical protein
bin008	SOY3_bin008_01029	864	14	29	26	1.937	3.404	3.197	putative inorganic polyphosphate/ATP-NAD kinase

bin008	SOY3_bin008_01030	1764	33	47	56	2.236	2.702	3.372	hypothetical protein
bin008	SOY3_bin008_01031	93	106	58	84	136.260	63.256	95.946	hypothetical protein
bin008	SOY3_bin008_01032	1395	0	25	14	0.000	1.818	1.066	Multidrug resistance protein stp
bin008	SOY3_bin008_01033	174	0	3	5	0.000	1.749	3.052	High molecular weight rubredoxin
bin008	SOY3_bin008_01034	831	28	74	84	4.028	9.032	10.738	hypothetical protein
bin008	SOY3_bin008_01035	375	15	93	101	4.782	25.154	28.610	hypothetical protein
bin008	SOY3_bin008_01036	1431	8	70	56	0.668	4.962	4.157	Aspartyl/glutamyl-tRNA(Asn/Gln) amidotransferase subunit B
bin008	SOY3_bin008_01037	1044	8	68	86	0.916	6.606	8.750	Methylthioribose-1-phosphate isomerase
bin008	SOY3_bin008_01038	1335	112	237	271	10.030	18.006	21.563	GTPase Der
bin008	SOY3_bin008_01039	1779	47	278	327	3.158	15.850	19.525	Methyl-accepting chemotaxis protein McpS
bin008	SOY3_bin008_01040	924	11	35	42	1.423	3.842	4.828	hypothetical protein
bin008	SOY3_bin008_01041	474	4	17	26	1.009	3.638	5.827	hypothetical protein
bin008	SOY3_bin008_01042	2106	26	60	68	1.476	2.890	3.430	Phosphoserine phosphatase RsbU
bin008	SOY3_bin008_01043	1851	12	29	36	0.775	1.589	2.066	H(+)/Cl(-) exchange transporter ClcA
bin008	SOY3_bin008_01044	939	1	14	21	0.127	1.512	2.376	Riboflavin biosynthesis protein RibF
bin008	SOY3_bin008_01045	522	2	15	8	0.458	2.915	1.628	PsbP
bin008	SOY3_bin008_01046	1155	63	303	342	6.521	26.608	31.454	Enterobacterial TraT complement resistance protein
bin008	SOY3_bin008_01047	1863	26	85	92	1.668	4.628	5.246	Serine/threonine-protein kinase PrkC
bin008	SOY3_bin008_01048	909	12	58	45	1.578	6.472	5.259	Serine/threonine phosphatase stp
bin008	SOY3_bin008_01049	1449	35	86	120	2.888	6.020	8.797	Serine/threonine-protein kinase PrkC
bin008	SOY3_bin008_01050	1917	73	388	445	4.552	20.529	24.659	Periplasmic serine endoprotease DegP precursor
bin008	SOY3_bin008_01051	498	79	214	281	18.965	43.585	59.939	hypothetical protein
bin008	SOY3_bin008_01052	3333	124	180	231	4.448	5.478	7.362	Autoinducer 2 sensor kinase/phosphatase LuxQ
bin008	SOY3_bin008_01053	1896	15	87	88	0.946	4.654	4.930	Chaperone protein HtpG
bin008	SOY3_bin008_01054	414	38	150	140	10.973	36.749	35.922	Spore protein SP21
bin008	SOY3_bin008_01055	369	43	119	135	13.931	32.710	38.863	Spore protein SP21
bin008	SOY3_bin008_01056	399	2	114	132	0.599	28.979	35.142	Transcriptional regulatory protein MucR
bin008	SOY3_bin008_01057	276	16	207	189	6.930	76.070	72.742	DNA-binding protein HU
bin008	SOY3_bin008_01058	1383	4	5	14	0.346	0.367	1.075	Hemolysin secretion protein D, chromosomal
bin008	SOY3_bin008_01059	2694	1	10	8	0.044	0.376	0.315	Alpha-hemolysin translocation ATP-binding protein HlyB
bin008	SOY3_bin008_01060	1728	13	34	51	0.899	1.996	3.135	Phage portal protein, SPP1 Gp6-like
bin008	SOY3_bin008_01061	369	4	4	2	1.296	1.099	0.576	Zinc ribbon domain protein
bin008	SOY3_bin008_01062	543	0	11	14	0.000	2.055	2.739	hypothetical protein
bin008	SOY3_bin008_01063	972	5	22	19	0.615	2.296	2.076	hypothetical protein
bin008	SOY3_bin008_01064	720	6	15	14	0.996	2.113	2.066	hypothetical protein
bin008	SOY3_bin008_01065	717	4	21	19	0.667	2.971	2.815	hypothetical protein
bin008	SOY3_bin008_01066	912	2	19	20	0.262	2.113	2.330	hypothetical protein
bin008	SOY3_bin008_01067	459	1	15	16	0.260	3.315	3.703	hypothetical protein
bin008	SOY3_bin008_01068	558	2	4	10	0.428	0.727	1.904	hypothetical protein
bin008	SOY3_bin008_01069	666	2	4	5	0.359	0.609	0.797	F5/8 type C domain protein
bin008	SOY3_bin008_01070	195	0	0	0	0.000	0.000	0.000	hypothetical protein
bin008	SOY3_bin008_01071	1707	2	8	10	0.140	0.475	0.622	Transglutaminase-like superfamily protein
bin008	SOY3_bin008_01072	468	3	6	2	0.766	1.300	0.454	hypothetical protein
bin008	SOY3_bin008_01073	1422	7	22	10	0.588	1.569	0.747	hypothetical protein
bin008	SOY3_bin008_01074	1923	32	44	50	1.989	2.321	2.762	Agglutinin receptor precursor
bin008	SOY3_bin008_01075	417	3	8	6	0.860	1.946	1.528	hypothetical protein
bin008	SOY3_bin008_01076	732	5	24	24	0.817	3.325	3.483	hypothetical protein
bin008	SOY3_bin008_01077	297	1	9	11	0.403	3.074	3.934	hypothetical protein
bin008	SOY3_bin008_01078	2067	2	19	32	0.116	0.932	1.645	hypothetical protein
bin008	SOY3_bin008_01079	684	3	12	15	0.524	1.779	2.330	hypothetical protein
bin008	SOY3_bin008_01080	1194	38	58	79	3.805	4.927	7.028	Ribosomal RNA large subunit methyltransferase I
bin008	SOY3_bin008_01081	1125	87	179	206	9.245	16.138	19.451	GTPase Obg
bin008	SOY3_bin008_01082	858	39	95	110	5.434	11.230	13.619	PBP superfamily domain protein
bin008	SOY3_bin008_01083	3558	118	280	301	3.965	7.982	8.986	Sensory/regulatory protein RpfC
bin008	SOY3_bin008_01084	354	9	21	26	3.039	6.017	7.802	Chemotaxis protein CheY
bin008	SOY3_bin008_01085	615	20	48	74	3.888	7.916	12.782	CheY-P phosphatase CheC
bin008	SOY3_bin008_01086	1299	9	24	33	0.828	1.874	2.699	Divalent metal cation transporter MntH
bin008	SOY3_bin008_01087	1257	4	17	22	0.380	1.372	1.859	Magnesium transporter MgtE
bin008	SOY3_bin008_01088	162	2	9	5	1.476	5.635	3.279	hypothetical protein
bin008	SOY3_bin008_01089	540	8	63	78	1.771	11.833	15.344	hypothetical protein
bin008	SOY3_bin008_01090	675	9	46	53	1.594	6.912	8.341	hypothetical protein
bin008	SOY3_bin008_01091	2445	7	20	24	0.342	0.830	1.043	Phytochrome-like protein cph2
bin008	SOY3_bin008_01092	1707	13	113	128	0.910	6.714	7.965	Glutamine--tRNA ligase
bin008	SOY3_bin008_01093	612	1	3	9	0.195	0.497	1.562	Ubiquinone/menaquinone biosynthesis C-methyltransferase UbiE
bin008	SOY3_bin008_01094	327	8	40	42	2.925	12.407	13.644	hypothetical protein
bin008	SOY3_bin008_01095	243	4	47	60	1.968	19.618	26.229	Nitrogen fixation protein of unknown function
bin008	SOY3_bin008_01096	2925	66	100	131	2.698	3.468	4.757	Autoinducer 2 sensor kinase/phosphatase LuxQ

bin008	SOY3_bin008_01097	297	177	361	414	71.246	123.284	148.072	hypothetical protein
bin008	SOY3_bin008_01098	378	117	218	250	37.003	58.495	70.255	hypothetical protein
bin008	SOY3_bin008_01099	192	50	70	98	31.133	36.979	54.219	hypothetical protein
bin008	SOY3_bin008_01100	2109	84	123	183	4.762	5.915	9.217	putative sensor histidine kinase pdtaS
bin008	SOY3_bin008_01101	267	16	71	62	7.164	26.971	24.667	hypothetical protein
bin008	SOY3_bin008_01102	558	19	57	75	4.071	10.361	14.278	phosphodiesterase
bin008	SOY3_bin008_01103	933	5	19	19	0.641	2.066	2.163	hypothetical protein
bin008	SOY3_bin008_01104	1179	6	35	21	0.608	3.011	1.892	hypothetical protein
bin008	SOY3_bin008_01105	927	8	48	50	1.032	5.252	5.730	Agglutinin receptor precursor
bin008	SOY3_bin008_01106	1086	2	26	34	0.220	2.428	3.326	GDSL-like Lipase/Acylhydrolase
bin008	SOY3_bin008_01107	1065	4	88	78	0.449	8.381	7.780	Localisation of periplasmic protein complexes
bin008	SOY3_bin008_01108	261	7	26	17	3.206	10.104	6.919	hypothetical protein
bin008	SOY3_bin008_01109	534	5	22	20	1.119	4.179	3.978	hypothetical protein
bin008	SOY3_bin008_01110	312	1	28	21	0.383	9.102	7.150	hypothetical protein
bin008	SOY3_bin008_01111	309	1	21	19	0.387	6.893	6.532	hypothetical protein
bin008	SOY3_bin008_01112	876	3	25	32	0.409	2.895	3.880	lipoprotein NlpI
bin008	SOY3_bin008_01113	1200	29	120	143	2.889	10.143	12.659	Arginine biosynthesis bifunctional protein ArgJ
bin008	SOY3_bin008_01114	642	3	10	18	0.559	1.580	2.978	5'-deoxynucleotidase YfbR
bin008	SOY3_bin008_01115	471	0	8	8	0.000	1.723	1.804	hypothetical protein
bin008	SOY3_bin008_01116	882	13	112	110	1.762	12.880	13.248	ATP phosphoribosyltransferase
bin008	SOY3_bin008_01117	450	4	29	42	1.063	6.536	9.914	phosphoribosyl-AMP cyclohydrolase
bin008	SOY3_bin008_01118	1053	1	25	23	0.114	2.408	2.320	Dual-specificity RNA methyltransferase RlmN
bin008	SOY3_bin008_01119	1323	10	60	79	0.904	4.600	6.343	CCA-adding enzyme
bin008	SOY3_bin008_01120	1296	13	70	68	1.199	5.478	5.574	Dihydroorotase
bin008	SOY3_bin008_01121	939	11	40	53	1.400	4.321	5.996	Aspartate carbamoyltransferase
bin008	SOY3_bin008_01122	1530	551	2751	3201	43.053	182.370	222.241	hypothetical protein
bin008	SOY3_bin008_01123	495	110	431	505	26.566	88.313	108.372	AmiS/UreI family transporter
bin008	SOY3_bin008_01124	1824	6	70	62	0.393	3.892	3.611	Glutamine--fructose-6-phosphate aminotransferase [isomerizing]
bin008	SOY3_bin008_01125	1839	21	86	65	1.365	4.743	3.755	N-acetylmuramoyl-L-alanine amidase AmiA precursor
bin008	SOY3_bin008_01126	687	5	29	21	0.870	4.282	3.247	Orotidine 5'-phosphate decarboxylase
bin008	SOY3_bin008_01127	240	0	8	8	0.000	3.381	3.541	hypothetical protein
bin008	SOY3_bin008_01128	1482	2	33	44	0.161	2.259	3.154	Nif-specific regulatory protein
bin008	SOY3_bin008_01129	1851	5	4	6	0.323	0.219	0.344	Acyltransferase
bin008	SOY3_bin008_01130	591	6	35	48	1.214	6.007	8.627	Putative ATPase subunit of terminase (gpP-like)
bin008	SOY3_bin008_01131	1002	542	2708	2918	64.666	274.117	309.348	hypothetical protein
bin008	SOY3_bin008_01132	234	99	662	715	50.578	286.944	324.579	hypothetical protein
bin008	SOY3_bin008_01133	1059	19	75	82	2.145	7.183	8.225	Chorismate synthase
bin008	SOY3_bin008_01134	1629	8	32	42	0.587	1.992	2.739	hypothetical protein
bin008	SOY3_bin008_01135	1218	76	198	272	7.460	16.488	23.722	hypothetical protein
bin008	SOY3_bin008_01136	1014	26	58	70	3.065	5.802	7.333	pyrroloquinoline quinone biosynthesis protein PqqE
bin008	SOY3_bin008_01137	255	19	37	51	8.908	14.717	21.245	hypothetical protein
bin008	SOY3_bin008_01138	975	13	22	55	1.594	2.289	5.992	Putative electron transport protein YccM
bin008	SOY3_bin008_01139	402	15	31	36	4.461	7.822	9.513	Chemotaxis protein CheY
bin008	SOY3_bin008_01140	885	0	12	20	0.000	1.375	2.401	hypothetical protein
bin008	SOY3_bin008_01141	1386	2	6	10	0.173	0.439	0.766	Multidrug resistance protein MdtK
bin008	SOY3_bin008_01142	369	2	18	14	0.648	4.948	4.030	Molybdenum-pterin-binding protein MopB
bin008	SOY3_bin008_01143	1188	7	98	65	0.704	8.367	5.812	Antilisterial bacteriocin subtilosin biosynthesis protein AlbA
bin008	SOY3_bin008_01144	2649	8	224	208	0.361	8.577	8.341	Alanine--tRNA ligase
bin008	SOY3_bin008_01145	1068	3	95	82	0.336	9.022	8.156	recombinase A
bin008	SOY3_bin008_01146	294	1	7	12	0.407	2.415	4.336	hypothetical protein
bin008	SOY3_bin008_01147	1092	10	33	26	1.095	3.065	2.529	putative tRNA sulfurtransferase
bin008	SOY3_bin008_01148	306	185	383	472	72.276	126.950	163.851	Bacterial SH3 domain protein
bin008	SOY3_bin008_01149	864	22	19	29	3.044	2.230	3.565	Ribonuclease
bin008	SOY3_bin008_01150	1929	26	109	104	1.611	5.731	5.727	1,4-alpha-glucan branching enzyme GlgB
bin008	SOY3_bin008_01151	1503	51	127	164	4.057	8.570	11.591	4-alpha-glucanotransferase
bin008	SOY3_bin008_01152	1116	17	41	48	1.821	3.726	4.569	hypothetical protein
bin008	SOY3_bin008_01153	1464	88	98	165	7.186	6.790	11.972	Cobyrinic acid A,C-diamide synthase
bin008	SOY3_bin008_01154	228	179	334	398	93.856	148.582	185.429	Dissimilatory sulfite reductase D (DsrD)
bin008	SOY3_bin008_01155	1164	1102	1807	2392	113.181	157.456	218.292	Sulfite reductase, dissimilatory-type subunit beta
bin008	SOY3_bin008_01156	1314	1183	2190	2805	107.630	169.045	226.760	Sulfite reductase, dissimilatory-type subunit alpha
bin008	SOY3_bin008_01157	660	1	3	6	0.181	0.461	0.966	Flagellin N-methylase
bin008	SOY3_bin008_01158	504	14	56	45	3.321	11.270	9.484	hypothetical protein
bin008	SOY3_bin008_01159	1299	3	13	7	0.276	1.015	0.572	Chromosome partition protein Smc
bin008	SOY3_bin008_01160	243	1	7	9	0.492	2.922	3.934	Ferredoxin, 2Fe-2S
bin008	SOY3_bin008_01161	1755	7	40	43	0.477	2.312	2.603	Iron hydrogenase 1
bin008	SOY3_bin008_01162	1176	2	34	26	0.203	2.932	2.349	Stage II sporulation protein E (SpoIIE)
bin008	SOY3_bin008_01163	351	6	15	9	2.044	4.334	2.724	Photosystem I assembly protein Ycf3

bin008	SOY3_bin008_01164	525	1	11	9	0.228	2.125	1.821	CYTH domain protein
bin008	SOY3_bin008_01165	1311	8	37	36	0.730	2.863	2.917	hypothetical protein
bin008	SOY3_bin008_01166	993	2	22	26	0.241	2.247	2.781	Energy-coupling factor transporter ATP-binding protein EcfA2
bin008	SOY3_bin008_01167	699	1	10	11	0.171	1.451	1.672	hydrogenase nickel incorporation protein HypB
bin008	SOY3_bin008_01168	495	3	33	27	0.725	6.762	5.794	putative tRNA (adenine(37)-N6)-methyltransferase
bin008	SOY3_bin008_01169	1185	2	4	6	0.202	0.342	0.538	putative diguanylate cyclase YdaM
bin008	SOY3_bin008_01170	1425	2	103	115	0.168	7.331	8.573	Adenosylhomocysteinase
bin008	SOY3_bin008_01171	924	2	42	39	0.259	4.610	4.484	hypothetical protein
bin008	SOY3_bin008_01172	618	3	5	5	0.580	0.821	0.859	HTH-type transcriptional repressor KstR2
bin008	SOY3_bin008_01173	627	6	6	7	1.144	0.971	1.186	Ubiquinone/menaquinone biosynthesis C-methyltransferase UbiE
bin008	SOY3_bin008_01174	2412	2	7	7	0.099	0.294	0.308	Methyl-accepting chemotaxis protein PctC
bin008	SOY3_bin008_01175	1830	4	13	21	0.261	0.721	1.219	putative ABC transporter ATP-binding protein
bin008	SOY3_bin008_01176	855	23	85	119	3.216	10.083	14.785	Epoxyqueuosine reductase
bin008	SOY3_bin008_01177	357	15	57	63	5.023	16.194	18.746	hypothetical protein
bin008	SOY3_bin008_01178	306	6	14	14	2.344	4.640	4.860	Bacterial SH3 domain protein
bin008	SOY3_bin008_01179	2031	337	789	1020	19.837	39.402	53.348	Methyl-accepting chemotaxis protein 4
bin008	SOY3_bin008_01180	489	28	41	70	6.845	8.504	15.206	Ribonuclease HI
bin008	SOY3_bin008_01181	522	8	12	23	1.832	2.332	4.680	hypothetical protein
bin008	SOY3_bin008_01182	672	9	32	33	1.601	4.830	5.216	putative ABC transporter ATP-binding protein/MT1014
bin008	SOY3_bin008_01183	603	30	50	66	5.948	8.410	11.627	Arylesterase precursor
bin008	SOY3_bin008_01184	774	12	63	45	1.853	8.256	6.176	Type III pantothenate kinase
bin008	SOY3_bin008_01185	735	17	43	59	2.765	5.934	8.527	Cellulose synthase operon protein C precursor
bin008	SOY3_bin008_01186	1314	162	825	895	14.739	63.682	72.353	Enolase
bin008	SOY3_bin008_01187	882	27	85	100	3.660	9.775	12.044	Bifunctional protein FolD protein
bin008	SOY3_bin008_01188	1671	26	48	66	1.860	2.914	4.196	putative protein kinase UbiB
bin008	SOY3_bin008_01189	1308	54	167	220	4.936	12.950	17.867	Methionine gamma-lyase
bin008	SOY3_bin008_01190	774	5	16	11	0.772	2.097	1.510	PGL/p-HBAD biosynthesis glycosyltransferase/MT3031
bin008	SOY3_bin008_01191	723	4	3	2	0.661	0.421	0.294	2-phospho-L-lactate guanylyltransferase
bin008	SOY3_bin008_01192	756	65	131	165	10.279	17.575	23.184	Mannose permease IID component
bin008	SOY3_bin008_01193	324	35	50	60	12.914	15.652	19.671	Phosphocarrier protein HPr
bin008	SOY3_bin008_01194	1782	65	143	175	4.361	8.139	10.432	Phosphoenolpyruvate-protein phosphotransferase
bin008	SOY3_bin008_01195	465	3	15	32	0.771	3.272	7.310	SsrA-binding protein
bin008	SOY3_bin008_01196	525	1	6	14	0.228	1.159	2.833	hypothetical protein
bin008	SOY3_bin008_01197	1389	6	47	49	0.516	3.432	3.747	putative FAD-linked oxidoreductase
bin008	SOY3_bin008_01198	1200	16	53	41	1.594	4.480	3.629	Anaerobic glycerol-3-phosphate dehydrogenase subunit C
bin008	SOY3_bin008_01199	2514	8	72	74	0.380	2.905	3.127	preprotein translocase subunit SecA
bin008	SOY3_bin008_01200	855	8	18	20	1.119	2.135	2.485	Motility protein A
bin008	SOY3_bin008_01201	795	9	30	28	1.353	3.827	3.741	Motility protein B
bin008	SOY3_bin008_01202	294	4	7	7	1.627	2.415	2.529	hypothetical protein
bin008	SOY3_bin008_01203	1449	74	304	434	6.105	21.279	31.816	Glutamate/gamma-aminobutyrate antiporter
bin008	SOY3_bin008_01204	1395	117	614	744	10.027	44.643	56.654	Glutamate decarboxylase beta
bin008	SOY3_bin008_01205	954	31	116	139	3.885	12.333	15.477	hypothetical protein
bin008	SOY3_bin008_01206	1671	39	168	201	2.790	10.197	12.778	Flagellar hook protein FlgE
bin008	SOY3_bin008_01207	723	7	47	53	1.157	6.593	7.787	Basal-body rod modification protein FlgD
bin008	SOY3_bin008_01208	2379	24	66	120	1.206	2.814	5.358	Flagellar hook-length control protein FliK
bin008	SOY3_bin008_01209	1419	2	19	30	0.168	1.358	2.246	ADP-heptose--LPS heptosyltransferase 2
bin008	SOY3_bin008_01210	1749	4	29	25	0.273	1.682	1.518	Glycosyl transferase family 2
bin008	SOY3_bin008_01211	1686	9	49	61	0.638	2.948	3.843	Spore protein YkvP
bin008	SOY3_bin008_01212	342	20	50	68	6.991	14.829	21.121	Putative anti-sigma factor antagonist
bin008	SOY3_bin008_01213	417	6	26	27	1.720	6.324	6.878	Serine-protein kinase RsbW
bin008	SOY3_bin008_01214	483	9	26	23	2.228	5.460	5.058	D-tyrosyl-tRNA(Tyr) deacylase
bin008	SOY3_bin008_01215	402	6	20	27	1.784	5.046	7.135	6-carboxy-5,6,7,8-tetrahydropterin synthase
bin008	SOY3_bin008_01216	3468	27	67	106	0.931	1.960	3.247	DNA polymerase III subunit alpha
bin008	SOY3_bin008_01217	450	20	18	47	5.313	4.057	11.095	putative lyase
bin008	SOY3_bin008_01218	357	21	53	72	7.032	15.058	21.424	Hpt domain protein
bin008	SOY3_bin008_01219	1503	235	442	503	18.692	29.828	35.550	Sensor protein ZraS
bin008	SOY3_bin008_01220	840	72	122	149	10.247	14.731	18.842	Ribosomal RNA small subunit methyltransferase I
bin008	SOY3_bin008_01221	1707	46	77	96	3.222	4.575	5.974	NADH peroxidase
bin008	SOY3_bin008_01222	1266	7	217	358	0.661	17.385	30.039	Periplasmic [Fe] hydrogenase large subunit
bin008	SOY3_bin008_01223	375	4	87	154	1.275	23.531	43.623	Periplasmic [Fe] hydrogenase small subunit precursor
bin008	SOY3_bin008_01224	582	1	7	3	0.205	1.220	0.548	hypothetical protein
bin008	SOY3_bin008_01225	1479	8	29	42	0.647	1.989	3.017	hypothetical protein
bin008	SOY3_bin008_01226	1752	12	13	27	0.819	0.753	1.637	Vitamin B12 transport ATP-binding protein BacA
bin008	SOY3_bin008_01227	603	3	15	19	0.595	2.523	3.347	hypothetical protein
bin008	SOY3_bin008_01228	1911	59	116	135	3.691	6.157	7.504	Globin-coupled histidine kinase
bin008	SOY3_bin008_01229	357	18	31	18	6.028	8.807	5.356	Response regulator MprA
bin008	SOY3_bin008_01230	930	40	44	35	5.142	4.799	3.998	universal stress protein UspE

bin008	SOY3_bin008_01231	1407	32	55	35	2.719	3.965	2.642	succinate dehydrogenase/fumarate reductase iron-sulfur subunit
bin008	SOY3_bin008_01232	678	18	27	13	3.174	4.039	2.037	Nitrate reductase gamma subunit
bin008	SOY3_bin008_01233	135	4	0	3	3.542	0.000	2.361	hypothetical protein
bin008	SOY3_bin008_01234	1179	23	38	30	2.332	3.269	2.703	putative Ni/Fe-hydrogenase 2 b-type cytochrome subunit
bin008	SOY3_bin008_01235	1056	26	24	32	2.943	2.305	3.219	Formate dehydrogenase, nitrate-inducible, iron-sulfur subunit
bin008	SOY3_bin008_01236	1743	37	46	38	2.538	2.677	2.316	High-molecular-weight cytochrome c precursor
bin008	SOY3_bin008_01237	1101	11	23	21	1.194	2.119	2.026	Transcriptional regulator ModE
bin008	SOY3_bin008_01238	2340	53	88	102	2.708	3.814	4.630	C4-dicarboxylate transport sensor protein DctB
bin008	SOY3_bin008_01239	387	34	107	118	10.503	28.043	32.389	Response regulator MprA
bin008	SOY3_bin008_01240	360	43	202	199	14.279	56.912	58.719	Phosphate regulon transcriptional regulatory protein PhoB
bin008	SOY3_bin008_01241	1344	0	9	6	0.000	0.679	0.474	Transcriptional regulatory protein ZraR
bin008	SOY3_bin008_01242	1431	3	4	7	0.251	0.284	0.520	Sensor protein ZraS
bin008	SOY3_bin008_01243	447	1	0	1	0.267	0.000	0.238	Universal stress protein family protein
bin008	SOY3_bin008_01244	939	1	7	8	0.127	0.756	0.905	hypothetical protein
bin008	SOY3_bin008_01245	759	0	3	2	0.000	0.401	0.280	Putative transmembrane protein (Alph_Pro_TM)
bin008	SOY3_bin008_01246	2586	11	18	30	0.509	0.706	1.232	Phosphoenolpyruvate synthase
bin008	SOY3_bin008_01247	420	0	11	1	0.000	2.656	0.253	C4-dicarboxylate transport transcriptional regulatory protein DctD
bin008	SOY3_bin008_01248	2295	11	15	28	0.573	0.663	1.296	Phosphoenolpyruvate synthase
bin008	SOY3_bin008_01249	2520	39	69	98	1.850	2.777	4.131	Phosphoenolpyruvate synthase
bin008	SOY3_bin008_01250	2268	46	106	147	2.425	4.740	6.885	hypothetical protein
bin008	SOY3_bin008_01251	2517	114	308	356	5.415	12.411	15.024	Sensory/regulatory protein RpfC
bin008	SOY3_bin008_01252	93	5	25	34	6.427	27.265	38.835	hypothetical protein
bin008	SOY3_bin008_01253	2622	64	171	205	2.918	6.615	8.305	Phosphoenolpyruvate synthase
bin008	SOY3_bin008_01254	786	13	32	43	1.977	4.129	5.811	Hydroxypyruvate isomerase
bin008	SOY3_bin008_01255	888	37	108	130	4.981	12.336	15.551	2-hydroxy-3-oxopropionate reductase
bin008	SOY3_bin008_01256	483	9	34	44	2.228	7.140	9.677	Bacterial SH3 domain protein
bin008	SOY3_bin008_01257	921	11	38	49	1.428	4.185	5.652	TPR repeat-containing protein YfgC precursor
bin008	SOY3_bin008_01258	2250	18	75	63	0.956	3.381	2.974	Adenylate cyclase 1
bin008	SOY3_bin008_01259	390	13	26	36	3.985	6.762	9.805	Alginate biosynthesis transcriptional regulatory protein AlgB
bin008	SOY3_bin008_01260	918	11	25	34	1.433	2.762	3.934	Sensor protein ZraS
bin008	SOY3_bin008_01261	396	26	52	55	7.849	13.319	14.754	Nitrogen assimilation regulatory protein
bin008	SOY3_bin008_01262	1653	34	81	83	2.459	4.970	5.334	Sensor protein ZraS
bin008	SOY3_bin008_01263	1806	35	95	102	2.317	5.335	5.999	Non-motile and phage-resistance protein
bin008	SOY3_bin008_01264	1005	3	28	30	0.357	2.826	3.171	Peptide methionine sulfoxide reductase MsrA/MsrB
bin008	SOY3_bin008_01265	900	30	44	51	3.985	4.959	6.019	Prenyltransferase and squalene oxidase repeat protein
bin008	SOY3_bin008_01266	1044	42	44	62	4.809	4.275	6.308	hypothetical protein
bin008	SOY3_bin008_01267	786	36	62	66	5.476	8.001	8.920	hypothetical protein
bin008	SOY3_bin008_01268	510	13	40	45	3.047	7.955	9.373	hypothetical protein
bin008	SOY3_bin008_01269	2088	24	93	100	1.374	4.518	5.087	Localization factor PodJL
bin008	SOY3_bin008_01270	303	0	0	1	0.000	0.000	0.351	hypothetical protein
bin008	SOY3_bin008_01271	855	767	1896	2550	107.244	224.919	316.814	Molybdenum storage protein subunit beta
bin008	SOY3_bin008_01272	207	11	16	31	6.353	7.840	15.908	hypothetical protein
bin008	SOY3_bin008_01273	372	6	14	27	1.928	3.817	7.710	lineage-specific thermal regulator protein
bin008	SOY3_bin008_01274	1146	4	23	18	0.417	2.036	1.668	Radical SAM superfamily protein
bin008	SOY3_bin008_01275	1311	2	9	3	0.182	0.696	0.243	Aspartate aminotransferase
bin008	SOY3_bin008_01276	1062	2	7	11	0.225	0.669	1.100	sn-glycerol-3-phosphate import ATP-binding protein UgpC
bin008	SOY3_bin008_01277	756	4	5	5	0.633	0.671	0.703	Sulfate transport system permease protein CysT
bin008	SOY3_bin008_01278	975	51	186	244	6.253	19.349	26.584	Molybdate-binding periplasmic protein precursor
bin008	SOY3_bin008_01279	1215	16	36	32	1.574	3.005	2.798	Light-independent protochlorophyllide reductase subunit B
bin008	SOY3_bin008_01280	1299	11	35	47	1.012	2.733	3.843	Nitrogenase vanadium-iron protein beta chain
bin008	SOY3_bin008_01281	768	4	16	26	0.623	2.113	3.596	Nitrogenase iron protein 1
bin008	SOY3_bin008_01282	1014	5	29	37	0.589	2.901	3.876	Oxygen-independent coproporphyrinogen-III oxidase 1
bin008	SOY3_bin008_01283	1548	12	18	29	0.927	1.179	1.990	Periplasmic [NiFe] hydrogenase large subunit precursor
bin008	SOY3_bin008_01284	504	7	29	34	1.660	5.836	7.166	hypothetical protein
bin008	SOY3_bin008_01285	648	8	14	16	1.476	2.191	2.623	GDSL-like Lipase/Acylhydrolase
bin008	SOY3_bin008_01286	405	0	4	1	0.000	1.002	0.262	CGGC domain protein
bin008	SOY3_bin008_01287	726	0	8	4	0.000	1.118	0.585	hypothetical protein
bin008	SOY3_bin008_01288	285	132	282	352	55.370	100.360	131.198	hypothetical protein
bin008	SOY3_bin008_01289	1728	14	113	107	0.969	6.633	6.578	putative oxidoreductase YdhV
bin008	SOY3_bin008_01290	1350	5	14	26	0.443	1.052	2.046	Cyclic pyranopterin monophosphate synthase
bin008	SOY3_bin008_01291	1188	15	27	23	1.509	2.305	2.057	undecaprenyldiphospho-muramoylpentapeptide beta-N- acetylglucosaminyltransferase
bin008	SOY3_bin008_01292	603	21	57	63	4.163	9.588	11.098	Putative phosphoserine phosphatase 2
bin008	SOY3_bin008_01293	4005	126	281	362	3.761	7.116	9.601	Aerobic respiration control sensor protein ArcB
bin008	SOY3_bin008_01294	1722	8	34	37	0.555	2.003	2.282	hypothetical protein
bin008	SOY3_bin008_01295	978	14	31	73	1.711	3.215	7.929	hypothetical protein
bin008	SOY3_bin008_01296	1119	16	28	49	1.709	2.538	4.652	GDP-mannose-dependent alpha-(1-6)-phosphatidylinositol monomannoside mannosyltransferase
bin008	SOY3_bin008_01297	1287	16	33	62	1.486	2.601	5.117	Putative teichuronic acid biosynthesis glycosyltransferase TuaC

bin008	SOY3_bin008_01298	1254	3	4	15	0.286	0.324	1.271	undecaprenyldiphospho-muramoylpentapeptide beta-N- acetylglucosaminyltransferase
bin008	SOY3_bin008_01299	1446	8	52	59	0.661	3.647	4.334	Transcriptional regulatory protein ZraR
bin008	SOY3_bin008_01300	729	8	50	53	1.312	6.957	7.723	High-affinity branched-chain amino acid transport ATP-binding protein LivF
bin008	SOY3_bin008_01301	768	9	62	68	1.401	8.188	9.405	Lipopolysaccharide export system ATP-binding protein LptB
bin008	SOY3_bin008_01302	1017	9	66	87	1.058	6.582	9.087	High-affinity branched-chain amino acid transport system permease protein LivH
bin008	SOY3_bin008_01303	924	8	47	36	1.035	5.159	4.139	High-affinity branched-chain amino acid transport system permease protein LivH
bin008	SOY3_bin008_01304	1167	137	805	913	14.034	69.965	83.105	Leucine-, isoleucine-, valine-, threonine-, and alanine-binding protein precursor
bin008	SOY3_bin008_01305	1155	23	92	101	2.381	8.079	9.289	Alanine dehydrogenase 2
bin008	SOY3_bin008_01306	858	6	29	24	0.836	3.428	2.971	Formyltetrahydrofolate deformylase
bin008	SOY3_bin008_01307	789	11	40	47	1.667	5.142	6.328	TPR repeat-containing protein YrrB
bin008	SOY3_bin008_01308	279	199	813	951	85.270	295.557	362.081	RNA recognition motif. (a.k.a. RRM, RBD, or RNP domain)
bin008	SOY3_bin008_01309	1083	3	20	16	0.331	1.873	1.569	putative peptidase
bin008	SOY3_bin008_01310	312	0	6	3	0.000	1.951	1.021	hypothetical protein
bin008	SOY3_bin008_01311	729	89	153	167	14.595	21.287	24.334	Signal transduction histidine-protein kinase BarA
bin008	SOY3_bin008_01312	390	43	85	107	13.181	22.106	29.144	CAI-1 autoinducer sensor kinase/phosphatase CqsS
bin008	SOY3_bin008_01313	642	52	96	128	9.683	15.167	21.179	hypothetical protein
bin008	SOY3_bin008_01314	825	10	20	24	1.449	2.459	3.090	putative deoxyribonuclease YcfH
bin008	SOY3_bin008_01315	723	8	24	20	1.323	3.367	2.938	hypothetical protein
bin008	SOY3_bin008_01316	462	8	10	10	2.070	2.195	2.299	putative voltage-gated ClC-type chloride channel ClcB
bin008	SOY3_bin008_01317	711	14	17	24	2.354	2.425	3.586	hypothetical protein
bin008	SOY3_bin008_01318	315	2	4	7	0.759	1.288	2.361	hypothetical protein
bin008	SOY3_bin008_01319	972	4	12	15	0.492	1.252	1.639	Calcineurin-like phosphoesterase superfamily domain protein
bin008	SOY3_bin008_01320	1359	5	17	18	0.440	1.269	1.407	DNA replication and repair protein RecF
bin008	SOY3_bin008_01321	702	3	12	22	0.511	1.734	3.329	Amidophosphoribosyltransferase precursor
bin008	SOY3_bin008_01322	615	6	27	33	1.166	4.453	5.700	Iron-sulfur flavoprotein
bin008	SOY3_bin008_01323	627	6	19	19	1.144	3.074	3.219	putative metallo-hydrolase
bin008	SOY3_bin008_01324	2409	9	93	98	0.447	3.916	4.321	TPR repeat-containing protein YrrB
bin008	SOY3_bin008_01325	405	2	31	36	0.590	7.764	9.442	hypothetical protein
bin008	SOY3_bin008_01326	627	0	76	79	0.000	12.294	13.384	Demethylrebeccamycin-D-glucose O-methyltransferase
bin008	SOY3_bin008_01327	579	6	134	138	1.239	23.474	25.318	FmdE, Molybdenum formylmethanofuran dehydrogenase operon
bin008	SOY3_bin008_01328	918	6	120	142	0.781	13.258	16.431	hypothetical protein
bin008	SOY3_bin008_01329	1077	27	424	673	2.997	39.931	66.379	Sorbitol dehydrogenase
bin008	SOY3_bin008_01330	2079	113	227	265	6.498	11.075	13.540	Acetoin dehydrogenase operon transcriptional activator AcoR
bin008	SOY3_bin008_01331	978	17	24	30	2.078	2.489	3.258	Lipoate-protein ligase LplJ
bin008	SOY3_bin008_01332	453	13	42	51	3.431	9.404	11.959	hypothetical protein
bin008	SOY3_bin008_01333	660	9	23	28	1.630	3.535	4.507	Fumarate reductase cytochrome b subunit
bin008	SOY3_bin008_01334	1824	33	119	129	2.163	6.617	7.513	Fumarate reductase flavoprotein subunit
bin008	SOY3_bin008_01335	756	14	31	30	2.214	4.159	4.215	Fumarate reductase iron-sulfur subunit
bin008	SOY3_bin008_01336	840	9	30	48	1.281	3.622	6.070	L(+)-tartrate dehydratase subunit alpha
bin008	SOY3_bin008_01337	543	42	74	110	9.247	13.823	21.519	Fumarate hydratase class I, anaerobic
bin008	SOY3_bin008_01338	855	33	98	81	4.614	11.626	10.063	Dihydroorotate dehydrogenase B (NAD(+)), electron transfer subunit
bin008	SOY3_bin008_01339	1476	66	157	179	5.346	10.789	12.882	Glutamate synthase [NADPH] small chain
bin008	SOY3_bin008_01340	1320	54	168	229	4.891	12.909	18.429	NADP-dependent malic enzyme
bin008	SOY3_bin008_01341	567	748	1647	2376	157.712	294.622	445.136	lipoprotein NlpI
bin008	SOY3_bin008_01342	693	43	95	73	7.418	13.904	11.190	Transcriptional regulatory protein DegU
bin008	SOY3_bin008_01343	1146	67	204	195	6.989	18.055	18.075	Sensor histidine kinase ComP
bin008	SOY3_bin008_01344	531	187	525	698	42.101	100.281	139.634	hypothetical protein
bin008	SOY3_bin008_01345	180	13	27	51	8.634	15.214	30.097	hypothetical protein
bin008	SOY3_bin008_01346	6072	263	497	578	5.178	8.302	10.112	Autoinducer 2 sensor kinase/phosphatase LuxQ
bin008	SOY3_bin008_01347	1131	34	102	101	3.594	9.147	9.486	putative xanthine dehydrogenase subunit A
bin008	SOY3_bin008_01348	825	3	12	22	0.435	1.475	2.833	hypothetical protein
bin008	SOY3_bin008_01349	207	276	794	1133	159.399	389.050	581.419	Molybdenum-pterin-binding protein 2
bin008	SOY3_bin008_01350	432	4	26	22	1.107	6.104	5.410	Transcriptional regulator ModE
bin008	SOY3_bin008_01351	894	20	51	62	2.674	5.786	7.367	2-hydroxymuconate semialdehyde hydrolase
bin008	SOY3_bin008_01352	180	12	12	16	7.970	6.762	9.442	hypothetical protein
bin008	SOY3_bin008_01353	174	5	37	38	3.435	21.568	23.199	Flp/Fap pilin component
bin008	SOY3_bin008_01354	1701	23	251	257	1.616	14.967	16.049	putative bifunctional SAT/APS kinase
bin008	SOY3_bin008_01355	1236	41	142	143	3.966	11.653	12.290	phosphodiesterase
bin008	SOY3_bin008_01356	498	8	20	20	1.920	4.073	4.266	Type 4 prepilin-like proteins leader peptide-processing enzyme
bin008	SOY3_bin008_01357	450	2	9	21	0.531	2.029	4.957	hypothetical protein
bin008	SOY3_bin008_01358	474	19	47	40	4.792	10.057	8.964	cAMP-activated global transcriptional regulator CRP
bin008	SOY3_bin008_01359	246	60	179	251	29.158	73.803	108.385	hypothetical protein
bin008	SOY3_bin008_01360	359	59587	66136	85374	19842.784	18685.221	25261.609	transfer-messenger RNA, SsrA
bin008	SOY3_bin008_01361	1185	7	48	57	0.706	4.108	5.110	Putative prophage CPS-53 integrase
bin008	SOY3_bin008_01362	672	5	22	44	0.890	3.321	6.955	HTH-type transcriptional regulator PrtR
bin008	SOY3_bin008_01363	699	2	14	13	0.342	2.031	1.976	Bacteriophage CI repressor helix-turn-helix domain protein
bin008	SOY3_bin008_01364	2340	7	18	28	0.358	0.780	1.271	Carbamoyltransferase HypF

bin008	SOY3_bin008_01365	468	9	19	30	2.299	4.118	6.809	hypothetical protein
bin008	SOY3_bin008_01366	804	9	21	31	1.338	2.649	4.096	Malonyl-[acyl-carrier protein] O-methyltransferase
bin008	SOY3_bin008_01367	612	4	7	10	0.781	1.160	1.736	Alpha/beta hydrolase family protein
bin008	SOY3_bin008_01368	1173	104	218	295	10.599	18.850	26.715	8-amino-7-oxononanoate synthase
bin008	SOY3_bin008_01369	1344	0	14	16	0.000	1.057	1.265	Adenosylmethionine-8-amino-7-oxononanoate aminotransferase
bin008	SOY3_bin008_01370	612	2	6	12	0.391	0.994	2.083	ATP-dependent dethiobiotin synthetase BioD
bin008	SOY3_bin008_01371	339	2	51	53	0.705	15.259	16.608	hypothetical protein
bin008	SOY3_bin008_01372	2871	0	33	29	0.000	1.166	1.073	WD domain, G-beta repeat
bin008	SOY3_bin008_01373	453	3	20	22	0.792	4.478	5.159	hypothetical protein
bin008	SOY3_bin008_01374	237	1	10	5	0.504	4.280	2.241	hypothetical protein
bin008	SOY3_bin008_01375	579	8	19	12	1.652	3.328	2.202	putative aromatic acid decarboxylase
bin008	SOY3_bin008_01376	495	3	11	9	0.725	2.254	1.931	hypothetical protein
bin008	SOY3_bin008_01377	588	4	19	13	0.813	3.277	2.349	DNA-3-methyladenine glycosylase 1
bin008	SOY3_bin008_01378	1446	17	74	67	1.405	5.191	4.922	3-octaprenyl-4-hydroxybenzoate carboxy-lyase
bin008	SOY3_bin008_01379	879	5	15	21	0.680	1.731	2.538	hypothetical protein
bin008	SOY3_bin008_01380	420	7	19	20	1.992	4.588	5.058	IncA protein
bin008	SOY3_bin008_01381	315	26	94	96	9.868	30.267	32.374	hypothetical protein
bin008	SOY3_bin008_01382	702	23	66	61	3.917	9.536	9.230	flagellar basal body-associated protein FliL
bin008	SOY3_bin008_01383	384	51	151	182	15.878	39.884	50.347	Chemotaxis protein CheY
bin008	SOY3_bin008_01384	678	34	112	156	5.995	16.755	24.441	RNA polymerase sigma factor FliA
bin008	SOY3_bin008_01385	867	45	164	154	6.205	19.186	18.868	Flagellum site-determining protein YlxH
bin008	SOY3_bin008_01386	1122	9	35	60	0.959	3.164	5.681	Flagellar biosynthesis protein FlhF
bin008	SOY3_bin008_01387	2112	17	68	84	0.962	3.266	4.225	Flagellar biosynthesis protein FlhA
bin008	SOY3_bin008_01388	1074	2	30	23	0.223	2.833	2.275	Flagellar biosynthetic protein FlhB
bin008	SOY3_bin008_01389	783	4	10	9	0.611	1.295	1.221	Flagellar biosynthetic protein FliR
bin008	SOY3_bin008_01390	897	7	27	27	0.933	3.053	3.197	Murein DD-endopeptidase MepM
bin008	SOY3_bin008_01391	768	6	26	30	0.934	3.434	4.149	tRNA 2-thiocytidine biosynthesis protein TtcA
bin008	SOY3_bin008_01392	855	11	39	37	1.538	4.627	4.597	HDOD domain protein
bin008	SOY3_bin008_01393	477	29	74	92	7.268	15.735	20.488	Chemoreceptor glutamine deamidase CheD
bin008	SOY3_bin008_01394	1128	18	31	37	1.908	2.787	3.484	Sporulation kinase E
bin008	SOY3_bin008_01395	639	3	10	10	0.561	1.587	1.662	Inner membrane protein YohD
bin008	SOY3_bin008_01396	1797	221	332	522	14.702	18.739	30.857	Methyl-accepting chemotaxis protein 4
bin008	SOY3_bin008_01397	2145	152	285	398	8.472	13.476	19.710	Chemotaxis protein CheA
bin008	SOY3_bin008_01398	513	41	103	122	9.555	20.365	25.262	Chemotaxis protein CheW
bin008	SOY3_bin008_01399	861	58	126	177	8.053	14.843	21.837	Chemotaxis protein methyltransferase
bin008	SOY3_bin008_01400	1074	93	175	215	10.352	16.527	21.265	Chemotaxis response regulator protein-glutamate methylesterase
bin008	SOY3_bin008_01401	603	12	18	30	2.379	3.028	5.285	CYTH domain protein
bin008	SOY3_bin008_01402	282	14	59	75	5.935	21.221	28.252	ATP-dependent Clp protease adapter protein ClpS
bin008	SOY3_bin008_01403	2292	108	253	334	5.633	11.196	15.480	ATP-dependent Clp protease ATP-binding subunit ClpA
bin008	SOY3_bin008_01404	687	4	12	8	0.696	1.772	1.237	Leucyl/phenylalanyl-tRNA--protein transferase
bin008	SOY3_bin008_01405	180	1	3	5	0.664	1.690	2.951	hypothetical protein
bin008	SOY3_bin008_01406	246	2	6	4	0.972	2.474	1.727	hypothetical protein
bin008	SOY3_bin008_01407	1530	9	41	30	0.703	2.718	2.083	2,3-bisphosphoglycerate-independent phosphoglycerate mutase
bin008	SOY3_bin008_01408	399	5	18	26	1.498	4.576	6.922	Ribosomal silencing factor RsfS
bin008	SOY3_bin008_01409	1302	25	169	191	2.295	13.165	15.583	Phenylacetate-coenzyme A ligase
bin008	SOY3_bin008_01410	432	14	99	124	3.874	23.244	30.491	acetolactate synthase 3 regulatory subunit
bin008	SOY3_bin008_01411	1437	57	96	146	4.742	6.776	10.793	RNA-splicing ligase RtcB
bin008	SOY3_bin008_01412	1896	71	109	155	4.477	5.831	8.684	Sensory/regulatory protein RpfC
bin008	SOY3_bin008_01413	789	59	200	234	8.940	25.710	31.504	4-hydroxy-tetrahydrodipicolinate reductase
bin008	SOY3_bin008_01414	549	4	36	34	0.871	6.651	6.579	Peroxyureidoacrylate/ureidoacrylate amidohydrolase RutB
bin008	SOY3_bin008_01415	1098	5	60	63	0.544	5.542	6.095	Nicotinate phosphoribosyltransferase pncB2
bin008	SOY3_bin008_01416	1551	18	63	57	1.387	4.120	3.904	hypothetical protein
bin008	SOY3_bin008_01417	1227	1	2	4	0.097	0.165	0.346	Ammonium transporter NrgA
bin008	SOY3_bin008_01418	339	1	2	5	0.353	0.598	1.567	Nitrogen regulatory protein P-II
bin008	SOY3_bin008_01419	492	94	231	288	22.841	47.621	62.181	hypothetical protein
bin008	SOY3_bin008_01420	465	332	528	773	85.355	115.169	176.586	hypothetical protein
bin008	SOY3_bin008_01421	345	7	7	6	2.426	2.058	1.847	DsrE/DsrF-like family protein
bin008	SOY3_bin008_01422	1653	17	58	75	1.229	3.559	4.820	Phosphoglucomutase
bin008	SOY3_bin008_01423	282	5	17	12	2.120	6.114	4.520	hypothetical protein
bin008	SOY3_bin008_01424	3894	51	108	165	1.566	2.813	4.501	Adenylate cyclase
bin008	SOY3_bin008_01425	1287	27	87	93	2.508	6.856	7.676	Cyclic di-GMP phosphodiesterase response regulator RpfG
bin008	SOY3_bin008_01426	843	17	39	41	2.411	4.692	5.166	Tetratricopeptide repeat protein
bin008	SOY3_bin008_01427	1791	46	64	100	3.070	3.624	5.931	Methyl-accepting chemotaxis protein McpS
bin008	SOY3_bin008_01428	1350	22	31	46	1.948	2.329	3.620	Na(+)/H(+) antiporter NhaA
bin008	SOY3_bin008_01429	978	30	52	66	3.667	5.393	7.169	2-deoxy-scyllo-inosamine dehydrogenase
bin008	SOY3_bin008_01430	1683	42	78	111	2.983	4.701	7.006	Ribosomal large subunit pseudouridine synthase D
bin008	SOY3_bin008_01431	726	6	10	24	0.988	1.397	3.512	Rhomboid protease GluP

bin008	SOY3_bin008_01432	585	12	13	15	2.452	2.254	2.724	LOG family protein YvdD
bin008	SOY3_bin008_01433	486	12	32	37	2.952	6.678	8.087	hypothetical protein
bin008	SOY3_bin008_01434	2040	7	20	20	0.410	0.994	1.041	UvrABC system protein B
bin008	SOY3_bin008_01435	1047	1	11	21	0.114	1.066	2.131	Voltage-gated potassium channel Kch
bin008	SOY3_bin008_01436	666	0	1	1	0.000	0.152	0.159	hypothetical protein
bin008	SOY3_bin008_01437	1215	16	57	59	1.574	4.758	5.158	glucans biosynthesis protein
bin008	SOY3_bin008_01438	228	17	38	62	8.914	16.905	28.886	Molybdopterin synthase sulfur carrier subunit
bin008	SOY3_bin008_01439	843	74	169	165	10.494	20.334	20.792	putative amino-acid-binding protein YxeM precursor
bin008	SOY3_bin008_01440	2652	158	216	268	7.122	8.261	10.735	Autoinducer 2 sensor kinase/phosphatase LuxQ
bin008	SOY3_bin008_01441	1284	24	148	167	2.235	11.691	13.816	hypothetical protein
bin008	SOY3_bin008_01442	750	69	260	286	10.998	35.161	40.507	FecR protein
bin008	SOY3_bin008_01443	765	9	68	84	1.406	9.016	11.664	Aggregation substance precursor
bin008	SOY3_bin008_01444	1035	6	16	19	0.693	1.568	1.950	Putative GTP cyclohydrolase 1 type 2
bin008	SOY3_bin008_01445	76	3	2	6	4.719	2.669	8.386	tRNA-Met(cat)
bin008	SOY3_bin008_01446	915	10	61	77	1.307	6.762	8.939	Thioredoxin reductase
bin008	SOY3_bin008_01447	2466	11	36	37	0.533	1.481	1.594	Sensory/regulatory protein RpfC
bin008	SOY3_bin008_01448	1113	6	15	35	0.644	1.367	3.340	hypothetical protein
bin008	SOY3_bin008_01449	489	9	33	66	2.200	6.845	14.337	hypothetical protein
bin008	SOY3_bin008_01450	504	18	66	74	4.270	13.282	15.597	hypothetical protein
bin008	SOY3_bin008_01451	369	2	29	28	0.648	7.971	8.060	DsrE/DsrF-like family protein
bin008	SOY3_bin008_01452	534	1	7	4	0.224	1.330	0.796	hypothetical protein
bin008	SOY3_bin008_01453	480	1	13	23	0.249	2.747	5.090	phosphoglycolate phosphatase
bin008	SOY3_bin008_01454	900	85	679	768	11.291	76.521	90.646	Flagellar filament 33 kDa core protein
bin008	SOY3_bin008_01455	843	966	2331	3121	136.992	280.459	393.275	PBP superfamily domain protein
bin008	SOY3_bin008_01456	696	103	186	225	17.692	27.106	34.340	Sulfate transport system permease protein CysW
bin008	SOY3_bin008_01457	717	95	249	303	15.840	35.224	44.890	Spermidine/putrescine import ATP-binding protein PotA
bin008	SOY3_bin008_01458	705	57	149	175	9.666	21.436	26.368	ferredoxin
bin008	SOY3_bin008_01459	1449	75	256	285	6.188	17.920	20.893	Sensor protein KdpD
bin008	SOY3_bin008_01460	339	11	70	84	3.879	20.944	26.321	Nitrogen regulatory protein P-II 1
bin008	SOY3_bin008_01461	1590	16	68	71	1.203	4.338	4.743	Ammonia channel precursor
bin008	SOY3_bin008_01462	1017	12	32	19	1.411	3.191	1.985	hypothetical protein
bin008	SOY3_bin008_01463	420	2	11	11	0.569	2.656	2.782	Coenzyme PQQ synthesis protein D
bin008	SOY3_bin008_01464	717	1	6	10	0.167	0.849	1.482	Sulfoxide reductase catalytic subunit YedY precursor
bin008	SOY3_bin008_01465	297	15	46	43	6.038	15.709	15.379	hypothetical protein
bin008	SOY3_bin008_01466	825	22	59	70	3.188	7.254	9.013	Purine nucleoside phosphorylase 1
bin008	SOY3_bin008_01467	759	3	21	26	0.473	2.806	3.639	Chemotaxis protein PomA
bin008	SOY3_bin008_01468	738	11	30	62	1.782	4.123	8.924	Motility protein B
bin008	SOY3_bin008_01469	429	33	85	131	9.196	20.096	32.437	Flagellar brake protein YcgR
bin008	SOY3_bin008_01470	1419	50	73	85	4.212	5.218	6.363	2-oxoglutarate carboxylase small subunit
bin008	SOY3_bin008_01471	2253	47	126	136	2.494	5.672	6.412	Acetyl-coenzyme A carboxylase carboxyl transferase subunit beta
bin008	SOY3_bin008_01472	651	25	54	79	4.591	8.413	12.891	acetyl-CoA carboxylase biotin carboxyl carrier protein subunit
bin008	SOY3_bin008_01473	348	16	62	71	5.497	18.070	21.672	hypothetical protein
bin008	SOY3_bin008_01474	537	77	183	211	17.142	34.565	41.739	Single-stranded DNA-binding protein
bin008	SOY3_bin008_01475	1893	78	173	203	4.926	9.269	11.391	putative diguanylate cyclase AdrA
bin008	SOY3_bin008_01476	2415	6930	6846	9709	343.053	287.524	427.058	Benzylsuccinate synthase alpha subunit
bin008	SOY3_bin008_01477	969	279	225	315	34.421	23.551	34.532	4-hydroxyphenylacetate decarboxylase activating enzyme
bin008	SOY3_bin008_01478	660	63	101	131	11.411	15.521	21.084	Transcriptional regulatory protein DegU
bin008	SOY3_bin008_01479	1671	155	134	187	11.089	8.134	11.888	Sensor protein kinase WalK
bin008	SOY3_bin008_01480	1149	68	79	137	7.075	6.974	12.666	hypothetical protein
bin008	SOY3_bin008_01481	402	13	25	30	3.866	6.308	7.927	TadE-like protein
bin008	SOY3_bin008_01482	429	19	19	35	5.295	4.492	8.666	TadE-like protein
bin008	SOY3_bin008_01483	657	8	21	26	1.456	3.242	4.204	Oxygen regulatory protein NreC
bin008	SOY3_bin008_01484	2118	37	60	100	2.088	2.873	5.015	Signal transduction histidine-protein kinase/phosphatase DegS
bin008	SOY3_bin008_01485	1944	56	142	143	3.444	7.409	7.814	Acetoin dehydrogenase operon transcriptional activator AcoR
bin008	SOY3_bin008_01486	567	0	1	3	0.000	0.179	0.562	hypothetical protein
bin008	SOY3_bin008_01487	1929	80	35	58	4.958	1.840	3.194	NADH oxidase
bin008	SOY3_bin008_01488	525	18	43	57	4.099	8.307	11.533	Aryl-alcohol dehydrogenase
bin008	SOY3_bin008_01489	645	24	79	107	4.448	12.423	17.622	Bifunctional polymyxin resistance protein ArnA
bin008	SOY3_bin008_01490	318	28	174	213	10.526	55.498	71.151	hypothetical protein
bin008	SOY3_bin008_01491	2679	10	6	4	0.446	0.227	0.159	Aldehyde oxidoreductase
bin008	SOY3_bin008_01492	2253	4	3	11	0.212	0.135	0.519	NAD-dependent dihydropyrimidine dehydrogenase subunit PreT
bin008	SOY3_bin008_01493	216	0	2	0	0.000	0.939	0.000	hypothetical protein
bin008	SOY3_bin008_01494	246	1	2	3	0.486	0.825	1.295	DNA replication protein DnaC
bin008	SOY3_bin008_01495	276	2	5	9	0.866	1.837	3.464	Formate dehydrogenase, nitrate-inducible, iron-sulfur subunit
bin008	SOY3_bin008_01496	399	9	14	24	2.697	3.559	6.390	putative oxidoreductase YdhV
bin008	SOY3_bin008_01497	1776	24	69	70	1.616	3.941	4.187	putative oxidoreductase YdhV
bin008	SOY3_bin008_01498	1467	0	12	10	0.000	0.830	0.724	Oxygen-independent coproporphyrinogen-III oxidase 1

bin008	SOY3_bin008_01499	1314	25	90	116	2.275	6.947	9.378	hypothetical protein
bin008	SOY3_bin008_01500	1110	68	52	77	7.324	4.752	7.369	Histidinol-phosphate aminotransferase 2
bin008	SOY3_bin008_01501	339	79	37	57	27.859	11.070	17.861	hypothetical protein
bin008	SOY3_bin008_01502	2271	898	395	660	47.272	17.641	30.871	Serine protease AprX
bin008	SOY3_bin008_01503	1050	17	36	39	1.936	3.478	3.946	hypothetical protein
bin008	SOY3_bin008_01504	804	6	37	29	0.892	4.668	3.832	Sulfur carrier protein ThiS adenylyltransferase
bin008	SOY3_bin008_01505	1473	11	52	51	0.893	3.581	3.678	hypothetical protein
bin008	SOY3_bin008_01506	474	2	3	3	0.504	0.642	0.672	hypothetical protein
bin008	SOY3_bin008_01507	126	1	0	0	0.949	0.000	0.000	hypothetical protein
bin008	SOY3_bin008_01508	426	19	64	70	5.332	15.238	17.455	hypothetical protein
bin008	SOY3_bin008_01509	399	28	63	72	8.389	16.015	19.169	site-specific tyrosine recombinase XerC
bin008	SOY3_bin008_01510	76	0	2	3	0.000	2.669	4.193	tRNA-Glu(ctc)
bin008	SOY3_bin008_01511	76	6	12	12	9.438	16.015	16.772	tRNA-Lys(ttt)
bin008	SOY3_bin008_01512	483	3	6	14	0.743	1.260	3.079	HTH-type transcriptional regulator ZntR
bin008	SOY3_bin008_01513	2253	43	38	68	2.282	1.711	3.206	putative cadmium-transporting ATPase
bin008	SOY3_bin008_01514	675	102	129	171	18.065	19.384	26.911	Methyltransferase domain protein
bin008	SOY3_bin008_01515	1461	80	81	139	6.546	5.623	10.106	Sensor protein ZraS
bin008	SOY3_bin008_01516	1998	70	89	120	4.188	4.518	6.380	Sporulation kinase E
bin008	SOY3_bin008_01517	1107	26	26	64	2.808	2.382	6.141	Cyclic di-GMP phosphodiesterase response regulator RpfG
bin008	SOY3_bin008_01518	390	22	18	36	6.744	4.681	9.805	C4-dicarboxylate transport transcriptional regulatory protein DctD
bin008	SOY3_bin008_01519	2274	68	80	133	3.575	3.568	6.213	Sensor protein FixL
bin008	SOY3_bin008_01520	2496	9	33	42	0.431	1.341	1.787	Transcriptional regulatory protein ZraR
bin008	SOY3_bin008_01521	411	123	178	258	35.777	43.927	66.682	Putative universal stress protein
bin008	SOY3_bin008_01522	297	77	132	171	30.994	45.079	61.160	hypothetical protein
bin008	SOY3_bin008_01523	1035	240	310	515	27.721	30.379	52.856	hypothetical protein
bin008	SOY3_bin008_01524	339	105	148	257	37.028	44.281	80.531	hypothetical protein
bin008	SOY3_bin008_01525	255	11	25	24	5.157	9.944	9.998	hypothetical protein
bin008	SOY3_bin008_01526	885	41	52	68	5.538	5.960	8.162	hypothetical protein
bin008	SOY3_bin008_01527	444	16	32	42	4.308	7.310	10.048	Peptidyl-tRNA hydrolase ArfB
bin008	SOY3_bin008_01528	201	2	3	0	1.190	1.514	0.000	hypothetical protein
bin008	SOY3_bin008_01529	756	5	10	11	0.791	1.342	1.546	Pyruvate dehydrogenase complex repressor
bin008	SOY3_bin008_01530	1665	22	9	15	1.580	0.548	0.957	Oxygen-dependent choline dehydrogenase
bin008	SOY3_bin008_01531	1476	16	17	22	1.296	1.168	1.583	Betaine aldehyde dehydrogenase
bin008	SOY3_bin008_01532	963	33	23	34	4.097	2.422	3.750	Glycine betaine-binding protein OpuAC precursor
bin008	SOY3_bin008_01533	756	15	31	33	2.372	4.159	4.637	hypothetical protein
bin008	SOY3_bin008_01534	999	4	11	18	0.479	1.117	1.914	Alpha/beta hydrolase family protein
bin008	SOY3_bin008_01535	87	0	2	2	0.000	2.332	2.442	tRNA-Leu(caa)
bin008	SOY3_bin008_01536	627	62	221	270	11.821	35.750	45.743	N-acetylmuramoyl-L-alanine amidase XlyA precursor
bin008	SOY3_bin008_01537	1215	47	109	156	4.625	9.099	13.639	Cyclic di-GMP phosphodiesterase response regulator RpfG
bin008	SOY3_bin008_01538	576	28	64	94	5.811	11.270	17.335	hypothetical protein
bin008	SOY3_bin008_01539	681	53	69	108	9.304	10.277	16.846	6-phosphogluconolactonase
bin008	SOY3_bin008_01540	1533	190	212	295	14.817	14.026	20.441	Glucose-6-phosphate 1-dehydrogenase
bin008	SOY3_bin008_01541	930	206	265	418	26.481	28.901	47.744	6-phosphogluconate dehydrogenase, decarboxylating
bin008	SOY3_bin008_01542	720	43	74	87	7.140	10.424	12.836	TVP38/TMEM64 family inner membrane protein YdjZ
bin008	SOY3_bin008_01543	1362	73	132	186	6.408	9.830	14.507	Deoxyribodipyrimidine photo-lyase
bin008	SOY3_bin008_01544	789	3	21	27	0.455	2.700	3.635	Nucleoside triphosphate pyrophosphohydrolase
bin008	SOY3_bin008_01545	816	3	27	31	0.440	3.356	4.036	Colicin V production protein
bin008	SOY3_bin008_01546	1122	26	108	88	2.770	9.763	8.331	ATP-dependent Clp protease ATP-binding subunit ClpX
bin008	SOY3_bin008_01547	1125	8	39	52	0.850	3.516	4.910	Riboflavin biosynthesis protein RibD
bin008	SOY3_bin008_01548	462	3	7	14	0.776	1.537	3.219	Riboflavin biosynthesis protein RibD
bin008	SOY3_bin008_01549	750	1	8	4	0.159	1.082	0.567	C-methyltransferase CouO
bin008	SOY3_bin008_01550	1239	21	176	190	2.026	14.408	16.290	Serine hydroxymethyltransferase
bin008	SOY3_bin008_01551	1245	13	176	166	1.248	14.338	14.163	3-oxoacyl-[acyl-carrier-protein] synthase 2
bin008	SOY3_bin008_01552	237	5	123	96	2.522	52.639	43.028	Acyl carrier protein
bin008	SOY3_bin008_01553	729	4	170	191	0.656	23.652	27.831	3-oxoacyl-[acyl-carrier-protein] reductase FabG
bin008	SOY3_bin008_01554	993	11	61	63	1.324	6.231	6.739	3-oxoacyl-[acyl-carrier-protein] synthase 3
bin008	SOY3_bin008_01555	1050	33	168	175	3.757	16.228	17.704	Phosphate acyltransferase
bin008	SOY3_bin008_01556	183	43	219	210	28.091	121.380	121.898	50S ribosomal protein L32
bin008	SOY3_bin008_01557	543	30	129	130	6.605	24.096	25.432	hypothetical protein
bin008	SOY3_bin008_01558	207	10	125	106	5.775	61.248	54.396	50S ribosomal protein L28
bin008	SOY3_bin008_01559	1338	5	23	20	0.447	1.744	1.588	Magnesium and cobalt efflux protein CorC
bin008	SOY3_bin008_01560	426	42	133	151	11.787	31.666	37.653	putative HTH-type transcriptional regulator YusO
bin008	SOY3_bin008_01561	408	103	229	251	30.180	56.929	65.350	Pyridoxamine 5'-phosphate oxidase
bin008	SOY3_bin008_01562	759	0	4	6	0.000	0.535	0.840	hypothetical protein
bin008	SOY3_bin008_01563	1395	3	62	61	0.257	4.508	4.645	Glutamate--tRNA ligase
bin008	SOY3_bin008_01564	222	5	66	60	2.693	30.154	28.710	Fe/S biogenesis protein NfuA
bin008	SOY3_bin008_01565	1338	47	137	150	4.199	10.385	11.909	Agglutinin receptor precursor

bin008	SOY3_bin008_01566	2037	78	189	200	4.578	9.411	10.430	Autoinducer 2 sensor kinase/phosphatase LuxQ
bin008	SOY3_bin008_01567	345	77	185	253	26.682	54.389	77.899	Ferredoxin thioredoxin reductase catalytic beta chain
bin008	SOY3_bin008_01568	264	55	161	202	24.906	61.855	81.279	Glutaredoxin-like protein NrdH
bin008	SOY3_bin008_01569	762	6	13	23	0.941	1.730	3.206	putative glutamine ABC transporter permease protein GlnM
bin008	SOY3_bin008_01570	387	3	12	23	0.927	3.145	6.313	hypothetical protein
bin008	SOY3_bin008_01571	813	18	91	105	2.647	11.353	13.719	Cystine-binding periplasmic protein precursor
bin008	SOY3_bin008_01572	435	0	5	11	0.000	1.166	2.686	hypothetical protein
bin008	SOY3_bin008_01573	645	0	7	7	0.000	1.101	1.153	Thymidylate kinase
bin008	SOY3_bin008_01574	1110	7	37	32	0.754	3.381	3.062	3'-5' exoribonuclease YhaM
bin008	SOY3_bin008_01575	783	9	18	35	1.374	2.332	4.748	5'-nucleotidase SurE
bin008	SOY3_bin008_01576	1020	57	135	159	6.681	13.424	16.559	Response regulator PleD
bin008	SOY3_bin008_01577	474	32	69	77	8.071	14.765	17.256	hypothetical protein
bin008	SOY3_bin008_01578	867	13	70	93	1.793	8.189	11.394	Motility protein A
bin008	SOY3_bin008_01579	987	29	162	208	3.513	16.648	22.386	Motility protein B
bin008	SOY3_bin008_01580	279	11	174	191	4.713	63.256	72.721	50S ribosomal protein L27
bin008	SOY3_bin008_01581	309	16	193	208	6.190	63.351	71.505	50S ribosomal protein L21
bin008	SOY3_bin008_01582	1524	42	80	79	3.295	5.324	5.506	hypothetical protein
bin008	SOY3_bin008_01583	444	9	12	18	2.423	2.741	4.306	hypothetical protein
bin008	SOY3_bin008_01584	810	5	15	13	0.738	1.878	1.705	Glutamate racemase
bin008	SOY3_bin008_01585	573	1	21	19	0.209	3.717	3.522	tetratricopeptide repeat protein
bin008	SOY3_bin008_01586	585	1	11	10	0.204	1.907	1.816	2'-5'-RNA ligase
bin008	SOY3_bin008_01587	486	37	200	255	9.101	41.740	55.736	hypothetical protein
bin008	SOY3_bin008_01588	453	41	212	249	10.820	47.467	58.389	Putative universal stress protein
bin008	SOY3_bin008_01589	1110	17	102	126	1.831	9.320	12.058	Histidinol-phosphate aminotransferase 2
bin008	SOY3_bin008_01590	681	9	74	105	1.580	11.021	16.378	Cytidylate kinase
bin008	SOY3_bin008_01591	489	3	65	71	0.733	13.482	15.423	Transcription elongation factor GreA
bin008	SOY3_bin008_01592	462	4	12	11	1.035	2.634	2.529	Nucleoside 2-deoxyribosyltransferase
bin008	SOY3_bin008_01593	264	92	170	217	41.661	65.313	87.314	hypothetical protein
bin008	SOY3_bin008_01594	2202	56	91	103	3.040	4.192	4.969	GTP pyrophosphokinase
bin008	SOY3_bin008_01595	1665	7	34	42	0.503	2.071	2.680	Oligopeptide-binding protein AppA precursor
bin008	SOY3_bin008_01596	3216	30	155	167	1.115	4.888	5.516	RNA polymerase-associated protein RapA
bin008	SOY3_bin008_01597	1416	7	24	56	0.591	1.719	4.201	Zeaxanthin glucosyltransferase
bin008	SOY3_bin008_01598	2148	36	59	83	2.004	2.786	4.105	Phosphoserine phosphatase RsbU
bin008	SOY3_bin008_01599	645	4	71	54	0.741	11.165	8.893	3,4-dihydroxy-2-butanone 4-phosphate synthase
bin008	SOY3_bin008_01600	624	227	176	272	43.490	28.608	46.304	zinc resistance protein
bin008	SOY3_bin008_01601	489	1316	1384	2268	321.731	287.066	492.679	Zinc resistance-associated protein precursor
bin008	SOY3_bin008_01602	399	29	68	30	8.689	17.286	7.987	putative HTH-type transcriptional regulator YtcD
bin008	SOY3_bin008_01603	516	10	23	24	2.317	4.521	4.941	Albonoursin synthase
bin008	SOY3_bin008_01604	585	5	19	24	1.022	3.294	4.358	Flavoredoxin
bin008	SOY3_bin008_01605	567	13	43	50	2.741	7.692	9.367	Flavoredoxin
bin008	SOY3_bin008_01606	573	15	30	37	3.130	5.310	6.859	Flavoredoxin
bin008	SOY3_bin008_01607	576	20	49	71	4.151	8.628	13.094	2-amino-4-deoxychorismate dehydrogenase
bin008	SOY3_bin008_01608	1251	32	58	62	3.058	4.702	5.265	Response regulator PleD
bin008	SOY3_bin008_01609	774	20	44	63	3.089	5.766	8.646	HTH-type transcriptional repressor of iron proteins A
bin008	SOY3_bin008_01610	1062	28	42	56	3.152	4.011	5.601	Sodium Bile acid symporter family protein
bin008	SOY3_bin008_01611	1803	6	28	38	0.398	1.575	2.239	hypothetical protein
bin008	SOY3_bin008_01612	729	7	18	13	1.148	2.504	1.894	hypothetical protein
bin008	SOY3_bin008_01613	1158	14	19	22	1.445	1.664	2.018	hypothetical protein
bin008	SOY3_bin008_01614	1740	15	28	35	1.031	1.632	2.137	tol-pal system protein YbgF
bin008	SOY3_bin008_01615	990	2	17	16	0.242	1.742	1.717	von Willebrand factor type A domain protein
bin008	SOY3_bin008_01616	495	3	9	8	0.725	1.844	1.717	hypothetical protein
bin008	SOY3_bin008_01617	942	12	15	13	1.523	1.615	1.466	VWA domain containing CoxE-like protein
bin008	SOY3_bin008_01618	1080	0	6	9	0.000	0.563	0.885	Inner membrane protein YeeA
bin008	SOY3_bin008_01619	1272	26	91	110	2.444	7.256	9.186	Sensor protein SrrB
bin008	SOY3_bin008_01620	702	69	174	218	11.751	25.140	32.987	cellulose synthase subunit BcsC
bin008	SOY3_bin008_01621	399	12	25	27	3.595	6.355	7.188	Chlorophenol reductase precursor
bin008	SOY3_bin008_01622	1245	22	75	80	2.113	6.110	6.826	ATPase RavA
bin008	SOY3_bin008_01623	501	6	34	30	1.432	6.883	6.361	NADH oxidase
bin008	SOY3_bin008_01624	867	627	620	962	86.456	72.532	117.865	hypothetical protein
bin008	SOY3_bin008_01625	1200	2171	2706	4467	216.284	228.719	395.425	Long-chain primary alcohol dehydrogenase AdhA
bin008	SOY3_bin008_01626	507	5	18	8	1.179	3.601	1.676	hypothetical protein
bin008	SOY3_bin008_01627	447	3	11	7	0.802	2.496	1.663	hypothetical protein
bin008	SOY3_bin008_01628	1218	12	31	29	1.178	2.581	2.529	Alpha-ribazole phosphatase
bin008	SOY3_bin008_01629	1980	34	68	82	2.053	3.483	4.399	putative protease YhbU precursor
bin008	SOY3_bin008_01630	1263	10	46	56	0.947	3.694	4.710	Aspartate aminotransferase
bin008	SOY3_bin008_01631	612	1	18	15	0.195	2.983	2.604	Flagellin N-methylase
bin008	SOY3_bin008_01632	297	6	14	15	2.415	4.781	5.365	RNA recognition motif. (a.k.a. RRM, RBD, or RNP domain)

bin008	SOY3_bin008_01633	1479	4	88	105	0.323	6.035	7.541	Signal recognition particle protein
bin008	SOY3_bin008_01634	240	23	153	170	11.457	64.660	75.243	30S ribosomal protein S16
bin008	SOY3_bin008_01635	234	211	500	560	107.798	216.725	254.215	hypothetical protein
bin008	SOY3_bin008_01636	579	37	63	95	7.640	11.036	17.429	Ribosome maturation factor RimM
bin008	SOY3_bin008_01637	177	10	15	28	6.754	8.596	16.804	hypothetical protein
bin008	SOY3_bin008_01638	261	5	13	22	2.290	5.052	8.954	Sec-independent protein translocase protein TatA
bin008	SOY3_bin008_01639	2352	126	224	286	6.404	9.660	12.917	Ubiquinone biosynthesis O-methyltransferase
bin008	SOY3_bin008_01640	657	16	38	47	2.911	5.866	7.599	hypothetical protein
bin008	SOY3_bin008_01641	471	10	91	119	2.538	19.596	26.838	hypothetical protein
bin008	SOY3_bin008_01642	378	386	864	1095	122.079	231.834	307.717	Desulfoferrodoxin
bin008	SOY3_bin008_01643	516	319	746	972	73.907	146.637	200.100	putative ferritin-1
bin008	SOY3_bin008_01644	750	42	78	115	6.695	10.548	16.288	Nitroreductase family protein
bin008	SOY3_bin008_01645	348	17	66	78	5.840	19.236	23.809	hypothetical protein
bin008	SOY3_bin008_01646	984	24	129	141	2.916	13.297	15.221	Fructose-1,6-bisphosphatase class 2
bin008	SOY3_bin008_01647	1992	48	181	204	2.881	9.216	10.879	Transketolase
bin008	SOY3_bin008_01648	465	9	32	43	2.314	6.980	9.823	Ribose-5-phosphate isomerase B
bin008	SOY3_bin008_01649	825	16	63	57	2.319	7.745	7.339	outer membrane lipoprotein LolB
bin008	SOY3_bin008_01650	1707	22	105	107	1.541	6.239	6.659	tetratricopeptide repeat protein
bin008	SOY3_bin008_01651	1005	55	100	135	6.542	10.092	14.269	RNA polymerase sigma factor RpoH
bin008	SOY3_bin008_01652	2424	17	106	91	0.838	4.435	3.988	Methionine synthase
bin008	SOY3_bin008_01653	474	8	34	39	2.018	7.275	8.740	Sporulation thiol-disulfide oxidoreductase A precursor
bin008	SOY3_bin008_01654	468	10	46	40	2.554	9.969	9.079	Amino-acid acetyltransferase
bin008	SOY3_bin008_01655	525	14	50	52	3.188	9.660	10.521	Hypoxanthine phosphoribosyltransferase
bin008	SOY3_bin008_01656	1068	20	122	133	2.239	11.586	13.228	preprotein translocase subunit SecG
bin008	SOY3_bin008_01657	1329	20	54	83	1.799	4.121	6.634	hypothetical protein
bin008	SOY3_bin008_01658	1458	2	38	49	0.164	2.644	3.570	Cysteine--tRNA ligase
bin008	SOY3_bin008_01659	453	275	1043	1073	72.574	233.529	251.612	Universal stress protein G
bin008	SOY3_bin008_01660	1626	8	75	77	0.588	4.678	5.030	2-isopropylmalate synthase
bin008	SOY3_bin008_01661	1233	10	76	80	0.970	6.252	6.892	Aspartokinase
bin008	SOY3_bin008_01662	510	4	24	27	0.938	4.773	5.624	tRNA threonylcarbamoyladenosine biosynthesis protein TsaE
bin008	SOY3_bin008_01663	456	4	42	50	1.049	9.342	11.648	Carnitine transport ATP-binding protein OpuCA
bin008	SOY3_bin008_01664	1560	31	85	93	2.376	5.526	6.333	Bifunctional NAD(P)H-hydrate repair enzyme Nnr
bin008	SOY3_bin008_01665	372	26	68	74	8.356	18.540	21.131	Holo-[acyl-carrier-protein] synthase
bin008	SOY3_bin008_01666	726	65	163	195	10.703	22.772	28.532	Pyridoxine 5'-phosphate synthase
bin008	SOY3_bin008_01667	1290	18	81	92	1.668	6.369	7.576	Citrate synthase 1
bin008	SOY3_bin008_01668	2448	35	71	71	1.709	2.942	3.081	Mechanosensitive channel MscK precursor
bin008	SOY3_bin008_01669	240	337	425	428	167.867	179.611	189.436	lipoprotein
bin008	SOY3_bin008_01670	1206	4	22	25	0.397	1.850	2.202	Immunoglobulin A1 protease autotransporter precursor
bin008	SOY3_bin008_01671	225	1	2	2	0.531	0.902	0.944	hypothetical protein
bin008	SOY3_bin008_01672	1653	30	94	95	2.170	5.768	6.105	Acetyl-coenzyme A synthetase
bin008	SOY3_bin008_01673	561	9	31	44	1.918	5.605	8.331	(S)-2-hydroxypropylphosphonic acid epoxidase
bin008	SOY3_bin008_01674	582	11	33	25	2.260	5.751	4.563	HTH-type transcriptional regulator PuuR
bin008	SOY3_bin008_01675	1674	12	56	78	0.857	3.393	4.950	Long-chain-fatty-acid--CoA ligase
bin008	SOY3_bin008_01676	819	9	21	13	1.314	2.601	1.686	YciI-like protein
bin008	SOY3_bin008_01677	1053	3	11	18	0.341	1.060	1.816	Bifunctional xylanase/deacetylase precursor
bin008	SOY3_bin008_01678	474	52	131	174	13.115	28.032	38.994	hypothetical protein
bin008	SOY3_bin008_01679	2841	98	170	206	4.124	6.069	7.702	putative FAD-linked oxidoreductase
bin008	SOY3_bin008_01680	660	1	9	7	0.181	1.383	1.127	Lactate utilization protein C
bin008	SOY3_bin008_01681	1386	5	18	14	0.431	1.317	1.073	Lactate utilization protein B
bin008	SOY3_bin008_01682	723	1	14	8	0.165	1.964	1.175	Lactate utilization protein A
bin008	SOY3_bin008_01683	1344	7	76	72	0.623	5.735	5.691	UDP-glucose 6-dehydrogenase TuaD
bin008	SOY3_bin008_01684	903	40	194	236	5.296	21.791	27.762	Flagellar filament 33 kDa core protein
bin008	SOY3_bin008_01685	699	16	53	80	2.736	7.690	12.157	Ribonuclease 3
bin008	SOY3_bin008_01686	1062	138	411	492	15.535	39.253	49.212	Phosphate acetyltransferase
bin008	SOY3_bin008_01687	297	6	60	85	2.415	20.490	30.401	hypothetical protein
bin008	SOY3_bin008_01688	3180	28	125	165	1.053	3.987	5.512	photosystem I assembly protein Ycf3
bin008	SOY3_bin008_01689	2466	307	547	689	14.883	22.498	29.679	Sensor histidine kinase TodS
bin008	SOY3_bin008_01690	1704	36	98	109	2.526	5.833	6.795	Cyclic di-GMP phosphodiesterase Gmr
bin008	SOY3_bin008_01691	183	37	1136	1463	24.171	629.625	849.226	Ferredoxin-2
bin008	SOY3_bin008_01692	153	1	15	17	0.781	9.944	11.803	hypothetical protein
bin008	SOY3_bin008_01693	1038	0	30	33	0.000	2.931	3.377	ATP-dependent (S)-NAD(P)H-hydrate dehydratase
bin008	SOY3_bin008_01694	867	1	2	2	0.138	0.234	0.245	Carboxymuconolactone decarboxylase family protein
bin008	SOY3_bin008_01695	915	2	14	10	0.261	1.552	1.161	Hca operon transcriptional activator
bin008	SOY3_bin008_01696	852	5	24	21	0.702	2.857	2.618	hypothetical protein
bin008	SOY3_bin008_01697	699	12	33	40	2.052	4.788	6.079	hypothetical protein
bin008	SOY3_bin008_01698	279	8	22	24	3.428	7.998	9.138	hypothetical protein
bin008	SOY3_bin008_01699	411	3	10	19	0.873	2.468	4.911	Putative flagellin YvzB

bin008	SOY3_bin008_01700	1398	32	83	75	2.736	6.022	5.699	Poly-beta-1,6-N-acetyl-D-glucosamine synthase
bin008	SOY3_bin008_01701	2382	21	101	111	1.054	4.301	4.950	hypothetical protein
bin008	SOY3_bin008_01702	1701	10	24	31	0.703	1.431	1.936	Capsule biosynthesis protein CapA
bin008	SOY3_bin008_01703	612	198	498	657	38.678	82.534	114.036	p-benzoquinone reductase
bin008	SOY3_bin008_01704	1386	5	25	21	0.431	1.829	1.609	Cobyrinic acid A,C-diamide synthase
bin008	SOY3_bin008_01705	1104	15	61	61	1.624	5.604	5.869	cobalt-precorrin-6A synthase
bin008	SOY3_bin008_01706	711	11	64	77	1.850	9.130	11.504	hypothetical protein
bin008	SOY3_bin008_01707	1227	8	29	33	0.779	2.397	2.857	Precorrin-6Y C(5,15)-methyltransferase [decarboxylating]
bin008	SOY3_bin008_01708	795	16	38	46	2.406	4.848	6.146	Cobalt-precorrin-4 C(11)-methyltransferase
bin008	SOY3_bin008_01709	444	11	64	66	2.962	14.620	15.790	hypothetical protein
bin008	SOY3_bin008_01710	993	52	130	126	6.260	13.278	13.479	Glycerol-3-phosphate dehydrogenase [NAD(P)+]
bin008	SOY3_bin008_01711	489	21	60	79	5.134	12.445	17.161	DNA-binding transcriptional regulator AsnC
bin008	SOY3_bin008_01712	825	38	169	247	5.506	20.777	31.803	putative enoyl-CoA hydratase echA17
bin008	SOY3_bin008_01713	2697	140	251	312	6.206	9.439	12.289	succinyl-CoA synthetase subunit alpha
bin008	SOY3_bin008_01714	1128	54	133	159	5.723	11.959	14.973	Cyclic di-GMP phosphodiesterase response regulator RpfG
bin008	SOY3_bin008_01715	786	2	11	30	0.304	1.419	4.054	Proline iminopeptidase
bin008	SOY3_bin008_01716	627	1	10	19	0.191	1.618	3.219	hypothetical protein
bin008	SOY3_bin008_01717	1269	4	49	60	0.377	3.916	5.022	Glutamate-1-semialdehyde 2,1-aminomutase
bin008	SOY3_bin008_01718	450	2	12	19	0.531	2.705	4.485	glyoxalase I
bin008	SOY3_bin008_01719	429	1	0	1	0.279	0.000	0.248	hypothetical protein
bin008	SOY3_bin008_01720	390	40	116	135	12.261	30.168	36.770	Hypoxic response protein 1
bin008	SOY3_bin008_01721	501	49	55	60	11.692	11.135	12.722	hypothetical protein
bin008	SOY3_bin008_01722	246	35	51	69	17.009	21.028	29.795	Gas vesicle protein G
bin008	SOY3_bin008_01723	471	19	41	50	4.823	8.829	11.277	Zinc resistance-associated protein precursor
bin008	SOY3_bin008_01724	243	1	3	1	0.492	1.252	0.437	hypothetical protein
bin008	SOY3_bin008_01725	1473	2	3	0	0.162	0.207	0.000	Terminase-like family protein
bin008	SOY3_bin008_01726	441	5	11	23	1.355	2.530	5.540	hypothetical protein
bin008	SOY3_bin008_01727	606	3	5	11	0.592	0.837	1.928	Terminase small subunit
bin008	SOY3_bin008_01728	126	1	3	2	0.949	2.415	1.686	hypothetical protein
bin008	SOY3_bin008_01729	270	2	1	9	0.886	0.376	3.541	hypothetical protein
bin008	SOY3_bin008_01730	222	6	6	11	3.231	2.741	5.263	hypothetical protein
bin008	SOY3_bin008_01731	249	3	7	5	1.440	2.851	2.133	hypothetical protein
bin008	SOY3_bin008_01732	1143	17	45	50	1.778	3.993	4.647	1,3-propanediol dehydrogenase
bin008	SOY3_bin008_01733	1215	10	34	45	0.984	2.838	3.934	Sporulation kinase E
bin008	SOY3_bin008_01734	1332	10	39	38	0.898	2.970	3.030	Transcriptional regulatory protein ZraR
bin008	SOY3_bin008_01735	477	2	62	82	0.501	13.183	18.261	Perchlorate reductase subunit gamma precursor
bin008	SOY3_bin008_01736	1779	21	303	314	1.411	17.275	18.749	Methyl-accepting chemotaxis protein McpS
bin008	SOY3_bin008_01737	477	21	2997	3193	5.263	637.269	711.067	NADP-reducing hydrogenase subunit HndA
bin008	SOY3_bin008_01738	381	22	2551	2660	6.903	679.110	741.629	NADP-reducing hydrogenase subunit HndB
bin008	SOY3_bin008_01739	1476	73	7993	8171	5.913	549.260	588.056	NADP-reducing hydrogenase subunit HndC
bin008	SOY3_bin008_01740	1758	81	9421	9699	5.508	543.542	586.054	NADP-reducing hydrogenase subunit HndC
bin008	SOY3_bin008_01741	1476	7	65	80	0.567	4.467	5.757	Aspartate ammonia-lyase
bin008	SOY3_bin008_01742	606	11	19	32	2.170	3.180	5.609	hypothetical protein
bin008	SOY3_bin008_01743	999	12	53	67	1.436	5.381	7.124	Biotin synthase
bin008	SOY3_bin008_01744	1209	23	106	100	2.274	8.893	8.786	tRNA modification GTPase MnmE
bin008	SOY3_bin008_01745	564	27	61	82	5.723	10.970	15.444	hypothetical protein
bin008	SOY3_bin008_01746	1458	19	75	83	1.558	5.217	6.047	GTPase Era
bin008	SOY3_bin008_01747	1143	6	22	40	0.628	1.952	3.717	Aspartate aminotransferase
bin008	SOY3_bin008_01748	1182	31	59	92	3.135	5.063	8.268	Purine-binding protein precursor
bin008	SOY3_bin008_01749	1254	10	43	50	0.953	3.478	4.235	Purine-binding protein precursor
bin008	SOY3_bin008_01750	1500	6	17	19	0.478	1.150	1.346	Autoinducer 2 import ATP-binding protein LsrA
bin008	SOY3_bin008_01751	1041	5	11	10	0.574	1.072	1.020	beta-methylgalactoside transporter inner membrane component
bin008	SOY3_bin008_01752	984	5	14	9	0.607	1.443	0.972	inner membrane ABC transporter permease protein YjfF
bin008	SOY3_bin008_01753	1878	11	117	151	0.700	6.319	8.541	Filamentous hemagglutinin
bin008	SOY3_bin008_01754	1470	5	9	10	0.407	0.621	0.723	Polysaccharide biosynthesis protein
bin008	SOY3_bin008_01755	1161	0	12	14	0.000	1.048	1.281	Poly-beta-1,6-N-acetyl-D-glucosamine synthase
bin008	SOY3_bin008_01756	423	2	11	5	0.565	2.638	1.256	hypothetical protein
bin008	SOY3_bin008_01757	1098	7	32	25	0.762	2.956	2.419	GDP-mannose-dependent alpha-(1-6)-phosphatidylinositol monomannoside mannosyltransferase
bin008	SOY3_bin008_01758	1215	12	72	72	1.181	6.011	6.295	Anaerobic sulfatase-maturating enzyme
bin008	SOY3_bin008_01759	444	17	47	56	4.577	10.737	13.398	Putative universal stress protein
bin008	SOY3_bin008_01760	732	6	26	35	0.980	3.603	5.079	metal-dependent hydrolase
bin008	SOY3_bin008_01761	573	29	50	65	6.050	8.851	12.050	periplasmic protein
bin008	SOY3_bin008_01762	303	16	39	34	6.313	13.055	11.920	hypothetical protein
bin008	SOY3_bin008_01763	591	21	60	71	4.248	10.297	12.761	ECF RNA polymerase sigma-E factor
bin008	SOY3_bin008_01764	516	47	62	69	10.889	12.187	14.205	periplasmic protein
bin008	SOY3_bin008_01765	1167	38	92	109	3.893	7.996	9.922	Chemotaxis protein CheA
bin008	SOY3_bin008_01766	783	24	73	86	3.664	9.456	11.667	Anti-sigma F factor

bin008	SOY3_bin008_01767	85	1	3	11	1.406	3.580	13.747	tRNA-Pro(tgg)
bin008	SOY3_bin008_01768	465	10	37	43	2.571	8.071	9.823	hypothetical protein
bin008	SOY3_bin008_01769	1284	31	82	129	2.886	6.477	10.672	Ribonuclease Y
bin008	SOY3_bin008_01770	816	7	53	50	1.026	6.588	6.509	Undecaprenyl-phosphate mannosyltransferase
bin008	SOY3_bin008_01771	459	2	23	19	0.521	5.082	4.397	hypothetical protein
bin008	SOY3_bin008_01772	1287	111	210	281	10.311	16.550	23.193	Response regulator PleD
bin008	SOY3_bin008_01773	405	20	31	76	5.904	7.764	19.934	hypothetical protein
bin008	SOY3_bin008_01774	1338	325	1129	1474	29.038	85.584	117.023	putative glutamine synthetase 2
bin008	SOY3_bin008_01775	1377	3	31	31	0.260	2.283	2.391	Ammonia channel precursor
bin008	SOY3_bin008_01776	342	0	6	10	0.000	1.779	3.106	Nitrogen regulatory protein P-II 2
bin008	SOY3_bin008_01777	846	8	15	12	1.130	1.798	1.507	hypothetical protein
bin008	SOY3_bin008_01778	498	0	3	6	0.000	0.611	1.280	putative tRNA (adenine(37)-N6)-methyltransferase
bin008	SOY3_bin008_01779	417	48	136	198	13.761	33.079	50.438	Autophagy protein Apg17
bin008	SOY3_bin008_01780	4164	111	152	181	3.187	3.702	4.617	Translocation and assembly module TamB
bin008	SOY3_bin008_01781	462	5	70	69	1.294	15.368	15.865	hypothetical protein
bin008	SOY3_bin008_01782	627	4	45	43	0.763	7.279	7.285	hypothetical protein
bin008	SOY3_bin008_01783	984	52	263	272	6.318	27.109	29.363	Glyceraldehyde-3-phosphate dehydrogenase
bin008	SOY3_bin008_01784	1167	11	89	107	1.127	7.735	9.740	Chemotaxis protein CheY
bin008	SOY3_bin008_01785	714	58	143	179	9.711	20.314	26.631	hypothetical protein
bin008	SOY3_bin008_01786	822	29	56	63	4.218	6.910	8.141	Coenzyme F420:L-glutamate ligase
bin008	SOY3_bin008_01787	1842	17	55	59	1.103	3.029	3.402	GTP-binding protein TypA/BipA
bin008	SOY3_bin008_01788	1719	12	33	22	0.835	1.947	1.359	Non-motile and phage-resistance protein
bin008	SOY3_bin008_01789	1167	25	48	54	2.561	4.172	4.915	NAD-dependent methanol dehydrogenase
bin008	SOY3_bin008_01790	795	35	54	65	5.263	6.889	8.685	hypothetical protein
bin008	SOY3_bin008_01791	663	47	130	172	8.475	19.888	27.558	Enhancing lycopene biosynthesis protein 2
bin008	SOY3_bin008_01792	1185	6	10	11	0.605	0.856	0.986	2-isopropylmalate synthase
bin008	SOY3_bin008_01793	1770	4	5	25	0.270	0.287	1.500	Putative multidrug export ATP-binding/permease protein
bin008	SOY3_bin008_01794	219	3	16	19	1.638	7.410	9.216	hypothetical protein
bin008	SOY3_bin008_01795	969	18	30	52	2.221	3.140	5.700	Selenide, water dikinase
bin008	SOY3_bin008_01796	357	3	10	8	1.005	2.841	2.380	hypothetical protein
bin008	SOY3_bin008_01797	1851	1	14	23	0.065	0.767	1.320	Phytochrome-like protein cph1
bin008	SOY3_bin008_01798	441	2	9	8	0.542	2.070	1.927	Response regulator rcp1
bin008	SOY3_bin008_01799	1002	2	12	13	0.239	1.215	1.378	putative diguanylate cyclase AdrA
bin008	SOY3_bin008_01800	786	1	9	10	0.152	1.161	1.351	Molybdate-binding periplasmic protein precursor
bin008	SOY3_bin008_01801	1983	119	367	414	7.174	18.771	22.177	Acetyl-coenzyme A synthetase
bin008	SOY3_bin008_01802	996	12	33	47	1.440	3.361	5.013	hypothetical protein
bin008	SOY3_bin008_01803	285	13	19	33	5.453	6.762	12.300	hypothetical protein
bin008	SOY3_bin008_01804	261	19	21	31	8.703	8.161	12.617	PQ loop repeat protein
bin008	SOY3_bin008_01805	570	25	31	46	5.243	5.516	8.573	hypothetical protein
bin008	SOY3_bin008_01806	519	27	56	61	6.219	10.944	12.485	Sel1 repeat protein
bin008	SOY3_bin008_01807	990	129	613	661	15.578	62.803	70.924	Ketol-acid reductoisomerase
bin008	SOY3_bin008_01808	483	66	173	201	16.336	36.329	44.206	Putative acetolactate synthase small subunit
bin008	SOY3_bin008_01809	1692	326	857	1015	23.034	51.373	63.723	Acetolactate synthase large subunit
bin008	SOY3_bin008_01810	234	82	316	378	41.893	136.970	171.595	hypothetical protein
bin008	SOY3_bin008_01811	306	43	196	214	16.799	64.966	74.289	hypothetical protein
bin008	SOY3_bin008_01812	555	75	229	252	16.155	41.850	48.232	Septum site-determining protein DivIVA
bin008	SOY3_bin008_01813	306	17	115	109	6.642	38.118	37.839	YGGT family protein
bin008	SOY3_bin008_01814	675	5	30	27	0.886	4.508	4.249	Phosphoglycolate phosphatase
bin008	SOY3_bin008_01815	837	7	56	75	1.000	6.786	9.518	hypothetical protein
bin008	SOY3_bin008_01816	366	5	9	19	1.633	2.494	5.514	hypothetical protein
bin008	SOY3_bin008_01817	195	3	14	25	1.839	7.282	13.619	Sec-independent protein translocase protein TatA
bin008	SOY3_bin008_01818	1134	8	22	34	0.843	1.968	3.185	N-acetylmuramoyl-L-alanine amidase AmiA precursor
bin008	SOY3_bin008_01819	927	4	15	13	0.516	1.641	1.490	tRNA dimethylallyltransferase
bin008	SOY3_bin008_01820	522	6	14	18	1.374	2.720	3.663	Phosphopantetheine adenylyltransferase
bin008	SOY3_bin008_01821	594	3	19	24	0.604	3.244	4.292	Ribosomal RNA small subunit methyltransferase D
bin008	SOY3_bin008_01822	660	8	62	55	1.449	9.528	8.852	Hydrogenase isoenzymes nickel incorporation protein HypB
bin008	SOY3_bin008_01823	354	1	15	14	0.338	4.298	4.201	Hydrogenase/urease nickel incorporation protein HypA
bin008	SOY3_bin008_01824	687	2	12	17	0.348	1.772	2.629	putative membrane protein YdfK
bin008	SOY3_bin008_01825	627	19	45	37	3.623	7.279	6.269	photosystem I assembly protein Ycf3
bin008	SOY3_bin008_01826	375	16	73	66	5.101	19.745	18.696	PilZ domain protein
bin008	SOY3_bin008_01827	210	33	189	184	18.786	91.285	93.074	Molybdenum-pterin-binding protein 2
bin008	SOY3_bin008_01828	222	21	22	24	11.309	10.051	11.484	hypothetical protein
bin008	SOY3_bin008_01829	1131	15	88	127	1.586	7.892	11.928	Nitric oxide synthase oxygenase
bin008	SOY3_bin008_01830	1008	11	47	56	1.305	4.729	5.901	molybdenum cofactor biosynthesis protein A
bin008	SOY3_bin008_01831	723	2	18	23	0.331	2.525	3.379	Energy-coupling factor transporter transmembrane protein EcfT
bin008	SOY3_bin008_01832	1404	26	114	133	2.214	8.236	10.063	Putative HMP/thiamine import ATP-binding protein YkoD
bin008	SOY3_bin008_01833	579	8	106	139	1.652	18.569	25.502	hypothetical protein

bin008	SOY3_bin008_01834	2088	7	448	553	0.401	21.762	28.134	Colicin I receptor precursor
bin008	SOY3_bin008_01835	759	6	24	30	0.945	3.207	4.199	hypothetical protein
bin008	SOY3_bin008_01836	1083	5	190	249	0.552	17.794	24.423	transport protein TonB
bin008	SOY3_bin008_01837	783	10	28	40	1.527	3.627	5.427	ribonuclease Z
bin008	SOY3_bin008_01838	1284	13	45	67	1.210	3.555	5.543	Folylpolyglutamate synthase
bin008	SOY3_bin008_01839	1410	14	25	19	1.187	1.798	1.431	L-seryl-tRNA(Sec) selenium transferase
bin008	SOY3_bin008_01840	1449	66	142	184	5.445	9.940	13.489	putative M18 family aminopeptidase 1
bin008	SOY3_bin008_01841	1920	24	73	84	1.494	3.856	4.647	Selenocysteine-specific elongation factor
bin008	SOY3_bin008_01842	114	9	28	34	9.438	24.912	31.681	hypothetical protein
bin008	SOY3_bin008_01843	360	4	9	32	1.328	2.536	9.442	hypothetical protein
bin008	SOY3_bin008_01844	888	3	16	24	0.404	1.828	2.871	Sirohydrochlorin cobaltochelatase CbiKP precursor
bin008	SOY3_bin008_01845	1047	2	11	9	0.228	1.066	0.913	Hemin transport system permease protein HmuU
bin008	SOY3_bin008_01846	831	3	12	13	0.432	1.465	1.662	putative siderophore transport system ATP-binding protein YusV
bin008	SOY3_bin008_01847	864	1	7	19	0.138	0.822	2.336	Hemin-binding periplasmic protein HmuT precursor
bin008	SOY3_bin008_01848	714	10	25	35	1.674	3.551	5.207	Cobalt-precorrin-2 C(20)-methyltransferase
bin008	SOY3_bin008_01849	447	13	27	37	3.477	6.126	8.793	hypothetical protein
bin008	SOY3_bin008_01850	462	9	34	37	2.329	7.464	8.507	PTS system mannose-specific EIIAB component
bin008	SOY3_bin008_01851	441	12	96	138	3.253	22.079	33.241	PTS system mannose-specific EIIAB component
bin008	SOY3_bin008_01852	888	19	43	68	2.558	4.911	8.134	glmZ(sRNA)-inactivating NTPase
bin008	SOY3_bin008_01853	453	76	205	253	20.057	45.900	59.327	PTS system fructose-specific EIIABC component
bin008	SOY3_bin008_01854	549	53	195	197	11.541	36.026	38.117	Ribosome-associated factor Y
bin008	SOY3_bin008_01855	1437	77	235	254	6.406	16.587	18.776	RNA polymerase sigma-54 factor
bin008	SOY3_bin008_01856	537	2	31	43	0.445	5.855	8.506	3-deoxy-D-manno-octulosonate 8-phosphate phosphatase KdsC
bin008	SOY3_bin008_01857	1635	39	157	169	2.852	9.739	10.980	CTP synthase
bin008	SOY3_bin008_01858	813	8	86	98	1.176	10.729	12.805	Phosphoribosylformylglycinamidine synthase
bin008	SOY3_bin008_01859	567	5	6	7	1.054	1.073	1.311	tRNA-specific adenosine deaminase
bin008	SOY3_bin008_01860	432	6	6	6	1.660	1.409	1.475	MOSC domain protein
bin008	SOY3_bin008_01861	519	1	3	1	0.230	0.586	0.205	hypothetical protein
bin008	SOY3_bin008_01862	522	3	3	8	0.687	0.583	1.628	hypothetical protein
bin008	SOY3_bin008_01863	204	2190	1324	1969	1283.392	658.283	1025.287	hypothetical protein
bin008	SOY3_bin008_01864	94	0	1	0	0.000	1.079	0.000	tRNA-Ser(cga)
bin008	SOY3_bin008_01865	1659	37	58	72	2.666	3.546	4.610	hypothetical protein
bin008	SOY3_bin008_01866	573	14	40	68	2.921	7.080	12.606	LemA family protein
bin008	SOY3_bin008_01867	1188	9	16	29	0.906	1.366	2.593	hypothetical protein
bin008	SOY3_bin008_01868	1956	7	11	25	0.428	0.570	1.358	Formate dehydrogenase H
bin008	SOY3_bin008_01869	327	1	9	12	0.366	2.792	3.898	hypothetical protein
bin008	SOY3_bin008_01870	1023	74	99	134	8.648	9.816	13.914	Cyclic di-GMP phosphodiesterase response regulator RpfG
bin008	SOY3_bin008_01871	864	26	37	70	3.598	4.344	8.606	hypothetical protein
bin008	SOY3_bin008_01872	1770	39	117	163	2.634	6.705	9.782	hypothetical protein
bin008	SOY3_bin008_01873	2304	25	65	85	1.297	2.861	3.919	Cysteine synthase
bin008	SOY3_bin008_01874	822	7	32	26	1.018	3.949	3.360	Demethylrebeccamycin-D-glucose O-methyltransferase
bin008	SOY3_bin008_01875	711	4	16	25	0.673	2.282	3.735	hypothetical protein
bin008	SOY3_bin008_01876	555	26	93	107	5.600	16.996	20.480	hypothetical protein
bin008	SOY3_bin008_01877	171	11	31	37	7.690	18.387	22.985	hypothetical protein
bin008	SOY3_bin008_01878	435	6	13	22	1.649	3.031	5.372	PilZ domain protein
bin008	SOY3_bin008_01879	405	34	92	117	10.036	23.040	30.687	hypothetical protein
bin008	SOY3_bin008_01880	1491	60	141	222	4.811	9.592	15.816	Non-motile and phage-resistance protein
bin008	SOY3_bin008_01881	879	22	85	108	2.992	9.808	13.052	dTDP-4-dehydrorhamnose reductase
bin008	SOY3_bin008_01882	1023	11	50	49	1.285	4.957	5.088	dTDP-glucose 4,6-dehydratase
bin008	SOY3_bin008_01883	76	0	1	4	0.000	1.335	5.591	tRNA-Val(cac)
bin008	SOY3_bin008_01884	327	2	19	31	0.731	5.893	10.070	hypothetical protein
bin008	SOY3_bin008_01885	423	1	12	10	0.283	2.877	2.511	Acyl-coenzyme A thioesterase PaaI
bin008	SOY3_bin008_01886	471	0	5	3	0.000	1.077	0.677	tRNA (cytidine(34)-2'-O)-methyltransferase
bin008	SOY3_bin008_01887	1128	0	7	4	0.000	0.629	0.377	Oxygen-independent coproporphyrinogen-III oxidase 1
bin008	SOY3_bin008_01888	1350	7	22	22	0.620	1.653	1.731	Murein DD-endopeptidase MepM
bin008	SOY3_bin008_01889	861	13	26	23	1.805	3.063	2.838	Glycine betaine-binding protein OpuAC precursor
bin008	SOY3_bin008_01890	837	2	8	8	0.286	0.969	1.015	Glycine betaine transport system permease protein OpuAB
bin008	SOY3_bin008_01891	1203	3	5	6	0.298	0.422	0.530	Glycine betaine/carnitine transport ATP-binding protein GbuA
bin008	SOY3_bin008_01892	537	50	63	82	11.131	11.899	16.221	hypothetical protein
bin008	SOY3_bin008_01893	1182	49	72	109	4.956	6.178	9.796	DisA bacterial checkpoint controller nucleotide-binding protein
bin008	SOY3_bin008_01894	1086	48	162	229	5.284	15.130	22.399	D-alanyl-D-alanine carboxypeptidase DacF precursor
bin008	SOY3_bin008_01895	1176	28	29	55	2.846	2.501	4.968	hypothetical protein
bin008	SOY3_bin008_01896	603	7	14	19	1.388	2.355	3.347	putative metallo-hydrolase
bin008	SOY3_bin008_01897	1500	8	31	46	0.638	2.096	3.258	Thermostable carboxypeptidase 1
bin008	SOY3_bin008_01898	972	16	53	68	1.968	5.530	7.431	hypothetical protein
bin008	SOY3_bin008_01899	747	25	59	63	4.001	8.011	8.959	Molybdate-binding periplasmic protein precursor
bin008	SOY3_bin008_01900	1344	11	100	142	0.978	7.547	11.223	Tryptophan synthase beta chain

bin008	SOY3_bin008_01901	1194	2	154	173	0.200	13.082	15.391	Transcriptional regulator ModE
bin008	SOY3_bin008_01902	717	2	26	41	0.333	3.678	6.074	Pyrethroid hydrolase
bin008	SOY3_bin008_01903	738	1	65	74	0.162	8.933	10.651	Sulfate/thiosulfate import ATP-binding protein CysA
bin008	SOY3_bin008_01904	720	1	132	160	0.166	18.595	23.606	Putative binding protein precursor
bin008	SOY3_bin008_01905	687	0	18	21	0.000	2.657	3.247	Sulfate transport system permease protein CysW
bin008	SOY3_bin008_01906	240	5	35	28	2.491	14.791	12.393	FeoA domain protein
bin008	SOY3_bin008_01907	249	6	7	9	2.881	2.851	3.839	Ferrous iron transport protein A
bin008	SOY3_bin008_01908	480	13	43	29	3.238	9.086	6.418	Putative redox-active protein (C_GCAxxG_C_C)
bin008	SOY3_bin008_01909	2049	54	151	170	3.151	7.475	8.813	Methyl-accepting chemotaxis protein III
bin008	SOY3_bin008_01910	1848	37	88	89	2.394	4.830	5.116	Sensor protein ZraS
bin008	SOY3_bin008_01911	1359	40	110	144	3.519	8.210	11.256	Transcriptional regulatory protein ZraR
bin008	SOY3_bin008_01912	1125	16	31	37	1.700	2.795	3.494	putative cell wall hydrolase LytN precursor
bin008	SOY3_bin008_01913	1173	14	35	39	1.427	3.026	3.532	Arylesterase precursor
bin008	SOY3_bin008_01914	216	19	55	53	10.516	25.826	26.065	hypothetical protein
bin008	SOY3_bin008_01915	207	1560	400	361	900.948	195.995	185.254	Molybdenum-pterin-binding protein 2
bin008	SOY3_bin008_01916	210	835	308	307	475.349	148.760	155.292	Molybdenum-pterin-binding protein 2
bin008	SOY3_bin008_01917	306	56	92	88	21.878	30.494	30.549	hypothetical protein
bin008	SOY3_bin008_01918	666	36	57	89	6.462	8.681	14.195	DNA polymerase/3'-5' exonuclease PolX
bin008	SOY3_bin008_01919	495	28	131	157	6.762	26.842	33.692	hypothetical protein
bin008	SOY3_bin008_01920	1338	60	124	193	5.361	9.400	15.323	(Dimethylallyl)adenosine tRNA methylthiotransferase MiaB
bin008	SOY3_bin008_01921	1146	2	9	6	0.209	0.797	0.556	Queuine tRNA-ribosyltransferase
bin008	SOY3_bin008_01922	2883	6	21	38	0.249	0.739	1.400	Spermidine synthase
bin008	SOY3_bin008_01923	639	0	8	9	0.000	1.270	1.496	Endonuclease III
bin008	SOY3_bin008_01924	555	26	139	149	5.600	25.403	28.518	pyruvoyl-dependent arginine decarboxylase
bin008	SOY3_bin008_01925	732	10	44	65	1.633	6.097	9.433	cell division protein FtsN
bin008	SOY3_bin008_01926	1659	7	84	83	0.504	5.136	5.314	Arginine--tRNA ligase
bin008	SOY3_bin008_01927	951	8	44	35	1.006	4.693	3.909	Polyketide biosynthesis malonyl CoA-acyl carrier protein transacylase BaeC
bin008	SOY3_bin008_01928	699	7	18	28	1.197	2.612	4.255	Ribosomal RNA small subunit methyltransferase G
bin008	SOY3_bin008_01929	231	0	7	4	0.000	3.074	1.839	hypothetical protein
bin008	SOY3_bin008_01930	465	7	14	17	1.800	3.054	3.884	Ribosomal RNA large subunit methyltransferase H
bin008	SOY3_bin008_01931	585	4	4	9	0.817	0.694	1.634	hypothetical protein
bin008	SOY3_bin008_01932	933	170	320	409	21.783	34.787	46.566	Epimerase family protein
bin008	SOY3_bin008_01933	1359	89	148	168	7.829	11.046	13.132	Cyclic di-GMP phosphodiesterase response regulator RpfG
bin008	SOY3_bin008_01934	741	14	21	35	2.259	2.874	5.017	Laccase domain protein YfiH
bin008	SOY3_bin008_01935	612	26	34	49	5.079	5.635	8.505	putative 5-formyltetrahydrofolate cyclo-ligase
bin008	SOY3_bin008_01936	681	93	140	194	16.326	20.851	30.261	Calcineurin-like phosphoesterase superfamily domain protein
bin008	SOY3_bin008_01937	1761	55	111	147	3.734	6.393	8.867	Cyclic di-GMP phosphodiesterase response regulator RpfG
bin008	SOY3_bin008_01938	765	16	34	48	2.500	4.508	6.665	Exodeoxyribonuclease
bin008	SOY3_bin008_01939	714	271	684	853	45.375	97.166	126.906	Carbonic anhydrase 2
bin008	SOY3_bin008_01940	1287	2	9	20	0.186	0.709	1.651	Diaminopimelate decarboxylase
bin008	SOY3_bin008_01941	453	2	5	16	0.528	1.120	3.752	hypothetical protein
bin008	SOY3_bin008_01942	285	1	0	8	0.419	0.000	2.982	cellulose synthase subunit BcsC
bin008	SOY3_bin008_01943	1167	19	103	109	1.946	8.952	9.922	LL-diaminopimelate aminotransferase
bin008	SOY3_bin008_01944	456	3	13	19	0.787	2.892	4.426	2-amino-4-hydroxy-6-hydroxymethyldihydropteridine pyrophosphokinase
bin008	SOY3_bin008_01945	312	10	42	53	3.832	13.654	18.045	YHS domain protein
bin008	SOY3_bin008_01946	1734	40	71	102	2.758	4.153	6.249	Adenine deaminase
bin008	SOY3_bin008_01947	603	7	36	31	1.388	6.055	5.461	Sulfurtransferase TusA
bin008	SOY3_bin008_01948	1134	15	51	47	1.581	4.562	4.403	Sporulation kinase E
bin008	SOY3_bin008_01949	786	4	11	25	0.608	1.419	3.379	Ribosomal large subunit pseudouridine synthase B
bin008	SOY3_bin008_01950	681	5	16	18	0.878	2.383	2.808	Outer-membrane lipoprotein carrier protein precursor
bin008	SOY3_bin008_01951	2328	26	60	63	1.335	2.614	2.875	DNA translocase FtsK
bin008	SOY3_bin008_01952	558	23	85	100	4.928	15.450	19.037	Elongation factor P
bin008	SOY3_bin008_01953	474	6	30	43	1.513	6.419	9.637	3-dehydroquinate dehydratase
bin008	SOY3_bin008_01954	684	53	142	177	9.263	21.057	27.488	putative GTP-binding protein EngB
bin008	SOY3_bin008_01955	192	52	142	159	32.378	75.014	87.968	hypothetical protein
bin008	SOY3_bin008_01956	813	4	8	10	0.588	0.998	1.307	Undecaprenyl-diphosphatase
bin008	SOY3_bin008_01957	77	0	1	0	0.000	1.317	0.000	tRNA-Met(cat)
bin008	SOY3_bin008_01958	759	0	1	1	0.000	0.134	0.140	Surfactin synthase thioesterase subunit
bin008	SOY3_bin008_01959	85	0	4	3	0.000	4.773	3.749	tRNA-Leu(tag)
bin008	SOY3_bin008_01960	861	6	23	31	0.833	2.709	3.825	Hydroxymethylpyrimidine/phosphomethylpyrimidine kinase
bin008	SOY3_bin008_01961	981	0	10	19	0.000	1.034	2.057	hypothetical protein
bin008	SOY3_bin008_01962	2175	10	46	48	0.550	2.145	2.344	ATP-dependent RecD-like DNA helicase
bin008	SOY3_bin008_01963	615	6	19	27	1.166	3.134	4.664	Recombination protein RecR
bin008	SOY3_bin008_01964	312	20	62	62	7.663	20.155	21.109	Nucleoid-associated protein
bin008	SOY3_bin008_01965	1743	22	62	80	1.509	3.608	4.876	DNA polymerase III subunit tau
bin008	SOY3_bin008_01966	927	21	255	263	2.708	27.901	30.137	Branched-chain-amino-acid aminotransferase
bin008	SOY3_bin008_01967	138	5	2	4	4.331	1.470	3.079	hypothetical protein

bin008	SOY3_bin008_01968	1002	11	12	22	1.312	1.215	2.332	tRNA-dihydrouridine synthase C
bin008	SOY3_bin008_01969	843	4	31	34	0.567	3.730	4.284	4-hydroxy-3-methylbut-2-enyl diphosphate reductase
bin008	SOY3_bin008_01970	990	80	259	340	9.661	26.535	36.482	Chemotaxis protein CheV
bin008	SOY3_bin008_01971	1005	25	64	89	2.974	6.459	9.407	Hydrogenase expression/formation protein HypE
bin008	SOY3_bin008_01972	1092	22	55	66	2.408	5.109	6.420	Hydrogenase expression/formation protein HypD
bin008	SOY3_bin008_01973	228	4	30	40	2.097	13.346	18.636	Hydrogenase isoenzymes formation protein HypC
bin008	SOY3_bin008_01974	1845	164	312	397	10.627	17.152	22.857	Methyl-accepting chemotaxis protein 4
bin008	SOY3_bin008_01975	369	16	47	66	5.184	12.919	19.000	hypothetical protein
bin008	SOY3_bin008_01976	645	17	49	77	3.151	7.705	12.681	phosphodiesterase
bin008	SOY3_bin008_01977	915	1	16	17	0.131	1.774	1.974	D-alanine--D-alanine ligase
bin008	SOY3_bin008_01978	1128	50	165	190	5.299	14.836	17.893	Carbamoyl-phosphate synthase small chain
bin008	SOY3_bin008_01979	819	89	195	324	12.991	24.149	42.023	Tetratricopeptide repeat protein
bin008	SOY3_bin008_01980	942	52	74	89	6.599	7.968	10.036	HPP family protein
bin008	SOY3_bin008_01981	849	4	29	35	0.563	3.465	4.379	hypothetical protein
bin008	SOY3_bin008_01982	1488	14	30	49	1.125	2.045	3.498	L-aspartate oxidase
bin008	SOY3_bin008_01983	948	29	93	116	3.657	9.950	12.998	hypothetical protein
bin008	SOY3_bin008_01984	489	30	90	101	7.334	18.668	21.940	Universal stress protein family protein
bin008	SOY3_bin008_01985	1002	121	211	256	14.437	21.358	27.140	4-hydroxythreonine-4-phosphate dehydrogenase 1
bin008	SOY3_bin008_01986	1929	82	227	287	5.082	11.936	15.804	1,4-alpha-glucan branching enzyme GlgB
bin008	SOY3_bin008_01987	1458	40	135	117	3.280	9.391	8.524	Glycogen synthase
bin008	SOY3_bin008_01988	462	47	119	156	12.162	26.125	35.868	Bifunctional NMN adenylyltransferase/Nudix hydrolase
bin008	SOY3_bin008_01989	915	9	15	13	1.176	1.663	1.509	Lipopolysaccharide heptosyltransferase 1
bin008	SOY3_bin008_01990	642	0	11	8	0.000	1.738	1.324	Threonylcarbamoyl-AMP synthase
bin008	SOY3_bin008_01991	1671	8	35	34	0.572	2.124	2.161	N-acetylglucosaminyl-diphospho-decaprenol L-rhamnosyltransferase
bin008	SOY3_bin008_01992	600	6	28	32	1.195	4.733	5.665	tRNA (guanosine(18)-2'-O)-methyltransferase
bin008	SOY3_bin008_01993	888	47	120	163	6.327	13.706	19.499	3-methyl-2-oxobutanoate hydroxymethyltransferase
bin008	SOY3_bin008_01994	1347	20	30	48	1.775	2.259	3.785	Phosphate regulon transcriptional regulatory protein PhoB
bin008	SOY3_bin008_01995	807	2	5	25	0.296	0.628	3.291	Prolipoprotein diacylglyceryl transferase
bin008	SOY3_bin008_01996	255	6	20	29	2.813	7.955	12.081	hypothetical protein
bin008	SOY3_bin008_01997	801	9	23	40	1.343	2.912	5.305	Glutamine cyclotransferase
bin008	SOY3_bin008_01998	543	26	92	135	5.724	17.185	26.410	hypothetical protein
bin008	SOY3_bin008_01999	396	14	65	110	4.226	16.648	29.507	hypothetical protein
bin008	SOY3_bin008_02000	675	3	12	17	0.531	1.803	2.675	cAMP receptor protein
bin008	SOY3_bin008_02001	1314	27	36	41	2.456	2.779	3.315	Ferredoxin
bin008	SOY3_bin008_02002	318	242	175	268	90.977	55.817	89.524	Cupin domain protein
bin008	SOY3_bin008_02003	870	140	127	205	19.238	14.806	25.030	NADH-dependent phenylglyoxylate dehydrogenase subunit beta
bin008	SOY3_bin008_02004	1617	717	1091	1456	53.010	68.434	95.649	Hydroxylamine reductase
bin008	SOY3_bin008_02005	768	30	71	83	4.670	9.377	11.480	hypothetical protein
bin008	SOY3_bin008_02006	1275	24	45	53	2.250	3.580	4.416	hypothetical protein
bin008	SOY3_bin008_02007	1428	34	86	119	2.846	6.108	8.852	hypothetical protein
bin008	SOY3_bin008_02008	165	6	22	40	4.347	13.524	25.752	hypothetical protein
bin008	SOY3_bin008_02009	411	13	27	45	3.781	6.663	11.631	Gamma-L-glutamyl-butirosin B gamma-glutamyl cyclotransferase
bin008	SOY3_bin008_02010	1182	11	23	33	1.113	1.974	2.966	L-fuculose phosphate aldolase
bin008	SOY3_bin008_02011	1755	27	43	42	1.839	2.485	2.542	putative oxidoreductase YdhV
bin008	SOY3_bin008_02012	789	5	74	97	0.758	9.513	13.059	hypothetical protein
bin008	SOY3_bin008_02013	1197	9	49	64	0.899	4.152	5.680	Response regulator PleD
bin008	SOY3_bin008_02014	1080	41	120	122	4.538	11.270	12.000	Cyclic di-GMP phosphodiesterase response regulator RpfG
bin008	SOY3_bin008_02015	2679	55	89	121	2.454	3.370	4.798	putative cation-transporting ATPase F
bin008	SOY3_bin008_02016	1827	53	94	168	3.468	5.218	9.768	Translocation and assembly module TamA precursor
bin008	SOY3_bin008_02017	585	0	0	0	0.000	0.000	0.000	hypothetical protein
bin008	SOY3_bin008_02018	417	1	9	13	0.287	2.189	3.312	hypothetical protein
bin008	SOY3_bin008_02019	858	4	23	32	0.557	2.719	3.962	Septum site-determining protein MinD
bin008	SOY3_bin008_02020	465	3	20	14	0.771	4.362	3.198	NifU-like protein
bin008	SOY3_bin008_02021	336	5	25	20	1.779	7.547	6.323	Dinitrogenase iron-molybdenum cofactor
bin008	SOY3_bin008_02022	882	6	40	33	0.813	4.600	3.974	Ferredoxin
bin008	SOY3_bin008_02023	882	5	30	27	0.678	3.450	3.252	Septum site-determining protein MinD
bin008	SOY3_bin008_02024	381	9	43	37	2.824	11.447	10.316	Dinitrogenase iron-molybdenum cofactor
bin008	SOY3_bin008_02025	246	2	6	4	0.972	2.474	1.727	Zinc ribbon domain protein
bin008	SOY3_bin008_02026	363	0	4	5	0.000	1.118	1.463	Response regulator receiver protein
bin008	SOY3_bin008_02027	828	5	10	13	0.722	1.225	1.668	Ribonuclease
bin008	SOY3_bin008_02028	903	2	6	8	0.265	0.674	0.941	hypothetical protein
bin008	SOY3_bin008_02029	396	2	4	7	0.604	1.025	1.878	RNA polymerase-binding transcription factor DksA
bin008	SOY3_bin008_02030	1065	12	33	50	1.347	3.143	4.987	Glycogen synthase
bin008	SOY3_bin008_02031	1140	27	64	71	2.831	5.694	6.616	Lipopolysaccharide core biosynthesis protein RfaG
bin008	SOY3_bin008_02032	1269	20	53	53	1.884	4.236	4.437	Glycogen synthase
bin008	SOY3_bin008_02033	1932	26	67	84	1.609	3.517	4.619	Asparagine synthetase [glutamine-hydrolyzing] 1
bin008	SOY3_bin008_02034	2073	42	95	137	2.422	4.648	7.020	Xyloglucanase Xgh74A precursor

bin008	SOY3_bin008_02035	651	11	29	30	2.020	4.518	4.895	hypothetical protein
bin008	SOY3_bin008_02036	1434	11	49	30	0.917	3.466	2.222	putative sugar transferase EpsL
bin008	SOY3_bin008_02037	1614	9	39	29	0.667	2.451	1.909	putative peptidoglycan biosynthesis protein MurJ
bin008	SOY3_bin008_02038	1155	17	104	140	1.760	9.133	12.876	Polysaccharide biosynthesis/export protein
bin008	SOY3_bin008_02039	1452	24	87	125	1.976	6.077	9.145	Tyrosine-protein kinase ptk
bin008	SOY3_bin008_02040	1407	12	37	53	1.020	2.667	4.001	O-Antigen ligase
bin008	SOY3_bin008_02041	798	15	39	62	2.247	4.957	8.253	putative S-adenosylmethionine-dependent methyltransferase/MSMEI_2290
bin008	SOY3_bin008_02042	852	11	45	44	1.543	5.357	5.486	31-O-demethyl-FK506 methyltransferase FkbM
bin008	SOY3_bin008_02043	162	1	9	13	0.738	5.635	8.524	hypothetical protein
bin008	SOY3_bin008_02044	1233	6	64	79	0.582	5.265	6.806	Capsular glucan synthase
bin008	SOY3_bin008_02045	1200	10	46	69	0.996	3.888	6.108	Alpha-D-kanosaminyltransferase
bin008	SOY3_bin008_02046	813	16	102	130	2.353	12.725	16.986	Tyrosine-protein kinase wzc
bin008	SOY3_bin008_02047	1098	13	40	44	1.415	3.695	4.257	Aminodeoxyfutalosine synthase
bin008	SOY3_bin008_02048	549	8	21	34	1.742	3.880	6.579	hypothetical protein
bin008	SOY3_bin008_02049	987	14	20	23	1.696	2.055	2.475	NMT1/THI5 like protein
bin008	SOY3_bin008_02050	2007	38	57	79	2.264	2.881	4.181	Phosphoserine phosphatase RsbU
bin008	SOY3_bin008_02051	402	196	764	794	58.288	192.762	209.809	Transcriptional regulatory protein MucR
bin008	SOY3_bin008_02052	678	67	118	136	11.814	17.653	21.308	hypothetical protein
bin008	SOY3_bin008_02053	1155	101	215	205	10.454	18.880	18.854	Hydrogenase transcriptional regulatory protein hupR1
bin008	SOY3_bin008_02054	1215	261	474	584	25.681	39.569	51.058	Hydrogenase transcriptional regulatory protein hupR1
bin008	SOY3_bin008_02055	1413	25	41	44	2.115	2.943	3.308	ribulose-1,5-biphosphate synthetase
bin008	SOY3_bin008_02056	696	10	30	41	1.718	4.372	6.258	Major phosphate-irrepressible acid phosphatase precursor
bin008	SOY3_bin008_02057	441	62	98	134	16.807	22.539	32.277	hypothetical protein
bin008	SOY3_bin008_02058	1362	33	71	106	2.897	5.287	8.267	Gamma-D-glutamyl-L-lysine endopeptidase
bin008	SOY3_bin008_02059	846	27	57	58	3.815	6.834	7.283	WLM domain protein
bin008	SOY3_bin008_02060	351	0	6	5	0.000	1.734	1.513	hypothetical protein
bin008	SOY3_bin008_02061	2223	31	94	135	1.667	4.289	6.451	Ribonuclease R
bin008	SOY3_bin008_02062	855	16	37	42	2.237	4.389	5.218	Phosphate-binding protein PstS 1 precursor
bin008	SOY3_bin008_02063	2223	25	34	54	1.344	1.551	2.580	Autoinducer 2 sensor kinase/phosphatase LuxQ
bin008	SOY3_bin008_02064	1278	58	180	212	5.426	14.286	17.621	hypothetical protein
bin008	SOY3_bin008_02065	456	10	57	39	2.622	12.678	9.085	Transcriptional regulatory protein TcrA
bin008	SOY3_bin008_02066	678	12	26	36	2.116	3.890	5.640	Transcriptional regulatory protein CusR
bin008	SOY3_bin008_02067	1374	37	62	72	3.219	4.577	5.566	Sensor kinase CusS
bin008	SOY3_bin008_02068	924	13	34	33	1.682	3.732	3.794	HTH-type transcriptional regulator GltC
bin008	SOY3_bin008_02069	405	0	1	0	0.000	0.250	0.000	Holin-like protein CidA
bin008	SOY3_bin008_02070	690	5	11	12	0.866	1.617	1.847	Inner membrane protein YohK
bin008	SOY3_bin008_02071	1083	8	24	22	0.883	2.248	2.158	Magnesium transport protein CorA
bin008	SOY3_bin008_02072	1965	23	86	85	1.399	4.439	4.595	Limonene hydroxylase
bin008	SOY3_bin008_02073	1197	5	20	18	0.499	1.695	1.597	putative transporter
bin008	SOY3_bin008_02074	672	8	14	15	1.423	2.113	2.371	putative HTH-type transcriptional regulator YttP
bin008	SOY3_bin008_02075	1182	2462	9821	12819	249.010	842.739	1152.037	1,3-propanediol dehydrogenase
bin008	SOY3_bin008_02076	1239	57	95	160	5.500	7.777	13.718	hypothetical protein
bin008	SOY3_bin008_02077	1176	46	82	130	4.676	7.072	11.743	Diguanylate cyclase DosC
bin008	SOY3_bin008_02078	813	8	27	24	1.176	3.368	3.136	Shikimate dehydrogenase
bin008	SOY3_bin008_02079	546	18	80	97	3.941	14.861	18.872	Pyruvate synthase subunit PorC
bin008	SOY3_bin008_02080	756	11	84	114	1.739	11.270	16.018	2-oxoglutarate oxidoreductase subunit KorB
bin008	SOY3_bin008_02081	1065	26	108	130	2.919	10.286	12.967	2-oxoglutarate oxidoreductase subunit KorA
bin008	SOY3_bin008_02082	228	2	22	24	1.049	9.787	11.182	Benzoyl-CoA oxygenase component A
bin008	SOY3_bin008_02083	1101	1	18	19	0.109	1.658	1.833	S-adenosylmethionine:tRNA ribosyltransferase-isomerase
bin008	SOY3_bin008_02084	321	29	456	535	10.800	144.084	177.043	hypothetical protein
bin008	SOY3_bin008_02085	945	16	97	127	2.024	10.411	14.276	hypothetical protein
bin008	SOY3_bin008_02086	573	99	389	506	20.655	68.857	93.805	TPR repeat-containing protein YrrB
bin008	SOY3_bin008_02087	411	4	43	40	1.163	10.612	10.338	Flagellar basal body rod protein FlgB
bin008	SOY3_bin008_02088	444	4	35	37	1.077	7.995	8.852	Flagellar basal-body rod protein FlgC
bin008	SOY3_bin008_02089	336	3	25	29	1.067	7.547	9.168	Flagellar hook-basal body complex protein FliE
bin008	SOY3_bin008_02090	1641	37	131	144	2.695	8.097	9.321	Flagellar M-ring protein
bin008	SOY3_bin008_02091	999	28	94	97	3.351	9.544	10.314	Flagellar motor switch protein FliG
bin008	SOY3_bin008_02092	741	18	54	57	2.904	7.391	8.171	Yop proteins translocation protein L
bin008	SOY3_bin008_02093	1329	11	29	60	0.989	2.213	4.796	putative ATP synthase YscN
bin008	SOY3_bin008_02094	1416	7	28	27	0.591	2.006	2.025	Alkaline phosphatase synthesis sensor protein PhoR
bin008	SOY3_bin008_02095	756	17	48	41	2.688	6.440	5.761	Transcriptional activator protein CopR
bin008	SOY3_bin008_02096	171	41	998	815	28.664	591.956	506.281	hypothetical protein
bin008	SOY3_bin008_02097	1245	137	214	268	13.155	17.434	22.866	Cyclic di-GMP phosphodiesterase response regulator RpfG
bin008	SOY3_bin008_02098	645	14	32	24	2.595	5.032	3.953	GDSL-like Lipase/Acylhydrolase
bin008	SOY3_bin008_02099	345	33	55	73	11.435	16.170	22.477	hypothetical protein
bin008	SOY3_bin008_02100	636	44	58	99	8.271	9.250	16.535	hypothetical protein
bin008	SOY3_bin008_02101	996	64	132	162	7.682	13.442	17.278	hypothetical protein

bin008	SOY3_bin008_02102	756	75	216	254	11.860	28.979	35.690	Chromosome partition protein Smc
bin008	SOY3_bin008_02103	3003	44	85	71	1.752	2.871	2.511	1-pyrroline-5-carboxylate dehydrogenase 1
bin008	SOY3_bin008_02104	474	10	9	9	2.522	1.926	2.017	Leucine-responsive regulatory protein
bin008	SOY3_bin008_02105	1611	32	95	127	2.375	5.981	8.374	Trifunctional nucleotide phosphoesterase protein YfkN precursor
bin008	SOY3_bin008_02106	630	6	32	20	1.139	5.152	3.372	Global nitrogen regulator
bin008	SOY3_bin008_02107	765	19	69	76	2.969	9.148	10.553	Lipopolysaccharide export system ATP-binding protein LptB
bin008	SOY3_bin008_02108	1125	107	377	502	11.370	33.989	47.400	Leucine-, isoleucine-, valine-, threonine-, and alanine-binding protein precursor
bin008	SOY3_bin008_02109	918	18	46	53	2.344	5.082	6.133	High-affinity branched-chain amino acid transport system permease protein LivH
bin008	SOY3_bin008_02110	1224	12	52	85	1.172	4.309	7.377	High-affinity branched-chain amino acid transport system permease protein LivH
bin008	SOY3_bin008_02111	717	4	35	39	0.667	4.951	5.778	High-affinity branched-chain amino acid transport ATP-binding protein LivF
bin008	SOY3_bin008_02112	279	3	8	16	1.285	2.908	6.092	hypothetical protein
bin008	SOY3_bin008_02113	1824	4	25	20	0.262	1.390	1.165	Signal transduction histidine-protein kinase BarA
bin008	SOY3_bin008_02114	498	7	28	34	1.680	5.703	7.252	hypothetical protein
bin008	SOY3_bin008_02115	918	16	29	43	2.084	3.204	4.976	putative inner membrane transporter YicL
bin008	SOY3_bin008_02116	288	392	840	1157	162.719	295.830	426.747	Cytochrome c-554(548)
bin008	SOY3_bin008_02117	1119	68	135	178	7.265	12.237	16.897	NADH oxidase
bin008	SOY3_bin008_02118	348	19	50	65	6.527	14.573	19.841	hypothetical protein
bin008	SOY3_bin008_02119	1329	12	33	40	1.079	2.519	3.197	Ribosomal RNA small subunit methyltransferase B
bin008	SOY3_bin008_02120	834	3	13	7	0.430	1.581	0.892	hypothetical protein
bin008	SOY3_bin008_02121	981	25	82	86	3.047	8.478	9.312	Methionyl-tRNA formyltransferase
bin008	SOY3_bin008_02122	525	5	60	52	1.139	11.592	10.521	Peptide deformylase 1
bin008	SOY3_bin008_02123	1812	11	101	99	0.726	5.654	5.804	Aspartate--tRNA ligase
bin008	SOY3_bin008_02124	1251	6	45	43	0.573	3.648	3.651	Histidine--tRNA ligase
bin008	SOY3_bin008_02125	771	23	62	70	3.566	8.156	9.644	Arabinose 5-phosphate isomerase KdsD
bin008	SOY3_bin008_02126	1263	17	96	118	1.609	7.709	9.924	biofilm formation regulator HmsP
bin008	SOY3_bin008_02127	699	3	4	7	0.513	0.580	1.064	putative dual-specificity RNA methyltransferase RlmN
bin008	SOY3_bin008_02128	2196	9	79	73	0.490	3.649	3.531	Anaerobic ribonucleoside-triphosphate reductase
bin008	SOY3_bin008_02129	924	2	18	15	0.259	1.976	1.724	Daunorubicin/doxorubicin resistance ATP-binding protein DrrA
bin008	SOY3_bin008_02130	786	1	4	9	0.152	0.516	1.216	Inner membrane transport permease YadH
bin008	SOY3_bin008_02131	834	3	13	13	0.430	1.581	1.656	Cephalosporin-C deacetylase
bin008	SOY3_bin008_02132	720	5	5	11	0.830	0.704	1.623	Inner membrane protein YgaZ
bin008	SOY3_bin008_02133	327	5	12	18	1.828	3.722	5.847	Branched-chain amino acid transport protein (AzlD)
bin008	SOY3_bin008_02134	2829	26	114	129	1.099	4.087	4.844	Sensory/regulatory protein RpfC
bin008	SOY3_bin008_02135	513	0	22	31	0.000	4.350	6.419	Murein DD-endopeptidase MepH precursor
bin008	SOY3_bin008_02136	486	38	71	87	9.347	14.818	19.016	Chemotaxis protein CheW
bin008	SOY3_bin008_02137	2334	46	108	158	2.356	4.693	7.191	Soluble lytic murein transglycosylase precursor
bin008	SOY3_bin008_02138	1269	120	268	325	11.305	21.420	27.205	translocation protein TolB
bin008	SOY3_bin008_02139	660	35	128	155	6.340	19.671	24.947	Nitrate reductase gamma subunit
bin008	SOY3_bin008_02140	1317	97	183	249	8.805	14.094	20.084	succinate dehydrogenase/fumarate reductase iron-sulfur subunit
bin008	SOY3_bin008_02141	381	52	77	87	16.316	20.498	24.256	Acidic cytochrome c3 precursor
bin008	SOY3_bin008_02142	825	3	26	37	0.435	3.196	4.764	hypothetical protein
bin008	SOY3_bin008_02143	414	13	90	126	3.754	22.049	32.330	Chemotaxis protein CheY
bin008	SOY3_bin008_02144	1512	23	45	64	1.819	3.019	4.496	hypothetical protein
bin008	SOY3_bin008_02145	2571	33	183	156	1.534	7.219	6.445	Maltodextrin phosphorylase
bin008	SOY3_bin008_02146	465	1	50	35	0.257	10.906	7.995	Deoxyuridine 5'-triphosphate nucleotidohydrolase
bin008	SOY3_bin008_02147	324	8	11	23	2.952	3.444	7.541	Transposase DDE domain protein
bin008	SOY3_bin008_02148	1404	4	29	26	0.341	2.095	1.967	putative diguanylate cyclase YdaM
bin008	SOY3_bin008_02149	183	0	0	0	0.000	0.000	0.000	hypothetical protein
bin008	SOY3_bin008_02150	654	41	152	175	7.495	23.573	28.424	hypothetical protein
bin008	SOY3_bin008_02151	2550	58	134	155	2.719	5.330	6.457	Autoinducer 2 sensor kinase/phosphatase LuxQ
bin008	SOY3_bin008_02152	77	0	0	2	0.000	0.000	2.759	tRNA-Met(cat)
bin008	SOY3_bin008_02153	255	14	44	55	6.563	17.501	22.911	hypothetical protein
bin008	SOY3_bin008_02154	138	22	131	136	19.059	96.282	104.686	50S ribosomal protein L34
bin008	SOY3_bin008_02155	1599	4	55	57	0.299	3.489	3.787	Membrane protein insertase YidC
bin008	SOY3_bin008_02156	1104	5	81	64	0.541	7.442	6.158	DNA polymerase III subunits gamma and tau
bin008	SOY3_bin008_02157	921	0	2	2	0.000	0.220	0.231	putative DMT superfamily transporter inner membrane protein
bin008	SOY3_bin008_02158	2532	67	156	148	3.163	6.249	6.209	Virulence sensor protein BvgS precursor
bin008	SOY3_bin008_02159	2976	151	264	318	6.066	8.998	11.351	Signal transduction histidine-protein kinase BarA
bin008	SOY3_bin008_02160	1227	127	155	230	12.374	12.813	19.912	Sensory/regulatory protein RpfC
bin008	SOY3_bin008_02161	1317	97	299	347	8.805	23.027	27.988	Muramidase-2 precursor
bin008	SOY3_bin008_02162	2508	27	76	105	1.287	3.074	4.447	Copper-exporting P-type ATPase A
bin008	SOY3_bin008_02163	360	8	34	45	2.657	9.579	13.278	hypothetical protein
bin008	SOY3_bin008_02164	78	0	0	0	0.000	0.000	0.000	tRNA-Pro(cgg)
bin008	SOY3_bin008_02165	1092	150	263	370	16.422	24.428	35.992	Chemotaxis response regulator protein-glutamate methylesterase of group 1 operon
bin008	SOY3_bin008_02166	1959	237	404	530	14.463	20.917	28.739	hypothetical protein
bin008	SOY3_bin008_02167	879	67	142	158	9.112	16.385	19.094	Chemotaxis protein methyltransferase Cher2
bin008	SOY3_bin008_02168	765	62	108	142	9.689	14.319	19.718	Sporulation initiation inhibitor protein Soj

bin008	SOY3_bin008_02169	744	58	135	179	9.320	18.404	25.557	Chemotaxis protein CheW
bin008	SOY3_bin008_02170	369	23	71	86	7.452	19.516	24.757	hypothetical protein
bin008	SOY3_bin008_02171	2934	158	409	448	6.438	14.139	16.220	Chemotaxis protein CheA
bin008	SOY3_bin008_02172	708	6	21	18	1.013	3.008	2.701	thiol:disulfide interchange protein precursor
bin008	SOY3_bin008_02173	363	3	2	2	0.988	0.559	0.585	Thioredoxin
bin008	SOY3_bin008_02174	234	7	4	5	3.576	1.734	2.270	Arsenical resistance operon trans-acting repressor ArsD
bin008	SOY3_bin008_02175	1089	4	3	5	0.439	0.279	0.488	putative permease
bin008	SOY3_bin008_02176	384	1	0	0	0.311	0.000	0.000	DGC domain protein
bin008	SOY3_bin008_02177	1011	689	1123	1717	81.473	112.664	180.405	hypothetical protein
bin008	SOY3_bin008_02178	624	6	5	14	1.150	0.813	2.383	hypothetical protein
bin008	SOY3_bin008_02179	1206	838	768	1324	83.070	64.591	116.619	Long-chain primary alcohol dehydrogenase AdhA
bin008	SOY3_bin008_02180	2730	758	552	971	33.193	20.508	37.782	Aldehyde oxidoreductase
bin008	SOY3_bin008_02181	1956	84	88	146	5.134	4.563	7.929	Acetoin dehydrogenase operon transcriptional activator AcoR
bin008	SOY3_bin008_02182	522	2	10	10	0.458	1.943	2.035	Bacterial SH3 domain protein
bin008	SOY3_bin008_02183	549	2	6	7	0.436	1.108	1.354	hypothetical protein
bin008	SOY3_bin008_02184	2691	16	51	51	0.711	1.922	2.013	Phosphoserine phosphatase RsbU
bin008	SOY3_bin008_02185	1299	20	22	37	1.841	1.718	3.026	Transcriptional regulatory protein ZraR
bin008	SOY3_bin008_02186	1944	10	36	35	0.615	1.878	1.913	Phosphoserine phosphatase RsbU
bin008	SOY3_bin008_02187	345	19	28	35	6.584	8.232	10.777	Putative anti-sigma factor antagonist BtrV
bin008	SOY3_bin008_02188	762	5	60	68	0.784	7.986	9.479	hypothetical protein
bin008	SOY3_bin008_02189	348	2	3	7	0.687	0.874	2.137	Development-specific protein S
bin008	SOY3_bin008_02190	417	6	25	19	1.720	6.081	4.840	Inhibitor of g-type lysozyme precursor
bin008	SOY3_bin008_02191	1656	11	51	51	0.794	3.124	3.271	Methyl-accepting chemotaxis protein PctC
bin008	SOY3_bin008_02192	1944	12	69	74	0.738	3.600	4.044	Methionine--tRNA ligase
bin008	SOY3_bin008_02193	1077	22	86	93	2.442	8.099	9.173	hypothetical protein
bin008	SOY3_bin008_02194	76	0	0	0	0.000	0.000	0.000	tRNA-Lys(ctt)
bin008	SOY3_bin008_02195	561	3	41	41	0.639	7.413	7.763	DNA-invertase hin
bin008	SOY3_bin008_02196	849	20	86	120	2.816	10.274	15.014	2-O-methyltransferase NoeI
bin008	SOY3_bin008_02197	924	16	60	50	2.070	6.586	5.748	Putative teichuronic acid biosynthesis glycosyltransferase TuaC
bin008	SOY3_bin008_02198	1803	18	105	113	1.193	5.907	6.658	Capsule polysaccharide biosynthesis protein
bin008	SOY3_bin008_02199	633	6	38	40	1.133	6.089	6.713	2,3-bisphosphoglycerate-dependent phosphoglycerate mutase
bin008	SOY3_bin008_02200	813	8	60	69	1.176	7.485	9.015	3-deoxy-manno-octulosonate cytidylyltransferase
bin008	SOY3_bin008_02201	1113	2	30	23	0.215	2.734	2.195	Glycosyl transferases group 1
bin008	SOY3_bin008_02202	3138	22	135	116	0.838	4.364	3.927	Glycosyltransferase Gtf1
bin008	SOY3_bin008_02203	849	19	121	110	2.675	14.455	13.763	2-dehydro-3-deoxyphosphooctonate aldolase
bin008	SOY3_bin008_02204	1029	21	328	330	2.440	32.331	34.067	Arabinose 5-phosphate isomerase KdsD
bin008	SOY3_bin008_02205	1092	42	247	281	4.598	22.942	27.335	GDP-mannose 4,6-dehydratase
bin008	SOY3_bin008_02206	981	27	104	147	3.290	10.753	15.918	GDP-mannose 4,6-dehydratase
bin008	SOY3_bin008_02207	1191	48	86	116	4.818	7.324	10.346	hypothetical protein
bin008	SOY3_bin008_02208	960	14	35	67	1.743	3.698	7.414	S-adenosyl-L-methionine-dependent 2-deoxy-scyllo-inosamine dehydrogenase
bin008	SOY3_bin008_02209	672	18	63	74	3.202	9.509	11.697	Polysialic acid transport ATP-binding protein KpsT
bin008	SOY3_bin008_02210	813	13	56	58	1.912	6.986	7.578	ABC-2 type transporter
bin008	SOY3_bin008_02211	1215	72	121	164	7.084	10.101	14.338	Chain length determinant protein
bin008	SOY3_bin008_02212	810	168	198	275	24.795	24.793	36.064	putative oxidoreductase
bin008	SOY3_bin008_02213	927	5	36	42	0.645	3.939	4.813	hypothetical protein
bin008	SOY3_bin008_02214	789	9	41	45	1.364	5.271	6.059	Putative glycosyltransferase EpsH
bin008	SOY3_bin008_02215	1410	4	64	66	0.339	4.604	4.972	hypothetical protein
bin008	SOY3_bin008_02216	753	3	98	106	0.476	13.200	14.953	Sulfotransferase domain protein
bin008	SOY3_bin008_02217	990	7	66	75	0.845	6.762	8.047	Ubiquinone biosynthesis O-methyltransferase
bin008	SOY3_bin008_02218	786	7	96	79	1.065	12.388	10.677	3-deoxy-manno-octulosonate cytidylyltransferase
bin008	SOY3_bin008_02219	777	10	121	111	1.539	15.795	15.175	4-hydroxy-2-oxo-heptane-1,7-dioate aldolase
bin008	SOY3_bin008_02220	732	29	79	116	4.736	10.946	16.834	3-deoxy-manno-octulosonate cytidylyltransferase
bin008	SOY3_bin008_02221	834	13	30	46	1.863	3.648	5.859	hypothetical protein
bin008	SOY3_bin008_02222	579	9	52	66	1.858	9.109	12.109	Chromosome partition protein Smc
bin008	SOY3_bin008_02223	3516	78	596	776	2.652	17.193	23.445	chromosome segregation protein
bin008	SOY3_bin008_02224	2019	53	94	128	3.138	4.722	6.734	Capsule polysaccharide biosynthesis protein
bin008	SOY3_bin008_02225	1140	27	45	38	2.831	4.004	3.541	UDP-galactopyranose mutase precursor
bin008	SOY3_bin008_02226	492	32	35	32	7.776	7.215	6.909	hypothetical protein
bin008	SOY3_bin008_02227	738	12	18	13	1.944	2.474	1.871	hypothetical protein
bin008	SOY3_bin008_02228	795	71	95	128	10.677	12.120	17.103	Chromosome-partitioning ATPase Soj
bin008	SOY3_bin008_02229	1530	8	13	17	0.625	0.862	1.180	Lipoteichoic acid synthase 1
bin008	SOY3_bin008_02230	657	36	134	193	6.551	20.687	31.205	Phosphoglycolate phosphatase
bin008	SOY3_bin008_02231	933	62	270	319	7.944	29.352	36.319	Glycerate dehydrogenase
bin008	SOY3_bin008_02232	879	42	170	184	5.712	19.616	22.236	dTDP-glucose 4,6-dehydratase
bin008	SOY3_bin008_02233	1200	69	161	205	6.874	13.608	18.147	Polysaccharide biosynthesis/export protein
bin008	SOY3_bin008_02234	7668	675	1830	2174	10.524	24.206	30.117	Erythronolide synthase, modules 3 and 4
bin008	SOY3_bin008_02235	1386	48	122	141	4.140	8.928	10.807	Putative pyridoxal phosphate-dependent acyltransferase

bin008	SOY3_bin008_02236	1371	37	64	93	3.226	4.735	7.206	Capsule polysaccharide biosynthesis protein
bin008	SOY3_bin008_02237	612	9	47	69	1.758	7.789	11.976	Protease synthase and sporulation negative regulatory protein PAI 1
bin008	SOY3_bin008_02238	927	33	76	81	4.256	8.316	9.282	hypothetical protein
bin008	SOY3_bin008_02239	360	0	14	4	0.000	3.944	1.180	hypothetical protein
bin008	SOY3_bin008_02240	1542	15	12	20	1.163	0.789	1.378	putative HTH-type transcriptional regulator YjiR
bin008	SOY3_bin008_02241	339	10	37	63	3.527	11.070	19.741	Anti-sigma F factor antagonist
bin008	SOY3_bin008_02242	1575	31	67	80	2.353	4.315	5.396	Cyclic di-GMP phosphodiesterase response regulator RpfG
bin008	SOY3_bin008_02243	897	11	31	18	1.466	3.505	2.132	Ribosomal RNA large subunit methyltransferase M
bin008	SOY3_bin008_02244	1635	180	351	471	13.161	21.774	30.601	Autoinducer 2 sensor kinase/phosphatase LuxQ
bin008	SOY3_bin008_02245	3111	5	65	54	0.192	2.119	1.844	Multidrug resistance protein MdtC
bin008	SOY3_bin008_02246	3120	21	71	89	0.805	2.308	3.030	Multidrug resistance protein MdtB
bin008	SOY3_bin008_02247	1173	6	31	46	0.612	2.681	4.166	Multidrug resistance protein MdtA precursor
bin008	SOY3_bin008_02248	204	2	8	11	1.172	3.978	5.728	hypothetical protein
bin008	SOY3_bin008_02249	618	2	23	24	0.387	3.775	4.125	recombination associated protein
bin008	SOY3_bin008_02250	528	1	28	16	0.226	5.379	3.219	hypothetical protein
bin008	SOY3_bin008_02251	753	2	14	12	0.318	1.886	1.693	putative tRNA/rRNA methyltransferase
bin008	SOY3_bin008_02252	1131	1	36	50	0.106	3.228	4.696	Ferredoxin-2
bin008	SOY3_bin008_02253	465	0	7	10	0.000	1.527	2.284	hypothetical protein
bin008	SOY3_bin008_02254	717	1	4	10	0.167	0.566	1.482	putative membrane protein YdfK
bin008	SOY3_bin008_02255	2625	34	127	160	1.548	4.907	6.475	cytochrome c nitrite reductase pentaheme subunit
bin008	SOY3_bin008_02256	1086	22	46	51	2.422	4.296	4.989	hypothetical protein
bin008	SOY3_bin008_02257	186	0	9	3	0.000	4.908	1.713	hypothetical protein
bin008	SOY3_bin008_02258	720	3	17	17	0.498	2.395	2.508	Ubiquinone/menaquinone biosynthesis C-methyltransferase UbiE
bin008	SOY3_bin008_02259	207	2	10	7	1.155	4.900	3.592	hypothetical protein
bin008	SOY3_bin008_02260	1314	13	91	69	1.183	7.024	5.578	UDP-N-acetyl-D-glucosamine 6-dehydrogenase
bin008	SOY3_bin008_02261	699	1	11	8	0.171	1.596	1.216	Futalosine hydrolase
bin008	SOY3_bin008_02262	858	0	8	15	0.000	0.946	1.857	1,4-dihydroxy-6-naphtoate synthase
bin008	SOY3_bin008_02263	969	21	62	89	2.591	6.490	9.757	Octaprenyl-diphosphate synthase
bin008	SOY3_bin008_02264	2997	61	242	273	2.433	8.190	9.676	Phosphoribosylformylglycinamidine synthase 2
bin008	SOY3_bin008_02265	492	38	148	154	9.233	30.511	33.250	Perchlorate reductase subunit gamma precursor
bin008	SOY3_bin008_02266	159	53	184	223	39.850	117.375	148.983	Rubredoxin
bin008	SOY3_bin008_02267	1206	177	742	828	17.546	62.404	72.931	Rubredoxin-oxygen oxidoreductase
bin008	SOY3_bin008_02268	351	10	80	117	3.406	23.117	35.409	hypothetical protein
bin008	SOY3_bin008_02269	606	33	230	269	6.510	38.496	47.153	hypothetical protein
bin008	SOY3_bin008_02270	1554	23	44	65	1.769	2.872	4.443	Lipopolysaccharide core heptosyltransferase RfaQ
bin008	SOY3_bin008_02271	1236	5	16	29	0.484	1.313	2.492	putative diguanylate cyclase YdaM
bin008	SOY3_bin008_02272	1704	7	31	41	0.491	1.845	2.556	putative lipoprotein YbbD precursor
bin008	SOY3_bin008_02273	2130	13	120	114	0.730	5.714	5.685	Methyl-accepting chemotaxis protein PctA
bin008	SOY3_bin008_02274	1029	3	11	17	0.349	1.084	1.755	Putative teichuronic acid biosynthesis glycosyltransferase TuaH
bin008	SOY3_bin008_02275	1737	10	24	34	0.688	1.401	2.079	N-glycosyltransferase
bin008	SOY3_bin008_02276	2049	51	122	161	2.976	6.039	8.347	putative sensor histidine kinase pdtaS
bin008	SOY3_bin008_02277	1092	76	146	175	8.320	13.561	17.023	hypothetical protein
bin008	SOY3_bin008_02278	2391	280	331	441	14.000	14.041	19.592	Primosomal protein N'
bin008	SOY3_bin008_02279	873	184	657	825	25.197	76.332	100.385	UTP--glucose-1-phosphate uridylyltransferase
bin008	SOY3_bin008_02280	1356	14	143	120	1.234	10.696	9.401	Phosphoglucosamine mutase
bin008	SOY3_bin008_02281	897	5	27	35	0.666	3.053	4.145	YbbR-like protein
bin008	SOY3_bin008_02282	771	3	24	19	0.465	3.157	2.618	DNA integrity scanning protein DisA
bin008	SOY3_bin008_02283	861	33	69	99	4.582	8.128	12.214	Dihydropteroate synthase
bin008	SOY3_bin008_02284	1983	81	290	324	4.883	14.833	17.356	ATP-dependent zinc metalloprotease FtsH
bin008	SOY3_bin008_02285	366	8	25	49	2.613	6.928	14.221	Chemotaxis protein CheY
bin008	SOY3_bin008_02286	1392	5	41	46	0.429	2.987	3.510	Cyclic di-GMP phosphodiesterase response regulator RpfG
bin008	SOY3_bin008_02287	651	1	1	2	0.184	0.156	0.326	Global nitrogen regulator
bin008	SOY3_bin008_02288	600	0	2	3	0.000	0.338	0.531	Prismane/CO dehydrogenase family protein
bin008	SOY3_bin008_02289	282	1	4	4	0.424	1.439	1.507	Anaerobic dimethyl sulfoxide reductase chain B
bin008	SOY3_bin008_02290	750	3	14	15	0.478	1.893	2.125	hypothetical protein
bin008	SOY3_bin008_02291	1359	6	15	17	0.528	1.120	1.329	Transcriptional regulatory protein ZraR
bin008	SOY3_bin008_02292	111	22	103	109	23.694	94.117	104.312	hypothetical protein
bin008	SOY3_bin008_02293	417	1	7	6	0.287	1.703	1.528	hypothetical protein
bin008	SOY3_bin008_02294	150	0	7	1	0.000	4.733	0.708	hypothetical protein
bin008	SOY3_bin008_02295	1866	3	43	34	0.192	2.337	1.936	Decarbamoylnovobiocin carbamoyltransferase
bin008	SOY3_bin008_02296	1248	8	24	22	0.766	1.951	1.873	Uracil permease
bin008	SOY3_bin008_02297	627	12	17	13	2.288	2.750	2.202	Uracil phosphoribosyltransferase
bin008	SOY3_bin008_02298	2274	196	301	336	10.304	13.426	15.696	Methyl-accepting chemotaxis protein PctC
bin008	SOY3_bin008_02299	249	32	417	426	15.364	169.860	181.736	periplasmic repressor CpxP
bin008	SOY3_bin008_02300	1287	4	14	13	0.372	1.103	1.073	Divalent metal cation transporter MntH
bin008	SOY3_bin008_02301	471	2	5	4	0.508	1.077	0.902	putative HTH-type transcriptional regulator/GBAA_1941/BAS1801
bin008	SOY3_bin008_02302	1209	4	9	8	0.396	0.755	0.703	putative multidrug resistance protein EmrK

bin008	SOY3_bin008_02303	1557	4	16	6	0.307	1.042	0.409	Multidrug export protein EmrB
bin008	SOY3_bin008_02304	711	0	8	13	0.000	1.141	1.942	hypothetical protein
bin008	SOY3_bin008_02305	411	10	45	49	2.909	11.105	12.664	Response regulator MprA
bin008	SOY3_bin008_02306	1026	15	66	81	1.748	6.525	8.386	Electron transfer flavoprotein subunit alpha
bin008	SOY3_bin008_02307	801	11	72	66	1.642	9.117	8.753	Electron transfer flavoprotein domain protein
bin008	SOY3_bin008_02308	801	12	68	73	1.791	8.611	9.681	Electron transfer flavoprotein domain protein
bin008	SOY3_bin008_02309	1026	15	61	72	1.748	6.030	7.454	Electron transfer flavoprotein subunit alpha
bin008	SOY3_bin008_02310	411	10	39	51	2.909	9.624	13.181	Response regulator MprA
bin008	SOY3_bin008_02311	711	1	6	10	0.168	0.856	1.494	hypothetical protein
bin008	SOY3_bin008_02312	1557	2	10	6	0.154	0.651	0.409	Multidrug export protein EmrB
bin008	SOY3_bin008_02313	1209	2	6	11	0.198	0.503	0.966	putative multidrug resistance protein EmrK
bin008	SOY3_bin008_02314	471	3	11	6	0.761	2.369	1.353	putative HTH-type transcriptional regulator/GBAA_1941/BAS1801
bin008	SOY3_bin008_02315	1287	3	15	15	0.279	1.182	1.238	Divalent metal cation transporter MntH
bin008	SOY3_bin008_02316	249	27	429	437	12.963	174.748	186.428	periplasmic repressor CpxP
bin008	SOY3_bin008_02317	2274	186	290	322	9.778	12.935	15.042	Methyl-accepting chemotaxis protein PctC
bin008	SOY3_bin008_02318	627	11	6	16	2.097	0.971	2.711	Uracil phosphoribosyltransferase
bin008	SOY3_bin008_02319	1248	11	21	19	1.054	1.707	1.617	Uracil permease
bin008	SOY3_bin008_02320	1866	7	54	34	0.448	2.935	1.936	Decarbamoylnovobiocin carbamoyltransferase
bin008	SOY3_bin008_02321	150	1	2	2	0.797	1.352	1.416	hypothetical protein
bin008	SOY3_bin008_02322	417	1	20	6	0.287	4.865	1.528	hypothetical protein
bin008	SOY3_bin008_02323	111	22	79	113	23.694	72.187	108.140	hypothetical protein
bin008	SOY3_bin008_02324	1359	7	10	17	0.616	0.746	1.329	Transcriptional regulatory protein ZraR
bin008	SOY3_bin008_02325	750	5	21	21	0.797	2.840	2.974	hypothetical protein
bin008	SOY3_bin008_02326	507	13	15	35	3.065	3.001	7.333	Anaerobic dimethyl sulfoxide reductase chain B
bin008	SOY3_bin008_02327	549	9	15	29	1.960	2.771	5.611	NADH-quinone oxidoreductase subunit I
bin008	SOY3_bin008_02328	2112	86	154	201	4.868	7.396	10.110	Polysulfide reductase chain A precursor
bin008	SOY3_bin008_02329	465	96	198	248	24.681	43.188	56.654	Organic hydroperoxide resistance transcriptional regulator
bin008	SOY3_bin008_02330	1644	320	453	599	23.270	27.948	38.704	Molybdopterin molybdenumtransferase
bin008	SOY3_bin008_02331	774	56	87	120	8.650	11.401	16.469	Soluble lytic murein transglycosylase precursor
bin008	SOY3_bin008_02332	2259	38	142	172	2.011	6.376	8.088	Alpha-amylase 1
bin008	SOY3_bin008_02333	1482	17	30	43	1.371	2.053	3.082	hypothetical protein
bin008	SOY3_bin008_02334	1167	13	58	58	1.332	5.041	5.279	HAMP domain protein
bin008	SOY3_bin008_02335	765	3	5	8	0.469	0.663	1.111	Phosphate import ATP-binding protein PstB
bin008	SOY3_bin008_02336	1425	47	49	50	3.943	3.488	3.727	Alkaline phosphatase synthesis sensor protein PhoR
bin008	SOY3_bin008_02337	684	11	28	13	1.923	4.152	2.019	Transcriptional regulatory protein SrrA
bin008	SOY3_bin008_02338	2514	188	526	647	8.940	21.221	27.338	Sensor protein SrrB
bin008	SOY3_bin008_02339	1905	213	571	762	13.367	30.402	42.490	hypothetical protein
bin008	SOY3_bin008_02340	387	495	2834	3737	152.911	742.752	1025.751	Cytochrome c3
bin008	SOY3_bin008_02341	765	13	22	37	2.032	2.917	5.138	Cobalt-precorrin-3B C(17)-methyltransferase
bin008	SOY3_bin008_02342	1035	7	40	66	0.809	3.920	6.774	cobalamin biosynthesis protein CbiG
bin008	SOY3_bin008_02343	630	17	35	37	3.226	5.635	6.239	Tetratricopeptide repeat protein
bin008	SOY3_bin008_02344	399	6	14	21	1.798	3.559	5.591	hypothetical protein
bin008	SOY3_bin008_02345	77	0	1	4	0.000	1.317	5.518	tRNA-Arg(tcg)
bin008	SOY3_bin008_02346	321	5	8	5	1.862	2.528	1.655	Hpt domain protein
bin008	SOY3_bin008_02347	1782	28	69	68	1.878	3.927	4.054	H(+)/Cl(-) exchange transporter ClcA
bin008	SOY3_bin008_02348	504	4	16	23	0.949	3.220	4.848	DinB family protein
bin008	SOY3_bin008_02349	1929	47	73	97	2.913	3.838	5.342	Acetyl-coenzyme A synthetase
bin008	SOY3_bin008_02350	882	27	56	64	3.660	6.440	7.708	Sensory transduction protein LytR
bin008	SOY3_bin008_02351	1209	0	22	10	0.000	1.846	0.879	Twitching mobility protein
bin008	SOY3_bin008_02352	1077	3	4	8	0.333	0.377	0.789	Twitching mobility protein
bin008	SOY3_bin008_02353	186	4	13	14	2.571	7.089	7.995	Helix-turn-helix domain protein
bin008	SOY3_bin008_02354	1641	21	49	68	1.530	3.029	4.402	Competence protein A
bin008	SOY3_bin008_02355	534	2	9	14	0.448	1.709	2.785	Pilus assembly protein, PilO
bin008	SOY3_bin008_02356	486	8	15	13	1.968	3.130	2.841	hypothetical protein
bin008	SOY3_bin008_02357	1803	12	43	60	0.796	2.419	3.535	Type IV pilus biogenesis and competence protein PilQ precursor
bin008	SOY3_bin008_02358	2130	22	135	168	1.235	6.428	8.378	Archaeal ATPase
bin008	SOY3_bin008_02359	1710	18	51	66	1.258	3.025	4.100	Type II secretion system protein E
bin008	SOY3_bin008_02360	1224	4	16	22	0.391	1.326	1.909	Type II secretion system protein F
bin008	SOY3_bin008_02361	1347	8	23	44	0.710	1.732	3.470	Nitrogen fixation protein VnfA
bin008	SOY3_bin008_02362	2154	1	23	25	0.056	1.083	1.233	TPR repeat-containing protein YrrB
bin008	SOY3_bin008_02363	534	1	1	4	0.224	0.190	0.796	hypothetical protein
bin008	SOY3_bin008_02364	432	1	0	3	0.277	0.000	0.738	hypothetical protein
bin008	SOY3_bin008_02365	1467	5	6	7	0.407	0.415	0.507	Legume lectin domain protein
bin008	SOY3_bin008_02366	558	2	6	9	0.428	1.091	1.713	Soluble lytic murein transglycosylase precursor
bin008	SOY3_bin008_02367	1215	19	46	53	1.869	3.840	4.634	Acyltransferase family protein
bin008	SOY3_bin008_02368	1392	46	72	94	3.951	5.246	7.173	NADH-quinone oxidoreductase subunit N
bin008	SOY3_bin008_02369	1500	69	101	120	5.499	6.829	8.498	NADH-quinone oxidoreductase subunit M

bin008	SOY3_bin008_02370	1866	79	106	159	5.061	5.762	9.051	NADH-quinone oxidoreductase subunit L
bin008	SOY3_bin008_02371	306	12	24	24	4.688	7.955	8.331	NADH-quinone oxidoreductase subunit K
bin008	SOY3_bin008_02372	522	28	33	49	6.413	6.412	9.971	NADH-quinone oxidoreductase subunit J
bin008	SOY3_bin008_02373	555	20	33	44	4.308	6.031	8.422	NADH-quinone oxidoreductase subunit I
bin008	SOY3_bin008_02374	990	26	51	55	3.140	5.225	5.901	NADH-quinone oxidoreductase subunit H
bin008	SOY3_bin008_02375	2316	111	156	202	5.730	6.832	9.265	NADH-quinone oxidoreductase subunit G
bin008	SOY3_bin008_02376	1365	52	80	108	4.554	5.944	8.405	NADH-quinone oxidoreductase subunit F
bin008	SOY3_bin008_02377	501	10	39	29	2.386	7.896	6.149	NADH-quinone oxidoreductase subunit E
bin008	SOY3_bin008_02378	228	69	66	100	36.179	29.361	46.590	hypothetical protein
bin008	SOY3_bin008_02379	2373	150	268	285	7.557	11.455	12.758	NADH-quinone oxidoreductase subunit C/D
bin008	SOY3_bin008_02380	420	44	81	96	12.524	19.561	24.280	NAD(P)H-quinone oxidoreductase subunit 3
bin008	SOY3_bin008_02381	1206	21	37	44	2.082	3.112	3.876	L-2-hydroxyglutarate oxidase LhgO
bin008	SOY3_bin008_02382	2349	4	6	9	0.204	0.259	0.407	Cyclic di-GMP phosphodiesterase YfgF
bin008	SOY3_bin008_02383	759	27	90	80	4.253	12.027	11.196	putative phospholipid import ATP-binding protein MlaF
bin008	SOY3_bin008_02384	762	5	33	27	0.784	4.393	3.764	Alpha-acetolactate decarboxylase precursor
bin008	SOY3_bin008_02385	453	9	23	19	2.375	5.150	4.455	hypothetical protein
bin008	SOY3_bin008_02386	1032	6	20	15	0.695	1.966	1.544	Poly-beta-1,6-N-acetyl-D-glucosamine N-deacetylase precursor
bin008	SOY3_bin008_02387	1170	10	37	50	1.022	3.208	4.540	hypothetical protein
bin008	SOY3_bin008_02388	1095	22	83	81	2.402	7.688	7.858	hypothetical protein
bin008	SOY3_bin008_02389	285	17	84	87	7.131	29.894	32.427	hypothetical protein
bin008	SOY3_bin008_02390	582	1056	2588	3258	216.913	451.020	594.645	hypothetical protein
bin008	SOY3_bin008_02391	372	37	283	328	11.891	77.161	93.661	30S ribosomal protein S12
bin008	SOY3_bin008_02392	471	41	215	207	10.407	46.299	46.685	30S ribosomal protein S7
bin008	SOY3_bin008_02393	2073	80	581	614	4.614	28.427	31.463	Elongation factor G
bin008	SOY3_bin008_02394	825	0	1	2	0.000	0.123	0.258	Nitrogenase iron protein 1
bin008	SOY3_bin008_02395	330	0	0	0	0.000	0.000	0.000	Nitrogen regulatory protein P-II
bin008	SOY3_bin008_02396	375	1	3	1	0.319	0.811	0.283	Nitrogen regulatory protein P-II 1
bin008	SOY3_bin008_02397	1626	5	10	9	0.368	0.624	0.588	Nitrogenase molybdenum-iron protein alpha chain
bin008	SOY3_bin008_02398	1386	6	10	11	0.518	0.732	0.843	Nitrogenase molybdenum-iron protein beta chain
bin008	SOY3_bin008_02399	312	0	2	4	0.000	0.650	1.362	Ferredoxin, 2Fe-2S
bin008	SOY3_bin008_02400	735	4	28	25	0.651	3.864	3.613	putative DNA endonuclease SmrA
bin008	SOY3_bin008_02401	1008	44	173	182	5.218	17.408	19.180	Curved DNA-binding protein
bin008	SOY3_bin008_02402	318	23	42	74	8.647	13.396	24.719	Putative heat shock protein HspR
bin008	SOY3_bin008_02403	2601	228	509	570	10.479	19.849	23.279	Chaperone protein ClpB 1
bin008	SOY3_bin008_02404	1650	127	283	407	9.202	17.396	26.202	Alpha-keto-acid decarboxylase
bin008	SOY3_bin008_02405	801	36	79	114	5.373	10.003	15.118	Hydroxyethylthiazole kinase
bin008	SOY3_bin008_02406	642	61	113	154	11.359	17.852	25.481	Thiamine-phosphate synthase
bin008	SOY3_bin008_02407	651	11	37	36	2.020	5.765	5.874	Localization factor PodJL
bin008	SOY3_bin008_02408	1119	4	21	8	0.427	1.903	0.759	pheromone autoinducer 2 transporter
bin008	SOY3_bin008_02409	243	5	11	24	2.460	4.591	10.491	hypothetical protein
bin008	SOY3_bin008_02410	1158	1	10	6	0.103	0.876	0.550	Capreomycidine synthase
bin008	SOY3_bin008_02411	744	79	591	676	12.694	80.569	96.517	ABC transporter arginine-binding protein 1 precursor
bin008	SOY3_bin008_02412	810	11	51	58	1.624	6.386	7.606	L-cystine transport system permease protein TcyB
bin008	SOY3_bin008_02413	741	3	40	44	0.484	5.475	6.308	Glutamine transport ATP-binding protein GlnQ
bin008	SOY3_bin008_02414	618	22	104	100	4.256	17.069	17.189	photosystem I assembly protein Ycf3
bin008	SOY3_bin008_02415	891	10	28	24	1.342	3.187	2.861	putative inner membrane transporter YedA
bin008	SOY3_bin008_02416	918	2	24	19	0.260	2.652	2.199	Phytochrome-like protein cph2
bin008	SOY3_bin008_02417	927	56	437	451	7.222	47.814	51.680	ABC transporter arginine-binding protein 1 precursor
bin008	SOY3_bin008_02418	714	3	26	18	0.502	3.693	2.678	Histidine transport system permease protein HisM
bin008	SOY3_bin008_02419	777	4	19	17	0.615	2.480	2.324	L-cystine import ATP-binding protein TcyC
bin008	SOY3_bin008_02420	693	9	13	14	1.553	1.903	2.146	Glutamine transport system permease protein GlnP
bin008	SOY3_bin008_02421	1368	14	30	36	1.223	2.224	2.795	putative FAD-linked oxidoreductase
bin008	SOY3_bin008_02422	936	1	20	26	0.128	2.167	2.951	2-hydroxy-6-oxo-6-phenylhexa-2,4-dienoate hydrolase
bin008	SOY3_bin008_02423	327	5	25	42	1.828	7.754	13.644	Glutaredoxin-3
bin008	SOY3_bin008_02424	882	17	26	33	2.304	2.990	3.974	EamA-like transporter family protein
bin008	SOY3_bin008_02425	1185	18	35	37	1.816	2.996	3.317	Aspartate aminotransferase
bin008	SOY3_bin008_02426	1278	30	92	122	2.806	7.301	10.140	Putative glycosyltransferase EpsH
bin008	SOY3_bin008_02427	1473	25	25	45	2.029	1.721	3.245	Multidrug resistance protein stp
bin008	SOY3_bin008_02428	489	14	94	97	3.423	19.497	21.071	Multidrug resistance operon repressor
bin008	SOY3_bin008_02429	1986	70	202	223	4.214	10.316	11.928	Glutathione-regulated potassium-efflux system protein KefC
bin008	SOY3_bin008_02430	861	48	194	212	6.665	22.854	26.155	L-lysine cyclodeaminase
bin008	SOY3_bin008_02431	3072	36	156	192	1.401	5.151	6.639	Multidrug resistance protein MdtB
bin008	SOY3_bin008_02432	1224	14	134	134	1.367	11.104	11.629	Multidrug resistance protein MdtA precursor
bin008	SOY3_bin008_02433	1122	10	34	51	1.065	3.074	4.828	Glutamine-binding periplasmic protein precursor
bin008	SOY3_bin008_02434	1299	11	56	79	1.012	4.373	6.460	Murein DD-endopeptidase MepM
bin008	SOY3_bin008_02435	420	19	41	58	5.408	9.901	14.669	hypothetical protein
bin008	SOY3_bin008_02436	588	268	843	1077	54.488	145.414	194.567	General stress protein 18

bin008	SOY3_bin008_02437	2469	0	4	10	0.000	0.164	0.430	Diflavin flavoprotein A 1
bin008	SOY3_bin008_02438	1008	6	44	41	0.712	4.427	4.321	Tryptophan--tRNA ligase
bin008	SOY3_bin008_02439	672	3	10	12	0.534	1.509	1.897	Peptidase family M50
bin008	SOY3_bin008_02440	378	98	307	336	30.994	82.376	94.423	Transcriptional regulatory protein YycF
bin008	SOY3_bin008_02441	198	5	16	28	3.019	8.196	15.022	hypothetical protein
bin008	SOY3_bin008_02442	909	14	25	35	1.841	2.790	4.090	tRNA 2-thiocytidine biosynthesis protein TtcA
bin008	SOY3_bin008_02443	1617	9	49	38	0.665	3.074	2.496	Putative glycosyltransferase EpsH
bin008	SOY3_bin008_02444	1134	7	38	43	0.738	3.399	4.028	Soluble hydrogenase 42 kDa subunit
bin008	SOY3_bin008_02445	1014	25	70	58	2.947	7.002	6.076	Phosphotransferase enzyme family protein
bin008	SOY3_bin008_02446	759	2	23	12	0.315	3.074	1.679	Phosphoglycolate phosphatase
bin008	SOY3_bin008_02447	528	6	17	18	1.359	3.266	3.621	hypothetical protein
bin008	SOY3_bin008_02448	2049	14	40	37	0.817	1.980	1.918	Beta-monoglucosyldiacylglycerol synthase
bin008	SOY3_bin008_02449	1860	2	17	15	0.129	0.927	0.857	Adenylate cyclase 1
bin008	SOY3_bin008_02450	669	20	80	91	3.574	12.129	14.449	hypothetical protein
bin008	SOY3_bin008_02451	456	137	223	188	35.917	49.601	43.795	Acid shock protein
bin008	SOY3_bin008_02452	960	11	24	20	1.370	2.536	2.213	hypothetical protein
bin008	SOY3_bin008_02453	639	0	6	7	0.000	0.952	1.164	Alpha-D-glucose-1-phosphate phosphatase YihX
bin008	SOY3_bin008_02454	648	6	16	18	1.107	2.504	2.951	phosphatidylserine decarboxylase
bin008	SOY3_bin008_02455	774	42	156	182	6.487	20.443	24.978	CDP-alcohol phosphatidyltransferase
bin008	SOY3_bin008_02456	1542	15	80	86	1.163	5.262	5.924	2-isopropylmalate synthase
bin008	SOY3_bin008_02457	1260	19	87	97	1.803	7.003	8.178	2,3-dimethylmalate dehydratase large subunit
bin008	SOY3_bin008_02458	498	5	33	40	1.200	6.721	8.532	2,3-dimethylmalate dehydratase small subunit
bin008	SOY3_bin008_02459	1077	18	80	89	1.998	7.534	8.778	3-isopropylmalate dehydrogenase
bin008	SOY3_bin008_02460	1980	6	36	33	0.362	1.844	1.770	putative ABC transporter ATP-binding protein YheS
bin008	SOY3_bin008_02461	414	1	9	9	0.289	2.205	2.309	CTP pyrophosphohydrolase
bin008	SOY3_bin008_02462	873	4	46	54	0.548	5.344	6.571	5,10-methylenetetrahydrofolate reductase
bin008	SOY3_bin008_02463	1035	9	146	173	1.040	14.308	17.756	Aspartate-semialdehyde dehydrogenase
bin008	SOY3_bin008_02464	942	3	22	14	0.381	2.369	1.579	Branched-chain-amino-acid aminotransferase
bin008	SOY3_bin008_02465	1170	19	52	66	1.941	4.508	5.992	Nitronate monooxygenase
bin008	SOY3_bin008_02466	516	1	16	20	0.232	3.145	4.117	NADH-quinone oxidoreductase subunit J
bin008	SOY3_bin008_02467	309	1	10	14	0.387	3.282	4.813	NADH-quinone oxidoreductase subunit K
bin008	SOY3_bin008_02468	1494	20	33	41	1.600	2.240	2.915	Na(+)/H(+) antiporter subunit A
bin008	SOY3_bin008_02469	270	3	11	17	1.328	4.132	6.688	hypothetical protein
bin008	SOY3_bin008_02470	1815	14	49	52	0.922	2.738	3.043	Na(+)/H(+) antiporter subunit D
bin008	SOY3_bin008_02471	1575	13	35	41	0.987	2.254	2.765	NAD(P)H-quinone oxidoreductase chain 4 1
bin008	SOY3_bin008_02472	1404	11	28	38	0.937	2.023	2.875	NADH-quinone oxidoreductase subunit N
bin008	SOY3_bin008_02473	393	4	15	11	1.217	3.871	2.973	NAD(P)H-quinone oxidoreductase subunit 3
bin008	SOY3_bin008_02474	531	6	20	21	1.351	3.820	4.201	NADH-quinone oxidoreductase subunit 6
bin008	SOY3_bin008_02475	537	2	11	17	0.445	2.078	3.363	NAD(P)H-quinone oxidoreductase subunit J
bin008	SOY3_bin008_02476	1158	5	23	40	0.516	2.015	3.669	NADH-quinone oxidoreductase subunit 4
bin008	SOY3_bin008_02477	987	6	16	21	0.727	1.644	2.260	NADH-quinone oxidoreductase subunit H
bin008	SOY3_bin008_02478	507	4	8	7	0.943	1.600	1.467	NAD(P)H-quinone oxidoreductase subunit I
bin008	SOY3_bin008_02479	561	42	171	159	8.950	30.916	30.107	fused phosphoenolpyruvate-protein phosphotransferase PtsP/GAF domain protein
bin008	SOY3_bin008_02480	1017	61	257	303	7.171	25.631	31.648	Glyceraldehyde-3-phosphate dehydrogenase
bin008	SOY3_bin008_02481	1695	139	213	288	9.804	12.746	18.049	Alkaline phosphatase synthesis sensor protein PhoR
bin008	SOY3_bin008_02482	906	134	194	250	17.682	21.718	29.312	hypothetical protein
bin008	SOY3_bin008_02483	1458	42	76	91	3.444	5.287	6.630	hypothetical protein
bin008	SOY3_bin008_02484	975	21	34	24	2.575	3.537	2.615	hypothetical protein
bin008	SOY3_bin008_02485	765	25	42	52	3.907	5.569	7.221	hypothetical protein
bin008	SOY3_bin008_02486	843	7	7	15	0.993	0.842	1.890	Protein-glutamine gamma-glutamyltransferase
bin008	SOY3_bin008_02487	3204	32	96	119	1.194	3.039	3.945	Gliding motility regulatory protein
bin008	SOY3_bin008_02488	522	11	23	26	2.519	4.469	5.291	CheC-like family protein
bin008	SOY3_bin008_02489	378	11	23	29	3.479	6.172	8.150	Chemotaxis protein CheY
bin008	SOY3_bin008_02490	1152	14	30	43	1.453	2.641	3.965	Alginate biosynthesis sensor protein KinB
bin008	SOY3_bin008_02491	900	8	11	17	1.063	1.240	2.006	putative diguanylate cyclase YdaM
bin008	SOY3_bin008_02492	555	12	147	151	2.585	26.865	28.901	Inner membrane protein YaaH
bin008	SOY3_bin008_02493	1146	16	41	55	1.669	3.629	5.098	3-dehydroquinate synthase
bin008	SOY3_bin008_02494	1110	98	111	120	10.555	10.143	11.484	Xylose isomerase-like TIM barrel
bin008	SOY3_bin008_02495	483	100	140	199	24.751	29.399	43.766	Transcriptional regulator SlyA
bin008	SOY3_bin008_02496	210	47	115	142	26.756	55.544	71.829	hypothetical protein
bin008	SOY3_bin008_02497	177	38	90	102	25.666	51.573	61.215	hypothetical protein
bin008	SOY3_bin008_02498	693	1	2	2	0.173	0.293	0.307	hypothetical protein
bin008	SOY3_bin008_02499	1506	1	1	2	0.079	0.067	0.141	DNA primase
bin008	SOY3_bin008_02500	345	0	0	2	0.000	0.000	0.616	hypothetical protein
bin008	SOY3_bin008_02501	387	0	1	0	0.000	0.262	0.000	Transposase zinc-ribbon domain protein
bin008	SOY3_bin008_02502	345	0	0	2	0.000	0.000	0.616	hypothetical protein
bin008	SOY3_bin008_02503	693	9	19	18	1.553	2.781	2.759	Phage regulatory protein Rha (Phage_pRha)

bin008	SOY3_bin008_02504	2856	24	114	120	1.005	4.049	4.463	Secreted effector protein PipB
bin008	SOY3_bin008_02505	90	6	7	19	7.970	7.889	22.425	tRNA-Ser(tga)
bin008	SOY3_bin008_02506	966	284	427	506	35.147	44.834	55.642	Chemotaxis protein CheV
bin008	SOY3_bin008_02507	1299	21	103	93	1.933	8.042	7.605	Alpha-D-kanosaminyltransferase
bin008	SOY3_bin008_02508	1911	7	59	54	0.438	3.131	3.002	Asparagine synthetase [glutamine-hydrolyzing] 1
bin008	SOY3_bin008_02509	1194	7	6	20	0.701	0.510	1.779	Major Facilitator Superfamily protein
bin008	SOY3_bin008_02510	306	9	19	29	3.516	6.298	10.067	hypothetical protein
bin008	SOY3_bin008_02511	876	113	416	546	15.421	48.166	66.209	putative transaldolase
bin008	SOY3_bin008_02512	441	6	11	22	1.627	2.530	5.299	Low molecular weight protein-tyrosine-phosphatase ptp
bin008	SOY3_bin008_02513	1182	5206	52736	59875	526.541	4525.270	5380.937	Alcohol dehydrogenase 2
bin008	SOY3_bin008_02514	561	35	244	269	7.458	44.115	50.935	succinate dehydrogenase/fumarate reductase iron-sulfur subunit
bin008	SOY3_bin008_02515	900	40	209	255	5.313	23.554	30.097	succinate dehydrogenase/fumarate reductase iron-sulfur subunit
bin008	SOY3_bin008_02516	1959	78	417	434	4.760	21.590	23.533	NADH-dependent phenylglyoxylate dehydrogenase subunit delta
bin008	SOY3_bin008_02517	489	23	92	100	5.623	19.082	21.723	Methyl-viologen-reducing hydrogenase, delta subunit
bin008	SOY3_bin008_02518	972	39	198	182	4.797	20.661	19.890	succinate dehydrogenase/fumarate reductase iron-sulfur subunit
bin008	SOY3_bin008_02519	1050	36	209	184	4.099	20.189	18.615	Anaerobic sulfite reductase subunit A
bin008	SOY3_bin008_02520	837	29	244	285	4.142	29.568	36.170	Anaerobic sulfite reductase subunit B
bin008	SOY3_bin008_02521	492	22	161	168	5.346	33.191	36.272	putative trifunctional 2-polyprenylphenol hydroxylase/glutamate synthase subunit beta/ferritin domain-containing protein
bin008	SOY3_bin008_02522	852	31	87	104	4.350	10.357	12.967	hypothetical protein
bin008	SOY3_bin008_02523	1356	24	83	110	2.116	6.208	8.617	Sensor protein ZraS
bin008	SOY3_bin008_02524	1332	33	92	122	2.962	7.005	9.729	Transcriptional regulatory protein ZraR
bin008	SOY3_bin008_02525	1023	144	1160	1097	16.828	115.010	113.910	Molybdopterin molybdenumtransferase
bin008	SOY3_bin008_02526	2751	1818	18661	17288	79.004	688.017	667.550	Aldehyde oxidoreductase
bin008	SOY3_bin008_02527	2313	118	659	635	6.099	28.898	29.163	NAD-dependent dihydropyrimidine dehydrogenase subunit PreT
bin008	SOY3_bin008_02528	213	12	135	132	6.735	64.285	65.830	hypothetical protein
bin008	SOY3_bin008_02529	723	35	130	141	5.787	18.237	20.716	Malonyl-[acyl-carrier protein] O-methyltransferase
bin008	SOY3_bin008_02530	438	13	53	60	3.548	12.273	14.551	Putative redox-active protein (C_GCAxxG_C_C)
bin008	SOY3_bin008_02531	1383	36	111	101	3.112	8.141	7.758	Cyclic pyranopterin monophosphate synthase
bin008	SOY3_bin008_02532	1068	6	66	84	0.672	6.268	8.355	Phenylacetate-coenzyme A ligase
bin008	SOY3_bin008_02533	1032	24	110	138	2.780	10.811	14.205	putative xanthine dehydrogenase subunit A
bin008	SOY3_bin008_02534	1239	21	97	93	2.026	7.941	7.973	Molybdenum cofactor cytidylyltransferase
bin008	SOY3_bin008_02535	645	17	80	95	3.151	12.580	15.646	Glucosyl-3-phosphoglycerate phosphatase
bin008	SOY3_bin008_02536	309	12	49	56	4.643	16.084	19.251	hypothetical protein
bin008	SOY3_bin008_02537	1212	23	85	96	2.269	7.113	8.414	Multidrug export protein AcrE precursor
bin008	SOY3_bin008_02538	3249	44	149	167	1.619	4.651	5.460	Multidrug export protein AcrF
bin008	SOY3_bin008_02539	1158	3	22	21	0.310	1.927	1.926	D-alanyl-D-alanine carboxypeptidase DacC precursor
bin008	SOY3_bin008_02540	162	6	8	17	4.428	5.009	11.147	hypothetical protein
bin008	SOY3_bin008_02541	1491	34	145	194	2.726	9.864	13.821	Sensor protein DivL
bin008	SOY3_bin008_02542	450	36	176	207	9.564	39.669	48.864	hypothetical protein
bin008	SOY3_bin008_02543	885	74	163	194	9.996	18.681	23.286	formate dehydrogenase accessory protein FdhE
bin008	SOY3_bin008_02544	711	143	242	362	24.044	34.522	54.084	formate dehydrogenase accessory protein
bin008	SOY3_bin008_02545	612	73	169	205	14.260	28.009	35.582	putative molybdenum cofactor guanylyltransferase
bin008	SOY3_bin008_02546	1005	53	220	253	6.305	22.203	26.741	Cyclic pyranopterin monophosphate synthase
bin008	SOY3_bin008_02547	867	19	55	55	2.620	6.434	6.739	50S ribosomal protein L37Ae
bin008	SOY3_bin008_02548	576	68	134	158	14.113	23.596	29.138	Formate dehydrogenase subunit alpha precursor
bin008	SOY3_bin008_02549	2415	204	334	476	10.099	14.028	20.937	Formate dehydrogenase subunit alpha precursor
bin008	SOY3_bin008_02550	723	74	174	192	12.236	24.410	28.209	Formate dehydrogenase subunit beta
bin008	SOY3_bin008_02551	1314	10	17	23	0.910	1.312	1.859	Leu/Ile/Val/Thr-binding protein precursor
bin008	SOY3_bin008_02552	2019	3	12	10	0.178	0.603	0.526	Sporulation kinase E
bin008	SOY3_bin008_02553	738	11	24	32	1.782	3.298	4.606	Nitrogen regulation protein NR(I)
bin008	SOY3_bin008_02554	324	4	34	42	1.476	10.644	13.770	hypothetical protein
bin008	SOY3_bin008_02555	321	4	38	30	1.490	12.007	9.928	hypothetical protein
bin008	SOY3_bin008_02556	837	3	24	20	0.428	2.908	2.538	Methyltransferase domain protein
bin008	SOY3_bin008_02557	1104	8	28	29	0.866	2.572	2.790	4-hydroxy-3-methylbut-2-en-1-yl diphosphate synthase
bin008	SOY3_bin008_02558	1728	11	62	62	0.761	3.639	3.811	Proline--tRNA ligase
bin008	SOY3_bin008_02559	1410	16	38	25	1.357	2.734	1.883	Exodeoxyribonuclease 7 large subunit
bin008	SOY3_bin008_02560	249	7	25	22	3.361	10.183	9.385	Exodeoxyribonuclease 7 small subunit
bin008	SOY3_bin008_02561	903	11	37	47	1.456	4.156	5.529	Farnesyl diphosphate synthase
bin008	SOY3_bin008_02562	627	7	26	49	1.335	4.206	8.302	NADH dehydrogenase
bin008	SOY3_bin008_02563	1902	42	113	132	2.640	6.026	7.372	1-deoxy-D-xylulose-5-phosphate synthase
bin008	SOY3_bin008_02564	756	8	26	19	1.265	3.488	2.670	Glycerophosphoryl diester phosphodiesterase
bin008	SOY3_bin008_02565	972	16	94	111	1.968	9.809	12.131	deoxyhypusine synthase-like protein
bin008	SOY3_bin008_02566	243	313	922	1105	153.987	384.839	483.043	hypothetical protein
bin008	SOY3_bin008_02567	390	4	48	52	1.226	12.483	14.163	OsmC-like protein
bin008	SOY3_bin008_02568	483	4	54	49	0.990	11.340	10.777	hypothetical protein
bin008	SOY3_bin008_02569	1734	50	147	196	3.447	8.599	12.007	phosphoenolpyruvate carboxykinase
bin008	SOY3_bin008_02570	1587	20	108	95	1.507	6.902	6.359	Nitrogen fixation protein VnfA

bin008	SOY3_bin008_02571	603	27	97	114	5.353	16.316	20.082	indolepyruvate oxidoreductase subunit beta
bin008	SOY3_bin008_02572	1839	52	161	230	3.380	8.880	13.285	Acetolactate synthase isozyme 1 large subunit
bin008	SOY3_bin008_02573	672	13	73	92	2.313	11.018	14.543	cellulose synthase subunit BcsC
bin008	SOY3_bin008_02574	1260	13	92	103	1.233	7.406	8.684	Gamma-glutamyl phosphate reductase
bin008	SOY3_bin008_02575	714	6	31	25	1.005	4.404	3.719	Nicotinate-nucleotide adenylyltransferase
bin008	SOY3_bin008_02576	1068	6	26	28	0.672	2.469	2.785	Hemolysin C
bin008	SOY3_bin008_02577	414	4	15	19	1.155	3.675	4.875	Cytochrome c-type biogenesis protein CcmE
bin008	SOY3_bin008_02578	1902	8	23	28	0.503	1.227	1.564	Cytochrome c-type biogenesis protein CcmF
bin008	SOY3_bin008_02579	672	6	17	20	1.067	2.566	3.161	Zinc import ATP-binding protein ZnuC
bin008	SOY3_bin008_02580	678	3	6	8	0.529	0.898	1.253	CcmB protein
bin008	SOY3_bin008_02581	684	1	13	15	0.175	1.928	2.330	Heme exporter protein C
bin008	SOY3_bin008_02582	141	0	5	3	0.000	3.597	2.260	hypothetical protein
bin008	SOY3_bin008_02583	612	4	20	25	0.781	3.315	4.339	Lipoprotein NlpI precursor
bin008	SOY3_bin008_02584	1173	40	194	217	4.077	16.775	19.651	Leucine-, isoleucine-, valine-, threonine-, and alanine-binding protein precursor
bin008	SOY3_bin008_02585	885	10	31	37	1.351	3.553	4.441	High-affinity branched-chain amino acid transport system permease protein LivH
bin008	SOY3_bin008_02586	1047	16	32	42	1.827	3.100	4.261	leucine/isoleucine/valine transporter permease subunit
bin008	SOY3_bin008_02587	786	3	30	25	0.456	3.871	3.379	Lipopolysaccharide export system ATP-binding protein LptB
bin008	SOY3_bin008_02588	708	6	16	11	1.013	2.292	1.650	High-affinity branched-chain amino acid transport ATP-binding protein LivF
bin008	SOY3_bin008_02589	1461	74	185	236	6.055	12.843	17.159	Inosine-5'-monophosphate dehydrogenase
bin008	SOY3_bin008_02590	1551	78	222	272	6.012	14.518	18.629	GMP synthase [glutamine-hydrolyzing]
bin008	SOY3_bin008_02591	312	35	90	131	13.411	29.258	44.601	Sec-independent protein translocase protein TatAd
bin008	SOY3_bin008_02592	585	43	107	140	8.787	18.552	25.422	Imidazoleglycerol-phosphate dehydratase
bin008	SOY3_bin008_02593	681	52	104	113	9.129	15.490	17.626	hypothetical protein
bin008	SOY3_bin008_02594	744	78	182	201	12.533	24.812	28.698	1-(5-phosphoribosyl)-5-[(5-phosphoribosylamino)methylideneamino] imidazole-4-carboxamide isomerase
bin008	SOY3_bin008_02595	207	26	95	106	15.016	46.549	54.396	Copper-transporting P-type ATPase
bin008	SOY3_bin008_02596	627	12	18	29	2.288	2.912	4.913	Malonyl-[acyl-carrier protein] O-methyltransferase
bin008	SOY3_bin008_02597	1650	4	57	64	0.290	3.504	4.120	Alkaline phosphatase synthesis sensor protein PhoR
bin008	SOY3_bin008_02598	636	2	29	21	0.376	4.625	3.507	Putative pit accessory protein
bin008	SOY3_bin008_02599	1011	34	58	54	4.020	5.819	5.674	Low-affinity inorganic phosphate transporter 1
bin008	SOY3_bin008_02600	1512	262	444	429	20.715	29.784	30.139	Vegetative catalase
bin008	SOY3_bin008_02601	447	0	3	5	0.000	0.681	1.188	Peroxide operon regulator
bin008	SOY3_bin008_02602	675	8	25	24	1.417	3.757	3.777	hypothetical protein
bin008	SOY3_bin008_02603	2037	145	269	342	8.510	13.394	17.835	Methyl-accepting chemotaxis protein McpS
bin008	SOY3_bin008_02604	1179	64	250	363	6.490	21.507	32.706	Multidrug export protein AcrE precursor
bin008	SOY3_bin008_02605	3111	119	328	463	4.573	10.694	15.809	Efflux pump membrane transporter BepE
bin008	SOY3_bin008_02606	504	20	33	53	4.744	6.641	11.171	hypothetical protein
bin008	SOY3_bin008_02607	2082	36	77	115	2.067	3.751	5.867	Signal transduction histidine-protein kinase BarA
bin008	SOY3_bin008_02608	801	94	370	476	14.029	46.852	63.125	hypothetical protein
bin008	SOY3_bin008_02609	3072	140	349	346	5.448	11.523	11.964	Non-motile and phage-resistance protein
bin008	SOY3_bin008_02610	429	1	2	5	0.279	0.473	1.238	hypothetical protein
bin008	SOY3_bin008_02611	3207	131	154	180	4.883	4.871	5.962	Trehalose synthase/amylase TreS
bin008	SOY3_bin008_02612	816	91	108	143	13.332	13.424	18.616	Trehalose-6-phosphate phosphatase
bin008	SOY3_bin008_02613	1509	72	72	113	5.704	4.839	7.955	Trehalose-phosphate synthase
bin008	SOY3_bin008_02614	945	20	18	46	2.530	1.932	5.171	N-acetylmuramoyl-L-alanine amidase XlyA precursor
bin008	SOY3_bin008_02615	960	62	124	171	7.721	13.101	18.921	Chaperone protein DnaJ
bin008	SOY3_bin008_02616	468	17	74	91	4.343	16.038	20.655	CheY-P phosphatase CheX
bin008	SOY3_bin008_02617	2031	94	164	197	5.533	8.190	10.304	Thiol:disulfide interchange protein DsbD precursor
bin008	SOY3_bin008_02618	819	33	64	78	4.817	7.926	10.117	Adenosyl-chloride synthase
bin008	SOY3_bin008_02619	726	33	44	54	5.434	6.147	7.901	Cobalamin synthase
bin008	SOY3_bin008_02620	444	11	81	95	2.962	18.504	22.728	Flagellar FliJ protein
bin008	SOY3_bin008_02621	684	46	92	125	8.040	13.642	19.413	phosphodiesterase
bin008	SOY3_bin008_02622	837	19	38	41	2.714	4.605	5.203	tRNA pseudouridine synthase A
bin008	SOY3_bin008_02623	852	13	31	32	1.824	3.690	3.990	Pantothenate synthetase
bin008	SOY3_bin008_02624	1170	70	486	481	7.152	42.131	43.671	S-adenosylmethionine synthase
bin008	SOY3_bin008_02625	507	25	158	149	5.895	31.608	31.218	hypothetical protein
bin008	SOY3_bin008_02626	348	16	228	243	5.497	66.452	74.175	hypothetical protein
bin008	SOY3_bin008_02627	74	4	13	10	6.462	17.818	14.355	tRNA-Gln(ctg)
bin008	SOY3_bin008_02628	78	7	5	7	10.729	6.502	9.533	tRNA-Glu(ttc)
bin008	SOY3_bin008_02629	1083	33	63	96	3.643	5.900	9.416	Tyrosine recombinase XerD
bin008	SOY3_bin008_02630	1914	7	13	14	0.437	0.689	0.777	hypothetical protein
bin008	SOY3_bin008_02631	1479	7	37	39	0.566	2.537	2.801	hypothetical protein
bin008	SOY3_bin008_02632	1590	0	2	3	0.000	0.128	0.200	Modification methylase MboII
bin008	SOY3_bin008_02633	942	8	46	43	1.015	4.953	4.849	Modulator of FtsH protease HflK
bin008	SOY3_bin008_02634	438	4	7	8	1.092	1.621	1.940	hypothetical protein
bin008	SOY3_bin008_02635	351	3	6	26	1.022	1.734	7.869	hypothetical protein
bin008	SOY3_bin008_02636	2034	4	18	13	0.235	0.898	0.679	Lon protease
bin008	SOY3_bin008_02637	1455	0	3	2	0.000	0.209	0.146	PglZ domain protein

bin008	SOY3_bin008_02638	723	11	45	66	1.819	6.313	9.697	hypothetical protein
bin008	SOY3_bin008_02639	375	8	17	34	2.550	4.598	9.631	hypothetical protein
bin008	SOY3_bin008_02640	3159	68	81	102	2.573	2.601	3.430	hypothetical protein
bin008	SOY3_bin008_02641	510	14	140	167	3.282	27.843	34.784	hypothetical protein
bin008	SOY3_bin008_02642	2367	57	150	163	2.879	6.428	7.315	Penicillin-binding protein 1A
bin008	SOY3_bin008_02643	618	31	55	66	5.997	9.027	11.344	hypothetical protein
bin008	SOY3_bin008_02644	255	16	36	53	7.501	14.319	22.078	hypothetical protein
bin008	SOY3_bin008_02645	654	72	167	227	13.161	25.900	36.870	Selenocysteine-containing peroxiredoxin PrxU
bin008	SOY3_bin008_02646	2655	15	83	69	0.675	3.171	2.761	Bifunctional uridylyltransferase/uridylyl-removing enzyme
bin008	SOY3_bin008_02647	339	87	409	492	30.681	122.371	154.168	Nitrogen regulatory protein P-II 1
bin008	SOY3_bin008_02648	1215	1	28	24	0.098	2.337	2.098	Ammonium transporter NrgA
bin008	SOY3_bin008_02649	1455	123	223	238	10.106	15.545	17.376	Sporulation kinase A
bin008	SOY3_bin008_02650	666	43	45	51	7.719	6.853	8.134	2-O-methyltransferase NoeI
bin008	SOY3_bin008_02651	2337	33	112	109	1.688	4.861	4.954	Methyl-accepting chemotaxis protein PctA
bin008	SOY3_bin008_02652	1284	19	61	64	1.769	4.819	5.295	Phosphoribosylamine--glycine ligase
bin008	SOY3_bin008_02653	498	6	30	36	1.440	6.110	7.679	N5-carboxyaminoimidazole ribonucleotide mutase
bin008	SOY3_bin008_02654	732	5	36	33	0.817	4.988	4.789	hypothetical protein
bin008	SOY3_bin008_02655	390	5	24	29	1.533	6.242	7.899	hypothetical protein
bin008	SOY3_bin008_02656	654	12	31	25	2.194	4.808	4.061	hypothetical protein
bin008	SOY3_bin008_02657	195	0	0	0	0.000	0.000	0.000	hypothetical protein
bin008	SOY3_bin008_02658	459	0	4	3	0.000	0.884	0.694	hypothetical protein
bin008	SOY3_bin008_02659	468	0	0	0	0.000	0.000	0.000	hypothetical protein
bin008	SOY3_bin008_02660	612	3	2	2	0.586	0.331	0.347	hypothetical protein
bin008	SOY3_bin008_02661	420	0	1	0	0.000	0.241	0.000	hypothetical protein
bin008	SOY3_bin008_02662	606	0	1	1	0.000	0.167	0.175	hypothetical protein
bin008	SOY3_bin008_02663	3075	1	3	3	0.039	0.099	0.104	hypothetical protein
bin008	SOY3_bin008_02664	345	1	0	0	0.347	0.000	0.000	hypothetical protein
bin008	SOY3_bin008_02665	219	1	7	8	0.546	3.242	3.880	hypothetical protein
bin008	SOY3_bin008_02666	294	1	2	8	0.407	0.690	2.890	hypothetical protein
bin008	SOY3_bin008_02667	1236	4	14	15	0.387	1.149	1.289	Prophage CP4-57 integrase
bin008	SOY3_bin008_02668	86	0	2	1	0.000	2.359	1.235	tRNA-Leu(gag)
bin008	SOY3_bin008_02669	390	44	193	259	13.488	50.193	70.545	Protein-export membrane protein SecG
bin008	SOY3_bin008_02670	756	36	150	162	5.693	20.124	22.763	Bifunctional PGK/TIM
bin008	SOY3_bin008_02671	1203	32	200	251	3.180	16.862	22.163	Bifunctional PGK/TIM
bin008	SOY3_bin008_02672	483	26	82	109	6.435	17.220	23.972	L-2,4-diaminobutyric acid acetyltransferase
bin008	SOY3_bin008_02673	537	35	83	102	7.792	15.677	20.177	Nudix hydrolase
bin008	SOY3_bin008_02674	777	32	65	78	4.924	8.485	10.664	Inositol-1-monophosphatase
bin008	SOY3_bin008_02675	1026	52	121	169	6.059	11.962	17.497	Rod shape-determining protein MreB
bin008	SOY3_bin008_02676	993	9	33	39	1.084	3.371	4.172	fused phosphoenolpyruvate-protein phosphotransferase PtsP/GAF domain protein
bin008	SOY3_bin008_02677	1761	27	128	138	1.833	7.372	8.324	hypothetical protein
bin008	SOY3_bin008_02678	1089	28	121	143	3.074	11.270	13.949	Aminomethyltransferase
bin008	SOY3_bin008_02679	717	5	13	17	0.834	1.839	2.519	Ribosomal RNA small subunit methyltransferase E
bin008	SOY3_bin008_02680	1227	10	30	30	0.974	2.480	2.597	Replication-associated recombination protein A
bin008	SOY3_bin008_02681	894	41	158	176	5.483	17.926	20.912	putative nicotinate-nucleotide pyrophosphorylase [carboxylating]
bin008	SOY3_bin008_02682	1032	50	163	182	5.792	16.020	18.734	Quinolinate synthase A
bin008	SOY3_bin008_02683	1581	33	154	177	2.495	9.880	11.892	L-aspartate oxidase
bin008	SOY3_bin008_02684	909	5	23	25	0.658	2.566	2.922	Spore coat polysaccharide biosynthesis protein SpsA
bin008	SOY3_bin008_02685	1188	2	16	16	0.201	1.366	1.431	hypothetical protein
bin008	SOY3_bin008_02686	612	2	12	12	0.391	1.989	2.083	hypothetical protein
bin008	SOY3_bin008_02687	669	5	22	30	0.893	3.335	4.763	CMP-N,N'-diacetyllegionaminic acid synthase
bin008	SOY3_bin008_02688	1161	10	66	54	1.030	5.766	4.941	UDP-4-amino-4-deoxy-L-arabinose--oxoglutarate aminotransferase
bin008	SOY3_bin008_02689	1041	22	66	75	2.526	6.431	7.653	D-glycero-alpha-D-manno-heptose 1-phosphate guanylyltransferase
bin008	SOY3_bin008_02690	339	10	22	18	3.527	6.582	5.640	HicB family protein
bin008	SOY3_bin008_02691	255	7	33	22	3.282	13.126	9.165	hypothetical protein
bin008	SOY3_bin008_02692	1194	30	45	67	3.004	3.823	5.961	MarR family protein
bin008	SOY3_bin008_02693	789	3	19	19	0.455	2.442	2.558	Sporulation initiation inhibitor protein Soj
bin008	SOY3_bin008_02694	1230	15	44	46	1.458	3.628	3.973	Cyclic pyranopterin monophosphate synthase
bin008	SOY3_bin008_02695	861	21	56	97	2.916	6.597	11.967	Putative glycosyltransferase EpsH
bin008	SOY3_bin008_02696	558	9	46	41	1.928	8.361	7.805	DNA-invertase hin
bin008	SOY3_bin008_02697	1071	13	36	47	1.451	3.409	4.662	Putative glycosyltransferase EpsE
bin008	SOY3_bin008_02698	1371	8	50	44	0.698	3.699	3.409	molybdenum cofactor biosynthesis protein A
bin008	SOY3_bin008_02699	1020	11	32	46	1.289	3.182	4.791	hypothetical protein
bin008	SOY3_bin008_02700	1086	16	73	75	1.761	6.818	7.336	tRNA (mo5U34)-methyltransferase
bin008	SOY3_bin008_02701	1137	21	48	85	2.208	4.282	7.941	Cyclic pyranopterin monophosphate synthase 1
bin008	SOY3_bin008_02702	855	17	60	84	2.377	7.118	10.436	molybdenum cofactor biosynthesis protein A
bin008	SOY3_bin008_02703	1359	6	31	41	0.528	2.314	3.205	Sulfotransferase family protein
bin008	SOY3_bin008_02704	1119	8	42	46	0.855	3.807	4.367	pyrroloquinoline quinone biosynthesis protein PqqE

bin008	SOY3_bin008_02705	1938	28	68	82	1.727	3.559	4.495	D-inositol 3-phosphate glycosyltransferase
bin008	SOY3_bin008_02706	645	7	19	25	1.297	2.988	4.117	hypothetical protein
bin008	SOY3_bin008_02707	1143	9	27	42	0.941	2.396	3.903	Chondroitin synthase
bin008	SOY3_bin008_02708	1002	4	47	39	0.477	4.758	4.135	GDP-6-deoxy-D-mannose reductase
bin008	SOY3_bin008_02709	1092	14	34	46	1.533	3.158	4.475	UDP-2,3-diacetamido-2,3-dideoxy-D-glucuronate 2-epimerase
bin008	SOY3_bin008_02710	1062	15	34	48	1.689	3.247	4.801	UDP-glucose 4-epimerase
bin008	SOY3_bin008_02711	888	14	68	100	1.885	7.767	11.962	dTDP-4-dehydrorhamnose reductase
bin008	SOY3_bin008_02712	1245	4	19	12	0.384	1.548	1.024	Glycosyl transferases group 1
bin008	SOY3_bin008_02713	633	7	23	25	1.322	3.685	4.195	Putative acetyltransferase
bin008	SOY3_bin008_02714	933	8	34	52	1.025	3.696	5.920	Cyclic pyranopterin monophosphate synthase 1
bin008	SOY3_bin008_02715	768	14	35	42	2.179	4.622	5.809	3-deoxy-manno-octulosonate cytidylyltransferase
bin008	SOY3_bin008_02716	1179	17	38	51	1.724	3.269	4.595	hypothetical protein
bin008	SOY3_bin008_02717	1062	14	30	41	1.576	2.865	4.101	UDP-N-acetylglucosamine--N-acetylmuramyl-(pentapeptide) pyrophosphoryl-undecaprenol N-acetylglucosamine transferase
bin008	SOY3_bin008_02718	972	29	108	134	3.567	11.270	14.644	N,N'-diacetyllegionaminic acid synthase
bin008	SOY3_bin008_02719	981	22	94	125	2.681	9.719	13.535	S-adenosyl-L-methionine-dependent 2-deoxy-scyllo-inosamine dehydrogenase
bin008	SOY3_bin008_02720	1716	41	147	188	2.856	8.689	11.638	Decarbamoylnovobiocin carbamoyltransferase
bin008	SOY3_bin008_02721	1056	24	69	85	2.717	6.627	8.550	Inositol 2-dehydrogenase
bin008	SOY3_bin008_02722	1029	23	79	115	2.672	7.787	11.872	Antilisterial bacteriocin subtilosin biosynthesis protein AlbA
bin008	SOY3_bin008_02723	1179	28	95	137	2.839	8.173	12.343	hypothetical protein
bin008	SOY3_bin008_02724	1293	15	141	163	1.387	11.061	13.391	L-glutamine:scyllo-inosose aminotransferase
bin008	SOY3_bin008_02725	1068	16	162	229	1.791	15.385	22.777	Ubiquinone biosynthesis O-methyltransferase
bin008	SOY3_bin008_02726	1329	11	62	69	0.989	4.732	5.515	(Dimethylallyl)adenosine tRNA methylthiotransferase MiaB
bin008	SOY3_bin008_02727	654	4	25	26	0.731	3.877	4.223	GlcNAc-PI de-N-acetylase
bin008	SOY3_bin008_02728	711	5	21	15	0.841	2.996	2.241	WbqC-like protein family protein
bin008	SOY3_bin008_02729	903	9	30	44	1.192	3.370	5.176	hypothetical protein
bin008	SOY3_bin008_02730	291	5	14	15	2.054	4.880	5.476	hypothetical protein
bin008	SOY3_bin008_02731	1080	20	49	67	2.214	4.602	6.590	Chemotaxis protein CheY
bin008	SOY3_bin008_02732	738	10	16	20	1.620	2.199	2.879	cellulose synthase subunit BcsC
bin008	SOY3_bin008_02733	387	179	222	323	55.295	58.183	88.659	hypothetical protein
bin008	SOY3_bin008_02734	1143	170	166	220	17.781	14.730	20.446	Inner membrane transport permease YbhS
bin008	SOY3_bin008_02735	918	29	33	48	3.777	3.646	5.554	putative ABC transporter ATP-binding protein YbhF
bin008	SOY3_bin008_02736	972	16	58	54	1.968	6.052	5.901	Multidrug resistance protein MdtN
bin008	SOY3_bin008_02737	651	11	37	36	2.020	5.765	5.874	HTH-type transcriptional repressor NicS
bin008	SOY3_bin008_02738	807	2	21	22	0.296	2.639	2.896	putative phospholipid ABC transporter permease protein MlaE
bin008	SOY3_bin008_02739	450	5	57	64	1.328	12.847	15.108	putative phospholipid ABC transporter-binding protein MlaD
bin008	SOY3_bin008_02740	591	6	74	65	1.214	12.700	11.683	putative phospholipid-binding protein MlaC precursor
bin008	SOY3_bin008_02741	843	14	62	62	1.985	7.460	7.813	putative phospholipid-binding lipoprotein MlaA precursor
bin008	SOY3_bin008_02742	579	1	3	0	0.206	0.526	0.000	Exoenzyme S synthesis regulatory protein ExsA
bin008	SOY3_bin008_02743	1017	0	3	4	0.000	0.299	0.418	EamA-like transporter family protein
bin008	SOY3_bin008_02744	993	25	82	111	3.010	8.376	11.874	Sporulation related domain protein
bin008	SOY3_bin008_02745	327	10	28	39	3.656	8.685	12.669	Hpt domain protein
bin008	SOY3_bin008_02746	960	44	105	118	5.479	11.094	13.057	putative L,D-transpeptidase YnhG precursor
bin008	SOY3_bin008_02747	906	75	124	134	9.896	13.882	15.711	Ferredoxin-2
bin008	SOY3_bin008_02748	720	50	79	97	8.302	11.129	14.311	Gamma-DL-glutamyl hydrolase precursor
bin008	SOY3_bin008_02749	1359	95	176	227	8.357	13.136	17.743	Sensor protein ZraS
bin008	SOY3_bin008_02750	801	6	76	51	0.895	9.624	6.763	Disulfide bond formation protein D precursor
bin008	SOY3_bin008_02751	75	0	4	9	0.000	5.409	12.747	tRNA-Cys(gca)
bin008	SOY3_bin008_02752	75	4	4	4	6.376	5.409	5.665	tRNA-Gly(gcc)
bin008	SOY3_bin008_02753	732	2	8	7	0.327	1.108	1.016	N5-glutamine S-adenosyl-L-methionine-dependent methyltransferase
bin008	SOY3_bin008_02754	1767	8	16	31	0.541	0.918	1.864	Ribosomal protein S12 methylthiotransferase accessory factor YcaO
bin008	SOY3_bin008_02755	318	1521	2703	3479	571.805	862.132	1162.136	Sulfite reductase, dissimilatory-type subunit gamma
bin008	SOY3_bin008_02756	2721	22	64	82	0.967	2.386	3.201	Tetratricopeptide repeat protein
bin008	SOY3_bin008_02757	360	16	37	46	5.313	10.424	13.573	MoaE protein
bin008	SOY3_bin008_02758	915	29	52	64	3.789	5.764	7.430	UDP-Glc:alpha-D-GlcNAc-diphosphoundecaprenol beta-1,3-glucosyltransferase WfgD
bin008	SOY3_bin008_02759	741	7	26	24	1.129	3.559	3.441	hypothetical protein
bin008	SOY3_bin008_02760	1377	60	794	766	5.209	58.485	59.091	Chromosomal replication initiator protein DnaA
bin008	SOY3_bin008_02761	747	2	15	16	0.320	2.037	2.275	Thymidylate synthase ThyX
bin008	SOY3_bin008_02762	993	10	40	46	1.204	4.086	4.921	Holliday junction ATP-dependent DNA helicase RuvB
bin008	SOY3_bin008_02763	609	6	14	17	1.178	2.332	2.965	Holliday junction ATP-dependent DNA helicase RuvA
bin008	SOY3_bin008_02764	510	3	12	9	0.703	2.387	1.875	Crossover junction endodeoxyribonuclease RuvC
bin008	SOY3_bin008_02765	738	15	69	93	2.430	9.483	13.386	putative transcriptional regulatory protein YebC
bin008	SOY3_bin008_02766	657	10	17	24	1.820	2.624	3.880	Ribosomal RNA large subunit methyltransferase E
bin008	SOY3_bin008_02767	1527	25	31	45	1.957	2.059	3.130	hypothetical protein
bin008	SOY3_bin008_02768	76	0	0	1	0.000	0.000	1.398	tRNA-Ala(cgc)
bin008	SOY3_bin008_02769	1644	166	307	433	12.071	18.940	27.978	Glucose-6-phosphate isomerase
bin008	SOY3_bin008_02770	2493	161	266	347	7.721	10.822	14.786	Magnesium-transporting ATPase, P-type 1
bin008	SOY3_bin008_02771	1761	121	250	311	8.214	14.399	18.760	putative oxidoreductase YdhV

bin008	SOY3_bin008_02772	450	35	92	133	9.298	20.736	31.396	putative ferredoxin-like protein YdhY
bin008	SOY3_bin008_02773	696	46	108	122	7.901	15.739	18.620	HTH-type transcriptional regulator LutR
bin008	SOY3_bin008_02774	1038	17	53	71	1.958	5.179	7.266	Heat-inducible transcription repressor HrcA
bin008	SOY3_bin008_02775	540	1	32	46	0.221	6.011	9.049	heat shock protein GrpE
bin008	SOY3_bin008_02776	1917	47	717	703	2.931	37.936	38.955	Chaperone protein DnaK
bin008	SOY3_bin008_02777	561	0	0	2	0.000	0.000	0.379	site-specific tyrosine recombinase XerC
bin008	SOY3_bin008_02778	1029	0	2	3	0.000	0.197	0.310	Transposase from transposon Tn916
bin008	SOY3_bin008_02779	76	3	0	0	4.719	0.000	0.000	tRNA-Ala(ggc)
bin008	SOY3_bin008_02780	996	26	34	36	3.121	3.462	3.839	Dipeptide transport system permease protein DppB
bin008	SOY3_bin008_02781	846	43	51	69	6.076	6.114	8.664	Dipeptide transport system permease protein DppC
bin008	SOY3_bin008_02782	1617	7	57	50	0.518	3.575	3.285	DNA repair protein RecN
bin008	SOY3_bin008_02783	1188	6	20	9	0.604	1.708	0.805	YibE/F-like protein
bin008	SOY3_bin008_02784	1614	8	20	23	0.593	1.257	1.514	Alkaline phosphatase 4 precursor
bin008	SOY3_bin008_02785	591	6	21	28	1.214	3.604	5.033	Thiol-disulfide oxidoreductase ResA
bin008	SOY3_bin008_02786	618	4	20	19	0.774	3.282	3.266	Putative peroxiredoxin/MT2298
bin008	SOY3_bin008_02787	318	13	80	108	4.887	25.516	36.077	Carboxymuconolactone decarboxylase family protein
bin008	SOY3_bin008_02788	576	97	494	596	20.132	86.988	109.914	Rubrerythrin
bin008	SOY3_bin008_02789	447	4	11	21	1.070	2.496	4.990	hypothetical protein
bin008	SOY3_bin008_02790	1080	7	24	33	0.775	2.254	3.246	Formate dehydrogenase, nitrate-inducible, iron-sulfur subunit
bin008	SOY3_bin008_02791	696	13	21	29	2.233	3.060	4.426	cAMP receptor protein
bin008	SOY3_bin008_02792	615	7	24	28	1.361	3.958	4.836	cAMP-activated global transcriptional regulator CRP
bin008	SOY3_bin008_02793	1176	51	137	172	5.185	11.816	15.536	hypothetical protein
bin008	SOY3_bin008_02794	663	5	11	7	0.902	1.683	1.122	hypothetical protein
bin008	SOY3_bin008_02795	342	3	12	12	1.049	3.559	3.727	Anti-sigma-B factor antagonist
bin008	SOY3_bin008_02796	1056	219	246	294	24.793	23.628	29.574	Phytochrome-like protein cph2
bin008	SOY3_bin008_02797	1200	79	162	178	7.870	13.693	15.757	Long-chain primary alcohol dehydrogenase AdhA
bin008	SOY3_bin008_02798	984	0	3	3	0.000	0.309	0.324	Magnesium transport protein CorA
bin008	SOY3_bin008_02799	273	98	269	328	42.915	99.941	127.627	hypothetical protein
bin008	SOY3_bin008_02800	231	4	12	15	2.070	5.269	6.898	Ferrous iron transport protein A
bin008	SOY3_bin008_02801	2190	19	91	93	1.037	4.215	4.511	Ferrous iron transport protein B
bin008	SOY3_bin008_02802	1200	18	35	35	1.793	2.958	3.098	L-threonine dehydratase catabolic TdcB
bin008	SOY3_bin008_02803	501	5	19	15	1.193	3.847	3.180	Glutaminase-asparaginase
bin008	SOY3_bin008_02804	705	10	26	23	1.696	3.741	3.466	High-affinity branched-chain amino acid transport ATP-binding protein LivF
bin008	SOY3_bin008_02805	786	8	10	12	1.217	1.290	1.622	Lipopolysaccharide export system ATP-binding protein LptB
bin008	SOY3_bin008_02806	1038	11	15	14	1.267	1.466	1.433	leucine/isoleucine/valine transporter permease subunit
bin008	SOY3_bin008_02807	894	13	24	32	1.738	2.723	3.802	High-affinity branched-chain amino acid transport system permease protein LivH
bin008	SOY3_bin008_02808	1134	109	334	340	11.491	29.874	31.849	hypothetical protein
bin008	SOY3_bin008_02809	768	7	14	20	1.090	1.849	2.766	Transcriptional regulator KdgR
bin008	SOY3_bin008_02810	774	58	242	326	8.958	31.712	44.741	CobQ/CobB/MinD/ParA nucleotide binding domain protein
bin008	SOY3_bin008_02811	1257	26	80	124	2.473	6.455	10.479	Nitrite reductase [NAD(P)H]
bin008	SOY3_bin008_02812	1020	8	28	17	0.938	2.784	1.770	outer membrane protease
bin008	SOY3_bin008_02813	186	14	43	45	8.998	23.448	25.700	hypothetical protein
bin008	SOY3_bin008_02814	1881	742	774	777	47.159	41.736	43.880	Carbon monoxide dehydrogenase 1
bin008	SOY3_bin008_02815	537	183	209	212	40.740	39.475	41.936	Iron-sulfur protein
bin008	SOY3_bin008_02816	339	1448	2244	2998	510.639	671.395	939.424	hypothetical protein
bin008	SOY3_bin008_02817	1221	28	63	76	2.741	5.233	6.612	Tyrosine recombinase XerD
bin008	SOY3_bin008_02818	2046	44	115	141	2.571	5.701	7.321	Membrane-bound lytic murein transglycosylase D precursor
bin008	SOY3_bin008_02819	651	51	235	301	9.366	36.614	49.115	Alkyl hydroperoxide reductase subunit C
bin008	SOY3_bin008_02820	948	6	61	57	0.757	6.526	6.387	Porphobilinogen deaminase
bin008	SOY3_bin008_02821	243	4	11	7	1.968	4.591	3.060	Zinc ribbon domain protein
bin008	SOY3_bin008_02822	615	1	23	14	0.194	3.793	2.418	Phosphoheptose isomerase
bin008	SOY3_bin008_02823	531	23	75	85	5.178	14.326	17.004	Shikimate kinase
bin008	SOY3_bin008_02824	603	6	26	23	1.190	4.373	4.052	hypothetical protein
bin008	SOY3_bin008_02825	540	6	26	26	1.328	4.884	5.115	hypothetical protein
bin008	SOY3_bin008_02826	1290	13	25	25	1.205	1.966	2.059	hypothetical protein
bin008	SOY3_bin008_02827	1704	47	61	66	3.297	3.631	4.114	hypothetical protein
bin008	SOY3_bin008_02828	309	501	884	1165	193.832	290.167	400.495	glycogen branching enzyme
bin008	SOY3_bin008_02829	429	43	140	200	11.983	33.100	49.522	Transcriptional activator protein CzcR
bin008	SOY3_bin008_02830	714	5	25	34	0.837	3.551	5.058	1-acyl-sn-glycerol-3-phosphate acyltransferase
bin008	SOY3_bin008_02831	1659	37	125	169	2.666	7.642	10.821	Ribonuclease J 1
bin008	SOY3_bin008_02832	783	16	43	56	2.443	5.570	7.597	Ureidoglycolate lyase
bin008	SOY3_bin008_02833	1089	11	55	73	1.208	5.123	7.121	Cyclic pyranopterin monophosphate synthase
bin008	SOY3_bin008_02834	846	16	264	267	2.261	31.651	33.525	30S ribosomal protein S2
bin008	SOY3_bin008_02835	603	11	299	248	2.181	50.293	43.688	Elongation factor Ts
bin008	SOY3_bin008_02836	732	99	277	279	16.168	38.382	40.488	Uridylate kinase
bin008	SOY3_bin008_02837	555	40	176	179	8.616	32.164	34.260	Ribosome-recycling factor
bin008	SOY3_bin008_02838	675	42	114	116	7.439	17.130	18.255	Ditrans,polycis-undecaprenyl-diphosphate synthase ((2E,6E)-farnesyl-diphosphate specific)

bin008	SOY3_bin008_02839	1074	30	48	75	3.339	4.533	7.418	Nicotinate-nucleotide--dimethylbenzimidazole phosphoribosyltransferase
bin008	SOY3_bin008_02840	1338	81	197	250	7.237	14.934	19.848	6-phosphofructokinase 1
bin008	SOY3_bin008_02841	1014	3	10	9	0.354	1.000	0.943	Glycosyl transferases group 1
bin008	SOY3_bin008_02842	1269	1	9	10	0.094	0.719	0.837	Spore protein YkvP
bin008	SOY3_bin008_02843	1197	50	111	141	4.994	9.406	12.513	2-aminoadipate transaminase
bin008	SOY3_bin008_02844	2271	36	75	108	1.895	3.350	5.052	Adenylate cyclase 1
bin008	SOY3_bin008_02845	1407	26	44	51	2.209	3.172	3.850	tRNA modification GTPase MnmE
bin008	SOY3_bin008_02846	1317	37	77	91	3.359	5.930	7.340	Di-/tripeptide transporter
bin008	SOY3_bin008_02847	837	42	164	180	5.999	19.873	22.844	photosystem I assembly protein Ycf3
bin008	SOY3_bin008_02848	954	28	169	170	3.509	17.968	18.929	UDP-glucose 4-epimerase
bin008	SOY3_bin008_02849	858	36	110	128	5.016	13.003	15.847	Ribosomal protein L11 methyltransferase
bin008	SOY3_bin008_02850	2073	420	685	824	24.221	33.516	42.224	D-gamma-glutamyl-meso-diaminopimelic acid endopeptidase CwlS precursor
bin008	SOY3_bin008_02851	1455	3	24	33	0.246	1.673	2.409	Peptidoglycan O-acetyltransferase
bin008	SOY3_bin008_02852	1143	13	21	39	1.360	1.863	3.625	1,3-propanediol dehydrogenase
bin008	SOY3_bin008_02853	1209	23	33	56	2.274	2.768	4.920	Sensor protein ZraS
bin008	SOY3_bin008_02854	1350	14	34	55	1.240	2.554	4.328	Transcriptional regulatory protein ZraR
bin008	SOY3_bin008_02855	1110	11	45	56	1.185	4.112	5.359	Minor endoglucanase Y precursor
bin008	SOY3_bin008_02856	1182	23	65	82	2.326	5.578	7.369	hypothetical protein
bin008	SOY3_bin008_02857	2859	58	137	217	2.425	4.860	8.063	Cellulose synthase operon protein C precursor
bin008	SOY3_bin008_02858	1590	18	88	130	1.353	5.614	8.685	Cellulose synthase catalytic subunit [UDP-forming]
bin008	SOY3_bin008_02859	1230	0	41	22	0.000	3.381	1.900	Multidrug resistance protein MexA precursor
bin008	SOY3_bin008_02860	3126	14	63	91	0.535	2.044	3.092	Multidrug efflux pump subunit AcrB
bin008	SOY3_bin008_02861	285	156	239	257	65.437	85.057	95.790	RNA recognition motif. (a.k.a. RRM, RBD, or RNP domain)
bin008	SOY3_bin008_02862	1035	2	5	9	0.231	0.490	0.924	corrinoid ABC transporter substrate-binding protein
bin008	SOY3_bin008_02863	2295	7	25	24	0.365	1.105	1.111	Pesticin receptor precursor
bin008	SOY3_bin008_02864	807	0	3	3	0.000	0.377	0.395	Iron(3+)-hydroxamate import ATP-binding protein FhuC
bin008	SOY3_bin008_02865	1005	0	3	3	0.000	0.303	0.317	putative ABC transporter permease protein
bin008	SOY3_bin008_02866	1263	1	2	5	0.095	0.161	0.421	hypothetical protein
bin008	SOY3_bin008_02867	1956	34	108	159	2.078	5.600	8.635	Magnesium-chelatase 38 kDa subunit
bin008	SOY3_bin008_02868	1032	4	46	71	0.463	4.521	7.308	Magnesium-chelatase 38 kDa subunit
bin008	SOY3_bin008_02869	3948	1	78	91	0.030	2.004	2.448	Aerobic cobaltochelatase subunit CobN
bin008	SOY3_bin008_02870	2304	3	57	53	0.156	2.509	2.444	Colicin I receptor precursor
bin008	SOY3_bin008_02871	771	1	13	12	0.155	1.710	1.653	putative siderophore transport system ATP-binding protein YusV
bin008	SOY3_bin008_02872	1080	1	15	20	0.111	1.409	1.967	Iron-uptake system permease protein FeuC
bin008	SOY3_bin008_02873	1107	2	32	40	0.216	2.932	3.838	putative ABC transporter solute-binding protein YclQ precursor
bin008	SOY3_bin008_02874	738	0	12	11	0.000	1.649	1.583	Inner membrane transport permease YadH
bin008	SOY3_bin008_02875	897	0	37	28	0.000	4.184	3.316	Daunorubicin/doxorubicin resistance ATP-binding protein DrrA
bin008	SOY3_bin008_02876	780	34	68	73	5.211	8.842	9.942	Yip1 domain protein
bin008	SOY3_bin008_02877	336	32	74	89	11.386	22.338	28.137	hypothetical protein
bin008	SOY3_bin008_02878	705	43	101	118	7.292	14.531	17.780	hypothetical protein
bin008	SOY3_bin008_02879	1089	2	5	18	0.220	0.466	1.756	Sodium Bile acid symporter family protein
bin008	SOY3_bin008_02880	447	17	53	87	4.547	12.026	20.675	HTH-type transcriptional regulator CymR
bin008	SOY3_bin008_02881	657	5	8	9	0.910	1.235	1.455	Thiamine-phosphate synthase
bin008	SOY3_bin008_02882	2943	9	28	24	0.366	0.965	0.866	ATP-dependent DNA helicase RecQ
bin008	SOY3_bin008_02883	234	9	35	60	4.598	15.171	27.237	hypothetical protein
bin008	SOY3_bin008_02884	1374	11	40	63	0.957	2.953	4.871	Transcriptional regulatory protein ZraR
bin008	SOY3_bin008_02885	1449	23	38	33	1.898	2.660	2.419	Sporulation kinase A
bin008	SOY3_bin008_02886	1176	52	129	120	5.286	11.126	10.839	Aspartate aminotransferase
bin008	SOY3_bin008_02887	753	306	426	567	48.582	57.381	79.987	Sugar fermentation stimulation protein A
bin008	SOY3_bin008_02888	210	236	401	442	134.350	193.678	223.580	Molybdenum-pterin-binding protein 2
bin008	SOY3_bin008_02889	1476	1	2	5	0.081	0.137	0.360	Limonene hydroxylase
bin008	SOY3_bin008_02890	369	9	2	2	2.916	0.550	0.576	NifU-like protein
bin008	SOY3_bin008_02891	2457	68	174	209	3.309	7.183	9.036	Maltodextrin phosphorylase
bin008	SOY3_bin008_02892	2496	101	126	161	4.838	5.120	6.852	Sensory/regulatory protein RpfC
bin008	SOY3_bin008_02893	1329	289	512	571	25.997	39.075	45.640	HDOD domain protein
bin008	SOY3_bin008_02894	678	1	3	6	0.176	0.449	0.940	hypothetical protein
bin008	SOY3_bin008_02895	2091	221	466	591	12.635	22.604	30.024	Methyl-accepting chemotaxis protein PctC
bin008	SOY3_bin008_02896	816	29	65	79	4.249	8.079	10.284	HTH-type transcriptional regulator PrtR
bin008	SOY3_bin008_02897	282	10	9	19	4.239	3.237	7.157	Mor transcription activator family protein
bin008	SOY3_bin008_02898	1701	121	206	254	8.504	12.283	15.862	Aerobic respiration control sensor protein ArcB
bin008	SOY3_bin008_02899	351	9	20	31	3.065	5.779	9.382	hypothetical protein
bin008	SOY3_bin008_02900	1407	3	34	30	0.255	2.451	2.265	Transcriptional regulatory protein QseF
bin008	SOY3_bin008_02901	315	14	73	78	5.313	23.505	26.304	hypothetical protein
bin008	SOY3_bin008_02902	951	30	189	204	3.771	20.157	22.787	Membrane-bound lytic murein transglycosylase B precursor
bin008	SOY3_bin008_02903	390	8	48	49	2.452	12.483	13.346	hypothetical protein
bin008	SOY3_bin008_02904	330	9	37	41	3.260	11.372	13.198	Membrane-associated protein TcaA
bin008	SOY3_bin008_02905	198	2	27	28	1.208	13.831	15.022	Ferredoxin-1

bin008	SOY3_bin008_02906	1980	18	68	69	1.087	3.483	3.702	Glutamine-dependent NAD(+) synthetase
bin008	SOY3_bin008_02907	1866	152	309	265	9.738	16.796	15.086	GTPase HflX
bin008	SOY3_bin008_02908	594	1	41	25	0.201	7.001	4.471	Bifunctional purine biosynthesis protein PurH
bin008	SOY3_bin008_02909	3174	9	26	24	0.339	0.831	0.803	Phage-related minor tail protein
bin008	SOY3_bin008_02910	1101	1	5	3	0.109	0.461	0.289	Phage Mu protein F like protein
bin008	SOY3_bin008_02911	387	3	3	1	0.927	0.786	0.274	hypothetical protein
bin008	SOY3_bin008_02912	408	2	10	5	0.586	2.486	1.302	hypothetical protein
bin008	SOY3_bin008_02913	921	8	15	10	1.038	1.652	1.153	hypothetical protein
bin008	SOY3_bin008_02914	381	1	5	7	0.314	1.331	1.952	hypothetical protein
bin008	SOY3_bin008_02915	1311	3	10	19	0.274	0.774	1.540	hypothetical protein
bin008	SOY3_bin008_02916	1701	6	18	37	0.422	1.073	2.311	hypothetical protein
bin008	SOY3_bin008_02917	342	0	0	3	0.000	0.000	0.932	hypothetical protein
bin008	SOY3_bin008_02918	489	1	1	4	0.244	0.207	0.869	hypothetical protein
bin008	SOY3_bin008_02919	243	1	1	0	0.492	0.417	0.000	hypothetical protein
bin008	SOY3_bin008_02920	2133	1	2	4	0.056	0.095	0.199	hypothetical protein
bin008	SOY3_bin008_02921	234	0	0	1	0.000	0.000	0.454	hypothetical protein
bin008	SOY3_bin008_02922	696	7	19	26	1.202	2.769	3.968	hypothetical protein
bin008	SOY3_bin008_02923	1254	145	301	260	13.823	24.346	22.024	Tyrosine recombinase XerD
bin008	SOY3_bin008_02924	207	137	182	178	79.122	89.178	91.344	Molybdenum-pterin-binding protein 2
bin008	SOY3_bin008_02925	189	7	5	3	4.428	2.683	1.686	hypothetical protein
bin008	SOY3_bin008_02926	252	2	1	3	0.949	0.402	1.265	hypothetical protein
bin008	SOY3_bin008_02927	324	0	1	0	0.000	0.313	0.000	Phage terminase, small subunit
bin008	SOY3_bin008_02928	372	0	0	0	0.000	0.000	0.000	hypothetical protein
bin008	SOY3_bin008_02929	2793	4	4	1	0.171	0.145	0.038	hypothetical protein
bin008	SOY3_bin008_02930	270	0	2	1	0.000	0.751	0.393	hypothetical protein
bin008	SOY3_bin008_02931	264	0	2	3	0.000	0.768	1.207	hypothetical protein
bin008	SOY3_bin008_02932	729	3	15	10	0.492	2.087	1.457	hypothetical protein
bin008	SOY3_bin008_02933	246	2	2	8	0.972	0.825	3.454	anaerobic benzoate catabolism transcriptional regulator
bin008	SOY3_bin008_02934	246	3	9	9	1.458	3.711	3.886	hypothetical protein
bin008	SOY3_bin008_02935	159	2	2	2	1.504	1.276	1.336	hypothetical protein
bin008	SOY3_bin008_02936	1233	4	7	16	0.388	0.576	1.378	Tyrosine recombinase XerD
bin008	SOY3_bin008_02937	927	30	59	76	3.869	6.455	8.709	hypothetical protein
bin008	SOY3_bin008_02938	1596	18	29	39	1.348	1.843	2.596	Putative electron transport protein YccM
bin008	SOY3_bin008_02939	1092	68	150	226	7.444	13.932	21.984	Spermidine/putrescine-binding periplasmic protein precursor
bin008	SOY3_bin008_02940	690	77	171	230	13.341	25.136	35.409	Peroxyureidoacrylate/ureidoacrylate amidohydrolase RutB
bin008	SOY3_bin008_02941	1368	45	148	164	3.933	10.973	12.735	2-methylcitrate dehydratase
bin008	SOY3_bin008_02942	396	19	96	122	5.736	24.588	32.726	SnoaL-like domain protein
bin008	SOY3_bin008_02943	2025	285	408	590	16.825	20.436	30.950	hypothetical protein
bin008	SOY3_bin008_02944	1326	9	52	59	0.811	3.978	4.726	3-phosphoshikimate 1-carboxyvinyltransferase
bin008	SOY3_bin008_02945	1143	1	21	20	0.105	1.863	1.859	P-protein
bin008	SOY3_bin008_02946	975	7	54	49	0.858	5.618	5.339	3-amino-4-hydroxybenzoic acid synthase
bin008	SOY3_bin008_02947	798	5	32	13	0.749	4.067	1.730	2-amino-4,5-dihydroxy-6-one-heptanoic acid-7-phosphate synthase
bin008	SOY3_bin008_02948	2358	2	27	23	0.101	1.161	1.036	5-methyltetrahydropteroyltriglutamate--homocysteine methyltransferase
bin008	SOY3_bin008_02949	492	1380	1216	1701	335.320	250.682	367.256	Ferredoxin-2
bin008	SOY3_bin008_02950	1992	4199	3925	5691	252.001	199.850	303.479	adenylylsulfate reductase subunit alpha
bin008	SOY3_bin008_02951	1242	89	159	198	8.567	12.985	16.935	2-enoate reductase FldZ
bin008	SOY3_bin008_02952	2307	174	292	358	9.017	12.838	16.484	putative ferredoxin-like protein YdhX precursor
bin008	SOY3_bin008_02953	1200	138	198	276	13.748	16.736	24.432	sn-glycerol-3-phosphate dehydrogenase subunit C
bin008	SOY3_bin008_02954	690	201	401	585	34.825	58.945	90.061	Chromosome partition protein Smc
bin008	SOY3_bin008_02955	3357	16	118	136	0.570	3.565	4.303	Extracellular serine protease precursor
bin008	SOY3_bin008_02956	1329	2	35	46	0.180	2.671	3.677	Methylenetetrahydrofolate--tRNA-(uracil-5-)-methyltransferase TrmFO
bin008	SOY3_bin008_02957	1365	4	22	24	0.350	1.635	1.868	ATP-dependent RNA helicase RhlE
bin008	SOY3_bin008_02958	408	44	138	197	12.893	34.306	51.290	Transcriptional regulatory protein ros
bin008	SOY3_bin008_02959	321	26	68	99	9.683	21.486	32.761	hypothetical protein
bin008	SOY3_bin008_02960	492	20	72	99	4.860	14.843	21.375	hypothetical protein
bin008	SOY3_bin008_02961	975	40	109	107	4.905	11.339	11.658	Oligopeptide transport ATP-binding protein OppF
bin008	SOY3_bin008_02962	966	20	45	67	2.475	4.725	7.368	Oligopeptide transport ATP-binding protein OppD
bin008	SOY3_bin008_02963	711	62	232	239	10.425	33.096	35.707	Inner membrane protein YbhL
bin008	SOY3_bin008_02964	1098	6	25	23	0.653	2.309	2.225	Tetraacyldisaccharide 4'-kinase
bin008	SOY3_bin008_02965	1173	5	56	61	0.510	4.842	5.524	Leucine-, isoleucine-, valine-, threonine-, and alanine-binding protein precursor
bin008	SOY3_bin008_02966	1467	2	12	12	0.163	0.830	0.869	hypothetical protein
bin008	SOY3_bin008_02967	1356	1	7	13	0.088	0.524	1.018	Peptidoglycan O-acetyltransferase
bin008	SOY3_bin008_02968	1140	6	10	18	0.629	0.890	1.677	Alginate biosynthesis protein AlgX precursor
bin008	SOY3_bin008_02969	501	12	14	21	2.863	2.834	4.453	Bacteriohemerythrin
bin008	SOY3_bin008_02970	624	13	24	30	2.491	3.901	5.107	hypothetical protein
bin008	SOY3_bin008_02971	231	24	48	56	12.421	21.076	25.752	hypothetical protein
bin008	SOY3_bin008_02972	417	172	594	778	49.310	144.479	198.186	hypothetical protein

bin008	SOY3_bin008_02973	714	3	5	12	0.502	0.710	1.785	acyl-CoA synthetase
bin008	SOY3_bin008_02974	648	5	15	26	0.922	2.348	4.262	Peptidoglycan endopeptidase LytF precursor
bin008	SOY3_bin008_02975	94	0	1	1	0.000	1.079	1.130	tRNA-seC(tca)
bin008	SOY3_bin008_02976	77	0	0	1	0.000	0.000	1.380	tRNA-Arg(ccg)
bin008	SOY3_bin008_02977	450	3	5	7	0.797	1.127	1.652	Protease synthase and sporulation negative regulatory protein PAI 1
bin008	SOY3_bin008_02978	657	2	13	20	0.364	2.007	3.234	putative lysine decarboxylase
bin008	SOY3_bin008_02979	1608	14	125	137	1.041	7.885	9.050	Ribonuclease
bin008	SOY3_bin008_02980	219	1	14	18	0.546	6.484	8.731	Translation initiation factor IF-1
bin008	SOY3_bin008_02981	1200	42	138	186	4.184	11.664	16.465	Aminopeptidase 2
bin008	SOY3_bin008_02982	489	28	116	132	6.845	24.060	28.674	hypothetical protein
bin008	SOY3_bin008_02983	477	261	493	620	65.414	104.829	138.071	Chemotaxis protein CheW
bin008	SOY3_bin008_02984	3468	45	92	118	1.551	2.691	3.614	Transcription-repair-coupling factor
bin008	SOY3_bin008_02985	1575	30	52	58	2.277	3.349	3.912	Chaperone SurA
bin008	SOY3_bin008_02986	1023	18	112	110	2.103	11.104	11.422	Chaperone SurA precursor
bin008	SOY3_bin008_02987	1419	25	120	115	2.106	8.577	8.609	cytoskeletal protein RodZ
bin008	SOY3_bin008_02988	747	10	66	98	1.600	8.961	13.936	DNA repair protein RecO
bin008	SOY3_bin008_02989	876	7	52	50	0.955	6.021	6.063	Glycine--tRNA ligase alpha subunit
bin008	SOY3_bin008_02990	2094	20	109	132	1.142	5.280	6.696	Glycine--tRNA ligase beta subunit
bin008	SOY3_bin008_02991	270	16	60	76	7.084	22.539	29.901	30S ribosomal protein S20
bin008	SOY3_bin008_02992	363	4	54	70	1.317	15.088	20.484	RNA polymerase-binding transcription factor DksA
bin008	SOY3_bin008_02993	1521	7	42	52	0.550	2.801	3.632	hypothetical protein
bin008	SOY3_bin008_02994	975	24	115	119	2.943	11.963	12.965	PhoH-like protein
bin008	SOY3_bin008_02995	2304	30	112	123	1.557	4.930	5.671	phosphodiesterase
bin008	SOY3_bin008_02996	420	22	27	47	6.262	6.520	11.887	Endoribonuclease YbeY
bin008	SOY3_bin008_02997	1644	128	218	213	9.308	13.450	13.763	Signal transduction histidine-protein kinase BarA
bin008	SOY3_bin008_02998	345	177	456	606	61.334	134.060	186.588	Serine/threonine-protein kinase D
bin008	SOY3_bin008_02999	909	3	47	40	0.395	5.244	4.674	Ornithine carbamoyltransferase
bin008	SOY3_bin008_03000	1197	9	144	151	0.899	12.202	13.400	Argininosuccinate synthase
bin008	SOY3_bin008_03001	1383	8	63	67	0.692	4.620	5.146	Argininosuccinate lyase 1
bin008	SOY3_bin008_03002	1563	14	77	105	1.071	4.997	7.136	Nitrogen fixation protein VnfA
bin008	SOY3_bin008_03003	1767	60	124	149	4.059	7.118	8.957	Methyl-accepting chemotaxis protein 3
bin008	SOY3_bin008_03004	76	0	3	3	0.000	4.004	4.193	tRNA-Thr(ggt)
bin008	SOY3_bin008_03005	77	4	8	7	6.210	10.538	9.657	tRNA-Gly(tcc)
bin008	SOY3_bin008_03006	86	2	22	15	2.780	25.947	18.528	tRNA-Tyr(gta)
bin008	SOY3_bin008_03007	76	3	15	25	4.719	20.019	34.943	tRNA-Thr(tgt)
bin008	SOY3_bin008_03008	1800	85	174	206	5.645	9.805	12.157	ATP-dependent zinc metalloprotease FtsH 4
bin008	SOY3_bin008_03009	1011	14	27	41	1.655	2.709	4.308	translocation protein TolB
bin008	SOY3_bin008_03010	888	6	38	39	0.808	4.340	4.665	Release factor glutamine methyltransferase
bin008	SOY3_bin008_03011	1074	7	47	40	0.779	4.439	3.956	Peptide chain release factor 1
bin008	SOY3_bin008_03012	960	22	95	97	2.740	10.037	10.733	hypothetical protein
bin008	SOY3_bin008_03013	216	6	137	141	3.321	64.331	69.342	50S ribosomal protein L31
bin008	SOY3_bin008_03014	1311	32	181	199	2.918	14.003	16.124	Phosphomethylpyrimidine synthase
bin008	SOY3_bin008_03015	750	36	174	164	5.738	23.531	23.228	2,3-bisphosphoglycerate-dependent phosphoglycerate mutase
bin008	SOY3_bin008_03016	762	196	343	489	30.750	45.656	68.168	hypothetical protein
bin008	SOY3_bin008_03017	777	330	667	977	50.774	87.068	133.568	Outer-membrane lipoprotein carrier protein
bin008	SOY3_bin008_03018	279	21	82	79	8.998	29.810	30.078	Late competence development protein ComFB
bin008	SOY3_bin008_03019	1011	1	34	33	0.118	3.411	3.467	UDP-glucose 4-epimerase
bin008	SOY3_bin008_03020	2022	5	13	17	0.296	0.652	0.893	hypothetical protein
bin008	SOY3_bin008_03021	1185	11	41	38	1.110	3.509	3.406	hypothetical protein
bin008	SOY3_bin008_03022	891	21	57	82	2.818	6.489	9.776	2,3-dimethylmalate lyase
bin008	SOY3_bin008_03023	1806	11	26	38	0.728	1.460	2.235	putative ABC transporter ATP-binding protein
bin008	SOY3_bin008_03024	2739	74	151	160	3.230	5.592	6.205	Putative glycosyltransferase EpsH
bin008	SOY3_bin008_03025	411	13	212	256	3.781	52.318	66.165	hypothetical protein
bin008	SOY3_bin008_03026	1512	3880	21265	25885	306.779	1426.489	1818.554	hypothetical protein
bin008	SOY3_bin008_03027	420	13	34	42	3.700	8.211	10.623	Enamine/imine deaminase
bin008	SOY3_bin008_03028	1077	38	86	77	4.218	8.099	7.595	Transcriptional regulator ModE
bin008	SOY3_bin008_03029	417	8	34	33	2.294	8.270	8.406	Putative universal stress protein
bin008	SOY3_bin008_03030	390	5	2	7	1.533	0.520	1.907	VanZ like family protein
bin008	SOY3_bin008_03031	876	24	59	65	3.275	6.831	7.882	hypothetical protein
bin008	SOY3_bin008_03032	981	5	8	16	0.609	0.827	1.733	Putative 2-hydroxyacid dehydrogenase
bin008	SOY3_bin008_03033	465	1	6	4	0.257	1.309	0.914	putative voltage-gated ClC-type chloride channel ClcB
bin008	SOY3_bin008_03034	1338	4	16	14	0.357	1.213	1.111	Inner membrane protein YbiR
bin008	SOY3_bin008_03035	171	15	57	73	10.487	33.809	45.348	Flp/Fap pilin component
bin008	SOY3_bin008_03036	384	40	141	158	12.453	37.243	43.707	Acyl-coenzyme A thioesterase PaaI
bin008	SOY3_bin008_03037	1008	22	55	53	2.609	5.534	5.585	HTH-type transcriptional regulator CdhR
bin008	SOY3_bin008_03038	792	50	49	52	7.547	6.275	6.974	hypothetical protein
bin008	SOY3_bin008_03039	1251	35	21	51	3.345	1.703	4.331	hypothetical protein

bin008	SOY3_bin008_03040	2496	355	172	211	17.003	6.989	8.980	Arylsulfatase
bin008	SOY3_bin008_03041	1011	23	13	18	2.720	1.304	1.891	Paraquat-inducible protein B
bin008	SOY3_bin008_03042	2274	27	22	33	1.419	0.981	1.542	Rhomboid protease GluP
bin008	SOY3_bin008_03043	174	1	0	0	0.687	0.000	0.000	hypothetical protein
bin008	SOY3_bin008_03044	414	1	5	7	0.289	1.225	1.796	hypothetical protein
bin008	SOY3_bin008_03045	3045	24	89	85	0.942	2.965	2.965	Autoinducer 2 sensor kinase/phosphatase LuxQ
bin008	SOY3_bin008_03046	5817	36	104	122	0.740	1.813	2.228	Phytochrome-like protein cph2
bin008	SOY3_bin008_03047	309	1	4	5	0.387	1.313	1.719	Tyrosine recombinase XerC
bin008	SOY3_bin008_03048	4926	23	90	88	0.558	1.853	1.898	type I restriction enzyme EcoKI subunit R
bin008	SOY3_bin008_03049	222	0	6	4	0.000	2.741	1.914	hypothetical protein
bin008	SOY3_bin008_03050	1464	3	37	41	0.245	2.563	2.975	hypothetical protein
bin008	SOY3_bin008_03051	234	1	0	2	0.511	0.000	0.908	hypothetical protein
bin008	SOY3_bin008_03052	1905	5	9	20	0.314	0.479	1.115	hypothetical protein
bin008	SOY3_bin008_03053	234	2	11	15	1.022	4.768	6.809	cardiolipin synthetase
bin008	SOY3_bin008_03054	495	5	18	11	1.208	3.688	2.361	Lipoprotein signal peptidase
bin008	SOY3_bin008_03055	2826	18	136	152	0.761	4.881	5.713	Isoleucine--tRNA ligase
bin008	SOY3_bin008_03056	189	0	3	2	0.000	1.610	1.124	Na+/Pi-cotransporter
bin008	SOY3_bin008_03057	1650	21	48	55	1.522	2.951	3.541	Na+/Pi-cotransporter
bin008	SOY3_bin008_03058	3237	32	233	199	1.182	7.301	6.530	Carbamoyl-phosphate synthase large chain
bin008	SOY3_bin008_03059	1416	12	91	101	1.013	6.518	7.577	Amidophosphoribosyltransferase precursor
bin008	SOY3_bin008_03060	360	2	9	9	0.664	2.536	2.656	hypothetical protein
bin008	SOY3_bin008_03061	2199	110	240	279	5.980	11.070	13.477	putative assembly protein
bin008	SOY3_bin008_03062	342	21	20	27	7.341	5.931	8.386	putative HTH-type transcriptional regulator/MT0088
bin008	SOY3_bin008_03063	1182	155	62	98	15.677	5.320	8.807	Sulfate transporter family protein
bin008	SOY3_bin008_03064	1581	17	98	105	1.285	6.287	7.055	Signal recognition particle receptor FtsY
bin008	SOY3_bin008_03065	96	0	1	1	0.000	1.057	1.107	tRNA-Pro(cgg)
bin008	SOY3_bin008_03066	1251	6	24	32	0.573	1.946	2.717	UDP-N-acetylglucosamine 1-carboxyvinyltransferase
bin008	SOY3_bin008_03067	76	1	1	0	1.573	1.335	0.000	tRNA-His(gtg)
bin008	SOY3_bin008_03068	492	1	7	12	0.243	1.443	2.591	hypothetical protein
bin008	SOY3_bin008_03069	1389	20	37	65	1.721	2.702	4.971	Asparagine--tRNA ligase
bin008	SOY3_bin008_03070	2310	140	370	467	7.245	16.246	21.475	Aerobic respiration control sensor protein ArcB
bin008	SOY3_bin008_03071	717	10	14	8	1.667	1.980	1.185	N-formylglutamate amidohydrolase
bin008	SOY3_bin008_03072	1263	8	30	32	0.757	2.409	2.691	Carboxylate-amine ligase YbdK
bin008	SOY3_bin008_03073	1473	5	39	39	0.406	2.685	2.812	Alpha-aminoadipate--LysW ligase LysX
bin008	SOY3_bin008_03074	744	4	7	9	0.643	0.954	1.285	Peptidase C39 family protein
bin008	SOY3_bin008_03075	981	24	104	113	2.925	10.753	12.236	Flagellar motor switch protein FliM
bin008	SOY3_bin008_03076	177	1	9	6	0.675	5.157	3.601	hypothetical protein
bin008	SOY3_bin008_03077	1428	16	66	92	1.339	4.688	6.844	Transcriptional regulatory protein ZraR
bin008	SOY3_bin008_03078	582	82	543	662	16.844	94.631	120.827	hypothetical protein
bin008	SOY3_bin008_03079	540	2	3	8	0.443	0.563	1.574	hypothetical protein
bin008	SOY3_bin008_03080	423	10	21	17	2.826	5.035	4.269	hypothetical protein
bin008	SOY3_bin008_03081	840	19	41	48	2.704	4.951	6.070	Chaperone protein DnaJ
bin008	SOY3_bin008_03082	492	34	54	72	8.262	11.132	15.545	Flagellin N-methylase
bin008	SOY3_bin008_03083	414	5	5	7	1.444	1.225	1.796	hypothetical protein
bin008	SOY3_bin008_03084	1047	6	26	35	0.685	2.519	3.551	Aminodeoxyfutalosine synthase
bin008	SOY3_bin008_03085	906	1	12	13	0.132	1.343	1.524	putative amino-acid metabolite efflux pump
bin008	SOY3_bin008_03086	1224	13	24	27	1.270	1.989	2.343	Xaa-Pro dipeptidase
bin008	SOY3_bin008_03087	615	2	8	7	0.389	1.319	1.209	FmdE, Molybdenum formylmethanofuran dehydrogenase operon
bin008	SOY3_bin008_03088	648	6	15	18	1.107	2.348	2.951	Kynurenine formamidase
bin008	SOY3_bin008_03089	408	9	54	59	2.637	13.424	15.361	27 kDa antigen Cfp30B
bin008	SOY3_bin008_03090	471	134	252	297	34.012	54.267	66.983	Cytochrome c3
bin008	SOY3_bin008_03091	459	50	172	228	13.023	38.008	52.766	Inosine-5'-monophosphate dehydrogenase
bin008	SOY3_bin008_03092	660	62	100	155	11.230	15.368	24.947	Protein-L-isoaspartate O-methyltransferase
bin008	SOY3_bin008_03093	891	19	36	67	2.549	4.098	7.988	Septum site-determining protein MinD
bin008	SOY3_bin008_03094	609	25	75	93	4.908	12.491	16.222	CDP-diacylglycerol--glycerol-3-phosphate 3-phosphatidyltransferase
bin008	SOY3_bin008_03095	357	41	91	133	13.730	25.854	39.574	hypothetical protein
bin008	SOY3_bin008_03096	327	23	46	53	8.409	14.268	17.217	cell division protein FtsB
bin008	SOY3_bin008_03097	945	69	138	132	8.729	14.812	14.838	hypothetical protein
bin008	SOY3_bin008_03098	819	20	59	55	2.919	7.307	7.134	hypothetical protein
bin008	SOY3_bin008_03099	1014	71	207	264	8.371	20.706	27.656	Fructose-1,6-bisphosphatase class 1
bin008	SOY3_bin008_03100	1095	30	57	64	3.275	5.280	6.209	tRNA N6-adenosine threonylcarbamoyltransferase
bin008	SOY3_bin008_03101	321	36	194	209	13.407	61.299	69.163	Thioredoxin
bin008	SOY3_bin008_03102	924	37	234	239	4.787	25.686	27.476	Thioredoxin reductase
bin008	SOY3_bin008_03103	738	15	94	115	2.430	12.919	16.553	Outer membrane protein assembly factor BamD precursor
bin008	SOY3_bin008_03104	885	15	28	44	2.026	3.209	5.281	hypothetical protein
bin008	SOY3_bin008_03105	2052	52	126	161	3.030	6.228	8.334	Elongation factor G
bin008	SOY3_bin008_03106	2085	63	112	121	3.612	5.448	6.165	DNA ligase

bin008	SOY3_bin008_03107	402	49	74	83	14.572	18.671	21.932	hypothetical protein
bin008	SOY3_bin008_03108	2076	12	52	48	0.691	2.541	2.456	Methyl-accepting chemotaxis protein McpS
bin008	SOY3_bin008_03109	246	3	10	12	1.458	4.123	5.182	hypothetical protein
bin008	SOY3_bin008_03110	384	4	24	25	1.245	6.339	6.916	Phage shock protein C
bin008	SOY3_bin008_03111	984	3	45	44	0.364	4.638	4.750	hypothetical protein
bin008	SOY3_bin008_03112	837	4	11	10	0.571	1.333	1.269	hypothetical protein
bin008	SOY3_bin008_03113	405	43	241	240	12.693	60.355	62.949	hypothetical protein
bin008	SOY3_bin008_03114	675	14	64	57	2.480	9.617	8.970	2-amino-4-deoxychorismate dehydrogenase
bin008	SOY3_bin008_03115	537	16	54	69	3.562	10.199	13.649	Bifunctional protein PyrR
bin008	SOY3_bin008_03116	99	3	35	27	3.623	35.858	28.971	hypothetical protein
bin008	SOY3_bin008_03117	204	7	56	50	4.102	27.843	26.036	hypothetical protein
bin008	SOY3_bin008_03118	336	19	160	174	6.760	48.299	55.010	Iron-sulfur cluster insertion protein ErpA
bin008	SOY3_bin008_03119	333	31	71	80	11.129	21.626	25.520	hypothetical protein
bin008	SOY3_bin008_03120	387	43	57	65	13.283	14.939	17.842	Alkaline phosphatase synthesis transcriptional regulatory protein PhoP
bin008	SOY3_bin008_03121	1725	25	37	53	1.733	2.176	3.264	Cyclic di-GMP phosphodiesterase response regulator RpfG
bin008	SOY3_bin008_03122	468	10	52	53	2.554	11.270	12.030	3-hydroxyacyl-[acyl-carrier-protein] dehydratase FabZ
bin008	SOY3_bin008_03123	507	37	384	462	8.724	76.821	96.797	Chaperone protein Skp precursor
bin008	SOY3_bin008_03124	2664	112	716	802	5.026	27.260	31.979	Outer membrane protein assembly factor BamA precursor
bin008	SOY3_bin008_03125	702	25	233	237	4.257	33.665	35.863	Lipoprotein-releasing system ATP-binding protein LolD
bin008	SOY3_bin008_03126	1230	10	40	33	0.972	3.298	2.850	Lipoprotein-releasing system transmembrane protein LolE
bin008	SOY3_bin008_03127	1590	18	113	100	1.353	7.208	6.681	Lysine--tRNA ligase, heat inducible
bin008	SOY3_bin008_03128	76	7	4	8	11.011	5.338	11.182	tRNA-Asn(gtt)
bin008	SOY3_bin008_03129	666	0	7	9	0.000	1.066	1.435	hypothetical protein
bin008	SOY3_bin008_03130	378	1	1	0	0.316	0.268	0.000	Helix-turn-helix domain protein
bin008	SOY3_bin008_03131	2373	1	5	3	0.050	0.214	0.134	hypothetical protein
bin008	SOY3_bin008_03132	177	0	0	0	0.000	0.000	0.000	hypothetical protein
bin008	SOY3_bin008_03133	489	0	2	0	0.000	0.415	0.000	hypothetical protein
bin008	SOY3_bin008_03134	339	1	2	4	0.353	0.598	1.253	hypothetical protein
bin008	SOY3_bin008_03135	687	2	8	11	0.348	1.181	1.701	hypothetical protein
bin008	SOY3_bin008_03136	309	5	12	18	1.934	3.939	6.188	Antitoxin igA-2
bin008	SOY3_bin008_03137	366	5	11	16	1.633	3.048	4.644	Toxin HigB-2
bin008	SOY3_bin008_03138	1164	4	26	20	0.411	2.266	1.825	Tyrosine recombinase XerD
bin008	SOY3_bin008_03139	327	455	1572	2157	166.345	487.595	700.700	Acid stress chaperone HdeA precursor
bin008	SOY3_bin008_03140	156	1	0	1	0.766	0.000	0.681	hypothetical protein
bin008	SOY3_bin008_03141	1932	30	35	43	1.856	1.837	2.364	Esterase EstP precursor
bin008	SOY3_bin008_03142	77	17	26	39	26.394	34.248	53.803	tRNA-Asp(gtc)
bin008	SOY3_bin008_03143	76	10	22	23	15.730	29.361	32.147	tRNA-Val(tac)
bin008	SOY3_bin008_03144	852	9	19	19	1.263	2.262	2.369	Lipase precursor
bin008	SOY3_bin008_03145	1674	10	18	29	0.714	1.091	1.840	Putative gamma-glutamyltransferase YwrD
bin008	SOY3_bin008_03146	993	0	5	7	0.000	0.511	0.749	Ferrous-iron efflux pump FieF
bin008	SOY3_bin008_03147	1164	2	4	14	0.205	0.349	1.278	FtsX-like permease family protein
bin008	SOY3_bin008_03148	723	3	9	8	0.496	1.263	1.175	putative ABC transporter ATP-binding protein/MT1014
bin008	SOY3_bin008_03149	993	0	5	7	0.000	0.511	0.749	Macrolide export protein MacA
bin008	SOY3_bin008_03150	1479	3	3	9	0.242	0.206	0.646	Outer membrane protein OprM precursor
bin008	SOY3_bin008_03151	1614	77	139	161	5.703	8.735	10.596	putative diguanylate cyclase YcdT
bin008	SOY3_bin008_03152	1278	7	44	56	0.655	3.492	4.655	tRNA (guanine-N(1)-)-methyltransferase
bin008	SOY3_bin008_03153	351	9	147	177	3.065	42.478	53.567	50S ribosomal protein L19
bin008	SOY3_bin008_03154	633	1	9	11	0.189	1.442	1.846	Ribonuclease HII
bin008	SOY3_bin008_03155	411	7	4	12	2.036	0.987	3.101	hypothetical protein
bin008	SOY3_bin008_03156	438	45	124	113	12.282	28.715	27.405	hypothetical protein
bin008	SOY3_bin008_03157	76	0	0	0	0.000	0.000	0.000	tRNA-Phe(gaa)
bin008	SOY3_bin008_03158	543	0	2	2	0.000	0.374	0.391	hypothetical protein
bin008	SOY3_bin008_03159	690	28	116	148	4.851	17.052	22.785	hypothetical protein
bin008	SOY3_bin008_03160	141	2	11	6	1.696	7.913	4.520	hypothetical protein
bin008	SOY3_bin008_03161	1092	10	36	54	1.095	3.344	5.253	Penicillin acylase precursor
bin008	SOY3_bin008_03162	615	36	137	154	6.998	22.594	26.600	hypothetical protein
bin008	SOY3_bin008_03163	624	8	18	18	1.533	2.926	3.064	hypothetical protein
bin008	SOY3_bin008_03164	2307	21	145	182	1.088	6.375	8.380	Methyl-accepting chemotaxis protein McpS
bin008	SOY3_bin008_03165	1182	19	59	74	1.922	5.063	6.650	Phosphoribosylglycinamide formyltransferase 2
bin008	SOY3_bin008_03166	1161	1	7	10	0.103	0.612	0.915	N-methyltryptophan oxidase
bin008	SOY3_bin008_03167	4161	131	663	719	3.764	16.161	18.355	DNA-directed RNA polymerase subunit beta'
bin008	SOY3_bin008_03168	4113	83	472	443	2.412	11.640	11.441	DNA-directed RNA polymerase subunit beta
bin008	SOY3_bin008_03169	390	24	489	485	7.357	127.174	132.101	50S ribosomal protein L7/L12
bin008	SOY3_bin008_03170	522	38	575	592	8.703	111.725	120.471	50S ribosomal protein L10
bin008	SOY3_bin008_03171	708	31	304	289	5.234	43.551	43.361	50S ribosomal protein L1
bin008	SOY3_bin008_03172	423	20	189	173	5.652	45.319	43.445	50S ribosomal protein L11
bin008	SOY3_bin008_03173	564	21	130	162	4.451	23.379	30.512	hypothetical protein

bin008	SOY3_bin008_03174	324	20	140	164	7.380	43.827	53.769	preprotein translocase subunit SecE
bin008	SOY3_bin008_03175	76	1	5	2	1.573	6.673	2.795	tRNA-Trp(cca)
bin008	SOY3_bin008_03176	150	14	125	118	11.158	84.523	83.564	50S ribosomal protein L33
bin008	SOY3_bin008_03177	138	13	119	115	11.262	87.463	88.521	Elongation factor Tu
bin008	SOY3_bin008_03178	1539	5	56	46	0.388	3.691	3.175	Methyl-accepting chemotaxis protein PctC
bin008	SOY3_bin008_03179	1986	8	34	42	0.482	1.736	2.246	Hydrogenase-4 component B
bin008	SOY3_bin008_03180	897	0	8	11	0.000	0.905	1.303	Formate hydrogenlyase subunit 4
bin008	SOY3_bin008_03181	642	0	12	11	0.000	1.896	1.820	Hydrogenase-4 component E
bin008	SOY3_bin008_03182	1479	6	15	13	0.485	1.029	0.934	Hydrogenase-4 component B
bin008	SOY3_bin008_03183	1485	17	35	59	1.369	2.391	4.220	Formate hydrogenlyase subunit 5 precursor
bin008	SOY3_bin008_03184	768	1	14	10	0.156	1.849	1.383	Formate hydrogenlyase subunit 7
bin008	SOY3_bin008_03185	624	184	382	457	35.252	62.092	77.797	hypothetical protein
bin008	SOY3_bin008_03186	1779	42	188	270	2.822	10.719	16.122	hypothetical protein
bin008	SOY3_bin008_03187	552	58	276	362	12.561	50.714	69.663	hypothetical protein
bin008	SOY3_bin008_03188	1128	8	58	44	0.848	5.215	4.144	Isopenicillin N epimerase
bin008	SOY3_bin008_03189	1320	4	49	49	0.362	3.765	3.943	Phenylacetate-coenzyme A ligase
bin008	SOY3_bin008_03190	744	2	25	17	0.321	3.408	2.427	High-affinity branched-chain amino acid transport ATP-binding protein LivF
bin008	SOY3_bin008_03191	786	0	15	15	0.000	1.936	2.027	Lipopolysaccharide export system ATP-binding protein LptB
bin008	SOY3_bin008_03192	978	0	15	12	0.000	1.556	1.303	High-affinity branched-chain amino acid transport system permease protein LivH
bin008	SOY3_bin008_03193	876	2	30	23	0.273	3.474	2.789	High-affinity branched-chain amino acid transport system permease protein LivH
bin008	SOY3_bin008_03194	1143	6	83	88	0.628	7.365	8.178	Leucine-, isoleucine-, valine-, threonine-, and alanine-binding protein precursor
bin008	SOY3_bin008_03195	498	18	102	146	4.321	20.774	31.142	hypothetical protein
bin008	SOY3_bin008_03196	951	15	35	48	1.886	3.733	5.362	Ribosomal large subunit pseudouridine synthase C
bin008	SOY3_bin008_03197	705	10	24	35	1.696	3.453	5.274	Putative TrmH family tRNA/rRNA methyltransferase
bin008	SOY3_bin008_03198	1995	45	122	136	2.697	6.203	7.241	Putative glycosyltransferase EpsH
bin008	SOY3_bin008_03199	2058	46	131	154	2.672	6.456	7.949	N-acetylglucosaminyl-diphospho-decaprenol L-rhamnosyltransferase
bin008	SOY3_bin008_03200	990	27	75	102	3.260	7.684	10.944	D-inositol-3-phosphate glycosyltransferase
bin008	SOY3_bin008_03201	3219	46	269	283	1.708	8.476	9.339	Glycosyl transferase family 2
bin008	SOY3_bin008_03202	1605	30	114	114	2.235	7.204	7.545	putative glycosyltransferase EpsJ
bin008	SOY3_bin008_03203	1224	20	109	128	1.953	9.032	11.109	Glycogen synthase
bin008	SOY3_bin008_03204	924	31	91	122	4.011	9.989	14.025	UDP-glucose 4-epimerase
bin008	SOY3_bin008_03205	231	1	9	9	0.518	3.952	4.139	hypothetical protein
bin008	SOY3_bin008_03206	669	11	60	68	1.966	9.097	10.797	Transposase zinc-ribbon domain protein
bin008	SOY3_bin008_03207	585	8	38	43	1.635	6.588	7.808	Transposase DDE domain protein
bin008	SOY3_bin008_03208	1473	51	122	172	4.139	8.401	12.404	Alginate biosynthesis protein AlgA
bin008	SOY3_bin008_03209	759	24	49	78	3.780	6.548	10.916	phosphoglycolate phosphatase
bin008	SOY3_bin008_03210	591	5	25	25	1.011	4.290	4.493	GDP-mannose 4,6-dehydratase
bin008	SOY3_bin008_03211	1563	12	22	23	0.918	1.428	1.563	Tn3 transposase DDE domain protein
bin008	SOY3_bin008_03212	1395	18	21	26	1.543	1.527	1.980	hypothetical protein
bin008	SOY3_bin008_03213	738	4	19	21	0.648	2.611	3.023	T-protein
bin008	SOY3_bin008_03214	1419	5	33	47	0.421	2.359	3.518	Anthranilate synthase component 1
bin008	SOY3_bin008_03215	579	1	9	16	0.206	1.577	2.935	Anthranilate synthase component 2
bin008	SOY3_bin008_03216	384	3	10	15	0.934	2.641	4.149	HEPN domain protein
bin008	SOY3_bin008_03217	1011	9	27	32	1.064	2.709	3.362	Anthranilate phosphoribosyltransferase
bin008	SOY3_bin008_03218	774	2	13	22	0.309	1.704	3.019	Indole-3-glycerol phosphate synthase
bin008	SOY3_bin008_03219	621	5	30	38	0.963	4.900	6.500	N-(5'-phosphoribosyl)anthranilate isomerase
bin008	SOY3_bin008_03220	1188	18	37	47	1.811	3.159	4.203	Tryptophan synthase beta chain
bin008	SOY3_bin008_03221	771	20	33	47	3.101	4.341	6.475	Tryptophan synthase alpha chain
bin008	SOY3_bin008_03222	621	30	49	64	5.775	8.003	10.948	Homoserine/homoserine lactone efflux protein
bin008	SOY3_bin008_03223	1101	76	140	190	8.252	12.897	18.331	Hydrogenase transcriptional regulatory protein hupR1
bin008	SOY3_bin008_03224	411	41	93	116	11.926	22.951	29.981	Chemotaxis protein CheY
bin008	SOY3_bin008_03225	657	16	53	59	2.911	8.182	9.539	hypothetical protein
bin008	SOY3_bin008_03226	1383	17	17	19	1.470	1.247	1.459	Multidrug resistance protein stp
bin008	SOY3_bin008_03227	621	6	7	20	1.155	1.143	3.421	hypothetical protein
bin008	SOY3_bin008_03228	735	2	11	15	0.325	1.518	2.168	hypothetical protein
bin008	SOY3_bin008_03229	1428	38	249	245	3.181	17.686	18.225	Pyruvate kinase
bin008	SOY3_bin008_03230	339	13	53	73	4.584	15.857	22.875	hypothetical protein
bin008	SOY3_bin008_03231	1053	19	94	120	2.157	9.054	12.105	N-acetyl-gamma-glutamyl-phosphate reductase
bin008	SOY3_bin008_03232	246	3	12	11	1.458	4.948	4.750	hypothetical protein
bin008	SOY3_bin008_03233	693	18	36	55	3.105	5.269	8.431	hypothetical protein
bin008	SOY3_bin008_03234	657	75	174	216	13.647	26.862	34.924	Cobalt-precorrin-8X methylmutase
bin008	SOY3_bin008_03235	867	1	5	5	0.138	0.585	0.613	aromatic amino acid exporter
bin008	SOY3_bin008_03236	1299	0	16	26	0.000	1.249	2.126	Phenylacetate-coenzyme A ligase
bin008	SOY3_bin008_03237	636	0	1	0	0.000	0.159	0.000	Phosphate import ATP-binding protein PstB
bin008	SOY3_bin008_03238	666	0	1	2	0.000	0.152	0.319	Phosphate-specific transport system accessory protein PhoU
bin008	SOY3_bin008_03239	1110	4	11	19	0.431	1.005	1.818	HPr kinase/phosphorylase
bin008	SOY3_bin008_03240	1212	5	10	8	0.493	0.837	0.701	Gluconeogenesis factor

bin008	SOY3_bin008_03241	2718	42	83	80	1.847	3.097	3.127	Histidine protein kinase DivJ
bin008	SOY3_bin008_03242	582	3	29	41	0.616	5.054	7.483	hypothetical protein
bin008	SOY3_bin008_03243	990	3	13	10	0.362	1.332	1.073	Cyclic di-GMP phosphodiesterase response regulator RpfG
bin008	SOY3_bin008_03244	777	2	4	7	0.308	0.522	0.957	Ion channel
bin008	SOY3_bin008_03245	1245	5	23	26	0.480	1.874	2.218	Nucleoside permease NupX
bin008	SOY3_bin008_03246	804	3	25	20	0.446	3.154	2.642	Sulfite exporter TauE/SafE
bin008	SOY3_bin008_03247	1362	15	43	57	1.317	3.202	4.446	hypothetical protein
bin008	SOY3_bin008_03248	261	93	324	417	42.598	125.910	169.717	hypothetical protein
bin008	SOY3_bin008_03249	420	7	33	34	1.992	7.969	8.599	S-adenosyl-L-methionine-binding protein
bin008	SOY3_bin008_03250	864	9	27	26	1.245	3.170	3.197	EamA-like transporter family protein
bin008	SOY3_bin008_03251	963	47	75	87	5.835	7.899	9.597	Thiamine-monophosphate kinase
bin008	SOY3_bin008_03252	666	4	8	9	0.718	1.218	1.435	D-glycero-beta-D-manno-heptose-1,7-bisphosphate 7-phosphatase
bin008	SOY3_bin008_03253	1068	6	18	15	0.672	1.709	1.492	tRNA-specific 2-thiouridylase MnmA
bin008	SOY3_bin008_03254	1473	19	50	68	1.542	3.443	4.904	Glutamyl-tRNA(Gln) amidotransferase subunit A
bin008	SOY3_bin008_03255	285	4	38	29	1.678	13.524	10.809	Glutamyl-tRNA(Gln) amidotransferase subunit C
bin008	SOY3_bin008_03256	1287	32	136	145	2.972	10.718	11.968	Penicillin-binding protein activator LpoA
bin008	SOY3_bin008_03257	687	1	8	14	0.174	1.181	2.165	hypothetical protein
bin008	SOY3_bin008_03258	2151	31	87	92	1.723	4.102	4.543	30S ribosomal protein S1
bin008	SOY3_bin008_03259	798	6	9	20	0.899	1.144	2.662	hypothetical protein
bin008	SOY3_bin008_03260	708	6	34	35	1.013	4.871	5.251	hypothetical protein
bin008	SOY3_bin008_03261	1377	11	36	37	0.955	2.652	2.854	GSCFA family protein
bin008	SOY3_bin008_03262	450	0	0	3	0.000	0.000	0.708	hypothetical protein
bin008	SOY3_bin008_03263	804	327	453	617	48.622	57.147	81.519	4'-phosphopantetheinyl transferase superfamily protein
bin008	SOY3_bin008_03264	1347	110	213	256	9.763	16.039	20.188	3-oxoacyl-[acyl-carrier-protein] synthase 2
bin008	SOY3_bin008_03265	249	43	101	119	20.645	41.141	50.767	Polyketide biosynthesis acyl-carrier-protein AcpK
bin008	SOY3_bin008_03266	1071	28	48	76	3.125	4.546	7.538	hypothetical protein
bin008	SOY3_bin008_03267	1290	5	27	33	0.463	2.123	2.717	3-aminobutyryl-CoA aminotransferase
bin008	SOY3_bin008_03268	1131	13	40	57	1.374	3.587	5.354	Lysine 6-dehydrogenase
bin008	SOY3_bin008_03269	1512	18	32	56	1.423	2.147	3.934	Benzoate--CoA ligase
bin008	SOY3_bin008_03270	249	15	31	33	7.202	12.627	14.078	hypothetical protein
bin008	SOY3_bin008_03271	1668	0	4	2	0.000	0.243	0.127	hypothetical protein
bin008	SOY3_bin008_03272	825	0	0	1	0.000	0.000	0.129	hypothetical protein
bin008	SOY3_bin008_03273	321	1	7	6	0.372	2.212	1.986	Helix-turn-helix domain protein
bin008	SOY3_bin008_03274	1092	8	12	23	0.876	1.115	2.237	Signal transduction histidine-protein kinase BaeS
bin008	SOY3_bin008_03275	687	16	33	51	2.784	4.872	7.886	Transcriptional regulatory protein BaeR
bin008	SOY3_bin008_03276	1158	11	67	80	1.136	5.868	7.339	Solvent efflux pump periplasmic linker SrpA precursor
bin008	SOY3_bin008_03277	2094	14	94	109	0.799	4.553	5.529	Multidrug export protein AcrF
bin008	SOY3_bin008_03278	924	1	22	17	0.129	2.415	1.954	Multidrug resistance protein MexB
bin008	SOY3_bin008_03279	402	2	6	3	0.595	1.514	0.793	hypothetical protein
bin008	SOY3_bin008_03280	765	8	25	37	1.250	3.315	5.138	Iron-sulfur flavoprotein
bin008	SOY3_bin008_03281	771	6	10	12	0.930	1.316	1.653	Regulatory protein SdiA
bin008	SOY3_bin008_03282	1077	38	260	303	4.218	24.486	29.885	Choloylglycine hydrolase
bin008	SOY3_bin008_03283	459	9	28	34	2.344	6.187	7.869	Transposase DDE domain protein
bin008	SOY3_bin008_03284	1302	211	1534	1806	19.374	119.500	147.345	Glycolipid anchored surface protein (GAS1)
bin008	SOY3_bin008_03285	297	3	6	18	1.208	2.049	6.438	Transposase
bin008	SOY3_bin008_03286	708	11	18	23	1.857	2.579	3.451	Serine/threonine phosphatase stp
bin008	SOY3_bin008_03287	1791	30	74	64	2.002	4.191	3.796	Serine/threonine-protein kinase PK-1
bin008	SOY3_bin008_03288	279	8	37	42	3.428	13.451	15.991	hypothetical protein
bin008	SOY3_bin008_03289	1248	2	11	11	0.192	0.894	0.936	hypothetical protein
bin008	SOY3_bin008_03290	4815	15	39	40	0.372	0.822	0.882	tetratricopeptide repeat protein
bin008	SOY3_bin008_03291	1167	33	58	60	3.381	5.041	5.461	Polysaccharide deacetylase
bin008	SOY3_bin008_03292	1302	21	55	55	1.928	4.285	4.487	Alginate lyase precursor
bin008	SOY3_bin008_03293	1665	39	51	68	2.800	3.107	4.338	Poly-beta-1,6-N-acetyl-D-glucosamine synthase
bin008	SOY3_bin008_03294	95	1	1	2	1.258	1.068	2.236	tRNA-Ser(gct)
bin008	SOY3_bin008_03295	1173	37	260	310	3.771	22.482	28.073	Glutamate-pyruvate aminotransferase AlaC
bin008	SOY3_bin008_03296	1293	59	141	146	5.455	11.061	11.995	Homoserine dehydrogenase
bin008	SOY3_bin008_03297	909	202	251	298	26.566	28.007	34.824	ADP-ribosyl-[dinitrogen reductase] glycohydrolase
bin008	SOY3_bin008_03298	330	27	62	69	9.781	19.056	22.211	hypothetical protein
bin008	SOY3_bin008_03299	204	13	16	23	7.618	7.955	11.976	hypothetical protein
bin008	SOY3_bin008_03300	369	12	28	28	3.888	7.696	8.060	Sporulation initiation phosphotransferase F
bin008	SOY3_bin008_03301	762	14	71	88	2.196	9.451	12.268	putative ABC transporter ATP-binding protein
bin008	SOY3_bin008_03302	1158	28	156	173	2.891	13.664	15.870	FeS cluster assembly protein SufB
bin008	SOY3_bin008_03303	1359	15	96	113	1.320	7.165	8.833	23S rRNA (uracil-C(5))-methyltransferase RlmCD
bin008	SOY3_bin008_03304	1755	14	80	77	0.954	4.623	4.661	Sensor protein ZraS
bin008	SOY3_bin008_03305	474	2	17	23	0.504	3.638	5.154	Pyridoxamine 5'-phosphate oxidase
bin008	SOY3_bin008_03306	705	7	18	14	1.187	2.590	2.109	GMP synthase [glutamine-hydrolyzing]
bin008	SOY3_bin008_03307	2736	132	318	437	5.768	11.789	16.967	Autoinducer 2 sensor kinase/phosphatase LuxQ

bin008	SOY3_bin008_03308	825	29	84	99	4.202	10.327	12.747	2-amino-4,5-dihydroxy-6-one-heptanoic acid-7-phosphate synthase
bin008	SOY3_bin008_03309	1293	9	28	29	0.832	2.196	2.382	Ribosomal RNA small subunit methyltransferase F
bin008	SOY3_bin008_03310	675	2	6	2	0.354	0.902	0.315	Inner membrane protein YghB
bin008	SOY3_bin008_03311	1644	22	179	143	1.600	11.043	9.240	FG-GAP repeat protein
bin008	SOY3_bin008_03312	870	2	3	6	0.275	0.350	0.733	High-affinity branched-chain amino acid transport system permease protein LivH
bin008	SOY3_bin008_03313	909	2	7	6	0.263	0.781	0.701	High-affinity branched-chain amino acid transport system permease protein LivH
bin008	SOY3_bin008_03314	1170	4	42	55	0.409	3.641	4.994	hypothetical protein
bin008	SOY3_bin008_03315	303	103	891	873	40.639	298.256	306.056	RNA recognition motif. (a.k.a. RRM, RBD, or RNP domain)
bin008	SOY3_bin008_03316	588	8	37	39	1.627	6.382	7.046	NYN domain protein
bin008	SOY3_bin008_03317	420	0	2	1	0.000	0.483	0.253	hypothetical protein
bin008	SOY3_bin008_03318	1698	8	38	44	0.563	2.270	2.753	Carbon starvation protein A
bin008	SOY3_bin008_03319	480	0	2	0	0.000	0.423	0.000	formate dehydrogenase accessory protein
bin008	SOY3_bin008_03320	699	5	13	10	0.855	1.886	1.520	UDP-3-O-[3-hydroxymyristoyl] glucosamine N-acyltransferase
bin008	SOY3_bin008_03321	1269	4	9	24	0.377	0.719	2.009	putative MFS-type transporter YhjX
bin008	SOY3_bin008_03322	2694	5	15	30	0.222	0.565	1.183	Formate dehydrogenase H
bin008	SOY3_bin008_03323	1470	3	18	12	0.244	1.242	0.867	NADP-reducing hydrogenase subunit HndC
bin008	SOY3_bin008_03324	381	0	5	2	0.000	1.331	0.558	NADP-reducing hydrogenase subunit HndB
bin008	SOY3_bin008_03325	222	0	3	1	0.000	1.371	0.478	NADP-reducing hydrogenase subunit HndA
bin008	SOY3_bin008_03326	345	4	2	5	1.386	0.588	1.540	Proline dehydrogenase
bin008	SOY3_bin008_03327	1044	8	68	69	0.916	6.606	7.021	Proline dehydrogenase 1
bin008	SOY3_bin008_03328	228	9	8	15	4.719	3.559	6.989	hypothetical protein
bin008	SOY3_bin008_03329	2430	121	142	229	5.953	5.927	10.011	Glycosyl hydrolase family 57
bin008	SOY3_bin008_03330	2820	152	204	253	6.444	7.337	9.530	Maltooligosyl trehalose synthase
bin008	SOY3_bin008_03331	1941	100	149	248	6.159	7.786	13.572	Malto-oligosyltrehalose trehalohydrolase
bin008	SOY3_bin008_03332	1776	27	49	83	1.817	2.798	4.964	Miniconductance mechanosensitive channel MscM precursor
bin008	SOY3_bin008_03333	1164	117	268	288	12.017	23.353	26.283	hypothetical protein
bin008	SOY3_bin008_03334	666	9	20	19	1.616	3.046	3.030	hypothetical protein
bin008	SOY3_bin008_03335	828	3	9	16	0.433	1.102	2.053	Inward rectifier potassium channel Kirbac3.1
bin008	SOY3_bin008_03336	77	0	0	0	0.000	0.000	0.000	tRNA-Arg(acg)
bin008	SOY3_bin008_03337	765	12	34	40	1.875	4.508	5.554	Enoyl-[acyl-carrier-protein] reductase [NADH] FabI
bin008	SOY3_bin008_03338	636	10	24	31	1.880	3.827	5.178	HTH-type transcriptional repressor FabR
bin008	SOY3_bin008_03339	420	10	14	10	2.846	3.381	2.529	Methylamine utilization protein MauE
bin008	SOY3_bin008_03340	507	6	23	16	1.415	4.601	3.352	Thiosulfate sulfurtransferase PspE precursor
bin008	SOY3_bin008_03341	849	23	99	97	3.239	11.827	12.137	putative adenylyltransferase/sulfurtransferase MoeZ
bin008	SOY3_bin008_03342	408	18	41	44	5.274	10.192	11.456	Inner membrane protein YccF
bin008	SOY3_bin008_03343	444	26	45	48	7.001	10.280	11.484	Inner membrane protein YdcZ
bin008	SOY3_bin008_03344	1113	13	12	20	1.396	1.094	1.909	Acyltransferase family protein
bin008	SOY3_bin008_03345	219	1	5	11	0.546	2.316	5.336	putative tautomerase
bin008	SOY3_bin008_03346	264	0	3	3	0.000	1.153	1.207	anaerobic benzoate catabolism transcriptional regulator
bin008	SOY3_bin008_03347	555	1	3	5	0.215	0.548	0.957	hypothetical protein
bin008	SOY3_bin008_03348	879	117	566	632	15.913	65.310	76.376	Right origin-binding protein
bin008	SOY3_bin008_03349	504	36	82	101	8.539	16.502	21.287	N-acyltransferase YncA
bin008	SOY3_bin008_03350	1005	16	47	77	1.903	4.743	8.139	inner membrane protein
bin008	SOY3_bin008_03351	1506	18	251	249	1.429	16.905	17.563	30S ribosomal protein S1
bin008	SOY3_bin008_03352	1686	12	60	60	0.851	3.610	3.780	putative ABC transporter ATP-binding protein
bin008	SOY3_bin008_03353	1641	40	442	416	2.914	27.319	26.929	hypothetical protein
bin008	SOY3_bin008_03354	573	7	14	18	1.460	2.478	3.337	hypothetical protein
bin008	SOY3_bin008_03355	348	1	1	0	0.344	0.291	0.000	hypothetical protein
bin008	SOY3_bin008_03356	924	5	71	63	0.647	7.794	7.243	Hydrogen peroxide-inducible genes activator
bin008	SOY3_bin008_03357	873	6	62	73	0.822	7.203	8.883	hypothetical protein
bin008	SOY3_bin008_03358	255	0	23	35	0.000	9.148	14.580	putative transcriptional regulator
bin008	SOY3_bin008_03359	801	9	33	36	1.343	4.179	4.774	Sirohydrochlorin cobaltochelatase CbiKC
bin008	SOY3_bin008_03360	864	7	28	33	0.969	3.287	4.057	Cell division protein FtsX
bin008	SOY3_bin008_03361	693	4	11	14	0.690	1.610	2.146	Cell division ATP-binding protein FtsE
bin008	SOY3_bin008_03362	2232	9	54	71	0.482	2.454	3.379	Ribonucleoside-diphosphate reductase NrdZ
bin008	SOY3_bin008_03363	273	9	27	35	3.941	10.031	13.619	hypothetical protein
bin008	SOY3_bin008_03364	1950	111	351	399	6.805	18.257	21.735	Molybdopterin molybdenumtransferase
bin008	SOY3_bin008_03365	888	27	103	141	3.635	11.765	16.867	N-carbamoyl-D-amino acid hydrolase
bin008	SOY3_bin008_03366	624	4	24	31	0.766	3.901	5.277	LysE type translocator
bin008	SOY3_bin008_03367	981	22	69	77	2.681	7.134	8.338	Glucokinase
bin008	SOY3_bin008_03368	1236	27	109	110	2.612	8.945	9.454	Molybdopterin molybdenumtransferase
bin008	SOY3_bin008_03369	273	5	14	19	2.190	5.201	7.393	hypothetical protein
bin008	SOY3_bin008_03370	333	4	8	15	1.436	2.437	4.785	hypothetical protein
bin008	SOY3_bin008_03371	1116	8	24	37	0.857	2.181	3.522	hypothetical protein
bin008	SOY3_bin008_03372	192	24	177	211	14.944	93.503	116.738	hypothetical protein
bin008	SOY3_bin008_03373	2640	5	37	36	0.226	1.422	1.449	Type III restriction enzyme, res subunit
bin008	SOY3_bin008_03374	498	2	10	6	0.480	2.037	1.280	hypothetical protein

bin008	SOY3_bin008_03375	576	0	6	15	0.000	1.057	2.766	hypothetical protein
bin008	SOY3_bin008_03376	474	1	2	3	0.252	0.428	0.672	hypothetical protein
bin008	SOY3_bin008_03377	456	0	2	2	0.000	0.445	0.466	hypothetical protein
bin008	SOY3_bin008_03378	669	2	1	6	0.357	0.152	0.953	hypothetical protein
bin008	SOY3_bin008_03379	2517	3	3	11	0.142	0.121	0.464	hypothetical protein
bin008	SOY3_bin008_03380	234	2	0	3	1.022	0.000	1.362	Helix-turn-helix domain protein
bin008	SOY3_bin008_03381	819	3	11	15	0.438	1.362	1.946	hypothetical protein
bin008	SOY3_bin008_03382	270	6	11	14	2.657	4.132	5.508	PemK-like protein
bin008	SOY3_bin008_03383	258	14	14	21	6.487	5.504	8.646	Phd_YefM
bin008	SOY3_bin008_03384	1188	6	19	22	0.604	1.622	1.967	Tyrosine recombinase XerD
bin008	SOY3_bin008_03385	1428	5	22	21	0.419	1.563	1.562	hypothetical protein
bin008	SOY3_bin008_03386	939	18	49	80	2.292	5.293	9.050	ribonuclease Z
bin008	SOY3_bin008_03387	1626	22	94	119	1.618	5.864	7.774	Benzoylformate decarboxylase
bin008	SOY3_bin008_03388	351	21	56	55	7.152	16.182	16.645	Oxalate decarboxylase OxdC
bin008	SOY3_bin008_03389	798	11	25	39	1.648	3.178	5.191	hypothetical protein
bin008	SOY3_bin008_03390	1983	69	116	157	4.160	5.933	8.410	Acetone carboxylase beta subunit
bin008	SOY3_bin008_03391	1152	50	143	171	5.189	12.590	15.768	Isocitrate dehydrogenase [NADP]
bin008	SOY3_bin008_03392	429	13	10	23	3.623	2.364	5.695	hypothetical protein
bin008	SOY3_bin008_03393	1377	38	93	118	3.299	6.850	9.103	Murein hydrolase activator EnvC precursor
bin008	SOY3_bin008_03394	513	154	589	703	35.888	116.454	145.569	Outer membrane protein P6 precursor
bin008	SOY3_bin008_03395	1317	125	265	338	11.347	20.409	27.262	translocation protein TolB
bin008	SOY3_bin008_03396	648	15	26	41	2.767	4.070	6.721	Tetracycline repressor protein class A from transposon 1721
bin008	SOY3_bin008_03397	1032	10	13	21	1.158	1.278	2.162	putative diguanylate cyclase AdrA
bin008	SOY3_bin008_03398	750	14	14	12	2.232	1.893	1.700	L-2,4-diaminobutyrate decarboxylase
bin008	SOY3_bin008_03399	216	8	56	68	4.428	26.296	33.441	hypothetical protein
bin008	SOY3_bin008_03400	378	2	6	2	0.633	1.610	0.562	HTH-type transcriptional repressor SmtB
bin008	SOY3_bin008_03401	990	6	11	13	0.725	1.127	1.395	putative quorum-quenching lactonase YtnP
bin008	SOY3_bin008_03402	3063	26	21	28	1.015	0.695	0.971	Multidrug export protein AcrF
bin008	SOY3_bin008_03403	1128	2	12	6	0.212	1.079	0.565	Multidrug resistance protein MdtE precursor
bin008	SOY3_bin008_03404	633	0	7	3	0.000	1.122	0.503	HTH-type transcriptional regulator TtgR
bin008	SOY3_bin008_03405	426	15	6	19	4.209	1.429	4.738	Tyrosine recombinase XerC
bin008	SOY3_bin008_03406	1848	119	182	251	7.698	9.989	14.428	Methyl-accepting chemotaxis protein McpS
bin008	SOY3_bin008_03407	564	192	269	357	40.697	48.376	67.239	hypothetical protein
bin008	SOY3_bin008_03408	783	38	123	142	5.802	15.933	19.264	Transposase DDE domain protein
bin008	SOY3_bin008_03409	77	3	1	0	4.658	1.317	0.000	tRNA-Pro(tgg)
bin008	SOY3_bin008_03410	1017	74	101	152	8.699	10.073	15.876	Benzylsuccinate synthase activating enzyme
bin008	SOY3_bin008_03411	372	64	148	161	20.568	40.353	45.974	hypothetical protein
bin008	SOY3_bin008_03412	1071	61	202	213	6.809	19.130	21.126	Phosphoribosylformylglycinamidine cyclo-ligase
bin008	SOY3_bin008_03413	1302	21	82	74	1.928	6.388	6.037	molybdenum cofactor biosynthesis protein A
bin008	SOY3_bin008_03414	426	3	5	7	0.842	1.190	1.745	Putative redox-active protein (C_GCAxxG_C_C)
bin008	SOY3_bin008_03415	393	14	243	265	4.259	62.715	71.628	30S ribosomal protein S9
bin008	SOY3_bin008_03416	429	8	199	182	2.229	47.049	45.065	50S ribosomal protein L13
bin008	SOY3_bin008_03417	1260	19	55	51	1.803	4.427	4.300	5'-nucleotidase
bin008	SOY3_bin008_03418	300	4	1	2	1.594	0.338	0.708	HTH-type transcriptional repressor YcgE
bin008	SOY3_bin008_03419	2181	18	160	166	0.987	7.441	8.085	Glutamine synthetase
bin008	SOY3_bin008_03420	1764	30	81	84	2.033	4.657	5.058	hypothetical protein
bin008	SOY3_bin008_03421	1002	9	81	89	1.074	8.199	9.435	IgA-specific serine endopeptidase autotransporter precursor
bin008	SOY3_bin008_03422	594	1	5	3	0.201	0.854	0.536	Glutaconyl-CoA decarboxylase subunit beta
bin008	SOY3_bin008_03423	2751	26	62	60	1.130	2.286	2.317	Multifunctional CCA protein
bin008	SOY3_bin008_03424	1308	28	65	81	2.559	5.040	6.578	Histidinol dehydrogenase
bin008	SOY3_bin008_03425	894	24	104	100	3.209	11.799	11.882	Phosphoribosylaminoimidazole-succinocarboxamide synthase
bin008	SOY3_bin008_03426	90	4	4	6	5.313	4.508	7.082	tRNA-Ser(gga)
bin008	SOY3_bin008_03427	627	14	22	32	2.669	3.559	5.421	Orotate phosphoribosyltransferase
bin008	SOY3_bin008_03428	1023	34	59	72	3.973	5.850	7.476	General stress protein 69
bin008	SOY3_bin008_03429	1311	16	21	35	1.459	1.625	2.836	Enterobactin exporter EntS
bin008	SOY3_bin008_03430	480	37	88	99	9.215	18.595	21.909	HTH-type transcriptional repressor NicR
bin008	SOY3_bin008_03431	2076	93	132	169	5.356	6.449	8.647	hypothetical protein
bin008	SOY3_bin008_03432	1329	31	80	106	2.789	6.105	8.472	succinate dehydrogenase/fumarate reductase iron-sulfur subunit
bin008	SOY3_bin008_03433	1611	1	18	14	0.074	1.133	0.923	Diguanylate cyclase DosC
bin008	SOY3_bin008_03434	303	111	134	189	43.795	44.856	66.260	transcriptional repressor DicA
bin008	SOY3_bin008_03435	2040	62	65	85	3.633	3.232	4.426	Blue-light-activated protein
bin008	SOY3_bin008_03436	2538	109	52	118	5.134	2.078	4.939	4-hydroxyphenylacetate decarboxylase large subunit
bin008	SOY3_bin008_03437	411	11	13	18	3.200	3.208	4.652	hypothetical protein
bin008	SOY3_bin008_03438	1017	16	15	34	1.881	1.496	3.551	Benzylsuccinate synthase activating enzyme
bin008	SOY3_bin008_03439	453	0	2	5	0.000	0.448	1.172	hypothetical protein
bin008	SOY3_bin008_03440	1107	10	36	37	1.080	3.298	3.550	Ribosome-binding ATPase YchF
bin008	SOY3_bin008_03441	636	12	47	54	2.256	7.495	9.019	hypothetical protein

bin008	SOY3_bin008_03442	516	205	677	871	47.495	133.074	179.307	putative thiol peroxidase
bin008	SOY3_bin008_03443	651	6	79	63	1.102	12.308	10.280	D-alanyl-D-alanine dipeptidase
bin008	SOY3_bin008_03444	2595	22	95	70	1.014	3.713	2.865	Wide host range VirA protein
bin008	SOY3_bin008_03445	1260	11	42	41	1.044	3.381	3.457	Response regulator PleD
bin008	SOY3_bin008_03446	324	6	27	26	2.214	8.452	8.524	hypothetical protein
bin008	SOY3_bin008_03447	846	2	18	21	0.283	2.158	2.637	4-hydroxybenzoate octaprenyltransferase
bin008	SOY3_bin008_03448	1200	7	126	112	0.697	10.650	9.914	Acetylornithine aminotransferase
bin008	SOY3_bin008_03449	2688	34	66	65	1.512	2.490	2.569	putative sensor histidine kinase pdtaS
bin008	SOY3_bin008_03450	1314	17	66	53	1.547	5.095	4.285	Histone deacetylase-like amidohydrolase
bin008	SOY3_bin008_03451	1683	60	89	109	4.262	5.364	6.880	Acetophenone carboxylase gamma subunit
bin008	SOY3_bin008_03452	696	11	139	148	1.889	20.256	22.588	Glutamyl-tRNA reductase
bin008	SOY3_bin008_03453	1227	37	220	255	3.605	18.186	22.076	N,N'-diacetyllegionaminic acid synthase
bin008	SOY3_bin008_03454	2376	15	41	57	0.755	1.750	2.548	Ubiquinone/menaquinone biosynthesis C-methyltransferase UbiE
bin008	SOY3_bin008_03455	1815	22	82	114	1.449	4.582	6.672	von Willebrand factor type A domain protein
bin008	SOY3_bin008_03456	1401	12	29	45	1.024	2.099	3.412	Succinylglutamate desuccinylase / Aspartoacylase family protein
bin008	SOY3_bin008_03457	1104	3	4	9	0.325	0.367	0.866	hypothetical protein
bin008	SOY3_bin008_03458	1143	5	1	5	0.523	0.089	0.465	DNA primase
bin008	SOY3_bin008_03459	327	1	2	1	0.366	0.620	0.325	hypothetical protein
bin008	SOY3_bin008_03460	693	2	3	6	0.345	0.439	0.920	hypothetical protein
bin008	SOY3_bin008_03461	729	24	51	49	3.936	7.096	7.140	hypothetical protein
bin008	SOY3_bin008_03462	912	12	38	34	1.573	4.226	3.960	hypothetical protein
bin008	SOY3_bin008_03463	711	1	2	1	0.168	0.285	0.149	Energy-coupling factor transporter transmembrane protein EcfT
bin008	SOY3_bin008_03464	1341	3	3	11	0.267	0.227	0.871	Putative HMP/thiamine import ATP-binding protein YkoD
bin008	SOY3_bin008_03465	732	2	2	3	0.327	0.277	0.435	hypothetical protein
bin008	SOY3_bin008_03466	564	2	3	2	0.424	0.540	0.377	hypothetical protein
bin008	SOY3_bin008_03467	1887	6	11	6	0.380	0.591	0.338	Pesticin receptor precursor
bin008	SOY3_bin008_03468	177	1	5	2	0.675	2.865	1.200	hypothetical protein
bin008	SOY3_bin008_03469	930	13	29	39	1.671	3.163	4.455	hypothetical protein
bin008	SOY3_bin008_03470	444	3	24	27	0.808	5.483	6.460	hypothetical protein
bin008	SOY3_bin008_03471	900	19	79	93	2.524	8.903	10.977	Acetylglutamate kinase
bin008	SOY3_bin008_03472	1374	30	90	100	2.610	6.644	7.731	ATP-dependent protease ATPase subunit HslU
bin008	SOY3_bin008_03473	546	14	62	52	3.065	11.517	10.117	ATP-dependent protease subunit ClpQ
bin008	SOY3_bin008_03474	558	4	20	12	0.857	3.635	2.284	O-acetyl-ADP-ribose deacetylase
bin008	SOY3_bin008_03475	1260	21	52	54	1.992	4.186	4.553	Protease 3 precursor
bin008	SOY3_bin008_03476	1023	0	0	0	0.000	0.000	0.000	UDP-N-acetylmuramate--L-alanine ligase
bin008	SOY3_bin008_03477	366	1	2	2	0.327	0.554	0.580	Dinitrogenase iron-molybdenum cofactor
bin008	SOY3_bin008_03478	969	9	22	37	1.110	2.303	4.056	hypothetical protein
bin008	SOY3_bin008_03479	729	22	52	76	3.608	7.235	11.074	Glutamine transport ATP-binding protein GlnQ
bin008	SOY3_bin008_03480	828	72	163	188	10.396	19.967	24.119	ABC transporter glutamine-binding protein GlnH precursor
bin008	SOY3_bin008_03481	702	18	9	15	3.065	1.300	2.270	putative glutamine ABC transporter permease protein GlnM
bin008	SOY3_bin008_03482	696	18	8	10	3.092	1.166	1.526	putative glutamine ABC transporter permease protein GlnP
bin008	SOY3_bin008_03483	258	10	0	1	4.634	0.000	0.412	hypothetical protein
bin008	SOY3_bin008_03484	363	5	26	27	1.647	7.265	7.901	hypothetical protein
bin008	SOY3_bin008_03485	837	13	48	34	1.857	5.817	4.315	phosphodiesterase
bin008	SOY3_bin008_03486	957	13	40	33	1.624	4.239	3.663	Response regulator PleD
bin008	SOY3_bin008_03487	975	2	22	17	0.245	2.289	1.852	Tyrosine recombinase XerD
bin008	SOY3_bin008_03488	76	0	0	0	0.000	0.000	0.000	tRNA-Thr(cgt)
bin008	SOY3_bin008_03489	120	0	0	0	0.000	0.000	0.000	hypothetical protein
bin008	SOY3_bin008_03490	930	5	26	34	0.643	2.836	3.884	hypothetical protein
bin008	SOY3_bin008_03491	3825	93	343	371	2.907	9.095	10.303	Extracellular serine protease precursor
bin008	SOY3_bin008_03492	495	3	40	72	0.725	8.196	15.451	Chaperone protein Skp precursor
bin008	SOY3_bin008_03493	1284	3	25	12	0.279	1.975	0.993	Putative tartrate transporter
bin008	SOY3_bin008_03494	276	3	7	4	1.299	2.572	1.540	Cysteine-rich small domain protein
bin008	SOY3_bin008_03495	1290	27	109	137	2.502	8.570	11.281	hypothetical protein
bin008	SOY3_bin008_03496	1356	45	127	148	3.967	9.499	11.594	Phosphoserine phosphatase RsbU
bin008	SOY3_bin008_03497	933	22	78	104	2.819	8.479	11.841	NAD-dependent protein deacetylase
bin008	SOY3_bin008_03498	705	11	68	47	1.865	9.783	7.082	Biopolymer transport protein ExbB
bin008	SOY3_bin008_03499	441	10	34	41	2.711	7.820	9.876	Biopolymer transport protein ExbD
bin008	SOY3_bin008_03500	1047	6	9	8	0.685	0.872	0.812	hypothetical protein
bin008	SOY3_bin008_03501	354	0	3	3	0.000	0.860	0.900	hypothetical protein
bin008	SOY3_bin008_03502	372	21	26	45	6.749	7.089	12.850	Helix-turn-helix domain protein
bin008	SOY3_bin008_03503	723	8	33	32	1.323	4.629	4.702	hypothetical protein
bin008	SOY3_bin008_03504	252	0	1	1	0.000	0.402	0.422	hypothetical protein
bin008	SOY3_bin008_03505	177	0	1	0	0.000	0.573	0.000	hypothetical protein
bin008	SOY3_bin008_03506	108	0	0	0	0.000	0.000	0.000	hypothetical protein
bin008	SOY3_bin008_03507	114	400	240	363	419.470	213.531	338.245	5S ribosomal RNA
bin008	SOY3_bin008_03508	2925	56916	38326	33826	2326.237	1328.992	1228.442	23S ribosomal RNA

bin008	SOY3_bin008_03509	76	2	25	14	3.146	33.364	19.568	tRNA-Ala(tgc)
bin008	SOY3_bin008_03510	77	5	23	24	7.763	30.296	33.109	tRNA-Ile(gat)
bin008	SOY3_bin008_03511	1456	8935	8185	7490	733.633	570.180	546.450	16S ribosomal RNA
bin008	SOY3_bin008_03512	939	12	25	41	1.528	2.700	4.638	Tyrosine recombinase XerD
bin009	SOY3_bin009_00001	492	2	5	2	0.486	1.031	0.432	Histidine-binding periplasmic protein precursor
bin009	SOY3_bin009_00002	972	1	0	0	0.123	0.000	0.000	L-cystine transport system permease protein TcyB
bin009	SOY3_bin009_00003	789	4	7	4	0.606	0.900	0.539	Glutamine transport ATP-binding protein GlnQ
bin009	SOY3_bin009_00004	177	866	1377	589	584.911	789.070	353.486	Antitoxin/MT0933
bin009	SOY3_bin009_00005	312	0	0	0	0.000	0.000	0.000	Branched-chain amino acid transport protein (AzlD)
bin009	SOY3_bin009_00006	714	0	0	0	0.000	0.000	0.000	AzlC protein
bin009	SOY3_bin009_00007	519	1	3	2	0.230	0.586	0.409	SNARE associated Golgi protein
bin009	SOY3_bin009_00008	936	0	0	1	0.000	0.000	0.113	Membrane transport protein
bin009	SOY3_bin009_00009	1011	0	0	0	0.000	0.000	0.000	HTH-type transcriptional regulator DegA
bin009	SOY3_bin009_00010	1617	1	1	1	0.074	0.063	0.066	Acetate CoA-transferase YdiF
bin009	SOY3_bin009_00011	837	0	0	0	0.000	0.000	0.000	putative enoyl-CoA hydratase
bin009	SOY3_bin009_00012	2493	2	5	0	0.096	0.203	0.000	Xylulose-5-phosphate phosphoketolase
bin009	SOY3_bin009_00013	3969	1	3	0	0.030	0.077	0.000	ATP-dependent RNA helicase HrpB
bin009	SOY3_bin009_00014	891	0	0	0	0.000	0.000	0.000	HTH-type transcriptional regulator CysL
bin009	SOY3_bin009_00015	1035	1	0	0	0.116	0.000	0.000	hypothetical protein
bin009	SOY3_bin009_00016	744	2	0	0	0.321	0.000	0.000	hypothetical protein
bin009	SOY3_bin009_00017	1725	5	3	4	0.347	0.176	0.246	Malto-oligosyltrehalose trehalohydrolase
bin009	SOY3_bin009_00018	2697	1	6	4	0.044	0.226	0.158	Maltooligosyl trehalose synthase
bin009	SOY3_bin009_00019	519	22	24	17	5.068	4.690	3.479	Lambda phage tail tape-measure protein (Tape_meas_lam_C)
bin009	SOY3_bin009_00020	891	1	5	2	0.134	0.569	0.238	Acyl-CoA thioesterase 2
bin009	SOY3_bin009_00021	936	24	30	11	3.065	3.251	1.248	RNA polymerase principal sigma factor HrdB
bin009	SOY3_bin009_00022	1437	4	7	6	0.333	0.494	0.444	RNA polymerase principal sigma factor HrdB
bin009	SOY3_bin009_00023	1359	1	0	0	0.088	0.000	0.000	Proton/sodium-glutamate symport protein
bin009	SOY3_bin009_00024	945	0	1	0	0.000	0.107	0.000	hypothetical protein
bin009	SOY3_bin009_00025	459	2	1	2	0.521	0.221	0.463	Cys-tRNA(Pro)/Cys-tRNA(Cys) deacylase YbaK
bin009	SOY3_bin009_00026	2088	1	8	6	0.057	0.389	0.305	DNA gyrase subunit B
bin009	SOY3_bin009_00027	1407	1	2	1	0.085	0.144	0.075	Vacuole effluxer Atg22 like protein
bin009	SOY3_bin009_00028	810	0	0	2	0.000	0.000	0.262	Pyridoxal phosphate phosphatase YigL
bin009	SOY3_bin009_00029	993	0	1	1	0.000	0.102	0.107	hypothetical protein
bin009	SOY3_bin009_00030	1059	2	4	0	0.226	0.383	0.000	ATPase family associated with various cellular activities (AAA)
bin009	SOY3_bin009_00031	1074	0	2	1	0.000	0.189	0.099	hypothetical protein
bin009	SOY3_bin009_00032	981	0	4	0	0.000	0.414	0.000	von Willebrand factor type A domain protein
bin009	SOY3_bin009_00033	963	1	2	0	0.124	0.211	0.000	von Willebrand factor type A domain protein
bin009	SOY3_bin009_00034	1959	2	5	1	0.122	0.259	0.054	S-formylglutathione hydrolase YeiG
bin009	SOY3_bin009_00035	501	0	4	1	0.000	0.810	0.212	hypothetical protein
bin009	SOY3_bin009_00036	1257	1	1	0	0.095	0.081	0.000	3-oxoacyl-[acyl-carrier-protein] synthase 2
bin009	SOY3_bin009_00037	246	2	2	0	0.972	0.825	0.000	Meromycolate extension acyl carrier protein
bin009	SOY3_bin009_00038	1005	0	0	3	0.000	0.000	0.317	3-oxoacyl-[acyl-carrier-protein] synthase 3
bin009	SOY3_bin009_00039	936	0	0	0	0.000	0.000	0.000	Malonyl CoA-acyl carrier protein transacylase
bin009	SOY3_bin009_00040	1212	0	0	2	0.000	0.000	0.175	Carbohydrate diacid regulator
bin009	SOY3_bin009_00041	1074	3	5	7	0.334	0.472	0.692	5-dehydro-2-deoxygluconokinase
bin009	SOY3_bin009_00042	2745	12	35	16	0.523	1.293	0.619	Pyruvate dehydrogenase E1 component
bin009	SOY3_bin009_00043	546	1	1	0	0.219	0.186	0.000	Dihydrofolate reductase
bin009	SOY3_bin009_00044	417	3	5	2	0.860	1.216	0.509	hypothetical protein
bin009	SOY3_bin009_00045	465	16	73	32	4.114	15.923	7.310	18 kDa heat shock protein
bin009	SOY3_bin009_00046	75	0	0	0	0.000	0.000	0.000	tRNA-Val(tac)
bin009	SOY3_bin009_00047	264	0	0	0	0.000	0.000	0.000	hypothetical protein
bin009	SOY3_bin009_00048	471	0	1	1	0.000	0.215	0.226	glutamyl-tRNA(Gln) amidotransferase subunit E
bin009	SOY3_bin009_00049	1107	4	8	8	0.432	0.733	0.768	Peptide chain release factor 2
bin009	SOY3_bin009_00050	687	1	2	1	0.174	0.295	0.155	Cell division ATP-binding protein FtsE
bin009	SOY3_bin009_00051	915	4	4	2	0.523	0.443	0.232	Cell division protein FtsX
bin009	SOY3_bin009_00052	1386	7	12	9	0.604	0.878	0.690	Murein DD-endopeptidase MepM
bin009	SOY3_bin009_00053	480	0	2	4	0.000	0.423	0.885	SsrA-binding protein
bin009	SOY3_bin009_00054	663	0	1	0	0.000	0.153	0.000	hypothetical protein
bin009	SOY3_bin009_00055	368	1489	2395	1395	483.718	660.104	402.676	transfer-messenger RNA, SsrA
bin009	SOY3_bin009_00056	1197	9	26	9	0.899	2.203	0.799	Sensor histidine kinase LiaS
bin009	SOY3_bin009_00057	654	3	5	2	0.548	0.775	0.325	Response regulator protein VraR
bin009	SOY3_bin009_00058	576	2	6	1	0.415	1.057	0.184	Multiple antibiotic resistance protein MarR
bin009	SOY3_bin009_00059	2280	1	0	1	0.052	0.000	0.047	Membrane protein YdfJ
bin009	SOY3_bin009_00060	852	0	0	1	0.000	0.000	0.125	hypothetical protein
bin009	SOY3_bin009_00061	564	0	5	2	0.000	0.899	0.377	hypothetical protein
bin009	SOY3_bin009_00062	729	2	4	0	0.328	0.557	0.000	DNA-binding response regulator MtrA
bin009	SOY3_bin009_00063	1602	0	2	0	0.000	0.127	0.000	Sensor histidine kinase MtrB

bin009	SOY3_bin009_00064	1761	2	0	0	0.136	0.000	0.000	Lipoprotein LpqB precursor
bin009	SOY3_bin009_00065	510	0	0	0	0.000	0.000	0.000	DNA utilization protein GntX
bin009	SOY3_bin009_00066	624	12	32	24	2.299	5.201	4.086	Putative sigma-54 modulation protein
bin009	SOY3_bin009_00067	2871	7	15	5	0.291	0.530	0.185	preprotein translocase subunit SecA
bin009	SOY3_bin009_00068	453	0	1	0	0.000	0.224	0.000	hypothetical protein
bin009	SOY3_bin009_00069	474	1	1	1	0.252	0.214	0.224	hypothetical protein
bin009	SOY3_bin009_00070	834	0	2	0	0.000	0.243	0.000	putative DNA repair protein YkoV
bin009	SOY3_bin009_00071	1308	4	11	6	0.366	0.853	0.487	putative translaldolase
bin009	SOY3_bin009_00072	468	1	1	1	0.255	0.217	0.227	UbiA prenyltransferase family protein
bin009	SOY3_bin009_00073	1335	1	7	3	0.090	0.532	0.239	L-fuculokinase
bin009	SOY3_bin009_00074	918	2	5	1	0.260	0.552	0.116	4-hydroxy-tetrahydrodipicolinate synthase
bin009	SOY3_bin009_00075	1494	6	17	6	0.480	1.154	0.427	manganese transport protein MntH
bin009	SOY3_bin009_00076	954	3	11	5	0.376	1.169	0.557	putative 3-hydroxybutyryl-CoA dehydrogenase
bin009	SOY3_bin009_00077	933	5	7	8	0.641	0.761	0.911	3-dehydroshikimate dehydratase
bin009	SOY3_bin009_00078	1290	4	1	2	0.371	0.079	0.165	hypothetical protein
bin009	SOY3_bin009_00079	1737	0	12	4	0.000	0.701	0.245	Dihydroxyacetone kinase
bin009	SOY3_bin009_00080	924	0	3	1	0.000	0.329	0.115	1-deoxy-D-xylulose-5-phosphate synthase
bin009	SOY3_bin009_00081	852	0	3	3	0.000	0.357	0.374	Ferredoxin fas2
bin009	SOY3_bin009_00082	441	0	3	4	0.000	0.690	0.963	Ribose-5-phosphate isomerase B
bin009	SOY3_bin009_00083	780	5	4	3	0.766	0.520	0.409	3-oxoacyl-[acyl-carrier-protein] reductase FabG
bin009	SOY3_bin009_00084	483	2	1	3	0.495	0.210	0.660	hypothetical protein
bin009	SOY3_bin009_00085	4815	6	12	11	0.149	0.253	0.243	NAD-specific glutamate dehydrogenase
bin009	SOY3_bin009_00086	858	0	1	2	0.000	0.118	0.248	Error-prone DNA polymerase
bin009	SOY3_bin009_00087	1122	0	1	1	0.000	0.090	0.095	Tryptophan--tRNA ligase 2
bin009	SOY3_bin009_00088	1080	2	3	2	0.221	0.282	0.197	LOG family protein ORF6 in fasciation locus
bin009	SOY3_bin009_00089	780	0	6	5	0.000	0.780	0.681	3-oxoacyl-[acyl-carrier-protein] reductase FabG
bin009	SOY3_bin009_00090	1431	3	8	6	0.251	0.567	0.445	DEAD-box ATP-dependent RNA helicase CshA
bin009	SOY3_bin009_00091	657	1	1	4	0.182	0.154	0.647	hypothetical protein
bin009	SOY3_bin009_00092	867	4	2	2	0.552	0.234	0.245	Shikimate dehydrogenase
bin009	SOY3_bin009_00093	228	1	2	1	0.524	0.890	0.466	hypothetical protein
bin009	SOY3_bin009_00094	207	43	96	85	24.834	47.039	43.619	putative cold shock protein A
bin009	SOY3_bin009_00095	684	0	0	0	0.000	0.000	0.000	hypothetical protein
bin009	SOY3_bin009_00096	101	0	0	1	0.000	0.000	1.052	tRNA-Ala(cgc)
bin009	SOY3_bin009_00097	546	2	0	0	0.438	0.000	0.000	Doxorubicin resistance ATP-binding protein DrrA
bin009	SOY3_bin009_00098	318	1	2	0	0.376	0.638	0.000	Transcriptional repressor SdpR
bin009	SOY3_bin009_00099	405	0	1	1	0.000	0.250	0.262	Arsenate-mycothiol transferase ArsC2
bin009	SOY3_bin009_00100	993	0	0	0	0.000	0.000	0.000	Arsenate-mycothiol transferase ArsC1
bin009	SOY3_bin009_00101	1116	0	0	0	0.000	0.000	0.000	Sodium Bile acid symporter family protein
bin009	SOY3_bin009_00102	324	0	0	0	0.000	0.000	0.000	Thioredoxin
bin009	SOY3_bin009_00103	972	0	0	0	0.000	0.000	0.000	Thioredoxin reductase
bin009	SOY3_bin009_00104	375	0	0	0	0.000	0.000	0.000	Arsenical resistance operon repressor
bin009	SOY3_bin009_00105	447	0	0	1	0.000	0.000	0.238	Arsenical resistance operon trans-acting repressor ArsD
bin009	SOY3_bin009_00106	2544	1	0	0	0.047	0.000	0.000	Arsenical pump-driving ATPase
bin009	SOY3_bin009_00107	933	0	1	0	0.000	0.109	0.000	Bifunctional transcriptional activator/DNA repair enzyme AdaA
bin009	SOY3_bin009_00108	924	0	0	0	0.000	0.000	0.000	hypothetical protein
bin009	SOY3_bin009_00109	720	0	0	0	0.000	0.000	0.000	Putative ATPase subunit of terminase (gpP-like)
bin009	SOY3_bin009_00110	384	0	0	0	0.000	0.000	0.000	hypothetical protein
bin009	SOY3_bin009_00111	465	0	0	0	0.000	0.000	0.000	hypothetical protein
bin009	SOY3_bin009_00112	666	0	0	1	0.000	0.000	0.159	hypothetical protein
bin009	SOY3_bin009_00113	828	0	0	0	0.000	0.000	0.000	hypothetical protein
bin009	SOY3_bin009_00114	1485	1	1	0	0.081	0.068	0.000	AAA-like domain protein
bin009	SOY3_bin009_00115	330	0	0	0	0.000	0.000	0.000	HTH-type transcriptional regulator CmtR
bin009	SOY3_bin009_00116	1905	1	0	1	0.063	0.000	0.056	putative cadmium-transporting ATPase
bin009	SOY3_bin009_00117	546	0	1	0	0.000	0.186	0.000	hypothetical protein
bin009	SOY3_bin009_00118	1428	0	0	0	0.000	0.000	0.000	Type IV secretory system Conjugative DNA transfer
bin009	SOY3_bin009_00119	486	0	0	0	0.000	0.000	0.000	Single-stranded DNA-binding protein
bin009	SOY3_bin009_00120	906	0	0	1	0.000	0.000	0.117	hypothetical protein
bin009	SOY3_bin009_00121	2151	1	1	1	0.056	0.047	0.049	DNA-invertase hin
bin009	SOY3_bin009_00122	549	0	1	1	0.000	0.185	0.193	hypothetical protein
bin009	SOY3_bin009_00123	297	0	1	0	0.000	0.342	0.000	hypothetical protein
bin009	SOY3_bin009_00124	75	0	0	0	0.000	0.000	0.000	tRNA-Ala(ggc)
bin009	SOY3_bin009_00125	828	2	6	1	0.289	0.735	0.128	hypothetical protein
bin009	SOY3_bin009_00126	1518	1	5	4	0.079	0.334	0.280	Gamma-glutamyl phosphate reductase
bin009	SOY3_bin009_00127	1257	0	2	1	0.000	0.161	0.085	Enterochelin esterase
bin009	SOY3_bin009_00128	999	0	1	1	0.000	0.102	0.106	Ferrienterobactin-binding periplasmic protein precursor
bin009	SOY3_bin009_00129	981	0	0	0	0.000	0.000	0.000	putative siderophore transport system permease protein YfiZ precursor
bin009	SOY3_bin009_00130	1077	0	0	1	0.000	0.000	0.099	Ferric enterobactin transport system permease protein FepG

bin009	SOY3_bin009_00131	867	1	4	5	0.138	0.468	0.613	putative siderophore transport system ATP-binding protein YusV
bin009	SOY3_bin009_00132	819	3	5	4	0.438	0.619	0.519	Ribonucleotide monophosphatase NagD
bin009	SOY3_bin009_00133	771	1	1	2	0.155	0.132	0.276	hypothetical protein
bin009	SOY3_bin009_00134	1107	0	1	1	0.000	0.092	0.096	hypothetical protein
bin009	SOY3_bin009_00135	699	13	19	10	2.223	2.757	1.520	VIT family protein
bin009	SOY3_bin009_00136	2493	1	2	2	0.048	0.081	0.085	Leucine--tRNA ligase
bin009	SOY3_bin009_00137	630	0	1	0	0.000	0.161	0.000	ComE operon protein 1
bin009	SOY3_bin009_00138	2346	1	0	0	0.051	0.000	0.000	ComEC family competence protein
bin009	SOY3_bin009_00139	1245	10	14	14	0.960	1.141	1.195	hypothetical protein
bin009	SOY3_bin009_00140	1020	2	0	2	0.234	0.000	0.208	DNA polymerase III subunit delta
bin009	SOY3_bin009_00141	261	5	6	7	2.290	2.332	2.849	30S ribosomal protein S20
bin009	SOY3_bin009_00142	708	3	10	8	0.507	1.433	1.200	hypothetical protein
bin009	SOY3_bin009_00143	666	2	5	3	0.359	0.761	0.478	hypothetical protein
bin009	SOY3_bin009_00144	1836	2	2	5	0.130	0.110	0.289	Elongation factor 4
bin009	SOY3_bin009_00145	735	0	0	0	0.000	0.000	0.000	putative S-adenosylmethionine-dependent methyltransferase/MSMEI_2290
bin009	SOY3_bin009_00146	360	0	0	0	0.000	0.000	0.000	hypothetical protein
bin009	SOY3_bin009_00147	1212	0	0	0	0.000	0.000	0.000	Oxygen-independent coproporphyrinogen-III oxidase 1
bin009	SOY3_bin009_00148	774	0	2	0	0.000	0.262	0.000	hypothetical protein
bin009	SOY3_bin009_00149	3123	1	9	6	0.038	0.292	0.204	Isoamylase precursor
bin009	SOY3_bin009_00150	792	1	4	3	0.151	0.512	0.402	Inositol-1-monophosphatase
bin009	SOY3_bin009_00151	1014	2	4	3	0.236	0.400	0.314	Heat-inducible transcription repressor HrcA
bin009	SOY3_bin009_00152	1215	3	2	5	0.295	0.167	0.437	Chaperone protein DnaJ
bin009	SOY3_bin009_00153	756	0	0	0	0.000	0.000	0.000	Ribosomal RNA small subunit methyltransferase E
bin009	SOY3_bin009_00154	1362	0	5	1	0.000	0.372	0.078	Calcineurin-like phosphoesterase
bin009	SOY3_bin009_00155	939	0	3	1	0.000	0.324	0.113	Diacylglycerol kinase
bin009	SOY3_bin009_00156	2745	0	3	5	0.000	0.111	0.193	ATP-dependent DNA helicase RecQ
bin009	SOY3_bin009_00157	768	0	2	1	0.000	0.264	0.138	Imidazole glycerol phosphate synthase subunit HisF
bin009	SOY3_bin009_00158	1749	2	11	5	0.137	0.638	0.304	Acetolactate synthase large subunit IlvB1
bin009	SOY3_bin009_00159	525	1	2	0	0.228	0.386	0.000	Acetolactate synthase small subunit
bin009	SOY3_bin009_00160	1029	7	11	9	0.813	1.084	0.929	Ketol-acid reductoisomerase
bin009	SOY3_bin009_00161	555	0	2	1	0.000	0.366	0.191	hypothetical protein
bin009	SOY3_bin009_00162	1530	4	19	4	0.313	1.260	0.278	Inner membrane protein YqiK
bin009	SOY3_bin009_00163	1242	6	7	1	0.578	0.572	0.086	DNA-directed RNA polymerase subunit P
bin009	SOY3_bin009_00164	1167	8	13	7	0.820	1.130	0.637	SPFH domain / Band 7 family protein
bin009	SOY3_bin009_00165	786	1	0	2	0.152	0.000	0.270	hypothetical protein
bin009	SOY3_bin009_00166	1062	0	1	2	0.000	0.096	0.200	3-isopropylmalate dehydrogenase
bin009	SOY3_bin009_00167	1083	1	8	2	0.110	0.749	0.196	Branched-chain-amino-acid aminotransferase
bin009	SOY3_bin009_00168	1614	1	4	4	0.074	0.251	0.263	2-isopropylmalate synthase
bin009	SOY3_bin009_00169	1278	1	2	1	0.094	0.159	0.083	Pyrimidine-nucleoside phosphorylase
bin009	SOY3_bin009_00170	840	0	1	2	0.000	0.121	0.253	Ureidoglycolate lyase
bin009	SOY3_bin009_00171	948	0	0	1	0.000	0.000	0.112	CAAX amino terminal protease self- immunity
bin009	SOY3_bin009_00172	75	0	3	0	0.000	4.057	0.000	tRNA-Gln(ctg)
bin009	SOY3_bin009_00173	76	2	0	2	3.146	0.000	2.795	tRNA-Glu(ctc)
bin009	SOY3_bin009_00174	1530	0	1	0	0.000	0.066	0.000	Beta-lactamase precursor
bin009	SOY3_bin009_00175	501	0	0	2	0.000	0.000	0.424	Isoprenylcysteine carboxyl methyltransferase (ICMT) family protein
bin009	SOY3_bin009_00176	264	0	0	0	0.000	0.000	0.000	Glycerol-3-phosphate dehydrogenase [NAD(P)+]
bin009	SOY3_bin009_00177	306	0	0	0	0.000	0.000	0.000	hypothetical protein
bin009	SOY3_bin009_00178	1929	2	0	0	0.124	0.000	0.000	NADH oxidase
bin009	SOY3_bin009_00179	846	0	0	0	0.000	0.000	0.000	hypothetical protein
bin009	SOY3_bin009_00180	366	0	0	0	0.000	0.000	0.000	hypothetical protein
bin009	SOY3_bin009_00181	1332	0	0	0	0.000	0.000	0.000	hypothetical protein
bin009	SOY3_bin009_00182	2049	0	0	0	0.000	0.000	0.000	hypothetical protein
bin009	SOY3_bin009_00183	678	0	0	0	0.000	0.000	0.000	hypothetical protein
bin009	SOY3_bin009_00184	1476	0	1	0	0.000	0.069	0.000	hypothetical protein
bin009	SOY3_bin009_00185	285	0	0	0	0.000	0.000	0.000	hypothetical protein
bin009	SOY3_bin009_00186	210	0	0	0	0.000	0.000	0.000	hypothetical protein
bin009	SOY3_bin009_00187	612	0	0	0	0.000	0.000	0.000	hypothetical protein
bin009	SOY3_bin009_00188	567	0	0	0	0.000	0.000	0.000	Penicillin-binding protein activator LpoB
bin009	SOY3_bin009_00189	942	0	0	1	0.000	0.000	0.113	hypothetical protein
bin009	SOY3_bin009_00190	699	0	0	0	0.000	0.000	0.000	SAF domain protein
bin009	SOY3_bin009_00191	789	0	0	0	0.000	0.000	0.000	hypothetical protein
bin009	SOY3_bin009_00192	1569	0	0	1	0.000	0.000	0.068	Type IV secretion system protein PtlH
bin009	SOY3_bin009_00193	861	0	0	0	0.000	0.000	0.000	Bacterial type II secretion system protein F domain protein
bin009	SOY3_bin009_00194	900	0	0	0	0.000	0.000	0.000	Bacterial type II secretion system protein F domain protein
bin009	SOY3_bin009_00195	216	0	0	0	0.000	0.000	0.000	hypothetical protein
bin009	SOY3_bin009_00196	414	0	0	0	0.000	0.000	0.000	TadE-like protein
bin009	SOY3_bin009_00197	426	0	0	0	0.000	0.000	0.000	TadE-like protein

bin009	SOY3_bin009_00198	429	0	0	0	0.000	0.000	0.000	hypothetical protein
bin009	SOY3_bin009_00199	3204	0	0	0	0.000	0.000	0.000	LysM domain/BON superfamily protein
bin009	SOY3_bin009_00200	189	1	0	0	0.633	0.000	0.000	Sec23/Sec24 zinc finger
bin009	SOY3_bin009_00201	339	0	0	0	0.000	0.000	0.000	hypothetical protein
bin009	SOY3_bin009_00202	933	0	0	0	0.000	0.000	0.000	hypothetical protein
bin009	SOY3_bin009_00203	438	0	0	0	0.000	0.000	0.000	hypothetical protein
bin009	SOY3_bin009_00204	261	0	0	0	0.000	0.000	0.000	Transposase, Mutator family
bin009	SOY3_bin009_00205	495	0	0	0	0.000	0.000	0.000	Transposase, Mutator family
bin009	SOY3_bin009_00206	813	0	0	0	0.000	0.000	0.000	hypothetical protein
bin009	SOY3_bin009_00207	198	0	0	0	0.000	0.000	0.000	hypothetical protein
bin009	SOY3_bin009_00208	639	0	0	0	0.000	0.000	0.000	hypothetical protein
bin009	SOY3_bin009_00209	279	0	0	0	0.000	0.000	0.000	hypothetical protein
bin009	SOY3_bin009_00210	819	0	0	0	0.000	0.000	0.000	hypothetical protein
bin009	SOY3_bin009_00211	1572	0	0	0	0.000	0.000	0.000	hypothetical protein
bin009	SOY3_bin009_00212	1512	0	1	0	0.000	0.067	0.000	hypothetical protein
bin009	SOY3_bin009_00213	1515	0	0	0	0.000	0.000	0.000	AAA-like domain protein
bin009	SOY3_bin009_00214	1923	1	3	1	0.062	0.158	0.055	Murein DD-endopeptidase MepM
bin009	SOY3_bin009_00215	237	0	0	0	0.000	0.000	0.000	hypothetical protein
bin009	SOY3_bin009_00216	417	0	0	0	0.000	0.000	0.000	hypothetical protein
bin009	SOY3_bin009_00217	462	0	0	0	0.000	0.000	0.000	hypothetical protein
bin009	SOY3_bin009_00218	1452	0	0	0	0.000	0.000	0.000	Coupling protein TraD
bin009	SOY3_bin009_00219	621	0	0	0	0.000	0.000	0.000	hypothetical protein
bin009	SOY3_bin009_00220	936	0	0	0	0.000	0.000	0.000	RNA polymerase principal sigma factor HrdB
bin009	SOY3_bin009_00221	312	0	0	0	0.000	0.000	0.000	hypothetical protein
bin009	SOY3_bin009_00222	1665	0	0	0	0.000	0.000	0.000	DNA helicase II
bin009	SOY3_bin009_00223	1089	0	0	0	0.000	0.000	0.000	hypothetical protein
bin009	SOY3_bin009_00224	246	0	0	0	0.000	0.000	0.000	transposase
bin009	SOY3_bin009_00225	990	0	0	0	0.000	0.000	0.000	Tyrosine recombinase XerD
bin009	SOY3_bin009_00226	933	0	0	0	0.000	0.000	0.000	Tyrosine recombinase XerC
bin009	SOY3_bin009_00227	1224	0	0	0	0.000	0.000	0.000	Tyrosine recombinase XerC
bin009	SOY3_bin009_00228	609	0	0	0	0.000	0.000	0.000	transposase
bin009	SOY3_bin009_00229	1191	0	1	0	0.000	0.085	0.000	Integrase core domain protein
bin009	SOY3_bin009_00230	474	1	0	0	0.252	0.000	0.000	phosphonate/organophosphate ester transporter subunit
bin009	SOY3_bin009_00231	825	0	1	1	0.000	0.123	0.129	hypothetical protein
bin009	SOY3_bin009_00232	1254	0	0	0	0.000	0.000	0.000	Helix-turn-helix
bin009	SOY3_bin009_00233	612	5	0	1	0.977	0.000	0.174	hypothetical protein
bin009	SOY3_bin009_00234	642	0	0	0	0.000	0.000	0.000	Type 4 prepilin-like proteins leader peptide-processing enzyme
bin009	SOY3_bin009_00235	5616	0	0	0	0.000	0.000	0.000	DNA primase
bin009	SOY3_bin009_00236	417	0	0	0	0.000	0.000	0.000	hypothetical protein
bin009	SOY3_bin009_00237	318	0	0	0	0.000	0.000	0.000	hypothetical protein
bin009	SOY3_bin009_00238	1182	0	0	0	0.000	0.000	0.000	hypothetical protein
bin009	SOY3_bin009_00239	723	0	0	0	0.000	0.000	0.000	SprT-like family protein
bin009	SOY3_bin009_00240	3093	0	0	0	0.000	0.000	0.000	Type-1 restriction enzyme R protein
bin009	SOY3_bin009_00241	1119	1	0	0	0.107	0.000	0.000	Type I restriction modification DNA specificity domain protein
bin009	SOY3_bin009_00242	2448	0	0	0	0.000	0.000	0.000	Type I restriction enzyme EcoKI M protein
bin009	SOY3_bin009_00243	1449	0	0	0	0.000	0.000	0.000	hypothetical protein
bin009	SOY3_bin009_00244	255	0	0	0	0.000	0.000	0.000	Helix-turn-helix domain protein
bin009	SOY3_bin009_00245	1167	0	0	0	0.000	0.000	0.000	Putative prophage phiRv2 integrase
bin009	SOY3_bin009_00246	85	1	1	0	1.406	1.193	0.000	tRNA-Ser(cag)
bin009	SOY3_bin009_00247	957	1	1	0	0.125	0.106	0.000	aromatic amino acid exporter
bin009	SOY3_bin009_00248	954	0	1	1	0.000	0.106	0.111	hypothetical protein
bin009	SOY3_bin009_00249	2553	1	1	0	0.047	0.040	0.000	ski2-like helicase
bin009	SOY3_bin009_00250	150	0	0	0	0.000	0.000	0.000	hypothetical protein
bin009	SOY3_bin009_00251	1185	0	0	1	0.000	0.000	0.090	NADH dehydrogenase subunit A
bin009	SOY3_bin009_00252	1494	0	0	0	0.000	0.000	0.000	hypothetical protein
bin009	SOY3_bin009_00253	528	0	0	0	0.000	0.000	0.000	Propanediol dehydratase small subunit
bin009	SOY3_bin009_00254	657	0	0	0	0.000	0.000	0.000	Propanediol dehydratase medium subunit
bin009	SOY3_bin009_00255	1662	0	0	0	0.000	0.000	0.000	Propanediol dehydratase large subunit
bin009	SOY3_bin009_00256	825	0	0	0	0.000	0.000	0.000	Propanediol utilization protein PduB
bin009	SOY3_bin009_00257	279	0	0	0	0.000	0.000	0.000	Propanediol utilization protein PduA
bin009	SOY3_bin009_00258	1131	0	3	0	0.000	0.269	0.000	NAD-dependent methanol dehydrogenase
bin009	SOY3_bin009_00259	1335	12	19	6	1.075	1.444	0.477	HTH-type transcriptional activator Btr
bin009	SOY3_bin009_00260	846	0	1	0	0.000	0.120	0.000	Putative phosphonates utilization ATP-binding protein PhnK
bin009	SOY3_bin009_00261	2187	0	1	0	0.000	0.046	0.000	Chromosome partition protein Smc
bin009	SOY3_bin009_00262	702	0	0	2	0.000	0.000	0.303	hypothetical protein
bin009	SOY3_bin009_00263	1539	1	0	0	0.078	0.000	0.000	DNA polymerase IV
bin009	SOY3_bin009_00264	3375	0	1	0	0.000	0.030	0.000	Error-prone DNA polymerase

bin009	SOY3_bin009_00265	624	3	2	1	0.575	0.325	0.170	hypothetical protein
bin009	SOY3_bin009_00266	1791	1	1	0	0.067	0.057	0.000	putative ABC transporter ATP-binding protein/MT2552
bin009	SOY3_bin009_00267	441	1	6	2	0.271	1.380	0.482	hypothetical protein
bin009	SOY3_bin009_00268	600	0	0	0	0.000	0.000	0.000	Iron import ATP-binding/permease protein IrtA
bin009	SOY3_bin009_00269	711	0	0	0	0.000	0.000	0.000	GMP synthase [glutamine-hydrolyzing]
bin009	SOY3_bin009_00270	1974	1	2	1	0.061	0.103	0.054	Dihydroxy-acid dehydratase
bin009	SOY3_bin009_00271	279	0	0	1	0.000	0.000	0.381	HicB family protein
bin009	SOY3_bin009_00272	978	1	7	0	0.122	0.726	0.000	SPFH domain / Band 7 family protein
bin009	SOY3_bin009_00273	1017	1	1	2	0.118	0.100	0.209	Magnesium transport protein CorA
bin009	SOY3_bin009_00274	2007	0	3	5	0.000	0.152	0.265	Threonine--tRNA ligase
bin009	SOY3_bin009_00275	594	0	1	1	0.000	0.171	0.179	AP-4-A phosphorylase
bin009	SOY3_bin009_00276	624	0	0	1	0.000	0.000	0.170	CDP-diacylglycerol--inositol 3-phosphatidyltransferase
bin009	SOY3_bin009_00277	873	0	1	1	0.000	0.116	0.122	Phosphatidylinositol mannoside acyltransferase
bin009	SOY3_bin009_00278	1152	0	1	0	0.000	0.088	0.000	GDP-mannose-dependent alpha-(1-2)-phosphatidylinositol mannosyltransferase
bin009	SOY3_bin009_00279	630	0	1	0	0.000	0.161	0.000	Putative CDP-diacylglycerol--glycerol-3-phosphate 3-phosphatidyl-transferase 2
bin009	SOY3_bin009_00280	747	1	4	2	0.160	0.543	0.284	hypothetical protein
bin009	SOY3_bin009_00281	333	1	2	0	0.359	0.609	0.000	hypothetical protein
bin009	SOY3_bin009_00282	741	2	7	3	0.323	0.958	0.430	hypothetical protein
bin009	SOY3_bin009_00283	387	1	0	1	0.309	0.000	0.274	Glycine cleavage system H protein
bin009	SOY3_bin009_00284	492	1	4	5	0.243	0.825	1.080	Oxoglutarate dehydrogenase inhibitor
bin009	SOY3_bin009_00285	711	3	2	1	0.504	0.285	0.149	Mercuric resistance operon regulatory protein
bin009	SOY3_bin009_00286	444	8	11	3	2.154	2.513	0.718	hypothetical protein
bin009	SOY3_bin009_00287	567	2	6	5	0.422	1.073	0.937	HTH-type transcriptional regulator HmrR
bin009	SOY3_bin009_00288	2820	1	7	4	0.042	0.252	0.151	Glycine dehydrogenase (decarboxylating)
bin009	SOY3_bin009_00289	1119	0	0	0	0.000	0.000	0.000	Inner membrane protein YhjD
bin009	SOY3_bin009_00290	1215	7	21	12	0.689	1.753	1.049	Pyrophosphate--fructose 6-phosphate 1-phosphotransferase
bin009	SOY3_bin009_00291	420	0	0	1	0.000	0.000	0.253	hypothetical protein
bin009	SOY3_bin009_00292	624	2	1	1	0.383	0.163	0.170	Threonylcarbamoyl-AMP synthase
bin009	SOY3_bin009_00293	804	1	3	3	0.149	0.378	0.396	PP2C-family Ser/Thr phosphatase
bin009	SOY3_bin009_00294	900	0	2	2	0.000	0.225	0.236	Rhomboid protease GluP
bin009	SOY3_bin009_00295	1527	0	6	3	0.000	0.399	0.209	Chaperone protein HscA
bin009	SOY3_bin009_00296	1134	1	11	3	0.105	0.984	0.281	Glutaconyl-CoA decarboxylase subunit beta
bin009	SOY3_bin009_00297	507	3	5	3	0.707	1.000	0.629	Glutaconyl-CoA decarboxylase subunit gamma
bin009	SOY3_bin009_00298	387	0	2	1	0.000	0.524	0.274	oxaloacetate decarboxylase subunit gamma
bin009	SOY3_bin009_00299	1578	9	19	7	0.682	1.221	0.471	putative propionyl-CoA carboxylase beta chain 5
bin009	SOY3_bin009_00300	1140	1	3	1	0.105	0.267	0.093	GDP-mannose-dependent alpha-mannosyltransferase
bin009	SOY3_bin009_00301	1203	2	2	4	0.199	0.169	0.353	UDP-glucose 4-epimerase
bin009	SOY3_bin009_00302	1104	0	1	0	0.000	0.092	0.000	Inner membrane protein YbjJ
bin009	SOY3_bin009_00303	831	1	4	2	0.144	0.488	0.256	Polyphosphate glucokinase
bin009	SOY3_bin009_00304	927	9	34	7	1.161	3.720	0.802	PAC2 family protein
bin009	SOY3_bin009_00305	1533	1	2	0	0.078	0.132	0.000	hypothetical protein
bin009	SOY3_bin009_00306	945	1	0	1	0.127	0.000	0.112	hypothetical protein
bin009	SOY3_bin009_00307	762	0	1	1	0.000	0.133	0.139	hypothetical protein
bin009	SOY3_bin009_00308	903	0	1	0	0.000	0.112	0.000	Protein-glutamine gamma-glutamyltransferase
bin009	SOY3_bin009_00309	2550	1	3	6	0.047	0.119	0.250	hypothetical protein
bin009	SOY3_bin009_00310	3492	5	7	0	0.171	0.203	0.000	hypothetical protein
bin009	SOY3_bin009_00311	744	0	1	1	0.000	0.136	0.143	Arginine transport ATP-binding protein ArtM
bin009	SOY3_bin009_00312	2184	0	4	3	0.000	0.186	0.146	Glutamine transport system permease protein GlnP
bin009	SOY3_bin009_00313	492	0	0	0	0.000	0.000	0.000	Response regulator protein VraR
bin009	SOY3_bin009_00314	1512	0	0	0	0.000	0.000	0.000	hypothetical protein
bin009	SOY3_bin009_00315	1509	0	0	0	0.000	0.000	0.000	AAA-like domain protein
bin009	SOY3_bin009_00316	1530	0	0	0	0.000	0.000	0.000	Murein DD-endopeptidase MepM
bin009	SOY3_bin009_00317	660	0	0	0	0.000	0.000	0.000	RNA polymerase sigma factor SigL
bin009	SOY3_bin009_00318	2019	0	0	0	0.000	0.000	0.000	Coupling protein TraD
bin009	SOY3_bin009_00319	651	0	0	0	0.000	0.000	0.000	hypothetical protein
bin009	SOY3_bin009_00320	921	0	0	0	0.000	0.000	0.000	RNA polymerase principal sigma factor HrdB
bin009	SOY3_bin009_00321	477	0	0	0	0.000	0.000	0.000	hypothetical protein
bin009	SOY3_bin009_00322	315	0	3	0	0.000	0.966	0.000	hypothetical protein
bin009	SOY3_bin009_00323	468	2	1	2	0.511	0.217	0.454	hypothetical protein
bin009	SOY3_bin009_00324	210	0	0	0	0.000	0.000	0.000	hypothetical protein
bin009	SOY3_bin009_00325	1149	0	1	0	0.000	0.088	0.000	hypothetical protein
bin009	SOY3_bin009_00326	687	0	0	0	0.000	0.000	0.000	hypothetical protein
bin009	SOY3_bin009_00327	291	0	0	0	0.000	0.000	0.000	hypothetical protein
bin009	SOY3_bin009_00328	684	0	1	0	0.000	0.148	0.000	hypothetical protein
bin009	SOY3_bin009_00329	795	0	0	0	0.000	0.000	0.000	Phospholipase D precursor
bin009	SOY3_bin009_00330	1839	1	0	0	0.065	0.000	0.000	hypothetical protein
bin009	SOY3_bin009_00331	3885	0	4	0	0.000	0.104	0.000	hypothetical protein

bin009	SOY3_bin009_00332	3450	0	2	0	0.000	0.059	0.000	N5-glutamine S-adenosyl-L-methionine-dependent methyltransferase
bin009	SOY3_bin009_00333	3159	0	1	0	0.000	0.032	0.000	RNA polymerase-associated protein RapA
bin009	SOY3_bin009_00334	1095	0	2	0	0.000	0.185	0.000	putative BsuMI modification methylase subunit YdiO
bin009	SOY3_bin009_00335	225	0	1	0	0.000	0.451	0.000	Helix-turn-helix domain protein
bin009	SOY3_bin009_00336	5664	0	0	1	0.000	0.000	0.019	DNA primase
bin009	SOY3_bin009_00337	390	0	0	0	0.000	0.000	0.000	hypothetical protein
bin009	SOY3_bin009_00338	306	0	0	0	0.000	0.000	0.000	hypothetical protein
bin009	SOY3_bin009_00339	453	0	0	0	0.000	0.000	0.000	hypothetical protein
bin009	SOY3_bin009_00340	1020	0	0	0	0.000	0.000	0.000	Endonuclease NucS
bin009	SOY3_bin009_00341	189	0	0	0	0.000	0.000	0.000	hypothetical protein
bin009	SOY3_bin009_00342	1092	0	0	0	0.000	0.000	0.000	DNA primase TraC
bin009	SOY3_bin009_00343	708	1	0	0	0.169	0.000	0.000	hypothetical protein
bin009	SOY3_bin009_00344	342	0	0	0	0.000	0.000	0.000	hypothetical protein
bin009	SOY3_bin009_00345	1347	0	0	1	0.000	0.000	0.079	hypothetical protein
bin009	SOY3_bin009_00346	282	0	1	0	0.000	0.360	0.000	hypothetical protein
bin009	SOY3_bin009_00347	198	0	0	0	0.000	0.000	0.000	Helix-turn-helix domain protein
bin009	SOY3_bin009_00348	768	0	0	0	0.000	0.000	0.000	hypothetical protein
bin009	SOY3_bin009_00349	168	0	0	0	0.000	0.000	0.000	hypothetical protein
bin009	SOY3_bin009_00350	1779	0	1	0	0.000	0.057	0.000	hypothetical protein
bin009	SOY3_bin009_00351	3162	3	6	2	0.113	0.192	0.067	hypothetical protein
bin009	SOY3_bin009_00352	1002	0	0	0	0.000	0.000	0.000	L-glyceraldehyde 3-phosphate reductase
bin009	SOY3_bin009_00353	1089	0	1	0	0.000	0.093	0.000	Lipase 2
bin009	SOY3_bin009_00354	387	0	1	1	0.000	0.262	0.274	hypothetical protein
bin009	SOY3_bin009_00355	465	1	1	0	0.257	0.218	0.000	NifU-like protein
bin009	SOY3_bin009_00356	1248	0	2	1	0.000	0.163	0.085	putative cysteine desulfurase
bin009	SOY3_bin009_00357	762	1	0	0	0.157	0.000	0.000	Vegetative protein 296
bin009	SOY3_bin009_00358	342	0	0	0	0.000	0.000	0.000	3-phenylpropionate/cinnamic acid dioxygenase ferredoxin subunit
bin009	SOY3_bin009_00359	1194	0	4	1	0.000	0.340	0.089	FeS cluster assembly protein SufB
bin009	SOY3_bin009_00360	1443	1	5	1	0.083	0.351	0.074	FeS cluster assembly protein SufB
bin009	SOY3_bin009_00361	729	6	4	2	0.984	0.557	0.291	Helix-turn-helix domain protein
bin009	SOY3_bin009_00362	1521	1	1	0	0.079	0.067	0.000	Xylulose kinase
bin009	SOY3_bin009_00363	1527	0	4	4	0.000	0.266	0.278	Glucose-6-phosphate 1-dehydrogenase
bin009	SOY3_bin009_00364	912	0	3	1	0.000	0.334	0.116	Glucose-6-phosphate dehydrogenase subunit
bin009	SOY3_bin009_00365	744	0	4	1	0.000	0.545	0.143	6-phosphogluconolactonase
bin009	SOY3_bin009_00366	309	2	2	1	0.774	0.656	0.344	RNA polymerase-binding protein RbpA
bin009	SOY3_bin009_00367	252	2	4	6	0.949	1.610	2.529	preprotein translocase subunit SecG
bin009	SOY3_bin009_00368	783	1	10	8	0.153	1.295	1.085	Triosephosphate isomerase
bin009	SOY3_bin009_00369	1224	4	14	6	0.391	1.160	0.521	Phosphoglycerate kinase
bin009	SOY3_bin009_00370	1005	33	88	47	3.925	8.881	4.968	Glyceraldehyde-3-phosphate dehydrogenase
bin009	SOY3_bin009_00371	984	5	10	5	0.607	1.031	0.540	Putative sporulation transcription regulator WhiA
bin009	SOY3_bin009_00372	936	0	0	2	0.000	0.000	0.227	Putative gluconeogenesis factor
bin009	SOY3_bin009_00373	900	1	3	1	0.133	0.338	0.118	glmZ(sRNA)-inactivating NTPase
bin009	SOY3_bin009_00374	1986	0	1	0	0.000	0.051	0.000	UvrABC system protein C
bin009	SOY3_bin009_00375	3009	2	5	6	0.079	0.169	0.212	UvrABC system protein A
bin009	SOY3_bin009_00376	735	2	7	3	0.325	0.966	0.434	mycothiol-dependent maleylpyruvate isomerase
bin009	SOY3_bin009_00377	663	0	2	2	0.000	0.306	0.320	putative metallo-hydrolase
bin009	SOY3_bin009_00378	1005	2	9	7	0.238	0.908	0.740	Inner membrane protein alx
bin009	SOY3_bin009_00379	2091	1	2	0	0.057	0.097	0.000	UvrABC system protein B
bin009	SOY3_bin009_00380	585	0	0	2	0.000	0.000	0.363	hypothetical protein
bin009	SOY3_bin009_00381	288	0	1	0	0.000	0.352	0.000	hypothetical protein
bin009	SOY3_bin009_00382	663	0	1	0	0.000	0.153	0.000	hypothetical protein
bin009	SOY3_bin009_00383	609	0	1	0	0.000	0.167	0.000	Dephospho-CoA kinase
bin009	SOY3_bin009_00384	1119	2	1	0	0.214	0.091	0.000	Ferrochelatase
bin009	SOY3_bin009_00385	1155	2	6	4	0.207	0.527	0.368	Antilisterial bacteriocin subtilosin biosynthesis protein AlbA
bin009	SOY3_bin009_00386	1446	14	27	19	1.157	1.894	1.396	hypothetical protein
bin009	SOY3_bin009_00387	726	0	2	0	0.000	0.279	0.000	Sortase family protein
bin009	SOY3_bin009_00388	2649	1	4	1	0.045	0.153	0.040	DNA polymerase I
bin009	SOY3_bin009_00389	534	1	2	2	0.224	0.380	0.398	Esterase YdiI
bin009	SOY3_bin009_00390	612	8	14	14	1.563	2.320	2.430	putative transcriptional regulatory protein pdtaR
bin009	SOY3_bin009_00391	84	0	4	2	0.000	4.830	2.529	tRNA-Leu(caa)
bin009	SOY3_bin009_00392	177	0	0	0	0.000	0.000	0.000	Helix-turn-helix domain protein
bin009	SOY3_bin009_00393	240	0	0	0	0.000	0.000	0.000	hypothetical protein
bin009	SOY3_bin009_00394	252	0	0	0	0.000	0.000	0.000	hypothetical protein
bin009	SOY3_bin009_00395	2097	0	0	0	0.000	0.000	0.000	Regulatory protein RepA
bin009	SOY3_bin009_00396	255	0	0	1	0.000	0.000	0.417	hypothetical protein
bin009	SOY3_bin009_00397	165	0	0	0	0.000	0.000	0.000	hypothetical protein
bin009	SOY3_bin009_00398	597	0	0	0	0.000	0.000	0.000	hypothetical protein

bin009	SOY3_bin009_00399	828	0	0	0	0.000	0.000	0.000	Phage capsid family protein
bin009	SOY3_bin009_00400	1179	0	1	0	0.000	0.086	0.000	Putative prophage phiRv2 integrase
bin009	SOY3_bin009_00401	1359	21	43	23	1.847	3.209	1.798	Pyruvate kinase
bin009	SOY3_bin009_00402	924	0	1	1	0.000	0.110	0.115	Ribosomal large subunit pseudouridine synthase D
bin009	SOY3_bin009_00403	447	0	2	1	0.000	0.454	0.238	Lipoprotein signal peptidase
bin009	SOY3_bin009_00404	156	0	0	1	0.000	0.000	0.681	RNA polymerase-binding transcription factor DksA
bin009	SOY3_bin009_00405	816	22	33	23	3.223	4.102	2.994	hypothetical protein
bin009	SOY3_bin009_00406	1509	22	73	44	1.743	4.907	3.097	Methylmalonyl-CoA carboxyltransferase 5S subunit
bin009	SOY3_bin009_00407	1911	3	6	10	0.188	0.318	0.556	Putative asparagine synthetase [glutamine-hydrolyzing]
bin009	SOY3_bin009_00408	1170	4	5	5	0.409	0.433	0.454	Acetoin dehydrogenase operon transcriptional activator AcoR
bin009	SOY3_bin009_00409	996	5	28	12	0.600	2.851	1.280	Acetoin:2,6-dichlorophenolindophenol oxidoreductase subunit alpha
bin009	SOY3_bin009_00410	1017	3	18	6	0.353	1.795	0.627	Acetoin:2,6-dichlorophenolindophenol oxidoreductase subunit beta
bin009	SOY3_bin009_00411	1539	2	19	4	0.155	1.252	0.276	Dihydrolipoyllysine-residue acetyltransferase component of pyruvate dehydrogenase complex
bin009	SOY3_bin009_00412	1473	8	22	10	0.649	1.515	0.721	Dihydrolipoyl dehydrogenase
bin009	SOY3_bin009_00413	1845	3	16	7	0.194	0.880	0.403	Dihydroxy-acid dehydratase
bin009	SOY3_bin009_00414	1053	2	2	1	0.227	0.193	0.101	Inner membrane transport permease YbhR
bin009	SOY3_bin009_00415	807	0	1	0	0.000	0.126	0.000	putative ABC transporter ATP-binding protein YbhF
bin009	SOY3_bin009_00416	864	0	0	0	0.000	0.000	0.000	putative ABC transporter ATP-binding protein YbhF
bin009	SOY3_bin009_00417	291	0	1	0	0.000	0.349	0.000	hypothetical protein
bin009	SOY3_bin009_00418	1152	1	6	0	0.104	0.528	0.000	D-xylose-proton symporter
bin009	SOY3_bin009_00419	990	1	9	7	0.121	0.922	0.751	2-dehydro-3-deoxygluconokinase
bin009	SOY3_bin009_00420	1023	29	52	23	3.389	5.156	2.388	Fructose-bisphosphate aldolase
bin009	SOY3_bin009_00421	630	1	1	3	0.190	0.161	0.506	hypothetical protein
bin009	SOY3_bin009_00422	1050	5	14	6	0.569	1.352	0.607	D-3-phosphoglycerate dehydrogenase
bin009	SOY3_bin009_00423	645	0	0	1	0.000	0.000	0.165	hypothetical protein
bin009	SOY3_bin009_00424	1158	0	0	2	0.000	0.000	0.183	Cystathionine beta-lyase PatB
bin009	SOY3_bin009_00425	663	0	1	0	0.000	0.153	0.000	tRNA (guanosine(18)-2'-O)-methyltransferase
bin009	SOY3_bin009_00426	540	3	4	0	0.664	0.751	0.000	Orotate phosphoribosyltransferase
bin009	SOY3_bin009_00427	714	0	4	6	0.000	0.568	0.893	Alpha/beta hydrolase family protein
bin009	SOY3_bin009_00428	681	0	0	4	0.000	0.000	0.624	Sortase family protein
bin009	SOY3_bin009_00429	807	1	2	1	0.148	0.251	0.132	Glutamine transport ATP-binding protein GlnQ
bin009	SOY3_bin009_00430	981	1	3	0	0.122	0.310	0.000	Inner membrane amino-acid ABC transporter permease protein YecS
bin009	SOY3_bin009_00431	903	2	10	8	0.265	1.123	0.941	Histidine-binding periplasmic protein precursor
bin009	SOY3_bin009_00432	807	1	2	0	0.148	0.251	0.000	spermidine synthase
bin009	SOY3_bin009_00433	2583	5	22	13	0.231	0.864	0.535	Chaperone protein ClpB
bin009	SOY3_bin009_00434	1080	15	21	14	1.660	1.972	1.377	Inositol-3-phosphate synthase
bin009	SOY3_bin009_00435	546	15	13	13	3.284	2.415	2.529	Transcriptional regulator PadR-like family protein
bin009	SOY3_bin009_00436	564	0	0	0	0.000	0.000	0.000	Alkyl hydroperoxide reductase subunit C
bin009	SOY3_bin009_00437	1587	0	0	0	0.000	0.000	0.000	Alkyl hydroperoxide reductase subunit F
bin009	SOY3_bin009_00438	1782	0	2	3	0.000	0.114	0.179	hypothetical protein
bin009	SOY3_bin009_00439	486	4	2	3	0.984	0.417	0.656	Putative oxidoreductase CatD
bin009	SOY3_bin009_00440	729	0	1	3	0.000	0.139	0.437	putative transcriptional regulatory protein TcrX
bin009	SOY3_bin009_00441	1515	0	2	0	0.000	0.134	0.000	putative sensor histidine kinase TcrY
bin009	SOY3_bin009_00442	657	0	0	1	0.000	0.000	0.162	Deoxyribose-phosphate aldolase
bin009	SOY3_bin009_00443	618	0	2	1	0.000	0.328	0.172	Energy-coupling factor transporter transmembrane protein BioN
bin009	SOY3_bin009_00444	696	0	4	0	0.000	0.583	0.000	Biotin transport ATP-binding protein BioM
bin009	SOY3_bin009_00445	639	1	0	0	0.187	0.000	0.000	Biotin transporter BioY
bin009	SOY3_bin009_00446	1506	21	48	21	1.667	3.233	1.481	Cytochrome bd-I ubiquinol oxidase subunit 1
bin009	SOY3_bin009_00447	1095	19	24	16	2.074	2.223	1.552	Cytochrome bd-I ubiquinol oxidase subunit 2
bin009	SOY3_bin009_00448	1701	2	4	0	0.141	0.239	0.000	ATP-binding/permease protein CydD
bin009	SOY3_bin009_00449	954	0	0	1	0.000	0.000	0.111	hypothetical protein
bin009	SOY3_bin009_00450	1422	1	0	1	0.084	0.000	0.075	Neutral endopeptidase
bin009	SOY3_bin009_00451	384	1	10	6	0.311	2.641	1.660	Inner membrane protein YhaH
bin009	SOY3_bin009_00452	1017	0	3	0	0.000	0.299	0.000	Hemin transport system permease protein HmuU
bin009	SOY3_bin009_00453	777	0	0	0	0.000	0.000	0.000	putative siderophore transport system ATP-binding protein YusV
bin009	SOY3_bin009_00454	945	0	0	1	0.000	0.000	0.112	Putative ABC transporter substrate-binding lipoprotein YhfQ precursor
bin009	SOY3_bin009_00455	966	2	8	4	0.248	0.840	0.440	Heptaprenyl diphosphate synthase component 2
bin009	SOY3_bin009_00456	1551	2	9	4	0.154	0.589	0.274	NADH-quinone oxidoreductase subunit N
bin009	SOY3_bin009_00457	1530	7	19	6	0.547	1.260	0.417	NADH-quinone oxidoreductase subunit M
bin009	SOY3_bin009_00458	1911	7	30	14	0.438	1.592	0.778	NADH-quinone oxidoreductase subunit 12
bin009	SOY3_bin009_00459	300	1	1	3	0.398	0.338	1.062	NADH-quinone oxidoreductase subunit 11
bin009	SOY3_bin009_00460	903	3	7	4	0.397	0.786	0.471	NADH-quinone oxidoreductase subunit J
bin009	SOY3_bin009_00461	552	3	3	1	0.650	0.551	0.192	NADH-quinone oxidoreductase subunit 9
bin009	SOY3_bin009_00462	1350	5	19	5	0.443	1.427	0.393	NADH-quinone oxidoreductase subunit H
bin009	SOY3_bin009_00463	2418	11	32	15	0.544	1.342	0.659	NADH-quinone oxidoreductase subunit 3
bin009	SOY3_bin009_00464	1341	5	11	14	0.446	0.832	1.109	NADH-quinone oxidoreductase subunit F
bin009	SOY3_bin009_00465	744	1	8	6	0.161	1.091	0.857	NADH-quinone oxidoreductase subunit E

bin009	SOY3_bin009_00466	1371	7	13	9	0.610	0.962	0.697	NADH-quinone oxidoreductase subunit 4
bin009	SOY3_bin009_00467	831	12	11	6	1.726	1.343	0.767	NADH-quinone oxidoreductase subunit C/D
bin009	SOY3_bin009_00468	558	3	10	8	0.643	1.818	1.523	NADH-quinone oxidoreductase subunit 6
bin009	SOY3_bin009_00469	360	6	5	5	1.992	1.409	1.475	NAD(P)H-quinone oxidoreductase subunit 3
bin009	SOY3_bin009_00470	1332	5	14	5	0.449	1.066	0.399	Putative oxidoreductase/MT0587
bin009	SOY3_bin009_00471	1254	0	1	1	0.000	0.081	0.085	Isochorismate synthase DhbC
bin009	SOY3_bin009_00472	1701	0	0	0	0.000	0.000	0.000	hypothetical protein
bin009	SOY3_bin009_00473	1761	1	4	2	0.068	0.230	0.121	hypothetical protein
bin009	SOY3_bin009_00474	690	0	1	1	0.000	0.147	0.154	SkfA peptide export ATP-binding protein SkfE
bin009	SOY3_bin009_00475	987	27	40	35	3.270	4.111	3.767	Ubiquinone/menaquinone biosynthesis C-methyltransferase UbiE
bin009	SOY3_bin009_00476	1854	0	1	2	0.000	0.055	0.115	hypothetical protein
bin009	SOY3_bin009_00477	1587	0	1	1	0.000	0.064	0.067	2-succinyl-5-enolpyruvyl-6-hydroxy-3-cyclohexene-1-carboxylate synthase
bin009	SOY3_bin009_00478	975	1	8	3	0.123	0.832	0.327	Muconate cycloisomerase 1
bin009	SOY3_bin009_00479	879	1	4	3	0.136	0.462	0.363	1,4-dihydroxy-2-naphthoate octaprenyltransferase
bin009	SOY3_bin009_00480	1065	0	0	0	0.000	0.000	0.000	2-succinylbenzoate--CoA ligase
bin009	SOY3_bin009_00481	252	2	6	3	0.949	2.415	1.265	Negative regulatory protein YxlE
bin009	SOY3_bin009_00482	1290	0	0	1	0.000	0.000	0.082	putative multidrug-efflux transporter/MT1297
bin009	SOY3_bin009_00483	336	1	1	0	0.356	0.302	0.000	PRC-barrel domain protein
bin009	SOY3_bin009_00484	786	10	17	10	1.521	2.194	1.351	PRC-barrel domain protein
bin009	SOY3_bin009_00485	951	0	0	0	0.000	0.000	0.000	Ferrous-iron efflux pump FieF
bin009	SOY3_bin009_00486	882	1	1	1	0.136	0.115	0.120	3-demethylubiquinone-9 3-methyltransferase
bin009	SOY3_bin009_00487	1350	0	1	0	0.000	0.075	0.000	D-serine dehydratase
bin009	SOY3_bin009_00488	906	0	0	0	0.000	0.000	0.000	Nuclease-related domain protein
bin009	SOY3_bin009_00489	1002	0	2	0	0.000	0.202	0.000	HTH-type transcriptional activator TipA
bin009	SOY3_bin009_00490	648	0	0	0	0.000	0.000	0.000	hypothetical protein
bin009	SOY3_bin009_00491	1476	1	4	0	0.081	0.275	0.000	hypothetical protein
bin009	SOY3_bin009_00492	699	0	0	0	0.000	0.000	0.000	hypothetical protein
bin009	SOY3_bin009_00493	1155	0	2	0	0.000	0.176	0.000	Acetyl esterase
bin009	SOY3_bin009_00494	522	0	0	0	0.000	0.000	0.000	Putative DNA ligase-like protein/MT0965
bin009	SOY3_bin009_00495	681	0	1	1	0.000	0.149	0.156	Response regulator ArlR
bin009	SOY3_bin009_00496	1044	1	1	0	0.115	0.097	0.000	Signal-transduction histidine kinase senX3
bin009	SOY3_bin009_00497	861	0	2	1	0.000	0.236	0.123	Thiamine biosynthesis lipoprotein ApbE precursor
bin009	SOY3_bin009_00498	483	0	0	0	0.000	0.000	0.000	Na(+)-translocating NADH-quinone reductase subunit C
bin009	SOY3_bin009_00499	1317	1	0	1	0.091	0.000	0.081	2-halobenzoate 1,2-dioxygenase electron transfer component
bin009	SOY3_bin009_00500	1017	1	3	1	0.118	0.299	0.104	Putative thiamine biosynthesis protein
bin009	SOY3_bin009_00501	807	0	0	0	0.000	0.000	0.000	Aliphatic sulfonates import ATP-binding protein SsuB
bin009	SOY3_bin009_00502	1632	3	4	3	0.220	0.249	0.195	Putative aliphatic sulfonates transport permease protein SsuC
bin009	SOY3_bin009_00503	1050	18	22	15	2.049	2.125	1.518	Mannosylfructose-phosphate synthase
bin009	SOY3_bin009_00504	1185	0	3	2	0.000	0.257	0.179	Alkanal monooxygenase alpha chain
bin009	SOY3_bin009_00505	540	0	0	1	0.000	0.000	0.197	Multiple antibiotic resistance protein MarR
bin009	SOY3_bin009_00506	732	0	1	0	0.000	0.139	0.000	NAD(P)H-dependent FAD/FMN reductase
bin009	SOY3_bin009_00507	621	0	0	0	0.000	0.000	0.000	Vitamin K epoxide reductase family protein
bin009	SOY3_bin009_00508	768	0	0	0	0.000	0.000	0.000	Serine/threonine-protein kinase PknE
bin009	SOY3_bin009_00509	501	2	0	2	0.477	0.000	0.424	hypothetical protein
bin009	SOY3_bin009_00510	156	0	0	0	0.000	0.000	0.000	Bacterial regulatory proteins, gntR family
bin009	SOY3_bin009_00511	687	0	0	0	0.000	0.000	0.000	hypothetical protein
bin009	SOY3_bin009_00512	591	2	5	2	0.405	0.858	0.359	putative HTH-type transcriptional regulator TtgW
bin009	SOY3_bin009_00513	660	3	8	1	0.543	1.229	0.161	putative sugar epimerase YhfK
bin009	SOY3_bin009_00514	690	0	0	0	0.000	0.000	0.000	Glycyl-glycine endopeptidase LytM precursor
bin009	SOY3_bin009_00515	528	0	1	0	0.000	0.192	0.000	Helix-turn-helix domain protein
bin009	SOY3_bin009_00516	879	0	1	2	0.000	0.115	0.242	hypothetical protein
bin009	SOY3_bin009_00517	336	0	0	0	0.000	0.000	0.000	hypothetical protein
bin009	SOY3_bin009_00518	1095	1	3	0	0.109	0.278	0.000	Carboxylate-amine ligase YbdK
bin009	SOY3_bin009_00519	1296	0	1	0	0.000	0.078	0.000	Proton glutamate symport protein
bin009	SOY3_bin009_00520	1257	0	0	0	0.000	0.000	0.000	N-carbamoyl-L-amino acid hydrolase
bin009	SOY3_bin009_00521	1116	0	0	0	0.000	0.000	0.000	Purine catabolism protein PucG
bin009	SOY3_bin009_00522	750	1	0	0	0.159	0.000	0.000	HTH-type transcriptional regulator LutR
bin009	SOY3_bin009_00523	82	0	0	0	0.000	0.000	0.000	tRNA-Val(gac)
bin009	SOY3_bin009_00524	1350	0	0	0	0.000	0.000	0.000	putative HTH-type transcriptional regulator YdcR
bin009	SOY3_bin009_00525	888	1	1	2	0.135	0.114	0.239	Pyridoxal biosynthesis lyase PdxS
bin009	SOY3_bin009_00526	675	0	1	1	0.000	0.150	0.157	Glutamine amidotransferase subunit PdxT
bin009	SOY3_bin009_00527	162	1	2	1	0.738	1.252	0.656	hypothetical protein
bin009	SOY3_bin009_00528	1698	1	7	6	0.070	0.418	0.375	Coenzyme A disulfide reductase
bin009	SOY3_bin009_00529	273	0	1	0	0.000	0.372	0.000	Copper-sensing transcriptional repressor CsoR
bin009	SOY3_bin009_00530	162	0	0	0	0.000	0.000	0.000	hypothetical protein
bin009	SOY3_bin009_00531	774	0	1	1	0.000	0.131	0.137	hypothetical protein
bin009	SOY3_bin009_00532	1251	0	0	0	0.000	0.000	0.000	D-amino acid dehydrogenase small subunit

bin009	SOY3_bin009_00533	381	0	0	0	0.000	0.000	0.000	Putative reactive intermediate deaminase TdcF
bin009	SOY3_bin009_00534	819	0	0	0	0.000	0.000	0.000	Glutamine transport ATP-binding protein GlnQ
bin009	SOY3_bin009_00535	864	3	2	3	0.415	0.235	0.369	Glutamine-binding periplasmic protein precursor
bin009	SOY3_bin009_00536	657	0	1	0	0.000	0.154	0.000	L-cystine transport system permease protein TcyB
bin009	SOY3_bin009_00537	639	0	1	0	0.000	0.159	0.000	Inner membrane amino-acid ABC transporter permease protein YecS
bin009	SOY3_bin009_00538	1065	0	1	0	0.000	0.095	0.000	Formaldehyde dismutase
bin009	SOY3_bin009_00539	990	0	1	1	0.000	0.102	0.107	D-lactate dehydrogenase
bin009	SOY3_bin009_00540	1293	0	0	1	0.000	0.000	0.082	5-aminovalerate aminotransferase DavT
bin009	SOY3_bin009_00541	1488	1	0	0	0.080	0.000	0.000	Purine catabolism regulatory protein
bin009	SOY3_bin009_00542	312	8	17	15	3.065	5.526	5.107	hypothetical protein
bin009	SOY3_bin009_00543	609	0	3	0	0.000	0.500	0.000	Uridine kinase
bin009	SOY3_bin009_00544	1860	0	0	1	0.000	0.000	0.057	Putative HMP/thiamine import ATP-binding protein YkoD
bin009	SOY3_bin009_00545	297	0	0	0	0.000	0.000	0.000	RNA polymerase-binding transcription factor DksA
bin009	SOY3_bin009_00546	2700	1	1	0	0.044	0.038	0.000	Magnesium-transporting ATPase, P-type 1
bin009	SOY3_bin009_00547	282	1	0	0	0.424	0.000	0.000	hypothetical protein
bin009	SOY3_bin009_00548	1338	0	0	1	0.000	0.000	0.079	Inner membrane transport protein YnfM
bin009	SOY3_bin009_00549	891	1	1	0	0.134	0.114	0.000	HTH-type transcriptional regulator GltC
bin009	SOY3_bin009_00550	1371	2	0	1	0.174	0.000	0.077	hypothetical protein
bin009	SOY3_bin009_00551	675	1	3	1	0.177	0.451	0.157	hypothetical protein
bin009	SOY3_bin009_00552	939	11	28	15	1.400	3.024	1.697	Universal stress protein/MT2698
bin009	SOY3_bin009_00553	1386	6	16	13	0.518	1.171	0.996	hypothetical protein
bin009	SOY3_bin009_00554	1311	0	1	1	0.000	0.077	0.081	Multidrug resistance protein MdtK
bin009	SOY3_bin009_00555	1437	0	5	3	0.000	0.353	0.222	Oxalate:formate antiporter
bin009	SOY3_bin009_00556	1533	1	3	3	0.078	0.198	0.208	Antiseptic resistance protein
bin009	SOY3_bin009_00557	933	1	5	3	0.128	0.544	0.342	Putative aliphatic sulfonates transport permease protein SsuC
bin009	SOY3_bin009_00558	1014	5	5	2	0.589	0.500	0.210	Putative thiamine biosynthesis protein
bin009	SOY3_bin009_00559	1302	2	1	2	0.184	0.078	0.163	Methionine gamma-lyase
bin009	SOY3_bin009_00560	1158	0	2	2	0.000	0.175	0.183	Homoserine O-acetyltransferase
bin009	SOY3_bin009_00561	642	0	2	1	0.000	0.316	0.165	Ubiquinone biosynthesis O-methyltransferase
bin009	SOY3_bin009_00562	933	1	0	1	0.128	0.000	0.114	DNA polymerase I
bin009	SOY3_bin009_00563	480	0	0	0	0.000	0.000	0.000	HTH-type transcriptional regulator LrpC
bin009	SOY3_bin009_00564	816	5	9	8	0.733	1.119	1.041	Undecaprenyl-phosphate mannosyltransferase
bin009	SOY3_bin009_00565	339	13	32	12	4.584	9.574	3.760	RNA polymerase-binding protein RbpA
bin009	SOY3_bin009_00566	789	1	1	1	0.152	0.129	0.135	putative glycerophosphoryl diester phosphodiesterase 1
bin009	SOY3_bin009_00567	459	0	6	1	0.000	1.326	0.231	Putative transmembrane protein (PGPGW)
bin009	SOY3_bin009_00568	930	0	2	0	0.000	0.218	0.000	Pyridoxamine kinase
bin009	SOY3_bin009_00569	1545	2	3	0	0.155	0.197	0.000	UDP-N-acetylglucosamine 1-carboxyvinyltransferase
bin009	SOY3_bin009_00570	1404	6	12	10	0.511	0.867	0.757	Dihydrolipoyl dehydrogenase
bin009	SOY3_bin009_00571	1161	16	52	37	1.648	4.543	3.385	Leucine-, isoleucine-, valine-, threonine-, and alanine-binding protein precursor
bin009	SOY3_bin009_00572	885	0	3	1	0.000	0.344	0.120	High-affinity branched-chain amino acid transport system permease protein LivH
bin009	SOY3_bin009_00573	981	0	3	0	0.000	0.310	0.000	High-affinity branched-chain amino acid transport system permease protein LivH
bin009	SOY3_bin009_00574	789	0	0	1	0.000	0.000	0.135	Lipopolysaccharide export system ATP-binding protein LptB
bin009	SOY3_bin009_00575	705	1	2	3	0.170	0.288	0.452	High-affinity branched-chain amino acid transport ATP-binding protein LivF
bin009	SOY3_bin009_00576	642	3	5	0	0.559	0.790	0.000	Inosine-5'-monophosphate dehydrogenase
bin009	SOY3_bin009_00577	1173	4	4	2	0.408	0.346	0.181	hypothetical protein
bin009	SOY3_bin009_00578	669	0	0	1	0.000	0.000	0.159	IMPACT family member YigZ
bin009	SOY3_bin009_00579	786	0	0	0	0.000	0.000	0.000	hypothetical protein
bin009	SOY3_bin009_00580	1932	14	19	7	0.866	0.997	0.385	NADH oxidase
bin009	SOY3_bin009_00581	849	5	27	4	0.704	3.226	0.500	acetoacetate decarboxylase
bin009	SOY3_bin009_00582	858	7	14	7	0.975	1.655	0.867	putative oxidoreductase
bin009	SOY3_bin009_00583	1053	9	22	3	1.022	2.119	0.303	hypothetical protein
bin009	SOY3_bin009_00584	2373	3	5	4	0.151	0.214	0.179	UvrABC system protein A
bin009	SOY3_bin009_00585	621	0	1	0	0.000	0.163	0.000	hypothetical protein
bin009	SOY3_bin009_00586	654	0	1	0	0.000	0.155	0.000	Copper-exporting P-type ATPase A
bin009	SOY3_bin009_00587	1347	241	490	309	21.389	36.896	24.368	hypothetical protein
bin009	SOY3_bin009_00588	1962	16	18	14	0.975	0.931	0.758	Isoniazid-induced protein IniA
bin009	SOY3_bin009_00589	1548	3	13	9	0.232	0.852	0.618	Isoniazid-induced protein IniC
bin009	SOY3_bin009_00590	639	18	45	17	3.368	7.143	2.826	hypothetical protein
bin009	SOY3_bin009_00591	810	48	104	45	7.084	13.023	5.901	hypothetical protein
bin009	SOY3_bin009_00592	2370	8	13	8	0.404	0.556	0.359	Spore germination protein GerE
bin009	SOY3_bin009_00593	1674	8	12	6	0.571	0.727	0.381	Chaperone protein DnaK
bin009	SOY3_bin009_00594	1833	0	5	7	0.000	0.277	0.406	Aspartate--tRNA ligase
bin009	SOY3_bin009_00595	780	0	4	4	0.000	0.520	0.545	Tyrosine-protein phosphatase precursor
bin009	SOY3_bin009_00596	1383	0	3	2	0.000	0.220	0.154	Replication-associated recombination protein A
bin009	SOY3_bin009_00597	969	0	0	1	0.000	0.000	0.110	hypothetical protein
bin009	SOY3_bin009_00598	2685	0	5	1	0.000	0.189	0.040	Alanine--tRNA ligase
bin009	SOY3_bin009_00599	468	0	1	2	0.000	0.217	0.454	Putative Holliday junction resolvase

bin009	SOY3_bin009_00600	1167	1	4	2	0.102	0.348	0.182	putative aminodeoxychorismate lyase
bin009	SOY3_bin009_00601	1197	0	4	6	0.000	0.339	0.532	Chorismate synthase
bin009	SOY3_bin009_00602	519	1	0	1	0.230	0.000	0.205	Shikimate kinase
bin009	SOY3_bin009_00603	1092	2	0	3	0.219	0.000	0.292	3-dehydroquinate synthase
bin009	SOY3_bin009_00604	687	3	0	1	0.522	0.000	0.155	Protease synthase and sporulation protein PAI 2
bin009	SOY3_bin009_00605	591	1	1	2	0.202	0.172	0.359	Elongation factor P
bin009	SOY3_bin009_00606	528	2	2	2	0.453	0.384	0.402	hypothetical protein
bin009	SOY3_bin009_00607	1137	0	2	3	0.000	0.178	0.280	Carbamoyl-phosphate synthase small chain
bin009	SOY3_bin009_00608	3324	3	8	6	0.108	0.244	0.192	Carbamoyl-phosphate synthase large chain
bin009	SOY3_bin009_00609	1098	3	3	3	0.327	0.277	0.290	Dihydroorotate dehydrogenase (quinone)
bin009	SOY3_bin009_00610	822	1	2	3	0.145	0.247	0.388	orotidine 5'-phosphate decarboxylase
bin009	SOY3_bin009_00611	312	5	9	8	1.916	2.926	2.724	30S ribosomal protein S13
bin009	SOY3_bin009_00612	558	3	6	6	0.643	1.091	1.142	Guanylate kinase
bin009	SOY3_bin009_00613	342	3	2	2	1.049	0.593	0.621	DNA-directed RNA polymerase subunit omega
bin009	SOY3_bin009_00614	1194	4	0	3	0.400	0.000	0.267	S-adenosylmethionine synthase
bin009	SOY3_bin009_00615	1977	0	2	0	0.000	0.103	0.000	Primosomal protein N'
bin009	SOY3_bin009_00616	930	0	1	2	0.000	0.109	0.228	Methionyl-tRNA formyltransferase
bin009	SOY3_bin009_00617	1365	0	1	0	0.000	0.074	0.000	Ribosomal RNA small subunit methyltransferase B
bin009	SOY3_bin009_00618	660	0	3	1	0.000	0.461	0.161	Ribulose-phosphate 3-epimerase
bin009	SOY3_bin009_00619	1002	1	2	0	0.119	0.202	0.000	hypothetical protein
bin009	SOY3_bin009_00620	138	0	0	0	0.000	0.000	0.000	hypothetical protein
bin009	SOY3_bin009_00621	501	1	1	0	0.239	0.202	0.000	hypothetical protein
bin009	SOY3_bin009_00622	861	2	7	0	0.278	0.825	0.000	hypothetical protein
bin009	SOY3_bin009_00623	363	0	0	0	0.000	0.000	0.000	hypothetical protein
bin009	SOY3_bin009_00624	366	0	0	0	0.000	0.000	0.000	hypothetical protein
bin009	SOY3_bin009_00625	801	3	1	1	0.448	0.127	0.133	hypothetical protein
bin009	SOY3_bin009_00626	240	0	0	0	0.000	0.000	0.000	hypothetical protein
bin009	SOY3_bin009_00627	1089	0	1	0	0.000	0.093	0.000	HTH-type transcriptional repressor CytR
bin009	SOY3_bin009_00628	1005	1	1	1	0.119	0.101	0.106	2,3-diketo-L-gulonate-binding periplasmic protein YiaO precursor
bin009	SOY3_bin009_00629	594	0	0	0	0.000	0.000	0.000	Sialic acid TRAP transporter permease protein SiaT
bin009	SOY3_bin009_00630	1290	0	1	0	0.000	0.079	0.000	Sialic acid TRAP transporter permease protein SiaT
bin009	SOY3_bin009_00631	74	0	0	0	0.000	0.000	0.000	tRNA-Thr(tgt)
bin009	SOY3_bin009_00632	996	0	0	0	0.000	0.000	0.000	Hca operon transcriptional activator
bin009	SOY3_bin009_00633	1017	0	0	1	0.000	0.000	0.104	methylcobalamin:coenzyme M methyltransferase
bin009	SOY3_bin009_00634	2019	0	1	1	0.000	0.050	0.053	Acetophenone carboxylase delta subunit
bin009	SOY3_bin009_00635	2112	0	0	0	0.000	0.000	0.000	Acetophenone carboxylase gamma subunit
bin009	SOY3_bin009_00636	810	0	0	0	0.000	0.000	0.000	Inner membrane ABC transporter permease protein YdcV
bin009	SOY3_bin009_00637	882	0	0	0	0.000	0.000	0.000	Spermidine/putrescine transport system permease protein PotB
bin009	SOY3_bin009_00638	1077	0	0	0	0.000	0.000	0.000	Spermidine/putrescine import ATP-binding protein PotA
bin009	SOY3_bin009_00639	1119	0	0	0	0.000	0.000	0.000	Spermidine/putrescine-binding periplasmic protein precursor
bin009	SOY3_bin009_00640	1191	0	5	3	0.000	0.426	0.268	Mannose-6-phosphate isomerase
bin009	SOY3_bin009_00641	285	0	0	0	0.000	0.000	0.000	hypothetical protein
bin009	SOY3_bin009_00642	444	0	0	0	0.000	0.000	0.000	hypothetical protein
bin009	SOY3_bin009_00643	1620	10	15	16	0.738	0.939	1.049	60 kDa chaperonin 1
bin009	SOY3_bin009_00644	780	1	1	2	0.153	0.130	0.272	Copper homeostasis protein CutC
bin009	SOY3_bin009_00645	534	0	0	1	0.000	0.000	0.199	Phosphatase NudJ
bin009	SOY3_bin009_00646	849	0	0	1	0.000	0.000	0.125	hypothetical protein
bin009	SOY3_bin009_00647	966	4	2	1	0.495	0.210	0.110	hypothetical protein
bin009	SOY3_bin009_00648	384	3	9	3	0.934	2.377	0.830	Cold shock-like protein
bin009	SOY3_bin009_00649	765	3	5	0	0.469	0.663	0.000	hypothetical protein
bin009	SOY3_bin009_00650	309	3	5	1	1.161	1.641	0.344	hypothetical protein
bin009	SOY3_bin009_00651	1536	0	8	7	0.000	0.528	0.484	Guanine/hypoxanthine permease PbuG
bin009	SOY3_bin009_00652	657	4	2	2	0.728	0.309	0.323	hypothetical protein
bin009	SOY3_bin009_00653	1023	2	3	3	0.234	0.297	0.312	hypothetical protein
bin009	SOY3_bin009_00654	693	0	4	0	0.000	0.585	0.000	peptide chain release factor 1
bin009	SOY3_bin009_00655	693	2	3	3	0.345	0.439	0.460	Iron-dependent repressor IdeR
bin009	SOY3_bin009_00656	76	0	0	0	0.000	0.000	0.000	tRNA-Arg(cct)
bin009	SOY3_bin009_00657	546	0	5	3	0.000	0.929	0.584	FMN-dependent NADPH-azoreductase
bin009	SOY3_bin009_00658	1011	0	4	1	0.000	0.401	0.105	Adenine deaminase
bin009	SOY3_bin009_00659	378	3	1	3	0.949	0.268	0.843	hypothetical protein
bin009	SOY3_bin009_00660	963	1	0	0	0.124	0.000	0.000	hypothetical protein
bin009	SOY3_bin009_00661	369	0	3	1	0.000	0.825	0.288	Large-conductance mechanosensitive channel
bin009	SOY3_bin009_00662	618	0	0	0	0.000	0.000	0.000	flagellar basal body P-ring biosynthesis protein FlgA
bin009	SOY3_bin009_00663	306	2	1	1	0.781	0.331	0.347	Zinc ribbon domain protein
bin009	SOY3_bin009_00664	639	0	1	2	0.000	0.159	0.332	5-formyltetrahydrofolate cyclo-ligase family protein
bin009	SOY3_bin009_00665	1266	1	4	0	0.094	0.320	0.000	Molybdopterin molybdenumtransferase
bin009	SOY3_bin009_00666	714	0	0	2	0.000	0.000	0.298	Putative ribosomal N-acetyltransferase YdaF

bin009	SOY3_bin009_00667	762	1	3	1	0.157	0.399	0.139	hypothetical protein
bin009	SOY3_bin009_00668	276	0	0	0	0.000	0.000	0.000	3-hydroxybenzoate--CoA/4-hydroxybenzoate--CoA ligase
bin009	SOY3_bin009_00669	915	0	0	0	0.000	0.000	0.000	3 beta-hydroxysteroid dehydrogenase/Delta 5-->4-isomerase
bin009	SOY3_bin009_00670	381	0	0	0	0.000	0.000	0.000	HTH-type transcriptional repressor YtrA
bin009	SOY3_bin009_00671	894	1	2	1	0.134	0.227	0.119	ABC transporter ATP-binding protein YtrB
bin009	SOY3_bin009_00672	696	1	2	0	0.172	0.291	0.000	hypothetical protein
bin009	SOY3_bin009_00673	705	1	2	1	0.170	0.288	0.151	hypothetical protein
bin009	SOY3_bin009_00674	1251	0	0	0	0.000	0.000	0.000	Aliphatic amidase expression-regulating protein
bin009	SOY3_bin009_00675	885	0	1	0	0.000	0.115	0.000	High-affinity branched-chain amino acid transport system permease protein LivH
bin009	SOY3_bin009_00676	1128	0	0	0	0.000	0.000	0.000	Autoinducer 2 import system permease protein LsrC
bin009	SOY3_bin009_00677	804	0	0	0	0.000	0.000	0.000	Sulfate/thiosulfate import ATP-binding protein CysA
bin009	SOY3_bin009_00678	696	0	0	0	0.000	0.000	0.000	High-affinity branched-chain amino acid transport ATP-binding protein LivF
bin009	SOY3_bin009_00679	708	0	0	0	0.000	0.000	0.000	Putative SOS response-associated peptidase YedK
bin009	SOY3_bin009_00680	501	1	3	1	0.239	0.607	0.212	hypothetical protein
bin009	SOY3_bin009_00681	1098	2	8	3	0.218	0.739	0.290	hypothetical protein
bin009	SOY3_bin009_00682	1647	27	69	41	1.960	4.249	2.644	Anaerobic glycerol-3-phosphate dehydrogenase subunit A
bin009	SOY3_bin009_00683	1230	12	30	17	1.166	2.474	1.468	Anaerobic glycerol-3-phosphate dehydrogenase subunit B
bin009	SOY3_bin009_00684	1347	11	32	18	0.976	2.410	1.419	Anaerobic glycerol-3-phosphate dehydrogenase subunit C
bin009	SOY3_bin009_00685	741	17	42	16	2.743	5.749	2.294	putative glycerol uptake facilitator protein
bin009	SOY3_bin009_00686	1530	44	107	40	3.438	7.093	2.777	Glycerol kinase
bin009	SOY3_bin009_00687	618	1	2	1	0.193	0.328	0.172	hypothetical protein
bin009	SOY3_bin009_00688	1218	1	2	0	0.098	0.167	0.000	p-aminobenzoyl-glutamate hydrolase subunit B
bin009	SOY3_bin009_00689	1374	1	0	0	0.087	0.000	0.000	Mitomycin radical oxidase
bin009	SOY3_bin009_00690	1116	1	2	0	0.107	0.182	0.000	Transcriptional regulatory protein DegU
bin009	SOY3_bin009_00691	1104	0	0	0	0.000	0.000	0.000	Putative prophage phiRv2 integrase
bin009	SOY3_bin009_00692	498	0	0	0	0.000	0.000	0.000	hypothetical protein
bin009	SOY3_bin009_00693	219	0	0	0	0.000	0.000	0.000	Helix-turn-helix domain protein
bin009	SOY3_bin009_00694	309	0	0	0	0.000	0.000	0.000	hypothetical protein
bin009	SOY3_bin009_00695	330	0	0	0	0.000	0.000	0.000	hypothetical protein
bin009	SOY3_bin009_00696	396	0	0	0	0.000	0.000	0.000	hypothetical protein
bin009	SOY3_bin009_00697	1446	0	0	0	0.000	0.000	0.000	DNA repair protein RadA
bin009	SOY3_bin009_00698	387	0	0	0	0.000	0.000	0.000	hypothetical protein
bin009	SOY3_bin009_00699	300	0	0	0	0.000	0.000	0.000	hypothetical protein
bin009	SOY3_bin009_00700	333	0	0	0	0.000	0.000	0.000	Transcriptional regulator WhiB
bin009	SOY3_bin009_00701	222	0	0	0	0.000	0.000	0.000	hypothetical protein
bin009	SOY3_bin009_00702	360	0	0	0	0.000	0.000	0.000	Helix-turn-helix domain protein
bin009	SOY3_bin009_00703	441	0	0	0	0.000	0.000	0.000	Mu-like prophage I protein
bin009	SOY3_bin009_00704	261	0	0	0	0.000	0.000	0.000	hypothetical protein
bin009	SOY3_bin009_00705	270	0	0	0	0.000	0.000	0.000	hypothetical protein
bin009	SOY3_bin009_00706	903	0	0	0	0.000	0.000	0.000	hypothetical protein
bin009	SOY3_bin009_00707	417	0	0	0	0.000	0.000	0.000	hypothetical protein
bin009	SOY3_bin009_00708	402	0	0	0	0.000	0.000	0.000	hypothetical protein
bin009	SOY3_bin009_00709	123	0	0	0	0.000	0.000	0.000	hypothetical protein
bin009	SOY3_bin009_00710	522	0	2	0	0.000	0.389	0.000	hypothetical protein
bin009	SOY3_bin009_00711	219	0	0	0	0.000	0.000	0.000	hypothetical protein
bin009	SOY3_bin009_00712	75	1	0	0	1.594	0.000	0.000	tRNA-Val(gac)
bin009	SOY3_bin009_00713	72	0	0	2	0.000	0.000	2.951	tRNA-Cys(gca)
bin009	SOY3_bin009_00714	74	0	0	0	0.000	0.000	0.000	tRNA-Gly(gcc)
bin009	SOY3_bin009_00715	74	2	0	0	3.231	0.000	0.000	tRNA-Val(cac)
bin009	SOY3_bin009_00716	801	0	2	2	0.000	0.253	0.265	sirohydrochlorin cobaltochelatase
bin009	SOY3_bin009_00717	1083	0	2	2	0.000	0.187	0.196	putative ATP-dependent DNA ligase YkoU
bin009	SOY3_bin009_00718	456	3	0	1	0.787	0.000	0.233	Peptide methionine sulfoxide reductase MsrB
bin009	SOY3_bin009_00719	573	0	2	1	0.000	0.354	0.185	hypothetical protein
bin009	SOY3_bin009_00720	1263	0	4	1	0.000	0.321	0.084	Ribonuclease D
bin009	SOY3_bin009_00721	903	0	2	1	0.000	0.225	0.118	1-deoxy-D-xylulose-5-phosphate synthase
bin009	SOY3_bin009_00722	1014	4	2	2	0.472	0.200	0.210	3-phosphoshikimate 1-carboxyvinyltransferase
bin009	SOY3_bin009_00723	1020	3	7	2	0.352	0.696	0.208	Putative ribosome biogenesis GTPase RsgA
bin009	SOY3_bin009_00724	843	1	4	2	0.142	0.481	0.252	Histidinol-phosphatase
bin009	SOY3_bin009_00725	1188	0	4	3	0.000	0.342	0.268	D-3-phosphoglycerate dehydrogenase
bin009	SOY3_bin009_00726	789	0	0	1	0.000	0.000	0.135	Oligopeptide transport ATP-binding protein OppF
bin009	SOY3_bin009_00727	846	0	0	0	0.000	0.000	0.000	putative D,D-dipeptide transport ATP-binding protein DdpD
bin009	SOY3_bin009_00728	819	0	1	0	0.000	0.124	0.000	Glutathione transport system permease protein GsiD
bin009	SOY3_bin009_00729	951	1	1	2	0.126	0.107	0.223	Glutathione transport system permease protein GsiC
bin009	SOY3_bin009_00730	1506	1	17	6	0.079	1.145	0.423	Heme-binding protein A precursor
bin009	SOY3_bin009_00731	75	0	0	1	0.000	0.000	1.416	tRNA-Met(cat)
bin009	SOY3_bin009_00732	969	0	0	0	0.000	0.000	0.000	hypothetical protein
bin009	SOY3_bin009_00733	1260	1	4	0	0.095	0.322	0.000	Divalent metal cation transporter MntH

bin009	SOY3_bin009_00734	1290	0	0	0	0.000	0.000	0.000	Protease 3 precursor
bin009	SOY3_bin009_00735	1350	1	0	2	0.089	0.000	0.157	Peptidase M16 inactive domain protein
bin009	SOY3_bin009_00736	2895	4	7	4	0.165	0.245	0.147	hypothetical protein
bin009	SOY3_bin009_00737	552	3	1	1	0.650	0.184	0.192	hypothetical protein
bin009	SOY3_bin009_00738	1047	2	5	1	0.228	0.484	0.101	Lon protease
bin009	SOY3_bin009_00739	1335	2	6	2	0.179	0.456	0.159	hypothetical protein
bin009	SOY3_bin009_00740	600	2	0	0	0.398	0.000	0.000	SprT-like family protein
bin009	SOY3_bin009_00741	162	1	14	11	0.738	8.765	7.213	Transcription elongation factor Elf1 like protein
bin009	SOY3_bin009_00742	1797	2	13	8	0.133	0.734	0.473	ATP-dependent DNA helicase UvrD2
bin009	SOY3_bin009_00743	3156	0	0	3	0.000	0.000	0.101	RecBCD enzyme subunit RecC
bin009	SOY3_bin009_00744	3276	0	5	0	0.000	0.155	0.000	RecBCD enzyme subunit RecB
bin009	SOY3_bin009_00745	1782	0	2	0	0.000	0.114	0.000	RecBCD enzyme subunit RecD
bin009	SOY3_bin009_00746	864	0	1	1	0.000	0.117	0.123	NADH pyrophosphatase
bin009	SOY3_bin009_00747	3222	0	2	1	0.000	0.063	0.033	ATP-dependent DNA helicase UvrD1
bin009	SOY3_bin009_00748	1728	0	1	2	0.000	0.059	0.123	ATP-dependent helicase/deoxyribonuclease subunit B
bin009	SOY3_bin009_00749	519	1	0	2	0.230	0.000	0.409	shikimate kinase
bin009	SOY3_bin009_00750	717	2	3	2	0.333	0.424	0.296	(alpha)-aspartyl dipeptidase
bin009	SOY3_bin009_00751	1173	4	0	8	0.408	0.000	0.724	N5-carboxyaminoimidazole ribonucleotide synthase
bin009	SOY3_bin009_00752	504	0	1	0	0.000	0.201	0.000	N5-carboxyaminoimidazole ribonucleotide mutase
bin009	SOY3_bin009_00753	573	0	3	1	0.000	0.531	0.185	hypothetical protein
bin009	SOY3_bin009_00754	1203	2	2	2	0.199	0.169	0.177	ABC-2 family transporter protein
bin009	SOY3_bin009_00755	882	1	3	1	0.136	0.345	0.120	ABC-type transporter ATP-binding protein EcsA
bin009	SOY3_bin009_00756	1248	0	2	0	0.000	0.163	0.000	Transcriptional regulator LytR
bin009	SOY3_bin009_00757	732	3	2	2	0.490	0.277	0.290	hypothetical protein
bin009	SOY3_bin009_00758	1059	3	2	1	0.339	0.192	0.100	Mannose-1-phosphate guanylyltransferase RfbM
bin009	SOY3_bin009_00759	807	0	3	1	0.000	0.377	0.132	hypothetical protein
bin009	SOY3_bin009_00760	264	1	4	2	0.453	1.537	0.805	Transcriptional regulator WhiB
bin009	SOY3_bin009_00761	3129	5	7	6	0.191	0.227	0.204	Glycosyl transferase family 2
bin009	SOY3_bin009_00762	1632	2	3	3	0.147	0.186	0.195	hypothetical protein
bin009	SOY3_bin009_00763	435	2	0	0	0.550	0.000	0.000	Possibl zinc metallo-peptidase
bin009	SOY3_bin009_00764	375	2	0	0	0.638	0.000	0.000	hypothetical protein
bin009	SOY3_bin009_00765	867	1	4	0	0.138	0.468	0.000	phosphoglycolate phosphatase
bin009	SOY3_bin009_00766	204	0	0	1	0.000	0.000	0.521	hypothetical protein
bin009	SOY3_bin009_00767	1038	0	1	3	0.000	0.098	0.307	hypothetical protein
bin009	SOY3_bin009_00768	1389	0	3	4	0.000	0.219	0.306	Adenosylhomocysteinase
bin009	SOY3_bin009_00769	264	3	2	2	1.359	0.768	0.805	hypothetical protein
bin009	SOY3_bin009_00770	1374	1	0	0	0.087	0.000	0.000	Multidrug resistance protein 3
bin009	SOY3_bin009_00771	813	0	1	1	0.000	0.125	0.131	RDD family protein
bin009	SOY3_bin009_00772	2154	1	2	0	0.056	0.094	0.000	4-alpha-glucanotransferase
bin009	SOY3_bin009_00773	585	0	1	0	0.000	0.173	0.000	Maleamate amidohydrolase
bin009	SOY3_bin009_00774	804	0	1	1	0.000	0.126	0.132	Hydroxyethylthiazole kinase
bin009	SOY3_bin009_00775	1440	0	2	1	0.000	0.141	0.074	Hydroxymethylpyrimidine/phosphomethylpyrimidine kinase
bin009	SOY3_bin009_00776	1356	2	2	4	0.176	0.150	0.313	Nicotinate phosphoribosyltransferase pncB1
bin009	SOY3_bin009_00777	312	0	1	0	0.000	0.325	0.000	ATP-dependent Clp protease adapter protein ClpS
bin009	SOY3_bin009_00778	624	1	2	0	0.192	0.325	0.000	hypothetical protein
bin009	SOY3_bin009_00779	819	0	4	3	0.000	0.495	0.389	Glutamate racemase
bin009	SOY3_bin009_00780	2841	9	22	14	0.379	0.785	0.523	putative FAD-linked oxidoreductase
bin009	SOY3_bin009_00781	702	3	0	0	0.511	0.000	0.000	Pyruvate dehydrogenase complex repressor
bin009	SOY3_bin009_00782	1695	26	39	28	1.834	2.334	1.755	L-lactate permease
bin009	SOY3_bin009_00783	669	0	0	2	0.000	0.000	0.318	Galactokinase
bin009	SOY3_bin009_00784	1293	5	2	0	0.462	0.157	0.000	D-alanyl-D-alanine carboxypeptidase precursor
bin009	SOY3_bin009_00785	1827	0	2	1	0.000	0.111	0.058	Putative multidrug export ATP-binding/permease protein
bin009	SOY3_bin009_00786	444	2	4	4	0.539	0.914	0.957	Multiple antibiotic resistance protein MarR
bin009	SOY3_bin009_00787	741	4	3	4	0.645	0.411	0.573	Chromosome partition protein Smc
bin009	SOY3_bin009_00788	1458	2	8	3	0.164	0.557	0.219	Xaa-Pro aminopeptidase 1
bin009	SOY3_bin009_00789	996	1	6	3	0.120	0.611	0.320	hypothetical protein
bin009	SOY3_bin009_00790	942	3	1	5	0.381	0.108	0.564	Malyl-CoA lyase
bin009	SOY3_bin009_00791	1299	0	1	0	0.000	0.078	0.000	Magnesium transporter MgtE
bin009	SOY3_bin009_00792	552	0	0	0	0.000	0.000	0.000	hypothetical protein
bin009	SOY3_bin009_00793	1185	1	0	0	0.101	0.000	0.000	Flagellum site-determining protein YlxH
bin009	SOY3_bin009_00794	1437	0	3	3	0.000	0.212	0.222	ATP-dependent RNA helicase RhlE
bin009	SOY3_bin009_00795	456	0	0	0	0.000	0.000	0.000	Sec-independent protein translocase protein TatB
bin009	SOY3_bin009_00796	882	1	7	2	0.136	0.805	0.241	4-hydroxy-tetrahydrodipicolinate synthase
bin009	SOY3_bin009_00797	642	1	5	1	0.186	0.790	0.165	Putative O-methyltransferase/MSMEI_4947
bin009	SOY3_bin009_00798	1224	22	40	14	2.149	3.315	1.215	Glucose-1-phosphate adenylyltransferase
bin009	SOY3_bin009_00799	1194	6	18	7	0.601	1.529	0.623	Capsular glucan synthase
bin009	SOY3_bin009_00800	168	2	5	3	1.423	3.019	1.897	hypothetical protein

bin009	SOY3_bin009_00801	336	0	1	1	0.000	0.302	0.316	DivIVA protein
bin009	SOY3_bin009_00802	1068	1	5	0	0.112	0.475	0.000	Succinyl-diaminopimelate desuccinylase
bin009	SOY3_bin009_00803	972	1	4	0	0.123	0.417	0.000	2,3,4,5-tetrahydropyridine-2,6-dicarboxylate N-succinyltransferase
bin009	SOY3_bin009_00804	885	2	0	1	0.270	0.000	0.120	hypothetical protein
bin009	SOY3_bin009_00805	1101	1	2	3	0.109	0.184	0.289	LL-diaminopimelate aminotransferase
bin009	SOY3_bin009_00806	318	4	4	4	1.504	1.276	1.336	Ferredoxin
bin009	SOY3_bin009_00807	1839	1	0	2	0.065	0.000	0.116	Vancomycin B-type resistance protein VanW
bin009	SOY3_bin009_00808	1401	9	25	9	0.768	1.810	0.682	Fumarate hydratase class II
bin009	SOY3_bin009_00809	1578	1	2	0	0.076	0.129	0.000	Putrescine importer PuuP
bin009	SOY3_bin009_00810	1377	0	2	1	0.000	0.147	0.077	Glutamate-1-semialdehyde 2,1-aminomutase
bin009	SOY3_bin009_00811	1425	0	6	2	0.000	0.427	0.149	Gamma-aminobutyraldehyde dehydrogenase
bin009	SOY3_bin009_00812	1341	0	3	2	0.000	0.227	0.158	5-aminovalerate aminotransferase DavT
bin009	SOY3_bin009_00813	3489	10	19	21	0.343	0.552	0.639	DNA-directed RNA polymerase subunit beta'
bin009	SOY3_bin009_00814	384	2	1	3	0.623	0.264	0.830	hypothetical protein
bin009	SOY3_bin009_00815	96	0	1	0	0.000	1.057	0.000	hypothetical protein
bin009	SOY3_bin009_00816	372	3	7	5	0.964	1.909	1.428	30S ribosomal protein S12
bin009	SOY3_bin009_00817	471	3	6	2	0.761	1.292	0.451	30S ribosomal protein S7
bin009	SOY3_bin009_00818	2100	7	11	9	0.398	0.531	0.455	Elongation factor G
bin009	SOY3_bin009_00819	1191	54	87	54	5.420	7.409	4.816	Elongation factor Tu
bin009	SOY3_bin009_00820	1362	2	2	1	0.176	0.149	0.078	chromosome segregation protein
bin009	SOY3_bin009_00821	489	0	0	1	0.000	0.000	0.217	hypothetical protein
bin009	SOY3_bin009_00822	429	10	25	14	2.787	5.911	3.467	Inosine-5'-monophosphate dehydrogenase
bin009	SOY3_bin009_00823	1464	0	2	2	0.000	0.139	0.145	Glutamate--tRNA ligase
bin009	SOY3_bin009_00824	312	7	9	3	2.682	2.926	1.021	30S ribosomal protein S10
bin009	SOY3_bin009_00825	660	6	8	4	1.087	1.229	0.644	50S ribosomal protein L3
bin009	SOY3_bin009_00826	699	1	18	9	0.171	2.612	1.368	50S ribosomal protein L4
bin009	SOY3_bin009_00827	309	1	4	2	0.387	1.313	0.688	50S ribosomal protein L23
bin009	SOY3_bin009_00828	837	3	11	8	0.428	1.333	1.015	50S ribosomal protein L2
bin009	SOY3_bin009_00829	282	4	4	5	1.696	1.439	1.883	30S ribosomal protein S19
bin009	SOY3_bin009_00830	420	1	4	3	0.285	0.966	0.759	50S ribosomal protein L22
bin009	SOY3_bin009_00831	837	4	12	7	0.571	1.454	0.888	30S ribosomal protein S3
bin009	SOY3_bin009_00832	417	2	7	4	0.573	1.703	1.019	50S ribosomal protein L16
bin009	SOY3_bin009_00833	246	2	4	3	0.972	1.649	1.295	50S ribosomal protein L29
bin009	SOY3_bin009_00834	276	1	8	4	0.433	2.940	1.540	30S ribosomal protein S17
bin009	SOY3_bin009_00835	369	5	7	3	1.620	1.924	0.864	50S ribosomal protein L14
bin009	SOY3_bin009_00836	369	1	5	4	0.324	1.374	1.151	50S ribosomal protein L24
bin009	SOY3_bin009_00837	684	4	9	6	0.699	1.335	0.932	50S ribosomal protein L5
bin009	SOY3_bin009_00838	186	1	4	4	0.643	2.181	2.284	30S ribosomal protein S14 type Z
bin009	SOY3_bin009_00839	408	1	6	1	0.293	1.492	0.260	30S ribosomal protein S8
bin009	SOY3_bin009_00840	543	1	7	8	0.220	1.308	1.565	50S ribosomal protein L6
bin009	SOY3_bin009_00841	384	0	3	6	0.000	0.792	1.660	50S ribosomal protein L18
bin009	SOY3_bin009_00842	612	3	10	5	0.586	1.657	0.868	30S ribosomal protein S5
bin009	SOY3_bin009_00843	183	0	1	1	0.000	0.554	0.580	50S ribosomal protein L30
bin009	SOY3_bin009_00844	444	5	10	9	1.346	2.284	2.153	50S ribosomal protein L15
bin009	SOY3_bin009_00845	1314	0	9	3	0.000	0.695	0.243	preprotein translocase subunit SecY
bin009	SOY3_bin009_00846	579	2	10	1	0.413	1.752	0.183	Adenylate kinase
bin009	SOY3_bin009_00847	837	2	7	5	0.286	0.848	0.635	Methionine aminopeptidase 1
bin009	SOY3_bin009_00848	288	0	2	1	0.000	0.704	0.369	YciI-like protein
bin009	SOY3_bin009_00849	222	5	12	6	2.693	5.483	2.871	Translation initiation factor IF-1
bin009	SOY3_bin009_00850	114	0	1	1	0.000	0.890	0.932	50S ribosomal protein L36
bin009	SOY3_bin009_00851	375	3	5	2	0.956	1.352	0.567	30S ribosomal protein S13
bin009	SOY3_bin009_00852	411	2	5	2	0.582	1.234	0.517	30S ribosomal protein S11
bin009	SOY3_bin009_00853	606	6	7	4	1.184	1.172	0.701	30S ribosomal protein S4
bin009	SOY3_bin009_00854	1026	1	6	1	0.117	0.593	0.104	DNA-directed RNA polymerase subunit alpha
bin009	SOY3_bin009_00855	567	3	12	4	0.633	2.147	0.749	50S ribosomal protein L17
bin009	SOY3_bin009_00856	1122	0	6	1	0.000	0.542	0.095	GDP-mannose-dependent alpha-(1-6)-phosphatidylinositol dimannoside mannosyltransferase
bin009	SOY3_bin009_00857	645	1	10	3	0.185	1.573	0.494	hypothetical protein
bin009	SOY3_bin009_00858	741	2	4	3	0.323	0.548	0.430	Resuscitation-promoting factor Rpf2 precursor
bin009	SOY3_bin009_00859	639	0	2	1	0.000	0.317	0.166	Response regulator protein VraR
bin009	SOY3_bin009_00860	1482	2	8	5	0.161	0.548	0.358	NAD(P) transhydrogenase subunit beta
bin009	SOY3_bin009_00861	1611	3	6	11	0.223	0.378	0.725	NAD(P) transhydrogenase subunit alpha
bin009	SOY3_bin009_00862	969	2	5	1	0.247	0.523	0.110	Putative 2-hydroxyacid dehydrogenase
bin009	SOY3_bin009_00863	588	0	0	0	0.000	0.000	0.000	Demethylmenaquinone methyltransferase
bin009	SOY3_bin009_00864	219	1	0	0	0.546	0.000	0.000	hypothetical protein
bin009	SOY3_bin009_00865	702	0	2	1	0.000	0.289	0.151	(S)-2-haloacid dehalogenase 4A
bin009	SOY3_bin009_00866	504	0	0	0	0.000	0.000	0.000	Acetyltransferase YpeA
bin009	SOY3_bin009_00867	1008	0	1	1	0.000	0.101	0.105	hypothetical protein

bin009	SOY3_bin009_00868	2046	0	2	1	0.000	0.099	0.052	Sodium, potassium, lithium and rubidium/H(+) antiporter
bin009	SOY3_bin009_00869	819	0	2	2	0.000	0.248	0.259	3-mercaptopyruvate sulfurtransferase
bin009	SOY3_bin009_00870	282	0	0	0	0.000	0.000	0.000	hypothetical protein
bin009	SOY3_bin009_00871	1008	0	1	1	0.000	0.101	0.105	Thioesterase domain protein
bin009	SOY3_bin009_00872	645	0	0	1	0.000	0.000	0.165	hypothetical protein
bin009	SOY3_bin009_00873	795	0	0	0	0.000	0.000	0.000	putative endonuclease 4
bin009	SOY3_bin009_00874	651	1	0	0	0.184	0.000	0.000	Streptothricin hydrolase
bin009	SOY3_bin009_00875	591	0	0	0	0.000	0.000	0.000	Arginine exporter protein ArgO
bin009	SOY3_bin009_00876	399	1	0	0	0.300	0.000	0.000	Inner membrane protein YgaP
bin009	SOY3_bin009_00877	291	2	8	2	0.822	2.788	0.730	30S ribosomal protein S6
bin009	SOY3_bin009_00878	552	8	18	3	1.733	3.307	0.577	Single-stranded DNA-binding protein
bin009	SOY3_bin009_00879	240	3	5	1	1.494	2.113	0.443	30S ribosomal protein S18 1
bin009	SOY3_bin009_00880	447	7	5	3	1.872	1.135	0.713	50S ribosomal protein L9
bin009	SOY3_bin009_00881	1050	5	9	2	0.569	0.869	0.202	Phosphoserine aminotransferase
bin009	SOY3_bin009_00882	1173	4	4	7	0.408	0.346	0.634	D-3-phosphoglycerate dehydrogenase
bin009	SOY3_bin009_00883	2547	2	3	4	0.094	0.119	0.167	phosphoenolpyruvate synthase
bin009	SOY3_bin009_00884	2106	7	11	10	0.397	0.530	0.504	Phosphate acetyltransferase
bin009	SOY3_bin009_00885	1284	0	0	4	0.000	0.000	0.331	hypothetical protein
bin009	SOY3_bin009_00886	1191	1	7	6	0.100	0.596	0.535	Acetate kinase
bin009	SOY3_bin009_00887	777	1	5	1	0.154	0.653	0.137	Gluconate 5-dehydrogenase
bin009	SOY3_bin009_00888	768	2	0	1	0.311	0.000	0.138	2-acyl-glycerophospho-ethanolamine acyltransferase
bin009	SOY3_bin009_00889	1923	2	4	1	0.124	0.211	0.055	Galactofuranosyl transferase GlfT1
bin009	SOY3_bin009_00890	756	0	2	2	0.000	0.268	0.281	Teichoic acids export ATP-binding protein TagH
bin009	SOY3_bin009_00891	894	0	0	0	0.000	0.000	0.000	Teichoic acid translocation permease protein TagG
bin009	SOY3_bin009_00892	519	0	1	0	0.000	0.195	0.000	hypothetical protein
bin009	SOY3_bin009_00893	2049	2	0	1	0.117	0.000	0.052	L-aspartate oxidase
bin009	SOY3_bin009_00894	1563	0	0	0	0.000	0.000	0.000	Acetate CoA-transferase YdiF
bin009	SOY3_bin009_00895	921	0	1	0	0.000	0.110	0.000	Prokaryotic cytochrome b561
bin009	SOY3_bin009_00896	984	0	2	0	0.000	0.206	0.000	Electron transfer flavoprotein subunit alpha
bin009	SOY3_bin009_00897	777	1	1	1	0.154	0.131	0.137	Electron transfer flavoprotein subunit beta
bin009	SOY3_bin009_00898	1008	0	0	1	0.000	0.000	0.105	HTH-type transcriptional repressor CytR
bin009	SOY3_bin009_00899	1155	5	2	2	0.518	0.176	0.184	Acyl-CoA dehydrogenase
bin009	SOY3_bin009_00900	1062	1	0	1	0.113	0.000	0.100	VanZ like family protein
bin009	SOY3_bin009_00901	630	0	0	0	0.000	0.000	0.000	HTH-type transcriptional repressor KstR
bin009	SOY3_bin009_00902	1671	2	1	1	0.143	0.061	0.064	Oleate hydratase
bin009	SOY3_bin009_00903	1482	0	0	0	0.000	0.000	0.000	Cardiolipin synthase
bin009	SOY3_bin009_00904	1446	5	9	10	0.413	0.631	0.735	Sucrose-6-phosphate hydrolase
bin009	SOY3_bin009_00905	1986	7	12	7	0.421	0.613	0.374	PTS system beta-glucoside-specific EIIBCA component
bin009	SOY3_bin009_00906	1008	0	2	0	0.000	0.201	0.000	HTH-type transcriptional regulator DegA
bin009	SOY3_bin009_00907	1797	1	2	4	0.067	0.113	0.236	putative ABC transporter ATP-binding protein YjjK
bin009	SOY3_bin009_00908	768	2	7	5	0.311	0.924	0.692	hypothetical protein
bin009	SOY3_bin009_00909	1749	15	21	25	1.025	1.218	1.518	Dihydrolipoyllysine-residue acetyltransferase component of pyruvate dehydrogenase complex
bin009	SOY3_bin009_00910	336	0	0	1	0.000	0.000	0.316	hypothetical protein
bin009	SOY3_bin009_00911	1527	1	4	3	0.078	0.266	0.209	Cytosol aminopeptidase
bin009	SOY3_bin009_00912	1098	3	1	0	0.327	0.092	0.000	Aminomethyltransferase
bin009	SOY3_bin009_00913	219	0	2	0	0.000	0.926	0.000	hypothetical protein
bin009	SOY3_bin009_00914	576	0	3	0	0.000	0.528	0.000	hypothetical protein
bin009	SOY3_bin009_00915	711	0	5	8	0.000	0.713	1.195	PspA/IM30 family protein
bin009	SOY3_bin009_00916	291	0	0	0	0.000	0.000	0.000	hypothetical protein
bin009	SOY3_bin009_00917	348	3	9	4	1.031	2.623	1.221	Iron-sulfur cluster insertion protein ErpA
bin009	SOY3_bin009_00918	1464	1	1	2	0.082	0.069	0.145	Inosine-5'-monophosphate dehydrogenase
bin009	SOY3_bin009_00919	489	4	10	3	0.978	2.074	0.652	putative response regulatory protein/MT3230
bin009	SOY3_bin009_00920	1095	14	44	21	1.528	4.076	2.037	Trehalose import ATP-binding protein SugC
bin009	SOY3_bin009_00921	1173	1	2	1	0.102	0.173	0.091	Cystathionine gamma-synthase
bin009	SOY3_bin009_00922	1389	0	3	1	0.000	0.219	0.076	Lipopolysaccharide kinase (Kdo/WaaP) family protein
bin009	SOY3_bin009_00923	366	0	2	2	0.000	0.554	0.580	hypothetical protein
bin009	SOY3_bin009_00924	1404	3	3	4	0.255	0.217	0.303	Cysteine--tRNA ligase
bin009	SOY3_bin009_00925	939	1	2	1	0.127	0.216	0.113	Putative TrmH family tRNA/rRNA methyltransferase
bin009	SOY3_bin009_00926	1533	7	9	13	0.546	0.595	0.901	Altronate dehydratase
bin009	SOY3_bin009_00927	735	3	4	2	0.488	0.552	0.289	putative HTH-type transcriptional regulator YdfH
bin009	SOY3_bin009_00928	1443	1	5	8	0.083	0.351	0.589	Serine hydroxymethyltransferase
bin009	SOY3_bin009_00929	600	1	2	3	0.199	0.338	0.531	Deoxycytidine triphosphate deaminase
bin009	SOY3_bin009_00930	822	0	1	0	0.000	0.123	0.000	Daunorubicin/doxorubicin resistance ABC transporter permease protein DrrB
bin009	SOY3_bin009_00931	822	0	2	0	0.000	0.247	0.000	Daunorubicin/doxorubicin resistance ATP-binding protein DrrA
bin009	SOY3_bin009_00932	77	1	0	0	1.553	0.000	0.000	tRNA-Ala(ggc)
bin009	SOY3_bin009_00933	672	0	0	0	0.000	0.000	0.000	Methionine import system permease protein MetP
bin009	SOY3_bin009_00934	984	2	2	1	0.243	0.206	0.108	Methionine import ATP-binding protein MetN

bin009	SOY3_bin009_00935	855	3	0	2	0.419	0.000	0.248	Methionine-binding lipoprotein MetQ precursor
bin009	SOY3_bin009_00936	1023	0	2	0	0.000	0.198	0.000	Hemin transport system permease protein HmuU
bin009	SOY3_bin009_00937	852	0	0	0	0.000	0.000	0.000	putative siderophore transport system ATP-binding protein YusV
bin009	SOY3_bin009_00938	585	0	0	2	0.000	0.000	0.363	hypothetical protein
bin009	SOY3_bin009_00939	1152	0	0	0	0.000	0.000	0.000	1-deoxy-D-xylulose 5-phosphate reductoisomerase
bin009	SOY3_bin009_00940	1062	1	0	1	0.113	0.000	0.100	Fe(3+)-citrate-binding protein YfmC precursor
bin009	SOY3_bin009_00941	2646	30	75	42	1.355	2.875	1.686	Pyruvate, phosphate dikinase
bin009	SOY3_bin009_00942	897	1	0	1	0.133	0.000	0.118	Putative pyruvate, phosphate dikinase regulatory protein
bin009	SOY3_bin009_00943	1014	0	0	1	0.000	0.000	0.105	Ribosomal RNA large subunit methyltransferase G
bin009	SOY3_bin009_00944	2136	0	0	0	0.000	0.000	0.000	RNA polymerase-associated protein RapA
bin009	SOY3_bin009_00945	1215	2	1	0	0.197	0.083	0.000	Endo-1,4-beta-xylanase Z precursor
bin009	SOY3_bin009_00946	573	1	1	0	0.209	0.177	0.000	hypothetical protein
bin009	SOY3_bin009_00947	165	0	0	0	0.000	0.000	0.000	Sodium/proline symporter
bin009	SOY3_bin009_00948	2817	2	4	0	0.085	0.144	0.000	CRISPR-associated nuclease/helicase Cas3
bin009	SOY3_bin009_00949	1602	0	1	1	0.000	0.063	0.066	CRISPR-associated protein CasA/Cse1
bin009	SOY3_bin009_00950	651	2	1	1	0.367	0.156	0.163	CRISPR-associated protein Cse2 (CRISPR_cse2)
bin009	SOY3_bin009_00951	1125	1	3	2	0.106	0.270	0.189	CRISPR system Cascade subunit CasC
bin009	SOY3_bin009_00952	702	1	2	0	0.170	0.289	0.000	CRISPR system Cascade subunit CasD
bin009	SOY3_bin009_00953	660	2	1	0	0.362	0.154	0.000	CRISPR-associated endoribonuclease Cse3
bin009	SOY3_bin009_00954	840	0	1	0	0.000	0.121	0.000	CRISPR-associated endonuclease Cas1
bin009	SOY3_bin009_00955	210	0	1	0	0.000	0.483	0.000	putative ssRNA endonuclease
bin009	SOY3_bin009_00956	1545	0	0	0	0.000	0.000	0.000	putative transcriptional regulator
bin009	SOY3_bin009_00957	750	0	0	0	0.000	0.000	0.000	Chromosomal replication initiator protein DnaA
bin009	SOY3_bin009_00958	1086	0	2	4	0.000	0.187	0.391	Mandelate racemase
bin009	SOY3_bin009_00959	963	1	0	0	0.124	0.000	0.000	putative DMT superfamily transporter inner membrane protein
bin009	SOY3_bin009_00960	2064	7	21	14	0.405	1.032	0.721	Transketolase
bin009	SOY3_bin009_00961	498	0	3	0	0.000	0.611	0.000	N-acyltransferase YncA
bin009	SOY3_bin009_00962	510	1	1	2	0.234	0.199	0.417	Xanthine phosphoribosyltransferase
bin009	SOY3_bin009_00963	1284	5	9	7	0.466	0.711	0.579	Citrate synthase 1
bin009	SOY3_bin009_00964	987	2	3	2	0.242	0.308	0.215	Diacylglycerol kinase
bin009	SOY3_bin009_00965	327	1	1	0	0.366	0.310	0.000	H(+)/Cl(-) exchange transporter ClcA
bin009	SOY3_bin009_00966	1695	0	2	0	0.000	0.120	0.000	hypothetical protein
bin009	SOY3_bin009_00967	933	0	0	0	0.000	0.000	0.000	Glutamate dehydrogenase
bin009	SOY3_bin009_00968	1158	0	0	0	0.000	0.000	0.000	Arginine--pyruvate transaminase AruH
bin009	SOY3_bin009_00969	621	0	0	0	0.000	0.000	0.000	4-hydroxy-4-methyl-2-oxoglutarate aldolase
bin009	SOY3_bin009_00970	861	0	0	0	0.000	0.000	0.000	Transketolase 2
bin009	SOY3_bin009_00971	981	0	0	0	0.000	0.000	0.000	1-deoxy-D-xylulose-5-phosphate synthase
bin009	SOY3_bin009_00972	1140	3	1	1	0.315	0.089	0.093	hypothetical protein
bin009	SOY3_bin009_00973	1329	1	0	1	0.090	0.000	0.080	Enterobactin exporter EntS
bin009	SOY3_bin009_00974	1398	2	4	1	0.171	0.290	0.076	Dihydrolipoyl dehydrogenase
bin009	SOY3_bin009_00975	987	0	0	0	0.000	0.000	0.000	Sodium/hydrogen exchanger family protein
bin009	SOY3_bin009_00976	891	0	0	0	0.000	0.000	0.000	2-dehydropantoate 2-reductase
bin009	SOY3_bin009_00977	963	3	6	3	0.372	0.632	0.331	Glycerate dehydrogenase
bin009	SOY3_bin009_00978	1095	2	1	3	0.218	0.093	0.291	putative HTH-type transcriptional regulator YbaQ
bin009	SOY3_bin009_00979	300	2	1	2	0.797	0.338	0.708	Toxin HigB-1
bin009	SOY3_bin009_00980	237	0	0	0	0.000	0.000	0.000	Glycerate dehydrogenase
bin009	SOY3_bin009_00981	981	0	1	0	0.000	0.103	0.000	HNH endonuclease
bin009	SOY3_bin009_00982	405	0	0	0	0.000	0.000	0.000	hypothetical protein
bin009	SOY3_bin009_00983	1314	1	0	0	0.091	0.000	0.000	hypothetical protein
bin009	SOY3_bin009_00984	603	0	1	0	0.000	0.168	0.000	hypothetical protein
bin009	SOY3_bin009_00985	963	2	1	2	0.248	0.105	0.221	hypothetical protein
bin009	SOY3_bin009_00986	606	1	2	1	0.197	0.335	0.175	hypothetical protein
bin009	SOY3_bin009_00987	2148	0	2	2	0.000	0.094	0.099	hypothetical protein
bin009	SOY3_bin009_00988	2499	0	1	2	0.000	0.041	0.085	PglZ domain protein
bin009	SOY3_bin009_00989	984	0	0	0	0.000	0.000	0.000	hypothetical protein
bin009	SOY3_bin009_00990	996	0	1	0	0.000	0.102	0.000	Putative prophage phiRv2 integrase
bin009	SOY3_bin009_00991	768	0	2	0	0.000	0.264	0.000	hypothetical protein
bin009	SOY3_bin009_00992	414	0	2	1	0.000	0.490	0.257	putative formaldehyde dehydrogenase AdhA
bin009	SOY3_bin009_00993	1320	4	3	1	0.362	0.231	0.080	L-fuconate dehydratase
bin009	SOY3_bin009_00994	1464	2	4	5	0.163	0.277	0.363	Glutamate synthase [NADPH] small chain
bin009	SOY3_bin009_00995	4524	6	22	13	0.159	0.493	0.305	Glutamate synthase [NADPH] large chain
bin009	SOY3_bin009_00996	879	1	2	1	0.136	0.231	0.121	Prolipoprotein diacylglyceryl transferase
bin009	SOY3_bin009_00997	858	1	0	1	0.139	0.000	0.124	Tryptophan synthase alpha chain
bin009	SOY3_bin009_00998	1245	0	0	1	0.000	0.000	0.085	Tryptophan synthase beta chain
bin009	SOY3_bin009_00999	801	1	1	0	0.149	0.127	0.000	Indole-3-glycerol phosphate synthase
bin009	SOY3_bin009_01000	1560	0	4	2	0.000	0.260	0.136	Anthranilate synthase component 1
bin009	SOY3_bin009_01001	396	0	1	1	0.000	0.256	0.268	phosphoribosyl-AMP cyclohydrolase

bin009	SOY3_bin009_01002	363	0	0	0	0.000	0.000	0.000	Transcriptional regulator YqjI
bin009	SOY3_bin009_01003	1374	0	2	1	0.000	0.148	0.077	THUMP domain protein
bin009	SOY3_bin009_01004	1065	0	0	0	0.000	0.000	0.000	hypothetical protein
bin009	SOY3_bin009_01005	1503	0	4	5	0.000	0.270	0.353	Aspartyl/glutamyl-tRNA(Asn/Gln) amidotransferase subunit B
bin009	SOY3_bin009_01006	1527	3	7	0	0.235	0.465	0.000	Glutamyl-tRNA(Gln) amidotransferase subunit A
bin009	SOY3_bin009_01007	297	2	4	3	0.805	1.366	1.073	Aspartyl/glutamyl-tRNA(Asn/Gln) amidotransferase subunit C
bin009	SOY3_bin009_01008	1416	0	3	2	0.000	0.215	0.150	Major myo-inositol transporter IolT
bin009	SOY3_bin009_01009	711	1	3	3	0.168	0.428	0.448	ACT domain protein
bin009	SOY3_bin009_01010	504	1	1	0	0.237	0.201	0.000	SNARE associated Golgi protein
bin009	SOY3_bin009_01011	240	1	1	3	0.498	0.423	1.328	hypothetical protein
bin009	SOY3_bin009_01012	996	0	2	0	0.000	0.204	0.000	methionine synthase
bin009	SOY3_bin009_01013	1092	0	1	2	0.000	0.093	0.195	tRNA-specific 2-thiouridylase MnmA
bin009	SOY3_bin009_01014	1170	0	0	1	0.000	0.000	0.091	Cysteine desulfurase
bin009	SOY3_bin009_01015	2130	3	8	5	0.168	0.381	0.249	Glycogen debranching enzyme
bin009	SOY3_bin009_01016	1455	0	7	4	0.000	0.488	0.292	Maltodextrin phosphorylase
bin009	SOY3_bin009_01017	252	3	1	0	1.423	0.402	0.000	Transcriptional regulator WhiB1
bin009	SOY3_bin009_01018	3729	12	27	8	0.385	0.734	0.228	Multifunctional 2-oxoglutarate metabolism enzyme
bin009	SOY3_bin009_01019	1314	0	2	0	0.000	0.154	0.000	hypothetical protein
bin009	SOY3_bin009_01020	1362	0	2	2	0.000	0.149	0.156	hypothetical protein
bin009	SOY3_bin009_01021	1125	0	1	2	0.000	0.090	0.189	pheromone autoinducer 2 transporter
bin009	SOY3_bin009_01022	73	0	0	0	0.000	0.000	0.000	tRNA-Arg(ccg)
bin009	SOY3_bin009_01023	1395	1	2	3	0.086	0.145	0.228	Diaminopimelate decarboxylase
bin009	SOY3_bin009_01024	1329	1	3	4	0.090	0.229	0.320	Homoserine dehydrogenase
bin009	SOY3_bin009_01025	618	2	2	0	0.387	0.328	0.000	phosphoserine phosphatase
bin009	SOY3_bin009_01026	1797	4	7	3	0.266	0.395	0.177	hypothetical protein
bin009	SOY3_bin009_01027	210	3	8	10	1.708	3.864	5.058	50S ribosomal protein L31
bin009	SOY3_bin009_01028	1086	0	2	1	0.000	0.187	0.098	Peptide chain release factor 1
bin009	SOY3_bin009_01029	861	0	1	1	0.000	0.118	0.123	Release factor glutamine methyltransferase
bin009	SOY3_bin009_01030	741	1	1	2	0.161	0.137	0.287	Threonylcarbamoyl-AMP synthase
bin009	SOY3_bin009_01031	1110	2	1	0	0.215	0.091	0.000	Decaprenyl-phosphate N-acetylglucosaminephosphotransferase
bin009	SOY3_bin009_01032	453	10	6	10	2.639	1.343	2.345	hypothetical protein
bin009	SOY3_bin009_01033	237	0	3	2	0.000	1.284	0.896	Putative F0F1-ATPase subunit (ATPase_gene1)
bin009	SOY3_bin009_01034	774	0	11	5	0.000	1.441	0.686	ATP synthase subunit a
bin009	SOY3_bin009_01035	222	1	9	2	0.539	4.112	0.957	ATP synthase subunit c
bin009	SOY3_bin009_01036	600	3	10	3	0.598	1.690	0.531	ATP synthase subunit b
bin009	SOY3_bin009_01037	777	4	7	1	0.615	0.914	0.137	ATP synthase subunit delta
bin009	SOY3_bin009_01038	1635	3	16	8	0.219	0.993	0.520	ATP synthase subunit alpha
bin009	SOY3_bin009_01039	927	1	3	6	0.129	0.328	0.688	ATP synthase gamma chain
bin009	SOY3_bin009_01040	1455	2	9	7	0.164	0.627	0.511	ATP synthase subunit beta
bin009	SOY3_bin009_01041	426	0	4	6	0.000	0.952	1.496	ATP synthase epsilon chain
bin009	SOY3_bin009_01042	465	1	3	4	0.257	0.654	0.914	hypothetical protein
bin009	SOY3_bin009_01043	1050	0	3	3	0.000	0.290	0.304	putative lipoate-protein ligase A
bin009	SOY3_bin009_01044	630	3	15	5	0.569	2.415	0.843	Cob(I)yrinic acid a,c-diamide adenosyltransferase
bin009	SOY3_bin009_01045	741	1	2	1	0.161	0.274	0.143	putative peptidyl-prolyl cis-trans isomerase
bin009	SOY3_bin009_01046	789	0	1	0	0.000	0.129	0.000	Esterase YbfF
bin009	SOY3_bin009_01047	276	1	0	0	0.433	0.000	0.000	hypothetical protein
bin009	SOY3_bin009_01048	630	2	3	1	0.380	0.483	0.169	Putative K(+)-stimulated pyrophosphate-energized sodium pump
bin009	SOY3_bin009_01049	840	0	5	3	0.000	0.604	0.379	putative oxidoreductase/MSMEI_2347
bin009	SOY3_bin009_01050	747	0	0	1	0.000	0.000	0.142	Acetylxylan esterase precursor
bin009	SOY3_bin009_01051	183	0	1	1	0.000	0.554	0.580	hypothetical protein
bin009	SOY3_bin009_01052	459	1	5	1	0.260	1.105	0.231	Putative reactive intermediate deaminase TdcF
bin009	SOY3_bin009_01053	666	1	1	1	0.180	0.152	0.159	Ultraviolet N-glycosylase/AP lyase
bin009	SOY3_bin009_01054	642	3	8	3	0.559	1.264	0.496	putative NUDIX hydrolase
bin009	SOY3_bin009_01055	510	6	5	4	1.406	0.994	0.833	hypothetical protein
bin009	SOY3_bin009_01056	978	2	1	0	0.244	0.104	0.000	putative inner membrane transporter YicL
bin009	SOY3_bin009_01057	618	4	13	2	0.774	2.134	0.344	hypothetical protein
bin009	SOY3_bin009_01058	204	0	0	1	0.000	0.000	0.521	hypothetical protein
bin009	SOY3_bin009_01059	141	0	0	0	0.000	0.000	0.000	hypothetical protein
bin009	SOY3_bin009_01060	777	1	1	1	0.154	0.131	0.137	hypothetical protein
bin009	SOY3_bin009_01061	924	0	0	0	0.000	0.000	0.000	Sugar phosphatase YidA
bin009	SOY3_bin009_01062	996	0	0	0	0.000	0.000	0.000	Septum site-determining protein MinD
bin009	SOY3_bin009_01063	1170	0	0	0	0.000	0.000	0.000	Putative conjugal transfer protein/MT3759
bin009	SOY3_bin009_01064	813	0	0	0	0.000	0.000	0.000	hypothetical protein
bin009	SOY3_bin009_01065	771	0	0	0	0.000	0.000	0.000	Bacterial type II secretion system protein F domain protein
bin009	SOY3_bin009_01066	228	0	0	1	0.000	0.000	0.466	hypothetical protein
bin009	SOY3_bin009_01067	381	0	0	0	0.000	0.000	0.000	hypothetical protein
bin009	SOY3_bin009_01068	339	0	0	0	0.000	0.000	0.000	hypothetical protein

bin009	SOY3_bin009_01069	1344	0	0	1	0.000	0.000	0.079	hypothetical protein
bin009	SOY3_bin009_01070	2268	2	7	1	0.105	0.313	0.047	ATP-dependent DNA helicase RecQ
bin009	SOY3_bin009_01071	1479	1	1	2	0.081	0.069	0.144	Ribosomal RNA small subunit methyltransferase C
bin009	SOY3_bin009_01072	2736	1	2	3	0.044	0.074	0.116	DNA topoisomerase 1
bin009	SOY3_bin009_01073	1473	1	7	4	0.081	0.482	0.288	hypothetical protein
bin009	SOY3_bin009_01074	663	0	2	2	0.000	0.306	0.320	Thymidylate kinase
bin009	SOY3_bin009_01075	1212	0	0	0	0.000	0.000	0.000	DNA polymerase III subunit tau
bin009	SOY3_bin009_01076	909	5	8	7	0.658	0.893	0.818	IgA-specific serine endopeptidase autotransporter precursor
bin009	SOY3_bin009_01077	831	1	4	0	0.144	0.488	0.000	1-acyl-sn-glycerol-3-phosphate acyltransferase
bin009	SOY3_bin009_01078	1152	1	2	0	0.104	0.176	0.000	Sensor histidine kinase LiaS
bin009	SOY3_bin009_01079	666	4	7	4	0.718	1.066	0.638	Transcriptional regulatory protein DegU
bin009	SOY3_bin009_01080	1347	4	6	0	0.355	0.452	0.000	Phospho-2-dehydro-3-deoxyheptonate aldolase
bin009	SOY3_bin009_01081	1044	0	0	0	0.000	0.000	0.000	L-allo-threonine aldolase
bin009	SOY3_bin009_01082	642	0	2	0	0.000	0.316	0.000	Putative Rieske 2Fe-2S iron-sulfur protein/MSMEI_6242
bin009	SOY3_bin009_01083	1020	3	2	0	0.352	0.199	0.000	putative DMT superfamily transporter inner membrane protein
bin009	SOY3_bin009_01084	2193	1	9	1	0.055	0.416	0.048	Serine/threonine-protein kinase PK-1
bin009	SOY3_bin009_01085	621	0	2	2	0.000	0.327	0.342	hypothetical protein
bin009	SOY3_bin009_01086	1092	1	2	1	0.109	0.186	0.097	Heptaprenyl diphosphate synthase component 2
bin009	SOY3_bin009_01087	900	0	3	1	0.000	0.338	0.118	5,10-methylenetetrahydrofolate reductase
bin009	SOY3_bin009_01088	2262	0	4	2	0.000	0.179	0.094	Helicase IV
bin009	SOY3_bin009_01089	948	0	1	0	0.000	0.107	0.000	hypothetical protein
bin009	SOY3_bin009_01090	963	0	1	1	0.000	0.105	0.110	hypothetical protein
bin009	SOY3_bin009_01091	1377	0	1	0	0.000	0.074	0.000	Adenylosuccinate synthetase
bin009	SOY3_bin009_01092	630	0	1	2	0.000	0.161	0.337	bifunctional nicotinamide mononucleotide adenylyltransferase/ADP-ribose pyrophosphatase
bin009	SOY3_bin009_01093	921	0	2	1	0.000	0.220	0.115	2-dehydropantoate 2-reductase
bin009	SOY3_bin009_01094	291	0	1	2	0.000	0.349	0.730	hypothetical protein
bin009	SOY3_bin009_01095	1311	1	1	1	0.091	0.077	0.081	Low-affinity inorganic phosphate transporter 1
bin009	SOY3_bin009_01096	747	2	1	2	0.320	0.136	0.284	Fumarate reductase iron-sulfur subunit
bin009	SOY3_bin009_01097	2049	3	3	3	0.175	0.149	0.156	Fumarate reductase flavoprotein subunit
bin009	SOY3_bin009_01098	774	4	2	3	0.618	0.262	0.412	Succinate dehydrogenase/Fumarate reductase transmembrane subunit
bin009	SOY3_bin009_01099	633	1	1	0	0.189	0.160	0.000	putative HTH-type transcriptional regulator TtgW
bin009	SOY3_bin009_01100	210	0	0	0	0.000	0.000	0.000	hypothetical protein
bin009	SOY3_bin009_01101	894	0	1	0	0.000	0.113	0.000	hypothetical protein
bin009	SOY3_bin009_01102	83	0	0	0	0.000	0.000	0.000	tRNA-Leu(tag)
bin009	SOY3_bin009_01103	525	1	1	1	0.228	0.193	0.202	Putative peroxiredoxin/MT2597
bin009	SOY3_bin009_01104	327	6	12	12	2.194	3.722	3.898	hypothetical protein
bin009	SOY3_bin009_01105	399	3	5	1	0.899	1.271	0.266	10 kDa chaperonin
bin009	SOY3_bin009_01106	76	0	0	0	0.000	0.000	0.000	tRNA-Lys(ctt)
bin009	SOY3_bin009_01107	1020	1	5	2	0.117	0.497	0.208	Fructosamine deglycase FrlB
bin009	SOY3_bin009_01108	828	0	4	3	0.000	0.490	0.385	Fructosamine kinase FrlD
bin009	SOY3_bin009_01109	2064	1	7	2	0.058	0.344	0.103	Acetophenone carboxylase gamma subunit
bin009	SOY3_bin009_01110	1644	1	6	2	0.073	0.370	0.129	Acetophenone carboxylase delta subunit
bin009	SOY3_bin009_01111	1599	4	14	9	0.299	0.888	0.598	Glutathione-binding protein GsiB precursor
bin009	SOY3_bin009_01112	954	0	3	6	0.000	0.319	0.668	Dipeptide transport system permease protein DppB
bin009	SOY3_bin009_01113	915	0	1	1	0.000	0.111	0.116	putative D,D-dipeptide transport system permease protein DdpC
bin009	SOY3_bin009_01114	1704	1	2	3	0.070	0.119	0.187	Glutathione import ATP-binding protein GsiA
bin009	SOY3_bin009_01115	1602	2	3	2	0.149	0.190	0.133	Gamma-glutamyltranspeptidase precursor
bin009	SOY3_bin009_01116	1059	1	0	1	0.113	0.000	0.100	Catabolite control protein A
bin009	SOY3_bin009_01117	546	0	0	1	0.000	0.000	0.195	hypothetical protein
bin009	SOY3_bin009_01118	732	1	2	1	0.163	0.277	0.145	Crotonobetainyl-CoA dehydrogenase
bin009	SOY3_bin009_01119	924	0	0	1	0.000	0.000	0.115	IS2 transposase TnpB
bin009	SOY3_bin009_01120	291	0	1	0	0.000	0.349	0.000	Transposase
bin009	SOY3_bin009_01121	438	0	0	1	0.000	0.000	0.243	oxidoreductase
bin009	SOY3_bin009_01122	291	0	0	0	0.000	0.000	0.000	NADH-plastoquinone oxidoreductase subunit
bin009	SOY3_bin009_01123	303	2	2	5	0.789	0.669	1.753	hypothetical protein
bin009	SOY3_bin009_01124	870	1	8	0	0.137	0.933	0.000	Exodeoxyribonuclease III
bin009	SOY3_bin009_01125	1380	0	1	1	0.000	0.073	0.077	hypothetical protein
bin009	SOY3_bin009_01126	750	2	0	3	0.319	0.000	0.425	Cobyric acid synthase
bin009	SOY3_bin009_01127	1287	2	2	3	0.186	0.158	0.248	UDP-N-acetylmuramoyl-L-alanyl-D-glutamate synthetase
bin009	SOY3_bin009_01128	1566	0	0	2	0.000	0.000	0.136	Aspartate/alanine antiporter
bin009	SOY3_bin009_01129	138	0	0	0	0.000	0.000	0.000	hypothetical protein
bin009	SOY3_bin009_01130	450	3	2	3	0.797	0.451	0.708	HIT-like protein
bin009	SOY3_bin009_01131	1551	0	4	1	0.000	0.262	0.068	Aminopeptidase S
bin009	SOY3_bin009_01132	126	0	0	0	0.000	0.000	0.000	hypothetical protein
bin009	SOY3_bin009_01133	1827	1	5	2	0.065	0.278	0.116	hypothetical protein
bin009	SOY3_bin009_01134	1338	2	0	0	0.179	0.000	0.000	Sensor histidine kinase ComP
bin009	SOY3_bin009_01135	660	0	1	1	0.000	0.154	0.161	Oxygen regulatory protein NreC

bin009	SOY3_bin009_01136	756	2	1	0	0.316	0.134	0.000	2-dehydro-3-deoxy-D-gluconate 5-dehydrogenase
bin009	SOY3_bin009_01137	894	2	2	2	0.267	0.227	0.238	2-hydroxy-3-oxopropionate reductase
bin009	SOY3_bin009_01138	738	1	1	2	0.162	0.137	0.288	HTH-type transcriptional repressor AllR
bin009	SOY3_bin009_01139	1023	6	14	9	0.701	1.388	0.935	L-idonate 5-dehydrogenase (NAD(P)(+))
bin009	SOY3_bin009_01140	1314	3	15	16	0.273	1.158	1.293	Sialic acid TRAP transporter permease protein SiaT
bin009	SOY3_bin009_01141	531	6	7	4	1.351	1.337	0.800	Sialic acid TRAP transporter permease protein SiaT
bin009	SOY3_bin009_01142	1038	29	80	60	3.340	7.817	6.140	Sialic acid-binding periplasmic protein SiaP precursor
bin009	SOY3_bin009_01143	1044	4	5	2	0.458	0.486	0.203	2,3-diketo-L-gulonate-binding periplasmic protein YiaO precursor
bin009	SOY3_bin009_01144	450	5	5	2	1.328	1.127	0.472	hypothetical protein
bin009	SOY3_bin009_01145	1320	0	2	0	0.000	0.154	0.000	2-acyl-glycerophospho-ethanolamine acyltransferase
bin009	SOY3_bin009_01146	2637	0	0	0	0.000	0.000	0.000	LysM domain/BON superfamily protein
bin009	SOY3_bin009_01147	447	0	0	0	0.000	0.000	0.000	TadE-like protein
bin009	SOY3_bin009_01148	336	0	0	0	0.000	0.000	0.000	TadE-like protein
bin009	SOY3_bin009_01149	228	0	0	0	0.000	0.000	0.000	hypothetical protein
bin009	SOY3_bin009_01150	912	0	0	0	0.000	0.000	0.000	hypothetical protein
bin009	SOY3_bin009_01151	873	0	1	0	0.000	0.116	0.000	Bacterial type II secretion system protein F domain protein
bin009	SOY3_bin009_01152	1455	0	1	0	0.000	0.070	0.000	Putative conjugal transfer protein/MT3759
bin009	SOY3_bin009_01153	795	1	0	0	0.150	0.000	0.000	hypothetical protein
bin009	SOY3_bin009_01154	657	1	2	0	0.182	0.309	0.000	flagellar basal body P-ring biosynthesis protein FlgA
bin009	SOY3_bin009_01155	663	0	0	1	0.000	0.000	0.160	zinc transporter ZitB
bin009	SOY3_bin009_01156	372	19	30	17	6.106	8.180	4.854	hypothetical protein
bin009	SOY3_bin009_01157	1134	11	14	21	1.160	1.252	1.967	Glycerate kinase
bin009	SOY3_bin009_01158	2160	2	4	3	0.111	0.188	0.148	Protein-glutamine gamma-glutamyltransferase
bin009	SOY3_bin009_01159	1242	0	0	0	0.000	0.000	0.000	hypothetical protein
bin009	SOY3_bin009_01160	1065	1	1	0	0.112	0.095	0.000	ATPase RavA
bin009	SOY3_bin009_01161	450	6	10	7	1.594	2.254	1.652	cell division protein MraZ
bin009	SOY3_bin009_01162	1044	1	3	1	0.115	0.291	0.102	Ribosomal RNA small subunit methyltransferase H
bin009	SOY3_bin009_01163	612	0	3	1	0.000	0.497	0.174	Cell division protein FtsL
bin009	SOY3_bin009_01164	1929	0	2	6	0.000	0.105	0.330	Penicillin-binding protein PbpB
bin009	SOY3_bin009_01165	1593	1	4	1	0.075	0.255	0.067	UDP-N-acetylmuramoyl-L-alanyl-D-glutamate--2,6-diaminopimelate ligase
bin009	SOY3_bin009_01166	1443	0	0	1	0.000	0.000	0.074	UDP-N-acetylmuramoyl-tripeptide--D-alanyl-D-alanine ligase
bin009	SOY3_bin009_01167	1071	0	0	2	0.000	0.000	0.198	Phospho-N-acetylmuramoyl-pentapeptide-transferase
bin009	SOY3_bin009_01168	1500	0	2	1	0.000	0.135	0.071	UDP-N-acetylmuramoylalanine--D-glutamate ligase
bin009	SOY3_bin009_01169	1317	3	4	0	0.272	0.308	0.000	Lipid II flippase FtsW
bin009	SOY3_bin009_01170	1113	1	1	0	0.107	0.091	0.000	UDP-N-acetylglucosamine--N-acetylmuramyl-(pentapeptide) pyrophosphoryl-undecaprenol N-acetylglucosamine transferase
bin009	SOY3_bin009_01171	1452	1	4	0	0.082	0.279	0.000	UDP-N-acetylmuramate--L-alanine ligase
bin009	SOY3_bin009_01172	729	1	2	2	0.164	0.278	0.291	Cell division protein FtsQ
bin009	SOY3_bin009_01173	1176	6	12	10	0.610	1.035	0.903	Cell division protein FtsZ
bin009	SOY3_bin009_01174	771	2	7	1	0.310	0.921	0.138	Laccase domain protein YfiH
bin009	SOY3_bin009_01175	504	9	6	13	2.135	1.207	2.740	Cell division protein SepF
bin009	SOY3_bin009_01176	291	2	1	2	0.822	0.349	0.730	YGGT family protein
bin009	SOY3_bin009_01177	621	10	26	11	1.925	4.247	1.882	hypothetical protein
bin009	SOY3_bin009_01178	258	1	0	0	0.463	0.000	0.000	hypothetical protein
bin009	SOY3_bin009_01179	366	0	0	0	0.000	0.000	0.000	hypothetical protein
bin009	SOY3_bin009_01180	333	0	1	0	0.000	0.305	0.000	hypothetical protein
bin009	SOY3_bin009_01181	972	0	0	0	0.000	0.000	0.000	Integrase core domain protein
bin009	SOY3_bin009_01182	312	0	0	0	0.000	0.000	0.000	Transposase
bin009	SOY3_bin009_01183	594	2	0	1	0.403	0.000	0.179	HTH-type transcriptional repressor KstR2
bin009	SOY3_bin009_01184	747	9	7	6	1.440	0.950	0.853	Global nitrogen regulator
bin009	SOY3_bin009_01185	237	1	0	0	0.504	0.000	0.000	Copper chaperone CopZ
bin009	SOY3_bin009_01186	1932	1	0	0	0.062	0.000	0.000	putative cadmium-transporting ATPase
bin009	SOY3_bin009_01187	318	0	0	0	0.000	0.000	0.000	hypothetical protein
bin009	SOY3_bin009_01188	894	0	0	0	0.000	0.000	0.000	hypothetical protein
bin009	SOY3_bin009_01189	336	2	4	2	0.712	1.207	0.632	hypothetical protein
bin009	SOY3_bin009_01190	996	2	2	2	0.240	0.204	0.213	Patatin-like phospholipase
bin009	SOY3_bin009_01191	1458	10	34	16	0.820	2.365	1.166	Catalase
bin009	SOY3_bin009_01192	1119	0	0	0	0.000	0.000	0.000	Purine catabolism regulatory protein
bin009	SOY3_bin009_01193	1395	0	0	0	0.000	0.000	0.000	5-aminovalerate aminotransferase DavT
bin009	SOY3_bin009_01194	603	0	1	0	0.000	0.168	0.000	cytidylate kinase
bin009	SOY3_bin009_01195	729	0	0	1	0.000	0.000	0.146	Glutamine transport ATP-binding protein GlnQ
bin009	SOY3_bin009_01196	699	0	0	0	0.000	0.000	0.000	Glutamine transport system permease protein GlnP
bin009	SOY3_bin009_01197	891	0	1	0	0.000	0.114	0.000	Cystine-binding periplasmic protein precursor
bin009	SOY3_bin009_01198	1170	2	1	0	0.204	0.087	0.000	hypothetical protein
bin009	SOY3_bin009_01199	522	0	1	1	0.000	0.194	0.203	Outer membrane lipoprotein Blc precursor
bin009	SOY3_bin009_01200	609	1	1	0	0.196	0.167	0.000	1-acyl-sn-glycerol-3-phosphate acyltransferase
bin009	SOY3_bin009_01201	3186	0	4	1	0.000	0.127	0.033	Macrolide export ATP-binding/permease protein MacB
bin009	SOY3_bin009_01202	798	0	1	0	0.000	0.127	0.000	hypothetical protein

bin009	SOY3_bin009_01203	156	0	2	0	0.000	1.300	0.000	hypothetical protein
bin009	SOY3_bin009_01204	276	1	4	7	0.433	1.470	2.694	hypothetical protein
bin009	SOY3_bin009_01205	459	1	1	2	0.260	0.221	0.463	Deoxyuridine 5'-triphosphate nucleotidohydrolase
bin009	SOY3_bin009_01206	816	0	4	3	0.000	0.497	0.391	hypothetical protein
bin009	SOY3_bin009_01207	1143	0	1	3	0.000	0.089	0.279	putative RNA methyltransferase/cg2084
bin009	SOY3_bin009_01208	264	5	16	6	2.264	6.147	2.414	hypothetical protein
bin009	SOY3_bin009_01209	1905	14	40	11	0.879	2.130	0.613	Phosphoenolpyruvate carboxykinase [GTP]
bin009	SOY3_bin009_01210	1467	8	23	14	0.652	1.590	1.014	Succinate-semialdehyde dehydrogenase [NADP(+)] GabD
bin009	SOY3_bin009_01211	750	7	6	2	1.116	0.811	0.283	3-oxoacyl-[acyl-carrier-protein] reductase FabG
bin009	SOY3_bin009_01212	2211	1	1	4	0.054	0.046	0.192	Pyruvate dehydrogenase E1 component subunit beta
bin009	SOY3_bin009_01213	1281	0	1	2	0.000	0.079	0.166	Dihydrolipoyllysine-residue acetyltransferase component of pyruvate dehydrogenase complex
bin009	SOY3_bin009_01214	1002	0	0	1	0.000	0.000	0.106	D-arabitol-phosphate dehydrogenase
bin009	SOY3_bin009_01215	525	0	1	1	0.000	0.193	0.202	putative lyase
bin009	SOY3_bin009_01216	684	0	0	0	0.000	0.000	0.000	HTH-type transcriptional regulator McbR
bin009	SOY3_bin009_01217	846	1	0	0	0.141	0.000	0.000	DNA-binding transcriptional repressor AcrR
bin009	SOY3_bin009_01218	477	0	0	0	0.000	0.000	0.000	flavodoxin
bin009	SOY3_bin009_01219	768	1	2	0	0.156	0.264	0.000	2-dehydro-3-deoxy-D-gluconate 5-dehydrogenase
bin009	SOY3_bin009_01220	612	0	0	0	0.000	0.000	0.000	Carminomycin 4-O-methyltransferase
bin009	SOY3_bin009_01221	1392	3	0	2	0.258	0.000	0.153	Vibriobactin utilization protein ViuB
bin009	SOY3_bin009_01222	285	6	5	3	2.517	1.779	1.118	hypothetical protein
bin009	SOY3_bin009_01223	2670	13	21	15	0.582	0.798	0.597	Aconitate hydratase
bin009	SOY3_bin009_01224	297	0	0	0	0.000	0.000	0.000	hypothetical protein
bin009	SOY3_bin009_01225	417	0	0	1	0.000	0.000	0.255	HIT-like protein
bin009	SOY3_bin009_01226	369	1	0	1	0.324	0.000	0.288	hypothetical protein
bin009	SOY3_bin009_01227	594	0	0	1	0.000	0.000	0.179	hypothetical protein
bin009	SOY3_bin009_01228	78	1	1	0	1.533	1.300	0.000	tRNA-Ala(cgc)
bin009	SOY3_bin009_01229	297	0	0	0	0.000	0.000	0.000	hypothetical protein
bin009	SOY3_bin009_01230	717	0	1	0	0.000	0.141	0.000	MYND finger
bin009	SOY3_bin009_01231	789	0	1	0	0.000	0.129	0.000	CAAX amino terminal protease self- immunity
bin009	SOY3_bin009_01232	612	0	0	0	0.000	0.000	0.000	HTH-type transcriptional repressor KstR2
bin009	SOY3_bin009_01233	750	0	1	0	0.000	0.135	0.000	hypothetical protein
bin009	SOY3_bin009_01234	183	0	0	0	0.000	0.000	0.000	hypothetical protein
bin009	SOY3_bin009_01235	759	0	0	1	0.000	0.000	0.140	DNA-invertase hin
bin009	SOY3_bin009_01236	903	1	3	0	0.132	0.337	0.000	hypothetical protein
bin009	SOY3_bin009_01237	567	2	4	1	0.422	0.716	0.187	hypothetical protein
bin009	SOY3_bin009_01238	1059	0	2	0	0.000	0.192	0.000	hypothetical protein
bin009	SOY3_bin009_01239	4470	4	5	1	0.107	0.113	0.024	Chromosome partition protein Smc
bin009	SOY3_bin009_01240	813	1	4	0	0.147	0.499	0.000	hypothetical protein
bin009	SOY3_bin009_01241	1641	1	1	0	0.073	0.062	0.000	hypothetical protein
bin009	SOY3_bin009_01242	153	0	0	0	0.000	0.000	0.000	hypothetical protein
bin009	SOY3_bin009_01243	390	1	0	0	0.307	0.000	0.000	Glyoxalase-like domain protein
bin009	SOY3_bin009_01244	756	2	2	1	0.316	0.268	0.141	Transglutaminase-like superfamily protein
bin009	SOY3_bin009_01245	1245	0	0	0	0.000	0.000	0.000	Inner membrane transport protein YeaN
bin009	SOY3_bin009_01246	645	0	1	0	0.000	0.157	0.000	Maleamate amidohydrolase
bin009	SOY3_bin009_01247	606	0	1	0	0.000	0.167	0.000	Putative HMP/thiamine permease protein YkoE
bin009	SOY3_bin009_01248	846	0	1	1	0.000	0.120	0.126	hypothetical protein
bin009	SOY3_bin009_01249	1911	2	4	3	0.125	0.212	0.167	Glutamate-ammonia-ligase adenylyltransferase
bin009	SOY3_bin009_01250	729	0	1	0	0.000	0.139	0.000	Acetoacetyl-CoA reductase
bin009	SOY3_bin009_01251	843	0	0	1	0.000	0.000	0.126	Cobalamin synthase
bin009	SOY3_bin009_01252	546	0	0	1	0.000	0.000	0.195	Bifunctional adenosylcobalamin biosynthesis protein CobU
bin009	SOY3_bin009_01253	1080	1	2	0	0.111	0.188	0.000	Nicotinate-nucleotide--dimethylbenzimidazole phosphoribosyltransferase
bin009	SOY3_bin009_01254	1083	0	0	0	0.000	0.000	0.000	Undecaprenyl-phosphate mannosyltransferase
bin009	SOY3_bin009_01255	906	0	0	2	0.000	0.000	0.234	hypothetical protein
bin009	SOY3_bin009_01256	1590	0	0	0	0.000	0.000	0.000	hypothetical protein
bin009	SOY3_bin009_01257	750	0	0	0	0.000	0.000	0.000	VTC domain protein
bin009	SOY3_bin009_01258	645	0	1	0	0.000	0.157	0.000	hypothetical protein
bin009	SOY3_bin009_01259	621	0	0	1	0.000	0.000	0.171	Glucosyl-3-phosphoglycerate phosphatase
bin009	SOY3_bin009_01260	1461	0	0	1	0.000	0.000	0.073	putative metabolite transport protein CsbC
bin009	SOY3_bin009_01261	687	0	0	1	0.000	0.000	0.155	HTH-type transcriptional regulator LutR
bin009	SOY3_bin009_01262	1215	1	0	0	0.098	0.000	0.000	Starvation-sensing protein RspA
bin009	SOY3_bin009_01263	1422	13	25	13	1.093	1.783	0.971	Glutamine synthetase
bin009	SOY3_bin009_01264	756	1	1	1	0.158	0.134	0.141	HTH-type transcriptional repressor YvoA
bin009	SOY3_bin009_01265	984	3	3	0	0.364	0.309	0.000	putative fructose-bisphosphate aldolase
bin009	SOY3_bin009_01266	786	3	0	0	0.456	0.000	0.000	Phosphorylated carbohydrates phosphatase
bin009	SOY3_bin009_01267	873	1	1	0	0.137	0.116	0.000	Inosose isomerase
bin009	SOY3_bin009_01268	771	0	0	0	0.000	0.000	0.000	3-oxoacyl-[acyl-carrier-protein] reductase FabG
bin009	SOY3_bin009_01269	1116	1	5	3	0.107	0.454	0.286	HTH-type transcriptional repressor CytR

bin009	SOY3_bin009_01270	1314	0	0	1	0.000	0.000	0.081	Sialic acid TRAP transporter permease protein SiaT
bin009	SOY3_bin009_01271	279	0	0	0	0.000	0.000	0.000	Tripartite ATP-independent periplasmic transporters, DctQ component
bin009	SOY3_bin009_01272	672	0	0	0	0.000	0.000	0.000	Haemolysin-III related
bin009	SOY3_bin009_01273	819	1	0	0	0.146	0.000	0.000	(2Z,6E)-farnesyl diphosphate synthase
bin009	SOY3_bin009_01274	1611	1	6	4	0.074	0.378	0.264	hypothetical protein
bin009	SOY3_bin009_01275	294	0	1	0	0.000	0.345	0.000	hypothetical protein
bin009	SOY3_bin009_01276	534	4	2	2	0.895	0.380	0.398	Acetyltransferase (GNAT) family protein
bin009	SOY3_bin009_01277	939	1	2	0	0.127	0.216	0.000	Mycothiol S-conjugate amidase
bin009	SOY3_bin009_01278	504	0	9	6	0.000	1.811	1.265	Transcription elongation factor GreA
bin009	SOY3_bin009_01279	849	1	4	3	0.141	0.478	0.375	D-alanine aminotransferase
bin009	SOY3_bin009_01280	717	0	2	0	0.000	0.283	0.000	Glutamine transport ATP-binding protein GlnQ
bin009	SOY3_bin009_01281	855	7	15	7	0.979	1.779	0.870	ABC transporter glutamine-binding protein GlnH precursor
bin009	SOY3_bin009_01282	648	3	2	0	0.553	0.313	0.000	putative glutamine ABC transporter permease protein GlnM
bin009	SOY3_bin009_01283	915	4	1	2	0.523	0.111	0.232	putative glutamine ABC transporter permease protein GlnP
bin009	SOY3_bin009_01284	1191	5	5	8	0.502	0.426	0.714	hypothetical protein
bin009	SOY3_bin009_01285	1209	0	4	3	0.000	0.336	0.264	pheromone autoinducer 2 transporter
bin009	SOY3_bin009_01286	1152	0	0	0	0.000	0.000	0.000	Multidrug efflux protein YfmO
bin009	SOY3_bin009_01287	696	2	3	1	0.344	0.437	0.153	Uracil-DNA glycosylase
bin009	SOY3_bin009_01288	909	2	1	3	0.263	0.112	0.351	NTE family protein RssA
bin009	SOY3_bin009_01289	636	1	2	2	0.188	0.319	0.334	Peptide methionine sulfoxide reductase MsrA
bin009	SOY3_bin009_01290	1323	5	13	11	0.452	0.997	0.883	Resuscitation-promoting factor Rpf2 precursor
bin009	SOY3_bin009_01291	696	1	1	0	0.172	0.146	0.000	60 kDa chaperonin
bin009	SOY3_bin009_01292	1506	3	12	5	0.238	0.808	0.353	putative endopeptidase p60 precursor
bin009	SOY3_bin009_01293	381	1	1	2	0.314	0.266	0.558	Arsenical resistance operon repressor
bin009	SOY3_bin009_01294	786	1	2	1	0.152	0.258	0.135	Malonyl-[acyl-carrier protein] O-methyltransferase
bin009	SOY3_bin009_01295	408	1	1	0	0.293	0.249	0.000	Arsenate-mycothiol transferase ArsC1
bin009	SOY3_bin009_01296	672	3	2	3	0.534	0.302	0.474	hypothetical protein
bin009	SOY3_bin009_01297	74	0	0	0	0.000	0.000	0.000	tRNA-Glu(ttc)
bin009	SOY3_bin009_01298	165	0	0	0	0.000	0.000	0.000	hypothetical protein
bin009	SOY3_bin009_01299	1017	5	7	6	0.588	0.698	0.627	putative GTPase/MT1543
bin009	SOY3_bin009_01300	2193	23	79	31	1.254	3.654	1.502	Methylmalonyl-CoA mutase large subunit
bin009	SOY3_bin009_01301	1905	19	52	33	1.192	2.769	1.840	Methylmalonyl-CoA mutase small subunit
bin009	SOY3_bin009_01302	936	4	14	12	0.511	1.517	1.362	2-(hydroxymethyl)glutarate dehydrogenase
bin009	SOY3_bin009_01303	909	1	1	0	0.132	0.112	0.000	Patatin-like phospholipase
bin009	SOY3_bin009_01304	1821	1	2	0	0.066	0.111	0.000	Beta-glucuronidase
bin009	SOY3_bin009_01305	720	0	2	2	0.000	0.282	0.295	putative HTH-type transcriptional regulator YdfH
bin009	SOY3_bin009_01306	1032	0	1	0	0.000	0.098	0.000	Sialic acid-binding periplasmic protein SiaP precursor
bin009	SOY3_bin009_01307	1074	0	0	0	0.000	0.000	0.000	Mannonate dehydratase
bin009	SOY3_bin009_01308	1044	0	2	2	0.000	0.194	0.203	Aspartate carbamoyltransferase
bin009	SOY3_bin009_01309	837	1	0	1	0.143	0.000	0.127	putative hydrolase YutF
bin009	SOY3_bin009_01310	1347	1	1	0	0.089	0.075	0.000	Glycerol-1-phosphate dehydrogenase [NAD(P)+]
bin009	SOY3_bin009_01311	237	0	0	0	0.000	0.000	0.000	Phosphocarrier protein HPr
bin009	SOY3_bin009_01312	369	0	0	0	0.000	0.000	0.000	PTS system glucitol/sorbitol-specific transporter subunit IIA
bin009	SOY3_bin009_01313	1065	0	1	0	0.000	0.095	0.000	Glucitol/sorbitol-specific phosphotransferase enzyme IIB component
bin009	SOY3_bin009_01314	552	0	0	1	0.000	0.000	0.192	Glucitol/sorbitol permease IIC component
bin009	SOY3_bin009_01315	459	0	0	0	0.000	0.000	0.000	DNA-binding transcriptional activator GutM
bin009	SOY3_bin009_01316	1899	1	0	0	0.063	0.000	0.000	putative licABCH operon regulator
bin009	SOY3_bin009_01317	786	2	0	1	0.304	0.000	0.135	DNA repair protein RecN
bin009	SOY3_bin009_01318	1578	3	1	0	0.227	0.064	0.000	D-serine/D-alanine/glycine transporter
bin009	SOY3_bin009_01319	417	0	0	0	0.000	0.000	0.000	hypothetical protein
bin009	SOY3_bin009_01320	1356	0	1	0	0.000	0.075	0.000	hypothetical protein
bin009	SOY3_bin009_01321	1161	0	3	1	0.000	0.262	0.091	putative hemoglobin and hemoglobin-haptoglobin-binding protein 3 precursor
bin009	SOY3_bin009_01322	516	0	2	3	0.000	0.393	0.618	hypothetical protein
bin009	SOY3_bin009_01323	315	0	3	0	0.000	0.966	0.000	hypothetical protein
bin009	SOY3_bin009_01324	1641	2	4	2	0.146	0.247	0.129	AAA-like domain protein
bin009	SOY3_bin009_01325	462	0	1	0	0.000	0.220	0.000	hypothetical protein
bin009	SOY3_bin009_01326	1341	1	3	0	0.089	0.227	0.000	NADP-specific glutamate dehydrogenase
bin009	SOY3_bin009_01327	1503	2	3	2	0.159	0.202	0.141	Serine/threonine-protein kinase PknB
bin009	SOY3_bin009_01328	600	1	3	3	0.199	0.507	0.531	Fatty acid desaturase
bin009	SOY3_bin009_01329	1971	0	1	2	0.000	0.051	0.108	putative ABC transporter ATP-binding protein
bin009	SOY3_bin009_01330	1734	0	0	0	0.000	0.000	0.000	putative ABC transporter ATP-binding protein
bin009	SOY3_bin009_01331	594	0	0	0	0.000	0.000	0.000	HTH-type transcriptional repressor KstR2
bin009	SOY3_bin009_01332	900	3	14	2	0.398	1.578	0.236	hypothetical protein
bin009	SOY3_bin009_01333	354	0	1	1	0.000	0.287	0.300	hypothetical protein
bin009	SOY3_bin009_01334	1530	1	1	1	0.078	0.066	0.069	Lysylphosphatidylglycerol biosynthesis bifunctional protein LysX
bin009	SOY3_bin009_01335	1338	0	2	2	0.000	0.152	0.159	hypothetical protein
bin009	SOY3_bin009_01336	1485	3	4	1	0.242	0.273	0.072	Inosine-5'-monophosphate dehydrogenase

bin009	SOY3_bin009_01337	1461	0	2	0	0.000	0.139	0.000	hypothetical protein
bin009	SOY3_bin009_01338	1599	2	26	20	0.150	1.649	1.329	60 kDa chaperonin 1
bin009	SOY3_bin009_01339	297	0	12	13	0.000	4.098	4.650	10 kDa chaperonin
bin009	SOY3_bin009_01340	1152	0	1	0	0.000	0.088	0.000	23S rRNA m(5)U1939 methyltransferase
bin009	SOY3_bin009_01341	489	0	1	0	0.000	0.207	0.000	8-oxo-dGTP diphosphatase
bin009	SOY3_bin009_01342	1044	0	1	1	0.000	0.097	0.102	tRNA N6-adenosine threonylcarbamoyltransferase
bin009	SOY3_bin009_01343	477	2	1	1	0.501	0.213	0.223	ribosomal-protein-alanine N-acetyltransferase
bin009	SOY3_bin009_01344	702	0	1	2	0.000	0.144	0.303	tRNA threonylcarbamoyladenosine biosynthesis protein TsaB
bin009	SOY3_bin009_01345	1065	0	0	2	0.000	0.000	0.199	tRNA threonylcarbamoyladenosine biosynthesis protein TsaE
bin009	SOY3_bin009_01346	1029	2	6	2	0.232	0.591	0.206	GDP-mannose-dependent alpha-mannosyltransferase
bin009	SOY3_bin009_01347	1452	6	16	13	0.494	1.118	0.951	Bifunctional NAD(P)H-hydrate repair enzyme Nnr
bin009	SOY3_bin009_01348	369	1	2	4	0.324	0.550	1.151	Holo-[acyl-carrier-protein] synthase
bin009	SOY3_bin009_01349	1887	10	22	16	0.634	1.183	0.901	Glutamine--fructose-6-phosphate aminotransferase [isomerizing]
bin009	SOY3_bin009_01350	924	0	1	1	0.000	0.110	0.115	Pantothenate kinase
bin009	SOY3_bin009_01351	846	1	1	2	0.141	0.120	0.251	Ion transport protein
bin009	SOY3_bin009_01352	1356	1	5	3	0.088	0.374	0.235	Phosphoglucosamine mutase
bin009	SOY3_bin009_01353	552	3	13	8	0.650	2.389	1.540	30S ribosomal protein S9
bin009	SOY3_bin009_01354	396	7	9	7	2.113	2.305	1.878	50S ribosomal protein L13
bin009	SOY3_bin009_01355	2037	1	5	2	0.059	0.249	0.104	O-acetyltransferase OatA
bin009	SOY3_bin009_01356	1488	0	1	2	0.000	0.068	0.143	hypothetical protein
bin009	SOY3_bin009_01357	1581	3	4	2	0.227	0.257	0.134	HTH-type transcriptional regulator MalT
bin009	SOY3_bin009_01358	456	0	0	0	0.000	0.000	0.000	Diadenosine hexaphosphate hydrolase
bin009	SOY3_bin009_01359	1527	2	1	0	0.157	0.066	0.000	Cyclomaltodextrinase
bin009	SOY3_bin009_01360	408	0	1	1	0.000	0.249	0.260	hypothetical protein
bin009	SOY3_bin009_01361	429	1	1	0	0.279	0.236	0.000	hypothetical protein
bin009	SOY3_bin009_01362	735	0	0	0	0.000	0.000	0.000	Sulfite exporter TauE/SafE
bin009	SOY3_bin009_01363	1380	1	2	1	0.087	0.147	0.077	Major Facilitator Superfamily protein
bin009	SOY3_bin009_01364	780	0	0	1	0.000	0.000	0.136	4-hydroxy-2-oxo-heptane-1,7-dioate aldolase
bin009	SOY3_bin009_01365	1821	1	4	2	0.066	0.223	0.117	Voltage-gated potassium channel Kch
bin009	SOY3_bin009_01366	384	3	3	4	0.934	0.792	1.107	hypothetical protein
bin009	SOY3_bin009_01367	654	2	1	0	0.366	0.155	0.000	mycothiol-dependent maleylpyruvate isomerase
bin009	SOY3_bin009_01368	3402	12	27	15	0.422	0.805	0.468	hypothetical protein
bin009	SOY3_bin009_01369	1368	1	5	2	0.087	0.371	0.155	Putative serine protease HtrA
bin009	SOY3_bin009_01370	1737	5	14	7	0.344	0.817	0.428	hypothetical protein
bin009	SOY3_bin009_01371	1182	11	17	5	1.113	1.459	0.449	Putative neutral zinc metallopeptidase
bin009	SOY3_bin009_01372	471	1	2	0	0.254	0.431	0.000	2-C-methyl-D-erythritol 2,4-cyclodiphosphate synthase
bin009	SOY3_bin009_01373	747	0	2	0	0.000	0.272	0.000	2-C-methyl-D-erythritol 4-phosphate cytidylyltransferase
bin009	SOY3_bin009_01374	495	0	0	0	0.000	0.000	0.000	hypothetical protein
bin009	SOY3_bin009_01375	459	1	1	0	0.260	0.221	0.000	Endoribonuclease YbeY
bin009	SOY3_bin009_01376	990	1	4	1	0.121	0.410	0.107	PhoH-like protein
bin009	SOY3_bin009_01377	471	6	7	3	1.523	1.507	0.677	hypothetical protein
bin009	SOY3_bin009_01378	1005	0	0	0	0.000	0.000	0.000	Ferrochelatase
bin009	SOY3_bin009_01379	1299	3	0	0	0.276	0.000	0.000	Queuine tRNA-ribosyltransferase
bin009	SOY3_bin009_01380	906	0	0	0	0.000	0.000	0.000	Sec-independent protein translocase protein TatC
bin009	SOY3_bin009_01381	387	0	3	0	0.000	0.786	0.000	Sec-independent protein translocase protein TatAd
bin009	SOY3_bin009_01382	948	0	1	0	0.000	0.107	0.000	hypothetical protein
bin009	SOY3_bin009_01383	984	2	1	4	0.243	0.103	0.432	hypothetical protein
bin009	SOY3_bin009_01384	948	1	1	0	0.126	0.107	0.000	Glutamate--tRNA ligase
bin009	SOY3_bin009_01385	1035	2	3	1	0.231	0.294	0.103	FK506-binding protein
bin009	SOY3_bin009_01386	744	5	4	0	0.803	0.545	0.000	Ribosomal large subunit pseudouridine synthase B
bin009	SOY3_bin009_01387	615	0	0	0	0.000	0.000	0.000	Segregation and condensation protein B
bin009	SOY3_bin009_01388	846	0	2	0	0.000	0.240	0.000	Segregation and condensation protein A
bin009	SOY3_bin009_01389	330	1	2	0	0.362	0.615	0.000	hypothetical protein
bin009	SOY3_bin009_01390	927	4	1	2	0.516	0.109	0.229	Sporulation initiation inhibitor protein Soj
bin009	SOY3_bin009_01391	1275	0	2	0	0.000	0.159	0.000	hypothetical protein
bin009	SOY3_bin009_01392	747	0	0	0	0.000	0.000	0.000	High-affinity branched-chain amino acid transport ATP-binding protein LivF
bin009	SOY3_bin009_01393	966	0	1	0	0.000	0.105	0.000	Lipopolysaccharide export system ATP-binding protein LptB
bin009	SOY3_bin009_01394	981	0	0	0	0.000	0.000	0.000	leucine/isoleucine/valine transporter permease subunit
bin009	SOY3_bin009_01395	1377	0	2	0	0.000	0.147	0.000	High-affinity branched-chain amino acid transport system permease protein LivH
bin009	SOY3_bin009_01396	726	0	2	1	0.000	0.279	0.146	VIT family protein
bin009	SOY3_bin009_01397	762	0	0	0	0.000	0.000	0.000	hypothetical protein
bin009	SOY3_bin009_01398	114	1	0	1	1.049	0.000	0.932	5S ribosomal RNA
bin009	SOY3_bin009_01399	1116	0	0	1	0.000	0.000	0.095	Glutamate-pyruvate aminotransferase AlaA
bin009	SOY3_bin009_01400	2496	1	4	1	0.048	0.163	0.043	Phenylalanine--tRNA ligase beta subunit
bin009	SOY3_bin009_01401	1104	0	2	0	0.000	0.184	0.000	Phenylalanine--tRNA ligase alpha subunit
bin009	SOY3_bin009_01402	816	0	0	1	0.000	0.000	0.130	23S rRNA (uridine(2479)-2'-O)-methyltransferase
bin009	SOY3_bin009_01403	366	1	6	10	0.327	1.663	2.902	50S ribosomal protein L20

bin009	SOY3_bin009_01404	219	1	9	5	0.546	4.168	2.425	50S ribosomal protein L35
bin009	SOY3_bin009_01405	603	3	7	11	0.595	1.177	1.938	Translation initiation factor IF-3
bin009	SOY3_bin009_01406	315	8	12	14	3.036	3.864	4.721	hypothetical protein
bin009	SOY3_bin009_01407	510	3	5	3	0.703	0.994	0.625	Tetraspanin family protein
bin009	SOY3_bin009_01408	855	1	3	1	0.140	0.356	0.124	ATP phosphoribosyltransferase
bin009	SOY3_bin009_01409	267	0	1	3	0.000	0.380	1.194	Phosphoribosyl-ATP pyrophosphatase
bin009	SOY3_bin009_01410	2382	0	5	3	0.000	0.213	0.134	Phosphoenolpyruvate-protein phosphotransferase
bin009	SOY3_bin009_01411	609	2	3	1	0.393	0.500	0.174	PTS-dependent dihydroxyacetone kinase, ADP-binding subunit DhaL
bin009	SOY3_bin009_01412	993	4	6	3	0.482	0.613	0.321	PTS-dependent dihydroxyacetone kinase, dihydroxyacetone-binding subunit DhaK
bin009	SOY3_bin009_01413	2061	1	8	7	0.058	0.394	0.361	ATP-dependent zinc metalloprotease FtsH
bin009	SOY3_bin009_01414	1257	2	0	2	0.190	0.000	0.169	L-cysteine:1D-myo-inositol 2-amino-2-deoxy-alpha-D-glucopyranoside ligase
bin009	SOY3_bin009_01415	567	2	2	2	0.422	0.358	0.375	hypothetical protein
bin009	SOY3_bin009_01416	891	3	3	0	0.403	0.342	0.000	Undecaprenyl-diphosphatase
bin009	SOY3_bin009_01417	963	0	2	2	0.000	0.211	0.221	General stress protein 69
bin009	SOY3_bin009_01418	609	1	2	0	0.196	0.333	0.000	Penicillin-binding protein activator LpoB
bin009	SOY3_bin009_01419	252	1	1	0	0.474	0.402	0.000	hypothetical protein
bin009	SOY3_bin009_01420	87	8	6	1	10.993	6.995	1.221	tRNA-Leu(gag)
bin009	SOY3_bin009_01421	903	0	0	0	0.000	0.000	0.000	hypothetical protein
bin009	SOY3_bin009_01422	648	0	0	0	0.000	0.000	0.000	Adenosylmethionine-8-amino-7-oxononanoate aminotransferase
bin009	SOY3_bin009_01423	780	1	2	1	0.153	0.260	0.136	Phosphate import ATP-binding protein PstB
bin009	SOY3_bin009_01424	1119	0	1	0	0.000	0.091	0.000	Phosphate transport system permease protein PstA
bin009	SOY3_bin009_01425	999	0	3	1	0.000	0.305	0.106	Phosphate transport system permease protein PstC
bin009	SOY3_bin009_01426	1095	1	1	0	0.109	0.093	0.000	Phosphate-binding protein PstS 3 precursor
bin009	SOY3_bin009_01427	879	0	3	1	0.000	0.346	0.121	Diadenosine hexaphosphate hydrolase
bin009	SOY3_bin009_01428	2097	1	5	2	0.057	0.242	0.101	Polyphosphate kinase
bin009	SOY3_bin009_01429	894	0	1	1	0.000	0.113	0.119	Mycothiol acetyltransferase
bin009	SOY3_bin009_01430	669	0	3	4	0.000	0.455	0.635	Transcriptional regulatory protein YycF
bin009	SOY3_bin009_01431	405	0	1	0	0.000	0.250	0.000	D-tyrosyl-tRNA(Tyr) deacylase
bin009	SOY3_bin009_01432	498	0	4	3	0.000	0.815	0.640	hypothetical protein
bin009	SOY3_bin009_01433	933	2	3	1	0.256	0.326	0.114	putative global regulator
bin009	SOY3_bin009_01434	1875	2	1	0	0.128	0.054	0.000	GTP-binding protein TypA/BipA
bin009	SOY3_bin009_01435	1065	1	1	0	0.112	0.095	0.000	Limonene 1,2-monooxygenase
bin009	SOY3_bin009_01436	1275	2	7	4	0.188	0.557	0.333	Toxic anion resistance protein (TelA)
bin009	SOY3_bin009_01437	534	0	2	1	0.000	0.380	0.199	hypothetical protein
bin009	SOY3_bin009_01438	1668	0	10	2	0.000	0.608	0.127	Stage V sporulation protein K
bin009	SOY3_bin009_01439	1275	0	3	0	0.000	0.239	0.000	hypothetical protein
bin009	SOY3_bin009_01440	825	1	0	1	0.145	0.000	0.129	hypothetical protein
bin009	SOY3_bin009_01441	963	0	1	3	0.000	0.105	0.331	Putative 2-hydroxyacid dehydrogenase
bin009	SOY3_bin009_01442	1155	1	0	0	0.104	0.000	0.000	hypothetical protein
bin009	SOY3_bin009_01443	600	0	1	0	0.000	0.169	0.000	Phosphoserine phosphatase 1
bin009	SOY3_bin009_01444	1197	0	0	0	0.000	0.000	0.000	Phosphoenolpyruvate synthase
bin009	SOY3_bin009_01445	396	0	0	0	0.000	0.000	0.000	LexA DNA binding domain protein
bin009	SOY3_bin009_01446	636	0	0	0	0.000	0.000	0.000	hypothetical protein
bin009	SOY3_bin009_01447	2058	0	1	0	0.000	0.049	0.000	Lon protease 2
bin009	SOY3_bin009_01448	2334	0	2	0	0.000	0.087	0.000	PglZ domain protein
bin009	SOY3_bin009_01449	3237	1	0	0	0.037	0.000	0.000	N-6 DNA Methylase
bin009	SOY3_bin009_01450	3459	1	0	1	0.035	0.000	0.031	hypothetical protein
bin009	SOY3_bin009_01451	558	0	0	0	0.000	0.000	0.000	hypothetical protein
bin009	SOY3_bin009_01452	756	0	0	0	0.000	0.000	0.000	hypothetical protein
bin009	SOY3_bin009_01453	1092	0	1	0	0.000	0.093	0.000	hypothetical protein
bin009	SOY3_bin009_01454	75	0	0	0	0.000	0.000	0.000	tRNA-Ala(tgc)
bin009	SOY3_bin009_01455	435	0	0	0	0.000	0.000	0.000	hypothetical protein
bin009	SOY3_bin009_01456	501	0	3	0	0.000	0.607	0.000	cytoplasmic glycerophosphodiester phosphodiesterase
bin009	SOY3_bin009_01457	1221	4	6	4	0.392	0.498	0.348	putative lipoprotein YbbD precursor
bin009	SOY3_bin009_01458	1464	2	4	1	0.163	0.277	0.073	Mycothione reductase
bin009	SOY3_bin009_01459	678	0	1	2	0.000	0.150	0.313	bifunctional RNase H/acid phosphatase
bin009	SOY3_bin009_01460	1608	1	5	2	0.074	0.315	0.132	Uroporphyrinogen-III C-methyltransferase
bin009	SOY3_bin009_01461	753	1	2	1	0.159	0.269	0.141	Pyrroline-5-carboxylate reductase
bin009	SOY3_bin009_01462	1101	3	10	3	0.326	0.921	0.289	Aspartate-semialdehyde dehydrogenase
bin009	SOY3_bin009_01463	774	0	0	0	0.000	0.000	0.000	CAAX amino terminal protease self- immunity
bin009	SOY3_bin009_01464	930	0	2	2	0.000	0.218	0.228	Proline dehydrogenase 1
bin009	SOY3_bin009_01465	798	1	2	0	0.150	0.254	0.000	fructoselysine 3-epimerase
bin009	SOY3_bin009_01466	639	3	6	4	0.561	0.952	0.665	hypothetical protein
bin009	SOY3_bin009_01467	1428	0	3	0	0.000	0.213	0.000	hypothetical protein
bin009	SOY3_bin009_01468	1059	0	2	2	0.000	0.192	0.201	DNA integrity scanning protein DisA
bin009	SOY3_bin009_01469	699	0	0	0	0.000	0.000	0.000	hypothetical protein
bin009	SOY3_bin009_01470	861	1	1	3	0.139	0.118	0.370	A/G-specific adenine glycosylase

bin009	SOY3_bin009_01471	2511	2	4	5	0.095	0.162	0.212	Alpha,alpha-trehalose phosphorylase
bin009	SOY3_bin009_01472	642	0	1	0	0.000	0.158	0.000	putative glycosyl hydrolase/MT2062
bin009	SOY3_bin009_01473	1644	0	0	0	0.000	0.000	0.000	Major cardiolipin synthase ClsA
bin009	SOY3_bin009_01474	624	0	0	0	0.000	0.000	0.000	hypothetical protein
bin009	SOY3_bin009_01475	1200	0	1	1	0.000	0.085	0.089	Alanine-anticapsin ligase BacD
bin009	SOY3_bin009_01476	459	1	1	1	0.260	0.221	0.231	Ribosome-binding factor A
bin009	SOY3_bin009_01477	2922	8	31	24	0.327	1.076	0.872	Translation initiation factor IF-2
bin009	SOY3_bin009_01478	234	1	0	1	0.511	0.000	0.454	hypothetical protein
bin009	SOY3_bin009_01479	978	6	14	10	0.733	1.452	1.086	hypothetical protein
bin009	SOY3_bin009_01480	492	2	7	6	0.486	1.443	1.295	Ribosome maturation factor RimP
bin009	SOY3_bin009_01481	1032	0	1	2	0.000	0.098	0.206	hypothetical protein
bin009	SOY3_bin009_01482	1749	0	1	1	0.000	0.058	0.061	Proline--tRNA ligase
bin009	SOY3_bin009_01483	1620	0	1	0	0.000	0.063	0.000	Lipid A export ATP-binding/permease protein MsbA
bin009	SOY3_bin009_01484	1695	0	0	2	0.000	0.000	0.125	putative ABC transporter ATP-binding protein
bin009	SOY3_bin009_01485	861	0	1	1	0.000	0.118	0.123	putative acetyltransferase
bin009	SOY3_bin009_01486	1143	1	4	4	0.105	0.355	0.372	4-hydroxy-3-methylbut-2-en-1-yl diphosphate synthase
bin009	SOY3_bin009_01487	1137	1	2	0	0.105	0.178	0.000	LOG family protein YgdH
bin009	SOY3_bin009_01488	636	0	0	0	0.000	0.000	0.000	hypothetical protein
bin009	SOY3_bin009_01489	1143	0	2	0	0.000	0.177	0.000	putative dual-specificity RNA methyltransferase RlmN
bin009	SOY3_bin009_01490	894	1	6	1	0.134	0.681	0.119	Phosphatidate cytidylyltransferase
bin009	SOY3_bin009_01491	258	0	5	1	0.000	1.966	0.412	Ribosome-recycling factor
bin009	SOY3_bin009_01492	915	0	0	0	0.000	0.000	0.000	hypothetical protein
bin009	SOY3_bin009_01493	1419	0	5	4	0.000	0.357	0.299	Glycine--tRNA ligase
bin009	SOY3_bin009_01494	1047	0	2	0	0.000	0.194	0.000	High-affinity zinc uptake system binding-protein ZnuA precursor
bin009	SOY3_bin009_01495	771	0	0	0	0.000	0.000	0.000	Sulfate/thiosulfate import ATP-binding protein CysA
bin009	SOY3_bin009_01496	981	1	0	1	0.122	0.000	0.108	High-affinity zinc uptake system membrane protein ZnuB
bin009	SOY3_bin009_01497	450	0	2	0	0.000	0.451	0.000	Zinc uptake regulation protein
bin009	SOY3_bin009_01498	1443	1	1	3	0.083	0.070	0.221	Periplasmic serine endoprotease DegP precursor
bin009	SOY3_bin009_01499	750	1	2	3	0.159	0.270	0.425	DNA repair protein RecO
bin009	SOY3_bin009_01500	792	0	1	0	0.000	0.128	0.000	Trans,polycis-polyprenyl diphosphate synthase ((2Z,6E)-farnesyl diphosphate specific)
bin009	SOY3_bin009_01501	510	0	1	0	0.000	0.199	0.000	putative nicotinate-nucleotide pyrophosphorylase [carboxylating]
bin009	SOY3_bin009_01502	897	0	0	1	0.000	0.000	0.118	Fluoroquinolones export ATP-binding protein/MT2762
bin009	SOY3_bin009_01503	864	0	1	0	0.000	0.117	0.000	ABC-2 family transporter protein
bin009	SOY3_bin009_01504	225	0	0	0	0.000	0.000	0.000	HTH-type transcriptional regulator SinR
bin009	SOY3_bin009_01505	1071	0	0	0	0.000	0.000	0.000	Biotin synthase
bin009	SOY3_bin009_01506	819	0	3	1	0.000	0.372	0.130	Inactive dihydropteroate synthase 2
bin009	SOY3_bin009_01507	1782	3	23	15	0.201	1.309	0.894	2-isopropylmalate synthase
bin009	SOY3_bin009_01508	1482	1	5	5	0.081	0.342	0.358	Polyol:NADP oxidoreductase
bin009	SOY3_bin009_01509	942	0	2	1	0.000	0.215	0.113	GTPase Era
bin009	SOY3_bin009_01510	735	1	2	0	0.163	0.276	0.000	Magnesium and cobalt efflux protein CorC
bin009	SOY3_bin009_01511	990	0	0	0	0.000	0.000	0.000	Oligopeptide-binding protein AppA precursor
bin009	SOY3_bin009_01512	1041	0	0	0	0.000	0.000	0.000	Nickel transport system permease protein NikB
bin009	SOY3_bin009_01513	849	0	0	0	0.000	0.000	0.000	Oligopeptide transport system permease protein OppC
bin009	SOY3_bin009_01514	1554	2	0	1	0.154	0.000	0.068	Glutathione import ATP-binding protein GsiA
bin009	SOY3_bin009_01515	696	0	1	0	0.000	0.146	0.000	Urease subunit alpha
bin009	SOY3_bin009_01516	1701	0	2	2	0.000	0.119	0.125	Urease subunit alpha
bin009	SOY3_bin009_01517	648	0	1	1	0.000	0.157	0.164	Urease accessory protein UreF
bin009	SOY3_bin009_01518	768	0	3	0	0.000	0.396	0.000	Urease accessory protein UreG
bin009	SOY3_bin009_01519	741	0	0	1	0.000	0.000	0.143	Urease accessory protein UreD
bin009	SOY3_bin009_01520	1245	1	2	0	0.096	0.163	0.000	Phosphoserine phosphatase
bin009	SOY3_bin009_01521	1284	2	8	2	0.186	0.632	0.165	Adenylosuccinate synthetase
bin009	SOY3_bin009_01522	774	0	1	1	0.000	0.131	0.137	Inner membrane protein YhhQ
bin009	SOY3_bin009_01523	864	0	0	0	0.000	0.000	0.000	hypothetical protein
bin009	SOY3_bin009_01524	1194	1	1	1	0.100	0.085	0.089	FHA domain-containing protein FhaB
bin009	SOY3_bin009_01525	1320	2	8	5	0.181	0.615	0.402	von Willebrand factor type A domain protein
bin009	SOY3_bin009_01526	1293	1	2	1	0.092	0.157	0.082	Putative protein phosphatase 2C-type
bin009	SOY3_bin009_01527	2490	0	3	0	0.000	0.122	0.000	Serine/threonine-protein kinase PknG
bin009	SOY3_bin009_01528	210	0	0	0	0.000	0.000	0.000	hypothetical protein
bin009	SOY3_bin009_01529	1554	4	7	14	0.308	0.457	0.957	hypothetical protein
bin009	SOY3_bin009_01530	1191	9	16	10	0.903	1.363	0.892	UDP-galactopyranose mutase
bin009	SOY3_bin009_01531	1278	1	0	0	0.094	0.000	0.000	putative deoxyribonuclease RhsB
bin009	SOY3_bin009_01532	681	1	0	1	0.176	0.000	0.156	hypothetical protein
bin009	SOY3_bin009_01533	2424	1	4	3	0.049	0.167	0.131	PGL/p-HBAD biosynthesis glycosyltransferase/MT3031
bin009	SOY3_bin009_01534	1203	0	0	1	0.000	0.000	0.088	hypothetical protein
bin009	SOY3_bin009_01535	1956	0	2	0	0.000	0.104	0.000	hypothetical protein
bin009	SOY3_bin009_01536	981	3	6	5	0.366	0.620	0.541	UDP-glucose 4-epimerase
bin009	SOY3_bin009_01537	375	0	1	0	0.000	0.270	0.000	hypothetical protein

bin009	SOY3_bin009_01538	672	6	7	3	1.067	1.057	0.474	Undecaprenyl-phosphate 4-deoxy-4-formamido-L-arabinose transferase
bin009	SOY3_bin009_01539	1113	18	27	20	1.933	2.460	1.909	6-phosphogluconolactonase
bin009	SOY3_bin009_01540	2112	19	44	22	1.075	2.113	1.107	Peptidoglycan-N-acetylglucosamine deacetylase
bin009	SOY3_bin009_01541	447	0	0	0	0.000	0.000	0.000	GtrA-like protein
bin009	SOY3_bin009_01542	993	0	1	0	0.000	0.102	0.000	dTDP-glucose 4,6-dehydratase
bin009	SOY3_bin009_01543	273	0	1	1	0.000	0.372	0.389	Acylphosphatase
bin009	SOY3_bin009_01544	543	15	23	10	3.302	4.296	1.956	Universal stress protein/MT2698
bin009	SOY3_bin009_01545	2181	1	2	1	0.055	0.093	0.049	DNA polymerase III subunit tau
bin009	SOY3_bin009_01546	309	0	1	1	0.000	0.328	0.344	Nucleoid-associated protein/MT3819
bin009	SOY3_bin009_01547	600	1	2	2	0.199	0.338	0.354	Recombination protein RecR
bin009	SOY3_bin009_01548	735	4	2	1	0.651	0.276	0.145	hypothetical protein
bin009	SOY3_bin009_01549	3183	1	8	3	0.038	0.255	0.100	Isoleucine--tRNA ligase
bin009	SOY3_bin009_01550	2880	4	9	1	0.166	0.317	0.037	Calcium-transporting ATPase
bin009	SOY3_bin009_01551	981	3	2	2	0.366	0.207	0.217	Quinone oxidoreductase 1
bin009	SOY3_bin009_01552	1350	0	0	0	0.000	0.000	0.000	DsdX permease
bin009	SOY3_bin009_01553	1614	0	0	1	0.000	0.000	0.066	Sensor histidine kinase DcuS
bin009	SOY3_bin009_01554	687	0	0	0	0.000	0.000	0.000	Transcriptional regulatory protein CitB
bin009	SOY3_bin009_01555	738	0	0	1	0.000	0.000	0.144	Purine nucleoside phosphorylase DeoD-type
bin009	SOY3_bin009_01556	1734	0	1	0	0.000	0.058	0.000	putative phosphomannomutase
bin009	SOY3_bin009_01557	1230	2	2	1	0.194	0.165	0.086	putative N-succinyldiaminopimelate aminotransferase DapC
bin009	SOY3_bin009_01558	972	1	2	0	0.123	0.209	0.000	L-lactate dehydrogenase 2
bin009	SOY3_bin009_01559	867	0	2	0	0.000	0.234	0.000	Polyphosphate kinase 2 (PPK2)
bin009	SOY3_bin009_01560	1839	0	0	0	0.000	0.000	0.000	ATP-dependent DNA helicase RecQ
bin009	SOY3_bin009_01561	1290	0	0	0	0.000	0.000	0.000	Phthalate 4,5-dioxygenase oxygenase reductase subunit
bin009	SOY3_bin009_01562	858	0	0	0	0.000	0.000	0.000	Thiamine biosynthesis lipoprotein ApbE precursor
bin009	SOY3_bin009_01563	204	0	0	0	0.000	0.000	0.000	hypothetical protein
bin009	SOY3_bin009_01564	609	0	0	0	0.000	0.000	0.000	hypothetical protein
bin009	SOY3_bin009_01565	1005	0	2	0	0.000	0.202	0.000	Glyceraldehyde-3-phosphate dehydrogenase
bin009	SOY3_bin009_01566	663	0	0	0	0.000	0.000	0.000	putative HTH-type transcriptional regulator YxaF
bin009	SOY3_bin009_01567	945	0	1	1	0.000	0.107	0.112	Daunorubicin/doxorubicin resistance ATP-binding protein DrrA
bin009	SOY3_bin009_01568	1623	0	0	0	0.000	0.000	0.000	ABC-2 family transporter protein
bin009	SOY3_bin009_01569	1239	0	0	0	0.000	0.000	0.000	putative inactive lipase/MT1628
bin009	SOY3_bin009_01570	366	3	2	0	0.980	0.554	0.000	glyoxalase I
bin009	SOY3_bin009_01571	1164	2	1	0	0.205	0.087	0.000	N-acetylglucosamine-6-phosphate deacetylase
bin009	SOY3_bin009_01572	786	0	0	0	0.000	0.000	0.000	Glucosamine-6-phosphate deaminase
bin009	SOY3_bin009_01573	939	0	0	0	0.000	0.000	0.000	PTS system glucose-specific EIICBA component
bin009	SOY3_bin009_01574	603	1	1	0	0.198	0.168	0.000	hypothetical protein
bin009	SOY3_bin009_01575	1953	1	4	1	0.061	0.208	0.054	Erythromycin C-12 hydroxylase
bin009	SOY3_bin009_01576	1002	0	1	0	0.000	0.101	0.000	3-oxoacyl-[acyl-carrier-protein] synthase 3
bin009	SOY3_bin009_01577	555	0	0	0	0.000	0.000	0.000	hypothetical protein
bin009	SOY3_bin009_01578	5037	2	18	15	0.047	0.362	0.316	Pyruvate-flavodoxin oxidoreductase
bin009	SOY3_bin009_01579	2829	7	19	4	0.296	0.681	0.150	Glutamate synthase [NADPH] small chain
bin009	SOY3_bin009_01580	1596	0	0	2	0.000	0.000	0.133	putative ABC transporter ATP-binding protein YheS
bin009	SOY3_bin009_01581	1080	0	0	0	0.000	0.000	0.000	putative carboxylesterase nap
bin009	SOY3_bin009_01582	615	0	3	1	0.000	0.495	0.173	HTH-type transcriptional regulator BetI
bin009	SOY3_bin009_01583	783	1	3	2	0.153	0.389	0.271	Ribonucleotide monophosphatase NagD
bin009	SOY3_bin009_01584	237	0	0	0	0.000	0.000	0.000	hypothetical protein
bin009	SOY3_bin009_01585	894	0	0	0	0.000	0.000	0.000	Phosphatidate cytidylyltransferase
bin009	SOY3_bin009_01586	558	4	2	2	0.857	0.364	0.381	Ribosome-recycling factor
bin009	SOY3_bin009_01587	714	5	5	4	0.837	0.710	0.595	Uridylate kinase
bin009	SOY3_bin009_01588	810	3	9	10	0.443	1.127	1.311	Elongation factor Ts
bin009	SOY3_bin009_01589	927	4	11	11	0.516	1.204	1.260	30S ribosomal protein S2
bin009	SOY3_bin009_01590	969	0	0	0	0.000	0.000	0.000	Murein DD-endopeptidase MepM
bin009	SOY3_bin009_01591	942	1	1	0	0.127	0.108	0.000	Tyrosine recombinase XerD
bin009	SOY3_bin009_01592	594	0	4	3	0.000	0.683	0.536	Peptide deformylase
bin009	SOY3_bin009_01593	1707	4	13	4	0.280	0.772	0.249	putative NAD-dependent malic enzyme 2
bin009	SOY3_bin009_01594	810	0	2	0	0.000	0.250	0.000	23S rRNA (guanosine-2'-O-)-methyltransferase RlmB
bin009	SOY3_bin009_01595	1005	0	0	0	0.000	0.000	0.000	Sodium Bile acid symporter family protein
bin009	SOY3_bin009_01596	783	1	6	4	0.153	0.777	0.543	putative ABC transporter ATP-binding protein YlmA
bin009	SOY3_bin009_01597	492	0	3	1	0.000	0.618	0.216	hypothetical protein
bin009	SOY3_bin009_01598	774	3	5	2	0.463	0.655	0.274	Enoyl-[acyl-carrier-protein] reductase [NADH]
bin009	SOY3_bin009_01599	729	3	5	5	0.492	0.696	0.729	3-oxoacyl-[acyl-carrier-protein] reductase FabG1
bin009	SOY3_bin009_01600	888	5	14	0	0.673	1.599	0.000	23S rRNA (guanine(748)-N(1))-methyltransferase
bin009	SOY3_bin009_01601	249	12	13	10	5.761	5.295	4.266	hypothetical protein
bin009	SOY3_bin009_01602	1611	0	1	3	0.000	0.063	0.198	putative ABC transporter ATP-binding protein YheS
bin009	SOY3_bin009_01603	627	1	1	1	0.191	0.162	0.169	2'-5'-RNA ligase
bin009	SOY3_bin009_01604	387	1	1	0	0.309	0.262	0.000	hypothetical protein

bin009	SOY3_bin009_01605	210	0	0	0	0.000	0.000	0.000	NifU-like protein
bin009	SOY3_bin009_01606	1107	0	3	2	0.000	0.275	0.192	5-dehydro-2-deoxygluconokinase
bin009	SOY3_bin009_01607	645	0	1	2	0.000	0.157	0.329	Putative KHG/KDPG aldolase
bin009	SOY3_bin009_01608	792	0	0	0	0.000	0.000	0.000	Transcriptional regulator KdgR
bin009	SOY3_bin009_01609	345	1	2	1	0.347	0.588	0.308	hypothetical protein
bin009	SOY3_bin009_01610	399	8	22	9	2.397	5.592	2.396	Putative thioredoxin-2
bin009	SOY3_bin009_01611	717	1	7	1	0.167	0.990	0.148	FHA domain-containing protein FhaA
bin009	SOY3_bin009_01612	522	2	5	3	0.458	0.972	0.610	FHA domain-containing protein FhaB
bin009	SOY3_bin009_01613	1518	4	2	6	0.315	0.134	0.420	PP2C-family Ser/Thr phosphatase
bin009	SOY3_bin009_01614	1410	3	3	3	0.254	0.216	0.226	Lipid II flippase FtsW
bin009	SOY3_bin009_01615	1407	2	3	3	0.170	0.216	0.226	Penicillin-binding protein A
bin009	SOY3_bin009_01616	1998	0	9	1	0.000	0.457	0.053	Serine/threonine-protein kinase PknB
bin009	SOY3_bin009_01617	633	0	5	2	0.000	0.801	0.336	Aminodeoxychorismate/anthranilate synthase component 2
bin009	SOY3_bin009_01618	834	2	1	2	0.287	0.122	0.255	hypothetical protein
bin009	SOY3_bin009_01619	297	3	4	2	1.208	1.366	0.715	Cell division protein CrgA
bin009	SOY3_bin009_01620	399	3	1	1	0.899	0.254	0.266	Na(+)/H(+) antiporter subunit G1
bin009	SOY3_bin009_01621	417	0	0	1	0.000	0.000	0.255	Na(+)/H(+) antiporter subunit F
bin009	SOY3_bin009_01622	681	0	0	1	0.000	0.000	0.156	Na(+)/H(+) antiporter subunit E
bin009	SOY3_bin009_01623	1656	0	1	1	0.000	0.061	0.064	Na(+)/H(+) antiporter subunit D
bin009	SOY3_bin009_01624	531	0	1	1	0.000	0.191	0.200	Na(+)/H(+) antiporter subunit C
bin009	SOY3_bin009_01625	744	0	1	1	0.000	0.136	0.143	Na(+)/H(+) antiporter subunit B
bin009	SOY3_bin009_01626	963	2	1	1	0.248	0.105	0.110	Glycerate dehydrogenase
bin009	SOY3_bin009_01627	1446	0	0	4	0.000	0.000	0.294	Amidophosphoribosyltransferase precursor
bin009	SOY3_bin009_01628	1056	0	0	0	0.000	0.000	0.000	Phosphoribosylformylglycinamidine cyclo-ligase
bin009	SOY3_bin009_01629	228	6	14	8	3.146	6.228	3.727	hypothetical protein
bin009	SOY3_bin009_01630	957	2	9	5	0.250	0.954	0.555	Phospholipase YtpA
bin009	SOY3_bin009_01631	76	1	0	0	1.573	0.000	0.000	tRNA-Phe(gaa)
bin009	SOY3_bin009_01632	77	1	0	1	1.553	0.000	1.380	tRNA-Asp(gtc)
bin009	SOY3_bin009_01633	696	1	0	0	0.172	0.000	0.000	3-oxoacyl-[acyl-carrier-protein] reductase FabG
bin009	SOY3_bin009_01634	771	1	0	0	0.155	0.000	0.000	N5-carboxyaminoimidazole ribonucleotide mutase
bin009	SOY3_bin009_01635	1356	3	2	1	0.264	0.150	0.078	hypothetical protein
bin009	SOY3_bin009_01636	804	1	7	0	0.149	0.883	0.000	Pyrroline-5-carboxylate reductase
bin009	SOY3_bin009_01637	504	2	6	6	0.474	1.207	1.265	DinB superfamily protein
bin009	SOY3_bin009_01638	909	2	1	1	0.263	0.112	0.117	Putative osmoprotectant uptake system substrate-binding protein OsmF precursor
bin009	SOY3_bin009_01639	969	0	1	0	0.000	0.105	0.000	Pyrimidine-specific ribonucleoside hydrolase RihA
bin009	SOY3_bin009_01640	930	0	1	0	0.000	0.109	0.000	Ribokinase
bin009	SOY3_bin009_01641	693	0	1	0	0.000	0.146	0.000	Glycine betaine/carnitine/choline transport system permease protein OpuCB
bin009	SOY3_bin009_01642	639	0	1	1	0.000	0.159	0.166	Putative osmoprotectant uptake system permease protein YehY
bin009	SOY3_bin009_01643	882	1	0	0	0.136	0.000	0.000	Carnitine transport ATP-binding protein OpuCA
bin009	SOY3_bin009_01644	1044	0	3	5	0.000	0.291	0.509	hypothetical protein
bin009	SOY3_bin009_01645	231	2	1	1	1.035	0.439	0.460	NIL domain protein
bin009	SOY3_bin009_01646	165	1	2	1	0.725	1.229	0.644	hypothetical protein
bin009	SOY3_bin009_01647	339	0	0	0	0.000	0.000	0.000	tRNA pseudouridine synthase B
bin009	SOY3_bin009_01648	546	3	5	6	0.657	0.929	1.167	acid-resistance membrane protein
bin009	SOY3_bin009_01649	600	1	2	1	0.199	0.338	0.177	putative antibiotic transporter
bin009	SOY3_bin009_01650	915	1	5	1	0.131	0.554	0.116	Riboflavin biosynthesis protein RibF
bin009	SOY3_bin009_01651	264	2	8	7	0.906	3.074	2.817	30S ribosomal protein S15
bin009	SOY3_bin009_01652	2220	5	24	9	0.269	1.097	0.431	Polyribonucleotide nucleotidyltransferase
bin009	SOY3_bin009_01653	1068	0	1	0	0.000	0.095	0.000	hypothetical protein
bin009	SOY3_bin009_01654	741	0	3	3	0.000	0.411	0.430	4-hydroxy-tetrahydrodipicolinate reductase
bin009	SOY3_bin009_01655	696	1	1	1	0.172	0.146	0.153	hypothetical protein
bin009	SOY3_bin009_01656	537	0	1	0	0.000	0.189	0.000	Acetyltransferase (GNAT) family protein
bin009	SOY3_bin009_01657	780	0	1	0	0.000	0.130	0.000	Iron(3+)-hydroxamate import ATP-binding protein FhuC
bin009	SOY3_bin009_01658	1098	0	1	0	0.000	0.092	0.000	Hemin transport system permease protein HmuU
bin009	SOY3_bin009_01659	969	0	0	0	0.000	0.000	0.000	Fe(3+)-citrate-binding protein YfmC precursor
bin009	SOY3_bin009_01660	1689	1	2	0	0.071	0.120	0.000	Putative ribonuclease J
bin009	SOY3_bin009_01661	186	0	2	0	0.000	1.091	0.000	50S ribosomal protein L28
bin009	SOY3_bin009_01662	2211	1	2	1	0.054	0.092	0.048	ATP-dependent DNA helicase RecG
bin009	SOY3_bin009_01663	525	0	2	4	0.000	0.386	0.809	Pyridoxamine 5'-phosphate oxidase
bin009	SOY3_bin009_01664	120	0	1	1	0.000	0.845	0.885	hypothetical protein
bin009	SOY3_bin009_01665	603	0	1	0	0.000	0.168	0.000	hypothetical protein
bin009	SOY3_bin009_01666	222	0	0	2	0.000	0.000	0.957	hypothetical protein
bin009	SOY3_bin009_01667	75	0	0	0	0.000	0.000	0.000	tRNA-Pro(ggg)
bin009	SOY3_bin009_01668	822	1	2	1	0.145	0.247	0.129	Ribonucleotide monophosphatase NagD
bin009	SOY3_bin009_01669	333	1	1	2	0.359	0.305	0.638	lineage-specific thermal regulator protein
bin009	SOY3_bin009_01670	813	5	7	2	0.735	0.873	0.261	hypothetical protein
bin009	SOY3_bin009_01671	744	8	7	5	1.285	0.954	0.714	NUDIX domain protein

bin009	SOY3_bin009_01672	147	6	12	8	4.880	8.280	5.781	hypothetical protein
bin009	SOY3_bin009_01673	426	15	23	12	4.209	5.476	2.992	Peroxiredoxin OsmC
bin009	SOY3_bin009_01674	2154	0	1	0	0.000	0.047	0.000	Helicase IV
bin009	SOY3_bin009_01675	348	3	1	3	1.031	0.291	0.916	Glyoxalase-like domain protein
bin009	SOY3_bin009_01676	1350	2	7	1	0.177	0.526	0.079	GTPase Der
bin009	SOY3_bin009_01677	699	0	0	1	0.000	0.000	0.152	Cytidylate kinase
bin009	SOY3_bin009_01678	1068	1	0	0	0.112	0.000	0.000	prephenate dehydrogenase
bin009	SOY3_bin009_01679	672	1	1	1	0.178	0.151	0.158	Proteasome subunit alpha
bin009	SOY3_bin009_01680	768	1	3	0	0.156	0.396	0.000	Proteasome subunit beta precursor
bin009	SOY3_bin009_01681	930	0	2	0	0.000	0.218	0.000	Gluconolactonase precursor
bin009	SOY3_bin009_01682	1602	2	5	1	0.149	0.317	0.066	Peptidoglycan-N-acetylglucosamine deacetylase
bin009	SOY3_bin009_01683	615	2	3	0	0.389	0.495	0.000	hypothetical protein
bin009	SOY3_bin009_01684	951	0	1	0	0.000	0.107	0.000	tRNA (adenine(58)-N(1))-methyltransferase TrmI
bin009	SOY3_bin009_01685	186	0	0	0	0.000	0.000	0.000	hypothetical protein
bin009	SOY3_bin009_01686	3741	8	12	9	0.256	0.325	0.256	Pyruvate-flavodoxin oxidoreductase
bin009	SOY3_bin009_01687	1635	4	3	8	0.292	0.186	0.520	NAD-dependent dihydropyrimidine dehydrogenase subunit PreT
bin009	SOY3_bin009_01688	306	0	0	4	0.000	0.000	1.389	hypothetical protein
bin009	SOY3_bin009_01689	768	0	1	0	0.000	0.132	0.000	Endonuclease 8 1
bin009	SOY3_bin009_01690	288	0	0	1	0.000	0.000	0.369	hypothetical protein
bin009	SOY3_bin009_01691	441	1	1	0	0.271	0.230	0.000	hypothetical protein
bin009	SOY3_bin009_01692	645	0	0	0	0.000	0.000	0.000	D-lactate dehydrogenase
bin009	SOY3_bin009_01693	645	0	0	0	0.000	0.000	0.000	D-lactate dehydrogenase
bin009	SOY3_bin009_01694	441	0	0	1	0.000	0.000	0.241	hypothetical protein
bin009	SOY3_bin009_01695	288	0	1	0	0.000	0.352	0.000	hypothetical protein
bin009	SOY3_bin009_01696	768	0	0	1	0.000	0.000	0.138	Endonuclease 8 1
bin009	SOY3_bin009_01697	306	1	2	2	0.391	0.663	0.694	hypothetical protein
bin009	SOY3_bin009_01698	1635	1	7	8	0.073	0.434	0.520	NAD-dependent dihydropyrimidine dehydrogenase subunit PreT
bin009	SOY3_bin009_01699	3741	4	13	9	0.128	0.352	0.256	Pyruvate-flavodoxin oxidoreductase
bin009	SOY3_bin009_01700	615	0	0	2	0.000	0.000	0.345	Holliday junction ATP-dependent DNA helicase RuvA
bin009	SOY3_bin009_01701	543	1	1	0	0.220	0.187	0.000	Crossover junction endodeoxyribonuclease RuvC
bin009	SOY3_bin009_01702	801	0	7	2	0.000	0.886	0.265	putative transcriptional regulatory protein/MSMEI_2866
bin009	SOY3_bin009_01703	1026	1	1	0	0.117	0.099	0.000	putative acetyltransferase
bin009	SOY3_bin009_01704	765	0	2	3	0.000	0.265	0.417	Phosphoribosyl isomerase A
bin009	SOY3_bin009_01705	615	1	1	0	0.194	0.165	0.000	Imidazole glycerol phosphate synthase subunit HisH
bin009	SOY3_bin009_01706	618	4	1	0	0.774	0.164	0.000	Imidazoleglycerol-phosphate dehydratase
bin009	SOY3_bin009_01707	1137	1	0	2	0.105	0.000	0.187	Histidinol-phosphate aminotransferase
bin009	SOY3_bin009_01708	1302	1	4	4	0.092	0.312	0.326	Histidinol dehydrogenase
bin009	SOY3_bin009_01709	774	1	0	0	0.154	0.000	0.000	putative membrane protein YdfK
bin009	SOY3_bin009_01710	510	0	1	1	0.000	0.199	0.208	Cys-tRNA(Pro)/Cys-tRNA(Cys) deacylase YbaK
bin009	SOY3_bin009_01711	699	1	3	6	0.171	0.435	0.912	hypothetical protein
bin009	SOY3_bin009_01712	3522	1	10	6	0.034	0.288	0.181	DNA polymerase III subunit alpha
bin009	SOY3_bin009_01713	324	3	4	2	1.107	1.252	0.656	putative quinol monooxygenase YgiN
bin009	SOY3_bin009_01714	1401	0	0	0	0.000	0.000	0.000	Inner membrane protein YjjP
bin009	SOY3_bin009_01715	1032	0	0	1	0.000	0.000	0.103	PTS system beta-glucoside-specific EIIBCA component
bin009	SOY3_bin009_01716	459	0	0	0	0.000	0.000	0.000	hypothetical protein
bin009	SOY3_bin009_01717	669	0	0	0	0.000	0.000	0.000	hypothetical protein
bin009	SOY3_bin009_01718	3237	1	4	0	0.037	0.125	0.000	LysM domain/BON superfamily protein
bin009	SOY3_bin009_01719	441	0	1	0	0.000	0.230	0.000	TadE-like protein
bin009	SOY3_bin009_01720	432	0	2	0	0.000	0.470	0.000	TadE-like protein
bin009	SOY3_bin009_01721	420	0	1	2	0.000	0.241	0.506	TadE-like protein
bin009	SOY3_bin009_01722	213	0	3	0	0.000	1.429	0.000	hypothetical protein
bin009	SOY3_bin009_01723	915	0	3	0	0.000	0.333	0.000	Bacterial type II secretion system protein F domain protein
bin009	SOY3_bin009_01724	861	3	5	1	0.417	0.589	0.123	Bacterial type II secretion system protein F domain protein
bin009	SOY3_bin009_01725	1557	1	8	1	0.077	0.521	0.068	Type IV secretion system protein PtlH
bin009	SOY3_bin009_01726	792	2	1	0	0.302	0.128	0.000	hypothetical protein
bin009	SOY3_bin009_01727	720	1	2	0	0.166	0.282	0.000	SAF domain protein
bin009	SOY3_bin009_01728	930	2	4	0	0.257	0.436	0.000	hypothetical protein
bin009	SOY3_bin009_01729	561	3	3	1	0.639	0.542	0.189	hypothetical protein
bin009	SOY3_bin009_01730	417	0	0	0	0.000	0.000	0.000	hypothetical protein
bin009	SOY3_bin009_01731	1197	0	1	0	0.000	0.085	0.000	Putative prophage phiRv2 integrase
bin009	SOY3_bin009_01732	76	0	0	1	0.000	0.000	1.398	tRNA-Thr(cgt)
bin009	SOY3_bin009_01733	792	2	1	2	0.302	0.128	0.268	Carboxylesterase A precursor
bin009	SOY3_bin009_01734	207	0	0	0	0.000	0.000	0.000	hypothetical protein
bin009	SOY3_bin009_01735	288	0	0	0	0.000	0.000	0.000	hypothetical protein
bin009	SOY3_bin009_01736	2175	1	3	1	0.055	0.140	0.049	hypothetical protein
bin009	SOY3_bin009_01737	420	0	1	0	0.000	0.241	0.000	hypothetical protein
bin009	SOY3_bin009_01738	2265	0	0	1	0.000	0.000	0.047	AAA-like domain protein

bin009	SOY3_bin009_01739	777	0	0	0	0.000	0.000	0.000	von Willebrand factor type A domain protein
bin009	SOY3_bin009_01740	732	0	0	0	0.000	0.000	0.000	hypothetical protein
bin009	SOY3_bin009_01741	816	0	0	0	0.000	0.000	0.000	hypothetical protein
bin009	SOY3_bin009_01742	711	0	0	0	0.000	0.000	0.000	hypothetical protein
bin009	SOY3_bin009_01743	1236	0	0	0	0.000	0.000	0.000	Photosystem I P700 chlorophyll a apoprotein A2
bin009	SOY3_bin009_01744	201	0	0	1	0.000	0.000	0.528	Chaperone protein DnaJ
bin009	SOY3_bin009_01745	1584	0	0	0	0.000	0.000	0.000	hypothetical protein
bin009	SOY3_bin009_01746	1110	1	2	1	0.108	0.183	0.096	CHAT domain protein
bin009	SOY3_bin009_01747	1686	1	1	0	0.071	0.060	0.000	hypothetical protein
bin009	SOY3_bin009_01748	846	0	0	2	0.000	0.000	0.251	GMP synthase [glutamine-hydrolyzing]
bin009	SOY3_bin009_01749	312	3	10	1	1.150	3.251	0.340	Phage shock protein C
bin009	SOY3_bin009_01750	192	0	1	0	0.000	0.528	0.000	hypothetical protein
bin009	SOY3_bin009_01751	1182	20	23	28	2.023	1.974	2.516	DNA-binding transcriptional activator PspC
bin009	SOY3_bin009_01752	1326	3	5	3	0.270	0.382	0.240	Sensor histidine kinase LiaS
bin009	SOY3_bin009_01753	687	1	2	1	0.174	0.295	0.155	Oxygen regulatory protein NreC
bin009	SOY3_bin009_01754	168	1	6	4	0.712	3.622	2.529	hypothetical protein
bin009	SOY3_bin009_01755	2388	0	1	0	0.000	0.042	0.000	ATP-dependent DNA helicase UvrD1
bin009	SOY3_bin009_01756	1221	2	7	6	0.196	0.581	0.522	Succinyl-CoA ligase [ADP-forming] subunit beta
bin009	SOY3_bin009_01757	879	6	4	2	0.816	0.462	0.242	Succinyl-CoA ligase [ADP-forming] subunit alpha
bin009	SOY3_bin009_01758	1386	3	4	4	0.259	0.293	0.307	hypothetical protein
bin009	SOY3_bin009_01759	573	1	1	2	0.209	0.177	0.371	Phosphoribosylglycinamide formyltransferase
bin009	SOY3_bin009_01760	1560	1	5	4	0.077	0.325	0.272	Bifunctional purine biosynthesis protein PurH
bin009	SOY3_bin009_01761	849	0	0	1	0.000	0.000	0.125	Bifunctional protein FolD protein
bin009	SOY3_bin009_01762	348	0	0	0	0.000	0.000	0.000	hypothetical protein
bin009	SOY3_bin009_01763	345	0	0	0	0.000	0.000	0.000	hypothetical protein
bin009	SOY3_bin009_01764	984	19	51	27	2.308	5.257	2.915	Malate dehydrogenase
bin009	SOY3_bin009_01765	825	0	1	0	0.000	0.123	0.000	Bifunctional ligase/repressor BirA
bin009	SOY3_bin009_01766	1572	3	6	2	0.228	0.387	0.135	putative propionyl-CoA carboxylase beta chain 5
bin009	SOY3_bin009_01767	207	1	0	0	0.578	0.000	0.000	hypothetical protein
bin009	SOY3_bin009_01768	669	2	2	1	0.357	0.303	0.159	Septum formation protein Maf
bin009	SOY3_bin009_01769	993	0	0	1	0.000	0.000	0.107	L-asparaginase 1
bin009	SOY3_bin009_01770	1488	1	0	1	0.080	0.000	0.071	Aromatic amino acid transport protein AroP
bin009	SOY3_bin009_01771	492	1	0	0	0.243	0.000	0.000	NADH pyrophosphatase
bin009	SOY3_bin009_01772	1752	1	8	9	0.068	0.463	0.546	2-oxoglutarate carboxylase small subunit
bin009	SOY3_bin009_01773	570	2	2	2	0.419	0.356	0.373	hypothetical protein
bin009	SOY3_bin009_01774	258	9	18	13	4.170	7.076	5.352	Phosphocarrier protein HPr
bin009	SOY3_bin009_01775	204	2	1	0	1.172	0.497	0.000	hypothetical protein
bin009	SOY3_bin009_01776	996	1	2	3	0.120	0.204	0.320	Dihydroorotate dehydrogenase B (NAD(+)), catalytic subunit
bin009	SOY3_bin009_01777	3573	5	8	4	0.167	0.227	0.119	Pyruvate-flavodoxin oxidoreductase
bin009	SOY3_bin009_01778	762	0	0	0	0.000	0.000	0.000	HTH-type transcriptional repressor GlcR
bin009	SOY3_bin009_01779	1218	0	2	1	0.000	0.167	0.087	(Dimethylallyl)adenosine tRNA methylthiotransferase MiaB
bin009	SOY3_bin009_01780	936	0	1	0	0.000	0.108	0.000	tRNA dimethylallyltransferase
bin009	SOY3_bin009_01781	828	0	1	3	0.000	0.122	0.385	Diaminopimelate epimerase
bin009	SOY3_bin009_01782	1482	4	5	4	0.323	0.342	0.287	GTPase HflX
bin009	SOY3_bin009_01783	2013	0	2	3	0.000	0.101	0.158	putative ATP-dependent helicase DinG
bin009	SOY3_bin009_01784	789	0	1	2	0.000	0.129	0.269	LexA repressor
bin009	SOY3_bin009_01785	357	1	2	0	0.335	0.568	0.000	hypothetical protein
bin009	SOY3_bin009_01786	1248	7	20	8	0.671	1.625	0.681	RDD family protein
bin009	SOY3_bin009_01787	459	0	0	0	0.000	0.000	0.000	hypothetical protein
bin009	SOY3_bin009_01788	528	3	4	3	0.679	0.768	0.604	Transcriptional repressor NrdR
bin009	SOY3_bin009_01789	2838	3	12	8	0.126	0.429	0.299	Vitamin B12-dependent ribonucleotide reductase
bin009	SOY3_bin009_01790	978	2	2	4	0.244	0.207	0.434	ABC transporter substrate binding protein
bin009	SOY3_bin009_01791	783	0	0	0	0.000	0.000	0.000	Cobalt transport protein CbiQ
bin009	SOY3_bin009_01792	363	0	0	1	0.000	0.000	0.293	Cobalt transport protein CbiN
bin009	SOY3_bin009_01793	699	0	0	0	0.000	0.000	0.000	Cobalt transport protein CbiM
bin009	SOY3_bin009_01794	657	3	3	4	0.546	0.463	0.647	Precorrin-8X methylmutase
bin009	SOY3_bin009_01795	1296	2	3	2	0.184	0.235	0.164	Inner membrane protein YbiR
bin009	SOY3_bin009_01796	450	2	4	7	0.531	0.902	1.652	CBS domain protein
bin009	SOY3_bin009_01797	819	0	0	0	0.000	0.000	0.000	Cobalt-precorrin-2 C(20)-methyltransferase
bin009	SOY3_bin009_01798	1227	0	1	0	0.000	0.083	0.000	Sirohydrochlorin cobaltochelatase
bin009	SOY3_bin009_01799	1761	0	3	0	0.000	0.173	0.000	Cobalt-precorrin-3B C(17)-methyltransferase
bin009	SOY3_bin009_01800	666	1	2	0	0.180	0.305	0.000	putative cobalt-precorrin-6Y C(5)-methyltransferase
bin009	SOY3_bin009_01801	909	0	1	0	0.000	0.112	0.000	Cobalt-precorrin-4 C(11)-methyltransferase
bin009	SOY3_bin009_01802	762	0	3	2	0.000	0.399	0.279	Precorrin-2 C(20)-methyltransferase
bin009	SOY3_bin009_01803	1038	0	1	0	0.000	0.098	0.000	putative acyl--CoA ligase YhfT
bin009	SOY3_bin009_01804	1140	0	0	1	0.000	0.000	0.093	Putative acetyl-CoA C-acetyltransferase YhfS
bin009	SOY3_bin009_01805	1227	1	2	0	0.097	0.165	0.000	Glutamyl-tRNA reductase

bin009	SOY3_bin009_01806	3489	19	28	18	0.651	0.814	0.548	DNA-directed RNA polymerase subunit beta
bin009	SOY3_bin009_01807	384	3	17	21	0.934	4.490	5.809	50S ribosomal protein L7/L12
bin009	SOY3_bin009_01808	705	8	30	20	1.357	4.316	3.013	50S ribosomal protein L10
bin009	SOY3_bin009_01809	714	6	6	3	1.005	0.852	0.446	50S ribosomal protein L1
bin009	SOY3_bin009_01810	429	0	5	0	0.000	1.182	0.000	50S ribosomal protein L11
bin009	SOY3_bin009_01811	846	2	8	5	0.283	0.959	0.628	hypothetical protein
bin009	SOY3_bin009_01812	492	2	10	1	0.486	2.062	0.216	preprotein translocase subunit SecE
bin009	SOY3_bin009_01813	74	1	1	0	1.616	1.371	0.000	tRNA-Trp(cca)
bin009	SOY3_bin009_01814	1200	0	3	1	0.000	0.254	0.089	Aspartate aminotransferase
bin009	SOY3_bin009_01815	1149	0	6	2	0.000	0.530	0.185	UDP-N-acetylenolpyruvoylglucosamine reductase
bin009	SOY3_bin009_01816	423	0	3	3	0.000	0.719	0.753	bifunctional enoyl-CoA hydratase/phosphate acetyltransferase
bin009	SOY3_bin009_01817	432	0	2	1	0.000	0.470	0.246	bifunctional enoyl-CoA hydratase/phosphate acetyltransferase
bin009	SOY3_bin009_01818	171	9	5	13	6.292	2.966	8.076	50S ribosomal protein L33 2
bin009	SOY3_bin009_01819	75	0	0	1	0.000	0.000	1.416	tRNA-Met(cat)
bin009	SOY3_bin009_01820	75	0	1	0	0.000	1.352	0.000	tRNA-Thr(ggt)
bin009	SOY3_bin009_01821	921	2	3	0	0.260	0.330	0.000	Pseudouridine-5'-phosphate glycosidase
bin009	SOY3_bin009_01822	85	0	1	1	0.000	1.193	1.250	tRNA-Tyr(gta)
bin009	SOY3_bin009_01823	492	2	1	2	0.486	0.206	0.432	putative nucleotide-binding protein
bin009	SOY3_bin009_01824	315	1	2	0	0.380	0.644	0.000	hypothetical protein
bin009	SOY3_bin009_01825	627	21	15	19	4.004	2.426	3.219	Lactate utilization protein C
bin009	SOY3_bin009_01826	741	0	1	0	0.000	0.137	0.000	Ribonuclease PH
bin009	SOY3_bin009_01827	639	0	1	0	0.000	0.159	0.000	Non-canonical purine NTP pyrophosphatase
bin009	SOY3_bin009_01828	816	1	3	0	0.147	0.373	0.000	Thymidylate synthase
bin009	SOY3_bin009_01829	528	2	0	0	0.453	0.000	0.000	Dihydrofolate reductase
bin009	SOY3_bin009_01830	426	2	0	2	0.561	0.000	0.499	(R)-specific enoyl-CoA hydratase
bin009	SOY3_bin009_01831	1362	1	2	2	0.088	0.149	0.156	DNA polymerase IV
bin009	SOY3_bin009_01832	996	4	8	5	0.480	0.815	0.533	Inner membrane ABC transporter permease protein YjfF
bin009	SOY3_bin009_01833	975	1	4	0	0.123	0.416	0.000	D-allose transport system permease protein AlsC
bin009	SOY3_bin009_01834	1542	0	5	8	0.000	0.329	0.551	Ribose import ATP-binding protein RbsA
bin009	SOY3_bin009_01835	1080	4	13	15	0.443	1.221	1.475	D-ribose-binding periplasmic protein precursor
bin009	SOY3_bin009_01836	870	5	4	3	0.687	0.466	0.366	L-arabinolactonase
bin009	SOY3_bin009_01837	1698	15	37	17	1.056	2.210	1.064	Phosphoenolpyruvate-protein phosphotransferase
bin009	SOY3_bin009_01838	1692	10	19	7	0.707	1.139	0.439	PTS system beta-glucoside-specific EIIBCA component
bin009	SOY3_bin009_01839	324	0	1	0	0.000	0.313	0.000	hypothetical protein
bin009	SOY3_bin009_01840	2199	0	2	0	0.000	0.092	0.000	hypothetical protein
bin009	SOY3_bin009_01841	1497	1	5	0	0.080	0.339	0.000	UDP-glucose:undecaprenyl-phosphate glucose-1-phosphate transferase
bin009	SOY3_bin009_01842	1449	2	5	2	0.165	0.350	0.147	Tyrosine-protein kinase YwqD
bin009	SOY3_bin009_01843	1461	3	2	2	0.245	0.139	0.145	UDP-glucose:undecaprenyl-phosphate glucose-1-phosphate transferase
bin009	SOY3_bin009_01844	759	1	2	2	0.158	0.267	0.280	Putative teichuronic acid biosynthesis glycosyltransferase TuaG
bin009	SOY3_bin009_01845	1326	1	2	1	0.090	0.153	0.080	UDP-N-acetyl-D-glucosamine 6-dehydrogenase
bin009	SOY3_bin009_01846	1212	0	2	0	0.000	0.167	0.000	O-Antigen ligase
bin009	SOY3_bin009_01847	975	0	2	0	0.000	0.208	0.000	N-acetylglucosaminyl-diphospho-decaprenol L-rhamnosyltransferase
bin009	SOY3_bin009_01848	654	0	1	0	0.000	0.155	0.000	Chloramphenicol acetyltransferase
bin009	SOY3_bin009_01849	1413	0	1	0	0.000	0.072	0.000	hypothetical protein
bin009	SOY3_bin009_01850	714	1	0	2	0.167	0.000	0.298	putative ATP-dependent helicase Lhr
bin009	SOY3_bin009_01851	264	0	4	4	0.000	1.537	1.609	Transcriptional regulator ClgR
bin009	SOY3_bin009_01852	555	2	0	2	0.431	0.000	0.383	Putative competence-damage inducible protein
bin009	SOY3_bin009_01853	690	2	3	2	0.347	0.441	0.308	Putative CDP-diacylglycerol--glycerol-3-phosphate 3-phosphatidyl-transferase 2
bin009	SOY3_bin009_01854	1434	2	5	2	0.167	0.354	0.148	Ribosomal protein S12 methylthiotransferase RimO
bin009	SOY3_bin009_01855	2385	6	7	11	0.301	0.298	0.490	DNA translocase SpoIIIE
bin009	SOY3_bin009_01856	975	0	1	0	0.000	0.104	0.000	Thiamine-monophosphate kinase
bin009	SOY3_bin009_01857	1119	0	3	1	0.000	0.272	0.095	D-alanine--D-alanine ligase
bin009	SOY3_bin009_01858	789	0	0	0	0.000	0.000	0.000	1-acyl-sn-glycerol-3-phosphate acyltransferase
bin009	SOY3_bin009_01859	606	0	0	1	0.000	0.000	0.175	3-isopropylmalate dehydratase small subunit
bin009	SOY3_bin009_01860	1398	1	8	4	0.086	0.580	0.304	3-isopropylmalate dehydratase large subunit
bin009	SOY3_bin009_01861	726	1	6	3	0.165	0.838	0.439	putative HTH-type transcriptional regulator RhmR
bin009	SOY3_bin009_01862	894	6	16	5	0.802	1.815	0.594	2-hydroxy-3-oxopropionate reductase
bin009	SOY3_bin009_01863	558	1	2	2	0.214	0.364	0.381	Methylated-DNA--protein-cysteine methyltransferase
bin009	SOY3_bin009_01864	960	4	6	1	0.498	0.634	0.111	Trehalose transport system permease protein SugA
bin009	SOY3_bin009_01865	816	2	6	1	0.293	0.746	0.130	Trehalose transport system permease protein SugB
bin009	SOY3_bin009_01866	1089	2	6	1	0.220	0.559	0.098	1,5-anhydro-D-fructose reductase
bin009	SOY3_bin009_01867	1056	1	3	1	0.113	0.288	0.101	L-threonine 3-dehydrogenase
bin009	SOY3_bin009_01868	2811	0	2	4	0.000	0.072	0.151	putative FAD-linked oxidoreductase
bin009	SOY3_bin009_01869	465	0	2	1	0.000	0.436	0.228	HTH-type transcriptional regulator MhqR
bin009	SOY3_bin009_01870	1872	0	7	1	0.000	0.379	0.057	Long-chain-fatty-acid--CoA ligase FadD15
bin009	SOY3_bin009_01871	867	0	1	0	0.000	0.117	0.000	Long-chain acyl-CoA thioesterase FadM
bin009	SOY3_bin009_01872	1443	12	19	7	0.994	1.335	0.515	6-phosphogluconate dehydrogenase, decarboxylating

bin009	SOY3_bin009_01873	1077	0	2	2	0.000	0.188	0.197	HTH-type transcriptional repressor CytR
bin009	SOY3_bin009_01874	186	1	0	0	0.643	0.000	0.000	hypothetical protein
bin009	SOY3_bin009_01875	219	0	0	0	0.000	0.000	0.000	hypothetical protein
bin009	SOY3_bin009_01876	1212	1	7	3	0.099	0.586	0.263	Signal recognition particle receptor FtsY
bin009	SOY3_bin009_01877	1566	2	3	3	0.153	0.194	0.203	Signal recognition particle protein
bin009	SOY3_bin009_01878	1074	0	1	2	0.000	0.094	0.198	phenylhydantoinase
bin009	SOY3_bin009_01879	615	2	4	6	0.389	0.660	1.036	Peroxyureidoacrylate/ureidoacrylate amidohydrolase RutB
bin009	SOY3_bin009_01880	1185	4	9	4	0.404	0.770	0.359	Anthranilate 1,2-dioxygenase system ferredoxin--NAD(+) reductase component
bin009	SOY3_bin009_01881	423	1	8	5	0.283	1.918	1.256	30S ribosomal protein S16
bin009	SOY3_bin009_01882	258	0	2	5	0.000	0.786	2.059	hypothetical protein
bin009	SOY3_bin009_01883	528	1	0	2	0.226	0.000	0.402	Ribosome maturation factor RimM
bin009	SOY3_bin009_01884	711	0	1	1	0.000	0.143	0.149	tRNA (guanine-N(1)-)-methyltransferase
bin009	SOY3_bin009_01885	651	11	27	15	2.020	4.207	2.448	hypothetical protein
bin009	SOY3_bin009_01886	2025	16	71	38	0.945	3.556	1.993	Fumarate reductase flavoprotein subunit
bin009	SOY3_bin009_01887	768	7	33	26	1.090	4.358	3.596	Fumarate reductase iron-sulfur subunit
bin009	SOY3_bin009_01888	348	1	7	6	0.344	2.040	1.831	50S ribosomal protein L19
bin009	SOY3_bin009_01889	819	1	3	3	0.146	0.372	0.389	Signal peptidase I
bin009	SOY3_bin009_01890	432	0	0	0	0.000	0.000	0.000	hypothetical protein
bin009	SOY3_bin009_01891	963	1	2	0	0.124	0.211	0.000	3-(3-hydroxyphenyl)propionate hydroxylase
bin009	SOY3_bin009_01892	801	0	0	0	0.000	0.000	0.000	ABC-2 family transporter protein
bin009	SOY3_bin009_01893	915	0	0	0	0.000	0.000	0.000	Putative osmoprotectant uptake system ATP-binding protein YehX
bin009	SOY3_bin009_01894	924	1	6	0	0.129	0.659	0.000	putative inactive lipase/MT1628
bin009	SOY3_bin009_01895	1371	0	4	1	0.000	0.296	0.077	Putative ABC transporter substrate-binding protein YesO
bin009	SOY3_bin009_01896	951	0	0	0	0.000	0.000	0.000	L-arabinose transport system permease protein AraQ
bin009	SOY3_bin009_01897	924	0	2	0	0.000	0.220	0.000	Inner membrane ABC transporter permease protein YcjO
bin009	SOY3_bin009_01898	2172	1	2	0	0.055	0.093	0.000	Alpha-galactosidase
bin009	SOY3_bin009_01899	1173	1	0	0	0.102	0.000	0.000	N-acetylglucosamine repressor
bin009	SOY3_bin009_01900	894	0	2	0	0.000	0.227	0.000	Aldose 1-epimerase
bin009	SOY3_bin009_01901	429	0	0	0	0.000	0.000	0.000	Ribose operon repressor
bin009	SOY3_bin009_01902	1044	1	1	1	0.115	0.097	0.102	Sensor histidine kinase DesK
bin009	SOY3_bin009_01903	981	1	8	0	0.122	0.827	0.000	Ribose-phosphate pyrophosphokinase
bin009	SOY3_bin009_01904	1734	0	10	6	0.000	0.585	0.368	Long-chain-fatty-acid--CoA ligase
bin009	SOY3_bin009_01905	1605	1	3	1	0.074	0.190	0.066	3-oxosteroid 1-dehydrogenase
bin009	SOY3_bin009_01906	579	0	0	2	0.000	0.000	0.367	HTH-type transcriptional repressor KstR2
bin009	SOY3_bin009_01907	4392	6	8	4	0.163	0.185	0.097	Activator of (R)-2-hydroxyglutaryl-CoA dehydratase
bin009	SOY3_bin009_01908	1350	0	0	0	0.000	0.000	0.000	hypothetical protein
bin009	SOY3_bin009_01909	750	0	0	0	0.000	0.000	0.000	RNA polymerase sigma factor SigA
bin009	SOY3_bin009_01910	1470	7	12	8	0.569	0.828	0.578	ATP-dependent Clp protease ATP-binding subunit ClpC1
bin009	SOY3_bin009_01911	708	0	2	2	0.000	0.287	0.300	nickel/cobalt efflux protein RcnA
bin009	SOY3_bin009_01912	798	0	1	0	0.000	0.127	0.000	ABC-2 family transporter protein
bin009	SOY3_bin009_01913	921	1	0	1	0.130	0.000	0.115	Daunorubicin/doxorubicin resistance ATP-binding protein DrrA
bin009	SOY3_bin009_01914	678	0	1	1	0.000	0.150	0.157	HTH-type transcriptional repressor AcnR
bin009	SOY3_bin009_01915	2409	19	39	11	0.943	1.642	0.485	Fumarate reductase flavoprotein subunit precursor
bin009	SOY3_bin009_01916	564	2	12	7	0.424	2.158	1.318	NADPH azoreductase
bin009	SOY3_bin009_01917	930	3	3	5	0.386	0.327	0.571	Thiamine biosynthesis lipoprotein ApbE precursor
bin009	SOY3_bin009_01918	1944	7	27	31	0.430	1.409	1.694	potassium transport protein Kup
bin009	SOY3_bin009_01919	507	2	8	2	0.472	1.600	0.419	Putative ribosomal N-acetyltransferase YdaF
bin009	SOY3_bin009_01920	774	3	7	2	0.463	0.917	0.274	3-dehydroquinate dehydratase
bin009	SOY3_bin009_01921	75	0	0	0	0.000	0.000	0.000	tRNA-Pro(ggg)
bin009	SOY3_bin009_01922	555	0	0	0	0.000	0.000	0.000	hypothetical protein
bin009	SOY3_bin009_01923	327	0	0	0	0.000	0.000	0.000	putative oxidoreductase
bin009	SOY3_bin009_01924	147	0	0	0	0.000	0.000	0.000	Mannose permease IID component
bin009	SOY3_bin009_01925	1719	0	0	0	0.000	0.000	0.000	Oligo-1,6-glucosidase 1
bin009	SOY3_bin009_01926	444	0	0	0	0.000	0.000	0.000	PTS system mannose-specific EIIAB component
bin009	SOY3_bin009_01927	1428	0	0	0	0.000	0.000	0.000	Bacitracin export ATP-binding protein BceA
bin009	SOY3_bin009_01928	642	0	0	0	0.000	0.000	0.000	Tetracycline repressor protein class A from transposon 1721
bin009	SOY3_bin009_01929	1230	0	1	0	0.000	0.082	0.000	putative chromate transport protein
bin009	SOY3_bin009_01930	1446	0	0	0	0.000	0.000	0.000	Amino-acid carrier protein AlsT
bin009	SOY3_bin009_01931	1110	0	0	0	0.000	0.000	0.000	hypothetical protein
bin009	SOY3_bin009_01932	1266	0	0	0	0.000	0.000	0.000	Putative diacyglycerol O-acyltransferase/MT0231
bin009	SOY3_bin009_01933	729	0	0	1	0.000	0.000	0.146	HTH-type transcriptional repressor DasR
bin009	SOY3_bin009_01934	216	0	0	0	0.000	0.000	0.000	hypothetical protein
bin009	SOY3_bin009_01935	1209	0	1	2	0.000	0.084	0.176	Putative peptidoglycan binding domain protein
bin009	SOY3_bin009_01936	762	0	4	0	0.000	0.532	0.000	hypothetical protein
bin009	SOY3_bin009_01937	873	0	1	0	0.000	0.116	0.000	Putative peptidoglycan binding domain protein
bin009	SOY3_bin009_01938	3270	3	3	2	0.110	0.093	0.065	hypothetical protein
bin009	SOY3_bin009_01939	5466	9	7	2	0.197	0.130	0.039	hypothetical protein

bin009	SOY3_bin009_01940	567	1	0	0	0.211	0.000	0.000	hypothetical protein
bin009	SOY3_bin009_01941	708	2	1	0	0.338	0.143	0.000	Sensor histidine kinase LiaS
bin009	SOY3_bin009_01942	732	0	1	0	0.000	0.139	0.000	Lipoprotein-releasing system ATP-binding protein LolD
bin009	SOY3_bin009_01943	1437	1	0	0	0.083	0.000	0.000	FtsX-like permease family protein
bin009	SOY3_bin009_01944	1320	2	5	4	0.181	0.384	0.322	putative succinyl-diaminopimelate desuccinylase
bin009	SOY3_bin009_01945	978	2	7	3	0.244	0.726	0.326	Uroporphyrinogen decarboxylase (URO-D)
bin009	SOY3_bin009_01946	603	0	4	1	0.000	0.673	0.176	hypothetical protein
bin009	SOY3_bin009_01947	1692	0	1	0	0.000	0.060	0.000	hypothetical protein
bin009	SOY3_bin009_01948	450	1	0	2	0.266	0.000	0.472	hypothetical protein
bin009	SOY3_bin009_01949	627	0	2	0	0.000	0.324	0.000	hypothetical protein
bin009	SOY3_bin009_01950	672	0	0	0	0.000	0.000	0.000	putative tRNA (adenine(37)-N6)-methyltransferase
bin009	SOY3_bin009_01951	642	2	3	0	0.372	0.474	0.000	hypothetical protein
bin009	SOY3_bin009_01952	726	3	3	2	0.494	0.419	0.293	Mycothiol S-conjugate amidase
bin009	SOY3_bin009_01953	900	0	0	1	0.000	0.000	0.118	putative transporter YfdV
bin009	SOY3_bin009_01954	780	0	1	0	0.000	0.130	0.000	Ribonuclease
bin009	SOY3_bin009_01955	429	0	0	0	0.000	0.000	0.000	Macrolide export ATP-binding/permease protein MacB
bin009	SOY3_bin009_01956	753	0	1	1	0.000	0.135	0.141	Lipoprotein-releasing system ATP-binding protein LolD
bin009	SOY3_bin009_01957	441	0	0	0	0.000	0.000	0.000	FMN-binding domain protein
bin009	SOY3_bin009_01958	903	0	0	0	0.000	0.000	0.000	Thiamine biosynthesis lipoprotein ApbE precursor
bin009	SOY3_bin009_01959	426	0	1	0	0.000	0.238	0.000	Thioredoxin
bin009	SOY3_bin009_01960	792	1	5	7	0.151	0.640	0.939	hypothetical protein
bin009	SOY3_bin009_01961	570	1	5	1	0.210	0.890	0.186	hypothetical protein
bin009	SOY3_bin009_01962	993	5	1	1	0.602	0.102	0.107	Pyruvate formate-lyase 1-activating enzyme
bin009	SOY3_bin009_01963	372	4	12	4	1.285	3.272	1.142	FK506-binding protein
bin009	SOY3_bin009_01964	330	1	2	2	0.362	0.615	0.644	methylated-DNA--protein-cysteine methyltransferase
bin009	SOY3_bin009_01965	948	1	1	0	0.126	0.107	0.000	hypothetical protein
bin009	SOY3_bin009_01966	327	0	0	0	0.000	0.000	0.000	Divalent-cation tolerance protein CutA
bin009	SOY3_bin009_01967	837	0	4	0	0.000	0.485	0.000	Serine 3-dehydrogenase
bin009	SOY3_bin009_01968	1344	2	2	1	0.178	0.151	0.079	Replicative DNA helicase
bin009	SOY3_bin009_01969	108	0	0	0	0.000	0.000	0.000	hypothetical protein
bin009	SOY3_bin009_01970	477	1	2	2	0.251	0.425	0.445	hypothetical protein
bin009	SOY3_bin009_01971	339	1	0	1	0.353	0.000	0.313	hypothetical protein
bin009	SOY3_bin009_01972	258	1	0	0	0.463	0.000	0.000	hypothetical protein
bin009	SOY3_bin009_01973	561	0	2	0	0.000	0.362	0.000	hypothetical protein
bin009	SOY3_bin009_01974	639	0	0	0	0.000	0.000	0.000	hypothetical protein
bin009	SOY3_bin009_01975	186	0	0	0	0.000	0.000	0.000	50S ribosomal protein L33 2
bin009	SOY3_bin009_01976	237	0	0	0	0.000	0.000	0.000	50S ribosomal protein L28
bin009	SOY3_bin009_01977	288	0	0	0	0.000	0.000	0.000	50S ribosomal protein L31 type B
bin009	SOY3_bin009_01978	555	0	0	0	0.000	0.000	0.000	Putative metal chaperone YciC
bin009	SOY3_bin009_01979	612	0	0	0	0.000	0.000	0.000	hypothetical protein
bin009	SOY3_bin009_01980	123	0	0	0	0.000	0.000	0.000	50S ribosomal protein L36 2
bin009	SOY3_bin009_01981	1191	1	2	2	0.100	0.170	0.178	Putative isomerase YitF
bin009	SOY3_bin009_01982	76	1	1	0	1.573	1.335	0.000	tRNA-Ile(gat)
bin009	SOY3_bin009_01983	720	1	0	2	0.166	0.000	0.295	flagellar accessory protein FlaH
bin009	SOY3_bin009_01984	387	1	0	1	0.309	0.000	0.274	hypothetical protein
bin009	SOY3_bin009_01985	621	4	15	4	0.770	2.450	0.684	hypothetical protein
bin009	SOY3_bin009_01986	2805	2	1	4	0.085	0.036	0.151	DNA gyrase subunit A
bin009	SOY3_bin009_01987	2034	3	7	6	0.176	0.349	0.313	DNA gyrase subunit B
bin009	SOY3_bin009_01988	867	0	1	0	0.000	0.117	0.000	Polyphosphate kinase 2 (PPK2)
bin009	SOY3_bin009_01989	1839	0	4	0	0.000	0.221	0.000	ATP-dependent DNA helicase RecQ
bin009	SOY3_bin009_01990	669	0	0	0	0.000	0.000	0.000	Phthalate 4,5-dioxygenase oxygenase reductase subunit
bin009	SOY3_bin009_01991	159	0	0	0	0.000	0.000	0.000	hypothetical protein
bin009	SOY3_bin009_01992	609	0	0	0	0.000	0.000	0.000	hypothetical protein
bin009	SOY3_bin009_01993	1854	1	0	0	0.064	0.000	0.000	Prolyl oligopeptidase family protein
bin009	SOY3_bin009_01994	1005	0	0	0	0.000	0.000	0.000	Glyceraldehyde-3-phosphate dehydrogenase
bin009	SOY3_bin009_01995	669	0	0	0	0.000	0.000	0.000	putative HTH-type transcriptional regulator YxaF
bin009	SOY3_bin009_01996	366	1	4	1	0.327	1.108	0.290	Glyoxalase-like domain protein
bin009	SOY3_bin009_01997	1164	1	4	1	0.103	0.349	0.091	N-acetylglucosamine-6-phosphate deacetylase
bin009	SOY3_bin009_01998	786	0	0	0	0.000	0.000	0.000	Glucosamine-6-phosphate deaminase
bin009	SOY3_bin009_01999	939	0	0	0	0.000	0.000	0.000	PTS system glucose-specific EIICBA component
bin009	SOY3_bin009_02000	921	0	3	0	0.000	0.330	0.000	hypothetical protein
bin009	SOY3_bin009_02001	408	1	2	0	0.293	0.497	0.000	hypothetical protein
bin009	SOY3_bin009_02002	696	1	1	0	0.172	0.146	0.000	hypothetical protein
bin009	SOY3_bin009_02003	159	0	0	0	0.000	0.000	0.000	hypothetical protein
bin009	SOY3_bin009_02004	1389	1	0	0	0.086	0.000	0.000	hypothetical protein
bin009	SOY3_bin009_02005	711	0	0	1	0.000	0.000	0.149	hypothetical protein
bin009	SOY3_bin009_02006	372	0	0	0	0.000	0.000	0.000	hypothetical protein

bin009	SOY3_bin009_02007	1527	1	1	1	0.078	0.066	0.070	Divergent AAA domain protein
bin009	SOY3_bin009_02008	498	0	0	0	0.000	0.000	0.000	HNH endonuclease
bin009	SOY3_bin009_02009	906	0	0	0	0.000	0.000	0.000	Transposase, Mutator family
bin009	SOY3_bin009_02010	1221	1	0	0	0.098	0.000	0.000	Tyrosine recombinase XerC
bin009	SOY3_bin009_02011	942	0	0	0	0.000	0.000	0.000	Tyrosine recombinase XerC
bin009	SOY3_bin009_02012	993	0	1	0	0.000	0.102	0.000	Tyrosine recombinase XerD
bin009	SOY3_bin009_02013	519	3	9	2	0.691	1.759	0.409	hypothetical protein
bin009	SOY3_bin009_02014	1284	16	47	27	1.490	3.713	2.234	putative M18 family aminopeptidase 2
bin009	SOY3_bin009_02015	1134	96	323	202	10.121	28.890	18.922	Alcohol dehydrogenase
bin009	SOY3_bin009_02016	1494	91	383	275	7.282	26.002	19.553	Methylmalonate-semialdehyde dehydrogenase [acylating]
bin009	SOY3_bin009_02017	765	1	6	6	0.156	0.796	0.833	HTH-type transcriptional repressor YvoA
bin009	SOY3_bin009_02018	1458	2	0	0	0.164	0.000	0.000	DNA recombination protein RmuC
bin009	SOY3_bin009_02019	978	0	4	0	0.000	0.415	0.000	4-hydroxy-3-methylbut-2-enyl diphosphate reductase
bin009	SOY3_bin009_02020	1236	0	0	0	0.000	0.000	0.000	Exodeoxyribonuclease 7 large subunit
bin009	SOY3_bin009_02021	207	2	0	0	1.155	0.000	0.000	Exodeoxyribonuclease 7 small subunit
bin009	SOY3_bin009_02022	1296	2	4	5	0.184	0.313	0.410	PhoH-like protein
bin009	SOY3_bin009_02023	336	0	0	0	0.000	0.000	0.000	hypothetical protein
bin009	SOY3_bin009_02024	102	1	0	0	1.172	0.000	0.000	hypothetical protein
bin009	SOY3_bin009_02025	324	0	0	0	0.000	0.000	0.000	hypothetical protein
bin009	SOY3_bin009_02026	231	0	0	0	0.000	0.000	0.000	hypothetical protein
bin009	SOY3_bin009_02027	1689	1	2	2	0.071	0.120	0.126	Coenzyme A disulfide reductase
bin009	SOY3_bin009_02028	276	0	0	0	0.000	0.000	0.000	Copper-sensing transcriptional repressor CsoR
bin009	SOY3_bin009_02029	819	0	0	1	0.000	0.000	0.130	2-dehydro-3-deoxygluconokinase
bin009	SOY3_bin009_02030	639	0	1	0	0.000	0.159	0.000	HTH-type transcriptional regulator LuxR
bin009	SOY3_bin009_02031	1248	1	9	7	0.096	0.731	0.596	Neopullulanase
bin009	SOY3_bin009_02032	297	1	7	1	0.403	2.391	0.358	Acetyltransferase (GNAT) family protein
bin009	SOY3_bin009_02033	276	0	0	1	0.000	0.000	0.385	glycogen branching enzyme
bin009	SOY3_bin009_02034	1185	0	4	0	0.000	0.342	0.000	cell division control protein 6
bin009	SOY3_bin009_02035	2247	1	6	2	0.053	0.271	0.095	6-phosphofructokinase
bin009	SOY3_bin009_02036	1800	0	0	0	0.000	0.000	0.000	hypothetical protein
bin009	SOY3_bin009_02037	903	0	0	0	0.000	0.000	0.000	hypothetical protein
bin009	SOY3_bin009_02038	2094	0	1	0	0.000	0.048	0.000	RyR domain protein
bin009	SOY3_bin009_02039	783	0	0	0	0.000	0.000	0.000	hypothetical protein
bin009	SOY3_bin009_02040	489	0	0	0	0.000	0.000	0.000	hypothetical protein
bin009	SOY3_bin009_02041	504	0	0	1	0.000	0.000	0.211	L-2,4-diaminobutyric acid acetyltransferase
bin009	SOY3_bin009_02042	2481	1	2	0	0.048	0.082	0.000	photosystem I assembly protein Ycf3
bin009	SOY3_bin009_02043	198	0	0	0	0.000	0.000	0.000	hypothetical protein
bin009	SOY3_bin009_02044	531	10	29	18	2.251	5.539	3.601	RNA polymerase-binding transcription factor CarD
bin009	SOY3_bin009_02045	453	1	1	6	0.264	0.224	1.407	Putative aminoacrylate peracid reductase RutC
bin009	SOY3_bin009_02046	894	6	2	13	0.802	0.227	1.545	Epimerase family protein
bin009	SOY3_bin009_02047	1698	3	6	3	0.211	0.358	0.188	Glutamine--tRNA ligase
bin009	SOY3_bin009_02048	390	0	0	1	0.000	0.000	0.272	Membrane protein of unknown function
bin009	SOY3_bin009_02049	1266	5	5	1	0.472	0.401	0.084	Telomeric repeat-binding factor 2
bin009	SOY3_bin009_02050	735	2	7	3	0.325	0.966	0.434	hypothetical protein
bin009	SOY3_bin009_02051	1758	0	2	1	0.000	0.115	0.060	reverse gyrase
bin009	SOY3_bin009_02052	1542	4	6	2	0.310	0.395	0.138	Glutathionyl-hydroquinone reductase YqjG
bin009	SOY3_bin009_02053	759	0	1	0	0.000	0.134	0.000	hypothetical protein
bin009	SOY3_bin009_02054	195	0	0	0	0.000	0.000	0.000	hypothetical protein
bin009	SOY3_bin009_02055	123	0	0	0	0.000	0.000	0.000	hypothetical protein
bin009	SOY3_bin009_02056	1014	0	1	0	0.000	0.100	0.000	hypothetical protein
bin009	SOY3_bin009_02057	720	1	0	0	0.166	0.000	0.000	SprT-like family protein
bin009	SOY3_bin009_02058	3117	0	0	0	0.000	0.000	0.000	Type-1 restriction enzyme R protein
bin009	SOY3_bin009_02059	1197	0	0	0	0.000	0.000	0.000	EcoKI restriction-modification system protein HsdS
bin009	SOY3_bin009_02060	2442	1	0	0	0.049	0.000	0.000	Type I restriction enzyme EcoKI M protein
bin009	SOY3_bin009_02061	924	0	0	0	0.000	0.000	0.000	Mrr restriction system protein
bin009	SOY3_bin009_02062	1635	0	5	1	0.000	0.310	0.065	Arginine--tRNA ligase
bin009	SOY3_bin009_02063	774	0	3	2	0.000	0.393	0.274	Polyphosphate glucokinase
bin009	SOY3_bin009_02064	720	1	2	4	0.166	0.282	0.590	putative HTH-type transcriptional regulator YdfH
bin009	SOY3_bin009_02065	1224	0	2	2	0.000	0.166	0.174	CAAX amino terminal protease self- immunity
bin009	SOY3_bin009_02066	1857	20	59	24	1.288	3.223	1.373	Chaperone protein DnaK
bin009	SOY3_bin009_02067	579	4	6	7	0.826	1.051	1.284	heat shock protein GrpE
bin009	SOY3_bin009_02068	1173	7	13	8	0.713	1.124	0.724	Chaperone protein DnaJ
bin009	SOY3_bin009_02069	423	0	2	3	0.000	0.480	0.753	Putative heat shock protein HspR
bin009	SOY3_bin009_02070	558	0	3	0	0.000	0.545	0.000	hypothetical protein
bin009	SOY3_bin009_02071	969	1	3	2	0.123	0.314	0.219	UDP-glucose 4-epimerase
bin009	SOY3_bin009_02072	996	1	2	7	0.120	0.204	0.747	Bacterial membrane flanked domain protein
bin009	SOY3_bin009_02073	77	0	1	0	0.000	1.317	0.000	tRNA-Met(cat)

bin009	SOY3_bin009_02074	960	0	0	0	0.000	0.000	0.000	hypothetical protein
bin009	SOY3_bin009_02075	735	1	1	1	0.163	0.138	0.145	hypothetical protein
bin009	SOY3_bin009_02076	1233	1	0	0	0.097	0.000	0.000	Coenzyme A biosynthesis bifunctional protein CoaBC
bin009	SOY3_bin009_02077	489	0	0	0	0.000	0.000	0.000	Beta-carbonic anhydrase 1
bin009	SOY3_bin009_02078	2451	2	1	0	0.098	0.041	0.000	DNA gyrase subunit A
bin009	SOY3_bin009_02079	1074	0	1	4	0.000	0.094	0.396	hypothetical protein
bin009	SOY3_bin009_02080	576	1	2	1	0.208	0.352	0.184	hypothetical protein
bin009	SOY3_bin009_02081	1155	0	0	3	0.000	0.000	0.276	putative mutase
bin009	SOY3_bin009_02082	1239	2	8	2	0.193	0.655	0.171	hypothetical protein
bin009	SOY3_bin009_02083	297	3	9	8	1.208	3.074	2.861	hypothetical protein
bin009	SOY3_bin009_02084	74	0	0	0	0.000	0.000	0.000	tRNA-Pro(tgg)
bin009	SOY3_bin009_02085	555	0	0	0	0.000	0.000	0.000	Putative ribosomal N-acetyltransferase YdaF
bin009	SOY3_bin009_02086	921	0	0	0	0.000	0.000	0.000	Tyrosine recombinase XerD
bin009	SOY3_bin009_02087	714	0	0	0	0.000	0.000	0.000	hypothetical protein
bin009	SOY3_bin009_02088	645	0	0	0	0.000	0.000	0.000	Helix-turn-helix
bin009	SOY3_bin009_02089	2370	1	0	1	0.050	0.000	0.045	excinuclease ABC subunit B
bin009	SOY3_bin009_02090	453	0	3	2	0.000	0.672	0.469	hypothetical protein
bin009	SOY3_bin009_02091	471	0	2	1	0.000	0.431	0.226	RNA polymerase sigma factor SigF
bin009	SOY3_bin009_02092	546	0	0	0	0.000	0.000	0.000	CAAX amino terminal protease self- immunity
bin009	SOY3_bin009_02093	1023	0	0	0	0.000	0.000	0.000	Ribose operon repressor
bin009	SOY3_bin009_02094	1593	0	0	1	0.000	0.000	0.067	Inner membrane symporter YicJ
bin009	SOY3_bin009_02095	228	0	1	0	0.000	0.445	0.000	hypothetical protein
bin009	SOY3_bin009_02096	711	0	1	1	0.000	0.143	0.149	hypothetical protein
bin009	SOY3_bin009_02097	228	0	0	0	0.000	0.000	0.000	Helix-turn-helix
bin009	SOY3_bin009_02098	234	0	0	0	0.000	0.000	0.000	hypothetical protein
bin009	SOY3_bin009_02099	300	0	0	0	0.000	0.000	0.000	hypothetical protein
bin009	SOY3_bin009_02100	495	0	1	0	0.000	0.205	0.000	Barstar (barnase inhibitor)
bin009	SOY3_bin009_02101	579	0	0	0	0.000	0.000	0.000	hypothetical protein
bin009	SOY3_bin009_02102	678	0	0	0	0.000	0.000	0.000	hypothetical protein
bin009	SOY3_bin009_02103	513	0	0	1	0.000	0.000	0.207	Barstar (barnase inhibitor)
bin009	SOY3_bin009_02104	504	0	3	2	0.000	0.604	0.422	Barstar (barnase inhibitor)
bin009	SOY3_bin009_02105	285	0	0	0	0.000	0.000	0.000	hypothetical protein
bin009	SOY3_bin009_02106	612	0	0	0	0.000	0.000	0.000	hypothetical protein
bin009	SOY3_bin009_02107	540	4	3	2	0.886	0.563	0.393	Endonuclease V
bin009	SOY3_bin009_02108	486	1	0	0	0.246	0.000	0.000	hypothetical protein
bin009	SOY3_bin009_02109	183	5	15	5	3.266	8.314	2.902	hypothetical protein
bin009	SOY3_bin009_02110	681	16	59	31	2.809	8.787	4.836	hypothetical protein
bin009	SOY3_bin009_02111	738	2	1	0	0.324	0.137	0.000	HTH-type transcriptional regulator LutR
bin009	SOY3_bin009_02112	1686	1	3	5	0.071	0.180	0.315	putative ABC transporter ATP-binding protein/MT2552
bin009	SOY3_bin009_02113	426	0	0	0	0.000	0.000	0.000	Single-stranded DNA-binding protein 1
bin009	SOY3_bin009_02114	1635	2	3	1	0.146	0.186	0.065	GTPase Era
bin009	SOY3_bin009_02115	1854	3	10	3	0.193	0.547	0.172	GTPase RsgA
bin009	SOY3_bin009_02116	1563	1	8	3	0.076	0.519	0.204	hypothetical protein
bin009	SOY3_bin009_02117	75	0	0	0	0.000	0.000	0.000	tRNA-Arg(tct)
bin009	SOY3_bin009_02118	621	0	1	1	0.000	0.163	0.171	Oligoribonuclease
bin009	SOY3_bin009_02119	417	2	0	1	0.573	0.000	0.255	putative cation-transporting ATPase F
bin009	SOY3_bin009_02120	1380	3	2	1	0.260	0.147	0.077	hypothetical protein
bin009	SOY3_bin009_02121	1800	2	3	2	0.133	0.169	0.118	ATP-dependent RNA helicase DeaD
bin009	SOY3_bin009_02122	321	0	2	0	0.000	0.632	0.000	HTH-type transcriptional regulator
bin009	SOY3_bin009_02123	978	0	6	0	0.000	0.622	0.000	hypothetical protein
bin009	SOY3_bin009_02124	705	1	2	1	0.170	0.288	0.151	Chlorite dismutase precursor
bin009	SOY3_bin009_02125	840	1	2	2	0.142	0.241	0.253	Flavin mononucleotide phosphatase YbjI
bin009	SOY3_bin009_02126	393	0	0	1	0.000	0.000	0.270	Ribonuclease VapC46
bin009	SOY3_bin009_02127	273	0	2	0	0.000	0.743	0.000	Antitoxin VapB46
bin009	SOY3_bin009_02128	651	3	4	4	0.551	0.623	0.653	Putative osmoprotectant uptake system permease protein YehW
bin009	SOY3_bin009_02129	1185	2	6	1	0.202	0.514	0.090	Glycine betaine/carnitine/choline transport ATP-binding protein OpuCA
bin009	SOY3_bin009_02130	786	3	3	4	0.456	0.387	0.541	Carnitine transport permease protein OpuCD
bin009	SOY3_bin009_02131	189	0	0	0	0.000	0.000	0.000	hypothetical protein
bin009	SOY3_bin009_02132	666	14	26	23	2.513	3.960	3.668	hypothetical protein
bin009	SOY3_bin009_02133	1074	4	11	11	0.445	1.039	1.088	hypothetical protein
bin009	SOY3_bin009_02134	645	0	1	0	0.000	0.157	0.000	Glutamine transport ATP-binding protein GlnQ
bin009	SOY3_bin009_02135	1020	1	2	1	0.117	0.199	0.104	hypothetical protein
bin009	SOY3_bin009_02136	465	0	1	2	0.000	0.218	0.457	Putative sugar phosphate isomerase YwlF
bin009	SOY3_bin009_02137	420	0	0	2	0.000	0.000	0.506	Nucleoside diphosphate kinase
bin009	SOY3_bin009_02138	1290	2	0	1	0.185	0.000	0.082	CAAX amino terminal protease self- immunity
bin009	SOY3_bin009_02139	1953	0	0	1	0.000	0.000	0.054	(Dimethylallyl)adenosine tRNA methylthiotransferase MiaB
bin009	SOY3_bin009_02140	1239	0	2	1	0.000	0.164	0.086	putative transporter YycB

bin009	SOY3_bin009_02141	666	0	0	0	0.000	0.000	0.000	Diaminopimelate epimerase
bin009	SOY3_bin009_02142	1017	0	0	0	0.000	0.000	0.000	CAAX amino terminal protease self- immunity
bin009	SOY3_bin009_02143	1008	0	1	0	0.000	0.101	0.000	hypothetical protein
bin009	SOY3_bin009_02144	357	0	0	0	0.000	0.000	0.000	HTH-type transcriptional repressor YtrA
bin009	SOY3_bin009_02145	1071	0	0	0	0.000	0.000	0.000	putative transporter
bin009	SOY3_bin009_02146	993	0	0	0	0.000	0.000	0.000	Lactose operon repressor
bin009	SOY3_bin009_02147	1176	6	10	1	0.610	0.862	0.090	Multiple sugar-binding periplasmic receptor ChvE precursor
bin009	SOY3_bin009_02148	1545	1	1	0	0.077	0.066	0.000	Xylose import ATP-binding protein XylG
bin009	SOY3_bin009_02149	1176	1	1	1	0.102	0.086	0.090	Xylose transport system permease protein XylH
bin009	SOY3_bin009_02150	1308	1	0	1	0.091	0.000	0.081	Transposase
bin009	SOY3_bin009_02151	291	0	0	0	0.000	0.000	0.000	hypothetical protein
bin009	SOY3_bin009_02152	904	913	1025	854	120.739	115.003	100.350	16S ribosomal RNA
bin009	SOY3_bin009_02153	918	1	0	0	0.130	0.000	0.000	Tyrosine recombinase XerD
bin009	SOY3_bin009_02154	663	1	0	0	0.180	0.000	0.000	ADP-ribose pyrophosphatase
bin009	SOY3_bin009_02155	1653	1	3	3	0.072	0.184	0.193	CTP synthase
bin009	SOY3_bin009_02156	1053	1	1	0	0.114	0.096	0.000	Putative DNA ligase-like protein/MT0965
bin009	SOY3_bin009_02157	336	0	1	0	0.000	0.302	0.000	HTH-type transcriptional regulator KmtR
bin009	SOY3_bin009_02158	1266	2	0	0	0.189	0.000	0.000	Major Facilitator Superfamily protein
bin009	SOY3_bin009_02159	2139	0	5	1	0.000	0.237	0.050	Putative multidrug export ATP-binding/permease protein
bin009	SOY3_bin009_02160	408	0	0	0	0.000	0.000	0.000	hypothetical protein
bin009	SOY3_bin009_02161	1053	3	4	5	0.341	0.385	0.504	Anthranilate phosphoribosyltransferase
bin009	SOY3_bin009_02162	717	0	0	1	0.000	0.000	0.148	Type IV leader peptidase family protein
bin009	SOY3_bin009_02163	555	0	1	0	0.000	0.183	0.000	hypothetical protein
bin009	SOY3_bin009_02164	921	48	56	24	6.231	6.167	2.768	putative membrane protein ArfC
bin009	SOY3_bin009_02165	510	2	11	4	0.469	2.188	0.833	Peptidyl-prolyl cis-trans isomerase B
bin009	SOY3_bin009_02166	282	1	2	1	0.424	0.719	0.377	Leucine-responsive regulatory protein
bin009	SOY3_bin009_02167	1746	0	1	0	0.000	0.058	0.000	DNA polymerase III PolC-type
bin009	SOY3_bin009_02168	1071	2	10	1	0.223	0.947	0.099	Murein DD-endopeptidase MepM
bin009	SOY3_bin009_02169	207	0	1	1	0.000	0.490	0.513	hypothetical protein
bin009	SOY3_bin009_02170	1113	0	0	0	0.000	0.000	0.000	hypothetical protein
bin009	SOY3_bin009_02171	450	2	6	3	0.531	1.352	0.708	hypothetical protein
bin009	SOY3_bin009_02172	225	0	2	2	0.000	0.902	0.944	hypothetical protein
bin009	SOY3_bin009_02173	645	0	0	0	0.000	0.000	0.000	RNA polymerase sigma factor SigA
bin009	SOY3_bin009_02174	504	2	0	2	0.474	0.000	0.422	hypothetical protein
bin009	SOY3_bin009_02175	1095	0	1	2	0.000	0.093	0.194	NADPH dehydrogenase
bin009	SOY3_bin009_02176	76	1	0	0	1.573	0.000	0.000	tRNA-Asn(gtt)
bin009	SOY3_bin009_02177	504	0	0	1	0.000	0.000	0.211	hypothetical protein
bin009	SOY3_bin009_02178	300	0	0	0	0.000	0.000	0.000	hypothetical protein
bin009	SOY3_bin009_02179	333	0	0	0	0.000	0.000	0.000	hypothetical protein
bin009	SOY3_bin009_02180	882	0	0	0	0.000	0.000	0.000	Phage capsid family protein
bin009	SOY3_bin009_02181	165	0	0	0	0.000	0.000	0.000	hypothetical protein
bin009	SOY3_bin009_02182	462	0	0	0	0.000	0.000	0.000	hypothetical protein
bin009	SOY3_bin009_02183	1287	0	0	0	0.000	0.000	0.000	Phage portal protein, SPP1 Gp6-like
bin009	SOY3_bin009_02184	1464	0	0	0	0.000	0.000	0.000	Phage Terminase
bin009	SOY3_bin009_02185	1149	0	2	0	0.000	0.177	0.000	hypothetical protein
bin009	SOY3_bin009_02186	1482	2	0	0	0.161	0.000	0.000	Acetyltransferase (GNAT) family protein
bin009	SOY3_bin009_02187	471	0	0	0	0.000	0.000	0.000	50S ribosomal protein L22/unknown domain fusion protein
bin009	SOY3_bin009_02188	240	0	0	0	0.000	0.000	0.000	hypothetical protein
bin009	SOY3_bin009_02189	588	1	1	0	0.203	0.172	0.000	hypothetical protein
bin009	SOY3_bin009_02190	2706	5	2	0	0.221	0.075	0.000	Deoxyguanosinetriphosphate triphosphohydrolase-like protein
bin009	SOY3_bin009_02191	414	1	1	0	0.289	0.245	0.000	hypothetical protein
bin009	SOY3_bin009_02192	915	0	0	0	0.000	0.000	0.000	Pyrimidine-specific ribonucleoside hydrolase RihA
bin009	SOY3_bin009_02193	351	0	0	0	0.000	0.000	0.000	Transposase, Mutator family
bin009	SOY3_bin009_02194	384	0	0	0	0.000	0.000	0.000	Transposase, Mutator family
bin009	SOY3_bin009_02195	753	0	0	2	0.000	0.000	0.282	hypothetical protein
bin009	SOY3_bin009_02196	864	1	1	3	0.138	0.117	0.369	putative formamidopyrimidine-DNA glycosylase-like protein
bin009	SOY3_bin009_02197	1257	0	0	0	0.000	0.000	0.000	hypothetical protein
bin009	SOY3_bin009_02198	1431	0	1	2	0.000	0.071	0.148	hypothetical protein
bin009	SOY3_bin009_02199	705	0	1	0	0.000	0.144	0.000	Leucyl/phenylalanyl-tRNA--protein transferase
bin009	SOY3_bin009_02200	1287	0	0	3	0.000	0.000	0.248	Citrate synthase 1
bin009	SOY3_bin009_02201	690	2	4	0	0.347	0.588	0.000	arginine repressor
bin009	SOY3_bin009_02202	1152	0	1	1	0.000	0.088	0.092	Alanine racemase
bin009	SOY3_bin009_02203	1155	0	0	0	0.000	0.000	0.000	Dihydrolipoyllysine-residue acetyltransferase component of pyruvate dehydrogenase complex
bin009	SOY3_bin009_02204	1434	2	7	4	0.167	0.495	0.296	Lactaldehyde dehydrogenase
bin009	SOY3_bin009_02205	1707	0	0	1	0.000	0.000	0.062	hypothetical protein
bin009	SOY3_bin009_02206	1701	1	0	1	0.070	0.000	0.062	Outer membrane protein assembly factor BamB
bin009	SOY3_bin009_02207	1110	6	24	8	0.646	2.193	0.766	Putative alcohol dehydrogenase D

bin009	SOY3_bin009_02208	420	6	25	5	1.708	6.037	1.265	Zinc ribbon domain protein
bin009	SOY3_bin009_02209	813	15	56	22	2.206	6.986	2.874	N-acyl homoserine lactonase
bin009	SOY3_bin009_02210	996	6	20	13	0.720	2.037	1.386	PTS-dependent dihydroxyacetone kinase, dihydroxyacetone-binding subunit DhaK
bin009	SOY3_bin009_02211	636	7	14	14	1.316	2.233	2.338	PTS-dependent dihydroxyacetone kinase, ADP-binding subunit DhaL
bin009	SOY3_bin009_02212	528	2	0	0	0.453	0.000	0.000	O-acetyl-ADP-ribose deacetylase
bin009	SOY3_bin009_02213	1620	163	438	263	12.029	27.423	17.245	Acetate CoA-transferase YdiF
bin009	SOY3_bin009_02214	420	45	109	51	12.809	26.323	12.899	(R)-specific enoyl-CoA hydratase
bin009	SOY3_bin009_02215	1137	106	266	152	11.145	23.729	14.201	Acyl-CoA dehydrogenase, short-chain specific
bin009	SOY3_bin009_02216	768	59	153	80	9.184	20.206	11.065	Electron transfer flavoprotein subunit beta
bin009	SOY3_bin009_02217	909	63	156	72	8.286	17.407	8.414	Acryloyl-CoA reductase electron transfer subunit beta
bin009	SOY3_bin009_02218	1305	71	211	129	6.504	16.399	10.500	Electron transfer flavoprotein-ubiquinone oxidoreductase
bin009	SOY3_bin009_02219	288	28	67	32	11.623	23.596	11.803	hypothetical protein
bin009	SOY3_bin009_02220	780	90	224	134	13.794	29.128	18.249	putative enoyl-CoA hydratase echA8
bin009	SOY3_bin009_02221	186	5	13	7	3.214	7.089	3.998	50S ribosomal protein L28
bin009	SOY3_bin009_02222	816	0	3	1	0.000	0.373	0.130	hypothetical protein
bin009	SOY3_bin009_02223	1257	0	0	0	0.000	0.000	0.000	Integrase core domain protein
bin009	SOY3_bin009_02224	76	0	0	0	0.000	0.000	0.000	tRNA-Arg(acg)
bin009	SOY3_bin009_02225	294	0	0	0	0.000	0.000	0.000	putative periplasmic iron-binding protein precursor
bin009	SOY3_bin009_02226	354	0	0	0	0.000	0.000	0.000	Periplasmic solute binding protein family protein
bin009	SOY3_bin009_02227	657	0	0	0	0.000	0.000	0.000	High-affinity zinc uptake system ATP-binding protein ZnuC
bin009	SOY3_bin009_02228	849	0	1	0	0.000	0.119	0.000	High-affinity zinc uptake system membrane protein ZnuB
bin009	SOY3_bin009_02229	762	0	0	0	0.000	0.000	0.000	Vitamin B12-binding protein precursor
bin009	SOY3_bin009_02230	483	0	0	1	0.000	0.000	0.220	hypothetical protein
bin009	SOY3_bin009_02231	840	0	0	1	0.000	0.000	0.126	Shikimate dehydrogenase
bin009	SOY3_bin009_02232	912	3	1	1	0.393	0.111	0.116	1,4-Dihydroxy-2-naphthoyl-CoA synthase
bin009	SOY3_bin009_02233	1428	2	2	5	0.167	0.142	0.372	hypothetical protein
bin009	SOY3_bin009_02234	495	2	1	0	0.483	0.205	0.000	hypothetical protein
bin009	SOY3_bin009_02235	1089	0	0	0	0.000	0.000	0.000	2-oxoglutarate oxidoreductase subunit KorB
bin009	SOY3_bin009_02236	1848	0	1	1	0.000	0.055	0.057	2-oxoglutarate oxidoreductase subunit KorA
bin009	SOY3_bin009_02237	1545	0	1	0	0.000	0.066	0.000	hypothetical protein
bin009	SOY3_bin009_02238	951	3	0	2	0.377	0.000	0.223	1,2-epoxyphenylacetyl-CoA isomerase
bin009	SOY3_bin009_02239	906	0	2	0	0.000	0.224	0.000	Exodeoxyribonuclease
bin009	SOY3_bin009_02240	375	0	0	0	0.000	0.000	0.000	hypothetical protein
bin009	SOY3_bin009_02241	222	0	0	0	0.000	0.000	0.000	ribosome-associated protein
bin009	SOY3_bin009_02242	969	0	1	1	0.000	0.105	0.110	hypothetical protein
bin009	SOY3_bin009_02243	624	0	0	0	0.000	0.000	0.000	hypothetical protein
bin009	SOY3_bin009_02244	210	0	0	0	0.000	0.000	0.000	hypothetical protein
bin009	SOY3_bin009_02245	3123	3	6	1	0.115	0.195	0.034	Type I restriction enzyme EcoR124II R protein
bin009	SOY3_bin009_02246	408	2	1	0	0.586	0.249	0.000	Ribonuclease VapC47
bin009	SOY3_bin009_02247	255	0	5	0	0.000	1.989	0.000	Antitoxin VapB47
bin009	SOY3_bin009_02248	951	0	1	0	0.000	0.107	0.000	hypothetical protein
bin009	SOY3_bin009_02249	1260	0	5	1	0.000	0.402	0.084	Putative type-1 restriction enzyme specificity protein MPN_089
bin009	SOY3_bin009_02250	1566	0	2	1	0.000	0.130	0.068	putative type I restriction enzymeP M protein
bin009	SOY3_bin009_02251	558	0	3	3	0.000	0.545	0.571	Low molecular weight protein-tyrosine-phosphatase etp
bin009	SOY3_bin009_02252	453	0	1	0	0.000	0.224	0.000	hypothetical protein
bin009	SOY3_bin009_02253	843	2	0	0	0.284	0.000	0.000	hypothetical protein
bin009	SOY3_bin009_02254	1458	0	0	1	0.000	0.000	0.073	Putative pyrimidine permease RutG
bin009	SOY3_bin009_02255	1170	0	1	0	0.000	0.087	0.000	hypothetical protein
bin009	SOY3_bin009_02256	1656	0	0	1	0.000	0.000	0.064	putative dolichyl-phosphate-mannose--protein mannosyltransferase
bin009	SOY3_bin009_02257	855	0	0	0	0.000	0.000	0.000	Ribosomal RNA small subunit methyltransferase I
bin009	SOY3_bin009_02258	888	0	1	2	0.000	0.114	0.239	putative deoxyribonuclease YcfH
bin009	SOY3_bin009_02259	507	0	0	0	0.000	0.000	0.000	putative transporter
bin009	SOY3_bin009_02260	495	0	0	0	0.000	0.000	0.000	Putative competence-damage inducible protein
bin009	SOY3_bin009_02261	804	1	0	1	0.149	0.000	0.132	Heat shock protein HslJ
bin009	SOY3_bin009_02262	2598	0	0	0	0.000	0.000	0.000	Calcium-transporting ATPase
bin009	SOY3_bin009_02263	186	0	1	0	0.000	0.545	0.000	Glutaminase 1
bin009	SOY3_bin009_02264	504	0	0	0	0.000	0.000	0.000	Glutaminase 1
bin009	SOY3_bin009_02265	2679	0	1	0	0.000	0.038	0.000	Serine/threonine-protein kinase PknK
bin009	SOY3_bin009_02266	453	0	0	0	0.000	0.000	0.000	hypothetical protein
bin009	SOY3_bin009_02267	1269	0	8	3	0.000	0.639	0.251	Tyrosine--tRNA ligase
bin009	SOY3_bin009_02268	1353	1	2	2	0.088	0.150	0.157	Argininosuccinate lyase
bin009	SOY3_bin009_02269	534	0	0	0	0.000	0.000	0.000	Arginine repressor
bin009	SOY3_bin009_02270	1197	0	3	1	0.000	0.254	0.089	Acetylornithine aminotransferase
bin009	SOY3_bin009_02271	912	0	2	2	0.000	0.222	0.233	Acetylglutamate kinase
bin009	SOY3_bin009_02272	1152	1	2	3	0.104	0.176	0.277	Arginine biosynthesis bifunctional protein ArgJ
bin009	SOY3_bin009_02273	1035	0	1	1	0.000	0.098	0.103	N-acetyl-gamma-glutamyl-phosphate reductase
bin009	SOY3_bin009_02274	873	2	1	2	0.274	0.116	0.243	NAD synthetase

bin009	SOY3_bin009_02275	474	1	0	0	0.252	0.000	0.000	Tetracycline repressor protein class E
bin009	SOY3_bin009_02276	726	0	2	1	0.000	0.279	0.146	2-C-methyl-D-erythritol 4-phosphate cytidylyltransferase
bin009	SOY3_bin009_02277	753	3	6	3	0.476	0.808	0.423	triosephosphate isomerase
bin009	SOY3_bin009_02278	372	3	2	0	0.964	0.545	0.000	PTS system glucitol/sorbitol-specific transporter subunit IIA
bin009	SOY3_bin009_02279	966	3	0	0	0.371	0.000	0.000	Sulfate/thiosulfate import ATP-binding protein CysA
bin009	SOY3_bin009_02280	810	0	0	0	0.000	0.000	0.000	Sulfate transport system permease protein CysW
bin009	SOY3_bin009_02281	894	1	0	0	0.134	0.000	0.000	Sulfate transport system permease protein CysT
bin009	SOY3_bin009_02282	1041	0	0	0	0.000	0.000	0.000	Sulfate-binding protein precursor
bin009	SOY3_bin009_02283	1680	1	3	5	0.071	0.181	0.316	Sulfite reductase [ferredoxin]
bin009	SOY3_bin009_02284	720	0	0	0	0.000	0.000	0.000	Phosphoadenosine phosphosulfate reductase
bin009	SOY3_bin009_02285	636	0	0	0	0.000	0.000	0.000	hypothetical protein
bin009	SOY3_bin009_02286	276	0	1	0	0.000	0.367	0.000	Antitoxin HigA-1
bin009	SOY3_bin009_02287	1020	0	0	1	0.000	0.000	0.104	hypothetical protein
bin009	SOY3_bin009_02288	1983	1	4	1	0.060	0.205	0.054	putative peptidoglycan biosynthesis protein MviN
bin009	SOY3_bin009_02289	1290	7	2	3	0.649	0.157	0.247	Aspartokinase
bin009	SOY3_bin009_02290	696	8	2	4	1.374	0.291	0.610	putative Mg(2+) transport ATPase
bin009	SOY3_bin009_02291	567	3	2	2	0.633	0.358	0.375	General stress protein 18
bin009	SOY3_bin009_02292	549	2	3	3	0.436	0.554	0.580	hypothetical protein
bin009	SOY3_bin009_02293	1023	3	13	4	0.351	1.289	0.415	Phosphoribosylformylglycinamidine synthase 2
bin009	SOY3_bin009_02294	999	2	7	4	0.239	0.711	0.425	Autoinducer 2 import system permease protein LsrC
bin009	SOY3_bin009_02295	498	16	13	22	3.841	2.648	4.693	hypothetical protein
bin009	SOY3_bin009_02296	360	1	0	0	0.332	0.000	0.000	hypothetical protein
bin009	SOY3_bin009_02297	951	0	0	0	0.000	0.000	0.000	hypothetical protein
bin009	SOY3_bin009_02298	834	1	0	0	0.143	0.000	0.000	3-mercaptopyruvate sulfurtransferase
bin009	SOY3_bin009_02299	1452	3	3	1	0.247	0.210	0.073	Tyrosine phenol-lyase
bin009	SOY3_bin009_02300	1056	0	0	0	0.000	0.000	0.000	Tyrosine recombinase XerC
bin009	SOY3_bin009_02301	429	0	0	0	0.000	0.000	0.000	hypothetical protein
bin009	SOY3_bin009_02302	678	1	3	1	0.176	0.449	0.157	Transposase DDE domain protein
bin009	SOY3_bin009_02303	76	0	0	0	0.000	0.000	0.000	tRNA-Glu(ttc)
bin009	SOY3_bin009_02304	543	0	0	0	0.000	0.000	0.000	hypothetical protein
bin009	SOY3_bin009_02305	966	0	3	1	0.000	0.315	0.110	Carboxylesterase NlhH
bin009	SOY3_bin009_02306	807	4	9	8	0.593	1.131	1.053	Levodione reductase
bin009	SOY3_bin009_02307	714	4	2	4	0.670	0.284	0.595	Helix-turn-helix domain protein
bin009	SOY3_bin009_02308	306	1	1	0	0.391	0.331	0.000	hypothetical protein
bin009	SOY3_bin009_02309	351	3	1	0	1.022	0.289	0.000	hypothetical protein
bin009	SOY3_bin009_02310	1191	0	1	1	0.000	0.085	0.089	Aspartate aminotransferase
bin009	SOY3_bin009_02311	495	7	18	10	1.691	3.688	2.146	Cytochrome c-type protein NrfH
bin009	SOY3_bin009_02312	1476	10	24	17	0.810	1.649	1.223	Cytochrome c-552 precursor
bin009	SOY3_bin009_02313	900	4	15	2	0.531	1.690	0.236	lipoprotein NlpI
bin009	SOY3_bin009_02314	576	1	0	0	0.208	0.000	0.000	L-threonine 3-dehydrogenase
bin009	SOY3_bin009_02315	882	1	3	0	0.136	0.345	0.000	Transketolase
bin009	SOY3_bin009_02316	957	0	0	0	0.000	0.000	0.000	1-deoxy-D-xylulose-5-phosphate synthase
bin009	SOY3_bin009_02317	861	0	1	0	0.000	0.118	0.000	D-tagatose 3-epimerase
bin009	SOY3_bin009_02318	786	1	0	1	0.152	0.000	0.135	2-dehydro-3-deoxy-D-gluconate 5-dehydrogenase
bin009	SOY3_bin009_02319	74	0	0	1	0.000	0.000	1.435	tRNA-Gly(tcc)
bin009	SOY3_bin009_02320	1218	0	3	0	0.000	0.250	0.000	Nuclease SbcCD subunit D
bin009	SOY3_bin009_02321	2979	1	2	0	0.040	0.068	0.000	Nuclease SbcCD subunit C
bin009	SOY3_bin009_02322	237	0	1	1	0.000	0.428	0.448	hypothetical protein
bin009	SOY3_bin009_02323	144	0	0	1	0.000	0.000	0.738	Flavin containing amine oxidoreductase
bin009	SOY3_bin009_02324	339	0	1	1	0.000	0.299	0.313	Nitrogen regulatory protein P-II
bin009	SOY3_bin009_02325	1347	1	3	0	0.089	0.226	0.000	Ammonia channel
bin009	SOY3_bin009_02326	1014	1	4	2	0.118	0.400	0.210	hypothetical protein
bin009	SOY3_bin009_02327	1113	3	5	2	0.322	0.456	0.191	hypothetical protein
bin009	SOY3_bin009_02328	519	0	1	1	0.000	0.195	0.205	phosphohistidine phosphatase
bin009	SOY3_bin009_02329	1068	0	1	1	0.000	0.095	0.099	DNA methylase
bin009	SOY3_bin009_02330	225	0	1	1	0.000	0.451	0.472	ferrous iron transport protein A
bin009	SOY3_bin009_02331	1941	0	3	1	0.000	0.157	0.055	Ferrous iron transport protein B
bin009	SOY3_bin009_02332	588	1	1	0	0.203	0.172	0.000	Fe/S biogenesis protein NfuA
bin009	SOY3_bin009_02333	735	2	0	0	0.325	0.000	0.000	4-formylbenzenesulfonate dehydrogenase TsaC1/TsaC2
bin009	SOY3_bin009_02334	1002	0	0	1	0.000	0.000	0.106	Alcohol dehydrogenase
bin009	SOY3_bin009_02335	723	0	0	0	0.000	0.000	0.000	Bicarbonate transport ATP-binding protein CmpD
bin009	SOY3_bin009_02336	429	0	0	0	0.000	0.000	0.000	NPCBM/NEW2 domain protein
bin009	SOY3_bin009_02337	1461	2	1	1	0.164	0.069	0.073	hypothetical protein
bin009	SOY3_bin009_02338	666	0	1	1	0.000	0.152	0.159	hypothetical protein
bin009	SOY3_bin009_02339	1059	1	2	7	0.113	0.192	0.702	hypothetical protein
bin009	SOY3_bin009_02340	822	0	2	4	0.000	0.247	0.517	hypothetical protein
bin009	SOY3_bin009_02341	504	0	0	0	0.000	0.000	0.000	Barstar (barnase inhibitor)

bin009	SOY3_bin009_02342	609	0	0	0	0.000	0.000	0.000	hypothetical protein
bin009	SOY3_bin009_02343	747	0	0	0	0.000	0.000	0.000	transposase
bin009	SOY3_bin009_02344	1566	0	0	0	0.000	0.000	0.000	Integrase core domain protein
bin009	SOY3_bin009_02345	327	0	0	0	0.000	0.000	0.000	hypothetical protein
bin009	SOY3_bin009_02346	867	0	0	0	0.000	0.000	0.000	Leucine Rich repeats (2 copies)
bin009	SOY3_bin009_02347	180	0	0	0	0.000	0.000	0.000	hypothetical protein
bin009	SOY3_bin009_02348	177	0	0	0	0.000	0.000	0.000	hypothetical protein
bin009	SOY3_bin009_02349	1311	0	1	0	0.000	0.077	0.000	Transposase, Mutator family
bin009	SOY3_bin009_02350	744	0	4	2	0.000	0.545	0.286	Ribonuclease 3
bin009	SOY3_bin009_02351	192	2	7	7	1.245	3.698	3.873	50S ribosomal protein L32
bin009	SOY3_bin009_02352	471	0	0	1	0.000	0.000	0.226	Thioredoxin
bin009	SOY3_bin009_02353	480	0	0	0	0.000	0.000	0.000	hypothetical protein
bin009	SOY3_bin009_02354	468	0	0	0	0.000	0.000	0.000	Cysteine synthase B
bin009	SOY3_bin009_02355	624	0	0	2	0.000	0.000	0.340	KipI antagonist
bin009	SOY3_bin009_02356	768	0	0	0	0.000	0.000	0.000	NADP-dependent 3-hydroxy acid dehydrogenase YdfG
bin009	SOY3_bin009_02357	537	0	0	1	0.000	0.000	0.198	hypothetical protein
bin009	SOY3_bin009_02358	498	0	3	2	0.000	0.611	0.427	Phosphopantetheine adenylyltransferase
bin009	SOY3_bin009_02359	609	0	1	0	0.000	0.167	0.000	Ribosomal RNA small subunit methyltransferase D
bin009	SOY3_bin009_02360	426	5	7	4	1.403	1.667	0.997	Pyridoxamine 5'-phosphate oxidase
bin009	SOY3_bin009_02361	828	3	7	3	0.433	0.857	0.385	fructoselysine 3-epimerase
bin009	SOY3_bin009_02362	234	0	0	0	0.000	0.000	0.000	hypothetical protein
bin009	SOY3_bin009_02363	1701	0	0	1	0.000	0.000	0.062	putative type I restriction enzymeP M protein
bin009	SOY3_bin009_02364	756	1	0	1	0.158	0.000	0.141	hypothetical protein
bin009	SOY3_bin009_02365	876	1	0	1	0.136	0.000	0.121	hypothetical protein
bin009	SOY3_bin009_02366	2679	0	0	1	0.000	0.000	0.040	5-methylcytosine-specific restriction enzyme B
bin009	SOY3_bin009_02367	1206	0	0	1	0.000	0.000	0.088	5-methylcytosine-specific restriction enzyme subunit McrC
bin009	SOY3_bin009_02368	267	0	0	0	0.000	0.000	0.000	PTS system galactitol-specific transporter subunit IIA
bin009	SOY3_bin009_02369	414	0	0	0	0.000	0.000	0.000	hypothetical protein
bin009	SOY3_bin009_02370	123	0	0	0	0.000	0.000	0.000	hypothetical protein
bin009	SOY3_bin009_02371	1695	3146	3391	2718	221.888	202.914	170.337	23S ribosomal RNA
bin009	SOY3_bin009_02372	116	1	1	0	1.031	0.874	0.000	5S ribosomal RNA
bin009	SOY3_bin009_02373	1563	0	3	5	0.000	0.195	0.340	Cornifin (SPRR) family protein
bin009	SOY3_bin009_02374	1014	1	2	4	0.118	0.200	0.419	Tetratricopeptide repeat protein
bin009	SOY3_bin009_02375	996	2	1	1	0.240	0.102	0.107	putative hydrolase YutF
bin009	SOY3_bin009_02376	237	2	0	0	1.009	0.000	0.000	hypothetical protein
bin009	SOY3_bin009_02377	765	0	1	0	0.000	0.133	0.000	16S/23S rRNA (cytidine-2'-O)-methyltransferase TlyA
bin009	SOY3_bin009_02378	927	1	1	0	0.129	0.109	0.000	Inorganic polyphosphate/ATP-NAD kinase
bin009	SOY3_bin009_02379	468	1	1	0	0.255	0.217	0.000	hypothetical protein
bin009	SOY3_bin009_02380	177	0	0	0	0.000	0.000	0.000	hypothetical protein
bin009	SOY3_bin009_02381	639	0	5	2	0.000	0.794	0.332	hypothetical protein
bin009	SOY3_bin009_02382	1491	0	1	3	0.000	0.068	0.214	hypothetical protein
bin009	SOY3_bin009_02383	1254	1	0	0	0.095	0.000	0.000	putative sulfoacetate transporter SauU
bin009	SOY3_bin009_02384	981	2	0	1	0.244	0.000	0.108	Calcineurin-like phosphoesterase superfamily domain protein
bin009	SOY3_bin009_02385	900	1	0	2	0.133	0.000	0.236	Cobalt-zinc-cadmium resistance protein CzcD
bin009	SOY3_bin009_02386	312	2	0	1	0.766	0.000	0.340	hypothetical protein
bin009	SOY3_bin009_02387	819	0	0	0	0.000	0.000	0.000	hypothetical protein
bin009	SOY3_bin009_02388	1794	2	9	3	0.133	0.509	0.178	hypothetical protein
bin009	SOY3_bin009_02389	831	0	0	0	0.000	0.000	0.000	hypothetical protein
bin009	SOY3_bin009_02390	1485	0	0	0	0.000	0.000	0.000	AAA-like domain protein
bin009	SOY3_bin009_02391	549	0	0	0	0.000	0.000	0.000	hypothetical protein
bin009	SOY3_bin009_02392	1779	0	0	0	0.000	0.000	0.000	Type IV secretory system Conjugative DNA transfer
bin009	SOY3_bin009_02393	441	0	0	0	0.000	0.000	0.000	Single-stranded DNA-binding protein 1
bin009	SOY3_bin009_02394	558	0	0	0	0.000	0.000	0.000	hypothetical protein
bin009	SOY3_bin009_02395	76	1	0	2	1.573	0.000	2.795	tRNA-His(gtg)
bin009	SOY3_bin009_02396	1032	2	4	2	0.232	0.393	0.206	Catabolite control protein A
bin009	SOY3_bin009_02397	1332	2	0	0	0.180	0.000	0.000	Lactose permease
bin009	SOY3_bin009_02398	1488	0	0	0	0.000	0.000	0.000	Sucrose-6-phosphate hydrolase
bin009	SOY3_bin009_02399	1173	1	1	2	0.102	0.086	0.181	hypothetical protein
bin009	SOY3_bin009_02400	327	0	2	2	0.000	0.620	0.650	hypothetical protein
bin009	SOY3_bin009_02401	303	0	0	0	0.000	0.000	0.000	hypothetical protein
bin009	SOY3_bin009_02402	1335	0	1	1	0.000	0.076	0.080	Sensor histidine kinase LiaS
bin009	SOY3_bin009_02403	822	0	0	1	0.000	0.000	0.129	hypothetical protein
bin009	SOY3_bin009_02404	759	0	0	0	0.000	0.000	0.000	ABC-2 type transporter
bin009	SOY3_bin009_02405	924	0	0	1	0.000	0.000	0.115	Daunorubicin/doxorubicin resistance ATP-binding protein DrrA
bin009	SOY3_bin009_02406	561	0	2	1	0.000	0.362	0.189	hypothetical protein
bin009	SOY3_bin009_02407	219	0	0	0	0.000	0.000	0.000	Glyoxalase-like domain protein
bin009	SOY3_bin009_02408	363	0	1	0	0.000	0.279	0.000	hypothetical protein

bin009	SOY3_bin009_02409	501	1	2	1	0.239	0.405	0.212	flavodoxin
bin009	SOY3_bin009_02410	393	2	0	1	0.608	0.000	0.270	Polyketide cyclase / dehydrase and lipid transport
bin009	SOY3_bin009_02411	474	1	2	0	0.252	0.428	0.000	Clp amino terminal domain protein
bin009	SOY3_bin009_02412	237	1	1	0	0.504	0.428	0.000	hypothetical protein
bin009	SOY3_bin009_02413	567	0	0	1	0.000	0.000	0.187	Alpha/beta hydrolase family protein
bin009	SOY3_bin009_02414	855	1	3	2	0.140	0.356	0.248	hypothetical protein
bin009	SOY3_bin009_02415	1314	3	4	3	0.273	0.309	0.243	Tryptophan synthase beta chain
bin009	SOY3_bin009_02416	831	0	0	1	0.000	0.000	0.128	Basic membrane protein
bin009	SOY3_bin009_02417	1086	0	2	2	0.000	0.187	0.196	Membrane lipoprotein TmpC precursor
bin009	SOY3_bin009_02418	1050	3	9	2	0.342	0.869	0.202	putative deferrochelatase/peroxidase YfeX
bin009	SOY3_bin009_02419	816	0	8	5	0.000	0.994	0.651	Linocin-M18
bin009	SOY3_bin009_02420	387	0	2	0	0.000	0.524	0.000	Cytidine deaminase
bin009	SOY3_bin009_02421	939	1	0	0	0.127	0.000	0.000	Ribonuclease HI
bin009	SOY3_bin009_02422	834	2	1	1	0.287	0.122	0.127	hypothetical protein
bin009	SOY3_bin009_02423	540	0	1	0	0.000	0.188	0.000	hypothetical protein
bin009	SOY3_bin009_02424	879	1	0	0	0.136	0.000	0.000	anaerobic benzoate catabolism transcriptional regulator
bin009	SOY3_bin009_02425	903	0	1	0	0.000	0.112	0.000	Morphine 6-dehydrogenase
bin009	SOY3_bin009_02426	1170	0	1	0	0.000	0.087	0.000	Putative isomerase YitF
bin009	SOY3_bin009_02427	423	0	1	0	0.000	0.240	0.000	Putative hydroxypyruvate isomerase YgbM
bin009	SOY3_bin009_02428	576	0	0	0	0.000	0.000	0.000	Putative hydrolase YdeN
bin009	SOY3_bin009_02429	75	0	1	0	0.000	1.352	0.000	tRNA-Lys(ttt)
bin009	SOY3_bin009_02430	786	0	0	0	0.000	0.000	0.000	Tyrosine-protein phosphatase precursor
bin009	SOY3_bin009_02431	234	0	0	1	0.000	0.000	0.454	hypothetical protein
bin009	SOY3_bin009_02432	399	0	0	0	0.000	0.000	0.000	Metallo-beta-lactamase superfamily protein
bin009	SOY3_bin009_02433	1026	0	0	0	0.000	0.000	0.000	Molybdopterin-synthase adenylyltransferase
bin009	SOY3_bin009_02434	516	0	0	0	0.000	0.000	0.000	hypothetical protein
bin009	SOY3_bin009_02435	1641	0	0	0	0.000	0.000	0.000	hypothetical protein
bin009	SOY3_bin009_02436	1212	0	0	0	0.000	0.000	0.000	hypothetical protein
bin009	SOY3_bin009_02437	2445	0	1	0	0.000	0.041	0.000	hypothetical protein
bin009	SOY3_bin009_02438	891	0	0	0	0.000	0.000	0.000	hypothetical protein
bin009	SOY3_bin009_02439	549	1	2	0	0.218	0.369	0.000	Spore coat protein SA
bin009	SOY3_bin009_02440	1461	0	2	1	0.000	0.139	0.073	hypothetical protein
bin009	SOY3_bin009_02441	1287	0	1	3	0.000	0.079	0.248	hypothetical protein
bin009	SOY3_bin009_02442	1419	0	1	0	0.000	0.071	0.000	Polysaccharide biosynthesis protein
bin009	SOY3_bin009_02443	1629	0	1	1	0.000	0.062	0.065	CMP-N,N'-diacetyllegionaminic acid synthase
bin009	SOY3_bin009_02444	1074	0	1	0	0.000	0.094	0.000	N,N'-diacetyllegionaminic acid synthase
bin009	SOY3_bin009_02445	1206	0	1	0	0.000	0.084	0.000	hypothetical protein
bin009	SOY3_bin009_02446	729	0	3	0	0.000	0.417	0.000	hypothetical protein
bin009	SOY3_bin009_02447	594	0	0	1	0.000	0.000	0.179	Peptidyl-tRNA hydrolase
bin009	SOY3_bin009_02448	627	1	6	4	0.191	0.971	0.678	50S ribosomal protein L25
bin009	SOY3_bin009_02449	987	3	8	2	0.363	0.822	0.215	Ribose-phosphate pyrophosphokinase
bin009	SOY3_bin009_02450	1500	2	12	7	0.159	0.811	0.496	Bifunctional protein GlmU
bin009	SOY3_bin009_02451	80	8	5	3	11.955	6.339	3.983	tRNA-Gln(ttg)
bin009	SOY3_bin009_02452	753	3	0	2	0.476	0.000	0.282	HTH-type transcriptional repressor Bm3R1
bin009	SOY3_bin009_02453	504	4	1	1	0.949	0.201	0.211	Multiple antibiotic resistance protein MarR
bin009	SOY3_bin009_02454	408	0	0	1	0.000	0.000	0.260	4-diphosphocytidyl-2-C-methyl-D-erythritol kinase
bin009	SOY3_bin009_02455	363	0	0	0	0.000	0.000	0.000	hypothetical protein
bin009	SOY3_bin009_02456	1578	0	0	0	0.000	0.000	0.000	hypothetical protein
bin009	SOY3_bin009_02457	2625	1	0	0	0.046	0.000	0.000	CRISPR-associated nuclease/helicase Cas3
bin009	SOY3_bin009_02458	990	0	1	0	0.000	0.102	0.000	hypothetical protein
bin009	SOY3_bin009_02459	1605	0	0	0	0.000	0.000	0.000	CRISPR-associated protein Cas4/endonuclease Cas1 fusion
bin009	SOY3_bin009_02460	303	0	0	0	0.000	0.000	0.000	CRISPR-associated endonuclease Cas2 2
bin009	SOY3_bin009_02461	474	0	0	0	0.000	0.000	0.000	Dihydroorotate dehydrogenase B (NAD(+)), electron transfer subunit
bin009	SOY3_bin009_02462	1194	2	1	3	0.200	0.085	0.267	Serine protease
bin009	SOY3_bin009_02463	1296	1	7	0	0.092	0.548	0.000	NADH dehydrogenase
bin009	SOY3_bin009_02464	369	0	5	1	0.000	1.374	0.288	hypothetical protein
bin009	SOY3_bin009_02465	1005	3	18	9	0.357	1.817	0.951	L-lactate dehydrogenase 2
bin009	SOY3_bin009_02466	1434	1	0	1	0.083	0.000	0.074	Signal transduction histidine-protein kinase/phosphatase MprB
bin009	SOY3_bin009_02467	708	0	5	0	0.000	0.716	0.000	Response regulator MprA
bin009	SOY3_bin009_02468	741	0	3	1	0.000	0.411	0.143	Cyanophycinase
bin009	SOY3_bin009_02469	522	1	1	0	0.229	0.194	0.000	hypothetical protein
bin009	SOY3_bin009_02470	246	1	0	2	0.486	0.000	0.864	hypothetical protein
bin009	SOY3_bin009_02471	240	0	0	0	0.000	0.000	0.000	Ppx/GppA phosphatase family protein
bin009	SOY3_bin009_02472	74	0	0	0	0.000	0.000	0.000	tRNA-Leu(taa)
bin009	SOY3_bin009_02473	432	0	0	0	0.000	0.000	0.000	2-hydroxy-3-oxopropionate reductase
bin009	SOY3_bin009_02474	378	0	0	0	0.000	0.000	0.000	2-hydroxy-3-oxopropionate reductase
bin009	SOY3_bin009_02475	591	0	0	0	0.000	0.000	0.000	Bacterial regulatory proteins, tetR family

bin009	SOY3_bin009_02476	846	0	0	0	0.000	0.000	0.000	2,5-diketo-D-gluconic acid reductase B
bin009	SOY3_bin009_02477	471	0	0	0	0.000	0.000	0.000	ATP-dependent Clp protease ATP-binding subunit ClpC1
bin009	SOY3_bin009_02478	237	0	0	0	0.000	0.000	0.000	Sigma-70, region 4
bin009	SOY3_bin009_02479	195	0	0	0	0.000	0.000	0.000	hypothetical protein
bin009	SOY3_bin009_02480	987	1	1	0	0.121	0.103	0.000	hypothetical protein
bin009	SOY3_bin009_02481	705	0	0	0	0.000	0.000	0.000	hypothetical protein
bin009	SOY3_bin009_02482	1407	1	0	0	0.085	0.000	0.000	Glutamate decarboxylase
bin009	SOY3_bin009_02483	2649	3	1	2	0.135	0.038	0.080	Lysylphosphatidylglycerol biosynthesis bifunctional protein LysX
bin009	SOY3_bin009_02484	861	1	0	0	0.139	0.000	0.000	hypothetical protein
bin009	SOY3_bin009_02485	1221	3	7	2	0.294	0.581	0.174	hypothetical protein
bin009	SOY3_bin009_02486	1686	8	28	10	0.567	1.684	0.630	Succinyl-CoA:coenzyme A transferase
bin009	SOY3_bin009_02487	651	0	1	1	0.000	0.156	0.163	Pyrophosphatase PpaX
bin009	SOY3_bin009_02488	552	1	0	1	0.217	0.000	0.192	Cys-tRNA(Pro)/Cys-tRNA(Cys) deacylase YbaK
bin009	SOY3_bin009_02489	633	0	0	0	0.000	0.000	0.000	hypothetical protein
bin009	SOY3_bin009_02490	750	0	2	0	0.000	0.270	0.000	hypothetical protein
bin009	SOY3_bin009_02491	867	1	3	0	0.138	0.351	0.000	Methionine aminopeptidase 2
bin009	SOY3_bin009_02492	867	0	4	1	0.000	0.468	0.123	hypothetical protein
bin009	SOY3_bin009_02493	1335	0	5	6	0.000	0.380	0.477	putative glutamine synthetase 2
bin009	SOY3_bin009_02494	717	0	0	1	0.000	0.000	0.148	hypothetical protein
bin009	SOY3_bin009_02495	1173	1	2	3	0.102	0.173	0.272	hypothetical protein
bin009	SOY3_bin009_02496	642	0	3	0	0.000	0.474	0.000	DNA polymerase III PolC-type
bin009	SOY3_bin009_02497	321	0	4	0	0.000	1.264	0.000	hypothetical protein
bin009	SOY3_bin009_02498	384	1	3	3	0.311	0.792	0.830	Dinitrogenase iron-molybdenum cofactor
bin009	SOY3_bin009_02499	711	0	5	1	0.000	0.713	0.149	Alkyl hydroperoxide reductase subunit C
bin009	SOY3_bin009_02500	330	0	0	0	0.000	0.000	0.000	Dinitrogenase iron-molybdenum cofactor
bin009	SOY3_bin009_02501	645	0	1	2	0.000	0.157	0.329	Peptide deformylase
bin009	SOY3_bin009_02502	996	1	1	1	0.120	0.102	0.107	hypothetical protein
bin009	SOY3_bin009_02503	456	0	3	1	0.000	0.667	0.233	Nucleotidyltransferase domain protein
bin009	SOY3_bin009_02504	402	0	1	0	0.000	0.252	0.000	HEPN domain protein
bin009	SOY3_bin009_02505	618	0	0	0	0.000	0.000	0.000	Sensor protein KdpD
bin009	SOY3_bin009_02506	1848	1	1	1	0.065	0.055	0.057	Serine/threonine-protein kinase PknA
bin009	SOY3_bin009_02507	1965	1	3	2	0.061	0.155	0.108	hypothetical protein
bin009	SOY3_bin009_02508	1494	0	0	1	0.000	0.000	0.071	putative MFS-type transporter EfpA
bin009	SOY3_bin009_02509	1506	1	2	3	0.079	0.135	0.212	CCA-adding enzyme
bin009	SOY3_bin009_02510	453	0	0	0	0.000	0.000	0.000	Threonine/homoserine exporter RhtA
bin009	SOY3_bin009_02511	810	169	464	552	24.943	58.102	72.391	Maritimacin
bin009	SOY3_bin009_02512	159	26	98	79	19.549	62.515	52.779	hypothetical protein
bin009	SOY3_bin009_02513	390	2	3	0	0.613	0.780	0.000	4'-phosphopantetheinyl transferase
bin009	SOY3_bin009_02514	1236	0	0	0	0.000	0.000	0.000	3-oxoacyl-[acyl-carrier-protein] synthase 2
bin009	SOY3_bin009_02515	282	0	0	0	0.000	0.000	0.000	Acyl carrier protein
bin009	SOY3_bin009_02516	768	0	0	0	0.000	0.000	0.000	Bacitracin export ATP-binding protein BceA
bin009	SOY3_bin009_02517	1293	2	0	1	0.185	0.000	0.082	Adenosine monophosphate-protein transferase SoFic
bin009	SOY3_bin009_02518	585	0	1	1	0.000	0.173	0.182	Acetyltransferase YpeA
bin009	SOY3_bin009_02519	360	0	1	0	0.000	0.282	0.000	hypothetical protein
bin009	SOY3_bin009_02520	1419	1	4	3	0.084	0.286	0.225	Chromosomal replication initiator protein DnaA
bin009	SOY3_bin009_02521	327	0	6	1	0.000	1.861	0.325	Ribonuclease P protein component
bin009	SOY3_bin009_02522	297	0	2	1	0.000	0.683	0.358	Putative membrane protein insertion efficiency factor
bin009	SOY3_bin009_02523	1092	0	7	2	0.000	0.650	0.195	Membrane protein insertase YidC
bin009	SOY3_bin009_02524	474	1	2	1	0.252	0.428	0.224	R3H domain protein
bin009	SOY3_bin009_02525	636	0	1	0	0.000	0.159	0.000	Ribosomal RNA small subunit methyltransferase G
bin009	SOY3_bin009_02526	873	0	1	1	0.000	0.116	0.122	Sporulation initiation inhibitor protein Soj
bin009	SOY3_bin009_02527	963	0	0	0	0.000	0.000	0.000	putative chromosome-partitioning protein ParB
bin009	SOY3_bin009_02528	114	0	1	0	0.000	0.890	0.000	Thioredoxin-1
bin009	SOY3_bin009_02529	2043	5	7	0	0.293	0.348	0.000	PTS system fructose-specific EIIABC component
bin009	SOY3_bin009_02530	1245	2	6	2	0.192	0.489	0.171	L-threonine dehydratase biosynthetic IlvA
bin009	SOY3_bin009_02531	840	3	2	1	0.427	0.241	0.126	putative oxidoreductase/MSMEI_2347
bin009	SOY3_bin009_02532	873	0	0	2	0.000	0.000	0.243	NAD-dependent protein deacetylase
bin009	SOY3_bin009_02533	1791	0	1	0	0.000	0.057	0.000	Methionine--tRNA ligase
bin009	SOY3_bin009_02534	165	0	0	0	0.000	0.000	0.000	hypothetical protein
bin009	SOY3_bin009_02535	813	1	0	0	0.147	0.000	0.000	ABC transporter ATP-binding protein YtrB
bin009	SOY3_bin009_02536	393	0	0	0	0.000	0.000	0.000	HTH-type transcriptional repressor YtrA
bin009	SOY3_bin009_02537	990	0	3	1	0.000	0.307	0.107	Capsular polysaccharide synthesis protein
bin009	SOY3_bin009_02538	348	0	0	0	0.000	0.000	0.000	hypothetical protein
bin009	SOY3_bin009_02539	810	0	0	0	0.000	0.000	0.000	Glyoxal reductase
bin009	SOY3_bin009_02540	783	0	2	1	0.000	0.259	0.136	Serine acetyltransferase
bin009	SOY3_bin009_02541	1626	2	4	1	0.147	0.250	0.065	Plasmid pRiA4b ORF-3-like protein
bin009	SOY3_bin009_02542	906	0	4	3	0.000	0.448	0.352	hypothetical protein

bin009	SOY3_bin009_02543	2010	12	23	12	0.714	1.161	0.634	Alpha-1,4-glucan:maltose-1-phosphate maltosyltransferase 1
bin009	SOY3_bin009_02544	1902	16	24	20	1.006	1.280	1.117	1,4-alpha-glucan branching enzyme GlgB
bin009	SOY3_bin009_02545	780	2	3	5	0.307	0.390	0.681	CTP pyrophosphohydrolase
bin009	SOY3_bin009_02546	915	0	6	2	0.000	0.665	0.232	Thioredoxin
bin009	SOY3_bin009_02547	372	2	1	2	0.643	0.273	0.571	Magnesium transport protein CorA
bin009	SOY3_bin009_02548	1722	1	2	0	0.069	0.118	0.000	Pyruvate dehydrogenase [ubiquinone]
bin009	SOY3_bin009_02549	1509	1	1	1	0.079	0.067	0.070	L-arabinose isomerase
bin009	SOY3_bin009_02550	690	0	3	0	0.000	0.441	0.000	L-ribulose-5-phosphate 4-epimerase UlaF
bin009	SOY3_bin009_02551	1608	2	2	0	0.149	0.126	0.000	Xylulose kinase
bin009	SOY3_bin009_02552	660	0	3	0	0.000	0.461	0.000	HTH-type transcriptional regulator DegA
bin009	SOY3_bin009_02553	1272	2	15	2	0.188	1.196	0.167	hypothetical protein
bin009	SOY3_bin009_02554	1563	1	0	0	0.076	0.000	0.000	Uronate isomerase
bin009	SOY3_bin009_02555	1395	0	3	1	0.000	0.218	0.076	Mannitol 2-dehydrogenase
bin009	SOY3_bin009_02556	1266	1	3	4	0.094	0.240	0.336	Dihydroorotase
bin009	SOY3_bin009_02557	852	0	4	3	0.000	0.476	0.374	Aminopeptidase N
bin009	SOY3_bin009_02558	732	0	4	1	0.000	0.554	0.145	hypothetical protein
bin009	SOY3_bin009_02559	3423	2	0	2	0.070	0.000	0.062	Methionine synthase
bin009	SOY3_bin009_02560	1794	2	5	3	0.133	0.283	0.178	L-arabonate dehydratase
bin009	SOY3_bin009_02561	918	0	0	0	0.000	0.000	0.000	Major Facilitator Superfamily protein
bin009	SOY3_bin009_02562	234	0	0	0	0.000	0.000	0.000	hypothetical protein
bin009	SOY3_bin009_02563	369	0	0	0	0.000	0.000	0.000	Helix-turn-helix domain protein
bin009	SOY3_bin009_02564	273	0	0	0	0.000	0.000	0.000	hypothetical protein
bin009	SOY3_bin009_02565	222	0	0	0	0.000	0.000	0.000	hypothetical protein
bin009	SOY3_bin009_02566	294	0	0	0	0.000	0.000	0.000	Transcriptional regulator WhiB7
bin009	SOY3_bin009_02567	660	0	0	0	0.000	0.000	0.000	hypothetical protein
bin009	SOY3_bin009_02568	384	0	0	0	0.000	0.000	0.000	hypothetical protein
bin009	SOY3_bin009_02569	390	0	0	0	0.000	0.000	0.000	hypothetical protein
bin009	SOY3_bin009_02570	1323	0	0	0	0.000	0.000	0.000	NACHT domain protein
bin009	SOY3_bin009_02571	237	0	0	0	0.000	0.000	0.000	hypothetical protein
bin009	SOY3_bin009_02572	336	0	0	0	0.000	0.000	0.000	hypothetical protein
bin009	SOY3_bin009_02573	228	0	0	0	0.000	0.000	0.000	Helix-turn-helix domain protein
bin009	SOY3_bin009_02574	1143	0	1	0	0.000	0.089	0.000	Putative prophage phiRv2 integrase
bin009	SOY3_bin009_02575	1629	3	2	3	0.220	0.125	0.196	hypothetical protein
bin009	SOY3_bin009_02576	360	0	0	0	0.000	0.000	0.000	hypothetical protein
bin009	SOY3_bin009_02577	747	1	0	2	0.160	0.000	0.284	hypothetical protein
bin009	SOY3_bin009_02578	1875	0	2	2	0.000	0.108	0.113	CRISPR-associated protein Cas4/endonuclease Cas1 fusion
bin009	SOY3_bin009_02579	279	0	2	0	0.000	0.727	0.000	CRISPR-associated endonuclease Cas2 2
bin009	SOY3_bin009_02580	1284	0	0	0	0.000	0.000	0.000	Neutral/alkaline non-lysosomal ceramidase
bin009	SOY3_bin009_02581	648	0	0	0	0.000	0.000	0.000	putative diguanylate cyclase
bin009	SOY3_bin009_02582	927	0	0	0	0.000	0.000	0.000	Energy-coupling factor transporter transmembrane protein EcfT
bin009	SOY3_bin009_02583	846	0	0	0	0.000	0.000	0.000	Energy-coupling factor transporter ATP-binding protein EcfA1
bin009	SOY3_bin009_02584	858	0	0	0	0.000	0.000	0.000	Energy-coupling factor transporter ATP-binding protein EcfA3
bin009	SOY3_bin009_02585	1587	0	0	0	0.000	0.000	0.000	N-acyl-D-aspartate deacylase
bin009	SOY3_bin009_02586	234	0	0	0	0.000	0.000	0.000	hypothetical protein
bin009	SOY3_bin009_02587	825	0	0	0	0.000	0.000	0.000	Nitrogenase iron protein 1
bin009	SOY3_bin009_02588	405	0	0	0	0.000	0.000	0.000	Molybdenum-pterin-binding protein 2
bin009	SOY3_bin009_02589	783	0	0	0	0.000	0.000	0.000	Sulfate-binding protein precursor
bin009	SOY3_bin009_02590	828	0	0	0	0.000	0.000	0.000	Molybdenum transport system permease protein ModB
bin009	SOY3_bin009_02591	1071	2	1	0	0.223	0.095	0.000	Putative 2-aminoethylphosphonate import ATP-binding protein PhnT
bin009	SOY3_bin009_02592	213	1	0	2	0.561	0.000	0.997	Molybdenum-pterin-binding protein MopA
bin009	SOY3_bin009_02593	1074	0	0	0	0.000	0.000	0.000	Mannosyltransferase (PIG-V))
bin009	SOY3_bin009_02594	171	0	0	0	0.000	0.000	0.000	hypothetical protein
bin009	SOY3_bin009_02595	282	0	0	0	0.000	0.000	0.000	hypothetical protein
bin009	SOY3_bin009_02596	1215	1	1	0	0.098	0.083	0.000	Cystathionine gamma-lyase
bin009	SOY3_bin009_02597	369	0	1	2	0.000	0.275	0.576	MarR family protein
bin009	SOY3_bin009_02598	171	2	1	0	1.398	0.593	0.000	YcfA-like protein
bin009	SOY3_bin009_02599	579	0	1	0	0.000	0.175	0.000	Methionine gamma-lyase
bin009	SOY3_bin009_02600	513	1	1	1	0.233	0.198	0.207	Putative acetyltransferase
bin009	SOY3_bin009_02601	474	0	1	0	0.000	0.214	0.000	Nucleotidyltransferase domain protein
bin009	SOY3_bin009_02602	240	1	0	0	0.498	0.000	0.000	Ribbon-helix-helix protein, copG family
bin009	SOY3_bin009_02603	324	0	0	0	0.000	0.000	0.000	hypothetical protein
bin009	SOY3_bin009_02604	558	0	1	0	0.000	0.182	0.000	Isopentenyl-diphosphate Delta-isomerase
bin009	SOY3_bin009_02605	747	0	0	0	0.000	0.000	0.000	HeH/LEM domain protein
bin009	SOY3_bin009_02606	1362	2	2	2	0.176	0.149	0.156	hypothetical protein
bin009	SOY3_bin009_02607	615	1	3	2	0.194	0.495	0.345	DSBA-like thioredoxin domain protein
bin009	SOY3_bin009_02608	741	4	10	7	0.645	1.369	1.003	hypothetical protein
bin009	SOY3_bin009_02609	2202	5	4	0	0.271	0.184	0.000	DNA ligase

bin009	SOY3_bin009_02610	459	1	6	2	0.260	1.326	0.463	Ribose-5-phosphate isomerase B
bin009	SOY3_bin009_02611	74	0	0	0	0.000	0.000	0.000	tRNA-Pro(tgg)
bin009	SOY3_bin009_02612	627	0	2	0	0.000	0.324	0.000	GTP cyclohydrolase 1
bin009	SOY3_bin009_02613	633	2	1	0	0.378	0.160	0.000	hypothetical protein
bin009	SOY3_bin009_02614	552	0	1	0	0.000	0.184	0.000	Hypoxanthine-guanine phosphoribosyltransferase
bin009	SOY3_bin009_02615	999	0	0	0	0.000	0.000	0.000	tRNA(Ile)-lysidine synthase
bin009	SOY3_bin009_02616	1044	0	0	0	0.000	0.000	0.000	hypothetical protein
bin009	SOY3_bin009_02617	1422	2	0	0	0.168	0.000	0.000	D-alanyl-D-alanine carboxypeptidase DacC precursor
bin009	SOY3_bin009_02618	561	7	19	11	1.492	3.435	2.083	Inorganic pyrophosphatase
bin009	SOY3_bin009_02619	1689	2	3	0	0.142	0.180	0.000	Putative ribonuclease J
bin009	SOY3_bin009_02620	1428	38	99	49	3.181	7.032	3.645	Citrate transporter
bin009	SOY3_bin009_02621	1302	4	6	10	0.367	0.467	0.816	carbohydrate diacid transcriptional activator CdaR
bin009	SOY3_bin009_02622	1407	2	9	6	0.170	0.649	0.453	Transposase DDE domain protein
bin009	SOY3_bin009_02623	723	0	0	0	0.000	0.000	0.000	putative metallo-hydrolase YflN
bin009	SOY3_bin009_02624	693	0	0	0	0.000	0.000	0.000	HTH-type transcriptional repressor KstR2
bin009	SOY3_bin009_02625	1242	0	1	2	0.000	0.082	0.171	Putative NAD(P)H nitroreductase acg
bin009	SOY3_bin009_02626	687	1	2	3	0.174	0.295	0.464	putative S-adenosyl-L-methionine-dependent methyltransferase TehB
bin009	SOY3_bin009_02627	489	0	0	0	0.000	0.000	0.000	putative N-acetyltransferase YafP
bin009	SOY3_bin009_02628	882	0	0	0	0.000	0.000	0.000	hypothetical protein
bin009	SOY3_bin009_02629	1047	0	2	2	0.000	0.194	0.203	Tropinesterase
bin009	SOY3_bin009_02630	129	0	0	0	0.000	0.000	0.000	NADP-dependent isopropanol dehydrogenase
bin009	SOY3_bin009_02631	1440	0	1	0	0.000	0.070	0.000	Putative niacin/nicotinamide transporter NaiP
bin009	SOY3_bin009_02632	1206	0	0	1	0.000	0.000	0.088	tRNA-dihydrouridine synthase C
bin009	SOY3_bin009_02633	1131	1	0	0	0.106	0.000	0.000	Deoxyguanosinetriphosphate triphosphohydrolase
bin009	SOY3_bin009_02634	1875	1	3	1	0.064	0.162	0.057	DNA primase
bin009	SOY3_bin009_02635	2268	0	1	0	0.000	0.045	0.000	RAMP superfamily protein
bin009	SOY3_bin009_02636	537	0	0	0	0.000	0.000	0.000	hypothetical protein
bin009	SOY3_bin009_02637	1518	1	1	0	0.079	0.067	0.000	RAMP superfamily protein
bin009	SOY3_bin009_02638	1542	1	1	1	0.078	0.066	0.069	hypothetical protein
bin009	SOY3_bin009_02639	1152	13	19	26	1.349	1.673	2.397	Periplasmic serine endoprotease DegP precursor
bin009	SOY3_bin009_02640	966	28	39	83	3.465	4.095	9.127	hypothetical protein
bin009	SOY3_bin009_02641	444	3	6	6	0.808	1.371	1.435	Phosphate-starvation-inducible E
bin009	SOY3_bin009_02642	1689	22	27	59	1.557	1.621	3.711	putative diguanylate cyclase YegE
bin009	SOY3_bin009_02643	1380	53	95	231	4.591	6.982	17.781	ATP-dependent zinc metalloprotease FtsH 4
bin009	SOY3_bin009_02644	1023	0	0	0	0.000	0.000	0.000	Serine/threonine-protein kinase PknD
bin009	SOY3_bin009_02645	3309	1	0	1	0.036	0.000	0.032	hypothetical protein
bin009	SOY3_bin009_02646	1287	0	0	0	0.000	0.000	0.000	Transcription-repair-coupling factor
bin009	SOY3_bin009_02647	522	0	3	0	0.000	0.583	0.000	hypothetical protein
bin009	SOY3_bin009_02648	633	1	0	1	0.189	0.000	0.168	Nucleoside triphosphate pyrophosphohydrolase
bin009	SOY3_bin009_02649	1290	40	93	40	3.707	7.312	3.294	Enolase
bin009	SOY3_bin009_02650	687	1	1	1	0.174	0.148	0.155	cell division protein FtsB
bin009	SOY3_bin009_02651	537	1	1	0	0.223	0.189	0.000	hypothetical protein
bin009	SOY3_bin009_02652	945	1	1	0	0.127	0.107	0.000	Guanosine-5'-triphosphate,3'-diphosphate pyrophosphatase
bin009	SOY3_bin009_02653	74	0	0	0	0.000	0.000	0.000	tRNA-Leu(taa)
bin009	SOY3_bin009_02654	1338	2	15	9	0.179	1.137	0.715	Phosphoglucomutase
bin009	SOY3_bin009_02655	708	2	17	11	0.338	2.435	1.650	preprotein translocase subunit SecG
bin009	SOY3_bin009_02656	444	7	18	15	1.885	4.112	3.589	Glyoxalase/Bleomycin resistance protein/Dioxygenase superfamily protein
bin009	SOY3_bin009_02657	1500	3	13	10	0.239	0.879	0.708	F0F1 ATP synthase subunit B
bin009	SOY3_bin009_02658	777	2	0	0	0.308	0.000	0.000	hypothetical protein
bin009	SOY3_bin009_02659	1422	0	5	3	0.000	0.357	0.224	Argininosuccinate synthase
bin009	SOY3_bin009_02660	1014	5	4	12	0.589	0.400	1.257	Quinone oxidoreductase 1
bin009	SOY3_bin009_02661	85	0	0	0	0.000	0.000	0.000	tRNA-Ser(tga)
bin009	SOY3_bin009_02662	468	0	0	0	0.000	0.000	0.000	hypothetical protein
bin009	SOY3_bin009_02663	1818	6	6	2	0.395	0.335	0.117	Transcriptional regulator LytR
bin009	SOY3_bin009_02664	92	4	3	0	5.198	3.307	0.000	tRNA-Ser(gct)
bin009	SOY3_bin009_02665	975	22	65	34	2.698	6.762	3.704	Universal stress protein/MSMEI_3859
bin009	SOY3_bin009_02666	88	1	1	0	1.359	1.153	0.000	tRNA-Ser(gga)
bin009	SOY3_bin009_02667	372	0	4	1	0.000	1.091	0.286	hypothetical protein
bin009	SOY3_bin009_02668	1380	1	7	4	0.087	0.514	0.308	Di-/tripeptide transporter
bin009	SOY3_bin009_02669	840	0	1	0	0.000	0.121	0.000	Vibriobactin utilization protein ViuB
bin009	SOY3_bin009_02670	954	1	3	0	0.125	0.319	0.000	4-carboxy-2-hydroxymuconate-6-semialdehyde dehydrogenase
bin009	SOY3_bin009_02671	1149	9	10	17	0.936	0.883	1.572	Dihydroorotate dehydrogenase B (NAD(+)), catalytic subunit
bin009	SOY3_bin009_02672	2247	2	2	0	0.106	0.090	0.000	Copper-exporting P-type ATPase A
bin009	SOY3_bin009_02673	207	0	0	0	0.000	0.000	0.000	Copper chaperone CopZ
bin009	SOY3_bin009_02674	294	2	0	0	0.813	0.000	0.000	Copper-sensing transcriptional repressor CsoR
bin009	SOY3_bin009_02675	519	0	1	0	0.000	0.195	0.000	Alpha/beta hydrolase family protein
bin009	SOY3_bin009_02676	1056	0	1	1	0.000	0.096	0.101	Lactose operon repressor

bin009	SOY3_bin009_02677	1116	7	5	1	0.750	0.454	0.095	Resuscitation-promoting factor Rpf2 precursor
bin009	SOY3_bin009_02678	141	0	0	0	0.000	0.000	0.000	hypothetical protein
bin009	SOY3_bin009_02679	603	21	37	14	4.163	6.224	2.466	Superoxide dismutase [Mn/Fe]
bin009	SOY3_bin009_02680	195	45	41	21	27.588	21.326	11.440	CsbD-like protein
bin009	SOY3_bin009_02681	600	0	0	0	0.000	0.000	0.000	Ribosomal RNA small subunit methyltransferase C
bin009	SOY3_bin009_02682	909	0	0	0	0.000	0.000	0.000	tRNA pseudouridine synthase A
bin009	SOY3_bin009_02683	744	1	0	0	0.161	0.000	0.000	tRNA (guanine-N(7)-)-methyltransferase
bin009	SOY3_bin009_02684	417	3	4	6	0.860	0.973	1.528	Inner membrane protein YccF
bin009	SOY3_bin009_02685	282	5	3	2	2.120	1.079	0.753	Elongation factor G
bin009	SOY3_bin009_02686	249	0	0	1	0.000	0.000	0.427	hypothetical protein
bin009	SOY3_bin009_02687	123	0	0	1	0.000	0.000	0.864	hypothetical protein
bin009	SOY3_bin009_02688	681	0	1	1	0.000	0.149	0.156	Transcriptional regulatory protein LiaR
bin009	SOY3_bin009_02689	780	0	1	0	0.000	0.130	0.000	1D-myo-inositol 2-acetamido-2-deoxy-alpha-D-glucopyranoside deacetylase
bin009	SOY3_bin009_02690	1131	0	1	1	0.000	0.090	0.094	D-inositol 3-phosphate glycosyltransferase
bin009	SOY3_bin009_02691	873	3	12	3	0.411	1.394	0.365	Putative glucose-6-phosphate 1-epimerase
bin009	SOY3_bin009_02692	558	3	5	2	0.643	0.909	0.381	hypothetical protein
bin009	SOY3_bin009_02693	246	0	0	1	0.000	0.000	0.432	hypothetical protein
bin009	SOY3_bin009_02694	135	0	0	0	0.000	0.000	0.000	hypothetical protein
bin009	SOY3_bin009_02695	846	2	6	2	0.283	0.719	0.251	Ethanolamine ammonia-lyase light chain
bin009	SOY3_bin009_02696	1365	8	16	8	0.701	1.189	0.623	Ethanolamine ammonia-lyase heavy chain
bin009	SOY3_bin009_02697	1431	3	9	3	0.251	0.638	0.223	reactivating factor for ethanolamine ammonia lyase
bin009	SOY3_bin009_02698	1029	3	6	3	0.349	0.591	0.310	Alcohol dehydrogenase
bin009	SOY3_bin009_02699	939	1	6	1	0.127	0.648	0.113	Phosphate acetyltransferase
bin009	SOY3_bin009_02700	750	0	1	0	0.000	0.135	0.000	hypothetical protein
bin009	SOY3_bin009_02701	804	3	9	2	0.446	1.135	0.264	DNA polymerase III subunit beta
bin009	SOY3_bin009_02702	1149	0	1	2	0.000	0.088	0.185	DNA replication and repair protein RecF
bin009	SOY3_bin009_02703	603	1	1	0	0.198	0.168	0.000	hypothetical protein
bin009	SOY3_bin009_02704	1956	0	0	0	0.000	0.000	0.000	hypothetical protein
bin009	SOY3_bin009_02705	525	0	0	0	0.000	0.000	0.000	hypothetical protein
bin009	SOY3_bin009_02706	567	0	1	0	0.000	0.179	0.000	hypothetical protein
bin009	SOY3_bin009_02707	279	0	0	0	0.000	0.000	0.000	Diol dehydratase-reactivating factor alpha subunit
bin009	SOY3_bin009_02708	360	0	0	0	0.000	0.000	0.000	hypothetical protein
bin009	SOY3_bin009_02709	567	0	0	0	0.000	0.000	0.000	hypothetical protein
bin009	SOY3_bin009_02710	279	0	0	0	0.000	0.000	0.000	Propanediol utilization protein PduA
bin009	SOY3_bin009_02711	645	0	0	0	0.000	0.000	0.000	Phosphate propanoyltransferase
bin009	SOY3_bin009_02712	837	0	0	0	0.000	0.000	0.000	Chaperone protein DnaK
bin009	SOY3_bin009_02713	828	0	0	0	0.000	0.000	0.000	bifunctional phosphopantothenoylcysteine decarboxylase/phosphopantothenate synthase
bin009	SOY3_bin009_02714	270	0	0	0	0.000	0.000	0.000	Carbon dioxide concentrating mechanism protein CcmL
bin009	SOY3_bin009_02715	1029	0	0	0	0.000	0.000	0.000	Cob(I)yrinic acid a,c-diamide adenosyltransferase
bin009	SOY3_bin009_02716	486	0	0	0	0.000	0.000	0.000	hypothetical protein
bin009	SOY3_bin009_02717	1368	2	0	1	0.175	0.000	0.078	hypothetical protein
bin009	SOY3_bin009_02718	2139	0	2	0	0.000	0.095	0.000	hypothetical protein
bin009	SOY3_bin009_02719	1044	0	4	3	0.000	0.389	0.305	recombinase A
bin009	SOY3_bin009_02720	318	5	13	10	1.880	4.146	3.340	Ethanolamine ammonia-lyase heavy chain
bin009	SOY3_bin009_02721	906	19	48	6	2.507	5.374	0.703	Ethanolamine ammonia-lyase light chain
bin009	SOY3_bin009_02722	657	17	43	17	3.093	6.638	2.749	Ethanolamine utilization protein EutL
bin009	SOY3_bin009_02723	1530	35	61	35	2.735	4.044	2.430	Aldehyde-alcohol dehydrogenase
bin009	SOY3_bin009_02724	294	20	32	17	8.133	11.040	6.142	Major carboxysome shell protein 1A
bin009	SOY3_bin009_02725	639	17	30	18	3.180	4.762	2.992	Ethanolamine utilization protein EutQ
bin009	SOY3_bin009_02726	531	1	8	3	0.225	1.528	0.600	hypothetical protein
bin009	SOY3_bin009_02727	249	0	2	0	0.000	0.815	0.000	hypothetical protein
bin009	SOY3_bin009_02728	921	0	1	0	0.000	0.110	0.000	hypothetical protein
bin009	SOY3_bin009_02729	258	0	0	0	0.000	0.000	0.000	hypothetical protein
bin009	SOY3_bin009_02730	360	0	0	0	0.000	0.000	0.000	hypothetical protein
bin009	SOY3_bin009_02731	195	0	0	0	0.000	0.000	0.000	hypothetical protein
bin009	SOY3_bin009_02732	1347	5	2	5	0.444	0.151	0.394	UDP-N-acetylmuramoylalanine--D-glutamate ligase
bin009	SOY3_bin009_02733	1356	0	5	6	0.000	0.374	0.470	hypothetical protein
bin009	SOY3_bin009_02734	903	2	2	3	0.265	0.225	0.353	hypothetical protein
bin009	SOY3_bin009_02735	243	0	0	1	0.000	0.000	0.437	Transposase, Mutator family
bin009	SOY3_bin009_02736	270	0	0	0	0.000	0.000	0.000	hypothetical protein
bin009	SOY3_bin009_02737	1374	0	2	0	0.000	0.148	0.000	Inner membrane metabolite transport protein YhjE
bin009	SOY3_bin009_02738	1191	0	1	0	0.000	0.085	0.000	putative hydrolase YxeP
bin009	SOY3_bin009_02739	1005	3	1	0	0.357	0.101	0.000	Zinc-type alcohol dehydrogenase-like protein
bin009	SOY3_bin009_02740	267	2	4	1	0.895	1.520	0.398	Ferredoxin
bin009	SOY3_bin009_02741	357	0	0	0	0.000	0.000	0.000	Rifampin ADP-ribosyl transferase
bin009	SOY3_bin009_02742	888	16	16	10	2.154	1.828	1.196	Fructose-bisphosphate aldolase class 1
bin009	SOY3_bin009_02743	1791	1	0	0	0.067	0.000	0.000	Helicase IV

bin009	SOY3_bin009_02744	348	0	0	0	0.000	0.000	0.000	HicB family protein
bin009	SOY3_bin009_02745	246	2	2	0	0.972	0.825	0.000	hypothetical protein
bin009	SOY3_bin009_02746	321	0	0	0	0.000	0.000	0.000	Transposase
bin009	SOY3_bin009_02747	828	1	0	1	0.144	0.000	0.128	IS2 transposase TnpB
bin009	SOY3_bin009_02748	228	0	0	0	0.000	0.000	0.000	hypothetical protein
bin009	SOY3_bin009_02749	924	9	19	6	1.164	2.086	0.690	hypothetical protein
bin009	SOY3_bin009_02750	2049	1	0	0	0.058	0.000	0.000	Acetyl-coenzyme A synthetase
bin009	SOY3_bin009_02751	1065	1	1	2	0.112	0.095	0.199	hypothetical protein
bin009	SOY3_bin009_02752	912	8	15	4	1.049	1.668	0.466	Calcineurin-like phosphoesterase superfamily domain protein
bin009	SOY3_bin009_02753	435	0	0	2	0.000	0.000	0.488	hypothetical protein
bin009	SOY3_bin009_02754	75	0	0	0	0.000	0.000	0.000	tRNA-Ala(tgc)
bin009	SOY3_bin009_02755	630	0	1	0	0.000	0.161	0.000	hypothetical protein
bin009	SOY3_bin009_02756	600	0	0	0	0.000	0.000	0.000	hypothetical protein
bin009	SOY3_bin009_02757	936	0	0	1	0.000	0.000	0.113	hypothetical protein
bin009	SOY3_bin009_02758	564	0	1	0	0.000	0.180	0.000	hypothetical protein
bin009	SOY3_bin009_02759	204	0	0	0	0.000	0.000	0.000	hypothetical protein
bin009	SOY3_bin009_02760	765	0	2	1	0.000	0.265	0.139	hypothetical protein
bin009	SOY3_bin009_02761	1083	0	1	1	0.000	0.094	0.098	2-oxoglutaramate amidase
bin009	SOY3_bin009_02762	2160	6	7	8	0.332	0.329	0.393	Copper-exporting P-type ATPase B
bin009	SOY3_bin009_02763	813	7	13	8	1.029	1.622	1.045	Putative osmoprotectant uptake system substrate-binding protein OsmF precursor
bin009	SOY3_bin009_02764	204	0	0	0	0.000	0.000	0.000	hypothetical protein
bin009	SOY3_bin009_02765	837	0	0	0	0.000	0.000	0.000	Aminodeoxychorismate synthase component 1
bin009	SOY3_bin009_02766	1263	2	2	0	0.189	0.161	0.000	DEAD/DEAH box helicase
bin009	SOY3_bin009_02767	1074	4	1	0	0.445	0.094	0.000	Transposase
bin009	SOY3_bin009_02768	561	5	0	0	1.065	0.000	0.000	hypothetical protein
bin009	SOY3_bin009_02769	744	0	0	0	0.000	0.000	0.000	hypothetical protein
bin009	SOY3_bin009_02770	2229	0	0	0	0.000	0.000	0.000	Beta-L-arabinobiosidase precursor
bin009	SOY3_bin009_02771	636	0	0	0	0.000	0.000	0.000	UTP--glucose-1-phosphate uridylyltransferase
bin009	SOY3_bin009_02772	852	1	0	0	0.140	0.000	0.000	Transcription antiterminator LicT
bin009	SOY3_bin009_02773	1437	0	0	0	0.000	0.000	0.000	Aryl-phospho-beta-D-glucosidase BglA
bin009	SOY3_bin009_02774	1959	0	1	0	0.000	0.052	0.000	PTS system beta-glucoside-specific EIIBCA component
bin009	SOY3_bin009_02775	597	2	2	0	0.400	0.340	0.000	Bacterial extracellular solute-binding protein, family 7
bin009	SOY3_bin009_02776	1158	0	2	2	0.000	0.175	0.183	putative GTP-binding protein EngB
bin009	SOY3_bin009_02777	282	0	0	0	0.000	0.000	0.000	hypothetical protein
bin009	SOY3_bin009_02778	438	2	0	0	0.546	0.000	0.000	hypothetical protein
bin009	SOY3_bin009_02779	408	0	1	0	0.000	0.249	0.000	hypothetical protein
bin009	SOY3_bin009_02780	366	1	4	3	0.327	1.108	0.871	Sigma-70, region 4
bin009	SOY3_bin009_02781	198	1	4	1	0.604	2.049	0.536	YcfA-like protein
bin009	SOY3_bin009_02782	213	0	0	0	0.000	0.000	0.000	hypothetical protein
bin009	SOY3_bin009_02783	768	1	0	0	0.156	0.000	0.000	hypothetical protein
bin009	SOY3_bin009_02784	336	0	0	0	0.000	0.000	0.000	Transposase
bin009	SOY3_bin009_02785	930	0	1	0	0.000	0.109	0.000	sn-glycerol-3-phosphate transport system permease protein UgpA
bin009	SOY3_bin009_02786	930	0	0	0	0.000	0.000	0.000	L-arabinose transport system permease protein AraQ
bin009	SOY3_bin009_02787	651	0	1	0	0.000	0.156	0.000	sn-glycerol-3-phosphate-binding periplasmic protein UgpB precursor
bin009	SOY3_bin009_02788	984	1	0	0	0.121	0.000	0.000	cobalamin biosynthesis protein
bin009	SOY3_bin009_02789	1050	0	3	0	0.000	0.290	0.000	Quinolinate synthase A
bin009	SOY3_bin009_02790	1635	2	1	0	0.146	0.062	0.000	L-aspartate oxidase
bin009	SOY3_bin009_02791	897	0	3	0	0.000	0.339	0.000	Nicotinate-nucleotide pyrophosphorylase [carboxylating]
bin009	SOY3_bin009_02792	405	0	0	0	0.000	0.000	0.000	VanZ like family protein
bin009	SOY3_bin009_02793	744	1	1	5	0.161	0.136	0.714	hypothetical protein
bin009	SOY3_bin009_02794	1158	0	1	4	0.000	0.088	0.367	hypothetical protein
bin009	SOY3_bin009_02795	1044	0	3	3	0.000	0.291	0.305	2,3-diketo-L-gulonate-binding periplasmic protein YiaO precursor
bin009	SOY3_bin009_02796	1035	0	0	0	0.000	0.000	0.000	2,3-diketo-L-gulonate-binding periplasmic protein YiaO precursor
bin009	SOY3_bin009_02797	174	0	0	1	0.000	0.000	0.610	hypothetical protein
bin009	SOY3_bin009_02798	993	0	6	2	0.000	0.613	0.214	cell envelope integrity inner membrane protein TolA
bin009	SOY3_bin009_02799	1050	1	5	3	0.114	0.483	0.304	putative endopeptidase p60 precursor
bin009	SOY3_bin009_02800	1674	6	10	16	0.428	0.606	1.015	Glucose-6-phosphate isomerase
bin009	SOY3_bin009_02801	642	2	1	4	0.372	0.158	0.662	vancomycin high temperature exclusion protein
bin009	SOY3_bin009_02802	639	0	1	0	0.000	0.159	0.000	Homoserine/homoserine lactone efflux protein
bin009	SOY3_bin009_02803	453	0	2	0	0.000	0.448	0.000	Leucine-responsive regulatory protein
bin009	SOY3_bin009_02804	258	0	0	0	0.000	0.000	0.000	hypothetical protein
bin009	SOY3_bin009_02805	168	0	0	0	0.000	0.000	0.000	hypothetical protein
bin009	SOY3_bin009_02806	669	0	0	0	0.000	0.000	0.000	Propanediol diffusion facilitator
bin009	SOY3_bin009_02807	651	1	0	0	0.184	0.000	0.000	Arsenate-mycothiol transferase ArsC2
bin009	SOY3_bin009_02808	657	0	1	1	0.000	0.154	0.162	Arsenate-mycothiol transferase ArsC1
bin009	SOY3_bin009_02809	318	0	0	0	0.000	0.000	0.000	hypothetical protein
bin009	SOY3_bin009_02810	807	0	2	1	0.000	0.251	0.132	prenyltransferase

bin009	SOY3_bin009_02811	1122	1	1	1	0.107	0.090	0.095	Pentachlorophenol 4-monooxygenase
bin009	SOY3_bin009_02812	708	0	0	2	0.000	0.000	0.300	hypothetical protein
bin009	SOY3_bin009_02813	1140	2	1	1	0.210	0.089	0.093	Alpha-pyrone synthesis polyketide synthase-like Pks18
bin009	SOY3_bin009_02814	600	0	1	0	0.000	0.169	0.000	5-dehydro-2-deoxygluconokinase
bin009	SOY3_bin009_02815	672	0	0	0	0.000	0.000	0.000	hypothetical protein
bin009	SOY3_bin009_02816	696	0	3	1	0.000	0.437	0.153	Nucleoside triphosphatase NudI
bin009	SOY3_bin009_02817	810	2	5	4	0.295	0.626	0.525	Bax inhibitor 1 like protein
bin009	SOY3_bin009_02818	1455	3	9	4	0.246	0.627	0.292	hypothetical protein
bin009	SOY3_bin009_02819	603	0	1	2	0.000	0.168	0.352	hypothetical protein
bin009	SOY3_bin009_02820	120	0	0	0	0.000	0.000	0.000	hypothetical protein
bin009	SOY3_bin009_02821	1530	0	2	1	0.000	0.133	0.069	von Willebrand factor type A domain protein
bin009	SOY3_bin009_02822	915	0	0	0	0.000	0.000	0.000	hypothetical protein
bin009	SOY3_bin009_02823	1296	1	3	2	0.092	0.235	0.164	Glutamate-1-semialdehyde 2,1-aminomutase
bin009	SOY3_bin009_02824	975	0	0	0	0.000	0.000	0.000	Delta-aminolevulinic acid dehydratase
bin009	SOY3_bin009_02825	369	0	0	0	0.000	0.000	0.000	Putative fluoride ion transporter CrcB
bin009	SOY3_bin009_02826	459	0	0	0	0.000	0.000	0.000	camphor resistance protein CrcB
bin009	SOY3_bin009_02827	534	1	0	1	0.224	0.000	0.199	Stress response protein NhaX
bin009	SOY3_bin009_02828	756	0	0	0	0.000	0.000	0.000	uroporphyrinogen-III synthase
bin009	SOY3_bin009_02829	267	0	0	0	0.000	0.000	0.000	porphobilinogen deaminase
bin009	SOY3_bin009_02830	2070	0	0	0	0.000	0.000	0.000	hypothetical protein
bin009	SOY3_bin009_02831	504	0	0	0	0.000	0.000	0.000	hypothetical protein
bin009	SOY3_bin009_02832	606	0	0	0	0.000	0.000	0.000	Putative hydrolase YdeN
bin009	SOY3_bin009_02833	885	0	1	0	0.000	0.115	0.000	hypothetical protein
bin009	SOY3_bin009_02834	660	0	0	0	0.000	0.000	0.000	hypothetical protein
bin009	SOY3_bin009_02835	1233	0	0	1	0.000	0.000	0.086	hypothetical protein
bin009	SOY3_bin009_02836	462	0	1	1	0.000	0.220	0.230	hypothetical protein
bin009	SOY3_bin009_02837	294	4	2	2	1.627	0.690	0.723	hypothetical protein
bin009	SOY3_bin009_02838	93	0	0	0	0.000	0.000	0.000	hypothetical protein
bin009	SOY3_bin009_02839	249	0	0	0	0.000	0.000	0.000	5'-methylthioadenosine/S-adenosylhomocysteine nucleosidase
bin009	SOY3_bin009_02840	861	1	1	2	0.139	0.118	0.247	Histidine kinase
bin009	SOY3_bin009_02841	579	1	0	1	0.206	0.000	0.183	Response regulator protein VraR
bin009	SOY3_bin009_02842	780	5	3	3	0.766	0.390	0.409	hypothetical protein
bin009	SOY3_bin009_02843	906	13	4	6	1.715	0.448	0.703	hypothetical protein
bin009	SOY3_bin009_02844	738	0	0	0	0.000	0.000	0.000	5'-methylthioadenosine/S-adenosylhomocysteine nucleosidase
bin009	SOY3_bin009_02845	1623	1	8	4	0.074	0.500	0.262	hypothetical protein
bin009	SOY3_bin009_02846	543	0	0	0	0.000	0.000	0.000	Adenine phosphoribosyltransferase
bin009	SOY3_bin009_02847	1230	0	1	0	0.000	0.082	0.000	preprotein translocase subunit SecF
bin009	SOY3_bin009_02848	852	1	3	1	0.140	0.357	0.125	preprotein translocase subunit SecD
bin009	SOY3_bin009_02849	1152	1	4	0	0.104	0.352	0.000	Threonine synthase
bin009	SOY3_bin009_02850	723	1	8	6	0.165	1.122	0.882	Octanoyltransferase
bin009	SOY3_bin009_02851	1005	3	0	3	0.357	0.000	0.317	Lipoyl synthase
bin009	SOY3_bin009_02852	1659	1	5	3	0.072	0.306	0.192	Valine--tRNA ligase
bin009	SOY3_bin009_02853	1485	0	4	0	0.000	0.273	0.000	putative sensor histidine kinase pdtaS
bin009	SOY3_bin009_02854	276	5	4	1	2.166	1.470	0.385	Anti-sigma factor RsrA
bin009	SOY3_bin009_02855	732	13	30	13	2.123	4.157	1.887	ECF RNA polymerase sigma factor SigR
bin009	SOY3_bin009_02856	594	0	12	7	0.000	2.049	1.252	DoxX
bin009	SOY3_bin009_02857	240	0	0	0	0.000	0.000	0.000	3-oxoacyl-[acyl-carrier-protein] reductase FabG
bin009	SOY3_bin009_02858	687	1	0	0	0.174	0.000	0.000	HEAT repeat protein
bin009	SOY3_bin009_02859	1416	0	0	0	0.000	0.000	0.000	putative transport protein HsrA
bin009	SOY3_bin009_02860	585	0	0	0	0.000	0.000	0.000	putative transcriptional regulator
bin009	SOY3_bin009_02861	1407	1	0	1	0.085	0.000	0.075	Glutamate decarboxylase
bin009	SOY3_bin009_02862	213	0	0	0	0.000	0.000	0.000	hypothetical protein
bin009	SOY3_bin009_02863	570	0	0	0	0.000	0.000	0.000	GMP synthase [glutamine-hydrolyzing]
bin009	SOY3_bin009_02864	903	0	1	0	0.000	0.112	0.000	Polyketide synthase PksR
bin009	SOY3_bin009_02865	1266	0	2	2	0.000	0.160	0.168	Gramicidin S synthase 2
bin009	SOY3_bin009_02866	921	1	3	4	0.130	0.330	0.461	hypothetical protein
bin009	SOY3_bin009_02867	555	1	0	0	0.215	0.000	0.000	2-amino-4-hydroxy-6-hydroxymethyldihydropteridine pyrophosphokinase
bin009	SOY3_bin009_02868	519	0	1	0	0.000	0.195	0.000	putative dihydroneopterin aldolase
bin009	SOY3_bin009_02869	798	1	2	3	0.150	0.254	0.399	Dihydropteroate synthase 1
bin009	SOY3_bin009_02870	627	1	3	5	0.191	0.485	0.847	GTP cyclohydrolase 1
bin009	SOY3_bin009_02871	1110	5	5	3	0.539	0.457	0.287	hypothetical protein
bin009	SOY3_bin009_02872	348	1	0	1	0.344	0.000	0.305	Hypoxanthine-guanine phosphoribosyltransferase
bin009	SOY3_bin009_02873	672	0	5	1	0.000	0.755	0.158	hypothetical protein
bin009	SOY3_bin009_02874	1287	3	7	2	0.279	0.552	0.165	putative sulfoacetate transporter SauU
bin009	SOY3_bin009_02875	588	0	1	1	0.000	0.172	0.181	Oxygen-insensitive NADPH nitroreductase
bin009	SOY3_bin009_02876	627	0	2	0	0.000	0.324	0.000	DNA-3-methyladenine glycosylase 1
bin009	SOY3_bin009_02877	681	1	2	2	0.176	0.298	0.312	Thiol-disulfide oxidoreductase ResA

bin009	SOY3_bin009_02878	156	0	0	0	0.000	0.000	0.000	Cytochrome c biogenesis protein CcsA
bin009	SOY3_bin009_02879	867	2	7	7	0.276	0.819	0.858	Trigger factor
bin009	SOY3_bin009_02880	1134	0	2	0	0.000	0.179	0.000	AI-2 transport protein TqsA
bin009	SOY3_bin009_02881	1296	1	4	1	0.092	0.313	0.082	AI-2 transport protein TqsA
bin009	SOY3_bin009_02882	615	1	1	1	0.194	0.165	0.173	ATP-dependent Clp protease proteolytic subunit 2
bin009	SOY3_bin009_02883	204	0	0	0	0.000	0.000	0.000	6-phosphogluconolactonase
bin009	SOY3_bin009_02884	1056	0	1	0	0.000	0.096	0.000	HTH-type transcriptional regulator GalR
bin009	SOY3_bin009_02885	1515	0	0	0	0.000	0.000	0.000	Ribose import ATP-binding protein RbsA
bin009	SOY3_bin009_02886	993	0	0	0	0.000	0.000	0.000	Ribose transport system permease protein RbsC
bin009	SOY3_bin009_02887	978	0	1	0	0.000	0.104	0.000	4-hydroxy-3-methylbut-2-enyl diphosphate reductase
bin009	SOY3_bin009_02888	1446	0	0	0	0.000	0.000	0.000	DNA recombination protein RmuC
bin009	SOY3_bin009_02889	780	0	0	0	0.000	0.000	0.000	HTH-type transcriptional repressor YvoA
bin009	SOY3_bin009_02890	1386	0	0	0	0.000	0.000	0.000	putative dTDP-4-dehydrorhamnose 3,5-epimerase
bin009	SOY3_bin009_02891	1002	1	1	0	0.119	0.101	0.000	dTDP-glucose 4,6-dehydratase
bin009	SOY3_bin009_02892	873	0	0	0	0.000	0.000	0.000	Glucose-1-phosphate thymidylyltransferase
bin009	SOY3_bin009_02893	753	24	25	29	3.810	3.367	4.091	hypothetical protein
bin009	SOY3_bin009_02894	720	0	2	1	0.000	0.282	0.148	Alkaline phosphatase synthesis transcriptional regulatory protein PhoP
bin009	SOY3_bin009_02895	1125	0	0	0	0.000	0.000	0.000	Signal transduction histidine-protein kinase BaeS
bin009	SOY3_bin009_02896	1131	0	0	0	0.000	0.000	0.000	hypothetical protein
bin009	SOY3_bin009_02897	1290	0	4	3	0.000	0.315	0.247	Chaperone protein DnaJ
bin009	SOY3_bin009_02898	237	0	0	0	0.000	0.000	0.000	hypothetical protein
bin009	SOY3_bin009_02899	474	1	1	0	0.252	0.214	0.000	hypothetical protein
bin009	SOY3_bin009_02900	1026	0	0	0	0.000	0.000	0.000	Lactose operon repressor
bin009	SOY3_bin009_02901	1275	1	0	0	0.094	0.000	0.000	putative MFS family transporter protein
bin009	SOY3_bin009_02902	357	1	0	0	0.335	0.000	0.000	HTH-type transcriptional repressor YtrA
bin009	SOY3_bin009_02903	1008	1	1	1	0.119	0.101	0.105	hypothetical protein
bin009	SOY3_bin009_02904	147	0	0	0	0.000	0.000	0.000	hypothetical protein
bin009	SOY3_bin009_02905	1308	9	4	2	0.823	0.310	0.162	Transposase
bin009	SOY3_bin009_02906	507	0	0	0	0.000	0.000	0.000	GTP pyrophosphokinase
bin009	SOY3_bin009_02907	225	0	0	0	0.000	0.000	0.000	hypothetical protein
bin009	SOY3_bin009_02908	318	0	0	0	0.000	0.000	0.000	hypothetical protein
bin009	SOY3_bin009_02909	273	0	0	0	0.000	0.000	0.000	hypothetical protein
bin009	SOY3_bin009_02910	363	0	0	0	0.000	0.000	0.000	hypothetical protein
bin009	SOY3_bin009_02911	627	0	0	0	0.000	0.000	0.000	Transposase, Mutator family
bin009	SOY3_bin009_02912	2340	0	0	0	0.000	0.000	0.000	Calcium-transporting ATPase 1
bin009	SOY3_bin009_02913	372	0	0	0	0.000	0.000	0.000	hypothetical protein
bin009	SOY3_bin009_02914	423	0	0	0	0.000	0.000	0.000	hypothetical protein
bin009	SOY3_bin009_02915	630	0	0	1	0.000	0.000	0.169	Phosphoserine phosphatase 1
bin009	SOY3_bin009_02916	414	0	3	3	0.000	0.735	0.770	Ribosomal silencing factor RsfS
bin009	SOY3_bin009_02917	708	0	1	3	0.000	0.143	0.450	putative nicotinate-nucleotide adenylyltransferase
bin009	SOY3_bin009_02918	1014	0	1	0	0.000	0.100	0.000	Putative thiamine biosynthesis protein
bin009	SOY3_bin009_02919	780	0	1	0	0.000	0.130	0.000	Putative aliphatic sulfonates transport permease protein SsuC
bin009	SOY3_bin009_02920	513	0	1	0	0.000	0.198	0.000	Bicarbonate transport ATP-binding protein CmpD
bin009	SOY3_bin009_02921	528	0	0	0	0.000	0.000	0.000	Bacterial membrane flanked domain protein
bin009	SOY3_bin009_02922	510	0	3	3	0.000	0.597	0.625	hypothetical protein
bin009	SOY3_bin009_02923	498	0	1	0	0.000	0.204	0.000	putative thiol peroxidase
bin009	SOY3_bin009_02924	74	0	0	0	0.000	0.000	0.000	tRNA-Gly(ccc)
bin009	SOY3_bin009_02925	543	0	2	0	0.000	0.374	0.000	PemK-like protein
bin009	SOY3_bin009_02926	474	0	1	0	0.000	0.214	0.000	hypothetical protein
bin009	SOY3_bin009_02927	420	0	2	3	0.000	0.483	0.759	UDP-glucose 4-epimerase
bin009	SOY3_bin009_02928	1509	1	7	2	0.079	0.471	0.141	Galactose-1-phosphate uridylyltransferase
bin009	SOY3_bin009_02929	585	0	0	1	0.000	0.000	0.182	hypothetical protein
bin009	SOY3_bin009_02930	348	0	0	1	0.000	0.000	0.305	Threonine efflux protein
bin009	SOY3_bin009_02931	74	0	0	0	0.000	0.000	0.000	tRNA-Leu(taa)
bin009	SOY3_bin009_02932	654	0	0	0	0.000	0.000	0.000	Guanosine-5'-triphosphate,3'-diphosphate pyrophosphatase
bin009	SOY3_bin009_02933	768	0	0	1	0.000	0.000	0.138	Energy-coupling factor transporter transmembrane protein EcfT
bin009	SOY3_bin009_02934	1698	0	0	1	0.000	0.000	0.063	Putative HMP/thiamine import ATP-binding protein YkoD
bin009	SOY3_bin009_02935	543	0	0	1	0.000	0.000	0.196	hypothetical protein
bin009	SOY3_bin009_02936	720	0	1	2	0.000	0.141	0.295	putative HTH-type transcriptional regulator YurK
bin009	SOY3_bin009_02937	2652	1	4	1	0.045	0.153	0.040	CHAT domain protein
bin009	SOY3_bin009_02938	399	2	0	1	0.599	0.000	0.266	hypothetical protein
bin009	SOY3_bin009_02939	396	0	0	0	0.000	0.000	0.000	Integrase core domain protein
bin009	SOY3_bin009_02940	1059	1	9	2	0.113	0.862	0.201	Cell wall synthesis protein Wag31
bin009	SOY3_bin009_02941	642	1	0	2	0.186	0.000	0.331	Uracil phosphoribosyltransferase
bin009	SOY3_bin009_02942	738	1	4	6	0.162	0.550	0.864	hypothetical protein
bin009	SOY3_bin009_02943	438	0	0	0	0.000	0.000	0.000	tRNA-specific adenosine deaminase
bin009	SOY3_bin009_02944	87	0	0	1	0.000	0.000	1.221	tRNA-Ser(cga)

bin009	SOY3_bin009_02945	1176	0	1	0	0.000	0.086	0.000	Multiple sugar-binding periplasmic receptor ChvE precursor
bin009	SOY3_bin009_02946	1545	0	0	0	0.000	0.000	0.000	Xylose import ATP-binding protein XylG
bin009	SOY3_bin009_02947	1176	0	0	0	0.000	0.000	0.000	Xylose transport system permease protein XylH
bin009	SOY3_bin009_02948	333	0	0	0	0.000	0.000	0.000	hypothetical protein
bin009	SOY3_bin009_02949	258	0	0	0	0.000	0.000	0.000	Citrate transporter
bin009	SOY3_bin009_02950	2079	3	2	2	0.173	0.098	0.102	Long-chain-fatty-acid--CoA ligase
bin009	SOY3_bin009_02951	825	0	1	1	0.000	0.123	0.129	Quercetin 2,3-dioxygenase
bin009	SOY3_bin009_02952	2100	20	53	30	1.139	2.560	1.518	PTS system mannitol-specific EIICBA component
bin009	SOY3_bin009_02953	1131	19	52	28	2.008	4.663	2.630	Mannitol-1-phosphate 5-dehydrogenase
bin009	SOY3_bin009_02954	297	1	5	3	0.403	1.708	1.073	hypothetical protein
bin009	SOY3_bin009_02955	216	0	0	1	0.000	0.000	0.492	hypothetical protein
bin009	SOY3_bin009_02956	426	2	2	0	0.561	0.476	0.000	Glucose-specific phosphotransferase enzyme IIA component
bin009	SOY3_bin009_02957	681	3	0	0	0.527	0.000	0.000	hypothetical protein
bin009	SOY3_bin009_02958	1275	1	3	1	0.094	0.239	0.083	Serine--tRNA ligase
bin009	SOY3_bin009_02959	930	1	3	1	0.129	0.327	0.114	Diacylglycerol kinase
bin009	SOY3_bin009_02960	1569	0	0	0	0.000	0.000	0.000	Cna protein B-type domain protein
bin009	SOY3_bin009_02961	1563	3	5	1	0.229	0.324	0.068	Transposase DDE domain protein
bin009	SOY3_bin009_02962	300	3	3	5	1.195	1.014	1.770	hypothetical protein
bin009	SOY3_bin009_02963	636	0	5	1	0.000	0.797	0.167	Trehalose transport system permease protein SugB
bin009	SOY3_bin009_02964	264	16	8	7	7.245	3.074	2.817	hypothetical protein
bin009	SOY3_bin009_02965	558	0	0	2	0.000	0.000	0.381	DNA protection during starvation protein 2
bin009	SOY3_bin009_02966	495	34	65	26	8.211	13.319	5.580	Fine tangled pili major subunit
bin009	SOY3_bin009_02967	870	0	0	0	0.000	0.000	0.000	putative oxidoreductase YghA
bin009	SOY3_bin009_02968	303	0	0	0	0.000	0.000	0.000	hypothetical protein
bin009	SOY3_bin009_02969	621	0	1	0	0.000	0.163	0.000	hypothetical protein
bin009	SOY3_bin009_02970	471	1	3	3	0.254	0.646	0.677	hypothetical protein
bin009	SOY3_bin009_02971	891	0	0	0	0.000	0.000	0.000	Glucose-1-phosphate thymidylyltransferase
bin009	SOY3_bin009_02972	498	0	0	2	0.000	0.000	0.427	hypothetical protein
bin009	SOY3_bin009_02973	471	0	0	3	0.000	0.000	0.677	undecaprenyldiphospho-muramoylpentapeptide beta-N- acetylglucosaminyltransferase
bin009	SOY3_bin009_02974	1461	3	9	8	0.245	0.625	0.582	UDP-glucose:undecaprenyl-phosphate glucose-1-phosphate transferase
bin009	SOY3_bin009_02975	642	0	0	0	0.000	0.000	0.000	Tyrosine-protein kinase YwqD
bin009	SOY3_bin009_02976	3639	1	4	2	0.033	0.111	0.058	DnaA regulatory inactivator Hda
bin009	SOY3_bin009_02977	288	5	9	15	2.076	3.170	5.533	Methylmalonyl-CoA carboxyltransferase 12S subunit
bin009	SOY3_bin009_02978	363	5	17	13	1.647	4.750	3.804	Methylmalonyl-CoA carboxyltransferase 1.3S subunit
bin009	SOY3_bin009_02979	762	1	7	4	0.157	0.932	0.558	NADP-dependent 3-hydroxy acid dehydrogenase YdfG
bin009	SOY3_bin009_02980	1116	0	6	2	0.000	0.545	0.190	hypothetical protein
bin009	SOY3_bin009_02981	750	0	5	4	0.000	0.676	0.567	2,3-bisphosphoglycerate-dependent phosphoglycerate mutase
bin009	SOY3_bin009_02982	1338	2	4	1	0.179	0.303	0.079	Histidine--tRNA ligase
bin009	SOY3_bin009_02983	693	2	2	1	0.345	0.293	0.153	putative metallo-hydrolase
bin009	SOY3_bin009_02984	1254	2	7	4	0.191	0.566	0.339	hypothetical protein
bin009	SOY3_bin009_02985	495	0	1	2	0.000	0.205	0.429	Bifunctional (p)ppGpp synthase/hydrolase relA
bin009	SOY3_bin009_02986	603	0	1	0	0.000	0.168	0.000	manganese efflux pump MntP
bin009	SOY3_bin009_02987	360	0	1	0	0.000	0.282	0.000	HTH-type transcriptional regulator CmtR
bin009	SOY3_bin009_02988	597	1	6	3	0.200	1.019	0.534	DNA-invertase hin
bin009	SOY3_bin009_02989	1398	1	2	0	0.086	0.145	0.000	Transposon Tn7 transposition protein TnsB
bin009	SOY3_bin009_02990	336	0	0	0	0.000	0.000	0.000	hypothetical protein
bin009	SOY3_bin009_02991	372	0	0	0	0.000	0.000	0.000	Chromate transport protein
bin009	SOY3_bin009_02992	1446	1	2	3	0.083	0.140	0.220	Amino-acid carrier protein AlsT
bin009	SOY3_bin009_02993	1107	2	0	1	0.216	0.000	0.096	CAAX amino terminal protease self- immunity
bin009	SOY3_bin009_02994	525	1	1	2	0.228	0.193	0.405	2-phosphoglycerate kinase
bin009	SOY3_bin009_02995	135	0	0	0	0.000	0.000	0.000	hypothetical protein
bin009	SOY3_bin009_02996	1218	2	6	2	0.196	0.500	0.174	Isocitrate dehydrogenase [NADP]
bin009	SOY3_bin009_02997	843	0	0	0	0.000	0.000	0.000	Formyltetrahydrofolate deformylase
bin009	SOY3_bin009_02998	819	0	0	0	0.000	0.000	0.000	Cobalt import ATP-binding protein CbiO
bin009	SOY3_bin009_02999	612	1	0	0	0.195	0.000	0.000	Precorrin-6Y C(5,15)-methyltransferase [decarboxylating]
bin009	SOY3_bin009_03000	771	0	0	0	0.000	0.000	0.000	Uroporphyrinogen-III C-methyltransferase
bin009	SOY3_bin009_03001	732	0	2	1	0.000	0.277	0.145	Precorrin-6A reductase
bin009	SOY3_bin009_03002	1017	3	3	1	0.353	0.299	0.104	hypothetical protein
bin009	SOY3_bin009_03003	1329	2	5	1	0.180	0.382	0.080	hypothetical protein
bin009	SOY3_bin009_03004	837	0	1	0	0.000	0.121	0.000	Adenylosuccinate lyase
bin009	SOY3_bin009_03005	1077	1	3	2	0.111	0.283	0.197	Branched-chain-amino-acid aminotransferase
bin009	SOY3_bin009_03006	1416	0	6	1	0.000	0.430	0.075	NAD(P)H dehydrogenase (quinone)
bin009	SOY3_bin009_03007	930	0	1	0	0.000	0.109	0.000	Glutamine-dependent NAD(+) synthetase
bin009	SOY3_bin009_03008	336	0	0	0	0.000	0.000	0.000	hypothetical protein
bin009	SOY3_bin009_03009	207	0	1	1	0.000	0.490	0.513	Helix-turn-helix domain protein
bin009	SOY3_bin009_03010	432	0	0	1	0.000	0.000	0.246	hypothetical protein
bin009	SOY3_bin009_03011	1230	0	1	1	0.000	0.082	0.086	Putative prophage phiRv2 integrase

bin009	SOY3_bin009_03012	864	1	5	2	0.138	0.587	0.246	CHAD domain protein
bin009	SOY3_bin009_03013	1344	0	0	0	0.000	0.000	0.000	Cobalt-precorrin-3B C(17)-methyltransferase
bin009	SOY3_bin009_03014	1227	1	1	1	0.097	0.083	0.087	Sirohydrochlorin cobaltochelatase
bin009	SOY3_bin009_03015	804	0	0	0	0.000	0.000	0.000	Cobalt-precorrin-2 C(20)-methyltransferase
bin009	SOY3_bin009_03016	432	0	1	1	0.000	0.235	0.246	Phosphoribosylaminoimidazole-succinocarboxamide synthase
bin009	SOY3_bin009_03017	222	4	4	4	2.154	1.828	1.914	phosphoribosylformylglycinamidine synthase subunit PurS
bin009	SOY3_bin009_03018	675	3	6	5	0.531	0.902	0.787	Phosphoribosylformylglycinamidine synthase 1
bin009	SOY3_bin009_03019	1530	2	5	4	0.156	0.331	0.278	hypothetical protein
bin009	SOY3_bin009_03020	300	0	1	0	0.000	0.338	0.000	hypothetical protein
bin009	SOY3_bin009_03021	909	0	0	0	0.000	0.000	0.000	Glycosyl hydrolase family 81
bin009	SOY3_bin009_03022	729	0	0	0	0.000	0.000	0.000	Alcohol acetyltransferase
bin009	SOY3_bin009_03023	672	0	0	0	0.000	0.000	0.000	hypothetical protein
bin009	SOY3_bin009_03024	156	0	0	0	0.000	0.000	0.000	hypothetical protein
bin009	SOY3_bin009_03025	1329	0	3	3	0.000	0.229	0.240	Putrescine oxidase
bin009	SOY3_bin009_03026	339	0	1	0	0.000	0.299	0.000	Nitrogen regulatory protein P-II
bin009	SOY3_bin009_03027	315	0	0	0	0.000	0.000	0.000	hypothetical protein
bin009	SOY3_bin009_03028	1524	0	2	0	0.000	0.133	0.000	Competence protein ComM
bin009	SOY3_bin009_03029	1140	0	1	0	0.000	0.089	0.000	hypothetical protein
bin009	SOY3_bin009_03030	276	0	1	0	0.000	0.367	0.000	Dinitrogenase iron-molybdenum cofactor
bin009	SOY3_bin009_03031	606	3	9	0	0.592	1.506	0.000	hypothetical protein
bin009	SOY3_bin009_03032	1284	7	22	3	0.652	1.738	0.248	Galactokinase
bin009	SOY3_bin009_03033	606	0	3	1	0.000	0.502	0.175	hypothetical protein
bin009	SOY3_bin009_03034	309	0	4	0	0.000	1.313	0.000	hypothetical protein
bin009	SOY3_bin009_03035	234	0	0	0	0.000	0.000	0.000	hypothetical protein
bin009	SOY3_bin009_03036	459	0	0	0	0.000	0.000	0.000	hypothetical protein
bin009	SOY3_bin009_03037	2547	1	4	5	0.047	0.159	0.209	Serine/threonine-protein kinase PknK
bin009	SOY3_bin009_03038	450	1	1	0	0.266	0.225	0.000	hypothetical protein
bin009	SOY3_bin009_03039	942	3	4	7	0.381	0.431	0.789	Cytochrome c biogenesis protein CcsA
bin009	SOY3_bin009_03040	1698	3	10	5	0.211	0.597	0.313	Cytochrome c biogenesis protein CcsB
bin009	SOY3_bin009_03041	720	4	2	3	0.664	0.282	0.443	thiol:disulfide interchange protein precursor
bin009	SOY3_bin009_03042	633	1	0	0	0.189	0.000	0.000	Tetracycline repressor protein class E
bin009	SOY3_bin009_03043	816	0	1	0	0.000	0.124	0.000	putative metallo-hydrolase YflN
bin009	SOY3_bin009_03044	933	2	1	0	0.256	0.109	0.000	O-acetylserine sulfhydrylase
bin009	SOY3_bin009_03045	1194	3	9	2	0.300	0.765	0.178	Riboflavin biosynthesis protein RibD
bin009	SOY3_bin009_03046	624	1	5	0	0.192	0.813	0.000	Riboflavin synthase
bin009	SOY3_bin009_03047	1230	3	5	6	0.292	0.412	0.518	Riboflavin biosynthesis protein RibBA
bin009	SOY3_bin009_03048	420	2	1	0	0.569	0.241	0.000	hypothetical protein
bin009	SOY3_bin009_03049	1302	4	7	0	0.367	0.545	0.000	Transposase
bin009	SOY3_bin009_03050	1236	1	1	0	0.097	0.082	0.000	DNA polymerase III PolC-type
bin009	SOY3_bin009_03051	1209	0	1	2	0.000	0.084	0.176	putative FAD-linked oxidoreductase
bin009	SOY3_bin009_03052	663	0	0	0	0.000	0.000	0.000	D-methionine transport system permease protein MetI
bin009	SOY3_bin009_03053	1071	0	1	0	0.000	0.095	0.000	Methionine import ATP-binding protein MetN 2
bin009	SOY3_bin009_03054	339	0	0	0	0.000	0.000	0.000	DL-methionine transporter substrate-binding subunit
bin009	SOY3_bin009_03055	1164	10	29	21	1.027	2.527	1.916	hypothetical protein
bin009	SOY3_bin009_03056	324	6	8	9	2.214	2.504	2.951	Nucleoid-associated protein Lsr2
bin009	SOY3_bin009_03057	555	0	1	3	0.000	0.183	0.574	2-amino-4-hydroxy-6-hydroxymethyldihydropteridine pyrophosphokinase
bin009	SOY3_bin009_03058	384	0	0	1	0.000	0.000	0.277	putative dihydroneopterin aldolase
bin009	SOY3_bin009_03059	597	0	0	0	0.000	0.000	0.000	Dihydropteroate synthase 1
bin009	SOY3_bin009_03060	1515	0	0	1	0.000	0.000	0.070	hypothetical protein
bin009	SOY3_bin009_03061	246	0	0	0	0.000	0.000	0.000	hypothetical protein
bin009	SOY3_bin009_03062	435	0	1	0	0.000	0.233	0.000	hypothetical protein
bin009	SOY3_bin009_03063	630	0	0	0	0.000	0.000	0.000	Cytosol aminopeptidase
bin009	SOY3_bin009_03064	1665	0	0	0	0.000	0.000	0.000	p-aminobenzoyl-glutamate transport protein
bin009	SOY3_bin009_03065	1026	0	0	0	0.000	0.000	0.000	Glutaminase 1
bin009	SOY3_bin009_03066	150	0	0	0	0.000	0.000	0.000	hypothetical protein
bin009	SOY3_bin009_03067	612	1	0	0	0.195	0.000	0.000	3-methyladenine DNA glycosylase
bin009	SOY3_bin009_03068	411	11	20	10	3.200	4.936	2.585	hypothetical protein
bin009	SOY3_bin009_03069	393	1	1	4	0.304	0.258	1.081	hypothetical protein
bin009	SOY3_bin009_03070	522	4	8	4	0.916	1.554	0.814	hypothetical protein
bin009	SOY3_bin009_03071	954	0	0	0	0.000	0.000	0.000	Diaminopropionate ammonia-lyase
bin009	SOY3_bin009_03072	1278	0	0	0	0.000	0.000	0.000	N-carbamoyl-L-amino acid hydrolase
bin009	SOY3_bin009_03073	651	0	0	0	0.000	0.000	0.000	Asp/Glu/Hydantoin racemase
bin009	SOY3_bin009_03074	375	0	0	0	0.000	0.000	0.000	Bacterial transcription activator, effector binding domain
bin009	SOY3_bin009_03075	879	0	0	0	0.000	0.000	0.000	anaerobic benzoate catabolism transcriptional regulator
bin009	SOY3_bin009_03076	891	1	1	3	0.134	0.114	0.358	Transposase
bin009	SOY3_bin009_03077	1050	0	0	0	0.000	0.000	0.000	Kinase A inhibitor
bin009	SOY3_bin009_03078	837	0	0	0	0.000	0.000	0.000	LamB/YcsF family protein

bin009	SOY3_bin009_03079	609	2	1	0	0.393	0.167	0.000	Bacterial regulatory proteins, tetR family
bin009	SOY3_bin009_03080	3144	0	1	1	0.000	0.032	0.034	hypothetical protein
bin009	SOY3_bin009_03081	402	0	1	0	0.000	0.252	0.000	HTH-type transcriptional repressor YtrA
bin009	SOY3_bin009_03082	408	0	0	0	0.000	0.000	0.000	hypothetical protein
bin009	SOY3_bin009_03083	1386	1	2	0	0.086	0.146	0.000	hypothetical protein
bin009	SOY3_bin009_03084	324	0	0	0	0.000	0.000	0.000	hypothetical protein
bin009	SOY3_bin009_03085	2808	1	2	0	0.043	0.072	0.000	Chromosome partition protein Smc
bin009	SOY3_bin009_03086	2448	0	0	0	0.000	0.000	0.000	Cobyrinic acid A,C-diamide synthase
bin009	SOY3_bin009_03087	360	0	0	0	0.000	0.000	0.000	hypothetical protein
bin009	SOY3_bin009_03088	798	0	0	0	0.000	0.000	0.000	hypothetical protein
bin009	SOY3_bin009_03089	276	0	0	0	0.000	0.000	0.000	hypothetical protein
bin009	SOY3_bin009_03090	795	0	0	0	0.000	0.000	0.000	hypothetical protein
bin009	SOY3_bin009_03091	198	0	0	0	0.000	0.000	0.000	hypothetical protein
bin009	SOY3_bin009_03092	165	0	0	0	0.000	0.000	0.000	hypothetical protein
bin009	SOY3_bin009_03093	381	0	0	0	0.000	0.000	0.000	Sensory transduction protein regX3
bin009	SOY3_bin009_03094	1128	0	0	0	0.000	0.000	0.000	Signal transduction histidine-protein kinase BaeS
bin009	SOY3_bin009_03095	303	0	0	0	0.000	0.000	0.000	hypothetical protein
bin009	SOY3_bin009_03096	540	0	0	0	0.000	0.000	0.000	hypothetical protein
bin009	SOY3_bin009_03097	1011	0	0	0	0.000	0.000	0.000	hypothetical protein
bin009	SOY3_bin009_03098	1518	0	0	0	0.000	0.000	0.000	hypothetical protein
bin009	SOY3_bin009_03099	240	0	0	0	0.000	0.000	0.000	hypothetical protein
bin009	SOY3_bin009_03100	1110	0	2	0	0.000	0.183	0.000	hypothetical protein
bin009	SOY3_bin009_03101	1107	1	1	6	0.108	0.092	0.576	putative oxidoreductase/MSMEI_1564
bin009	SOY3_bin009_03102	327	2	2	2	0.731	0.620	0.650	T-protein
bin009	SOY3_bin009_03103	555	2	4	5	0.431	0.731	0.957	Serine acetyltransferase
bin009	SOY3_bin009_03104	426	0	1	0	0.000	0.238	0.000	hypothetical protein
bin009	SOY3_bin009_03105	312	0	0	0	0.000	0.000	0.000	hypothetical protein
bin009	SOY3_bin009_03106	408	0	2	1	0.000	0.497	0.260	hypothetical protein
bin009	SOY3_bin009_03107	1539	0	1	1	0.000	0.066	0.069	GTPase Obg
bin009	SOY3_bin009_03108	291	0	0	0	0.000	0.000	0.000	Scramblase
[truncated: 642,747 more chars]
